# Dissection of molecular and histological subtypes of papillary thyroid cancer using alternative splicing profiles

**DOI:** 10.1038/s12276-022-00740-0

**Published:** 2022-03-11

**Authors:** Jiyeon Park, Dongmoung Kim, Jin-Ok Lee, Hyeon-Chun Park, Brian Y. Ryu, Ju Han Kim, Sug Hyung Lee, Yeun-Jun Chung

**Affiliations:** 1 Precision Medicine Research Center, College of Medicine, The Catholic University of Korea, Seoul, Republic of Korea; 2 Integrated Research Center for Genome Polymorphism, College of Medicine, The Catholic University of Korea, Seoul, Republic of Korea; 3 Department of Biomedicine & Health Sciences, Graduate School, The Catholic University of Korea, Seoul, Republic of Korea; 4 Department of Microbiology, College of Medicine, The Catholic University of Korea, Seoul, Republic of Korea; 5 Seoul National University Biomedical Informatics, Division of Biomedical Informatics, Seoul National University College of Medicine, Seoul, Republic of Korea; 6 Department of Pathology, College of Medicine, The Catholic University of Korea, Seoul, Republic of Korea

**Keywords:** Cancer genomics, RNA splicing

## Abstract

Despite growing evidence of the relevance of alternative splicing (AS) to cancer development and progression, the biological implications of AS for tumor behaviors, including papillary thyroid cancer (PTC), remain elusive. With the aim of further understanding the molecular and histological subtypes of PTC, we in this study explored whether AS events might act as new molecular determinants. For this purpose, AS profiles were analyzed in RNA-sequencing data from The Cancer Genome Atlas (TCGA) and from a Korean patient dataset. A total of 23 distinct exon-skipping (ES) events that correlated significantly with PTC oncogenic activity and differentiation scores were identified. The two top-ranked ES events, NUMA1_17515 in exon 18 of *NUMA1* and TUBB3_38175 in exon 6 of *TUBB3*, showed high correlations with oncogenic activities and discriminated histological and molecular subtypes of PTC. Furthermore, two novel intron-retention (IR) events for *TUBB3* were uncovered. All ES and IR events for the *TUBB3* gene were predicted to induce nonsense-mediated mRNA decay. The relative abundances of intron reads in the PTC dataset from TCGA showed IR levels to differ significantly among PTC subtypes, possibly reflecting their different tumor behaviors. This study provides a landscape of AS changes among PTC subtypes and identified two significant AS events, NUMA1_17515 and TUBB3_38175, as potential AS biomarkers for PTC subclassification and characterization. The AS events identified in this study may be involved in the development of phenotypic differences underlying the functional characteristics and histological differentiation of PTCs.

## Thyroid cancer: Biomarkers could improve subtype classification

Two potential biomarkers uncovered by scientists in South Korea may help more accurately classify subtypes of papillary thyroid cancer, the most common form of thyroid cancer, and improve treatment regimens. Ascertaining the correct papillary thyroid cancer (PTC) subtype is important for patient prognoses and treatment plans. Growing evidence suggests that cancer progression may be influenced by ‘alternative splicing’ events, alterations to mRNA that change the structure of mRNA transcripts and affect the function of encoded proteins. Yeun-Jun Chung and Sug Hyung Lee at the Catholic University of Korea, Seoul, and co-workers explored alternative splicing events in PTC patient samples. They identified 25 distinct events associated with oncogenic activity and differentiation between PTC subtypes. Of these, two events associated with two separate genes are particularly significant and could prove useful as biomarkers for disease classification and characterisation.

## Introduction

There are four main types of thyroid cancer (papillary, follicular, medullary, and anaplastic) based on histology. Papillary thyroid cancer (PTC), which accounts for ~90% of thyroid cancers, has a better prognosis than the other types^1^. Approximately 50% of PTCs are the classical subtype (cPTC); the other 50% consist of less common histological variants, including follicular (fvPTC) and tall cell (tPTC) variants. The course of tPTC is more aggressive than that of cPTC, including increased lymph node metastasis and higher mortality. Histological classification of fvPTC is often problematic because it shares histological features with follicular thyroid cancer (FTC)^2,3^. Therefore, determining PTC subtypes is an important step in predicting patient prognoses and designing proper treatment plans.

The frequency of somatic mutations is relatively low in PTC compared with that in other thyroid cancers. *BRAF, RAS*, and *RET* are the most commonly mutated genes in PTCs, but their distribution in PTC subtypes does not determine the subtypes^4^. Recent next-generation sequencing (NGS)-based comprehensive genomic characterization of PTC has allowed its reclassification into five molecular subgroups, providing better information about molecular characteristics than conventional histological classification^5^. However, ambiguities in both histological subtypes and molecular subgroups remain^5,6^. Therefore, a more sophisticated molecular classification of PTC is needed for the precise diagnosis and treatment of thyroid cancers.

Since thyroid cancer has a low mutation burden^7^, RNA alterations, such as alternative splicing (AS), are likely to play an important role in its tumorigenesis and biological features^8,9^. Most eukaryotic multiexon genes undergo AS after transcription, which expands transcriptomic diversity. Many AS events involve exon skipping (ES) and intron retention (IR), producing structural differences in mRNA transcripts and affecting the functions of the encoded proteins^10^. In addition, some AS events introduce premature stop codons, triggering nonsense-mediated mRNA decay (NMD) or encoding truncated proteins^11^. Recent progress in NGS technology has enabled genome-wide profiling of AS events^12–14^, and pancancer analyses using data from The Cancer Genome Atlas (TCGA) have identified a substantial number of AS events and somatic mutations in splicing factor genes in cancers^15–18^. Furthermore, AS has recently been highlighted as a source of neoantigens, which are candidate targets for immunotherapy^17,19^. Despite growing evidence for the involvement of AS regulation in cancer development and progression, its biological implications on tumor behavior remain elusive. Therefore, the aim of this study was to identify AS events that would allow for a better understanding of PTC.

## Materials and methods

### RNA-sequencing datasets

We used two independent datasets based on tissues from patients with cancer from TCGA and the Seoul National University Hospital (SNUH), Korea. All thyroid carcinoma (THCA) samples in the dataset from TCGA were PTCs (*n* = 505); those in the SNUH dataset were PTCs (76 cPTC and 49 fvPTC) and FTCs (*n* = 30) from Korean patients. TCGA raw sequencing files (fastq format) and clinical data were obtained through the Genomic Data Commons portal (https://portal.gdc.cancer.gov/legacy-archive), and SNUH data were downloaded from the European Nucleotide Archive database (http://www.ebi.ac.uk/ena/data/view/PRJEB11591). Information on TCGA molecular subtypes was retrieved using the R package TCGAbiolinks^20^.

### Quantification and functional annotation of AS events

For TCGA data, percent spliced in (PSI) values were used to quantify each AS event in the database TCGA SpliceSeq, which classifies AS events into seven splicing types: alternate promoter (AP), alternate terminator (AT), exon skip, retained intron, alternate donor site, alternate acceptor site, and mutually exclusive exons^21^. Significant ES events were annotated using ASpedia, a functional annotation database for human AS events^22^. ES events were visualized with the Genotype–Tissue Expression (GTEx) portal (https://gtexportal.org/home/)^23^. To identify upstream regulators of splicing events, motifs of RNA-binding proteins were analyzed using rMAPS^24^. For SNUH data, PSI values were calculated using SpliceSeq software^25^. To calculate gene-level expression values for both TCGA and SNUH data, sequencing reads were aligned to a human reference genome (hg19) using HISAT2 software^26^, and counts were calculated at the gene level using featureCounts software^27^.

### Identification of PTC subtype-discriminating ES events

To discover ES events with significant differences among PTC molecular subtypes, analysis of variance (ANOVA) was performed using PSI values. Both an ANOVA *p* value of <0.0001 and adjusted *R*^2^ > 0.1 were used to identify statistically significant ES events. ANOVA was followed by Tukey’s honest significant difference test to identify pairs of molecular subtypes that would lead to a difference in ES events. To explore whether ES events might serve as a new classifier of PTC subtypes, a random forest analysis was performed using the ‘randomForest’ R package. A random forest of classification trees was built, and the importance of variables for the model was determined by comparing Gini importance scores (mean decrease in Gini), as described elsewhere^28^. The higher the Gini importance score is, the higher the importance of the variable in the model is. To reduce variance in predictions, random forest analysis was performed 10 times under different random seeds, with an average of 10 Gini importance scores calculated for each variable.

### Analysis of the correlation between ES events and the biological characteristics of PTC

The thyroid differentiation score (TDS) was calculated based on the mRNA expression levels of 16 genes related to thyroid metabolism and function^5^. The extracellular signal-regulated kinase (ERK) score was calculated by summarizing the mRNA expression levels of 52 genes that are responsive to MEK inhibition, as described elsewhere^29^. RAS scores for RAS pathway activation were obtained from a previous TCGA pancancer analysis^30^. Pearson’s correlation coefficients were calculated between PSI values of ES events and the TDS, as well as ERK and RAS scores. To assess the clinical implications of AS events, a *t test* or ANOVA was applied to compare PSI values in the given clinical categories.

### Analysis of IR using RNA-seq data

The numbers of sequencing reads around AS events were calculated using BAM format alignment data and SAMtools^31^, and the numbers of reads within intronic regions and across junctions of two flanking exons were calculated. The total library size was used to normalize counts. The IR ratio was calculated as the normalized intron read count divided by the normalized exon–exon junction read count^32^. A low IR ratio indicates that a junction is primarily spliced, whereas a high ratio indicates that a junction is primarily unspliced.

### Gene set analysis

Biological pathways associated with significant genes were analyzed using Gene Ontology (GO) annotations (http://www.geneontology.org). Mapping between genes and GO terms was obtained from the National Center for Biotechnology Information Gene database (http://www.ncbi.nlm.nih.gov/gene). Associations between genes and pathways were assessed using Fisher’s exact test. Redundant concepts in the GO results were removed by comparing the overlapping portions between genes related to GO terms.

### Validation of AS events in thyroid cancer cell lines

Three thyroid cancer cell lines were used: TT2609-C02, FTC-133, and ML-1. RNA was extracted and reverse transcribed into cDNA using SuperScript™ IV VILO™ master mix (Thermo Fisher Scientific, USA). RT–PCR primers were designed using the Primer3 program^33^. The primer sequences are shown in Supplementary Table 1. RT–PCR was performed using KOD FX PCR master mix (TOYOBO, Japan). Amplified PCR products were purified from 3% agarose gels using the QIAquick gel extraction kit (Qiagen, Germany) and then subjected to Sanger sequencing. Sequences were aligned to a human reference genome (hg38) using the UCSC BLAT search tool^34^.

## Results

### Splicing events in molecular subtypes of PTC

**Fig. 1:**
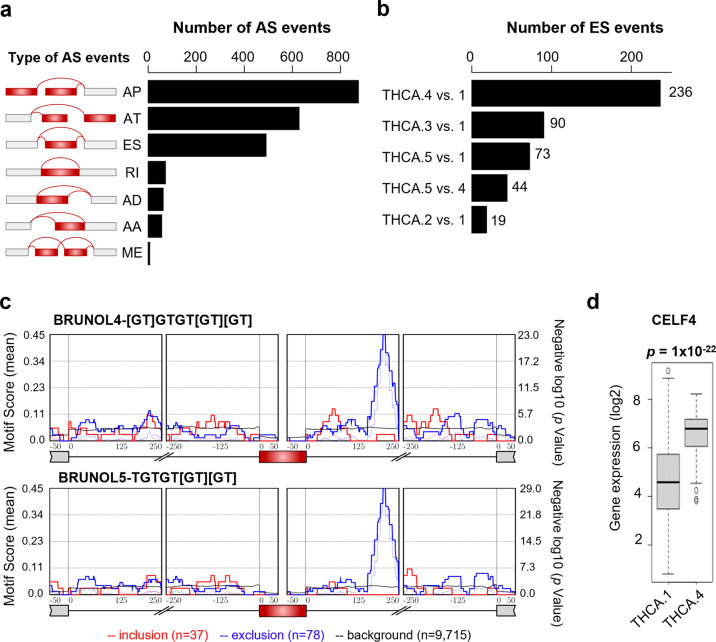
Overall features of alternative splicing (AS) events in molecular subtypes of papillary thyroid cancer (PTC). **a** Numbers of AS events that differed significantly among molecular subtypes of PTC (by ANOVA). AP alternate promoter, AT alternate terminator, ES exon skip, RI retained intron, AD alternate donor site, AA alternate acceptor site, ME mutually exclusive exons. **b** Top five molecular subtype pairs that exhibited differences in ES events (by Tukey’s test). **c** Motif enrichment profiles of BRUNOL4 and BRUNOL5 for significant ES events in THCA.1 vs. THCA.4. The X-axis indicates the intron position up/downstream of the target exon (red box). Solid red and blue lines indicate motif scores for 37 included and 78 excluded ES events between THCA.1 and THCA.4, respectively. **d** Comparison of *CELF4* mRNA expression levels between THCA.1 and THCA.4 cells. The *p* value was determined using the *t test*.

In this study, we explored whether AS events can serve as new molecular determinants for understanding the clinical implications of molecular and histological subtypes of PTC. Regarding molecular subtypes, we obtained information from an earlier TCGA marker paper in which PTCs were reclassified as five molecular subtypes (THCA.1–THCA.5) based on multidimensional analysis of mRNA, microRNA, DNA methylation, and protein profiles^5^. Histological subtype data are also available from TCGA. The overall workflow for the identification and characterization of AS events in thyroid cancer is shown in Supplementary Fig. 1. In the THCA dataset from TCGA^5^, 2,190 of 45,150 AS events were found to be significantly different among the five molecular subtypes of PTC [p < 0.0001 by ANOVA, ΔPSI > 0.2, and adjusted *R*^2^ > 0.1] (Supplementary Table 2). Of the 2,190 AS events, AP was the most common type, followed by AT and ES (Fig. 1a). Consistently, AP, AT, and ES were also the most common AS events that were significantly different among the histological subtypes of PTC (Supplementary Fig. 2a). Our analysis focused on 490 ES events that can occur solely by splicing regulation; AP and AT events are regulated by transcription initiation and termination, in addition to splicing. Of 490 ES events, 25% were thyroid-specific; the other 75% (369) were found to be universal across diverse cancers (Supplementary Fig. 2b). GO terms for actin filaments, cell adhesion, and cell junctions were significantly associated with ES events (Supplementary Fig. 2c). Among molecular subtypes, the THCA.1 vs. THCA.4 subtype pair exhibited differences in 48% of ES events (236/490), followed by THCA.1 vs. THCA.3 (90/490) and THCA.1 vs. THCA.5 (73/490) (Fig. 1b). Moreover, THCA.1 and THCA.4 molecular subtypes showed the largest differences in histological subtype and thyroid differentiation (Supplementary Fig. 2d and 2e).

Of the ES events detected in THCA.1, 37 and 78 events were included and excluded in THCA.4 transcripts, respectively (*p* < 0.001 by the *t test* and ΔPSI > 0.15) (Supplementary Table 3). To predict upstream splicing regulators for ES events, we examined their binding sites and found high enrichment for GT-rich sequence motifs at positions 125–250 of the downstream introns of the 78 ES events excluded in THCA.4 (Fig. 1c). The top two significant motifs are for binding of the proteins BRUNOL4 and BRUNOL5 (Supplementary Table 4), which are encoded by CUGBP Elav-like family member (*CELF*) 4 and 5 genes, respectively. Accordingly, expression of the *CELF4* gene was significantly different between the THCA.1 and THCA.4 subtypes (fold change > 2 and *p* < 10^−20^ by the *t test*) (Fig. 1d).

### Correlation of ES events with biological features of thyroid cancers

When we examined the potential involvement of ES events in the biological features of PTC, such as TDS, ERK and RAS oncogenic activity scores, extrathyroidal extension, and lymph node metastasis, 216 of 490 were matched with functional annotation information (Supplementary Table 5). Notably, 85% of the ES events (184/216) had posttranslational modification sites, which suggested functional implications in protein synthesis. Furthermore, 23 ES events exhibited significant correlations with the TDS, as well as ERK and RAS oncogenic activity scores (Pearson correlation, *r* > 0.5 or < −0.5 in biological scores and *p* < 0.001 by the *t test* in clinical outcomes) (Table 1).

**Table 1:** Twenty-three ES events showing significant correlations with the TDS, ERK score, or RAS score.

Gene symbol	AS ID	Exon	TDS		ERK score		RAS score	
			*r*^a^	*p* value	*r*	*p* value	*r*	*p* value
*NUMA1*	17515	18	−0.54	7E − 40	0.50	9E − 33	−0.62	4E − 52
*LSR*	49086	5	0.53	6E − 37	−0.59	5E − 49	0.49	9E − 30
*TUBB3*	38175	6.1:6.2	0.52	1E − 32	−0.45	8E − 24	0.62	3E − 47
*KIAA1217*	11009	12	0.58	3E − 46	−0.29	3E − 11	0.55	2E − 38
*ARPC1B*	80609	8	0.47	1E − 28	−0.52	1E − 36	0.58	6E − 45
*CACNB3*	21476	9	−0.58	3E − 44	0.32	4E − 13	−0.55	2E − 38
*SSBP3*	3144	7	0.57	2E − 43	−0.39	7E − 20	0.41	8E − 21
*DYSF*	53937	19	−0.54	3E − 33	0.41	2E − 18	−0.61	1E − 42
*LUC7L*	32844	4	0.38	7E − 19	−0.55	9E − 41	0.40	2E − 19
*CADM1*	18851	10	0.44	1E − 25	−0.45	3E − 26	0.55	3E − 39
*SSBP4*	48431	7	−0.53	2E − 38	0.42	9E − 23	−0.50	7E − 32
*TBC1D15*	23415	10	0.54	3E − 38	−0.41	1E − 20	0.46	1E − 25
*ITGB4*	43489	35	0.52	4E − 36	−0.37	1E − 17	0.52	2E − 34
*PCYT2*	44230	7	−0.47	3E − 29	0.51	3E − 35	−0.37	1E − 16
*EXOC7*	43569	7	0.41	2E − 21	−0.50	2E − 33	0.52	5E − 35
*SH3BP1*	62140	16	0.48	9E − 30	−0.47	2E − 29	0.52	3E − 34
*MROH1*	85546	26	−0.50	5E − 33	0.32	2E − 13	−0.52	3E − 34
*ARHGAP17*	35664	18	0.35	3E − 16	−0.51	4E − 34	0.38	1E − 17
*OSBPL3*	79027	9	0.52	4E − 34	−0.45	1E − 25	0.51	1E − 31
*UNC5B*	12054	8	−0.52	4E − 31	0.43	1E − 20	−0.55	1E − 33
*CCNDBP1*	30220	10.1	0.47	2E − 28	−0.38	8E − 19	0.50	7E − 32
*ABLIM1*	13209	16	0.50	3E − 31	−0.37	5E − 17	0.42	5E − 20
*SYTL2*	18153	11.2	0.48	3E − 25	−0.43	3E − 20	0.50	4E − 27

^a^Pearson’s correlation coefficient.

### Correlation of an ES event for exon 18 of NUMA1 with biological features of PTC

**Fig. 2:**
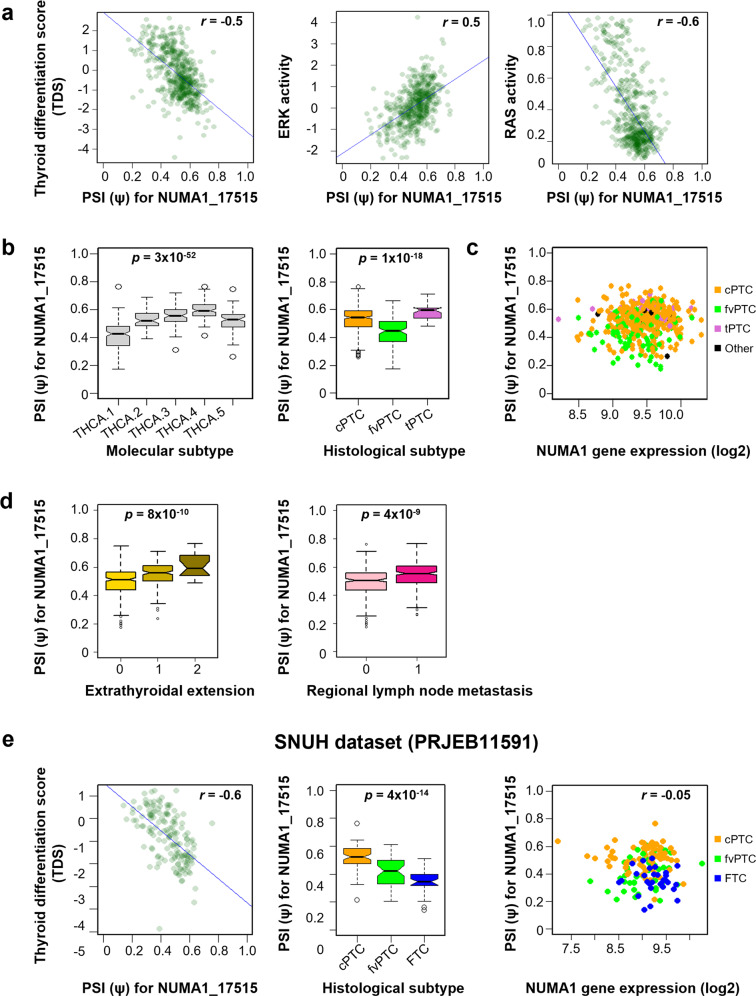
Association of the NUMA1_17515 exon skipping event with biological features of papillary thyroid cancer (PTC). **a** Correlations of the NUMA1_17515 event with thyroid differentiation scores (TDSs) and ERK and RAS activity scores. Each dot represents an individual patient sample. Pearson’s correlation coefficient (*r*) and the linear regression line are shown for each plot. **b** Percent spliced-in (PSI) values for molecular (left) and histological (right) subtypes. The p values were determined by ANOVA. **c** Correlation between NUMA1_17515 PSI values and *NUMA1* expression levels. Histological subtypes are indicated by different colors. **d** Association of NUMA1_17515 PSI values with progression of extrathyroidal extension (left) and regional lymph node metastasis (right). Extrathyroidal extension: 0 = none; 1 = minimum (T3); 2 = advanced (T4a and T4b). Regional lymph node metastasis: 0 = negative; 1 = positive. **e** Correlations of the NUMA1_17515 event with TDSs, histological subtype, and *NUMA1* expression level in the Seoul National University Hospital (SNUH) thyroid cancer dataset.

NUMA1_17515, an ES event in exon 18 of the nuclear mitotic apparatus protein 1 (*NUMA1*) gene, exhibited the strongest correlation with both the TDS (*r* = −0.5) and oncogenic activities (*r* = 0.5 and *r* = −0.6 for ERK and RAS scores, respectively) (Table 1 and Fig. 2a). The PSI values of NUMA1_17515 were significantly different among molecular subtypes (*p* = 3 × 10^−52^), being lowest in THCA.1 and highest in THCA.4 (Fig. 2b, left). The PSI values of this ES event were also differed significantly among histological subtypes (*p* = 1 × 10^−18^) (Fig. 2b, right). However, NUMA1_17515 did not correlate with the *NUMA1* expression level (*r* = 0.06) (Fig. 2c), and *NUMA1* expression did not show any correlation with the TDS and ERK and RAS activity scores (Supplementary Fig. 3a), indicating that the correlation of this ES event with biological features is independent of gene expression. Regarding progression of THCA, NUMA1_17515 was significantly associated with extrathyroidal extension (*p* = 8 × 10^−10^) and lymph node metastasis (*p* = 4 × 10^−9^) (Fig. 2d). Consistent results were obtained using an independent Korean thyroid cancer dataset (PRJEB11591), including a strong correlation with the TDS (*r* = −0.6), a significant difference among histological subtypes (*p* = 4 × 10^−14^), and no correlation with gene expression levels (*r* = −0.05) (Fig. 2e). Notably, the PSI values of fvPTC were intermediate between those of cPTC and FTC, consistent with the intermediate features of fvPTC, as previously reported^3^.

**Fig. 3:**
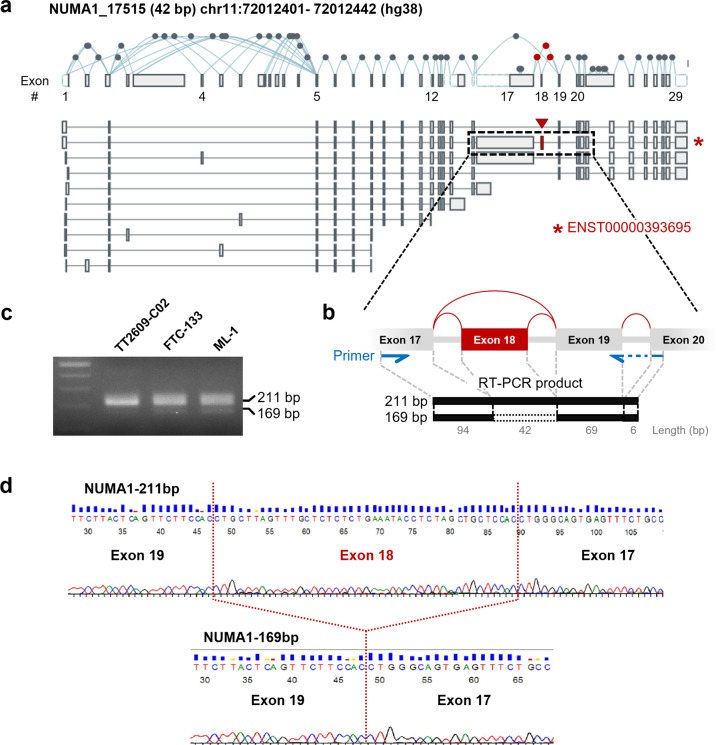
Validation of the NUMA1_17515 exon-skipping event in thyroid cancer cell lines. **a** Isoforms of the *NUMA1* gene in the exon expression panel of the GTEx portal. The location of NUMA1_17515 is marked by a red arrowhead (chr11:72012401–72012442, hg38). Only one transcript (ENST00000393695, red asterisk) had an NUMA1_17515-regulated exon. Exon numbers below the *NUMA1* gene structure are in accordance with TCGA SpliceSeq. **b** Primers were designed to amplify sequences in neighboring exons of exon 18 (blue arrows). The exon targeted by NUMA1_17515 is colored in red. A primer (right) across exons 19 and 20 was designed. Numbers below each exon represent sizes produces using the primer set. The expected lengths of the RT–PCR products are 169 and 211 bp, excluding or including exon 18 (42 bp), respectively. **c** Gel electrophoresis of RT–PCR products from three thyroid cancer cell lines (TT2609-C02, FTC-133, and ML-1). **d** Confirmation of the inclusion of exon 18 in the 211-bp amplicon and its exclusion from the 169-bp amplicon based on Sanger sequencing.

Exon 18, which is regulated by NUMA1_17515, is 42 base pairs (bp) in length, and only one transcript (ENST00000393695) is known to harbor this exon (Fig. 3a). To confirm the occurrence of the NUMA1_17515 ES event in thyroid cancer, we performed RT–PCR and observed two bands of the expected sizes (211 and 169 bp) across the tumor cell lines tested (Fig. 3b, c), and DNA sequencing confirmed the presence of exon 18 in the 211-bp amplicon and its exclusion in the 169-bp amplicon (Fig. 3d).

### Discrimination of molecular subtypes of PTC by ES and IR events for the TUBB3 gene

**Fig. 4:**
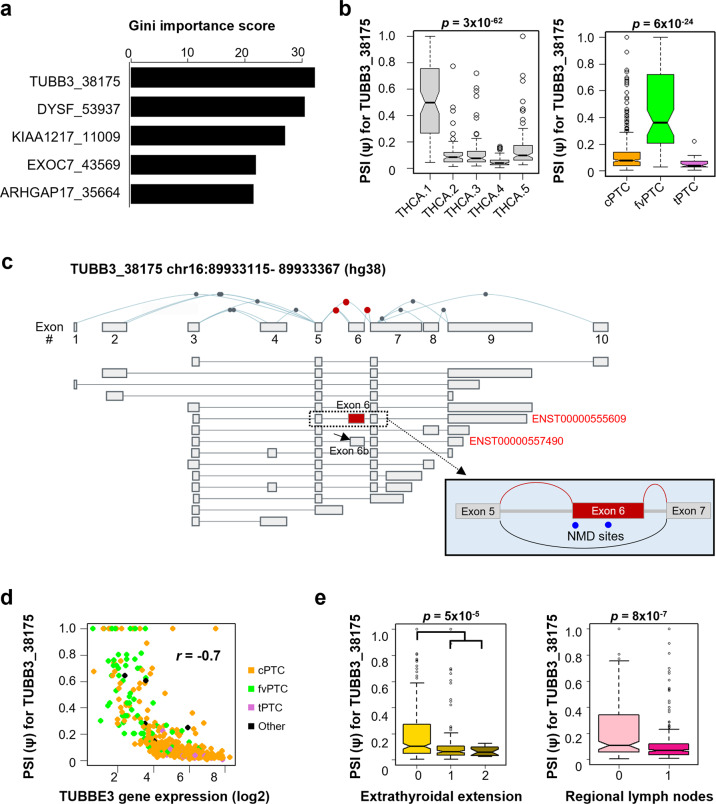
TUBB3_38175 exon skipping (ES) event as a classifier for molecular subtypes of papillary thyroid cancer (PTC). **a** Random forest variable importance plot showing significant ES events for classification using the Gini importance score as an indicator of the subtype-discriminating ability. **b** TUBB3_38175 percent spliced-in (PSI) value profiles for molecular (left) and histological (right) subtypes. The *p* values were determined by ANOVA. **c** Isoforms of the *TUBB3* gene from the exon expression panel of the GTEx portal. The location of TUBB3_38175 is marked by the red box (chr11:72012401–72012442, hg38) (ENST00000555609). Sequence-based annotation of exon 6 in the ASpedia database indicates the sites of nonsense-mediated decay (NMD) (blue dots). The transcript with a shorter form of exon 6 (exon 6b) is indicated by a solid arrow (ENST00000557490). **d** Correlation between TUBB3_38175 PSI values and *TUBB3* expression levels. Histological subtypes are indicated by different colors. **e** Association of TUBB3_38175 PSI values with progression of extrathyroidal extension (left) and regional lymph node metastasis (right).

To further explore ES events as a classifier for PTC subtype, we performed random forest classification analysis with the 23 ES events and observed which was able to discriminate the molecular subtypes of PTC (Supplementary Table 6). According to the Gini importance score, TUBB3_38175, an ES event for the tubulin beta 3 class III (*TUBB3*) gene, was the top-ranked event (Fig. 4a). The PSI values for TUBB3_38175 were significantly different among molecular subtypes (*p* = 3 × 10^−62^), being highest in THCA.1 (Fig. 4b, left). The differences among histological PTC subtypes were also significant (*p* = 6 × 10^−24^), with the highest value found in fvPTC (Fig. 4b, right). A transcript (ENST00000555609) that is known to harbor this exon contains two NMD sites according to ASpedia annotation^22^ (Fig. 4c). Among the NMD factors tested (*UPF1*, *UPF2*, *UPF3A*, and *UPF3B*)^35^, TUBB3_38175 correlated positively with expression of the *UPF3A* and *UPF3B* genes (Supplementary Fig. 4a), which supports the hypothesis that exon 6 is an NMD target. Another transcript (ENST00000557490) is also known to harbor part of exon 6, and it is 24 bp shorter (hereafter referred to as exon 6b) than exon 6 in the ENST00000555609 transcript^23^ (Fig. 4c). PSI values for TUBB3_38175 correlated negatively with gene expression levels (*r* = −0.7), indicating that this ES event plays a role in the regulation of *TUBB3* gene expression (Fig. 4d). Notably, PSI values for TUBB3_38175 were associated with the progression of thyroid cancers, whereby lower values were significantly associated with advanced extrathyroidal extension (*p* = 5 × 10^−5^) and regional lymph node metastasis (*p* = 8 × 10^−7^) (Fig. 4e).

**Fig. 5:**
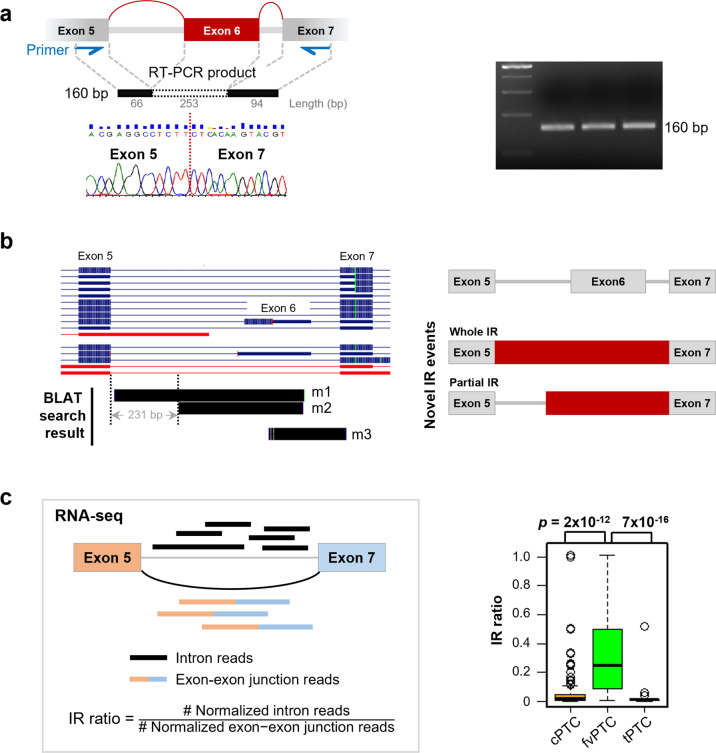
Validation of exon-skipping events and identification of novel intron retention (IR) events in *TUBB3*. **a** Validation of exon 6 skipping. Primers were designed to amplify sequences in neighboring exons of exon 6 (blue arrows). The exon targeted by TUBB3_38175 is colored in red. Numbers below each exon represent sizes from the primer. Agarose gel electrophoresis of RT–PCR products from three thyroid cancer cell lines showed a transcript of the expected size (160 bp), which was confirmed by Sanger sequencing. **b** Identification of new IR events between exons 5 and 7 of the *TUBB3* gene (details available in Supplementary Additional file 1). Sequence alignment of three RT–PCR amplicons (m1–3) to the human genome (hg38) confirmed two types of novel IR events between exons 5 and 7, namely, retention of a full-length intron and an intron that was 231 bp shorter. For GENCODE transcripts (left), blue lines indicate coding transcripts, and red lines display problem transcripts. In the right plots, red colors indicate IR regions. **c** IR ratios among papillary thyroid cancer (PTC) histological subtypes. The *p* values were calculated using the *t test*.

Next, we validated the occurrence of TUBB3_38175; details of the validation strategies and findings are provided in the Supplementary text. In brief, skipping of exon 6 was confirmed by RT–PCR between neighboring exons, and sequencing of the products was performed (Fig. 5a). Inclusion of exon 6 (253 bp) and exon 6b (229 bp) was also confirmed (Supplementary Fig. 5).

Interestingly, in addition to ES, novel IR events were discovered between exons 5 and 7 of *TUBB3* (Fig. 5b and Supplementary Figs. 5–7). In one IR event, a full-length intron was retained; in the other, the intron retained was 231 bp shorter. To verify that these IR events occurred in thyroid cancer samples, the IR ratio, which represents the relative abundance of intron reads to exon 5/exon 7 junction reads, was calculated as previously described^32^ using TCGA RNA-seq data (Fig. 5c, left). Among PTC subtypes, the IR ratio for *TUBB3* was significantly higher in fvPTC than in cPTC and tPTC (*p* < 10^−10^) (Fig. 5c, right).

**Fig. 6:**
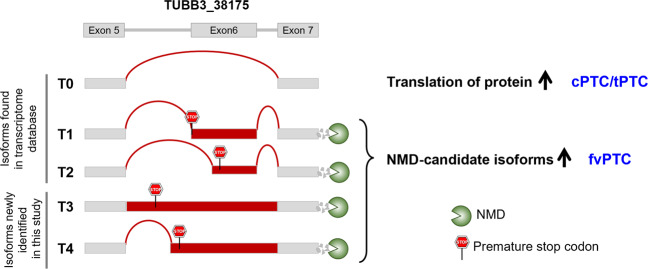
Model of dynamic alternative splicing regulation of the *TUBB3* gene. Five kinds of transcripts (T0–T4) were identified between exon 5 and exon 7. Three (T0, T1, and T2) were generated by exon-skipping events, and two (T3 and T4) were produced by intron-retention events. Red boxes represent unique sequences compared with that of the T0 transcript. The positions of premature stop codons are shown as red stop signs. The green Pac-Man denotes isoforms subject to nonsense-mediated decay (NMD). Only the T0 transcript is predicted to avoid NMD, whereas the others are likely to be subjected to transcriptional degradation due to premature stop codons.

Taken together, these data support a model of dynamic AS regulation of the *TUBB3* gene (Fig. 6), in which five transcripts (T0–T4) of varying lengths may be spliced between exons 5 and 7. T0 is a transcript with exon 6 skipped, the T1 and T2 transcripts the inclusion of exon 6 or 6b, respectively, and the T3 and T4 transcripts are products of IR events. Many premature stop codons between exons 5 and 7 were detected (Supplementary Fig. 6). Accordingly, four transcripts (T1–T4) containing premature stop codons can undergo transcriptional degradation via NMD, though the T0 transcript avoids NMD and serves as a template for protein production.

## Discussion

Recent studies exploring global splicing changes in association with the prognosis of PTC^8,9^ have provided evidence for widespread AS events and their implications for PTC prognosis. Nevertheless, no data on AS events associated with the molecular/histological subtypes and oncogenic activities of PTC are available. In this study, we found 23 distinct ES events associated with the oncogenic activities of PTC and two ES events, NUMA1_17515 for *NUMA1* and TUBB3_38175 for *TUBB3*, that discriminated the histological and molecular subtypes of PTC. Our data indicate that the histological or molecular subtype alone is not sufficient for the subclassification of PTC and that AS events may serve as a PTC classifier.

Among PTC molecular subtypes, THCA.1 and THCA.4 showed the largest differences in ES events. Of note, THCA.1 and THCA.4 consisted of mixed and contrasting histological subtypes; THCA.1 had the highest proportion of fvPTC and the lowest proportion of tPTC, whereas THCA.4 had the lowest proportion of fvPTC and the highest proportion of tPTC. In addition, both subtypes showed the largest differences in their TDSs. These data suggest that the ES events identified in this study may lead to phenotypic differences underlying functional characteristics and histological differentiation in PTC.

Of 216 functionally annotated ES events, 23 correlated significantly with the TDS and ERK and RAS activity scores. TDSs have been used to assess thyroid differentiation levels, which are important for histological subtyping. Although *BRAF* and *RAS* are mitogen-activated protein kinase-related genes and mutations in them are the most common somatic alterations in PTCs^36^, these mutations are mutually exclusive^37^. Therefore, we adopted the ERK score for activation of the ERK transcriptional program and the RAS score for RAS pathway activation^5,30^. However, we were unable to implicate molecular functions (TDS and ERK and RAS activity scores) with ES events for patient survival and distant metastasis because these events are too rare in PTC to enable a reliable interpretation. Instead, as an indicator of aggressiveness, correlations of ES events with extrathyroidal extension and regional lymph node metastasis were found, indicating that ES events might be related to the biological behaviors of PTC and possibly to clinical outcomes.

Of the 23 ES events that correlated significantly with oncogenic activity and differentiation stage, the most significant was NUMA1_17515 in exon 18 of the *NUMA1* gene. The exon regulated by NUMA1_17515 is 42 bp in length; its inclusion is predicted to lead to a gain of 14 amino acid residues. The NUMA1 protein is a structural component of the nuclear matrix, which is involved in mitotic spindle formation during cell division by interacting with microtubules^38,39^. NUMA1 is overexpressed in ovarian cancer, and its upregulation correlates with increased mitotic defects and aneuploidy^40^, suggesting that this protein possesses oncogenic functions. NUMA1_17515 has been reported to promote mammary epithelial cell proliferation and tumorigenesis^41^. In this study, NUMA1_17515 did not correlate with *NUMA1* expression levels, which indicates that this ES event might contribute to PTC tumorigenesis via a mechanism unrelated to *NUMA1* expression. When we examined the subtype-specific regulation of NUMA1_17515 in other TCGA cancers, it was also detected in other cancers, including breast and esophageal carcinomas (Supplementary Fig. 3b). In addition, this ES event showed different patterns of expression during organ development (Supplementary Fig. 3c), which suggests that NUMA1_17515 might play a fundamental role in the development of diverse organs. The correlation between NUMA1_17515 PSI values and levels of thyroid differentiation was also observed in an independent Korean dataset (PRJEB11591), which suggests that the NUMA1_17515 ES event may play a role in PTC phenotypes by affecting thyroid differentiation.

The TUBB3_38175 ES event for the *TUBB3* gene was predicted to be the most significant classifier for PTC subtype. The incidence of TUBB3_38175 was also significantly different between molecular subtypes of other cancers, including renal cell carcinoma and head and neck carcinoma (Supplementary Fig. 4b). Previous studies have reported that upregulation of *TUBB3* expression is common and associated with aggressiveness in many cancers, including thyroid cancer^42–45^. For example, *TUBB3* gene expression was used to predict invasive potential in association with the epithelial–mesenchymal transition in thyroid cancer^45^. In our study, TUBB3_38175 correlated significantly with expression of *TUBB3*, suggesting that this ES event might play a role as a regulator of gene expression. Indeed, our data showed higher levels of the exon 5–exon 7 junction, leading to upregulation of the *TUBB3* gene, in cPTC and tPTC, which have less favorable clinical outcomes, than in fvPTC, which has more favorable outcomes^46^.

In this study, we uncovered novel IR events between exons 5 and 7 of *TUBB3* through experimental validation. Except for the T0 transcript, the other transcripts (T1–T4) harbored premature stop codons, which might result in NMD. NMD, often coupled with AS, is a quality control mechanism that decreases the abundance of mRNA transcripts carrying a premature stop codon^47^. The positive correlations between TUBB3_38175 and expression levels of NMD factors (*UPF3A* and *UPF3B*) found in this study support the possibility of AS-NMD coupling for the *TUBB3* gene. Although we identified complex patterns of AS of the *TUBB3* gene, leading to a possible loss of its expression due to NMD in PTC, the roles of these patterns in tumorigenesis in thyroid cancers and the clinical implications remain to be further elucidated.

Our study has several limitations. First, the identified AS-based subclassifiers for histological/molecular subtypes may not constitute a complete classification system for PTC. Thus, integrated analysis of AS and histological/molecular subtypes of PTC in a larger cohort of patients with detailed clinical information is required. Second, because RNA-seq only measures transcript levels, without analyzing actual cellular phenotypes, we could only infer the functional implications of AS events. Although our data suggest heterogeneity among PTC subsets, the functional impacts of AS profiles require further investigation.

In summary, this study provides a landscape of AS changes among PTC subtypes and identifies two significant AS events, NUMA1_17515 and TUBB3_38175, as potential AS biomarkers for PTC subclassification and characterization. Our findings indicate that the histological or molecular subtype alone is not sufficient for subclassification of PTC and that AS events may be additional classifiers.

## Supplementary information

Supplementary Information
