# Supplementary material for: Dissection of molecular and histological subtypes of papillary thyroid cancer using alternative splicing profiles
Source: Exp Mol Med. 2022 Mar 11;54(3):263–72. doi: 10.1038/s12276-022-00740-0 (PMC8980103; doi:10.1038/s12276-022-00740-0)
Supplement: Supplementary file 1 — Supplementary Information [file 12276_2022_740_MOESM1_ESM.pdf]

Dissection of molecular and histological subtypes of papillary thyroid cancer using alternative splicing profiles

Supplementary Text

Validation of TUBB3 exon skipping events and discovery of intron retention events

To validate the occurrence of TUBB3_38175 (exon 6), we designed a primer set at neighboring exons. In case of the skipping of exon 6, the expected length of the reverse transcription–polymerase chain reaction (RT-PCR) product was 160 bp (Fig. 5a, left). In case of the presence of exon 6 or 6b, the length will be 413 bp or 389 bp, respectively (Supplementary Fig. 5a). When we performed RT-PCR, the expected 160 bp sized amplicon band was clearly detected (Fig. 5a, right). The ES of exon 6 was confirmed by Sanger sequencing (Fig. 5a, bottom). However, the expected bands representing exon 6 or 6b (413 bp or 389 bp bands) were not detected. Since the failure of detecting exon 6 containing amplicons would be due to relatively low expression of the transcripts, we designed a primer inside the exon 6 to verify the existence of exon 6: expected amplicon lengths was 201 bp (Supplementary Fig. 5b). Indeed, expected 201 bp amplicon representing exon 6 inclusion was detected by RT-PCR (Supplementary Fig. 5b). Sanger sequencing of the 201 bp product revealed existence of exon 6 without intron sequence (Supplementary Fig. 5b). Notably, in addition to the 201 bp band, another amplicon which is ~100 bp longer than the expected one was also detected. Sanger sequencing of the 301 bp product revealed existence of exon 6 with retention of the intron between exon 6 and 7 (Supplementary Fig. 5b).

Subsequently, we checked the rest part of the transcript by designing the primers between exon 5 and exon 6: in the presence of exon 6 or 6b, 261 or 237 bp amplicon will be

produced, respectively (Supplementary Fig. 5c). When we performed RT-PCR, we observed more complex alternative splicing events than expectation. As expected, two amplicons around 250 bp were detected and turned out to have the sequence of exon 6 and 6b (Supplementary Fig. 5c). Interestingly, two more bigger sized amplicon bands were also detected (Supplementary Fig. 5c). The longest amplicon (696bp) was found to be the product of intron retention (IR) (BLAT search result in Supplementary Fig. 6). To confirm this, we designed the primer at exon 5-intron junction and exon 7 and confirmed the whole IR between exon 5 and exon 7 by Sanger sequencing (Supplementary Fig. 7a). Sanger sequencing of the second largest band revealed partial IR lacking 5' end of the intron (BLAT search result in Supplementary Fig. 6). To further verify the structure of the partial IR, we designed a primer set at the expected IR junction and the intron between exon 5 and 7 (Supplementary Fig. 7b). When we performed RT-PCR, we detected 571 bp amplicon and confirmed the partial IR lacking 231 bp at the 5' end of intron (Supplementary Fig. 7b). All amplicon bands in Supplementary Fig. 5a and 5b were sequenced and visualized in UCSC genome browser using UCSC BLAT search (Supplementary Fig. 6). Sequence information of all the primers used in this study is available in Supplementary Table 1.

Supplementary Figures

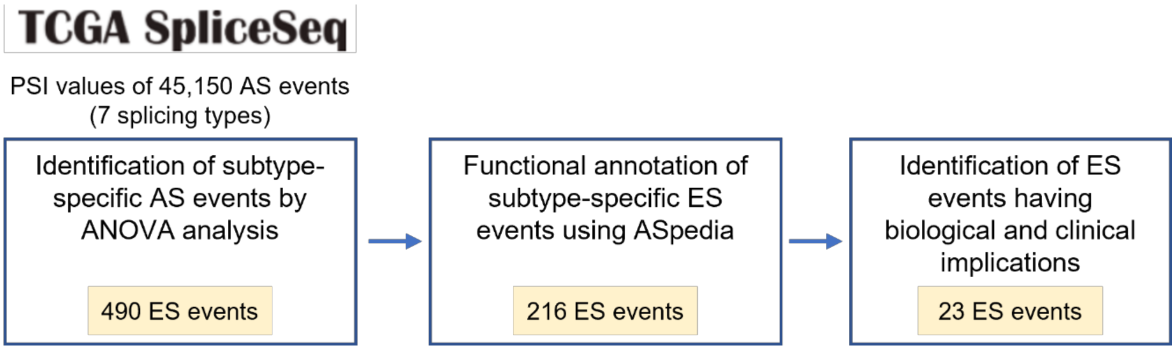

Supplementary Fig. 1. Workflow to identify AS events that could allow further classificationof PTC and their functional implication.

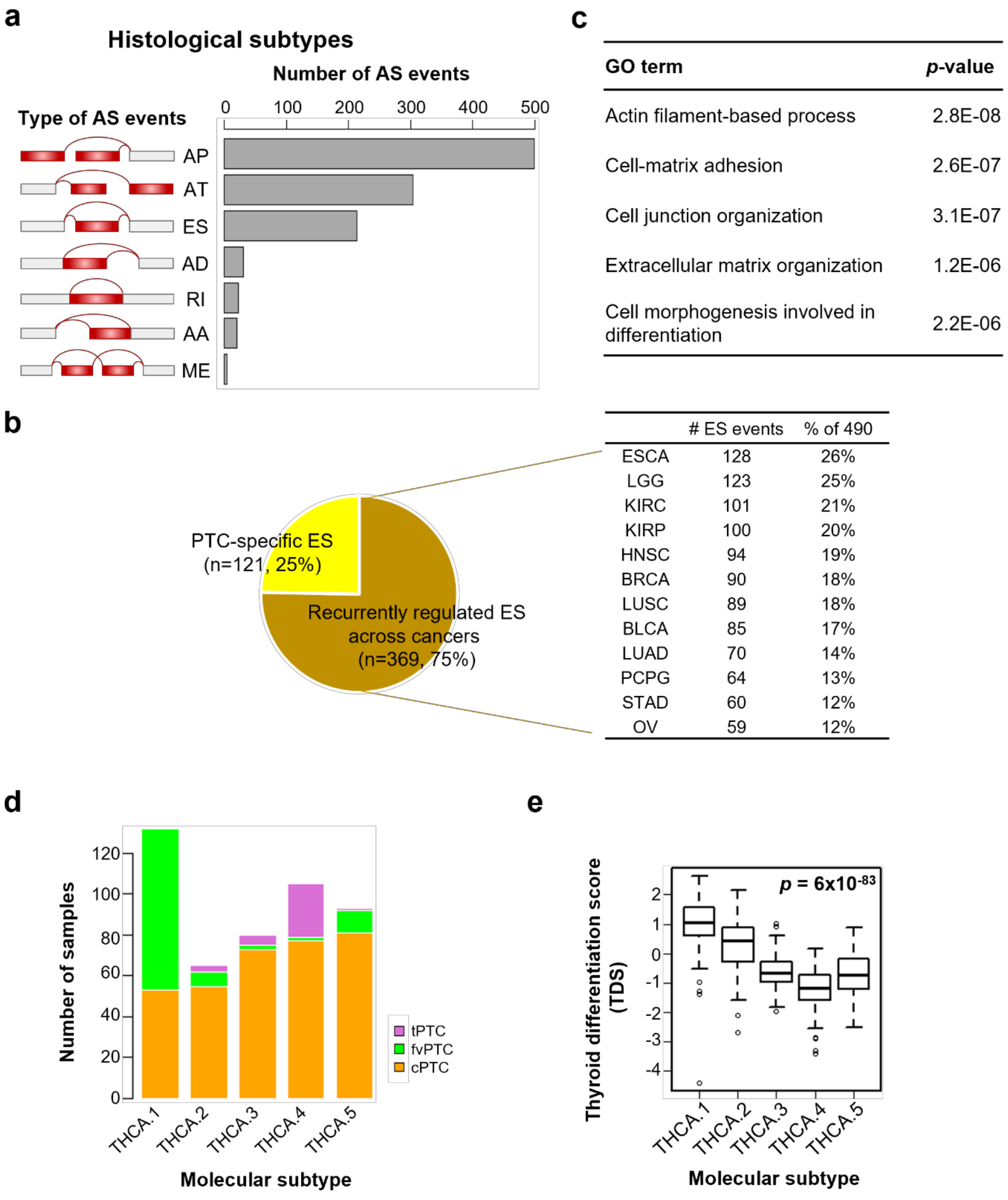

Supplementary Fig. 2. Analysis of subtype-specific AS in papillary thyroid cancer (PTC). **a** Numbers of AS events that were significantly different between histological subtypes. The events were selected using analysis of variance (ANOVA). The threshold for the selection was p < 0.0001 and adjusted R^2> 0.05. AP, alternate promoter; AT, alternate terminator; ES, exon skip; RI, retained intron; AD, alternate donor site; AA, alternate acceptor site; and ME, mutually exclusive exons. **b** Relative fraction of the recurrence in other cancer types among 490 molecular subtype-specific ES events. A table on the right shows the number of events sharing with other cancer type and the fraction. **c** Significant Gene Ontology (GO) terms

associated with genes having significant ES changes. Top 5 GO terms were shown in the category of biological pathways. **d** Relationship of molecular subtypes with histological subtypes in TCGA PTC data. X-axis indicates molecular subtypes and y-axis shows the number of samples. Each bar is separated by histological subtypes, which are marked by color. **e.** Comparison of thyroid differentiation scores (TDSs) between PTC molecular subtypes. The p-value on the plot was determined using ANOVA.

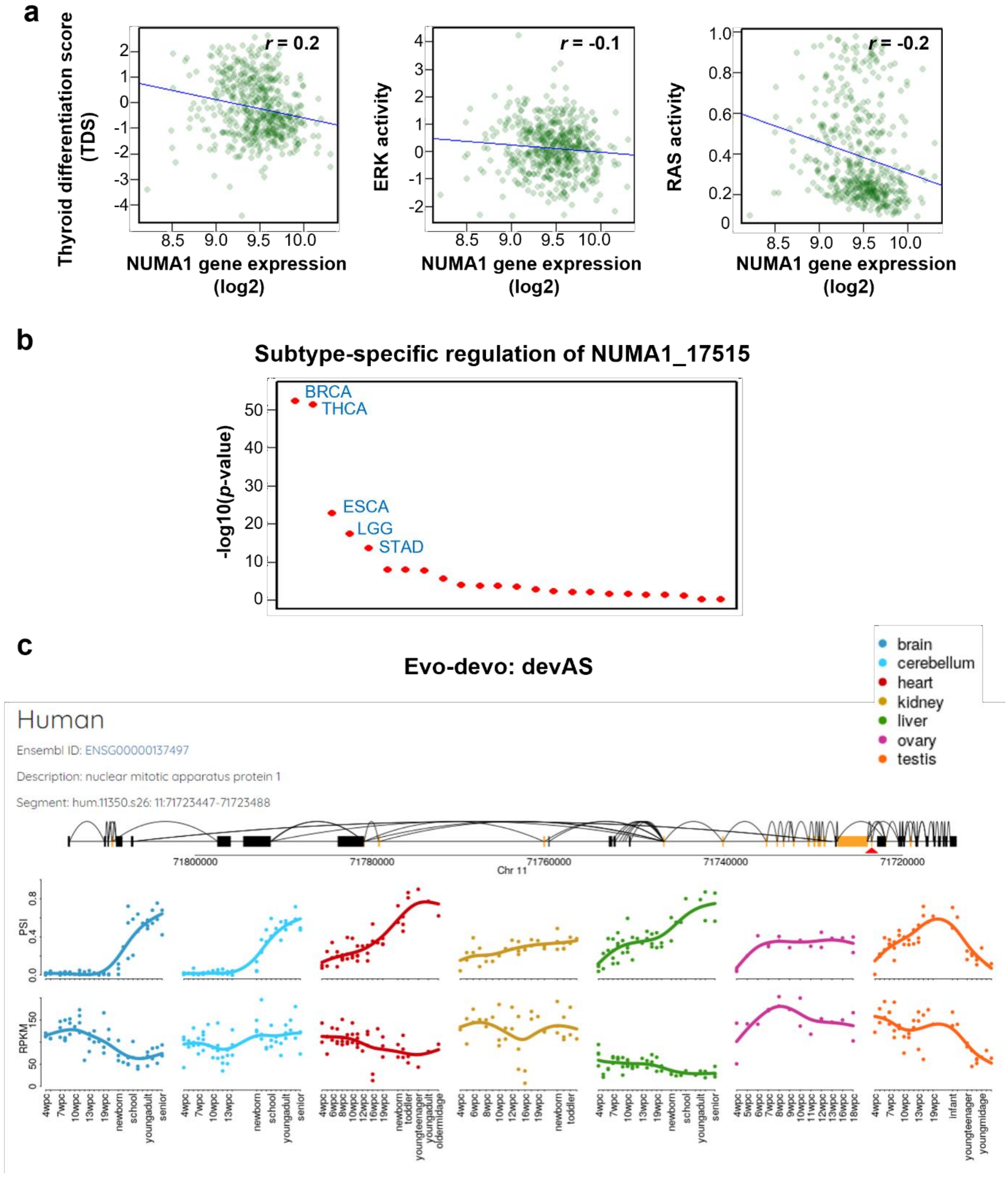

Supplementary Fig. 3. Correlation of NUMA1_17515 with biological features of PTC. **a** Correlation of *NUMA1* gene expression with TDS, ERK activity, and RAS activity. X-axis indicates gene expression value and y-axis indicates scores of each feature. Pearson correlation coefficients were shown on the top right. **b** Subtype-specific regulation of NUMA1_17515 across diverse cancers. Total 24 cancer types were analyzed. The p-values from ANOVA were converted into -log10(p-value). The five most significant cancer types are labelled (blue). BRCA: Breast invasive carcinoma, THCA: Thyroid carcinoma, ESCA: Esophageal carcinoma, LGG: Brain Lower Grade Glioma, STAD: Stomach adenocarcinoma. **c** Visualization of NUMA1_17515 in developmental AS atlas database

(https://apps.kaessmannlab.org/alternative-splicing/)[1]. The location of NUMA1_17515 was marked by red arrow in gene structure. Upper panel indicates developmental changes of percent spliced in (PSI) values and lower panel shows gene expression changes across seven human organs (brain, cerebellum, heart, kidney, liver, ovary, testis).

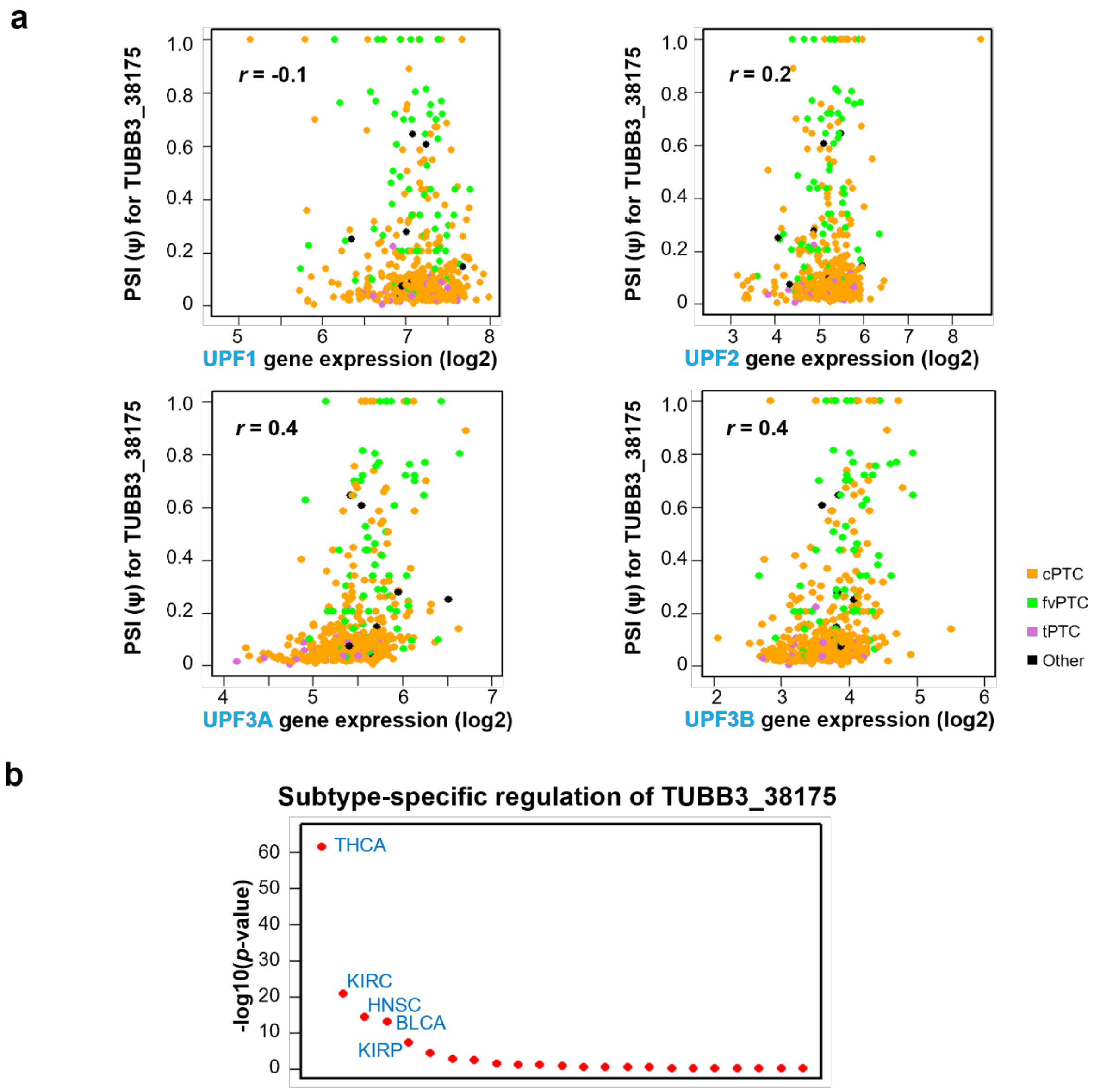

Supplementary Fig. 4. Characteristics of TUBBE3_38175. **a** Correlation analysis of TUBB3_38175 with NMD factor genes (*UPF1*, *UPF2*, *UBF3A* and *UBF3B*). Pearson correlation coefficients are displayed for each plot. **b** Subtype-specific regulation of TUBBE3_38175 across diverse cancers. The five most significant cancer types are labelled (blue). THCA: Thyroid carcinoma, KIRC: Kidney renal clear cell carcinoma, HNSC: Head and Neck squamous cell carcinoma, BLCA: Bladder Urothelial Carcinoma, KIRP: Kidney renal papillary cell carcinoma.

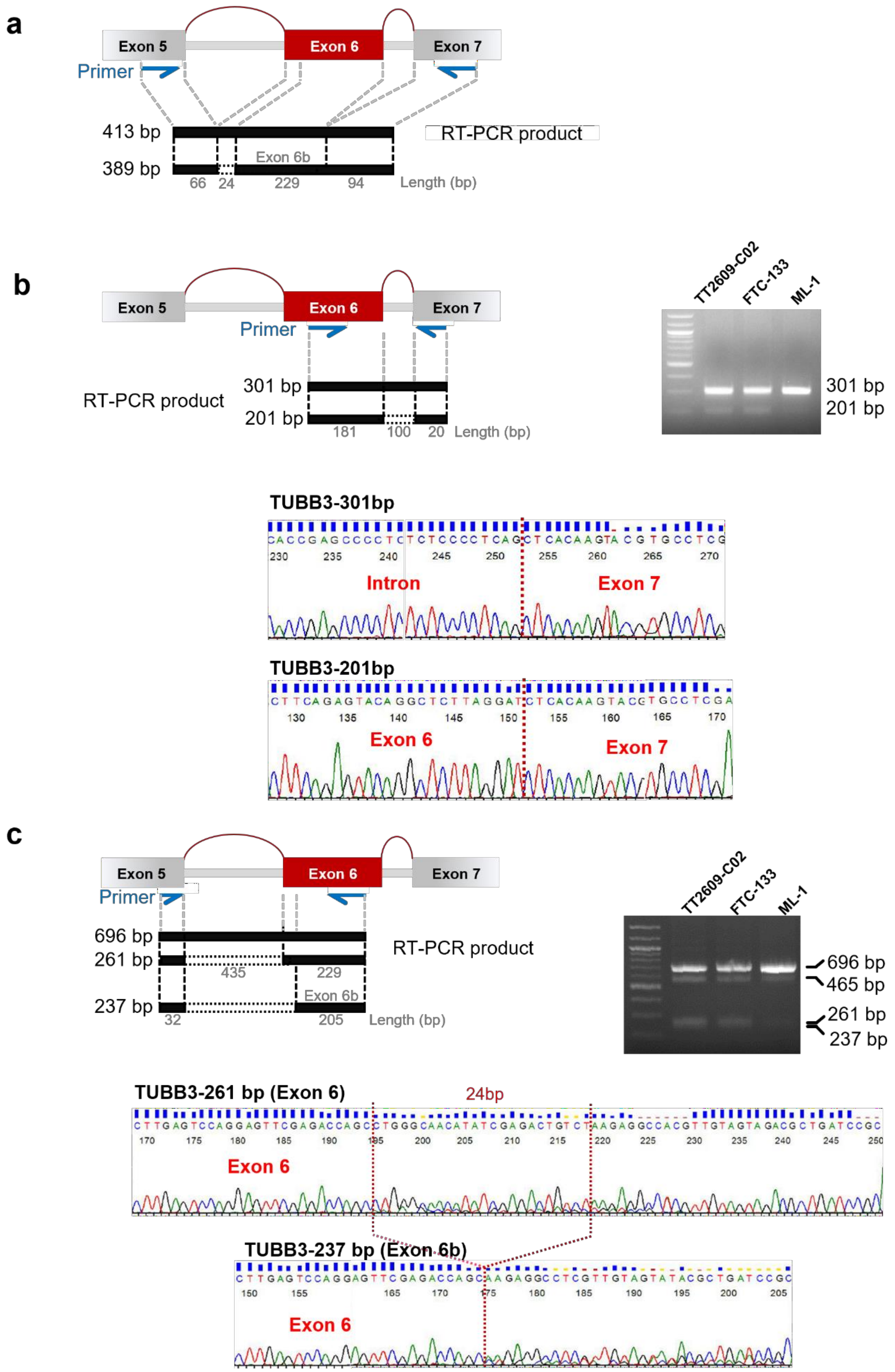

Supplementary Fig. 5. Validation of *TUBB3* exon skipping events and discovery of intron

retention events. **a** The expected sizes of PCR products in the presence of exon 6 or exon 6b. The length of exon 6b is 229 bp, 24 bp shorter than the one of exon 6 (253 bp). Primers were designed to amplify sequences in neighboring exons of exon 6 (blue arrows). Boxes indicate exons and lines indicate introns. The exon targeted by TUBB3_38175 is colored in red. Numbers below each exon represent sizes from the primer. **b** Primers were designed in exon 6 and exon 7. Location of a primer set and the expected PCR products are on the left, and the gel electrophoresis image of RT-PCR products is on the right. The Sanger sequencing results of gel purified RT-PCR amplicons were on the bottom. Two amplicons (201 bp and 301 bp) were detected and sequenced. **c** Primers were designed in exon 5 and exon 6. Four amplicons were found. Sanger sequencing results for two amplicons (237 bp and 261 bp) shown. 261 bp amplicon contains exon 6 and 237 bp (24 bp shorter than exon 6) amplicon contains exon 6b.

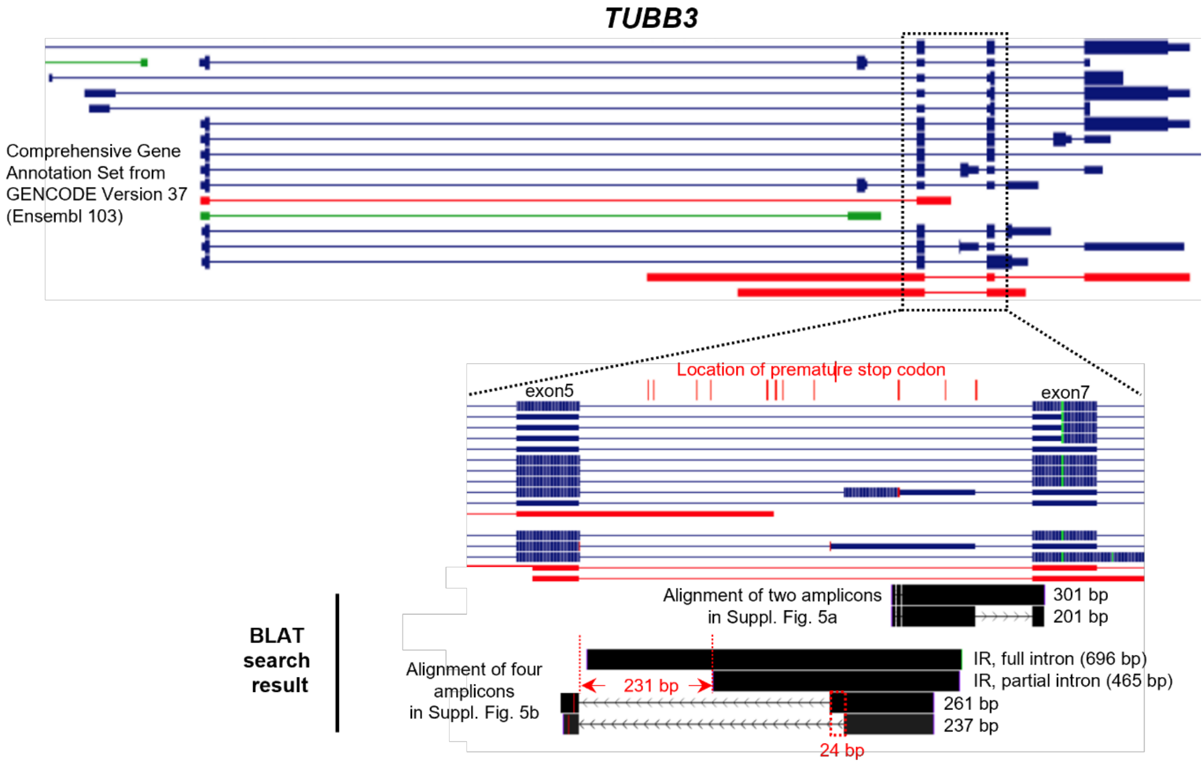

Supplementary Fig. 6. BLAT analysis for the amplicons shown in Supplementary Fig. 5b and 5c. Upper panel shows the transcripts of *TUBB3* gene, which derived from GENCODE Comprehensive set. The genomic region between exon 5 and exon 7 is marked by dotted box. Vertical red lines in the lower panel indicate the location of in-frame stop codons, which can lead to premature termination of transcripts. The black think lines in lower panel show BLAT search results of all amplicons shown in Supplementary Fig. 5b and 5c.

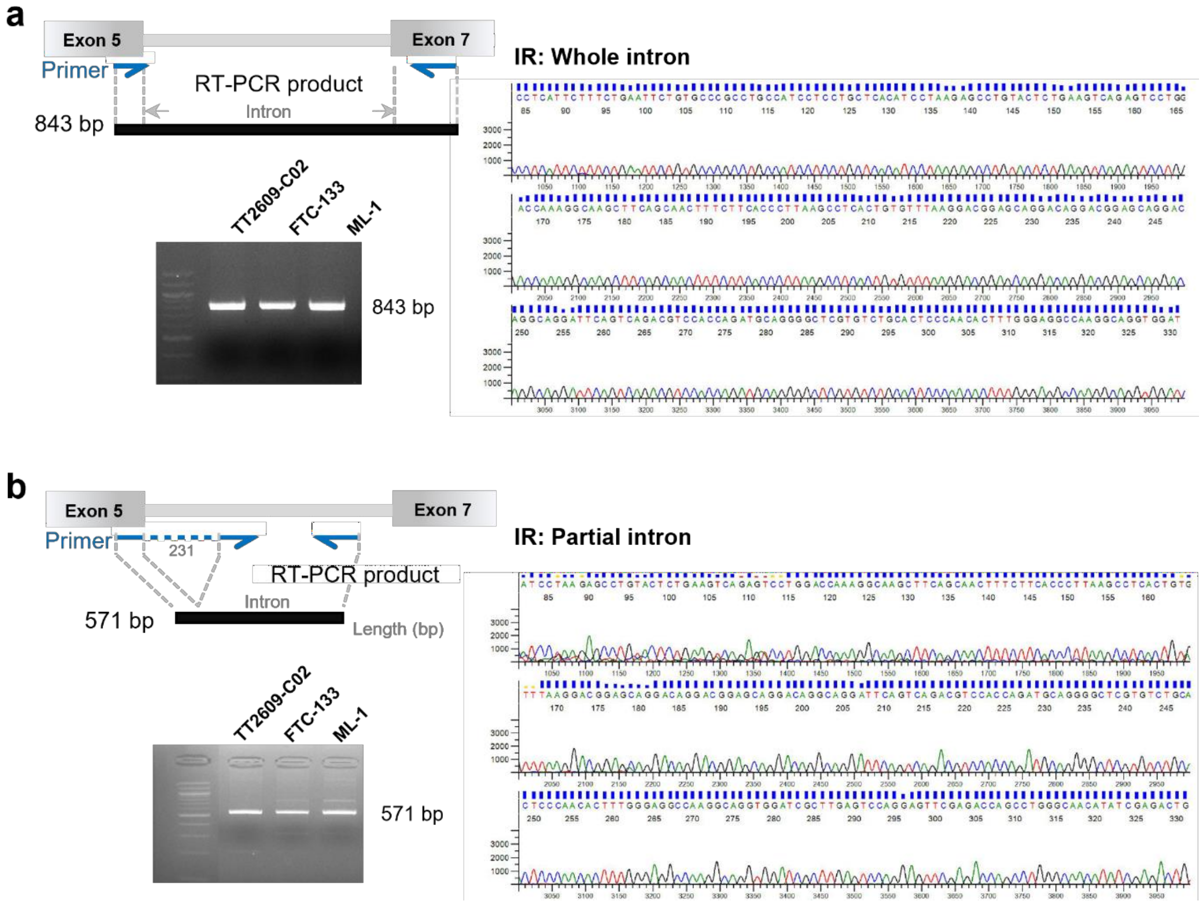

Supplementary Fig. 7. Experimental validation of the two novel intron-retention (IR) events in the *TUBB3* gene. **a** Validation of whole IR. We designed a primer set at exon 5-intron junction and exon 7 (expected size: 843 bp). RT-PCR products showed expected-sized band. Sanger sequencing result are seen on the right. **b** Validation of partial IR. We designed a primer set at the expected IR junction and the intron between exon 5 and 7. We detected 571 bp amplicon and confirmed the partial IR lacking 231 bp by Sanger sequencing.

Reference

1 Mazin, P. V., Khaitovich, P., Cardoso-Moreira, M. & Kaessmann, H. Alternative splicing during mammalian organ development. *Nat. Genet.* **53**, 925-934 (2021).

Supplementary Tables

Supplementary Table 1. List of primers for reverse transcription–polymerase chain reaction (RT-PCR)

Name	Figure in the paper	Forward	Reverse
NUMA1-P2	Fig. 3b	GGAGGTGATGACTGCCAAGT	GACAGCCTTCAGCTTCTGCT
TUBB3-P2	Fig. 5a	GCAACTACGTGGGCGACT	GGCCTGAAGAGATGTCCAAA
TUBB3-P3	Fig. S5a	CAAAGTGTTGGGAGTGCAGA	TCGAGGCACGTACTTGTGAG
TUBB3-P4	Fig. S5b	GCGGATCAGCGTCTACTACA	TCAGAGTCCTGGACCAAAGG
TUBB3-P5	Fig. S6a	AACGAGGCCTCTTGTGAGTG	GTTCCAGGTCCACCAGAATG
TUBB3-P7	Fig. S7b	CTCTTGGCCTGTGCGTGT	ACTTGTGAGCTGAGGGGAGA

Supplementary Table 2. List of 2,190 alternative splicing (AS) events regulated by papillary thyroid cancer (PTC) molecular subtypes.

Gene Symbol	as_id	splice_type	exons	from_exon	to_exon	P-value	adjusted.r2
KIAA1217	10994	AP	10	NA	NA	2.4E-199	0.86
KIAA1217	10995	AP	3	NA	NA	1.2E-176	0.82
SERPINA1	29130	ES	1:2.2:2.3:2.4:2	1.1	3.2	4.2E-110	0.65
TUBB3	38167	AP	4	NA	NA	1.8E-92	0.59
NFYC	2015	AP	4	NA	NA	3.9E-91	0.59
NFYC	2013	AP	1	NA	NA	1.5E-90	0.59
IL1RAP	68107	AT	13	NA	NA	1.5E-89	0.58
TMC6	43757	AT	14.4	NA	NA	3.9E-88	0.57
TMC6	43758	AT	21	NA	NA	3.9E-88	0.57
NEDD4L	45649	AP	6	NA	NA	8.3E-88	0.57
CRLF2	88358	AT	7	NA	NA	1.7E-82	0.57
CRLF2	88357	AT	5	NA	NA	1.7E-82	0.57
STRA6	31688	AD	18.2	18.1	19	1.5E-81	0.54
SHROOM4	89139	AP	3	NA	NA	6.0E-81	0.56
SHROOM4	89138	AP	1	NA	NA	6.0E-81	0.56
S100A2	7720	AP	3	NA	NA	1.3E-77	0.53
NAV2	14695	AP	1	NA	NA	9.7E-76	0.52
ARHGAP24	69815	AP	6	NA	NA	1.8E-75	0.54
KLK10	51264	AP	5	NA	NA	1.2E-73	0.51
KRT15	40913	RI	7.2	7.1	7.3	1.8E-73	0.51
S100A2	7717	AP	2	NA	NA	6.6E-73	0.50
TMEM79	8217	AP	2	NA	NA	1.4E-72	0.52
TMEM79	8218	AP	1	NA	NA	1.4E-72	0.52
DYSF	53935	AP	2	NA	NA	2.7E-72	0.52
DYSF	53934	AP	1	NA	NA	2.7E-72	0.52
PPHLN1	21214	AT	19	NA	NA	3.6E-72	0.50
EPS15L1	48153	AT	16.3	NA	NA	2.1E-71	0.50
LTBR	19846	AP	1	NA	NA	1.8E-70	0.50
LTBR	19848	AP	2	NA	NA	1.8E-70	0.50
CBX5	22140	AP	1	NA	NA	2.5E-69	0.49
CBX5	22139	AP	2.1	NA	NA	2.5E-69	0.49
BCL2L1	58900	AP	2.1	NA	NA	5.8E-69	0.49
BCL2L1	58899	AP	1	NA	NA	5.8E-69	0.49
GPER1	78566	AP	3.1	NA	NA	3.2E-68	0.50
GRB7	40693	AP	2	NA	NA	1.3E-67	0.48
GPER1	78565	AP	2	NA	NA	3.4E-67	0.50
SULT2B1	50773	AP	1	NA	NA	8.9E-67	0.48
SULT2B1	50774	AP	2.1	NA	NA	8.9E-67	0.48
LYNX1	85361	AP	7	NA	NA	3.1E-66	0.47
RNH1	13664	AP	1	NA	NA	1.2E-65	0.47
BEAN1	36708	AT	6.3	NA	NA	5.8E-65	0.47
KLK10	51263	AP	3	NA	NA	5.5E-64	0.46
GRB7	40691	AP	1	NA	NA	9.3E-64	0.46
BID	61002	AP	3	NA	NA	9.8E-64	0.47
ELMO1	79266	AP	1.1	NA	NA	1.0E-63	0.46
EPS15L1	48158	AT	24	NA	NA	1.0E-63	0.46
TUBB3	38166	AP	5.1	NA	NA	1.2E-63	0.46
BID	61000	AP	2	NA	NA	4.3E-63	0.47
TUBB3	38175	ES	6.1:6.2	5.4	7.1	3.5E-62	0.49

NEDD4L	45651	AP	1	NA	NA	3.7E-62	0.45
LSR	49086	ES	5	3	6	8.0E-62	0.45
LYNX1	85363	AP	1	NA	NA	9.3E-62	0.45
RNH1	13662	AP	2	NA	NA	3.3E-61	0.45
SAA2	14577	AT	5	NA	NA	3.3E-60	0.44
SEPT9	43715	AP	8	NA	NA	3.6E-59	0.44
BEAN1	36710	AT	5.3	NA	NA	5.9E-59	0.43
POLR2J3	81113	ES	6:07	4.3	8	3.2E-58	0.43
SAA2	14580	AT	4	NA	NA	3.8E-58	0.43
HES2	401	AT	5	NA	NA	5.0E-58	0.43
HES2	400	AT	4.3	NA	NA	5.0E-58	0.43
TANK	55730	AP	2.1	NA	NA	5.5E-57	0.42
TANK	55731	AP	1	NA	NA	5.5E-57	0.42
SPTBN1	53576	AP	1	NA	NA	9.7E-57	0.45
SPTBN1	53577	AP	3	NA	NA	9.7E-57	0.45
ICAM3	47503	RI	3.2	3.1	3.3	1.9E-56	0.42
CACNB3	21476	ES	9	8	10	2.5E-56	0.43
BCAR3	3796	AP	4	NA	NA	7.3E-56	0.43
MYO1B	56608	ES	23:24	22	25	8.2E-56	0.43
CITED1	89452	AP	3	NA	NA	5.8E-55	0.42
ACAP1	38921	AP	1	NA	NA	1.9E-54	0.41
TRIM46	7953	AT	13	NA	NA	3.2E-54	0.41
RCAN2	76415	AP	4	NA	NA	6.6E-54	0.47
PARD6G	46292	AT	4	NA	NA	1.3E-53	0.40
PARD6G	46293	AT	3	NA	NA	1.3E-53	0.40
ERBB3	22355	AT	5.2	NA	NA	2.2E-53	0.40
ERBB3	22357	AT	29	NA	NA	2.2E-53	0.40
MGAT1	75022	AP	5	NA	NA	3.5E-53	0.40
PRICKLE1	21233	AP	2	NA	NA	5.1E-53	0.41
NUMA1	17515	ES	18	17	19	3.4E-52	0.40
FN1	57393	AA	40.1:40.2:40.3	39	40.4	3.6E-52	0.40
CITED1	89451	AP	1	NA	NA	6.9E-52	0.40
SCIMP	38666	AT	5	NA	NA	8.7E-52	0.39
SCIMP	38667	AT	6	NA	NA	8.7E-52	0.39
DNASE1L1	90575	AP	3.1	NA	NA	3.3E-51	0.39
ZNF430	48741	AT	6	NA	NA	5.3E-51	0.39
ZNF581	52118	AP	1	NA	NA	5.6E-51	0.39
LPXN	16010	AP	2	NA	NA	1.0E-50	0.39
LPXN	16009	AP	1.1	NA	NA	1.0E-50	0.39
FGF1	73871	AP	1	NA	NA	1.2E-50	0.41
INF2	29547	ES	22	21	23	2.0E-50	0.39
NAV2	14696	AP	2	NA	NA	5.1E-50	0.39
RAD23B	87147	AP	2	NA	NA	1.4E-49	0.38
RAD23B	87146	AP	1	NA	NA	1.4E-49	0.38
ALDOA	36038	AP	6.1	NA	NA	1.5E-49	0.38
CACNB3	21469	AP	2	NA	NA	1.7E-49	0.39
OXR1	84844	AP	15.1	NA	NA	1.8E-49	0.38
SSBP3	3144	ES	7	6	8	1.8E-49	0.38
ST6GAL1	68068	AP	1	NA	NA	2.2E-49	0.41
ST6GAL1	68067	AP	3	NA	NA	2.2E-49	0.41
ZNF581	52119	AP	2	NA	NA	2.8E-49	0.38
DYSF	53937	ES	19	18	20	3.8E-49	0.43
ELMO1	79267	AP	15	NA	NA	3.9E-49	0.38

PLXNC1	23721	AP	21	NA	NA	4.9E-49	0.39
SEC31A	100881	ES	27	26.1	28	5.5E-49	0.38
SH3BP1	62140	ES	16	15	17.2	6.1E-49	0.38
MPPED2	14794	AT	10	NA	NA	6.7E-49	0.38
MPPED2	14793	AT	9	NA	NA	6.7E-49	0.38
PI4K2A	12728	AP	1	NA	NA	7.8E-49	0.38
PI4K2A	12729	AP	3	NA	NA	7.8E-49	0.38
P2RX5	38486	AT	13	NA	NA	9.3E-49	0.40
P2RX5	38487	AT	12	NA	NA	9.3E-49	0.40
MXRA8	143	AP	2	NA	NA	1.9E-48	0.38
MXRA8	142	AP	1	NA	NA	1.9E-48	0.38
CADM1	18851	ES	10	9	12	3.9E-48	0.37
ALDOA	36035	AP	7.1	NA	NA	1.0E-47	0.37
ACOX3	68765	AP	1	NA	NA	1.8E-47	0.38
ACOX3	68766	AP	2	NA	NA	1.8E-47	0.38
TUBB6	44667	AT	5.5	NA	NA	2.1E-47	0.37
PLXNC1	23719	AP	1	NA	NA	2.9E-47	0.38
DNAJB6	82510	AT	9.2	NA	NA	3.3E-47	0.37
DNAJB6	82508	AT	11	NA	NA	3.3E-47	0.37
LY75	55707	AT	40	NA	NA	3.6E-47	0.37
LY75	55708	AT	35	NA	NA	3.6E-47	0.37
TUBB6	44669	AT	6	NA	NA	7.0E-47	0.36
SSBP4	48431	ES	7	6	8	1.2E-46	0.36
ARHGAP24	69814	AP	1	NA	NA	3.7E-46	0.38
RARG	21980	AP	4	NA	NA	4.6E-46	0.36
RARG	21979	AP	1	NA	NA	4.6E-46	0.36
MUC20	68182	AP	1	NA	NA	5.6E-46	0.36
MUC20	68183	AP	2.1	NA	NA	5.6E-46	0.36
SEC31A	69727	ES	27	26.2	28	7.2E-46	0.36
SERPINA1	29134	ES	2.1:2.4:2.5	1.1	3.2	7.4E-46	0.36
BTNL9	75035	AT	7.2	NA	NA	1.0E-45	0.36
KIAA1217	11009	ES	12	11	13	1.2E-45	0.36
OSBPL3	79027	ES	9	8	10	1.4E-45	0.37
ARPC1B	80609	ES	8	7	9	1.7E-45	0.36
HNRNPF	11325	AP	3	NA	NA	8.6E-45	0.35
LSR	49085	ES	4:05	3	6	1.5E-44	0.35
TMED4	79538	AT	5	NA	NA	1.8E-44	0.35
TMED4	79539	AT	4.2	NA	NA	1.8E-44	0.35
HNRNPC	26552	ES	2.4:2.5:2.6	1	3.2	1.9E-44	0.35
P2RY6	17683	AP	1	NA	NA	2.1E-44	0.37
TSPAN4	13789	AP	2	NA	NA	2.1E-44	0.35
TNFRSF13B	39450	AT	12	NA	NA	2.3E-44	0.35
PIK3R1	72291	AP	9	NA	NA	2.4E-44	0.36
LAT2	80065	AT	14	NA	NA	3.1E-44	0.35
LAT2	80064	AT	15	NA	NA	3.1E-44	0.35
LYNX1	85365	AT	6.2	NA	NA	5.7E-44	0.35
LYNX1	85366	AT	9.2	NA	NA	5.7E-44	0.35
CCND3	76156	AP	2	NA	NA	5.7E-44	0.35
ECE1	961	AP	1	NA	NA	5.9E-44	0.35
RASA4	81120	AP	2.1	NA	NA	6.7E-44	0.35
RASA4	81121	AP	1.1	NA	NA	6.7E-44	0.35
EPB41L1	59264	AP	6	NA	NA	7.3E-44	0.37
FAM47E	69601	AT	11	NA	NA	1.1E-43	0.35

FAM47E	69602	AT	10	NA	NA	1.1E-43	0.35
DGUOK	54003	ES	5:06	4	7	1.2E-43	0.34
CCND3	76154	AP	3.1	NA	NA	2.8E-43	0.34
NPIPA8	34239	ES	22	20	23	2.8E-43	0.34
FAM86B1	82692	ES	06:07.1	4	8.1	3.2E-43	0.34
ABLIM2	68744	AT	25	NA	NA	4.6E-43	0.34
ABLIM2	68745	AT	18.4	NA	NA	4.6E-43	0.34
PTPRE	13458	AP	1	NA	NA	6.2E-43	0.40
TNFRSF13B	39449	AT	5.2	NA	NA	7.3E-43	0.34
RAG1	15432	AT	9.2	NA	NA	8.2E-43	0.34
RAG1	15433	AT	11	NA	NA	8.2E-43	0.34
KIF16B	58721	AT	23	NA	NA	1.1E-42	0.34
KIF16B	58720	AT	27	NA	NA	1.1E-42	0.34
TUBB3	38168	AP	1	NA	NA	1.6E-42	0.34
AKT2	49869	AP	2	NA	NA	3.1E-42	0.34
AKT2	49868	AP	1	NA	NA	3.1E-42	0.34
ARAP1	17639	AP	2	NA	NA	3.3E-42	0.34
NDUFC1	70618	AP	1	NA	NA	4.1E-42	0.34
NDUFC1	70617	AP	2.1	NA	NA	4.1E-42	0.34
RTKN2	11871	AT	14	NA	NA	4.3E-42	0.33
PPP2R4	87834	AP	14.1	NA	NA	4.5E-42	0.33
C12orf76	24403	AT	10.2	NA	NA	5.0E-42	0.33
TPST2	61518	AP	3	NA	NA	6.3E-42	0.34
TPST2	61516	AP	2	NA	NA	6.5E-42	0.34
PIK3R1	72292	AP	8	NA	NA	1.2E-41	0.35
DGUOK	54004	ES	5	4	7	1.4E-41	0.33
ARAP1	17640	AP	5	NA	NA	1.6E-41	0.33
TRIM46	7951	AT	11	NA	NA	1.8E-41	0.33
BCAR3	3793	AP	11	NA	NA	3.8E-41	0.34
PCYT2	44230	ES	7	6	8	4.0E-41	0.33
SH3BP1	62132	AT	21	NA	NA	4.3E-41	0.33
SH3BP1	62134	AT	20.3	NA	NA	4.3E-41	0.33
TSTD1	8526	ES	2.2:2.3	1	3.1	5.1E-41	0.33
HYDIN	37416	AT	87	NA	NA	8.7E-41	0.33
SPTAN1	87772	ES	38	36.1	39	8.7E-41	0.33
RPS6KA1	1281	AP	7	NA	NA	8.7E-41	0.33
ITGB4	43489	ES	35	34	36	1.3E-40	0.33
UNC5B	12054	ES	8	7	9	1.4E-40	0.37
PCNA	58649	AP	2.1	NA	NA	1.4E-40	0.33
PCNA	58648	AP	1	NA	NA	1.4E-40	0.33
FGF1	73869	AP	3	NA	NA	1.6E-40	0.35
OXR1	84842	AP	6.1	NA	NA	2.0E-40	0.33
PPHLN1	21217	AT	14.2	NA	NA	3.1E-40	0.32
TNK2	68201	AP	4	NA	NA	3.2E-40	0.32
P2RY6	17682	AP	3.1	NA	NA	3.5E-40	0.35
HNRNPC	26556	ES	2.2:2.3:2.4	1	3.2	4.9E-40	0.32
MFF	57806	ES	8:09:10	7	11	5.4E-40	0.32
VPS13D	706	AP	1	NA	NA	1.0E-39	0.34
S100A13	7731	AP	6	NA	NA	1.2E-39	0.32
RBM47	69087	AP	5	NA	NA	2.8E-39	0.32
TLN2	30978	AT	59	NA	NA	3.2E-39	0.32
TLN2	30977	AT	45.2	NA	NA	3.2E-39	0.32
FGD3	86892	AP	2	NA	NA	3.8E-39	0.35

CSF2RA	88369	ES	13	12	14	5.9E-39	0.31
ERBB3	22353	AP	24.1	NA	NA	7.0E-39	0.31
BTNL9	75037	AT	13	NA	NA	7.7E-39	0.31
USP15	22828	AT	7	NA	NA	1.4E-38	0.31
USP15	22829	AT	23	NA	NA	1.4E-38	0.31
C11orf74	15435	AP	2	NA	NA	1.5E-38	0.31
C11orf74	15436	AP	1.1	NA	NA	1.5E-38	0.31
TRAK1	64266	AP	5	NA	NA	1.6E-38	0.35
SERPINA1	29123	AA	2.1:2.2:2.3	1.1	2.4	1.8E-38	0.33
ASPHD1	35983	AT	5	NA	NA	3.1E-38	0.31
ASPHD1	35984	AT	3.2	NA	NA	3.1E-38	0.31
GPR110	76436	AT	17	NA	NA	3.2E-38	0.31
GPR110	76437	AT	8	NA	NA	3.2E-38	0.31
ATF5	51134	AP	1	NA	NA	3.7E-38	0.31
ATF5	51133	AP	2.1	NA	NA	3.7E-38	0.31
TCF20	62501	ES	4.1:4.2	3	5	3.7E-38	0.31
HCFC1R1	33353	AA	3.1	1.5	3.2	4.8E-38	0.31
HMGA2	22880	AT	10	NA	NA	5.9E-38	0.31
TRIM14	87031	AT	8	NA	NA	6.2E-38	0.31
TRIM14	87030	AT	7.3	NA	NA	6.2E-38	0.31
SYNPO2	70455	AT	6	NA	NA	6.3E-38	0.31
PAK6	29958	AP	2	NA	NA	1.1E-37	0.32
TRAK1	64265	AP	1	NA	NA	1.2E-37	0.34
CD44	15142	ES	7:12.1:13:14	5	15	1.2E-37	0.32
CCDC108	57579	AT	34	NA	NA	1.2E-37	0.33
CHEK2	61534	ME	6|7.1:7.2	5	9	1.3E-37	0.31
TTC40	13507	AT	9.2	NA	NA	1.3E-37	0.34
BTBD11	24190	AP	1	NA	NA	1.4E-37	0.32
ARHGAP17	35664	ES	18	17	19	1.5E-37	0.31
PAK6	29956	AP	1	NA	NA	1.6E-37	0.31
CTNNBIP1	578	AP	2	NA	NA	1.8E-37	0.31
PPP2R4	87831	AP	12	NA	NA	2.7E-37	0.30
EXOC7	43569	ES	7	6	8.2	4.2E-37	0.30
EMR2	48028	AT	21	NA	NA	4.3E-37	0.30
EMR2	48029	AT	22	NA	NA	4.3E-37	0.30
PTPRC	9320	AT	34	NA	NA	5.6E-37	0.30
RTKN2	11869	AT	13.2	NA	NA	5.7E-37	0.30
RILPL1	25077	AP	1	NA	NA	6.6E-37	0.30
PTPRC	9321	AT	4	NA	NA	6.8E-37	0.30
CD96	66048	AT	9.2	NA	NA	7.7E-37	0.30
ACAP1	38919	AP	22.1	NA	NA	9.0E-37	0.30
CPNE5	75991	AP	1	NA	NA	9.9E-37	0.32
CPNE5	75990	AP	13	NA	NA	9.9E-37	0.32
ERBB3	22351	AP	1.1	NA	NA	1.3E-36	0.30
LMO7	26061	AP	5.1	NA	NA	1.4E-36	0.32
LMO7	26060	AP	1	NA	NA	1.4E-36	0.32
RPS6KA1	1280	AP	1	NA	NA	2.1E-36	0.30
SRP68	43546	AP	9	NA	NA	2.1E-36	0.30
SRP68	43547	AP	1.1	NA	NA	2.1E-36	0.30
MROH1	85546	ES	26	25.2	27	2.3E-36	0.30
NXNL2	86787	AT	2	NA	NA	2.8E-36	0.30
NXNL2	86786	AT	4	NA	NA	2.8E-36	0.30
PTPN6	20023	AP	2	NA	NA	2.9E-36	0.30

PTPN6	20022	AP	1	NA	NA	2.9E-36	0.30
RILPL1	25079	AP	3	NA	NA	3.2E-36	0.30
ASAP2	52611	ES	23	22	24	3.2E-36	0.30
CD33	51314	AT	6.3	NA	NA	3.3E-36	0.30
CD33	51313	AT	7	NA	NA	3.3E-36	0.30
CLDN11	67617	AT	5	NA	NA	4.1E-36	0.30
CLDN11	67616	AT	3	NA	NA	4.1E-36	0.30
AGAP2	22721	AP	1	NA	NA	4.2E-36	0.35
AGAP2	22720	AP	2	NA	NA	4.2E-36	0.35
LRRC2	64456	AP	1	NA	NA	4.5E-36	0.31
LRRC2	64455	AP	2	NA	NA	4.5E-36	0.31
UBTF	41831	AP	1	NA	NA	4.9E-36	0.30
LDB1	12934	AP	2	NA	NA	6.6E-36	0.30
LDB1	12935	AP	1	NA	NA	6.6E-36	0.30
KIAA1468	45699	ME	24|25	23	26	8.5E-36	0.30
TMEM180	12954	ES	6	5	7	1.1E-35	0.29
PDLIM7	74777	AT	9	NA	NA	1.1E-35	0.29
ARHGEF10L	857	AP	4.1	NA	NA	1.3E-35	0.33
RCAN2	76416	AP	2	NA	NA	1.4E-35	0.35
NPIPA8	34240	ES	21	20	23	1.4E-35	0.29
GBP2	3712	AP	1	NA	NA	1.8E-35	0.31
GBP2	3713	AP	4.1	NA	NA	1.8E-35	0.31
C1S	20068	AP	1	NA	NA	2.6E-35	0.30
C1S	20067	AP	5	NA	NA	2.6E-35	0.30
RANGRF	39163	RI	3.4	3.3	3.5	3.1E-35	0.29
PLAC8	69771	AP	5	NA	NA	4.1E-35	0.34
ELMO1	79269	AD	1.2	1.1	3	4.7E-35	0.32
SEC31A	100885	ES	26.1:27	25.1	28	4.7E-35	0.29
PLCD1	64008	AP	2	NA	NA	4.8E-35	0.29
PLCD1	64009	AP	1	NA	NA	4.8E-35	0.29
TTC7A	53482	AP	3	NA	NA	5.2E-35	0.29
TTC7A	53483	AP	1	NA	NA	5.2E-35	0.29
CLN3	35708	AP	3.1	NA	NA	5.8E-35	0.30
CLN3	35707	AP	1	NA	NA	5.8E-35	0.30
SYTL2	18153	ES	11.2	10.3	12.2	6.9E-35	0.33
TMEM107	39131	ES	2:3.2:3.4:3.5	1	3.7	7.2E-35	0.29
PSTPIP1	31973	ES	3	2.2	5	8.0E-35	0.29
NARF	44391	AP	1	NA	NA	8.7E-35	0.29
NARF	44392	AP	2	NA	NA	8.7E-35	0.29
SLC14A2	45330	AT	13.2	NA	NA	1.1E-34	0.29
SLC14A2	45329	AT	23	NA	NA	1.1E-34	0.29
IPO11	72190	AT	32	NA	NA	1.2E-34	0.29
IPO11	72191	AT	35	NA	NA	1.2E-34	0.29
TNIP1	74126	AP	2	NA	NA	1.4E-34	0.29
TMUB2	41785	AP	2.1	NA	NA	1.4E-34	0.29
CCNDBP1	30220	ES	10.1	9	10.3	1.5E-34	0.29
NRP2	57107	AT	20.2	NA	NA	1.7E-34	0.28
NALCN	26204	AT	7	NA	NA	1.8E-34	0.29
LCN6	88202	AP	1	NA	NA	1.8E-34	0.32
LCN6	88203	AP	3.1	NA	NA	1.8E-34	0.32
NRP2	57106	AT	18	NA	NA	2.2E-34	0.28
GRAMD1C	66223	AP	8	NA	NA	2.4E-34	0.28
CD44	15131	ES	.2:10:11:12.1:	5	15	3.1E-34	0.28

TNIP1	74127	AP	3	NA	NA	6.6E-34	0.28
PDLIM7	74778	AT	15	NA	NA	7.8E-34	0.28
EVL	29241	AP	3	NA	NA	8.2E-34	0.29
TTC40	13506	AT	58	NA	NA	1.1E-33	0.31
CD44	15128	ES	:9.2:10:11:12.	5	15	1.5E-33	0.28
KCTD7	79881	AT	4.4	NA	NA	1.7E-33	0.28
KCTD7	79880	AT	17	NA	NA	1.7E-33	0.28
NALCN	26202	AT	46	NA	NA	1.8E-33	0.28
CLK3	31725	AP	2	NA	NA	1.9E-33	0.28
CLK3	31726	AP	1	NA	NA	1.9E-33	0.28
PRICKLE1	21232	AP	3	NA	NA	3.2E-33	0.29
NCS1	87899	AP	2	NA	NA	3.9E-33	0.28
NCS1	87898	AP	1	NA	NA	3.9E-33	0.28
OAS3	24604	AT	3.2	NA	NA	4.2E-33	0.27
TNK2	68202	AP	1	NA	NA	4.8E-33	0.28
PAK1	17950	AP	2	NA	NA	5.2E-33	0.28
PAK1	17951	AP	1	NA	NA	5.2E-33	0.28
NAV2	14691	AP	3	NA	NA	5.4E-33	0.28
SCNN1A	19842	ES	10	9	11	5.5E-33	0.28
CDC25B	58601	AP	1	NA	NA	6.2E-33	0.28
CDC25B	58600	AP	2	NA	NA	6.2E-33	0.28
COPS7B	57936	AP	2	NA	NA	6.5E-33	0.27
COPS7B	57937	AP	1	NA	NA	6.5E-33	0.27
AGPAT9	69803	AP	1	NA	NA	6.9E-33	0.32
AGPAT9	69804	AP	2.1	NA	NA	6.9E-33	0.32
S100A13	7733	AP	4	NA	NA	7.0E-33	0.27
ACSL5	13109	AP	2	NA	NA	7.3E-33	0.29
ACSL5	13108	AP	3	NA	NA	8.2E-33	0.29
L3MBTL4	44551	AT	22	NA	NA	9.9E-33	0.27
L3MBTL4	44550	AT	19	NA	NA	9.9E-33	0.27
CYP46A1	29229	AT	17	NA	NA	1.4E-32	0.27
CYP46A1	29230	AT	9.2	NA	NA	1.4E-32	0.27
UGP2	53745	AP	2	NA	NA	1.4E-32	0.27
UGP2	53744	AP	1	NA	NA	1.6E-32	0.27
MRPL33	53046	ES	3	2	4	2.4E-32	0.27
PYHIN1	8389	AT	5	NA	NA	3.1E-32	0.27
PYHIN1	8390	AT	10	NA	NA	3.1E-32	0.27
GPR116	76428	AP	1	NA	NA	3.1E-32	0.28
GPR116	76427	AP	2	NA	NA	3.1E-32	0.28
TMEM130	80589	AP	3	NA	NA	3.2E-32	0.27
PRKG1	11580	AP	1	NA	NA	3.3E-32	0.28
PRKG1	11579	AP	2	NA	NA	3.9E-32	0.28
EPB41L1	59268	AP	2	NA	NA	4.1E-32	0.28
MYO1B	56609	ES	24	22	25	4.4E-32	0.27
SCNN1A	19840	AP	2	NA	NA	4.5E-32	0.27
HMGA2	22877	AT	7	NA	NA	5.4E-32	0.27
MITF	65584	AP	4	NA	NA	6.7E-32	0.31
ARHGAP24	69813	AP	4	NA	NA	6.7E-32	0.29
MORF4L2	89765	AP	1	NA	NA	7.9E-32	0.27
POLR2L	13788	AT	3	NA	NA	8.3E-32	0.27
POLR2L	13787	AT	2.2	NA	NA	8.3E-32	0.27
GBA	8041	AP	1	NA	NA	9.0E-32	0.27
GBA	8040	AP	2.1	NA	NA	9.1E-32	0.27

IL1RAP	68105	AT	10.2	NA	NA	1.0E-31	0.27
WSB1	39835	RI	5.2	5.1	5.3	1.1E-31	0.27
DLC1	82763	AP	6	NA	NA	1.1E-31	0.28
FAM102B	3941	AT	11	NA	NA	1.3E-31	0.26
FAM102B	3940	AT	12	NA	NA	1.3E-31	0.26
ZNF524	52115	AP	2.1	NA	NA	1.3E-31	0.26
ZNF524	52116	AP	1	NA	NA	1.3E-31	0.26
P4HA2	73262	ES	2.1:2.2	1	3	1.6E-31	0.27
PPRC1	12938	ES	8:09	7	10	1.7E-31	0.26
TBC1D15	23415	ES	10	9	11	1.9E-31	0.27
CACNB3	21470	AP	3	NA	NA	2.2E-31	0.27
AMPD3	14346	AP	4	NA	NA	2.3E-31	0.31
BAIAP2	44095	ES	17.1:17.2	16.1	18.1	2.4E-31	0.26
CD44	15127	ES	1:9.2:10:11:12	5	15	2.6E-31	0.26
URGCP	79353	AP	1.1	NA	NA	2.9E-31	0.26
LAMB4	81392	AT	18	NA	NA	3.5E-31	0.26
LAMB4	81393	AT	36	NA	NA	3.6E-31	0.26
OXCT1	71882	AP	14	NA	NA	5.2E-31	0.27
PTK2B	83150	AP	6.1	NA	NA	5.8E-31	0.29
MAF	37687	RI	1.2	1.1	1.3	6.7E-31	0.26
ST7	81556	AT	19	NA	NA	7.5E-31	0.26
LUC7L	32844	ES	4	3	5	7.5E-31	0.26
FAM86B1	82686	ES	5:6:7.1:7.2:7.3	4	8.1	7.8E-31	0.26
NLRP1	38720	AT	19	NA	NA	9.8E-31	0.26
MAPRE3	52921	AD	5.2	5.1	6	1.0E-30	0.26
TMEM130	80590	AP	1	NA	NA	1.2E-30	0.26
ST7	81558	AT	20	NA	NA	1.2E-30	0.26
NRP1	11200	AD	14.2	14.1	15.1	1.5E-30	0.26
MORF4L2	89764	AP	2	NA	NA	1.5E-30	0.26
SLAIN2	69214	ES	8	6	9	1.8E-30	0.26
LUC7L	32848	ES	1.3:1.4	1.1	2.2	2.2E-30	0.26
FLT4	75016	AT	31	NA	NA	2.6E-30	0.26
FLT4	75015	AT	30.2	NA	NA	2.6E-30	0.26
MINA	65746	AD	1.2	1.1	2	2.7E-30	0.26
RAC1	78720	ES	4	3	5	3.7E-30	0.25
FMNL3	21605	ES	6	5	7	4.4E-30	0.26
TACC1	83434	AP	5.1	NA	NA	4.6E-30	0.26
ELMO1	79264	AP	2	NA	NA	4.7E-30	0.25
NLRP1	38719	AT	17	NA	NA	5.6E-30	0.25
FMO5	7369	AT	9	NA	NA	6.0E-30	0.25
ARHGEF3	65361	AP	1	NA	NA	8.8E-30	0.27
DENND2D	4141	AP	2	NA	NA	8.9E-30	0.25
DENND2D	4142	AP	1	NA	NA	8.9E-30	0.25
CD44	15130	ES	9.2:10:11:12.1	5	15	9.3E-30	0.25
KCNAB2	361	AP	3	NA	NA	1.0E-29	0.27
KCTD13	35987	AT	2.2	NA	NA	1.0E-29	0.25
PPCDC	31827	AP	1	NA	NA	1.1E-29	0.25
PPCDC	31829	AP	3.1	NA	NA	1.1E-29	0.25
SLC25A37	83083	AD	2.2	2.1	3.2	1.1E-29	0.25
PTK2B	83149	AP	1	NA	NA	1.2E-29	0.28
C16orf13	32915	ES	4	3	5	1.2E-29	0.25
CCDC148	55662	AT	17	NA	NA	1.3E-29	0.25
CTAGE5	27373	AP	3	NA	NA	1.3E-29	0.25

HYDIN	37420	AT	20.2	NA	NA	1.5E-29	0.25
SULF2	59728	ES	20	19.2	21	1.6E-29	0.25
KCNN3	7841	AP	3	NA	NA	2.0E-29	0.25
LCN12	127791	ES	4	3	5	2.5E-29	0.25
MCPH1	82575	AT	14	NA	NA	2.7E-29	0.25
MCPH1	82574	AT	8.2	NA	NA	2.7E-29	0.25
CHEK1	19311	AT	13.3	NA	NA	3.6E-29	0.25
CHEK1	19312	AT	14	NA	NA	3.6E-29	0.25
ARHGAP22	11485	AP	3	NA	NA	3.8E-29	0.25
DCN	23655	AT	6	NA	NA	3.8E-29	0.25
IGSF1	90114	AT	28	NA	NA	4.2E-29	0.25
DCN	23652	AT	12	NA	NA	4.2E-29	0.25
IGSF1	90115	AT	13.2	NA	NA	4.2E-29	0.25
PARP3	65116	ES	02:03.1	1	3.2	4.2E-29	0.26
BCKDHB	76828	RI	11.2	11.1	11.3	5.6E-29	0.25
MORF4L2	89774	ES	04:05.3	3.2	6.2	5.7E-29	0.25
PCBD2	73439	AT	5	NA	NA	6.3E-29	0.25
PCBD2	73438	AT	4.2	NA	NA	6.3E-29	0.25
TGIF1	44502	AP	3	NA	NA	6.7E-29	0.25
ELF5	14951	AT	4.2	NA	NA	7.1E-29	0.26
ELF5	14952	AT	8	NA	NA	7.1E-29	0.26
DIXDC1	18709	AT	7.2	NA	NA	8.4E-29	0.24
DIXDC1	18710	AT	24	NA	NA	8.4E-29	0.24
GULP1	56499	AT	9.2	NA	NA	9.7E-29	0.24
GULP1	56500	AT	15	NA	NA	9.7E-29	0.24
EXOC7	43571	ES	07:08.2	6	9	1.0E-28	0.24
MCFD2	53471	AP	3	NA	NA	1.2E-28	0.24
DYSF	53936	ES	43	42	44	1.2E-28	0.25
EVA1A	54148	AP	2	NA	NA	1.3E-28	0.26
SH3BP2	68592	AP	1	NA	NA	1.4E-28	0.25
KCNN3	7840	AP	1	NA	NA	1.5E-28	0.24
FBLN5	28888	AP	2	NA	NA	1.5E-28	0.25
FBLN5	28887	AP	1	NA	NA	1.5E-28	0.25
PTGR1	87215	AP	2	NA	NA	1.7E-28	0.24
PTGR1	87213	AP	1	NA	NA	1.7E-28	0.24
LUC7L	32850	ES	1.3	1.1	2.2	1.7E-28	0.25
INPP5J	61825	ES	2	1	3.1	1.7E-28	0.24
SEC31A	69728	ES	26.2:27	26.1	28	1.8E-28	0.24
GRAMD1C	66220	AP	1	NA	NA	1.9E-28	0.24
OAS3	24605	AT	16	NA	NA	1.9E-28	0.24
CCDC36	64848	AT	12	NA	NA	1.9E-28	0.24
CCDC36	64847	AT	10.2	NA	NA	1.9E-28	0.24
BTBD11	24189	AP	13	NA	NA	2.3E-28	0.25
ERLIN2	83344	AP	1	NA	NA	2.4E-28	0.24
MIER1	3333	AP	1	NA	NA	2.6E-28	0.26
MIER1	3332	AP	4	NA	NA	2.6E-28	0.26
ESR1	78167	AT	13	NA	NA	3.1E-28	0.24
ESR1	78166	AT	14	NA	NA	3.1E-28	0.24
VGLL4	63393	AP	1	NA	NA	3.1E-28	0.24
LARP7	70384	AP	1	NA	NA	3.2E-28	0.24
LARP7	70383	AP	2	NA	NA	3.2E-28	0.24
LIMK1	80060	AP	4	NA	NA	3.4E-28	0.25
SERPINA1	29115	AA	2.1:2.2:2.3:2.4	1.1	2.5	3.8E-28	0.26

PLAC8	69770	AP	6	NA	NA	4.0E-28	0.28
KCNAB1	67357	AP	2	NA	NA	4.1E-28	0.26
SNAPC3	85916	RI	9.2	9.1	9.3	4.1E-28	0.24
CCL28	71946	AT	6	NA	NA	4.7E-28	0.24
CCL28	71947	AT	5.2	NA	NA	4.7E-28	0.24
CLNK	68788	AT	15	NA	NA	5.3E-28	0.24
CLNK	68789	AT	24	NA	NA	5.3E-28	0.24
KCNAB2	360	AP	2	NA	NA	5.5E-28	0.26
LIMK1	80059	AP	2	NA	NA	5.8E-28	0.25
ZNF333	48017	AT	17	NA	NA	6.4E-28	0.24
NFATC1	46239	AT	11	NA	NA	7.1E-28	0.24
NFATC1	46240	AT	9.2	NA	NA	7.1E-28	0.24
MAST4	72283	AT	9	NA	NA	8.2E-28	0.24
INCENP	16337	ES	11	10	12	8.6E-28	0.25
CLCF1	17173	AP	1	NA	NA	8.7E-28	0.24
OXCT1	71881	AP	1	NA	NA	9.3E-28	0.24
ADAL	30234	AT	13	NA	NA	1.0E-27	0.24
ADAL	30232	AT	11.2	NA	NA	1.0E-27	0.24
ST5	14263	AP	19	NA	NA	1.1E-27	0.24
ACYP1	28474	AP	1	NA	NA	1.1E-27	0.24
ACYP1	28473	AP	2.1	NA	NA	1.1E-27	0.24
ARAP1	17641	ES	32	31	33	1.4E-27	0.24
ABI1	11032	ES	12	11.2	13	1.6E-27	0.24
SAMD3	77517	AT	19	NA	NA	1.8E-27	0.24
C6orf123	78445	AT	4	NA	NA	2.0E-27	0.23
C6orf123	78444	AT	3.2	NA	NA	2.0E-27	0.23
ABLIM1	13209	ES	16	15	17	2.1E-27	0.25
SEC23A	27347	AT	23	NA	NA	2.3E-27	0.23
SEC23A	27348	AT	4.2	NA	NA	2.4E-27	0.23
NPIPB5	35567	ES	3.3	2.5	5	2.4E-27	0.25
CCDC108	57577	AT	12.2	NA	NA	2.5E-27	0.26
ACLY	40960	ES	14	13	15	2.6E-27	0.24
BRE	53054	ES	14	12	15	2.6E-27	0.23
PCCB	66900	AT	18.2	NA	NA	2.7E-27	0.23
PCCB	66901	AT	19.2	NA	NA	2.7E-27	0.23
DOCK9	26172	AP	3	NA	NA	3.3E-27	0.25
RABGGTA	26950	RI	1.2:1.3	1.1	1.4	3.3E-27	0.23
FAM47E	69609	ES	4	3	5	3.8E-27	0.23
ZNF506	48683	AT	8	NA	NA	4.7E-27	0.23
ALG13	89905	AT	4	NA	NA	5.0E-27	0.23
ALG13	89906	AT	28	NA	NA	5.0E-27	0.23
SIRT3	13591	AP	1	NA	NA	5.0E-27	0.23
SIRT3	13592	AP	2.1	NA	NA	5.0E-27	0.23
RGS3	87291	AP	5	NA	NA	5.3E-27	0.23
RBM4	17096	ES	4.1	3.2	5	6.4E-27	0.23
SAMD3	77518	AT	13.2	NA	NA	6.7E-27	0.23
NADK	228	AP	1	NA	NA	6.9E-27	0.24
DOCK9	26171	AP	1	NA	NA	7.7E-27	0.25
SYTL2	18148	ES	13	12.2	14	7.7E-27	0.25
PDE5A	70465	AP	1	NA	NA	8.1E-27	0.24
MAPK10	69826	ES	9	8.2	10	1.0E-26	0.24
SVEP1	87187	AT	49	NA	NA	1.1E-26	0.23
MAST4	72281	AT	36	NA	NA	1.1E-26	0.23

MITF	65588	AP	1	NA	NA	1.1E-26	0.26
RPL31	54729	AT	2.6	NA	NA	1.1E-26	0.23
B4GALT3	8588	AA	3.1	2.2	3.2	1.2E-26	0.23
RAB34	39953	AP	1	NA	NA	1.4E-26	0.23
RAB34	39954	AP	2.1	NA	NA	1.4E-26	0.23
ACSL6	73248	AT	39	NA	NA	1.4E-26	0.23
ACSL6	73246	AT	27	NA	NA	1.4E-26	0.23
MAP3K4	78357	ES	18	17	19	1.5E-26	0.23
PCSK5	86634	AT	21	NA	NA	1.5E-26	0.23
ERCC1	50442	AT	10.2	NA	NA	1.5E-26	0.23
ERCC1	50441	AT	11	NA	NA	1.5E-26	0.23
SYNE1	78183	ES	151	150	152	1.9E-26	0.23
IRF5	81735	ES	5	4	6.1	2.1E-26	0.24
CLCF1	17175	AP	2	NA	NA	2.3E-26	0.23
PTK2	85305	ES	39.2	37	39.5	2.3E-26	0.23
BANF1	16979	AP	1.1	NA	NA	2.5E-26	0.23
BANF1	16980	AP	2.1	NA	NA	2.5E-26	0.23
PPFIBP1	20893	ES	11	10	12	2.6E-26	0.25
SVEP1	87186	AT	28.2	NA	NA	2.9E-26	0.23
IGSF3	4366	ES	6	5	7	2.9E-26	0.26
TACC1	83437	AP	1	NA	NA	3.4E-26	0.23
ADCK4	49944	AP	1	NA	NA	3.6E-26	0.23
MYO5A	30660	ES	35	34	36	3.7E-26	0.25
CFLAR	56799	ES	10	8.1	11	3.9E-26	0.22
LYST	10361	AP	1	NA	NA	3.9E-26	0.23
LYST	10360	AP	2.1	NA	NA	3.9E-26	0.23
TMEM126B	18122	ES	3	1	4	3.9E-26	0.23
CD96	66049	AT	16	NA	NA	4.0E-26	0.22
PDE4D	72140	AP	13	NA	NA	4.0E-26	0.23
ERLIN2	83343	AP	2.1	NA	NA	4.1E-26	0.23
KIF13A	75458	ES	28	27	29	4.2E-26	0.24
PRKCA	43100	AT	19	NA	NA	4.4E-26	0.22
PRKCA	43101	AT	10	NA	NA	4.4E-26	0.22
MKL1	62348	AP	1	NA	NA	5.3E-26	0.23
MKL1	62349	AP	4.1	NA	NA	5.3E-26	0.23
SGK1	77770	AP	6.1	NA	NA	5.5E-26	0.23
NFIX	47903	AP	1	NA	NA	6.2E-26	0.24
VPS13D	704	AP	70	NA	NA	6.3E-26	0.24
NAAA	69561	AT	12	NA	NA	8.6E-26	0.22
DNASE1L1	90574	AP	1	NA	NA	8.7E-26	0.22
S100PBP	1636	AT	7	NA	NA	1.0E-25	0.22
S100PBP	1635	AT	9	NA	NA	1.0E-25	0.22
NSMCE4A	13328	AP	1	NA	NA	1.1E-25	0.22
NSMCE4A	13329	AP	2	NA	NA	1.1E-25	0.22
DST	76560	ES	106	105	107	1.3E-25	0.22
ZNF575	50204	AP	4	NA	NA	1.3E-25	0.23
ZNF575	50203	AP	1	NA	NA	1.3E-25	0.23
SLC14A2	45328	AP	1	NA	NA	1.3E-25	0.23
SLC14A2	45327	AP	3.1	NA	NA	1.3E-25	0.23
C6orf141	76449	AT	5	NA	NA	1.5E-25	0.22
ITGA7	22216	ES	29	28	30	1.5E-25	0.22
ZFAND1	84309	ES	3	2	4	1.5E-25	0.22
TRIM16	39364	AP	6.1	NA	NA	1.6E-25	0.24

TRIM16	39362	AP	1.1	NA	NA	1.6E-25	0.24
MATN2	84633	AP	1.1	NA	NA	1.6E-25	0.23
MATN2	84632	AP	2.1	NA	NA	1.6E-25	0.23
DTNA	45092	AP	2	NA	NA	1.7E-25	0.23
KLHL7	78952	AT	7	NA	NA	2.0E-25	0.22
SSBP4	48426	AP	1	NA	NA	2.2E-25	0.22
SSBP4	48427	AP	17.1	NA	NA	2.2E-25	0.22
PRICKLE1	21231	AP	4	NA	NA	2.5E-25	0.23
EVA1A	54150	AP	3	NA	NA	2.6E-25	0.23
GUSB	79855	ES	6	5.1	7	2.6E-25	0.22
CORO1A	36091	ES	2	1	3	2.7E-25	0.22
LRRC23	20002	ES	8:09	7.1	10	3.5E-25	0.22
ABI1	11037	ES	11.1:11.2:12	9	13	3.9E-25	0.22
APOD	68181	ES	3	2	4	4.0E-25	0.22
RALGDS	88016	AP	8	NA	NA	4.2E-25	0.22
RAPGEF1	87965	AP	3	NA	NA	5.1E-25	0.23
FN1	57398	ES	25	24	26	5.7E-25	0.22
PCDH1	73831	AT	7	NA	NA	5.8E-25	0.22
PCDH1	73832	AT	4.2	NA	NA	5.8E-25	0.22
EEF1B2	57144	AA	1.3	1.1	1.4	6.2E-25	0.22
MACROD2	58714	AP	6	NA	NA	6.5E-25	0.22
CDH23	12058	AT	15.2	NA	NA	6.9E-25	0.22
FBXL2	63846	AD	17.2	17.1	18	7.2E-25	0.22
F8	90667	AP	23	NA	NA	7.4E-25	0.25
F8	90666	AP	1	NA	NA	7.4E-25	0.25
COL6A3	58104	ES	6	5	7	7.6E-25	0.24
MACROD2	58715	AP	15	NA	NA	8.1E-25	0.21
EPB41L3	44533	ES	25	24	26	8.2E-25	0.22
TMUB2	41784	AP	1	NA	NA	8.4E-25	0.22
TRIM73	80116	AT	7.3	NA	NA	9.5E-25	0.21
TRIM73	80115	AT	9	NA	NA	9.5E-25	0.21
TMC6	43751	AP	2	NA	NA	1.1E-24	0.22
KLHL7	78950	AT	14	NA	NA	1.2E-24	0.21
ABI1	11038	ES	11.2:12	9	13	1.3E-24	0.22
AHCYL2	81744	AP	3	NA	NA	1.3E-24	0.22
METTL21A	57188	AP	2.1	NA	NA	1.5E-24	0.22
PTPRE	13457	AP	6	NA	NA	1.7E-24	0.25
CHCHD7	83913	ES	2.1:2.2	1	4	1.7E-24	0.21
DYSF	53938	ES	7	6	8	1.8E-24	0.24
CCDC108	57578	AT	4.2	NA	NA	1.9E-24	0.23
CRB3	47050	RI	5.2	5.1	5.3	2.0E-24	0.21
MYL6	22378	ES	4.3	4.1	5	2.0E-24	0.21
IL12RB1	48393	AT	19	NA	NA	2.1E-24	0.21
IL12RB1	48392	AT	11	NA	NA	2.1E-24	0.21
COA1	79330	AT	9.2	NA	NA	2.2E-24	0.21
C11orf74	15442	AD	1.2	1.1	4	2.3E-24	0.21
RAPGEF1	87967	AP	1	NA	NA	2.6E-24	0.23
KCNAB1	67355	AP	3	NA	NA	3.0E-24	0.23
FAM104B	89278	AT	4.2	NA	NA	3.0E-24	0.21
FAM104B	89277	AT	5	NA	NA	3.0E-24	0.21
RAP1A	4176	AP	3	NA	NA	3.1E-24	0.22
MPZL2	18984	AT	5.2	NA	NA	3.3E-24	0.21
MPZL2	18983	AT	6	NA	NA	3.3E-24	0.21

RAP1A	4178	AP	2	NA	NA	3.4E-24	0.22
KLHL3	73482	AT	11	NA	NA	3.7E-24	0.21
KLHL3	73481	AT	18	NA	NA	3.7E-24	0.21
ELP3	83201	AP	2	NA	NA	3.7E-24	0.22
ELP3	83202	AP	1	NA	NA	3.7E-24	0.22
TMEM234	1571	RI	5.5	5.4	5.6	3.8E-24	0.21
C19orf24	46447	AP	1	NA	NA	5.5E-24	0.21
C19orf24	46446	AP	2	NA	NA	5.5E-24	0.21
MAP4K1	49669	ES	31	30	32	5.9E-24	0.21
RPL31	54732	AT	4	NA	NA	5.9E-24	0.21
KCTD13	35989	AT	6.2	NA	NA	6.1E-24	0.21
TEKT4	54479	AT	2	NA	NA	7.0E-24	0.21
TEKT4	54480	AT	7	NA	NA	7.0E-24	0.21
ZNF626	48724	AT	5	NA	NA	7.3E-24	0.21
ZNF626	48723	AT	4	NA	NA	7.3E-24	0.21
KATNAL2	45429	AT	20	NA	NA	7.8E-24	0.21
KATNAL2	45430	AT	17	NA	NA	7.8E-24	0.21
PLB1	53064	AT	35	NA	NA	7.9E-24	0.21
ANXA6	74148	ES	22	21	23	7.9E-24	0.21
PLB1	53065	AT	60	NA	NA	7.9E-24	0.21
CCDC148	55661	AT	12	NA	NA	8.7E-24	0.21
LGALS9	39858	ES	5	4	6	9.0E-24	0.21
SMARCC2	22390	RI	28.2	28.1	28.3	9.2E-24	0.21
SLC35C2	59672	AA	3.1	1	3.2	9.4E-24	0.21
ADK	12258	AP	1	NA	NA	9.4E-24	0.21
ADK	12257	AP	2	NA	NA	9.5E-24	0.21
LMF1	33028	AP	2	NA	NA	1.1E-23	0.21
LMF1	33029	AP	1	NA	NA	1.1E-23	0.21
EVL	29239	AP	1	NA	NA	1.1E-23	0.21
FIP1L1	69311	AT	31	NA	NA	1.2E-23	0.21
ADAMTS2	74891	AT	11	NA	NA	1.3E-23	0.21
ADAMTS2	74892	AT	23	NA	NA	1.3E-23	0.21
PTGIR	50569	AT	4.2	NA	NA	1.3E-23	0.21
PTGIR	50570	AT	3	NA	NA	1.3E-23	0.21
IQCH	31314	AT	13.2	NA	NA	1.6E-23	0.20
CASP8	56813	AP	3	NA	NA	1.6E-23	0.22
SLC25A36	67052	ES	6.1:6.2	4.1	7	1.7E-23	0.22
C4orf19	69000	AT	6	NA	NA	1.9E-23	0.20
C4orf19	69001	AT	5	NA	NA	1.9E-23	0.20
MROH6	85423	AP	1	NA	NA	1.9E-23	0.22
MROH6	85424	AP	9.1	NA	NA	1.9E-23	0.22
FLT3LG	50945	AT	11.2	NA	NA	2.2E-23	0.20
CAST	72841	AP	3	NA	NA	2.2E-23	0.21
ATP2C1	66756	AP	1	NA	NA	2.4E-23	0.23
PARP3	65119	ES	2	1	3.1	2.4E-23	0.22
SMARCD3	82359	AP	1	NA	NA	2.5E-23	0.20
NPIPB6	35702	AP	1	NA	NA	2.5E-23	0.20
NPIPB6	35703	AP	2	NA	NA	2.5E-23	0.20
FIP1L1	69310	AT	19	NA	NA	2.7E-23	0.20
MAPK9	75012	ES	3	2	4	2.7E-23	0.21
TPM2	98133	ES	6	5	7	2.7E-23	0.24
IQCH	31311	AT	24	NA	NA	2.9E-23	0.20
ANK3	11843	AP	3	NA	NA	3.0E-23	0.21

BAIAP2	44096	ES	17.1	16.1	18.1	3.2E-23	0.20
HSD17B14	50818	ES	5	4.2	6	3.2E-23	0.20
DUSP18	61795	AT	4	NA	NA	3.6E-23	0.20
RASA1	72723	AD	1.2	1.1	4	4.0E-23	0.22
ETS1	19409	AP	4	NA	NA	4.1E-23	0.20
ETS1	19408	AP	1	NA	NA	4.1E-23	0.20
PRPSAP2	39657	AP	1	NA	NA	4.5E-23	0.21
PRPSAP2	39659	AP	3	NA	NA	4.5E-23	0.21
SMARCD3	82361	AP	4	NA	NA	4.6E-23	0.20
TMC6	43759	AD	14.2:14.3	14.1	15	4.7E-23	0.21
CAST	72863	ES	8.2	7.1	9	4.7E-23	0.20
LEPR	3295	AT	22	NA	NA	4.7E-23	0.20
FLT1	25556	AT	16	NA	NA	5.3E-23	0.20
COL16A1	1493	ES	44	43	45.1	5.5E-23	0.20
GRB7	40698	ES	16	15	17	5.9E-23	0.20
PEX26	61021	AT	9	NA	NA	6.5E-23	0.20
PEX26	61022	AT	5	NA	NA	6.5E-23	0.20
LEPR	3297	AT	21	NA	NA	6.6E-23	0.20
MXI1	13080	AP	1	NA	NA	7.4E-23	0.20
FLT1	25558	AT	35	NA	NA	7.4E-23	0.20
PFDN5	22006	ES	2	1	4.1	7.6E-23	0.20
SLC11A2	21725	AP	6	NA	NA	7.7E-23	0.20
CASZ1	622	AT	21	NA	NA	7.8E-23	0.20
CASZ1	621	AT	16.2	NA	NA	7.8E-23	0.20
ACSL1	71323	ME	16|17	15	18.1	8.2E-23	0.20
SHF	30409	AP	3	NA	NA	8.3E-23	0.20
ZNF124	10514	AT	5	NA	NA	8.4E-23	0.20
NPIPB4	35510	ES	3.3	2.5	4	8.4E-23	0.22
BCCIP	13433	AT	8.2	NA	NA	9.3E-23	0.20
IPO11	72193	ES	30	29	33	9.5E-23	0.21
COL4A6	89858	AT	51.2	NA	NA	9.5E-23	0.21
COL4A6	89859	AT	52	NA	NA	9.5E-23	0.21
MAST4	72278	AP	7	NA	NA	9.6E-23	0.20
PDS5B	25604	ES	33.1	32	34	1.1E-22	0.21
CASC4	30336	ES	10	9	11	1.4E-22	0.20
CD55	9632	AT	10.2	NA	NA	1.5E-22	0.20
CD55	9631	AT	15	NA	NA	1.5E-22	0.20
DLC1	82764	AP	1	NA	NA	1.8E-22	0.21
FAM63A	7539	AA	4.1	2.2	4.2	1.8E-22	0.20
VPS13D	705	AP	64	NA	NA	2.1E-22	0.21
ZBTB7C	45452	AP	6	NA	NA	2.4E-22	0.21
ADCK4	49942	AP	2	NA	NA	2.5E-22	0.20
KIF4A	89373	AT	32	NA	NA	2.6E-22	0.20
KIF4A	89372	AT	29	NA	NA	2.6E-22	0.20
KIFC3	36605	AP	4	NA	NA	2.8E-22	0.20
LRRC23	20003	ES	8	7.1	10	3.0E-22	0.20
SH3BP2	68590	AP	3	NA	NA	3.1E-22	0.20
AHCYL2	81742	AP	1	NA	NA	3.1E-22	0.20
EPB41L5	55146	AT	26	NA	NA	3.4E-22	0.19
EPB41L5	55145	AT	17	NA	NA	3.4E-22	0.19
OGG1	63158	AT	8	NA	NA	3.5E-22	0.19
OGG1	63159	AT	6.4	NA	NA	3.5E-22	0.19
CREM	11230	AP	12	NA	NA	3.6E-22	0.19

MEF2BNB	48602	AT	6.2	NA	NA	3.7E-22	0.19
MEF2BNB	48603	AT	4.2	NA	NA	3.7E-22	0.19
NME4	32879	ES	4	2	5	3.9E-22	0.19
PKN1	47977	AP	1	NA	NA	4.1E-22	0.20
PKN1	47976	AP	2	NA	NA	4.1E-22	0.20
ZNF530	52302	AT	4.2	NA	NA	4.4E-22	0.19
ZNF530	52303	AT	5	NA	NA	4.4E-22	0.19
OPA1	68139	ES	7	6	8	4.4E-22	0.21
LYPLA2	1071	AP	5.1	NA	NA	4.8E-22	0.19
LYPLA2	1070	AP	1	NA	NA	4.8E-22	0.19
ARHGAP27	41961	AP	7	NA	NA	5.0E-22	0.22
EPB41L1	59273	ES	20	19	21	5.1E-22	0.19
ELN	80043	AA	24.1	23	24.2	5.2E-22	0.22
ARMCX3	89663	AA	3.1	1	3.2	5.5E-22	0.20
LRRFIP1	58130	AT	26	NA	NA	5.7E-22	0.19
LRRFIP1	58131	AT	21	NA	NA	5.7E-22	0.19
TANGO2	61114	AP	3.1	NA	NA	5.8E-22	0.21
TANGO2	61112	AP	1	NA	NA	5.8E-22	0.21
LRRFIP2	63960	ES	20	19	21	5.9E-22	0.19
CREB3L4	7769	AP	2	NA	NA	6.6E-22	0.19
CREB3L4	7768	AP	1.1	NA	NA	6.6E-22	0.19
FAM178A	12842	AT	20	NA	NA	6.9E-22	0.19
DNAJB2	57675	RI	9.2	9.1	9.3	7.0E-22	0.19
COX19	78556	AT	13	NA	NA	7.1E-22	0.19
COX19	78557	AT	3	NA	NA	7.1E-22	0.19
RNH1	13673	ES	3	2	4.2	7.9E-22	0.19
FIP1L1	69312	ES	14	13.1	15	8.0E-22	0.19
MYO9B	48232	ES	37	36	38.1	8.1E-22	0.22
BBIP1	13098	ES	3:04	1	5.1	8.3E-22	0.19
ZNF174	33571	AT	2.3	NA	NA	8.5E-22	0.19
ZNF174	33572	AT	3	NA	NA	8.5E-22	0.19
DIXDC1	18708	AP	3	NA	NA	8.6E-22	0.21
WIPF1	56026	AP	1	NA	NA	8.8E-22	0.20
FADS2	16291	AP	6	NA	NA	8.9E-22	0.19
PGAP2	14010	ES	7	6	10	9.0E-22	0.20
TRIM16L	39631	AP	6.1	NA	NA	9.4E-22	0.21
TRIM16L	39629	AP	1.1	NA	NA	9.5E-22	0.21
SYNE1	78184	ES	143	142	144	9.6E-22	0.20
CRYGN	152609	AD	2.2	2.1	3	1.2E-21	0.20
SLCO3A1	32546	AT	10.2	NA	NA	1.2E-21	0.19
SLCO3A1	32547	AT	11	NA	NA	1.2E-21	0.19
PTK2B	83156	ES	28	27	29	1.2E-21	0.19
NPIPB5	35566	ES	3.1:3.2:3.3	2.5	5	1.2E-21	0.19
GRIN2C	43309	AT	14	NA	NA	1.3E-21	0.19
GRIN2C	43310	AT	13.2	NA	NA	1.3E-21	0.19
STXBP6	27043	AD	1.2	1.1	6	1.3E-21	0.20
SLC44A2	47559	AP	2	NA	NA	1.3E-21	0.19
SLC44A2	47558	AP	1	NA	NA	1.3E-21	0.19
C1orf54	7454	AP	3.1	NA	NA	1.3E-21	0.19
C1orf54	7455	AP	1	NA	NA	1.3E-21	0.19
ANKRD18A	86459	AT	22	NA	NA	1.3E-21	0.19
ANKRD18A	86460	AT	17.2	NA	NA	1.3E-21	0.19
DPYSL3	73957	AP	2.1	NA	NA	1.4E-21	0.19

DPYSL3	73958	AP	1	NA	NA	1.4E-21	0.19
MAPKAPK3	65093	AP	1	NA	NA	1.4E-21	0.19
MAPKAPK3	65092	AP	4	NA	NA	1.4E-21	0.19
FMO5	7367	AT	11	NA	NA	1.4E-21	0.19
MYO5B	45495	AP	1	NA	NA	1.4E-21	0.20
MYO5B	45493	AP	32.1	NA	NA	1.4E-21	0.20
SEC31A	69730	ES	26.1:26.2:27	25.1	28	1.5E-21	0.19
GSE1	37883	ES	3	2	4	1.6E-21	0.21
NPHP1	54927	AT	4.2	NA	NA	1.7E-21	0.19
NPHP1	54928	AT	21	NA	NA	1.7E-21	0.19
C1orf85	8221	AT	6.3	NA	NA	1.8E-21	0.19
C1orf85	8222	AT	7	NA	NA	1.8E-21	0.19
E2F5	84325	AP	1	NA	NA	2.0E-21	0.20
E2F5	84324	AP	2	NA	NA	2.0E-21	0.20
FAM86B1	82688	ES	7.1:7.2:7.3	4	8.1	2.1E-21	0.20
ABCA9	43157	AT	39	NA	NA	2.2E-21	0.19
ABCA9	43158	AT	6.2	NA	NA	2.2E-21	0.19
GLIPR1	23464	RI	5.2	5.1	5.3	2.2E-21	0.19
NPIPA5	34150	ES	8.3	8.1	9	2.3E-21	0.19
MAP3K12	22063	AP	3.1	NA	NA	2.3E-21	0.19
ASPH	83961	AP	1	NA	NA	2.7E-21	0.19
ASPH	83962	AP	2	NA	NA	2.7E-21	0.19
GEMIN2	27356	ES	5	4	6	2.8E-21	0.19
RGS3	87289	AP	25	NA	NA	2.9E-21	0.19
ADAP1	78554	AP	1	NA	NA	2.9E-21	0.20
PLEC	85511	AP	1	NA	NA	3.1E-21	0.20
MAP3K12	22061	AP	1	NA	NA	3.2E-21	0.19
ZNF83	51479	AP	1	NA	NA	3.2E-21	0.19
RBCK1	58451	AT	13	NA	NA	3.3E-21	0.19
RBCK1	58450	AT	3	NA	NA	3.3E-21	0.19
NR1H3	15695	AP	3.1	NA	NA	3.4E-21	0.19
NAIP	72432	AT	7.2	NA	NA	3.5E-21	0.19
NAIP	72431	AT	18	NA	NA	3.5E-21	0.19
FAM195B	44172	RI	4.4	4.3	4.5	3.7E-21	0.19
TNFSF13	38970	AP	2.1	NA	NA	3.8E-21	0.19
TNFSF13	38969	AP	1	NA	NA	3.8E-21	0.19
GBA2	86289	RI	14.5	14.4	14.6	4.0E-21	0.19
SERPINA1	29136	ES	2.4:2.5	1.1	3.2	4.9E-21	0.19
WTAP	78313	AT	7.2	NA	NA	5.1E-21	0.19
WTAP	78312	AT	9	NA	NA	5.1E-21	0.19
GANC	30141	AT	29	NA	NA	5.3E-21	0.19
DNAJC5	60176	ES	5	4	6	5.5E-21	0.18
TUSC3	82771	ES	11	10	12	5.5E-21	0.19
TDRKH	7662	AT	15.3	NA	NA	5.5E-21	0.18
DOCK9	26178	ES	48	47	49	5.9E-21	0.19
LYRM1	34404	AP	3	NA	NA	6.8E-21	0.19
TJP3	46725	AP	1	NA	NA	6.9E-21	0.19
ARHGAP27	41957	AP	6	NA	NA	6.9E-21	0.21
GNPDA1	73865	ES	2.4	1	2.6	7.0E-21	0.19
SYTL2	18156	AD	8.3	8.2	9	7.7E-21	0.19
CASK	88866	ES	20	19.1	22	8.2E-21	0.21
ATP8B3	46543	AT	14.2	NA	NA	8.6E-21	0.18
ATP8B3	46544	AT	29	NA	NA	8.6E-21	0.18

ITGAE	38490	ES	28	27	29	8.8E-21	0.18
ZNF7	85657	AT	8.2	NA	NA	8.8E-21	0.18
RFX5	7601	AP	2	NA	NA	9.1E-21	0.19
PADI4	851	AT	17	NA	NA	9.1E-21	0.18
PADI4	852	AT	4	NA	NA	9.1E-21	0.18
SULT1A3	94137	AA	11.1	10	11.2	1.1E-20	0.19
DCAKD	41927	AP	1	NA	NA	1.1E-20	0.18
PIWIL4	18357	AP	1	NA	NA	1.2E-20	0.18
RALGPS2	9094	ES	15	14	16	1.2E-20	0.21
CD44	15268	ES	2:10:11:12.1:1	2.1	17.2	1.3E-20	0.19
ITGB1BP1	52617	AA	8.1:8.2	7	8.3	1.3E-20	0.18
XYLB	64043	AT	3	NA	NA	1.3E-20	0.18
XYLB	64044	AT	20	NA	NA	1.3E-20	0.18
CDH23	12059	AT	27.2	NA	NA	1.3E-20	0.18
GPD2	55624	AP	2	NA	NA	1.3E-20	0.19
CSMD2	1689	AT	78	NA	NA	1.4E-20	0.18
SKA2	42757	ES	2	1.1	5	1.5E-20	0.18
GNAS	60003	ES	06:08.1	5	8.2	1.5E-20	0.18
ANKRD30B	44760	AT	31	NA	NA	1.6E-20	0.18
ANKRD30B	44762	AT	42	NA	NA	1.6E-20	0.18
GANC	30142	AT	7	NA	NA	1.6E-20	0.18
ACAP1	38918	AP	21.1	NA	NA	1.7E-20	0.18
HOPX	69364	AP	1.1	NA	NA	1.8E-20	0.18
MBD6	22634	RI	13.4	13.3	13.5	1.8E-20	0.18
WIPF1	56028	AP	2	NA	NA	2.0E-20	0.19
CCDC50	68126	ES	6	5	7	2.1E-20	0.18
CEP57	18391	AD	10.2:10.3	10.1	11	2.1E-20	0.18
TJP3	46727	AP	2	NA	NA	2.1E-20	0.18
DMKN	49144	ES	21	20.1	22	2.2E-20	0.18
FAM195A	32927	ES	3	2	4	2.2E-20	0.18
NUBPL	27126	ES	3	2	4	2.2E-20	0.19
DBNDD1	38196	AP	3.1	NA	NA	2.3E-20	0.18
DMKN	49134	AT	14	NA	NA	2.4E-20	0.18
DENND2A	82009	AT	20	NA	NA	2.4E-20	0.18
DENND2A	82008	AT	14.2	NA	NA	2.4E-20	0.18
HOPX	69367	AP	4.1	NA	NA	2.6E-20	0.18
OSBPL1A	44876	AP	1.1	NA	NA	2.7E-20	0.18
CYHR1	85617	AT	6	NA	NA	2.7E-20	0.18
CYHR1	85616	AT	2.2	NA	NA	2.7E-20	0.18
ADAP1	78553	AP	2	NA	NA	2.8E-20	0.20
GPD2	55623	AP	1	NA	NA	2.8E-20	0.19
HMHA1	46375	AP	15.1	NA	NA	2.8E-20	0.18
OBSL1	57728	AT	9	NA	NA	2.9E-20	0.18
KSR1	39851	AA	24.1	22	24.2	3.0E-20	0.18
LARP6	31443	AT	2	NA	NA	3.2E-20	0.18
LARP6	31442	AT	4	NA	NA	3.2E-20	0.18
FBLN1	62663	AT	16	NA	NA	3.7E-20	0.18
MFSD12	46695	AT	15	NA	NA	4.2E-20	0.18
MFSD12	46697	AT	13.3	NA	NA	4.2E-20	0.18
TTC8	28742	ES	10.1	9	11	4.3E-20	0.18
FAM107A	65463	AP	1	NA	NA	4.5E-20	0.18
OSBPL1A	44877	AP	19	NA	NA	4.7E-20	0.18
PIWIL4	18359	AP	2	NA	NA	5.3E-20	0.18

CDC42SE1	7556	ES	5	4	6	5.5E-20	0.18
LRRC23	19998	AT	7.2	NA	NA	5.6E-20	0.18
LRRC23	20000	AT	10	NA	NA	5.6E-20	0.18
FLT3LG	50943	AT	10	NA	NA	5.8E-20	0.18
ISLR	31677	AP	1	NA	NA	6.0E-20	0.18
ISLR	31676	AP	2	NA	NA	6.0E-20	0.18
TMEM107	39135	ES	2:3.2:3.4	1	3.7	6.2E-20	0.18
TMEM161B	72730	AT	12.2	NA	NA	6.4E-20	0.18
TMEM161B	72731	AT	13	NA	NA	6.4E-20	0.18
PFKFB2	9613	AP	1	NA	NA	6.4E-20	0.19
APOLD1	20516	AT	4	NA	NA	6.8E-20	0.18
APOLD1	20517	AT	17	NA	NA	6.8E-20	0.18
FAM86B1	82715	AA	3.1	1.2	3.2	7.4E-20	0.18
CHCHD4	63518	ES	2	1	4	7.4E-20	0.18
PFKFB2	9616	AT	18	NA	NA	8.4E-20	0.18
PFKFB2	9617	AT	17	NA	NA	8.4E-20	0.18
GBA2	86285	AP	2.1	NA	NA	8.9E-20	0.18
GBA2	86283	AP	1	NA	NA	8.9E-20	0.18
C16orf91	33097	RI	4.2	4.1	4.3	9.0E-20	0.18
SNX14	76924	ES	16	15	17	9.2E-20	0.18
FGD3	86889	AP	12	NA	NA	9.7E-20	0.19
SP140	57870	AT	28	NA	NA	1.0E-19	0.18
BCCIP	13431	AT	7	NA	NA	1.1E-19	0.17
GMEB2	60137	AP	2.1	NA	NA	1.1E-19	0.18
TNC	87340	ES	12:13:14:15	11	16	1.1E-19	0.20
CLSPN	1731	AT	26	NA	NA	1.2E-19	0.17
CLSPN	1730	AT	25	NA	NA	1.2E-19	0.17
SLC16A3	44301	AP	4.1	NA	NA	1.2E-19	0.17
RUFY3	69446	AP	3	NA	NA	1.2E-19	0.18
DMKN	49138	AT	22	NA	NA	1.3E-19	0.17
UNK	43509	AT	5	NA	NA	1.3E-19	0.17
SLC16A3	44300	AP	2	NA	NA	1.4E-19	0.17
GMEB2	60138	AP	1	NA	NA	1.5E-19	0.18
ARHGEF10L	858	AP	10	NA	NA	1.6E-19	0.19
FBLN1	62666	AT	22	NA	NA	1.7E-19	0.17
FAM86B1	82694	ES	7.1	4	8.1	1.7E-19	0.18
PINLYP	50209	AP	5.1	NA	NA	1.8E-19	0.18
DIO2	28647	AP	1	NA	NA	1.8E-19	0.20
FAM86B1	82689	ES	5:6:7.1:7.3	4	8.1	1.9E-19	0.18
CLSTN1	575	ES	11	10	12	1.9E-19	0.17
GRK4	68625	AT	2	NA	NA	1.9E-19	0.17
GRK4	68624	AT	17	NA	NA	1.9E-19	0.17
DMKN	49143	ES	20.2:21	20.1	22	2.0E-19	0.17
DCAKD	41928	AP	2	NA	NA	2.1E-19	0.17
CAPN3	30157	ES	23:24	21	25	2.1E-19	0.18
MAP2K6	43187	AP	2	NA	NA	2.4E-19	0.19
MAP2K6	43186	AP	1	NA	NA	2.4E-19	0.19
MBNL1	67324	ES	8	7	9	2.5E-19	0.18
NAV1	9389	AP	3.1	NA	NA	2.5E-19	0.19
S100A4	7711	AP	2	NA	NA	2.6E-19	0.17
ACTG1	44121	RI	1.3:1.4	1.2	1.5	2.7E-19	0.17
S100A4	7713	AP	4	NA	NA	2.9E-19	0.17
CABIN1	61387	AP	2	NA	NA	3.3E-19	0.18

BRD8	73509	ES	10	9	11	3.4E-19	0.17
DUOXA2	30383	AA	2.1	1	2.2	3.5E-19	0.17
PAM	72894	ES	23.2:25.2	22	26.1	3.7E-19	0.17
DIO2	28649	AP	3.1	NA	NA	3.8E-19	0.20
TIMM8B	18730	ES	2	1	3	3.8E-19	0.17
GOLGA8M	29753	AT	18	NA	NA	4.3E-19	0.17
GOLGA8M	29752	AT	12.2	NA	NA	4.3E-19	0.17
SBF1	62828	ES	29	28	30	4.5E-19	0.17
GBA2	86290	RI	14.3	14.2	14.4	4.5E-19	0.17
NAA60	33528	RI	10.4:10.5	10.3	10.6	4.7E-19	0.17
ABCB8	82287	AT	11.2	NA	NA	4.7E-19	0.17
ABCB8	82288	AT	18	NA	NA	4.7E-19	0.17
ARHGEF11	8338	ES	39	38	40	4.7E-19	0.17
CAST	72838	AP	6	NA	NA	5.1E-19	0.17
IYD	78139	AT	7	NA	NA	5.5E-19	0.17
IYD	78140	AT	6.4	NA	NA	5.5E-19	0.17
WTAP	78310	AP	1	NA	NA	5.7E-19	0.17
WTAP	78311	AP	2	NA	NA	5.7E-19	0.17
TCHP	24365	AT	16	NA	NA	6.1E-19	0.17
TCHP	24366	AT	14.2	NA	NA	6.1E-19	0.17
PFKFB3	10690	AP	3	NA	NA	6.1E-19	0.19
PAM	72891	ES	23.2:25.1:25.2	22	26.1	6.7E-19	0.17
CD44	15274	ES	10:11:12.1:13	2.1	17.2	6.7E-19	0.18
MAGED2	89247	AP	1	NA	NA	6.7E-19	0.17
PIDD	13764	AP	1	NA	NA	7.0E-19	0.17
PIDD	13765	AP	2.1	NA	NA	7.0E-19	0.17
NUMB	28288	ES	13	12	14	7.3E-19	0.17
ABCC6	34220	AT	32	NA	NA	7.4E-19	0.17
ABCC6	34219	AT	2.2	NA	NA	7.4E-19	0.17
EGFL7	88188	AP	4	NA	NA	8.1E-19	0.17
RAPGEF2	71001	ES	17	16	18	8.6E-19	0.19
UNK	43507	AT	21	NA	NA	8.7E-19	0.17
GGT1	61437	AA	15.3	14	15.4	8.7E-19	0.17
DMKN	49145	AD	20.2	20.1	22	8.8E-19	0.17
IVNS1ABP	9218	ES	9	8	10	8.8E-19	0.17
DLG1	68295	ES	9	7	10	9.4E-19	0.18
FAM86C1	17436	ES	4	3.2	5.1	9.8E-19	0.17
PRDM1	77110	AP	1.1	NA	NA	1.0E-18	0.17
PRDM1	77111	AP	4	NA	NA	1.0E-18	0.17
RNF167	94344	ES	2.2:2.3	1.2	2.5	1.1E-18	0.19
RARA	40858	AP	5	NA	NA	1.1E-18	0.17
LYRM1	34402	AP	2.1	NA	NA	1.1E-18	0.17
LMO7	26065	AA	19.1	18	19.2	1.1E-18	0.17
TSPAN14	12366	AP	1	NA	NA	1.1E-18	0.17
TSPAN14	12367	AP	2	NA	NA	1.1E-18	0.17
SLC6A12	19581	AP	3	NA	NA	1.1E-18	0.19
LCN10	123359	ES	4.2	2.2	5	1.3E-18	0.19
LLGL2	43461	AT	12.2	NA	NA	1.3E-18	0.17
LLGL2	43460	AT	28.2	NA	NA	1.3E-18	0.17
NWD1	48207	AD	7.2	7.1	8	1.4E-18	0.17
FDFT1	82641	AP	2.1	NA	NA	1.4E-18	0.17
EGFL7	88186	AP	2	NA	NA	1.5E-18	0.17
SRSF4	1424	ES	6	5	7	1.5E-18	0.17

ERICH1	82554	AT	7	NA	NA	1.5E-18	0.17
ERICH1	82553	AT	6	NA	NA	1.5E-18	0.17
IMMP1L	14817	ES	4:05:06	1	7	1.5E-18	0.17
PCSK5	86633	AT	38	NA	NA	1.6E-18	0.17
PPP1R13L	50436	AP	1	NA	NA	1.6E-18	0.17
PPP1R13L	50435	AP	2	NA	NA	1.6E-18	0.17
ARSA	62897	RI	1.3:1.4	1.2	1.5	1.6E-18	0.17
PHLDB2	66064	ES	14:15	13	16	1.7E-18	0.17
CHCHD7	83914	ES	2.1	1	4	1.8E-18	0.16
PILRB	80935	ES	10	9	11	1.8E-18	0.17
ARSG	43135	AP	1	NA	NA	1.9E-18	0.18
ARSG	43136	AP	2	NA	NA	1.9E-18	0.18
CORIN	69182	AT	16.2	NA	NA	1.9E-18	0.17
CORIN	69181	AT	23	NA	NA	1.9E-18	0.17
PDE4D	72138	AP	20	NA	NA	2.0E-18	0.17
EPS15L1	48160	ES	22:23.1	21	24	2.0E-18	0.16
UAP1	8750	ES	9.1:9.2	8	10	2.0E-18	0.16
TMUB2	41810	ES	2.3:2.4:2.5:3	2.2	4.3	2.1E-18	0.17
DYX1C1	30734	AT	12	NA	NA	2.1E-18	0.16
S100A14	7727	AP	3	NA	NA	2.1E-18	0.17
S100A14	7729	AP	1	NA	NA	2.1E-18	0.17
MYO19	40481	ES	23	22	24	2.1E-18	0.17
ZC3HAV1	81939	AT	9.2	NA	NA	2.3E-18	0.16
ZC3HAV1	81940	AT	13	NA	NA	2.3E-18	0.16
BIN1	55193	ES	16:17	12	18	2.5E-18	0.19
C2orf81	54051	AP	2.1	NA	NA	2.5E-18	0.16
C2orf81	54050	AP	1	NA	NA	2.5E-18	0.16
NEU3	17812	AT	6	NA	NA	2.7E-18	0.16
ATP5S	27466	AT	7.2	NA	NA	2.9E-18	0.16
SMCO4	18299	AP	1	NA	NA	3.0E-18	0.16
SMCO4	18300	AP	2	NA	NA	3.0E-18	0.16
PFKFB3	10692	AP	1	NA	NA	3.2E-18	0.18
SMTN	61810	AP	12	NA	NA	3.2E-18	0.17
SMTN	61811	AP	1	NA	NA	3.2E-18	0.17
SLC6A12	19579	AP	1	NA	NA	3.3E-18	0.18
ZSCAN20	1679	AT	8	NA	NA	3.4E-18	0.16
ZSCAN20	1678	AT	6.2	NA	NA	3.4E-18	0.16
TP53I3	52811	RI	1.2	1.1	1.3	3.4E-18	0.16
YBX3	20481	ES	6	5	7	3.5E-18	0.16
BMP1	82988	ES	17.1:17.2	16	18.1	3.5E-18	0.16
PTBP2	3832	ES	12	11.2	13	4.2E-18	0.18
MRPL52	26637	ES	4.1:4.2	2	5	4.2E-18	0.16
ZNF215	14189	AT	7.2	NA	NA	4.3E-18	0.16
ZNF215	14188	AT	8	NA	NA	4.3E-18	0.16
MYO1G	79577	AT	22	NA	NA	4.5E-18	0.16
MYO1G	79578	AT	12.2	NA	NA	4.5E-18	0.16
VAV1	47087	AP	2	NA	NA	4.6E-18	0.18
VAV1	47088	AP	1	NA	NA	4.6E-18	0.18
SMAGP	21827	AP	1	NA	NA	4.6E-18	0.16
SSPN	20844	AP	2.1	NA	NA	4.9E-18	0.17
SSPN	20842	AP	1	NA	NA	4.9E-18	0.17
MGAT1	75019	AP	2	NA	NA	4.9E-18	0.16
INPP5F	13270	AP	16	NA	NA	5.0E-18	0.16

MYO1C	38307	AP	1	NA	NA	5.1E-18	0.16
ZBTB16	18812	AP	2	NA	NA	5.3E-18	0.18
ZBTB16	18811	AP	1	NA	NA	5.3E-18	0.18
PTPRE	13459	AP	3	NA	NA	5.4E-18	0.19
DDX52	40542	ES	3	2	5	5.6E-18	0.17
CACNB3	21468	AP	1	NA	NA	5.6E-18	0.16
AP1S2	88569	AT	5.2	NA	NA	5.6E-18	0.16
AP1S2	88571	AT	6	NA	NA	5.6E-18	0.16
MEIS3	50645	AA	7.1	6	7.2	5.8E-18	0.17
ARHGEF9	89301	AP	1	NA	NA	6.1E-18	0.17
ARHGEF9	89302	AP	2.1	NA	NA	6.1E-18	0.17
MCC	73004	AP	1	NA	NA	6.2E-18	0.18
FBXO7	61930	AP	1	NA	NA	6.3E-18	0.16
FBXO7	61931	AP	2	NA	NA	6.3E-18	0.16
NASP	2706	ES	9	8	10	6.4E-18	0.16
RNH1	13674	ES	03:04.2	2	4.3	6.5E-18	0.16
MORF4L2	89771	ES	4:5.1:5.2:5.3	3.2	6.2	6.6E-18	0.16
EMC10	51201	ES	7	6	8.1	6.7E-18	0.16
SLC25A45	16820	AP	3.1	NA	NA	6.7E-18	0.16
URGCP	79354	AP	3	NA	NA	7.3E-18	0.16
PUS10	53675	AT	20	NA	NA	7.5E-18	0.16
PUS10	53676	AT	4.2	NA	NA	7.5E-18	0.16
FAM86B1	82706	ES	4:5:6:7.1	3.2	8.1	8.2E-18	0.16
KCTD7	79882	ES	13	12	14	8.8E-18	0.16
ZXDC	66597	AT	10	NA	NA	8.8E-18	0.16
ZXDC	66596	AT	6.2	NA	NA	8.8E-18	0.16
NR1H3	15692	AP	1	NA	NA	8.9E-18	0.17
RNH1	13677	ES	3	1	4.2	8.9E-18	0.16
SLA	85211	AP	1	NA	NA	9.0E-18	0.18
SLA	85212	AP	3.1	NA	NA	9.0E-18	0.18
CTAGE5	27371	AP	1	NA	NA	9.4E-18	0.16
FAM134B	71598	AP	1	NA	NA	9.7E-18	0.17
FAM134B	71599	AP	4	NA	NA	9.7E-18	0.17
PFDN5	21992	ES	02:04.1	1	4.2	9.8E-18	0.16
CAV1	81529	AP	2.1	NA	NA	1.0E-17	0.16
MALT1	45679	ES	7	6	8	1.0E-17	0.17
CAV1	81528	AP	1.1	NA	NA	1.0E-17	0.16
KIAA0195	43444	AP	1	NA	NA	1.0E-17	0.17
KIAA0195	43443	AP	3	NA	NA	1.0E-17	0.17
CALD1	81858	AP	5	NA	NA	1.1E-17	0.16
GIT2	24370	ES	19	18.2	20	1.1E-17	0.16
ANK3	11842	AP	1	NA	NA	1.1E-17	0.17
ATP5S	27467	AT	5.2	NA	NA	1.2E-17	0.16
CEP78	86656	AT	16.2	NA	NA	1.2E-17	0.16
CEP78	86655	AT	17	NA	NA	1.2E-17	0.16
ISOC2	52105	ES	03:04.1	2	4.2	1.2E-17	0.16
C6orf141	76447	AT	6	NA	NA	1.2E-17	0.16
C8orf34	84098	AT	16	NA	NA	1.2E-17	0.16
COX4I1	156373	AA	5.1:5.2:5.3	4.1	5.4	1.2E-17	0.16
SLC29A1	76356	AP	3.1	NA	NA	1.3E-17	0.16
SLC2A4	38901	AT	11.2	NA	NA	1.3E-17	0.16
SLC2A4	38902	AT	12	NA	NA	1.3E-17	0.16
C6orf132	76170	AT	3	NA	NA	1.4E-17	0.16

C6orf132	76171	AT	6	NA	NA	1.4E-17	0.16
LARP1B	70566	AT	9.3	NA	NA	1.5E-17	0.16
FAM180A	81892	AT	4	NA	NA	1.5E-17	0.18
FAM180A	81893	AT	3.3	NA	NA	1.5E-17	0.18
MCM7	80881	AP	1	NA	NA	1.5E-17	0.16
MCM7	80880	AP	2	NA	NA	1.5E-17	0.16
RNH1	13672	AA	4.2	3	4.3	1.6E-17	0.16
GOLGA8M	29754	RI	5.2	5.1	5.3	1.6E-17	0.16
CALD1	81856	AP	2	NA	NA	1.7E-17	0.16
PSPC1	25404	AT	12	NA	NA	1.7E-17	0.16
KHDC1	76735	AP	1	NA	NA	1.8E-17	0.16
KHDC1	76736	AP	3	NA	NA	1.8E-17	0.16
BDNF	14758	AP	11.1	NA	NA	1.9E-17	0.17
HHLA3	3402	ES	4	3	5	2.0E-17	0.16
PAM	72906	ES	14	13	15	2.1E-17	0.16
NPIPB4	35509	ES	3.1:3.2:3.3	2.5	4	2.2E-17	0.16
TRAK1	64269	AT	19	NA	NA	2.4E-17	0.16
SYK	86821	ES	9	8	10	2.5E-17	0.16
TGFB1I1	36268	AP	1	NA	NA	2.6E-17	0.16
IGBP1	89367	RI	1.2	1.1	1.3	2.7E-17	0.16
GOLGA8J	93641	ES	5.2:6.1	5.1	6.2	2.9E-17	0.16
COX20	10475	ES	3	1	4	2.9E-17	0.15
UBXN2A	52794	AP	3	NA	NA	3.0E-17	0.15
UBXN2A	52795	AP	1	NA	NA	3.0E-17	0.15
ARPC1A	80601	AT	12	NA	NA	3.0E-17	0.15
ARPC1A	80602	AT	15	NA	NA	3.0E-17	0.15
EPHB6	99887	ES	3	2	4.2	3.0E-17	0.18
NADK	225	AP	3	NA	NA	3.6E-17	0.16
MRPL55	10148	ES	2.2:2.3:2.4:2.5	1.1	2.9	3.7E-17	0.15
UPP1	79637	ES	6.2:7	6.1	9	3.7E-17	0.16
CRELD1	63293	RI	1.2:1.3	1.1	1.4	4.0E-17	0.15
PFKFB2	9614	AP	10.1	NA	NA	4.1E-17	0.17
SCRIB	98107	ES	17	16	18	4.1E-17	0.16
TRAK1	64268	AT	17.2	NA	NA	4.2E-17	0.15
OSBP2	61799	AP	3	NA	NA	4.4E-17	0.16
HYDIN	37418	AT	16.3	NA	NA	4.5E-17	0.16
RASSF5	9585	AP	1	NA	NA	4.5E-17	0.16
RASSF5	9586	AP	3	NA	NA	4.5E-17	0.16
CAMK1D	10773	AT	10.2	NA	NA	4.7E-17	0.15
CAMK1D	10772	AT	11	NA	NA	4.7E-17	0.15
NSRP1	40102	ES	3	1	8	5.2E-17	0.18
FAM71D	28039	AT	6	NA	NA	5.3E-17	0.16
MID1	88461	AP	3.1	NA	NA	5.3E-17	0.19
MAFF	62216	AP	3	NA	NA	5.5E-17	0.15
SNAPC5	31277	ES	1.2:2.1	1.1	3.1	5.9E-17	0.15
C14orf159	28858	ES	6	5.2	7	6.0E-17	0.16
UGDH	69064	AP	1	NA	NA	6.1E-17	0.16
RTKN2	11870	AT	5.2	NA	NA	6.2E-17	0.15
HSD11B1L	46870	AT	6	NA	NA	6.4E-17	0.15
HSD11B1L	46868	AT	11.2	NA	NA	6.4E-17	0.15
STAMBPL1	12471	AT	11	NA	NA	6.7E-17	0.15
STAMBPL1	12472	AT	12	NA	NA	6.7E-17	0.15
CCDC40	44017	AT	23	NA	NA	6.9E-17	0.15

ZNF438	11129	AP	2	NA	NA	7.2E-17	0.18
ZNF438	11127	AP	1	NA	NA	7.2E-17	0.18
MFF	57807	ES	9:10	7	11	7.7E-17	0.15
IL18BP	17481	RI	1.7	1.6	1.8	7.7E-17	0.16
ZNF124	10515	AT	4.2	NA	NA	7.9E-17	0.15
TTLL11	87448	AT	4.2	NA	NA	8.2E-17	0.15
TTLL11	87447	AT	9	NA	NA	8.2E-17	0.15
PHF19	87400	AP	7	NA	NA	8.2E-17	0.16
PHF19	87399	AP	1	NA	NA	8.2E-17	0.16
PIK3R1	72294	AP	1.1	NA	NA	8.4E-17	0.16
SLC29A1	76359	AP	1	NA	NA	8.6E-17	0.15
GLS2	22441	AA	9.1	8.3	9.2	8.9E-17	0.16
TARBP2	22068	AP	2	NA	NA	9.2E-17	0.16
TARBP2	22067	AP	1.1	NA	NA	9.2E-17	0.16
NNMT	18813	AP	3.1	NA	NA	9.4E-17	0.18
LMO3	20630	AD	4.2:4.3	4.1	6.3	9.6E-17	0.15
HDAC8	89464	AT	9	NA	NA	1.0E-16	0.15
RDH13	52001	AT	12	NA	NA	1.0E-16	0.15
RDH13	52000	AT	10	NA	NA	1.0E-16	0.15
SLC11A2	21729	AT	23.3	NA	NA	1.0E-16	0.15
SLC11A2	21730	AT	22.2	NA	NA	1.0E-16	0.15
PINLYP	50208	AP	4	NA	NA	1.1E-16	0.16
MBNL1	67318	ES	10	9	11	1.1E-16	0.16
SHC1	7855	AP	2.1	NA	NA	1.1E-16	0.15
SHC1	7854	AP	1	NA	NA	1.1E-16	0.15
KIAA0513	37877	AT	9.2	NA	NA	1.2E-16	0.15
KIAA0513	37878	AT	14	NA	NA	1.2E-16	0.15
CCDC84	19052	AD	6.2	6.1	7	1.2E-16	0.15
ARHGEF3	65360	AP	10	NA	NA	1.2E-16	0.16
MAFF	62214	AP	1.1	NA	NA	1.3E-16	0.15
PSPC1	25402	AT	9	NA	NA	1.3E-16	0.15
FAM71D	28041	AT	14	NA	NA	1.4E-16	0.15
CD44	15058	ES	:9.2:10:11:12	5	14	1.4E-16	0.16
TGFB1I1	36265	AP	4.1	NA	NA	1.5E-16	0.15
SLC25A37	83082	AA	3.1	2.1	3.2	1.5E-16	0.15
RPS3	17835	AT	7.2	NA	NA	1.5E-16	0.15
ITGB2	60853	ES	2	1	4.2	1.6E-16	0.15
PTK2	85287	AP	1	NA	NA	1.6E-16	0.15
FANCD2	63307	AT	45	NA	NA	1.7E-16	0.15
PKP2	21062	ES	6	5	7	1.9E-16	0.17
CDC14B	86972	AP	2	NA	NA	1.9E-16	0.17
CDC14B	86971	AP	1	NA	NA	1.9E-16	0.17
CYB561A3	16164	AA	6.6	6.4	6.7	2.0E-16	0.15
DNM1L	21046	ES	18	16	19	2.0E-16	0.15
NFKBIB	49718	RI	5.2	5.1	5.3	2.0E-16	0.15
APOC2	50375	RI	4.4	4.3	4.5	2.1E-16	0.16
DTNB	52862	ES	21	20	22	2.2E-16	0.15
LRRC32	17897	AP	1	NA	NA	2.2E-16	0.15
LRRC32	17898	AP	2	NA	NA	2.2E-16	0.15
NDUFV3	60762	ES	3	2	4	2.3E-16	0.15
C9orf9	87993	AP	1	NA	NA	2.5E-16	0.15
C9orf9	87992	AP	2	NA	NA	2.5E-16	0.15
METTL21A	57183	AP	1	NA	NA	2.6E-16	0.15

NT5DC2	65224	AP	1.1	NA	NA	2.7E-16	0.15
NT5DC2	65225	AP	2	NA	NA	2.7E-16	0.15
LIMS2	55227	AP	2	NA	NA	2.7E-16	0.15
BPGM	81853	ES	3	1	4	2.8E-16	0.15
FAM49B	85137	AP	2	NA	NA	2.8E-16	0.15
ZNF655	80660	AP	2	NA	NA	2.9E-16	0.15
ZNF655	80659	AP	1	NA	NA	2.9E-16	0.15
RPRD2	7472	ES	4	3.1	5	3.0E-16	0.16
GBA2	86287	RI	15.2:15.3	15.1	15.4	3.2E-16	0.15
SYNGR1	62299	AT	8	NA	NA	3.3E-16	0.15
GRK6	74765	AA	17.1	15	17.2	3.4E-16	0.15
CHN2	79084	AP	1	NA	NA	3.4E-16	0.15
TMC6	43754	AP	3	NA	NA	3.5E-16	0.15
LGMN	29011	ES	2	1	3	3.5E-16	0.15
LCN10	88199	ES	03:04.2	2.2	5	3.5E-16	0.15
RAPH1	57074	AT	18	NA	NA	3.7E-16	0.15
UGDH	69065	AP	2	NA	NA	3.7E-16	0.15
ZNF385A	22177	AP	4.1	NA	NA	3.8E-16	0.15
EXOC7	43573	ES	7	6	9	3.8E-16	0.15
DGKZ	15541	AP	2	NA	NA	3.9E-16	0.15
ERBB2	40677	AP	6	NA	NA	3.9E-16	0.15
FADS2	16289	AP	2.1	NA	NA	4.0E-16	0.15
GINS4	83515	AT	6.3	NA	NA	4.1E-16	0.15
GINS4	83516	AT	3.2	NA	NA	4.1E-16	0.15
DEDD	8558	AP	2	NA	NA	4.2E-16	0.15
DEDD	8560	AP	1	NA	NA	4.2E-16	0.15
CCL28	71944	AP	1	NA	NA	4.2E-16	0.15
CCL28	71945	AP	2	NA	NA	4.2E-16	0.15
ZNF365	11879	AT	5	NA	NA	4.3E-16	0.15
C2CD3	17761	AT	8	NA	NA	4.4E-16	0.14
FAM86B1	82693	ES	06:07.1	4	8.1	4.5E-16	0.15
SLC7A2	82807	ME	9|10	8	11	4.7E-16	0.16
UACA	31439	AP	1	NA	NA	5.0E-16	0.15
UACA	31438	AP	2	NA	NA	5.0E-16	0.15
VPS13A	86648	AT	76	NA	NA	5.1E-16	0.14
RAPH1	57075	AT	17	NA	NA	5.2E-16	0.14
MAST4	72277	AP	10	NA	NA	5.3E-16	0.15
NDUFAF6	84596	ES	12	11	14	5.3E-16	0.14
TRMT10A	70055	AP	2	NA	NA	5.3E-16	0.16
TRMT10A	70054	AP	1	NA	NA	5.3E-16	0.16
PLEKHA4	50820	ES	18	17	19	5.3E-16	0.15
SKA2	42754	ES	02:04.1	1.1	5	5.4E-16	0.14
RAB9A	88512	ES	2	1	3	5.4E-16	0.15
LYNX1	85364	AP	2	NA	NA	5.5E-16	0.15
RPS24	12297	AA	5.1	4	5.2	5.7E-16	0.14
C16orf46	37750	AT	4.2	NA	NA	5.7E-16	0.14
C16orf46	37751	AT	5	NA	NA	5.7E-16	0.14
RABEP2	35894	ES	3	2.2	4	6.2E-16	0.14
QKI	78405	AT	9	NA	NA	6.3E-16	0.14
QKI	78404	AT	8.7	NA	NA	6.3E-16	0.14
DNAH2	39053	AT	14.2	NA	NA	6.4E-16	0.14
DNAH2	39052	AT	88	NA	NA	6.4E-16	0.14
C2CD3	17760	AT	35	NA	NA	6.4E-16	0.14

CLASP1	55177	ES	22	21	24	6.8E-16	0.17
KIF6	76048	AT	25	NA	NA	7.1E-16	0.14
KIF6	76049	AT	24	NA	NA	7.1E-16	0.14
CD44	15107	ES	6:7:8:9.2	5	10	7.4E-16	0.15
NAV1	9387	AP	6	NA	NA	7.5E-16	0.16
SERPINA1	29137	ES	2.5	1.1	3.2	7.7E-16	0.15
ATP2C1	66758	AP	2	NA	NA	7.9E-16	0.16
NPIPA5	34148	RI	8.2	8.1	8.3	7.9E-16	0.14
TSPAN4	13792	AP	1	NA	NA	8.0E-16	0.14
MLPH	58111	AP	1	NA	NA	8.0E-16	0.14
MLPH	58112	AP	2.1	NA	NA	8.0E-16	0.14
ERBB2	40678	AP	1	NA	NA	8.5E-16	0.14
PLS3	89920	AP	2	NA	NA	8.7E-16	0.14
PLS3	89921	AP	1	NA	NA	8.7E-16	0.14
EPHA4	57747	AP	1	NA	NA	8.9E-16	0.15
EPHA4	57746	AP	2.1	NA	NA	9.0E-16	0.15
CD44	15133	ES	0:11:12.1:13:1	5	15	9.0E-16	0.15
HDAC8	89459	AT	14.2	NA	NA	9.1E-16	0.14
NDUFAF6	84598	ES	9.2:10	9.1	11	9.3E-16	0.14
INPP5F	13271	AP	1	NA	NA	9.5E-16	0.14
SEPT8	73303	AA	12.1	11	12.2	9.6E-16	0.14
NNMT	18817	AP	1	NA	NA	9.7E-16	0.17
SIAH1	36340	AP	6	NA	NA	9.7E-16	0.14
KIFC3	36604	AP	8	NA	NA	9.9E-16	0.14
UGT1A1	58054	AT	10	NA	NA	1.0E-15	0.15
UGT1A1	58055	AT	9	NA	NA	1.0E-15	0.15
FGFR1	83430	ES	6	4	8.2	1.0E-15	0.15
LIG4	26234	AP	1	NA	NA	1.1E-15	0.15
NFIC	46675	AP	1	NA	NA	1.1E-15	0.15
NFIC	46676	AP	2	NA	NA	1.1E-15	0.15
MFSD6	56564	AP	1	NA	NA	1.2E-15	0.16
TSHZ2	59816	AP	2	NA	NA	1.2E-15	0.14
TSHZ2	59817	AP	1	NA	NA	1.2E-15	0.14
METTL15	14787	AA	10.1	9	10.2	1.2E-15	0.15
RPS3A	96814	ES	2.2	1.3	4.1	1.2E-15	0.15
FAM178A	12839	AT	1.2	NA	NA	1.2E-15	0.14
RNF165	45394	AT	8	NA	NA	1.2E-15	0.14
RNF165	45395	AT	1.5	NA	NA	1.2E-15	0.14
PFDN5	22007	ES	2:4.1:4.2	1	5	1.3E-15	0.14
EHBP1	53718	ES	18	17	19	1.3E-15	0.14
MFSD6	56566	AP	4	NA	NA	1.3E-15	0.16
ST7	81563	ES	10	9	11.1	1.3E-15	0.14
ARHGEF1	50101	ES	15	14	16	1.3E-15	0.14
RANGRF	39166	AD	3.2:3.3	3.1	3.5	1.3E-15	0.14
NONO	89420	ES	2:03	1	4	1.4E-15	0.15
SPAG9	42494	ES	30	29	31	1.4E-15	0.14
DBNDD1	38197	AP	2	NA	NA	1.4E-15	0.14
METTL21A	57191	AT	7	NA	NA	1.4E-15	0.14
ADORA1	9442	ES	4	3.2	5	1.5E-15	0.15
SEMA4D	86803	AP	20.1	NA	NA	1.5E-15	0.14
CD44	15115	ES	7:8:9.2:10:11	5	12.1	1.5E-15	0.14
CD46	9652	ES	13	12	14	1.5E-15	0.14
D2HGDH	95828	ES	7.1	6	8	1.6E-15	0.15

ZNF814	52354	AT	9	NA	NA	1.7E-15	0.14
AMPD3	14347	AP	1	NA	NA	1.7E-15	0.17
TMSB15B	89787	AT	6	NA	NA	1.7E-15	0.14
ZNF365	11877	AT	15	NA	NA	1.8E-15	0.14
TMEM107	39110	RI	3.3	3.2	3.4	1.8E-15	0.14
SERPING1	15866	AP	1	NA	NA	1.9E-15	0.14
SERPING1	15865	AP	2.1	NA	NA	1.9E-15	0.14
PPP1R7	58339	AD	1.2:1.3	1.1	3	1.9E-15	0.15
SYNCRIP	76933	AT	11.2	NA	NA	1.9E-15	0.14
SYNCRIP	76934	AT	12	NA	NA	1.9E-15	0.14
PTPN4	55140	AP	1	NA	NA	1.9E-15	0.14
PTPN4	55141	AP	14	NA	NA	1.9E-15	0.14
MTHFR	683	AP	1	NA	NA	2.0E-15	0.15
FAM86B1	82685	ES	5	4	6	2.0E-15	0.14
ZNF397	45144	AT	6	NA	NA	2.1E-15	0.14
PRMT2	60964	ES	1.3	1.1	2	2.2E-15	0.14
TMSB4X	88498	AD	1.3	1.2	1.5	2.2E-15	0.16
RPL19	40632	ES	1.2:1.3:2.1	1.1	2.2	2.3E-15	0.14
ANAPC11	44217	ES	3.3:6	3.2	7.2	2.5E-15	0.14
LCN10	123361	ES	3	2.2	4.2	2.6E-15	0.16
RNF43	42671	AT	9.2	NA	NA	2.6E-15	0.14
RNF43	42672	AT	10	NA	NA	2.6E-15	0.14
CD44	15112	ES	:8:9.1:9.2:10:1	5	12.1	2.7E-15	0.14
PI4KA	61189	ES	38:39:40:41:4	36	44	2.7E-15	0.17
ATXN2L	35855	ES	22.2:22.4:22.5	22.1	22.6	2.8E-15	0.14
ZGLP1	47493	RI	1.4	1.3	1.5	2.9E-15	0.15
COX11	42569	RI	3.2	3.1	3.3	2.9E-15	0.14
TACC2	13333	AP	6	NA	NA	2.9E-15	0.14
PHACTR1	75366	AT	13.3	NA	NA	2.9E-15	0.14
MRAS	66989	AP	1	NA	NA	3.0E-15	0.14
ESCO2	83185	AT	11.2	NA	NA	3.1E-15	0.14
ESCO2	83184	AT	14	NA	NA	3.1E-15	0.14
FAM13A	69912	AA	16.1	15	16.2	3.1E-15	0.15
CSMD2	1687	AT	50	NA	NA	3.2E-15	0.14
SLK	13030	ES	13	12	14	3.2E-15	0.14
CYGB	43589	AP	1	NA	NA	3.3E-15	0.14
CENPM	62466	AP	6	NA	NA	3.4E-15	0.14
LIG4	26235	AP	3	NA	NA	3.4E-15	0.14
DGKZ	15540	AP	3	NA	NA	3.4E-15	0.14
PLA2G16	16517	AP	2.1	NA	NA	3.6E-15	0.14
PLA2G16	16516	AP	1	NA	NA	3.6E-15	0.14
FLNA	90565	ES	30	29	31	4.2E-15	0.14
CXCL12	11343	AT	3.3	NA	NA	4.5E-15	0.14
DCAF11	26847	ES	1.2:2.2	1.1	2.4	4.6E-15	0.14
B3GNT5	67799	AP	1	NA	NA	4.7E-15	0.15
CENPK	72213	AT	6.2	NA	NA	4.8E-15	0.14
CENPK	72212	AT	13	NA	NA	4.8E-15	0.14
MPST	62073	ES	4	3	5	4.9E-15	0.14
CHRNB1	38958	AP	11.1	NA	NA	5.1E-15	0.14
DMKN	49168	ES	11:12	7	13	5.1E-15	0.14
DAP3	8123	AD	2.3	2.2	3	5.2E-15	0.14
PXN	24750	AA	14.1	9	14.2	5.2E-15	0.14
MAPK10	69825	ME	12|13	11	14	5.3E-15	0.14

CAPN12	49690	AT	5	NA	NA	5.4E-15	0.14
CAPN12	49691	AT	23	NA	NA	5.4E-15	0.14
PILRA	80941	ES	3	2	4	5.5E-15	0.14
CCND3	76160	AD	3.2	3.1	4	5.7E-15	0.14
LARP1B	70567	AT	21	NA	NA	5.7E-15	0.14
FAM129C	48336	AA	14.1	13	14.2	5.7E-15	0.14
IGFLR1	49257	AP	1.1	NA	NA	5.8E-15	0.16
IGFLR1	49256	AP	2.1	NA	NA	5.8E-15	0.16
CD44	15055	ES	.1:9.2:10:11:1	5	14	5.8E-15	0.14
SCRIB	85500	ES	36	35	37	5.8E-15	0.14
SWI5	87732	AA	3.1	2	3.2	5.9E-15	0.14
PRKDC	83791	ES	81	80	82	5.9E-15	0.14
DMKN	49186	ES	7:11:12	6.4	13	6.4E-15	0.14
ABHD11	80028	ES	5	3	6	6.7E-15	0.14
SESN1	77155	AP	1	NA	NA	7.0E-15	0.14
ZNF655	80663	AT	10	NA	NA	7.1E-15	0.13
ZNF655	80662	AT	7	NA	NA	7.1E-15	0.13
CD44	15104	ES	6:7:8:9.1:9.2	5	10	7.2E-15	0.14
SEC31A	69734	ES	16:17	15	18	7.5E-15	0.14
TPD52L1	77416	ES	08:09.1	6	10	7.7E-15	0.13
FYN	77269	AP	1	NA	NA	7.9E-15	0.14
SHF	30407	AP	1	NA	NA	8.3E-15	0.13
RGS12	68643	ES	19	18	20.1	8.3E-15	0.13
SMAGP	21829	AP	2	NA	NA	8.5E-15	0.14
CD44	15108	ES	08:09.2	5	10	8.6E-15	0.14
TACC2	13344	ES	12	11	13	8.6E-15	0.13
UAP1	8751	ES	9.2	8	10	8.7E-15	0.14
SPATA24	73604	AT	4.3	NA	NA	8.7E-15	0.13
SPATA24	73605	AT	6	NA	NA	8.8E-15	0.13
ABCA2	88259	ES	4.1	3	4.6	8.8E-15	0.16
OSBPL5	13950	AP	1.1	NA	NA	8.8E-15	0.14
PRKAR1B	78508	AP	2	NA	NA	9.3E-15	0.14
TENC1	21923	AP	1	NA	NA	9.4E-15	0.13
SARDH	88078	AT	24	NA	NA	9.4E-15	0.13
KIAA0391	27214	AP	1	NA	NA	9.7E-15	0.14
KIAA0391	27213	AP	2.1	NA	NA	9.7E-15	0.14
TMEM145	50152	AT	14	NA	NA	1.0E-14	0.13
TMEM145	50151	AT	16	NA	NA	1.0E-14	0.13
CTNNBIP1	577	AP	1	NA	NA	1.0E-14	0.14
MTHFD1L	78151	AT	8	NA	NA	1.1E-14	0.13
MTHFD1L	78150	AT	29	NA	NA	1.1E-14	0.13
KIAA0040	9045	AP	1	NA	NA	1.2E-14	0.14
KIAA0040	9046	AP	2	NA	NA	1.2E-14	0.14
GNLY	54377	ES	2.1:2.2	1	3	1.2E-14	0.14
AP1B1	61603	ES	24	23	25	1.2E-14	0.13
ZBTB45	52479	AD	1.2	1.1	3	1.2E-14	0.14
CCDC40	44016	AT	11	NA	NA	1.2E-14	0.13
LIMCH1	69109	AP	2	NA	NA	1.3E-14	0.14
FBLN2	63511	ES	11	10	12	1.3E-14	0.14
AP1G2	26771	RI	1.3	1.2	1.4	1.3E-14	0.13
FGFR2	13318	ES	4	3	5	1.3E-14	0.13
FAM63A	7537	AA	4.2:4.3	2.2	4.4	1.3E-14	0.14
CYGB	43591	AP	2	NA	NA	1.4E-14	0.13

ZNF385A	22176	AP	1	NA	NA	1.4E-14	0.13
CCDC124	48385	AP	1	NA	NA	1.5E-14	0.13
CCDC124	48386	AP	2	NA	NA	1.5E-14	0.13
ZNF197	64376	AT	7	NA	NA	1.5E-14	0.13
ZNF197	64375	AT	8	NA	NA	1.5E-14	0.13
RNF146	77452	ES	03:05.1	2	6	1.6E-14	0.13
SRGAP1	22855	AP	1	NA	NA	1.6E-14	0.13
SRGAP1	22854	AP	2.1	NA	NA	1.6E-14	0.13
WDR96	13040	AT	38	NA	NA	1.6E-14	0.13
PLD3	49892	ES	3	1.2	5.2	1.6E-14	0.13
TDRKH	7661	AT	17	NA	NA	1.6E-14	0.13
HIST2H2BF	7400	AT	1.2	NA	NA	1.6E-14	0.13
LINC00908	45828	AT	3	NA	NA	1.6E-14	0.13
RPL39L	68071	AP	3	NA	NA	1.7E-14	0.13
RPL39L	68070	AP	1	NA	NA	1.7E-14	0.13
DNASE1L1	90576	AD	3.3	3.2	4	1.7E-14	0.13
NF2	61626	ES	16.1	15	17	1.7E-14	0.13
PCDP1	55125	AT	28	NA	NA	1.7E-14	0.16
BIN1	55200	ES	13	12	17	1.8E-14	0.13
SH3KBP1	88641	AP	1	NA	NA	1.8E-14	0.14
RGS12	68637	AP	7.1	NA	NA	1.8E-14	0.13
IER2	47929	AP	2.1	NA	NA	1.8E-14	0.13
IER2	47930	AP	1	NA	NA	1.8E-14	0.13
PSMA2	79316	AT	10	NA	NA	1.9E-14	0.13
MACF1	1881	ES	107	106	108	1.9E-14	0.13
THTPA	26761	AD	1.2:1.3:1.4:1.5	1.1	2	2.0E-14	0.13
MSR1	82778	AT	10.2	NA	NA	2.1E-14	0.13
MSR1	82779	AT	12	NA	NA	2.1E-14	0.13
THTPA	26763	AD	1.2:1.3:1.4	1.1	2	2.1E-14	0.13
STAMBP	53988	AT	12	NA	NA	2.1E-14	0.13
STAMBP	53987	AT	11.2	NA	NA	2.1E-14	0.13
SLC25A25	87693	AP	3.1	NA	NA	2.2E-14	0.13
ATL3	16518	AP	1	NA	NA	2.2E-14	0.16
ATL3	16519	AP	2.1	NA	NA	2.2E-14	0.16
GLS2	22442	RI	8.2	8.1	8.3	2.3E-14	0.14
SESN1	77156	AP	2	NA	NA	2.3E-14	0.13
MTHFR	682	AP	4.1	NA	NA	2.4E-14	0.14
LINC00908	45829	AT	4	NA	NA	2.4E-14	0.13
DBF4B	41884	AT	13.2	NA	NA	2.4E-14	0.13
DBF4B	41883	AT	14.3	NA	NA	2.4E-14	0.13
TCTN1	24458	AT	19	NA	NA	2.5E-14	0.13
TCTN1	24461	AT	7.3	NA	NA	2.5E-14	0.13
SEC31A	69731	ES	26.1:26.2	25.1	28	2.5E-14	0.13
SLC9A3R2	33187	AP	1	NA	NA	2.5E-14	0.13
VGLL4	63395	AP	6	NA	NA	2.5E-14	0.13
ANKLE2	25300	RI	1.2	1.1	1.3	2.5E-14	0.13
AP1G2	99354	ES	5	4	6	2.5E-14	0.13
GGT1	61432	RI	17.3:17.4	17.2	17.5	2.6E-14	0.13
PCGF3	68404	RI	5.2	5.1	5.3	2.7E-14	0.13
PPIP5K1	30267	ES	31	30	32	2.8E-14	0.13
EMID1	61575	ME	14|15	13	16	2.8E-14	0.13
SYNGR1	62298	AT	7	NA	NA	2.9E-14	0.13
CYP4B1	2829	AP	1	NA	NA	2.9E-14	0.13

CYP4B1	2830	AP	3.1	NA	NA	2.9E-14	0.13
PTGER3	3415	AT	4	NA	NA	3.0E-14	0.13
OSGEP	26442	AD	4.5	4.4	5	3.0E-14	0.13
RAI14	71716	ES	18	17	19	3.0E-14	0.14
PSMG4	75171	AT	5.6	NA	NA	3.2E-14	0.13
APP	60283	ES	10	9	11	3.2E-14	0.13
CLU	83171	RI	3.2	3.1	3.3	3.3E-14	0.14
C11orf80	17129	ES	2:03	1	4	3.4E-14	0.13
MCMDC2	84041	AT	15	NA	NA	3.4E-14	0.13
DERL3	61334	RI	5.3	5.2	5.4	3.4E-14	0.13
FAM47E	69604	AD	6.2	6.1	7	3.4E-14	0.13
RAB30	18041	AP	3.1	NA	NA	3.4E-14	0.15
DNM1L	21045	ES	17:18	16	19	3.4E-14	0.13
GLB1L	57659	ES	6:07	5	8	3.5E-14	0.13
TCF20	62502	ES	4.2	3	5	3.5E-14	0.13
CGREF1	52934	AT	7.4	NA	NA	3.5E-14	0.13
CGREF1	52933	AT	9	NA	NA	3.5E-14	0.13
ZBTB8OS	1614	ES	2:03:04	1	6	3.6E-14	0.14
FAM104B	89276	AP	1	NA	NA	3.7E-14	0.13
FAM104B	89275	AP	2.1	NA	NA	3.7E-14	0.13
SDCBP2	58484	AP	1	NA	NA	3.7E-14	0.13
CADM1	18849	ES	11	10	12	3.7E-14	0.13
SERPINA1	29127	ES	2.1	1.1	2.4	4.0E-14	0.15
CD44	15143	ES	12.1:13:14	5	15	4.3E-14	0.13
NDUFS7	46465	RI	9.2	9.1	9.3	4.4E-14	0.13
BSCL2	16400	AP	4.1	NA	NA	4.4E-14	0.13
CCDC65	21489	AT	8.2	NA	NA	4.4E-14	0.13
CCDC65	21490	AT	9	NA	NA	4.4E-14	0.13
CXCL12	11342	AT	5.2	NA	NA	4.4E-14	0.13
PDDC1	13755	ES	4.1:4.2	3	5	4.5E-14	0.15
NMRK1	86630	ES	3	2	4	4.5E-14	0.13
WIBG	22288	AP	3	NA	NA	4.5E-14	0.13
TCF7	73350	ES	6	5	7	4.6E-14	0.14
MFSD11	43692	AD	2.2	2.1	2.6	4.6E-14	0.15
C6orf203	77125	ES	1.2:2	1.1	3	4.6E-14	0.13
COASY	41068	RI	1.2:1.3:1.4	1.1	1.5	4.8E-14	0.13
FAM69B	88195	AP	1	NA	NA	4.8E-14	0.13
FAM69B	88196	AP	3.1	NA	NA	4.8E-14	0.13
ARHGEF7	26280	AP	2.1	NA	NA	4.8E-14	0.15
THTPA	26762	ES	1.3:1.4:1.5	1.1	2	4.9E-14	0.13
GBP3	3709	AD	5.2	5.1	6	5.0E-14	0.13
CSF2RA	88361	AT	16	NA	NA	5.0E-14	0.13
CSF2RA	88362	AT	17.2	NA	NA	5.0E-14	0.13
TBC1D23	65817	ES	15	14	16	5.2E-14	0.13
S100A5	7709	AP	3	NA	NA	5.2E-14	0.13
S100A5	7710	AP	1	NA	NA	5.2E-14	0.13
PTOV1	51095	AP	2	NA	NA	5.3E-14	0.13
PTOV1	51094	AP	1	NA	NA	5.3E-14	0.13
RNF6	25512	AP	1	NA	NA	5.3E-14	0.13
KIFC3	36606	AP	12	NA	NA	5.5E-14	0.13
YIF1B	49607	AT	10	NA	NA	5.5E-14	0.13
YIF1B	49606	AT	9.2	NA	NA	5.5E-14	0.13
EIF3C	35825	AP	1	NA	NA	5.6E-14	0.13

EIF3C	35827	AP	2.1	NA	NA	5.6E-14	0.13
EVL	29240	AP	2	NA	NA	5.8E-14	0.13
PAM	72902	ES	23.2	22	25.2	5.8E-14	0.13
SMAGP	21832	AT	7	NA	NA	5.9E-14	0.13
SMAGP	21831	AT	6.2	NA	NA	5.9E-14	0.13
DCAF6	8886	ES	11:13.1	10	14	6.0E-14	0.13
TENC1	21925	AP	3	NA	NA	6.0E-14	0.13
NINJ2	19605	AP	2	NA	NA	6.3E-14	0.13
RNF167	38614	AD	1.2	1.1	2.2	6.3E-14	0.13
TPM1	30980	AP	4	NA	NA	6.3E-14	0.13
CHEK2	61544	ES	2	1	3	6.4E-14	0.14
RIMBP2	25199	AT	16	NA	NA	6.4E-14	0.13
RIMBP2	25200	AT	24	NA	NA	6.4E-14	0.13
GGT1	61440	AA	7.1:7.2	6.2	7.3	6.4E-14	0.13
AP1G2	26772	RI	1.2:1.3	1.1	1.4	6.5E-14	0.13
SLC11A2	21722	AP	4	NA	NA	6.5E-14	0.13
CSTF3	14883	AT	22	NA	NA	7.0E-14	0.13
THEMIS2	1352	ES	3:4.1:4.2:4.3	2	5	7.0E-14	0.13
MGLL	66622	AP	1.1	NA	NA	7.1E-14	0.14
MGLL	66624	AP	4	NA	NA	7.1E-14	0.14
CSTF3	14884	AT	4.4	NA	NA	7.5E-14	0.13
BCAR1	37598	AP	6	NA	NA	7.5E-14	0.13
LAT	35908	AP	1	NA	NA	7.6E-14	0.13
LAT	35911	AP	2.1	NA	NA	7.6E-14	0.13
ZC2HC1C	28481	AD	2.2:2.3	2.1	3	7.9E-14	0.13
ANAPC5	24862	AA	9.1	8.3	9.2	8.1E-14	0.13
TMSB15B	89788	AT	4	NA	NA	8.4E-14	0.13
C7orf41	79113	ES	3	1	4.1	8.4E-14	0.13
NPIPA5	34144	AP	1	NA	NA	8.6E-14	0.13
NPIPA5	34143	AP	2	NA	NA	8.6E-14	0.13
TMEM63B	76351	AP	2	NA	NA	8.7E-14	0.14
TMEM63B	76352	AP	1	NA	NA	8.7E-14	0.14
PPAPDC1B	83387	AT	4.2	NA	NA	8.7E-14	0.13
GRIK3	1791	AT	15.2	NA	NA	8.8E-14	0.13
GRIK3	1790	AT	16	NA	NA	8.8E-14	0.13
CTSB	97871	ES	2	1.1	3.1	8.8E-14	0.13
XRRA1	17786	AA	18.1:18.2	17	18.3	8.8E-14	0.13
ITGB3	42068	AT	19	NA	NA	8.8E-14	0.13
XRRA1	17785	RI	18.2	18.1	18.3	8.9E-14	0.13
NLRP1	38721	AT	18.2	NA	NA	9.0E-14	0.13
PDGFA	78504	ES	6	5	7	9.3E-14	0.13
HSPB7	786	AP	2.1	NA	NA	9.6E-14	0.13
HSPB7	785	AP	1	NA	NA	9.6E-14	0.13
H2AFV	79570	AT	5	NA	NA	9.6E-14	0.13
ZSCAN5A	52162	AP	4	NA	NA	9.9E-14	0.12
ARID1B	78237	ES	12	11	13	1.0E-13	0.13
RPL28	52094	AT	5	NA	NA	1.1E-13	0.12
PFKM	21421	AD	16.2:16.3	16.1	17	1.1E-13	0.13
COA1	79332	AT	7.2	NA	NA	1.1E-13	0.12
YIPF1	3079	ES	3	2	4	1.1E-13	0.13
ZSCAN5A	52163	AP	1	NA	NA	1.1E-13	0.12
MAP4K4	54762	ES	17	16.2	19	1.2E-13	0.13
NXF1	16435	RI	11.2	11.1	11.3	1.2E-13	0.12

TLE6	46632	AT	3.2	NA	NA	1.3E-13	0.12
TLE6	46631	AT	17	NA	NA	1.3E-13	0.12
STAG3	80918	ES	13	12	14	1.3E-13	0.13
TSPEAR	60826	AT	12	NA	NA	1.3E-13	0.14
TSPEAR	60827	AT	14	NA	NA	1.3E-13	0.14
ZMIZ2	79559	ES	9	8	10	1.3E-13	0.13
SYNE4	102891	ES	2	1	3	1.4E-13	0.12
SEC23A	27346	AP	1	NA	NA	1.4E-13	0.12
SEC23A	27345	AP	7	NA	NA	1.4E-13	0.12
CHI3L2	4144	AP	4.1	NA	NA	1.4E-13	0.15
GK	88735	ES	23	22	24	1.4E-13	0.13
PDE5A	70467	AP	3	NA	NA	1.4E-13	0.13
ERMAP	2124	RI	3.3	3.2	3.4	1.4E-13	0.13
RFX5	7603	AP	1	NA	NA	1.4E-13	0.13
CAST	196583	ES	7.1:8.2	5.2	9	1.5E-13	0.14
ZNF331	51725	AP	4	NA	NA	1.5E-13	0.12
SULT1A2	35811	RI	1.2:1.3	1.1	1.4	1.5E-13	0.12
POLR2J2	81129	ES	3.1	2	3.3	1.5E-13	0.12
UPP1	79641	ES	05:06.1	4	9	1.6E-13	0.13
LLGL2	43458	AP	1	NA	NA	1.6E-13	0.12
LLGL2	43459	AP	2	NA	NA	1.6E-13	0.12
FOXK1	78642	AP	5	NA	NA	1.7E-13	0.12
NT5C2	12992	ES	5	4	6	1.7E-13	0.13
FOXK1	78641	AP	1	NA	NA	1.7E-13	0.12
HIPK3	14893	ES	14	13	15	1.7E-13	0.14
TPM1	30979	AP	1	NA	NA	1.7E-13	0.12
LIMS1	54885	AP	1	NA	NA	1.7E-13	0.12
SYNJ2	78249	AD	19.3	19.2	20	1.8E-13	0.12
TXLNA	1558	AP	1	NA	NA	1.8E-13	0.13
TXLNA	1559	AP	2.1	NA	NA	1.8E-13	0.13
OSMR	71850	AT	18	NA	NA	1.8E-13	0.12
OSMR	71849	AT	7.2	NA	NA	1.8E-13	0.12
ANAPC11	44218	ES	6	3.2	7.2	1.9E-13	0.12
HAUS1	45389	ES	3	2.1	4	1.9E-13	0.12
EPOR	47693	ES	4.2	3	5	1.9E-13	0.13
UBE2D3	70141	AD	2.4	2.3	3.3	1.9E-13	0.12
RNH1	13676	ES	3	2	4.3	2.0E-13	0.12
DGUOK	54011	ES	4:05	1	7	2.0E-13	0.12
WEE1	14328	AP	1	NA	NA	2.1E-13	0.13
WEE1	14327	AP	2	NA	NA	2.1E-13	0.13
RPL30	84636	ES	2.2:3.1	2.1	3.2	2.1E-13	0.12
TMEM91	50044	AP	3	NA	NA	2.1E-13	0.12
SLC16A1	4265	AT	5.2	NA	NA	2.1E-13	0.12
SLC16A1	4266	AT	6	NA	NA	2.1E-13	0.12
DBI	55115	AD	1.2:1.3	1.1	3.1	2.2E-13	0.12
DERL3	61333	AA	5.4	5.2	5.5	2.2E-13	0.12
GTF2IRD1	80080	AP	2	NA	NA	2.3E-13	0.12
GTF2IRD1	80081	AP	1	NA	NA	2.3E-13	0.12
CYTH1	43891	ES	12	11.1	13.2	2.4E-13	0.12
FAM49B	85136	AP	1	NA	NA	2.4E-13	0.12
RPL10	90568	AP	1	NA	NA	2.6E-13	0.13
RPL10	90570	AP	3	NA	NA	2.6E-13	0.13
NDUFAF6	84597	ES	10	9.2	11	2.7E-13	0.12

C8orf34	84100	AT	18	NA	NA	2.7E-13	0.12
FCHSD1	73817	ES	19	18	20	2.8E-13	0.12
DMD	88772	ES	82	81	83	2.8E-13	0.13
ZNF141	68345	AT	4.2	NA	NA	2.8E-13	0.12
ZNF141	68344	AT	5	NA	NA	2.8E-13	0.12
PHACTR1	75368	AT	18	NA	NA	2.9E-13	0.12
WDR66	24918	AT	23	NA	NA	2.9E-13	0.12
WDR66	24919	AT	18	NA	NA	2.9E-13	0.12
BSCL2	16403	AP	1	NA	NA	2.9E-13	0.12
LIMA1	21688	AP	4.1	NA	NA	3.0E-13	0.12
SLC11A2	21724	AP	3.1	NA	NA	3.0E-13	0.12
EPOR	47692	ES	4.1:4.2	3	5	3.1E-13	0.12
SLC9A3R2	33186	AP	3	NA	NA	3.1E-13	0.12
COL14A1	85015	AP	1	NA	NA	3.2E-13	0.13
REPS1	77956	ES	9.3	9.1	10	3.2E-13	0.12
FBXO16	83216	AT	19	NA	NA	3.2E-13	0.12
DCTN2	22644	ES	8	2	10	3.2E-13	0.12
APBB2	69101	ES	8	7.2	9	3.3E-13	0.12
PCDP1	55127	AT	9	NA	NA	3.3E-13	0.14
ARMC8	66961	AT	13.2	NA	NA	3.3E-13	0.12
ARMC8	66960	AT	23	NA	NA	3.3E-13	0.12
MSL1	40842	AT	3.2	NA	NA	3.5E-13	0.12
MSL1	40843	AT	9	NA	NA	3.5E-13	0.12
CSNK1A1	74045	AD	12.2	12.1	13	3.6E-13	0.12
FDFT1	82638	AP	1	NA	NA	3.6E-13	0.12
SUGT1	26006	ES	7	6	8	3.6E-13	0.12
ANKRD42	18050	AT	6.2	NA	NA	3.6E-13	0.12
CYTH1	43890	ES	11.2:13.1	11.1	13.2	3.6E-13	0.12
RNF145	74403	AP	2	NA	NA	3.7E-13	0.12
PCBP4	65125	AA	15.1	14	15.2	3.7E-13	0.12
SLC25A25	87692	AP	1	NA	NA	3.8E-13	0.12
SLC4A4	69463	AT	14.2	NA	NA	3.9E-13	0.12
SLC4A4	69464	AT	26	NA	NA	3.9E-13	0.12
ELF2	70614	AA	10.1	9	10.2	4.0E-13	0.12
UGGT2	26129	AT	42	NA	NA	4.0E-13	0.12
CCPG1	30723	AP	2	NA	NA	4.1E-13	0.12
CCPG1	30722	AP	1	NA	NA	4.1E-13	0.12
PPP2R4	87850	ES	4:5:6:7:8:9:10	3.1	15.1	4.2E-13	0.12
MYO6	76806	ES	29:30:31	28.1	32	4.2E-13	0.13
GPM6B	88529	AA	11.1	10	11.2	4.3E-13	0.12
CHTF8	37269	ES	4.3:4.4	4.1	4.6	4.3E-13	0.12
LUC7L	32849	AD	1.2:1.3	1.1	2.2	4.3E-13	0.12
SUPT20H	25663	AA	21.1	20	21.2	4.3E-13	0.12
RELL1	69002	AT	7	NA	NA	4.3E-13	0.12
RELL1	69003	AT	8	NA	NA	4.3E-13	0.12
KCNJ2	43196	AP	2	NA	NA	4.3E-13	0.12
KCNJ2	43195	AP	1	NA	NA	4.3E-13	0.12
MGAT1	75021	AP	6	NA	NA	4.7E-13	0.12
BRIP1	42892	AT	19.2	NA	NA	4.7E-13	0.12
BRIP1	42893	AT	20	NA	NA	4.7E-13	0.12
SIAH1	36339	AP	8.1	NA	NA	4.7E-13	0.12
CLEC16A	34006	AD	11.2	11.1	12	4.9E-13	0.13
C7orf55-LUC7L2	81956	AP	3.1	NA	NA	4.9E-13	0.12

TIMM17B	89016	AA	4.1:4.2	3	4.3	5.0E-13	0.12
DUSP18	61793	AT	3	NA	NA	5.1E-13	0.12
CD44	15105	ES	7:8:9.1:9.2	5	10	5.2E-13	0.12
TIA1	53874	ES	6	5	7	5.3E-13	0.12
PLEKHG5	465	AP	8	NA	NA	5.7E-13	0.15
ABCG2	69880	AP	1	NA	NA	5.7E-13	0.13
ABCG2	69881	AP	2	NA	NA	5.7E-13	0.13
CARD8	50715	ES	7.2	5	8	5.9E-13	0.13
BCL2L1	58902	AA	2.2	1	2.3	5.9E-13	0.12
VWA5A	19211	AT	10.2	NA	NA	6.0E-13	0.12
VWA5A	19212	AT	18	NA	NA	6.0E-13	0.12
FBF1	43529	AT	2.2	NA	NA	6.1E-13	0.12
FBF1	43530	AT	29	NA	NA	6.1E-13	0.12
POLR2J3	81118	ES	4.1	2	4.3	6.1E-13	0.12
COL14A1	85013	AP	5	NA	NA	6.3E-13	0.12
ZNF100	48787	AP	1	NA	NA	6.4E-13	0.13
ZNF100	48786	AP	3	NA	NA	6.4E-13	0.13
FOXP1	65594	AP	2	NA	NA	6.6E-13	0.12
ERRFI1	534	AA	3.1:3.2:3.3	2	3.4	6.6E-13	0.13
DMKN	49187	ES	11:12	6.4	13	6.7E-13	0.12
SUPT4H1	42661	AP	1	NA	NA	6.8E-13	0.12
SDHAF2	16230	ES	3:4.1:5.1:5.2	1	6	6.8E-13	0.12
ACOT7	389	AP	1.1	NA	NA	7.0E-13	0.12
TMEM201	564	AT	11	NA	NA	7.2E-13	0.12
TMEM201	565	AT	6.2	NA	NA	7.2E-13	0.12
SAR1B	73412	AP	7.1	NA	NA	7.3E-13	0.12
FUT8	28012	AP	1	NA	NA	7.3E-13	0.12
FUT8	28010	AP	2	NA	NA	7.3E-13	0.12
B9D1	39710	AT	8.5	NA	NA	7.6E-13	0.12
CABIN1	61386	AP	31	NA	NA	7.7E-13	0.12
CACNB3	21480	ES	4	3	5	7.9E-13	0.14
MRPL21	17344	AD	2.2	2.1	3	7.9E-13	0.12
PNPLA6	47110	AP	2	NA	NA	8.1E-13	0.12
TFIP11	61515	ES	3	2.2	4	8.6E-13	0.12
APEH	64895	ES	2	1	4	9.0E-13	0.12
PPP1R12A	23528	AP	2.1	NA	NA	9.0E-13	0.13
PDHX	14972	AP	1	NA	NA	9.1E-13	0.12
PDHX	14971	AP	2	NA	NA	9.1E-13	0.12
METTL21A	57189	AT	8.3	NA	NA	9.3E-13	0.12
CD44	14981	ES	8:9.2:10:11	7	12.1	9.4E-13	0.13
CDKN2AIP	71270	AD	2.2	2.1	3	9.4E-13	0.12
ERLIN2	83350	RI	7.2	7.1	7.3	9.7E-13	0.12
MAD2L2	665	ES	2:03	1	4	9.7E-13	0.12
EIF4A2	96628	ES	9.1:9.2	8	10	1.0E-12	0.12
RCC1	1386	AP	1	NA	NA	1.0E-12	0.12
RCC1	1387	AP	5	NA	NA	1.0E-12	0.12
CEP170	10451	AD	16.3	16.2	17	1.0E-12	0.13
STRADA	42961	RI	12.6	12.5	12.7	1.0E-12	0.12
RPS6KA3	88667	AP	2	NA	NA	1.0E-12	0.12
NUMA1	17518	ES	4	3	5	1.1E-12	0.12
UBTF	41827	AP	2	NA	NA	1.1E-12	0.12
TRIM7	75047	AT	3.3	NA	NA	1.1E-12	0.12
TRIM7	75046	AT	8	NA	NA	1.1E-12	0.12

CD44	15276	ES	0:11:12.1:13:	2.1	17.2	1.1E-12	0.12
OARD1	76082	AP	3.1	NA	NA	1.2E-12	0.12
TJP2	86532	AP	3	NA	NA	1.2E-12	0.12
CAMK2B	79489	ES	18:19:20	17	21.1	1.2E-12	0.12
CHN2	79083	AP	3	NA	NA	1.2E-12	0.12
UPF3B	89980	ES	8	7	9	1.2E-12	0.12
ATP9B	46234	ES	30	29	31.1	1.2E-12	0.12
CD44	15111	ES	7:8:9.1:9.2:10:	5	12.1	1.2E-12	0.12
PPIP5K2	72915	ES	28:29:00	27	30	1.2E-12	0.12
INSR	47099	ES	11	10	12	1.2E-12	0.12
DMKN	49142	AD	20.2	20.1	21	1.3E-12	0.12
BAG5	29456	AP	2.1	NA	NA	1.3E-12	0.12
BAG5	29457	AP	1	NA	NA	1.3E-12	0.12
ZCCHC10	73327	AT	6.2	NA	NA	1.3E-12	0.12
ZCCHC10	73326	AT	3	NA	NA	1.3E-12	0.12
TJP1	29765	AD	30.2	30.1	31	1.3E-12	0.12
CCDC158	69613	AT	9	NA	NA	1.3E-12	0.12
CCDC158	69612	AT	25	NA	NA	1.3E-12	0.12
YPEL3	36066	AP	2.1	NA	NA	1.4E-12	0.12
YPEL3	36068	AP	1.1	NA	NA	1.4E-12	0.12
SNUPN	31884	AD	1.2	1.1	4	1.4E-12	0.12
SIGIRR	13650	AP	2	NA	NA	1.4E-12	0.12
SIGIRR	13651	AP	1.1	NA	NA	1.4E-12	0.12
RPS25	19057	ES	2.1:2.2	1	3.1	1.5E-12	0.12
DHX8	41705	AT	24	NA	NA	1.5E-12	0.11
DHX8	41706	AT	23	NA	NA	1.5E-12	0.11
PPIL3	127875	ES	2.2:3:4.1	1.1	4.2	1.5E-12	0.13
NPR3	71661	AP	2	NA	NA	1.5E-12	0.12
NPR3	71662	AP	1	NA	NA	1.5E-12	0.12
SULF1	84103	AP	2	NA	NA	1.5E-12	0.12
SULF1	84104	AP	1	NA	NA	1.5E-12	0.12
CTCFL	59903	AT	14.2	NA	NA	1.5E-12	0.12
PCGF5	12510	AT	12	NA	NA	1.5E-12	0.11
PCGF5	12509	AT	4	NA	NA	1.5E-12	0.11
CD44	15114	ES	6:7:8:9.2:10:1	5	12.1	1.5E-12	0.12
AP1G1	37485	ES	12	10.1	13.2	1.5E-12	0.14
PDE4DIP	4432	ES	5.2:5.3	4	6.1	1.5E-12	0.13
PPP1R12A	23526	AP	1	NA	NA	1.5E-12	0.12
OARD1	76080	AP	2.1	NA	NA	1.6E-12	0.11
TTC31	54097	ES	3	2	4	1.6E-12	0.12
RRN3	34139	ES	12:13	11.1	14	1.7E-12	0.12
ZNF185	90401	ES	15	14	16	1.7E-12	0.12
IL32	33439	AD	1.2:1.3:1.4:1.5	1.1	1.9	1.7E-12	0.12
PICALM	18172	ES	14.1:14.2	13	15	1.7E-12	0.12
CDYL	75231	AP	4	NA	NA	1.8E-12	0.11
TMEM33	69133	RI	8.2	8.1	8.3	1.8E-12	0.11
GNB1L	61083	RI	1.2	1.1	1.3	1.8E-12	0.12
FAM86C1	17441	ES	3.2	2	5.1	1.8E-12	0.11
VSTM1	51760	ME	3|4	2	5	1.8E-12	0.12
RAB34	94477	ES	03:04.1	2.3	4.2	1.9E-12	0.12
AGO3	1740	AT	8	NA	NA	1.9E-12	0.11
CDYL	75229	AP	5	NA	NA	1.9E-12	0.11
ARHGAP22	11484	AP	7	NA	NA	2.0E-12	0.11

CD44	14980	ES	8:9.1:9.2:10:1	7	12.1	2.0E-12	0.12
MEST	81804	AP	3	NA	NA	2.0E-12	0.12
IGFLR1	49261	ES	3:4.1:4.2:4.3	2.2	5	2.1E-12	0.11
RUFY3	69444	AP	1	NA	NA	2.1E-12	0.11
PISD	61881	AP	4	NA	NA	2.1E-12	0.12
LRRC23	20005	ES	7.1:8	6	10	2.1E-12	0.11
SH2D3C	87662	AP	1	NA	NA	2.1E-12	0.12
C6orf203	77126	AD	1.2	1.1	3	2.2E-12	0.11
CD44	15270	ES	:10:11:12.1:1	2.1	17.2	2.2E-12	0.11
GGT1	61444	ES	6.2	4	7.3	2.2E-12	0.12
LIMS2	55226	AP	1	NA	NA	2.3E-12	0.12
IP6K2	64760	ES	11.4:11.5	11.2	11.9	2.3E-12	0.11
PHYHIP	82997	ES	2	1	3	2.3E-12	0.13
NR3C2	70802	AP	1	NA	NA	2.3E-12	0.12
NR3C2	70801	AP	2	NA	NA	2.3E-12	0.12
BEND6	76573	AT	4	NA	NA	2.4E-12	0.11
BEND6	76572	AT	10	NA	NA	2.4E-12	0.11
ATL2	53249	AA	17.1	15	17.2	2.4E-12	0.11
DUSP18	61794	AT	2.5	NA	NA	2.4E-12	0.11
DNAJB5	86220	AP	1	NA	NA	2.4E-12	0.12
DNAJB5	86221	AP	2.1	NA	NA	2.4E-12	0.12
ANKRD13D	17158	ES	2	1	3.1	2.5E-12	0.12
ZNF789	80647	AT	4.3	NA	NA	2.5E-12	0.11
ACBD4	41948	RI	3.4	3.3	3.5	2.5E-12	0.11
CCDC90B	18088	AD	1.2:1.3	1.1	2	2.5E-12	0.11
GABRG3	93624	AT	6.2	NA	NA	2.6E-12	0.14
GABRG3	93623	AT	10	NA	NA	2.6E-12	0.14
EIF4E2	58001	ES	8	6.1	9	2.7E-12	0.11
SUPT4H1	42659	AP	2.1	NA	NA	2.8E-12	0.11
BIN1	55192	ES	13:16:17	12	18	2.8E-12	0.13
ZNF814	95403	ES	02:03.1	1	9	2.8E-12	0.11
RWDD2B	60299	ES	3	2	4	2.9E-12	0.12
BEX2	89725	AD	1.2:1.3	1.1	2	3.0E-12	0.11
CEP170	10453	AD	16.2	16.1	17	3.1E-12	0.12
MVD	38010	ES	3:04	1	5	3.2E-12	0.11
SLC35E2	223	AT	7	NA	NA	3.2E-12	0.11
SLC35E2	222	AT	8	NA	NA	3.2E-12	0.11
C1D	53822	ES	2.2	1.1	3.1	3.3E-12	0.11
PAM16	33645	AP	6.1	NA	NA	3.5E-12	0.11
TCAIM	64356	AP	2	NA	NA	3.7E-12	0.11
TCAIM	64355	AP	1	NA	NA	3.7E-12	0.11
RBBP8	44787	AP	2.1	NA	NA	3.8E-12	0.12
RPS6KB2	17203	ES	6.1:6.2	5.1	7	3.9E-12	0.11
FAM86B1	82687	ES	6:7.1:7.2:7.3	4	8.1	3.9E-12	0.12
COQ4	87738	RI	2.2	2.1	2.3	3.9E-12	0.11
ADAM15	7897	ES	21.1:21.2	20	22.1	4.0E-12	0.11
ZNF333	48018	AT	11	NA	NA	4.0E-12	0.11
MEST	81803	AP	1	NA	NA	4.0E-12	0.11
PLEKHA8	79105	AT	14	NA	NA	4.1E-12	0.11
ITGB3	42067	AT	15	NA	NA	4.1E-12	0.11
ZNF207	40205	ES	10	9	11	4.2E-12	0.11
ABCB9	24998	AT	14.2	NA	NA	4.2E-12	0.11
EXOC7	43568	ES	07:08.1	6	8.2	4.3E-12	0.11

ANKRD42	18051	AT	13.2	NA	NA	4.5E-12	0.11
MOK	29362	AP	14	NA	NA	4.6E-12	0.11
FBLN5	28893	ES	7	5	8	4.8E-12	0.11
DTD2	27119	RI	3.2	3.1	3.3	4.9E-12	0.11
SLC25A45	16822	AP	4.1	NA	NA	4.9E-12	0.11
DAG1	64873	AP	2.1	NA	NA	5.0E-12	0.11
DAG1	64874	AP	1	NA	NA	5.0E-12	0.11
CASP8	56814	AP	4	NA	NA	5.0E-12	0.12
C7orf55-LUC7L2	81954	AP	2	NA	NA	5.0E-12	0.11
MAX	27935	RI	5.6:5.7	5.5	5.8	5.3E-12	0.11
THUMPD2	53337	ES	4	3	5	5.3E-12	0.12
GNPDA1	73862	RI	2.5	2.4	2.6	5.4E-12	0.11
OBSL1	57732	ES	13	12	14.1	5.4E-12	0.11
LMO7	26067	ES	12	9	13	5.5E-12	0.12
PLEKHA8	79104	AT	16	NA	NA	5.5E-12	0.11
MFF	57810	ES	9	7	11	5.6E-12	0.11
RREB1	75253	ES	12	11	13	5.7E-12	0.12
DISC1	10280	AT	17	NA	NA	5.7E-12	0.11
LIMCH1	69111	AP	10.1	NA	NA	5.7E-12	0.12
TCF20	62500	AA	4.1	3	4.2	5.7E-12	0.11
MAP3K7	77020	ES	11	10	12	5.9E-12	0.11
NPIPB5	35568	ES	3.1	2.5	5	5.9E-12	0.12
C1orf50	90934	ES	1.3	1.1	2.2	6.0E-12	0.13
C11orf49	15609	RI	14.2	14.1	14.3	6.0E-12	0.11
KIAA0513	37876	AP	2	NA	NA	6.0E-12	0.11
CHRNB1	38960	AP	1	NA	NA	6.0E-12	0.11
PFDN5	93149	ES	2	1	4.2	6.1E-12	0.11
ZNF678	10051	AT	6	NA	NA	6.2E-12	0.11
ZNF678	10052	AT	5.2	NA	NA	6.2E-12	0.11
ARRDC1	88335	RI	2.2	2.1	2.3	6.4E-12	0.11
ERG	60589	AP	5	NA	NA	6.4E-12	0.13
PLD3	49889	ES	04:05.1	1.2	5.2	6.6E-12	0.11
SH3KBP1	88642	AP	3	NA	NA	6.6E-12	0.11
H2AFV	79572	AT	6	NA	NA	6.7E-12	0.11
CNTN4	62954	AP	1	NA	NA	7.1E-12	0.11
STRADA	42962	RI	12.4	12.3	12.5	7.1E-12	0.11
RAPGEF3	21352	AT	30	NA	NA	7.1E-12	0.11
RAPGEF3	21351	AT	18.2	NA	NA	7.1E-12	0.11
TMEM116	24566	ES	3	2	4	7.1E-12	0.12
SULT1A1	35813	AP	6.1	NA	NA	7.1E-12	0.11
SYNJ2	78242	AP	3	NA	NA	7.5E-12	0.11
ZNF250	85676	AT	11	NA	NA	7.6E-12	0.11
CNTN4	62950	AP	4	NA	NA	7.6E-12	0.11
EVC	68701	ES	23	22.1	24	7.8E-12	0.11
ULK3	31757	RI	6.2	6.1	6.3	8.0E-12	0.11
ENSA	7493	AT	6	NA	NA	8.0E-12	0.11
CCNL2	155	AT	12	NA	NA	8.1E-12	0.11
CCNL2	157	AT	6.3	NA	NA	8.1E-12	0.11
ZDHHC11	71448	AT	5.2	NA	NA	8.2E-12	0.11
RNF167	38606	AP	1.1	NA	NA	8.3E-12	0.11
RNF167	38605	AP	2.1	NA	NA	8.3E-12	0.11
OXR1	84847	AP	5	NA	NA	8.3E-12	0.11
TROAP	21551	AT	10.2	NA	NA	8.3E-12	0.11

PPAPDC1B	83388	AT	7	NA	NA	8.3E-12	0.11
NIPA1	29688	AP	2	NA	NA	8.4E-12	0.11
WNT9B	42038	AT	4.2	NA	NA	8.4E-12	0.11
WNT9B	42039	AT	5	NA	NA	8.4E-12	0.11
MRAS	66991	AP	3	NA	NA	8.8E-12	0.11
LETMD1	21780	ES	02:03.2	1.2	7	8.9E-12	0.11
PBX1	8790	ES	11	10	12	8.9E-12	0.11
FAM65B	75542	AT	28	NA	NA	8.9E-12	0.11
IDS	90287	AP	4	NA	NA	9.1E-12	0.11
IDS	90286	AP	1	NA	NA	9.1E-12	0.11
LUC7L	32846	RI	1.2	1.1	1.3	9.3E-12	0.11
DNASE1L1	90573	AP	2.1	NA	NA	9.5E-12	0.11
MTUS1	82818	AP	12	NA	NA	9.5E-12	0.11
SMURF2	43074	ES	4	3	5	9.8E-12	0.11
HYAL2	65003	RI	1.4	1.3	1.5	1.0E-11	0.11
MIA3	9888	AP	7	NA	NA	1.0E-11	0.11
MIA3	9887	AP	1	NA	NA	1.0E-11	0.11
MRPL55	10177	AD	1.2	1.1	2.2	1.0E-11	0.11
PLA2G6	62205	ES	14	13	15	1.0E-11	0.11
RBM19	24646	AT	25	NA	NA	1.1E-11	0.11
RBM19	24645	AT	24.3	NA	NA	1.1E-11	0.11
ZCCHC8	24958	AP	2.1	NA	NA	1.1E-11	0.11
NAGA	62493	RI	1.2	1.1	1.3	1.1E-11	0.11
REPIN1	82230	AP	5.1	NA	NA	1.1E-11	0.11
INO80C	45175	AA	4.1	3	4.2	1.2E-11	0.11
SNCA	69932	AD	2.3	2.2	4.2	1.2E-11	0.13
DCAF6	8887	ES	13.1	10	14	1.2E-11	0.11
IKBIP	23864	AT	3	NA	NA	1.2E-11	0.11
IKBIP	23865	AT	4	NA	NA	1.2E-11	0.11
SARDH	88080	AT	8	NA	NA	1.2E-11	0.11
CFLAR	56787	AP	2	NA	NA	1.2E-11	0.11
KIAA0513	37875	AP	1.1	NA	NA	1.2E-11	0.11
RHOA	64860	ES	4	2	6	1.2E-11	0.11
IFIT3	12489	AP	1	NA	NA	1.2E-11	0.11
IFIT3	12488	AP	2	NA	NA	1.2E-11	0.11
CLYBL	26188	RI	9.2	9.1	9.3	1.2E-11	0.11
EXOC7	43570	ES	7:8.1:8.2	6	9	1.3E-11	0.11
ING2	71272	AP	2	NA	NA	1.3E-11	0.11
ING2	71271	AP	1	NA	NA	1.3E-11	0.11
HSF1	85559	ES	10	9	11	1.3E-11	0.11
BCL11A	53659	AT	6	NA	NA	1.3E-11	0.11
BCL11A	53658	AT	5.5	NA	NA	1.3E-11	0.11
FANCA	38142	AT	10.4	NA	NA	1.3E-11	0.11
FANCA	38143	AT	43	NA	NA	1.3E-11	0.11
KRT8	21907	AP	3.1	NA	NA	1.3E-11	0.11
KRT8	21908	AP	1.1	NA	NA	1.3E-11	0.11
VDAC1	73335	AP	1	NA	NA	1.3E-11	0.11
VDAC1	73334	AP	2	NA	NA	1.3E-11	0.11
TBC1D22A	62720	AP	2	NA	NA	1.3E-11	0.11
SLC35G1	12570	AT	3	NA	NA	1.3E-11	0.11
NAT16	81045	AT	5	NA	NA	1.3E-11	0.12
NAT16	81044	AT	3.2	NA	NA	1.3E-11	0.12
RABGAP1	87493	AP	2	NA	NA	1.3E-11	0.13

RWDD1	77326	ES	2	1	3	1.3E-11	0.11
CRELD1	63294	AD	1.2	1.1	1.4	1.3E-11	0.11
ZNF789	80648	AT	8	NA	NA	1.4E-11	0.11
IL18BP	17471	AT	5	NA	NA	1.4E-11	0.11
IL18BP	17473	AT	4.7	NA	NA	1.4E-11	0.11
POLL	12902	ES	1.2:1.3:1.5	1.1	3	1.5E-11	0.13
YY1AP1	8104	AA	12.1	11	12.2	1.5E-11	0.11
BCKDHA	50063	AP	2.1	NA	NA	1.5E-11	0.11
BCKDHA	50062	AP	1	NA	NA	1.5E-11	0.11
COQ10A	22415	AP	2	NA	NA	1.5E-11	0.11
RPL21	25524	AD	1.4	1.3	2	1.5E-11	0.11
COQ10A	22414	AP	1	NA	NA	1.6E-11	0.11
ZNF254	48837	AP	3	NA	NA	1.6E-11	0.11
ZNF254	48836	AP	1	NA	NA	1.6E-11	0.11
ANKRD11	38078	AT	8	NA	NA	1.6E-11	0.11
VGLL3	65659	AT	4	NA	NA	1.6E-11	0.11
VGLL3	65658	AT	5	NA	NA	1.6E-11	0.11
FAM92A1	84516	AP	1	NA	NA	1.7E-11	0.11
IFNAR1	60399	AP	2	NA	NA	1.8E-11	0.11
IFNAR1	60398	AP	1	NA	NA	1.8E-11	0.11
PISD	61880	AP	1	NA	NA	1.8E-11	0.11
ANAPC11	44211	ES	6	3.3	7.2	1.8E-11	0.11
BMP8B	1912	AT	8	NA	NA	1.8E-11	0.11
BMP8B	1911	AT	6.2	NA	NA	1.8E-11	0.11
CENPP	86873	AP	6	NA	NA	1.8E-11	0.11
CD44	15117	ES	10:11	5	12.1	1.8E-11	0.11
OAZ1	46599	ES	3.2:3.3:3.4:3.5	1	4	1.8E-11	0.11
D2HGDH	58421	ES	8:09	6	11.2	1.9E-11	0.11
ESRP1	84567	ES	14:15	13	16	1.9E-11	0.11
SRSF4	1425	ES	2:03:04	1	5	1.9E-11	0.11
TERF1	84141	ES	7	6	8	1.9E-11	0.11
RASGRP3	53188	AP	3	NA	NA	2.0E-11	0.11
ABCC3	42464	RI	16.2	16.1	16.3	2.0E-11	0.11
COPS5	84071	ES	2.3	2.1	5	2.0E-11	0.13
SSH2	40062	AT	3	NA	NA	2.0E-11	0.10
ARVCF	96084	ES	20	19	21	2.0E-11	0.11
STRADB	56840	AA	12.1	11	12.2	2.0E-11	0.11
RTN4	53584	AP	2.1	NA	NA	2.1E-11	0.11
SSH2	40060	AT	19	NA	NA	2.1E-11	0.10
FCGRT	50957	AP	1	NA	NA	2.1E-11	0.10
FCGRT	50958	AP	2.1	NA	NA	2.1E-11	0.10
MRPL55	10117	ES	2:2.3:2.4:2.5:2	1.2	2.9	2.1E-11	0.10
ZNF684	2009	AT	6	NA	NA	2.1E-11	0.10
ZNF684	2008	AT	5.2	NA	NA	2.1E-11	0.10
NEK2	9718	AT	7.2	NA	NA	2.1E-11	0.10
NEK2	9717	AT	8	NA	NA	2.1E-11	0.10
PPP1CA	17183	ES	2.1:2.2	1	3	2.1E-11	0.11
MYO1C	38306	AP	4	NA	NA	2.2E-11	0.11
SAR1B	73415	AP	2	NA	NA	2.2E-11	0.10
ING5	58408	AT	9	NA	NA	2.2E-11	0.10
ING5	58407	AT	8	NA	NA	2.2E-11	0.10
TOP3B	61270	ES	6	5	7	2.2E-11	0.11
LIAS	69062	ES	7	6	8	2.2E-11	0.11

PLD3	49893	ES	1.2:4:5.1	1.1	5.2	2.3E-11	0.11
FAM92A1	84515	AP	6	NA	NA	2.3E-11	0.11
SERTAD3	49901	AP	2	NA	NA	2.3E-11	0.10
SERTAD3	49902	AP	1	NA	NA	2.3E-11	0.10
USP1	3252	AP	1	NA	NA	2.4E-11	0.11
USP1	3251	AP	2	NA	NA	2.4E-11	0.11
PRKAG1	21508	ES	03:04.1	2	4.2	2.4E-11	0.11
RAB3GAP1	55433	ES	4:05	3.1	6	2.6E-11	0.10
MSI2	94619	ES	19	18.2	20	2.6E-11	0.11
FN1	57397	ES	33	32	34	2.7E-11	0.10
SMAGP	21834	ES	5	3.3	6.1	2.8E-11	0.10
SMAD4	45557	AP	4	NA	NA	2.8E-11	0.10
SMARCC2	22393	ES	18	17	19	2.8E-11	0.11
CFLAR	56791	AT	9	NA	NA	2.8E-11	0.10
TMEM205	47677	AD	2.2:2.3	2.1	2.6	2.9E-11	0.10
KCNC3	51171	AP	2	NA	NA	2.9E-11	0.11
KCNC3	51170	AP	1	NA	NA	2.9E-11	0.11
PALLD	71133	ES	14	12	15	2.9E-11	0.11
KIF12	102262	ES	5	4.1	6	2.9E-11	0.12
SP140	57871	AT	30	NA	NA	3.0E-11	0.10
SPECC1	39786	AP	1	NA	NA	3.0E-11	0.13
FOXJ3	2067	AP	1	NA	NA	3.1E-11	0.10
ZNF667	52180	ES	02:03.2	1	6	3.1E-11	0.10
FEZ2	53195	AP	2	NA	NA	3.1E-11	0.10
FEZ2	53193	AP	1	NA	NA	3.1E-11	0.10
OSGIN2	84387	AP	1	NA	NA	3.1E-11	0.10
OSGIN2	84388	AP	2	NA	NA	3.1E-11	0.10
NPHP3	66808	AT	29.2	NA	NA	3.2E-11	0.10
ZNF519	44757	AT	4	NA	NA	3.2E-11	0.10
MCTP2	32584	AP	2	NA	NA	3.2E-11	0.11
ARL17B	42015	AT	5	NA	NA	3.2E-11	0.10
SPTBN4	49911	AT	30.2	NA	NA	3.3E-11	0.10
ORAOV1	17371	RI	5.2	5.1	5.3	3.3E-11	0.10
VRK3	51145	AT	17	NA	NA	3.3E-11	0.10
SPECC1	39789	AP	5	NA	NA	3.4E-11	0.13
BCO2	18750	AT	14	NA	NA	3.5E-11	0.10
BCO2	18749	AT	13.2	NA	NA	3.5E-11	0.10
URGCP	79355	AP	4.1	NA	NA	3.6E-11	0.10
PALLD	71123	AP	3	NA	NA	3.6E-11	0.11
PACS2	29630	AP	2	NA	NA	3.6E-11	0.10
CASP8	56812	AP	1.1	NA	NA	3.6E-11	0.11
PI4K2B	68958	AP	1	NA	NA	3.6E-11	0.11
PI4K2B	68959	AP	2	NA	NA	3.6E-11	0.11
SLC35G1	12567	AT	5.2	NA	NA	3.6E-11	0.10
KIAA1522	1632	AP	1	NA	NA	3.7E-11	0.10
ZNF783	82182	AT	16	NA	NA	3.8E-11	0.10
ZNF783	82183	AT	7	NA	NA	3.8E-11	0.10
DMKN	49149	AA	18.3:18.4	18.1	18.5	3.8E-11	0.10
RPL21	25525	AD	1.3:1.4	1.2	2	3.9E-11	0.10
CFLAR	56783	AP	1	NA	NA	3.9E-11	0.10
PSAP	12067	AA	8.1	7	8.2	3.9E-11	0.10
MID1	88463	AP	1	NA	NA	4.0E-11	0.12
FKBP11	21495	AT	8	NA	NA	4.0E-11	0.10

AIDA	9892	AP	1	NA	NA	4.1E-11	0.10
AIDA	9891	AP	2	NA	NA	4.1E-11	0.10
ABI1	11042	ES	11.2	9	13	4.1E-11	0.11
OBSCN	10197	AT	92	NA	NA	4.1E-11	0.10
OBSCN	10196	AT	118	NA	NA	4.1E-11	0.10
PRKAG2	82384	AP	7.1	NA	NA	4.2E-11	0.10
KRTCAP3	53014	AT	7.2	NA	NA	4.2E-11	0.10
KRTCAP3	53013	AT	8	NA	NA	4.2E-11	0.10
MYL6B	22371	RI	7.2	7.1	7.3	4.2E-11	0.10
ST5	14266	AP	5	NA	NA	4.3E-11	0.10
FAM72A	9576	AP	3.1	NA	NA	4.3E-11	0.10
FAM72A	9575	AP	1	NA	NA	4.3E-11	0.10
THYN1	19549	AD	1.2	1.1	1.4	4.3E-11	0.10
ADAM15	7898	ES	21.2	20	22.1	4.3E-11	0.10
ANKRD44	56670	AT	27	NA	NA	4.5E-11	0.10
SLC6A13	19586	AT	16	NA	NA	4.5E-11	0.10
SLC6A13	19588	AT	2.2	NA	NA	4.5E-11	0.10
AKAP2	87175	AP	8	NA	NA	4.6E-11	0.12
SCRN2	42120	RI	7.2	7.1	7.3	4.7E-11	0.10
SAE1	50630	AD	1.2:1.3	1.1	2	4.9E-11	0.11
TGFA	53894	AP	1	NA	NA	5.0E-11	0.11
TGFA	53893	AP	2	NA	NA	5.0E-11	0.11
D2HGDH	58422	ES	7.1:7.2:7.3	6	8	5.1E-11	0.10
C18orf56	44456	AT	2	NA	NA	5.2E-11	0.10
C18orf56	44455	AT	3	NA	NA	5.2E-11	0.10
CAPN3	30149	AP	1	NA	NA	5.2E-11	0.10
IL1R1	54771	AP	3	NA	NA	5.4E-11	0.11
ATXN2L	35846	ES	.2:22.4:22.5:2	22.1	22.7	5.5E-11	0.10
ZNF223	50272	AT	13	NA	NA	5.5E-11	0.10
PDGFRA	69319	AT	24	NA	NA	5.7E-11	0.10
METTL21A	57184	AP	4.1	NA	NA	5.9E-11	0.10
RNH1	13678	ES	03:04.2	1	4.3	6.0E-11	0.10
MRPL55	10128	ES	2.3:2.4:2.5:2.6	1.1	2.9	6.2E-11	0.10
ZNF580	52123	RI	2.2	2.1	2.3	6.2E-11	0.10
RBMS1	55726	AP	4	NA	NA	6.3E-11	0.10
PDGFRA	69320	AT	17	NA	NA	6.3E-11	0.10
ULK4	64260	AT	40	NA	NA	6.4E-11	0.10
KIAA1522	1631	AP	2	NA	NA	6.4E-11	0.10
CNTLN	85938	AT	7.3	NA	NA	6.4E-11	0.10
MAP2	57224	ES	16	15	17	6.4E-11	0.10
SGK3	84032	AP	2	NA	NA	6.5E-11	0.10
SGK3	84031	AP	1	NA	NA	6.5E-11	0.10
GALK1	43493	AT	8	NA	NA	6.6E-11	0.10
GALK1	43494	AT	7.2	NA	NA	6.6E-11	0.10
MMP28	40332	AT	3.2	NA	NA	6.7E-11	0.10
SUPT20H	25665	AA	21.2	20	21.3	7.1E-11	0.10
FAM214A	30696	AD	12.2	12.1	13	7.1E-11	0.10
RGS3	87285	AP	24	NA	NA	7.2E-11	0.10
NFIX	47902	AP	4.1	NA	NA	7.5E-11	0.11
TMEM107	39130	ES	:3.1:3.2:3.4:3.	1	3.7	7.6E-11	0.10
FOXP1	65598	AP	14	NA	NA	7.6E-11	0.10
PPP2R4	87853	ES	6:7:8:9:10	3.1	15.1	7.7E-11	0.11
ZNF667	52179	ES	9	8	12	7.7E-11	0.10

NREP	72972	AP	4	NA	NA	7.8E-11	0.11
TMUB2	41811	AD	2.3:2.4:2.5	2.2	4.3	7.8E-11	0.10
PALLD	71121	AP	13	NA	NA	8.0E-11	0.10
SALL1	36401	AP	2	NA	NA	8.4E-11	0.10
SALL1	36400	AP	1	NA	NA	8.4E-11	0.10
NDRG2	26503	RI	4.3:4.4	4.2	4.5	8.5E-11	0.11
RASGRP3	53186	AP	1	NA	NA	8.5E-11	0.10
RHEB	82377	AP	2	NA	NA	8.6E-11	0.11
RHEB	82378	AP	1.1	NA	NA	8.6E-11	0.11
SMARCA2	85723	AP	29.1	NA	NA	9.5E-11	0.11
RNF216	78681	ES	4:5.1:5.2:6.2	2	7	9.6E-11	0.10
BIN1	55184	ES	16	13	17	9.6E-11	0.10
KANK1	85711	AP	1	NA	NA	9.6E-11	0.10
NPIPB4	35511	ES	3.1	2.5	4	9.9E-11	0.11
LPIN1	52712	ES	10	9	12	9.9E-11	0.11
CHTF8	37272	AA	4.1:4.2	3	4.3	1.0E-10	0.10
DIXDC1	18706	AP	1	NA	NA	1.0E-10	0.11
PSMC3IP	41081	RI	1.2	1.1	1.3	1.1E-10	0.10
ERG	60587	AP	1	NA	NA	1.1E-10	0.11
C4orf22	69679	AT	11	NA	NA	1.1E-10	0.10
STRADA	42973	ES	4	3	6	1.1E-10	0.11
TDRD3	26018	AP	2	NA	NA	1.2E-10	0.11
TDRD3	26017	AP	1	NA	NA	1.2E-10	0.11
FAM49B	85138	AP	4	NA	NA	1.2E-10	0.10
PPIL3	127874	ES	1.2:2.2:3:4.1	1.1	4.2	1.2E-10	0.11
CAPN3	30161	ES	11	10	12	1.3E-10	0.12
LYRM1	34413	ES	5:07	3	8.1	1.3E-10	0.10
TMUB2	41803	AA	4.2	3	4.3	1.3E-10	0.10
MON2	22839	ES	31	30	32	1.3E-10	0.10
ARHGEF10	82562	ES	10	9	11	1.3E-10	0.11
CHERP	48175	AA	6.1	5	6.2	1.4E-10	0.10
MYO1B	56607	ES	23	22	24	1.4E-10	0.10
MLH1	63953	AD	1.2	1.1	2.1	1.4E-10	0.10
DOK4	36567	AP	1	NA	NA	1.4E-10	0.12
DOK4	36566	AP	2.1	NA	NA	1.4E-10	0.12
CDKL1	27475	AA	9.1	8	9.2	1.7E-10	0.10
TBC1D10C	17192	AA	6.1	5	6.2	1.7E-10	0.10
PRDM16	303	ES	17	16	18.1	1.9E-10	0.10
CKMT2	72660	ES	2	1.1	3	2.1E-10	0.11
MAP2	57225	ES	13	12	14	2.2E-10	0.10
MFSD11	43691	AD	2.2:2.3	2.1	2.6	2.5E-10	0.11
B3GALNT1	67502	ES	6:07	5	9.1	2.6E-10	0.11
THEMIS2	1354	ES	3	2	5	2.7E-10	0.10
LEF1	70294	ES	7	6.1	8	2.7E-10	0.10
CAMTA2	38637	AA	7.1	6	7.2	2.8E-10	0.11
CAMTA2	38634	ES	7.1:7.2:7.3	6	8	2.9E-10	0.10
REPIN1	82235	ES	4.1:4.2:5.2	3.2	5.3	2.9E-10	0.10
C4orf22	69683	ES	6	4	8	3.0E-10	0.11
C22orf29	61086	AD	1.2	1.1	1.4	3.0E-10	0.11
PTPRK	77503	ES	2	1	4	3.1E-10	0.11
PLEC	85512	AP	8	NA	NA	3.2E-10	0.10
C11orf49	15633	ES	3:04	1	7	3.3E-10	0.11
EFCAB2	10481	ES	2:03	1.3	4	3.3E-10	0.10

KRBOX1	64326	AT	8	NA	NA	3.6E-10	0.10
KRBOX1	64325	AT	10	NA	NA	3.6E-10	0.10
DNASE1L2	33239	RI	1.2:1.3	1.1	1.4	4.0E-10	0.10
TNRC6A	94083	ES	7	6	8	4.0E-10	0.10
KCTD7	79878	AP	1	NA	NA	4.9E-10	0.10
KCTD7	79879	AP	5	NA	NA	4.9E-10	0.10
DOCK7	3256	ES	24	23	25	5.2E-10	0.11
ACPL2	67063	ES	6	5	9	5.3E-10	0.11
FAM86C1	17437	ES	3.1:3.2:4	2	5.1	5.7E-10	0.11
TNC	87360	ES	19	11	20	6.2E-10	0.11
RNF38	86345	AP	1	NA	NA	6.4E-10	0.10
RGL1	9193	AP	4	NA	NA	7.5E-10	0.10
RGL1	9194	AP	1	NA	NA	7.5E-10	0.10
ANKRD28	63623	AP	3	NA	NA	8.3E-10	0.10
ANKRD28	63624	AP	1	NA	NA	8.3E-10	0.10
NVL	9942	ME	11|12	10	13	8.4E-10	0.11
MCC	73006	AP	5	NA	NA	8.7E-10	0.10
ZNF664	25131	ES	2.2:4	1.4	5	9.6E-10	0.11
TNIK	67629	ES	22	21.1	23	1.1E-09	0.10
ZNF185	90396	AP	1	NA	NA	1.3E-09	0.10
STRADA	42976	ES	3:04	2.2	6	2.0E-09	0.10

The Analysis of variance (ANOVA) results were shown.

Supplementary Table 3. Result of t-test comparing exon skipping (ES) regulation between THCA.1 and THCA.4.

Gene Symbol	as_id	splice_type	exons	from_exon	to_exon	ΔPSI	P-value	regulation
LSR	49086	ES	5	3	6	-0.18	2.2E-44	excluded
SERPINA1	29130	ES	2.1:2.2:2.3:2.4:2.5	1.1	3.2	0.55	6.3E-44	included
POLR2J3	81113	ES	6:07	4.3	8	0.33	1.6E-43	included
NUMA1	17515	ES	18	17	19	0.18	8.9E-41	included
SSBP4	48431	ES	7	6	8	0.15	1.4E-38	included
FAM86B1	82692	ES	06:07.1	4	8.1	-0.33	2.5E-38	excluded
SERPINA1	29134	ES	2.1:2.4:2.5	1.1	3.2	0.12	7.7E-38	included
SH3BP1	62140	ES	16	15	17.2	-0.23	7.7E-38	excluded
OSBPL3	79027	ES	9	8	10	-0.21	3.7E-36	excluded
SYNGR2	43776	ES	4.1	3.2	4.3	0.03	9.7E-36	included
CADM1	18851	ES	10	9	12	-0.16	5.1E-35	excluded
MYO1B	56608	ES	23:24	22	25	-0.20	1.5E-34	excluded
ARPC1B	80609	ES	8	7	9	-0.12	1.8E-34	excluded
DGUOK	54003	ES	5:06	4	7	-0.11	5.7E-34	excluded
SYTL2	18153	ES	11.2	10.3	12.2	-0.31	1.5E-33	excluded
LSR	49085	ES	4:05	3	6	-0.20	1.5E-33	excluded
SEC31A	100881	ES	27	26.1	28	-0.28	1.6E-33	excluded
DYSF	53937	ES	19	18	20	0.26	3.8E-33	included
NPIPA8	34239	ES	22	20	23	-0.15	3.9E-32	excluded
CACNB3	21476	ES	9	8	10	0.21	4.3E-32	included
PCYT2	44230	ES	7	6	8	0.17	6.8E-31	included
DGUOK	54004	ES	5	4	7	-0.11	9.0E-31	excluded
FAM86B1	82686	ES	5:6:7.1:7.2:7.3	4	8.1	-0.27	1.8E-30	excluded
TMEM180	12954	ES	6	5	7	-0.23	2.4E-30	excluded
HNRNPC	26558	ES	2.4	1	3.2	-0.08	8.5E-30	excluded
FN1	57395	ES	40.1:40.2	39	40.4	0.07	9.0E-30	included
TMEM126B	18122	ES	3	1	4	-0.12	9.2E-30	excluded
ITGB4	43489	ES	35	34	36	-0.28	1.8E-29	excluded
RANGRF	39167	ES	3.3	3.1	3.5	0.08	8.6E-29	included
SNAPC5	31278	ES	2.1	1.1	3.1	0.08	1.6E-28	included
MROH1	85546	ES	26	25.2	27	0.14	2.8E-28	included
TSTD1	8526	ES	2.2:2.3	1	3.1	0.16	2.9E-28	included
INF2	29547	ES	22	21	23	0.12	3.8E-28	included
KIAA1217	11009	ES	12	11	13	-0.17	4.7E-28	excluded
VKORC1	36229	ES	5	4.2	6	0.02	5.8E-28	included
UNC5B	12054	ES	8	7	9	0.28	8.5E-28	included
SEC31A	69727	ES	27	26.2	28	-0.21	1.2E-27	excluded
CSF2RA	88369	ES	13	12	14	-0.24	7.5E-27	excluded
HNRNPC	26552	ES	2.4:2.5:2.6	1	3.2	-0.17	2.1E-26	excluded
HCFC1R1	33354	ES	2	1.5	3.2	0.05	6.4E-26	included
TUBB3	38175	ES	6.1:6.2	5.4	7.1	-0.48	7.2E-26	excluded
LUC7L	32844	ES	4	3	5	-0.21	1.0E-25	excluded
KIF13A	75458	ES	28	27	29	0.13	1.4E-25	included
SLAIN2	69214	ES	8	6	9	0.12	2.5E-25	included
SSBP3	3144	ES	7	6	8	-0.11	2.5E-25	excluded
DYSF	53936	ES	43	42	44	0.14	2.7E-25	included
TBC1D15	23415	ES	10	9	11	-0.23	4.9E-25	excluded
DCTN1	54045	ES	27	26	28	0.01	5.3E-25	included
INCENP	16337	ES	11	10	12	0.17	7.2E-25	included
TUBA1B	21536	ES	2	1	3	-0.01	1.7E-24	excluded
SPTAN1	87770	ES	52	51.1	53	-0.04	1.7E-24	excluded
TCF20	62501	ES	4.1:4.2	3	5	-0.16	1.9E-24	excluded
NPIPA8	34240	ES	21	20	23	-0.17	1.9E-24	excluded
HNRNPC	26556	ES	2.2:2.3:2.4	1	3.2	-0.16	1.9E-24	excluded
EXOC7	43569	ES	7	6	8.2	-0.19	2.8E-24	excluded
MRPL33	53046	ES	3	2	4	0.08	3.0E-24	included
LUC7L	32848	ES	1.3:1.4	1.1	2.2	-0.28	7.1E-24	excluded
DST	76560	ES	106	105	107	-0.13	9.7E-24	excluded
TMEM107	39131	ES	2:3.2:3.4:3.5	1	3.7	0.12	1.2E-23	included

PARP3	65116	ES	02:03.1	1	3.2	0.24	1.3E-23	included
SEC31A	100885	ES	26.1:27	25.1	28	-0.24	1.5E-23	excluded
CD44	15142	ES	7:12.1:13:14	5	15	-0.27	1.5E-23	excluded
ARHGAP17	35664	ES	18	17	19	-0.16	3.3E-23	excluded
ABI1	11032	ES	12	11.2	13	-0.15	3.5E-23	excluded
MEAF6	1804	ES	7	5	8.1	-0.06	3.5E-23	excluded
COL6A3	58104	ES	6	5	7	0.24	6.4E-23	included
MORF4L2	89774	ES	04:05.3	3.2	6.2	0.15	8.4E-23	included
PSTPIP1	31973	ES	3	2.2	5	-0.29	8.8E-23	excluded
VEGFA	76330	ES	8.1:8.2	6	9.1	-0.08	1.2E-22	excluded
SYNE1	78183	ES	151	150	152	0.15	1.5E-22	included
TXNL4A	46276	ES	8	7.2	9	0.02	1.8E-22	included
SLC35F5	55070	ES	15	14	16.1	0.06	2.1E-22	included
CCNDBP1	30220	ES	10.1	9	10.3	-0.13	2.1E-22	excluded
SYTL2	18148	ES	13	12.2	14	0.27	2.2E-22	included
FAM86B1	82688	ES	7.1:7.2:7.3	4	8.1	-0.21	2.4E-22	excluded
PARP3	65119	ES	2	1	3.1	0.24	2.7E-22	included
ARAP1	17641	ES	32	31	33	0.11	4.3E-22	included
PRMT1	51044	ES	4.1	3	6	-0.06	8.9E-22	excluded
NPIPB5	35567	ES	3.3	2.5	5	-0.30	9.8E-22	excluded
FAM47E	69609	ES	4	3	5	0.15	1.5E-21	included
TSTD1	8527	ES	2.3	1	3.1	0.03	1.6E-21	included
RILP	38360	ES	7	6	8	-0.03	1.6E-21	excluded
PARP6	31525	ES	20	19	21	0.04	2.0E-21	included
ABLIM1	13209	ES	16	15	17	-0.14	3.5E-21	excluded
FAM86B1	82694	ES	7.1	4	8.1	-0.25	4.2E-21	excluded
LGALS9	39858	ES	5	4	6	0.16	4.8E-21	included
SNRPB	58543	ES	3	2	4	-0.03	6.0E-21	excluded
HN1	43378	ES	3	1	4	-0.03	6.4E-21	excluded
OFD1	98311	ES	21	20	22	-0.04	7.4E-21	excluded
MAP7D1	1761	ES	6	5	7.1	-0.05	9.2E-21	excluded
SHARPIN	85531	ES	7	6	8	0.03	1.4E-20	included
MYO1B	56609	ES	24	22	25	-0.12	1.6E-20	excluded
TPCN1	24630	ES	19	18	20	-0.05	1.9E-20	excluded
P4HA2	73262	ES	2.1:2.2	1	3	0.12	2.4E-20	included
EPB41L3	44533	ES	25	24	26	0.27	2.4E-20	included
GUSB	79855	ES	6	5.1	7	0.08	3.1E-20	included
CD44	15131	ES	7:8:9.2:10:11:12.1:13:	5	15	-0.25	3.3E-20	excluded
BRE	53054	ES	14	12	15	-0.07	3.4E-20	excluded
ACLY	40960	ES	14	13	15	0.11	4.3E-20	included
CAMTA1	507	ES	3	1	4	0.03	4.4E-20	included
ZFAND1	84309	ES	3	2	4	0.13	5.0E-20	included
G6PC3	41762	ES	5	4	6	-0.04	5.1E-20	excluded
EIF4A2	68056	ES	4	3	5	0.06	5.4E-20	included
PDS5B	25604	ES	33.1	32	34	-0.07	7.6E-20	excluded
ZFYVE21	29513	ES	6.1	5	7	-0.04	8.5E-20	excluded
MAP3K4	78357	ES	18	17	19	-0.15	1.0E-19	excluded
SBDS	79906	ES	2.1:2.2	1	3	0.03	1.0E-19	included
GPS1	44284	ES	1.3:1.4:1.5:1.6	1.1	2.3	0.04	1.1E-19	included
IPO11	72193	ES	30	29	33	-0.19	1.3E-19	excluded
SCNN1A	19842	ES	10	9	11	-0.20	1.8E-19	excluded
SYNE1	78184	ES	143	142	144	0.19	2.3E-19	included
IGSF3	4366	ES	6	5	7	-0.17	2.3E-19	excluded
MRPL52	26639	ES	4.1	2	5	0.01	2.7E-19	included
ASAP2	52611	ES	23	22	24	0.12	2.7E-19	included
GGCT	79138	ES	4	3	5	0.06	2.8E-19	included
DYSF	53938	ES	7	6	8	-0.13	3.0E-19	excluded
GALT	86203	ES	3.2:3.3:4	2	5	0.03	3.8E-19	included
PEX19	8457	ES	3	1	4	0.06	4.6E-19	included
IRF5	81735	ES	5	4	6.1	-0.20	4.7E-19	excluded
CFLAR	56799	ES	10	8.1	11	-0.17	5.2E-19	excluded
SERPINA1	29136	ES	2.4:2.5	1.1	3.2	0.15	5.4E-19	included

GRB7	40698	ES	16	15	17	0.09	5.6E-19	included
CD44	15128	ES	7:8:9.1:9.2:10:11:12.1	5	15	-0.25	6.4E-19	excluded
MFF	57806	ES	8:09:10	7	11	0.13	6.6E-19	included
HSD17B14	50818	ES	5	4.2	6	0.08	6.7E-19	included
CHCHD7	83913	ES	2.1:2.2	1	4	-0.09	7.0E-19	excluded
LUC7L	32850	ES	1.3	1.1	2.2	-0.20	1.2E-18	excluded
TTC8	28742	ES	10.1	9	11	-0.09	1.6E-18	excluded
ASPH	83975	ES	12	11	13	0.07	2.3E-18	included
CHID1	13810	ES	12	11	13	-0.02	2.7E-18	excluded
AK2	1652	ES	9	8.1	10	-0.06	3.1E-18	excluded
APEH	64893	ES	3	2	4	0.03	3.5E-18	included
CASC4	30336	ES	10	9	11	-0.10	3.6E-18	excluded
INPP5J	61825	ES	2	1	3.1	-0.12	4.7E-18	excluded
CIZ1	87718	ES	6	5	7	0.06	5.1E-18	included
MFF	57805	ES	8	7	9	0.07	5.5E-18	included
PFDN1	73648	ES	2	1	3.1	0.04	5.8E-18	included
CMTM8	63813	ES	2	1	3	-0.02	7.5E-18	excluded
CHCHD4	63518	ES	2	1	4	0.16	8.3E-18	included
SEC31A	69728	ES	26.2:27	26.1	28	-0.10	8.9E-18	excluded
MAP4K1	49669	ES	31	30	32	-0.16	1.1E-17	excluded
TTC8	101216	ES	20	19	21	0.04	1.1E-17	included
EXOC7	43571	ES	07:08.2	6	9	-0.16	1.2E-17	excluded
TMEM107	39135	ES	2:3.2:3.4	1	3.7	0.16	1.3E-17	included
PTBP2	3832	ES	12	11.2	13	-0.25	1.5E-17	excluded
FAM86B1	82706	ES	4:5:6:7.1	3.2	8.1	-0.19	1.5E-17	excluded
PKP2	21062	ES	6	5	7	-0.20	1.6E-17	excluded
PHLDB1	19040	ES	18.3	18.1	19	0.06	1.7E-17	included
NPIPB4	35510	ES	3.3	2.5	4	-0.29	1.8E-17	excluded
SRSF4	1424	ES	6	5	7	-0.11	2.1E-17	excluded
EPB41L1	59273	ES	20	19	21	-0.14	2.3E-17	excluded
GNPDA1	73865	ES	2.4	1	2.6	0.07	2.8E-17	included
BBIP1	13098	ES	3:04	1	5.1	-0.20	3.1E-17	excluded
NME4	32879	ES	4	2	5	0.12	3.1E-17	included
OPA1	68139	ES	7	6	8	-0.15	3.2E-17	excluded
RBM4	17096	ES	4.1	3.2	5	0.08	4.3E-17	included
RPS24	12296	ES	5.2	4	6	-0.07	4.4E-17	excluded
BPGM	81853	ES	3	1	4	0.07	4.5E-17	included
ZBTB8OS	1614	ES	2:03:04	1	6	0.08	4.6E-17	included
FAM86B1	82689	ES	5:6:7.1:7.3	4	8.1	-0.20	4.7E-17	excluded
TUSC3	82771	ES	11	10	12	0.10	4.7E-17	included
SBF1	62828	ES	29	28	30	-0.10	5.0E-17	excluded
PFDN5	22008	ES	4.1:4.2	1	5	-0.06	5.7E-17	excluded
ISOC2	52105	ES	03:04.1	2	4.2	-0.17	7.3E-17	excluded
PBX3	87592	ES	4	3	5	-0.05	7.5E-17	excluded
PPFIBP1	20893	ES	11	10	12	-0.16	7.7E-17	excluded
DNM2	47587	ES	15	14	17	0.07	7.9E-17	included
TPT1	25801	ES	1.4	1.1	2	0.00	9.0E-17	included
CHCHD7	83914	ES	2.1	1	4	-0.07	1.0E-16	excluded
BRD8	73509	ES	10	9	11	0.08	1.1E-16	included
TNC	87340	ES	12:13:14:15	11	16	-0.19	1.2E-16	excluded
PILRB	80935	ES	10	9	11	-0.09	1.2E-16	excluded
TPD52L1	77408	ES	9.1	8	10	0.06	1.2E-16	included
APOD	68181	ES	3	2	4	-0.16	1.2E-16	excluded
NPIPA5	34150	ES	8.3	8.1	9	-0.12	1.4E-16	excluded
BAIAP2	44095	ES	17.1:17.2	16.1	18.1	0.10	1.4E-16	included
TPM2	98133	ES	6	5	7	-0.27	1.4E-16	excluded
FAM86C1	17436	ES	4	3.2	5.1	-0.11	1.4E-16	excluded
SCRIB	85500	ES	36	35	37	0.09	1.5E-16	included
OARD1	76087	ES	5:06	4.1	7	0.03	1.5E-16	included
LRRC23	20003	ES	8	7.1	10	-0.18	1.8E-16	excluded
LCN12	127791	ES	4	3	5	-0.13	1.9E-16	excluded
LSR	49088	ES	3:04:05	2.2	6	-0.04	1.9E-16	excluded

LSR	49089	ES	3:05	2.2	6	-0.01	1.9E-16	excluded
CTNNA1	73565	ES	20	19	21	0.00	2.1E-16	excluded
PRMT1	51043	ES	4.1:4.2:5	3	6	-0.06	2.3E-16	excluded
LRRC23	20002	ES	8:09	7.1	10	-0.10	2.4E-16	excluded
DOCK9	26178	ES	48	47	49	0.11	2.6E-16	included
NSRP1	40102	ES	3	1	8	-0.13	2.8E-16	excluded
IVNS1ABP	9218	ES	9	8	10	-0.12	2.9E-16	excluded
FAS	12481	ES	6	5	7	-0.04	3.1E-16	excluded
FAM21A	11559	ES	28	27	29	0.06	3.1E-16	included
BIN1	55202	ES	7	6	8	-0.06	3.7E-16	excluded
PTK2	85305	ES	39.2	37	39.5	-0.14	4.3E-16	excluded
NPIPB5	35566	ES	3.1:3.2:3.3	2.5	5	-0.07	4.8E-16	excluded
PEA15	8435	ES	2:03	1	4.1	-0.02	4.8E-16	excluded
BIN1	55193	ES	16:17	12	18	-0.26	5.5E-16	excluded
MRPL52	26637	ES	4.1:4.2	2	5	0.14	6.0E-16	included
SERPINA1	29137	ES	2.5	1.1	3.2	0.11	6.5E-16	included
DBN1	74775	ES	14	13	15	0.01	9.5E-16	included
CD55	9637	ES	11:12.1:12.2	9	15	-0.05	1.1E-15	excluded
CAST	72863	ES	8.2	7.1	9	-0.08	1.3E-15	excluded
STAG3	80918	ES	13	12	14	-0.13	1.3E-15	excluded
HHLA3	3402	ES	4	3	5	0.10	1.4E-15	included
LYRM1	34414	ES	7	3	8.1	0.06	1.5E-15	included
ST7	81563	ES	10	9	11.1	0.13	1.6E-15	included
MFF	57797	ES	10	9	11	0.04	1.6E-15	included
CADM1	18850	ES	10:11	9	12	-0.05	1.7E-15	excluded
FIP1L1	69312	ES	14	13.1	15	-0.07	1.9E-15	excluded
SNRPD2	50513	ES	4	1.3	5.1	0.00	2.1E-15	excluded
TMUB2	41810	ES	2.3:2.4:2.5:3	2.2	4.3	-0.14	2.2E-15	excluded
MAP4K4	54762	ES	17	16.2	19	0.11	2.3E-15	included
EPS15L1	48160	ES	22:23.1	21	24	-0.13	2.3E-15	excluded
C11orf80	17129	ES	2:03	1	4	-0.13	2.3E-15	excluded
FMNL3	21605	ES	6	5	7	-0.12	2.3E-15	excluded
MAPK9	75012	ES	3	2	4	-0.11	2.4E-15	excluded
PAPOLA	29206	ES	19:20	18	21	0.04	2.9E-15	included
ZNHIT3	40478	ES	2	1	3.1	0.03	3.1E-15	included
GSE1	37883	ES	3	2	4	-0.16	3.5E-15	excluded
PTPRF	2190	ES	14	13	15	0.06	3.8E-15	included
CD164	77187	ES	6	5	7.1	0.02	4.1E-15	included
DNM1L	21046	ES	18	16	19	0.13	4.3E-15	included
SERPINA1	29132	ES	2.1:2.2:2.4:2.5	1.1	3.2	0.05	4.3E-15	included
HNRNPC	26550	ES	2.2:2.3:2.4:2.5:2.6	1	3.2	-0.05	4.3E-15	excluded
CD151	13783	ES	1.2:2	1.1	3	0.03	4.4E-15	included
VRK2	53645	ES	18	17	19	0.06	5.1E-15	included
D2HGDH	95828	ES	7.1	6	8	0.15	5.4E-15	included
UQCRQ	73320	ES	1.2:1.4	1.1	2	0.06	5.9E-15	included
AK2	1656	ES	2	1	3	-0.04	7.9E-15	excluded
PILRA	80941	ES	3	2	4	-0.13	8.8E-15	excluded
ISOC2	52106	ES	3	2	4.2	-0.02	1.1E-14	excluded
COX4I1	37910	ES	3	2.2	4.1	0.02	1.2E-14	included
GPX4	46389	ES	5	4	6	0.00	1.2E-14	included
STRAP	20589	ES	3	1	4	-0.03	1.4E-14	excluded
DSTN	58734	ES	2	1	3	-0.01	1.4E-14	excluded
CD44	15127	ES	6:7:8:9.1:9.2:10:11:12	5	15	-0.18	1.9E-14	excluded
LRRC28	32636	ES	11	10	12	0.05	2.0E-14	included
PFDN5	22006	ES	2	1	4.1	0.13	2.0E-14	included
RPS19BP1	62314	ES	3	2	4	0.01	2.1E-14	included
SLC25A36	67052	ES	6.1:6.2	4.1	7	0.17	2.6E-14	included
LRRFIP2	63960	ES	20	19	21	0.14	2.6E-14	included
CERS5	21686	ES	2	1	8	-0.08	2.6E-14	excluded
ANAPC16	12087	ES	2	1	3	0.01	2.7E-14	included
C1orf50	90934	ES	1.3	1.1	2.2	0.09	2.7E-14	included
PI4KA	61189	ES	37:38:39:40:41:42:43	36	44	0.10	2.8E-14	included

SSBP4	48432	ES	4	3	5	0.01	2.8E-14	included
FAM86B1	82693	ES	06:07.1	4	8.1	-0.17	3.2E-14	excluded
RAC1	78720	ES	4	3	5	0.06	4.4E-14	included
GEMIN2	27356	ES	5	4	6	-0.13	4.6E-14	excluded
PAM	72906	ES	14	13	15	0.11	5.1E-14	included
MORF4L2	89771	ES	4:5.1:5.2:5.3	3.2	6.2	0.14	5.1E-14	included
DLG1	68295	ES	9	7	10	0.12	5.3E-14	included
BMP1	82988	ES	17.1:17.2	16	18.1	-0.09	5.4E-14	excluded
RHEB	82380	ES	3	1.2	4	-0.01	5.6E-14	excluded
LCN10	123359	ES	4.2	2.2	5	0.13	5.6E-14	included
ABCA2	88259	ES	4.1	3	4.6	0.16	6.2E-14	included
SARNP	22254	ES	3	2	4	0.01	6.3E-14	included
RAPGEF2	71001	ES	17	16	18	-0.16	7.9E-14	excluded
CD44	15130	ES	6:7:8:9.2:10:11:12.1:1	5	15	-0.16	8.1E-14	excluded
RGS12	68643	ES	19	18	20.1	-0.09	1.0E-13	excluded
CDC42SE1	7556	ES	5	4	6	-0.08	1.1E-13	excluded
SEC31A	69730	ES	26.1:26.2:27	25.1	28	-0.11	1.2E-13	excluded
POLR3H	62434	ES	4	3	5.1	-0.05	1.2E-13	excluded
NONO	89420	ES	2:03	1	4	0.13	1.2E-13	included
SYNE4	102891	ES	2	1	3	0.08	1.3E-13	included
MRPL55	10071	ES	3	2.9	4.1	0.03	1.4E-13	included
ADORA1	9442	ES	4	3.2	5	-0.14	1.4E-13	excluded
TCF20	62502	ES	4.2	3	5	-0.12	1.5E-13	excluded
ATP6V0B	2511	ES	2.2	1.1	3.2	-0.02	1.7E-13	excluded
CPSF6	23304	ES	7	6.2	8.1	-0.05	1.7E-13	excluded
SNAPC5	31277	ES	1.2:2.1	1.1	3.1	0.14	1.7E-13	included
ATXN2L	35855	ES	22.2:22.4:22.5	22.1	22.6	0.09	1.8E-13	included
ITGA7	22216	ES	29	28	30	0.14	1.9E-13	included
GK	88735	ES	23	22	24	0.12	1.9E-13	included
ITGAE	38490	ES	28	27	29	0.14	2.0E-13	included
FAM86B1	82685	ES	5	4	6	-0.16	2.0E-13	excluded
HDDC2	77424	ES	3.1	2	5	0.06	2.1E-13	included
SYK	86821	ES	9	8	10	0.16	2.2E-13	included
PAM	72894	ES	23.2:25.2	22	26.1	0.09	2.3E-13	included
RPS3A	70825	ES	2.2:3.1:3.2	1.3	4.1	0.00	2.4E-13	included
BIN1	55200	ES	13	12	17	0.11	2.4E-13	included
LAMTOR3	70083	ES	2	1	3	0.03	2.5E-13	included
PPRC1	12938	ES	8:09	7	10	0.10	2.8E-13	included
CLSTN1	575	ES	11	10	12	-0.10	2.8E-13	excluded
MYL9	59288	ES	3	2	4	-0.02	2.9E-13	excluded
EHBP1	53718	ES	18	17	19	0.10	2.9E-13	included
OCEL1	48244	ES	4	3	5	0.03	3.3E-13	included
PDLIM3	71367	ES	5:06	4	7	-0.03	3.3E-13	excluded
PRKRA	56161	ES	4	3.1	5	-0.05	3.3E-13	excluded
MFGE8	32404	ES	8	7	9.1	-0.03	3.4E-13	excluded
NDUFV3	60764	ES	2	1	4	0.02	3.7E-13	included
MORF4L2	89775	ES	5.3	3.2	6.2	0.03	4.6E-13	included
PICALM	18172	ES	14.1:14.2	13	15	0.08	4.7E-13	included
MYO5A	30660	ES	35	34	36	0.09	4.8E-13	included
MPST	62073	ES	4	3	5	-0.06	4.9E-13	excluded
MRPL55	10148	ES	1.2:2.2:2.3:2.4:2.5:2.6	1.1	2.9	0.14	5.4E-13	included
EIF4A2	96628	ES	9.1:9.2	8	10	-0.14	5.4E-13	excluded
MORF4L2	89780	ES	4	3.2	5.3	0.05	5.6E-13	included
RAB34	39961	ES	4.2	3	5	-0.01	6.6E-13	excluded
ABI1	11038	ES	11.2:12	9	13	-0.14	6.7E-13	excluded
POLR2J3	81118	ES	4.1	2	4.3	0.12	6.9E-13	included
RCE1	17132	ES	7	6	8	0.03	7.1E-13	included
STAG2	90034	ES	35	34	36	-0.04	7.2E-13	excluded
CLTB	74653	ES	6	5	7	-0.02	7.3E-13	excluded
B3GALNT1	67502	ES	6:07	5	9.1	-0.18	7.9E-13	excluded
KIN	10723	ES	2	1	3	0.05	7.9E-13	included
LGMN	29004	ES	13:14	12	15	0.01	8.5E-13	included

GLB1L	57659	ES	6:07	5	8	0.07	8.9E-13	included
PTS	18764	ES	4	3	5	-0.02	9.2E-13	excluded
TFIP11	61515	ES	3	2.2	4	-0.13	9.5E-13	excluded
MUC15	14740	ES	4	3	5	-0.03	9.5E-13	excluded
DDX52	40542	ES	3	2	5	-0.12	9.9E-13	excluded
CLASP1	55177	ES	22	21	24	0.16	1.0E-12	included
MYO1B	56607	ES	23	22	24	-0.12	1.1E-12	excluded
MCCC2	72445	ES	7	6	8	-0.04	1.1E-12	excluded
PDDC1	13755	ES	4.1:4.2	3	5	0.23	1.2E-12	included
NRP1	11202	ES	14.1	12	15.1	-0.05	1.2E-12	excluded
MPRIP	39458	ES	20	19	21	-0.02	1.3E-12	excluded
HNRNPK	86714	ES	9	8	10	0.02	1.4E-12	included
CARD8	50715	ES	7.2	5	8	0.17	1.4E-12	included
PTBP1	46317	ES	9.1:9.2	8	10	0.04	1.4E-12	included
POLR2J2	81129	ES	3.1	2	3.3	0.13	1.4E-12	included
MEAF6	1801	ES	8.1:9.1	5	9.2	0.02	1.4E-12	included
SEP15	3687	ES	5	4	6	0.01	1.5E-12	included
PRDX5	16638	ES	2	1	3	-0.01	1.5E-12	excluded
BRE	53055	ES	13	12	15	-0.04	1.5E-12	excluded
UPP1	79637	ES	6.2:7	6.1	9	0.08	1.6E-12	included
U2AF1	60780	ES	3	2.2	4	-0.02	1.6E-12	excluded
PAM	72891	ES	23.2:25.1:25.2	22	26.1	0.11	1.7E-12	included
EIF4E2	58001	ES	8	6.1	9	-0.10	1.7E-12	excluded
LETMD1	21782	ES	2	1.2	7	0.05	1.8E-12	included
BIN1	55192	ES	13:16:17	12	18	-0.22	1.9E-12	excluded
PHLDB2	66064	ES	14:15	13	16	0.09	2.1E-12	included
SUB1	71656	ES	7	6	8	0.00	2.3E-12	excluded
DNM1L	21045	ES	17:18	16	19	0.13	2.3E-12	included
ABI1	11037	ES	11.1:11.2:12	9	13	-0.08	2.8E-12	excluded
DMKN	49191	ES	12	6.4	13	0.04	3.1E-12	included
SERPINA1	29127	ES	2.1	1.1	2.4	0.17	3.4E-12	included
RABEP2	35894	ES	3	2.2	4	-0.11	3.7E-12	excluded
BSG	46304	ES	3	2	4	-0.01	3.8E-12	excluded
DGUOK	54012	ES	3:04	1	7	0.05	4.0E-12	included
PAM	72893	ES	23.1:23.2:25.2	22	26.1	0.01	4.0E-12	included
PGAP2	14010	ES	7	6	10	0.13	4.1E-12	included
UBQLN1	86688	ES	8	7	9	0.03	4.1E-12	included
PKP4	55680	ES	25	24	26	-0.07	4.1E-12	excluded
ETHE1	50201	ES	3	2	4	0.04	4.1E-12	included
PLD3	49889	ES	04:05.1	1.2	5.2	0.12	4.5E-12	included
ANAPC11	44217	ES	3.3:6	3.2	7.2	0.07	5.0E-12	included
SCRIB	98107	ES	17	16	18	-0.10	5.0E-12	excluded
FAM49B	85147	ES	7.1:7.2:8	6	9	-0.01	5.1E-12	excluded
RPRD2	7472	ES	4	3.1	5	-0.10	5.6E-12	excluded
MXRA7	43613	ES	5	4	6	-0.04	5.8E-12	excluded
C9orf142	98264	ES	3	2	4	0.02	6.0E-12	included
NPIPB4	35509	ES	3.1:3.2:3.3	2.5	4	-0.07	6.2E-12	excluded
ANAPC11	44218	ES	6	3.2	7.2	0.06	6.3E-12	included
FGFR1	83430	ES	6	4	8.2	-0.19	6.6E-12	excluded
ECHDC1	77467	ES	7	6.3	10.2	0.05	7.1E-12	included
AMDHD2	93845	ES	8.3	8.1	9	0.06	7.2E-12	included
PPIL2	61252	ES	3	2	4	-0.05	7.3E-12	excluded
MAPK10	69826	ES	9	8.2	10	-0.16	7.5E-12	excluded
GPR137	16623	ES	9	8	10.1	-0.01	7.5E-12	excluded
BAIAP2	44096	ES	17.1	16.1	18.1	0.11	7.7E-12	included
PPIP5K2	72915	ES	28:29:00	27	30	-0.11	8.1E-12	excluded
FAM86C1	17441	ES	3.2	2	5.1	0.10	8.2E-12	included
HSF1	85559	ES	10	9	11	0.05	8.3E-12	included
RPL24	65970	ES	5.1	4	6	0.00	9.2E-12	included
DNAJC5	60176	ES	5	4	6	-0.06	9.6E-12	excluded
GNLY	54377	ES	2.1:2.2	1	3	-0.08	9.7E-12	excluded
UPP1	79641	ES	05:06.1	4	9	-0.06	1.1E-11	excluded

NDUFV3	60762	ES	3	2	4	0.10	1.1E-11	included
MGST2	70634	ES	3	2	4	-0.02	1.2E-11	excluded
HOPX	69379	ES	3.2	1.2	4.6	0.02	1.2E-11	included
RAB9A	88512	ES	2	1	3	-0.07	1.2E-11	excluded
FGFR2	13318	ES	4	3	5	-0.09	1.2E-11	excluded
CHEK2	61544	ES	2	1	3	-0.16	1.3E-11	excluded
EMC3	63300	ES	2	1	3	-0.01	1.3E-11	excluded
APEH	64892	ES	7	6	8	0.02	1.3E-11	included
SLC50A1	7944	ES	4	3	5	-0.04	1.4E-11	excluded
PSMA4	32105	ES	9	8	10	0.00	1.4E-11	included
CD44	15406	ES	3.1:3.2:4:5:15:16.1:16	2.1	17.2	0.06	1.4E-11	included
MAGOH	3055	ES	3	2	4	-0.02	1.5E-11	excluded
TIMM8B	18730	ES	2	1	3	0.06	1.5E-11	included
MROH7-TTC4	3152	ES	4	3	5	0.06	1.6E-11	included
PFKM	21422	ES	16.3	16.1	17	-0.05	1.6E-11	excluded
RREB1	75253	ES	12	11	13	0.13	1.7E-11	included
NDUFAF7	53222	ES	5.2:6	5.1	7	0.08	1.7E-11	included
PAM	72890	ES	23.1:23.2:25.1:25.2	22	26.1	0.02	1.8E-11	included
BIN1	55195	ES	17	12	18	-0.06	1.9E-11	excluded
RAB25	8171	ES	2	1	3	0.01	1.9E-11	included
SETD9	72117	ES	2	1	3	0.08	1.9E-11	included
GIT2	24370	ES	19	18.2	20	0.09	2.0E-11	included
FABP3	1467	ES	3	2	4	-0.08	2.2E-11	excluded
IGFLR1	49261	ES	3:4.1:4.2:4.3	2.2	5	-0.08	2.3E-11	excluded
LMO7	26067	ES	12	9	13	0.17	2.3E-11	included
GSN	87433	ES	11	10	16	-0.01	2.5E-11	excluded
WAC	11105	ES	9	8	10	0.04	2.5E-11	included
GMPR2	26924	ES	4	3.2	5	-0.03	2.5E-11	excluded
DMKN	49143	ES	20.2:21	20.1	22	0.05	2.6E-11	included
DMKN	49187	ES	11:12	6.4	13	0.14	2.6E-11	included
B3GALNT1	67503	ES	7	5	9.1	-0.07	2.7E-11	excluded
RPS3A	96814	ES	2.2	1.3	4.1	0.07	2.8E-11	included
CD44	14976	ES	14	13	15	-0.01	2.9E-11	excluded
IMMP1L	14817	ES	4:05:06	1	7	0.11	2.9E-11	included
TOP3B	61270	ES	6	5	7	-0.14	2.9E-11	excluded
BBIP1	13089	ES	6	5.2	7	-0.04	3.1E-11	excluded
CTSB	97871	ES	2	1.1	3.1	0.08	3.1E-11	included
EPB41L1	59274	ES	18	17	19	-0.02	3.2E-11	excluded
APP	60283	ES	10	9	11	0.07	3.2E-11	included
APBB2	69101	ES	8	7.2	9	0.09	3.3E-11	included
LGMN	29005	ES	14	12	15	0.15	3.3E-11	included
BCAS4	59782	ES	6	5	7	-0.05	3.4E-11	excluded
PLD3	49893	ES	1.2:4:5.1	1.1	5.2	0.15	3.7E-11	included
NARF	44397	ES	11.2:12	11.1	13.1	0.03	3.7E-11	included
TMEM107	39130	ES	2:3.1:3.2:3.4:3.5	1	3.7	0.11	3.8E-11	included
PRMT2	60964	ES	1.3	1.1	2	-0.10	3.8E-11	excluded
APOC1	50359	ES	6	5.1	7	-0.03	3.8E-11	excluded
SRSF4	1425	ES	2:03:04	1	5	-0.11	3.9E-11	excluded
MRPL22	74297	ES	2.2	1.3	3	0.02	4.1E-11	included
UGP2	53757	ES	8	6	9	-0.02	4.3E-11	excluded
FLNA	90565	ES	30	29	31	0.07	4.3E-11	included
GOLGA8J	93641	ES	5.2:6.1	5.1	6.2	0.09	4.4E-11	included
PPP1CA	17184	ES	2.2	1	3	0.00	4.5E-11	excluded
DGUOK	54011	ES	4:05	1	7	-0.11	4.6E-11	excluded
TBCA	72602	ES	05:06.1	4	6.3	0.10	4.9E-11	included
EIF4A2	68054	ES	11	10	12	-0.04	5.2E-11	excluded
PPIL3	127875	ES	2.2:3:4.1	1.1	4.2	0.13	5.3E-11	included
RHOA	64860	ES	4	2	6	-0.07	5.4E-11	excluded
PFDN5	22001	ES	4.1:4.2:5	1	6.2	-0.13	5.6E-11	excluded
LIAS	69062	ES	7	6	8	0.05	6.1E-11	included
D2HGDH	58422	ES	7.1:7.2:7.3	6	8	0.17	6.1E-11	included
RNF167	94344	ES	2.2:2.3	1.2	2.5	0.07	6.3E-11	included

TMEM126A	18130	ES	2.1:2.2	1	3.2	-0.02	6.5E-11	excluded
EXOC7	43568	ES	07:08.1	6	8.2	-0.13	7.3E-11	excluded
PBX3	87590	ES	9	8	10	-0.05	7.3E-11	excluded
COPS7B	57943	ES	9.1:9.2	8	10	-0.03	7.6E-11	excluded
HIPK3	14893	ES	14	13	15	-0.10	7.8E-11	excluded
AMZ2	43129	ES	6:07	5	8	0.03	7.9E-11	included
RNF146	77452	ES	03:05.1	2	6	-0.12	8.0E-11	excluded
NDUFAF6	84598	ES	9.2:10	9.1	11	-0.12	8.2E-11	excluded
EXOC7	43570	ES	7:8.1:8.2	6	9	-0.12	8.2E-11	excluded
PQBP1	89026	ES	6	5	7	0.01	8.3E-11	included
SP100	57907	ES	3	1	5	0.04	9.0E-11	included
ACSF3	38061	ES	6	5	7	0.03	9.2E-11	included
DCAF6	8886	ES	11:13.1	10	14	0.07	9.4E-11	included
EEF1D	85442	ES	9	8.3	10.1	0.01	9.5E-11	included
CDC42BPB	29428	ES	23	22	24	-0.05	1.0E-10	excluded
RPS25	19057	ES	2.1:2.2	1	3.1	-0.08	1.1E-10	excluded
C12orf73	24076	ES	5	2.4	6	-0.14	1.1E-10	excluded
COBLL1	55787	ES	13:14	12	15	-0.06	1.1E-10	excluded
MYO6	76802	ES	34	33	35	-0.02	1.1E-10	excluded
PHPT1	88225	ES	4	3	5	-0.03	1.2E-10	excluded
MPI	31777	ES	7	6.1	8	0.04	1.2E-10	included
OARD1	76086	ES	6	5	7	0.03	1.2E-10	included
MTA1	29645	ES	18	17	20	0.01	1.3E-10	included
MORF4L2	89776	ES	4	3.2	5.1	0.06	1.4E-10	included
EXOC7	43573	ES	7	6	9	-0.08	1.4E-10	excluded
U2AF1	60778	ES	4	3	5	-0.06	1.4E-10	excluded
C21orf59	60360	ES	5.3	5.1	5.5	0.01	1.5E-10	included
RNF10	24772	ES	2	1	3	-0.05	1.5E-10	excluded
PAPOLA	29205	ES	19	18	20	0.04	1.6E-10	included
SMYD2	9791	ES	9:10	8	11	0.01	1.6E-10	included
KIF12	102262	ES	5	4.1	6	-0.09	1.6E-10	excluded
THEMIS2	1352	ES	3:4.1:4.2:4.3	2	5	0.11	1.7E-10	included
ADAM15	7897	ES	21.1:21.2	20	22.1	-0.10	1.7E-10	excluded
TIA1	53874	ES	6	5	7	-0.12	1.8E-10	excluded
DMKN	49168	ES	11:12	7	13	0.07	1.8E-10	included
CC2D2A	68809	ES	5	4	6	-0.09	2.0E-10	excluded
FAM86B1	82687	ES	6:7.1:7.2:7.3	4	8.1	-0.11	2.0E-10	excluded
S100A2	7721	ES	5	4.3	6	0.02	2.1E-10	included
TPD52L1	77417	ES	9.1	6	10	0.04	2.1E-10	included
CASK	88866	ES	20	19.1	22	-0.13	2.4E-10	excluded
NRD1	2986	ES	26	25	27	-0.03	2.6E-10	excluded
SLC22A17	102809	ES	7	6	8.1	0.03	2.7E-10	included
ANXA6	74148	ES	22	21	23	0.06	2.8E-10	included
PPHLN1	21221	ES	10	9	11	0.03	2.8E-10	included
C12orf73	24074	ES	04:01.1	2.4	6	-0.19	2.9E-10	excluded
ADAM15	7905	ES	22.1	20	23	0.05	2.9E-10	included
RWDD1	77328	ES	3	1	4	0.06	2.9E-10	included
NUMA1	17517	ES	17	16	19	-0.01	2.9E-10	excluded
S100A4	7714	ES	3	2	5	-0.02	2.9E-10	excluded
NDUFA3	95376	ES	4.1:4.3	2	5.1	0.07	3.0E-10	included
TARSL2	32772	ES	19	18	20	-0.03	3.1E-10	excluded
SMAGP	21834	ES	5	3.3	6.1	-0.10	3.1E-10	excluded
BBIP1	13099	ES	3	1	5.1	-0.04	3.2E-10	excluded
RHOC	4232	ES	4	3	5	-0.01	3.3E-10	excluded
TPO	52537	ES	17	16	18	-0.05	3.4E-10	excluded
CADM1	18856	ES	9:10	8	12	-0.05	3.5E-10	excluded
DMKN	49186	ES	7:11:12	6.4	13	0.09	3.5E-10	included
ADAM15	7898	ES	21.2	20	22.1	-0.10	3.7E-10	excluded
HSD17B14	50817	ES	8	7	9	0.04	3.7E-10	included
KCTD7	79882	ES	13	12	14	-0.16	3.9E-10	excluded
RWDD2B	60299	ES	3	2	4	0.06	3.9E-10	included
DCTN2	22644	ES	8	2	10	-0.06	4.0E-10	excluded

CCDC74A	55391	ES	1.2:2	1.1	3.1	-0.05	4.1E-10	excluded
PAM	72902	ES	23.2	22	25.2	0.05	4.2E-10	included
CARM1	47599	ES	16.1	15	16.3	-0.06	4.3E-10	excluded
CHD3	39069	ES	34	33	35	0.05	4.4E-10	included
GPX8	72028	ES	2	1.2	3	0.02	4.5E-10	included
ZNF814	95403	ES	02:03.1	1	9	0.08	4.5E-10	included
RALY	59017	ES	5	4	6	0.03	4.8E-10	included
KIF12	102260	ES	7	6	8	-0.04	5.0E-10	excluded
RHOA	64861	ES	3	2	4	0.00	5.0E-10	included
ALG8	18011	ES	14	13	15	0.08	5.2E-10	included
TSPAN14	12377	ES	5	1	6	0.07	5.2E-10	included
C1D	53822	ES	2.2	1.1	3.1	-0.06	5.4E-10	excluded
ARHGAP8	62627	ES	13	11	14	0.08	5.4E-10	included
DMKN	49171	ES	12	7	13	0.01	6.0E-10	included
APOPT1	29463	ES	2:03	1	5	0.03	6.1E-10	included
BIN1	55184	ES	16	13	17	-0.08	6.2E-10	excluded
DGUOK	54010	ES	2:04:05	1	7	-0.02	6.2E-10	excluded
SGSM2	38394	ES	11	10	12	0.08	6.4E-10	included
SDHAF2	16234	ES	03:04.1	1	6	0.05	6.5E-10	included
ACD	94172	ES	9	8	10	0.03	6.5E-10	included
ERCC1	50443	ES	9	8	10.1	-0.01	6.8E-10	excluded
REPS1	77956	ES	9.3	9.1	10	0.09	6.9E-10	included
PHYHIP	82997	ES	2	1	3	-0.20	7.0E-10	excluded
RWDD1	77326	ES	2	1	3	-0.06	7.1E-10	excluded
CHTF8	37269	ES	4.3:4.4	4.1	4.6	0.07	7.6E-10	included
MON2	22839	ES	31	30	32	0.11	7.9E-10	included
FBXO44	657	ES	5.2:6	5.1	7	0.06	8.1E-10	included
SUGT1	26006	ES	7	6	8	0.07	9.0E-10	included
MTMR3	61690	ES	20	19	21	0.04	9.1E-10	included
MPP1	90665	ES	2	1	4	-0.02	9.1E-10	excluded
NMRK1	86630	ES	3	2	4	-0.07	9.4E-10	excluded
PPIL3	127874	ES	1.2:2.2:3:4.1	1.1	4.2	0.13	9.6E-10	included
PAM	72896	ES	23.1:23.2	22	26.1	0.04	9.9E-10	included
SERPINA1	29135	ES	1.2:2.4:2.5	1.1	3.2	0.02	9.9E-10	included
ARVCF	96084	ES	20	19	21	-0.07	1.0E-09	excluded
DNM1L	21047	ES	17	16	19	0.11	1.0E-09	included
PSMD13	13633	ES	3.1	2	3.3	0.01	1.0E-09	included
HSP90B1	24063	ES	3	2	4	0.00	1.0E-09	excluded
WNK1	19617	ES	14:15	13.2	16	-0.13	1.0E-09	excluded
ARID1B	78237	ES	12	11	13	0.10	1.1E-09	included
P4HA2	73260	ES	2.1:2.2:2.3	1	3	0.03	1.1E-09	included
DDX49	48529	ES	3.1:3.2	2.2	4	-0.04	1.3E-09	excluded
BMP1	82989	ES	17.2	16	18.1	-0.05	1.3E-09	excluded
GPR137	16625	ES	6:07	5	8	-0.01	1.3E-09	excluded
SEPT10	54907	ES	12.1:12.2	11.1	13	-0.06	1.3E-09	excluded
NSMCE1	35680	ES	4.1:4.3	3	5	0.00	1.4E-09	excluded
APP	60286	ES	9:10	8	11	0.01	1.4E-09	included
ACPL2	67063	ES	6	5	9	-0.08	1.4E-09	excluded
TBCA	72604	ES	5	4	6.1	0.00	1.5E-09	included
NPIPB5	35568	ES	3.1	2.5	5	-0.11	1.5E-09	excluded
PFDN5	22009	ES	4.2	1	5	-0.03	1.5E-09	excluded
RUFY1	74895	ES	9	8	10	-0.03	1.5E-09	excluded
CCDC50	68126	ES	6	5	7	-0.12	1.5E-09	excluded
SUGT1	26007	ES	4	3	5	-0.02	1.6E-09	excluded
PSMB5	26688	ES	3	2	4	-0.01	1.6E-09	excluded
C7orf41	79113	ES	3	1	4.1	-0.07	1.6E-09	excluded
POLL	12902	ES	1.2:1.3:1.5	1.1	3	0.20	1.7E-09	included
FCHSD1	73817	ES	19	18	20	-0.08	1.7E-09	excluded
ATP5C1	10726	ES	9	8.1	10	-0.02	1.7E-09	excluded
RANBP3	46966	ES	5:06	4.1	7	-0.05	1.7E-09	excluded
C16orf13	32915	ES	4	3	5	0.06	1.7E-09	included
PACRGL	68883	ES	12	9	13.1	0.10	1.8E-09	included

SMURF2	43074	ES	4	3	5	-0.10	1.8E-09	excluded
LETMD1	21773	ES	2:4:5:6	1.2	7	0.09	1.8E-09	included
FERMT2	27560	ES	14	13	15	-0.02	1.8E-09	excluded
PAK1	17954	ES	16	15	17.1	0.02	1.8E-09	included
RAB34	39960	ES	4.1:4.2	3	5	-0.09	1.9E-09	excluded
BCL2L12	51035	ES	3.1:3.2	2	4	0.04	1.9E-09	included
HAUS1	45389	ES	3	2.1	4	-0.08	1.9E-09	excluded
SERPINH1	17871	ES	2.2	1	3	-0.02	1.9E-09	excluded
SRSF2	43666	ES	2.3	2.1	2.5	-0.05	2.1E-09	excluded
SKA2	42757	ES	2	1.1	5	0.09	2.1E-09	included
WHSC1	68535	ES	7	6	8	0.03	2.1E-09	included
C11orf49	15633	ES	3:04	1	7	-0.12	2.2E-09	excluded
LPHN2	3565	ES	28	27	31	0.13	2.2E-09	included
MRPL55	10128	ES	1.2:2.2:2.3:2.4:2.5:2.6:	1.1	2.9	0.13	2.2E-09	included
OAZ1	46598	ES	3.1:3.2:3.3:3.4:3.5	1	4	0.00	2.3E-09	included
EIF4G1	67910	ES	2.2	1	2.4	0.05	2.3E-09	included
SMARCE1	40871	ES	12	11	13	-0.01	2.5E-09	excluded
DUSP6	23618	ES	2.2	1	3	0.01	2.5E-09	included
NF2	61626	ES	16.1	15	17	0.08	2.5E-09	included
CERS5	21681	ES	6.1:6.2	1	8	-0.11	2.7E-09	excluded
SETD4	60518	ES	4	3.4	5	-0.10	2.7E-09	excluded
RIPK2	84385	ES	3	1	4	-0.07	2.8E-09	excluded
SNRPD2	50514	ES	3	1.3	5.1	0.00	2.8E-09	excluded
PLK3	2578	ES	13	12	14	0.05	2.8E-09	included
DCAF6	8887	ES	13.1	10	14	0.08	2.8E-09	included
PPIL2	61250	ES	12	11	13	-0.02	3.1E-09	excluded
FBLN5	28893	ES	7	5	8	-0.13	3.1E-09	excluded
ALDH5A1	75516	ES	5	4	6	0.06	3.3E-09	included
ELP6	64531	ES	4.1	3	5	0.02	3.3E-09	included
FAM86C1	17437	ES	3.1:3.2:4	2	5.1	-0.11	3.5E-09	excluded
MGST1	20606	ES	6	5	7	0.01	3.6E-09	included
PAM	72904	ES	23.2	22	25.1	0.03	3.6E-09	included
TRAPPC2L	38050	ES	3.3:4.4	3.2	5.1	0.00	3.8E-09	included
SRSF7	53274	ES	7	6	8	0.02	3.9E-09	included
ZNF667	52180	ES	02:03.2	1	6	-0.14	4.1E-09	excluded
MDP1	26908	ES	4.2:5	4.1	6	0.02	4.1E-09	included
CACNB3	21480	ES	4	3	5	-0.13	4.2E-09	excluded
USE1	48241	ES	2	1	3	0.01	4.2E-09	included
WNK1	19618	ES	15	13.2	16	-0.11	4.5E-09	excluded
OCIAD1	69246	ES	2.2:3:4	2.1	6	0.12	4.5E-09	included
C14orf2	29533	ES	3:05	2	7.1	0.11	4.6E-09	included
ITGB4	43490	ES	33	32	34	-0.04	4.6E-09	excluded
RPN2	59332	ES	17	16	18	0.00	5.1E-09	included
EIF4H	80063	ES	5	4	6	-0.02	5.1E-09	excluded
D2HGDH	58421	ES	8:09	6	11.2	-0.06	5.1E-09	excluded
RPL8	85642	ES	4	3.2	5	0.00	5.1E-09	included
UPP1	97460	ES	7	6.2	9	0.04	5.2E-09	included
PAM	72899	ES	23.2:25.1	22	25.2	0.04	5.2E-09	included
TBC1D1	69017	ES	13:14	12	15	-0.03	5.4E-09	excluded
TPM1	115012	ES	12.1	11.1	13.1	0.17	5.6E-09	included
HADH	70285	ES	2.2	1	4	-0.01	5.6E-09	excluded
NDUFAF6	84597	ES	10	9.2	11	-0.12	5.6E-09	excluded
IL17RE	63251	ES	3	2.2	4	0.10	5.6E-09	included
ARRB2	38564	ES	10	9	11	0.01	5.9E-09	included
FAM86B1	82698	ES	4:5:6:7.1:7.2:7.3	3.2	8.1	-0.13	5.9E-09	excluded
BZW2	78864	ES	6.1:6.2	5	8	0.01	6.0E-09	included
VDAC2	12273	ES	7	6	8	-0.01	6.0E-09	excluded
PUM2	52774	ES	16	15	17	0.07	6.2E-09	included
SDHAF2	16230	ES	3:4.1:5.1:5.2	1	6	0.05	6.3E-09	included
CEP57	18392	ES	10.3	10.1	11	-0.04	6.4E-09	excluded
APMAP	58855	ES	8	7	9	0.02	6.4E-09	included
CERS5	21661	ES	15	14	16	-0.01	6.4E-09	excluded

CAMK2B	79489	ES	18:19:20	17	21.1	0.13	6.8E-09	included
ITGB2	60853	ES	2	1	4.2	-0.04	6.8E-09	excluded
OS9	22693	ES	13	12	14	-0.04	7.1E-09	excluded
NR1H3	15701	ES	8.1	7	8.3	0.03	7.2E-09	included
PBX1	8790	ES	11	10	12	-0.07	7.2E-09	excluded
PAM	72903	ES	23.1:23.2	22	25.1	0.05	7.4E-09	included
CCNDBP1	30222	ES	3	2	4	0.01	7.4E-09	included
LYRM1	34413	ES	5:07	3	8.1	0.11	7.6E-09	included
KLHL12	9424	ES	12	11	13	0.02	7.8E-09	included
TOM1	61965	ES	4	3	5	-0.01	7.8E-09	excluded
STYXL1	80150	ES	5	4	6	0.03	7.9E-09	included
PEX2	84241	ES	4.2	2.2	5	0.16	8.1E-09	included
PRDM16	303	ES	17	16	18.1	-0.09	8.7E-09	excluded
NPIPB4	35511	ES	3.1	2.5	4	-0.11	8.9E-09	excluded
LETMD1	21780	ES	02:03.2	1.2	7	0.13	9.0E-09	included
CLK3	31728	ES	5	4	6	0.02	9.1E-09	included
ATXN2L	35846	ES	22.2:22.4:22.5:22.6	22.1	22.7	0.11	9.3E-09	included
HDAC7	21370	ES	27	26	28	-0.02	9.3E-09	excluded
ARAP3	73825	ES	32	31	33	-0.04	9.7E-09	excluded
CHTOP	7749	ES	4.4	4.2	5	0.01	9.8E-09	included
ANAPC11	44211	ES	6	3.3	7.2	0.05	9.9E-09	included
ZNF267	36293	ES	4	3	5	-0.08	9.9E-09	excluded
THUMPD2	53337	ES	4	3	5	-0.12	1.0E-08	excluded
PFDN5	21992	ES	02:04.1	1	4.2	0.08	1.1E-08	included
MAGI2	80218	ES	5	4	6	-0.05	1.1E-08	excluded
RMDN1	84375	ES	9.1	8	10	0.02	1.2E-08	included
NRD1	2987	ES	4:05	3	6	-0.08	1.2E-08	excluded
GGCT	79135	ES	4:05	3	7	0.01	1.3E-08	included
CADM1	18854	ES	9:10:11	8	12	-0.11	1.3E-08	excluded
TBC1D22A	62728	ES	8	7	9	-0.01	1.3E-08	excluded
EIF4B	21918	ES	8	7	9	0.00	1.3E-08	excluded
CHTF8	37273	ES	4.1	3	4.3	-0.04	1.3E-08	excluded
EMC10	51201	ES	7	6	8.1	-0.08	1.4E-08	excluded
GPS1	44286	ES	1.5:1.6	1.1	2.3	0.02	1.4E-08	included
PTDSS2	13660	ES	2.1:2.2	1	3	0.04	1.4E-08	included
CAPN3	30157	ES	23:24	21	25	0.13	1.4E-08	included
ZNF185	90401	ES	15	14	16	-0.09	1.6E-08	excluded
TRAPPC2	88518	ES	2.1:2.2	1	3	0.11	1.6E-08	included
COPZ1	22173	ES	3.2	1	4	0.01	1.6E-08	included
RRBP1	58743	ES	2	1	3.1	-0.05	1.6E-08	excluded
MXD4	68555	ES	3	2	4	-0.01	1.7E-08	excluded
NBPF12	7351	ES	67:68:69:70:71:72:73:<br>74:75:76:77:78:79:80:	66	85	-0.03	1.7E-08	excluded
GIPC1	47980	ES	4	3	5	0.04	1.7E-08	included
APOPT1	29461	ES	3	2	5	0.02	1.7E-08	included
ABI2	57021	ES	11	10	12	-0.02	1.8E-08	excluded
MYO6	76806	ES	29:30:31	28.1	32	0.07	1.9E-08	included
TIMP1	88929	ES	2	1	3.1	0.00	1.9E-08	included
ABHD11	80027	ES	4:05	3	6	0.02	2.0E-08	included
SAE1	50623	ES	7:08	6	9	-0.07	2.0E-08	excluded
HEXA	31552	ES	2	1	3.2	-0.01	2.0E-08	excluded
NDUFAF6	84596	ES	12	11	14	-0.05	2.0E-08	excluded
PLXDC1	40623	ES	13:14.1	12	14.2	-0.12	2.0E-08	excluded
PRPF40A	55608	ES	8	7	9	-0.07	2.1E-08	excluded
PFDN5	22002	ES	4.2:5	1	6.2	-0.11	2.1E-08	excluded
FBXO25	82548	ES	4	3	5	0.03	2.1E-08	included
BRD4	48070	ES	5	4	6	-0.04	2.2E-08	excluded
NUMB	28293	ES	11	10	12	0.02	2.2E-08	included
TPRA1	66613	ES	9	8	10	0.02	2.2E-08	included
NPTN	31606	ES	2	1	3	-0.01	2.2E-08	excluded
SPAG16	57337	ES	7	5	8.1	0.01	2.2E-08	included
RHBDD2	80128	ES	2	1	3	0.05	2.3E-08	included

MPPE1	44652	ES	9.2	8	10	0.03	2.5E-08	included
CSF2RA	88367	ES	13:14	12	15	-0.05	2.5E-08	excluded
RPL30	84636	ES	2.2:3.1	2.1	3.2	-0.05	2.6E-08	excluded
SYNE4	122025	ES	5	4	6	-0.04	2.6E-08	excluded
BLOC1S6	30437	ES	05:07.1	3	7.2	-0.16	2.7E-08	excluded
RPL19	40632	ES	1.2:1.3:2.1	1.1	2.2	-0.08	2.7E-08	excluded
STX5	16448	ES	2.1:2.2	1	4	0.00	2.8E-08	included
LARP7	70388	ES	6	5.2	7	0.02	2.8E-08	included
MAP2	57225	ES	13	12	14	-0.10	2.8E-08	excluded
PPP4C	36058	ES	3.2	2.2	4	0.01	2.8E-08	included
CD44	15108	ES	08:09.2	5	10	-0.11	2.9E-08	excluded
COMMD9	15421	ES	4	3	5	0.01	2.9E-08	included
DGUOK	54002	ES	6	5	7	0.02	3.0E-08	included
CPSF7	16217	ES	4	3	6.1	-0.04	3.0E-08	excluded
ARMC4	11085	ES	24	23	25	-0.05	3.0E-08	excluded
RRN3	34139	ES	12:13	11.1	14	0.15	3.0E-08	included
CD59	14916	ES	2	1	3	-0.02	3.0E-08	excluded
TJP2	86537	ES	23:24	22.1	25	0.01	3.1E-08	included
NVL	9944	ES	11	10	13	-0.05	3.1E-08	excluded
REPIN1	82240	ES	4.1:4.2	3.2	5.2	-0.09	3.1E-08	excluded
GTPBP8	66128	ES	5	4	6	0.04	3.2E-08	included
PRKAG1	21508	ES	03:04.1	2	4.2	0.12	3.2E-08	included
ZNF608	73151	ES	9	8	10	-0.09	3.2E-08	excluded
PICALM	18170	ES	19	18	20	-0.03	3.3E-08	excluded
ATXN2L	35858	ES	22.2:22.4	22.1	22.6	0.01	3.4E-08	included
CAMTA2	38634	ES	7.1:7.2:7.3	6	8	0.10	3.5E-08	included
RNF216	78681	ES	4:5.1:5.2:6.2	2	7	0.11	3.5E-08	included
PIGT	59573	ES	2.1:2.2:3	1	4	-0.01	3.6E-08	excluded
PALLD	71133	ES	14	12	15	0.12	3.7E-08	included
CCT7	53959	ES	6	5.2	7	0.00	3.7E-08	included
RABL6	88222	ES	6	5	7	-0.01	4.0E-08	excluded
SMAD2	45451	ES	4	3.2	5	-0.01	4.0E-08	excluded
SPTAN1	87774	ES	23	22	24	0.06	4.0E-08	included
FXYD5	49073	ES	2	1.6	3.1	0.00	4.0E-08	excluded
PPA1	12045	ES	7	6	8	-0.02	4.1E-08	excluded
ESD	25844	ES	4	3	5	-0.01	4.1E-08	excluded
GPBP1	72129	ES	4	3.1	6	0.04	4.2E-08	included
INSR	47099	ES	11	10	12	0.09	4.2E-08	included
ACOT9	88695	ES	5.1:6	4	7.1	0.05	4.3E-08	included
GNAS	60006	ES	6	5	8.1	-0.04	4.3E-08	excluded
EXOC1	69335	ES	11	10	12	-0.09	4.4E-08	excluded
ANKRD13D	17158	ES	2	1	3.1	0.07	4.5E-08	included
EPB41L1	59271	ES	22.1:22.2	21	23	-0.06	4.7E-08	excluded
AGPAT2	88194	ES	4	3	5	-0.01	4.7E-08	excluded
LETMD1	21771	ES	2:3.2:4:5:6	1.2	7	0.05	4.8E-08	included
RCSD1	8870	ES	3	2	4	0.08	4.9E-08	included
FAM175A	69802	ES	3	2	4	-0.12	5.0E-08	excluded
DMKN	49203	ES	7:08	6.4	12	-0.07	5.0E-08	excluded
FAXC	77051	ES	3:04	2	5	-0.06	5.0E-08	excluded
CD44	15058	ES	6:7:8:9.2:10:11:12.1:1	5	14	-0.17	5.0E-08	excluded
CSDE1	4333	ES	6	5	7	-0.02	5.1E-08	excluded
DMKN	49190	ES	7:12	6.4	13	0.02	5.2E-08	included
HERC4	11916	ES	19	18	20	0.03	5.3E-08	included
ARFIP2	14139	ES	2.1:2.2	1	3	-0.02	5.3E-08	excluded
VPS51	16760	ES	2	1	4	0.01	5.4E-08	included
BCAP29	81361	ES	10	9.1	12	-0.03	5.4E-08	excluded
AP1G2	26770	ES	2	1.4	3	0.03	5.5E-08	included
TM2D3	32771	ES	1.3	1.1	2.2	-0.06	5.6E-08	excluded
PRKDC	83791	ES	81	80	82	-0.06	5.7E-08	excluded
MFF	57810	ES	9	7	11	-0.05	5.9E-08	excluded
ZFAND5	86600	ES	3	1	4.1	-0.05	5.9E-08	excluded
RHOT1	40190	ES	3	2.2	4	0.05	6.0E-08	included

TFR2	80979	ES	10	9	11	-0.06	6.1E-08	excluded
RPS24	12295	ES	5.1:5.2	4	6	-0.04	6.1E-08	excluded
ZDHHC7	37873	ES	4	3	5	0.07	6.3E-08	included
HIRIP3	36001	ES	4	3	5	0.04	6.4E-08	included
OAZ1	46599	ES	3.2:3.3:3.4:3.5	1	4	0.13	6.5E-08	included
ZC3H14	28722	ES	12:13:14.1:14.2	10	15	0.02	6.6E-08	included
SYTL2	18147	ES	15	14	16	-0.11	6.6E-08	excluded
ATP5A1	300062	ES	3.3:4.2	3.2	6.1	-0.09	6.8E-08	excluded
FCGR2B	8681	ES	9	8	10	0.13	7.1E-08	included
DENND4C	85968	ES	20	19	21	0.07	7.3E-08	included
C14orf166	27538	ES	3	2	4	0.00	7.3E-08	included
COX20	10475	ES	3	1	4	0.06	7.4E-08	included
ZMIZ2	79559	ES	9	8	10	0.06	7.4E-08	included
TARBP2	22072	ES	8	7.2	9.1	-0.02	7.8E-08	excluded
SFTA3	121951	ES	02:04.2	1.3	6	0.08	7.8E-08	included
TNPO3	81740	ES	2	1	3	-0.04	7.9E-08	excluded
RNF146	77450	ES	3	2	5.1	-0.10	7.9E-08	excluded
SULF2	59728	ES	20	19.2	21	-0.07	8.0E-08	excluded
GSTK1	82081	ES	4.3	4.1	5	0.01	8.0E-08	included
APEH	64895	ES	2	1	4	-0.11	8.1E-08	excluded
TXNL1	45630	ES	5	4	6	-0.01	8.3E-08	excluded
IP6K2	64760	ES	11.4:11.5	11.2	11.9	0.07	8.4E-08	included
NFU1	53853	ES	4	2	5	0.03	8.4E-08	included
FAM86B1	82710	ES	4	3.2	8.1	0.11	8.4E-08	included
PAM	72887	ES	25.2	23.2	26.1	0.02	8.7E-08	included
FAHD2A	54505	ES	2	1	3	0.07	8.8E-08	included
ZDHHC20	25452	ES	14	13.2	15	-0.07	8.8E-08	excluded
TTC9C	16416	ES	2	1.3	3.1	-0.03	8.9E-08	excluded
DNASE1L1	90577	ES	2.2:3.2:3.3	1	4	0.20	9.0E-08	included
SNX17	52994	ES	3:04	2.2	5	0.12	9.0E-08	included
MACF1	1883	ES	103	102	104	0.04	9.6E-08	included
CKMT2	72660	ES	2	1.1	3	0.18	1.0E-07	included
MTCH2	15788	ES	4	3	5	0.02	1.0E-07	included
STAT6	22523	ES	5	4	6	0.01	1.0E-07	included
MFF	57808	ES	10	7	11	0.04	1.1E-07	included
TPCN1	24631	ES	2	1	3	-0.09	1.1E-07	excluded
MRPL55	10137	ES	1.2:2.2:2.3:2.4:2.5:2.6:	1.1	2.9	0.09	1.1E-07	included
LRRC23	20004	ES	7.1:8:9	6	10	-0.04	1.1E-07	excluded
SPATS2L	56737	ES	10	9	11	-0.05	1.2E-07	excluded
PPFIBP1	20894	ES	9	8	10	-0.04	1.2E-07	excluded
EEF1B2	57139	ES	3	2.2	4.2	-0.01	1.2E-07	excluded
ATP9B	46234	ES	30	29	31.1	-0.07	1.2E-07	excluded
CCND3	76159	ES	4	3.1	5	-0.02	1.3E-07	excluded
ZNF211	52315	ES	3.1:3.2	2.2	4	0.14	1.3E-07	included
SLC15A4	93375	ES	3.2	2	3.4	0.06	1.3E-07	included
THTPA	26762	ES	1.3:1.4:1.5	1.1	2	0.10	1.3E-07	included
ADD1	68609	ES	15	14	16	-0.01	1.3E-07	excluded
DBI	55116	ES	1.2:1.3:3.1:3.2	1.1	5	0.00	1.4E-07	included
HSP90B1	192393	ES	3:04	2	5	-0.11	1.4E-07	excluded
CREM	11261	ES	9.2:10.1:11	4	15	0.11	1.4E-07	included
ECHDC2	3037	ES	5.1	2.1	6.2	-0.03	1.4E-07	excluded
C6orf203	77125	ES	1.2:2	1.1	3	-0.08	1.5E-07	excluded
TMEM107	39134	ES	2:3.1:3.2:3.4	1	3.7	0.04	1.5E-07	included
PAM	72901	ES	23.1:23.2	22	25.2	0.02	1.5E-07	included
EPN2	39700	ES	3	2.3	4	-0.03	1.6E-07	excluded
SAE1	50625	ES	7	6	8	0.00	1.7E-07	excluded
NADK2	71810	ES	10	9	11	-0.10	1.7E-07	excluded
TPD52L1	77416	ES	08:09.1	6	10	0.06	1.7E-07	included
MOV10	4225	ES	03:04.1	2.3	4.2	-0.04	1.7E-07	excluded
FCGR1B	4403	ES	2:03	1	4	-0.05	1.7E-07	excluded
ORAOV1	17380	ES	2	1	3	0.06	1.8E-07	included
ISY1	66700	ES	9	8	10.1	-0.07	1.8E-07	excluded

CORO1B	17217	ES	5.7	5.5	6	-0.01	1.8E-07	excluded
UPF3B	89980	ES	8	7	9	-0.09	1.8E-07	excluded
SFTA3	27278	ES	4.2	2	6	0.05	1.9E-07	included
ARFIP2	14140	ES	2.2	1	3	-0.03	1.9E-07	excluded
UCKL1	60181	ES	9	8	11.2	0.03	2.0E-07	included
CD151	13782	ES	2	1.2	3	0.05	2.0E-07	included
GMIP	48669	ES	14	13.2	15	0.06	2.1E-07	included
GATSL3	61705	ES	7	6	8	0.03	2.1E-07	included
ARHGEF10	82562	ES	10	9	11	0.11	2.1E-07	included
C4orf36	69844	ES	7.1:7.2	6	8	-0.13	2.2E-07	excluded
CLK4	74871	ES	5	4	6	0.06	2.3E-07	included
PLEKHA4	50820	ES	18	17	19	0.05	2.3E-07	included
CD44	15055	ES	6:7:8:9.1:9.2:10:11:12	5	14	-0.15	2.4E-07	excluded
PAM	72886	ES	25.1:25.2	23.2	26.1	0.03	2.4E-07	included
MAP2	57224	ES	16	15	17	0.05	2.4E-07	included
PPP2R5C	29321	ES	21	20.1	22	-0.07	2.4E-07	excluded
MRPL52	26641	ES	3	2	4.1	-0.01	2.5E-07	excluded
KBTBD3	18560	ES	2	1	3.2	0.06	2.5E-07	included
GTF3A	25533	ES	5	4.2	6	-0.01	2.6E-07	excluded
COQ3	77054	ES	5	4	6	0.02	2.6E-07	included
SREK1	72274	ES	4	3.2	5	-0.12	2.7E-07	excluded
VKORC1	36231	ES	4.2:5	2	6	0.05	2.7E-07	included
LRRC27	13498	ES	11	10	12	-0.07	2.7E-07	excluded
YBX3	20481	ES	6	5	7	-0.05	2.7E-07	excluded
RAB35	24720	ES	6	5	7	-0.02	2.8E-07	excluded
PAM	72897	ES	23.2	22	26.1	0.07	2.8E-07	included
LEF1	70294	ES	7	6.1	8	0.11	2.8E-07	included
FAM204A	13247	ES	2	1	3	0.05	2.8E-07	included
TIMMDC1	66311	ES	2:03:04	1.1	5	0.01	2.9E-07	included
ZCCHC11	573886	ES	16:17:18:19:20:21	3.2	22	-0.08	2.9E-07	excluded
LDHA	14636	ES	3	2.4	4	-0.08	3.0E-07	excluded
C4orf22	69683	ES	6	4	8	0.13	3.0E-07	included
IGFLR1	49259	ES	3:4.1:4.2	2.2	4.3	-0.05	3.1E-07	excluded
MRPL47	67700	ES	2.1:2.2	1	3	0.02	3.1E-07	included
RTN4	53592	ES	6.1:6.2	5	8	-0.09	3.2E-07	excluded
LPIN1	52712	ES	10	9	12	-0.12	3.2E-07	excluded
PIGT	59552	ES	2.2:3	2.1	4	0.00	3.3E-07	excluded
PLA2G4C	50685	ES	2.1:2.2	1	3	-0.11	3.3E-07	excluded
LRRC23	20005	ES	7.1:8	6	10	-0.10	3.3E-07	excluded
MEF2D	8275	ES	10	9	11	0.02	3.5E-07	included
ANKMY1	58257	ES	13:14	12	15	-0.15	3.5E-07	excluded
DNAJC10	56464	ES	11	10	13	-0.03	3.5E-07	excluded
BIN1	55199	ES	16	12	17	-0.03	3.6E-07	excluded
TNC	87357	ES	12:13:14:15:16:19	11	20	0.14	3.6E-07	included
PRKRIP1	81089	ES	5:06	4	7	0.06	3.6E-07	included
GGACT	93447	ES	2	1	3	-0.07	3.6E-07	excluded
TPM1	30990	ES	8	7	9	-0.05	3.6E-07	excluded
TXNL4A	46289	ES	4	3	7.2	-0.02	3.7E-07	excluded
EMC8	37897	ES	4	3	5	-0.02	3.7E-07	excluded
NONO	89416	ES	3	2	4	0.01	3.7E-07	included
ORC3	76971	ES	3	2	4	-0.09	3.7E-07	excluded
FAM86B1	82691	ES	7.1:7.3	4	8.1	-0.12	3.8E-07	excluded
CIRBP	46431	ES	9.6	9.3	9.8	-0.01	3.8E-07	excluded
SRP68	43552	ES	1.2:2.1:2.2	1.1	3	0.01	4.0E-07	included
DMKN	49180	ES	8	7	12	-0.03	4.1E-07	excluded
ZC3H14	28716	ES	13:14.1:14.2	12	15	0.03	4.1E-07	included
CFI	70340	ES	7:08	6	9	-0.07	4.2E-07	excluded
RNF181	534110	ES	02:03.2	1	4.1	-0.05	4.2E-07	excluded
ITGA6	55968	ES	27	26	28	0.06	4.2E-07	included
DGUOK	54014	ES	4	1	7	0.04	4.2E-07	included
MRPL55	10133	ES	1.2:2.2:2.5:2.6:2.7:2.8	1.1	2.9	0.06	4.2E-07	included
COX14	21650	ES	3	1.1	4	0.01	4.2E-07	included

ERRFI1	536	ES	3.1:3.2	2	3.4	-0.10	4.3E-07	excluded
ANXA3	69643	ES	3	2	4	-0.01	4.3E-07	excluded
DMKN	49144	ES	21	20.1	22	0.04	4.4E-07	included
BEX4	89722	ES	2	1	3	0.01	4.4E-07	included
GBAS	79770	ES	4	3	5	-0.01	4.4E-07	excluded
KLC1	29482	ES	15:16	13.2	18	-0.05	4.5E-07	excluded
RCOR3	9709	ES	14.1	13	15	0.06	4.5E-07	included
YAF2	21146	ES	5.1:5.2:6:7	2	9.1	-0.05	4.6E-07	excluded
TRMT1L	9212	ES	11	10	12	-0.03	4.7E-07	excluded
DEDD2	50136	ES	3	2	4.1	0.07	4.7E-07	included
DPH5	3897	ES	7	6.2	8.1	0.02	4.7E-07	included
DDX49	48528	ES	4	2.2	5	0.01	4.7E-07	included
ZNF682	48709	ES	3:04	2	5.2	0.16	4.8E-07	included
PTPN3	87168	ES	5	4	6.2	0.05	4.8E-07	included
MSI2	94619	ES	19	18.2	20	0.06	4.8E-07	included
DMKN	101873	ES	8	6.4	12	-0.01	4.8E-07	excluded
TCTN1	93321	ES	14:16.2	12	17.2	0.10	4.8E-07	included
ANK3	11853	ES	26	25	27	-0.13	4.8E-07	excluded
ZDHHC4	78750	ES	1.2:1.3:2.1	1.1	2.2	0.03	4.8E-07	included
R3HDM2	22576	ES	12:13	11	15	0.03	4.9E-07	included
MTERFD2	58318	ES	2	1	3.1	0.05	4.9E-07	included
EWSR1	61584	ES	9.1:9.2:11.2	8	12	0.00	5.1E-07	excluded
MED24	40831	ES	7	6	8	-0.05	5.1E-07	excluded
ACTB	78668	ES	4	3	5	0.00	5.3E-07	excluded
HEXA	31548	ES	5:06	4	7	0.01	5.3E-07	included
PSMC5	43007	ES	5	4	6	0.00	5.3E-07	included
MPRIP	39457	ES	24	23	25	-0.08	5.3E-07	excluded
MACF1	1881	ES	107	106	108	0.05	5.3E-07	included
PTPRK	77503	ES	2	1	4	-0.06	5.4E-07	excluded
C11orf49	15626	ES	4	3	7	-0.02	5.4E-07	excluded
FANK1	13451	ES	5	1	6.1	0.08	5.6E-07	included
C5orf45	74964	ES	4	2.1	5.1	0.03	5.6E-07	included
MRPL55	10153	ES	1.2:2.2:2.5:2.6	1.1	2.9	0.09	5.7E-07	included
TYMS	44457	ES	4	3	5	0.03	5.7E-07	included
NEK1	71148	ES	17	16	18	-0.12	5.7E-07	excluded
PAM	72898	ES	23.1:23.2:25.1	22	25.2	0.04	5.8E-07	included
DGCR6	61030	ES	4	3.1	5.1	-0.02	5.8E-07	excluded
ASS1	87902	ES	2	1	4	-0.01	5.8E-07	excluded
SEPP1	71916	ES	3	2	4	0.00	5.9E-07	excluded
EBPL	25912	ES	4.1:4.2	1	6	-0.07	6.0E-07	excluded
VKORC1	36230	ES	4.1:4.2:5	2	6	0.02	6.1E-07	included
USP21	8566	ES	2	1	3	0.07	6.2E-07	included
EPS15L1	48159	ES	22	21	23.1	-0.05	6.2E-07	excluded
RASA4	97680	ES	6	5	7	0.04	6.2E-07	included
ETV1	78838	ES	5	4	6	0.13	6.3E-07	included
HNRNPA1	22146	ES	8	7.2	9.1	-0.01	6.3E-07	excluded
PLA2G6	62205	ES	14	13	15	0.09	6.3E-07	included
C16orf13	32917	ES	3	2	5	-0.02	6.3E-07	excluded
TMEM168	81457	ES	3	1	4	-0.03	6.3E-07	excluded
RALGAPA1	27239	ES	42	41	43	-0.10	6.3E-07	excluded
REPIN1	82235	ES	4.1:4.2:5.2	3.2	5.3	-0.10	6.4E-07	excluded
CD44	15107	ES	6:7:8:9.2	5	10	-0.06	6.4E-07	excluded
CAST	196583	ES	7.1:8.2	5.2	9	-0.12	6.5E-07	excluded
SDHAF2	16236	ES	3	1	6	0.06	6.6E-07	included
PATZ1	61847	ES	5.1:5.2	4	6	0.06	6.8E-07	included
ERMARD	78487	ES	11	10	12	0.02	6.9E-07	included
ALG13	89907	ES	25	24	26	0.06	7.1E-07	included
TMC6	43765	ES	9	8	10	0.02	7.2E-07	included
PPIP5K2	72914	ES	29	28	30	-0.11	7.2E-07	excluded
MAPK13	75953	ES	8:09	7	10.1	0.01	7.2E-07	included
ING4	19911	ES	5.2:6.1	5.1	6.2	-0.05	7.3E-07	excluded
RFX5	7609	ES	7	6.2	8	-0.08	7.4E-07	excluded

BBIP1	13094	ES	4	3	5.1	-0.08	7.4E-07	excluded
PSMA3	27691	ES	3.1:3.2	2	4	0.00	7.4E-07	included
FDPS	8068	ES	1.2:2	1.1	3.1	0.03	7.5E-07	included
ATG4B	58404	ES	5	3	6	-0.04	7.6E-07	excluded
PRPF3	7468	ES	4	3	5	-0.08	7.6E-07	excluded
SCAMP3	8052	ES	2	1	3	-0.02	7.7E-07	excluded
PPP2R4	87853	ES	6:7:8:9:10	3.1	15.1	-0.15	7.7E-07	excluded
DGUOK	54007	ES	2:4:5:6	1	7	-0.05	7.8E-07	excluded
SNRPC	75790	ES	2.2	1	3	0.00	7.9E-07	excluded
NVL	9953	ES	2	1	3.1	0.05	7.9E-07	included
VPS29	24433	ES	4	3.2	5	0.08	7.9E-07	included
EWSR1	61583	ES	9.1:9.2:11.1:11.2	8	12	0.00	8.5E-07	excluded
API5	15453	ES	2	1	3	-0.02	8.5E-07	excluded
CCDC112	73026	ES	10	9	11	-0.06	8.6E-07	excluded
VPS51	16759	ES	2:03	1	4	0.11	8.7E-07	included
ACIN1	26706	ES	9.3	9.1	10	-0.12	8.8E-07	excluded
TNC	87360	ES	19	11	20	0.14	9.0E-07	included
YAF2	21157	ES	5.1:5.2:6	2	9.1	-0.07	9.0E-07	excluded
OFD1	88522	ES	11	10	13	0.05	9.0E-07	included
CAPN3	30161	ES	11	10	12	-0.14	9.1E-07	excluded
PAM	72892	ES	25.1:25.2	22	26.1	0.02	9.1E-07	included
MUC1	7960	ES	6.1:6.2	5	7	0.00	9.1E-07	excluded
COG4	37403	ES	4	3	5.1	-0.04	9.2E-07	excluded
ACTN1	28118	ES	20	19	21	0.04	9.3E-07	included
FANK1	13444	ES	12:13	11	14	-0.04	9.3E-07	excluded
MRPL48	17726	ES	6	5	8	-0.02	9.3E-07	excluded
NDRG2	26511	ES	4.2:4.5	4.1	5.2	-0.05	9.5E-07	excluded
EPHB6	99887	ES	3	2	4.2	-0.06	9.6E-07	excluded
PPA2	70215	ES	3	2	4	-0.01	9.7E-07	excluded
PHYKPL	74855	ES	14	13	15	-0.02	9.8E-07	excluded
APRT	38028	ES	3	2.1	4	0.00	9.9E-07	included
IL17RC	63260	ES	12	11	13	0.01	9.9E-07	included
ZBTB1	27894	ES	3	2.2	4.1	-0.10	9.9E-07	excluded
VPS29	24446	ES	3.1	1	5	0.07	9.9E-07	included
YAP1	18442	ES	7	6.1	8	0.04	1.0E-06	included
RBP7	592	ES	2	1	3	0.07	1.0E-06	included
THBS3	8027	ES	14	13	15	0.01	1.0E-06	included
UBE2A	89957	ES	4.2	3	5	-0.02	1.0E-06	excluded
C2orf74	95530	ES	3	1	4	0.02	1.0E-06	included
CADM1	18849	ES	11	10	12	0.05	1.1E-06	included
ATL2	389880	ES	15:17.1	14	17.2	0.10	1.1E-06	included
ZNF584	52455	ES	2.1:2.2:2.3	1	3	-0.14	1.1E-06	excluded
MBD1	45522	ES	12	11	13.1	0.01	1.1E-06	included
RBM39	59241	ES	11	10	12.1	-0.01	1.1E-06	excluded
CBWD2	55056	ES	2	1	3	-0.05	1.1E-06	excluded
TNC	87348	ES	12:13:14:15:16	11	19	-0.14	1.1E-06	excluded
NBPF12	7355	ES	24.3:25	24.2	26	0.06	1.1E-06	included
MCL1	7489	ES	2	1	3	0.01	1.1E-06	included
CPNE1	59198	ES	1.2:2.1:2.2:3	1.1	5	-0.08	1.1E-06	excluded
TCF25	38160	ES	2	1	3	-0.02	1.1E-06	excluded
ZNF584	52456	ES	2.2:2.3	1	3	-0.12	1.1E-06	excluded
ITGB5	66543	ES	5	4	6	0.00	1.1E-06	excluded
FEZ2	53198	ES	8	7	9	0.02	1.1E-06	included
SLC25A45	16828	ES	8	7	10	-0.04	1.1E-06	excluded
NASP	2706	ES	9	8	10	0.07	1.2E-06	included
CD47	66013	ES	9:10	8	11	0.07	1.2E-06	included
AHDC1	1331	ES	03:04.1	2	4.2	0.12	1.2E-06	included
SKA2	42754	ES	02:04.1	1.1	5	0.07	1.2E-06	included
LCN10	88199	ES	03:04.2	2.2	5	0.13	1.2E-06	included
PDE4DIP	4432	ES	5.2:5.3	4	6.1	-0.12	1.2E-06	excluded
SERGEF	14559	ES	8	7	9	-0.02	1.2E-06	excluded
DMTN	82930	ES	6	5.2	7	0.05	1.2E-06	included

SNRNP200	54521	ES	45	44	46.1	-0.04	1.2E-06	excluded
CDC42	1006	ES	2	1	3	-0.02	1.2E-06	excluded
RNMT	44755	ES	2.2	1	3.2	0.08	1.2E-06	included
WIZ	48090	ES	9	8	10	-0.06	1.2E-06	excluded
CBWD1	85693	ES	2	1	3	-0.04	1.2E-06	excluded
KMT2E	81271	ES	26	25	27	0.01	1.2E-06	included
MEAF6	1800	ES	6:8.1:9.1	5	9.2	0.11	1.3E-06	included
CERS5	21659	ES	17.1:17.2	16	18	-0.03	1.3E-06	excluded
MLST8	33221	ES	9	8.2	10.1	0.00	1.3E-06	excluded
SSR4	90499	ES	4	3	5	0.00	1.3E-06	excluded
TLE2	114945	ES	11	10.5	12	0.02	1.3E-06	included
KANSL3	54543	ES	23.1:23.2:23.3	22	24	-0.03	1.3E-06	excluded
NCOR2	25144	ES	46.1:46.2	45	47	-0.03	1.3E-06	excluded
USF2	49098	ES	3	2.3	4	-0.02	1.3E-06	excluded
CD44	15105	ES	7:8:9.1:9.2	5	10	-0.10	1.3E-06	excluded
TMUB2	41807	ES	3	2.5	4.3	-0.06	1.4E-06	excluded
PIGQ	32905	ES	12	11	13	0.04	1.4E-06	included
PRDX5	16640	ES	3	1	4	0.03	1.4E-06	included
SPATS2	21586	ES	2	1	5	-0.07	1.4E-06	excluded
UPP1	101244	ES	6.1	4	9	-0.02	1.4E-06	excluded
CD44	15133	ES	10:11:12.1:13:14	5	15	-0.12	1.4E-06	excluded
ABI2	57069	ES	4	1	5.1	-0.01	1.4E-06	excluded
TRAF3IP2	77268	ES	2	1	3	-0.08	1.4E-06	excluded
HAUS4	26677	ES	3	2	4	0.02	1.4E-06	included
VEGFA	76338	ES	6:7.1:7.2:7.3:8.1:8.2	5	9.1	0.12	1.5E-06	included
CD59	14917	ES	2:03	1	5.4	-0.01	1.5E-06	excluded
BRD8	73507	ES	22	21	23	-0.05	1.5E-06	excluded
ZNF638	53930	ES	23	22	24	0.01	1.5E-06	included
ERBB2IP	72264	ES	24.1:24.2:24.3	21	25	0.03	1.5E-06	included
MRPL55	10117	ES	2.2:2.3:2.4:2.5:2.6	1.2	2.9	0.08	1.6E-06	included
AKAP8L	48076	ES	10	9	11	-0.07	1.6E-06	excluded
HNRNPUL1	50031	ES	16.1:16.2:16.3	15	17	0.03	1.6E-06	included
SEC31A	69734	ES	16:17	15	18	0.05	1.6E-06	included
PAM	72895	ES	25.2	22	26.1	0.03	1.6E-06	included
RSRC2	24970	ES	04:05.1	3	5.2	-0.04	1.7E-06	excluded
AK2	1654	ES	2:03	1	4	-0.02	1.7E-06	excluded
RNF14	73846	ES	6	4	7	0.05	1.7E-06	included
MARCH8	11362	ES	7	6	8	-0.07	1.7E-06	excluded
TRIM65	43525	ES	5	4	6	0.07	1.7E-06	included
STK16	57666	ES	4	3.2	5	0.02	1.7E-06	included
FAM49B	85146	ES	9	6	10	0.04	1.7E-06	included
ACYP1	28478	ES	4	3.1	6	-0.03	1.8E-06	excluded
POLL	12904	ES	1.5	1.1	3	0.08	1.8E-06	included
TECR	564143	ES	4:5.1:5.2	1	5.3	0.09	1.8E-06	included
C17orf49	38825	ES	3	2.2	4	0.01	1.8E-06	included
RNF14	73845	ES	5:06	4	7	0.01	1.8E-06	included
FLAD1	91164	ES	2.2:4.1:4.3	1.3	6.1	0.11	1.9E-06	included
SIPA1L2	10315	ES	20	19	21	0.02	1.9E-06	included
ARHGAP23	40578	ES	23	22	25	0.04	1.9E-06	included
LYRM5	20815	ES	2.1:2.2	1.1	3.1	-0.02	2.0E-06	excluded
ZNF197	64377	ES	3	2.3	4	-0.08	2.0E-06	excluded
MND1	70870	ES	5:06	4	7	-0.09	2.0E-06	excluded
MAN2C1	31867	ES	7.1:7.2	6	8	0.01	2.0E-06	included
PLS3	89927	ES	5	3	6	-0.10	2.0E-06	excluded
MRPL55	10135	ES	1.2:2.5:2.6:2.7:2.8	1.1	2.9	0.02	2.0E-06	included
SYNRG	40526	ES	22	21	23	0.08	2.0E-06	included
SUN1	78549	ES	5.2	3.2	6	-0.02	2.0E-06	excluded
TMC6	43760	ES	14.3	14.1	15	-0.03	2.1E-06	excluded
NGLY1	63754	ES	13	12	14	0.02	2.1E-06	included
SH3TC1	68759	ES	8	7	9	0.06	2.1E-06	included
ELP2	45219	ES	6	5	7	0.08	2.1E-06	included
TCEAL8	89723	ES	2	1	3	0.03	2.1E-06	included

SAMD4B	49805	ES	4	1	5	-0.04	2.2E-06	excluded
PLD3	49891	ES	4	1.2	5.2	0.03	2.2E-06	included
NSFL1C	58507	ES	3	2	4	-0.01	2.2E-06	excluded
KIF12	87305	ES	15	14	16	0.07	2.2E-06	included
C2CD5	20733	ES	26:27.1:27.2	25	28	0.07	2.2E-06	included
MRPS18C	69795	ES	3	2	4	-0.02	2.3E-06	excluded
CD44	15104	ES	6:7:8:9.1:9.2	5	10	-0.07	2.3E-06	excluded
AMZ2	43131	ES	2	1.5	3.2	-0.01	2.3E-06	excluded
WFDC2	59581	ES	3	2	4.3	-0.02	2.3E-06	excluded
CTNNB1	64254	ES	5	4	6	0.00	2.3E-06	excluded
SH3D21	1767	ES	12:13	11	14	0.02	2.3E-06	included
ZRANB2	3436	ES	10	9	11	-0.05	2.3E-06	excluded
APLP2	19476	ES	16	15	17	-0.02	2.3E-06	excluded
C11orf57	18729	ES	1.3	1.1	2.1	0.02	2.3E-06	included
PDCD6IP	63894	ES	2	1	3	-0.07	2.4E-06	excluded
EML2	50501	ES	9:10	8	11	0.01	2.4E-06	included
ATP6V1F	81725	ES	2	1	3	0.00	2.4E-06	included
KIF21A	93029	ES	29	28	33.2	0.07	2.4E-06	included
PPP6R3	17320	ES	16	15	17.1	-0.04	2.4E-06	excluded
RAB34	94477	ES	03:04.1	2.3	4.2	0.06	2.4E-06	included
PFDN5	93149	ES	2	1	4.2	0.07	2.5E-06	included
SNX1	139174	ES	6:7:8:9:10.2:11:12:13.	3	16.1	0.09	2.5E-06	included
GIT1	40046	ES	8	7	9	0.04	2.5E-06	included
GLRX3	13476	ES	11.1	10	12.1	-0.03	2.5E-06	excluded
CNN2	46362	ES	5.1:5.2	4.2	6	-0.14	2.6E-06	excluded
RNF135	40137	ES	3	2	5	0.04	2.6E-06	included
DECR1	84410	ES	4.1:4.2	1	5.2	-0.02	2.6E-06	excluded
TP53	39039	ES	10.1:10.2	9	11	-0.01	2.6E-06	excluded
AVL9	79198	ES	14	13	15	0.03	2.6E-06	included
MAG	49104	ES	11	10	12	0.11	2.7E-06	included
UPP1	97464	ES	5:6.1:6.2	4	9	-0.04	2.7E-06	excluded
POLR2H	67944	ES	05:06.1	4	6.2	0.05	2.8E-06	included
ZFYVE21	29516	ES	02:03.2	1	4	0.00	2.8E-06	included
MYO9B	48232	ES	37	36	38.1	-0.09	2.8E-06	excluded
CAST	196584	ES	7.1	5.2	9	0.03	2.9E-06	included
C5orf45	74963	ES	2.3:4	2.1	5.1	0.15	2.9E-06	included
BPNT1	9865	ES	5	4	6	0.02	2.9E-06	included
CD46	9659	ES	9	6	10	-0.04	2.9E-06	excluded
REPIN1	82244	ES	3.2:4.1:4.2	2.1	5.2	-0.06	2.9E-06	excluded
PKP4	95717	ES	26	24	27	0.01	3.0E-06	included
D2HGDH	58414	ES	10:11.1	9	11.2	0.11	3.0E-06	included
RBM6	64952	ES	3.2:4:5	2	7	0.08	3.0E-06	included
FAM92A1	84523	ES	11	10	12.1	-0.03	3.0E-06	excluded
MROH7-TTC4	3150	ES	21.2:22	21.1	23	0.03	3.0E-06	included
NBPF11	7333	ES	19.1:19.2	18	20.2	-0.03	3.0E-06	excluded
FAM195A	32927	ES	3	2	4	-0.07	3.0E-06	excluded
SNRNP70	50888	ES	8.1	7	9	-0.04	3.1E-06	excluded
YIPF1	3079	ES	3	2	4	-0.06	3.1E-06	excluded
COPS7A	19947	ES	6	2.4	7	0.01	3.2E-06	included
U2AF1L4	49268	ES	08:09.1	7	9.2	-0.03	3.2E-06	excluded
DEDD	8563	ES	3.1:3.2	1	4	-0.03	3.3E-06	excluded
ANAPC11	44205	ES	8	7.2	9	0.00	3.3E-06	included
PPHLN1	21223	ES	9:10	7	11	0.00	3.3E-06	included
MRPL48	17730	ES	5	4	8	0.02	3.3E-06	included
MALT1	45679	ES	7	6	8	-0.05	3.3E-06	excluded
RBM39	59250	ES	4	3	6	-0.07	3.3E-06	excluded
CAPN10	58271	ES	10.1:10.2	9	11	0.02	3.4E-06	included
DONSON	60445	ES	5	4.2	6	0.06	3.4E-06	included
ANAPC11	44216	ES	3.3:3.4:6	3.2	7.2	0.05	3.4E-06	included
RASA1	72717	ES	14	13	15	-0.04	3.4E-06	excluded
MYL6	22383	ES	2.1:2.2	1.4	3.1	0.00	3.5E-06	included
RHNO1	19727	ES	2.1	1	4	-0.10	3.5E-06	excluded

EDEM2	59067	ES	12	11	13	-0.04	3.5E-06	excluded
SLC38A2	21331	ES	4	3	5	-0.02	3.5E-06	excluded
CCNDBP1	30224	ES	2	1	4	-0.09	3.6E-06	excluded
KIFC3	100255	ES	6:07	5	13	0.07	3.6E-06	included
MRPL43	12858	ES	2	1	3.1	0.00	3.6E-06	included
BCLAF1	77907	ES	11	10	12	-0.07	3.6E-06	excluded
E2F6	52689	ES	4	3	5.1	-0.07	3.7E-06	excluded
PEX2	84243	ES	3	2.2	4.2	-0.01	3.7E-06	excluded
UBE2V1	59759	ES	4	3	5	-0.01	3.8E-06	excluded
WDR54	54055	ES	2	1	3	-0.03	3.8E-06	excluded
STXBP6	27042	ES	3	1.1	6	-0.10	3.9E-06	excluded
ZNF664	25130	ES	2.1:2.2:4	1.4	5	-0.09	4.0E-06	excluded
RSRC2	24972	ES	4	3	5.2	-0.03	4.0E-06	excluded
SDCBP	83938	ES	2	1	3.2	0.00	4.0E-06	excluded
CCDC25	83179	ES	7	6	8.1	0.02	4.1E-06	included
TNFRSF12A	33348	ES	3.2	2.1	4	0.01	4.1E-06	included
TNIK	67629	ES	22	21.1	23	-0.11	4.1E-06	excluded
CALD1	81862	ES	8.3:9	8.2	10	-0.06	4.2E-06	excluded
RQCD1	57499	ES	2	1	3	-0.08	4.2E-06	excluded
FAM219B	31802	ES	1.3:1.4	1.1	2	0.03	4.2E-06	included
SCMH1	2047	ES	17	16	18	-0.04	4.2E-06	excluded
CACFD1	88067	ES	5	4	6	0.02	4.2E-06	included
GOPC	77347	ES	3	2.1	5.1	0.03	4.2E-06	included
HDAC3	73809	ES	3	2	4	0.01	4.2E-06	included
UBE2E1	63718	ES	4	2	5	0.00	4.4E-06	excluded
NDUFA3	51782	ES	4.3	4.1	5.1	0.02	4.5E-06	included
CD44	15115	ES	7:8:9.2:10:11	5	12.1	-0.10	4.5E-06	excluded
LRP8	3058	ES	20	19	21	-0.08	4.5E-06	excluded
ZNF664	25129	ES	2.2:3:4	1.4	5	-0.08	4.5E-06	excluded
RGS6	28219	ES	20.1:20.2	18	26.1	0.06	4.6E-06	included
NSMCE1	35679	ES	4.1:4.2:4.3	3	5	-0.01	4.6E-06	excluded
ZNF789	80649	ES	7	6	8	-0.08	4.6E-06	excluded
RPS7	52573	ES	1.2:2.1	1.1	2.2	-0.09	4.6E-06	excluded
PARP8	71982	ES	11	10	12.1	-0.02	4.7E-06	excluded
BCL2L12	51036	ES	3.2	2	4	0.11	4.7E-06	included
EEF1D	85456	ES	04:08.1	1	8.2	-0.02	4.7E-06	excluded
NME1-NME2	42513	ES	2	1	3	0.01	4.7E-06	included
TBC1D9B	74991	ES	20	19	21	-0.01	4.7E-06	excluded
CD59	14919	ES	2	1	5.4	0.00	4.8E-06	excluded
MIS18BP1	27410	ES	8	7	9	-0.11	4.8E-06	excluded
DOK1	54115	ES	5	4	6	0.03	4.8E-06	included
KLC1	29480	ES	14.1:15:16	13.2	18	0.00	4.8E-06	excluded
IL15	70676	ES	3	2	4.2	-0.05	5.0E-06	excluded
CCT7	53962	ES	3:4:5.1:5.2:6:7	1	8	0.00	5.1E-06	included
DOCK7	3256	ES	24	23	25	0.12	5.1E-06	included
IFI44	91013	ES	7.1	6	8	0.05	5.1E-06	included
DGCR6L	61146	ES	3	2.2	4	0.01	5.2E-06	included
FAM21C	11374	ES	26	25	27	0.01	5.2E-06	included
CALD1	81863	ES	9	8.2	10	-0.01	5.2E-06	excluded
NUP98	13997	ES	29	28	30	-0.01	5.2E-06	excluded
NCOA1	52829	ES	24	23	25.1	0.05	5.2E-06	included
SHF	30417	ES	7	6	8.1	-0.02	5.2E-06	excluded
IL32	33440	ES	1.3:1.4:1.5	1.1	1.9	0.13	5.3E-06	included
ABCD4	28378	ES	8.2	7	9	0.03	5.3E-06	included
ELK1	88937	ES	2	1	3	0.06	5.3E-06	included
TRIQK	84493	ES	5	4	6.1	0.05	5.3E-06	included
ZNF528	51457	ES	9	7.1	10	0.11	5.3E-06	included
NONO	89421	ES	2	1	4	0.01	5.3E-06	included
CD59	14918	ES	3	1	5.4	0.03	5.4E-06	included
CIZ1	87713	ES	15	14	16	0.00	5.4E-06	included
SERGEF	102751	ES	1.2:2	1.1	3	-0.07	5.5E-06	excluded
FAM86B1	82684	ES	7.3	7.1	8.1	0.10	5.5E-06	included

SCUBE2	14293	ES	20	19	21	0.02	5.5E-06	included
TPO	52539	ES	11	10	12	-0.03	5.5E-06	excluded
TTLL5	28523	ES	21	20	22	0.07	5.5E-06	included
RBBP8	94795	ES	18	17.2	19.2	0.01	5.6E-06	included
DDX50	11971	ES	2.1:2.2	1	3	-0.05	5.7E-06	excluded
DGUOK	54009	ES	3:04:05	1	7	-0.07	5.7E-06	excluded
MORF4L2	89777	ES	4:5.1:5.2	3.2	5.3	0.03	5.8E-06	included
CTNNA1	73567	ES	4	2	5	0.00	5.8E-06	included
TPM2	98132	ES	7	6	8	-0.07	5.8E-06	excluded
PTRH2	42793	ES	2.2	1	2.4	-0.04	5.8E-06	excluded
CARD8	50713	ES	7.1:7.2	5	8	0.14	5.9E-06	included
DLG3	89382	ES	18	16	19	-0.02	5.9E-06	excluded
YWHAE	38301	ES	2	1	3	-0.01	5.9E-06	excluded
BAG1	86098	ES	3	2	4	0.01	5.9E-06	included
NDUFA7	47219	ES	4	2	5.1	0.00	5.9E-06	included
SPATC1L	60904	ES	2	1	3	-0.04	6.0E-06	excluded
NOD1	79117	ES	10	9	11	0.04	6.0E-06	included
PIGF	53467	ES	6	5	7	0.04	6.0E-06	included
TMEM141	88206	ES	2	1	3	0.02	6.0E-06	included
MAD2L2	665	ES	2:03	1	4	-0.14	6.0E-06	excluded
SUCO	9011	ES	13	12	14	0.10	6.0E-06	included
HSBP1L1	46269	ES	2.2:3.1	2.1	3.2	-0.05	6.1E-06	excluded
PLEKHB1	17700	ES	7	6	8	-0.03	6.1E-06	excluded
POLL	12903	ES	1.2:1.5	1.1	3	0.14	6.2E-06	included
ZNF384	19923	ES	8	7	9	-0.04	6.3E-06	excluded
PLCB4	58679	ES	20	19	21	-0.08	6.3E-06	excluded
FAM122B	90158	ES	3	2	4	-0.07	6.4E-06	excluded
MRPL55	10157	ES	1.2:2.2:2.3:2.4:2.5	1.1	2.9	0.05	6.4E-06	included
ZNF664	25131	ES	2.2:4	1.4	5	-0.09	6.4E-06	excluded
BNIPL	7552	ES	8	7	9	-0.06	6.5E-06	excluded
RAB11FIP3	32895	ES	7	6	8	0.06	6.6E-06	included
ZNF227	50300	ES	3	2.2	4.1	0.07	6.8E-06	included
FAM96A	31084	ES	4	3	5.1	0.02	6.8E-06	included
SAMD4A	27599	ES	4	3	5	0.06	6.9E-06	included
GNB2L1	75060	ES	4.1:4.2	3	5	0.00	6.9E-06	excluded
IL4R	35690	ES	2	1	3.2	-0.05	7.0E-06	excluded
NUMA1	17518	ES	4	3	5	-0.05	7.1E-06	excluded
ZNF76	75902	ES	12.1	11	12.3	-0.03	7.1E-06	excluded
TNRC18	78664	ES	15	14	16	0.03	7.3E-06	included
ZNF496	10518	ES	6	5	7	-0.02	7.3E-06	excluded
CADPS2	81608	ES	25	24.1	27	-0.05	7.3E-06	excluded
RRAGB	89285	ES	4	3	5	0.04	7.3E-06	included
COL1A1	402821	ES	29:30:31:32:33:34:35:<br>36:37:38:41:42:43:44:	28	50	-0.03	7.4E-06	excluded
GALK2	30524	ES	15	14	16	-0.06	7.4E-06	excluded
SMG7	9178	ES	20	19	21	-0.05	7.4E-06	excluded
RSPO4	58472	ES	4	3	5	-0.13	7.4E-06	excluded
HNRNPDL	69706	ES	6	5	7	0.00	7.5E-06	excluded
BTBD3	58696	ES	3	2.3	4	0.02	7.5E-06	included
RGN	88903	ES	2	1	3	-0.04	7.7E-06	excluded
MVD	38010	ES	3:04	1	5	-0.07	7.8E-06	excluded
PTK2B	83156	ES	28	27	29	-0.05	7.9E-06	excluded
NRP1	11201	ES	14.1:14.2	12	15.1	-0.01	7.9E-06	excluded
CCDC53	24024	ES	4	2	6	-0.03	7.9E-06	excluded
AP1M2	47557	ES	2	1	3	-0.01	8.0E-06	excluded
EBPL	25911	ES	2:4.1:4.2	1	6	-0.02	8.1E-06	excluded
COPS5	84071	ES	2.3	2.1	5	0.07	8.1E-06	included
BCAS4	59787	ES	4	3	5	-0.04	8.2E-06	excluded
HSD11B1L	46874	ES	5.2:5.3:7	4.2	8	0.09	8.3E-06	included
TXNL4A	46285	ES	04:07.2	3	9	-0.03	8.3E-06	excluded
MRPL55	10149	ES	2.2:2.3:2.4:2.5:2.6	1.1	2.9	0.08	8.3E-06	included
ABHD11	80028	ES	5	3	6	0.09	8.4E-06	included

MORF4L2	89772	ES	5.1:5.2:5.3	3.2	6.2	0.03	8.6E-06	included
TMEM116	24566	ES	3	2	4	-0.08	8.7E-06	excluded
SMARCE1	40872	ES	5	4.1	6	0.00	8.9E-06	excluded
ZCWPW1	80945	ES	16:17	15	18	0.07	8.9E-06	included
GSN	87434	ES	10:15.1:15.2	9	16	-0.07	9.0E-06	excluded
NBPF1	835	ES	25	24	26	-0.03	9.0E-06	excluded
SMUG1	22138	ES	2.2	1.1	4.1	-0.06	9.0E-06	excluded
EFCAB2	10481	ES	2:03	1.3	4	-0.08	9.1E-06	excluded
RAP1GAP	993	ES	2	1	4	-0.10	9.3E-06	excluded
PPP1R7	58337	ES	1.2:1.3:2	1.1	3	0.09	9.3E-06	included
NBPF11	7346	ES	3	2	4	0.02	9.3E-06	included
LGALS9	39856	ES	5:06	4	7	0.05	9.4E-06	included
MFF	57813	ES	3:04:05	1	6	0.09	9.4E-06	included
UTRN	78027	ES	67	66	68	-0.05	9.6E-06	excluded
B9D2	50061	ES	3	2	4	0.03	9.6E-06	included
IDH3A	32015	ES	5	4.2	6.1	0.02	9.6E-06	included
ANKHD1	73655	ES	31	30	32	0.01	9.7E-06	included
COA1	79347	ES	3.1	1	4.2	0.09	9.7E-06	included
MOK	29397	ES	04:06.1	2	7	0.12	9.8E-06	included
DCTN2	22643	ES	7:08	2	10	-0.01	9.9E-06	excluded
CMTM7	63816	ES	3	2	4.2	0.02	9.9E-06	included
PPP1R13B	29521	ES	6	5	7	-0.04	1.0E-05	excluded
LGMN	29011	ES	2	1	3	0.08	1.0E-05	included
ODF2L	3675	ES	15	14	17	0.08	1.0E-05	included
ZNF195	13975	ES	9:11:12	5.1	13	-0.03	1.0E-05	excluded
CAV2	81527	ES	2.1	1	3	0.00	1.0E-05	included
C16orf62	34324	ES	27	26	28	-0.01	1.1E-05	excluded
COL1A1	402696	ES	29:30:31:32:33:43:44:45:46:47:48:49	28	50	-0.07	1.1E-05	excluded
SLTM	30918	ES	5	4.2	6	0.01	1.1E-05	included
P4HA2	73263	ES	2.2	1	3	0.09	1.1E-05	included
FAM109A	24510	ES	3	2	5	-0.05	1.1E-05	excluded
PDE4DIP	4431	ES	5.1:5.2:5.3	4	6.1	-0.10	1.1E-05	excluded
EEF1D	85450	ES	8.3:10.1:11:12.2:13.1	8.2	13.2	0.00	1.1E-05	excluded
ZNF814	52367	ES	2	1	3.1	0.04	1.1E-05	included
DUOXA1	30390	ES	7	6	8	-0.04	1.1E-05	excluded
VEGFA	76328	ES	7.1:7.2:8.1:8.2	6	9.1	-0.05	1.2E-05	excluded
CCDC84	19053	ES	4	3	5	0.07	1.2E-05	included
CPPED1	34061	ES	3	2	4.1	0.02	1.2E-05	included
C14orf159	28844	ES	16	15	17	-0.01	1.2E-05	excluded
FUBP3	87903	ES	17	16	18	-0.02	1.2E-05	excluded
FUZ	51088	ES	2.1:2.2:2.3	1.2	3	0.02	1.2E-05	included
RIBC1	89221	ES	5.1	4	6	0.12	1.2E-05	included
RBMS2	93175	ES	15	14	16	0.02	1.2E-05	included
PPP2R4	87850	ES	4:5:6:7:8:9:10	3.1	15.1	-0.06	1.2E-05	excluded
DMD	88772	ES	82	81	83	-0.08	1.2E-05	excluded
TG	319452	ES	29	27	31	-0.08	1.2E-05	excluded
VPS53	38222	ES	8	7	9	-0.03	1.2E-05	excluded
C11orf74	15441	ES	5	4	7	-0.11	1.2E-05	excluded
HTRA2	54103	ES	7	6	8	0.01	1.2E-05	included
ZNF844	47775	ES	3	2	4	-0.05	1.3E-05	excluded
RECQL5	43469	ES	1.2:2.1	1.1	2.2	-0.07	1.3E-05	excluded
ICA1	78794	ES	8	7	10	-0.04	1.3E-05	excluded
BCAT2	50815	ES	4:05	1	6	-0.03	1.3E-05	excluded
COPZ1	22169	ES	5	4	6	0.00	1.3E-05	included
GRAMD1A	49013	ES	10	9	11	0.01	1.3E-05	included
SS18	44969	ES	4	3	9	-0.02	1.3E-05	excluded
CKB	273160	ES	4:05	3	7	0.07	1.3E-05	included
DIO2	28651	ES	4	3.3	5.1	-0.03	1.3E-05	excluded
DHX30	64544	ES	4	3	5	-0.09	1.3E-05	excluded
GNAS	60003	ES	06:08.1	5	8.2	-0.05	1.3E-05	excluded
ATP6V0D1	37080	ES	3	1	4	0.00	1.3E-05	excluded

NHLRC3	25701	ES	4	3	5	-0.06	1.3E-05	excluded
APTX	86078	ES	9	8	10	0.01	1.3E-05	included
TMCO1	8809	ES	7	6	8	0.00	1.4E-05	excluded
GSTK1	82083	ES	3	2	4.1	0.00	1.4E-05	included
NDRG3	59303	ES	16	15	17	0.01	1.4E-05	included
EIF3E	84879	ES	2.1:2.2	1	3	-0.01	1.4E-05	excluded
TPM1	115011	ES	12.1:12.2	11.1	13.1	0.06	1.4E-05	included
MRPL55	10155	ES	1.2:2.5:2.6	1.1	2.9	0.06	1.4E-05	included
MLST8	33219	ES	09:10.1	8.2	11.1	-0.09	1.4E-05	excluded
COX17	66352	ES	2	1	3	0.00	1.4E-05	included
NEK11	66786	ES	9:10:11	8	12	-0.02	1.4E-05	excluded
CATSPERG	49635	ES	5	4	6.1	-0.11	1.4E-05	excluded
FASTKD1	55868	ES	11	10	12	0.04	1.4E-05	included
ZNF410	28328	ES	14	13	16	-0.05	1.4E-05	excluded
FMR1	90274	ES	13	12	14	-0.03	1.4E-05	excluded
PAFAH1B1	38410	ES	2	1	3	-0.02	1.4E-05	excluded
GTF2I	80087	ES	12	11.1	13	0.04	1.4E-05	included
BRD9	71462	ES	18.2:19	17	20	0.01	1.4E-05	included
ERBB2IP	72261	ES	22	21	24.1	-0.05	1.4E-05	excluded
OGFOD2	25008	ES	6.3:8.1	6.2	8.2	0.09	1.5E-05	included
PPP1CA	17183	ES	2.1:2.2	1	3	-0.07	1.5E-05	excluded
LMO3	20622	ES	9.2:10:11.1:11.2	9.1	12	-0.06	1.5E-05	excluded
SNRPA	50001	ES	3	2	4	-0.01	1.5E-05	excluded
ADNP	59791	ES	2	1	4.2	0.08	1.5E-05	included
EML2	50498	ES	19:20	18	21	-0.02	1.5E-05	excluded
CD44	15112	ES	7:8:9.1:9.2:10:11	5	12.1	-0.10	1.5E-05	excluded
SETMAR	62998	ES	2.1:2.2:2.3:2.4	1	3	-0.06	1.5E-05	excluded
SAP130	55254	ES	16	15.2	17	-0.04	1.5E-05	excluded
MPND	46796	ES	11.2	10	12	0.00	1.5E-05	included
NSFL1C	58509	ES	2	1	4	0.01	1.5E-05	included
RHBDD1	57793	ES	2:03	1	4	-0.10	1.6E-05	excluded
DECR1	84409	ES	2:3:4.1:4.2	1	5.2	-0.08	1.6E-05	excluded
HEXA	31550	ES	5	4	6	0.01	1.6E-05	included
EEF1D	85462	ES	4	1	8.1	0.00	1.6E-05	excluded
BTF3L4	2997	ES	2	1	4	0.02	1.6E-05	included
BLOC1S6	30440	ES	5	3	7.1	-0.07	1.6E-05	excluded
CIAPIN1	36550	ES	7	6	8	-0.02	1.6E-05	excluded
SUMF2	79785	ES	9	8	10.2	0.01	1.6E-05	included
CD44	15111	ES	6:7:8:9.1:9.2:10:11	5	12.1	-0.08	1.7E-05	excluded
RNF130	74995	ES	8	7	9	0.00	1.7E-05	included
LRRC23	20007	ES	5	4	6	-0.05	1.7E-05	excluded
CCDC25	83180	ES	5	4	6	0.03	1.7E-05	included
RBM6	64940	ES	3.2	2	4	0.03	1.7E-05	included
COMMD4	31847	ES	7:08	6.2	9.1	0.00	1.7E-05	included
IRF7	13715	ES	4	3.2	5.1	0.07	1.8E-05	included
YAF2	21147	ES	3.1:3.2:5.2:6:7	2	9.1	-0.02	1.8E-05	excluded
ADAM15	7907	ES	21.2	20	23	-0.04	1.8E-05	excluded
RAF1	63446	ES	6	5	7.1	-0.02	1.8E-05	excluded
SNX1	139166	ES	4.2:5:6:7:8:9:10.1:10.2:11:12:13.1:14:15	3	16.1	-0.01	1.8E-05	excluded
PCM1	82842	ES	7	6	8	-0.06	1.8E-05	excluded
CNOT7	82787	ES	3.1:3.2	2	4	0.01	1.8E-05	included
PKIG	59481	ES	2.2	1	4	0.04	1.8E-05	included
AFTPH	53773	ES	8	7	9	0.03	1.8E-05	included
SEMA3F	64964	ES	7	6	8	0.05	1.9E-05	included
ZNF655	80676	ES	8:09	3.2	10	-0.14	1.9E-05	excluded
ABCD4	28393	ES	3	2	4.1	-0.06	1.9E-05	excluded
SDHA	71420	ES	5	4	6	0.04	1.9E-05	included
BZW2	78865	ES	6.2	5	8	0.11	2.0E-05	included
DMKN	49183	ES	7:8:10:11:12	6.4	13	0.12	2.0E-05	included
RNH1	13674	ES	03:04.2	2	4.3	0.09	2.0E-05	included
UIMC1	74693	ES	6.1:7.1:7.2	5	8	-0.04	2.0E-05	excluded

ALCAM	65991	ES	14	13	15	-0.03	2.0E-05	excluded
PLOD2	67137	ES	15	14	16	0.05	2.0E-05	included
LPXN	16011	ES	6	5	7	-0.08	2.0E-05	excluded
BBX	66009	ES	16	15	17	0.06	2.0E-05	included
TXN	87183	ES	3	2	4	0.00	2.1E-05	included
THTPA	26764	ES	1.3:1.4	1.1	2	0.03	2.1E-05	included
ARFGAP2	15642	ES	05:06.1	4.2	6.2	0.06	2.1E-05	included
MATR3	73583	ES	7.2:9	7.1	10	-0.10	2.1E-05	excluded
BCL7B	79959	ES	2	1	3	-0.07	2.1E-05	excluded
MKRN1	82004	ES	3	1	6	-0.04	2.1E-05	excluded
SLC2A11	61346	ES	10.1:10.2	9.1	11	-0.07	2.1E-05	excluded
CTAGE5	27377	ES	21	20	22	-0.02	2.1E-05	excluded
NUDT13	12125	ES	8:09	7	10	0.07	2.2E-05	included
ARRB2	38576	ES	2	1	4.2	-0.07	2.2E-05	excluded
POMT1	87947	ES	3	2	4	-0.03	2.2E-05	excluded
PGS1	43881	ES	2	1	3.1	-0.07	2.2E-05	excluded
PPHLN1	21228	ES	4	2	5	0.03	2.2E-05	included
SUPT5H	49834	ES	6	5	7	0.02	2.2E-05	included
TTC18	12138	ES	25	24.2	26	0.04	2.2E-05	included
FKBP5	75922	ES	4:5:6:7	3.1	8	0.02	2.3E-05	included
TCEA1	83856	ES	3.2	2	4.1	0.00	2.3E-05	excluded
CD44	15114	ES	6:7:8:9.2:10:11	5	12.1	-0.06	2.3E-05	excluded
DMKN	49160	ES	11	8	12	0.08	2.3E-05	included
C17orf62	44358	ES	2:4.2:4.3:5.2:6	1.1	7.1	-0.10	2.3E-05	excluded
SNX1	139168	ES	7:8:9:10.1:10.2:11:12:<br>13.1:14:15	3	16.1	-0.01	2.4E-05	excluded
MPND	46798	ES	7:08	6	9	0.01	2.4E-05	included
RNF115	7297	ES	3	2	4	0.03	2.4E-05	included
RCHY1	69523	ES	7.1	6	8.1	0.02	2.4E-05	included
CREB3L4	7773	ES	4	3.2	5	0.02	2.4E-05	included
CCNB1IP1	26417	ES	9	8	10	0.01	2.4E-05	included
HDAC8	89468	ES	6.1	5	8.1	0.02	2.4E-05	included
RNLS	12466	ES	2:03	1	4	-0.02	2.5E-05	excluded
ZNF530	52304	ES	4.1	3	5	0.13	2.5E-05	included
TMEM120A	80146	ES	10	9	11	0.02	2.5E-05	included
PTPRS	46839	ES	28	27.2	29	0.05	2.5E-05	included
SF1	16682	ES	14.1	13	14.3	-0.04	2.5E-05	excluded
CERS5	21684	ES	4	1	8	-0.08	2.6E-05	excluded
PARL	67809	ES	6	5	7	-0.01	2.6E-05	excluded
PACSIN2	62559	ES	12	11	13	0.05	2.6E-05	included
EEF1D	85448	ES	8.3:10.1:10.2:11:12.2:	8.2	13.2	-0.07	2.6E-05	excluded
HYI	2184	ES	3	2.1	4	-0.05	2.6E-05	excluded
SNRNP70	50886	ES	8.1:8.2:8.3	7	9	-0.08	2.6E-05	excluded
PSMA6	27231	ES	4	2.1	5	0.00	2.7E-05	included
TXNL4A	46282	ES	7.2:8	3	9	0.08	2.7E-05	included
C11orf58	14507	ES	3	2	4	0.00	2.7E-05	included
PPP2R2A	83122	ES	7	6	8	-0.01	2.7E-05	excluded
ACIN1	26708	ES	4	3	5	-0.04	2.7E-05	excluded
CCDC104	53623	ES	2	1	3	0.00	2.7E-05	included
ENPP2	85007	ES	14	13	15	0.06	2.7E-05	included
SCNN1A	19841	ES	12	11	13	0.01	2.8E-05	included
CD200	66102	ES	2:03	1	4	-0.07	2.8E-05	excluded
DMTN	82928	ES	18.2	16	19	0.03	2.8E-05	included
DCAF6	8885	ES	12:13.1	10	14	0.06	2.9E-05	included
REPIN1	82246	ES	3.2:4.2	2.1	5.2	-0.09	2.9E-05	excluded
PRRC2B	87934	ES	31	30	32	0.04	2.9E-05	included
SERPINA1	29129	ES	1.2:2.1:2.2:2.3:2.4:2.5	1.1	3.2	0.13	2.9E-05	included
CRTC1	106128	ES	14	13	15	0.02	2.9E-05	included
SON	60438	ES	11	10	12	-0.06	2.9E-05	excluded
RAD23A	47893	ES	4.1:4.2	3	5	0.00	2.9E-05	included
CLSTN1	576	ES	3	2	4	-0.06	2.9E-05	excluded
HPS1	91779	ES	9	8	10.1	0.03	3.0E-05	included

CYB5R2	14208	ES	10	9	11	-0.04	3.0E-05	excluded
RPS14	74092	ES	1.2:1.3:3	1.1	4	0.05	3.0E-05	included
KHDRBS1	1542	ES	3	2	4	0.01	3.0E-05	included
PRKRIP1	97667	ES	5	4	6	0.07	3.0E-05	included
FAM13A	69911	ES	23	22	24	-0.02	3.0E-05	excluded
HNRNPA1	212643	ES	3:4:5:6.1:6.2:7.1:7.2:8:9.1:9.2:10:11.2	2	11.3	0.00	3.0E-05	excluded
TUBB6	44678	ES	4.1:4.2:5.1:5.2	3	5.3	-0.02	3.0E-05	excluded
SNX1	139169	ES	4.1:4.2:5:6:8:9:10.1:10.2:11:12:13.1:14:15	3	16.1	-0.01	3.0E-05	excluded
DLG5	12292	ES	15	14	16	-0.01	3.1E-05	excluded
SNX1	31096	ES	4.1:4.2:5	3	6	0.00	3.1E-05	excluded
COL1A1	402801	ES	29:30:31:32:33:34:35:36:37:38:39:40:41:42:43:44:45:46:48:49	28	50	-0.06	3.1E-05	excluded
PSTK	91868	ES	4.5	4.3	5	0.06	3.1E-05	included
SNX1	139170	ES	4.2:5:6:8:9:10.1:10.2:11:12:13.1:14:15	3	16.1	-0.01	3.1E-05	excluded
ILDR1	66415	ES	6	5	7	-0.05	3.1E-05	excluded
CITED1	89453	ES	4	3	5	-0.05	3.1E-05	excluded
RTN2	50466	ES	5	4.1	6	-0.10	3.2E-05	excluded
UNK	43510	ES	3	2	4	0.03	3.2E-05	included
TAF15	40340	ES	10	9	11	0.01	3.2E-05	included
CTTN	17403	ES	12	10	13	-0.08	3.2E-05	excluded
TEAD2	50921	ES	6	5	7.1	0.07	3.2E-05	included
COA1	79345	ES	3.1:3.2	1	4.2	0.06	3.2E-05	included
ARHGDIB	20566	ES	2	1	4	0.00	3.2E-05	excluded
ARFGAP2	15643	ES	5	4.2	6.2	0.01	3.2E-05	included
RPS6KB1	42840	ES	2	1	3	-0.04	3.2E-05	excluded
ABCD3	3815	ES	2	1	3	-0.02	3.3E-05	excluded
GMFG	49769	ES	6	5	7.1	0.01	3.3E-05	included
ZNF185	90406	ES	11	10	12	-0.07	3.3E-05	excluded
GATAD2A	48634	ES	14	13.2	15	-0.02	3.3E-05	excluded
RPL27	41175	ES	4.2	3.2	5	0.00	3.3E-05	included
CAPRIN2	20950	ES	5	4	6	-0.09	3.3E-05	excluded
RNF146	77451	ES	04:05.1	2	6	-0.08	3.3E-05	excluded
FRG1	71416	ES	2	1	3	-0.03	3.3E-05	excluded
MADD	15716	ES	36	35.1	37	-0.02	3.3E-05	excluded
TPD52L1	77407	ES	9.1:9.2	8	10	0.03	3.3E-05	included
ZDHHC4	78751	ES	1.3:2.1	1.1	2.2	0.04	3.4E-05	included
IDI1	10610	ES	2	1	3	-0.04	3.4E-05	excluded
ACP5	47750	ES	2.1:2.2	1	3.2	-0.10	3.4E-05	excluded
DNAJC21	71746	ES	10	9.2	11	0.02	3.4E-05	included
SYNE4	114854	ES	7	6	8	-0.02	3.4E-05	excluded
ADAM15	7906	ES	21.1:21.2	20	23	-0.03	3.5E-05	excluded
ABHD14B	65147	ES	3.1	2.2	3.3	0.01	3.5E-05	included
TMEM256-PLSC	38938	ES	6	5.5	7	0.03	3.5E-05	included
USMG5	13004	ES	2.2	1	4	0.01	3.5E-05	included
AASS	81603	ES	20	19	21	0.08	3.5E-05	included
TBC1D23	65817	ES	15	14	16	0.07	3.6E-05	included
BUD31	80622	ES	4	3.2	6	-0.02	3.6E-05	excluded
CDC42	1005	ES	4	3	5	0.00	3.6E-05	excluded
NRSN2	58447	ES	2	1	3	-0.06	3.6E-05	excluded
CCDC91	99554	ES	4	3	8.2	0.05	3.6E-05	included
GPR116	76429	ES	22	21	23	-0.07	3.7E-05	excluded
MAP3K6	1323	ES	3	2	4	-0.07	3.7E-05	excluded
RETSAT	54199	ES	2	1	3	0.00	3.7E-05	excluded
SAT1	88700	ES	2.3	2.1	3	0.00	3.7E-05	included
TCF3	94884	ES	18.1:18.2	17.3	19.1	-0.04	3.7E-05	excluded
TCF7	73347	ES	11:14.1	10	14.2	-0.02	3.7E-05	excluded
GUK1	10185	ES	11.1:11.2	9.2	12	0.00	3.8E-05	excluded
TRIP4	31123	ES	3	2.2	4	0.01	3.8E-05	included

MLLT4	251631	ES	25.1:25.2:26:27:28:29<br>:30:31:33.1:33.2:34.1	24.2	35	-0.11	3.8E-05	excluded
NAB2	22517	ES	3	2	4	0.00	3.8E-05	included
MTFR1L	1213	ES	6	4.2	7.2	0.04	3.9E-05	included
PAPOLA	29207	ES	20	18	21	-0.07	3.9E-05	excluded
CCDC41	23730	ES	15	14	16	0.03	3.9E-05	included
PXK	65444	ES	17.2	16	20	-0.06	3.9E-05	excluded
SFTA3	121949	ES	2:4.1:4.2	1.3	6	0.07	3.9E-05	included
CLUAP1	33593	ES	2	1	3	0.02	3.9E-05	included
FAM98A	53191	ES	4	3	5	0.01	4.0E-05	included
PTP4A3	85338	ES	5.1:5.2	4	6	0.01	4.0E-05	included
TMBIM4	22909	ES	2.2	1	4.1	0.00	4.0E-05	included
CASP9	761	ES	4:5:6:7	3.2	8	-0.01	4.0E-05	excluded
ZNF664	25128	ES	2.1:2.2:3:4	1.4	5	-0.07	4.0E-05	excluded
CDC37	47515	ES	2	1	3	0.00	4.0E-05	excluded
ICMT	387	ES	3	2	4	-0.03	4.0E-05	excluded
MPG	32795	ES	3	2	4	0.01	4.0E-05	included
RABGAP1	87498	ES	19	18	20	-0.02	4.0E-05	excluded
PLD3	49888	ES	4	1.2	5.1	0.08	4.1E-05	included
SNRPG	53887	ES	4	1	5	0.01	4.1E-05	included
D2HGDH	58423	ES	7.1:7.3	6	8	0.10	4.1E-05	included
POLM	79450	ES	9.4:9.5	9.2	9.7	0.07	4.1E-05	included
RAI14	71716	ES	18	17	19	0.10	4.1E-05	included
MBNL1	67309	ES	12	11	13	-0.02	4.1E-05	excluded
ZMAT1	89675	ES	6:07:08	5	9	-0.07	4.2E-05	excluded
BLOC1S1	22234	ES	3	2	4.1	-0.05	4.2E-05	excluded
PPP2R4	87852	ES	4:6:7:8:9:10	3.1	15.1	-0.01	4.2E-05	excluded
NFIB	85888	ES	12.1:12.2:13:14	11	15	0.08	4.3E-05	included
SCAMP5	31823	ES	7	6	8.2	-0.09	4.3E-05	excluded
ACOT8	59634	ES	2	1	4	0.01	4.4E-05	included
WDR53	68249	ES	2.1:2.2:3	1	4	0.05	4.4E-05	included
RANBP3	47007	ES	2	1	4.1	-0.08	4.4E-05	excluded
RNF181	534111	ES	2	1	4.1	-0.05	4.4E-05	excluded
ASPSCR1	44258	ES	12	11	13	-0.02	4.5E-05	excluded
CD46	9658	ES	8:09	6	10	-0.01	4.5E-05	excluded
FTH1	16335	ES	1.2:3.1	1.1	3.2	0.02	4.5E-05	included
PACRGL	68881	ES	11	9	12	-0.17	4.5E-05	excluded
TCF12	30789	ES	18	17	19	0.05	4.6E-05	included
GLOD4	38275	ES	3	1	4.2	-0.05	4.7E-05	excluded
PCNT	60937	ES	45:46:00	44	47	0.03	4.7E-05	included
ASCC2	61681	ES	7	5	8	0.02	4.7E-05	included
FGFR1OP	78436	ES	7	6	8	0.06	4.8E-05	included
SHMT1	39617	ES	9	7	10	0.05	4.8E-05	included
CD74	74077	ES	8	7.1	9	0.03	4.9E-05	included
RHOQ	53466	ES	2	1	3	-0.01	4.9E-05	excluded
TMEM126B	18121	ES	4	1	5.1	0.02	4.9E-05	included
MTMR14	63115	ES	19	18	21	-0.04	4.9E-05	excluded
SFXN2	12967	ES	10:11	9	12	0.01	4.9E-05	included
PTGES3	22479	ES	5	4.2	6	0.00	5.0E-05	included
SEZ6L2	35977	ES	15	14	16	0.04	5.0E-05	included
FAM228B	52815	ES	4:5:6:7:8	3	9.1	0.05	5.0E-05	included
PPP1R12A	23532	ES	15	14	16	0.06	5.1E-05	included
NBPF12	7362	ES	4	3	5	0.02	5.1E-05	included
COPZ1	22165	ES	7	6	8	0.00	5.2E-05	included
FAM122C	90167	ES	7	6	8	0.00	5.2E-05	excluded
TCF7L2	13147	ES	16	14	17	-0.04	5.2E-05	excluded
LEMD1	91352	ES	6	3	7	-0.08	5.2E-05	excluded
CD47	66014	ES	8:09:10	7	11	0.02	5.3E-05	included
COQ9	36560	ES	4.1:4.2:5:6	3	7	0.00	5.3E-05	excluded
MRPS33	82033	ES	5.2	2	6	0.06	5.3E-05	included
TIMMDC1	66313	ES	1.2:2:3:4:5	1.1	6	-0.09	5.4E-05	excluded
NKX2-1	102506	ES	3.2	1	3.4	-0.05	5.4E-05	excluded

P4HA2	73261	ES	2.2:2.3	1	3	0.03	5.4E-05	included
FAM21A	11561	ES	27:28:00	26	29	0.02	5.4E-05	included
KRIT1	80427	ES	3	2.2	4	-0.05	5.5E-05	excluded
DCK	69458	ES	7	6	8	-0.02	5.6E-05	excluded
ARL6IP5	65578	ES	2	1	4.1	0.00	5.6E-05	excluded
MYH14	51169	ES	7	6.1	9	-0.04	5.7E-05	excluded
RAB3GAP1	55433	ES	4:05	3.1	6	-0.10	5.7E-05	excluded
MRPS22	67027	ES	7	6	8	-0.01	5.7E-05	excluded
CDKN1A	75988	ES	3	2.1	4	0.00	5.7E-05	excluded
VPS18	30060	ES	3	2	4	-0.03	5.8E-05	excluded
SAR1A	12041	ES	2.2	1	3	0.02	5.8E-05	included
PCNP	65960	ES	2.2:2.3	1	3	0.02	5.9E-05	included
POLB	83715	ES	2	1	3	0.04	5.9E-05	included
CLCN2	67937	ES	23	22	24	-0.03	6.0E-05	excluded
AMY2B	3911	ES	2	1	3	0.03	6.0E-05	included
C7orf55-LUC7L2	81960	ES	5:06	3.2	7	-0.07	6.0E-05	excluded
NFU1	53854	ES	3	2	4	-0.01	6.0E-05	excluded
METTL4	44482	ES	8	7	9	-0.03	6.0E-05	excluded
MPP1	90664	ES	3	1	4	-0.03	6.0E-05	excluded
MFF	57814	ES	3:05	1	6	0.02	6.0E-05	included
PRDX2	47871	ES	5	4	6	0.00	6.1E-05	excluded
NIN	27495	ES	18	17	19	-0.09	6.1E-05	excluded
MOK	29394	ES	3:04	2	6.1	0.13	6.1E-05	included
LRRC27	13499	ES	8	7	9	-0.04	6.1E-05	excluded
MTMR14	63114	ES	20	18	21	-0.06	6.2E-05	excluded
SDR39U1	27013	ES	4.1	3.2	5	0.01	6.2E-05	included
MMAB	24331	ES	2	1	4	0.01	6.2E-05	included
C14orf159	28870	ES	4	1	7	-0.06	6.2E-05	excluded
TMEM54	1644	ES	03:04.1	2	4.2	0.00	6.3E-05	included
ENOSF1	44467	ES	15:16	13	17	-0.10	6.3E-05	excluded
TRMT2B	89624	ES	5	4	6	0.03	6.4E-05	included
VTA1	77963	ES	7	6	8	-0.01	6.4E-05	excluded
TRMT10B	86435	ES	3.2:4.1:4.2	2	5	0.05	6.4E-05	included
PDDC1	13756	ES	4.1	3	5	0.02	6.4E-05	included
HAX1	7818	ES	2.3:3.1	2.2	3.2	0.09	6.5E-05	included
C8orf58	83032	ES	6.1:6.2	5	7	0.07	6.5E-05	included
MARK2	16540	ES	18:19	17	20	0.09	6.5E-05	included
MYL12A	44491	ES	1.2:2	1.1	4	0.06	6.6E-05	included
OBSL1	57732	ES	13	12	14.1	-0.06	6.6E-05	excluded
INO80C	45176	ES	3:4.1:4.2:5.1:5.2:6:7	1	8	-0.06	6.6E-05	excluded
INO80E	36006	ES	10	6.3	11	-0.03	6.6E-05	excluded
ABCC6	34222	ES	25	24	26	-0.04	6.6E-05	excluded
CORO1B	387275	ES	4:5.1:5.3:5.5:5.7:6	3	7	-0.10	6.6E-05	excluded
MTRF1L	78212	ES	6	5	7	0.03	6.7E-05	included
VAPA	44622	ES	6	5	7	0.01	6.7E-05	included
SLC52A2	85570	ES	3.1:3.2:3.3:4.1	2.4	5	0.00	6.7E-05	excluded
TTC31	54097	ES	3	2	4	0.03	6.7E-05	included
BLOC1S5	75279	ES	5	4	6	-0.03	6.8E-05	excluded
PSMG1	60602	ES	2	1	3	0.01	6.9E-05	included
CLPB	17598	ES	6	5	7	-0.02	7.0E-05	excluded
GLIPR1	23465	ES	3	2	4	0.01	7.0E-05	included
FMR1	90271	ES	15:16.1:16.2	14	16.3	0.01	7.0E-05	included
FRA10AC1	12563	ES	13:15	12	16	0.01	7.0E-05	included
ATP6V0D1	37079	ES	4	1	7	0.00	7.1E-05	included
MCCC1	67777	ES	18	17	19	-0.04	7.1E-05	excluded
CKLF	36732	ES	3.2	1	4	0.01	7.1E-05	included
DCTN3	86189	ES	3.3	3.1	3.5	0.00	7.1E-05	included
PGF	28457	ES	7	6	8	-0.04	7.2E-05	excluded
PLSCR1	67165	ES	6	5	7	0.03	7.2E-05	included
STK11	46395	ES	2	1	3	-0.01	7.2E-05	excluded
PLSCR1	67164	ES	8.1	7	8.3	0.00	7.3E-05	excluded
SNRNP70	50887	ES	8.1:8.2	7	9	-0.02	7.3E-05	excluded

HEXA	31544	ES	11	10	12	0.00	7.4E-05	included
TMX3	45761	ES	6	5	8	0.03	7.4E-05	included
TNFSF12	38968	ES	6	5	7	0.02	7.4E-05	included
DPM1	59792	ES	7	6	8	-0.02	7.4E-05	excluded
ATXN2L	35859	ES	22.4	22.1	22.6	0.01	7.4E-05	included
APBB3	73676	ES	6.3:6.4:6.5:6.6:6.8	6.2	6.9	-0.08	7.5E-05	excluded
LRRC37A	42023	ES	13:14	12	15	0.04	7.5E-05	included
NUP62	51126	ES	1.5	1.2	2.1	-0.07	7.6E-05	excluded
ASL	79868	ES	3	2.2	4	-0.01	7.6E-05	excluded
INPP5J	206702	ES	02:03.1	1	3.3	-0.07	7.6E-05	excluded
ZNF468	51633	ES	3.1:3.2	2	4	-0.09	7.7E-05	excluded
MKNK2	46571	ES	5	4	6	0.00	7.7E-05	included
TBCA	72605	ES	4:05	2	6.1	0.05	7.7E-05	included
TNIP1	74141	ES	4.1:4.2	3	5	-0.01	7.7E-05	excluded
FAM86B1	82708	ES	04:07.1	3.2	8.1	-0.10	7.8E-05	excluded
PI4KB	7591	ES	5	4	6	-0.04	7.8E-05	excluded
SCMH1	2057	ES	6:07	5	8	0.07	8.1E-05	included
MRPL2	76238	ES	3:4:5:6	2.1	7	0.02	8.2E-05	included
RBM39	95936	ES	4:05	3	6	-0.07	8.2E-05	excluded
CD44	15059	ES	7:8:9.2:10:11:12.1:13	5	14	-0.06	8.3E-05	excluded
MRPL53	54074	ES	2	1	3	0.00	8.3E-05	included
COMMD4	31848	ES	7	6.2	9.1	0.02	8.3E-05	included
REPIN1	82243	ES	2.2:3.2:4.1:4.2	2.1	5.2	-0.04	8.4E-05	excluded
HHAT	9694	ES	7	6	8	-0.04	8.4E-05	excluded
PMS2	78701	ES	4	3	5	0.02	8.5E-05	included
TMEM98	40217	ES	2	1	3	0.02	8.5E-05	included
THNSL2	54474	ES	7:08	6	11	0.02	8.6E-05	included
KIAA0430	34165	ES	23	22	24	-0.01	8.6E-05	excluded
CCNC	77078	ES	13.1	12	14	0.01	8.6E-05	included
PABPC1L	59498	ES	12	11	13	0.06	8.7E-05	included
FAM86A	33893	ES	2	1	3.1	-0.05	8.7E-05	excluded
C14orf2	29532	ES	4:05	2	7.1	0.06	8.7E-05	included
DMKN	101856	ES	8:9:11:12	6.4	13	0.13	8.8E-05	included
ARHGEF1	50101	ES	15	14	16	0.06	8.8E-05	included
DNASE1	33613	ES	6.1	5	7	0.11	8.8E-05	included
POLL	12891	ES	3:4:5.1:5.2	1.5	6.1	0.03	8.9E-05	included
C16orf93	94144	ES	4.1:4.3:5:6	3.3	7	0.08	8.9E-05	included
TRAPPC2L	38048	ES	4.1:4.3:4.4	3.2	5.1	0.13	8.9E-05	included
NDUFC1	70625	ES	4	1	5	-0.03	9.0E-05	excluded
IL32	33432	ES	1.2:1.3:1.4:1.5:1.8	1.1	1.9	0.02	9.0E-05	included
STRADA	42973	ES	4	3	6	0.06	9.0E-05	included
NPRL3	32816	ES	2:03	1.4	4	-0.03	9.0E-05	excluded
TRIP6	81014	ES	2.1:2.2	1	3	0.01	9.0E-05	included
LMO3	20620	ES	9.2:10:11.1	9.1	11.2	-0.04	9.1E-05	excluded
FAM86B1	82695	ES	5:06	4	7.1	-0.08	9.1E-05	excluded
NEK4	65260	ES	8	7	9	-0.04	9.1E-05	excluded
PDE8B	72567	ES	8	7	9	-0.02	9.2E-05	excluded
LETMD1	21761	ES	5:06	2	7	-0.03	9.2E-05	excluded
CELF1	15768	ES	3:04	1	6.2	-0.08	9.4E-05	excluded
ARFGAP1	60114	ES	3	2	4	-0.01	9.5E-05	excluded
SUPT7L	53039	ES	2.4	2.1	3	0.00	9.5E-05	included
CD46	9652	ES	13	12	14	0.03	9.6E-05	included
WDR45B	44415	ES	3	2	5	-0.01	9.6E-05	excluded
TXNL4A	46284	ES	05:07.2	3	9	-0.10	9.6E-05	excluded
BBS9	79220	ES	18	17	19.2	0.01	9.6E-05	included
NACA	22500	ES	1.2:2.1	1.1	2.2	0.00	9.6E-05	excluded
ATP5C1	10727	ES	2	1	3	0.00	9.7E-05	included
ASCC2	61682	ES	6	5	7	-0.03	9.7E-05	excluded
FANCL	53657	ES	2:3:4:5	1	6	0.02	9.7E-05	included
LRRC28	32637	ES	9	8	10	-0.01	9.8E-05	excluded
ENOSF1	44472	ES	7:08	5	9.2	-0.02	9.8E-05	excluded
CTSB	82672	ES	3.1	1.1	5.3	0.00	9.8E-05	excluded

BMP1	82991	ES	17.2:18.1	16	18.2	-0.03	9.9E-05	excluded
ARFGAP1	60111	ES	13	12	14.1	-0.05	9.9E-05	excluded
WIPI2	78656	ES	2	1	3	-0.04	9.9E-05	excluded
AAMDC	17981	ES	2	1.3	4	-0.03	1.0E-04	excluded
ANKRD54	62164	ES	8	7	9	0.00	1.0E-04	included
RANBP3	46967	ES	5	4.1	7	-0.05	1.0E-04	excluded
FAIM	67016	ES	3	1	6	-0.07	1.0E-04	excluded
VWDE	78810	ES	9:10:11	8.1	12	0.05	1.0E-04	included
PAF1	49810	ES	12	11	13	0.00	1.0E-04	excluded
ANKS3	33838	ES	2.2	1.1	3	-0.07	1.0E-04	excluded
VWA9	31223	ES	1.2:2.1	1.1	2.2	0.09	1.0E-04	included
ATP5SL	50074	ES	4	3	5	0.03	1.0E-04	included
SFTA3	27276	ES	4.1:4.2	2	6	0.02	1.0E-04	included
ERBB2IP	72267	ES	24.1:24.3	21	25	0.10	1.0E-04	included
ARFRP1	60156	ES	3.2:3.3	1	4	-0.02	1.0E-04	excluded
NDUFA7	47218	ES	3:04	2	5.1	0.03	1.0E-04	included
MED29	49819	ES	2	1	4	0.00	1.0E-04	included
PDLIM2	83026	ES	10	9	11.1	0.02	1.0E-04	included
PQLC1	46263	ES	6	5	9	-0.04	1.0E-04	excluded
MYO5A	30661	ES	33	32	34	0.08	1.1E-04	included
RABEP2	35892	ES	11	10	12	0.01	1.1E-04	included
GLYR1	33864	ES	9	8	10	0.01	1.1E-04	included
CASP8	56825	ES	10	8	11	-0.05	1.1E-04	excluded
ETFA	31930	ES	12	11	13	-0.01	1.1E-04	excluded
ATP5H	120235	ES	3	1	4	-0.08	1.1E-04	excluded
SUGP2	48549	ES	12.5	12.3	14	0.08	1.1E-04	included
NFIB	85889	ES	12.1:13:14	11	15	0.08	1.1E-04	included
FAAH2	89299	ES	8	7	9	0.03	1.1E-04	included
CORO7	33710	ES	3	1.2	4	0.01	1.1E-04	included
GNAS	60005	ES	6	5	8.2	-0.02	1.1E-04	excluded
DMKN	49151	ES	18.3	18.1	18.5	0.08	1.1E-04	included
FANCI	32419	ES	1.2:2:3	1.1	4	-0.13	1.1E-04	excluded
TMEM234	1575	ES	4	3	5.1	-0.04	1.1E-04	excluded
PAOX	13557	ES	5	3	6	0.04	1.1E-04	included
CTNND1	15979	ES	3:4.1:4.2:4.3:5	2.1	6	-0.08	1.1E-04	excluded
SLC41A3	66574	ES	6	3	7	-0.02	1.1E-04	excluded
RHOC	4242	ES	2.2:2.3:2.4	1.1	3	-0.01	1.1E-04	excluded
METTL5	100318	ES	5	4	6.1	0.01	1.1E-04	included
ARFRP1	60157	ES	3.3	1	4	-0.02	1.1E-04	excluded
INO80C	45189	ES	3:4.1:4.2	1	5.1	-0.06	1.1E-04	excluded
C20orf24	59296	ES	03:04.1	2	5	-0.03	1.1E-04	excluded
MRPL55	10166	ES	1.2:2.2	1.1	2.9	0.05	1.1E-04	included
CTSB	97875	ES	02:03.1	1.1	5.3	0.01	1.1E-04	included
FDPS	8071	ES	1.2:2:3.1	1.1	3.2	0.02	1.2E-04	included
AP2B1	40319	ES	16	15	17	-0.04	1.2E-04	excluded
AKIP1	14279	ES	2	1.3	3	0.04	1.2E-04	included
ZNRF1	37579	ES	3	2	4.1	0.00	1.2E-04	excluded
TSSC1	52553	ES	5	4	6	-0.01	1.2E-04	excluded
RTKN	54058	ES	3	2	4	0.07	1.2E-04	included
SMARCC2	22393	ES	18	17	19	-0.04	1.2E-04	excluded
SIVA1	29556	ES	2	1	3	0.00	1.2E-04	included
SORBS2	71381	ES	20:22	19	23	-0.06	1.2E-04	excluded
HNRNPL	49699	ES	8	7	9	-0.02	1.2E-04	excluded
HSCB	61548	ES	2.1:2.2:3.1:3.2:4	1	5	0.05	1.2E-04	included
CADM1	18859	ES	2	1	3	-0.01	1.2E-04	excluded
SUPT7L	53038	ES	2.3:2.4	2.1	3	0.04	1.2E-04	included
PPP6R3	17316	ES	16:17.1:17.2	15	17.3	-0.01	1.2E-04	excluded
CD151	13784	ES	2	1.1	3	0.04	1.2E-04	included
TRIM13	25919	ES	2	1.4	3	-0.04	1.2E-04	excluded
NDUFA11	46955	ES	2	1	3	-0.01	1.2E-04	excluded
SSR2	8148	ES	6.1:6.2	5	7	0.00	1.2E-04	excluded
FOXRED1	19378	ES	2	1.1	3	0.02	1.2E-04	included

NMNAT3	67037	ES	7	5	8	0.05	1.2E-04	included
SLTM	30919	ES	5:06	4.2	7	0.01	1.3E-04	included
TANK	55742	ES	5	4	6.2	-0.01	1.3E-04	excluded
RAB1A	53787	ES	6	5	7	0.00	1.3E-04	excluded
HDAC7	21371	ES	11	10.2	12	0.04	1.3E-04	included
MBD1	45520	ES	13.1:13.2	12	14	-0.02	1.3E-04	excluded
RALGPS2	9094	ES	15	14	16	-0.07	1.3E-04	excluded
PSMA4	32109	ES	5.1:5.2	4	6	0.00	1.3E-04	included
MLLT4	78457	ES	9	8	10	-0.01	1.3E-04	excluded
ZNF667	52179	ES	9	8	12	-0.10	1.3E-04	excluded
CCDC115	55334	ES	5	4	6	-0.02	1.3E-04	excluded
MDH1	53736	ES	5	4	6	0.00	1.3E-04	included
CHMP7	83072	ES	10	9	11	-0.02	1.3E-04	excluded
NDUFB5	67704	ES	3.1:3.2:4.1:4.2	1	5	-0.06	1.3E-04	excluded
OTUD6B	84425	ES	4	3	5	-0.07	1.3E-04	excluded
MBNL1	67324	ES	8	7	9	-0.07	1.4E-04	excluded
CARD8	50714	ES	06:07.2	5	8	0.10	1.4E-04	included
NPM1	74521	ES	9	8	10	0.00	1.4E-04	included
SRPK2	97698	ES	19	18	20	-0.08	1.4E-04	excluded
CCT7	53965	ES	3:5.2:6:7	1	8	0.10	1.4E-04	included
UBP1	63866	ES	13	12	14	-0.03	1.4E-04	excluded
USP54	12167	ES	13	12	14	-0.05	1.4E-04	excluded
RAB1A	53788	ES	6:07	5	8	0.00	1.4E-04	excluded
SFTA3	27280	ES	03:04.1	2	4.2	-0.06	1.4E-04	excluded
TUFM	35864	ES	3	2	4	0.00	1.4E-04	included
TCF3	94888	ES	17.3	17.1	18.1	-0.03	1.4E-04	excluded
ABCB9	25001	ES	10	9	11	-0.04	1.4E-04	excluded
PPP1R7	58335	ES	2	1.3	3	-0.01	1.4E-04	excluded
FLAD1	91161	ES	2.1:2.2:3:4.1:4.3	1.3	6.1	0.06	1.4E-04	included
C17orf62	44357	ES	3.2:4.2:4.3:5.2:6	1.1	7.1	-0.06	1.5E-04	excluded
PBXIP1	7849	ES	2.1:2.2:3.1:3.2:4:5.1	1	5.2	0.00	1.5E-04	excluded
TRAPPC6B	27360	ES	4	3	5	0.03	1.5E-04	included
LMO7	26068	ES	10:11	9	12	-0.07	1.5E-04	excluded
TRMT11	77437	ES	10:11	9.1	12	-0.01	1.5E-04	excluded
AP2A2	13821	ES	15	14	16	0.03	1.5E-04	included
ATP13A2	841	ES	22	21	23	0.01	1.5E-04	included
DERL2	38704	ES	5.2	4.3	6	-0.08	1.6E-04	excluded
CERS4	47209	ES	3.2	2	4	-0.08	1.6E-04	excluded
DPF2	16815	ES	7	6	8	0.01	1.6E-04	included
IL32	33437	ES	1.3:1.4:1.5:1.6	1.1	1.9	0.04	1.6E-04	included
GSN	87430	ES	15.1:15.2	10	16	-0.04	1.6E-04	excluded
HMGN1	60621	ES	6.2:8.2	5	9	0.00	1.6E-04	included
UQCRB	84615	ES	5	4	6.4	0.00	1.6E-04	excluded
DLG1	68293	ES	9:10	7	11	0.04	1.6E-04	included
TANK	55744	ES	2.2:3	2.1	4	-0.08	1.6E-04	excluded
ZNF142	57508	ES	4	3.2	5	-0.08	1.6E-04	excluded
CCSER2	12409	ES	11	10	12	-0.04	1.6E-04	excluded
FAM86C1	17443	ES	3.2	2	4	0.07	1.6E-04	included
TCEB2	33303	ES	3	2.2	4.1	0.00	1.6E-04	included
GATC	24756	ES	3	2	4	-0.05	1.6E-04	excluded
KIAA1217	11002	ES	23:24	22	25.1	0.01	1.6E-04	included
UPP1	97465	ES	6.1:6.2	4	9	-0.01	1.6E-04	excluded
MAPK9	75011	ES	9	7	10.1	-0.02	1.6E-04	excluded
DHPS	47832	ES	8	7	9.1	-0.01	1.6E-04	excluded
ARL17A	42031	ES	4.1	3	7	-0.10	1.6E-04	excluded
FAM49B	85161	ES	5	3	6	-0.06	1.6E-04	excluded
SNX1	139167	ES	6:7:8:9:10.1:10.2:11:12:13.1:14:15	3	16.1	-0.01	1.6E-04	excluded
POP5	24773	ES	3	2	4	0.02	1.6E-04	included
ATXN2L	35849	ES	22.2:22.4:22.6	22.1	22.7	0.01	1.6E-04	included
TNFSF10	67647	ES	3:04	2	5	-0.01	1.7E-04	excluded
ATXN2	24518	ES	21	20	22	0.05	1.7E-04	included

TMEM25	19014	ES	5	4	6	0.02	1.7E-04	included
TMEM126B	18123	ES	2.1:2.2	1	4	-0.02	1.7E-04	excluded
DPAGT1	19116	ES	3	2.3	4.2	0.01	1.7E-04	included
RABL2B	62929	ES	2.1:2.2:3.1	1	3.2	0.07	1.7E-04	included
NDUFA3	95374	ES	4.1:4.2:4.3	2	5.1	0.08	1.7E-04	included
C12orf76	24408	ES	9.1	8	10.2	0.05	1.8E-04	included
SFTA3	27265	ES	4.2	2	5	0.04	1.8E-04	included
SAMD4A	27598	ES	13	12	14	0.02	1.8E-04	included
ABI2	57061	ES	5.3:8:10:12	5.2	14	0.09	1.8E-04	included
PIGQ	32904	ES	13	11	14	-0.06	1.8E-04	excluded
MMS19	12716	ES	23	22	24	0.01	1.8E-04	included
RNF121	17461	ES	5	3	6.1	-0.02	1.8E-04	excluded
CTBS	3607	ES	3	2	4	0.03	1.8E-04	included
NBPF11	7340	ES	5:8.1:8.2	4	8.3	0.14	1.8E-04	included
EEF1B2	57138	ES	03:04.1	2.2	4.2	0.00	1.8E-04	excluded
STAU2	84167	ES	5	4	7	0.05	1.8E-04	included
KIF3C	52880	ES	6	5	8	-0.10	1.8E-04	excluded
SMARCE1	40880	ES	3	2.2	4.1	0.00	1.8E-04	included
TMEM107	39122	ES	3.2:3.4	2	3.7	0.07	1.8E-04	included
IL32	33433	ES	1.3:1.4:1.5:1.8	1.1	1.9	0.02	1.8E-04	included
VWA9	31211	ES	1.2:2.1:2.2:2.3	1.1	3.1	0.06	1.8E-04	included
RNASEK	38817	ES	2.2	1	3	0.01	1.8E-04	included
IRF2	71294	ES	4	3	5	-0.02	1.8E-04	excluded
TMEM180	12955	ES	4	3	5	-0.05	1.8E-04	excluded
ADPGK	31586	ES	5	4	7	0.01	1.8E-04	included
SNAP23	30177	ES	7.1	6	8	-0.01	1.8E-04	excluded
ACTB	264670	ES	3:04	1	5	-0.01	1.8E-04	excluded
SSB	55885	ES	6	5	7	-0.05	1.8E-04	excluded
WARS	29299	ES	4	1	7	0.02	1.9E-04	included
HADHB	52890	ES	4	3	5	-0.02	1.9E-04	excluded
C20orf24	59297	ES	4.1	2	5	0.00	1.9E-04	excluded
CCDC90B	18068	ES	3	2	4.2	-0.02	1.9E-04	excluded
MRPL55	10138	ES	2.2:2.3:2.4:2.5:2.6:2.8	1.1	2.9	0.04	1.9E-04	included
TMUB2	41808	ES	2.3:2.4:2.5:3:4.2	2.2	4.3	-0.07	1.9E-04	excluded
ZNF207	40205	ES	10	9	11	0.04	1.9E-04	included
SETD5	63093	ES	07:08.1	6	8.2	0.06	1.9E-04	included
CD55	9639	ES	10.1	9	15	0.01	1.9E-04	included
FMR1	90272	ES	15:16.2	14	16.3	0.04	2.0E-04	included
WDR26	9960	ES	10	9	11	-0.01	2.0E-04	excluded
ACP1	52510	ES	5	4.2	6	0.01	2.0E-04	included
TMEM8B	86322	ES	5:06	4.2	7	-0.03	2.0E-04	excluded
ZNF655	80682	ES	9	3.2	10	-0.09	2.0E-04	excluded
LRRCC1	84319	ES	5:06:07	4	8	0.02	2.0E-04	included
PPP1R32	16242	ES	8	7	9	0.07	2.0E-04	included
SRSF7	53284	ES	4.3:4.4	4.1	4.6	0.05	2.0E-04	included
RBCK1	58456	ES	2	1	3	0.01	2.0E-04	included
BRD7	36380	ES	10	9	11	0.01	2.0E-04	included
GGT1	61444	ES	6.2	4	7.3	-0.07	2.0E-04	excluded
ANK2	70397	ES	42	41	43	-0.16	2.0E-04	excluded
PPIP5K1	30267	ES	31	30	32	-0.06	2.0E-04	excluded
ALDH7A1	73172	ES	2	1	3.2	-0.01	2.0E-04	excluded
ZDHHC17	23508	ES	8	7	9	0.07	2.0E-04	included
CD46	9657	ES	7:08:09	6	10	-0.08	2.1E-04	excluded
TMEM14B	75315	ES	10:11	8	12	0.14	2.1E-04	included
CREM	11285	ES	4:9.2:10.1:11	1	15	0.10	2.1E-04	included
SIRT3	13609	ES	2.2:3.1:3.2:4:5.1:5.2:5.	2.1	6.1	0.12	2.1E-04	included
HOPX	69368	ES	5	4.6	6	0.00	2.1E-04	included
CIRBP	46430	ES	9.5:9.6	9.3	9.8	-0.05	2.1E-04	excluded
HNRNPH1	74905	ES	13	12	14	0.01	2.1E-04	included
SORBS2	71382	ES	22	19	23	-0.02	2.1E-04	excluded
DCAF8	91248	ES	8.1:8.2	7.2	9	-0.04	2.1E-04	excluded
SLC25A13	80550	ES	4	3	5	-0.02	2.1E-04	excluded

ST3GAL5	122013	ES	7	6	9.2	-0.03	2.1E-04	excluded
SLC25A26	65549	ES	3	2	4	-0.08	2.1E-04	excluded
NUDT9	69867	ES	5	4	6	0.00	2.2E-04	excluded
SNAPC5	31276	ES	2.1:2.2	1.1	3.1	0.04	2.2E-04	included
C3orf17	66151	ES	4.1:4.2:5.1	3.1	6.1	0.01	2.2E-04	included
ARMC6	48581	ES	2.2	1.1	5	0.04	2.2E-04	included
KPNA2	43124	ES	5:6:7:8	4.2	9	0.07	2.2E-04	included
GADD45A	3356	ES	2	1	3	0.00	2.2E-04	included
COL1A1	265303	ES	15:16:17:18:19:20:21:<br>22:25:26:27	14	28	-0.05	2.2E-04	excluded
PSME3	41153	ES	6	4	7	-0.01	2.2E-04	excluded
C1orf86	250	ES	6	5	7.1	-0.04	2.2E-04	excluded
GRAMD3	73164	ES	7	5.2	8	0.01	2.2E-04	included
PAX8	533932	ES	9.2:10	8	11	-0.03	2.2E-04	excluded
FMR1	90273	ES	15	14	16.3	0.03	2.2E-04	included
TMEM50B	60420	ES	6	5	7	-0.01	2.2E-04	excluded
IDUA	68443	ES	2	1	4	0.01	2.2E-04	included
NUCB2	14526	ES	16	15	18	-0.01	2.2E-04	excluded
YIF1A	17008	ES	7	6	8.2	0.00	2.2E-04	excluded
CPNE1	59190	ES	2.1:2.2:3	1.2	5	-0.06	2.3E-04	excluded
SUPT20H	25668	ES	4	3	5	0.06	2.3E-04	included
ECHDC2	3036	ES	2.2:5.1	2.1	6.2	-0.01	2.3E-04	excluded
SULF1	84105	ES	3	2	4	-0.06	2.3E-04	excluded
TAPT1	68830	ES	12	11	13	0.01	2.3E-04	included
CTSB	82673	ES	2	1.1	5.3	0.03	2.3E-04	included
TRIQK	84490	ES	05:06.1	4	6.2	0.07	2.3E-04	included
PRPSAP2	39673	ES	4	3	5	-0.03	2.4E-04	excluded
EIF3M	14854	ES	2.1:2.2:3:4	1	5	0.00	2.4E-04	included
NABP1	56614	ES	04:05.1	3	5.2	-0.12	2.4E-04	excluded
ASCC1	12080	ES	14:15	13	16	-0.04	2.4E-04	excluded
REPIN1	82236	ES	4.2:5.2	3.2	5.3	-0.08	2.4E-04	excluded
RABEP1	38672	ES	16	15	17	0.01	2.4E-04	included
UBE2V1	59762	ES	3	2	5	0.02	2.4E-04	included
DTNB	52867	ES	17	16	19	0.01	2.4E-04	included
SEC24C	12178	ES	8	7	9	0.02	2.4E-04	included
SMARCC2	22392	ES	28.3	28.1	29	0.08	2.4E-04	included
TIA1	53875	ES	5:06	4	7	-0.06	2.4E-04	excluded
THTPA	26766	ES	1.3	1.1	2	0.02	2.5E-04	included
CHEK2	61529	ES	12	11	13	0.05	2.5E-04	included
PGAP2	14025	ES	6	4	10	-0.04	2.5E-04	excluded
ZNF195	13979	ES	9:11	5.1	13	-0.05	2.5E-04	excluded
STEAP3	55102	ES	2	1.1	5.3	-0.09	2.5E-04	excluded
ST5	14273	ES	8	7	9	0.02	2.5E-04	included
CC2D2A	68812	ES	2	1.1	3.3	-0.04	2.5E-04	excluded
NME2	42514	ES	8	7	9	-0.04	2.5E-04	excluded
AFAP1	68741	ES	13	12	14	-0.03	2.5E-04	excluded
NOP2	19889	ES	16	15	17	-0.01	2.5E-04	excluded
TSPAN4	13798	ES	7	5.2	8	0.00	2.6E-04	excluded
MGAT4C	23586	ES	8:09:10	6.2	11.1	-0.07	2.6E-04	excluded
C20orf24	59298	ES	3	2	5	-0.09	2.6E-04	excluded
CREM	11264	ES	9.2:10.1	4	15	0.07	2.6E-04	included
NKX2-1	27289	ES	3.2	2	3.4	-0.07	2.6E-04	excluded
LYRM1	34415	ES	5	3	8.1	0.08	2.6E-04	included
E2F4	36968	ES	03:04.2	2.2	5	0.01	2.6E-04	included
TGFBR2	63806	ES	2	1	3	0.04	2.6E-04	included
ELMOD3	54206	ES	9	8	10	0.05	2.6E-04	included
TIMM17B	89021	ES	4.3	3	5	-0.01	2.6E-04	excluded
REXO2	18838	ES	4:5.2:5.3:6:7	2	8	0.00	2.6E-04	included
RHNO1	19722	ES	3	2.2	4	-0.02	2.6E-04	excluded
ACTB	204989	ES	2:03:04	1	5	-0.01	2.6E-04	excluded
ACTG1	44119	ES	3	2	4	0.00	2.6E-04	excluded
DGUOK	54008	ES	4:05:06	1	7	-0.04	2.7E-04	excluded

DEPDC5	61901	ES	36	35	37	-0.02	2.7E-04	excluded
ISCU	24235	ES	5.3	5.1	6	0.00	2.7E-04	included
DOCK9	26179	ES	40	39	41	-0.02	2.7E-04	excluded
AP2S1	50600	ES	4.1:4.2	3.2	5	0.05	2.7E-04	included
ETV4	41712	ES	8	7	9	0.01	2.7E-04	included
OGFOD3	44324	ES	9	8	10	-0.04	2.7E-04	excluded
NUDT22	16586	ES	2:03	1.4	4	0.00	2.7E-04	included
C8orf59	84333	ES	5	3.2	6	0.01	2.8E-04	included
PSMD7	37562	ES	2	1	3	-0.03	2.8E-04	excluded
CD44	15274	ES	3.1:3.2:4:5:6:7:8:9.2:10:11:12.1:13:14:15:16	2.1	17.2	-0.13	2.8E-04	excluded
POSTN	25673	ES	18:19	16	20	0.02	2.8E-04	included
ANKRD10	26274	ES	4	3	6.1	-0.07	2.8E-04	excluded
MINK1	38598	ES	19	18	20	0.03	2.8E-04	included
THEMIS2	1354	ES	3	2	5	0.08	2.8E-04	included
PKIG	59480	ES	2.2:3	1	4	0.01	2.8E-04	included
CINP	29414	ES	5	4	6	0.00	2.8E-04	excluded
METTL1	22747	ES	4	3	5	0.01	2.8E-04	included
EFCAB4A	13776	ES	8	7	9	-0.02	2.8E-04	excluded
TRAPPC6A	50410	ES	1.2:2	1.1	3	-0.04	2.9E-04	excluded
CPSF7	16215	ES	5.1:5.2	3	6.1	-0.02	2.9E-04	excluded
CENPC	69412	ES	10	9	11	-0.01	2.9E-04	excluded
SMS	88682	ES	3	2	4	0.00	2.9E-04	included
ABHD14B	65144	ES	3.1:3.3	2.2	6.1	0.06	2.9E-04	included
PSMD12	43113	ES	3	1	4	-0.01	2.9E-04	excluded
SLC47A1	39739	ES	16	15	17	0.04	2.9E-04	included
CD44	15268	ES	3.1:3.2:4:5:6:7:8:9.1:9.2:10:11:12.1:13:14:15:16.1:16.2:17.1	2.1	17.2	-0.13	2.9E-04	excluded
VLDLR	85739	ES	16	15	17	0.05	2.9E-04	included
AMN1	21016	ES	2:03:04	1	5	-0.02	3.0E-04	excluded
RHOC	4247	ES	2.2:2.3	1.1	3	0.03	3.0E-04	included
CAMTA2	38636	ES	7.3	6	8	0.03	3.0E-04	included
LDB2	68844	ES	9.2:11.1	9.1	11.2	0.11	3.0E-04	included
TTC38	62703	ES	3	2	4	-0.03	3.0E-04	excluded
IST1	37519	ES	12	11	14.1	0.00	3.0E-04	excluded
EP400	25233	ES	24	23	25	0.06	3.0E-04	included
ILK	14174	ES	2	1.4	3	-0.01	3.0E-04	excluded
MON1B	37653	ES	3.2:3.3	2	4	0.01	3.0E-04	included
RNF13	67242	ES	2	1	3	0.01	3.0E-04	included
FAM86B1	82700	ES	4:7.1:7.2:7.3	3.2	8.1	-0.09	3.0E-04	excluded
OCIAD2	69259	ES	3	2	4.1	0.01	3.1E-04	included
ABHD17A	46559	ES	03:04.1	2.2	4.2	0.07	3.1E-04	included
GSTO2	13052	ES	5	4	8	0.04	3.1E-04	included
PTPN18	55346	ES	3	1	7	-0.07	3.1E-04	excluded
LYPLAL1	91406	ES	3.2	2	4	0.06	3.1E-04	included
SETD3	29218	ES	4	3	5	0.01	3.1E-04	included
FAM92A1	84522	ES	13	12.1	14	-0.02	3.1E-04	excluded
PPHLN1	21222	ES	8:09:10	7	11	0.07	3.1E-04	included
PTK2	85316	ES	20	19	21.2	-0.01	3.1E-04	excluded
NAP1L1	23489	ES	4	2	5	-0.05	3.1E-04	excluded
IMMP1L	14816	ES	3:4:5:6	1	7	0.06	3.1E-04	included
RBMS2	22466	ES	4	3	5	0.02	3.1E-04	included
ACP1	52514	ES	4.1	3	4.4	0.05	3.2E-04	included
FAM211B	61458	ES	2	1	3	-0.06	3.2E-04	excluded
RAI2	88597	ES	3.2	2	4.1	-0.05	3.2E-04	excluded
MEF2BNB-MEF2	95081	ES	13	12	14	0.05	3.2E-04	included
OGG1	63170	ES	4:5:6.1:7.2	3	8	-0.09	3.2E-04	excluded
EFCAB2	10483	ES	2	1.3	3	-0.06	3.2E-04	excluded
DARS	55452	ES	2	1	3	0.00	3.2E-04	included
PPP1R2	68180	ES	3	2	4	-0.01	3.2E-04	excluded
MPZL1	8871	ES	5	4	6	-0.02	3.3E-04	excluded

POLR2B	69390	ES	3	2	4	-0.01	3.3E-04	excluded
NDRG2	26510	ES	4.2:4.3:4.5	4.1	5.2	-0.01	3.3E-04	excluded
C6orf1	75776	ES	3	2.2	4	0.01	3.3E-04	included
AKAP2	87180	ES	11	10	13	0.04	3.3E-04	included
CWC25	40599	ES	3	2	4	-0.02	3.3E-04	excluded
ITGB6	55712	ES	11:12	10	13	-0.01	3.3E-04	excluded
PARP11	19764	ES	4	3	5	-0.07	3.3E-04	excluded
C16orf13	265880	ES	3	1	4	0.08	3.3E-04	included
FBXO44	660	ES	3.1:3.2	2	4	-0.03	3.3E-04	excluded
CEP164	18916	ES	8	7	9	-0.06	3.4E-04	excluded
ARMC6	48567	ES	4	2.2	5	-0.05	3.4E-04	excluded
GLB1	63833	ES	8	7	9	0.00	3.4E-04	excluded
ACAD10	24536	ES	6	5	7	0.05	3.4E-04	included
TBCA	72603	ES	6.1	4	6.3	0.00	3.4E-04	included
CCL28	71949	ES	4	3	5.1	-0.03	3.4E-04	excluded
TPD52L1	77419	ES	8	6	10	-0.03	3.4E-04	excluded
DAP3	8121	ES	5.1	4	6	0.01	3.4E-04	included
FGFR1OP2	20856	ES	5.1	4	6	-0.05	3.4E-04	excluded
TEX264	65102	ES	2:03	1.1	4	-0.07	3.5E-04	excluded
NSMCE1	35681	ES	2	1	3	-0.01	3.5E-04	excluded
SHMT1	39615	ES	8	7	9	-0.02	3.5E-04	excluded
LIMCH1	69117	ES	28	27	29	-0.06	3.5E-04	excluded
CKLF	36731	ES	02:03.2	1	4	0.01	3.5E-04	included
CEP63	66880	ES	16:17	15	18	0.04	3.5E-04	included
GRAMD3	73166	ES	6	5.2	7	-0.01	3.5E-04	excluded
RSRC2	24969	ES	4	3	5.1	-0.07	3.5E-04	excluded
C8orf44	84030	ES	3	1	4	0.06	3.5E-04	included
SFTA3	27254	ES	4.3:5	4.2	6	-0.06	3.5E-04	excluded
OGFOD2	25009	ES	7	6.2	8.2	0.09	3.6E-04	included
BRD9	71461	ES	18.1:18.2:19	17	20	0.00	3.6E-04	included
HDLBP	58348	ES	11	10	12	0.00	3.6E-04	included
TREM2	76098	ES	4.2	3	5	-0.01	3.6E-04	excluded
PIP5K1C	46724	ES	18	16	19	-0.05	3.6E-04	excluded
IL17RE	63247	ES	4:05	2.2	6	-0.07	3.6E-04	excluded
CAMTA2	38633	ES	21	20	22	0.00	3.6E-04	included
PPARD	75913	ES	3	2	4.1	-0.01	3.7E-04	excluded
KLC1	29484	ES	14.1:15	13.2	18	-0.01	3.7E-04	excluded
ECHDC2	90972	ES	10	8.1	11	0.01	3.7E-04	included
PARP8	71983	ES	9	8	10	-0.05	3.7E-04	excluded
BCKDK	36239	ES	10	9	11	0.01	3.7E-04	included
GKAP1	86693	ES	11	10	12	0.04	3.7E-04	included
SGCB	69277	ES	2	1	3	0.02	3.7E-04	included
ELOF1	94992	ES	4	1	7	-0.01	3.7E-04	excluded
COX7A2	76780	ES	4.1	3	5	0.00	3.8E-04	included
PIGT	59574	ES	2.1:2.2	1	4	-0.09	3.8E-04	excluded
MAZ	35942	ES	6.1	5.1	7.1	0.03	3.8E-04	included
CNOT2	23381	ES	3.2	2	6	0.02	3.8E-04	included
SMN1	72425	ES	6	5	7	-0.01	3.8E-04	excluded
MYB	77818	ES	17	16	19.1	-0.07	3.8E-04	excluded
R3HDM2	22577	ES	13	11	15	0.07	3.8E-04	included
TIMMDC1	66314	ES	2:3:4:5	1.1	6	0.00	3.8E-04	excluded
PPCDC	31830	ES	4	3.2	5	-0.01	3.9E-04	excluded
MRPL55	10109	ES	2.2:2.5:2.6:2.7:2.8	1.2	2.9	0.02	3.9E-04	included
NMRAL1	33734	ES	3	2.6	5	0.02	3.9E-04	included
PCM1	82839	ES	25:26:00	24	27	0.04	3.9E-04	included
COL1A1	402802	ES	29:30:31:33:34:35:36:<br>37:38:39:40:41:42:43:	28	50	-0.02	3.9E-04	excluded
NAB2	22516	ES	6	5	7	0.01	4.0E-04	included
LDHA	14634	ES	3:04	2.4	6.1	-0.02	4.0E-04	excluded
TMEM107	39118	ES	3.2:3.4:3.5	2	3.7	0.01	4.0E-04	included
TP53BP2	9930	ES	3	2	4	-0.08	4.0E-04	excluded
NAT9	43295	ES	6.5:6.6:6.7:7.1	6.2	7.2	0.01	4.0E-04	included

TMEM44	68161	ES	14.1:14.2	13.2	15	-0.05	4.0E-04	excluded
NR1D2	63736	ES	6	5	7	-0.03	4.0E-04	excluded
TALDO1	13738	ES	2	1	3	-0.03	4.0E-04	excluded
EML2	50500	ES	19	18	20	-0.05	4.0E-04	excluded
SFTA3	121950	ES	03:04.2	1.3	6	0.05	4.0E-04	included
KANSL2	21458	ES	3	2	4	0.03	4.1E-04	included
ENTPD6	58866	ES	2	1	3.1	0.06	4.1E-04	included
PTPRK	77502	ES	3	1	4	-0.03	4.1E-04	excluded
C12orf73	24072	ES	03:04.1	2.4	5	-0.08	4.1E-04	excluded
MORF4L1	32130	ES	6	5	7	0.00	4.1E-04	included
SNX1	139171	ES	6:8:9:10.1:10.2:11:12:13.1:14:15	3	16.1	-0.01	4.1E-04	excluded
PPP3CC	83012	ES	14	13	15	0.05	4.2E-04	included
C11orf49	15612	ES	9	7	10	-0.01	4.2E-04	excluded
FOXP1	65603	ES	27	26	28	-0.01	4.2E-04	excluded
GOLT1B	20703	ES	4	2	5	-0.05	4.2E-04	excluded
KRBA1	82204	ES	13:14	12	15	-0.04	4.2E-04	excluded
AZI1	44107	ES	19	18	20	-0.02	4.2E-04	excluded
PLXNB2	96240	ES	2.1	1	3	-0.04	4.2E-04	excluded
RNF146	77449	ES	4	2	5.1	-0.06	4.3E-04	excluded
ZNF160	51656	ES	3.2:3.3	2	4	0.08	4.3E-04	included
RPL35A	68337	ES	4	3.1	5	0.00	4.3E-04	excluded
PTBP1	46318	ES	9.2	8	10	0.03	4.3E-04	included
RBM5	100789	ES	16	15	17	0.01	4.3E-04	included
MEF2BNB-MEF2	95084	ES	5	4	7	-0.07	4.3E-04	excluded
PHF20L1	85200	ES	6.2	5	7	-0.05	4.3E-04	excluded
GSTO2	13054	ES	8	4	9	-0.04	4.3E-04	excluded
ENPP5	76412	ES	2	1	3	-0.05	4.3E-04	excluded
CASK	88865	ES	21	19.1	22	-0.07	4.3E-04	excluded
PLSCR1	67169	ES	4	1	5	0.01	4.4E-04	included
CHTOP	91132	ES	4.1:4.2	3.2	5	-0.09	4.4E-04	excluded
PFKFB2	9618	ES	15	14	16	0.01	4.4E-04	included
TIE1	2161	ES	9	6	10	0.04	4.4E-04	included
MAP2K3	39819	ES	4	1	5	-0.03	4.4E-04	excluded
TANGO2	61122	ES	7.2:8	6	9	-0.02	4.4E-04	excluded
KLHDC10	97728	ES	2	1	3	-0.06	4.4E-04	excluded
ENOSF1	123119	ES	17	13	18.1	0.08	4.5E-04	included
NUMB	28294	ES	7	6	8.2	-0.05	4.5E-04	excluded
SKP1	73361	ES	3	1	4	0.00	4.5E-04	included
KANSL2	93039	ES	7	6	8.1	0.03	4.5E-04	included
FAM60A	20989	ES	3	1	4	0.05	4.6E-04	included
FBXO18	10672	ES	21	20	22	0.01	4.6E-04	included
SMN2	72411	ES	6	5	7	-0.01	4.6E-04	excluded
CD44	15056	ES	7:8:9.1:9.2:10:11:12.1	5	14	-0.05	4.6E-04	excluded
SENP6	76797	ES	8	6	9	0.05	4.6E-04	included
UBE2J2	54	ES	3	2.1	5	0.00	4.6E-04	excluded
SEPT2	58373	ES	6	2	7	0.00	4.6E-04	excluded
MRPL52	26644	ES	1.2:1.4	1.1	1.5	-0.03	4.6E-04	excluded
CD200	66101	ES	2	1	3	-0.04	4.6E-04	excluded
RPL18A	48384	ES	2.2:2.4	1	3	0.09	4.6E-04	included
MKRN1	152272	ES	3:06	1	7	-0.11	4.7E-04	excluded
TMEM66	83265	ES	2.2:2.3:2.4	1.2	3	-0.05	4.7E-04	excluded
CRELD2	62760	ES	6	5	7	-0.02	4.7E-04	excluded
RHNO1	19725	ES	2.1:2.2:3	1	4	-0.07	4.7E-04	excluded
BLNK	12676	ES	9	8	10	0.03	4.7E-04	included
BLOC1S6	30435	ES	7.1:7.2	3	8	0.01	4.7E-04	included
ATF2	56065	ES	15	14	16	-0.01	4.7E-04	excluded
TANGO2	61123	ES	8	6	9	-0.06	4.8E-04	excluded
YAF2	21162	ES	5.1:5.2	2	9.1	-0.02	4.8E-04	excluded
STRADA	42976	ES	3:04	2.2	6	0.09	4.8E-04	included
VWA5A	19219	ES	1.3	1.1	2.2	0.07	4.8E-04	included
LMAN2L	54575	ES	3	2	4	-0.04	4.8E-04	excluded

PLA2G15	37203	ES	2	1	3	-0.07	4.8E-04	excluded
DNAJC19	67762	ES	3	1	4	0.01	4.8E-04	included
IFI27	29087	ES	3.2	1	4.1	-0.04	4.8E-04	excluded
ATP5J2	80643	ES	4	3.2	5.1	0.00	4.9E-04	excluded
FMNL1	41954	ES	26	25	27	-0.02	4.9E-04	excluded
TGS1	83880	ES	2	1	3	-0.02	4.9E-04	excluded
DMKN	49178	ES	11	7	12	0.02	4.9E-04	included
AP2A1	51066	ES	16	15	17	-0.02	4.9E-04	excluded
FAM26F	77318	ES	2	1	3	0.09	5.0E-04	included
PKM	31520	ES	4	2.1	5.1	0.00	5.0E-04	excluded
C5orf28	71956	ES	4	1	5	-0.02	5.0E-04	excluded
ECHDC2	3023	ES	13	12	14	-0.01	5.0E-04	excluded
PDCD10	67562	ES	3.2:3.3	1.1	5	0.02	5.0E-04	included
HYOU1	19084	ES	18.1:18.2	17	19	0.09	5.0E-04	included
WDSUB1	55693	ES	5:6:7:8	4	9	-0.01	5.1E-04	excluded
MEFV	33476	ES	6	5	7	-0.02	5.1E-04	excluded
SMAP1	76647	ES	6	5.2	7	-0.04	5.1E-04	excluded
HMOX2	33746	ES	3:07	2.2	8	-0.01	5.1E-04	excluded
MARK3	29449	ES	18	16	19	-0.02	5.1E-04	excluded
RFC5	24686	ES	2.1	1	3	0.04	5.1E-04	included
DYNC1I2	55943	ES	8	7.3	9	-0.06	5.2E-04	excluded
LGMN	29006	ES	13	12	15	-0.05	5.2E-04	excluded
BAZ1A	27174	ES	13	12	14	-0.05	5.2E-04	excluded
RNF7	67074	ES	2:03	1.2	4	-0.06	5.2E-04	excluded
ZNF664	25127	ES	4	2.2	5	-0.02	5.2E-04	excluded
RNF121	17451	ES	6.1:6.2	3	7	0.01	5.3E-04	included
PIGP	60543	ES	4	3.2	5	-0.02	5.3E-04	excluded
MRPL55	10144	ES	1.2:2.5:2.6:2.8	1.1	2.9	0.01	5.3E-04	included
ABCC6	34224	ES	3	2.1	4	0.07	5.3E-04	included
KLC1	29479	ES	13.3:14.1:15:16	13.2	18	-0.01	5.4E-04	excluded
CHTOP	91133	ES	4.2	3.2	5	-0.08	5.4E-04	excluded
TSFM	22759	ES	8	6	9	-0.01	5.4E-04	excluded
DMKN	49169	ES	8:09:12	7	13	0.13	5.4E-04	included
ZNF185	90411	ES	8	7	9.1	-0.01	5.5E-04	excluded
TMEM107	39129	ES	2:3.2:3.3:3.4:3.5	1	3.7	0.05	5.5E-04	included
MAVS	58609	ES	4	3	5	0.02	5.5E-04	included
ATAD1	12463	ES	7	6	8	0.01	5.5E-04	included
GGCT	79129	ES	5	4	7	0.00	5.5E-04	included
COL1A1	402675	ES	29:30:31:33:34:35:36:<br>37:38:41:42:43:44:45:	28	50	-0.03	5.5E-04	excluded
WDR41	72585	ES	4	2	5	0.02	5.6E-04	included
STEAP3	55101	ES	3	1.1	5.3	-0.10	5.6E-04	excluded
HNMT	55472	ES	3.1:4	2.2	5	-0.04	5.6E-04	excluded
DDX42	42993	ES	2	1.3	3	-0.04	5.6E-04	excluded
B3GALNT2	10355	ES	2	1	3	0.07	5.6E-04	included
TNK2	68209	ES	16	15.2	17	-0.03	5.6E-04	excluded
GABARAPL2	37627	ES	4.1	3	5	0.00	5.6E-04	included
TARS	71682	ES	4	2	6.1	0.01	5.6E-04	included
INADL	3241	ES	46	45	47.1	-0.03	5.6E-04	excluded
ZNF253	48693	ES	2:03	1	4	-0.03	5.6E-04	excluded
TRA2A	78979	ES	3.1	1	4	-0.02	5.6E-04	excluded
RPS5	52443	ES	5	2.3	6	0.04	5.6E-04	included
PDCD2	78500	ES	4	3.1	5	-0.01	5.7E-04	excluded
RNMT	44753	ES	2.2:3.2	1	4	0.01	5.7E-04	included
FLAD1	91163	ES	2.1:2.2:4.1:4.3	1.3	6.1	0.04	5.7E-04	included
CPNE1	59200	ES	1.2:2.2:3	1.1	5	-0.05	5.7E-04	excluded
DCN	23664	ES	2	1	3	-0.05	5.8E-04	excluded
C12orf73	24075	ES	4.1:5	2.4	6	-0.08	5.8E-04	excluded
SKA2	42745	ES	2	1.1	4.1	0.02	5.8E-04	included
KLC1	29474	ES	15:16	13.3	18	-0.03	5.8E-04	excluded
TRA2A	78977	ES	3.1:3.2	1	4	-0.06	5.8E-04	excluded
NDUFS8	17281	ES	3:4.1:5:6	1	7	0.00	5.8E-04	included

AP1B1	61603	ES	24	23	25	0.04	5.8E-04	included
KLF8	89289	ES	7	6	8	-0.02	5.8E-04	excluded
IFT88	25431	ES	2.1:2.2:2.3	1	3	-0.10	5.8E-04	excluded
DDX49	48526	ES	3.1:3.2:4	2.2	5	0.03	5.9E-04	included
VEGFA	76335	ES	7.1:7.2:7.3	6	8.1	0.02	5.9E-04	included
PEX2	84240	ES	03:04.2	2.2	5	0.01	5.9E-04	included
CLIP1	24956	ES	10	8	11.1	-0.02	5.9E-04	excluded
EIF3I	99866	ES	5	4	7	0.06	5.9E-04	included
CCT4	53708	ES	2	1	3	-0.07	5.9E-04	excluded
MTMR10	29794	ES	7	6	9	-0.04	6.0E-04	excluded
BBIP1	13093	ES	5.1:5.2	3	7	0.01	6.0E-04	included
JKAMP	27750	ES	1.2:2.1	1.1	2.2	-0.08	6.0E-04	excluded
ELP2	45250	ES	2	1	3	0.00	6.0E-04	included
TBCB	49356	ES	3	2.3	4	0.00	6.0E-04	excluded
TATDN1	85090	ES	3	2	4.1	0.01	6.1E-04	included
ADAM15	7902	ES	22.1:22.2	20	23	0.01	6.2E-04	included
COL1A2	484902	ES	15:16:17:18:19:20:21:<br>22:23:24:25:26:34:35:	14	38	-0.04	6.2E-04	excluded
GPR75-ASB3	53556	ES	3	2.1	4	-0.03	6.2E-04	excluded
LIMCH1	69123	ES	20	12.1	21	-0.08	6.2E-04	excluded
SPAG9	42494	ES	30	29	31	0.04	6.2E-04	included
BLOC1S6	30456	ES	3	1	7.1	0.04	6.3E-04	included
CCNL1	67381	ES	8	7	9	-0.05	6.3E-04	excluded
SMPD4	55298	ES	11:13	10	14	-0.07	6.3E-04	excluded
SMPD4	95699	ES	21	20	22	0.03	6.3E-04	included
ZDHHC16	12705	ES	07:08.1	6	8.2	0.02	6.4E-04	included
ANAPC11	44210	ES	3.4:6	3.3	7.2	0.03	6.4E-04	included
PHF20L1	85199	ES	6.1:6.2	5	7	-0.05	6.4E-04	excluded
SEC11A	32321	ES	2	1	3	-0.01	6.4E-04	excluded
SETD5	63100	ES	4	3	6	-0.05	6.4E-04	excluded
PICALM	18169	ES	20	18	21	0.05	6.4E-04	included
MT1E	36481	ES	2.1	1	2.3	0.00	6.4E-04	excluded
FAM76A	1342	ES	7	6	8	-0.03	6.4E-04	excluded
DAZAP1	46479	ES	2	1	3	-0.04	6.4E-04	excluded
RABL5	81063	ES	3.2	1	4	0.01	6.5E-04	included
SEC24B	70326	ES	5	4	6	-0.05	6.5E-04	excluded
FLNB	65420	ES	27	26	28	0.06	6.5E-04	included
CTSB	82671	ES	3.1:3.2	1.1	5.3	0.00	6.5E-04	excluded
SH3GLB2	87809	ES	12	11	13	0.00	6.5E-04	included
EEF1D	85446	ES	8.3:10.1:11:12.1:12.2:	8.2	13.2	-0.05	6.5E-04	excluded
ZNF354A	74880	ES	4	3	5	-0.06	6.5E-04	excluded
MATR3	96940	ES	7.2:8	7.1	9	0.07	6.5E-04	included
FAM193A	68579	ES	22	21	23	-0.03	6.5E-04	excluded
FAM104A	43212	ES	3.1:3.2:4	1	5	0.03	6.6E-04	included
NUB1	82371	ES	5	4	6	-0.01	6.6E-04	excluded
RNMT	44754	ES	3.2	1	4	0.02	6.6E-04	included
FAM179B	27392	ES	6	5	7	0.03	6.6E-04	included
USP33	3534	ES	2	1	3	-0.04	6.6E-04	excluded
EMD	90566	ES	2	1.2	3	0.00	6.6E-04	excluded
HNRNPA1	301521	ES	7.1:7.2:8:9.1:9.2	6.2	10	-0.03	6.6E-04	excluded
UBXN11	1260	ES	5:7:8:9	2	10	0.07	6.7E-04	included
KLC1	29473	ES	14.1:15:16	13.3	18	-0.01	6.7E-04	excluded
RCC1	1388	ES	7	6	8	0.05	6.7E-04	included
PPIE	1903	ES	11	9.1	12	-0.04	6.7E-04	excluded
NPRL3	32813	ES	3	2	4	-0.05	6.7E-04	excluded
MFF	57815	ES	5	1	6	0.05	6.7E-04	included
MECP2	90551	ES	2	1	3	0.03	6.8E-04	included
RMND5B	102686	ES	2	1	4.1	0.02	6.8E-04	included
IWS1	55221	ES	6	5	7	0.01	6.8E-04	included
MPPE1	44656	ES	2	1	3.1	-0.01	6.8E-04	excluded
PARK7	526	ES	5	4	6	0.00	6.8E-04	excluded
ATG4A	89854	ES	3	2	4	0.03	6.8E-04	included

SCMH1	2051	ES	7	6	8	0.05	6.8E-04	included
LETMD1	21757	ES	3.2:3.3:4:5:6	2	7	-0.04	6.9E-04	excluded
BCAP29	81362	ES	7	6	8	0.00	6.9E-04	excluded
KLHDC4	37961	ES	5	4.2	6.1	-0.05	6.9E-04	excluded
RHOC	4243	ES	2.3:2.4	1.1	3	0.00	6.9E-04	excluded
AIMP2	78706	ES	2	1.2	4	-0.01	6.9E-04	excluded
PQLC1	46259	ES	6:09	5	10	-0.04	7.0E-04	excluded
RAB5C	41014	ES	2	1	4	-0.04	7.0E-04	excluded
LZTFL1	64417	ES	5	4	6	0.01	7.0E-04	included
VWA9	31212	ES	2.1:2.2:2.3	1.1	3.1	0.01	7.0E-04	included
TNFRSF12A	33347	ES	3.1:3.2	2.1	4	0.06	7.0E-04	included
POLR2E	46388	ES	2.1	1	3	0.00	7.0E-04	included
BABAM1	48257	ES	3	2.4	4	0.01	7.1E-04	included
ARHGAP6	88480	ES	15	14	16.1	-0.01	7.1E-04	excluded
TRNT1	62979	ES	4	2	5	-0.06	7.2E-04	excluded
EPS15L1	48161	ES	23.1	21	24	-0.04	7.2E-04	excluded
ANKRD65	167	ES	2	1.4	3	0.14	7.3E-04	included
PIGK	3519	ES	3:04	2	5	0.00	7.3E-04	included
MAFF	62218	ES	4	1.1	5	0.01	7.3E-04	included
SPTAN1	87772	ES	38	36.1	39	-0.04	7.3E-04	excluded
CKLF	36735	ES	2	1	3.2	0.03	7.3E-04	included
MPZL1	8873	ES	3:04:05	2	6	-0.01	7.3E-04	excluded
MRPL55	10164	ES	1.2:2.5	1.1	2.9	0.01	7.4E-04	included
PQLC1	46257	ES	6:07:09	5	10	-0.05	7.4E-04	excluded
VEGFA	76329	ES	7.1:8.1:8.2	6	9.1	-0.03	7.4E-04	excluded
FDPS	8072	ES	02:03.1	1.1	3.2	-0.02	7.4E-04	excluded
PGAP3	40670	ES	4:5:6:7	3	8	0.00	7.5E-04	excluded
ACOT9	88694	ES	6	5.1	7.1	0.06	7.6E-04	included
DMKN	49167	ES	8:11:12	7	13	0.07	7.6E-04	included
ZNF485	11338	ES	3:04	2	5	-0.03	7.6E-04	excluded
CNOT2	23380	ES	3.1:3.2	2	6	0.02	7.7E-04	included
EED	18181	ES	8:09:10	7	11.2	-0.07	7.7E-04	excluded
AFMID	43850	ES	5	2	6	0.01	7.7E-04	included
BOD1	74586	ES	2	1	4	-0.08	7.8E-04	excluded
C1orf159	22	ES	5.1:5.2:5.3	4.4	5.5	-0.03	7.8E-04	excluded
HMGN1	107413	ES	8.2	7	9	0.08	7.8E-04	included
SDHAF2	16233	ES	03:04.1	1	6	0.03	7.8E-04	included
BAI2	1502	ES	8	7	9	0.07	7.8E-04	included
SKA2	42731	ES	3	2	4.1	-0.02	7.8E-04	excluded
PMM1	62439	ES	2	1	3	-0.03	7.8E-04	excluded
DCAF8	8442	ES	15	14	16	0.00	7.9E-04	included
SH3BP2	68600	ES	8.1	7	9	-0.03	7.9E-04	excluded
NBPF11	7342	ES	05:08.1	4	8.3	0.08	7.9E-04	included
LRRCC1	84321	ES	5:06	4	7	0.01	8.0E-04	included
SNX21	59618	ES	3	2	4.1	0.05	8.0E-04	included
FAM189B	8050	ES	2:03:04	1	5	-0.04	8.0E-04	excluded
PRKAB1	24709	ES	3	1.3	4	-0.04	8.1E-04	excluded
HNRNPC	26557	ES	2.2:2.4	1	3.2	-0.02	8.1E-04	excluded
ZDHHC4	78749	ES	1.3	1.1	2.1	0.10	8.1E-04	included
C16orf45	34159	ES	8	7	10	0.00	8.1E-04	excluded
MACF1	1888	ES	43.2:44:45:46:47	42	48	-0.10	8.2E-04	excluded
MPI	31784	ES	2.2:2.3	1.1	3	0.01	8.2E-04	included
GPS1	44285	ES	1.4:1.5:1.6	1.1	2.3	0.01	8.2E-04	included
ALKBH3	15467	ES	7	6	9	0.02	8.2E-04	included
MRPL55	10111	ES	2.2:2.3:2.4:2.5:2.6:2.8	1.2	2.9	0.03	8.2E-04	included
PAX8	102557	ES	9.2	8	10	-0.04	8.2E-04	excluded
SEC14L2	61748	ES	2	1	3	0.02	8.3E-04	included
GRB10	79720	ES	10	5.2	11	0.12	8.3E-04	included
CCDC24	2530	ES	4.1:4.2:5	3.2	6.2	0.03	8.3E-04	included
ELN	80047	ES	23	21	24.2	-0.05	8.4E-04	excluded
ALDH3A2	39753	ES	11	10	12	0.02	8.4E-04	included
COG4	37401	ES	9	8	10	-0.05	8.4E-04	excluded

UVRAG	17885	ES	20	19.2	21	-0.03	8.4E-04	excluded
PLA2G10	34100	ES	4	3	5	-0.10	8.5E-04	excluded
EP400NL	25252	ES	4	3.2	5.2	-0.08	8.5E-04	excluded
HMGN1	60610	ES	07:08.2	6.2	9	0.09	8.6E-04	included
COL1A1	402674	ES	29:30:31:32:33:34:35:<br>36:37:38:41:42:43:44:	28	50	-0.04	8.6E-04	excluded
CLK4	74877	ES	3	2.1	4	-0.06	8.6E-04	excluded
SSFA2	56442	ES	17	16	18	0.02	8.6E-04	included
TMEM106B	78806	ES	2	1	3	-0.06	8.6E-04	excluded
SRSF11	3392	ES	5:6.1:6.2:6.3	4.2	6.5	-0.07	8.6E-04	excluded
PGBD2	10572	ES	03:04.1	2	4.2	0.10	8.6E-04	included
DTNB	52862	ES	21	20	22	-0.03	8.7E-04	excluded
NEDD4L	45660	ES	18	17	19	0.03	8.7E-04	included
TXNL4A	46286	ES	7.2	3	9	0.00	8.7E-04	excluded
HNRNPM	94942	ES	13	12	14	0.01	8.7E-04	included
ANKRD10	26273	ES	5	3	6.1	-0.07	8.8E-04	excluded
MBD1	45515	ES	18.1	17	18.4	0.04	8.8E-04	included
DMKN	49185	ES	7:8:11:12	6.4	13	0.08	8.8E-04	included
FHL2	54830	ES	3.2	2.3	5.1	-0.06	8.8E-04	excluded
ATF3	9738	ES	5.1	4	5.3	-0.01	8.8E-04	excluded
FAM208A	65357	ES	17	16	18.2	-0.03	8.8E-04	excluded
UTP11L	1854	ES	6	5	7	0.00	8.9E-04	included
WDR27	78474	ES	9	8	10	-0.09	8.9E-04	excluded
CCL14	40385	ES	5.4	5.2	5.6	-0.01	8.9E-04	excluded
LDLRAD3	15419	ES	5	4	6	-0.06	8.9E-04	excluded
RPS3	17853	ES	2.1	1	3.1	0.05	8.9E-04	included
MLPH	58115	ES	10	9	11	-0.03	9.0E-04	excluded
CCP110	34316	ES	15	14	16	0.06	9.0E-04	included
PDE6D	57935	ES	4	3	5	0.01	9.0E-04	included
RAPGEF3	21355	ES	5:6:7:8	4	9	0.00	9.1E-04	excluded
FBXO4	71889	ES	3	2	4	-0.05	9.1E-04	excluded
PMS2	97368	ES	10	9	11.1	-0.04	9.1E-04	excluded
SEC16A	88173	ES	25	24	26	0.04	9.2E-04	included
ARMC8	66964	ES	3	2.2	4	0.05	9.2E-04	included
EPB41L1	59272	ES	22.2	21	23	-0.04	9.2E-04	excluded
ZDHHC4	78754	ES	1.3	1.1	2.2	0.08	9.2E-04	included
PTPN18	55342	ES	4:05:06	3	7	0.04	9.2E-04	included
SPOP	42307	ES	7	6	8	-0.01	9.3E-04	excluded
RGS14	74759	ES	14	13	15	0.03	9.3E-04	included
ZNF786	82165	ES	2:03	1	4	-0.09	9.3E-04	excluded
LTBP4	49936	ES	25	24	28	-0.03	9.3E-04	excluded
MYNN	67581	ES	2	1	3	0.05	9.3E-04	included
CKLF	36729	ES	3.2	2	4	0.02	9.4E-04	included
RAB40C	32913	ES	6	5	7	-0.04	9.4E-04	excluded
NDUFV2	44591	ES	1.3	1.1	4	0.00	9.4E-04	excluded
ARMC10	81156	ES	8:09	6	10	0.00	9.4E-04	excluded
NASP	2729	ES	5:6:7.2:8:9	3	10	0.05	9.4E-04	included
SHMT2	22548	ES	5	4	6.1	0.02	9.5E-04	included
HNRNPC	26551	ES	2.2:2.4:2.5:2.6	1	3.2	0.00	9.5E-04	excluded
GBA	8042	ES	5	4	6	0.01	9.5E-04	included
PLEKHH1	28067	ES	29:30:00	28	31	0.01	9.5E-04	included
MAGOHB	20478	ES	1.2:2.1:2.2	1.1	3	0.06	9.6E-04	included
CHORDC1	18269	ES	9.2	8.3	9.4	-0.04	9.6E-04	excluded
CDPF1	62696	ES	4	3.2	5	-0.03	9.6E-04	excluded
SFTA3	27273	ES	4.2:5	2	6	0.03	9.6E-04	included
SFTA3	27277	ES	03:04.2	2	6	0.02	9.7E-04	included
PMPCB	81180	ES	7	6	8	0.01	9.7E-04	included
FKTN	87134	ES	10:11:12	9	13.1	0.03	9.7E-04	included
RNH1	13673	ES	3	2	4.2	0.03	9.8E-04	included
EXOSC9	70505	ES	10.3	10.1	10.5	0.03	9.8E-04	included
ILK	14167	ES	5.5	5.3	6	0.00	9.8E-04	included
MPPE1	44651	ES	9.1:9.2	8	10	0.04	9.8E-04	included

SORBS2	71380	ES	21:22	19	23	0.01	9.8E-04	included
HMGN1	60625	ES	4	3	5	0.00	9.9E-04	included
MRRF	87470	ES	5:06	4	7	-0.06	9.9E-04	excluded
FAM86B1	82702	ES	4:5:6:7.1:7.3	3.2	8.1	-0.08	9.9E-04	excluded
NUP85	43388	ES	2	1	3	0.01	9.9E-04	included
FHL2	54829	ES	3.2:4	2.3	5.1	-0.07	9.9E-04	excluded
PLAT	83578	ES	5	4	6	0.01	9.9E-04	included
MFF	57811	ES	8	7	11	0.02	1.0E-03	included
RHOC	4237	ES	1.2:2.2	1.1	2.3	-0.04	1.0E-03	excluded
HNRNPA1	212647	ES	3:4:5:6.1:9.2:10:11.2	2	11.3	-0.01	1.0E-03	excluded
DAGLB	78732	ES	4:05	3	6	-0.01	1.0E-03	excluded
IL32	33430	ES	1.3:1.4:1.5:1.6:1.8	1.1	1.9	0.01	1.0E-03	included
RPAP3	21339	ES	12	11	13	-0.01	1.0E-03	excluded
PRCC	8324	ES	4	3	5	0.00	1.0E-03	excluded
ARPC4	63183	ES	5:06	4	7	0.07	1.0E-03	included
VEGFA	76346	ES	6	5	9.1	0.01	1.0E-03	included
MFSD12	46699	ES	14	12	15	-0.07	1.0E-03	excluded
ANKS3	33825	ES	5.1:5.2	3	6	-0.03	1.0E-03	excluded
LRP8	3059	ES	17	16	18	0.02	1.0E-03	included
LYRM1	34421	ES	7	2.2	8.1	0.05	1.0E-03	included
BOLA1	7408	ES	2.2:3.1	2.1	3.2	-0.05	1.0E-03	excluded
PQLC3	52674	ES	7	5	8	-0.04	1.0E-03	excluded
ANXA2	30952	ES	3	2	4.2	-0.01	1.0E-03	excluded
RPE	57252	ES	3	1.2	4	0.01	1.0E-03	included
SYF2	1139	ES	3	2	4	0.00	1.0E-03	included
ITCH	59022	ES	7	6	8	-0.08	1.0E-03	excluded
GLB1	63839	ES	3	1	4.2	-0.02	1.1E-03	excluded
CFI	70342	ES	7	6	8	-0.02	1.1E-03	excluded
MUTYH	2601	ES	8:09	7	10	0.01	1.1E-03	included
SDHAF2	16235	ES	2:03	1	6	0.00	1.1E-03	included
DGUOK	54016	ES	2	1	4	-0.01	1.1E-03	excluded
MRPL45	40572	ES	4	3	5	-0.05	1.1E-03	excluded
ADAM9	83484	ES	19	18	20	0.00	1.1E-03	included
UBL5	47434	ES	2.2:3	2.1	4	0.00	1.1E-03	excluded
YAF2	21148	ES	3.2:5.2:6:7	2	9.1	0.00	1.1E-03	excluded
TACC2	13341	ES	14:15.1	13	15.2	0.07	1.1E-03	included
RPL7L1	76196	ES	2	1	3	0.03	1.1E-03	included
ECHDC2	319385	ES	12	11	14	0.04	1.1E-03	included
HNRNPH3	11933	ES	3	2	4.1	0.01	1.1E-03	included
HNRNPA1	485364	ES	3:4:5:6.1:6.2:10:11.2	2	11.3	0.00	1.1E-03	excluded
AKAP1	42609	ES	7	6	8	-0.02	1.1E-03	excluded
TPT1	25800	ES	1.2:1.4	1.1	2	0.01	1.1E-03	included
RPS6KB2	17208	ES	2	1	3	0.01	1.1E-03	included
ZNF140	25327	ES	7	5.3	8.1	-0.05	1.1E-03	excluded
RBM25	28257	ES	8	7	9	-0.02	1.1E-03	excluded
REPIN1	82238	ES	4.1:4.2	3.2	5.3	-0.08	1.1E-03	excluded
GGPS1	10348	ES	5.1:5.2	3	6	0.01	1.1E-03	included
TACC2	13348	ES	04:05.1	3	8	0.04	1.1E-03	included
STYXL1	80149	ES	8	7	10	0.01	1.1E-03	included
GEMIN8	88533	ES	2	1	3	0.04	1.1E-03	included
NID2	27539	ES	12	11	13	-0.03	1.1E-03	excluded
EED	18180	ES	10	9	11.2	-0.03	1.2E-03	excluded
APLP2	19479	ES	9	8	10	0.04	1.2E-03	included
CYTH1	43891	ES	12	11.1	13.2	-0.04	1.2E-03	excluded
GBGT1	88022	ES	5.2	3	6	0.03	1.2E-03	included
TRMT11	77439	ES	8	7	9.1	-0.05	1.2E-03	excluded
PHYH	100582	ES	7.1:7.2:8	6	9	-0.06	1.2E-03	excluded
EAF2	66410	ES	4	3	5	0.05	1.2E-03	included
SH3GLB1	3685	ES	6:07	5	8	-0.01	1.2E-03	excluded
PIP5K1A	7576	ES	13	12	14	0.00	1.2E-03	included
C16orf93	94146	ES	4.1:4.3:5	3.3	7	0.06	1.2E-03	included
DMKN	49141	ES	21	20.2	22	0.05	1.2E-03	included

SPNS1	35904	ES	7	6	8	0.01	1.2E-03	included
RHEB	82381	ES	1.2:3	1.1	4	-0.06	1.2E-03	excluded
RPGR	88808	ES	14.1:14.3	13	16	0.04	1.2E-03	included
TRIQK	84499	ES	4:05	2.1	6.1	0.07	1.2E-03	included
SEPT6	89975	ES	12:13.1	11.1	13.2	0.05	1.2E-03	included
TATDN1	85082	ES	10	9.1	11	-0.02	1.2E-03	excluded
MAN2C1	124833	ES	14	13	15	0.01	1.2E-03	included
C14orf2	29530	ES	3:05:06	2	7.1	0.01	1.2E-03	included
SNRNP200	563710	ES	44:45:00	43	46.1	-0.03	1.2E-03	excluded
LONRF1	82753	ES	7	6	8	-0.02	1.2E-03	excluded
RAB1A	53795	ES	5	4	6	0.00	1.2E-03	included
PLEKHA5	20649	ES	27	26	28	-0.02	1.2E-03	excluded
DMD	88774	ES	76:77:78	74	79	-0.03	1.2E-03	excluded
STAC3	22562	ES	2:03:04	1	5	0.03	1.2E-03	included
MYO19	40490	ES	8	7	9	0.02	1.2E-03	included
KIF13A	75457	ES	40	39	41.1	-0.03	1.2E-03	excluded
PXN	24748	ES	13	9	14.1	-0.08	1.2E-03	excluded
SS18	44912	ES	15	14	16	0.04	1.2E-03	included
SPATA20	42435	ES	4.2	3	6	-0.04	1.2E-03	excluded
MPPE1	44647	ES	12	11	13	-0.04	1.2E-03	excluded
TMEM64	84417	ES	6	5	7	0.02	1.2E-03	included
ESRP1	84566	ES	15	14	16	-0.03	1.3E-03	excluded
BBS1	17053	ES	4	3	5.1	0.00	1.3E-03	included
AGAP1	58088	ES	14	13	15	0.05	1.3E-03	included
NSFL1C	58501	ES	5.2:7.2	4	7.3	0.07	1.3E-03	included
XRN2	58835	ES	3	1	4	0.01	1.3E-03	included
GSTA4	76485	ES	4	3.2	5.1	0.01	1.3E-03	included
PTP4A2	1532	ES	5	3	6	0.00	1.3E-03	excluded
ANGEL2	9777	ES	8	7	9	0.02	1.3E-03	included
GMFG	49792	ES	3.2	2	4	0.00	1.3E-03	included
DGUOK	54015	ES	3	1	4	0.03	1.3E-03	included
SCAP	64519	ES	4:05:06	3	7	-0.01	1.3E-03	excluded
YTHDC2	73010	ES	3:04	2	5	0.05	1.3E-03	included
C20orf24	59299	ES	3	2	4.1	-0.02	1.3E-03	excluded
NFYC	2020	ES	18	17	19	0.01	1.3E-03	included
DAPK2	31076	ES	13.2:13.3:13.4:14:15.1	13.1	15.3	-0.02	1.3E-03	excluded
EIF3G	47465	ES	5	4	6	-0.02	1.3E-03	excluded
PEMT	39491	ES	9.1:9.2	8	10	0.00	1.3E-03	included
XAF1	38808	ES	4.1:4.2:4.3	2.1	6	-0.07	1.3E-03	excluded
EWSR1	61586	ES	5	4	6	0.00	1.3E-03	excluded
MLLT1	47022	ES	5	4	6	0.02	1.3E-03	included
SS18	44959	ES	4:5:8.1:8.2	3	9	0.00	1.3E-03	excluded
PCBP2	22049	ES	15:16.1	14.1	16.2	0.00	1.3E-03	included
GPHN	28034	ES	5	3	6	0.01	1.3E-03	included
HDHD2	45441	ES	3	1	4.1	-0.05	1.3E-03	excluded
FRG1	71413	ES	5	4	6	0.01	1.3E-03	included
SRSF11	3394	ES	5:6.1:6.3	4.2	6.5	-0.02	1.3E-03	excluded
LMO3	20616	ES	10:11.1	9.2	11.2	-0.05	1.3E-03	excluded
RABL2B	62925	ES	2.1:2.2	1	3.1	0.06	1.3E-03	included
SUCO	9012	ES	4	3	5	0.07	1.3E-03	included
CTSB	82670	ES	4	1.1	5.3	0.00	1.3E-03	excluded
SH3YL1	52504	ES	7	6	8	-0.05	1.3E-03	excluded
ST7L	4218	ES	4.1:4.2	2.2	5	0.03	1.3E-03	included
SP110	57867	ES	11.1:11.2	10	12	0.01	1.3E-03	included
TMEM159	34426	ES	6	5	7	-0.04	1.3E-03	excluded
CHMP7	83073	ES	4	3	5	-0.06	1.3E-03	excluded
MAP4K3	53330	ES	16	15	18	0.05	1.3E-03	included
SPIDR	83780	ES	9.1	8	10	-0.01	1.3E-03	excluded
SUMF2	79812	ES	3:5.1:5.2	2	6	0.02	1.3E-03	included
MRPL55	10146	ES	1.2:2.2:2.8	1.1	2.9	0.07	1.3E-03	included
BCAT2	50814	ES	3:04:05	1	6	-0.06	1.4E-03	excluded
PAPLN	28283	ES	8	7	9	0.02	1.4E-03	included

USP10	37864	ES	3	1	4	-0.05	1.4E-03	excluded
TSC22D2	67269	ES	2	1	3	-0.04	1.4E-03	excluded
SLC25A45	16839	ES	4.3:5.1:5.2	4.2	6.2	-0.09	1.4E-03	excluded
HM13	58891	ES	12.1	11	13	0.00	1.4E-03	excluded
GGT2	100019	ES	5	4	6	-0.05	1.4E-03	excluded
KLHDC3	76212	ES	4	3	5	0.00	1.4E-03	included
BMP1	82990	ES	17.1:17.2:18.1	16	18.2	-0.04	1.4E-03	excluded
GORASP1	64157	ES	03:04.2	1	5	-0.01	1.4E-03	excluded
PLD3	49896	ES	1.2:3	1.1	5.2	0.02	1.4E-03	included
NAV2	14700	ES	23:24	22	25	0.06	1.4E-03	included
CSNK1D	44308	ES	11	10	12	0.01	1.4E-03	included
IL32	33377	ES	2.4	2.1	3	-0.01	1.4E-03	excluded
UBR4	881	ES	94	93	95	0.00	1.4E-03	excluded
FDXR	43316	ES	12.4	12.2	13	0.00	1.4E-03	excluded
TXN2	62050	ES	5	4	6	0.00	1.4E-03	excluded
ZNF655	80690	ES	04:05.3	3.2	7	0.05	1.4E-03	included
RNF7	67075	ES	3	1.2	4	0.00	1.4E-03	excluded
CRTC1	48503	ES	12:13:14	11	15	0.01	1.4E-03	included
EPS8L1	52015	ES	4:05	3.2	6	0.01	1.4E-03	included
PSMD13	13634	ES	2	1	3.1	0.00	1.4E-03	included
MYO9A	31489	ES	27	26	28	-0.02	1.4E-03	excluded
MYCBPAP	42406	ES	20:21	19	22	0.06	1.4E-03	included
LRRFIP2	63959	ES	23	22	25	0.00	1.4E-03	excluded
C16orf13	32921	ES	2:03	1	5	-0.01	1.4E-03	excluded
C3orf52	66087	ES	4:05	3	6	-0.01	1.4E-03	excluded
HNRNPD	69700	ES	7	6	8.1	-0.02	1.4E-03	excluded
TMEM185A	90321	ES	2:3.1:3.2:6.1	1	7	-0.01	1.4E-03	excluded
WARS	29289	ES	4	2.3	7	0.01	1.4E-03	included
DSTN	58733	ES	5	4	6	0.00	1.4E-03	excluded
HOPX	69381	ES	1.2:3.2	1.1	4.6	0.04	1.4E-03	included
CAPN3	30153	ES	24	23	25	0.03	1.4E-03	included
KAT7	42322	ES	7	6	8	0.02	1.4E-03	included
HEXA	31545	ES	11:12	10	13.1	0.00	1.4E-03	included
PEX5	20089	ES	9	8	10	0.04	1.4E-03	included
GMFG	49794	ES	3.2:4	2	5	0.00	1.4E-03	included
NDUFB5	67705	ES	3.2:4.1:4.2	1	5	0.01	1.4E-03	included
RHOT2	32942	ES	5	4.2	6	-0.01	1.4E-03	excluded
KLC1	29486	ES	15	13.2	18	-0.04	1.4E-03	excluded
ERLEC1	53558	ES	12	11	13	0.00	1.4E-03	excluded
LCN10	88197	ES	4.2	3	5	0.02	1.4E-03	included
QPCT	53228	ES	2	1	3	0.00	1.5E-03	excluded
TECR	564146	ES	2:04	1	5.3	0.06	1.5E-03	included
PARD3	11216	ES	13	12	14	-0.04	1.5E-03	excluded
SIRT2	49714	ES	02:03.1	1	3.2	-0.05	1.5E-03	excluded
TCOF1	74072	ES	7	6	8	0.03	1.5E-03	included
DCUN1D5	18472	ES	4	3	5	-0.03	1.5E-03	excluded
KLC1	29481	ES	13.3:15:16	13.2	18	-0.04	1.5E-03	excluded
PTPRF	2189	ES	23	22	24	0.00	1.5E-03	included
MAP4K4	54757	ES	18	17	19	-0.02	1.5E-03	excluded
CMC2	37706	ES	10	9	12	-0.02	1.5E-03	excluded
GRIPAP1	89052	ES	18	17	19	0.01	1.5E-03	included
MRPL55	10142	ES	1.2:2.2:2.5:2.6:2.8	1.1	2.9	0.03	1.5E-03	included
MKNK1	2809	ES	11	10	12	-0.04	1.5E-03	excluded
STXBP6	27040	ES	5	1.1	6	-0.02	1.5E-03	excluded
MTMR10	29793	ES	7:08	6	9	-0.09	1.5E-03	excluded
SRCAP	36172	ES	21	20	22	0.01	1.5E-03	included
PLXNC1	23724	ES	28	27	29	0.01	1.5E-03	included
THEM4	7677	ES	3	2.1	4	-0.03	1.5E-03	excluded
NSL1	9745	ES	6	4	7	-0.01	1.5E-03	excluded
WDR53	68252	ES	2.1:2.2	1	3	0.05	1.5E-03	included
CCDC24	2527	ES	4.1:4.2:5:6.1	3.2	6.2	0.06	1.5E-03	included
CMC2	37705	ES	10:11	9	12	0.00	1.6E-03	excluded

SMUG1	22130	ES	2.2:2.3	1.1	3	-0.09	1.6E-03	excluded
POLDIP3	62524	ES	9	8	10.1	0.01	1.6E-03	included
FLOT2	40016	ES	3	2	5	-0.02	1.6E-03	excluded
USF2	49096	ES	04:05.1	2.3	5.2	0.00	1.6E-03	excluded
RPAIN	38683	ES	5	4	7	-0.04	1.6E-03	excluded
C16orf13	32919	ES	2:03:04	1	5	0.00	1.6E-03	excluded
GPATCH1	48926	ES	9	8	10	-0.01	1.6E-03	excluded
TTLL5	28526	ES	17	16	18	0.06	1.6E-03	included
MLH1	63934	ES	7	6	8	0.01	1.6E-03	included
EEF1D	85449	ES	8.3:9:10.1:11:12.2:13.	8.2	13.2	-0.02	1.6E-03	excluded
TMEM14B	75316	ES	4	3	5.1	0.00	1.6E-03	included
B3GALNT1	67504	ES	6	5	7	-0.04	1.6E-03	excluded
BRD8	96935	ES	16	15	17	0.01	1.6E-03	included
FBXO4	71888	ES	7	6.1	8	0.02	1.6E-03	included
CBS	60774	ES	19	18	20.1	-0.01	1.6E-03	excluded
ATXN7	65518	ES	15	14	16	-0.05	1.6E-03	excluded
HNRNPA1	264682	ES	3:4:5:6.1:6.2:8:9.1:9.2 :10:11.2	2	11.3	0.00	1.6E-03	excluded
RPS3A	96809	ES	5.2	4.1	6	0.06	1.6E-03	included
EIF4A1	38993	ES	2	1	3	0.00	1.6E-03	excluded
ZSWIM7	94447	ES	7.3:7.4:7.5	7.1	7.7	-0.06	1.6E-03	excluded
DMTF1	80300	ES	15	14	16	-0.01	1.6E-03	excluded
UBXN11	1257	ES	5:6:7:8:9	2	10	0.07	1.6E-03	included
TXNDC9	54706	ES	4	3	5.1	-0.01	1.6E-03	excluded
HGSNAT	83764	ES	6	5	7	-0.02	1.6E-03	excluded
PPP1CB	53077	ES	1.4	1.1	2	0.01	1.6E-03	included
TMEM129	68504	ES	3	2	4	-0.01	1.6E-03	excluded
MKRN1	82002	ES	7:08	6	9.1	0.00	1.6E-03	included
RRNAD1	8312	ES	3	2.3	4	0.00	1.6E-03	included
IL17RC	63276	ES	4	3.3	5.1	0.03	1.6E-03	included
SRCAP	36171	ES	23	22	24	-0.03	1.6E-03	excluded
PPCDC	31835	ES	2	1	3.2	0.03	1.7E-03	included
DCAF11	26847	ES	1.2:2.2	1.1	2.4	0.05	1.7E-03	included
YIF1A	17009	ES	5	4	6	0.00	1.7E-03	excluded
DCTN2	22641	ES	7	2	8	-0.01	1.7E-03	excluded
TRMT61B	53080	ES	5	4	6	0.01	1.7E-03	included
TMEM43	63521	ES	2	1	3	-0.04	1.7E-03	excluded
TPM1	30988	ES	9	8	10	-0.01	1.7E-03	excluded
COL1A1	132060	ES	47	46	48	0.01	1.7E-03	included
SLCO3A1	32548	ES	10.1	9	11	0.01	1.7E-03	included
BCL2L13	96058	ES	7	6	8.1	0.02	1.7E-03	included
KLHL7	78959	ES	3	1	4	-0.04	1.7E-03	excluded
HIATL1	86935	ES	10	9	11	-0.01	1.7E-03	excluded
RMND5B	102685	ES	2:03	1	4.1	0.04	1.7E-03	included
ATP5H	43344	ES	5	3	6	0.09	1.7E-03	included
HMGN1	60611	ES	8.2	6.2	9	0.08	1.7E-03	included
CLN3	35745	ES	4	3.4	5	0.01	1.7E-03	included
C11orf49	15623	ES	7	3	10	0.01	1.7E-03	included
TMEM205	47675	ES	2.2:2.5	2.1	2.6	0.03	1.7E-03	included
SKA2	42753	ES	1.2:2:4.1	1.1	5	0.06	1.7E-03	included
PTGR1	87222	ES	6	5	7	0.00	1.7E-03	excluded
RIC3	14236	ES	2	1	3.1	0.07	1.7E-03	included
ITGB1BP1	52620	ES	5	4	6.1	0.01	1.7E-03	included
ORMDL2	22258	ES	3.1:3.2	1	4	0.00	1.7E-03	included
GLMN	3758	ES	12	11	13	-0.02	1.7E-03	excluded
FN3KRP	44418	ES	5	4	6	-0.02	1.7E-03	excluded
CNP	40964	ES	2.1:2.2	1	3	0.00	1.7E-03	included
TOP1MT	85418	ES	4	3	5	-0.06	1.7E-03	excluded
STXBP6	27033	ES	7	6	8	-0.01	1.7E-03	excluded
LMNA	8184	ES	17.1	16	18.1	0.00	1.8E-03	excluded
CYB561D1	4026	ES	2	1.2	3	-0.07	1.8E-03	excluded
BBS9	79224	ES	17:18	15	19.2	0.01	1.8E-03	included

MIF4GD	43425	ES	3.1:3.2	2	4	0.03	1.8E-03	included
COMMD1	53712	ES	2	1	3	-0.01	1.8E-03	excluded
LDLRAD4	44741	ES	8	6	9	-0.05	1.8E-03	excluded
TCF3	94886	ES	17.3:18.1	17.1	18.2	-0.06	1.8E-03	excluded
RAB5A	63691	ES	3	2	4.2	-0.01	1.8E-03	excluded
KLHL24	67806	ES	2	1	3	-0.09	1.8E-03	excluded
ARGLU1	26233	ES	4	3	5	-0.06	1.8E-03	excluded
WBP2	43522	ES	3	2.1	4	-0.02	1.8E-03	excluded
FAM86C1	17439	ES	4	2	5.1	-0.06	1.8E-03	excluded
C16orf45	34160	ES	5	4	6	-0.01	1.8E-03	excluded
FLAD1	91158	ES	2.2:3:4.1:4.2:4.3	1.3	6.1	0.08	1.8E-03	included
NFIX	47907	ES	10	9	11	-0.02	1.8E-03	excluded
SRSF11	3389	ES	5:6.1:6.3:6.4	4.2	6.5	-0.02	1.8E-03	excluded
DDX17	62241	ES	11.2:12.2:12.3	11.1	12.4	-0.04	1.8E-03	excluded
VMP1	42797	ES	6	5	7	0.00	1.8E-03	included
NAP1L4	13938	ES	16	15	17	-0.02	1.8E-03	excluded
CHID1	13816	ES	6.1:6.2	5.2	7	0.00	1.8E-03	included
GIGYF2	58026	ES	2	1	3.2	-0.04	1.9E-03	excluded
SERINC4	115972	ES	5:6:9:10	4	11	0.02	1.9E-03	included
ZNF207	40208	ES	02:03.1	1	3.2	0.00	1.9E-03	excluded
SNF8	42249	ES	6	5	7.1	-0.01	1.9E-03	excluded
ADAL	30235	ES	10	9	11.1	0.03	1.9E-03	included
ZBTB7B	7878	ES	5	2.2	6.2	-0.06	1.9E-03	excluded
MUC1	7964	ES	4.1:4.2:4.3	3.4	5	0.00	1.9E-03	excluded
MAP2K3	39818	ES	3:04	1	5	-0.02	1.9E-03	excluded
CASP1	18522	ES	8	7	9.2	0.00	1.9E-03	excluded
TIE1	2162	ES	7	6	9	-0.03	1.9E-03	excluded
SLC25A25	87697	ES	7	6	8	-0.01	1.9E-03	excluded
HMOX2	33747	ES	7	2.2	8	0.00	1.9E-03	excluded
PAF1	49812	ES	4	3	5	0.00	1.9E-03	included
ZYX	82117	ES	5	4	6	0.00	1.9E-03	included
CMC2	37738	ES	3	2	5	-0.01	1.9E-03	excluded
DMKN	49158	ES	10:11	8	12	0.08	1.9E-03	included
TMED3	32155	ES	3	2	4	0.00	1.9E-03	excluded
DDX55	25093	ES	2.2:3:4	1	5.1	0.02	2.0E-03	included
ZSWIM7	94445	ES	7.3:7.5:7.6	7.1	7.7	-0.05	2.0E-03	excluded
AP4M1	80892	ES	3.2	2.1	4	-0.03	2.0E-03	excluded
CDK5	82324	ES	6	5	7	0.01	2.0E-03	included
NDUFB3	56781	ES	2.1:2.2	1	3	0.00	2.0E-03	excluded
IL15	70674	ES	6	5	7	-0.04	2.0E-03	excluded
NT5C3A	79217	ES	3	1	5	-0.05	2.0E-03	excluded
USP8	30585	ES	13	12	14	-0.04	2.0E-03	excluded
FAM86B1	82690	ES	6:7.1:7.3	4	8.1	-0.06	2.0E-03	excluded
MFF	57798	ES	9:10	8	11	0.03	2.0E-03	included
TBC1D10A	61719	ES	11	10	12	-0.02	2.0E-03	excluded
MBD1	45523	ES	10	9	11	-0.01	2.0E-03	excluded
ACTR6	23910	ES	5	4	6	-0.04	2.0E-03	excluded
CYLD	36396	ES	3	2.3	4.2	0.05	2.0E-03	included
METTL23	43647	ES	2	1.1	3	0.04	2.0E-03	included
NEDD4L	45672	ES	4:8:9:10:11:12:13:14:<br>15:16:17:18:19:20:21:<br>22:23:24:26:27:28:29:	1	35	0.03	2.0E-03	included
TECR	564145	ES	04:05.2	1	5.3	0.03	2.0E-03	included
MFF	57804	ES	9	7	10	-0.01	2.0E-03	excluded
HAUS2	30187	ES	04:05.1	3	6	0.01	2.0E-03	included
CD97	47968	ES	5:06	4	7	0.03	2.0E-03	included
BCL2L13	96057	ES	8.1	6	9	-0.04	2.0E-03	excluded
NDUFS4	72012	ES	2	1	3	0.00	2.0E-03	excluded
SUPT6H	39943	ES	2	1	3.1	-0.06	2.0E-03	excluded
PDCD6	71429	ES	2	1	4	0.00	2.0E-03	included
SEPT10	54908	ES	12.1	11.1	13	-0.01	2.0E-03	excluded
RPL30	84639	ES	2.1:2.2	1.3	3.1	-0.01	2.0E-03	excluded

NOSIP	50977	ES	2	1	3	-0.01	2.0E-03	excluded
GGT1	61441	ES	5:6.2:7.1:7.2	4	7.3	0.05	2.1E-03	included
UBXN2B	83925	ES	6	5	7	-0.07	2.1E-03	excluded
CCDC91	20919	ES	10	9	11	-0.03	2.1E-03	excluded
LTBP4	385946	ES	31:32:33:34	28	35	0.06	2.1E-03	included
ACOT9	88696	ES	5.1	4	7.1	0.01	2.1E-03	included
LSM14B	95990	ES	6.1:6.2	5	7	-0.02	2.1E-03	excluded
MEF2BNB	48604	ES	5.2:6.1	5.1	6.2	-0.02	2.1E-03	excluded
RPLP0	24730	ES	5.2:5.3	4.2	6.2	0.00	2.1E-03	included
SDC4	59523	ES	2	1	3	0.00	2.1E-03	included
CARF	56983	ES	4.1:4.2:5.1:5.2:6.1:6.2	3.1	7	0.09	2.1E-03	included
MFF	57807	ES	9:10	7	11	0.03	2.1E-03	included
PDE8B	72568	ES	8:09:10	7	11	-0.01	2.1E-03	excluded
DIAPH1	73806	ES	2	1	3	0.07	2.1E-03	included
STRBP	87506	ES	2	1	3	-0.06	2.1E-03	excluded
SREBF1	39507	ES	2	1	3.2	0.05	2.1E-03	included
PTTG1IP	60840	ES	5:06	4	7	0.00	2.1E-03	included
FAM49A	52726	ES	3	2	4	-0.01	2.1E-03	excluded
SMPD4	55292	ES	13	11	14	-0.05	2.2E-03	excluded
TAZ	90590	ES	5:06:07	4	8.1	-0.02	2.2E-03	excluded
MTHFD1	27878	ES	27	26	28	0.00	2.2E-03	included
ADAM15	7896	ES	22.1	21.2	23	0.04	2.2E-03	included
CDKL3	73374	ES	9	8	10.1	-0.05	2.2E-03	excluded
FAM129C	48337	ES	13:14.1	12	14.2	-0.02	2.2E-03	excluded
SIRT2	49709	ES	10	9	11	-0.01	2.2E-03	excluded
NDUFA13	302227	ES	4.3:6:7	4.1	8	-0.01	2.2E-03	excluded
SYTL2	18158	ES	2	1	3	0.03	2.2E-03	included
LMBR1	82487	ES	3	2.1	4.1	0.02	2.2E-03	included
RAP1GDS1	69999	ES	6	5	7	-0.02	2.2E-03	excluded
MBNL2	26144	ES	7	6.3	8	-0.04	2.2E-03	excluded
ZC3H14	28724	ES	13:14.1	10	15	0.06	2.2E-03	included
CAPZB	908	ES	10	9	12	0.00	2.2E-03	included
CUL2	11224	ES	2	1.2	4	-0.05	2.2E-03	excluded
BOLA3	54017	ES	3	2	4	-0.02	2.2E-03	excluded
CDIPT	35972	ES	2.1:2.2	1.5	3.2	0.00	2.2E-03	excluded
ABCD4	98496	ES	13	12	14	0.02	2.2E-03	included
PTPRA	58567	ES	12	11	13	-0.01	2.2E-03	excluded
CCDC91	20916	ES	16	15	17	-0.03	2.2E-03	excluded
MRPL13	85021	ES	3	2	4	0.00	2.2E-03	included
LOXL1	31618	ES	2	1	3	-0.01	2.3E-03	excluded
CPNE1	59202	ES	1.2:2.1:2.2	1.1	5	-0.04	2.3E-03	excluded
SLC25A45	16827	ES	8:09	7	10	-0.07	2.3E-03	excluded
INO80E	36004	ES	7:08:09	6.3	10	0.10	2.3E-03	included
EIF3H	84957	ES	5:06	3.2	7	0.04	2.3E-03	included
SLC2A8	87631	ES	2:03	1	4	-0.05	2.3E-03	excluded
OXNAD1	63642	ES	5	4	6	-0.05	2.3E-03	excluded
FN1	57396	ES	40.2	39	40.4	-0.03	2.3E-03	excluded
CTSE	9581	ES	7	6	8	0.05	2.3E-03	included
CTNND1	16000	ES	4.1:4.2:4.3	2.1	5	-0.03	2.3E-03	excluded
SLC25A40	80340	ES	11	10.2	12	-0.03	2.3E-03	excluded
PTPN13	69834	ES	21	20	22	0.06	2.3E-03	included
ZNF446	52470	ES	5	4	6	-0.01	2.3E-03	excluded
RPS20	83889	ES	2.1	1.4	2.3	0.00	2.3E-03	included
POMT1	87950	ES	2:03	1.1	4	-0.03	2.3E-03	excluded
CA2	84357	ES	3	2	4	-0.01	2.3E-03	excluded
PI4KB	7600	ES	2	1	4	-0.05	2.3E-03	excluded
LETMD1	21775	ES	2:05:06	1.2	7	0.10	2.3E-03	included
GLYR1	33866	ES	4	3	5	-0.01	2.3E-03	excluded
RPLP0	24733	ES	4.2:5.1	4.1	5.2	-0.07	2.4E-03	excluded
STX17	87068	ES	8	7	9	-0.01	2.4E-03	excluded
FLNB	65418	ES	32.1:32.2	31	33	0.00	2.4E-03	excluded
SORBS2	71383	ES	17	16	18	0.02	2.4E-03	included

ITGB1BP1	52618	ES	8.1	7	8.3	0.00	2.4E-03	excluded
NUP43	78099	ES	5	4	6	0.01	2.4E-03	included
SAP30BP	43482	ES	4	3	6	-0.01	2.4E-03	excluded
SORBS2	71390	ES	8:9.1:9.2	7	10	-0.03	2.4E-03	excluded
MTMR3	61691	ES	18	17	19	-0.02	2.4E-03	excluded
REPIN1	82239	ES	4.2	3.2	5.3	-0.06	2.4E-03	excluded
C1orf63	1147	ES	5.1:5.2:5.3	4.2	6.2	-0.07	2.4E-03	excluded
DCAF6	91282	ES	14	10	15	-0.02	2.4E-03	excluded
VDAC3	83725	ES	5.2	4.1	6	0.01	2.4E-03	included
WARS	29278	ES	6	4	7	-0.01	2.5E-03	excluded
PAK4	49760	ES	3.2	2.3	4	-0.01	2.5E-03	excluded
MTCH2	15787	ES	6	5	7	0.00	2.5E-03	included
RABEP2	35895	ES	2.2:3	1	4	-0.06	2.5E-03	excluded
TNC	87338	ES	16:19	15	20	0.03	2.5E-03	included
ZDHHC16	12708	ES	3	1	4	-0.04	2.5E-03	excluded
RABEPK	87555	ES	5	4	7.1	-0.01	2.5E-03	excluded
KCTD6	65459	ES	2	1	3.2	-0.05	2.5E-03	excluded
VEGFA	76336	ES	7.1:7.2	6	8.1	0.03	2.5E-03	included
RNF167	38607	ES	7:08	6	9	0.00	2.5E-03	excluded
C16orf62	34325	ES	15:16	14	17	0.00	2.5E-03	excluded
OSBPL9	2979	ES	6.1	5	7	0.03	2.5E-03	included
SH2D4A	82871	ES	6	5	7	-0.04	2.5E-03	excluded
IDH3A	32018	ES	3	2	4.1	-0.03	2.5E-03	excluded
GPR107	87896	ES	15	14	17	0.03	2.5E-03	included
VPS29	24444	ES	3.1:3.2	1	5	-0.02	2.6E-03	excluded
ACBD5	11069	ES	6	5	7	-0.02	2.6E-03	excluded
MYL6	22378	ES	4.3	4.1	5	0.05	2.6E-03	included
KARS	37641	ES	3:5:6:7:8:9:10:11:12:13:14:15.1	2	15.2	-0.07	2.6E-03	excluded
CD55	9635	ES	14	9	15	0.00	2.6E-03	excluded
MON1A	64928	ES	4	3	5	0.03	2.6E-03	included
PQLC1	46266	ES	4.1:4.2	2	5	-0.04	2.6E-03	excluded
HNRNPUL1	50033	ES	16.3	15	17	0.03	2.6E-03	included
HOPX	69380	ES	2	1.2	4.6	0.00	2.6E-03	included
PRIMPOL	71311	ES	3.1:3.2	2	4	0.02	2.6E-03	included
ZFAT	85268	ES	3	1	4	-0.10	2.6E-03	excluded
TMEM134	17232	ES	6.2	4.1	7	0.01	2.6E-03	included
GPX3	74125	ES	2	1	3	0.00	2.6E-03	included
PACRGL	68923	ES	2.3:2.4:3	2.2	4	-0.09	2.6E-03	excluded
ARNT	7514	ES	4	3	5	-0.06	2.6E-03	excluded
FRA10AC1	12564	ES	15	12	16	-0.01	2.6E-03	excluded
SCARB1	25156	ES	14	13.2	15	-0.02	2.6E-03	excluded
POSTN	25676	ES	18	16	19	0.03	2.7E-03	included
EGLN1	10279	ES	4	3	5	-0.02	2.7E-03	excluded
C1orf86	251	ES	4	3	5	0.00	2.7E-03	included
AKAP11	25751	ES	10	9	11	0.05	2.7E-03	included
ZC3HC1	81762	ES	9	8	10	0.00	2.7E-03	excluded
KANSL3	54546	ES	8	7	9	0.04	2.7E-03	included
PSMD6	65521	ES	3	2.2	4	0.01	2.7E-03	included
EBPL	25914	ES	2	1	6	-0.08	2.7E-03	excluded
PTK2	85315	ES	22	21.2	23	-0.01	2.7E-03	excluded
TBL2	79984	ES	2.3:2.4:3	1	4.3	0.01	2.7E-03	included
UBE2D3	70130	ES	9.2	8.2	10	0.00	2.7E-03	excluded
NRCAM	81400	ES	30:31:32	29	33	-0.01	2.7E-03	excluded
UBE2I	33061	ES	4.2:4.3	2	5.2	0.01	2.7E-03	included
SETD5	63098	ES	4:05	3	6	-0.08	2.7E-03	excluded
BAIAP2	44102	ES	2	1	3	-0.02	2.7E-03	excluded
CTNND1	16004	ES	03:04.3	2.1	5	-0.01	2.7E-03	excluded
ATP2B4	9450	ES	21	20	22	-0.05	2.8E-03	excluded
PRRC2C	8988	ES	34	33	35	0.04	2.8E-03	included
CNOT1	36671	ES	45	44	46	-0.01	2.8E-03	excluded
PITPNC1	43114	ES	10	9	11	-0.01	2.8E-03	excluded

COL1A1	265304	ES	15:16:17:18:19:20:21:22:23:24	14	28	-0.05	2.8E-03	excluded
ALAS1	65186	ES	2	1	3.1	-0.03	2.8E-03	excluded
NUP88	38676	ES	2	1	3	-0.04	2.8E-03	excluded
FIP1L1	69315	ES	2	1	3	0.04	2.8E-03	included
NAE1	36853	ES	5	4	6	0.01	2.8E-03	included
MRPS18A	76317	ES	5	4	6	0.01	2.8E-03	included
FLOT2	40004	ES	5	3	6	-0.06	2.8E-03	excluded
FAM193A	68581	ES	20	18	21	-0.01	2.8E-03	excluded
TCF7	73349	ES	11	10	14.2	-0.02	2.8E-03	excluded
TMUB2	41789	ES	4.7:4.8	4.5	5	-0.05	2.8E-03	excluded
DCLK2	70807	ES	17	16	18	0.07	2.8E-03	included
CAMLG	73422	ES	2:03	1	4	0.02	2.9E-03	included
CUTC	12778	ES	7	6	8	0.03	2.9E-03	included
ZNF211	52312	ES	3.1:3.2:4	2.2	5	0.08	2.9E-03	included
DSC2	45007	ES	16	15	17	0.06	2.9E-03	included
REPS2	88583	ES	13:14	12	15	-0.06	2.9E-03	excluded
OGG1	63167	ES	5:6.1:7.2	4	8	-0.08	2.9E-03	excluded
SORBS2	71376	ES	25	24	26	0.00	2.9E-03	excluded
NQO2	75160	ES	3:04:06	1	7	0.01	2.9E-03	included
LDHA	14627	ES	3:4:6.1:6.2	2.4	7	0.00	2.9E-03	excluded
SYNRG	40530	ES	7	6	8	-0.02	2.9E-03	excluded
CARM1	47600	ES	4	3	5	-0.03	2.9E-03	excluded
CHEK2	61543	ES	4	3	5	0.03	2.9E-03	included
CDK5RAP1	58983	ES	9	7	10	0.00	2.9E-03	included
SORBS2	71389	ES	8	7	9.1	-0.02	2.9E-03	excluded
KDM5B	9420	ES	6	5	7	-0.08	2.9E-03	excluded
TKT	65305	ES	6.1:6.2	5	7.1	-0.02	2.9E-03	excluded
ARHGAP27	41966	ES	12	11	13	0.03	3.0E-03	included
SMC5	86553	ES	19	18	20	-0.05	3.0E-03	excluded
HKR1	49488	ES	16.1	13	17.2	0.02	3.0E-03	included
TEP1	26436	ES	49	48	50	0.03	3.0E-03	included
DMKN	101865	ES	8	6.4	11	-0.05	3.0E-03	excluded
CREM	11291	ES	4:9.2:10.1	1	15	0.06	3.0E-03	included
CASP10	56807	ES	8	7	9	0.01	3.0E-03	included
MRPL55	10090	ES	2.3:2.4:2.5:2.6:2.8	2.2	2.9	0.02	3.0E-03	included
CLK1	56755	ES	5	4	6	0.02	3.0E-03	included
AKAP13	32356	ES	12	11	14	-0.06	3.0E-03	excluded
NAP1L1	23486	ES	6	5	7.4	0.00	3.0E-03	included
SDHAF2	16237	ES	2	1	3	-0.01	3.0E-03	excluded
ARL6IP4	25030	ES	2.2:3.1	2.1	3.2	0.04	3.1E-03	included
ADAL	30238	ES	2	1	3	0.02	3.1E-03	included
SUPT16H	26575	ES	4	3	5.1	-0.01	3.1E-03	excluded
GAK	68415	ES	04:05.1	3	5.2	-0.02	3.1E-03	excluded
EPOR	47694	ES	2	1	3	0.04	3.1E-03	included
AFMID	43851	ES	3	2	5	-0.01	3.1E-03	excluded
C8orf47	84642	ES	2	1	3	0.04	3.1E-03	included
ABI1	11035	ES	11.2	9	12	-0.03	3.1E-03	excluded
LIN7B	50889	ES	4	3	5	0.01	3.1E-03	included
TMEM107	39133	ES	2:3.2:3.3:3.4	1	3.7	0.02	3.1E-03	included
PIP4K2C	22652	ES	5	4	6	-0.02	3.1E-03	excluded
C11orf73	18187	ES	2:03	1	4	0.07	3.1E-03	included
HADHB	52889	ES	6	5	7	0.00	3.1E-03	included
TMEM161B	72743	ES	6	5	7	-0.01	3.2E-03	excluded
FBXW4	91812	ES	5	4	6	-0.01	3.2E-03	excluded
PACSIN2	62560	ES	5	4	6	0.00	3.2E-03	excluded
STRADA	42967	ES	10	9	11.1	0.00	3.2E-03	included
CD44	15143	ES	12.1:13:14	5	15	-0.07	3.2E-03	excluded
HM13	58895	ES	6	5	7	0.00	3.2E-03	excluded
ALKBH3	15469	ES	4	3	5.1	0.01	3.2E-03	included
FAM35A	12447	ES	7	6	8	0.02	3.2E-03	included
DST	76565	ES	70	69	71	-0.01	3.2E-03	excluded

TMEM39A	66294	ES	5	4	6	-0.01	3.2E-03	excluded
TBC1D5	63664	ES	8	7	9.2	0.04	3.2E-03	included
MRPL48	17724	ES	8	5	10	0.00	3.3E-03	included
MYOF	12552	ES	17	16	18	-0.06	3.3E-03	excluded
ACTR10	27682	ES	3	2	4	-0.05	3.3E-03	excluded
HERPUD1	36506	ES	3.2	2.2	4	0.03	3.3E-03	included
MLLT10	10974	ES	4	3	5	-0.05	3.3E-03	excluded
ENDOV	44066	ES	5	2.4	6.2	-0.06	3.4E-03	excluded
ARHGEF40	26522	ES	21	20	22	0.01	3.4E-03	included
TPD52L1	77418	ES	7:08	6	10	0.00	3.4E-03	excluded
ATG4A	89849	ES	9.2:10	9.1	11	0.02	3.4E-03	included
PTPN2	44726	ES	2	1	5	0.00	3.4E-03	included
CD74	152981	ES	6:7.1:8:9	4	10.2	0.02	3.4E-03	included
SORBS2	71387	ES	9.1:9.2	8	10	-0.03	3.4E-03	excluded
MMAB	24323	ES	3	2	4	-0.02	3.4E-03	excluded
HNRNPA1	212645	ES	3:4:5:6.1:6.2:7.1:7.2:9<br>.1:9.2:10:11.2	2	11.3	-0.04	3.4E-03	excluded
NSL1	9748	ES	4:06	3	7	0.00	3.4E-03	excluded
MSH6	53505	ES	4	3	5.1	0.01	3.4E-03	included
NAPA	50654	ES	4	3	5	0.00	3.4E-03	excluded
FKBP10	40948	ES	6.3	6.1	7	0.00	3.4E-03	excluded
APOL3	61992	ES	4.1:4.2:4.3	3.3	5	-0.07	3.4E-03	excluded
RNMTL1	38277	ES	2	1	3	0.02	3.4E-03	included
CNN2	46365	ES	4.2:5.1	4.1	5.2	-0.09	3.4E-03	excluded
C1orf43	7808	ES	2	1	3	-0.02	3.4E-03	excluded
POLB	83711	ES	10	9	11	-0.01	3.4E-03	excluded
OSBP2	61807	ES	12	11.2	13	-0.03	3.5E-03	excluded
BCL2L11	54956	ES	9	7	11	-0.05	3.5E-03	excluded
CLK4	74876	ES	2.3:3	2.1	4	-0.05	3.5E-03	excluded
COL1A1	402552	ES	34:35:36:37:38:41:42	33	43	0.03	3.5E-03	included
PTPN12	80200	ES	10	9	11	0.01	3.5E-03	included
NAA10	90537	ES	6	5	7	0.00	3.5E-03	included
EXD3	88302	ES	5:06:07	4	8	-0.04	3.5E-03	excluded
MORC2	61809	ES	1.2:2	1.1	3	-0.04	3.5E-03	excluded
MDH2	80161	ES	3	1	4	0.00	3.5E-03	included
CRYL1	25426	ES	5	4	6	0.01	3.5E-03	included
PHF11	25893	ES	11	10	12	-0.01	3.5E-03	excluded
SPCS2	17803	ES	04:05.1	3	5.2	0.00	3.5E-03	excluded
DNAJC7	40974	ES	4	1	5	0.00	3.5E-03	included
BMP7	59887	ES	4	3	5	0.00	3.5E-03	excluded
ACO1	86056	ES	2	1	3	0.02	3.5E-03	included
HMGCL	1080	ES	7:08	6	9	0.00	3.5E-03	included
ZNF346	74724	ES	2	1	3	-0.02	3.5E-03	excluded
FHL1	90190	ES	9	8	10	-0.02	3.5E-03	excluded
GRN	573794	ES	11	9	12	-0.02	3.6E-03	excluded
ZNF160	51655	ES	3.1:3.2:3.3	2	4	0.08	3.6E-03	included
SUMF2	79798	ES	5.1:5.2	3	6	0.04	3.6E-03	included
MKNK1	2810	ES	9.1	8	10	0.02	3.6E-03	included
DST	76561	ES	104	103	105	-0.03	3.6E-03	excluded
ARMC10	81158	ES	9	6	10	-0.02	3.6E-03	excluded
TIA1	53873	ES	8	7	9.1	-0.06	3.6E-03	excluded
GSTO2	13055	ES	3.2	2	4	0.04	3.6E-03	included
SUN1	78550	ES	4	3.2	5.2	0.01	3.6E-03	included
GOLGA2	87727	ES	7	6	8	-0.03	3.6E-03	excluded
FAM219B	31804	ES	1.3	1.1	2	0.05	3.6E-03	included
GIPC1	47989	ES	2	1	3	0.02	3.7E-03	included
MAGOHB	20472	ES	5	4	6	-0.02	3.7E-03	excluded
SMUG1	22137	ES	1.2:2.2	1.1	4.1	-0.02	3.7E-03	excluded
LSM12	41759	ES	6	5	7	-0.04	3.7E-03	excluded
ZNF789	80652	ES	3	2	4.1	0.06	3.7E-03	included
CD55	9636	ES	13	9	15	0.00	3.7E-03	excluded
MRPL55	10134	ES	2.2:2.5:2.6:2.7:2.8	1.1	2.9	0.02	3.7E-03	included

HSCB	61550	ES	2.1:2.2:3.1:4	1	5	0.02	3.7E-03	included
TIMMDC1	66310	ES	1.2:2:3:4	1.1	5	0.05	3.7E-03	included
KIAA1217	11006	ES	22	21	25.1	-0.03	3.7E-03	excluded
ACOT8	59632	ES	2:3.1:3.2	1	4	0.06	3.7E-03	included
BCCIP	13434	ES	4	3	5	0.00	3.7E-03	included
NT5C3A	79213	ES	4.1:4.2	3	5	0.02	3.7E-03	included
SMPDL3A	97281	ES	4	3	5	0.04	3.7E-03	included
SUMO1	56942	ES	3	1	4	-0.01	3.7E-03	excluded
TRIP12	57852	ES	4.2:5:6:8.1:8.2:9:10:1 1:12:13:14:15:16:17:1 8:19.2:20:21:22:23.1	4.1	23.2	-0.03	3.7E-03	excluded
NBPF10	4449	ES	79:80	78	81	-0.05	3.7E-03	excluded
SPAST	53147	ES	4	3	5	0.03	3.7E-03	included
CNIH4	9958	ES	4	3	5.1	0.00	3.7E-03	excluded
SCP2	3045	ES	12	11	13	0.06	3.8E-03	included
CFI	70341	ES	8	6	9	-0.01	3.8E-03	excluded
NUDT9	69868	ES	2:03	1.2	4	0.00	3.8E-03	included
BTF3L4	2996	ES	2:03	1	4	0.04	3.8E-03	included
MMADHC	55561	ES	7	6	8	-0.01	3.8E-03	excluded
MEF2A	32715	ES	13	10	14	0.01	3.8E-03	included
IFT46	19025	ES	4	3	5	0.03	3.8E-03	included
PUF60	85503	ES	6	5	7	0.02	3.8E-03	included
METTL8	55929	ES	9	8	10	0.02	3.8E-03	included
ATP6AP2	88832	ES	5	3	6	0.06	3.8E-03	included
SEC16A	88176	ES	24:25:00	23.12	26	0.05	3.8E-03	included
PRDX5	16636	ES	3	2	4	0.00	3.8E-03	included
ANXA2	30956	ES	3	1.1	4.2	0.00	3.9E-03	excluded
DTNA	45099	ES	31:32.1	30	32.2	-0.01	3.9E-03	excluded
TANGO2	61121	ES	7.1:7.2:8	6	9	-0.08	3.9E-03	excluded
CTNND1	15980	ES	4.1:4.2:4.3:5	2.1	6	-0.07	3.9E-03	excluded
PRKCZ	243	ES	22	21	23	-0.01	3.9E-03	excluded
CASP6	70332	ES	4	3	5	0.02	3.9E-03	included
MED15	61171	ES	6	5	7	-0.01	3.9E-03	excluded
KDELR2	78738	ES	2	1	3	0.00	3.9E-03	excluded
AXIN1	32866	ES	9	8	10	0.04	3.9E-03	included
EMC4	29841	ES	4.2	3.2	6	0.01	3.9E-03	included
CD99	88410	ES	4	3	5	0.00	3.9E-03	included
SLCO2A1	66864	ES	4	3	5	-0.01	3.9E-03	excluded
CDC16	26407	ES	13	12	14	0.00	4.0E-03	included
C1orf86	248	ES	6:7.1:8:9	5	10	-0.04	4.0E-03	excluded
ACSF2	42389	ES	5.1:5.2	4	6.1	-0.01	4.0E-03	excluded
NBPF1	836	ES	10:11:12:13:14:15	9	16	-0.04	4.0E-03	excluded
TM7SF3	20859	ES	9	8	10	-0.01	4.0E-03	excluded
LYPLAL1	91405	ES	3.1:3.2	2	4	0.02	4.0E-03	included
TBC1D25	88993	ES	3	2	4	-0.02	4.0E-03	excluded
SQSTM1	74941	ES	4	3	5.2	0.00	4.0E-03	excluded
KARS	37643	ES	2	1	3	-0.03	4.0E-03	excluded
SGMS1	11575	ES	9:10	8	11	-0.02	4.0E-03	excluded
PSMG4	75189	ES	5.1:5.4	4	5.6	0.01	4.0E-03	included
C11orf74	15440	ES	5:06	4	7	-0.03	4.0E-03	excluded
ZNF568	49436	ES	3	2	4	0.04	4.0E-03	included
BRD9	71464	ES	18.2	17	20	0.01	4.0E-03	included
TMPO	23846	ES	6	5.1	7	0.02	4.0E-03	included
ZFAND1	84308	ES	7	6	8.1	-0.02	4.0E-03	excluded
TSPAN19	23575	ES	5	4	6	0.03	4.0E-03	included
AHDC1	90856	ES	3	2	4.2	0.03	4.0E-03	included
BCAR1	37603	ES	4.2:4.3	3	8.1	-0.07	4.1E-03	excluded
PLOD1	695	ES	2	1	3	-0.01	4.1E-03	excluded
P4HA3	17769	ES	10	9	11	0.01	4.1E-03	included
TFDP1	26393	ES	7	6	8	0.01	4.1E-03	included
RASA1	72721	ES	1.2:3	1.1	4	0.00	4.1E-03	excluded
WBP5	89728	ES	2	1	3	0.01	4.1E-03	included

DMKN	49204	ES	7	6.4	12	-0.02	4.1E-03	excluded
DTNB	52868	ES	12	11	13	0.01	4.1E-03	included
NEK3	25994	ES	12.2	11	13	-0.09	4.1E-03	excluded
CPM	23300	ES	4	3.2	5	-0.03	4.2E-03	excluded
BAX	50837	ES	5	4	6.1	0.00	4.2E-03	excluded
WNK1	19630	ES	12	9	13.1	-0.04	4.2E-03	excluded
PSMB5	26692	ES	2	1.2	4	0.00	4.2E-03	included
GLA	89642	ES	5	4	6	0.01	4.2E-03	included
SNUPN	31881	ES	2	1.2	4	-0.08	4.2E-03	excluded
TBC1D22A	62731	ES	5	1	6	-0.04	4.2E-03	excluded
DEPDC5	61899	ES	40	39	41	0.02	4.2E-03	included
POLR2C	36565	ES	3	2	4	-0.01	4.2E-03	excluded
TAZ	90591	ES	6:07	4	8.1	-0.02	4.2E-03	excluded
ARHGEF6	90216	ES	8	7	9	-0.08	4.2E-03	excluded
MCCC1	67781	ES	6	5	7	-0.01	4.2E-03	excluded
HBS1L	77809	ES	3	2	4	0.00	4.2E-03	included
FAM3A	90639	ES	3.2	2	4	0.03	4.3E-03	included
ASH2L	83371	ES	4	3	5	0.00	4.3E-03	included
LSM4	48416	ES	4	3	5	0.00	4.3E-03	included
FN1	57397	ES	33	32	34	-0.04	4.3E-03	excluded
ELOF1	47744	ES	5	1	7	-0.01	4.3E-03	excluded
ARID4A	27700	ES	23.1:23.2	22	24	0.01	4.3E-03	included
SNRPA1	32758	ES	6	5	7	0.02	4.3E-03	included
LMO7	26062	ES	30	29	31	0.00	4.4E-03	included
ACADM	3492	ES	6	4.2	7	-0.01	4.4E-03	excluded
PCBP4	65137	ES	3	2.2	5	-0.08	4.4E-03	excluded
IRF3	50994	ES	5.1:6.1	4	6.2	-0.01	4.4E-03	excluded
RPAIN	38681	ES	05:06.1	4	7	-0.01	4.4E-03	excluded
KDM5C	89196	ES	4	3	5	-0.05	4.4E-03	excluded
PI4KB	7599	ES	2:03	1	4	-0.05	4.4E-03	excluded
TUBGCP2	13534	ES	7	6	8	-0.03	4.4E-03	excluded
CDIPT	35970	ES	5	4	6	0.00	4.4E-03	included
SLC30A9	69138	ES	4	3	5	0.00	4.4E-03	included
PPP2R1A	51422	ES	3	1	5	0.00	4.4E-03	excluded
NDRG2	26507	ES	4.2:4.5:4.6	4.1	5.2	0.00	4.4E-03	excluded
ANXA2	30957	ES	2	1.1	4.2	0.01	4.4E-03	included
FLAD1	91160	ES	2.2:4.1:4.2:4.3	1.3	6.1	0.03	4.4E-03	included
TJP2	86539	ES	23	22.1	24	0.00	4.4E-03	included
GABPB2	7559	ES	6	5	7	0.05	4.4E-03	included
SNX1	139184	ES	4.2:5:6:8:9:10.1:10.2:11:12:14:15	3	16.1	0.00	4.4E-03	excluded
ATP13A2	840	ES	28	27	29	0.01	4.4E-03	included
SRSF11	3396	ES	6.3	4.2	6.5	-0.02	4.5E-03	excluded
INCA1	38644	ES	3	2.2	4	0.01	4.5E-03	included
FAM162A	66435	ES	2	1	3	0.00	4.5E-03	excluded
ABCD4	28390	ES	03:04.1	2	5	-0.01	4.5E-03	excluded
TNFRSF11B	84998	ES	3	2	4	0.06	4.5E-03	included
ABI1	11042	ES	11.2	9	13	-0.05	4.5E-03	excluded
TMEM39B	1546	ES	6	5	7.2	0.01	4.5E-03	included
CD40	59663	ES	5	4	7	0.05	4.5E-03	included
LMO3	20617	ES	10:11.1:11.2	9.2	12	-0.02	4.5E-03	excluded
CAMK2B	79490	ES	16	15	17	-0.03	4.5E-03	excluded
TMEM134	17231	ES	6.1:6.2	4.1	7	0.03	4.6E-03	included
EIF2A	67287	ES	3	2	4	-0.05	4.6E-03	excluded
PPP4R1	44611	ES	5	4.2	6	-0.05	4.6E-03	excluded
C14orf2	29529	ES	4:05:06	2	7.1	0.01	4.6E-03	included
DMKN	49188	ES	7:8:9:12	6.4	13	0.11	4.6E-03	included
BIN1	55198	ES	13:16	12	17	-0.02	4.6E-03	excluded
PCM1	82838	ES	26	25	27	0.02	4.6E-03	included
DDX19A	37374	ES	7	5.2	8.1	-0.01	4.6E-03	excluded
AP1AR	70363	ES	6	5	7	0.01	4.6E-03	included
C14orf2	29536	ES	3	2	5	0.00	4.7E-03	included

VPS25	41127	ES	2.2:3	2.1	4	0.00	4.7E-03	excluded
DDX11	20967	ES	25:26:27:28.1:28.2:29.	24	29.3	0.02	4.7E-03	included
NFIC	46681	ES	10	9	11	0.03	4.7E-03	included
AFMID	43798	ES	7:08:09	6	10	0.03	4.7E-03	included
MACF1	1885	ES	77	76.1	79	0.01	4.7E-03	included
TG	85209	ES	22	21	23	0.00	4.7E-03	included
PISD	61885	ES	2	1	3	0.01	4.7E-03	included
PPM1K	69887	ES	2	1	3.1	-0.04	4.7E-03	excluded
ILK	14172	ES	2:03	1.4	4	-0.06	4.7E-03	excluded
CRLF3	40126	ES	2	1	3	0.00	4.7E-03	included
PQLC1	46261	ES	6:07	5	9	-0.02	4.7E-03	excluded
SLC24A1	31234	ES	11	10	12	-0.05	4.8E-03	excluded
CACNB1	40630	ES	7	6	9	0.05	4.8E-03	included
APP	60282	ES	17	16	18	0.00	4.8E-03	excluded
ANKRD11	38082	ES	6	5.3	7	0.01	4.8E-03	included
TBL2	79985	ES	3	1	4.3	-0.03	4.8E-03	excluded
RPS9	51825	ES	4.1:4.3	3	4.5	0.03	4.8E-03	included
ZSWIM7	39396	ES	5.1:5.2	4	6	0.01	4.8E-03	included
MLLT4	251635	ES	25.1:25.2:26:27:28:29<br>:30:31:33.2:34.1	24.2	35	-0.07	4.8E-03	excluded
AGFG1	57820	ES	7	6	8	-0.01	4.8E-03	excluded
STXBP6	27041	ES	3:04	1.1	6	-0.04	4.8E-03	excluded
CDH11	36699	ES	3	2	4	-0.01	4.8E-03	excluded
PPIL6	77196	ES	2	1	3	0.03	4.8E-03	included
MAPKAPK5	24545	ES	4:5:6:7:8	1	9	0.00	4.8E-03	excluded
RPS5	52446	ES	3	2.3	4	0.00	4.8E-03	excluded
SNF8	42247	ES	8	7.1	9	-0.01	4.8E-03	excluded
ERCC1	50444	ES	09:10.1	7	11	0.00	4.8E-03	excluded
TXNDC12	2991	ES	4	2	5	0.00	4.8E-03	excluded
CPNE1	59191	ES	2.2:3	1.2	5	-0.03	4.8E-03	excluded
WARS	29279	ES	5	4	7	0.00	4.9E-03	excluded
SSBP2	72676	ES	5	4	6	0.01	4.9E-03	included
GSTZ1	28590	ES	5	1	6	0.00	4.9E-03	excluded
MOK	29375	ES	16	15.6	17.1	-0.03	4.9E-03	excluded
GPN1	53035	ES	5:06	4.2	7	0.02	4.9E-03	included
PPFIBP1	20890	ES	20	19	21	0.05	4.9E-03	included
ARHGAP12	11152	ES	9	8	10	-0.03	4.9E-03	excluded
SLC37A4	19077	ES	2.1:2.2	1	3.1	-0.04	4.9E-03	excluded
SLC25A14	90099	ES	8	7	9.1	-0.05	4.9E-03	excluded
ABHD14A-ACY1	65157	ES	5	4	6	0.04	4.9E-03	included
MAX	27957	ES	5.5	5.1	5.8	0.02	4.9E-03	included
SENP5	68262	ES	2	1	3	-0.02	4.9E-03	excluded
USF2	49097	ES	04:05.1	2.3	5.2	0.00	4.9E-03	excluded
KARS	37639	ES	4	3	5	0.00	5.0E-03	included
MMAB	24322	ES	5	4	6	0.03	5.0E-03	included
U2AF1L4	49273	ES	3.1:3.2:4	2.2	5	0.11	5.0E-03	included
PTPRG	65493	ES	14	13	15	0.04	5.0E-03	included
JOSD2	51205	ES	4	3.2	5	-0.01	5.0E-03	excluded
RFWD2	9054	ES	9	8	10	0.02	5.0E-03	included
ZNF655	80664	ES	6.1:6.2	5.3	7	-0.05	5.0E-03	excluded
SMAD6	31298	ES	5	4	6	0.00	5.0E-03	excluded
GNB2L1	190583	ES	5:06	3	9	0.04	5.0E-03	included
GCNT1	86638	ES	4	3	5	0.05	5.0E-03	included
FGD4	21038	ES	6	5	7.1	-0.02	5.0E-03	excluded
CENPJ	25487	ES	11	10	12	-0.03	5.0E-03	excluded
TMEM62	30214	ES	5	4	6	0.03	5.0E-03	included
TMEM106C	21393	ES	4.1:4.2:5.1:5.2	3	6	0.00	5.1E-03	excluded
TLK2	42903	ES	10	9	11	0.03	5.1E-03	included
DROSHA	71628	ES	2	1	3	0.05	5.1E-03	included
TMEM194A	22513	ES	3.2:4	3.1	5	-0.05	5.1E-03	excluded
PHKB	36324	ES	2	1	3	-0.02	5.1E-03	excluded
CALM1	28823	ES	3	1.2	4	0.00	5.1E-03	included

PCTP	42589	ES	3.2:4.1	3.1	4.2	-0.01	5.1E-03	excluded
NSMF	88316	ES	10.1	9.2	10.3	-0.01	5.1E-03	excluded
PRDM2	728	ES	8	7	9	-0.03	5.1E-03	excluded
PPOX	8581	ES	3:4:5:6:7:8:9:10	2	11	0.01	5.1E-03	included
METAP1	70019	ES	2	1	3	-0.01	5.1E-03	excluded
FLNA	205372	ES	37:38:39:41:42:43:44:	36	46	-0.03	5.1E-03	excluded
ZNF140	25328	ES	6	5.3	8.1	-0.04	5.1E-03	excluded
FAM86B1	82711	ES	4:05:06	3.2	7.1	-0.09	5.1E-03	excluded
COBLL1	55788	ES	14	12	15	-0.04	5.1E-03	excluded
KLC1	29475	ES	14.1:15	13.3	18	-0.01	5.1E-03	excluded
ASPSCR1	44262	ES	3	1	5	0.00	5.1E-03	included
PDCD6	71427	ES	5.1:5.2:6.1	4	7	0.00	5.1E-03	included
NDUFB1	28987	ES	3	1	4	0.01	5.2E-03	included
NIPA2	29683	ES	2:03	1.1	4	-0.05	5.2E-03	excluded
BCAS3	42873	ES	28	27	30	-0.03	5.2E-03	excluded
IK	73709	ES	3	2	4	-0.04	5.2E-03	excluded
KIAA0101	31115	ES	3	2	5	-0.02	5.2E-03	excluded
YAF2	21163	ES	3.1:3.2:5.2	2	9.1	-0.01	5.2E-03	excluded
ATL2	389879	ES	16:17.1	14	17.2	-0.11	5.2E-03	excluded
ANGPTL4	47224	ES	4	3	5	0.01	5.2E-03	included
ITGB1BP1	52621	ES	3	2	4	0.03	5.2E-03	included
NDUFA12	23739	ES	2.2:3:5.1	2.1	5.2	0.00	5.2E-03	excluded
PGAP2	14016	ES	6	5	10	-0.03	5.2E-03	excluded
TMEM209	81772	ES	11	10	12	-0.01	5.2E-03	excluded
TANK	55739	ES	12	11	14	0.00	5.2E-03	excluded
MKRN2	63439	ES	2.1:2.2	1	3	-0.01	5.3E-03	excluded
MPDZ	85873	ES	41	40	42	-0.02	5.3E-03	excluded
TOR1AIP2	9125	ES	3	2	4	-0.02	5.3E-03	excluded
PAPLN	28278	ES	21.1:21.2	20	22	0.03	5.3E-03	included
SNX3	77146	ES	3	2	4	0.00	5.3E-03	excluded
RAD1	71742	ES	7	5	8	0.03	5.3E-03	included
CAPN7	99511	ES	16	15	17	-0.02	5.3E-03	excluded
PBRM1	65236	ES	28:29:00	27	30	0.04	5.3E-03	included
DNAJC11	495	ES	12	11	13	0.00	5.3E-03	included
RPS5	52445	ES	4	2.3	5	0.00	5.3E-03	excluded
HRAS	13685	ES	6	5	7.1	0.03	5.3E-03	included
CA12	31026	ES	9	8	10	-0.04	5.3E-03	excluded
TACC2	13340	ES	20	19	22	-0.01	5.4E-03	excluded
NDUFA12	23740	ES	2.2:5.1	2.1	5.2	-0.01	5.4E-03	excluded
CLEC1A	20304	ES	2	1	3	0.08	5.4E-03	included
NAPA	50662	ES	3	1	5	0.02	5.4E-03	included
SS18	44966	ES	6	3	9	-0.04	5.4E-03	excluded
DNAJC2	81189	ES	5	4	6	-0.03	5.4E-03	excluded
ANKS3	33804	ES	7.1:7.2	6	8	-0.04	5.4E-03	excluded
NOSTRIN	55836	ES	8	6	9	0.01	5.4E-03	included
WDR61	32081	ES	8	7	9	0.00	5.5E-03	excluded
RBMX	90223	ES	4	3.2	5	0.00	5.5E-03	included
SNRNP40	1459	ES	10	9	11	-0.01	5.5E-03	excluded
FAM210A	44743	ES	2	1	3	-0.06	5.5E-03	excluded
SELT	67291	ES	3.1:3.2	2.1	4	-0.02	5.5E-03	excluded
DYM	45471	ES	7:8:10:11:12	6	15.1	0.00	5.5E-03	included
MLPH	58114	ES	12	11	13	0.02	5.5E-03	included
MRPL35	54421	ES	3	2	4.1	0.00	5.5E-03	included
NBPF12	7360	ES	9	8	10	-0.04	5.6E-03	excluded
KIF9	64506	ES	2.3	2.1	4	0.01	5.6E-03	included
ENOX2	90109	ES	3	2	5	0.03	5.6E-03	included
SP100	57905	ES	4	3	5	-0.01	5.6E-03	excluded
MBD1	45518	ES	15	14	16.1	-0.02	5.6E-03	excluded
MTMR14	63113	ES	19:20	18	21	-0.02	5.6E-03	excluded
CPSF6	23307	ES	3	2.2	4	-0.01	5.6E-03	excluded
DECR1	84406	ES	2:3:4.1:4.2:5.1	1	5.2	0.00	5.6E-03	excluded
EFEMP1	53636	ES	7:08	6	9	-0.01	5.6E-03	excluded

JAG2	29601	ES	10	9	11	-0.03	5.7E-03	excluded
TARS	71683	ES	3.1:3.2	2	4	0.00	5.7E-03	excluded
EIF4G1	67905	ES	2.2:2.4:3.2:5	1	6	0.01	5.7E-03	included
MINA	65745	ES	6	5	7	-0.02	5.7E-03	excluded
CBWD5	86503	ES	11	10	12.1	0.00	5.7E-03	included
DGCR2	61036	ES	6	5	7	0.00	5.7E-03	excluded
TARS	71684	ES	3.2	2	4	0.00	5.7E-03	excluded
MKS1	42650	ES	6.2	5	7	0.01	5.8E-03	included
RWDD1	77327	ES	2:03	1	4	-0.02	5.8E-03	excluded
ST6GALNAC4	87685	ES	2	1	3	0.02	5.8E-03	included
MIF4GD	43418	ES	3.1:3.2:4:5	2	6.1	0.01	5.8E-03	included
TPD52L1	77420	ES	7	6	8	0.00	5.8E-03	excluded
SLC25A26	65548	ES	7	6	9	-0.01	5.8E-03	excluded
SNX1	139182	ES	7:8:9:10.1:10.2:11:12:	3	16.1	0.00	5.8E-03	excluded
RHOT2	32945	ES	3	2.2	4.1	0.00	5.8E-03	included
TCF7L2	13146	ES	15:16	14	17	-0.05	5.8E-03	excluded
GIPC1	47988	ES	3	1	5	-0.06	5.8E-03	excluded
MGRN1	33781	ES	17.1	16	17.3	-0.03	5.8E-03	excluded
CREM	11294	ES	3	1	4	-0.03	5.8E-03	excluded
NGLY1	63755	ES	11	10	12	-0.01	5.8E-03	excluded
ANKDD1A	31141	ES	15	14	16	-0.03	5.9E-03	excluded
METTL23	43638	ES	1.4:2	1.3	3	0.04	5.9E-03	included
CFL1	16925	ES	3	2	6	0.00	5.9E-03	included
ATL2	53248	ES	16:17.1	15	17.2	-0.03	5.9E-03	excluded
TBRG1	19224	ES	5	3	6	0.04	5.9E-03	included
IMPDH1	81676	ES	7.1:7.2	6	8.1	0.01	5.9E-03	included
GNB2L1	75061	ES	4.2	3	5	0.00	5.9E-03	excluded
HMGN1	60620	ES	6.1:6.2:8.2	5	9	0.00	5.9E-03	included
ATP6V0B	2510	ES	2.1:2.2	1.1	3.2	-0.05	5.9E-03	excluded
FGF1	73873	ES	7.1:7.2	6	8	-0.03	5.9E-03	excluded
SCMH1	2049	ES	7:08	6	9	0.03	5.9E-03	included
INO80C	45177	ES	3:4.2:5.1:5.2:6:7	1	8	0.06	6.0E-03	included
KCTD21	18014	ES	2	1	3	-0.03	6.0E-03	excluded
FAM86C1	17442	ES	3.1:3.2	2	4	-0.05	6.0E-03	excluded
CHEK2	61532	ES	9	6	10	0.02	6.0E-03	included
SUN2	62262	ES	6	3	7	0.04	6.0E-03	included
NUPR1	35752	ES	2	1	3.2	0.00	6.0E-03	included
BCL2L11	54955	ES	8:09	7	11	-0.02	6.0E-03	excluded
TMUB2	41821	ES	2.5:4.2	1	4.3	0.07	6.0E-03	included
OSBPL10	63809	ES	4	3	5	0.02	6.0E-03	included
USO1	69551	ES	14	13	15	-0.05	6.1E-03	excluded
CAST	72862	ES	7.2:8.2	7.1	9	-0.01	6.1E-03	excluded
CCT7	53973	ES	2	1	3	0.00	6.1E-03	included
C16orf13	32923	ES	2	1	5	-0.04	6.1E-03	excluded
ADAM15	7901	ES	21.2:22.1:22.2	20	23	-0.02	6.2E-03	excluded
NFIC	46680	ES	11	9	12	-0.03	6.2E-03	excluded
ZNF692	10556	ES	9	8	10	0.01	6.2E-03	included
OAZ3	7654	ES	5	4.5	6	0.02	6.2E-03	included
UBE2F	58156	ES	11	10	12.1	0.00	6.2E-03	excluded
IFT122	66727	ES	19	18	20	0.03	6.2E-03	included
CHFR	25318	ES	05:06.2	4	8	0.04	6.2E-03	included
TMEM116	24550	ES	10	9	11	-0.01	6.2E-03	excluded
IFRD2	64980	ES	4	3	5	0.01	6.2E-03	included
AGTRAP	672	ES	4.2	3	5	-0.02	6.3E-03	excluded
MFSD11	43677	ES	7	6	8	0.01	6.3E-03	included
IRF3	50996	ES	5.1	4	6.2	0.00	6.3E-03	excluded
ARFIP1	70858	ES	4	3	5	-0.02	6.3E-03	excluded
NLRP1	38722	ES	14	13	15	-0.02	6.3E-03	excluded
IDE	12534	ES	20	19	21	-0.03	6.3E-03	excluded
INTS6	25943	ES	14	13	15	-0.01	6.3E-03	excluded
AFMID	43793	ES	11.1	10	12	0.03	6.3E-03	included
AFMID	43808	ES	8:09:10	6	12	-0.01	6.3E-03	excluded

GPR56	107347	ES	5.1:5.2	2	7.2	-0.04	6.4E-03	excluded
SDHC	8663	ES	3	2	5	0.00	6.4E-03	excluded
PTBP1	46320	ES	3:4:5:6:7:8:9.2:10	2	11	0.05	6.4E-03	included
CYP20A1	57008	ES	3	2	4	-0.02	6.4E-03	excluded
TPD52L1	77410	ES	7:08	6	9.1	0.00	6.4E-03	excluded
SPATA7	28693	ES	8	6.2	9	-0.04	6.4E-03	excluded
MARCH7	55702	ES	12	11	13	-0.01	6.5E-03	excluded
COX4I1	156374	ES	4.2:5.1:5.3	4.1	5.4	0.00	6.5E-03	excluded
VSIG4	89325	ES	3	2	4	-0.03	6.5E-03	excluded
QARS	64826	ES	10	9	11	0.01	6.5E-03	included
NDEL1	39189	ES	11	10	12.1	-0.03	6.5E-03	excluded
HCFC1R1	33356	ES	1.5	1.2	2	0.02	6.5E-03	included
DPH3	63634	ES	2	1	3	0.04	6.5E-03	included
PPP6C	87545	ES	4	3	5	0.00	6.5E-03	excluded
PLEKHN1	1	ES	11	10	12	-0.03	6.5E-03	excluded
NECAB3	102496	ES	7.3	7.1	8	0.02	6.5E-03	included
MRPL55	10169	ES	1.2:2.2:2.3:2.4	1.1	2.5	0.01	6.5E-03	included
MTMR2	18403	ES	5	3	6	-0.06	6.5E-03	excluded
CIB2	32001	ES	2:03	1	4	-0.01	6.6E-03	excluded
DNM2	47586	ES	16	14	17	0.02	6.6E-03	included
ATIC	57352	ES	6	5	7	0.00	6.6E-03	included
ZNF687	7588	ES	2	1	3	-0.07	6.6E-03	excluded
INSIG1	82433	ES	6	5	7	0.03	6.6E-03	included
STAC3	22561	ES	7	6	8	0.01	6.6E-03	included
FAM208A	65356	ES	17:18.1	16	18.2	-0.01	6.6E-03	excluded
YTHDC1	69421	ES	6	5	7	0.02	6.6E-03	included
KIFC3	36616	ES	6	5	13	-0.03	6.6E-03	excluded
MRPL10	42103	ES	1.2:2.2	1.1	3	-0.01	6.6E-03	excluded
GIT2	24385	ES	17.1:17.2:18.1	15	18.2	0.04	6.6E-03	included
KDM5C	89208	ES	2.2	1	3	0.01	6.6E-03	included
RNF2	9211	ES	4	3	5	0.00	6.7E-03	included
MKNK1	2812	ES	7.1	5	8	0.01	6.7E-03	included
LIG1	50696	ES	3	2	4	0.01	6.7E-03	included
MAP3K3	42944	ES	3	2.2	4	-0.04	6.7E-03	excluded
GABARAPL1	20402	ES	2.7:2.8:2.9:2.10:2.12:2	2.6	3	0.00	6.7E-03	excluded
COX7A2	76779	ES	4.1:4.2	3	5	0.07	6.7E-03	included
WDR35	52748	ES	27	26	28	0.01	6.7E-03	included
CCDC148	55664	ES	5:6:7:8:9.1	4	10	-0.02	6.7E-03	excluded
ERBB2IP	72262	ES	23:24.1:24.2:24.3	21	25	0.08	6.7E-03	included
DMKN	101858	ES	8:11:12	6.4	13	0.06	6.7E-03	included
SS18	44960	ES	5:8.1:8.2	3	9	0.00	6.7E-03	excluded
ABI1	11034	ES	11.1:11.2	9	12	-0.01	6.8E-03	excluded
MYEF2	30483	ES	3	2	4	-0.02	6.8E-03	excluded
RPL7L1	76195	ES	5.1	4	6.1	0.00	6.8E-03	included
ZNF280D	30778	ES	22	21	23.1	-0.01	6.8E-03	excluded
CPNE1	59188	ES	3	2.2	5	-0.02	6.8E-03	excluded
LDHA	14628	ES	4:6.1:6.2	2.4	7	0.00	6.8E-03	excluded
IAH1	52634	ES	3	2	4.1	0.02	6.8E-03	included
DUSP22	75135	ES	2:03:05	1	6	0.00	6.8E-03	excluded
YAF2	21155	ES	7	2	9.1	-0.02	6.8E-03	excluded
CEP112	43098	ES	24:25:00	22	26	0.00	6.8E-03	included
KLC1	29477	ES	14.1	13.3	15	-0.02	6.8E-03	excluded
LIF	61698	ES	2	1	3	-0.02	6.8E-03	excluded
ABHD3	44779	ES	4:5.1:5.2	3	6	-0.05	6.8E-03	excluded
ZFYVE21	29515	ES	2:3.1:3.2	1	4	0.08	6.8E-03	included
EPN3	42412	ES	4	3.2	5	0.02	6.8E-03	included
TMEM205	47674	ES	2.2:2.3:2.5	2.1	2.6	0.03	6.9E-03	included
GGA3	43403	ES	6	5	8	-0.01	6.9E-03	excluded
MRPL2	76239	ES	3:04:05	2.1	7	0.04	6.9E-03	included
UBXN11	101235	ES	5	2	7	0.05	6.9E-03	included
UBE2K	69079	ES	2:03	1	4	0.00	6.9E-03	included
STX8	39220	ES	2	1	3	0.00	6.9E-03	included

IL32	33429	ES	1.2:1.3:1.4:1.5:1.6:1.8	1.1	1.9	0.01	7.0E-03	included
TNRC6A	94083	ES	7	6	8	-0.05	7.0E-03	excluded
STX16	59970	ES	7	6	8	-0.03	7.0E-03	excluded
PACS2	29635	ES	20	19	21	0.00	7.0E-03	excluded
SCAP	64521	ES	7:08:09	3	10	0.09	7.0E-03	included
PLAC8	69774	ES	7	5	8	-0.02	7.0E-03	excluded
SPOP	42313	ES	5	1	6	0.03	7.0E-03	included
C16orf93	94142	ES	6	5	7	0.04	7.0E-03	included
NUBP2	33138	ES	4	3	5	0.02	7.1E-03	included
TAF1C	37840	ES	2.1:2.2:3	1	4	-0.01	7.1E-03	excluded
MPDU1	39003	ES	5.1:5.2	3.2	6.1	0.01	7.1E-03	included
PTER	10876	ES	5	4	6	-0.01	7.1E-03	excluded
INPP5K	38335	ES	2:03	1	4	-0.01	7.1E-03	excluded
SKA2	42756	ES	1.2:2	1.1	5	0.04	7.1E-03	included
NPNT	70260	ES	11	10	12	0.01	7.1E-03	included
TCF7	73350	ES	6	5	7	0.06	7.1E-03	included
SMC2	87101	ES	25	24	26	-0.01	7.1E-03	excluded
ATP2C1	66766	ES	5	4	6	0.00	7.1E-03	excluded
KXD1	48463	ES	6	5	7.1	0.00	7.1E-03	included
SMARCD2	130243	ES	12	11	13	0.00	7.2E-03	included
SULT1A1	35821	ES	3:6.2:7:8	2	9	-0.01	7.2E-03	excluded
DNM1L	21051	ES	8	7	9	0.01	7.2E-03	included
RTN3	16531	ES	3.1:3.2	1	4	-0.01	7.2E-03	excluded
SHF	30416	ES	8.1:9.1	6	10	0.04	7.2E-03	included
EVC	99901	ES	24	23	25	-0.03	7.2E-03	excluded
TPD52L1	77412	ES	7:8:9.1:9.2	6	10	0.00	7.2E-03	excluded
CAP2	75432	ES	7:08	6	10	-0.03	7.2E-03	excluded
MSMO1	71042	ES	2	1	3	0.02	7.2E-03	included
PDDC1	13757	ES	2	1	3	-0.01	7.2E-03	excluded
AFMID	43814	ES	5:6:7:8:9:10:11.1:11.2	2	13	0.09	7.3E-03	included
HSFX2	90316	ES	2:03	1	4	-0.06	7.3E-03	excluded
PSMD13	13627	ES	10	9	11	0.00	7.3E-03	included
INPP5J	61820	ES	11	10	12	0.00	7.3E-03	included
FCGRT	50961	ES	6	4	7	0.00	7.3E-03	included
MTFR1L	1214	ES	5	4.2	7.2	0.03	7.3E-03	included
COPS4	69767	ES	8	7	9	-0.01	7.3E-03	excluded
PAAF1	17743	ES	4.1:4.2	3.2	5	-0.02	7.3E-03	excluded
GOSR1	40116	ES	9	7.2	10.1	0.00	7.3E-03	included
ARL1	23947	ES	4	3	5	0.00	7.3E-03	excluded
TPGS2	45269	ES	7.1:7.2	6	8	0.00	7.3E-03	excluded
ABHD10	66071	ES	2	1	3	0.01	7.3E-03	included
RAB7L1	9565	ES	2:03	1.4	4	0.01	7.3E-03	included
ELP3	83206	ES	3	2	4	0.01	7.3E-03	included
TMEM66	83267	ES	2.2:2.4	1.2	3	0.00	7.3E-03	excluded
DCAF8	8443	ES	8.1:8.2	7.3	9	-0.03	7.3E-03	excluded
RNFT1	42843	ES	3	2	5	0.07	7.3E-03	included
ACP5	47748	ES	4.2:5:6:7.1	4.1	7.2	0.00	7.3E-03	excluded
TIMM50	49836	ES	2:3:4:5:6:7:8	1	9	0.00	7.4E-03	included
IMPA1	84296	ES	7	6.2	8	-0.04	7.4E-03	excluded
PDLIM4	73264	ES	6	5	7	0.01	7.4E-03	included
RPS9	51823	ES	4.1:4.3:4.4	3	4.5	-0.05	7.4E-03	excluded
GTF2H2C	72391	ES	10.1:11:12:13:14:15:16:17:18.1	8	18.2	0.01	7.4E-03	included
PPP6R3	17323	ES	4	3	5	0.01	7.4E-03	included
CTH	3410	ES	5	4	6	-0.01	7.5E-03	excluded
SEPT2	58377	ES	4	2	7	-0.02	7.5E-03	excluded
RPH3AL	38215	ES	2	1	3	-0.06	7.5E-03	excluded
NUDT22	16587	ES	3	1.4	4	0.01	7.5E-03	included
LETMD1	21770	ES	1.3:2:3.2:4:5:6	1.2	7	0.08	7.5E-03	included
ZNF554	46627	ES	3	2	4	-0.04	7.5E-03	excluded
HPS3	67212	ES	2	1	3	0.01	7.5E-03	included
GLG1	37568	ES	2	1	3	0.00	7.5E-03	included

CD55	9638	ES	12.2	9	15	-0.02	7.5E-03	excluded
NAPA	50661	ES	2:03	1	5	0.00	7.5E-03	included
APIP	14970	ES	2	1	3	-0.02	7.5E-03	excluded
TMX2	15906	ES	4:5.1:5.3	2	6	0.02	7.5E-03	included
BLCAP	59357	ES	4	1	6	-0.01	7.5E-03	excluded
MAMLD1	90333	ES	5	4	6	0.05	7.5E-03	included
COPS7A	19950	ES	4	2.4	6	-0.01	7.6E-03	excluded
ANKRD17	69482	ES	16	15	17	0.04	7.6E-03	included
NR2C2AP	48614	ES	3	2	4.1	0.01	7.6E-03	included
GGT1	61445	ES	5	4	6.2	0.03	7.6E-03	included
THBS3	8028	ES	12	11	13	0.01	7.6E-03	included
DNAJC7	40973	ES	3:04	1	5	0.05	7.6E-03	included
NBPF11	7338	ES	9	8.3	10	-0.04	7.6E-03	excluded
MPP7	11093	ES	19	18	20	-0.05	7.6E-03	excluded
TPGS2	45276	ES	2	1	4.2	-0.01	7.6E-03	excluded
SERPINB1	75145	ES	3	2	4	-0.01	7.7E-03	excluded
HDAC10	62796	ES	14	13	15	-0.02	7.7E-03	excluded
RPE	57242	ES	8	7	10.1	-0.03	7.7E-03	excluded
IFI44	102199	ES	8	6	9	-0.05	7.7E-03	excluded
SMARCE1	40874	ES	4.1	3	6	0.00	7.7E-03	included
ATP5H	43345	ES	4	3	6	0.00	7.7E-03	included
CLCN3	71155	ES	3	2	4	-0.02	7.7E-03	excluded
TMEM106C	21395	ES	4.1:4.2:5.1	3	6	0.00	7.7E-03	excluded
DECR1	84411	ES	4.2	1	5.2	-0.04	7.7E-03	excluded
UBOX5	58571	ES	4	3	5	-0.02	7.7E-03	excluded
COL1A2	484887	ES	16:17:18:19:20:21:22:<br>23:24:25:26:34:35:36	15	37	-0.04	7.7E-03	excluded
MRPS5	54487	ES	7	6	8	0.00	7.8E-03	included
DDX19B	37359	ES	4	3	5.2	0.02	7.8E-03	included
C7orf55-LUC7L2	81961	ES	5	3.2	7	-0.04	7.8E-03	excluded
CLEC2D	20237	ES	7.3:7.4:7.5	7.1	8	-0.07	7.8E-03	excluded
LETMD1	21767	ES	2:3.2:3.3:4:5:6	1.2	7	0.08	7.8E-03	included
TMX2	15904	ES	3.2:3.3:4:5.1:5.3	2	6	0.01	7.8E-03	included
ILK	14171	ES	5.1	4	5.3	0.00	7.8E-03	included
TRAPPC13	72245	ES	9	7	10	0.03	7.9E-03	included
C21orf33	60806	ES	5	4	7	0.00	7.9E-03	excluded
LETMD1	21754	ES	4	2	5	0.03	7.9E-03	included
TMX2	15921	ES	3.3	2	4	-0.01	8.0E-03	excluded
NACA	100419	ES	3.3	2.2	4.2	0.00	8.0E-03	included
PFKFB4	64709	ES	11.1:11.2	10	12	-0.01	8.0E-03	excluded
NIPAL3	1112	ES	3	2	4	-0.01	8.0E-03	excluded
AARSD1	94557	ES	11	10	12	0.01	8.0E-03	included
CD97	47967	ES	6	5	7	0.04	8.0E-03	included
RAP1B	22958	ES	2	1.1	3.1	-0.04	8.0E-03	excluded
KLC1	29489	ES	14.1	13.2	15	-0.01	8.0E-03	excluded
C5orf45	74972	ES	5.1:5.2	2.1	6	-0.06	8.0E-03	excluded
HES4	5	ES	1.3	1.1	2	0.02	8.0E-03	included
GNA12	78639	ES	6	4	7	-0.01	8.0E-03	excluded
ATXN1	75423	ES	2	1	3	-0.03	8.0E-03	excluded
SIK3	18877	ES	16	15	17	-0.01	8.0E-03	excluded
SFTA3	27279	ES	3	2	4.1	-0.05	8.0E-03	excluded
MR1	9138	ES	4	3.2	5	0.03	8.0E-03	included
MFF	57799	ES	9	8	11	-0.06	8.0E-03	excluded
PPIP5K1	30268	ES	28	27	29	0.03	8.1E-03	included
IGFBP6	21939	ES	2	1	3.1	-0.01	8.1E-03	excluded
PNPLA2	13773	ES	2	1	3	-0.04	8.1E-03	excluded
CAMLG	73424	ES	2	1	3	0.02	8.1E-03	included
D2HGDH	58425	ES	2	1	3	0.01	8.1E-03	included
CSGALNACT1	82882	ES	7	5	8	0.02	8.1E-03	included
RNASEH1	52566	ES	2	1.1	3	0.00	8.1E-03	excluded
CCDC25	83177	ES	07:08.1	6	9	0.00	8.1E-03	included
OPN3	10431	ES	3	1	4	0.03	8.1E-03	included

ACOX1	43538	ES	4	2	5	0.01	8.1E-03	included
LBH	53112	ES	4	3.1	5	-0.03	8.2E-03	excluded
USP3	31038	ES	4	3.2	5	-0.03	8.2E-03	excluded
TMEM185A	90324	ES	2	1	3.1	-0.02	8.2E-03	excluded
SUMF1	62990	ES	3	2	4	-0.01	8.2E-03	excluded
TATDN3	9762	ES	5	4	6.1	-0.01	8.2E-03	excluded
GALM	53267	ES	3	2	4	-0.03	8.3E-03	excluded
SMPD4	55299	ES	11:12	10	14	-0.05	8.3E-03	excluded
MBNL1	67325	ES	6	5	7	0.00	8.3E-03	excluded
WDR41	72586	ES	3	2	4	-0.04	8.3E-03	excluded
CMPK1	2908	ES	3	1	4	0.00	8.3E-03	included
FLNA	499334	ES	37:38:39:41:43:44:45	36	46	0.00	8.3E-03	excluded
CERS5	21676	ES	4:08	1	9	-0.03	8.3E-03	excluded
MARCH2	47231	ES	5	4	6.1	0.00	8.4E-03	included
CDCA3	19985	ES	4	3.2	5	0.04	8.4E-03	included
CCDC18	3780	ES	28	27	29	-0.07	8.4E-03	excluded
SNX16	84312	ES	2	1	3	-0.04	8.4E-03	excluded
ABI1	11041	ES	11.1:11.2	9	13	-0.03	8.4E-03	excluded
PCNX	28194	ES	7:08	6	9	0.05	8.4E-03	included
GABARAP	38868	ES	1.2:1.3:1.5	1.1	2.1	0.04	8.4E-03	included
SEMA4F	54132	ES	5:6:7:8	4	9	-0.03	8.4E-03	excluded
PCCB	66902	ES	15	14	16	0.00	8.5E-03	included
SLC47A1	39738	ES	17	15	18.1	-0.05	8.5E-03	excluded
RASA1	72722	ES	1.2:2	1.1	4	0.00	8.5E-03	excluded
ATP5S	27469	ES	6	5.1	7.1	0.01	8.5E-03	included
ANAPC10	96806	ES	5	4	7	0.01	8.5E-03	included
HINT1	73217	ES	2.3:3.1:3.2	2.1	4	0.00	8.5E-03	included
PDE8A	32338	ES	11	9	12	-0.01	8.5E-03	excluded
PIEZO1	38024	ES	45	44	46	-0.01	8.6E-03	excluded
ALDOA	36045	ES	7.2	6.2	9	0.00	8.6E-03	included
ETFA	31944	ES	2:03	1	4	-0.07	8.6E-03	excluded
HIPK1	4317	ES	13	12	14	0.00	8.6E-03	included
CGGBP1	65669	ES	5.2	4	5.4	-0.03	8.6E-03	excluded
POSTN	25674	ES	19	16	20	0.02	8.6E-03	included
RNF7	67076	ES	2	1.2	3	-0.01	8.7E-03	excluded
COL3A1	206507	ES	45:46:00	44	47	0.00	8.7E-03	included
LONP2	36337	ES	3	2	4	0.01	8.7E-03	included
ZNF213	33456	ES	4	3.2	5	0.01	8.7E-03	included
BFAR	34099	ES	3	2	4	0.01	8.7E-03	included
MRPL52	26636	ES	3:4.1:4.2	2	5	0.01	8.7E-03	included
RAP1GDS1	70001	ES	3	2.2	4.1	-0.01	8.7E-03	excluded
MAP2	57227	ES	9.1:9.2:10:11	8	12	-0.03	8.7E-03	excluded
SH3D19	70836	ES	13	12	14	0.02	8.8E-03	included
HKR1	49489	ES	14:15	13	17.2	-0.04	8.8E-03	excluded
APTX	86087	ES	6.1:6.2:6.3:7.2:7.3	5.2	7.4	0.04	8.8E-03	included
NCOA4	11538	ES	13	12	14	0.00	8.8E-03	excluded
ARHGEF10L	862	ES	18	17	19	0.04	8.8E-03	included
DAZAP1	46477	ES	8	7.1	9	0.01	8.8E-03	included
SLC25A46	72946	ES	8	7.2	9	-0.01	8.8E-03	excluded
ADCK4	49950	ES	10	9	11.1	0.01	8.9E-03	included
TG	319465	ES	25:27:29:31:32:33:34:<br>35:37:38:39:40:41:42:	24	46	-0.01	8.9E-03	excluded
ADAM15	7900	ES	21.1:21.2:22.1:22.2	20	23	-0.01	8.9E-03	excluded
TM7SF2	16763	ES	3	2	4	0.00	8.9E-03	included
EDC3	31735	ES	3	1	5	-0.04	8.9E-03	excluded
DTNB	52866	ES	18	16	19	0.00	8.9E-03	included
SELENBP1	7620	ES	6	5	7	-0.01	8.9E-03	excluded
SPOP	42314	ES	2:03	1	6	-0.05	8.9E-03	excluded
PSMD12	43112	ES	2:03	1	4	-0.01	9.0E-03	excluded
RPS15A	34254	ES	4	3.3	5.1	0.00	9.0E-03	excluded
RAPGEF3	21354	ES	20	19	21	0.00	9.0E-03	included
ARID5A	54532	ES	4	3	5	0.04	9.0E-03	included

ACAD8	19559	ES	2:04	1	5	-0.04	9.0E-03	excluded
CALU	81714	ES	2	1	3	-0.01	9.1E-03	excluded
BICD1	21021	ES	7.2:8	7.1	9	0.06	9.1E-03	included
RIC8B	24161	ES	14:15:16	13	17	0.07	9.1E-03	included
ARHGAP10	70799	ES	21	20	22	0.02	9.1E-03	included
DNM1L	21060	ES	4	2	5	-0.01	9.1E-03	excluded
PTP4A2	1536	ES	3:05	2	6	-0.01	9.1E-03	excluded
CPSF4	80637	ES	4	3	5	-0.05	9.1E-03	excluded
POGK	8825	ES	4:05	3	6.1	-0.01	9.1E-03	excluded
LEF1	70292	ES	12	11	13	0.03	9.1E-03	included
VWA5A	19216	ES	1.2:1.3:2.1	1.1	2.2	-0.01	9.1E-03	excluded
BCAS4	59783	ES	4:05:06	3	7	-0.07	9.2E-03	excluded
BLVRA	79349	ES	2	1	3	-0.02	9.2E-03	excluded
TOM1L1	42540	ES	14	13	15	0.00	9.2E-03	excluded
SNRPN	93602	ES	8:09	7	10.3	0.04	9.2E-03	included
GABARAPL1	20399	ES	2.7:2.8:2.9:2.10:2.11:2.12:2.14	2.6	3	0.00	9.2E-03	excluded
RMDN2	53239	ES	4.2	3	6	-0.01	9.2E-03	excluded
KIF9	64504	ES	2.3:3	2.1	4	0.00	9.2E-03	included
ENOSF1	44474	ES	6	5	7	0.06	9.2E-03	included
YAF2	21149	ES	5.2:6:7	2	9.1	-0.02	9.2E-03	excluded
RPL6	399926	ES	1.2:2:3:4	1.1	5	0.03	9.2E-03	included
EIF3M	14856	ES	2.1:2.2:3	1	5	0.04	9.2E-03	included
BLNK	12674	ES	18	16	19	0.00	9.3E-03	excluded
SFI1	61867	ES	20	19	21	0.00	9.3E-03	included
MCFD2	53475	ES	5:06	3	7	0.01	9.3E-03	included
SFI1	61868	ES	13	12	14	0.03	9.3E-03	included
CDIPT	35973	ES	2.2	1.5	3.2	-0.06	9.3E-03	excluded
DDX11	20965	ES	25:26:27:28.1:28.2:29.	24	29.3	0.07	9.3E-03	included
ZNF814	95404	ES	3.1	1	9	0.03	9.3E-03	included
KCNJ16	43192	ES	5	3	6	-0.05	9.3E-03	excluded
CAMK2D	70405	ES	22	21.1	23	-0.03	9.3E-03	excluded
AHI1	77894	ES	31:32:00	30.1	33	-0.05	9.3E-03	excluded
MKNK1	2807	ES	16	15.2	17	0.03	9.3E-03	included
STX16	59984	ES	3	1.4	5.1	-0.04	9.3E-03	excluded
APBB3	73677	ES	6.5:6.6:6.8	6.2	6.9	0.00	9.3E-03	excluded
CERS5	21662	ES	10	9	11	0.01	9.4E-03	included
SERGEF	102749	ES	2	1.2	3	-0.03	9.4E-03	excluded
PLD3	49892	ES	3	1.2	5.2	0.04	9.4E-03	included
GSTO1	13046	ES	5	4	6	0.00	9.4E-03	included
ST3GAL5	54388	ES	3	1	5	-0.03	9.5E-03	excluded
EVL	29247	ES	6	5	7	0.00	9.5E-03	excluded
ESAM	19238	ES	2:03:04	1	5.1	0.00	9.5E-03	excluded
YIPF1	3074	ES	12	11	13	-0.02	9.5E-03	excluded
ERI2	34397	ES	4	3	5	-0.03	9.5E-03	excluded
IFI44	3557	ES	2	1	3	0.03	9.6E-03	included
SIRT3	13619	ES	2.2:3.1:3.2:4:5.3	2.1	6.1	0.01	9.6E-03	included
LAS1L	89322	ES	7	6	8	0.01	9.6E-03	included
TANK	55743	ES	3	2.2	4	-0.04	9.6E-03	excluded
LETMD1	21759	ES	3.2:4:5:6	2	7	-0.03	9.6E-03	excluded
SKA2	42749	ES	2:4.1:4.2	1.1	5	0.03	9.6E-03	included
SCEL	26084	ES	9	8	10	-0.01	9.6E-03	excluded
AGAP3	82353	ES	15:16	14	17	0.02	9.7E-03	included
ITPA	58578	ES	2:03	1.2	4	0.00	9.7E-03	excluded
TMEM67	84538	ES	4	3.2	6	0.10	9.7E-03	included
TTLL3	63211	ES	10	9	12.1	0.04	9.7E-03	included
PDLIM3	71369	ES	2	1	3	0.00	9.7E-03	included
KIAA1468	45700	ES	21	20	22	-0.02	9.7E-03	excluded
SECISBP2L	30513	ES	8	7	9	-0.05	9.8E-03	excluded
METTL23	43630	ES	2	1.4	3	0.04	9.8E-03	included
TERF1	84141	ES	7	6	8	0.03	9.8E-03	included
ALDOA	36049	ES	2	1.3	3	0.02	9.8E-03	included

POLR2H	67945	ES	5	4	6.2	0.00	9.8E-03	included
XPNPEP1	13072	ES	15	14	16	-0.01	9.8E-03	excluded
CRCP	79873	ES	4	3	5	-0.06	9.8E-03	excluded
FLNB	65419	ES	32.1	31	33	-0.03	9.8E-03	excluded
RASGEF1B	69697	ES	4	3	5.1	0.00	9.8E-03	excluded
PUF60	85504	ES	3	1	4	-0.01	9.8E-03	excluded
TMEM126B	18124	ES	2.1	1	4	0.00	9.9E-03	excluded
EXOSC8	25656	ES	2	1	3	0.01	9.9E-03	included
ASRGL1	16341	ES	3	2.2	4	-0.02	9.9E-03	excluded
APOPT1	29462	ES	2:03:04	1	5	0.03	9.9E-03	included
LENG8	51899	ES	5	4	6	-0.01	1.0E-02	excluded
PSMC5	43011	ES	2.1	1	2.3	0.02	1.0E-02	included
ENOSF1	44475	ES	3	2	4	-0.01	1.0E-02	excluded
LETMD1	21746	ES	3.1:3.2:3.3	2	4	-0.01	1.0E-02	excluded
ZNF621	64242	ES	3	2	4	-0.04	1.0E-02	excluded
ATG12	73038	ES	2	1.1	3	-0.03	1.0E-02	excluded
PACRGL	68902	ES	6:7:8:9	5	13.1	-0.01	1.0E-02	excluded
PEX10	267	ES	4.2:5.1	4.1	5.2	0.06	1.0E-02	included
ZNF737	48722	ES	2	1	3	-0.07	1.0E-02	excluded
ST7	81565	ES	5:6:7:8:9:10:11.1:12	1	13	0.01	1.0E-02	included
MPHOSPH6	37772	ES	5	4	6	-0.01	1.0E-02	excluded
GCSH	37755	ES	2	1	3	0.00	1.0E-02	included
APBB3	73684	ES	3:04	2	5	0.00	1.0E-02	included
RCBTB2	25862	ES	10	9	11	0.01	1.0E-02	included
CIDEB	26959	ES	2	1	3	0.06	1.0E-02	included
TUSC2	65013	ES	2.1:2.2:2.3	1	3.1	0.01	1.0E-02	included
CCNT1	21462	ES	7	6	8	0.03	1.0E-02	included
RNF8	76012	ES	9	8	10	-0.03	1.0E-02	excluded
RPS5	52441	ES	3:04:05	2.3	6	-0.03	1.0E-02	excluded
CMC2	37730	ES	7	5	9	0.00	1.0E-02	excluded
MAP2K7	47194	ES	2	1	3	0.04	1.0E-02	included
CCT6B	40224	ES	6	5	7	-0.04	1.0E-02	excluded
TDP1	28811	ES	11	10.1	12	-0.04	1.0E-02	excluded
INO80E	36016	ES	6.2:6.3	5	11	0.02	1.0E-02	included
TPP2	26212	ES	24	23	25	0.02	1.0E-02	included
MUC1	7959	ES	8	7	9	-0.01	1.0E-02	excluded
SRRM1	1128	ES	16	15	17	0.03	1.0E-02	included
TACC1	83447	ES	9	8	10.1	0.01	1.0E-02	included
TMX2	15903	ES	3.1:3.2:3.3:4:5.1:5.3	2	6	0.04	1.0E-02	included
IST1	37515	ES	13:14.1	12	14.2	0.05	1.0E-02	included
RNF135	40135	ES	4	3	5	-0.02	1.0E-02	excluded
SEC13	63362	ES	2.1:2.2	1.1	3	0.00	1.0E-02	excluded
NUDT22	16588	ES	2	1.4	3	0.01	1.0E-02	included
ODF2L	3674	ES	15:16	14	17	0.04	1.0E-02	included
MARK2	16541	ES	19	17	20	0.01	1.0E-02	included
CRELD2	62757	ES	9	8	10	0.00	1.0E-02	excluded
SPAG16	57338	ES	4	3.2	5	0.00	1.0E-02	excluded
TXNL4A	46288	ES	4:05	3	7.2	0.00	1.0E-02	excluded
FBXO22	31902	ES	2	1	3	0.01	1.0E-02	included
MS4A6A	16064	ES	5.1	4	7	0.00	1.0E-02	excluded
CTSA	59640	ES	3	2	4	0.00	1.0E-02	included
TOM1L1	42544	ES	5	4	6.1	0.00	1.0E-02	included
C14orf159	28843	ES	20	19	21	-0.01	1.0E-02	excluded
DMKN	49201	ES	11	6.4	12	0.02	1.0E-02	included
XPO1	53698	ES	4.2:5	4.1	6	-0.01	1.0E-02	excluded
FKBP3	27404	ES	3	2	4	-0.03	1.0E-02	excluded
FKBP8	48448	ES	4:5:6.1:6.2	3	7	0.00	1.0E-02	excluded
RILPL2	25073	ES	2	1	3	0.01	1.1E-02	included
CPSF3L	138	ES	2.1:2.2:3	1	4	-0.01	1.1E-02	excluded
PPP3CB	12158	ES	2	1	3	0.02	1.1E-02	included
ANKMY2	78857	ES	10	9	11	-0.01	1.1E-02	excluded
OCIAD2	69257	ES	6	5	7	0.00	1.1E-02	included

BCL7B	79952	ES	4	3	6	-0.01	1.1E-02	excluded
ASPSCR1	44260	ES	4	3	5	-0.01	1.1E-02	excluded
TMEM107	39124	ES	2:3.1:3.2:3.3:3.4:3.5:3.	1	3.7	0.00	1.1E-02	excluded
FAM104A	43210	ES	4	3.2	5	0.03	1.1E-02	included
ABHD3	44780	ES	04:05.1	3	6	-0.01	1.1E-02	excluded
TMEM185A	90322	ES	3.1:3.2:6.1	1	7	-0.06	1.1E-02	excluded
SULT1A1	35820	ES	3:4:6.2:7:8	2	9	-0.06	1.1E-02	excluded
HAUS7	90441	ES	4	3	5	0.01	1.1E-02	included
CMC2	37723	ES	9:10	5	12	-0.02	1.1E-02	excluded
AIFM1	90074	ES	3	2	4	0.00	1.1E-02	excluded
COX5A	31810	ES	5.1	4	6	0.00	1.1E-02	included
FXR1	67756	ES	2	1	5	-0.01	1.1E-02	excluded
FAM49B	85149	ES	7.1	6	9	0.01	1.1E-02	included
IL15	70678	ES	2	1.2	3	-0.03	1.1E-02	excluded
MAX	28008	ES	2	1.2	3	-0.02	1.1E-02	excluded
ARL13B	65692	ES	3.1:3.2	2	4	-0.05	1.1E-02	excluded
ABCD4	28377	ES	8.1:8.2	7	9	0.05	1.1E-02	included
NOX4	18233	ES	21	20	22.1	0.04	1.1E-02	included
HAUS5	49219	ES	11	10	12	0.01	1.1E-02	included
TOM1L2	39518	ES	5	4	6	0.01	1.1E-02	included
TATDN1	138623	ES	2:4.1:4.2:5:6	1.1	7	-0.04	1.1E-02	excluded
HSCB	61552	ES	2.1:2.2:3.1:3.2	1	5	0.05	1.1E-02	included
PAK4	49761	ES	2.3:3.2	2.2	4	-0.01	1.1E-02	excluded
PKM	31519	ES	2.2:2.3:4	2.1	5.1	0.00	1.1E-02	excluded
ERC1	19657	ES	16	15	17	0.01	1.1E-02	included
CEP57L1	77168	ES	10.1	9	12	-0.02	1.1E-02	excluded
TMEM189	59776	ES	2	1	3	-0.03	1.1E-02	excluded
ELP2	45226	ES	5:06	4.2	7	0.05	1.1E-02	included
COASY	41065	ES	1.2:1.3:1.5	1.1	1.6	0.09	1.1E-02	included
FBXO38	73980	ES	15.1:15.2	14	16	0.03	1.1E-02	included
MIF4GD	43420	ES	4:05	2	6.1	0.00	1.1E-02	included
NAA60	33537	ES	6.2	5	7	0.01	1.1E-02	included
RUFY2	11940	ES	4	3	5	0.02	1.1E-02	included
TBCE	10349	ES	8	7	9	-0.03	1.1E-02	excluded
POLR3D	83000	ES	7	6	8	0.00	1.1E-02	included
APBB3	73678	ES	6.6:6.8	6.2	6.9	0.00	1.1E-02	excluded
ARHGAP8	62628	ES	12	11	14	0.01	1.1E-02	included
MED16	46339	ES	12	11	13	0.00	1.1E-02	included
NAT10	14948	ES	2.2:3	1	4	0.00	1.1E-02	excluded
ZNF644	3733	ES	4:05	3	6	-0.02	1.1E-02	excluded
GPBP1	72131	ES	3.1:4	2	6	0.03	1.1E-02	included
USP4	64854	ES	9	6	10	-0.02	1.1E-02	excluded
STARD3NL	79285	ES	6	5	7	-0.01	1.1E-02	excluded
LTBP4	49933	ES	26:27:00	25	28	0.01	1.1E-02	included
ARL6IP5	65577	ES	3	1	4.1	-0.03	1.1E-02	excluded
EPDR1	79273	ES	5	3	6	0.02	1.1E-02	included
ARHGAP12	11153	ES	8:09	7	10	-0.03	1.1E-02	excluded
ARL13B	65690	ES	4	2	5.1	0.03	1.1E-02	included
ARPC4	63185	ES	3	2	4	0.00	1.1E-02	excluded
THSD1	25996	ES	4	3	5	0.05	1.1E-02	included
VRK2	53646	ES	7	6.2	8	-0.02	1.1E-02	excluded
ERCC1	50446	ES	4	3.2	5	0.00	1.1E-02	included
ANAPC15	17575	ES	3.2:3.3	1.2	4.1	-0.01	1.1E-02	excluded
SEMA4A	8192	ES	3.2	2	4	-0.02	1.2E-02	excluded
C19orf47	49882	ES	6	5	7	-0.03	1.2E-02	excluded
NDUFAB1	35613	ES	2	1	3	0.00	1.2E-02	excluded
RBCK1	58453	ES	8.1:8.2	7	9	0.01	1.2E-02	included
SLC38A6	27793	ES	4	3	5	-0.03	1.2E-02	excluded
SIDT2	18891	ES	14	13	15.1	-0.02	1.2E-02	excluded
HDAC11	63490	ES	6.1:6.2:7:8	5	9	-0.01	1.2E-02	excluded
COQ9	36557	ES	5	4.2	6	0.00	1.2E-02	included
GPNMB	78967	ES	4.1	3	5	0.00	1.2E-02	excluded

FAM227B	30544	ES	8	7	9.1	0.08	1.2E-02	included
TMEM66	83261	ES	05:06.1	3	6.2	0.00	1.2E-02	excluded
ATF2	56063	ES	17:18.1	16	18.2	-0.02	1.2E-02	excluded
GNPTG	33076	ES	4	3	6	-0.03	1.2E-02	excluded
ETFA	31946	ES	2	1	3	-0.03	1.2E-02	excluded
RRNAD1	8311	ES	5	4	6	0.02	1.2E-02	included
PPP6R2	62825	ES	17	16	18	-0.02	1.2E-02	excluded
RQCD1	57497	ES	7	6	8	-0.04	1.2E-02	excluded
POLR2H	67948	ES	2.1	1	3	0.00	1.2E-02	included
CNOT10	63824	ES	14	13	15	-0.01	1.2E-02	excluded
ACTR1A	12957	ES	9	8	10	0.00	1.2E-02	included
MALL	54923	ES	3:04	1	5	0.00	1.2E-02	excluded
MS4A7	16089	ES	3	2	4	-0.08	1.2E-02	excluded
TARDBP	635	ES	3	2	4	-0.05	1.2E-02	excluded
GPBP1	72127	ES	9	8	10	0.02	1.2E-02	included
RNF121	17450	ES	4:6.1:6.2	3	7	0.01	1.2E-02	included
KLC1	29469	ES	16	15	18	-0.04	1.2E-02	excluded
USP4	64853	ES	23	22	24	-0.01	1.2E-02	excluded
TBC1D3	40564	ES	17	16	18.1	0.02	1.2E-02	included
SFTA3	27263	ES	4.1:4.2	2	5	0.03	1.2E-02	included
C20orf96	58439	ES	3	2.2	4	-0.02	1.2E-02	excluded
TMEM179B	16427	ES	4	3	5	0.00	1.2E-02	excluded
B3GALNT2	10354	ES	4	3	5	-0.02	1.2E-02	excluded
VPS29	24441	ES	3.1:3.2:4	1	5	0.00	1.2E-02	included
DTWD1	30557	ES	6	3	8	-0.03	1.2E-02	excluded
EPB41L2	77557	ES	20.1:20.2	14	21	0.00	1.2E-02	excluded
MEMO1	95465	ES	10	9	11	0.01	1.2E-02	included
KNTC1	24977	ES	58	57	59	-0.01	1.2E-02	excluded
ZFYVE27	12744	ES	2.1:2.2:3	1.1	4	0.00	1.2E-02	included
NUDT1	78611	ES	3.2	1	4	0.02	1.2E-02	included
PPP1R21	53525	ES	19	18	20	0.01	1.2E-02	included
ARMCX6	89659	ES	2:03	1	4	-0.04	1.2E-02	excluded
CIRH1A	37277	ES	10:11:12	9	13	0.00	1.2E-02	included
PCMT1	78114	ES	3.2	2	5	0.00	1.2E-02	excluded
SUOX	22343	ES	2:03	1	5.2	0.03	1.2E-02	included
RPL35	87540	ES	4.1	3	5	0.00	1.2E-02	included
TCTN1	93315	ES	13:14:15:16.1:16.2:17.	12	17.2	0.06	1.2E-02	included
DYM	45472	ES	3:05	2	6	-0.08	1.2E-02	excluded
PIGS	39919	ES	4	3	5	0.00	1.2E-02	included
PRUNE	7542	ES	8	7	9	-0.01	1.2E-02	excluded
UBP1	63867	ES	8	7	9	-0.03	1.2E-02	excluded
PSMD9	24914	ES	3.1	2.2	4	0.00	1.2E-02	included
PDE9A	60721	ES	6	5	7	0.03	1.2E-02	included
SRSF11	3387	ES	5:6.1:6.2:6.3:6.4	4.2	6.5	-0.05	1.2E-02	excluded
SNX1	139183	ES	4.1:4.2:5:6:8:9:10.1:1<br>0.2:11:12:14:15	3	16.1	0.00	1.2E-02	excluded
MARCH6	71564	ES	4	3	5	-0.01	1.2E-02	excluded
TSPAN4	13795	ES	10	9	11	0.00	1.3E-02	included
NAP1L1	23497	ES	2	1	3	0.00	1.3E-02	included
YPEL5	53106	ES	3.1:3.2	1	5	0.01	1.3E-02	included
PDGFA	78504	ES	6	5	7	0.06	1.3E-02	included
NUBP2	33139	ES	2	1	3	-0.02	1.3E-02	excluded
GLG1	234290	ES	13:14:15:16:17:18:19:<br>20:21:22:23:24:25:26:	12	27.2	-0.04	1.3E-02	excluded
MIER1	3340	ES	6.1	5	7	-0.01	1.3E-02	excluded
UQCRB	84614	ES	6.1	4	6.4	0.00	1.3E-02	included
TNK2	68211	ES	7	6	8	0.02	1.3E-02	included
APH1B	31024	ES	5	4	6	-0.01	1.3E-02	excluded
CERS4	47210	ES	2	1	3.1	-0.02	1.3E-02	excluded
SNRNP25	32783	ES	4	3	5	0.00	1.3E-02	included
CHID1	13820	ES	4.2	3.1	5.2	0.02	1.3E-02	included
NBPF10	5531	ES	17:18	16	19	0.03	1.3E-02	included

PABPC1L	59497	ES	14	13	15	-0.01	1.3E-02	excluded
PIGT	59575	ES	2.1	1	4	-0.07	1.3E-02	excluded
RNH1	13678	ES	03:04.2	1	4.3	0.03	1.3E-02	included
SS18	44962	ES	8.2	3	9	0.00	1.3E-02	excluded
NECAP2	825	ES	6	5	7	-0.01	1.3E-02	excluded
ATRIP	64661	ES	3	2	4	-0.02	1.3E-02	excluded
C5orf45	74971	ES	4:5.1:5.2	2.1	6	0.01	1.3E-02	included
TFR2	80980	ES	8:09:10	7	11	-0.02	1.3E-02	excluded
B4GALT4	66287	ES	6.2:7:8.1:8.2:9.1:9.2:9.	6.1	9.4	-0.07	1.3E-02	excluded
SEPT2	58376	ES	3:04	2	7	-0.02	1.3E-02	excluded
NFIB	85884	ES	14	13	15	0.05	1.3E-02	included
RPGRIP1L	36425	ES	21	20	22	0.08	1.3E-02	included
PSEN1	28271	ES	5	4.1	7	-0.02	1.3E-02	excluded
ADRM1	60057	ES	3	2	4	0.00	1.3E-02	included
MATR3	96937	ES	8	7.2	9	0.03	1.3E-02	included
ZBTB7B	7879	ES	3:04	2.2	6.2	0.04	1.3E-02	included
LDHA	14616	ES	9	8.3	10.1	0.00	1.3E-02	included
EZR	78290	ES	4	3	5	0.00	1.3E-02	excluded
DMKN	49166	ES	8:9:11:12	7	13	0.06	1.3E-02	included
PGPEP1	48424	ES	2	1	4	0.01	1.3E-02	included
IPO7	188728	ES	2	1	3	-0.06	1.3E-02	excluded
PDDC1	13745	ES	7:8.1:8.2	6.2	8.3	0.01	1.3E-02	included
EPRS	9853	ES	14:15	13	16	0.00	1.3E-02	included
EPB41L2	77556	ES	17:18:20.1:20.2	14	21	-0.01	1.3E-02	excluded
GNB2	80995	ES	3.2	1	4.1	0.00	1.3E-02	included
SUMF2	79811	ES	3:4:5.1:5.2	2	6	0.00	1.3E-02	included
SYNE2	27854	ES	105	103	109	0.00	1.3E-02	included
FAM86B1	82707	ES	06:07.1	3.2	8.1	-0.06	1.3E-02	excluded
NPIPA8	100474	ES	26	25	27	0.00	1.3E-02	excluded
ZNF415	51670	ES	7.1:7.2:8.1	6.1	8.2	0.08	1.3E-02	included
LMAN2L	54569	ES	6	5	7	0.01	1.3E-02	included
EIF4G1	67902	ES	2.2:2.4:3.1:3.2:5	1	6	0.06	1.3E-02	included
EEF1D	85455	ES	5:7.2:8.1	1	8.2	0.00	1.3E-02	excluded
TMUB2	41823	ES	2.5:3	1	4.3	-0.05	1.3E-02	excluded
YAF2	21158	ES	3.1:3.2:5.2:6	2	9.1	-0.02	1.3E-02	excluded
PRKAR1A	43151	ES	3.2	2.1	3.4	0.01	1.3E-02	included
CAST	72861	ES	8.1:8.2	7.1	9	-0.01	1.3E-02	excluded
TMPRSS4	18957	ES	11	10	12	0.02	1.3E-02	included
UBR7	29026	ES	2	1	3	0.01	1.3E-02	included
RNF14	73850	ES	6:07	4	8	-0.03	1.3E-02	excluded
USP3	31064	ES	2	1	3.2	-0.04	1.3E-02	excluded
HYAL2	65008	ES	1.3	1.1	1.5	0.04	1.3E-02	included
LAMA5	60063	ES	76	75	77	0.00	1.3E-02	included
L3MBTL2	62413	ES	2	1	3.1	-0.03	1.3E-02	excluded
EEF1D	85461	ES	05:07.2	1	8.1	0.00	1.3E-02	excluded
APTX	86082	ES	6.1:6.2	5.2	7.1	0.03	1.3E-02	included
UBE2C	59606	ES	02:03.2	1.4	4	0.00	1.3E-02	excluded
HPS5	14593	ES	1.3:2	1.2	3	-0.03	1.3E-02	excluded
FBRS	36170	ES	3:04	2	5	0.01	1.3E-02	included
VPS53	38231	ES	5	4	6	0.02	1.3E-02	included
ANO1	17389	ES	15	13	16	0.00	1.3E-02	excluded
TPM3	7796	ES	5.1:5.2:5.3	3.2	6.1	0.00	1.3E-02	included
DCAF11	26846	ES	1.2:2.2:2.3	1.1	2.4	0.02	1.3E-02	included
TUSC3	82772	ES	10:11	9	12	0.01	1.3E-02	included
FAM110A	58470	ES	3	1	4.2	-0.06	1.3E-02	excluded
CEP76	44708	ES	7	6	8	-0.04	1.4E-02	excluded
ATXN3	28902	ES	11	10.2	13.2	-0.01	1.4E-02	excluded
TMEM248	79904	ES	5	4	6	0.00	1.4E-02	included
PTBP1	94868	ES	11	10	12	0.01	1.4E-02	included
ECHDC2	3024	ES	9.1	8.1	10	-0.03	1.4E-02	excluded
C5orf45	74951	ES	5.1:5.2	4	6	0.00	1.4E-02	included
UBXN11	1258	ES	3:4:5:7:8:9	2	10	0.07	1.4E-02	included

LARP4	21703	ES	10	9	11	-0.03	1.4E-02	excluded
CTAGE5	27379	ES	5	4.3	6	0.00	1.4E-02	included
TOX2	59455	ES	9	8	10	-0.01	1.4E-02	excluded
DECR1	84412	ES	2:03	1	4.1	-0.01	1.4E-02	excluded
GLIS3	85749	ES	4	2	5	0.05	1.4E-02	included
MAPKAP1	87584	ES	7	6	8	0.00	1.4E-02	excluded
SMPD4	55293	ES	12	11	14	-0.04	1.4E-02	excluded
ARHGEF9	89306	ES	9	8	10	-0.01	1.4E-02	excluded
STRN3	27096	ES	8:09:10	7	11	-0.06	1.4E-02	excluded
KNSTRN	29976	ES	7	6	8	0.00	1.4E-02	included
C9orf3	86946	ES	19.1	18	20	-0.02	1.4E-02	excluded
ZDHHC16	12706	ES	5	4	6	0.00	1.4E-02	included
SHROOM1	73312	ES	3.1	2	3.3	-0.05	1.4E-02	excluded
STAT1	56596	ES	9	8	10	0.00	1.4E-02	included
ODF2L	3678	ES	4	3.2	5	-0.02	1.4E-02	excluded
SMARCA4	47611	ES	35	34	36.2	0.01	1.4E-02	included
TYW3	3472	ES	4	3	5	0.00	1.4E-02	included
ZNF207	40206	ES	7	6	8	-0.01	1.4E-02	excluded
FEZ2	53196	ES	8:09	7	10	0.04	1.4E-02	included
RBM28	81665	ES	2:3:4:5	1	6	0.01	1.4E-02	included
OGG1	63171	ES	05:06.1	3	8	-0.01	1.4E-02	excluded
SH3GL1	46800	ES	4.1:4.2	3	5.1	0.00	1.4E-02	included
SFXN2	12968	ES	8	7	9	0.02	1.4E-02	included
CMTM3	36813	ES	2.3:5:6:7.1:7.2:9.1	2.2	9.2	-0.01	1.4E-02	excluded
PACRGL	68882	ES	11:12	9	13.1	-0.07	1.4E-02	excluded
LY6E	85390	ES	3	2	5.2	0.00	1.4E-02	excluded
IL17RE	63249	ES	3:04	2.2	5	0.03	1.4E-02	included
EYA3	1369	ES	7	6	8	-0.05	1.4E-02	excluded
PDHB	65456	ES	2	1	3	0.01	1.4E-02	included
TMX2	15905	ES	3.3:4:5.1:5.3	2	6	0.00	1.4E-02	included
MUTYH	2603	ES	8	7	9	0.01	1.4E-02	included
SIDT2	18892	ES	11	10	12	0.01	1.4E-02	included
RNF7	67079	ES	2	1.1	3	0.02	1.4E-02	included
DNASE1L1	90581	ES	2.2	1	3.2	-0.04	1.4E-02	excluded
NT5C3B	40954	ES	3	2	4	-0.03	1.4E-02	excluded
ZNF664	25126	ES	3:04	2.2	5	-0.05	1.4E-02	excluded
FAM219B	31799	ES	3.2	2	4	-0.01	1.4E-02	excluded
RPS9	51821	ES	4.3	4.1	4.5	0.01	1.4E-02	included
FLOT2	40013	ES	5	2	6	0.01	1.4E-02	included
KRTCAP3	53016	ES	2.2	1	3	0.01	1.4E-02	included
SNX6	27163	ES	4	3	5	0.01	1.4E-02	included
MXRA8	146	ES	4	3	5	0.01	1.4E-02	included
CHEK2	61528	ES	14	13	15	0.01	1.5E-02	included
DTYMK	58406	ES	3	2	4	0.01	1.5E-02	included
DUSP22	75137	ES	2:03	1	6	-0.01	1.5E-02	excluded
KBTBD3	18558	ES	02:03.2	1	5	0.00	1.5E-02	included
C10orf68	11182	ES	7	5	8	0.02	1.5E-02	included
PACRGL	68907	ES	6	5	7	-0.01	1.5E-02	excluded
C18orf8	44834	ES	3	2	4	-0.01	1.5E-02	excluded
SCAP	64517	ES	4:5:6:7:8	3	9	0.00	1.5E-02	excluded
TUBB6	44679	ES	4.2:5.1:5.2	3	5.3	-0.01	1.5E-02	excluded
DMKN	49184	ES	7:8:9:11:12	6.4	13	0.07	1.5E-02	included
DCAF11	26839	ES	2.5:3.1	2.4	3.2	-0.01	1.5E-02	excluded
IQCK	93918	ES	5	4	7.1	-0.06	1.5E-02	excluded
TSNARE1	85346	ES	3:04	2	5	-0.02	1.5E-02	excluded
MSANTD3	87083	ES	3	2	4	-0.01	1.5E-02	excluded
CRAT	87828	ES	2:03	1	4.1	-0.05	1.5E-02	excluded
NUP107	22962	ES	22:23	21	24	0.00	1.5E-02	excluded
HDGFRP3	32274	ES	2	1	4	-0.01	1.5E-02	excluded
SELENBP1	7625	ES	3	2.2	4.1	0.00	1.5E-02	included
NIP7	37283	ES	4	3.1	5	-0.01	1.5E-02	excluded
HPS5	14592	ES	2	1.3	3	-0.04	1.5E-02	excluded

HDLBP	58349	ES	5	4	6	0.00	1.5E-02	excluded
SERP1	67274	ES	3.1	2	4	0.00	1.5E-02	included
RPS3A	70823	ES	2.1:2.2:3.1:3.2	1.3	4.1	0.03	1.5E-02	included
WARS	29288	ES	3:04	2.3	7	-0.15	1.5E-02	excluded
PLEKHM1	41977	ES	4.1	3	5	0.01	1.5E-02	included
MBD1	45521	ES	13.2	12	14	-0.08	1.5E-02	excluded
COL1A2	484905	ES	15:16:17:18:19:20:21:<br>22:23:24:25:26:27:28:	14	38	-0.03	1.5E-02	excluded
AKT2	49876	ES	3.1:3.2	1	4	0.00	1.5E-02	excluded
LETMD1	21753	ES	3.2:4	2	5	0.01	1.5E-02	included
DCN	23657	ES	7:8:9:10:11	3	12	0.00	1.5E-02	excluded
C12orf23	24185	ES	2.2	1	4.2	0.04	1.5E-02	included
TMEM18	52526	ES	3	1	4	-0.02	1.5E-02	excluded
EIF4ENIF1	61860	ES	6:07	5	8	0.00	1.5E-02	included
STX8	39219	ES	3:04	2	5	0.00	1.5E-02	included
FAM13B	73501	ES	3	2	4	-0.04	1.5E-02	excluded
EIF3K	107086	ES	5	4	7.1	0.05	1.5E-02	included
CLK4	74875	ES	2.2:2.3:3	2.1	4	-0.03	1.5E-02	excluded
RANBP3	46995	ES	2:4.1:5:6:7:8	1	10.2	-0.08	1.5E-02	excluded
EPSTI1	25763	ES	2	1	3	0.01	1.5E-02	included
LTBR	19854	ES	8	7	9.1	0.01	1.5E-02	included
TBCK	70267	ES	8	7	9.1	0.03	1.5E-02	included
GLTP	24362	ES	3.1	2	4.1	0.00	1.5E-02	excluded
FAM193A	68587	ES	6	5	7	-0.04	1.5E-02	excluded
PAF1	49813	ES	2	1	3	0.00	1.5E-02	included
TRDMT1	10887	ES	5	4	6	0.02	1.5E-02	included
MUTYH	2614	ES	6.4:6.5:7:8:9:10:11:12<br>:13:14:15:16:17:18	5	19	0.00	1.5E-02	included
CTNNA1	73568	ES	3	2	4	0.00	1.5E-02	excluded
PAN3	25552	ES	5	4	6	-0.04	1.5E-02	excluded
PARP8	71988	ES	4	2.2	8	0.00	1.5E-02	included
PQLC1	46268	ES	3	2	5	0.00	1.5E-02	excluded
ODF2L	3676	ES	12	11	13	0.01	1.5E-02	included
DBI	55112	ES	1.2:1.3:3.1	1.1	3.2	0.05	1.5E-02	included
RPS2	33174	ES	2	1.3	3	0.00	1.5E-02	included
CREB1	57181	ES	5	4	6	0.04	1.5E-02	included
CASP8	56824	ES	9:10	8	11	-0.02	1.5E-02	excluded
EPSTI1	25759	ES	12	11	13	-0.02	1.5E-02	excluded
PCCA	26190	ES	22	21	23	0.01	1.6E-02	included
ZZZ3	3527	ES	6	5	7	-0.01	1.6E-02	excluded
CRYZL1	60462	ES	7	6	9	0.00	1.6E-02	excluded
CALU	81711	ES	3:05	1	6	0.00	1.6E-02	included
DECR2	32887	ES	5.1:5.2:5.3	4.2	6	-0.03	1.6E-02	excluded
FAM98C	49644	ES	4:05:06	3	7	0.00	1.6E-02	included
MAPK3	36088	ES	6.1	5	7.1	0.00	1.6E-02	excluded
HDAC7	21379	ES	4	2	5.2	-0.03	1.6E-02	excluded
ADSL	62342	ES	13	12	14	0.01	1.6E-02	included
MAGI1	65533	ES	19.1:19.2	18	20	-0.05	1.6E-02	excluded
HAUS2	30188	ES	5.1	3	6	0.01	1.6E-02	included
ABCD4	28383	ES	6	5	7	0.00	1.6E-02	excluded
ATP6V1D	28054	ES	2	1	3	0.00	1.6E-02	included
ACTR3B	82418	ES	3	2	4	0.02	1.6E-02	included
LDHA	14635	ES	4	2.4	6.1	0.00	1.6E-02	included
SLC6A6	63528	ES	10	9	11	-0.02	1.6E-02	excluded
FLOT2	40009	ES	3:06	2	7	0.01	1.6E-02	included
SCEL	26087	ES	6	5	7	0.01	1.6E-02	included
PCYT2	44229	ES	12	11	13	0.01	1.6E-02	included
CARS2	26265	ES	4	3	5	-0.02	1.6E-02	excluded
ZNF283	50252	ES	2	1	3.2	0.06	1.6E-02	included
C1orf86	249	ES	7.1:8:9	5	10	-0.04	1.6E-02	excluded
MAD2L1	70468	ES	3	2	4	0.01	1.6E-02	included
ADCK1	28635	ES	4	3	5	-0.04	1.6E-02	excluded

NECAP2	824	ES	8	7	9.1	0.00	1.6E-02	included
EFNA1	7939	ES	3	2	4	0.01	1.6E-02	included
TRAP1	33632	ES	2	1	4	-0.01	1.6E-02	excluded
MARK2	16538	ES	19	18	20	0.04	1.6E-02	included
TMBIM4	22904	ES	2.2:2.3:4.1	1	4.2	0.09	1.6E-02	included
SLC38A6	27792	ES	8	7	9	-0.03	1.6E-02	excluded
ORC4	55533	ES	3	2.1	4	-0.01	1.6E-02	excluded
LRRCC1	84322	ES	2.2	1.1	3	0.07	1.6E-02	included
ZMYND11	10586	ES	12	11.2	13	0.00	1.6E-02	included
DHX40	42779	ES	3	2.2	4	0.01	1.6E-02	included
TMEM175	68433	ES	2:3:4.1:4.2	1	5.1	0.04	1.6E-02	included
EGFL7	88191	ES	5	3.2	6	-0.02	1.6E-02	excluded
SNX1	139185	ES	6:8:9:10.1:10.2:11:12:	3	16.1	0.00	1.6E-02	excluded
SUMF2	79794	ES	6	4	7	-0.06	1.6E-02	excluded
SH2B1	35869	ES	9.2:10	9.1	11	0.01	1.6E-02	included
PIGB	30721	ES	2:03	1	4	0.01	1.6E-02	included
DERL2	38707	ES	3:4.1:5.1:5.2	2	6	0.00	1.6E-02	excluded
FAM114A2	74204	ES	1.3:1.4	1.1	2.1	0.02	1.6E-02	included
DHX30	399746	ES	18	17	19	0.01	1.6E-02	included
PRKCQ	10705	ES	4	3	5	0.02	1.6E-02	included
C1RL	20076	ES	2	1	3.1	0.01	1.6E-02	included
DOCK3	120842	ES	34	33	35	-0.01	1.6E-02	excluded
NPRL3	32804	ES	5	4	6	-0.02	1.6E-02	excluded
MORF4L2	89773	ES	5.2:5.3	3.2	6.2	0.03	1.6E-02	included
STRADA	42980	ES	2.2:3	2.1	6	-0.04	1.6E-02	excluded
COG6	25714	ES	2:03	1	4.1	-0.01	1.6E-02	excluded
BACE2	60652	ES	7	6	8	0.00	1.6E-02	excluded
TSR1	38389	ES	2	1	3	0.01	1.6E-02	included
CCL4L2	40467	ES	2	1	3.3	0.03	1.6E-02	included
PECR	57401	ES	4	3	5	0.00	1.6E-02	excluded
ERI3	2552	ES	3	2.2	5	0.01	1.6E-02	included
IFT88	25429	ES	5	4	6	0.02	1.6E-02	included
PAX8	533938	ES	8	7	11	0.04	1.6E-02	included
GTF2E2	83297	ES	3:04	2	5	0.00	1.6E-02	excluded
ARL13B	65693	ES	3.2	2	4	-0.05	1.6E-02	excluded
SEC24D	70445	ES	27	26	28	-0.03	1.6E-02	excluded
DTNB	52864	ES	20	19	21	0.00	1.6E-02	excluded
MLF1	67442	ES	3	1.1	5	-0.01	1.6E-02	excluded
UBAP2	86137	ES	8:09	7	10	-0.01	1.6E-02	excluded
MBNL1	67311	ES	11	10	13	-0.02	1.7E-02	excluded
ZNF561	47365	ES	7	6.2	8	0.01	1.7E-02	included
ARHGEF28	72494	ES	37	36.1	38	0.03	1.7E-02	included
TBC1D3F	40555	ES	10	9	11.1	0.02	1.7E-02	included
WDSUB1	55692	ES	10	9	11	0.01	1.7E-02	included
MTO1	76748	ES	8	7	9	-0.03	1.7E-02	excluded
MON1B	37656	ES	2:3.2:3.3	1	4	0.00	1.7E-02	included
C12orf52	24621	ES	2.1:2.2	1	3	-0.01	1.7E-02	excluded
LMAN2L	54574	ES	5	4	7	0.02	1.7E-02	included
BBS4	31580	ES	1.3	1.1	2	0.01	1.7E-02	included
C21orf59	60362	ES	3	2	4	0.00	1.7E-02	included
GAB1	70700	ES	8	7	9	-0.05	1.7E-02	excluded
ARHGEF7	26285	ES	25.1	24	26	0.00	1.7E-02	excluded
NASP	2731	ES	6:7.2:8:9	3	10	0.01	1.7E-02	included
CERS4	47206	ES	10:11	9	12	0.00	1.7E-02	excluded
ZMYND11	10591	ES	7	6	8	-0.01	1.7E-02	excluded
TOM1L2	39511	ES	15	13	16	0.00	1.7E-02	excluded
FAM172A	72786	ES	4	3	5	0.02	1.7E-02	included
ARID4B	10343	ES	18	17	19	-0.02	1.7E-02	excluded
KIF21A	21077	ES	18	17	19	0.02	1.7E-02	included
ACOT8	59633	ES	02:03.2	1	4	0.07	1.7E-02	included
EIF6	59076	ES	2.3:2.4:3	2.2	4	0.01	1.7E-02	included
UGP2	53760	ES	5	1	6	-0.01	1.7E-02	excluded

GALE	1077	ES	3	2	4	0.01	1.7E-02	included
CTBP2	13417	ES	5	2	6	-0.02	1.7E-02	excluded
UBE2D2	73622	ES	3.2	2	4	0.01	1.7E-02	included
MYLIP	75422	ES	2	1	3	0.00	1.7E-02	included
ERMARD	78489	ES	3	1	4.1	-0.02	1.7E-02	excluded
TOLLIP	13826	ES	7	6	8	0.00	1.7E-02	excluded
SNX1	139188	ES	6:7:8:9:10.2:11:12:14:	3	16.1	0.00	1.7E-02	included
ACTR2	53799	ES	4	2	5	0.00	1.7E-02	excluded
PARD3	11210	ES	24	22	25	0.02	1.7E-02	included
EVC	68701	ES	23	22.1	24	-0.03	1.7E-02	excluded
PDCD4	13086	ES	3	2	4	-0.01	1.7E-02	excluded
LETMD1	21747	ES	3.2:3.3	2	4	-0.05	1.7E-02	excluded
RPUSD1	33015	ES	2.2	1	3.1	0.01	1.7E-02	included
TOM1L2	39512	ES	14	13	15	-0.01	1.7E-02	excluded
METTL22	33897	ES	4	3.4	5	-0.05	1.7E-02	excluded
MAP4	64557	ES	19:20	18	21	0.00	1.7E-02	excluded
KDM4C	85833	ES	12	11	13	-0.03	1.7E-02	excluded
ZNF664	25133	ES	2.2	1.4	5	-0.03	1.7E-02	excluded
PCNP	65959	ES	2.1:2.2:2.3	1	3	0.03	1.7E-02	included
IRAK4	21254	ES	4:05	3	6	0.06	1.7E-02	included
ETFA	31940	ES	3:04	1	5	0.00	1.7E-02	included
DNMT1	47475	ES	5	4.2	6	0.03	1.7E-02	included
SULT1A1	235342	ES	3:08	2	9	-0.06	1.7E-02	excluded
TRIP10	47082	ES	11.2:12.1	10	12.2	0.00	1.8E-02	excluded
TPRA1	66611	ES	10	8	11	-0.02	1.8E-02	excluded
SFI1	61877	ES	3	2.2	6	-0.05	1.8E-02	excluded
CAMK2G	12249	ES	13	12	14	0.01	1.8E-02	included
NCOA4	11540	ES	7	6	8	0.00	1.8E-02	included
TRAPPC4	19063	ES	3.1:3.2	2.3	4.1	0.00	1.8E-02	included
C12orf73	24073	ES	4.1	2.4	5	0.05	1.8E-02	included
NDUFS4	72011	ES	4.2	3	5	0.00	1.8E-02	excluded
CBWD6	86492	ES	2	1	3.1	-0.01	1.8E-02	excluded
UGDH	69069	ES	6	5.2	7	0.00	1.8E-02	excluded
CCDC53	24017	ES	11	9	12	-0.01	1.8E-02	excluded
ZNF721	68351	ES	4.1:5	3	6	-0.02	1.8E-02	excluded
PCNXL4	27768	ES	11	10	12	-0.01	1.8E-02	excluded
ZSWIM7	39394	ES	6	5.2	7.1	0.03	1.8E-02	included
VCL	12254	ES	19	18.2	20	-0.02	1.8E-02	excluded
DCAF6	8884	ES	11	10	13.1	0.03	1.8E-02	included
UBE2V1	59761	ES	3:04	2	5	-0.03	1.8E-02	excluded
CS	22418	ES	6	5.2	7	-0.05	1.8E-02	excluded
VPS29	24443	ES	2:3.1:3.2	1	5	0.00	1.8E-02	excluded
KCTD20	75982	ES	3	2	4	0.02	1.8E-02	included
ZNF280D	30779	ES	10.1:10.2	9.2	11	-0.02	1.8E-02	excluded
SNX11	42185	ES	1.2:2	1.1	3.1	0.07	1.8E-02	included
INIP	87253	ES	2	1	3	0.01	1.8E-02	included
VAV3	3934	ES	19	18.2	21	-0.01	1.8E-02	excluded
BID	61007	ES	5	2	7	-0.06	1.8E-02	excluded
THAP9	69749	ES	2:03	1	4.1	0.04	1.8E-02	included
TMEM66	83269	ES	1.2:2.2	1.1	2.3	-0.04	1.8E-02	excluded
ABI2	57070	ES	3	1	5.1	-0.01	1.8E-02	excluded
HMGXB4	61945	ES	5	4	6	0.05	1.8E-02	included
ZC3H14	28723	ES	13:14.1:14.2	10	15	0.04	1.8E-02	included
SERPINA1	29131	ES	1.2:2.1:2.2:2.4:2.5	1.1	3.2	0.00	1.8E-02	included
GUCD1	61415	ES	6	5	7.2	0.00	1.8E-02	excluded
IMPA1	84302	ES	2	1	3	-0.02	1.8E-02	excluded
ANKRD54	62166	ES	5.2	4	6	-0.04	1.8E-02	excluded
SDCCAG3	88163	ES	2:03	1	4	-0.04	1.8E-02	excluded
KARS	37637	ES	4:05	3	6	0.00	1.8E-02	included
CITED1	89454	ES	4	2.2	5	-0.04	1.8E-02	excluded
ARPC2	57444	ES	6	5	7	0.00	1.8E-02	excluded
ZNF92	79851	ES	2	1	3	0.05	1.8E-02	included

INO80E	36011	ES	6.3:7:8:9:10	5	11	0.03	1.8E-02	included
MADD	15721	ES	23.2	22	24	-0.01	1.8E-02	excluded
UBXN11	1255	ES	3:4:5:6:7:8:9	2	10	0.07	1.8E-02	included
C1orf50	2116	ES	1.3:2.2	1.1	3	0.01	1.8E-02	included
MPV17	52974	ES	6.3	3.2	7	-0.03	1.8E-02	excluded
C16orf89	33879	ES	6	5	7	0.00	1.8E-02	included
KLC1	29483	ES	13.3:14.1:15	13.2	18	-0.01	1.8E-02	excluded
KLHL42	20900	ES	3	2	4	0.04	1.8E-02	included
FBXO33	27381	ES	2:03	1	4	-0.01	1.8E-02	excluded
ATP6AP2	88831	ES	4:05	3	6	0.00	1.8E-02	included
NUPL2	78963	ES	2	1	3	0.01	1.8E-02	included
PLEKHH2	53393	ES	4	3	5	-0.07	1.8E-02	excluded
ECHDC1	77477	ES	4	1.1	6.1	0.00	1.8E-02	included
PAX8	55050	ES	8:9.1:9.2:10	7	11	0.00	1.8E-02	included
DDX11	20968	ES	25:26:27:28.2:29.1	24	29.3	0.05	1.8E-02	included
TBX3	24654	ES	3	2	4	0.03	1.8E-02	included
FAM86A	33883	ES	5.2	4	6	0.04	1.8E-02	included
MFN1	67692	ES	11	10	12	-0.01	1.8E-02	excluded
PRKAG1	21509	ES	3	2	4.2	0.00	1.9E-02	included
FAM221A	78990	ES	3.1:3.2:3.3	2	4	-0.04	1.9E-02	excluded
DTX2	80177	ES	3	2	4	-0.03	1.9E-02	excluded
AFMID	43823	ES	5:6:7:8:9:10:11.1:12	2	13	0.05	1.9E-02	included
RHOC	4231	ES	6.2:7.1	6.1	7.2	0.00	1.9E-02	included
LETMD1	21743	ES	3.3:4:5:6	3.2	7	-0.06	1.9E-02	excluded
ENTPD6	58867	ES	2:3.1:3.2	1	4	0.02	1.9E-02	included
GYS1	50847	ES	1.2:2:3:4:5:6	1.1	7	0.00	1.9E-02	included
ZC3H14	28717	ES	13:14.1	12	15	0.04	1.9E-02	included
MAX	27952	ES	5.5:5.6	5.1	5.8	0.00	1.9E-02	included
GMIP	48671	ES	6	5	7	0.01	1.9E-02	included
GPR89A	7318	ES	2	1	4	-0.01	1.9E-02	excluded
CHORDC1	18274	ES	3	2	4	-0.01	1.9E-02	excluded
MUC1	8019	ES	2.3:3.1:3.2	2.2	3.3	0.01	1.9E-02	included
SFI1	61876	ES	3:05	2.2	6	-0.04	1.9E-02	excluded
CDKN2A	86009	ES	7	5.5	8	0.00	1.9E-02	included
PLOD2	67138	ES	3	2	4	-0.01	1.9E-02	excluded
SLC37A3	81991	ES	4	3	5	-0.05	1.9E-02	excluded
GABARAPL1	20403	ES	2.7:2.8:2.10:2.12:2.14	2.6	3	0.00	1.9E-02	excluded
NSRP1	40086	ES	5	3	8	-0.07	1.9E-02	excluded
PABPC4	1894	ES	10.2:11	10.1	12	0.00	1.9E-02	included
TMEM62	30215	ES	3	2	4	0.02	1.9E-02	included
CNOT2	23374	ES	7:8:9:10	6	11.2	0.00	1.9E-02	included
MTMR14	63112	ES	19	18	20	0.01	1.9E-02	included
SLC27A5	100951	ES	9	8.2	10.1	0.01	1.9E-02	included
EXOC7	43564	ES	14	13	15	0.00	1.9E-02	excluded
TOX4	26587	ES	4	3.1	5.1	-0.01	1.9E-02	excluded
CGRRF1	27593	ES	3:04	2	5	0.00	1.9E-02	included
PPP3CA	70094	ES	9	8	10	0.00	1.9E-02	excluded
CCM2	79585	ES	3	1	4	0.02	1.9E-02	included
RBFA	46291	ES	5	4	6	0.01	1.9E-02	included
SLC25A39	41839	ES	4	3	5.2	0.00	1.9E-02	included
TMEM14B	75318	ES	3	2	5.1	-0.02	1.9E-02	excluded
PLEKHB2	55373	ES	9.2	8.1	10	-0.03	1.9E-02	excluded
TPM3	7794	ES	6.1:6.2:7:8.1	5.3	8.2	0.00	1.9E-02	included
DNASE1L3	65425	ES	5	4	6	0.02	1.9E-02	included
GTPBP10	80392	ES	5	4	6	0.01	1.9E-02	included
CBLB	66001	ES	18.4	18.2	19	0.03	1.9E-02	included
COPE	48515	ES	5	4	6	0.00	1.9E-02	excluded
FOPNL	34203	ES	3.1:3.2	2	4	-0.02	1.9E-02	excluded
IFT122	66740	ES	4	3	5	-0.02	1.9E-02	excluded
DCTD	71248	ES	1.2:2.1:2.2:3	1.1	5	-0.03	1.9E-02	excluded
PRMT5	26671	ES	2	1.1	3	0.00	1.9E-02	included
SLC35A1	76961	ES	5:06	4	7	0.00	1.9E-02	included

ARRDC3	72781	ES	3	2	4	0.01	1.9E-02	included
GABARAP	38870	ES	1.5	1.1	2.1	0.00	1.9E-02	included
ATF7IP2	33977	ES	11	10	12	0.02	1.9E-02	included
CD33	51316	ES	2	1	3	-0.06	1.9E-02	excluded
IFI27	29086	ES	3.1:3.2	1	4.1	-0.03	1.9E-02	excluded
PYROXD1	20699	ES	4	3	5	-0.04	2.0E-02	excluded
UBE2Q2	31898	ES	4	3	5	0.01	2.0E-02	included
SNX4	66556	ES	2	1	3	0.00	2.0E-02	included
BRD8	73508	ES	12	11	13	-0.02	2.0E-02	excluded
CLK3	31727	ES	06:07.1	5	7.2	0.00	2.0E-02	included
NDST1	74099	ES	13	12	14	0.01	2.0E-02	included
VEGFA	76333	ES	7.1:8.1	6	9.1	0.00	2.0E-02	excluded
GLG1	37566	ES	14	13	15	-0.01	2.0E-02	excluded
LMO3	20618	ES	11.2	9.2	12	0.00	2.0E-02	included
HSD17B7	8757	ES	7	6	8	0.01	2.0E-02	included
MAP1LC3B	37943	ES	3	2	4.1	0.00	2.0E-02	excluded
SCARB2	69597	ES	3	2	4	-0.01	2.0E-02	excluded
INTS8	84574	ES	21	20.3	22	-0.01	2.0E-02	excluded
C12orf29	23603	ES	3	1	4	-0.03	2.0E-02	excluded
MRPL55	10115	ES	2.5:2.6:2.8	1.2	2.9	0.00	2.0E-02	included
FLOT2	40008	ES	5:06	2	7	0.00	2.0E-02	included
ANAPC11	44215	ES	3.3:5:6	3.2	7.2	0.01	2.0E-02	included
NUBPL	27126	ES	3	2	4	0.04	2.0E-02	included
NPRL3	32825	ES	1.4:2:3:4	1.3	6	-0.01	2.0E-02	excluded
FXR1	67746	ES	19	18	20	0.02	2.0E-02	included
HYKK	32100	ES	4	3	5.1	0.05	2.0E-02	included
IKBKB	83593	ES	15	14	16	0.00	2.0E-02	excluded
ENDOV	44068	ES	2.3:2.4:5	2.2	6.2	-0.07	2.0E-02	excluded
MRPL55	10158	ES	2.2:2.3:2.4:2.5	1.1	2.9	0.01	2.0E-02	included
N6AMT1	60296	ES	4	3	5	-0.03	2.0E-02	excluded
NR1H2	51182	ES	10.1:10.2	9	11	0.00	2.0E-02	included
BTD	63620	ES	3:04	1.2	5	0.00	2.0E-02	included
NBPF12	7361	ES	6	5	7	0.03	2.0E-02	included
ARMCX4	89651	ES	17	15	18.1	0.08	2.0E-02	included
CD44	15276	ES	3.1:3.2:4:5:7:8:9.2:10:<br>11:12.1:13:14:15:16.1	2.1	17.2	-0.07	2.0E-02	excluded
EIF3M	14851	ES	3:04	2.2	5	0.00	2.0E-02	included
ATG13	15582	ES	13:14	12	15	0.00	2.0E-02	excluded
RNF8	76015	ES	2	1	3	-0.01	2.0E-02	excluded
REV1	54714	ES	4	3	5	0.03	2.0E-02	included
RABL5	97662	ES	4	1	5	-0.03	2.0E-02	excluded
TSG101	14663	ES	2:03:04	1	5	0.00	2.0E-02	included
ZNF567	49420	ES	3	2.2	5.2	0.04	2.0E-02	included
TBRG4	79586	ES	4:05	3	6	0.01	2.0E-02	included
MRPL52	26638	ES	03:04.1	2	5	0.04	2.0E-02	included
HNRNPA1	22147	ES	6.2:7.1:7.2:8:9.1	6.1	9.2	-0.02	2.0E-02	excluded
APH1A	7445	ES	3	2.2	4	0.00	2.1E-02	included
HMGN1	60617	ES	6.2	5	8.2	0.02	2.1E-02	included
NBPF15	7397	ES	4:05	3	6	-0.05	2.1E-02	excluded
TPGS2	45275	ES	3	1	4.2	0.00	2.1E-02	excluded
TPO	52540	ES	9	8	10	0.00	2.1E-02	included
MS4A6A	16061	ES	5.1:7	4	8.1	0.00	2.1E-02	excluded
LRRC23	20001	ES	9	8	10	0.02	2.1E-02	included
SLC20A2	83733	ES	6	5	7	-0.02	2.1E-02	excluded
GALK2	30526	ES	9	7.2	10	0.00	2.1E-02	excluded
IL1R1	54783	ES	7	6	8	-0.01	2.1E-02	excluded
ASH1L	8088	ES	4	3	5	-0.06	2.1E-02	excluded
USP7	33965	ES	5	4	6.1	-0.02	2.1E-02	excluded
ATXN2	24524	ES	4	3	5	0.01	2.1E-02	included
SPATS2	21579	ES	14	13	15	-0.01	2.1E-02	excluded
UBE2V2	83798	ES	2	1.1	3.2	0.00	2.1E-02	included
DDOST	932	ES	2	1	3	0.00	2.1E-02	included

ZMYM5	25407	ES	5	4	6.1	0.02	2.1E-02	included
ESRP1	84568	ES	14	13	16	0.03	2.1E-02	included
CBR4	71142	ES	3	1	4	0.01	2.1E-02	included
DUSP22	75132	ES	5	3	6	0.01	2.1E-02	included
RARG	21981	ES	5	4	6	0.01	2.1E-02	included
AP4M1	80890	ES	9:10	8	11	0.00	2.1E-02	included
FXYD6	18944	ES	5	2	6	0.01	2.1E-02	included
USE1	48236	ES	6	5.6	7	0.00	2.1E-02	included
PUM1	1452	ES	5:06	4	7	-0.01	2.1E-02	excluded
TBCA	72606	ES	4	2	6.1	0.00	2.1E-02	included
COPS7A	19949	ES	3:04	2.4	6	-0.01	2.1E-02	excluded
SUOX	22340	ES	3	2	5.2	0.03	2.1E-02	included
DCTD	71239	ES	2.1:2.2:3:4	1.2	5	0.01	2.1E-02	included
MMP19	22276	ES	5	4.2	6.1	-0.01	2.1E-02	excluded
KDELC2	18618	ES	7	6	8	-0.03	2.1E-02	excluded
IMMP1L	14814	ES	5:06	4	7	0.02	2.1E-02	included
NINL	58875	ES	17	16	18	0.02	2.1E-02	included
HEATR5B	53213	ES	32	31	33	0.01	2.1E-02	included
INO80C	45190	ES	03:04.2	1	5.1	0.01	2.1E-02	included
DCTD	71240	ES	3:04	1.2	5	0.00	2.1E-02	included
IRF3	51011	ES	1.5:2	1.1	3	-0.03	2.1E-02	excluded
NTMT1	87868	ES	4.1:4.2	2	5.1	0.00	2.1E-02	included
RAD51C	42716	ES	5	4	6	0.02	2.1E-02	included
DEF8	38189	ES	4	2.1	5	0.00	2.1E-02	excluded
DCP2	73000	ES	9	8	10	-0.01	2.1E-02	excluded
TGFBR3	3746	ES	5	4.2	6	0.03	2.1E-02	included
SART3	24219	ES	7	6	8.1	-0.01	2.1E-02	excluded
PCMT1	78118	ES	2	1	5	0.00	2.1E-02	included
MRPS11	32376	ES	3	2	4.1	0.00	2.2E-02	excluded
SPIDR	83782	ES	08:09.1	5	10	-0.01	2.2E-02	excluded
ATP5A1	45374	ES	5	4.2	6.1	-0.01	2.2E-02	excluded
PSMG4	75183	ES	5.4	5.1	5.6	0.03	2.2E-02	included
LGALS8	10382	ES	11	10.1	12	0.04	2.2E-02	included
EPC1	11158	ES	13	12	14	-0.03	2.2E-02	excluded
ILF3	47579	ES	4	3	5	0.00	2.2E-02	included
DHRS4	26789	ES	3:05:06	2	7.1	0.07	2.2E-02	included
BRAP	24530	ES	5	4	6	0.00	2.2E-02	included
SPATS2	21580	ES	8	7	9	-0.04	2.2E-02	excluded
VWA9	31210	ES	1.2:1.3:2.1:2.2:2.3	1.1	3.1	0.06	2.2E-02	included
LRRC37A3	43082	ES	4.2	3.2	5	-0.03	2.2E-02	excluded
SLC25A29	29262	ES	2:3.2:3.3:3.4:3.5:3.6	1	3.7	0.03	2.2E-02	included
ALG8	18012	ES	6	5	7	0.00	2.2E-02	included
CTU2	38020	ES	3	2	4	0.01	2.2E-02	included
CD320	47212	ES	2.1:2.2	1	3.1	-0.04	2.2E-02	excluded
MEAF6	1802	ES	6	5	7	0.01	2.2E-02	included
SCMH1	2055	ES	8	5	9	-0.04	2.2E-02	excluded
SUMF2	79790	ES	5.1:5.2	4	6	0.00	2.2E-02	included
ARPC1B	80613	ES	3	1	4.2	0.01	2.2E-02	included
IL17RE	63250	ES	4	2.2	5	-0.05	2.2E-02	excluded
ELP3	83204	ES	4	3	5	0.00	2.2E-02	included
CUL9	76256	ES	5:06	4	7	0.02	2.2E-02	included
NTMT1	87869	ES	4.1	2	5.1	-0.04	2.2E-02	excluded
RBM6	64950	ES	4:05:06	2	7	-0.03	2.2E-02	excluded
MVP	35961	ES	3:4:5.1:6.1	2.3	6.2	0.00	2.2E-02	excluded
GGT1	61443	ES	05:06.2	4	7.3	0.02	2.2E-02	included
PARN	34080	ES	4:5.1:5.2:6:7:8:9:10	3.2	11	-0.01	2.2E-02	excluded
ERMP1	85803	ES	10	9	11	0.01	2.2E-02	included
LTBP4	385945	ES	29:31:32:33:34	28	35	0.04	2.2E-02	included
SPATA20	42434	ES	4.1:4.2	3	6	-0.02	2.2E-02	excluded
ARAP1	17642	ES	16	15	17	-0.01	2.2E-02	excluded

COL1A1	306631	ES	29:30:31:33:34:35:36:37:38:39:40:41:42:43:44:45:46:47:48:49	28	50	-0.03	2.2E-02	excluded
DCTD	71250	ES	1.2:3	1.1	5	-0.03	2.2E-02	excluded
YWHAE	38297	ES	2:03:06	1	7	-0.03	2.2E-02	excluded
TMBIM4	22900	ES	2.2:4.1:4.2:5	1	6	0.06	2.2E-02	included
REXO4	88043	ES	3:04	2.2	5	0.01	2.2E-02	included
IP6K2	64762	ES	11.4	11.2	11.9	0.01	2.2E-02	included
ZSWIM7	94444	ES	7.2:7.3:7.5:7.6	7.1	7.7	-0.04	2.2E-02	excluded
CNOT8	74248	ES	5	3	6	0.01	2.2E-02	included
MUTYH	2652	ES	6.4:6.5:7:8:9	5	10	0.01	2.2E-02	included
RBM6	64949	ES	3.2:4:5:6	2	7	0.02	2.2E-02	included
PDE9A	60719	ES	6:07	5	8	0.01	2.2E-02	included
TLE2	46642	ES	22	21	23.1	0.00	2.2E-02	excluded
B3GALNT1	67499	ES	8	7	9.1	0.03	2.2E-02	included
TKT	65307	ES	2	1.3	3	0.00	2.2E-02	included
CD3D	18990	ES	2	1	3	0.00	2.2E-02	excluded
URGCP	79367	ES	4.2:4.3	3	4.6	0.03	2.3E-02	included
PC	17139	ES	2	1	3	-0.02	2.3E-02	excluded
SNX1	139180	ES	4.2:5:6:7:8:9:10.1:10.2:11:12:14:15	3	16.1	0.00	2.3E-02	excluded
IYD	78145	ES	6.2	5	7	-0.02	2.3E-02	excluded
SFTA3	27255	ES	5	4.2	6	-0.01	2.3E-02	excluded
GIT2	24374	ES	18.2	17.2	20	-0.03	2.3E-02	excluded
PIGT	59558	ES	2.1:2.2:3:4	1	5.2	0.00	2.3E-02	excluded
ERLEC1	53559	ES	9	8	10	0.01	2.3E-02	included
LMCD1	63055	ES	2	1	3	-0.01	2.3E-02	excluded
CLEC2D	20251	ES	7.1:7.2:7.3:7.4:7.5	6.1	8	-0.05	2.3E-02	excluded
TMBIM6	21614	ES	2.2:2.3	1	3.2	0.00	2.3E-02	excluded
SUZ12	40169	ES	4	3	5	0.01	2.3E-02	included
DYNC2LI1	53401	ES	6	5	7	0.00	2.3E-02	included
MTX2	56119	ES	5	4	6	0.00	2.3E-02	excluded
GBP3	3710	ES	3	2.1	4	0.03	2.3E-02	included
ZNF260	49390	ES	3	2.2	5	-0.06	2.3E-02	excluded
LMBR1	82470	ES	20	19	21	0.01	2.3E-02	included
IMMP1L	14819	ES	4	1	7	0.03	2.3E-02	included
CTSB	97870	ES	3.1	2	5.3	0.01	2.3E-02	included
NOL10	52662	ES	11	10	12	0.01	2.3E-02	included
TG	319464	ES	25:26:27:29:31:32:33:34:35:37:38:39:40:41:	24	46	-0.05	2.3E-02	excluded
BAX	50838	ES	3	2	4	0.00	2.3E-02	excluded
SPIN1	86785	ES	2:03:04	1	5	-0.07	2.3E-02	excluded
SOGA1	59325	ES	15.2:16.1:16.2:16.3	15.1	16.4	0.00	2.3E-02	excluded
ATP2C1	66763	ES	29.1:29.2	28	30	0.01	2.3E-02	included
TRIM68	121243	ES	2	1	3.1	-0.03	2.3E-02	excluded
EIF3H	84958	ES	4:06	3.2	7	0.03	2.3E-02	included
SEC63	77141	ES	2	1	3	-0.02	2.3E-02	excluded
MTMR2	18405	ES	2	1	3	-0.04	2.3E-02	excluded
NAT9	43294	ES	6.3:6.4:6.5:6.6:6.7:7.1	6.2	7.2	0.04	2.3E-02	included
COMMD4	31846	ES	8	7	9.1	0.01	2.3E-02	included
SUMF2	79809	ES	4	3	5.1	0.00	2.3E-02	excluded
C16orf45	34161	ES	2	1	4	-0.02	2.3E-02	excluded
ANO1	17387	ES	17	16	18	0.06	2.3E-02	included
ARMC10	81161	ES	7	6	9	-0.01	2.3E-02	excluded
PARN	34077	ES	10	9	11	0.00	2.3E-02	included
SPHK2	50788	ES	3.3:3.4:3.5	2	4	0.01	2.3E-02	included
SETX	87976	ES	26	25	27	-0.03	2.3E-02	excluded
HMGCS1	71943	ES	2	1	3	0.04	2.3E-02	included
STXBP4	42574	ES	4	3	5	-0.02	2.3E-02	excluded
LAT2	80066	ES	10	9	11	0.00	2.3E-02	excluded
STRA13	44265	ES	3.1:3.2	2	4.1	0.03	2.3E-02	included
ECE1	965	ES	19	18	20	0.00	2.3E-02	included

LARP4B	10606	ES	4	3	5	-0.02	2.3E-02	excluded
PODXL	81824	ES	4.2:4.4:4.6:4.7	4.1	4.8	0.00	2.3E-02	included
UPRT	89523	ES	5	4	6	-0.03	2.3E-02	excluded
PCBP2	22051	ES	15	14.1	16.2	0.01	2.3E-02	included
LTA4H	23822	ES	18.1:18.2	17	19	-0.01	2.3E-02	excluded
ATP6V0D1	37071	ES	6	4	7	0.00	2.4E-02	excluded
KIAA0930	62653	ES	3	1	5.3	0.00	2.4E-02	included
ATP6V0E1	74573	ES	3.1	2	4	0.00	2.4E-02	excluded
PARP8	71984	ES	5:06	4	7	0.02	2.4E-02	included
DDX11	20969	ES	24	23.2	25	0.02	2.4E-02	included
PXK	65443	ES	17.1:17.2	16	20	-0.02	2.4E-02	excluded
SKP2	71800	ES	3:4:5:6:7	2	8	0.00	2.4E-02	excluded
PTBP1	46319	ES	3:4:5:6:7:8:9.1:9.2:10	2	11	0.00	2.4E-02	included
TDP1	28808	ES	21	19	22	-0.01	2.4E-02	excluded
SETMAR	63000	ES	2.3:2.4	1	3	-0.05	2.4E-02	excluded
ZNF185	90408	ES	12	10	13	0.05	2.4E-02	included
XAF1	38796	ES	4.1:4.2:4.3:6:7	2.1	8	-0.02	2.4E-02	excluded
KLF6	10632	ES	3	2.1	4	0.01	2.4E-02	included
LTBP4	49935	ES	26:27:00	24	28	-0.02	2.4E-02	excluded
SLC25A19	43433	ES	3	1	4.2	0.05	2.4E-02	included
C1D	53820	ES	1.2:2.1	1.1	2.2	0.02	2.4E-02	included
RNFT1	42841	ES	4	3	5	-0.02	2.4E-02	excluded
PYROXD1	20698	ES	7	6	8	-0.01	2.4E-02	excluded
PPP4R1	44610	ES	6	4.2	7	0.01	2.4E-02	included
SERPINA1	29133	ES	1.2:2.1:2.4:2.5	1.1	3.2	0.01	2.4E-02	included
RARG	21982	ES	3	2.2	5	-0.01	2.4E-02	excluded
PSAT1	86659	ES	8	7	9	-0.01	2.4E-02	excluded
APOC1	50369	ES	5.1	3.2	7	0.00	2.4E-02	included
ACCS	15475	ES	4	3	5	-0.01	2.4E-02	excluded
TTC32	52745	ES	2	1	3	0.00	2.4E-02	included
HMGN1	60615	ES	6.2:7	5	8.2	0.02	2.4E-02	included
PPIP5K2	72917	ES	26	25	27	-0.04	2.4E-02	excluded
NEK11	66788	ES	4	3	5	-0.02	2.4E-02	excluded
TMUB2	41813	ES	2.2:2.3:2.4:2.5:4.2	2.1	4.3	0.05	2.4E-02	included
HAT1	55964	ES	3	2	4	0.01	2.4E-02	included
IQUB	81626	ES	9	8	10	0.03	2.4E-02	included
ECHDC2	3030	ES	4:5.1:6.1	2.1	6.2	0.02	2.4E-02	included
CSNK1A1	74047	ES	7	6	8.2	-0.01	2.4E-02	excluded
ADARB1	60866	ES	5	4	6	-0.01	2.4E-02	excluded
FLOT2	40012	ES	3:05	2	6	-0.06	2.4E-02	excluded
SUGP2	48552	ES	10	9.3	11	0.01	2.4E-02	included
DTNBP1	75418	ES	4.2	3	5	0.01	2.4E-02	included
PPP4C	36057	ES	3.1:3.2	2.2	4	0.03	2.4E-02	included
MEN1	16701	ES	9	8	10	0.00	2.4E-02	excluded
SMUG1	22135	ES	1.2:1.3:3	1.1	4.1	-0.02	2.4E-02	excluded
ARL13B	65699	ES	2:3.1:3.2:4	1	5.1	-0.06	2.4E-02	excluded
IFI27	29076	ES	4.1	3.2	6.1	0.00	2.4E-02	excluded
G6PC3	41767	ES	1.2:2.1:2.2	1.1	3	0.00	2.4E-02	included
ZNF254	48842	ES	4	3	6	0.05	2.5E-02	included
CERS5	21682	ES	6.2	1	8	-0.01	2.5E-02	excluded
OCIAD1	69244	ES	2.2:2.3:2.4:3:4	2.1	6	0.06	2.5E-02	included
SRP9	9995	ES	4	2	5	0.01	2.5E-02	included
FZD6	84800	ES	7	6.3	8	0.00	2.5E-02	included
GOLGA4	63983	ES	19	18	20	0.01	2.5E-02	included
VAMP8	54291	ES	4	3	5	0.00	2.5E-02	excluded
NDUFAF5	58708	ES	5	4.2	6	0.02	2.5E-02	included
TECR	48003	ES	2	1	4	0.00	2.5E-02	included
CTNND1	15958	ES	4.1:4.2:4.3:5:6:7	2.1	8	-0.03	2.5E-02	excluded
DLST	28439	ES	4	3	5.1	0.00	2.5E-02	included
DCAF8	8450	ES	7.2:8.2	7.1	9	-0.01	2.5E-02	excluded
PPP2R3C	27210	ES	3	1.3	4.2	-0.02	2.5E-02	excluded
LIMCH1	69126	ES	7	6	8	0.00	2.5E-02	included

RAB3IL1	16309	ES	6	5	7	0.02	2.5E-02	included
SFSWAP	25215	ES	12	11	13	0.01	2.5E-02	included
RAD1	71744	ES	6	5	7	-0.02	2.5E-02	excluded
C17orf80	43221	ES	5	4	6	-0.03	2.5E-02	excluded
TUBB6	44682	ES	4.1:4.2	3	5.3	0.00	2.5E-02	excluded
ZNF584	52451	ES	4.1:4.2	3	5	-0.02	2.5E-02	excluded
STXBP5	78057	ES	22:23	21	24	-0.06	2.5E-02	excluded
SEC11A	32314	ES	8	5	9	0.00	2.5E-02	included
MAD1L1	78597	ES	2.1:2.2	1	3	0.01	2.5E-02	included
MRPS35	20898	ES	6	5	7	0.00	2.5E-02	included
GPR107	87895	ES	15:16	14	17	0.00	2.5E-02	excluded
RAB1A	53794	ES	5	4	7	0.03	2.5E-02	included
ZNF880	51450	ES	4.1	3.2	5	-0.06	2.5E-02	excluded
DMKN	49174	ES	8	7	11	-0.04	2.5E-02	excluded
SPAG9	42496	ES	7	6	8	0.03	2.5E-02	included
NUMB	28296	ES	2	1	3	0.01	2.5E-02	included
PGM3	76866	ES	4	2	5.1	0.01	2.5E-02	included
FIS1	81056	ES	3	1	4	0.06	2.5E-02	included
BCS1L	57545	ES	1.4:1.5:1.6:1.7:1.8	1.1	2	-0.04	2.5E-02	excluded
PSEN1	28270	ES	13	12	14	-0.01	2.5E-02	excluded
CCDC24	2531	ES	4.2:5	3.2	6.2	0.02	2.5E-02	included
SCHIP1	67468	ES	4:05:06	3	7	-0.02	2.6E-02	excluded
PLEKHJ1	46588	ES	4	3.3	5.1	0.00	2.6E-02	included
ATL2	53253	ES	5	1	6	0.00	2.6E-02	included
SFTA3	121944	ES	2:4.1:4.2:5	1.3	6	0.01	2.6E-02	included
GNB1	231	ES	2	1	3	0.01	2.6E-02	included
SAMD4B	49806	ES	2:03	1	4	0.01	2.6E-02	included
GABARAPL1	20407	ES	2.10:2.11:2.12	2.6	3	0.00	2.6E-02	included
SYNE2	27846	ES	118	117.2	119.2	0.04	2.6E-02	included
STAU1	59741	ES	2:03	1	5	-0.01	2.6E-02	excluded
TCTEX1D2	68236	ES	4	3	5	-0.02	2.6E-02	excluded
ZFP64	59814	ES	3	2.2	4	-0.01	2.6E-02	excluded
FBXL4	77048	ES	2	1	3	0.03	2.6E-02	included
MEF2B	48596	ES	14.2	13	14.4	0.04	2.6E-02	included
ARHGEF7	26286	ES	20	19	21	0.01	2.6E-02	included
GLTP	24361	ES	3.1:3.2	2	4.1	0.00	2.6E-02	excluded
METTL23	43643	ES	1.2:1.3:1.4:2	1.1	3	0.05	2.6E-02	included
CKLF	36733	ES	2	1	4	0.04	2.6E-02	included
POLM	79456	ES	6.3:7:8	6.2	9.1	0.05	2.6E-02	included
EPN3	42410	ES	6.2	5	7	-0.01	2.6E-02	excluded
BRD9	71466	ES	12	10	13	0.00	2.6E-02	included
ELN	80050	ES	13	12	14	-0.02	2.6E-02	excluded
SEC61A2	10762	ES	15	13	16	0.03	2.6E-02	included
ZNF195	13976	ES	5.2:11:12	5.1	13	-0.01	2.6E-02	excluded
GUK1	10186	ES	10:11.2	9.2	12	-0.01	2.6E-02	excluded
NDUFB5	67703	ES	3.2	1	4.1	0.00	2.6E-02	included
C14orf159	28869	ES	04:05.2	1	7	-0.04	2.6E-02	excluded
PAX8	102556	ES	9.1:9.2	8	10	0.01	2.6E-02	included
AMBRA1	15578	ES	9	8.3	10	-0.03	2.6E-02	excluded
PLEKHM2	767	ES	7	6	8	0.03	2.6E-02	included
FAM107A	65465	ES	5	4	6	0.05	2.6E-02	included
RAD1	71743	ES	6	5	8	-0.04	2.6E-02	excluded
KIF9	64497	ES	19	18	20	-0.03	2.6E-02	excluded
C11orf73	18186	ES	3	2	4	-0.01	2.6E-02	excluded
CBR4	71144	ES	2.2	1	3	-0.01	2.6E-02	excluded
ANKRD53	53910	ES	4	3	5	-0.03	2.6E-02	excluded
CASP8	56827	ES	9	8	10	0.04	2.6E-02	included
IST1	37518	ES	12:13	11	14.1	-0.05	2.6E-02	excluded
RPAIN	38696	ES	4	3	7	0.03	2.6E-02	included
RPAP1	30096	ES	22.2:23.1	22.1	23.2	-0.03	2.6E-02	excluded
TBRG1	19226	ES	3:05	2	6	0.05	2.6E-02	included
LETMD1	21748	ES	3.1:3.2	2	4	-0.03	2.6E-02	excluded

LETMD1	21758	ES	3.1:3.2:4:5:6	2	7	-0.01	2.6E-02	excluded
RUVBL2	50861	ES	3	2	4	-0.03	2.6E-02	excluded
SIMC1	74640	ES	2:03	1	4	-0.05	2.6E-02	excluded
TMEM107	39125	ES	2:3.2:3.3:3.4:3.5:3.6	1	3.7	-0.01	2.6E-02	excluded
MPHOSPH6	37774	ES	2	1.1	3.1	0.00	2.6E-02	excluded
LRCH3	68324	ES	21	20	22.1	-0.01	2.6E-02	excluded
UBXN11	1259	ES	4:5:7:8:9	2	10	0.02	2.7E-02	included
TULP4	78272	ES	13	12	14	0.02	2.7E-02	included
AP1G1	37485	ES	12	10.1	13.2	-0.03	2.7E-02	excluded
DDB2	15675	ES	4:05:07	3	8	-0.05	2.7E-02	excluded
APOBEC3D	62274	ES	4:05:06	3	7	-0.05	2.7E-02	excluded
MPND	46795	ES	11.1:11.2	10	12	0.06	2.7E-02	included
ATP5J	60272	ES	1.2:1.3:1.4:1.5:2	1.1	3	0.03	2.7E-02	included
ING4	19919	ES	2	1	4	0.01	2.7E-02	included
NAP1L1	23487	ES	3	2	4	0.00	2.7E-02	included
FKBP8	48450	ES	05:06.2	3	7	0.00	2.7E-02	excluded
TM6SF1	32251	ES	6.2:7:8	5.3	9	0.01	2.7E-02	included
LDB2	68851	ES	3	2	5	-0.01	2.7E-02	excluded
RRN3	34141	ES	5	4	6	-0.01	2.7E-02	excluded
THNSL2	54470	ES	10	8	11	-0.03	2.7E-02	excluded
GGT5	61395	ES	3	2	4	-0.01	2.7E-02	excluded
RNF4	68575	ES	7	6	8.1	-0.01	2.7E-02	excluded
ELOVL5	76495	ES	6	5.3	7	0.00	2.7E-02	excluded
SLC25A23	47044	ES	5	4	6	-0.02	2.7E-02	excluded
MRPS28	84266	ES	4.1:4.2	1	5	0.01	2.7E-02	included
FAM104A	43214	ES	3.1:3.2	1	5	0.01	2.7E-02	included
MX1	60669	ES	5	4	6	0.01	2.7E-02	included
DMKN	49195	ES	7:08	6.4	11	-0.05	2.7E-02	excluded
MTFP1	61750	ES	3.2:3.3	2	4	-0.01	2.7E-02	excluded
DNAJC19	67760	ES	5.1:5.2	4	6	0.01	2.7E-02	included
SLC4A5	54039	ES	24	23	25	0.01	2.7E-02	included
DCUN1D4	69268	ES	13	11	14	0.02	2.7E-02	included
TCEB1	84208	ES	5	1.2	6	0.02	2.7E-02	included
TFDP2	67093	ES	7:08	6	9	-0.04	2.7E-02	excluded
PLCB2	29965	ES	29	28	30	0.01	2.7E-02	included
SENP2	68027	ES	4.2	3	5.1	0.00	2.7E-02	included
CHKA	17289	ES	3	2	4	-0.01	2.7E-02	excluded
RPL29	65169	ES	3.3	3.1	4	0.00	2.7E-02	excluded
SH3GLB1	3686	ES	2	1	3	0.01	2.7E-02	included
ZNF135	52397	ES	2.2:2.3	1	3	0.04	2.7E-02	included
ABHD12	58873	ES	3	2	4	0.00	2.7E-02	included
TMEM107	39127	ES	2:3.2:3.4:3.5:3.6	1	3.7	0.03	2.7E-02	included
SEC22C	64298	ES	4:6.1:6.2	3	7	-0.01	2.7E-02	excluded
CCNJL	74419	ES	6	5	8	-0.05	2.7E-02	excluded
RRM1	14038	ES	3	2	4	0.00	2.7E-02	excluded
GUCD1	61413	ES	06:07.1	5	7.2	-0.02	2.7E-02	excluded
MVK	24336	ES	9.1:9.2	8.1	10	0.00	2.7E-02	excluded
HMGN1	60616	ES	6.1:6.2	5	8.2	0.03	2.7E-02	included
YLPM1	28429	ES	5	4.2	6	0.01	2.7E-02	included
DDHD1	27563	ES	13	12	14	0.04	2.7E-02	included
ZNF331	51731	ES	9	6	11	-0.03	2.8E-02	excluded
CTNND1	15999	ES	3:4.1:4.2:4.3	2.1	5	-0.04	2.8E-02	excluded
MGEA5	12920	ES	13	12.1	14.1	-0.01	2.8E-02	excluded
MTA3	53371	ES	7	6	8	-0.05	2.8E-02	excluded
ODF2	87762	ES	8.1:8.2	6	9.1	0.00	2.8E-02	included
CEP192	44732	ES	14	13	15	0.03	2.8E-02	included
NUMB	28295	ES	4	3	5.2	0.02	2.8E-02	included
PRPF39	27399	ES	4.1:4.3	3	5	-0.06	2.8E-02	excluded
THNSL2	54473	ES	7:08:09	6	11	0.01	2.8E-02	included
SLC38A7	36680	ES	8:09:10	7.2	11	0.01	2.8E-02	included
NDRG1	85254	ES	2.2:14:15:16.1	2.1	16.2	-0.04	2.8E-02	excluded
PCBP2	22052	ES	15	14.1	16.1	0.02	2.8E-02	included

POLDIP3	62533	ES	3.2:4:6:7.1:7.2:8:10.1	2	10.2	-0.06	2.8E-02	excluded
ANKRD11	38081	ES	9	7	10	-0.03	2.8E-02	excluded
FYN	102082	ES	2	1	4	0.01	2.8E-02	included
TOR1AIP1	9126	ES	9	8	10	0.00	2.8E-02	included
LILRB4	51931	ES	13.2	12.2	14	0.05	2.8E-02	included
FBXL12	47420	ES	4.2	2.4	5	0.03	2.8E-02	included
TTC12	18779	ES	7	6	8	-0.02	2.8E-02	excluded
CCM2	79584	ES	6:07	5	8	0.00	2.8E-02	excluded
UXS1	54855	ES	3.1:3.2	2	4	0.01	2.8E-02	included
LGALS3BP	234088	ES	2.4:3.1:3.2	2.2	4.1	0.01	2.8E-02	included
BFAR	34098	ES	4	2	5	-0.03	2.8E-02	excluded
KIAA0430	34179	ES	8.2:8.3	7	9	-0.06	2.8E-02	excluded
CD44	15200	ES	3.2:4:5:7:8:9.2:10:11:<br>12.1:13:14:15:16.1	3.1	16.2	-0.01	2.8E-02	excluded
MCFD2	53477	ES	6	3	7	0.00	2.8E-02	included
AP3S2	32456	ES	4	3	6	0.01	2.8E-02	included
HNRNPA1	120410	ES	6.3:7.2	6.2	8	0.00	2.8E-02	excluded
FMNL3	21603	ES	26	25	27	-0.03	2.8E-02	excluded
PKIG	59478	ES	5	4	6.2	0.00	2.8E-02	excluded
MRPS27	72452	ES	5	4.1	6	0.00	2.8E-02	included
CMC2	37729	ES	8	5	9	0.00	2.8E-02	included
ZNF691	2136	ES	2.1:2.3	1	4	0.04	2.8E-02	included
CES4A	36918	ES	10	9	11	-0.01	2.8E-02	excluded
EXOSC3	86446	ES	3	2	4.1	-0.02	2.8E-02	excluded
NUP54	69587	ES	7	6	8	0.01	2.8E-02	included
CACNB1	40629	ES	8	6	9	-0.04	2.8E-02	excluded
MFSD8	70556	ES	2	1	3	0.03	2.9E-02	included
PROM2	54498	ES	15	14	16	0.00	2.9E-02	included
NSFL1C	58498	ES	8	7.3	9	0.00	2.9E-02	excluded
COPS7A	19948	ES	5	2.4	6	0.00	2.9E-02	excluded
SFTA3	27266	ES	3:4.1:4.2:4.3:5	2	6	-0.01	2.9E-02	excluded
FBLN5	28894	ES	6	5	8	-0.01	2.9E-02	excluded
KLHL18	64510	ES	3	1	4	0.02	2.9E-02	included
CORO1B	387277	ES	4:5.1:5.3:5.4:5.5:6	3	7	0.06	2.9E-02	included
TM4SF1	67225	ES	2.3	2.1	3.1	0.00	2.9E-02	included
NAGK	53923	ES	2	1	3	0.00	2.9E-02	included
UQCC1	59124	ES	5	3	6.1	0.00	2.9E-02	included
C14orf159	28852	ES	11.1:11.2:11.3:11.4	10.2	12.1	0.00	2.9E-02	included
ELMOD3	54248	ES	2.1	1	2.3	-0.04	2.9E-02	excluded
CDIP1	33759	ES	5.2:6.1	5.1	6.2	0.00	2.9E-02	included
YWHAE	38294	ES	4:05	3	6	0.00	2.9E-02	included
B3GALNT1	67501	ES	7:08	5	9.1	-0.06	2.9E-02	excluded
CALU	81707	ES	4	3	5	-0.02	2.9E-02	excluded
UPRT	89524	ES	3	1	4	-0.01	2.9E-02	excluded
NOLC1	12943	ES	2:3.1:3.2:4.1:4.2:5	1	6.1	0.00	2.9E-02	included
IFT122	66733	ES	5	3	7	0.01	2.9E-02	included
CDC14B	86979	ES	14	13	16	0.04	2.9E-02	included
RALB	55153	ES	4	3.3	5	0.00	2.9E-02	excluded
SLC25A10	44162	ES	7	6	8.1	0.03	2.9E-02	included
COX4I1	156376	ES	4.2:5.1	4.1	5.4	-0.03	2.9E-02	excluded
CTNNBL1	59362	ES	2	1	3	0.00	2.9E-02	excluded
ADARB1	60865	ES	9	8	10	-0.03	2.9E-02	excluded
MPZL1	8875	ES	3:04	2	6	-0.04	2.9E-02	excluded
OGG1	63162	ES	7.1:7.2	6.1	8	-0.03	2.9E-02	excluded
SMC6	52732	ES	9	8	10	-0.03	2.9E-02	excluded
SLC25A17	62363	ES	10	9	11	0.00	2.9E-02	excluded
DTX2	80174	ES	7	6	8	0.03	2.9E-02	included
HNRNPLL	53261	ES	5.2:6.1	4.2	6.2	0.00	2.9E-02	excluded
ORAOV1	17374	ES	3	1	4.1	-0.01	2.9E-02	excluded
SMPD1	14102	ES	3	2.3	4	0.00	2.9E-02	included
SVIL	11113	ES	21	20	22	-0.01	2.9E-02	excluded
MRPL9	7650	ES	5	4	6	0.00	2.9E-02	included

MBOAT7	51804	ES	4	3	5	0.00	2.9E-02	excluded
BAI2	1499	ES	33	32.2	34	0.01	2.9E-02	included
MRPL55	10120	ES	2.2:2.5:2.6	1.2	2.9	0.02	2.9E-02	included
ASCC1	12079	ES	15	14	16	-0.04	2.9E-02	excluded
EIF2D	9588	ES	6:07	5	8	0.00	3.0E-02	included
BCAS3	42872	ES	28:29:00	27	30	-0.03	3.0E-02	excluded
NQO1	37299	ES	5	4	6.1	0.00	3.0E-02	excluded
METTL2B	81694	ES	3	2	4	0.00	3.0E-02	excluded
YAF2	21151	ES	5.1:5.2:7	2	9.1	0.00	3.0E-02	excluded
CTNND1	15957	ES	3:4.1:4.2:4.3:5:6:7	2.1	8	-0.02	3.0E-02	excluded
AK2	1655	ES	3	1	4	0.00	3.0E-02	excluded
MCTP1	72803	ES	21	19	22	0.04	3.0E-02	included
PTP4A2	1527	ES	6	3	8	-0.03	3.0E-02	excluded
MRPL55	10130	ES	1.2:2.2:2.4:2.5:2.6:2.7:	1.1	2.9	0.00	3.0E-02	included
MTMR14	63116	ES	16	15	17	0.00	3.0E-02	included
PISD	61882	ES	11	10	12	0.00	3.0E-02	excluded
RIC8B	24160	ES	15:16	14	17	0.03	3.0E-02	included
PRKCD	65294	ES	2	1	3	-0.02	3.0E-02	excluded
VEZT	23786	ES	5	4	6.1	-0.03	3.0E-02	excluded
GNB2L1	190578	ES	4.1:4.2:5:6:7.2:8.1:8.2	3	9	-0.03	3.0E-02	excluded
IMMP1L	14818	ES	3:04	1	7	0.02	3.0E-02	included
PPP3CA	70093	ES	13	12	14	0.00	3.0E-02	included
PPP4R1	44608	ES	15	14	16	-0.02	3.0E-02	excluded
WDR41	72583	ES	7	6	8	0.00	3.0E-02	excluded
CCPG1	30728	ES	8	7.2	9.1	0.00	3.0E-02	included
TCEB1	84206	ES	1.3:6	1.2	7	-0.05	3.0E-02	excluded
TRIP10	47083	ES	11.1:11.2	10	12.2	-0.03	3.0E-02	excluded
ORC5	81263	ES	3.1	2	4	0.00	3.0E-02	excluded
CITED1	89456	ES	2.2	1	5	0.02	3.0E-02	included
PPP2R3C	27198	ES	11	10	12	0.00	3.0E-02	included
PGPEP1	48422	ES	3	2	4	-0.01	3.0E-02	excluded
CMTM3	36814	ES	2.3:5:6:7.2:9.1	2.2	9.2	0.00	3.0E-02	excluded
NDUFA12	23737	ES	3	2.2	5.1	0.00	3.0E-02	included
FDPS	8069	ES	2	1.1	3.1	-0.02	3.0E-02	excluded
NAV2	14699	ES	23	22	24	0.04	3.0E-02	included
DTNA	45112	ES	19	17	22	-0.03	3.0E-02	excluded
CTNND1	16003	ES	2.2:2.3:3:4.3	2.1	5	0.00	3.0E-02	excluded
FLOT2	40007	ES	3:05:06	2	7	0.01	3.0E-02	included
C5orf24	73433	ES	3	2	4.1	-0.01	3.0E-02	excluded
ZNF431	48758	ES	5	4	7	0.02	3.1E-02	included
SHISA5	64691	ES	6	5	8	0.00	3.1E-02	excluded
POLR3H	62433	ES	5.1	3	6	0.00	3.1E-02	included
RPH3AL	38214	ES	6	5	7	0.01	3.1E-02	included
ZNF706	84747	ES	3.2:3.3:3.4	2	4	0.00	3.1E-02	excluded
STAU2	84184	ES	3	2	4	-0.02	3.1E-02	excluded
ZNF346	74706	ES	04:05.1	3	5.2	0.00	3.1E-02	excluded
APTX	86084	ES	6.1:6.2:7.1:7.2:7.3	5.2	7.4	0.04	3.1E-02	included
TCTN3	12650	ES	4:05	3	6	0.00	3.1E-02	excluded
CCM2	79583	ES	8	7	9	0.00	3.1E-02	included
IRF3	51008	ES	1.2:1.3:1.4:1.5:2	1.1	3	-0.07	3.1E-02	excluded
FGFR2	13303	ES	16	15	17.1	-0.01	3.1E-02	excluded
ACTR3	55080	ES	4	3	5	0.00	3.1E-02	included
MTIF2	53609	ES	2	1.1	3	-0.01	3.1E-02	excluded
TYW1	79908	ES	9	8	10	0.01	3.1E-02	included
SDCBP	83936	ES	02:03.2	1	3.3	-0.02	3.1E-02	excluded
TTC23	32615	ES	3	2.3	4	0.02	3.1E-02	included
ANKS1A	75794	ES	7:8:9:10:11:12:13:14:<br>15:16:17:18:19:20:21:	6	24.2	0.01	3.1E-02	included
RNF121	17462	ES	4	3	6.1	-0.05	3.1E-02	excluded
SLC25A29	29259	ES	3.2:3.3:3.4:3.5	2	3.7	0.06	3.1E-02	included
IRF3	50995	ES	5.1:5.2	4	6.2	-0.01	3.1E-02	excluded
ZNF763	47764	ES	6	5.2	7	0.02	3.1E-02	included

ZSCAN32	33552	ES	3	2.2	4	0.03	3.1E-02	included
CCL4	40387	ES	2	1	3	0.01	3.1E-02	included
ARNT	7513	ES	7	6	8	0.03	3.1E-02	included
IQCB1	66404	ES	10	9	11	0.01	3.1E-02	included
ZNF263	33510	ES	3	2	4	0.01	3.1E-02	included
TPRA1	66610	ES	9:10	8	11	0.00	3.1E-02	excluded
REPIN1	82248	ES	3.2	2.1	5.2	-0.03	3.1E-02	excluded
CRCP	79874	ES	3:4:5:6	1.2	7	-0.02	3.1E-02	excluded
MTMR14	63121	ES	5.2:6	5.1	7	0.00	3.1E-02	excluded
MAP2K4	39301	ES	6	5	7	0.00	3.1E-02	included
RBPJ	68982	ES	14	13	15	0.00	3.1E-02	excluded
KIAA1217	119028	ES	22	21	23	0.01	3.1E-02	included
DBI	55117	ES	3.1:3.2	1.1	5	0.00	3.1E-02	excluded
PUM1	1448	ES	6:07:08	5	9	0.00	3.1E-02	included
KTN1	27638	ES	26	25	27	0.00	3.2E-02	excluded
WDR86	82376	ES	5.2	4	6	-0.01	3.2E-02	excluded
MLEC	24778	ES	3:04	2	5	0.00	3.2E-02	excluded
CTNND1	15936	ES	19	18	20	0.00	3.2E-02	excluded
NBPF15	91080	ES	2	1	3	-0.04	3.2E-02	excluded
EVI5	3765	ES	12	11	13	0.05	3.2E-02	included
COPS8	58098	ES	4	3	5	0.00	3.2E-02	excluded
CATSPER2	105993	ES	4	3	5	0.04	3.2E-02	included
ITGB1BP1	52625	ES	3	1	4	0.03	3.2E-02	included
SELP	8932	ES	11	10	12	0.01	3.2E-02	included
SLC25A11	38602	ES	2	1	3	0.01	3.2E-02	included
DYNC2LI1	53403	ES	2	1	3	0.04	3.2E-02	included
RCBTB2	25865	ES	3:04	2	5	0.02	3.2E-02	included
AFMID	43804	ES	7:8:9:10:11.1	6	12	0.04	3.2E-02	included
CD44	14980	ES	8:9.1:9.2:10:11	7	12.1	0.06	3.2E-02	included
SPARCL1	69872	ES	2	1	3	-0.01	3.2E-02	excluded
KAT5	16913	ES	4	3	5	0.02	3.2E-02	included
EEF1D	85460	ES	06:07.2	1	8.1	-0.01	3.2E-02	excluded
SNRPD1	44773	ES	2	1	3.1	0.00	3.2E-02	included
NBN	84397	ES	6	5	7	0.03	3.2E-02	included
ANKRD6	77007	ES	10.1	9	11	0.00	3.2E-02	included
UNG	24279	ES	4	3	5	0.00	3.2E-02	included
CCNB1IP1	26420	ES	7.1:7.2	5	8	-0.01	3.2E-02	excluded
ACAD8	19557	ES	2:03:04	1	5	0.00	3.2E-02	excluded
PREPL	53440	ES	2.2:2.3	1.3	3	-0.03	3.2E-02	excluded
KLHDC4	37958	ES	6.1:6.2	4.2	7.1	0.01	3.2E-02	included
ZNF528	51459	ES	3	2	4	-0.01	3.2E-02	excluded
CARD8	50718	ES	4.1	3	5	0.04	3.2E-02	included
ANXA5	70491	ES	3:04	2	5	0.00	3.2E-02	included
HNRNPH1	74908	ES	2	1.5	3	-0.01	3.2E-02	excluded
ARHGDIA	44195	ES	4:05:06	3.2	7.1	0.00	3.2E-02	included
HYAL3	64985	ES	4	3.2	5	-0.02	3.2E-02	excluded
EMP3	50726	ES	2:03	1	4.1	-0.05	3.3E-02	excluded
TJP1	29766	ES	23	22	24	-0.02	3.3E-02	excluded
RAB11B	47228	ES	2	1	3	0.00	3.3E-02	included
CD22	49115	ES	6	5	7	-0.01	3.3E-02	excluded
NADK	229	ES	9.2:10.1	9.1	10.2	-0.02	3.3E-02	excluded
DDX42	42991	ES	2:03	1.3	4	-0.01	3.3E-02	excluded
RBPJ	68984	ES	4.2	2.3	5	0.03	3.3E-02	included
TMEM220	39286	ES	2	1	3	-0.02	3.3E-02	excluded
MRPL55	10121	ES	2.5:2.6	1.2	2.9	0.01	3.3E-02	included
LMBR1L	21526	ES	4.1	3	5	0.02	3.3E-02	included
CCNG1	74438	ES	2.1:2.2	1.5	3.1	0.00	3.3E-02	included
TSPAN4	13800	ES	5.2	1	7	0.01	3.3E-02	included
EEF1D	85444	ES	8.3:10.1:10.2:11:12.1:12.2:13.1	8.2	13.2	-0.01	3.3E-02	excluded
CTNND1	15937	ES	12	11	13	0.00	3.3E-02	excluded
EMP3	50728	ES	2	1	3	0.00	3.3E-02	excluded

DDX52	40539	ES	3:05	2	6	-0.03	3.3E-02	excluded
TSPAN7	88816	ES	6	1	7	0.00	3.3E-02	excluded
KTN1	27637	ES	35	34.2	36	0.02	3.3E-02	included
DDX3X	88853	ES	9	8	10	0.00	3.3E-02	included
SKA2	42751	ES	1.2:2:3:4.1	1.1	5	0.01	3.3E-02	included
RNF121	17449	ES	5:6.1:6.2	3	7	0.05	3.3E-02	included
KIAA0430	34169	ES	13	12	14	0.00	3.3E-02	included
SIRT6	46773	ES	4	3	5	0.01	3.3E-02	included
ATP1B3	67084	ES	2	1	3	0.00	3.3E-02	excluded
MAP2	57228	ES	9.2:10:11	8	12	-0.03	3.3E-02	excluded
WDR45	89084	ES	06:07.1	5	7.2	-0.04	3.3E-02	excluded
EFEMP2	16933	ES	11.1	10	11.3	0.00	3.3E-02	excluded
IRAK4	21255	ES	5	3	6	0.04	3.3E-02	included
AP4S1	27101	ES	7	5.2	8	0.05	3.3E-02	included
LAT2	80068	ES	2.1:2.2	1	3	-0.05	3.3E-02	excluded
DPP9	46826	ES	4	3	6	-0.05	3.3E-02	excluded
CREB1	57178	ES	8	7.2	9	-0.01	3.3E-02	excluded
PTGES2	87700	ES	3	2	4	-0.01	3.3E-02	excluded
ACTN4	49687	ES	2.1:2.2:3:4:5:6:7:8	1	9	0.00	3.3E-02	included
ZNF136	47785	ES	2	1	3	-0.02	3.4E-02	excluded
PPIL6	77195	ES	7	6	9	-0.04	3.4E-02	excluded
BTRC	12874	ES	2	1	3	-0.04	3.4E-02	excluded
HADHB	52888	ES	9:10	8	11	0.00	3.4E-02	included
DMPK	50522	ES	13:14.1:14.2	12	15	-0.03	3.4E-02	excluded
HKR1	49490	ES	15	13	17.2	0.03	3.4E-02	included
CERS5	21673	ES	6.1:6.2:8	1	9	0.00	3.4E-02	excluded
BTN3A2	75630	ES	2.2:3.1:3.2	1	4.2	-0.01	3.4E-02	excluded
CRYAB	18700	ES	4	3	5.2	-0.01	3.4E-02	excluded
USP10	37865	ES	2	1	4	0.01	3.4E-02	included
GATSL3	61708	ES	3	2	4	0.00	3.4E-02	included
ZKSCAN1	80872	ES	2	1	3.1	-0.05	3.4E-02	excluded
CPSF3L	132	ES	4:5.2:6.2	1	7.1	-0.02	3.4E-02	excluded
SEC22C	64299	ES	6.2	3	7	0.00	3.4E-02	included
MVD	38011	ES	2	1	5	-0.01	3.4E-02	excluded
HERPUD1	36504	ES	5:06	4	7	0.00	3.4E-02	included
MACROD1	117288	ES	10	9	11	0.01	3.4E-02	included
ZNF337	58878	ES	4	3	5	0.02	3.4E-02	included
NPL	9168	ES	02:03.2	1	4	-0.01	3.4E-02	excluded
INTS9	83230	ES	2	1.1	3	0.00	3.4E-02	included
LETMD1	21756	ES	3.1:3.2:3.3:4:5:6	2	7	0.00	3.4E-02	excluded
WDR91	81881	ES	14	13	15	0.00	3.4E-02	included
TACC1	83449	ES	7:08	5.2	10.1	0.03	3.4E-02	included
PRUNE	7543	ES	4:05:06	3	7	-0.01	3.4E-02	excluded
TRMT1	47918	ES	9	8	10	0.03	3.4E-02	included
YWHAE	38296	ES	3:4:5:6	1	7	0.00	3.4E-02	included
CD74	152982	ES	07:01.1	4	10.2	-0.01	3.4E-02	excluded
UBXN11	101232	ES	5:06	2	7	0.04	3.4E-02	included
REPIN1	82241	ES	4.2	3.2	5.2	-0.04	3.4E-02	excluded
TBXAS1	81971	ES	8	7.1	9	-0.06	3.4E-02	excluded
NBPF10	7265	ES	19:20:21:22	16	23	0.00	3.4E-02	excluded
TRAF3	29424	ES	8	7	9	-0.01	3.4E-02	excluded
BZRAP1	42656	ES	31	30	32	-0.03	3.4E-02	excluded
PGAP2	14024	ES	6:07	4	10	0.06	3.4E-02	included
CCBL2	3703	ES	6	5	7	-0.01	3.4E-02	excluded
RNF216	78683	ES	4:5.2:6.2	2	7	0.04	3.4E-02	included
RMDN2	53238	ES	4.2:5.1	3	6	0.00	3.4E-02	excluded
NPTN	31605	ES	3:04	1	5	0.00	3.5E-02	included
MAPKAP1	87583	ES	10	8	11	0.01	3.5E-02	included
MVB12A	48306	ES	4	3	5	0.00	3.5E-02	included
INPP5J	206703	ES	3.1	1	3.3	0.04	3.5E-02	included
BCKDK	36238	ES	12	11	13.1	0.00	3.5E-02	included
LDHA	14620	ES	6.1:6.2:7:8.1	4	8.2	0.00	3.5E-02	excluded

TSEN15	9205	ES	4:5.1:5.2	3	6	-0.02	3.5E-02	excluded
YIPF1	3077	ES	3:04	2	5	-0.01	3.5E-02	excluded
EIF3J	30343	ES	3:04	2	5	0.00	3.5E-02	included
CDK2	22324	ES	1.3	1.1	2.1	0.01	3.5E-02	included
CD164	77192	ES	5	4	6	0.00	3.5E-02	included
DEPTOR	85012	ES	2:03	1	4	0.00	3.5E-02	included
DMKN	49161	ES	9	8	12	0.08	3.5E-02	included
MPI	31780	ES	1.2:2.2	1.1	2.3	0.02	3.5E-02	included
CAPZB	907	ES	10:11	9	12	0.04	3.5E-02	included
PRMT2	60961	ES	8	6.1	12	-0.06	3.5E-02	excluded
RAP1B	22947	ES	1.2:3.1:3.2:4:5	1.1	6	0.00	3.5E-02	included
CCDC176	28365	ES	2	1	3	-0.02	3.5E-02	excluded
PRSS16	75693	ES	3:4:5:6:7:8:9	2	10	0.02	3.5E-02	included
SERINC4	115970	ES	5:6:7:8:9:10	4	11	-0.01	3.5E-02	excluded
IKBKAP	87148	ES	2:3:4:5:6:7:8	1	9	0.02	3.5E-02	included
ALKBH8	18572	ES	11	10.2	12	-0.03	3.5E-02	excluded
EEF1D	98099	ES	6	1	7.2	-0.02	3.5E-02	excluded
C19orf12	48857	ES	3	1	5.1	-0.05	3.5E-02	excluded
AMPD3	14353	ES	7	6	8	0.01	3.5E-02	included
UQCC1	59094	ES	8	7.2	9	0.00	3.5E-02	included
STARD3	40660	ES	5	4.2	6	0.00	3.5E-02	included
ZNF232	38658	ES	3	2	4.1	0.05	3.5E-02	included
ANXA7	12147	ES	2	1	3.1	0.00	3.5E-02	included
CEP41	81798	ES	12	11	13	0.02	3.5E-02	included
ZBTB17	782	ES	4	3	5.2	-0.01	3.5E-02	excluded
PIGQ	32903	ES	12:13	11	14	-0.05	3.5E-02	excluded
NF2	61625	ES	16.1:16.2	15	17	0.02	3.6E-02	included
PPP2R2A	83123	ES	4	3.1	5	-0.01	3.6E-02	excluded
PDE9A	60728	ES	4	3	5	-0.01	3.6E-02	excluded
SRSF3	75985	ES	4.1	3	5	0.03	3.6E-02	included
UBE2F	58164	ES	8	7	9	0.00	3.6E-02	included
MACF1	1887	ES	59	58	60	-0.02	3.6E-02	excluded
CAMK2G	12241	ES	19.1:19.2	18	21	-0.03	3.6E-02	excluded
ACOT7	395	ES	12.1:12.2	11	13	0.00	3.6E-02	excluded
SMU1	86093	ES	2:03	1	4	0.00	3.6E-02	excluded
AFMID	43795	ES	10:11.1	8	12	0.04	3.6E-02	included
MAGED1	89147	ES	4	3.2	5	0.00	3.6E-02	included
MOSPD1	90172	ES	5	4.1	6	0.03	3.6E-02	included
TMUB2	41809	ES	2.3:2.4:2.5:4.2	2.2	4.3	-0.04	3.6E-02	excluded
GGT5	61397	ES	2	1	4	0.01	3.6E-02	included
ZNF763	47766	ES	5.2:6	4	7	0.00	3.6E-02	included
NBPF11	7344	ES	6:07	4	8.3	0.07	3.6E-02	included
DTNA	45100	ES	31	30	32.2	-0.01	3.6E-02	excluded
FAM49B	85160	ES	5	4	6	-0.05	3.6E-02	excluded
DHRS12	25953	ES	3	2	4	-0.03	3.6E-02	excluded
KIDINS220	52604	ES	27	26	29	-0.01	3.6E-02	excluded
D2HGDH	58416	ES	7.1:7.2:7.3:8:9:10:11.	6	11.2	0.08	3.6E-02	included
SLC27A2	30569	ES	5	3	6	-0.01	3.6E-02	excluded
CMAS	20723	ES	6	5	7	0.00	3.6E-02	excluded
DMWD	50528	ES	4	3	5	-0.02	3.6E-02	excluded
NAE1	36876	ES	2.1:2.2	1	3.1	-0.01	3.6E-02	excluded
ZNF738	48767	ES	2	1	3	0.04	3.6E-02	included
PQLC3	52673	ES	6:07	5	8	-0.04	3.6E-02	excluded
VOPP1	79758	ES	3.1:3.2	1	9	0.00	3.6E-02	included
IL11RA	86212	ES	6	5	7	0.00	3.6E-02	included
PNPO	42131	ES	4:05	3	6	0.00	3.6E-02	excluded
STK33	14243	ES	15	14	16	-0.01	3.6E-02	excluded
ASNS	80575	ES	2.3	1	3	0.04	3.6E-02	included
L3MBTL3	77509	ES	8	7	9	0.04	3.6E-02	included
ZSWIM7	94449	ES	7.3:7.5	7.1	7.7	-0.04	3.6E-02	excluded
NME6	64588	ES	5.2	4	6	0.02	3.6E-02	included
CCDC176	28364	ES	7	6	8	0.04	3.6E-02	included

THUMPD1	34383	ES	3	2	4	-0.04	3.6E-02	excluded
UBAP1	86147	ES	6	5	7	0.00	3.6E-02	excluded
HNRNPA1	301522	ES	6.3:7.2:8:9.1:9.2	6.2	10	0.00	3.6E-02	excluded
TSPAN7	88817	ES	5	1	7	0.00	3.6E-02	excluded
FAM120A	86917	ES	16	15	17	0.00	3.7E-02	included
HARS	73736	ES	4	2	5	0.01	3.7E-02	included
HSD17B4	73084	ES	3	2	4	0.00	3.7E-02	excluded
ARL6IP5	65575	ES	4.2:5.1	4.1	5.2	0.00	3.7E-02	excluded
RNF14	73851	ES	5:07	4	8	-0.07	3.7E-02	excluded
METTL1	22748	ES	2	1	3	0.02	3.7E-02	included
CHCHD7	83904	ES	5.5	5.3	6	0.00	3.7E-02	excluded
ECD	12129	ES	10	9	11	0.00	3.7E-02	excluded
RABL5	97659	ES	3.1:3.2:4	1	5	-0.05	3.7E-02	excluded
NBPF20	114047	ES	2:3:4:5:6	1	7	-0.03	3.7E-02	excluded
EPS8L2	13732	ES	2.2	1	3	-0.02	3.7E-02	excluded
GANC	30145	ES	4	3	5	-0.01	3.7E-02	excluded
XRRA1	17792	ES	11:12	10	13	-0.05	3.7E-02	excluded
TTLL3	63210	ES	10:11	9	12.1	0.03	3.7E-02	included
TTLL3	63209	ES	11	10	12.1	-0.04	3.7E-02	excluded
MAP4K4	54754	ES	24	23	25	0.02	3.7E-02	included
PFKL	60815	ES	2:03:04	1	5	-0.01	3.7E-02	excluded
MFSD4	9558	ES	8	7	9	0.01	3.7E-02	included
GUK1	10183	ES	11.1:11.2:11.3	9.2	12	-0.02	3.7E-02	excluded
AFMID	43792	ES	11.1:11.2	10	12	0.05	3.7E-02	included
PIFO	4162	ES	3	2	4	0.02	3.7E-02	included
MDM2	23100	ES	5.1:5.2	3	6	0.01	3.7E-02	included
TM7SF2	16761	ES	8	7	9	0.00	3.7E-02	included
TDP1	28813	ES	9	8	10.1	0.01	3.7E-02	included
AIG1	77976	ES	3	1	4	0.00	3.7E-02	excluded
EIF3F	14214	ES	4	3.2	5	0.00	3.7E-02	included
RPL15	63733	ES	2	1.1	3.2	0.00	3.7E-02	excluded
TCEAL4	89746	ES	4.1:4.2:5.1	3.1	5.2	0.01	3.7E-02	included
FAXC	99934	ES	4	3	5	-0.03	3.7E-02	excluded
FAM161A	53702	ES	5	4.2	6	0.04	3.7E-02	included
PGAP3	40672	ES	4	3	5	0.00	3.7E-02	excluded
CYB561D1	4032	ES	1.2:2:3	1.1	4.2	0.01	3.7E-02	included
GTPBP10	80396	ES	4:05	3	6	0.02	3.7E-02	included
NAPA	50660	ES	4	1	5	-0.02	3.7E-02	excluded
ARL17B	42020	ES	4.1	3	6	-0.01	3.7E-02	excluded
TRIQK	84492	ES	5	4	6.2	0.03	3.7E-02	included
MTA1	29647	ES	4	3	5	0.01	3.8E-02	included
SRPX	88797	ES	10	9	11	-0.01	3.8E-02	excluded
LLGL2	43463	ES	26	25	27	0.01	3.8E-02	included
CTNNBIP1	584	ES	3	1	5	0.02	3.8E-02	included
PSMB5	26691	ES	1.3:2	1.2	4	-0.05	3.8E-02	excluded
OCIAD1	69235	ES	4:05	3	6	0.01	3.8E-02	included
DCAF11	26835	ES	5	4	6	0.00	3.8E-02	included
CALU	81705	ES	5	4	6	-0.01	3.8E-02	excluded
NELFA	68541	ES	2:03	1	4	0.02	3.8E-02	included
COL23A1	74865	ES	17	16	18	0.01	3.8E-02	included
MRPS28	97992	ES	4.2	1	5	0.04	3.8E-02	included
FNBP1	87879	ES	12	10.3	14.2	0.03	3.8E-02	included
TMEM230	58644	ES	1.2:2	1.1	4	0.00	3.8E-02	included
ZFYVE27	12743	ES	1.2:2.1:2.2:3	1.1	4	0.02	3.8E-02	included
CBWD3	86512	ES	11	10	12	0.00	3.8E-02	included
EEF1D	85454	ES	6:7.2:8.1	1	8.2	-0.02	3.8E-02	excluded
BABAM1	48260	ES	1.2:2.1	1.1	2.2	-0.02	3.8E-02	excluded
DMXL1	73066	ES	34	33	35	0.03	3.8E-02	included
UXS1	54856	ES	3.2	2	4	0.00	3.8E-02	included
TAZ	90593	ES	5:06	4	8.1	-0.04	3.8E-02	excluded
KIAA1191	74644	ES	3	2	4	-0.01	3.8E-02	excluded
GZF1	58842	ES	2	1	3	0.00	3.8E-02	included

FAM13B	73500	ES	14	13	15	-0.04	3.8E-02	excluded
LMF2	62835	ES	9.2:10:11.1	9.1	11.2	0.00	3.8E-02	excluded
PDCD10	67554	ES	7	6	8	0.00	3.8E-02	excluded
PPT2-EGFL8	75759	ES	3	2	4	0.01	3.8E-02	included
DRG2	39573	ES	2	1	3.1	-0.01	3.8E-02	excluded
EIF2A	67283	ES	5.1:5.2	4	6	0.00	3.8E-02	included
TSPAN9	19753	ES	4	3.2	5	-0.01	3.8E-02	excluded
IQCK	93914	ES	5:6.1:6.2:6.3	4	7.1	-0.05	3.8E-02	excluded
F3	3816	ES	5	4	6	0.01	3.8E-02	included
ETS1	19411	ES	9.1:9.2	8.1	10.1	0.00	3.8E-02	excluded
TMUB2	41814	ES	2.2:2.3:2.4:2.5:3	2.1	4.3	-0.03	3.8E-02	excluded
USP8	30593	ES	4	3	5	0.01	3.9E-02	included
MST1	64900	ES	4	3	5	0.01	3.9E-02	included
VGLL4	63399	ES	4:05	3	8	-0.01	3.9E-02	excluded
KRIT1	80428	ES	1.5:2.1	1.4	2.2	0.02	3.9E-02	included
ARMC8	66966	ES	2.2:3	1	4	0.04	3.9E-02	included
CDC123	10771	ES	9	8	10	0.00	3.9E-02	included
GTF2IRD2B	80106	ES	14	13.2	15	0.01	3.9E-02	included
MUM1	46457	ES	5.1:5.2	4	6	-0.03	3.9E-02	excluded
HSD17B4	73082	ES	14	13	15	-0.01	3.9E-02	excluded
PUM1	1449	ES	5:6:7:8	4	9	-0.01	3.9E-02	excluded
NAPA	50657	ES	3	1	4	0.00	3.9E-02	included
FAXDC2	74240	ES	4	3	5.2	0.00	3.9E-02	included
LAMTOR2	8170	ES	3	2	4.2	0.00	3.9E-02	included
IKBKG	90654	ES	7	6.2	8.1	0.00	3.9E-02	included
NAGPA	33873	ES	6	5	7	-0.02	3.9E-02	excluded
FBXO11	53519	ES	16	15	17	0.00	3.9E-02	excluded
TMEM39B	1549	ES	5:06	3	7.2	0.02	3.9E-02	included
PTDSS2	13661	ES	2.2	1	3	0.01	3.9E-02	included
ING4	19915	ES	4	2	5.1	0.00	3.9E-02	included
YLPM1	101298	ES	18	17.1	19	-0.02	3.9E-02	excluded
ANXA11	12346	ES	6	5.3	7	0.00	3.9E-02	included
ZNF410	28329	ES	12	11	13	-0.01	3.9E-02	excluded
MRPL48	17725	ES	7	5	8	-0.01	3.9E-02	excluded
RNF4	68572	ES	11	9.1	12	0.00	3.9E-02	included
CCNL1	67383	ES	7	6	9	0.04	3.9E-02	included
SERPINB8	45738	ES	1.2:2	1.1	3	-0.03	3.9E-02	excluded
RHOC	4246	ES	1.2:2.2:2.3	1.1	3	0.00	3.9E-02	excluded
EXD3	88299	ES	26	25	27	-0.01	3.9E-02	excluded
DCAF11	26837	ES	3.1:3.2	2.5	4	0.00	3.9E-02	included
ZNF692	10559	ES	5.1:5.2	4	6.1	0.01	4.0E-02	included
IL3RA	88385	ES	3:04	2	5	0.01	4.0E-02	included
SUV420H1	17298	ES	6	5	7	-0.02	4.0E-02	excluded
TGFBR1	87053	ES	4.2	3	5	0.01	4.0E-02	included
BIN1	55196	ES	13:14:15:16	12	17	0.00	4.0E-02	included
SLC12A4	37160	ES	4:5:6:7:8	1	9	0.00	4.0E-02	excluded
TRPT1	16583	ES	2.2	1	3	0.02	4.0E-02	included
PKP4	55685	ES	4	3	5	0.00	4.0E-02	excluded
NME1	42503	ES	3	1	4	0.00	4.0E-02	included
SFTA3	27264	ES	03:04.2	2	5	0.04	4.0E-02	included
SNX2	73124	ES	3	2	4	0.00	4.0E-02	included
ATXN2L	35850	ES	22.4:22.6	22.1	22.7	0.01	4.0E-02	included
VPS51	16758	ES	3	2	4	-0.01	4.0E-02	excluded
RPS11	50954	ES	2.1:2.2:2.3	1	3.1	0.03	4.0E-02	included
RPRD1A	45206	ES	3	1	4	-0.01	4.0E-02	excluded
LRR1	27428	ES	5	3	7	-0.03	4.0E-02	excluded
MRPL55	10174	ES	1.2:2.2	1.1	2.5	0.03	4.0E-02	included
SRPX	88798	ES	5	4	6	0.00	4.0E-02	included
NEO1	31597	ES	27	26	28	0.01	4.0E-02	included
BRF1	29618	ES	12.3:13.2:14.2:15.2:16:17:18:19:20.1	12.2	20.2	0.00	4.0E-02	included
ST7L	4209	ES	17	16.1	18	-0.06	4.0E-02	excluded

MRPL55	10122	ES	2.2:2.3:2.4:2.5	1.2	2.9	0.01	4.0E-02	included
SCYL1	16857	ES	17.1	16.2	17.3	0.00	4.0E-02	included
ABCB6	57633	ES	2	1	3	0.02	4.0E-02	included
CYTH1	43890	ES	11.2:13.1	11.1	13.2	-0.03	4.0E-02	excluded
DLD	81382	ES	06:07.1	5	7.2	-0.02	4.0E-02	excluded
UBXN11	1256	ES	4:5:6:7:8:9	2	10	0.03	4.0E-02	included
MARK2	16543	ES	18	17	19	0.01	4.0E-02	included
GSR	83300	ES	8	7	9	0.00	4.0E-02	excluded
NPRL3	32818	ES	1.4:2:3:4:5	1.3	6	0.00	4.0E-02	excluded
FAM168A	17695	ES	6:07	5	8	-0.01	4.0E-02	excluded
XAF1	38791	ES	7	6	8	-0.05	4.0E-02	excluded
DNAJC19	67761	ES	5.1	4	6	-0.03	4.0E-02	excluded
ZMYND12	2070	ES	3	2	4	-0.02	4.1E-02	excluded
ATF1	21714	ES	5	4	6	-0.01	4.1E-02	excluded
DAPK2	31077	ES	13.3:13.4:14:15.1:15.2	13.1	15.3	0.01	4.1E-02	included
MT1A	36483	ES	2	1	3	0.03	4.1E-02	included
ETFA	31941	ES	4	1	5	0.01	4.1E-02	included
SHISA5	64695	ES	3.2	1.3	4.1	0.00	4.1E-02	excluded
HMGN1	60614	ES	6.1:6.2:7	5	8.2	0.04	4.1E-02	included
MVK	24337	ES	6	5	7	-0.01	4.1E-02	excluded
RNF14	73849	ES	5:06:07	4	8	0.00	4.1E-02	excluded
CCND3	76158	ES	3.2:4	3.1	5	-0.06	4.1E-02	excluded
UBE2F	58188	ES	3	2	5	0.00	4.1E-02	excluded
TPD52	84281	ES	5	1	6	0.00	4.1E-02	included
VPS37A	82797	ES	3	1	4	-0.01	4.1E-02	excluded
TIMM23B	11532	ES	2:03	1	4	0.06	4.1E-02	included
TFDP1	26388	ES	13.1:13.2:13.3	12	14	-0.03	4.1E-02	excluded
VEZT	23759	ES	7	6.2	9	0.00	4.1E-02	included
ECM1	7482	ES	7	6	8	-0.01	4.1E-02	excluded
PTPN12	80201	ES	5	4	6	0.00	4.1E-02	excluded
TUBD1	42815	ES	4	3	5	-0.04	4.1E-02	excluded
MRPL48	17718	ES	10:11.1	8	12	0.00	4.1E-02	included
ACY1	390891	ES	8.1:11:12:13:14	7.2	15	0.03	4.1E-02	included
AGTRAP	679	ES	3	1	5	0.00	4.1E-02	included
RRM2B	84774	ES	2	1	3.1	-0.06	4.1E-02	excluded
FRG1B	58885	ES	4	3	5	0.02	4.1E-02	included
MLLT4	78456	ES	16	15	17	-0.04	4.1E-02	excluded
PCCA	26191	ES	2	1	3	0.01	4.1E-02	included
IQCE	78628	ES	3:04	1	5.2	0.02	4.1E-02	included
NPHP1	54931	ES	8.2:9.1	8.1	9.2	-0.07	4.1E-02	excluded
DCTD	71246	ES	2.1:2.2:3:4	1.1	5	0.01	4.1E-02	included
PSMG2	44703	ES	5:06	4	7.1	0.00	4.2E-02	included
LGALS3BP	43969	ES	2.4	2.2	3.1	0.00	4.2E-02	included
GNLY	54378	ES	2.2	1	3	-0.02	4.2E-02	excluded
C19orf66	47453	ES	4.1:4.3	3	5	-0.01	4.2E-02	excluded
CDC25B	58603	ES	7	6	8	0.00	4.2E-02	included
CELF2	10740	ES	15	14.2	16.1	0.00	4.2E-02	excluded
TMEM159	34427	ES	5:06	4	7	-0.04	4.2E-02	excluded
TRDMT1	10888	ES	5:06	4	7	0.02	4.2E-02	included
TRIM16L	39635	ES	9:10	8.2	11	0.00	4.2E-02	included
MYD88	64031	ES	3.1	2	4	0.00	4.2E-02	included
SALL1	36402	ES	3	1	4	0.00	4.2E-02	excluded
DOLPP1	87822	ES	6	5	7	-0.01	4.2E-02	excluded
ZNF384	19926	ES	5	4	6.1	0.02	4.2E-02	included
FAM175A	69801	ES	7	6	8	0.01	4.2E-02	included
BTBD10	14453	ES	2	1	4.1	-0.03	4.2E-02	excluded
NAP1L1	23480	ES	7.2:7.4	6	7.5	0.01	4.2E-02	included
NF2	61628	ES	15:16.1	14	17	0.01	4.2E-02	included
COPS5	84063	ES	3	2.3	5	0.00	4.2E-02	excluded
ELF2	70613	ES	11	10.2	12	-0.01	4.2E-02	excluded
FLNA	90563	ES	36	35	37	0.00	4.2E-02	excluded
SLC25A19	43434	ES	2.1:2.2	1	4.2	0.05	4.2E-02	included

PPP2R5C	29324	ES	8	4	9	-0.03	4.2E-02	excluded
NCBP2	68266	ES	3.2:4.1	3.1	4.2	0.01	4.2E-02	included
UPP1	79638	ES	7	6.1	9	0.02	4.2E-02	included
AAAS	22020	ES	5	4	6	0.00	4.2E-02	included
PSMA4	32107	ES	7.2:8	6	10	-0.03	4.2E-02	excluded
IFT57	66018	ES	2	1	3	0.00	4.2E-02	included
SS18	44965	ES	7	3	9	0.00	4.2E-02	excluded
ECHDC2	3034	ES	04:05.1	2.1	6.2	-0.01	4.2E-02	excluded
RELA	16905	ES	4.1:4.2:5	3	6.1	0.00	4.2E-02	included
NASP	2717	ES	06:07.2	3	8	-0.01	4.2E-02	excluded
GMFG	49779	ES	4	3.2	5	0.00	4.2E-02	included
TMEM106B	78805	ES	4	3	5	0.00	4.2E-02	excluded
FKBP10	40949	ES	2	1	3	0.00	4.3E-02	included
MYO5B	45496	ES	30	29	31	0.01	4.3E-02	included
RFX5	7608	ES	9	8	11	0.00	4.3E-02	included
GOPC	77346	ES	2.2:4	2.1	5.1	0.00	4.3E-02	excluded
ATP5A1	300057	ES	4.2:5	3.2	6.1	-0.05	4.3E-02	excluded
ZNF540	49544	ES	5:06	4	7	-0.01	4.3E-02	excluded
CBWD3	86513	ES	8:09	7	10	0.01	4.3E-02	included
WWOX	37683	ES	4	3	5	0.00	4.3E-02	included
RBM10	98324	ES	6	5	7	0.02	4.3E-02	included
SEPT6	89969	ES	12	11.1	13.1	0.02	4.3E-02	included
SPATA20	42425	ES	10	9	11.1	0.00	4.3E-02	excluded
EIF3M	14852	ES	3	2.2	5	0.01	4.3E-02	included
CARS	13949	ES	2	1	4	0.00	4.3E-02	excluded
C14orf159	28860	ES	5.2	4	7	0.03	4.3E-02	included
SAP30BP	43479	ES	9:10	8	11	0.00	4.3E-02	excluded
SERGEF	14558	ES	12	11	14	0.01	4.3E-02	included
DMKN	49159	ES	9:11	8	12	0.07	4.3E-02	included
MYL4	42065	ES	4	3.2	5	0.01	4.3E-02	included
ERGIC3	59177	ES	9	8	12	0.00	4.3E-02	included
INIP	87252	ES	4	3	5	-0.01	4.3E-02	excluded
PIP5K1A	7580	ES	3	2.2	4	0.02	4.3E-02	included
CCT2	23325	ES	16	15	17	0.00	4.3E-02	included
VEZT	23788	ES	3	1	4	-0.01	4.3E-02	excluded
ST3GAL3	2298	ES	7.2:8	6	9	0.00	4.3E-02	included
TTC27	53178	ES	2:3:4:5:6:7:8	1	9	0.00	4.3E-02	included
GTPBP8	66132	ES	4	1	6	-0.03	4.3E-02	excluded
COMMD7	58959	ES	2	1	3.2	-0.01	4.3E-02	excluded
TMEM135	18207	ES	5	4	7	0.02	4.3E-02	included
KIF27	86702	ES	12.1	11	13	0.01	4.3E-02	included
RBMS3	63803	ES	11	10	12	0.02	4.3E-02	included
SPATS2L	56739	ES	3	1.3	4	-0.02	4.3E-02	excluded
MORN3	24888	ES	5	4	6	0.04	4.3E-02	included
SURF4	88039	ES	5.1:5.2	4	7.1	0.00	4.3E-02	excluded
SNAPC5	31275	ES	1.2:2.1:2.2	1.1	3.1	0.01	4.3E-02	included
CPNE1	59186	ES	8.2	7	9	0.02	4.3E-02	included
ATG4D	47541	ES	4.1	3.2	5	0.05	4.3E-02	included
PDCD6	71428	ES	5.1:6.1	4	7	0.01	4.3E-02	included
DOLPP1	87823	ES	3	2	4	0.00	4.4E-02	included
EIF4G3	960	ES	10	9	12	-0.02	4.4E-02	excluded
CCDC24	2528	ES	4.2:5:6.1	3.2	6.2	0.03	4.4E-02	included
R3HDM4	46355	ES	2:3.1:3.2	1	3.3	-0.01	4.4E-02	excluded
SPAG16	57340	ES	02:03.1	1.1	3.2	0.00	4.4E-02	excluded
METTL23	43645	ES	1.2:1.3:2	1.1	3	0.03	4.4E-02	included
CTNND1	15964	ES	2.2:2.3:5:6:7	2.1	8	-0.01	4.4E-02	excluded
RFC2	80075	ES	5	4	6	0.01	4.4E-02	included
AIMP2	78705	ES	4	1.2	5	0.00	4.4E-02	included
EIF6	59077	ES	2.4:3	2.2	4	0.00	4.4E-02	included
NDUFC1	70626	ES	3	1	5	0.02	4.4E-02	included
CAD	52954	ES	13	12	14	-0.01	4.4E-02	excluded
UBA5	66823	ES	3:04	2.1	5	0.01	4.4E-02	included

SH3GL3	32281	ES	2:03:04	1	5	0.01	4.4E-02	included
FUT8	28013	ES	10:11	9	12	0.00	4.4E-02	included
PORCN	88986	ES	5:06	4	9	0.00	4.4E-02	included
MAPKAP1	87581	ES	11	10	12	0.00	4.4E-02	included
RNPS1	33251	ES	8	7	9	0.00	4.4E-02	included
GTPBP3	48291	ES	6.1	5.2	6.3	0.00	4.4E-02	included
GABARAPL1	20406	ES	2.7:2.8:2.10:2.11:2.12	2.6	3	0.00	4.4E-02	included
DCAF8	8441	ES	18	17	19	-0.02	4.4E-02	excluded
CSNK2A1	58463	ES	2	1	3	-0.01	4.4E-02	excluded
PLAGL1	77995	ES	7	6.4	8	-0.04	4.4E-02	excluded
COMMD2	67230	ES	5	4	6	-0.01	4.4E-02	excluded
ABI2	57054	ES	5.3:7:8:10:11:12	5.2	14	-0.05	4.4E-02	excluded
BCL2L13	60994	ES	5	4	6	0.00	4.4E-02	excluded
ATP6V1D	28050	ES	7	6	8	0.00	4.4E-02	excluded
SLC25A45	16837	ES	4.3:5.1:5.2:6.1	4.2	6.2	-0.06	4.5E-02	excluded
BBS2	36471	ES	14	13	15	0.00	4.5E-02	included
RBM39	59249	ES	5	3	6	-0.02	4.5E-02	excluded
ATP2C1	66764	ES	29.1	28	30	0.02	4.5E-02	included
FAM193B	74801	ES	7:08	6	9	-0.03	4.5E-02	excluded
STAT6	22526	ES	4	2.1	5	0.00	4.5E-02	excluded
FLAD1	91157	ES	2.1:2.2:3:4.1:4.2:4.3	1.3	6.1	0.03	4.5E-02	included
TVP23C-CDRT4	39350	ES	3.1:3.2	2	4	0.00	4.5E-02	included
ABCE1	70753	ES	14	13	15	-0.04	4.5E-02	excluded
CPSF7	16216	ES	5.2	3	6.1	-0.01	4.5E-02	excluded
GLYR1	33865	ES	6	5	7	0.01	4.5E-02	included
FCGR2B	8682	ES	7	6	8	-0.01	4.5E-02	excluded
ARHGEF12	19165	ES	5	4	6	-0.03	4.5E-02	excluded
MLH3	28471	ES	5	4	6	-0.03	4.5E-02	excluded
ALOX15B	39085	ES	9	8	10.1	-0.03	4.5E-02	excluded
TPM1	31005	ES	3.1:3.2	2.2	5.2	0.02	4.5E-02	included
ZNF131	71930	ES	7.1:7.4	6	8	-0.01	4.5E-02	excluded
WDR35	52751	ES	23:24.1:24.2	22.1	25	0.00	4.5E-02	included
ATL2	53252	ES	3:05	1	6	0.01	4.5E-02	included
MRPL48	17728	ES	5:07	4	8	0.05	4.5E-02	included
ARPC1B	80610	ES	5	4.2	6	-0.01	4.5E-02	excluded
WDR44	89937	ES	15	14	16	0.00	4.5E-02	included
TMEM134	17235	ES	2.2:3.1	2.1	3.2	-0.02	4.5E-02	excluded
SFTA3	27258	ES	3:4.1:4.2:4.3	2	5	-0.01	4.5E-02	excluded
SLC25A29	29265	ES	2:3.2:3.3:3.4:3.5	1	3.7	0.03	4.5E-02	included
RHOC	4239	ES	2.1:2.2:2.3:2.4	1.1	3	0.00	4.5E-02	excluded
ZNF302	48984	ES	3:4:5.1:5.2:6.1	2	6.2	-0.01	4.5E-02	excluded
ZNF74	61152	ES	3	2	4	-0.05	4.5E-02	excluded
TSC2	33192	ES	33	32	34	0.01	4.5E-02	included
C11orf74	15439	ES	6	5	7	0.01	4.5E-02	included
PCMT1	78113	ES	3.1:3.2	2	5	0.00	4.5E-02	excluded
FAM131A	67936	ES	4	2	6.2	0.04	4.5E-02	included
PRSS16	75694	ES	4:5:6:7:8:9	2	10	0.02	4.5E-02	included
HADH	70284	ES	9	8	10	0.00	4.5E-02	excluded
STK16	57665	ES	6	5	7	0.00	4.6E-02	included
ISY1	66701	ES	7	6	8	0.00	4.6E-02	excluded
ZFR	96876	ES	16	15	17	0.01	4.6E-02	included
ARMC6	48573	ES	5:06	1.1	7	0.00	4.6E-02	excluded
NME6	64632	ES	2.1	1.1	3.2	0.01	4.6E-02	included
MCTP2	32588	ES	21:22	19	23	0.01	4.6E-02	included
TMBIM6	21615	ES	2.2	1	3.2	0.00	4.6E-02	excluded
HAUS1	45382	ES	3:04:05	2.1	6	-0.01	4.6E-02	excluded
COL1A1	106078	ES	32	31	33	0.01	4.6E-02	included
ASPH	83976	ES	4	3	5	-0.01	4.6E-02	excluded
NPHP3	66812	ES	17	16	18	-0.05	4.6E-02	excluded
GGA3	43400	ES	9	8	10	0.00	4.6E-02	included
C19orf43	47855	ES	2.1:2.2	1.2	3	0.01	4.6E-02	included
POLR2D	55249	ES	4	3	5	0.01	4.6E-02	included

PACRGL	68904	ES	6:08:09	5	13.1	-0.02	4.6E-02	excluded
DNAJC1	10978	ES	3	2	4.1	0.00	4.6E-02	included
ANAPC10	70751	ES	1.2:2	1.1	3	0.01	4.6E-02	included
AREL1	28415	ES	5	4	6	0.00	4.6E-02	excluded
PLEKHB2	55379	ES	07:08.1	6	9.2	0.00	4.6E-02	excluded
STX16	59983	ES	1.5:3	1.4	5.1	0.03	4.6E-02	included
EED	18182	ES	8:09	7	11.2	-0.01	4.6E-02	excluded
CLEC16A	34007	ES	6	5	7	0.00	4.6E-02	included
TARS2	7479	ES	5:06	4.2	7	0.00	4.6E-02	excluded
PRPF3	7467	ES	5	3	6	0.01	4.6E-02	included
NHP2	74843	ES	3	2	4	0.00	4.6E-02	included
NFXL1	69189	ES	19	18	20	0.00	4.6E-02	excluded
RAP1B	22940	ES	5	4	6	0.00	4.7E-02	excluded
GDI1	90612	ES	2	1	3.2	0.00	4.7E-02	included
SYNE4	49323	ES	3:04	2	5	0.01	4.7E-02	included
CX3CL1	36543	ES	2.3	1	3	0.00	4.7E-02	included
ENOSF1	44469	ES	7	5	8	-0.01	4.7E-02	excluded
MTMR3	61692	ES	3	2	4	-0.01	4.7E-02	excluded
EIF3C	190574	ES	2.4:3:4:5.1:5.2:6:7:8:9<br>:10:11:12	1	13	-0.07	4.7E-02	excluded
CD46	9663	ES	8	6	9	-0.04	4.7E-02	excluded
LMBR1L	21522	ES	4.1:4.2:5	3	6.1	0.02	4.7E-02	included
RAD51AP1	19780	ES	10.1	9.2	11	-0.02	4.7E-02	excluded
MS4A7	16087	ES	4:5:6:7.1	3	7.2	-0.01	4.7E-02	excluded
SLC2A11	61351	ES	5	4	6	0.00	4.7E-02	excluded
PSMC1	28816	ES	3	2	4	0.00	4.7E-02	included
PLS3	89928	ES	4	3	6	0.00	4.7E-02	excluded
AMN1	21014	ES	5	1	6	0.02	4.7E-02	included
URI1	48867	ES	3	2	5	0.02	4.7E-02	included
PDXK	60790	ES	8	7	9	0.00	4.7E-02	included
C17orf62	44387	ES	1.2:3.2	1.1	4.2	0.00	4.7E-02	excluded
ZNF480	51442	ES	3	2	4	0.01	4.7E-02	included
ELP2	45229	ES	4.1:4.2:5:6:7:8.1:9:10:<br>11:12:13:14:15:16:17	3	18	0.00	4.7E-02	included
LRR1	27427	ES	4:05	3	7	-0.05	4.7E-02	excluded
NTAN1	34131	ES	2	1	3	0.02	4.7E-02	included
TTLL3	63224	ES	5:6.3:6.5:7	3	8.1	-0.02	4.7E-02	excluded
RREB1	75251	ES	13	12	14	0.02	4.7E-02	included
PDIA5	66460	ES	11	10	12	0.01	4.7E-02	included
DNAJA3	33726	ES	2	1	3	-0.04	4.7E-02	excluded
PHB2	20045	ES	5.3:6.1	5.1	6.2	0.00	4.7E-02	included
OCRL	90046	ES	19	18	20	0.02	4.8E-02	included
NARFL	32995	ES	1.3	1.1	1.5	-0.01	4.8E-02	excluded
MYL12A	44490	ES	2	1.2	4	0.00	4.8E-02	excluded
NELFA	68542	ES	3	1	4	0.00	4.8E-02	included
INPP4A	54637	ES	5.2:6:7:8:9:10:11:12:1<br>3:16.2:17:18.1:19:20:	5.1	22.2	-0.03	4.8E-02	excluded
ARAP2	68995	ES	24	23	25	0.03	4.8E-02	included
CNOT4	81887	ES	13	10.2	14	0.03	4.8E-02	included
ARFGAP2	15659	ES	3	2	4.2	0.00	4.8E-02	included
ABCB8	82312	ES	3	1	5	-0.01	4.8E-02	excluded
C16orf13	32916	ES	3:04	2	5	0.00	4.8E-02	excluded
CHEK1	19314	ES	12	11	13.1	0.01	4.8E-02	included
PCBP2	22053	ES	13	12	14.1	0.00	4.8E-02	excluded
MEGF6	317	ES	26:27:00	25	28	-0.02	4.8E-02	excluded
C1orf116	9608	ES	2	1	3	-0.03	4.8E-02	excluded
YPEL5	53094	ES	4	3.2	5	-0.05	4.8E-02	excluded
PAIP2	73593	ES	4	3	5	0.00	4.8E-02	included
PPP2CB	83304	ES	2.2:3:4:5:6:7.1	2.1	7.2	0.00	4.8E-02	excluded
SLC12A6	29861	ES	7	6	8	-0.01	4.8E-02	excluded
CMTM7	63815	ES	03:04.1	2	4.2	0.04	4.8E-02	included
PHF8	89236	ES	15	14	16	0.02	4.8E-02	included

RABL3	66379	ES	6	5.2	7	-0.01	4.8E-02	excluded
TSC2	33199	ES	3	1	4	0.00	4.8E-02	excluded
CHP1	30075	ES	5	4	6	0.00	4.8E-02	included
ECHDC1	77466	ES	7:08	6.3	10.2	0.03	4.8E-02	included
NDUFS2	8598	ES	3	2	4	0.00	4.8E-02	included
SP140L	57891	ES	12	11	13	-0.02	4.8E-02	excluded
FAM86A	33886	ES	04:05.2	3.1	6	0.02	4.9E-02	included
CLASP1	55174	ES	25	24	26	-0.01	4.9E-02	excluded
C1orf54	7456	ES	7	6	8	0.01	4.9E-02	included
GBAS	79772	ES	3	2	5	0.00	4.9E-02	included
SOS1	53315	ES	24	23	25	0.01	4.9E-02	included
NDUFS4	72010	ES	4.1:4.2	3	5	0.00	4.9E-02	excluded
RHOT1	40186	ES	19.3:20	19.1	22	0.02	4.9E-02	included
GRIPAP1	89053	ES	13	12	14	0.00	4.9E-02	excluded
TRIP4	31122	ES	8	7	9	0.01	4.9E-02	included
METTL14	70436	ES	10	9	11	0.01	4.9E-02	included
C11orf49	15625	ES	5	3	7	0.00	4.9E-02	excluded
NDUFA3	51783	ES	3	2	4.1	0.00	4.9E-02	excluded
UQCC1	59091	ES	09:10.1	7.2	11.1	0.00	4.9E-02	included
ENY2	84886	ES	5	4.3	6	0.00	4.9E-02	excluded
SMARCD1	21643	ES	12	11	13	0.00	4.9E-02	included
NFE2L1	42160	ES	6	5.2	7	0.02	4.9E-02	included
ZNF280D	30780	ES	10.2	9.2	11	-0.03	4.9E-02	excluded
ATXN2L	35848	ES	22.2:22.3:22.4:22.6	22.1	22.7	0.03	4.9E-02	included
BANP	37989	ES	16	15	17	-0.03	4.9E-02	excluded
IKBKB	83599	ES	03:04.1	1.4	5	0.00	4.9E-02	excluded
RPS3A	96812	ES	2.1:2.2	1.3	4.1	0.00	4.9E-02	included
DIO1	3080	ES	3	2.2	4	0.02	4.9E-02	included
RDX	18651	ES	3.2	2	4	0.00	4.9E-02	included
TP53I11	15496	ES	5.1:5.2	1.2	6.2	-0.03	4.9E-02	excluded
TOM1L1	42548	ES	3.2:3.3	2	4	0.00	4.9E-02	included
IMPA2	44664	ES	4	3.2	5	-0.01	4.9E-02	excluded
SRSF6	59434	ES	3	2	4	0.04	4.9E-02	included
AFMID	43800	ES	7	6	8	0.03	5.0E-02	included
TMEM107	39117	ES	3.1:3.2:3.4:3.5	2	3.7	0.04	5.0E-02	included
CRAT	87827	ES	3	2	4.1	-0.03	5.0E-02	excluded
KDM2B	24881	ES	20	19	21	0.01	5.0E-02	included
CDK10	38118	ES	4:05	2.2	6	-0.05	5.0E-02	excluded
BCAP29	81364	ES	3	1	4	0.00	5.0E-02	excluded
CORO7	33672	ES	8	7.1	9	-0.04	5.0E-02	excluded
IFT43	28536	ES	2	1	3	0.01	5.0E-02	included
TSEN15	9209	ES	4	3	5.1	-0.01	5.0E-02	excluded
ZNF821	37507	ES	4	3.3	6	0.02	5.0E-02	included
MAPKAPK5	93328	ES	7	6	8	0.01	5.0E-02	included
ELP2	45235	ES	4.1:4.2:7:8.1:9:10:11:12:13:14:15:16:17	3	18	0.01	5.0E-02	included
RHOA	64858	ES	4:05	2	6	0.00	5.0E-02	excluded
TBC1D5	63663	ES	22	21	23	0.02	5.0E-02	included
TMEM147	49214	ES	3:04	2	5.1	0.00	5.0E-02	excluded
SUV420H2	52072	ES	3	2	4	-0.02	5.0E-02	excluded
UBE2D4	79374	ES	4	3	5.1	0.01	5.0E-02	included
C16orf74	37893	ES	3.4:3.5	2	4	-0.02	5.0E-02	excluded
FAM219B	31798	ES	3.1:3.2	2	4	-0.02	5.1E-02	excluded
TMX3	45757	ES	8	6	9.1	0.01	5.1E-02	included
CD44	15300	ES	3.1:3.2:4:5:12.1:13:14:15:16.1:16.2:17.1	2.1	17.2	0.01	5.1E-02	included
LRRFIP2	63958	ES	23:24	22	25	-0.01	5.1E-02	excluded
MAEA	68473	ES	8	7	9	0.00	5.1E-02	included
R3HDM1	55443	ES	16	15	17	0.03	5.1E-02	included
UBA3	65565	ES	6:07	5	8	0.00	5.1E-02	included
EPS8L1	52012	ES	16	15.3	17	-0.02	5.1E-02	excluded
ATP6V1H	83833	ES	8	7	9	0.00	5.1E-02	included

HN1L	33122	ES	3.1:3.2	1.2	4	0.00	5.1E-02	excluded
RANBP3	46965	ES	6	5	7	-0.03	5.1E-02	excluded
IRF6	9687	ES	2:03	1	4	0.00	5.1E-02	excluded
ROGDI	33859	ES	4.1	3	5	0.01	5.1E-02	included
ARL5A	55596	ES	5	4	6	0.00	5.1E-02	included
FOXJ3	2069	ES	10	9	11	-0.02	5.1E-02	excluded
PQLC1	46267	ES	4.2	2	5	-0.02	5.1E-02	excluded
NT5E	76918	ES	7	6	8	0.00	5.1E-02	excluded
PCM1	82836	ES	37:38:00	36	39	0.00	5.1E-02	included
STIM2	68991	ES	13	12	14	-0.01	5.1E-02	excluded
SUMF2	79815	ES	3:04	2	6	0.02	5.1E-02	included
RNF135	40139	ES	2:03	1	5	0.00	5.1E-02	included
UCP2	17756	ES	7	6	8	0.00	5.1E-02	included
MED1	40648	ES	3	2	4	-0.06	5.1E-02	excluded
TCEAL4	89753	ES	4.2	3.1	5.2	0.00	5.2E-02	included
DTNBP1	75420	ES	2	1	3	-0.02	5.2E-02	excluded
HDAC8	89469	ES	5:6.1:8.1:8.2	4.1	9	0.01	5.2E-02	included
GABARAPL1	20404	ES	2.10:2.12:2.14	2.6	3	0.00	5.2E-02	excluded
FBXO44	658	ES	6	5.1	7	0.03	5.2E-02	included
MERTK	54978	ES	2.1:2.2	1	3	0.00	5.2E-02	excluded
SLC25A26	65546	ES	8	7	9	0.00	5.2E-02	included
SYNE2	27850	ES	106:107:108	105	109	0.00	5.2E-02	excluded
PHACTR2	77987	ES	8	6	9	-0.03	5.2E-02	excluded
ST3GAL3	2201	ES	16.1:16.2:19.1:19.2:20	15.1	21	-0.03	5.2E-02	excluded
GTF2H5	78271	ES	2	1	3	0.01	5.2E-02	included
KIFC3	36608	ES	28	27	29	0.00	5.2E-02	included
CETP	36511	ES	10	9	11	0.03	5.2E-02	included
MRPL27	42376	ES	5.2:5.3:5.5	5.1	5.6	0.00	5.2E-02	included
NRBF2	11887	ES	2	1	3	0.00	5.2E-02	included
UBE2V1	59758	ES	5	3	6	0.00	5.2E-02	excluded
TMX4	58671	ES	2	1	3	0.00	5.2E-02	excluded
ARMC10	81159	ES	8	6	10	-0.05	5.2E-02	excluded
ZNF561	47373	ES	4	3	6.2	-0.01	5.2E-02	excluded
MUTYH	2615	ES	6.5:7:8:9:10:11:12:13:14:15:16:17:18	5	19	0.02	5.2E-02	included
TATDN1	138625	ES	1.2:2:3:4.1:5:6	1.1	7	0.04	5.2E-02	included
GSN	87431	ES	15.2	10	16	0.00	5.2E-02	excluded
APOPT1	29460	ES	3:04	2	5	0.03	5.2E-02	included
DNMT3B	58965	ES	22:23	21	24	-0.05	5.2E-02	excluded
ARPP19	30683	ES	2.3:2.4:2.5:2.6:4	2.2	5.2	0.05	5.2E-02	included
RAPGEF1	87968	ES	5	4	6	-0.02	5.2E-02	excluded
YIPF3	76290	ES	3.1	2.5	4.1	-0.05	5.2E-02	excluded
TEX264	65103	ES	1.2:1.3:1.4:3	1.1	4	-0.03	5.2E-02	excluded
TMBIM1	57471	ES	6	5	7	0.00	5.2E-02	included
TAF11	75793	ES	4	3	5	0.00	5.2E-02	excluded
INO80E	36010	ES	6.2:6.3:7:8:9:10	5	11	0.06	5.2E-02	included
SNRNP200	563711	ES	44	43	46.1	0.00	5.2E-02	included
ITM2C	57914	ES	3	1	4	0.00	5.2E-02	excluded
SEC11C	45685	ES	5	4	6	0.00	5.2E-02	included
BIN1	55194	ES	13:17	12	18	-0.02	5.2E-02	excluded
BEST1	16319	ES	8.1:8.2:9:10	7	11.1	-0.01	5.2E-02	excluded
RAF1	63445	ES	8	7.1	9	0.00	5.2E-02	excluded
CASS4	59878	ES	6	5	7	-0.05	5.2E-02	excluded
GCAT	62158	ES	2.1:2.2	1	3	0.03	5.3E-02	included
TUBE1	77292	ES	5	3	6.1	0.01	5.3E-02	included
GOLT1B	20704	ES	3	2	5	0.01	5.3E-02	included
NDUFA13	302226	ES	4.2:4.3:6:7	4.1	8	0.00	5.3E-02	excluded
RAP1B	22948	ES	3.1:3.2:4:5	1.1	6	0.00	5.3E-02	included
SLC25A29	29260	ES	3.4:3.5	2	3.7	0.03	5.3E-02	included
LTC4S	74925	ES	2.1:2.2:3	1	4	0.00	5.3E-02	excluded
UPP1	97461	ES	5	4	6.1	0.02	5.3E-02	included
NEK9	28486	ES	4	3	5	0.00	5.3E-02	excluded

EXOC7	43572	ES	8.2	6	9	0.03	5.3E-02	included
PKD2	69879	ES	6	5	7	-0.01	5.3E-02	excluded
ASB1	58228	ES	2	1	3	0.00	5.3E-02	included
CLN3	35730	ES	7	6	8	0.00	5.3E-02	included
SCAP	64520	ES	4:5:6:7:8:9	3	10	0.00	5.3E-02	included
GTDC1	55510	ES	6.2:7	5.2	10	0.00	5.3E-02	included
WDR20	29345	ES	7.1:7.2	4	9	-0.04	5.3E-02	excluded
MAGOHB	20476	ES	2.1:2.2	1.2	3	0.02	5.3E-02	included
IL17RC	63262	ES	7	6	8.1	0.02	5.3E-02	included
ITM2C	57913	ES	5	4	6	0.00	5.3E-02	included
TMEM107	39136	ES	2	1	3.7	0.03	5.3E-02	included
SAT2	39031	ES	4	3	5.1	0.03	5.3E-02	included
YAF2	21160	ES	5.2:6	2	9.1	-0.02	5.3E-02	excluded
SGCE	80508	ES	3	1	4	-0.01	5.3E-02	excluded
LRRFIP2	63970	ES	7:8:9:10:14:15:16:17	5	18	0.00	5.3E-02	included
CLIP1	24955	ES	9:10	8	11.1	-0.01	5.3E-02	excluded
XRRA1	17802	ES	2	1	4	0.03	5.3E-02	included
SNX1	139181	ES	6:7:8:9:10.1:10.2:11:1	3	16.1	0.00	5.3E-02	excluded
POLR2E	46386	ES	2.1:2.2	1	3	0.05	5.3E-02	included
ODF2	87766	ES	5	2	6	-0.04	5.3E-02	excluded
MEAF6	1798	ES	6:7:8.1:9.1	5	9.2	0.05	5.3E-02	included
BPNT1	9864	ES	8	7	9	0.00	5.3E-02	included
PUM1	1453	ES	3	2.2	4	-0.01	5.3E-02	excluded
KLHL5	69044	ES	2.2	1	3	-0.05	5.3E-02	excluded
TNFAIP8	73071	ES	4	3.3	5	0.00	5.3E-02	included
BCAS4	59785	ES	4:05	3	7	-0.01	5.3E-02	excluded
PRDX5	16641	ES	2	1	4	-0.01	5.4E-02	excluded
ZNF512	53026	ES	2:03	1	4.1	-0.02	5.4E-02	excluded
PPM1B	53416	ES	2	1	3.1	0.00	5.4E-02	included
AGAP8	402438	ES	4	3	5	-0.02	5.4E-02	excluded
ZMIZ1	12300	ES	24	23	25	-0.02	5.4E-02	excluded
FAM195B	44179	ES	2.2	1	2.4	0.00	5.4E-02	excluded
LSR	49087	ES	4	3	5	-0.01	5.4E-02	excluded
LRRFIP1	58132	ES	18:19	17	20	0.00	5.4E-02	included
SLC2A11	61347	ES	10.2	9.1	11	-0.04	5.4E-02	excluded
DMD	88773	ES	75:76:77:78	74	79	-0.01	5.4E-02	excluded
RBM41	89818	ES	5	4	6.1	0.04	5.4E-02	included
EBAG9	84901	ES	7	6	8	0.00	5.4E-02	excluded
EIF5A	38910	ES	4.2	3	5.2	0.00	5.4E-02	excluded
TCEAL4	89749	ES	4.2:5.1	3.1	5.2	0.00	5.4E-02	included
NCAPD2	19871	ES	4	3	5	0.00	5.4E-02	included
MED29	49817	ES	3	2	4	0.00	5.4E-02	excluded
FOSL1	16942	ES	3.1:3.2	2	4	0.02	5.4E-02	included
CNIH4	9957	ES	4	3	5.2	-0.01	5.4E-02	excluded
SKA2	42734	ES	03:04.1	2	5	-0.02	5.4E-02	excluded
TJP2	86538	ES	24	22.1	25	0.05	5.4E-02	included
DNAJA3	33720	ES	12	11	13	-0.02	5.4E-02	excluded
PBDC1	89529	ES	5	4	6	0.00	5.4E-02	included
C5orf28	71957	ES	02:03.2	1	5	-0.02	5.4E-02	excluded
NSFL1C	58500	ES	5.1:5.2:7.2	4	7.3	0.03	5.5E-02	included
MAX	27955	ES	5.2:5.3:5.5	5.1	5.8	0.00	5.5E-02	included
PON3	80530	ES	5	4	6	0.02	5.5E-02	included
FBLN5	28895	ES	4	3	5	-0.01	5.5E-02	excluded
DOCK6	47644	ES	23	22	24	-0.02	5.5E-02	excluded
IL6ST	72082	ES	10	8	11	0.00	5.5E-02	excluded
IRF7	13712	ES	04:05.1	3.2	5.3	0.01	5.5E-02	included
HMCN1	9219	ES	104	103	105	0.04	5.5E-02	included
SEC22C	64300	ES	4	3	7	-0.01	5.5E-02	excluded
ZCCHC17	1464	ES	1.2:2	1.1	4	0.06	5.5E-02	included
TANK	55740	ES	8	7.1	10.1	0.00	5.5E-02	excluded
SMPDL3A	77390	ES	2	1	3	0.02	5.5E-02	included
LRRC42	3101	ES	2	1	3	-0.02	5.5E-02	excluded

USP3	31036	ES	4:05	3.2	7	-0.02	5.5E-02	excluded
CDR2	35555	ES	2	1	3	-0.01	5.5E-02	excluded
ATP5G2	22101	ES	03:04.1	1.3	4.2	0.04	5.5E-02	included
ASMTL	88390	ES	4	3	5	0.00	5.5E-02	included
LAYN	18670	ES	4	3	5	0.01	5.5E-02	included
ATL1	27486	ES	15	13	16	-0.03	5.5E-02	excluded
KIF27	86701	ES	13:14	12.1	15	0.01	5.5E-02	included
REXO2	18841	ES	2:4:5.2:5.3	1	6	0.00	5.6E-02	included
NSMF	88322	ES	7	4	9.2	0.02	5.6E-02	included
MYO5C	30655	ES	5	4	6	0.00	5.6E-02	excluded
PABPC4	1893	ES	11	10.2	12	0.01	5.6E-02	included
POSTN	25675	ES	17:18	16	19	0.05	5.6E-02	included
ARF4	65384	ES	2:3.1:3.2	1	4	-0.05	5.6E-02	excluded
AGAP6	11553	ES	2	1	3	0.02	5.6E-02	included
SUN1	78524	ES	12:13	11	14	-0.01	5.6E-02	excluded
BBS4	31565	ES	7	6	8	0.01	5.6E-02	included
POLR2M	30879	ES	13.2:14.1	13.1	14.2	0.04	5.6E-02	included
FAIM	67015	ES	4	1	6	-0.01	5.6E-02	excluded
VRK3	51151	ES	4	3	5	0.00	5.6E-02	excluded
VEGFA	76332	ES	7.1:7.2:8.1	6	9.1	0.00	5.6E-02	excluded
BAZ2B	55698	ES	10	9	11	-0.02	5.6E-02	excluded
CLEC2D	20253	ES	7.1:7.3:7.4:7.5	6.1	8	-0.04	5.6E-02	excluded
ITGB3BP	3267	ES	2	1	3	0.00	5.6E-02	included
MED12	89397	ES	39.2:40.1	39.1	40.2	0.01	5.6E-02	included
MLH1	63940	ES	3	2.2	4.1	-0.01	5.6E-02	excluded
TMEM258	16286	ES	2.2:2.3:3.1	2.1	3.2	0.00	5.6E-02	excluded
SLC30A6	53157	ES	4	3	6	-0.01	5.6E-02	excluded
DECR1	84407	ES	4.1:4.2:5.1	1	5.2	-0.03	5.6E-02	excluded
POLR2H	67947	ES	2.1:2.2	1	3	0.03	5.6E-02	included
CBWD1	85691	ES	9.1	8	10	0.00	5.6E-02	included
AFMID	43803	ES	10:11.1:11.2	6	12	-0.01	5.7E-02	excluded
AZIN1	84781	ES	3	2	4	-0.02	5.7E-02	excluded
RPL37	71872	ES	3.1	2	4	0.00	5.7E-02	excluded
SLC10A7	70775	ES	13	12	14	-0.04	5.7E-02	excluded
MIF4GD	43419	ES	3.2:4:5	2	6.1	0.02	5.7E-02	included
MARK4	50421	ES	9:10	8	11	0.00	5.7E-02	included
HN1	43370	ES	6	5.1	8.2	0.00	5.7E-02	included
DYNC2LI1	53402	ES	4	3	5	-0.01	5.7E-02	excluded
AGO2	85285	ES	2	1	3	0.01	5.7E-02	included
DDB2	15673	ES	6	5	7	0.01	5.7E-02	included
IP6K2	64777	ES	8.1	7	11.1	0.00	5.7E-02	included
FLOT2	40003	ES	4:05	3	6	-0.03	5.7E-02	excluded
CYFIP1	29680	ES	2	1	3.2	-0.01	5.7E-02	excluded
ST3GAL3	2273	ES	10	9	13	0.01	5.7E-02	included
C10orf137	13422	ES	18	17	19	-0.03	5.7E-02	excluded
RPS10	75781	ES	6	5	7	0.00	5.7E-02	included
FAM86B1	82699	ES	4:6:7.1:7.2:7.3	3.2	8.1	-0.04	5.7E-02	excluded
SUV420H1	17300	ES	5	4	7	0.01	5.7E-02	included
THRAP3	1762	ES	2	1	3	0.01	5.7E-02	included
FAM57A	38254	ES	2	1.3	3	0.01	5.7E-02	included
EPHX2	83164	ES	3:4:5:6	2.2	7	0.00	5.7E-02	included
CPSF3L	94	ES	5.1:5.2:6.1:6.2	4	7.1	0.00	5.7E-02	included
VEGFA	76337	ES	7.1	6	8.1	0.01	5.7E-02	included
EXOC6	12547	ES	10	9	11	-0.01	5.7E-02	excluded
CCBL1	87785	ES	6	5	7	0.00	5.7E-02	excluded
TMEM214	52923	ES	4	3	5	0.00	5.7E-02	included
CDK11A	216	ES	6.2	5	7.1	0.01	5.7E-02	included
IFNGR2	60414	ES	2.1:2.2	1	4	0.00	5.7E-02	excluded
ABLIM1	13211	ES	12	10	13	0.00	5.7E-02	included
UBR3	55899	ES	31	30	32	-0.02	5.7E-02	excluded
EI24	19302	ES	10	9	11.1	0.00	5.8E-02	included
PHKA1	89509	ES	19	18	20	-0.01	5.8E-02	excluded

DAPK2	31078	ES	13.2:13.3:15.2	13.1	15.3	-0.01	5.8E-02	excluded
TCEAL4	89750	ES	4.1:4.2	3.1	5.2	0.04	5.8E-02	included
CIB2	32004	ES	2	1	3	-0.02	5.8E-02	excluded
ARMC6	48565	ES	6	5	7	0.00	5.8E-02	excluded
NOLC1	12944	ES	2:3.2:4.1:4.2:5	1	6.1	0.01	5.8E-02	included
GGA1	62125	ES	13:14	12	15	0.00	5.8E-02	included
SLC43A1	15852	ES	4.3:5:6:7:8:9:10.1	4.2	10.2	0.01	5.8E-02	included
INTS6	25948	ES	06:07.2	5	8	-0.02	5.8E-02	excluded
KIAA0895L	36959	ES	2.1:2.2	1	3.1	-0.02	5.8E-02	excluded
ITGB3BP	3266	ES	9	8	10	-0.02	5.8E-02	excluded
BTN2A1	75671	ES	4	3	5	-0.03	5.8E-02	excluded
ZNF185	90407	ES	11:12	10	13	-0.03	5.8E-02	excluded
EIF3K	49682	ES	1.3:2	1.2	3	0.00	5.8E-02	included
DNAJC17	30047	ES	2	1	3.1	-0.01	5.8E-02	excluded
MFSD1	67454	ES	3.2	2.2	4	0.01	5.8E-02	included
HN1L	33123	ES	3.1	1.2	4	-0.01	5.8E-02	excluded
NVL	9943	ES	12	10	13	0.02	5.8E-02	included
CCT5	71556	ES	4	3	5	0.00	5.9E-02	excluded
RBM26	26100	ES	14	13.2	15	-0.01	5.9E-02	excluded
UIMC1	74696	ES	6.1	5	8	-0.02	5.9E-02	excluded
IL15RA	10679	ES	4	3	5	0.03	5.9E-02	included
HACL1	63587	ES	6	5	7	-0.01	5.9E-02	excluded
OPHN1	89352	ES	21	20	22	0.03	5.9E-02	included
PFDN5	22007	ES	2:4.1:4.2	1	5	0.02	5.9E-02	included
SLC16A4	4111	ES	3	2	4	0.02	5.9E-02	included
TBRG1	19225	ES	4	3	6	0.03	5.9E-02	included
TUBD1	42828	ES	2	1	3	0.03	5.9E-02	included
POLR3E	35549	ES	21.1:21.2	20	22	0.00	5.9E-02	excluded
ME1	76869	ES	2	1	3	0.01	5.9E-02	included
TMEM259	46360	ES	10	9	11	0.00	5.9E-02	excluded
RANBP3	47005	ES	4.1:7:8	1	10.2	0.01	5.9E-02	included
ATXN2	24520	ES	18.1:18.2	17	19	0.01	5.9E-02	included
NBPF1	273490	ES	4	3	8	-0.02	5.9E-02	excluded
DMKN	49175	ES	8:10:11	7	12	0.00	5.9E-02	included
LRRFIP1	58133	ES	15	14	16	-0.01	5.9E-02	excluded
EZH1	41112	ES	5	4.2	6	0.01	5.9E-02	included
DAZAP1	46476	ES	13	12	14	0.01	5.9E-02	included
ZBTB8OS	1612	ES	5	4	6	0.01	5.9E-02	included
LZTFL1	64416	ES	12	11	13	0.00	5.9E-02	included
EPOR	47692	ES	4.1:4.2	3	5	0.05	5.9E-02	included
GYG1	67204	ES	7	6	8	0.00	5.9E-02	excluded
TARBP2	22086	ES	3.1:3.2:3.3	1.2	4.1	0.00	5.9E-02	included
C12orf23	24183	ES	2.2:3.1:3.2	1	4.2	0.03	5.9E-02	included
AMN1	21017	ES	2:03	1	5	-0.03	5.9E-02	excluded
CHID1	13819	ES	3.2:4.2	3.1	5.2	0.00	5.9E-02	excluded
ANAPC11	44208	ES	6	3.4	7.2	0.03	6.0E-02	included
REEP4	82943	ES	6	5	7	0.00	6.0E-02	included
SCEL	26083	ES	12	11	13	-0.02	6.0E-02	excluded
MEAF6	1805	ES	6	5	8.1	0.00	6.0E-02	included
SLMO1	44688	ES	4	3.2	5	0.01	6.0E-02	included
CAPZA2	81551	ES	7	6.1	8.2	0.00	6.0E-02	included
TOM1L1	42557	ES	3.2:3.3:4	2	6.1	0.00	6.0E-02	included
SLC35D2	86966	ES	11	10	12	-0.01	6.0E-02	excluded
INO80E	36009	ES	6.1:6.2:6.3:7:8:9:10	5	11	0.04	6.0E-02	included
HNRNPAB	74844	ES	6	5.3	7	0.01	6.0E-02	included
PIP5K1A	7582	ES	1.2:2.1	1.1	2.2	-0.01	6.0E-02	excluded
GNS	22866	ES	2	1	3.2	0.00	6.0E-02	included
RPS15	46492	ES	1.2:1.4	1.1	1.5	-0.04	6.0E-02	excluded
CCDC180	87000	ES	37:38:39	35	40	-0.04	6.0E-02	excluded
MRPL55	10114	ES	2.2:2.5:2.6:2.8	1.2	2.9	0.01	6.0E-02	included
WDR20	29355	ES	3.1:3.2	1.2	4	-0.01	6.0E-02	excluded
AXL	50027	ES	11	10	12	-0.03	6.0E-02	excluded

FKBP7	56168	ES	3	2	4.1	-0.03	6.0E-02	excluded
CBWD1	271014	ES	5	4.3	6	0.01	6.0E-02	included
ORC3	76970	ES	4	2	5	0.01	6.0E-02	included
THBS3	8033	ES	2.2:3:4	2.1	5	-0.01	6.1E-02	excluded
ECI2	75221	ES	5	4.2	6	0.00	6.1E-02	excluded
ZNF195	13983	ES	9	5.1	13	-0.01	6.1E-02	excluded
TNIP1	74139	ES	18	17	20	0.00	6.1E-02	included
LGALSL	53769	ES	2:03	1	4.2	0.00	6.1E-02	included
TAX1BP1	79062	ES	16.1:16.2:17	14	18	-0.01	6.1E-02	excluded
SRSF11	3395	ES	6.1:6.3	4.2	6.5	0.00	6.1E-02	excluded
IFNLR1	1096	ES	7.1	6	8.1	0.01	6.1E-02	included
TMBIM4	22899	ES	2.2:2.3:4.1:4.2:5	1	6	0.06	6.1E-02	included
RABL2B	62930	ES	2.2:3.1	1	3.2	0.01	6.1E-02	included
AGAP3	82354	ES	10	9	11	0.01	6.1E-02	included
PDIA6	52669	ES	6	5	7	0.00	6.1E-02	excluded
NAP1L1	23483	ES	7.2	6	7.4	0.00	6.1E-02	included
JMJD6	43622	ES	5.2	4	6	-0.02	6.1E-02	excluded
PPIL3	56762	ES	5.1:5.2	3	6	-0.02	6.1E-02	excluded
WDR74	16457	ES	11	10	12	0.00	6.1E-02	excluded
ABI3BP	65842	ES	57	56	58	0.02	6.1E-02	included
UBAP1	86151	ES	2:05	1	6	-0.02	6.1E-02	excluded
XAF1	38802	ES	4.1:4.2:4.3:6	2.1	8	-0.01	6.1E-02	excluded
SNX3	77147	ES	2:03	1.2	4	-0.01	6.1E-02	excluded
GGT1	100018	ES	12	11	13	-0.03	6.1E-02	excluded
CTNND1	15935	ES	21	20	22.1	-0.03	6.2E-02	excluded
CYB561A3	16183	ES	3	2	4.2	0.01	6.2E-02	included
DZIP3	66036	ES	28	27	29	0.00	6.2E-02	excluded
CEP192	44733	ES	13:14	12	15	0.02	6.2E-02	included
ZCCHC10	73330	ES	5.2	4	6.1	0.02	6.2E-02	included
PARP6	31527	ES	18.2	17	19	0.02	6.2E-02	included
SLC4A5	54040	ES	22	21	23	-0.01	6.2E-02	excluded
SORBS3	83020	ES	12.2	11	13	0.01	6.2E-02	included
ARV1	10263	ES	2	1.2	3	0.00	6.2E-02	included
YAF2	21154	ES	5.2:7	2	9.1	-0.01	6.2E-02	excluded
YAF2	21152	ES	3.1:3.2:5.2:7	2	9.1	0.00	6.2E-02	excluded
ARFGAP1	60113	ES	5	4	6	0.00	6.2E-02	excluded
PTK7	76247	ES	17	16.2	18	0.00	6.2E-02	included
ITFG2	19718	ES	2:3:4.1:4.2	1	5	0.05	6.2E-02	included
RBFOX2	61985	ES	12	11.2	14	-0.01	6.2E-02	excluded
FHL1	90191	ES	8:09	7	10	-0.04	6.2E-02	excluded
ATG4D	47538	ES	4.1:5	3.2	6	0.05	6.2E-02	included
UBE2G1	38532	ES	4	3	5	0.01	6.2E-02	included
TTI1	59368	ES	2	1	3.2	-0.02	6.2E-02	excluded
ATG4D	47534	ES	4.1:5:6	3.2	7	0.02	6.2E-02	included
ETFA	31934	ES	3:04:05	1	6	0.00	6.2E-02	included
MPDZ	85874	ES	38	37	39	0.01	6.2E-02	included
MARCH2	47234	ES	2.2	1	3.1	-0.03	6.2E-02	excluded
MAP2K3	39813	ES	10	9	11	0.00	6.2E-02	included
QSOX1	9131	ES	4	3	5	0.00	6.2E-02	included
WIZ	48091	ES	8:09	4	10	-0.03	6.3E-02	excluded
DNM1L	21043	ES	18	17	19	0.03	6.3E-02	included
ZKSCAN5	80657	ES	6	5	7	0.04	6.3E-02	included
HNRNPD	69701	ES	2:03	1.3	4	0.00	6.3E-02	included
TMX2	15919	ES	3.1:3.2:3.3	2	4	-0.04	6.3E-02	excluded
ZNF610	51445	ES	6	5	7	0.01	6.3E-02	included
CCDC159	47685	ES	4	3.1	5	0.02	6.3E-02	included
COL1A2	306246	ES	27:28:29:30:31:33	26	34	0.02	6.3E-02	included
INADL	3245	ES	36	35	37	0.02	6.3E-02	included
CLDND1	65783	ES	3.3:3.4	1	4.1	-0.01	6.3E-02	excluded
GSN	87436	ES	10:12	9	16	-0.05	6.3E-02	excluded
D2HGDH	58418	ES	8:9:10:11.1	6	11.2	-0.04	6.3E-02	excluded
PTGR2	102630	ES	6	5	7	0.01	6.3E-02	included

TRMT11	77438	ES	10	9.1	12	-0.01	6.3E-02	excluded
MYL6	22384	ES	2.1	1.4	3.1	0.02	6.3E-02	included
DLST	28444	ES	2	1	3	0.00	6.3E-02	included
CALCOCO2	42229	ES	5	2	6	0.00	6.3E-02	excluded
KDM6A	88880	ES	14	12	15	-0.02	6.3E-02	excluded
HMGN1	60622	ES	8.2	5	9	0.00	6.3E-02	included
TCEA1	83855	ES	3.1:3.2	2	4.1	0.00	6.3E-02	excluded
CD44	15199	ES	3.2:4:5:6:7:8:9.2:10:1<br>1:12.1:13:14:15:16.1	3.1	16.2	-0.03	6.3E-02	excluded
CAMKK2	24853	ES	17	16	19.1	0.02	6.3E-02	included
UBE2G2	60833	ES	2	1	3	-0.01	6.3E-02	excluded
GDI2	10663	ES	5	4	6	0.00	6.3E-02	included
LSM1	83373	ES	4	2	5	-0.01	6.3E-02	excluded
SEC61A2	10764	ES	9	6	10	-0.04	6.4E-02	excluded
U2AF1L4	49276	ES	3.2	2.2	5	-0.03	6.4E-02	excluded
PIGT	59546	ES	9	8	10	0.00	6.4E-02	excluded
ABHD14A	65160	ES	3:04	2	5	0.00	6.4E-02	excluded
PTK2	85322	ES	6	1	8	0.02	6.4E-02	included
PTPRB	23388	ES	12	11	13	-0.01	6.4E-02	excluded
NLN	72252	ES	2	1	3	-0.05	6.4E-02	excluded
CCNB2	30929	ES	7	6	8	0.00	6.4E-02	included
ITFG2	19719	ES	03:04.1	1	5	0.00	6.4E-02	included
CPSF3L	95	ES	5.2:6.1:6.2	4	7.1	0.00	6.4E-02	included
SDCCAG8	10458	ES	6	5	7	0.01	6.4E-02	included
GMFG	49771	ES	5:6:7.1:7.2	4	7.3	-0.03	6.4E-02	excluded
GGT2	121590	ES	6	4	7	0.04	6.4E-02	included
TMEM258	16287	ES	2.2:3.1	2.1	3.2	0.00	6.4E-02	included
PSMG4	75190	ES	5.1	4	5.6	0.00	6.4E-02	included
CTSB	82669	ES	1.2:5.2	1.1	5.3	0.00	6.4E-02	excluded
STAU2	84166	ES	6	4	7	0.00	6.4E-02	included
LDHA	14617	ES	8.3:9:10.1	8.2	10.2	0.00	6.4E-02	included
ANXA11	12350	ES	4	1.1	5.3	0.01	6.4E-02	included
PGS1	43876	ES	02:03.1	1	3.2	0.01	6.4E-02	included
PNPLA7	88332	ES	2	1	3	-0.01	6.4E-02	excluded
CHCHD3	81836	ES	10:11	9	12	0.00	6.4E-02	excluded
KIAA1191	74646	ES	2	1	3	0.01	6.4E-02	included
APP	60287	ES	10	8	11	0.01	6.4E-02	included
MAN2A2	32518	ES	11	10	12	0.00	6.4E-02	excluded
DDX49	48530	ES	3.2	2.2	4	0.00	6.4E-02	excluded
RASSF7	13693	ES	6.3	6.1	6.5	0.03	6.4E-02	included
AGK	82044	ES	4	2	6	0.00	6.4E-02	included
CD44	15270	ES	3.1:3.2:4:5:7:8:9.1:9.2<br>:10:11:12.1:13:14:15:	2.1	17.2	-0.05	6.4E-02	excluded
CTSB	97868	ES	4	2	5.3	0.00	6.4E-02	excluded
BTN3A2	75634	ES	2.2	1	4.2	-0.02	6.4E-02	excluded
SNX29	34058	ES	5	4	6	-0.05	6.4E-02	excluded
SH3D21	1768	ES	7:08	6	9	0.01	6.4E-02	included
DMKN	101867	ES	8:10:11	6.4	12	0.00	6.4E-02	included
ERGIC1	74565	ES	6.3	6.1	8	0.00	6.4E-02	excluded
ECHDC2	3035	ES	03:05.1	2.1	6.2	0.00	6.4E-02	excluded
CEP57	18400	ES	5	2	6.2	0.00	6.4E-02	included
RBMS1	55728	ES	13	12	14	0.03	6.5E-02	included
NECAP1	20177	ES	4	3	5	0.01	6.5E-02	included
ZNF148	66555	ES	2	1	3.1	-0.01	6.5E-02	excluded
ARHGAP8	62625	ES	20	19	21	0.00	6.5E-02	excluded
COPS8	58099	ES	2	1	3	-0.01	6.5E-02	excluded
TXNRD1	24101	ES	22	21	23	-0.01	6.5E-02	excluded
DCTD	71247	ES	1.2:3:4	1.1	5	0.01	6.5E-02	included
ZNF346	74704	ES	5.1:5.2:6.2	4	7	0.01	6.5E-02	included
CCDC125	72340	ES	10:11	9	12	-0.01	6.5E-02	excluded
CD276	31615	ES	3	1	4.1	0.00	6.5E-02	included
STRAP	20588	ES	2:03	1	4	-0.02	6.5E-02	excluded

COPS7B	57957	ES	5	3	6	0.01	6.5E-02	included
URGCP	79361	ES	4.2:4.3:4.6	3	5	0.01	6.5E-02	included
PLSCR1	67171	ES	3	1	4	0.00	6.5E-02	included
FANCL	53656	ES	06:07.1	5	8	0.00	6.5E-02	included
UHMK1	8746	ES	8	7	9	-0.01	6.5E-02	excluded
KATNA1	78087	ES	5:06	4	7	0.00	6.5E-02	included
TRMT10B	86438	ES	3.2:4.1	2	5	0.03	6.5E-02	included
ORAOV1	17376	ES	03:04.1	1	5.1	-0.01	6.5E-02	excluded
ZFYVE9	3000	ES	6	5	7	0.01	6.5E-02	included
ZNF664	25132	ES	2.1:2.2	1.4	5	-0.01	6.5E-02	excluded
GAK	68414	ES	8	7	9	0.00	6.5E-02	included
YWHAE	38292	ES	4:05:06	3	7	0.00	6.5E-02	included
ZSCAN29	30244	ES	3.1:3.2:4.2:4.3	2	5	0.00	6.5E-02	excluded
ETFA	31935	ES	4:05	1	6	0.01	6.5E-02	included
MYO19	40481	ES	23	22	24	-0.01	6.5E-02	excluded
ANGPTL1	9095	ES	2	1	3	-0.02	6.5E-02	excluded
PXN	24752	ES	4	1	5	-0.02	6.6E-02	excluded
ZNF701	51473	ES	3.1:3.2	1	4	-0.05	6.6E-02	excluded
ATXN2L	35840	ES	22.3:22.4:22.6	22.2	22.7	-0.04	6.6E-02	excluded
RPAIN	38692	ES	04:06.1	3	7	-0.01	6.6E-02	excluded
CDADC1	25877	ES	5	4.2	6	0.02	6.6E-02	included
STOML1	31621	ES	8.1:8.2	7	9	0.00	6.6E-02	excluded
NIPA2	29685	ES	2	1.1	4	-0.03	6.6E-02	excluded
ZNF37A	11312	ES	02:03.1	1	3.2	0.05	6.6E-02	included
NPM2	82919	ES	6	5	7	0.00	6.6E-02	included
PSEN1	28269	ES	14	12	15	0.00	6.6E-02	included
MAX	27951	ES	5.3:5.5:5.6	5.1	5.8	0.00	6.6E-02	excluded
BTF3L4	2994	ES	5	4	6.1	0.00	6.6E-02	excluded
IRF3	51012	ES	1.2:1.3:1.4:2	1.1	3	-0.01	6.6E-02	excluded
IP6K2	64770	ES	8.1:8.2:11.1	7	11.2	-0.01	6.6E-02	excluded
PKIG	59479	ES	3	2.2	4	0.01	6.7E-02	included
B4GALT4	66288	ES	6.2:7:8.1:9.1:9.2:9.3	6.1	9.4	-0.07	6.7E-02	excluded
GPBP1L1	2769	ES	2	1	3	-0.02	6.7E-02	excluded
RAD9A	17182	ES	2	1	3	0.00	6.7E-02	excluded
EVC	96676	ES	23:24	22.1	25	-0.02	6.7E-02	excluded
EEF1D	85457	ES	03:08.1	1	8.2	-0.01	6.7E-02	excluded
ETV1	78837	ES	11	9	12	0.00	6.7E-02	excluded
SIRT3	13597	ES	6.2:7	6.1	8.1	0.00	6.7E-02	excluded
ACOT7	396	ES	12.2	11	13	0.00	6.7E-02	excluded
FCN3	1325	ES	4	3	5	-0.02	6.7E-02	excluded
ZDHHC20	25456	ES	2	1	3	0.01	6.7E-02	included
COL1A2	115448	ES	32	31	33	0.01	6.7E-02	included
NARFL	32988	ES	2.2	1.5	2.4	0.00	6.7E-02	excluded
YIF1B	49608	ES	9.1	8	10	0.01	6.7E-02	included
IL17RC	63273	ES	04:05.1	3.3	5.2	0.00	6.7E-02	included
ARHGEF7	26288	ES	7	6	8	-0.01	6.7E-02	excluded
ABLIM1	13212	ES	11	10	12	0.00	6.7E-02	included
FAM3A	90638	ES	3.1:3.2	2	4	-0.01	6.7E-02	excluded
ZNF138	79835	ES	5	4.2	6	-0.04	6.8E-02	excluded
SAP30BP	43485	ES	3	1	4	0.00	6.8E-02	included
RBBP4	1627	ES	4:06	3.2	7	0.00	6.8E-02	included
SNX1	139178	ES	6:8:9:10.2:11:12:13.1:	3	16.1	0.00	6.8E-02	included
PRUNE	7544	ES	5:06	3	7	-0.02	6.8E-02	excluded
FUZ	51086	ES	2.1:2.2:2.3:2.4	1.2	3	0.05	6.8E-02	included
PAX8	55049	ES	08:09.2	7	10	0.00	6.8E-02	excluded
PML	31663	ES	6.1	4	6.4	0.01	6.8E-02	included
FAM172A	72785	ES	6	5	7	0.01	6.8E-02	included
DUOXA1	30397	ES	1.2:2.1:2.2:3	1.1	5	0.07	6.8E-02	included
PLAUR	50235	ES	6	5	7	0.00	6.8E-02	included
TCF12	30790	ES	12	11	13	0.00	6.8E-02	included
METTL23	43635	ES	1.4:3	1.3	4.1	-0.01	6.8E-02	excluded
GPX3	74124	ES	3	1	4	0.00	6.8E-02	excluded

HSPBAP1	66451	ES	5	4	6	-0.02	6.8E-02	excluded
NUP54	69585	ES	7:08	6	9	0.00	6.8E-02	excluded
RTN3	16533	ES	2	1	4	0.00	6.8E-02	excluded
OCIAD1	69247	ES	3:04	2.1	6	0.00	6.8E-02	included
FAM133B	80446	ES	3	1	4	-0.01	6.9E-02	excluded
PTPRK	77498	ES	19	18	21	0.00	6.9E-02	excluded
C20orf194	58587	ES	26	25	27	0.01	6.9E-02	included
ZSWIM7	39398	ES	3	2	4	-0.02	6.9E-02	excluded
ELN	80054	ES	6:7:8.2:9:10	5	11	0.00	6.9E-02	excluded
NEDD1	23836	ES	03:04.2	2.3	5	-0.02	6.9E-02	excluded
C12orf43	24819	ES	2	1	3.1	-0.03	6.9E-02	excluded
SH2B1	35870	ES	10	9.1	11	0.00	6.9E-02	included
EHD3	53139	ES	4:05	3	6	0.00	6.9E-02	included
GRN	522182	ES	4:5:6.2:7:8:9:10	3	11	0.01	6.9E-02	included
TSPAN31	22729	ES	2.2:3:5.1:5.2	2.1	6.1	0.00	6.9E-02	included
NDUFB5	67707	ES	02:04.2	1	5	0.00	6.9E-02	included
ZSWIM7	39405	ES	1.2:2:3	1.1	4	0.05	6.9E-02	included
FPGS	87671	ES	7	6	8	0.00	6.9E-02	included
CBR4	71143	ES	2.1:2.2	1	3	-0.02	6.9E-02	excluded
NIPA2	29682	ES	3	2	4	-0.03	6.9E-02	excluded
EIF6	59079	ES	2.4	2.2	4	0.01	6.9E-02	included
FHL2	54827	ES	6	5.1	7	0.00	6.9E-02	excluded
EIF2A	67285	ES	3:04	2	5.1	-0.02	6.9E-02	excluded
EDNRA	70793	ES	2	1	3	-0.01	6.9E-02	excluded
XPO6	35701	ES	2	1	3	-0.01	6.9E-02	excluded
GGT1	61442	ES	6.2:7.1:7.2	4	7.3	0.03	6.9E-02	included
LRCH3	68327	ES	2:03	1	4	0.00	6.9E-02	excluded
TIMM17B	89019	ES	4.1:4.2:4.3	3	5	0.01	7.0E-02	included
HMOX2	93852	ES	7:8:9:10	6	11	0.03	7.0E-02	included
RPS14	74093	ES	1.2:3	1.1	4	0.03	7.0E-02	included
GABARAPL1	20401	ES	2.10:2.11:2.12:2.14	2.6	3	0.00	7.0E-02	excluded
COPS4	69765	ES	10:11	9	12	0.02	7.0E-02	included
PGM2	69008	ES	5	4	6.1	-0.01	7.0E-02	excluded
NPRL3	32817	ES	2	1.4	4	-0.01	7.0E-02	excluded
PTPN3	87167	ES	13	12	14	-0.02	7.0E-02	excluded
MFF	57816	ES	3:04	1	5	0.03	7.0E-02	included
WDR6	64798	ES	3.1:3.2:4.2:4.3	1	4.4	-0.01	7.0E-02	excluded
STX17	87070	ES	4	3	5	-0.01	7.0E-02	excluded
PCBP2	22055	ES	12:13	11.2	14.1	0.00	7.0E-02	excluded
NUP50	62643	ES	4.1:4.2	3.2	5	-0.02	7.0E-02	excluded
IL17RC	63259	ES	18	17	19	0.02	7.0E-02	included
MLH1	63950	ES	1.2:2.1	1.1	2.2	-0.04	7.0E-02	excluded
PASK	58326	ES	4:05	3	6	0.00	7.0E-02	included
MAP4K1	49676	ES	11:12	10	13	0.01	7.0E-02	included
TRIQK	84502	ES	4	2.1	6.1	0.01	7.0E-02	included
SLC2A8	87630	ES	5	4	6	-0.01	7.0E-02	excluded
FIG4	77215	ES	3:04	2	5	0.00	7.1E-02	excluded
CPSF4	80636	ES	6	5	7	0.00	7.1E-02	included
RNH1	13670	ES	5.3:6.2:7:8:9.1	5.2	9.2	-0.01	7.1E-02	excluded
SEC23IP	13278	ES	2	1	3	0.00	7.1E-02	included
PUS7L	21243	ES	2	1.1	3	-0.02	7.1E-02	excluded
ECHDC2	3031	ES	3:5.1:6.1	2.1	6.2	0.01	7.1E-02	included
FLRT3	58719	ES	2	1	3	-0.04	7.1E-02	excluded
PXN	24751	ES	13	9	14.2	-0.02	7.1E-02	excluded
RPE	57245	ES	7	6	10.1	0.00	7.1E-02	excluded
PKNOX1	60767	ES	4	3	5	0.01	7.1E-02	included
ING4	19908	ES	7	6.2	8	0.00	7.1E-02	included
TRAF3IP1	58224	ES	08:09.1	7	11	0.00	7.1E-02	excluded
BCL2L13	60990	ES	6	4	7	0.01	7.1E-02	included
DLG1	68287	ES	23	21	24	-0.02	7.1E-02	excluded
HIRA	61053	ES	2	1	4	-0.01	7.1E-02	excluded
LIPG	45489	ES	6	5	7.1	0.00	7.1E-02	excluded

TMEM68	83875	ES	02:03.1	1.1	4	0.00	7.1E-02	excluded
INTS9	83228	ES	4	3	5	-0.01	7.1E-02	excluded
TLK1	55927	ES	8	7	9	0.00	7.1E-02	excluded
MYO1G	79580	ES	2	1	3	0.02	7.1E-02	included
COX20	10474	ES	2:03	1	4	0.01	7.1E-02	included
IVD	29978	ES	2	1	3	0.00	7.1E-02	included
MTA1	29643	ES	19	18	20	0.00	7.1E-02	included
ATP2A3	38515	ES	21.1:21.2	20	23.1	0.00	7.2E-02	excluded
RPS6KB2	17203	ES	6.1:6.2	5.1	7	-0.02	7.2E-02	excluded
TESC	24677	ES	3	2	4	0.00	7.2E-02	excluded
RHOA	64856	ES	5	4	6	0.00	7.2E-02	excluded
MEGF8	50157	ES	41	40	42	-0.02	7.2E-02	excluded
PEPD	48940	ES	3:04	2	5	0.00	7.2E-02	excluded
MEF2BNB	48605	ES	2:03	1	4.1	0.01	7.2E-02	included
MKRN1	152273	ES	6	1	7	0.00	7.2E-02	included
UPP1	97463	ES	6.1:7	4	9	0.00	7.2E-02	excluded
NF1	40147	ES	51	50	52	0.00	7.2E-02	included
WDR52	66203	ES	3:04:05	2	6	0.02	7.2E-02	included
DUSP10	9882	ES	2	1	4	0.01	7.2E-02	included
CKMT1B	30285	ES	3.2	2.2	4	0.00	7.2E-02	included
KDM6A	88878	ES	16	15	17	0.03	7.2E-02	included
LRMP	20767	ES	5	4.2	6	-0.02	7.2E-02	excluded
FNTA	83757	ES	3:04	1	5	0.00	7.2E-02	excluded
SH3KBP1	88646	ES	7:08	6	9	0.00	7.2E-02	included
NDRG1	85238	ES	2.3:3:4:5.1:5.2:6:7.1:7 .2:8:9:10:11:12:13	2.2	14	-0.04	7.2E-02	excluded
CLDND1	65777	ES	4.1:4.2	1	5.2	0.00	7.2E-02	excluded
HDAC7	21374	ES	9:10.1:10.2:11	8	12	0.00	7.2E-02	included
FBXO22	31901	ES	3:04	2	5	0.00	7.2E-02	included
DHX30	64543	ES	6	5	10	0.03	7.3E-02	included
TATDN1	138629	ES	2:4.1:5:6	1.1	7	-0.03	7.3E-02	excluded
PSMD6	65522	ES	2.2:3	2.1	4	0.01	7.3E-02	included
ORC5	81262	ES	3.1:3.2	2	4	0.00	7.3E-02	excluded
LYRM1	34410	ES	7	5	8.1	0.03	7.3E-02	included
MRPL55	10162	ES	1.2:2.2:2.5	1.1	2.9	0.01	7.3E-02	included
GNS	22865	ES	3.2	1	4	0.00	7.3E-02	included
NSUN4	2793	ES	4	3	5	0.01	7.3E-02	included
DYNC1LI2	36838	ES	3	2.3	4	0.00	7.3E-02	excluded
COG4	37404	ES	3:04	2	5.1	-0.01	7.3E-02	excluded
MRPL55	10126	ES	2.5	1.2	2.9	0.00	7.3E-02	included
ZNF211	52317	ES	2.2:3.1:3.2	2.1	4	0.02	7.3E-02	included
UBA2	48974	ES	3	1	4	-0.01	7.3E-02	excluded
CAPN10	58290	ES	2:3.1:3.2:4:5:6:7:8:9	1	11	-0.08	7.3E-02	excluded
PLEKHG2	49826	ES	15:16	14	17	0.00	7.3E-02	included
USP10	37859	ES	7:08	6	9	0.00	7.3E-02	included
KCNJ15	60586	ES	4	3	5	0.00	7.3E-02	excluded
ABCD4	28386	ES	4.1	3	5	-0.01	7.3E-02	excluded
PTPMT1	15769	ES	2	1.3	3	0.01	7.3E-02	included
PSME1	534014	ES	2	1.1	3	0.00	7.3E-02	included
TPK1	82139	ES	15	13	16	-0.02	7.4E-02	excluded
IL17RC	63265	ES	4:5.1:5.2:5.3	3.3	6	0.00	7.4E-02	included
COL4A3BP	72518	ES	12	11	13	0.01	7.4E-02	included
CD44	15117	ES	10:11	5	12.1	-0.04	7.4E-02	excluded
TMX2	15920	ES	3.2:3.3	2	4	-0.03	7.4E-02	excluded
ATPAF1	2821	ES	9:10	8	11	-0.01	7.4E-02	excluded
CCDC92	25124	ES	2.2	1	2.4	0.01	7.4E-02	included
VTA1	77964	ES	2	1	3	0.00	7.4E-02	included
EYA2	59707	ES	4.2:5.1	4.1	5.2	0.00	7.4E-02	excluded
SUMO2	43381	ES	4	3	5	0.00	7.4E-02	included
MLTK	56000	ES	4	3	5	-0.01	7.4E-02	excluded
TPRG1L	319	ES	3	2	4	0.00	7.4E-02	excluded
FAM211A	39437	ES	3	2	4	-0.01	7.4E-02	excluded

CDKAL1	75497	ES	13	12	14	0.02	7.4E-02	included
JMJD8	32952	ES	8	7	9	0.00	7.4E-02	included
SLC11A2	21734	ES	8:9.1:9.2	7.2	10	-0.02	7.4E-02	excluded
TFIP11	61514	ES	4	2.2	5	0.01	7.4E-02	included
ASPM	9286	ES	18	17	19	-0.03	7.4E-02	excluded
SCP2	3043	ES	15	14.2	16.1	0.00	7.4E-02	included
PLEKHB2	55378	ES	7:8.1:9.1	6	9.2	0.00	7.4E-02	excluded
RHOC	4245	ES	1.2:1.3:2.2:2.3	1.1	3	0.00	7.4E-02	included
CLDN16	68104	ES	2:03:04	1	5	0.00	7.4E-02	excluded
GYS1	50843	ES	6	5	7	0.00	7.4E-02	excluded
SLC27A1	48321	ES	3:4.1:4.4	2.2	5	-0.01	7.4E-02	excluded
ATP6V0A1	41046	ES	20	18	21.2	0.00	7.5E-02	included
ZMYM2	25414	ES	17	16	18	0.00	7.5E-02	excluded
USP47	14403	ES	3	1	4	0.01	7.5E-02	included
TMUB2	41819	ES	2.5:3:4.2	1	4.3	0.04	7.5E-02	included
PPP5C	50558	ES	5	4	6	0.00	7.5E-02	included
RRM2B	84772	ES	2:3.1:3.2	1	4	-0.01	7.5E-02	excluded
LMBR1L	21525	ES	4.1:4.2	3	5	0.04	7.5E-02	included
TMBIM4	22907	ES	3	1	4.1	0.00	7.5E-02	excluded
RAD51D	40267	ES	5:07:08	4	9	-0.03	7.5E-02	excluded
PHTF1	4281	ES	6	5	7	-0.01	7.5E-02	excluded
CTBP2	13418	ES	4	2	6	0.04	7.5E-02	included
FAM211B	61457	ES	4	3	5	-0.01	7.5E-02	excluded
TARBP2	22079	ES	3.1:3.2:3.3:4.1:4.2	1.2	5.1	0.01	7.5E-02	included
C6orf141	76450	ES	2:03	1.1	4	-0.01	7.5E-02	excluded
EDEM2	59068	ES	10:11:12	9	13	-0.02	7.5E-02	excluded
SRSF11	3382	ES	5:6.1:6.2	4.2	6.3	-0.02	7.5E-02	excluded
ZMYND11	10592	ES	5	4.2	6	0.00	7.5E-02	excluded
RBCK1	58455	ES	2	1	4	0.00	7.5E-02	excluded
FAM131A	67934	ES	04:06.2	2	6.4	0.01	7.5E-02	included
EXOSC10	647	ES	4	3	5	-0.04	7.5E-02	excluded
PARP6	31526	ES	18.1:18.2	17	19	0.03	7.5E-02	included
INPP5K	38315	ES	8.1:8.2:9	7	10	-0.01	7.5E-02	excluded
CCDC64	24717	ES	6	5	8	-0.02	7.5E-02	excluded
ZNF106	30164	ES	4.2:5	4.1	6	0.00	7.5E-02	excluded
CCDC43	41881	ES	4.2	3	5	0.01	7.6E-02	included
RDX	18650	ES	3.1:3.2	2	4	-0.02	7.6E-02	excluded
RHOT1	40176	ES	21	20	22	-0.02	7.6E-02	excluded
HAUS8	48227	ES	4	3.2	5.1	0.02	7.6E-02	included
TPM3	7797	ES	3.2:5.1	3.1	5.2	0.00	7.6E-02	included
C5orf45	74970	ES	2.3:4:5.1:5.2	2.1	6	0.05	7.6E-02	included
SNX1	139187	ES	4.2:5:6:7:8:9:10.2:11:	3	16.1	0.00	7.6E-02	excluded
B4GALT4	66290	ES	6.2:7:8.1:9.1:9.3	6.1	9.4	-0.02	7.6E-02	excluded
FAM32A	48144	ES	3	1.3	4	0.00	7.6E-02	excluded
SHISA5	64694	ES	2:3.1:3.2	1.3	4.1	-0.01	7.6E-02	excluded
FAM204A	13246	ES	8	7	9	-0.01	7.6E-02	excluded
ZNF83	51498	ES	8	7	9.6	0.03	7.6E-02	included
RAP1B	22943	ES	02:03.1	1.1	3.2	-0.02	7.6E-02	excluded
PNN	27366	ES	4.3	4.1	5	0.00	7.6E-02	included
PRCP	18028	ES	3	2	4	0.00	7.6E-02	included
GPRASP1	89705	ES	2:03:04	1	5	0.02	7.6E-02	included
ZNF195	13980	ES	5.2:11	5.1	13	-0.02	7.6E-02	excluded
POR	80140	ES	11:12:13:14.1:14.2:15.	10	15.2	0.00	7.6E-02	excluded
MRPL55	10124	ES	2.4:2.5	1.2	2.9	0.00	7.6E-02	excluded
LTBR	19853	ES	9.1	7	10	0.00	7.6E-02	excluded
WAC	11106	ES	4	3	5.2	0.00	7.6E-02	included
FUS	36247	ES	7	6	8	-0.01	7.6E-02	excluded
SLC27A1	48318	ES	4.1:4.2:4.3:4.4	2.2	5	-0.01	7.6E-02	excluded
SNCA	69930	ES	7	6.1	8	0.00	7.6E-02	excluded
TG	319504	ES	25:26:27:29:31:32:33:<br>34:35:36:37:38:39:40:	24	46	-0.01	7.6E-02	excluded
PRMT2	60953	ES	9:10:11	8	12	0.00	7.6E-02	included

PIK3R3	2779	ES	10	9	11	0.01	7.6E-02	included
MICU1	12098	ES	6	5	8	0.00	7.6E-02	included
FLOT2	40015	ES	3:04	2	5	0.00	7.6E-02	excluded
PDLIM7	74780	ES	10.2	8	11	0.00	7.6E-02	included
TMEM194A	22512	ES	6	5	7	-0.01	7.7E-02	excluded
LUC7L3	102328	ES	12.1	11	12.5	-0.01	7.7E-02	excluded
YWHAZ	84732	ES	3.2	2	7.1	0.01	7.7E-02	included
ANKS3	33828	ES	4	3	5.1	0.03	7.7E-02	included
PLEKHB2	55372	ES	9.1:9.2	8.1	10	0.00	7.7E-02	excluded
NASP	2719	ES	06:07.2	3	8	0.00	7.7E-02	excluded
TBCB	49352	ES	3:4:5.1:5.2	2.3	6	-0.03	7.7E-02	excluded
PRR5	62617	ES	9	8	10	0.00	7.7E-02	excluded
METTL23	43639	ES	2	1.3	3	0.02	7.7E-02	included
ADAMTS13	88054	ES	17:18:19	16	20	-0.03	7.7E-02	excluded
COG6	25716	ES	2	1	4.1	0.00	7.7E-02	excluded
BCL7B	79956	ES	3:04	1	6	0.00	7.7E-02	included
FBXO36	57858	ES	2	1	3	-0.04	7.7E-02	excluded
ACTR1A	12960	ES	2	1	3	0.00	7.7E-02	included
RANBP3	46964	ES	9	8	10.2	-0.01	7.7E-02	excluded
TRABD2A	54190	ES	3	2	4	-0.01	7.7E-02	excluded
DCAF6	8883	ES	12	10	13.1	0.03	7.7E-02	included
MAZ	35952	ES	3.1:3.2	2.2	4.2	0.00	7.7E-02	included
ABCB8	82315	ES	2.1:2.2	1	5	0.00	7.7E-02	included
BCAP29	81363	ES	5	4	6	0.00	7.7E-02	excluded
PARD3B	57101	ES	11	10	12	-0.02	7.7E-02	excluded
GNPDA2	69153	ES	2	1	3	0.01	7.7E-02	included
PRR14L	61888	ES	7	6	8	-0.01	7.7E-02	excluded
CD74	74081	ES	6:7.1:9:10.1	4	10.2	0.00	7.8E-02	excluded
MTHFSD	37920	ES	6	5	7	-0.03	7.8E-02	excluded
GIT2	24378	ES	17.2:18.1:18.2:19	17.1	20	0.01	7.8E-02	included
SFT2D2	8905	ES	4	3	5	0.00	7.8E-02	excluded
OCIAD1	69236	ES	4	3	6	0.00	7.8E-02	included
GPX3	74123	ES	2:03	1	4	0.03	7.8E-02	included
CSNK1D	44311	ES	02:03.2	1	4	-0.04	7.8E-02	excluded
DCTD	96834	ES	3	2.2	5	-0.03	7.8E-02	excluded
ARMCX6	89660	ES	2	1	4	0.00	7.8E-02	excluded
FAM76B	18380	ES	09:10.1	8	10.2	-0.03	7.8E-02	excluded
FN1	57368	ES	40.1:40.2:40.4:41	39	42	0.00	7.8E-02	included
ABI2	57017	ES	15	14	16	-0.01	7.8E-02	excluded
ABCB8	82293	ES	12	9	13.1	0.00	7.8E-02	included
TNIP2	68588	ES	4	3	5	0.00	7.8E-02	included
ATP2B4	9452	ES	7	6	8	0.01	7.8E-02	included
AAMDC	17978	ES	5:06	4	7	-0.01	7.8E-02	excluded
PTP4A2	1518	ES	7	6	8	0.00	7.8E-02	included
MLLT4	78450	ES	32.1:32.2:33.1	31	33.2	0.00	7.8E-02	excluded
RSPO1	507795	ES	5:6:8.1:8.2	4.2	8.3	0.00	7.8E-02	included
TNFRSF25	461	ES	3	2	4	-0.01	7.9E-02	excluded
RBPMS	83292	ES	14	13	15	0.00	7.9E-02	excluded
SLC7A6	37209	ES	7:08	6	9	0.00	7.9E-02	included
IL32	33409	ES	1.2:1.3:1.4:1.5:1.9	1.1	2.1	0.01	7.9E-02	included
KANSL3	54547	ES	6	5.2	7	0.02	7.9E-02	included
ECI2	75220	ES	7:08	6	9	0.00	7.9E-02	included
LONP2	36335	ES	13	12	14	0.00	7.9E-02	excluded
PDLIM5	69978	ES	13	8.2	14	0.02	7.9E-02	included
ZNF268	25357	ES	9:10.1:10.2	7	11	0.00	7.9E-02	excluded
MRPL46	32374	ES	2	1	3	0.00	7.9E-02	included
APTX	86085	ES	6.2:7.1:7.2:7.3	5.2	7.4	0.03	7.9E-02	included
ARMC10	81163	ES	3:04	1	5.1	0.00	7.9E-02	excluded
MZT2B	55331	ES	3	2	4	0.00	7.9E-02	excluded
GLS2	22440	ES	9.2	8.3	10	0.01	7.9E-02	included
UCK1	87957	ES	6	5	7	0.00	7.9E-02	excluded
C8orf59	84339	ES	2.2	1	3.2	0.00	7.9E-02	included

GATM	30422	ES	3	2	4	0.02	7.9E-02	included
BIN1	55197	ES	14:15:16	12	17	0.00	7.9E-02	included
CALM1	28822	ES	6	5	7.1	0.00	7.9E-02	included
OCIAD1	69245	ES	2.2:2.3:3:4	2.1	6	0.00	7.9E-02	included
SLC25A17	62392	ES	2.1:2.2:4	1	5.1	0.00	7.9E-02	included
PPA2	70208	ES	4:07:08	2	9	-0.02	7.9E-02	excluded
TMX2	15897	ES	3.3:4:5.1:5.2:5.3	2	6	0.04	7.9E-02	included
RAF1	63447	ES	3	2	4	0.00	7.9E-02	excluded
ZC3H14	28721	ES	12	10	13	0.01	7.9E-02	included
BCL7B	79955	ES	2:03:04	1	6	0.00	7.9E-02	included
GALNT10	74218	ES	5	4	6	0.00	7.9E-02	included
CISD2	70154	ES	3	1	4	-0.02	7.9E-02	excluded
MRPL21	17346	ES	2.1	1	3	0.00	7.9E-02	included
XRRA1	17789	ES	11:12:13	10	14	-0.05	7.9E-02	excluded
RPL21	25523	ES	3	2	4.1	0.00	8.0E-02	included
MEF2B	48601	ES	2:03	1	4	0.01	8.0E-02	included
BRD9	71467	ES	11	10	12	-0.01	8.0E-02	excluded
MARK2	16544	ES	16.1:16.2	15.2	17	-0.02	8.0E-02	excluded
FRG1B	58883	ES	4:05	3	6	0.04	8.0E-02	included
SAE1	50626	ES	4	3	5	0.00	8.0E-02	included
DBI	55119	ES	3.2	1.1	5	0.00	8.0E-02	included
FGGY	3212	ES	15	14	16	-0.01	8.0E-02	excluded
RRNAD1	8315	ES	2.1	1	2.3	0.00	8.0E-02	included
RRP12	12697	ES	05:02.2	3	7	0.00	8.0E-02	included
NDRG1	85235	ES	9	8	10	0.00	8.0E-02	excluded
C1orf159	15	ES	9	8	10.1	0.00	8.0E-02	excluded
SLC30A6	53155	ES	5	4	6	0.02	8.0E-02	included
ATP5S	27470	ES	2	1	4	0.01	8.0E-02	included
USP15	22830	ES	8	6	9	-0.02	8.0E-02	excluded
AKAP13	32355	ES	13	11	14	-0.02	8.0E-02	excluded
MED1	40647	ES	6	5	7	-0.01	8.1E-02	excluded
FAM114A1	69035	ES	2:03	1	4	0.03	8.1E-02	included
ASGR1	38841	ES	5	4	6	0.02	8.1E-02	included
MIEF1	62310	ES	4	3	5	0.02	8.1E-02	included
ADCK1	28631	ES	6	5	7	-0.01	8.1E-02	excluded
UGGT2	26132	ES	9	7	10	0.00	8.1E-02	included
U2AF1L4	49270	ES	8	7	9.1	-0.02	8.1E-02	excluded
SELT	67292	ES	3.1	2.1	4	0.00	8.1E-02	excluded
ASL	79867	ES	8	7	9	0.01	8.1E-02	included
DMTF1	80304	ES	7	6	8	0.01	8.1E-02	included
ZMYND8	59717	ES	16	15	17	-0.01	8.1E-02	excluded
AAMDC	17979	ES	6	4	7	0.00	8.1E-02	excluded
PLA1A	66341	ES	3	1	4.1	-0.01	8.1E-02	excluded
BCL7B	79958	ES	3	1	6	0.00	8.1E-02	included
GOSR2	42045	ES	5.1	4.1	6.1	-0.01	8.1E-02	excluded
BCAS4	59784	ES	5:06	3	7	-0.03	8.1E-02	excluded
KIAA1737	28578	ES	3	2	4.2	0.00	8.1E-02	included
POT1	81643	ES	12	11	13	-0.01	8.1E-02	excluded
UBA3	65571	ES	2	1	3	-0.02	8.1E-02	excluded
ANKS3	33819	ES	4:5.1:5.2:6	3	8	0.01	8.1E-02	included
MMAB	24325	ES	2:04:05	1	6	0.01	8.1E-02	included
DLST	28441	ES	3	2	4	0.00	8.2E-02	excluded
ABCB8	82313	ES	2.1:2.2:2.3	1	5	-0.01	8.2E-02	excluded
NUDT7	37669	ES	2	1	5	0.01	8.2E-02	included
C11orf54	18323	ES	8	7	9	-0.01	8.2E-02	excluded
CABYR	44862	ES	7.2:8.1	7.1	8.2	-0.02	8.2E-02	excluded
CPNE1	59192	ES	2.1:2.2	1.2	5	-0.02	8.2E-02	excluded
BDP1	72440	ES	34	33	35	0.01	8.2E-02	included
ZNF415	51685	ES	6.1:8.2	4	9	-0.04	8.2E-02	excluded
PHB	42297	ES	5.2:6:7.1	5.1	7.2	0.00	8.2E-02	excluded
RWDD4	71274	ES	3.1:3.2	2	4	0.00	8.2E-02	included
SFTA3	121942	ES	2:4.2:4.3:5	1.3	6	0.03	8.2E-02	included

RBKS	53051	ES	3	1	4	0.00	8.2E-02	excluded
MLST8	33225	ES	6	5.2	7	0.00	8.2E-02	included
MRPL55	10085	ES	2.4:2.5:2.6	2.2	2.8	0.00	8.2E-02	included
DEGS2	29248	ES	2	1	3	0.00	8.2E-02	excluded
HMG20A	31991	ES	2	1	3	-0.01	8.2E-02	excluded
TRIT1	1923	ES	4	3.1	6.1	0.01	8.2E-02	included
DMKN	49162	ES	10	8	11	0.04	8.2E-02	included
MTRF1	25729	ES	12	11	13	-0.01	8.2E-02	excluded
TCTN1	24463	ES	15:16.1	14	16.2	-0.01	8.2E-02	excluded
YPEL5	53104	ES	3.1:3.2:4	1	5	-0.01	8.2E-02	excluded
TOLLIP	13831	ES	4	3.2	5	0.00	8.3E-02	included
ZNF195	13986	ES	3	1	4	0.03	8.3E-02	included
CC2D2B	12661	ES	20:21	19	22	0.01	8.3E-02	included
SLC44A2	47565	ES	4	3	5	0.00	8.3E-02	excluded
RDH11	28093	ES	2.1:2.2	1	3	0.00	8.3E-02	excluded
ANKMY1	58261	ES	6:07	5	8	0.04	8.3E-02	included
FAM221A	78986	ES	7	5	8	0.04	8.3E-02	included
COG2	10244	ES	5	4	6	0.00	8.3E-02	excluded
FDFT1	82653	ES	3.2:5.2:6.3	2.2	6.4	0.05	8.3E-02	included
CAPN10	58274	ES	8:9:10.1:10.2	7	11	0.01	8.3E-02	included
SMG7	9180	ES	7	6	8	-0.02	8.3E-02	excluded
UBR4	886	ES	54	53	55	-0.02	8.3E-02	excluded
R3HDM2	22572	ES	17	16	18	0.00	8.3E-02	included
SLC39A1	7766	ES	5	4.3	6	0.00	8.3E-02	excluded
TCF3	46542	ES	04:05.1	3	6.1	-0.01	8.3E-02	excluded
ST3GAL3	2202	ES	16.2:19.1:19.2:20	15.1	21	0.00	8.4E-02	excluded
DGKA	22305	ES	8	7	10	0.00	8.4E-02	included
LANCL1	57269	ES	3:04	2	5	0.00	8.4E-02	excluded
TMEM55A	84424	ES	2	1	3	-0.02	8.4E-02	excluded
SRSF11	3393	ES	6.1:6.2:6.3	4.2	6.5	-0.02	8.4E-02	excluded
MUC1	8015	ES	2.3:3.1:3.2:3.3	2.2	3.4	0.00	8.4E-02	included
ZNF615	51394	ES	4	1	5	0.03	8.4E-02	included
SFSWAP	25214	ES	15	14	16.1	0.01	8.4E-02	included
WDR45	89079	ES	6:7.2:8:9.1	5	9.2	-0.01	8.4E-02	excluded
YME1L1	11062	ES	3.1	2	3.3	-0.01	8.4E-02	excluded
NFIA	3229	ES	6	5	7	0.00	8.4E-02	included
TYMS	44458	ES	2:03	1	4	0.00	8.4E-02	included
MAPK13	75954	ES	5	4	6	0.00	8.4E-02	included
CAMKK1	38496	ES	9	8	10	0.02	8.4E-02	included
CHPT1	23996	ES	5	4	6	0.00	8.4E-02	included
AP5M1	27653	ES	2	1	3	0.01	8.4E-02	included
FANCI	32417	ES	34	33	35	0.01	8.4E-02	included
CAPN7	63560	ES	2	1	3	0.01	8.4E-02	included
AP2S1	50606	ES	3.2	1.1	4.2	0.00	8.4E-02	excluded
DBNL	79393	ES	4.1	3	5.2	0.00	8.5E-02	included
FBLN2	63511	ES	11	10	12	0.03	8.5E-02	included
COPS4	69766	ES	10	9	12	0.00	8.5E-02	included
GAK	68417	ES	03:05.2	2	6	0.01	8.5E-02	included
SNRPD2	50511	ES	03:05.1	1.3	5.2	-0.02	8.5E-02	excluded
CHTOP	91129	ES	4.2:4.3:4.4	3.2	5	-0.04	8.5E-02	excluded
C12orf29	23601	ES	6.1	5	7	0.00	8.5E-02	excluded
DTNB	52869	ES	7	6	8	0.00	8.5E-02	included
TRAP1	33631	ES	2:03	1	4	0.00	8.5E-02	excluded
SIK3	18879	ES	4	3	5	-0.01	8.5E-02	excluded
SPATA20	42426	ES	7	6	8	0.00	8.5E-02	included
ACTR3B	82417	ES	10:11	9	12	0.00	8.5E-02	excluded
ATP6V0A1	41048	ES	05:06.1	4	6.2	-0.02	8.5E-02	excluded
NARF	44406	ES	5	3.1	6	0.00	8.5E-02	excluded
DSN1	59315	ES	2	1.1	3.1	0.02	8.5E-02	included
FAM49B	85148	ES	7.1:7.2	6	9	0.00	8.5E-02	excluded
AGO3	1742	ES	6	5	9	0.00	8.6E-02	excluded
TRIM35	83148	ES	2	1	3	0.00	8.6E-02	excluded

PDPR	37330	ES	20	19	21	-0.02	8.6E-02	excluded
DCTN4	74115	ES	4	3	5	0.01	8.6E-02	included
UPF3A	101239	ES	6	5	7	0.00	8.6E-02	included
ETFA	31931	ES	4	3	5	0.00	8.6E-02	included
COPZ2	42155	ES	5:06:07	4	8	-0.01	8.6E-02	excluded
MT1G	36489	ES	2.1:2.2	1	2.4	0.02	8.6E-02	included
PARP11	19765	ES	2	1	3	-0.03	8.6E-02	excluded
COPS3	39474	ES	3.2	1	4	0.00	8.6E-02	included
WDR78	3331	ES	15	14	16	0.03	8.7E-02	included
ABCC1	34215	ES	17:18	16	19	-0.01	8.7E-02	excluded
DCAF6	91279	ES	13:01.1	10	15	0.03	8.7E-02	included
RAP1GAP	991	ES	22.1:22.2	21	22.4	0.00	8.7E-02	excluded
MFF	57803	ES	8:09	7	10	0.02	8.7E-02	included
NASP	2752	ES	5	3	6	-0.01	8.7E-02	excluded
MSTO1	8093	ES	4.2:5	4.1	6	-0.01	8.7E-02	excluded
LRRFIP2	63966	ES	7:8:9:10:11:12:13:14:	5	18	0.00	8.7E-02	included
CETN3	72763	ES	5	4.2	6	0.01	8.7E-02	included
CABIN1	61389	ES	37	36	38	0.00	8.7E-02	included
KCTD9	83109	ES	3	2.1	4	0.00	8.7E-02	included
MAPKBP1	30106	ES	12	11	13	0.01	8.7E-02	included
PHB2	20044	ES	5.2:5.3:6.1	5.1	6.2	0.04	8.7E-02	included
TESC	24676	ES	5	4	6.1	0.00	8.7E-02	excluded
DNALI1	1806	ES	3	2	4	0.01	8.7E-02	included
GULP1	56502	ES	08:09.1	6	10	0.01	8.7E-02	included
PBRM1	65239	ES	28	27	29	0.02	8.7E-02	included
PFKP	10622	ES	6	5	7	-0.01	8.7E-02	excluded
EIF4A1	38991	ES	5.1	4.2	6	0.00	8.7E-02	excluded
SERGEF	14561	ES	2	1.1	3	-0.02	8.7E-02	excluded
SENP6	76795	ES	21	20	22	0.00	8.7E-02	included
MORF4L1	32129	ES	8	7	9	0.00	8.7E-02	included
U2AF1L4	49272	ES	4	3.2	5	0.05	8.7E-02	included
ECHDC2	3027	ES	4	2.1	5.1	0.04	8.7E-02	included
PTGES3	22481	ES	03:04.1	2	4.2	0.00	8.7E-02	excluded
GABPB1	30578	ES	2	1	3	0.03	8.8E-02	included
RHOA	64857	ES	3:04:05	2	6	-0.02	8.8E-02	excluded
NBPF11	7336	ES	9:10:11:12:13	8.3	14	-0.01	8.8E-02	excluded
SNRPN	93600	ES	09:10.2	7	10.3	-0.01	8.8E-02	excluded
NEK8	39970	ES	9.2:10:11:12:13.1	9.1	13.2	0.00	8.8E-02	included
RSPO1	507794	ES	5:6:7:8.1:8.2	4.2	8.3	0.00	8.8E-02	included
BCL7B	79957	ES	2:03	1	6	0.01	8.8E-02	included
CAMTA1	508	ES	2	1	4	0.01	8.8E-02	included
OGFOD2	25011	ES	07:08.2	6.2	8.3	0.03	8.8E-02	included
GZF1	58840	ES	2:03	1	4	0.01	8.8E-02	included
SLC25A17	62386	ES	3.1	2.2	4	-0.01	8.8E-02	excluded
BECN1	41137	ES	11	10	12	0.00	8.8E-02	excluded
ZNF414	47242	ES	2	1	3	0.00	8.8E-02	excluded
HDAC10	62802	ES	7:8:9:10	6.2	11	0.00	8.8E-02	excluded
ACSS2	59042	ES	9	8	10	0.01	8.8E-02	included
MRPL55	10150	ES	1.2:2.2:2.4:2.5:2.6	1.1	2.9	0.01	8.8E-02	included
IFNGR2	60412	ES	2.1:2.2:3	1	4	0.00	8.8E-02	excluded
CTNND1	15975	ES	6:07	2.1	8	-0.02	8.8E-02	excluded
PCCB	66916	ES	5	3	6	0.00	8.8E-02	excluded
KLHL22	61159	ES	3	2	4	-0.01	8.8E-02	excluded
SEC31A	69731	ES	26.1:26.2	25.1	28	0.02	8.8E-02	included
DTNA	45108	ES	19:20:21	17	22	0.00	8.8E-02	included
TRAPPC3	1752	ES	4	3	5	0.00	8.8E-02	excluded
MEMO1	53145	ES	5	4	7	0.00	8.8E-02	excluded
SPINT2	49599	ES	2	1.2	3	0.00	8.8E-02	excluded
ARL16	44156	ES	3	2.2	5	0.04	8.8E-02	included
CMSS1	65807	ES	3	1	4	0.00	8.8E-02	excluded
SSR2	8157	ES	4.1:5:6.1:6.2	2	7	-0.04	8.9E-02	excluded
CCDC53	24018	ES	10	9	12	0.00	8.9E-02	excluded

HAUS1	45380	ES	7	6	8	0.00	8.9E-02	included
GOLGA4	63982	ES	24	23	25	0.02	8.9E-02	included
TTC8	28740	ES	13	11	14	-0.01	8.9E-02	excluded
CLUAP1	33591	ES	3	2	4	0.00	8.9E-02	included
TBC1D13	87781	ES	8:09	7	10	0.00	8.9E-02	included
PACRGL	68892	ES	6:7:8:9:12	5	13.1	-0.01	8.9E-02	excluded
SLC12A2	73194	ES	21	20	22	0.01	8.9E-02	included
SFTA3	121939	ES	2:3:4.1:4.2:4.3:5	1.3	6	-0.02	8.9E-02	excluded
TDG	24082	ES	7	6	8	-0.01	8.9E-02	excluded
TMEM230	58643	ES	3	1.1	4	0.01	8.9E-02	included
TP53BP1	30260	ES	21	20	22	0.00	8.9E-02	included
CIR1	56020	ES	8	7	9	0.00	8.9E-02	included
ANXA7	12145	ES	6	5	7	0.00	8.9E-02	excluded
SDHC	8660	ES	6	5	7	0.00	8.9E-02	excluded
NAGK	53920	ES	2:03:04	1	5	0.00	8.9E-02	included
EDA2R	89345	ES	4:05	3.2	6.1	-0.02	8.9E-02	excluded
ZNF195	13974	ES	6:9:11:12	5.1	13	-0.01	8.9E-02	excluded
ZNF846	47405	ES	2.2:3.1	2.1	3.2	-0.02	9.0E-02	excluded
PSMD1	57924	ES	4	3	5	0.00	9.0E-02	excluded
SEMA4A	8191	ES	6	5	7	0.00	9.0E-02	included
MAST2	2775	ES	10	9	11	0.00	9.0E-02	included
CROCC	101747	ES	18:19:20	17	21	0.02	9.0E-02	included
TEK	86044	ES	7	6	8	-0.02	9.0E-02	excluded
AFMID	43806	ES	10:11.1	6	12	-0.01	9.0E-02	excluded
GSN	87432	ES	12	10	16	0.00	9.0E-02	excluded
ELN	80055	ES	5	4	6	-0.01	9.0E-02	excluded
TMEM44	68164	ES	7	6.1	8	-0.02	9.0E-02	excluded
SFXN4	13257	ES	4	3	5	0.02	9.0E-02	included
C16orf13	32920	ES	3:04	1	5	-0.03	9.0E-02	excluded
DEF8	38188	ES	2.2:4	2.1	5	-0.02	9.0E-02	excluded
CERS6	55828	ES	10	9	11	0.00	9.0E-02	included
CAST	72857	ES	10:11.1	9	11.2	0.00	9.0E-02	excluded
CD44	15197	ES	3.2:4:5:7:8:9.1:9.2:10:11:12.1:13:14:15:16.1	3.1	16.2	-0.01	9.0E-02	excluded
KIAA0922	70873	ES	17:18	16	19	0.01	9.0E-02	included
GMPR2	26922	ES	4:05	3.2	6.1	-0.01	9.0E-02	excluded
TAF5	12996	ES	8	7	9	0.01	9.0E-02	included
IFI16	8395	ES	9	8	10	0.02	9.0E-02	included
LRP8	3066	ES	6	5	7	-0.04	9.0E-02	excluded
ELP2	45233	ES	4.1:4.2:5:7:8.1:9:10:11:12:13:14:15:16:17	3	18	0.00	9.0E-02	included
RBM6	64938	ES	6	5	7	-0.02	9.0E-02	excluded
TMEM143	50747	ES	4	2	5	-0.02	9.0E-02	excluded
TMEM39B	1552	ES	4	3	5	0.01	9.0E-02	included
NME6	64630	ES	2.1:2.2	1.1	3.2	0.01	9.0E-02	included
ATP5F1	4167	ES	2:03	1	4	0.00	9.0E-02	included
ZMYM2	25415	ES	7	6	8.1	0.00	9.0E-02	included
TRIT1	1972	ES	03:01.1	1	6.1	0.01	9.0E-02	included
ZNF506	48692	ES	3	2	6	-0.02	9.0E-02	excluded
FN1	57366	ES	40.1:40.2:40.3:40.4:41	39	42	0.00	9.1E-02	included
CBWD3	86515	ES	2	1	3	-0.01	9.1E-02	excluded
PCMTD1	83811	ES	2	1	3.1	0.00	9.1E-02	included
USP19	64836	ES	6.1:6.2	5.2	7	-0.01	9.1E-02	excluded
HAUS1	45385	ES	3:4:5:6	2.1	7	-0.01	9.1E-02	excluded
YAF2	21159	ES	3.2:5.2:6	2	9.1	0.00	9.1E-02	excluded
ATF2	56090	ES	7	6	8	0.02	9.1E-02	included
EIF3H	84961	ES	4	3.2	6	0.00	9.1E-02	excluded
KDM5C	89195	ES	5	3	6	0.00	9.1E-02	included
PTBP1	46321	ES	3:4:5:6:7:8:10	2	11	0.00	9.1E-02	included
NOL3	36955	ES	2:03	1	4.1	-0.02	9.1E-02	excluded
PITRM1	10627	ES	16.1:17	15	18	0.01	9.1E-02	included
CNIH1	27589	ES	2	1	3	0.00	9.1E-02	excluded

ATP5SL	50073	ES	6.1	5	7	0.02	9.1E-02	included
MAP4K3	53329	ES	16:17	15	18	-0.01	9.1E-02	excluded
CIAPIN1	36549	ES	8	6	9	0.02	9.1E-02	included
NAPA	50659	ES	3:04	1	5	0.00	9.1E-02	excluded
LRRFIP2	63971	ES	6:7:8:10:14:15:16:17	5	18	0.00	9.1E-02	included
AP1M1	48147	ES	10	9	11	0.00	9.1E-02	included
CAND2	63457	ES	3:04	2	5.2	0.00	9.1E-02	excluded
HEATR5A	27114	ES	34	33	35	0.01	9.1E-02	included
DMPK	50523	ES	13:14.2	12	15	-0.03	9.1E-02	excluded
SLC37A3	81985	ES	12:13:14	11	15	-0.01	9.1E-02	excluded
QRICH1	64821	ES	3	1	4	-0.02	9.1E-02	excluded
SULT1C2	54875	ES	5.2:5.3:6.1	3	6.2	0.00	9.1E-02	excluded
PORCN	88985	ES	5:06:07	4	9	0.02	9.2E-02	included
VEGFA	76334	ES	8.1	6	9.1	0.00	9.2E-02	excluded
ALDH1A3	32742	ES	4:05:06	3	7	0.00	9.2E-02	excluded
KXD1	48464	ES	3.2	1.1	4.2	0.01	9.2E-02	included
ARNTL	14446	ES	3	2	4	0.03	9.2E-02	included
NCOA4	11548	ES	5	1	6	0.00	9.2E-02	excluded
INTS8	84573	ES	24	23	25	0.00	9.2E-02	excluded
PLEKHB2	55374	ES	8.2:9.1	8.1	9.2	0.03	9.2E-02	included
MGP	20561	ES	2	1	3	0.00	9.2E-02	excluded
SLC17A5	76763	ES	4	3	5	0.00	9.2E-02	included
C7orf63	80383	ES	8	7	9	-0.02	9.2E-02	excluded
FHL2	54832	ES	2.3:3.2:4	2.2	5.1	-0.04	9.2E-02	excluded
ACOT8	59629	ES	03:02.2	1	5	0.02	9.2E-02	included
GTPBP1	62254	ES	2	1	3	0.02	9.2E-02	included
MPPE1	44649	ES	9.2:10	8	11	0.00	9.2E-02	included
CYFIP2	74363	ES	8.1:10.1	6.2	10.2	0.00	9.2E-02	excluded
DRAM1	24011	ES	4	3	5	0.00	9.2E-02	included
ATP9B	46235	ES	12	11	13.1	0.02	9.2E-02	included
TPP1	14184	ES	2.4	2.2	3	0.00	9.2E-02	included
AMIGO2	21335	ES	2.1:2.2	1	3.1	-0.03	9.2E-02	excluded
SSBP2	72672	ES	16	15	17	0.00	9.2E-02	included
MAX	27946	ES	5.3:5.5:5.6:5.7	5.1	5.8	0.00	9.3E-02	excluded
RAB3IP	23351	ES	9.1:9.2:10	8.1	11	0.00	9.3E-02	included
WBSCR22	80002	ES	12	10.1	13	0.00	9.3E-02	included
IQCB1	96476	ES	12	11	13	0.01	9.3E-02	included
RNF8	76014	ES	4	3	5.2	0.00	9.3E-02	excluded
HMOX2	33749	ES	3	2.2	7	-0.01	9.3E-02	excluded
TSPAN31	22730	ES	2.2:3:5.2	2.1	6.1	0.02	9.3E-02	included
RHOT2	32943	ES	03:04.1	2.2	4.2	0.00	9.3E-02	included
ARCN1	19030	ES	3.1:3.2	1	4	0.00	9.3E-02	included
FUZ	51076	ES	2.4:3	2.3	4	-0.03	9.3E-02	excluded
CDK11A	221	ES	2.4	2.2	3	-0.02	9.3E-02	excluded
NUP54	69589	ES	6	5	7	0.00	9.3E-02	excluded
WDR70	71831	ES	11	10	12.1	0.00	9.3E-02	excluded
KBTBD3	18559	ES	3.2	1	5	0.01	9.3E-02	included
STX2	25202	ES	10	9	11	-0.03	9.3E-02	excluded
SKA2	42744	ES	1.2:2	1.1	4.1	0.04	9.3E-02	included
MAP1S	48356	ES	4	3.2	5	0.00	9.3E-02	included
CCNYL1	57201	ES	7	6	8	-0.01	9.3E-02	excluded
ARHGEF28	72495	ES	31	30	32	0.00	9.3E-02	excluded
TPM3	7792	ES	12	11	15	0.00	9.3E-02	included
TEX9	30760	ES	10	9	11.1	-0.02	9.3E-02	excluded
MTX1	8038	ES	4	3	5	0.00	9.3E-02	excluded
NPIPB3	93952	ES	8.5:8.6:8.7:8.8:9:10:1	8.4	12.1	0.02	9.3E-02	included
ADPGK	31587	ES	2:03	1.3	4	-0.01	9.3E-02	excluded
LRRC8A	87789	ES	3	1	4	-0.01	9.3E-02	excluded
EIF6	59075	ES	3	2.4	4	0.00	9.3E-02	included
GNB2L1	75087	ES	1.2:2.1:2.2:2.3:3:5:6:7	1.1	8.2	0.00	9.4E-02	excluded
ORC4	55525	ES	7	6	8	0.00	9.4E-02	included
CYP2R1	14484	ES	2.2:3	2.1	4.2	-0.05	9.4E-02	excluded

TRIT1	1924	ES	2:3.1:4:6.1:6.2	1	7.1	0.01	9.4E-02	included
RRBP1	131581	ES	3.3:3.4:4:5:7:8:9:10:1<br>1:12:13:14:15:16:17:1	3.2	19	-0.03	9.4E-02	excluded
MVK	24342	ES	4	3	5	-0.01	9.4E-02	excluded
RAB2A	83946	ES	6	5	7	0.00	9.4E-02	included
SETDB1	7522	ES	7	6	8	0.00	9.4E-02	included
ARIH2	64786	ES	5	4.2	6	-0.02	9.4E-02	excluded
P2RX4	24843	ES	3	1	4	-0.01	9.4E-02	excluded
IQCK	34335	ES	7.1	6.3	8.1	0.00	9.4E-02	included
CD44	15196	ES	3.2:4:5:6:7:8:9.1:9.2:1<br>0:11:12.1:13:14:15:16	3.1	16.2	-0.03	9.4E-02	excluded
DENND4A	31238	ES	20	19	21	-0.03	9.4E-02	excluded
MFSD10	68616	ES	9	8	10	0.00	9.4E-02	included
LARS	73907	ES	4	3	5	0.00	9.4E-02	excluded
ANAPC11	44209	ES	5:06	3.3	7.2	0.01	9.4E-02	included
ABLIM2	68752	ES	14:15:16	13	17	0.02	9.4E-02	included
C2CD5	20736	ES	11	10	12	0.00	9.4E-02	excluded
ZNF92	79846	ES	3	2	4	-0.01	9.4E-02	excluded
TOP3B	61271	ES	5:06:07	4	8	0.00	9.5E-02	excluded
CRYL1	25427	ES	2	1	3	0.00	9.5E-02	excluded
TUSC2	65015	ES	2.1:2.2	1	3.1	0.00	9.5E-02	included
ECI2	75225	ES	1.2:1.3:2.1	1.1	2.2	0.00	9.5E-02	excluded
MED30	84990	ES	3	2	4	0.00	9.5E-02	included
DRG1	61852	ES	3	2	4	0.00	9.5E-02	included
GORASP2	55920	ES	2.2	1	4.1	0.00	9.5E-02	excluded
IRF1	73285	ES	4	3	5	0.00	9.5E-02	included
DBI	55120	ES	2.2	1.1	5	0.00	9.5E-02	excluded
MR1	9137	ES	5	3.2	6	0.00	9.5E-02	excluded
SLC25A32	84815	ES	3:04	2	5	0.00	9.5E-02	excluded
CBWD5	86508	ES	2	1	3	-0.01	9.5E-02	excluded
ARHGAP4	90524	ES	18:19	17	20	0.00	9.5E-02	included
RBM47	69091	ES	8.2	7.2	9	-0.01	9.5E-02	excluded
SIRT6	46772	ES	7	6	8.1	0.00	9.5E-02	excluded
MRPL55	10086	ES	2.5:2.6	2.2	2.8	0.01	9.5E-02	included
RASA4B	81102	ES	17	16	18	0.02	9.5E-02	included
KIF27	86703	ES	4	3	5	0.04	9.5E-02	included
SYPL1	81328	ES	6	5	7	-0.02	9.5E-02	excluded
FBXO8	71181	ES	4	3	5	0.00	9.5E-02	excluded
ZNF461	49408	ES	6	5	7	-0.03	9.5E-02	excluded
CSE1L	59734	ES	9	8	10	0.00	9.5E-02	included
COL1A1	316104	ES	20:21:22:23:24:28:29:<br>30:31:32:33:34:35:36:	19	41	-0.03	9.5E-02	excluded
TNK2	68208	ES	18	17	19	-0.04	9.6E-02	excluded
ATXN10	62671	ES	2	1	3	0.00	9.6E-02	included
TMEM63A	10003	ES	10:11.1	9.2	11.2	-0.03	9.6E-02	excluded
DLD	81380	ES	8	7.2	9.1	0.00	9.6E-02	included
TAMM41	63406	ES	11.1	9.1	12	-0.01	9.6E-02	excluded
UBE2K	69075	ES	6	5	7	0.00	9.6E-02	excluded
RSRC1	67422	ES	11	10	12	0.00	9.6E-02	excluded
SETD5	63091	ES	11	10	12	0.02	9.6E-02	included
ZDHHC3	64392	ES	7	6	8	0.01	9.6E-02	included
CAST	72858	ES	10	9	11.2	0.01	9.6E-02	included
CCT7	53972	ES	3:04	1	5.1	0.00	9.6E-02	excluded
SUMF2	79797	ES	4:5.1:5.2	3	6	0.00	9.6E-02	included
NPRL3	32820	ES	1.4:2:4:5	1.3	6	0.00	9.6E-02	excluded
SCLT1	70590	ES	12:13:14:15:16:17:18:	11	21	0.00	9.6E-02	included
GTPBP8	66133	ES	4	1	5	0.01	9.6E-02	included
CFLAR	56796	ES	15	13	17	0.00	9.6E-02	excluded
SMAD9	25650	ES	4	3	5	-0.02	9.6E-02	excluded
ST3GAL3	2302	ES	5.1:5.2	4	6	0.00	9.7E-02	included
ACOX1	43540	ES	3	2	4	-0.02	9.7E-02	excluded
WDR26	9961	ES	7	6	8	0.00	9.7E-02	excluded

MIF4GD	43417	ES	5	4	6.1	0.00	9.7E-02	included
SH3BP2	68599	ES	8.1:8.2	7	9	-0.01	9.7E-02	excluded
DNMBP	12786	ES	11.2	10.1	12	-0.01	9.7E-02	excluded
NARS	45639	ES	6	5.2	7	0.00	9.7E-02	excluded
SMIM11	60481	ES	4	3	6	-0.02	9.7E-02	excluded
MRPL55	10154	ES	2.2:2.5:2.6	1.1	2.9	0.02	9.7E-02	included
BID	61003	ES	6	5	7	0.00	9.7E-02	excluded
TBC1D13	87782	ES	3:4:5:6	2	7	0.00	9.7E-02	included
GPATCH4	8298	ES	3	2.2	4	0.00	9.7E-02	included
OXR1	84855	ES	16	14	17	-0.02	9.7E-02	excluded
SH3GLB2	87814	ES	2:03:04	1	5	-0.04	9.7E-02	excluded
C19orf44	48174	ES	3	2	4	0.01	9.7E-02	included
CCT3	8240	ES	2	1	4	0.00	9.7E-02	excluded
TMUB2	41812	ES	2.2:2.3:2.4:2.5:3:4.2	2.1	4.3	0.03	9.7E-02	included
ECSIT	47718	ES	6.3:6.4	6.1	7	0.00	9.7E-02	included
RELA	16903	ES	4.1:4.2	3	5	0.00	9.7E-02	excluded
PPARG	63421	ES	10	9	11	-0.01	9.8E-02	excluded
SZRD1	805	ES	2:03	1	4.1	0.00	9.8E-02	excluded
RCAN3	1118	ES	3:4.1:4.2	2	5	0.00	9.8E-02	included
SNUPN	31883	ES	1.2:2	1.1	4	-0.04	9.8E-02	excluded
IRF9	26880	ES	2.1	1	3	-0.02	9.8E-02	excluded
MASTL	11063	ES	10	9.2	11	-0.01	9.8E-02	excluded
RFFL	40236	ES	7	6	8	-0.01	9.8E-02	excluded
SH3TC1	68758	ES	17	16	18	0.00	9.8E-02	excluded
MDH1	53737	ES	4:05:06	1	7	0.00	9.8E-02	included
EPG5	45358	ES	36	35	37	0.01	9.8E-02	included
DMKN	49197	ES	7:8:10:11	6.4	12	0.01	9.8E-02	included
MUC1	8011	ES	2.3:3.1:3.2:3.3:3.4:4.1	2.2	4.2	0.00	9.8E-02	included
CAPN10	58280	ES	8	7	9	0.03	9.8E-02	included
ITGB6	55717	ES	2	1.3	3.1	0.01	9.8E-02	included
BROX	9896	ES	13	12	14	0.00	9.8E-02	included
KIFAP3	8964	ES	5	4	6	0.00	9.8E-02	excluded
ARFGAP3	62553	ES	4	3	5	0.00	9.8E-02	included
PCP4	60649	ES	3	2	4	0.00	9.8E-02	excluded
ARSD	88419	ES	3	2	4	-0.01	9.8E-02	excluded
C1orf43	7800	ES	4	3	5	0.00	9.9E-02	included
PGM2	69010	ES	04:06.1	2	6.2	0.00	9.9E-02	excluded
IFI27L1	29069	ES	03:04.2	1	4.3	0.01	9.9E-02	included
ZNF135	52395	ES	2.2:2.3:2.4	1	3	0.02	9.9E-02	included
SMUG1	22136	ES	3	1.1	4.1	0.03	9.9E-02	included
LRRC61	82222	ES	2	1	3	0.00	9.9E-02	excluded
MLST8	33224	ES	7	5.2	8.1	0.00	9.9E-02	included
PBRM1	65237	ES	29	27	30	0.02	9.9E-02	included
SDSL	24644	ES	2	1	3	0.02	9.9E-02	included
TRIM16	39367	ES	2	1.3	3	-0.03	9.9E-02	excluded
SULT1A1	35823	ES	3:04	2	6.2	0.05	9.9E-02	included
PML	31662	ES	05:06.1	4	6.4	0.00	9.9E-02	included
PHF20L1	85196	ES	9	8.2	10.1	0.00	9.9E-02	excluded
SEMA6A	73059	ES	20.2	18	22	0.01	1.0E-01	included
RWDD4	71275	ES	3.2	2	4	0.03	1.0E-01	included
EMC4	29846	ES	3.1:3.2	2.2	6	0.00	1.0E-01	included
HMGB1	25571	ES	7.1	6	7.3	0.00	1.0E-01	excluded
HDAC11	63478	ES	6.1:6.2:7:8:9:10	5	11	0.00	1.0E-01	excluded
KLHDC8A	9536	ES	3:04:05	2.2	6	0.00	1.0E-01	included
PARP9	66443	ES	4.2:5:6:7.1	4.1	7.2	0.00	1.0E-01	included
RTFDC1	59881	ES	9	8	10	0.00	1.0E-01	included
ADAM15	7930	ES	4	3.2	5.1	0.00	1.0E-01	included
ST3GAL3	2303	ES	5.1	4	6	0.00	1.0E-01	included
MRPL38	43528	ES	6	5	7	0.00	1.0E-01	excluded
LRRFIP2	63972	ES	7:8:10:14:15:16:17	5	18	0.00	1.0E-01	included
VCAM1	3888	ES	5	4	6	0.01	1.0E-01	included
RABL5	81061	ES	3.1:3.2	1	4	-0.02	1.0E-01	excluded

NPEPPS	42084	ES	6.1:6.2	5	7.1	-0.01	1.0E-01	excluded
ABI1	11051	ES	4	3	7	0.00	1.0E-01	excluded
TMUB2	41804	ES	3	2.5	4.2	-0.03	1.0E-01	excluded
TYW5	56727	ES	6	5	7	-0.02	1.0E-01	excluded
GDI2	10664	ES	2	1	3	0.00	1.0E-01	excluded
DMD	88775	ES	75	74	76	0.03	1.0E-01	included
GPR56	36588	ES	3.2:5.2	3.1	7.2	0.02	1.0E-01	included
SCMH1	2053	ES	6:07:08	5	9	0.02	1.0E-01	included
SNX19	19511	ES	5.2:5.3	4	6	0.00	1.0E-01	included
ANKS3	33824	ES	4:5.1:5.2	3	6	-0.01	1.0E-01	excluded
ERCC6	11520	ES	9	8	10	-0.01	1.0E-01	excluded
PRMT5	26664	ES	1.2:2:3:4.1	1.1	4.2	0.00	1.0E-01	included
SEPT2	58374	ES	3:04:05	2	7	0.00	1.0E-01	excluded
PDE9A	60736	ES	2:03	1	5	0.01	1.0E-01	included
FUK	37393	ES	4.2	3	5	0.00	1.0E-01	excluded
BRAT1	78623	ES	4	3	5	0.01	1.0E-01	included
HKR1	49493	ES	9	8.2	12	-0.02	1.0E-01	excluded
NFYC	2023	ES	6	1	11	-0.02	1.0E-01	excluded
NPRL3	32803	ES	7	6	8	0.00	1.0E-01	included
DDX11	20980	ES	1.2:3.1	1.1	3.2	-0.01	1.0E-01	excluded
TTC13	10259	ES	6:07	5	8	0.02	1.0E-01	included
RHOD	17150	ES	2:03	1	4.2	0.00	1.0E-01	excluded
CD59	14910	ES	7	6	8.1	0.00	1.0E-01	excluded
DAB2	71863	ES	10	9	11	0.01	1.0E-01	included
SMG1	93909	ES	2	1	3	-0.03	1.0E-01	excluded
CHCHD3	81838	ES	5	4	6.1	0.00	1.0E-01	excluded
SENP6	76799	ES	3	2	4	0.02	1.0E-01	included
NEK3	25993	ES	12.1:12.2	11	13	-0.07	1.0E-01	excluded
IL32	33443	ES	1.3:1.4	1.1	1.9	0.01	1.0E-01	included
PPP6R2	62827	ES	3:04	2	5	-0.01	1.0E-01	excluded
PPIF	12305	ES	05:06.1	4.1	6.2	-0.01	1.0E-01	excluded
CD22	49116	ES	5:06	4.2	7	-0.01	1.0E-01	excluded
RPE	57244	ES	7:08	6	10.1	-0.01	1.0E-01	excluded
RMDN1	84377	ES	6	4	7	-0.02	1.0E-01	excluded
PKP4	95716	ES	25:26:00	24	27	-0.01	1.0E-01	excluded
NFIB	85890	ES	14	11	15	-0.02	1.0E-01	excluded
BIN1	55201	ES	11	10	12	0.01	1.0E-01	included
POLD1	51192	ES	22	21	23	0.00	1.0E-01	included
DECR1	84408	ES	4.2:5.1	1	5.2	-0.01	1.0E-01	excluded
ATF2	56064	ES	17	16	18.2	-0.01	1.0E-01	excluded
CLASRP	50394	ES	3.1:3.2:4:5.1:5.2	2.2	6	0.00	1.0E-01	included
HBS1L	77792	ES	5.1:5.2	4	7.1	0.00	1.0E-01	excluded
PCBP2	22056	ES	13	11.2	14.1	-0.01	1.0E-01	excluded
RPE	57239	ES	08:10.1	7	10.2	-0.02	1.0E-01	excluded
FBXO3	14933	ES	4	3	5	-0.01	1.0E-01	excluded
ACTR6	23908	ES	5:06	4	7	-0.02	1.0E-01	excluded
WDR90	32930	ES	38	37	39	0.00	1.0E-01	excluded
ASCC2	61683	ES	4	3	5	-0.02	1.0E-01	excluded
MOK	29381	ES	15.2:15.4	15.1	15.5	0.01	1.0E-01	included
UBE2F	58169	ES	7	5	9	0.00	1.0E-01	excluded
TMUB2	41790	ES	4.8	4.5	5	-0.03	1.0E-01	excluded
PEX10	269	ES	2	1	3	0.01	1.0E-01	included
RAD51	30021	ES	5	3	6	0.01	1.0E-01	included
KCTD7	79891	ES	7	5	10	-0.02	1.0E-01	excluded
CTSB	97869	ES	3.1:3.2	2	5.3	0.00	1.0E-01	excluded
CDK2	22320	ES	5	4	6	0.00	1.0E-01	excluded
GIPC1	47982	ES	2:03	1	4	0.00	1.0E-01	included
HDAC11	63495	ES	5:6.1:6.2:7:8:9:10	4	11	0.00	1.0E-01	excluded
IFT122	66728	ES	10	9	11	0.01	1.0E-01	included
QDPR	68858	ES	3	1	4	0.01	1.0E-01	included
SIRT3	13620	ES	3.1:3.2:4:5.3	2.1	6.1	0.01	1.0E-01	included
WARS	29291	ES	3	2.3	4	-0.04	1.0E-01	excluded

CTTN	92714	ES	10	9	13	0.01	1.0E-01	included
PSMA6	27228	ES	6	5	7	0.00	1.0E-01	excluded
YIPF2	47604	ES	4	3	5	-0.01	1.0E-01	excluded
PLEKHA5	20660	ES	19	15	21	0.00	1.0E-01	included
MYO5C	30651	ES	22	21	23	-0.01	1.0E-01	excluded
TARDBP	634	ES	4	2	5	0.00	1.0E-01	included
GSTZ1	28587	ES	7.1:7.2	6	8	0.00	1.0E-01	included
RUVBL2	50869	ES	1.2:1.4:1.5:1.6	1.1	2	0.08	1.0E-01	included
USP8	30594	ES	4:05	3	6	0.00	1.0E-01	excluded
ZSCAN25	80705	ES	6:07	5	8	0.00	1.0E-01	included
LRRC28	32648	ES	5:6:7.1:7.2:8:10:11	3	12	0.00	1.0E-01	included
ITSN2	52821	ES	17	16	18	0.00	1.0E-01	excluded
PPOX	8572	ES	4:5:6:7:8:9:10	3	11	0.00	1.0E-01	excluded
TFIP11	61513	ES	3:04	2.2	5	0.01	1.0E-01	included
MAP2K4	39305	ES	3	1	4	-0.01	1.0E-01	excluded
EIF4A1	38987	ES	9.5:9.6	9.3	10	0.00	1.0E-01	included
ZNF133	58761	ES	7	6.2	8.1	-0.01	1.0E-01	excluded
CECR5	60968	ES	8	7	9	0.00	1.0E-01	included
FNTB	27916	ES	3	2	5	0.00	1.0E-01	included
ANP32E	7432	ES	7	6	8.1	0.00	1.0E-01	excluded
PLEKHA5	20661	ES	16:17	15	21	0.00	1.0E-01	excluded
ATL2	53255	ES	3	1	5	-0.04	1.0E-01	excluded
ERC1	19656	ES	20	19	21	0.00	1.0E-01	excluded
ENTPD6	58864	ES	11	10	12	-0.01	1.0E-01	excluded
TPRA1	66608	ES	10	9	11	0.00	1.0E-01	included
PLXNB2	96239	ES	2.1:2.2	1	3	-0.01	1.0E-01	excluded
DLG1	68285	ES	24	23	25.1	-0.02	1.0E-01	excluded
GKAP1	86694	ES	7	6	8	0.02	1.1E-01	included
SZRD1	808	ES	03:04.1	1	4.2	-0.02	1.1E-01	excluded
GGA3	43410	ES	3	2	4	-0.01	1.1E-01	excluded
SLC10A7	70777	ES	4	2	5	-0.01	1.1E-01	excluded
TCTN1	24473	ES	6.2	5	7.1	0.00	1.1E-01	excluded
RBL2	36416	ES	15.2:16:17:18:19:20:2	15.1	22.2	0.00	1.1E-01	included
CASP2	82100	ES	3.2:4:6.1	3.1	6.2	0.01	1.1E-01	included
ZNF195	13982	ES	6:09	5.1	13	0.00	1.1E-01	excluded
SUMO1	56941	ES	4	1	5.1	0.00	1.1E-01	included
METTL23	43636	ES	3	1.3	4.1	-0.01	1.1E-01	excluded
CYB561D2	65051	ES	1.2:1.4	1.1	1.5	0.00	1.1E-01	included
TMEM143	50732	ES	5	4	6	0.01	1.1E-01	included
TIAL1	13268	ES	6	5.1	7	-0.02	1.1E-01	excluded
RUVBL2	50867	ES	1.4:1.6	1.2	2	0.00	1.1E-01	included
CTTN	92712	ES	10:12	9	13	-0.05	1.1E-01	excluded
COL1A1	409549	ES	20:21:22:23:24:25:26:<br>27:28:29:30:31:33:34:	19	41	-0.02	1.1E-01	excluded
EFTUD1	32215	ES	3:04	2	5	0.00	1.1E-01	excluded
DCUN1D2	26378	ES	5	4	6	0.04	1.1E-01	included
SMTN	61813	ES	21.1:21.2	20	22	0.01	1.1E-01	included
SHANK3	62904	ES	19	18.1	21	-0.01	1.1E-01	excluded
RAD51	30018	ES	10	9	11	-0.01	1.1E-01	excluded
GLS2	22457	ES	2:03	1	5	-0.02	1.1E-01	excluded
ATXN2	24516	ES	25	23	26	-0.01	1.1E-01	excluded
HKR1	49502	ES	6:12	5	13	-0.01	1.1E-01	excluded
DSTYK	9525	ES	12	11.2	13	-0.01	1.1E-01	excluded
SIRT3	13606	ES	2.2:3.1	2.1	3.2	0.00	1.1E-01	included
RPGR	88803	ES	15	14.3	16	-0.01	1.1E-01	excluded
LIMCH1	69121	ES	29:30.2	26	31	0.00	1.1E-01	included
HSF4	36951	ES	4	3	5	0.00	1.1E-01	included
HNRNPA1L2	26005	ES	4	3	5	0.04	1.1E-01	included
IKBKB	83603	ES	2:3:4.1:5:6.1:6.2:7:9:1<br>0:11:12.1:12.2:13:14:<br>15:16:17:18:19:20:21.	1.4	22.2	0.00	1.1E-01	included
SLC16A4	4109	ES	4	2	5	-0.03	1.1E-01	excluded

NPL	9165	ES	11:12	10	13	-0.01	1.1E-01	excluded
GPI	48958	ES	17:18	16	19	0.00	1.1E-01	included
KCTD10	24301	ES	2.2	1	3	0.00	1.1E-01	included
C8orf58	83033	ES	6.2	5	7	0.03	1.1E-01	included
MUC1	8018	ES	2.3:3.1	2.2	3.2	0.00	1.1E-01	included
CEP70	67005	ES	3.1:3.2:3.3	2	4	-0.02	1.1E-01	excluded
SLC27A1	48313	ES	6	5	7	0.01	1.1E-01	included
TMEM135	18210	ES	1.2:2:3:4	1.1	5	0.01	1.1E-01	included
IRAK1	90546	ES	10.1:10.2:10.3	9	11.1	0.00	1.1E-01	excluded
BOD1	74584	ES	3	2	4	0.00	1.1E-01	included
ATF2	56069	ES	9	8	10.1	0.00	1.1E-01	included
RANBP10	37111	ES	2	1	3	0.00	1.1E-01	included
C11orf49	15624	ES	6	3	7	0.00	1.1E-01	excluded
RNF135	40136	ES	3:04	2	5	0.03	1.1E-01	included
TPRKB	53980	ES	3	2.2	4	0.02	1.1E-01	included
TTC8	28788	ES	4	2	7	0.00	1.1E-01	excluded
WDR24	32954	ES	1.6:2:3	1.5	4	-0.02	1.1E-01	excluded
MCM3	76465	ES	2	1	3	0.00	1.1E-01	included
ETS1	19412	ES	5:6:7:8.1	4	9.1	0.00	1.1E-01	excluded
NDRG1	85242	ES	2.3:3:4:5.1:5.2:6:7.1:7.2:8:10:11:12:13	2.2	14	0.01	1.1E-01	included
INADL	3246	ES	31	30	32	0.01	1.1E-01	included
GARS	79141	ES	2	1	3	0.00	1.1E-01	excluded
WDR61	32084	ES	04:05.2	3	6	0.00	1.1E-01	included
MAPK3	36089	ES	4	3	5	0.00	1.1E-01	excluded
AASDH	69346	ES	13:14	11.1	15	-0.04	1.1E-01	excluded
CDK5RAP2	87385	ES	21.2	20	22	0.01	1.1E-01	included
ENDOV	44060	ES	11:12.1	10.2	13	-0.01	1.1E-01	excluded
MAPKAP1	87585	ES	2:03	1	4	0.00	1.1E-01	included
SPTLC3	58702	ES	8	7	9	0.00	1.1E-01	included
CTNND1	15998	ES	2.2:2.3:3:4.1:4.2:4.3	2.1	5	-0.01	1.1E-01	excluded
PKIA	84246	ES	2	1	4	0.02	1.1E-01	included
FAM110B	83922	ES	3	2	4	0.01	1.1E-01	included
C17orf72	43028	ES	4.1:4.2	3	5	0.01	1.1E-01	included
SIGMAR1	86191	ES	2	1.4	3	0.00	1.1E-01	excluded
TBCEL	19168	ES	7	6.2	8	0.01	1.1E-01	included
G6PC3	41764	ES	2.1:2.2	1.2	3	0.02	1.1E-01	included
SCNN1A	19844	ES	5.1:5.2	4.2	6	0.00	1.1E-01	included
EDC3	31733	ES	3:04	1	5	-0.01	1.1E-01	excluded
SCARB1	25159	ES	4:05	1	6	0.00	1.1E-01	included
PHLDB1	19041	ES	16	14	17	0.00	1.1E-01	excluded
ZNF35	64379	ES	3	2.2	4	-0.03	1.1E-01	excluded
NDUFC2	18007	ES	2.1:2.2:2.3	1	3.1	0.00	1.1E-01	included
STAU1	59742	ES	3	1	5	-0.01	1.1E-01	excluded
IFI27L1	29058	ES	7.1	6	8	0.00	1.1E-01	excluded
GTF2IRD2	80094	ES	14	13	15	0.01	1.1E-01	included
METTL5	55891	ES	4:05	3	6.1	0.00	1.1E-01	excluded
OSBPL9	2971	ES	14	13	15	0.00	1.1E-01	excluded
IQCK	34338	ES	6.1:6.3	5	7.1	0.00	1.1E-01	included
ZNF415	51674	ES	7.1:7.2:8.1:8.2	6.1	9	0.04	1.1E-01	included
BANP	37991	ES	7	6.2	9	-0.01	1.1E-01	excluded
HACL1	63592	ES	5:06:07	4	8	-0.01	1.1E-01	excluded
AFMID	43809	ES	10	6	12	0.00	1.1E-01	excluded
TBC1D31	85037	ES	14:15.1	13	15.2	0.02	1.1E-01	included
CARD8	50717	ES	6	5	7.2	-0.03	1.1E-01	excluded
SEPT2	58356	ES	11	10	12.2	0.00	1.1E-01	excluded
TSPAN31	22727	ES	4	3	5.1	0.00	1.1E-01	excluded
TP53I11	15489	ES	7	6.2	8.1	0.00	1.1E-01	excluded
AGTRAP	675	ES	3:4.1:4.2	1	5	0.03	1.1E-01	included
YIPF3	76289	ES	3.1:3.2	2.5	4.1	0.00	1.1E-01	excluded
LRRC37A3	43083	ES	3.2:4.2	2	5	0.03	1.1E-01	included
NFS1	59219	ES	7	5	8	-0.03	1.1E-01	excluded

GABARAPL1	20400	ES	2.7:2.8:2.10:2.11:2.12:	2.6	3	0.00	1.1E-01	excluded
STAM	10936	ES	4:05	2	6	0.01	1.1E-01	included
TFAM	11814	ES	5	4	6	0.01	1.1E-01	included
ECHDC1	77478	ES	3	1.1	4	0.01	1.1E-01	included
RHOT1	40189	ES	19.3	19.1	22	0.02	1.1E-01	included
EIF3L	62171	ES	3	2	4	0.00	1.1E-01	excluded
EMP1	20541	ES	2:03	1	4.1	0.00	1.1E-01	included
CMC2	37737	ES	4.4	2	5	-0.01	1.1E-01	excluded
FKBP7	56173	ES	2	1	4.1	-0.01	1.1E-01	excluded
LPAR2	48666	ES	3:04	2.2	5.2	0.04	1.1E-01	included
THAP1	83742	ES	2	1	3	0.01	1.1E-01	included
COG4	37405	ES	3	2	5.1	0.00	1.1E-01	excluded
VSTM2L	59363	ES	3	2	4	0.01	1.1E-01	included
UBAP2	86138	ES	5:6:7:8:9:10:11	4	12	0.00	1.1E-01	included
OXSR1	64042	ES	4	3	5	0.01	1.1E-01	included
NDRG3	59308	ES	3	2.2	4	0.00	1.1E-01	included
RBM39	95934	ES	5	4	6	-0.02	1.1E-01	excluded
TOM1L2	39513	ES	6	5	7	0.00	1.1E-01	included
TARBP2	22075	ES	4.2:5.1	4.1	5.2	0.04	1.1E-01	included
RBKS	53052	ES	2	1	4	-0.02	1.1E-01	excluded
GRN	522183	ES	4:05:10	3	11	0.02	1.1E-01	included
HNRNPR	1049	ES	2.2	1	3	0.02	1.1E-01	included
SHMT1	39616	ES	8:09	7	10	0.00	1.1E-01	included
GSN	87438	ES	10	9	16	0.00	1.1E-01	included
PMPCB	81179	ES	11:12.1	9	12.3	0.00	1.1E-01	included
APLP2	19482	ES	5.2:6.1	5.1	6.2	0.00	1.1E-01	included
ANKRD28	63629	ES	24:25:00	23	26	0.00	1.1E-01	included
SCLY	58203	ES	3.1:3.2:4	2	6	-0.01	1.1E-01	excluded
DUSP3	41719	ES	2	1	3	0.00	1.1E-01	excluded
MRRF	87469	ES	5	4	6	-0.01	1.1E-01	excluded
LTBP3	16865	ES	25	24	26	-0.03	1.1E-01	excluded
SULT1C2	54874	ES	5.1:5.2:5.3:6.1	3	6.2	-0.01	1.1E-01	excluded
ACSL3	57755	ES	3	2	4	-0.03	1.1E-01	excluded
STAG1	66932	ES	34	33	35	0.00	1.1E-01	excluded
ACSF2	42393	ES	4:5.1:5.2	1	6.1	0.00	1.1E-01	excluded
CSF2RA	88377	ES	5	4	7	0.01	1.1E-01	included
PTCD3	54398	ES	8	7	9	0.00	1.1E-01	included
CXADR	60229	ES	5:06	4	7.1	-0.01	1.1E-01	excluded
BCLAF1	77908	ES	7	6	8	-0.01	1.1E-01	excluded
ELAC2	39330	ES	5	4	6	0.00	1.1E-01	excluded
CCDC91	20918	ES	15	14	17	0.00	1.1E-01	included
SNX17	52997	ES	2.2	1	3	0.00	1.1E-01	excluded
APP	60288	ES	9	8	11	0.00	1.1E-01	included
KATNA1	78086	ES	10	9	11	0.00	1.1E-01	included
KIAA1731	18306	ES	17	16	18	0.02	1.1E-01	included
SEC31A	69738	ES	5.1:5.2	4	6	0.00	1.1E-01	included
SKP2	71799	ES	5	4	6	0.00	1.1E-01	included
RIT1	8132	ES	6	5	7	0.00	1.1E-01	excluded
RPS15A	34261	ES	3.2:3.3	2.2	5.1	0.00	1.1E-01	included
MLF1	67431	ES	6	5	7	0.00	1.1E-01	included
PRPF38B	3947	ES	2	1	3	0.02	1.1E-01	included
DHX32	13437	ES	4.2	3	5	0.00	1.1E-01	excluded
KCNJ16	43194	ES	3:06	1	7	-0.01	1.1E-01	excluded
PINX1	82611	ES	6	5	8	0.01	1.1E-01	included
TMEM14B	97116	ES	04:05.1	3	8	0.03	1.1E-01	included
ZFAND3	76020	ES	4	3	5	0.00	1.1E-01	included
CLDND1	65782	ES	3.2:3.3:3.4	1	4.1	0.00	1.1E-01	excluded
SMAP1	76648	ES	04:05.1	3	5.2	0.00	1.1E-01	excluded
FDFT1	82654	ES	5.2:6.3	2.2	6.4	0.00	1.1E-01	included
AGPAT5	82581	ES	2	1	3	0.01	1.1E-01	included
PORCN	88988	ES	3.2	2.2	4	0.00	1.1E-01	included
ZNF250	85681	ES	05:06.1	4	6.2	0.01	1.1E-01	included

ADAM15	7913	ES	20:22.1:22.2	19	23	0.10	1.1E-01	included
CMC1	63791	ES	2	1.1	3	0.01	1.1E-01	included
ULK3	31768	ES	2.2:2.3	1	3.2	0.02	1.1E-01	included
CNOT8	74259	ES	2:03	1.4	5	0.00	1.1E-01	excluded
DHX35	59384	ES	20	19	21	0.00	1.1E-01	included
ECHDC1	77475	ES	3:04	1.1	6.1	0.03	1.1E-01	included
SERPING1	15870	ES	05:06.2	4	7.1	0.00	1.1E-01	included
CAP1	1984	ES	1.2:2.1	1.1	2.2	0.02	1.1E-01	included
FXYD6	18937	ES	10.1:10.2	9	10.5	0.00	1.1E-01	included
GYG1	67207	ES	4.2:5	4.1	6	0.00	1.2E-01	excluded
NUP43	78100	ES	5:06	4	7.1	0.00	1.2E-01	included
SNRPN	29701	ES	11	10.4	12	0.00	1.2E-01	excluded
TCEB1	84212	ES	1.2:5	1.1	6	-0.02	1.2E-01	excluded
SLC43A2	38350	ES	13	12	14.2	0.00	1.2E-01	included
UQCC1	59121	ES	6.1:6.2:7.1:7.2	3	8	0.00	1.2E-01	excluded
ABCC3	42466	ES	5	2.2	6	-0.01	1.2E-01	excluded
CDK10	38119	ES	5	2.2	6	-0.02	1.2E-01	excluded
MAD1L1	78598	ES	2.2	1	3	0.01	1.2E-01	included
APOPT1	29459	ES	4	3	5	0.00	1.2E-01	excluded
C2CD5	20735	ES	13	12	14	0.00	1.2E-01	excluded
OCIAD1	69234	ES	5	4	6	-0.02	1.2E-01	excluded
SLC52A2	85576	ES	1.3:1.4:1.5:2.2	1.2	2.3	0.00	1.2E-01	excluded
YPEL5	53107	ES	3.1	1	5	0.00	1.2E-01	included
CEP57	18401	ES	3	2	5	0.00	1.2E-01	excluded
LGMN	29007	ES	13	12	14	0.00	1.2E-01	excluded
TPD52	84279	ES	7	6	8	0.00	1.2E-01	excluded
ACAD9	66671	ES	1.2:2.1:2.2	1.1	2.3	-0.01	1.2E-01	excluded
LRRFIP2	63978	ES	7:8:9:10:14	5	18	0.00	1.2E-01	included
TDP1	28810	ES	14	13	15	0.00	1.2E-01	excluded
SLAMF8	8418	ES	2	1	3	-0.01	1.2E-01	excluded
C3orf33	67352	ES	2	1	3	0.03	1.2E-01	included
RNPEP	9393	ES	4	3	5	0.00	1.2E-01	included
TDRD3	26024	ES	4.1:4.2	3	5	0.01	1.2E-01	included
LGR6	9410	ES	7:08	6	9	-0.01	1.2E-01	excluded
OGFOD2	25016	ES	4	3	5.2	0.01	1.2E-01	included
TMEM106A	41702	ES	2.2	1	3.1	-0.02	1.2E-01	excluded
SLC12A8	136740	ES	15	14	16.1	0.01	1.2E-01	included
EFCAB2	10482	ES	3	1.3	4	-0.02	1.2E-01	excluded
ACPP	66804	ES	5	4	6	0.01	1.2E-01	included
SNX7	3841	ES	5	4	6	0.00	1.2E-01	excluded
TP53BP2	9932	ES	2	1	4	0.00	1.2E-01	included
SLC24A1	31236	ES	6:7:8:9	5	10	-0.01	1.2E-01	excluded
RRP7A	62507	ES	2	1	3	0.00	1.2E-01	excluded
EXOSC1	12703	ES	3	2	4	0.00	1.2E-01	excluded
PPHLN1	21226	ES	8	7	9	-0.01	1.2E-01	excluded
ABHD10	66069	ES	2:03	1	4	0.00	1.2E-01	included
FAM192A	36534	ES	3.2	1.2	4.2	0.00	1.2E-01	excluded
TCEB1	84209	ES	4	1.2	6	0.00	1.2E-01	included
SMUG1	22134	ES	2.2:2.3:3	1.1	4.1	-0.03	1.2E-01	excluded
SLC8B1	24640	ES	8	7	9	0.01	1.2E-01	included
SS18	44913	ES	10	9	11	-0.01	1.2E-01	excluded
TES	81524	ES	4:05	3	6	0.00	1.2E-01	included
C9orf89	86902	ES	3.3	3.1	3.5	0.01	1.2E-01	included
SH3GLB2	87815	ES	2:03	1	5	0.00	1.2E-01	excluded
ACACA	40512	ES	48	47	49	0.01	1.2E-01	included
CYFIP2	74338	ES	11:12:13.1:13.2	10.3	14	0.00	1.2E-01	excluded
MRPL48	17717	ES	11.1	10	12	0.00	1.2E-01	included
HNRNPLL	53263	ES	4.2:5.2	4.1	6.1	0.00	1.2E-01	included
FNTA	83756	ES	3	1	4	0.00	1.2E-01	excluded
SCP2	3048	ES	3	2	4	0.00	1.2E-01	included
MAP2K7	47193	ES	4.2:5.1	4.1	5.2	-0.01	1.2E-01	excluded
DMTF1	80313	ES	1.2:2:3	1.1	4.2	-0.04	1.2E-01	excluded

RAD51C	42717	ES	3	2.1	4	0.00	1.2E-01	included
C12orf49	24664	ES	2	1	3	0.00	1.2E-01	excluded
SMUG1	22128	ES	2.3:3	2.2	4.1	0.03	1.2E-01	included
OS9	22714	ES	3	2.2	4	0.00	1.2E-01	excluded
MRPL55	10096	ES	2.5:2.6	2.2	2.9	0.01	1.2E-01	included
SULT1A1	35822	ES	6.2:7:8	2	9	-0.05	1.2E-01	excluded
PCGF2	40590	ES	3.2	2.2	5	0.02	1.2E-01	included
SPG21	31152	ES	5	4.2	6	0.00	1.2E-01	included
SRD5A1	71519	ES	3	1	4	-0.01	1.2E-01	excluded
KMT2E	81274	ES	1.2:2:3.1	1.1	3.2	0.01	1.2E-01	included
TAZ	90594	ES	6	4	8.1	-0.02	1.2E-01	excluded
ZCCHC8	24963	ES	6.1	5	6.3	0.00	1.2E-01	included
INPP5K	38316	ES	8.1:9	7	10	0.00	1.2E-01	excluded
TMEM205	47662	ES	2.3:2.6	2.2	3	0.01	1.2E-01	included
MPI	31783	ES	1.2:2.2:2.3	1.1	3	0.03	1.2E-01	included
ARL8B	63025	ES	2	1	3.1	0.00	1.2E-01	excluded
IKBIP	23866	ES	2	1	4	-0.01	1.2E-01	excluded
GK	88738	ES	7	5	8	0.00	1.2E-01	included
IFNAR1	60400	ES	10.4:11	10.3	12	0.00	1.2E-01	excluded
FNTA	83758	ES	4	1	5	-0.04	1.2E-01	excluded
VWA9	31222	ES	1.2:1.3:2.1	1.1	2.2	0.04	1.2E-01	included
DCTN6	83284	ES	3	2	4	-0.02	1.2E-01	excluded
ALKBH6	49325	ES	3	2	4	0.00	1.2E-01	excluded
TIMM23	11552	ES	04:05.1	3	5.2	0.00	1.2E-01	excluded
PIGS	39924	ES	1.3	1.1	1.5	0.00	1.2E-01	included
SRSF11	3386	ES	5	4.2	6.1	-0.02	1.2E-01	excluded
ANGPT2	82580	ES	2	1	3	0.01	1.2E-01	included
TFCP2	21801	ES	9:10:11	8	12	0.00	1.2E-01	excluded
CRYZL1	60463	ES	2	1	4	0.00	1.2E-01	included
WDHD1	27611	ES	13	12	14	0.01	1.2E-01	included
IDH2	32467	ES	3:04	1	5	0.00	1.2E-01	included
ENPP2	85005	ES	24	23	25	0.02	1.2E-01	included
VEGFA	76340	ES	6:7.1:8.1:8.2	5	9.1	0.02	1.2E-01	included
TP53I11	15494	ES	5.1:5.2:5.3	1.2	6.2	-0.02	1.2E-01	excluded
PPP6R3	17313	ES	16:17.1	15	17.2	0.00	1.2E-01	excluded
FIP1L1	69314	ES	9	8	10	-0.01	1.2E-01	excluded
ARRB2	38571	ES	3	2	4.2	0.02	1.2E-01	included
USP54	12165	ES	23	22	24	-0.01	1.2E-01	excluded
YTHDF1	60099	ES	5	3	6	0.01	1.2E-01	included
KLHL2	71039	ES	4	3	5	0.01	1.2E-01	included
EIF3D	62058	ES	4.2:5:6.1	4.1	6.2	0.00	1.2E-01	included
TMBIM4	22908	ES	2.2:2.3	1	4.1	0.00	1.2E-01	included
S100A13	7735	ES	5	4	8	0.00	1.2E-01	included
TMEM159	34429	ES	3	2.3	4	-0.01	1.2E-01	excluded
LRRFIP2	63965	ES	6:7:8:9:10:11:12:13:14:15:16:17	5	18	0.00	1.2E-01	included
SIRT5	75394	ES	9	8	10	0.00	1.2E-01	included
G6PC3	41769	ES	1.2:2.1	1.1	3	0.00	1.2E-01	included
NDRG1	85256	ES	2.1:2.2:2.3:3	1.1	4	0.00	1.2E-01	excluded
SLC15A2	66412	ES	4	3	5	0.01	1.2E-01	included
MTM1	90341	ES	4:05	3	6	0.01	1.2E-01	included
EEF1D	85463	ES	3	1	8.1	0.00	1.2E-01	excluded
CAMK2D	70415	ES	15:17	14.2	18	0.00	1.2E-01	excluded
PTGES3	22477	ES	6	5	7	0.00	1.2E-01	included
RNPS1	33253	ES	04:05.1	3	5.2	0.00	1.2E-01	included
TMEM25	19019	ES	3.1:3.2:3.3	2.3	4	0.02	1.2E-01	included
UQCRB	84613	ES	6.1:6.2	4	6.4	0.00	1.2E-01	included
ST3GAL3	2213	ES	14:15.1:16.2:19.1:19.2	13	21	0.00	1.2E-01	excluded
EVA1C	60346	ES	5	4	6.1	0.00	1.2E-01	excluded
P2RX4	24840	ES	7	5	8	0.00	1.2E-01	included
ENTHD2	44111	ES	8	7	9	0.01	1.2E-01	included
TRAPPC9	85282	ES	6	5	7	-0.02	1.2E-01	excluded

LRTOMT	17537	ES	4	3	5	-0.01	1.2E-01	excluded
STK40	1774	ES	2	1	3	-0.02	1.2E-01	excluded
TDRKH	7664	ES	6.3:7.1	6.2	7.2	0.00	1.2E-01	included
GPR137	16624	ES	7	6	8	0.00	1.2E-01	excluded
WFDC2	59584	ES	2	1	4.3	0.00	1.2E-01	included
CHEK2	61537	ES	4:5:7.1:7.2	3	9	-0.01	1.2E-01	excluded
NPLOC4	44135	ES	5	4	6	0.00	1.2E-01	included
KCNJ16	43193	ES	3:05:06	1	7	-0.01	1.2E-01	excluded
PUS7	81290	ES	15	14	16	0.02	1.2E-01	included
TMEM184A	78587	ES	6	5.2	7	0.01	1.2E-01	included
SFMBT1	65291	ES	22	21	23	0.00	1.2E-01	included
SARS	3999	ES	10	9	11	0.00	1.2E-01	excluded
PRMT5	26665	ES	03:04.1	1.1	4.2	0.00	1.2E-01	included
PPP6R3	17327	ES	2	1	3	-0.03	1.2E-01	excluded
NPIPB3	93953	ES	8.5:8.7:8.8:9:10:11	8.4	12.1	-0.02	1.2E-01	excluded
ZC3H14	28725	ES	13:14.1	10	15	0.01	1.2E-01	included
CDKL2	69546	ES	11:12	10	13	-0.03	1.2E-01	excluded
C19orf82	47383	ES	2	1	4.1	0.02	1.2E-01	included
EPB41L2	77596	ES	17:18	14	20.1	-0.03	1.2E-01	excluded
CCZ1	78687	ES	3	2	4	0.00	1.2E-01	included
NDUFS8	17280	ES	3:4.1:4.2:5:6	1	7	0.03	1.2E-01	included
MRPL55	10091	ES	2.4:2.5:2.6:2.8	2.2	2.9	0.00	1.2E-01	included
C5orf45	74967	ES	3.1:3.2:4:5.1:5.2	2.1	6	0.03	1.2E-01	included
PPA2	70241	ES	2:04	1	5	0.00	1.2E-01	excluded
PDIA3	30312	ES	3	1	4	0.00	1.2E-01	excluded
RORC	7674	ES	2	1	3	-0.02	1.2E-01	excluded
KIF9	64501	ES	3	2.3	4	0.04	1.2E-01	included
RPS20	83893	ES	1.4	1.1	2.1	0.00	1.2E-01	included
KIAA1407	66243	ES	2:03	1	4.1	0.02	1.2E-01	included
FAM169A	72509	ES	8	7	9	-0.01	1.2E-01	excluded
INO80E	36005	ES	7:8:9:10	6.3	11	0.03	1.2E-01	included
DHX33	38699	ES	6	5	7	-0.01	1.2E-01	excluded
PPARD	75909	ES	5:06	4.2	7	0.00	1.2E-01	excluded
SLC25A39	41840	ES	2.1:2.2:3:4:5.1:5.2	1	6	-0.01	1.2E-01	excluded
STOM	87439	ES	4:5:6:7	3	8	0.00	1.2E-01	excluded
ARIH1	31555	ES	3	2	4	0.00	1.2E-01	excluded
LRRC23	20006	ES	7.1	6	10	-0.01	1.2E-01	excluded
TRIM35	83147	ES	4	3	5	0.01	1.2E-01	included
EPB41L2	77576	ES	20.1:20.2:21:22	14	23	0.00	1.2E-01	included
SH2B1	35873	ES	3.4:3.5:3.6:4.1	3.3	4.2	0.01	1.2E-01	included
CMTM3	36812	ES	6	5	7.2	0.00	1.2E-01	included
AP4B1	4307	ES	2:03:04	1.3	5.1	0.00	1.2E-01	included
ZNF189	87092	ES	2	1	3.2	0.03	1.2E-01	included
CYB561A3	16180	ES	5.1:5.2	4.2	6.1	0.00	1.2E-01	excluded
NPIPB3	34463	ES	8.8:9:10:11:12.1	8.7	12.2	0.02	1.2E-01	included
ZC3HC1	81769	ES	2	1	3	0.00	1.2E-01	excluded
ZNF286A	39383	ES	1.4	1.1	2	0.02	1.2E-01	included
WDR20	29353	ES	4	1.2	7.1	-0.02	1.2E-01	excluded
CRYZ	3471	ES	2	1	3	-0.01	1.2E-01	excluded
RPS3A	96813	ES	1.4:2.2	1.3	4.1	0.00	1.2E-01	included
TSPAN14	12373	ES	7	6	8	0.00	1.2E-01	excluded
RTN4	53594	ES	5	2.5	8	0.01	1.2E-01	included
ANKMY2	78858	ES	2	1	3	-0.01	1.2E-01	excluded
TCF7L2	13141	ES	17	14	19	-0.02	1.2E-01	excluded
LITAF	34020	ES	9	8.2	10	0.00	1.2E-01	excluded
YWHAZ	84730	ES	3.2:7.1	2	7.2	0.00	1.2E-01	excluded
COL1A2	257687	ES	15:16:17:18:19:20:21:<br>22:23:24:25:26:27:28:<br>29:30:31:33:34:35:36:	14	38	-0.02	1.2E-01	excluded
TRIT1	1920	ES	8	7.2	9	0.00	1.2E-01	included
TXNDC11	34029	ES	6:07	5	8	0.01	1.2E-01	included
ARPC4	63182	ES	6	5	7	0.00	1.2E-01	included

ZNF808	51469	ES	4	3	5.1	0.02	1.2E-01	included
MAP3K7	77020	ES	11	10	12	-0.01	1.2E-01	excluded
NUDT2	86162	ES	2.1:2.2	1	3	-0.03	1.2E-01	excluded
CCZ1B	78768	ES	3	2	4	0.00	1.2E-01	included
FNTA	83755	ES	6	5	7.1	0.00	1.2E-01	included
ITIH4	65280	ES	15.1:15.2:15.3:15.4	14	16	0.04	1.2E-01	included
NEK11	66789	ES	2.2	1	3	-0.04	1.2E-01	excluded
LRRFIP2	63967	ES	6:7:8:10:11:12:13:14:	5	18	0.00	1.2E-01	included
ACOT13	75529	ES	2	1	3	0.00	1.2E-01	excluded
SFTA3	121948	ES	2:3:4.1:4.2	1.3	6	0.02	1.2E-01	included
INPP5K	38336	ES	3	1	4	0.00	1.2E-01	excluded
CS	22421	ES	3	1	4.1	0.00	1.2E-01	excluded
ICMT	385	ES	3:04	2	5	-0.03	1.2E-01	excluded
HAUS2	30197	ES	2	1	3	-0.02	1.2E-01	excluded
ZNF691	2135	ES	2.1:2.2:2.3	1	4	-0.03	1.2E-01	excluded
IP6K2	64776	ES	8.1:8.2	7	11.1	0.00	1.2E-01	excluded
LIPT1	54680	ES	3:04	1	6	-0.04	1.2E-01	excluded
PHF15	73403	ES	12:13.1	11	13.2	0.01	1.2E-01	included
PUS1	25226	ES	3:04	2.2	5	0.00	1.3E-01	excluded
MYH14	51166	ES	19	18	20	0.00	1.3E-01	excluded
CNDP2	45814	ES	5:06	4	7	0.00	1.3E-01	excluded
PBRM1	65244	ES	10	9	11	0.01	1.3E-01	included
HEATR1	10396	ES	23	22	24	0.00	1.3E-01	included
NDUFB5	67706	ES	4.1:4.2	1	5	-0.01	1.3E-01	excluded
ZFYVE20	63554	ES	2	1	3	-0.02	1.3E-01	excluded
ZNF397	45147	ES	5	3.1	6	-0.02	1.3E-01	excluded
SNURF	29714	ES	3.1:5:6:7:8	2	9	0.00	1.3E-01	included
ZMYND8	59712	ES	26	25	27	0.02	1.3E-01	included
NAA20	58808	ES	5	4	6	0.00	1.3E-01	excluded
MAP7	77915	ES	9	8	10	0.02	1.3E-01	included
SLC27A1	48322	ES	4.1:4.4	2.2	5	0.00	1.3E-01	excluded
LETMD1	100274	ES	8.1:8.2	7	9	-0.03	1.3E-01	excluded
NPIPA8	266561	ES	3	2	4	-0.01	1.3E-01	excluded
CLCN6	689	ES	13	12	14	-0.03	1.3E-01	excluded
RHOT1	40187	ES	20	19.1	22	0.02	1.3E-01	included
SLTM	30923	ES	2	1	4.2	0.00	1.3E-01	included
LPXN	16012	ES	4	3	5	0.01	1.3E-01	included
DFNA5	79021	ES	6	5	7	0.01	1.3E-01	included
MMP19	22271	ES	6.6	6.4	6.8	-0.01	1.3E-01	excluded
GGA1	62127	ES	6	5.1	8	0.00	1.3E-01	included
ZNF561	47368	ES	5.1:5.2	4	6.2	0.02	1.3E-01	included
EIF4E	70011	ES	8	7.2	9	0.00	1.3E-01	excluded
MEF2C	72753	ES	10.2:12.1	10.1	12.2	0.00	1.3E-01	included
LGALS3BP	234085	ES	2.3:2.4:2.5:3.1:3.2	2.2	4.1	-0.02	1.3E-01	excluded
FAM221A	78994	ES	2:3.1:3.2:3.3	1	4	-0.02	1.3E-01	excluded
ERBB2IP	72263	ES	22:24.1:24.2:24.3	21	25	0.04	1.3E-01	included
IKBKE	9584	ES	3	2	4	-0.01	1.3E-01	excluded
MFSD8	70551	ES	8	7	9	0.00	1.3E-01	included
MPV17	52967	ES	10	9	12	0.00	1.3E-01	included
GNB2L1	75080	ES	2.1:2.2:2.3:3:4.2:5:6:7	1.1	8.2	-0.02	1.3E-01	excluded
SF3A1	61728	ES	3.1:4.2	2	5	-0.01	1.3E-01	excluded
OCIAD1	69241	ES	2.2:2.3:3:4:5	2.1	6	-0.04	1.3E-01	excluded
TCP1	78323	ES	4.2:5:6	3.2	7	0.00	1.3E-01	included
RBMS3	63804	ES	8	7	9.1	-0.01	1.3E-01	excluded
HDAC7	21375	ES	9:10.1:10.2	8	12	0.00	1.3E-01	included
DCP1B	19691	ES	4	3	5	0.01	1.3E-01	included
HDAC7	21378	ES	5.2	2	6	-0.01	1.3E-01	excluded
GRB10	79725	ES	5.2:6	1	11	-0.03	1.3E-01	excluded
PELP1	38555	ES	10.2:11.1	10.1	11.2	-0.01	1.3E-01	excluded
USP25	60220	ES	19:20	18	21	-0.01	1.3E-01	excluded
PCCB	66915	ES	6	3	7.1	0.00	1.3E-01	included
DENND5A	14304	ES	14	13	15	0.00	1.3E-01	excluded

N6AMT2	25435	ES	4	3	5	-0.01	1.3E-01	excluded
ADH5	70025	ES	6	5.2	7	0.00	1.3E-01	excluded
ICOSLG	60812	ES	3	2	4	-0.01	1.3E-01	excluded
MPI	31778	ES	5.1	4	6.1	0.00	1.3E-01	included
MCM5	61973	ES	3	2	4	0.00	1.3E-01	excluded
NPRL2	65045	ES	3.2	2.1	3.4	-0.02	1.3E-01	excluded
LARP4	21702	ES	12.1	11	13	-0.01	1.3E-01	excluded
POLK	72536	ES	5	4	6	-0.01	1.3E-01	excluded
CDK8	25515	ES	5	4	6	0.01	1.3E-01	included
NDE1	34187	ES	6	5	7	-0.01	1.3E-01	excluded
C1GALT1C1	90008	ES	2	1	3	0.00	1.3E-01	excluded
HERPUD1	36505	ES	3.1:3.2	2.2	4	0.00	1.3E-01	included
ACP2	15690	ES	3	1	4.1	0.00	1.3E-01	excluded
ZC4H2	89318	ES	6	5	7	0.00	1.3E-01	excluded
CERS5	21685	ES	3	1	8	-0.02	1.3E-01	excluded
RMND5B	74842	ES	3	2	4.1	0.01	1.3E-01	included
MOSPD3	80975	ES	4	3.2	5.1	0.00	1.3E-01	included
HCLS1	66396	ES	6	5	7	0.00	1.3E-01	included
CAPZB	909	ES	3	1	4.2	0.00	1.3E-01	excluded
EEF1A1	319302	ES	1.4:2:3:4:5	1.3	6	-0.02	1.3E-01	excluded
SFTA3	27271	ES	4.1:4.2:5	2	6	0.02	1.3E-01	included
GNB2L1	75091	ES	2.1:3:5:6:7.2:8.1	1.1	8.2	-0.02	1.3E-01	excluded
SDR39U1	100871	ES	4.2:5	4.1	7	-0.03	1.3E-01	excluded
TMEM14B	75317	ES	3:04	2	5.1	0.00	1.3E-01	excluded
DIS3L2	57991	ES	15	14	16.1	0.00	1.3E-01	excluded
MRPL55	10139	ES	1.2:2.2:2.4:2.5:2.6:2.8	1.1	2.9	0.00	1.3E-01	included
TUBD1	42826	ES	2:03:05	1	6	-0.01	1.3E-01	excluded
POLB	83712	ES	4:05:06	3	7	0.00	1.3E-01	included
RNASET2	78435	ES	3	2.2	4	0.00	1.3E-01	excluded
ZFAND6	32175	ES	4	1	6	-0.02	1.3E-01	excluded
MFF	57812	ES	4	3	5	0.01	1.3E-01	included
MX1	60668	ES	7	6	8.2	0.00	1.3E-01	included
LTBP4	49937	ES	25	24	26	0.01	1.3E-01	included
ACADM	3510	ES	2.2	1	3	0.01	1.3E-01	included
ASB8	21441	ES	3.1:3.2	2.2	4.1	-0.01	1.3E-01	excluded
TTC3	60551	ES	3.2:4:5:6:7:8:9:10:11	1	13	-0.03	1.3E-01	excluded
C1orf159	14	ES	11.3	11.1	12	-0.04	1.3E-01	excluded
PPARD	75912	ES	4.1:4.2	2	5	0.00	1.3E-01	included
NOL6	86119	ES	16:17:18:19:20:21.2:2<br>2:23.1:23.2:24:25	15	26.1	0.00	1.3E-01	included
KIAA1217	11001	ES	24	23	25.1	0.00	1.3E-01	included
NELL2	21292	ES	4.2	3.3	5	0.00	1.3E-01	included
VWA9	31201	ES	3.1:3.2	2.3	4.1	0.00	1.3E-01	excluded
CLTCL1	61045	ES	30	29	31	-0.02	1.3E-01	excluded
PTN	81903	ES	5	4	6	0.00	1.3E-01	excluded
FIG4	77211	ES	22	21	23	0.00	1.3E-01	excluded
IFI16	8401	ES	4	3	5	0.00	1.3E-01	included
IP6K2	64774	ES	8.1	7	11.2	0.01	1.3E-01	included
NBPF10	7269	ES	5	4.2	6	-0.01	1.3E-01	excluded
ST3GAL3	2299	ES	7.1:7.2	6	9	0.01	1.3E-01	included
LSM14A	48951	ES	11	10	12	0.01	1.3E-01	included
ADCK5	85594	ES	5	4	6.1	-0.02	1.3E-01	excluded
TANGO2	61117	ES	9	8	10	0.00	1.3E-01	excluded
HYOU1	19085	ES	18.2	17	19	0.00	1.3E-01	included
NOMO2	34245	ES	2	1	3	0.00	1.3E-01	excluded
KIF16B	58724	ES	24	22	25	-0.01	1.3E-01	excluded
REPIN1	82247	ES	2.2:3.2	2.1	5.2	0.03	1.3E-01	included
ZCCHC6	86765	ES	15:16:17	14	18	0.00	1.3E-01	excluded
TMED1	47591	ES	4	3.2	5	0.00	1.3E-01	excluded
RNASE1	26472	ES	2.3	1	3.1	0.00	1.3E-01	included
TLN1	86281	ES	41	40	42	0.00	1.3E-01	excluded
SLC11A2	21737	ES	9.1	7.2	10	0.00	1.3E-01	excluded

TNFRSF18	36	ES	4.1:4.2:5.1	3	5.2	0.00	1.3E-01	excluded
IP6K2	64758	ES	11.7	11.2	11.9	-0.02	1.3E-01	excluded
AAED1	86980	ES	5	4	6	0.01	1.3E-01	included
ZDHHC16	12704	ES	9	8.2	10	0.01	1.3E-01	included
ZNF507	48870	ES	6	5	7	0.01	1.3E-01	included
ELN	80051	ES	11	10	12	0.00	1.3E-01	excluded
DPP4	55757	ES	12	11	13	0.00	1.3E-01	excluded
TRIM33	4324	ES	20	19	21	0.02	1.3E-01	included
OGFOD2	25010	ES	6.3:8.1:8.2	6.2	8.3	0.02	1.3E-01	included
COL1A1	190090	ES	39:40:00	38	41	0.00	1.3E-01	included
FAM195A	32925	ES	3:04	2	5	0.00	1.3E-01	excluded
YY1AP1	8105	ES	11	10.2	12.1	0.01	1.3E-01	included
PTPRK	77494	ES	24	23.1	25	0.00	1.3E-01	excluded
DBF4B	41888	ES	6	5	7	-0.03	1.3E-01	excluded
C18orf8	44832	ES	3:04	2	6	0.00	1.3E-01	excluded
WDR45	89081	ES	6:7.1:7.2:8	5	9.2	0.00	1.3E-01	excluded
RNF135	40138	ES	2:03:04	1	5	0.02	1.3E-01	included
PPP4R1L	59958	ES	5	4	6	0.02	1.3E-01	included
FYTTD1	68314	ES	8	7	9	0.00	1.3E-01	excluded
H2AFY	73445	ES	8	6.3	9	0.00	1.3E-01	excluded
GIT2	24373	ES	18.1:18.2	17.2	20	-0.02	1.3E-01	excluded
PRUNE	7548	ES	3:05	1	6	0.00	1.3E-01	included
SAFB2	46851	ES	3	2	4.1	0.00	1.3E-01	included
DCAF8	8444	ES	7.3:8.1	7.2	8.2	0.00	1.3E-01	included
CACNB3	21477	ES	7	6	8	0.00	1.3E-01	excluded
TXNDC9	54710	ES	3	2.1	5.1	0.00	1.3E-01	included
NOL8	86857	ES	6.1:6.2:6.3	5	7	0.01	1.3E-01	included
PLAT	83574	ES	6:7:8.1:8.2	5	9	0.00	1.3E-01	excluded
CD44	15071	ES	12.1:13	5	14	0.00	1.3E-01	excluded
TCTN1	24480	ES	4	1.1	5	0.01	1.3E-01	included
PTPRB	23387	ES	16	15	17	-0.01	1.3E-01	excluded
POLM	79451	ES	9.5	9.2	9.7	0.04	1.3E-01	included
OBSL1	57733	ES	11	10	12	-0.01	1.3E-01	excluded
CMC1	63790	ES	1.2:2	1.1	3	-0.02	1.3E-01	excluded
VIPAS39	28602	ES	1.3:1.4	1.1	2	0.02	1.3E-01	included
C8orf59	84337	ES	2.2:2.3	1	3.2	0.01	1.3E-01	included
SON	60439	ES	3	2	4	0.00	1.3E-01	included
CLTA	86334	ES	2:03	1	4	0.00	1.3E-01	included
CHTOP	91128	ES	4.1:4.2:4.3:4.4	3.2	5	-0.03	1.3E-01	excluded
SPRYD3	21933	ES	6	5	7	0.00	1.3E-01	included
SCLT1	70591	ES	8:9:10:11:12:13:14:15<br>:16:17:18:19:20	6	21	0.00	1.3E-01	included
TSSC4	13923	ES	2	1	3.2	-0.01	1.3E-01	excluded
DMKN	49199	ES	7:08:11	6.4	12	-0.03	1.3E-01	excluded
UBE2F	58163	ES	9	7	10	0.00	1.3E-01	excluded
MUTYH	2608	ES	6.3:6.4:6.5	5	7	0.01	1.3E-01	included
FANCG	86230	ES	4	3	5	0.01	1.3E-01	included
FBRS	36169	ES	7:8:9:10:11:12	6	13	0.00	1.3E-01	included
RABEPK	87553	ES	6	5	7.1	-0.01	1.3E-01	excluded
SMIM14	69072	ES	4	3	5	0.00	1.3E-01	included
FAM57A	38255	ES	2:03	1.3	4	0.00	1.3E-01	included
APLP2	19485	ES	3	1	4	0.00	1.3E-01	included
PLEKHA3	56176	ES	3	2	4	0.01	1.3E-01	included
NASP	2753	ES	4	3	6	0.00	1.4E-01	excluded
SERGEF	14557	ES	12:13	11	14	-0.01	1.4E-01	excluded
CNOT8	74249	ES	4	3	5	0.00	1.4E-01	excluded
SEPT5	61077	ES	10	9	11.1	0.00	1.4E-01	included
COMMD6	26059	ES	6	5.2	7	0.00	1.4E-01	excluded
ATP1B3	67083	ES	4:5:6:8	3	9	0.00	1.4E-01	included
TSPAN3	31976	ES	4	3	5	0.00	1.4E-01	excluded
TAMM41	63410	ES	5	4	7	0.02	1.4E-01	included
GRN	522184	ES	4:5:6.1:6.2:7:8:9	3	11	-0.02	1.4E-01	excluded

EFTUD1	32214	ES	4	3	5	0.01	1.4E-01	included
TCTN1	24464	ES	14:15	13	16.1	0.00	1.4E-01	included
IRF3	50989	ES	5.1:6.1:6.2	4	7	0.00	1.4E-01	excluded
CKAP5	15603	ES	36	35	37	0.00	1.4E-01	included
DKK3	14410	ES	6	5	7	0.00	1.4E-01	excluded
PPIP5K2	72916	ES	28	27	30	0.03	1.4E-01	included
CALD1	81864	ES	7.2:8.1	7.1	8.2	0.00	1.4E-01	excluded
SEC23A	27350	ES	18	17	19	0.01	1.4E-01	included
UBE2F	58187	ES	4	2	5	0.00	1.4E-01	excluded
PCBP4	65136	ES	4.1	2.2	5	0.04	1.4E-01	included
TRAPPC6A	50411	ES	2	1.1	3	-0.01	1.4E-01	excluded
MUTYH	2613	ES	6.3:6.4:6.5:7:8:9:10:1<br>1:12:13:14:15:16:17:1	5	19	0.02	1.4E-01	included
CTSB	97872	ES	2	1.1	4	0.02	1.4E-01	included
PTK2	85311	ES	24	23	25	0.00	1.4E-01	excluded
CLPB	17600	ES	4.1:4.2	2	5	-0.01	1.4E-01	excluded
CYB5A	45807	ES	4	3	6	0.00	1.4E-01	excluded
PSME4	53562	ES	14	13	15	0.00	1.4E-01	excluded
ZNF195	13966	ES	12	11	13	-0.03	1.4E-01	excluded
C5orf63	73185	ES	4	3	5.1	0.02	1.4E-01	included
SPARCL1	69871	ES	4	3	5	0.00	1.4E-01	excluded
MRPS35	20899	ES	2	1	3	0.00	1.4E-01	excluded
DDX19B	37361	ES	04:05.2	3	6	0.01	1.4E-01	included
LYRM1	34417	ES	5	3	7	-0.02	1.4E-01	excluded
TASP1	58706	ES	6:7:8:9:10:11	4	12	0.00	1.4E-01	included
PLEKHB2	55377	ES	7:8.1:8.2:9.1	6	9.2	0.00	1.4E-01	excluded
ARMC6	48568	ES	3	2.2	5	-0.02	1.4E-01	excluded
MTIF2	53607	ES	4	3	5	0.03	1.4E-01	included
TEAD4	19744	ES	6	5	7	-0.01	1.4E-01	excluded
DNAL1	28309	ES	2.2	1	3	-0.03	1.4E-01	excluded
DMKN	101870	ES	8:11	6.4	12	0.00	1.4E-01	excluded
ZNF805	52224	ES	3	2.2	4	-0.01	1.4E-01	excluded
XRRA1	17798	ES	7	6	8	0.02	1.4E-01	included
PHF15	73402	ES	12	11	13.1	0.02	1.4E-01	included
DONSON	60443	ES	9.1:9.2	8	10.1	0.00	1.4E-01	excluded
EXOC6B	53945	ES	2	1	3	0.00	1.4E-01	excluded
SOD2	78305	ES	7	6	8.1	0.00	1.4E-01	included
PPP1R12C	52021	ES	15.2:16:17.2	15.1	18	0.00	1.4E-01	excluded
CD200	66103	ES	3	1	4	-0.02	1.4E-01	excluded
LMF1	33035	ES	7	5.1	8.1	0.00	1.4E-01	included
MED15	61168	ES	13:14	11	15	0.00	1.4E-01	included
CD74	74078	ES	07:01.1	4	9	0.00	1.4E-01	included
AAMDC	17983	ES	1.2:1.3:2	1.1	4	0.00	1.4E-01	excluded
ODF3B	62862	ES	3.1:3.2	2.2	4.2	0.02	1.4E-01	included
CASP6	70334	ES	2:03	1	5	-0.02	1.4E-01	excluded
RPS9	51824	ES	4.1:4.2:4.3	3	4.5	-0.03	1.4E-01	excluded
VEZT	23758	ES	10	9	11	0.00	1.4E-01	excluded
CDCA7L	78916	ES	2	1	3	-0.01	1.4E-01	excluded
USP45	77072	ES	10.1	9	11	0.01	1.4E-01	included
BCS1L	57546	ES	1.2:1.8	1.1	2	0.02	1.4E-01	included
STIM2	68992	ES	9	8	10	0.02	1.4E-01	included
MARK4	50422	ES	2:3:4:5:6:7	1	8	0.00	1.4E-01	included
DDX5	43062	ES	4.2:5:6.1	4.1	6.2	0.00	1.4E-01	excluded
EIF4B	21919	ES	6	5.2	7	0.00	1.4E-01	excluded
SLMAP	65403	ES	13	12	14	0.03	1.4E-01	included
TMEM260	27644	ES	12	11	13	-0.01	1.4E-01	excluded
MMRN1	69938	ES	8	7	9	-0.01	1.4E-01	excluded
MLF1	67440	ES	4.1:4.2	1.1	5	-0.03	1.4E-01	excluded
RIC3	14228	ES	3.1:4:5	1	7.2	-0.01	1.4E-01	excluded
RWDD3	3828	ES	2	1	3.1	-0.02	1.4E-01	excluded
PHF6	90151	ES	3	2	4	-0.01	1.4E-01	excluded
STAU2	84159	ES	17	16	18.1	0.00	1.4E-01	excluded

ABCB8	82310	ES	5	1	6.1	0.01	1.4E-01	included
STAM	10937	ES	3	2	4	-0.01	1.4E-01	excluded
EXOC7	43563	ES	19	18	20	0.00	1.4E-01	included
HNRNPA1	301525	ES	6.3:7.2:9.1:9.2	6.2	10	0.00	1.4E-01	excluded
DHRS4L2	26800	ES	8.1	7	9	0.00	1.4E-01	included
GDI1	90610	ES	6	5	7.1	0.00	1.4E-01	included
DDX19A	37382	ES	2	1	3	-0.03	1.4E-01	excluded
TNFRSF10B	83060	ES	2	1	3	0.00	1.4E-01	included
STX8	39216	ES	3:04:05	2	6	0.00	1.4E-01	included
ZNF19	37457	ES	04:05.1	2	5.2	0.03	1.4E-01	included
PRR5L	15428	ES	9	8	10	0.01	1.4E-01	included
RHNO1	19726	ES	2.1:2.2	1	4	0.01	1.4E-01	included
TRIT1	1971	ES	02:03.1	1	4	0.01	1.4E-01	included
NEIL2	82634	ES	1.3:2.1	1.1	3	-0.03	1.4E-01	excluded
MBNL1	67320	ES	8:09:10	7	11	0.00	1.4E-01	excluded
POLM	79467	ES	3	2	4	0.00	1.4E-01	excluded
PKN2	3702	ES	2	1	3	0.00	1.4E-01	included
MAP3K4	78359	ES	4	3	5	-0.04	1.4E-01	excluded
TTLL3	63225	ES	4	3	5	0.02	1.4E-01	included
CTNND1	15942	ES	3:4.1:4.2:4.3	2.3	5	-0.03	1.4E-01	excluded
GK	88743	ES	2:03	1	5	-0.01	1.4E-01	excluded
FAM90A1	20184	ES	3	2	4	-0.02	1.4E-01	excluded
APOL1	62043	ES	2	1	3.2	-0.01	1.4E-01	excluded
ITGA2	71994	ES	2	1	3	0.00	1.4E-01	included
FBXO44	661	ES	3.2	2	4	-0.01	1.4E-01	excluded
NBR1	41697	ES	4.1	3	6	0.00	1.4E-01	included
MAEA	68475	ES	7:09	6	11	0.00	1.4E-01	included
SEC31B	12810	ES	26	25	27	0.01	1.4E-01	included
RRP1	60794	ES	2	1	3	0.00	1.4E-01	excluded
DCAF8	91247	ES	7.3:8.1:8.2	7.2	9	-0.01	1.4E-01	excluded
MYEF2	30482	ES	15	14	16	-0.02	1.4E-01	excluded
MRPL48	17733	ES	2	1	4	-0.01	1.4E-01	excluded
TRDMT1	10915	ES	4:05:06	2	7	0.01	1.4E-01	included
KLC2	16995	ES	4.2	3.3	5	0.00	1.4E-01	included
DYNC1I2	55951	ES	7.3	4	9	0.00	1.4E-01	included
ATXN2	24521	ES	18.1	17	19	0.01	1.4E-01	included
GPR56	36586	ES	3.2:3.3:7.1	3.1	7.2	-0.01	1.4E-01	excluded
CAST	116465	ES	35	34	36	0.00	1.4E-01	included
PLA2G12A	70336	ES	2.1:2.2	1	3	0.00	1.4E-01	included
C5orf45	74950	ES	5.2:6:7.1:7.3	5.1	7.4	0.00	1.4E-01	included
ARNTL	14444	ES	6.1:6.2	5	7	-0.02	1.4E-01	excluded
FPGS	87670	ES	11	10	12	0.00	1.4E-01	excluded
STK3	84657	ES	8:09	7	10	0.00	1.4E-01	included
SFTA3	121952	ES	2	1.3	6	0.01	1.4E-01	included
SKA2	42748	ES	1.2:2:4.1:4.2	1.1	5	0.00	1.4E-01	included
ZNF17	52248	ES	3	2	4	0.01	1.4E-01	included
UBXN11	101229	ES	7:08:09	2	10	0.02	1.4E-01	included
FAM207A	96051	ES	5	4	6	-0.01	1.4E-01	excluded
CSTF2	89612	ES	9	8	10	0.01	1.4E-01	included
PPM1M	65193	ES	4	3	5	0.01	1.4E-01	included
DHRS7B	39806	ES	3	2.2	4	0.00	1.4E-01	excluded
SIRT1	11909	ES	6.2	5	7	-0.01	1.4E-01	excluded
COQ5	24767	ES	3	2	4	0.00	1.4E-01	included
CSF1R	74059	ES	15	14	16	0.00	1.4E-01	included
RBPMS	83293	ES	11	9.1	13	0.00	1.4E-01	included
MRPL11	17030	ES	5	4	6	0.00	1.4E-01	included
ATPAF1	2822	ES	4	3	5	-0.01	1.4E-01	excluded
AIF1L	87918	ES	8.2:8.3:9.1	8.1	9.2	0.00	1.4E-01	excluded
AFMID	43801	ES	7:8:9:10:11.1:11.2	6	12	0.02	1.4E-01	included
ADA	59482	ES	9	8	10	0.01	1.4E-01	included
APTX	86089	ES	6.1:6.2	5.2	7.4	0.02	1.4E-01	included
ELP4	14825	ES	10:11	9	12	-0.02	1.4E-01	excluded

NPEPPS	42086	ES	4	3	5	0.00	1.4E-01	excluded
STRN3	27098	ES	8:09	7	10	-0.02	1.4E-01	excluded
HSD17B4	73081	ES	20	19	21	0.00	1.4E-01	excluded
RAB8B	31019	ES	10	9	11	0.00	1.4E-01	excluded
PDLIM5	69976	ES	17:18:19	15	20	0.00	1.4E-01	included
UBAP1	86153	ES	3	1	5	-0.01	1.4E-01	excluded
DHRS4	26790	ES	3:04:05	2	7.1	0.05	1.4E-01	included
ERC1	19659	ES	7	6	8	0.02	1.4E-01	included
LETMD1	21762	ES	3.1:3.2	2	7	0.00	1.4E-01	excluded
LDHA	14619	ES	5:6.1:6.2:7:8.1	4	8.2	-0.01	1.4E-01	excluded
BBS9	79227	ES	14	13	15	0.00	1.4E-01	excluded
COA1	79348	ES	2	1	4.2	-0.02	1.4E-01	excluded
DLAT	18711	ES	5.1:5.2:6	4	7	0.00	1.4E-01	included
LGALS9	39855	ES	10	9	11	0.00	1.4E-01	excluded
C17orf49	38824	ES	5	4	6.1	-0.01	1.4E-01	excluded
CNIH1	27582	ES	5	3	6.1	0.00	1.4E-01	included
IST1	37517	ES	13	12	14.1	-0.02	1.4E-01	excluded
OARD1	76088	ES	5	4.1	7	0.03	1.4E-01	included
STAT6	22531	ES	4	2.1	6	-0.02	1.4E-01	excluded
PPP3CA	70096	ES	2	1	3	0.00	1.4E-01	included
GTF2F1	47032	ES	5	4	6	0.00	1.4E-01	included
DENND1A	87519	ES	4	3	5	0.01	1.4E-01	included
FUZ	51081	ES	3	2.2	4	-0.01	1.4E-01	excluded
FBXO38	73981	ES	15.1	14	16	0.02	1.4E-01	included
IKBKB	83604	ES	3:4.1:5:6.1:6.2:7:9:10:11:12.1:12.2:13:14:15:16:17:18:19:20:21.1:	1.4	22.2	0.00	1.4E-01	included
GIT2	24379	ES	17.2:18.2:19	17.1	20	0.01	1.4E-01	included
FAM86C1	17440	ES	3.1:3.2	2	5.1	-0.03	1.4E-01	excluded
TPM1	30997	ES	3.2:5.2:6:7:8:10:11.1:12.1:12.2:13.1	3.1	13.2	0.02	1.4E-01	included
LITAF	34028	ES	6	4	7.1	0.00	1.4E-01	excluded
IRF7	13714	ES	4	3.2	5.3	0.03	1.4E-01	included
DNAJC24	14812	ES	8	7	9.1	-0.01	1.4E-01	excluded
CSAD	21968	ES	8:9:10.1:10.4	7.2	11	0.01	1.4E-01	included
ANXA7	91713	ES	2:3.1:3.2:4:5:6	1	7	-0.03	1.4E-01	excluded
PHF12	40028	ES	10	9.1	11	0.00	1.4E-01	excluded
MRPS18C	107824	ES	4	3	5	0.00	1.4E-01	excluded
CLASP1	55173	ES	27	26	28.1	0.00	1.4E-01	excluded
SEC16A	88177	ES	25	23.12	26	0.03	1.4E-01	included
ZC3H15	56485	ES	3	2	4	0.00	1.4E-01	excluded
DAB2	71865	ES	9	8	10	0.01	1.4E-01	included
FGD6	23746	ES	2	1	3	0.01	1.4E-01	included
BUD31	80621	ES	6	3.2	7	0.00	1.4E-01	excluded
RRP8	14161	ES	2.2:3.1:3.3	1	4	0.01	1.4E-01	included
ZNF821	37505	ES	7.1:7.2	6	8.1	-0.01	1.5E-01	excluded
CLN3	35736	ES	6:7:8:9	5	11	0.02	1.5E-01	included
NCAPH2	62843	ES	2	1.2	3	0.00	1.5E-01	included
ST13	62398	ES	2:03	1	4	0.00	1.5E-01	included
PCK2	26822	ES	4	2.3	5	0.01	1.5E-01	included
RBM42	49227	ES	05:06.1	4	6.2	0.01	1.5E-01	included
UBE2J2	53	ES	2.2:3	2.1	5	0.00	1.5E-01	excluded
PDE9A	60725	ES	5:07	3	8	0.00	1.5E-01	excluded
RRBP1	131582	ES	3.3:4:5:7:8:9:10:11:12:13:14:15:16:17:18	3.2	19	-0.02	1.5E-01	excluded
NFIA	3226	ES	10	9	11.2	0.00	1.5E-01	excluded
TRIT1	1926	ES	2:3.1:6.1:6.2	1	7.1	0.03	1.5E-01	included
C4orf29	70559	ES	12	11	13	-0.01	1.5E-01	excluded
CLCC1	3983	ES	2	1	3.1	0.02	1.5E-01	included
THOC7	65513	ES	2	1	3	0.00	1.5E-01	excluded
UBXN7	68245	ES	2	1	3	0.01	1.5E-01	included
STX16	59981	ES	4.1:4.2	1.4	5.1	0.00	1.5E-01	included

FAM13A	69917	ES	8:10:11:12:13	6	14	0.00	1.5E-01	included
DCTD	71241	ES	2.1:2.2:3	1.2	5	-0.01	1.5E-01	excluded
C22orf34	62745	ES	1.2:2	1.1	3	0.00	1.5E-01	excluded
CRCP	79871	ES	4:05	3	6	-0.01	1.5E-01	excluded
GALK2	30534	ES	5	1	6	-0.03	1.5E-01	excluded
ADC	1663	ES	3	2.2	4	-0.02	1.5E-01	excluded
SRSF11	3390	ES	6.1:6.3:6.4	4.2	6.5	0.00	1.5E-01	excluded
SETD5	63097	ES	4	3	5	-0.03	1.5E-01	excluded
LRCH3	68328	ES	2:03:04	1	5	0.00	1.5E-01	excluded
FCGBP	263959	ES	17:18:19:20:21:22:23:	8	25	-0.01	1.5E-01	excluded
TTC26	81947	ES	6.2:7:8.1	5	9	0.00	1.5E-01	included
TMEM150A	54305	ES	7	5	8	0.01	1.5E-01	included
HFE	75574	ES	03:05.1	2.2	5.2	0.01	1.5E-01	included
SLMAP	65397	ES	19	18.1	20	0.00	1.5E-01	excluded
ANKRD46	84711	ES	3	2.2	4	0.01	1.5E-01	included
SSBP4	48433	ES	2	1	3	0.00	1.5E-01	excluded
FOXP1	65607	ES	16:17	15	18	-0.01	1.5E-01	excluded
PTPN13	69835	ES	19:20:21	18	22	0.01	1.5E-01	included
TRDMT1	10914	ES	3:4:5:6	2	7	0.01	1.5E-01	included
SENP2	68024	ES	5.1:5.2	4.2	6	0.00	1.5E-01	included
SEC24C	12175	ES	12:13	11	14	0.00	1.5E-01	included
BMPR2	56947	ES	12	11	13	0.01	1.5E-01	included
PDCD6IP	63892	ES	2:03	1	4	-0.01	1.5E-01	excluded
CAST	72854	ES	16	15	17	0.06	1.5E-01	included
IMPA2	44663	ES	6.1	5	7	0.01	1.5E-01	included
WDR48	64124	ES	5.2	4.2	6	0.00	1.5E-01	excluded
SDC1	52763	ES	5.2:6:7.1	5.1	7.2	0.00	1.5E-01	included
TAF8	76168	ES	9	8.2	10	-0.03	1.5E-01	excluded
PPP2R5C	29322	ES	8	5	9	-0.03	1.5E-01	excluded
TANGO2	61118	ES	9:10	8	11	0.00	1.5E-01	excluded
SUPT4H1	42664	ES	4	3.2	5	0.00	1.5E-01	included
PA2G4	22362	ES	9.2:10:11:12:13.1	9.1	13.2	0.00	1.5E-01	excluded
RPE	57241	ES	9	7	10.1	0.00	1.5E-01	included
BSDC1	1592	ES	10.1	9	11.1	0.00	1.5E-01	excluded
LSM14A	48955	ES	3	2	4	-0.02	1.5E-01	excluded
RSPH1	60716	ES	2	1	3	-0.02	1.5E-01	excluded
PHB2	20047	ES	3.2:4.1	3.1	4.2	0.00	1.5E-01	excluded
SUSD1	87237	ES	16	15	17	-0.01	1.5E-01	excluded
MEF2BNB-MEF2	95082	ES	6	5	7	0.03	1.5E-01	included
PPAP2A	72040	ES	2	1	3	0.00	1.5E-01	included
KIAA0226	68307	ES	14	13	15	-0.03	1.5E-01	excluded
HPN	49020	ES	6:07:08	5	9	0.00	1.5E-01	included
ATP6V1A	66219	ES	3	2	4	0.00	1.5E-01	excluded
PIK3R1	72298	ES	10	9	12	0.01	1.5E-01	included
METAP2	23792	ES	3	2.2	4.1	0.00	1.5E-01	included
UEVLD	14668	ES	13	12	14	0.01	1.5E-01	included
RPS3	17850	ES	3.2:4.2	3.1	4.3	0.00	1.5E-01	excluded
EIF2AK2	53217	ES	11	10	12	0.00	1.5E-01	included
USP3	31042	ES	3.2:5:7:8:9:10:11	1	12	0.00	1.5E-01	included
RABEP2	35896	ES	2.2	1	4	0.00	1.5E-01	excluded
PRUNE	7545	ES	4	3	5	-0.01	1.5E-01	excluded
TBC1D2	87040	ES	2	1	3	0.01	1.5E-01	included
PTAR1	86548	ES	3:04	1	5	0.00	1.5E-01	excluded
SFTA3	27261	ES	4.2:4.3	2	5	0.01	1.5E-01	included
MAP9	70915	ES	9	8	10	0.00	1.5E-01	included
SAP30BP	43486	ES	2	1	3	0.00	1.5E-01	included
C2orf43	52788	ES	6	5	7	0.01	1.5E-01	included
BAD	16613	ES	2:03	1.3	4	-0.01	1.5E-01	excluded
YAF2	21156	ES	4:5.1:5.2:6	2	9.1	-0.01	1.5E-01	excluded
CAMK2B	79502	ES	14.1:14.2:15:17:18:19	12	21.1	0.01	1.5E-01	included
LIG1	50692	ES	6	5	7.1	0.00	1.5E-01	included
PNKP	51106	ES	11	10	12	0.00	1.5E-01	excluded

OXR1	84854	ES	17	14	18	0.00	1.5E-01	excluded
UBR2	76183	ES	43	42	44	0.00	1.5E-01	excluded
C12orf23	24184	ES	2.2:3.2	1	4.2	0.02	1.5E-01	included
POC1B	23632	ES	6:07:08	4	9	0.00	1.5E-01	excluded
TRPC4AP	59059	ES	9.2	8	10	-0.01	1.5E-01	excluded
LCORL	68868	ES	4	3	5	-0.01	1.5E-01	excluded
NMRK1	86628	ES	6.3	6.1	6.5	0.01	1.5E-01	included
TRAPPC1	39077	ES	2	1.3	3	0.00	1.5E-01	included
DBNL	79400	ES	3	2.2	4.1	0.00	1.5E-01	included
UBXN11	1261	ES	3:04	2	5	-0.03	1.5E-01	excluded
ATRIP	64659	ES	12	11	13	0.01	1.5E-01	included
ASB8	21442	ES	3.1	2.2	4.1	0.00	1.5E-01	included
HELQ	69792	ES	4	3	5	0.02	1.5E-01	included
GRK4	68626	ES	16	15	17	-0.03	1.5E-01	excluded
MOK	29393	ES	5	2	6.1	0.03	1.5E-01	included
DESI2	10470	ES	03:04.1	2	4.2	-0.01	1.5E-01	excluded
PDCD5	48882	ES	5.1	4.2	5.3	0.00	1.5E-01	included
PSPH	79779	ES	2.2:2.3:3	1	4	0.00	1.5E-01	included
FXR1	67752	ES	2:5:6:7:8:9:10:11:12:1	1	16	-0.02	1.5E-01	excluded
GTF2H2C	72393	ES	7:08	6	10.1	0.00	1.5E-01	included
STK38L	20869	ES	5	4.1	6	0.01	1.5E-01	included
CAMSAP1	88144	ES	6	4	7	0.02	1.5E-01	included
PYCARD	36249	ES	2	1	3	0.01	1.5E-01	included
BTBD7	29034	ES	2.2:3	1	5	-0.02	1.5E-01	excluded
TJAP1	76278	ES	3	2	5	-0.01	1.5E-01	excluded
SFTA3	121943	ES	2:3:4.1:4.2:5	1.3	6	-0.02	1.5E-01	excluded
ACY1	390895	ES	8.1:11:12	7.2	15	0.00	1.5E-01	included
SPATA7	28701	ES	5	3	6.1	-0.03	1.5E-01	excluded
ARFIP1	70859	ES	3:4:5:6	2	7	0.00	1.5E-01	excluded
GRB10	79716	ES	17	16	18	0.00	1.5E-01	included
TMEM128	68654	ES	4	3	5	0.00	1.5E-01	included
MRPL48	17729	ES	5:06	4	8	0.03	1.5E-01	included
TXNDC11	34030	ES	7	5	8	0.01	1.5E-01	included
SH2D3A	47086	ES	3	2	4	0.01	1.5E-01	included
CNTRL	98213	ES	41	40	42	0.01	1.5E-01	included
ITGA7	22218	ES	11	10	12	0.00	1.5E-01	included
SKA2	42735	ES	4.1	2	5	0.02	1.5E-01	included
PHF15	73408	ES	11	10	12	0.00	1.5E-01	excluded
COPS5	84061	ES	4.1:4.2	2.3	5	0.00	1.5E-01	excluded
WWP2	37320	ES	1.2:4.2	1.1	4.3	0.00	1.5E-01	excluded
ZNF114	50701	ES	6	5.2	7	0.00	1.5E-01	included
MTM1	90340	ES	4	3	5	0.00	1.5E-01	excluded
MIF4GD	43426	ES	3.2	2	4	-0.01	1.5E-01	excluded
EXOC7	43566	ES	8.2	7	9	-0.02	1.5E-01	excluded
C18orf25	45391	ES	3	2	4	0.02	1.5E-01	included
ARHGAP5	27134	ES	3	2	4.2	-0.02	1.5E-01	excluded
HSD17B7	8759	ES	3	2.1	4	-0.01	1.5E-01	excluded
WDR27	78475	ES	5:06	4	7	-0.01	1.5E-01	excluded
METTL20	21003	ES	6	5	7	0.01	1.5E-01	included
SRPR	19374	ES	2	1	3	0.00	1.5E-01	included
SS18	44963	ES	4:05:07	3	9	0.00	1.5E-01	excluded
TOMM7	78942	ES	3:04	1	5	-0.03	1.5E-01	excluded
SFTA3	27274	ES	5	2	6	-0.01	1.5E-01	excluded
IFNGR1	77935	ES	3	1	4	0.00	1.5E-01	excluded
EPB41L1	59275	ES	9	8	10	0.00	1.5E-01	excluded
TRIM33	4325	ES	12	11	13	-0.02	1.5E-01	excluded
PDE8A	32342	ES	9:11	8	12	0.01	1.5E-01	included
PPP1R8	1345	ES	4	3	5	0.00	1.5E-01	included
LRRFIP2	63969	ES	6:7:8:9:10:14:15:16:1	5	18	0.00	1.5E-01	included
HMBS	19106	ES	2	1.1	4	0.00	1.5E-01	excluded
NAPSA	51187	ES	3	2	4	0.00	1.5E-01	included
DRG2	39558	ES	4:5.1:6.1:6.2:6.4:7:8.1	3.2	10	0.00	1.5E-01	included

MDM1	22924	ES	7	6	8	-0.04	1.5E-01	excluded
PRMT3	14720	ES	3	2	4	-0.01	1.5E-01	excluded
GMPS	67354	ES	2:03	1	4	0.00	1.5E-01	included
PPP2R5D	76200	ES	3.4:4.1	3.3	4.2	0.00	1.5E-01	included
CIRBP	46438	ES	8.1:8.2	7.5	8.4	-0.03	1.5E-01	excluded
BLM	32491	ES	18:19	17	20	0.00	1.5E-01	excluded
CTNND1	16001	ES	2.2:2.3:3:4.2:4.3	2.1	5	0.00	1.5E-01	excluded
TAX1BP1	79066	ES	5	4	6	0.00	1.5E-01	included
C19orf57	47944	ES	6	5	7	-0.01	1.5E-01	excluded
ATP6V0D1	37072	ES	5	4	7	0.00	1.5E-01	excluded
GGA3	43401	ES	7	6	8	0.00	1.5E-01	included
NME6	64587	ES	5.1:5.2	4	6	-0.03	1.5E-01	excluded
ZC3H14	28727	ES	5	4	6	0.00	1.5E-01	included
AGBL5	52926	ES	14	13	15	0.02	1.5E-01	included
ZNF333	48022	ES	7	6	8	-0.03	1.5E-01	excluded
ARMC8	66971	ES	2.2	1	3	0.03	1.5E-01	included
POMT2	28581	ES	2	1	3	-0.01	1.5E-01	excluded
SMG7	9184	ES	2	1	3	-0.02	1.5E-01	excluded
FOPNL	34190	ES	5	4	6	0.00	1.6E-01	included
SMAD4	45563	ES	14:15	13	16	0.00	1.6E-01	excluded
NOSTRIN	55834	ES	14	13	15	0.01	1.6E-01	included
PFDN5	21990	ES	4.1:4.2	2	5	0.02	1.6E-01	included
MANEAL	1836	ES	3	2.3	4.1	0.01	1.6E-01	included
SORBS2	71379	ES	20	19	22	0.01	1.6E-01	included
PNPO	42138	ES	2.1:2.2	1.1	3	0.00	1.6E-01	included
PQLC1	46254	ES	7	6	9	-0.01	1.6E-01	excluded
LRRC28	32681	ES	3:5:6:7.2:8:10:11	2	12	0.01	1.6E-01	included
FBXW2	87390	ES	4	3	5	0.00	1.6E-01	excluded
OXR1	84853	ES	16:17	14	18	0.00	1.6E-01	excluded
UQCC1	59108	ES	6.1:6.2:7.1:7.2:8	3	9	0.00	1.6E-01	included
STYXL1	80152	ES	4	3	6	-0.01	1.6E-01	excluded
C4orf21	70381	ES	4	3	6	0.01	1.6E-01	included
TNFRSF25	451	ES	3:4:5:6.2:6.5:7.1	2	7.2	-0.01	1.6E-01	excluded
ARHGEF4	55358	ES	9	8	10	0.00	1.6E-01	included
AP1M1	48148	ES	6	5	7	0.00	1.6E-01	excluded
PDCD10	67563	ES	3.3	1.1	5	0.00	1.6E-01	included
SGSH	44035	ES	3	2.2	4	0.00	1.6E-01	included
POT1	81642	ES	17	16	18.1	0.01	1.6E-01	included
VPS29	24440	ES	2:3.1:3.2:4	1	5	0.00	1.6E-01	included
EXD2	28136	ES	3	1	4	0.02	1.6E-01	included
BRD1	62752	ES	11.1	10	11.3	-0.01	1.6E-01	excluded
ZBTB44	19495	ES	5.2:7.1:7.2	5.1	7.3	0.01	1.6E-01	included
CTNND1	15965	ES	2.2:5:6:7	2.1	8	-0.02	1.6E-01	excluded
STRADA	42971	ES	6	3	7	0.00	1.6E-01	excluded
HNRNPD	69702	ES	3	1.3	4	0.00	1.6E-01	included
NKIRAS2	40978	ES	6	5.6	7	-0.03	1.6E-01	excluded
USP3	31035	ES	9:10	8	11	0.00	1.6E-01	included
C16orf87	36315	ES	3	2	4	0.02	1.6E-01	included
ZNF608	73152	ES	7	6	8	0.00	1.6E-01	included
ARMC6	48579	ES	2.2:4	1.1	5	0.00	1.6E-01	excluded
CBY1	62251	ES	2	1	3	0.01	1.6E-01	included
TIMM9	27709	ES	3	2	4.2	-0.01	1.6E-01	excluded
VEGFA	76339	ES	6:7.1:7.2:8.1:8.2	5	9.1	0.01	1.6E-01	included
AGAP8	11529	ES	3	2.2	4	-0.01	1.6E-01	excluded
COPZ1	22171	ES	4	3.2	6	0.02	1.6E-01	included
POMZP3	80187	ES	4	3	5	-0.02	1.6E-01	excluded
CDS2	58653	ES	2:03:04	1	5	0.00	1.6E-01	included
ATG16L2	17657	ES	4.1:4.2:4.3:5:6.1:6.3:6.4:7:8:9:10:11:12:13	3	14	0.00	1.6E-01	included
MANBAL	59338	ES	5	4.2	6	0.00	1.6E-01	excluded
MAP3K7	77019	ES	15	14	16	0.00	1.6E-01	excluded
RSAD1	42403	ES	2:03	1	4	0.00	1.6E-01	excluded

ANXA2	30955	ES	2:03	1.1	4.2	0.00	1.6E-01	excluded
VEZT	23763	ES	6.1:6.2	5	9	-0.01	1.6E-01	excluded
CNOT1	36674	ES	4.2:5:6:7:8:9:10:11:12	4.1	14.2	-0.01	1.6E-01	excluded
AZI2	63796	ES	2	1	3	0.00	1.6E-01	included
EDEM1	63033	ES	5	4	6	0.00	1.6E-01	included
FANK1	13449	ES	2	1	5	-0.04	1.6E-01	excluded
PML	31642	ES	6.1:6.4:6.5:6.6:6.7	4	6.8	0.00	1.6E-01	included
TLK1	55928	ES	5	4	7	0.01	1.6E-01	included
ZNF766	51430	ES	7	6	8.1	-0.03	1.6E-01	excluded
ARL2	16727	ES	4	3	5.1	0.00	1.6E-01	excluded
ECE1	196613	ES	3.2:4.2:5:6:7:8:9	3.1	10	-0.03	1.6E-01	excluded
MAX	27950	ES	5.2:5.3:5.5:5.6	5.1	5.8	0.00	1.6E-01	excluded
NRG1	83321	ES	6:07	5	10	0.00	1.6E-01	included
ZNF268	25345	ES	12	11	13	0.02	1.6E-01	included
MDM2	23285	ES	3:5.1:5.2	2.1	6	0.00	1.6E-01	included
BLOC1S6	30432	ES	8	7.2	9	0.00	1.6E-01	included
UBXN11	101234	ES	4:05	2	7	0.02	1.6E-01	included
EMC4	29844	ES	3.1:3.2:4.2	2.2	6	0.00	1.6E-01	included
HLCS	60539	ES	4	3	6	-0.02	1.6E-01	excluded
MARK3	29453	ES	7	6	8	0.00	1.6E-01	excluded
SLC35A3	3863	ES	8:09	7	10.1	0.00	1.6E-01	excluded
ADAM10	30906	ES	2:3:4:5:6:7:8.1	1	8.2	0.00	1.6E-01	excluded
HSP90AA1	29335	ES	2	1	4	-0.02	1.6E-01	excluded
YY1AP1	8109	ES	5	4.1	6	-0.01	1.6E-01	excluded
DRG1	61851	ES	6	5	7	0.00	1.6E-01	excluded
TATDN1	85099	ES	2	1.1	3	0.00	1.6E-01	included
ITGB7	21974	ES	11:12:13	10	14	0.00	1.6E-01	included
KDM1B	75463	ES	9:10	8	11	0.00	1.6E-01	excluded
MRPL42	23703	ES	3.1	2.2	4	0.00	1.6E-01	excluded
MOK	29396	ES	05:06.1	2	7	0.03	1.6E-01	included
FBXL5	68813	ES	3	2.2	4	0.00	1.6E-01	included
KANSL1L	57262	ES	10	9.1	11	-0.01	1.6E-01	excluded
OGG1	63168	ES	05:06.1	4	8	-0.01	1.6E-01	excluded
EXOSC2	87905	ES	06:07.1	5	7.2	0.00	1.6E-01	included
NCOA4	11542	ES	5:06:07	1	8	0.00	1.6E-01	excluded
BCL7B	79948	ES	6	4	7	0.00	1.6E-01	excluded
MYO3A	11029	ES	18:19:20:21:22:23:24:<br>25:26:27:28:29:30:31:	17	35	0.02	1.6E-01	included
PCID2	26363	ES	4.2:5.1	4.1	5.2	0.00	1.6E-01	excluded
ZSCAN9	75722	ES	4	3.2	5	0.02	1.6E-01	included
AKAP10	39785	ES	3	2	4.1	-0.03	1.6E-01	excluded
RCBTB2	25868	ES	2:03	1	5	-0.03	1.6E-01	excluded
BOD1	74585	ES	2:03	1	4	0.00	1.6E-01	excluded
ZNF195	13978	ES	6:09:11	5.1	13	-0.01	1.6E-01	excluded
PPIL3	56764	ES	4.1:4.2	3	6	0.02	1.6E-01	included
SUMO1	56939	ES	3:04	1	5.1	-0.03	1.6E-01	excluded
TMPO	23850	ES	7:08	5.1	9	-0.02	1.6E-01	excluded
YAF2	21165	ES	5.2	2	9.1	-0.01	1.6E-01	excluded
SKA2	42739	ES	02:04.1	1.2	5	0.03	1.6E-01	included
ACAA1	64018	ES	9.3	9.1	10	0.00	1.6E-01	excluded
PRKAG2	82395	ES	8	7.2	9.1	-0.01	1.6E-01	excluded
WDR27	78473	ES	26	25	27	0.03	1.6E-01	included
ACAD11	66807	ES	10:11.1	9	12	0.00	1.6E-01	excluded
EEF1D	85443	ES	8.3:9:10.1:10.2:11:12.<br>1:12.2:13.1	8.2	13.2	0.00	1.6E-01	included
NSRP1	40083	ES	7	3	8	0.01	1.6E-01	included
MORN4	12733	ES	2.1	1	3	0.01	1.6E-01	included
MALL	54926	ES	2	1	3	0.00	1.6E-01	excluded
ANAPC15	17571	ES	3.2:3.3:4.1:4.2	1.2	5	0.00	1.6E-01	excluded
ATP6V0E1	74574	ES	2	1	3.1	0.00	1.6E-01	included
FAU	16774	ES	2.4	2.2	2.6	0.00	1.6E-01	excluded
POLL	12888	ES	4	3	5.1	0.00	1.6E-01	included

GNB2L1	264680	ES	5:7.2:8.1:8.2	3	9	0.01	1.6E-01	included
BAZ2B	55697	ES	13	12	14	-0.02	1.6E-01	excluded
FGFR2	13311	ES	15:17.1	14	18	0.00	1.6E-01	included
ELN	80040	ES	32	31	33	-0.02	1.6E-01	excluded
ATP5J	60263	ES	1.5:2	1.4	3	0.02	1.6E-01	included
CAMLG	96929	ES	3	2	4	0.00	1.6E-01	included
IQCE	78627	ES	3	1	4	0.01	1.6E-01	included
METTL3	26597	ES	4.1:4.2	3.3	5	0.00	1.6E-01	excluded
MPZL3	18977	ES	3	2.2	4	-0.01	1.6E-01	excluded
RIC8B	24164	ES	12:13:14:15:16	11	17	0.02	1.6E-01	included
RNF40	36187	ES	9	8	10.1	0.00	1.6E-01	included
ZNHIT3	40471	ES	02:03.1	1	3.2	0.00	1.6E-01	included
RPS15	46489	ES	2	1.5	3.2	0.00	1.6E-01	excluded
BBS1	17050	ES	7:09:10	6.2	11	0.01	1.6E-01	included
MSTO1	8094	ES	4.1:4.2	3	5	0.00	1.6E-01	included
MCCC2	72444	ES	11.2:12.1	11.1	12.2	-0.01	1.6E-01	excluded
FKBP1A	58491	ES	1.3	1.1	2	0.00	1.6E-01	included
ASCC1	12081	ES	14	13	16	-0.01	1.6E-01	excluded
SCRN1	79101	ES	8	7	9	0.00	1.6E-01	included
ACAA1	64022	ES	6	5	7	0.01	1.6E-01	included
TIMELESS	22432	ES	10.2:11:12:13:14:15:1<br>6:17:18:19:20:21.1	10.1	21.2	0.00	1.6E-01	excluded
NAP1L1	23493	ES	3:04	1	5	-0.04	1.6E-01	excluded
MORF4L1	32132	ES	4:05:06	3.1	7	0.01	1.6E-01	included
PAX8	55048	ES	8:9.1:9.2	7	10	0.00	1.6E-01	included
HVCN1	24495	ES	5.2	4.2	6.1	-0.01	1.6E-01	excluded
DOK3	74789	ES	4	3.2	5	-0.01	1.6E-01	excluded
STRN3	27097	ES	10	7	11	-0.01	1.6E-01	excluded
ADC	1662	ES	6:07:08	5	9	0.00	1.6E-01	included
SDHAF2	16229	ES	2:3:4.1:5.1:5.2	1	6	0.00	1.6E-01	included
TMEM55A	84421	ES	6	5	7	0.00	1.6E-01	included
CDC23	73520	ES	4	3.2	5	0.00	1.6E-01	included
MARK3	29448	ES	17:18	16	19	-0.01	1.6E-01	excluded
SLC35F6	52911	ES	3	2	4	0.00	1.6E-01	included
SSR2	8151	ES	5:6.1:6.2:7	4.1	8	-0.02	1.6E-01	excluded
EEF1D	85447	ES	8.3:9:10.1:10.2:11:12.	8.2	13.2	-0.02	1.6E-01	excluded
UBE2D3	70146	ES	2.2:2.3:2.4:3.2	2.1	3.3	0.01	1.6E-01	included
ZCCHC17	1461	ES	3	2	4	0.01	1.6E-01	included
MTF2	3770	ES	11	10	12	-0.01	1.6E-01	excluded
MBNL1	67315	ES	10:11	9	13	0.02	1.6E-01	included
MYH11	34188	ES	42	41	43	-0.03	1.6E-01	excluded
SORBS1	12621	ES	27	26	28	-0.01	1.6E-01	excluded
GPBP1	72128	ES	3.2:5	3.1	6	0.00	1.6E-01	included
POLL	12889	ES	3:04	1.5	5.1	0.00	1.6E-01	included
PDPR	37333	ES	8	7	9	-0.03	1.6E-01	excluded
PAFAH1B1	38408	ES	10	9	11	0.00	1.6E-01	included
ZNF268	25364	ES	9:10.1:10.2:11:12	7	13	0.00	1.6E-01	excluded
KLC2	16993	ES	7:8:9:10:11:12:13:14:<br>15:16.1:16.2:16.3	6	16.4	-0.02	1.6E-01	excluded
RABL5	81065	ES	2	1	3.2	0.00	1.6E-01	included
ZNF821	37506	ES	7.1	6	8.1	-0.01	1.6E-01	excluded
NFATC3	37189	ES	2:03	1	4.1	0.00	1.6E-01	included
LRRFIP2	63968	ES	7:8:10:11:12:13:14:15	5	18	0.00	1.6E-01	included
TBP	78495	ES	2.2	1	3	0.00	1.6E-01	excluded
TXNL4A	46277	ES	5	4	7.2	-0.01	1.7E-01	excluded
THAP6	69538	ES	2	1.1	3	-0.01	1.7E-01	excluded
TMEM218	19278	ES	4.3:4.4:5.1	4.2	5.2	0.03	1.7E-01	included
SMARCD3	82365	ES	5	3.2	6	-0.01	1.7E-01	excluded
C12orf23	24179	ES	5	4.2	6	-0.03	1.7E-01	excluded
MX2	60663	ES	5	4	6	-0.01	1.7E-01	excluded
GRN	546752	ES	10	9	12	-0.01	1.7E-01	excluded
SREBF1	39505	ES	8	7	9	0.01	1.7E-01	included

GNPNAT1	27553	ES	2	1	3	0.00	1.7E-01	excluded
CADM1	18858	ES	9	8	12	0.00	1.7E-01	excluded
MCCC1	67778	ES	16	15	17	0.00	1.7E-01	excluded
MRPL48	17722	ES	7:08	5	10	0.02	1.7E-01	included
POMT1	87942	ES	5.2:6:7:8.1	4	9	0.01	1.7E-01	included
ETV7	75974	ES	3	2	4	-0.02	1.7E-01	excluded
HPS5	14595	ES	1.2:1.3:2	1.1	3	0.03	1.7E-01	included
RPAIN	38680	ES	5	4	6.1	0.00	1.7E-01	included
DCAF6	8881	ES	18	17	19	0.00	1.7E-01	included
TNFRSF25	443	ES	3:4:5:6.2:6.3:6.4:6.5:7	2	7.2	-0.01	1.7E-01	excluded
C8orf31	85393	ES	6	5	7	0.01	1.7E-01	included
RAB6A	17709	ES	5	4	7	0.00	1.7E-01	excluded
TSPAN3	31981	ES	3	2.2	4	0.00	1.7E-01	included
NPC2	28405	ES	4.1:4.2	3	4.4	0.00	1.7E-01	excluded
CNOT10	63825	ES	6	5	7	0.00	1.7E-01	included
POLR3D	83001	ES	5	4	6	0.01	1.7E-01	included
SLC26A1	68438	ES	2	1	3	-0.02	1.7E-01	excluded
NKIRAS2	40998	ES	5.2:5.3:5.4:5.5:5.6	3.2	7	0.00	1.7E-01	included
C12orf10	22014	ES	4	3	5	0.00	1.7E-01	included
MLXIPL	79990	ES	11.1:11.2	10	12	0.01	1.7E-01	included
RPL8	85643	ES	02:03.1	1.4	3.2	0.00	1.7E-01	included
SULT1C2	54876	ES	4:5.3:6.1	3	6.2	-0.01	1.7E-01	excluded
TLE2	46651	ES	10.2:10.3:10.4:10.5:11	10.1	12	-0.02	1.7E-01	excluded
MGEA5	12921	ES	10	9	11	0.00	1.7E-01	included
ATG4D	47533	ES	4.1:4.2:5:6	3.2	7	0.00	1.7E-01	included
PIGT	59560	ES	2.1:4	1	5.2	-0.02	1.7E-01	excluded
SLC25A45	16826	ES	9	8	10	0.02	1.7E-01	included
ZNF280D	30776	ES	26	25	27.1	-0.01	1.7E-01	excluded
DPP3	17037	ES	3	2.3	4	0.00	1.7E-01	excluded
ARMC8	66962	ES	12	11	13.1	0.00	1.7E-01	included
IKBKB	83703	ES	1.4	1.1	2	0.00	1.7E-01	included
PLS3	89924	ES	14	13	15	0.00	1.7E-01	included
PARP8	71981	ES	16	15	17	0.00	1.7E-01	excluded
ATG4B	58403	ES	4:05	3	6	0.00	1.7E-01	excluded
GNB2	80991	ES	3.2:4.1:4.2:5	1	6	0.00	1.7E-01	included
ZNF415	51666	ES	8.1:8.2	7.2	9	-0.02	1.7E-01	excluded
RABL2B	62918	ES	7.1:9	6	10.1	0.00	1.7E-01	included
TARBP2	22082	ES	3.1:3.2:3.3:4.1	1.2	5.1	0.00	1.7E-01	included
DENND1B	9307	ES	3	2	4	0.04	1.7E-01	included
HAUS4	26676	ES	5	4	6	-0.01	1.7E-01	excluded
DENND1A	87521	ES	3	2	4	0.00	1.7E-01	excluded
AARSD1	41167	ES	3	2	4	0.00	1.7E-01	excluded
TBCD	44422	ES	37	36	38	0.00	1.7E-01	excluded
INO80E	36017	ES	6.3	5	11	0.01	1.7E-01	included
FAM86A	33890	ES	3.2:5.1	3.1	5.2	-0.04	1.7E-01	excluded
C11orf49	15635	ES	3	1	7	-0.01	1.7E-01	excluded
ICA1	78791	ES	13.1:14:16:17:18	12	19	0.00	1.7E-01	included
PMF1	8203	ES	2:3:4:5	1	6	0.00	1.7E-01	excluded
USP39	54317	ES	13	12	14.1	0.00	1.7E-01	excluded
PRMT7	37222	ES	7:08	6	9	0.00	1.7E-01	included
IRAK4	21264	ES	3:05	1.1	6	0.01	1.7E-01	included
RWDD4	71280	ES	02:03.2	1	4	0.02	1.7E-01	included
TIRAP	19386	ES	5	4	6.1	0.02	1.7E-01	included
LIMS2	55234	ES	14	13.2	15	0.00	1.7E-01	excluded
BUD31	80620	ES	4:06	3.2	7	0.00	1.7E-01	excluded
SDC1	52764	ES	3	2.2	4	0.00	1.7E-01	excluded
MAP4	64562	ES	12.1:12.2	11	14	0.01	1.7E-01	included
NUDT16L1	33788	ES	2.2:2.4	2.1	2.5	0.01	1.7E-01	included
CHEK2	61538	ES	5:7.1:7.2	3	9	0.00	1.7E-01	excluded
TMEM254	12338	ES	5	3	6	-0.01	1.7E-01	excluded
NARF	44398	ES	10	9	11.1	-0.01	1.7E-01	excluded
BBS1	17048	ES	8	7	9	-0.01	1.7E-01	excluded

ZCCHC7	86397	ES	3	1.1	5	-0.01	1.7E-01	excluded
WDR45	89083	ES	7.2:8	5	9.2	-0.01	1.7E-01	excluded
ODF3B	62860	ES	3.1:3.2:4.1	2.2	4.2	0.04	1.7E-01	included
COPS7B	57959	ES	4.2:4.3:4.4	3	5	-0.04	1.7E-01	excluded
CARD8	50720	ES	2.3	2.1	3	0.02	1.7E-01	included
PML	31654	ES	6.1:6.2:6.4:6.7	4	6.8	0.01	1.7E-01	included
IQCK	34337	ES	6.1:6.2:6.3	5	7.1	0.04	1.7E-01	included
PEMT	39495	ES	5	4	6	0.00	1.7E-01	excluded
TRAPPC13	72247	ES	4	3	5	0.01	1.7E-01	included
CNTD2	95212	ES	2.2	1	3	0.03	1.7E-01	included
ARAP1	92727	ES	3	2	4	-0.01	1.7E-01	excluded
GPR56	36590	ES	5.2	2	7.2	0.01	1.7E-01	included
IL6ST	72106	ES	4.1:4.2:5	3.2	6	0.00	1.7E-01	excluded
ZNF260	49392	ES	2.2:3	2.1	5	-0.02	1.7E-01	excluded
PHYHD1	87799	ES	9.1	8	10	0.01	1.7E-01	included
DNAJC4	16600	ES	3	2	4	-0.01	1.7E-01	excluded
RIN3	28998	ES	5	4	6	-0.01	1.7E-01	excluded
EWSR1	61582	ES	9.2:11.2:12:13.1	9.1	13.2	0.00	1.7E-01	excluded
PCCB	66924	ES	1.2:2:3:6:7.1	1.1	7.2	0.00	1.7E-01	excluded
FUBP1	3546	ES	3	2	4	0.01	1.7E-01	included
MBNL1	67322	ES	8:09	7	11	0.00	1.7E-01	excluded
IRF9	26877	ES	8	7	9	-0.01	1.7E-01	excluded
YAF2	21143	ES	5.2:7:8	2	9.1	0.00	1.7E-01	excluded
SPNS1	35905	ES	2	1.4	3	0.00	1.7E-01	included
TMEM9B	14289	ES	3	2	4	0.00	1.7E-01	excluded
PPP1R1A	22198	ES	4:05	3	6	0.01	1.7E-01	included
HPS1	12762	ES	5	4	6	-0.01	1.7E-01	excluded
LMBR1	82480	ES	4.1:5:7.2:8	2.1	9	-0.01	1.7E-01	excluded
GATAD2A	48636	ES	9	8	10	0.00	1.7E-01	excluded
TMEM176B	82258	ES	5	4.3	6	0.00	1.7E-01	excluded
YAF2	21110	ES	7	6	9.1	-0.03	1.7E-01	excluded
ERBB2IP	72257	ES	25	24.3	26	0.00	1.7E-01	excluded
OBSL1	140012	ES	6	5	7	0.00	1.7E-01	excluded
WDR41	72584	ES	3:04	2	5	0.01	1.7E-01	included
VPS29	24434	ES	3.2:4	3.1	5	0.00	1.7E-01	included
ALDH3A2	39758	ES	9	8	10	0.00	1.7E-01	excluded
EXOC2	75139	ES	2:3:4:5:6:7:8:9:10:11	1	12	0.00	1.7E-01	excluded
HNRNPC	26537	ES	8.1:8.2	7.2	8.4	0.00	1.7E-01	excluded
TMBIM4	22894	ES	08:09.1	7	9.2	0.00	1.7E-01	included
FAM76B	18382	ES	9	8	10.1	-0.03	1.7E-01	excluded
GPRASP2	89710	ES	2	1	3	-0.01	1.7E-01	excluded
CD74	152983	ES	9	4	10.2	0.00	1.7E-01	excluded
APOL3	61994	ES	4.3	3.3	5	0.02	1.7E-01	included
HDAC7	21377	ES	04:05.2	2	6	0.00	1.7E-01	excluded
BBS9	79222	ES	17	15	18	0.00	1.7E-01	included
TSPAN9	19754	ES	2	1	3.2	0.01	1.7E-01	included
CHFR	25317	ES	5:6.1:6.2	4	8	0.03	1.7E-01	included
PICALM	18173	ES	14.2	13	15	0.01	1.7E-01	included
DDX52	40540	ES	5	2	6	0.01	1.7E-01	included
TPD52L1	77414	ES	9.1:9.2	6	10	0.00	1.7E-01	included
AGO3	1743	ES	4	3	5	-0.01	1.7E-01	excluded
FUT8	28014	ES	7	6	8	0.00	1.7E-01	excluded
SEP15	3688	ES	3	2	4	0.00	1.7E-01	excluded
RPUSD1	33011	ES	4	3.2	5.1	0.00	1.7E-01	excluded
MRPS18C	69793	ES	3:04	2	5	0.00	1.7E-01	excluded
DCUN1D5	18480	ES	2:03	1.1	5	0.01	1.7E-01	included
POGLUT1	66301	ES	05:06.1	4	6.2	0.02	1.7E-01	included
EWSR1	61581	ES	9.2:11.1:11.2:12:13.1	9.1	13.2	0.00	1.7E-01	excluded
SYNE2	27856	ES	102	101	103	0.00	1.7E-01	excluded
SCMH1	2054	ES	6:08	5	9	-0.03	1.7E-01	excluded
USP3	31041	ES	2:3.2:5:7:8:9:10:11	1	12	0.00	1.7E-01	included
RFESD	72814	ES	4	3	5	-0.03	1.7E-01	excluded

CNIH4	9955	ES	04:05.1	3	5.2	0.00	1.7E-01	excluded
VCPKMT	27450	ES	5	4	6	-0.02	1.7E-01	excluded
ZC3HC1	81766	ES	3:07	1	8	0.01	1.7E-01	included
PFDN5	21997	ES	2:3:5:6.1	1	6.2	-0.03	1.7E-01	excluded
G3BP1	74191	ES	7	5	8	0.00	1.7E-01	excluded
CNNM2	12985	ES	7	6	8	0.02	1.7E-01	included
DCUN1D5	18484	ES	2	1.1	3	0.00	1.7E-01	included
CLEC1A	20299	ES	2:03:04	1	5	0.01	1.7E-01	included
PPHLN1	21225	ES	9	7	11	0.01	1.7E-01	included
AGTRAP	671	ES	4.1:4.2	3	5	-0.01	1.7E-01	excluded
CARF	56985	ES	5.1:5.2:6.1:6.2	3.1	7	0.02	1.7E-01	included
POLM	79459	ES	6.2:6.3:7:8	6.1	9.1	0.04	1.7E-01	included
STX17	87069	ES	6	5	7	-0.02	1.7E-01	excluded
PEX19	8456	ES	5	4	6	0.00	1.7E-01	included
SH2B1	35875	ES	3.6:4.1	3.3	4.2	0.01	1.7E-01	included
CARD8	50712	ES	09:10.2	8	11	0.01	1.7E-01	included
PRSS8	36243	ES	3.2:4.1	3.1	4.2	0.00	1.7E-01	excluded
PFKM	21429	ES	8	7.3	9	0.03	1.7E-01	included
IFT88	100187	ES	27	26	28	-0.01	1.7E-01	excluded
TCN2	61789	ES	2	1	3	0.00	1.7E-01	excluded
PLA2G6	62210	ES	4	3.2	5	-0.01	1.7E-01	excluded
CHID1	13817	ES	6.2	5.2	7	0.00	1.7E-01	included
ZNF208	48802	ES	2	1	3	-0.02	1.7E-01	excluded
RAD23A	47891	ES	8	7.2	9	0.00	1.7E-01	included
MGA	30104	ES	15	14	16.1	0.01	1.7E-01	included
HACL1	63586	ES	10	9	11	-0.01	1.7E-01	excluded
AHI1	77893	ES	34	33	36	0.01	1.7E-01	included
DHRS4	26788	ES	4:05:06	2	7.1	0.04	1.7E-01	included
IRF9	26879	ES	2.1:2.2	1	3	0.00	1.7E-01	excluded
CYB561D1	4027	ES	1.2:2	1.1	3	0.02	1.7E-01	included
SAMD12	84995	ES	3	2	4.1	0.01	1.7E-01	included
RAB36	61297	ES	4	3	5	0.00	1.7E-01	included
DCAF17	55933	ES	10:11	9	12	0.01	1.8E-01	included
FANCI	32418	ES	26	25	27	0.01	1.8E-01	included
ATE1	13323	ES	5	4	6	0.00	1.8E-01	included
SLC12A8	139156	ES	13:14	12.1	16.1	-0.02	1.8E-01	excluded
USP14	44437	ES	3	2	4	0.00	1.8E-01	included
PICALM	18175	ES	3:04:05	2	6	0.00	1.8E-01	excluded
APP	60285	ES	9	8	10	0.00	1.8E-01	included
PDE8B	72570	ES	2	1	3	0.00	1.8E-01	excluded
TSPO	62572	ES	3	1	4	0.00	1.8E-01	excluded
LGALS1	62146	ES	3	2.2	4	0.00	1.8E-01	included
NCOA4	11549	ES	2	1	5	0.00	1.8E-01	excluded
DTX2	80176	ES	4	2	5	0.00	1.8E-01	included
ARHGAP30	8539	ES	4	3	5	-0.01	1.8E-01	excluded
RBM6	64948	ES	3.1:3.2:4:5:6	2	7	-0.02	1.8E-01	excluded
UBXN4	55451	ES	3.1:3.3	2	4	0.00	1.8E-01	included
ASCC2	61687	ES	2	1	5	-0.01	1.8E-01	excluded
UNK	43511	ES	6	2	7	-0.02	1.8E-01	excluded
ISCU	24231	ES	5.6	5.1	6	0.00	1.8E-01	excluded
AHSA2	53691	ES	5	4	6	-0.01	1.8E-01	excluded
ST6GALNAC2	43600	ES	3	1	4	0.01	1.8E-01	included
DYRK1B	49847	ES	10.1	9	10.3	-0.01	1.8E-01	excluded
ZNF148	66548	ES	9.2:10.1	9.1	10.2	0.00	1.8E-01	excluded
YWHAZ	84729	ES	7.1:7.2	3.2	11	0.00	1.8E-01	excluded
TACC1	83470	ES	2:03	1	7	0.05	1.8E-01	included
TCOF1	96962	ES	16	15	17	0.01	1.8E-01	included
TMA16	71020	ES	5	4	6.1	-0.01	1.8E-01	excluded
ANKRD13C	3399	ES	3	2	4	0.00	1.8E-01	included
FBL	49850	ES	2	1	3	0.00	1.8E-01	excluded
SCFD1	27063	ES	9	8	10	-0.01	1.8E-01	excluded
MANBA	70115	ES	2	1	3	-0.04	1.8E-01	excluded

TCEAL4	89738	ES	4.3:5.1	4.2	5.2	0.00	1.8E-01	excluded
TFDP1	26390	ES	13.1:13.2	12	14	-0.01	1.8E-01	excluded
DNAJB6	82512	ES	08:09.1	5	10	0.00	1.8E-01	excluded
RABL5	81062	ES	02:03.2	1	4	0.01	1.8E-01	included
NADK	230	ES	6:07	5	9.1	0.00	1.8E-01	excluded
MAP4K3	53331	ES	6:7:8:9	5	10	0.00	1.8E-01	excluded
USP34	53693	ES	74:75	73	76	-0.01	1.8E-01	excluded
IQCD	24625	ES	3.1:4	2.2	5	0.02	1.8E-01	included
RDX	18638	ES	5:06	4	7.1	-0.01	1.8E-01	excluded
SNX1	139177	ES	4.2:5:6:8:9:10.2:11:12 :13.1:14:15	3	16.1	0.00	1.8E-01	excluded
CPSF3L	139	ES	2.1:3	1	4	0.00	1.8E-01	excluded
ZC3H15	56484	ES	5:06	4	7	0.00	1.8E-01	included
MOCS1	76070	ES	11.1:11.2	10	12.1	-0.01	1.8E-01	excluded
ZNF707	85477	ES	9:10	8.2	11	-0.01	1.8E-01	excluded
ZNF786	82164	ES	2	1	3	-0.04	1.8E-01	excluded
VCAN	72704	ES	7:08	6.1	9	-0.02	1.8E-01	excluded
GNB2L1	75057	ES	7.2:8.1:8.2	6	9	0.00	1.8E-01	excluded
SENP7	65952	ES	2	1	3	0.02	1.8E-01	included
SENP7	65949	ES	5:06	4	7	0.01	1.8E-01	included
TP53INP1	84604	ES	5	3.1	6.1	0.02	1.8E-01	included
RPAIN	38687	ES	5	3	6.1	-0.01	1.8E-01	excluded
TMCO4	916	ES	11	10	12	0.01	1.8E-01	included
REPS1	77957	ES	5:06:07	4	8	0.00	1.8E-01	included
ANKRD54	62165	ES	5.1:5.2	4	6	0.00	1.8E-01	excluded
SH3GLB2	87812	ES	7	6	8	0.00	1.8E-01	included
SNRPN	29705	ES	8	7	9	0.00	1.8E-01	excluded
CDH1	37248	ES	9.1	8	10	0.00	1.8E-01	excluded
LMBR1	82499	ES	2.1	1	4.1	0.00	1.8E-01	excluded
TCP1	78328	ES	2	1	3.2	0.00	1.8E-01	excluded
DNAL1	28304	ES	4	3	5	0.01	1.8E-01	included
FCHO2	72483	ES	7	6	8	0.00	1.8E-01	included
DUSP16	20510	ES	4	3	5	-0.01	1.8E-01	excluded
TUSC2	65014	ES	2.2:2.3	1	3.1	0.00	1.8E-01	included
PFDN5	22010	ES	2:03	1	5	0.00	1.8E-01	included
PPA2	70226	ES	2:4:7:8:9	1	11	-0.02	1.8E-01	excluded
CRELD2	390167	ES	8:09	7	10	0.00	1.8E-01	excluded
ARHGAP9	22586	ES	21.1:21.2	20	22	-0.02	1.8E-01	excluded
MANBA	70114	ES	5	4	6	0.00	1.8E-01	included
ARL13B	65700	ES	03:02.2	1	5.1	-0.03	1.8E-01	excluded
C1orf85	8223	ES	5.1:5.2	4	6.1	-0.02	1.8E-01	excluded
CREM	11251	ES	11	10.1	15	0.02	1.8E-01	included
ARHGEF7	26290	ES	6:07	2.3	8	-0.01	1.8E-01	excluded
CHN2	79085	ES	16	15	17	0.00	1.8E-01	excluded
AP3M1	12256	ES	2	1	3	0.02	1.8E-01	included
CHP1	30071	ES	7	6	8	0.00	1.8E-01	excluded
UBE2J2	47	ES	6.1:6.2	5	7	0.00	1.8E-01	included
PCDH18	70602	ES	1.2:1.3:1.4:2.1	1.1	2.2	-0.03	1.8E-01	excluded
TMEM150A	54306	ES	6	5	7	-0.01	1.8E-01	excluded
MOK	29385	ES	8	7	10.1	0.01	1.8E-01	included
ENDOV	44078	ES	2.4:6.2	1	7	0.01	1.8E-01	included
PMM2	33934	ES	6:07	3	8	0.00	1.8E-01	excluded
MLH3	28470	ES	7	6	8	0.02	1.8E-01	included
NCSTN	8463	ES	7	6	8	0.00	1.8E-01	included
RPAIN	38694	ES	4:05	3	7	-0.02	1.8E-01	excluded
DUOXA1	30392	ES	1.2:2.1	1.1	2.2	0.04	1.8E-01	included
ARFGAP1	60109	ES	13:14.1	12	14.2	-0.02	1.8E-01	excluded
KANSL1	42014	ES	11	10	12	0.01	1.8E-01	included
TSPAN7	88819	ES	2:03	1	7	0.00	1.8E-01	included
ZNF140	25333	ES	04:05.2	2	5.3	0.00	1.8E-01	excluded
SLC25A40	80342	ES	10.2	9	12	0.00	1.8E-01	included
FANK1	13445	ES	9	8	10	0.01	1.8E-01	included

LPHN2	3569	ES	27	26	31	0.00	1.8E-01	excluded
KANK2	47640	ES	2.2	1	3	-0.02	1.8E-01	excluded
FANK1	13448	ES	6.3	6.1	7.1	0.01	1.8E-01	included
RBM14-RBM4	17102	ES	02:03.1	1	4	0.01	1.8E-01	included
PCDH1	73834	ES	4.1	3.3	5	0.00	1.8E-01	included
SCMH1	2056	ES	6	5	9	-0.04	1.8E-01	excluded
VMP1	42803	ES	3	2	4	0.00	1.8E-01	included
HNRNPA1	212642	ES	3:4:5:6.1:9.2:10:11.1:	2	11.3	0.02	1.8E-01	included
ERO1L	27545	ES	15	14	16	0.00	1.8E-01	included
TMEM55A	84423	ES	3:04:05	1	6	0.00	1.8E-01	included
RAB30	18045	ES	4	3.2	5.1	-0.01	1.8E-01	excluded
SDHD	18742	ES	2	1	3.1	0.00	1.8E-01	included
CSAD	21964	ES	8:9:10.1:10.2:10.3:10.	7.2	11	0.02	1.8E-01	included
PIGT	59567	ES	2.1:2.2:3:4:5.2:6	1	7	0.00	1.8E-01	excluded
TYMP	96246	ES	2.1:2.2:2.3:3:4	1	5	-0.01	1.8E-01	excluded
LIPC	30897	ES	2	1	3.2	0.02	1.8E-01	included
EIF5A	38912	ES	4.2	2	5.2	0.00	1.8E-01	excluded
PHTF1	4282	ES	4	3	5	0.00	1.8E-01	excluded
TMEM5	22852	ES	3	2	4	-0.01	1.8E-01	excluded
CAMK2G	12250	ES	4:5:6:7:8:9:10	3	11	0.00	1.8E-01	excluded
PRKAA1	71869	ES	4	3	5	-0.01	1.8E-01	excluded
TINF2	26934	ES	4:05	3	6.1	0.00	1.8E-01	included
PHC3	67606	ES	6.1	5.2	7	0.01	1.8E-01	included
SHMT2	22555	ES	4:05	1	6.1	0.00	1.8E-01	included
AK2	1653	ES	5	4	7	0.00	1.8E-01	included
ATR	67117	ES	6	5	7	0.01	1.8E-01	included
HM13	58890	ES	12.1:12.2	11	13	0.00	1.8E-01	excluded
ST3GAL6	65796	ES	12	9	13	-0.01	1.8E-01	excluded
ANGEL2	9780	ES	3	1	5	0.01	1.8E-01	included
MED15	61167	ES	12:13:14	11	15	0.00	1.8E-01	included
BCAT2	50816	ES	3	1	4	-0.01	1.8E-01	excluded
SIRT5	75397	ES	5	4	6	-0.02	1.8E-01	excluded
HPN	49021	ES	3:04	2.3	5	0.00	1.8E-01	excluded
ELN	80057	ES	3	2.1	4	-0.01	1.8E-01	excluded
WAC	11103	ES	12	11.2	13	-0.01	1.8E-01	excluded
SMARCB1	61328	ES	4.1	3	5	0.00	1.8E-01	included
GNPDA1	73861	ES	3.1:3.2	2.6	4	0.00	1.8E-01	included
EPB41L2	77563	ES	17:18:20.1:20.2:21	14	22	0.01	1.8E-01	included
CRELD2	390169	ES	8	7	10	0.01	1.8E-01	included
ABI2	57025	ES	7	5.3	8	-0.01	1.8E-01	excluded
ZSWIM7	39397	ES	5.2	4	6	0.00	1.8E-01	included
MEF2C	72758	ES	6.1:6.2	5	8	0.00	1.8E-01	included
DNAJC7	40975	ES	3	1	4	0.00	1.8E-01	included
KDM4C	85835	ES	4	3	5.1	0.00	1.8E-01	excluded
TMBIM1	57473	ES	4	1	5	0.00	1.8E-01	included
TEX264	65107	ES	2	1.1	3	0.00	1.8E-01	excluded
STIP1	16571	ES	5:6.1:7:8:9:10:11:12:1	4.2	14.2	0.00	1.8E-01	included
SNX24	73129	ES	2	1	3	0.01	1.8E-01	included
APCDD1	44627	ES	2:3.1:3.2	1	4.1	0.00	1.8E-01	included
RMDN1	84378	ES	5	4	7	0.00	1.8E-01	excluded
PILRB	80934	ES	11	9	12.2	0.00	1.8E-01	included
ANKRD33B	71570	ES	3	2	4	-0.01	1.8E-01	excluded
CLPTM1L	71484	ES	8.2:9	8.1	10	0.00	1.8E-01	excluded
PLEKHA4	50823	ES	9	8	10	-0.01	1.8E-01	excluded
OSBPL1A	44896	ES	3.1:3.2:4	2	6	0.00	1.8E-01	excluded
APLP2	19480	ES	6.1:6.2:7:8:9	5.2	10	0.00	1.8E-01	included
SLC35F6	52913	ES	2	1	3	0.00	1.8E-01	excluded
NAP1L4	13939	ES	2	1	3	0.01	1.8E-01	included
HYOU1	19089	ES	1.2:2	1.1	3	-0.01	1.8E-01	excluded
ZNF589	64578	ES	3:04	2	5	0.04	1.8E-01	included
POMT1	87936	ES	17	16	18	0.00	1.8E-01	excluded
SNX13	78884	ES	3	2	4	0.00	1.8E-01	excluded

TEX264	65104	ES	1.2:1.3:3	1.1	4	-0.02	1.8E-01	excluded
TBC1D19	68990	ES	2:03:04	1	5	-0.01	1.8E-01	excluded
RBMXL1	3705	ES	2	1	3	-0.03	1.8E-01	excluded
RAB35	24721	ES	2	1	3	-0.01	1.8E-01	excluded
TSPAN14	12368	ES	9	8	10	0.00	1.8E-01	excluded
TTC23	32626	ES	2.2:3:4	2.1	6	0.02	1.8E-01	included
RAB1A	53792	ES	5:06	4	7	0.00	1.8E-01	included
EIF2A	67281	ES	6:07	5.2	8	0.00	1.8E-01	excluded
KIAA0753	38764	ES	2.2	1	3	0.01	1.8E-01	included
CORO7	33670	ES	18.4	18.2	19	0.02	1.8E-01	included
MUTYH	2650	ES	6.2:6.3:6.4:6.5:7:8:9	5	10	0.01	1.8E-01	included
COPE	48517	ES	4:06:07	3	8	0.00	1.8E-01	included
MICAL2	14427	ES	23	18	25	-0.03	1.9E-01	excluded
MRPS18C	69794	ES	4	2	5	0.02	1.9E-01	included
CD44	15265	ES	3.2:4:5:15:16.1	3.1	16.2	0.00	1.9E-01	included
HAUS2	30189	ES	4	3	5.1	0.02	1.9E-01	included
ELP2	45228	ES	5	4.2	7	-0.01	1.9E-01	excluded
PPIA	79568	ES	2.1:2.2	1	3	0.00	1.9E-01	included
C3orf17	66139	ES	7	6.2	8	-0.01	1.9E-01	excluded
GNB2L1	75122	ES	1.2:2.1:2.2	1.1	2.3	0.00	1.9E-01	excluded
CXorf56	89953	ES	3	2	4	0.00	1.9E-01	included
PGRMC1	89951	ES	2	1	3	0.00	1.9E-01	excluded
CSNK2A1	58461	ES	2:03	1	4	-0.03	1.9E-01	excluded
MICAL1	77199	ES	9	8	10	0.00	1.9E-01	excluded
NBEAL2	64488	ES	24	23	25	-0.01	1.9E-01	excluded
NASP	2751	ES	4:05	3	6	0.00	1.9E-01	excluded
AGTPBP1	86733	ES	9:10	8	11.1	0.00	1.9E-01	included
ANK3	11849	ES	42	41	43	-0.01	1.9E-01	excluded
LSG1	68168	ES	6	5	7	0.00	1.9E-01	excluded
PET112	70845	ES	6	5	7	0.00	1.9E-01	excluded
NLRP3	10522	ES	7	6	8	-0.01	1.9E-01	excluded
SLCO2B1	17823	ES	6:07	5	8	0.00	1.9E-01	included
HHLA3	3407	ES	1.2:1.3:2.2	1.1	3	0.00	1.9E-01	included
CRYZ	3468	ES	5	4	6	0.00	1.9E-01	included
MGAT4C	23591	ES	8:9:10:11.1	6.2	11.2	-0.02	1.9E-01	excluded
CTU2	38018	ES	14	13	15	0.00	1.9E-01	included
ORMDL1	56539	ES	2.2	1	2.4	0.02	1.9E-01	included
DIS3	26044	ES	14	13	15	0.00	1.9E-01	excluded
TNS1	57434	ES	23	22	25	-0.01	1.9E-01	excluded
BCS1L	57553	ES	1.4	1.1	2	-0.02	1.9E-01	excluded
BRF1	29617	ES	12.3:13.2:14.1:14.2:15 .2:16:17:18:19:20.1	12.2	20.2	0.00	1.9E-01	included
NOX4	18243	ES	10:11:12:13	8	14	0.00	1.9E-01	excluded
NME4	32881	ES	3.2	2	5	0.00	1.9E-01	excluded
LETMD1	100275	ES	8.2	7	9	-0.01	1.9E-01	excluded
RBM14	17085	ES	3	1	4.1	-0.02	1.9E-01	excluded
NBPF3	970	ES	3:04:05	2	6	-0.01	1.9E-01	excluded
COPRS	40167	ES	3	1	5	0.00	1.9E-01	included
ST6GALNAC6	87684	ES	2.2	1	4	0.02	1.9E-01	included
DCAF6	91281	ES	13.1:14	10	15	0.01	1.9E-01	included
SLC47A1	39740	ES	8	7	9	0.00	1.9E-01	included
ME3	18199	ES	10	8	11	-0.03	1.9E-01	excluded
MAP4	64567	ES	4	3	5	0.00	1.9E-01	included
HAUS7	90439	ES	06:07.1	5	8	0.00	1.9E-01	included
ENDOV	44077	ES	2.4:5:6.2	1	7	0.01	1.9E-01	included
TUSC3	82774	ES	7	6	9	0.00	1.9E-01	included
SP4	78912	ES	3	2	4	0.01	1.9E-01	included
MYO5C	30654	ES	8	7	9	-0.01	1.9E-01	excluded
PIGL	39430	ES	2	1	5	0.01	1.9E-01	included
UAP1	8750	ES	9.1:9.2	8	10	-0.01	1.9E-01	excluded
RBM23	26657	ES	2:03	1	4	0.00	1.9E-01	excluded
TMEM205	47660	ES	2.3:2.5:2.6	2.2	3	0.03	1.9E-01	included

GIT2	24384	ES	17.1:17.2	15	18.1	0.01	1.9E-01	included
MYD88	64035	ES	02:03.1	1	4	0.00	1.9E-01	included
CMTM3	36818	ES	1.3:1.4:2.2:2.3	1.2	5	0.01	1.9E-01	included
KIAA0586	27718	ES	17	16	18	0.01	1.9E-01	included
SRRM1	1133	ES	4:05	3	6	-0.02	1.9E-01	excluded
PREB	52944	ES	6	5	7	0.00	1.9E-01	included
NDUFA5	81628	ES	4	3	5	0.00	1.9E-01	excluded
SLC37A3	81988	ES	09:10.1	8	10.2	-0.03	1.9E-01	excluded
CCDC61	50540	ES	4	3.2	5	0.00	1.9E-01	included
SACM1L	64407	ES	4	3	5	0.00	1.9E-01	excluded
PPP3CB	12155	ES	16	15.1	17	0.02	1.9E-01	included
SYNRG	40527	ES	20	19	21	0.00	1.9E-01	included
NSUN4	2794	ES	2.2	1	3	-0.02	1.9E-01	excluded
MADD	15717	ES	28	27	29	0.00	1.9E-01	included
SEMA6C	7561	ES	9	8	10	0.01	1.9E-01	included
ATP5A1	45372	ES	6.2:7.1:7.2:8:9:10:11.	6.1	11.2	0.00	1.9E-01	included
YIF1A	17010	ES	2:03	1	4	0.00	1.9E-01	excluded
PORCN	88987	ES	3.1:3.2	2.2	4	0.00	1.9E-01	included
MRPL4	47486	ES	4	3	5	0.00	1.9E-01	included
C11orf80	17125	ES	6	5.1	7	-0.01	1.9E-01	excluded
RBPJ	68983	ES	7	6	8	0.00	1.9E-01	excluded
PML	31639	ES	5:6.1:6.2:6.4:6.5:6.6:6.	4	6.8	0.01	1.9E-01	included
ICA1L	56958	ES	6.1:6.2:7	5.2	8.1	-0.01	1.9E-01	excluded
SLC25A16	11952	ES	2.1:2.2	1	3	0.04	1.9E-01	included
C12orf23	24182	ES	3.2	2.2	4.2	0.01	1.9E-01	included
FAM86A	33885	ES	3.2:5.1:5.2	3.1	6	-0.04	1.9E-01	excluded
NQO2	75161	ES	6	1	7	0.01	1.9E-01	included
TP53BP2	9929	ES	13	12	14	0.00	1.9E-01	excluded
CAV1	81533	ES	1.2:1.3:2.3	1.1	2.4	0.01	1.9E-01	included
TRIP12	57850	ES	4.2:5:6:8.1:8.2:9:10:1<br>1:12:13:14:15:16:17:1<br>8:19.1:19.2:20:21:22:	4.1	23.2	0.00	1.9E-01	excluded
USP10	37862	ES	5	4	6	0.00	1.9E-01	included
PML	31656	ES	6.1:6.4:6.7	4	6.8	0.00	1.9E-01	included
NAP1L1	23492	ES	2:03:04	1	5	0.00	1.9E-01	included
BTN3A2	75623	ES	3.1:3.2	2.2	4.2	-0.01	1.9E-01	excluded
MBNL2	26142	ES	10	8	11	0.02	1.9E-01	included
GOLGA8B	100047	ES	2	1	3	-0.03	1.9E-01	excluded
PROSER1	25700	ES	2	1	3	-0.02	1.9E-01	excluded
SCARB2	69595	ES	3:4:5:6	2	7	-0.01	1.9E-01	excluded
ZNF415	51688	ES	6.1	4	9	-0.02	1.9E-01	excluded
PCMT1	78112	ES	4	2	5	0.00	1.9E-01	excluded
NPRL3	32827	ES	1.4:2:4	1.3	6	-0.01	1.9E-01	excluded
FAM156B	89172	ES	3.1:3.2	2.5	4	0.00	1.9E-01	excluded
DDX3X	88854	ES	6.2:7:8:9:10:11:12:13:<br>14:15:16:17	6.1	18	0.00	1.9E-01	excluded
FOPNL	34209	ES	2	1	4	-0.01	1.9E-01	excluded
RDX	18637	ES	5	4	6	0.00	1.9E-01	excluded
KIAA0368	87206	ES	46:47:48:49:50.1	45	50.2	0.00	1.9E-01	included
PRPF39	27396	ES	10	9	11	0.02	1.9E-01	included
RAB5C	41013	ES	3	1	4	0.00	1.9E-01	excluded
P2RX4	24841	ES	6	5	7	0.00	1.9E-01	excluded
DYNLL1	24766	ES	2:03	1	4.4	-0.03	1.9E-01	excluded
TINAGL1	1479	ES	3	2	5.1	0.00	1.9E-01	excluded
C5orf45	74949	ES	5.2:6:7.1:7.2:7.3	5.1	7.4	0.00	1.9E-01	included
MARVELD2	72373	ES	3	2.4	4	-0.01	1.9E-01	excluded
PSMA4	32106	ES	7.2:8:9	6	10	0.00	1.9E-01	included
PHB2	20042	ES	7.1:8:9.2	6.2	9.3	0.00	1.9E-01	included
LPHN2	3564	ES	29	27	31	0.02	1.9E-01	included
PTPN2	44720	ES	12.1	11	13	0.00	1.9E-01	included
MED15	61175	ES	5:07:08	3.1	9	0.00	1.9E-01	included
CENPN	37743	ES	7	6	9	-0.02	1.9E-01	excluded

STAU2	84164	ES	5:07	4	8	0.01	1.9E-01	included
EOGT	65562	ES	9:10	8	11	0.00	1.9E-01	excluded
NCBP1	87021	ES	4	3	5	0.00	1.9E-01	included
MVK	24343	ES	2.2:3:4:5:6:7:8.1:9.1	1	9.2	-0.01	1.9E-01	excluded
CALU	81713	ES	3:04	1	6	0.00	1.9E-01	included
ZNF274	52417	ES	2:03:04	1	5	0.00	1.9E-01	excluded
BTN2A2	75651	ES	3.1:3.2	2	4	0.01	1.9E-01	included
PAX8	533933	ES	10	8	11	-0.01	1.9E-01	excluded
SS18	44967	ES	4:05	3	9	0.00	1.9E-01	excluded
LMAN2L	54576	ES	2:03	1	4	-0.03	1.9E-01	excluded
MST4	90124	ES	3	2	5	-0.01	1.9E-01	excluded
MAP2K3	39820	ES	3	1	5	0.00	1.9E-01	included
RPL37A	57420	ES	3	2.4	4	0.00	1.9E-01	included
ISYNA1	48436	ES	4	3.2	5	0.00	1.9E-01	included
NPEPPS	42081	ES	8	7.2	9.1	0.00	1.9E-01	excluded
CCNDBP1	30221	ES	5	4	6	0.00	1.9E-01	included
MANBAL	59344	ES	3	1	4.2	0.01	1.9E-01	included
NAA50	66213	ES	5	4.3	6	0.00	1.9E-01	excluded
RGS4	8767	ES	6.2:7.1	6.1	7.2	-0.01	1.9E-01	excluded
RNF146	77453	ES	5.1	2	6	-0.02	1.9E-01	excluded
NMU	69333	ES	5:06	4	7	0.00	1.9E-01	included
GNG7	46616	ES	6	5	7	0.00	1.9E-01	excluded
CMYA5	99732	ES	5	4	6	0.02	1.9E-01	included
DAPK2	31079	ES	13.3:15.2	13.1	15.3	0.00	1.9E-01	excluded
ZNF512	53024	ES	6	5	7	0.01	1.9E-01	included
SF3A1	61727	ES	3.1:3.2:4.1:4.2	2	5	0.00	1.9E-01	excluded
CCDC82	18411	ES	2	1	3.2	-0.01	1.9E-01	excluded
SNURF	29712	ES	14	13	15	0.02	1.9E-01	included
MED23	77617	ES	16:17	15	18	0.00	1.9E-01	included
DNAJC10	56465	ES	10:11	9	13	0.00	2.0E-01	included
DRG2	39553	ES	5.1	4	6.1	0.00	2.0E-01	included
ALDH16A1	50937	ES	7	6	8	-0.01	2.0E-01	excluded
ELMOD3	54250	ES	2.1:2.3:2.4:2.5:2.6:2.7:	1	2.9	-0.03	2.0E-01	excluded
PTK2	85309	ES	25	24	26	0.00	2.0E-01	excluded
KIFC3	36615	ES	7	5	13	0.03	2.0E-01	included
TRIM16L	39638	ES	4:05	3	6.2	-0.01	2.0E-01	excluded
STAU2	84165	ES	7	4	8	0.00	2.0E-01	included
COQ6	28347	ES	8:9:10.1:10.2:10.3:11:	6	14.1	0.02	2.0E-01	included
DIS3L2	57993	ES	7	6	8.1	-0.01	2.0E-01	excluded
FRG1	71414	ES	5:06	4	7	0.01	2.0E-01	included
C19orf40	48918	ES	2:3.1:3.2	1.1	4	-0.01	2.0E-01	excluded
TACC2	13339	ES	20:21	19	22	0.01	2.0E-01	included
CDADC1	25878	ES	4.1:4.2:5	3	6	0.02	2.0E-01	included
RRAS2	14467	ES	6	2	7.2	0.00	2.0E-01	excluded
SNRPN	124116	ES	10.3:10.4	9	11	0.03	2.0E-01	included
HERC3	69901	ES	14	13	15	0.00	2.0E-01	included
LY6E	85387	ES	03:05.1	2	5.2	0.00	2.0E-01	included
PLEKHA5	20657	ES	16:17:19:20	15	21	0.00	2.0E-01	excluded
CORO1B	387278	ES	4:5.1:5.2:5.3:5.5:6	3	7	0.02	2.0E-01	included
ATXN7L1	81314	ES	2	1	3	0.01	2.0E-01	included
AHNAK	16349	ES	6	4	7	-0.01	2.0E-01	excluded
DHRS4	26783	ES	5:06	3	7.1	0.01	2.0E-01	included
SENP7	65951	ES	5	4	7	0.03	2.0E-01	included
AFMID	43807	ES	7:8:9:10	6	12	-0.01	2.0E-01	excluded
LIMA1	21694	ES	10	9.2	11.1	0.00	2.0E-01	included
MMS19	12724	ES	2	1.3	3	0.00	2.0E-01	included
SLC44A2	47564	ES	6:7:8:9:10:11	5	12	0.00	2.0E-01	included
EML4	53353	ES	19:20	18	21	0.00	2.0E-01	included
ERICH1	82555	ES	2:03	1	4	0.00	2.0E-01	excluded
IFNGR2	60410	ES	4	1	5	0.00	2.0E-01	included
WSB2	24695	ES	6	5	7.1	0.00	2.0E-01	excluded
KRBOX4	88892	ES	5.2:6	5.1	8.1	0.02	2.0E-01	included

AASDH	69345	ES	12:13:14	11.1	15	-0.01	2.0E-01	excluded
ENOSF1	44471	ES	6:07:08	5	9.2	-0.02	2.0E-01	excluded
ERBB2	40684	ES	9:10:11:12:13	8.1	14	0.00	2.0E-01	included
VAV2	88084	ES	16	15	17	0.00	2.0E-01	included
CDK10	38125	ES	2.2:4	2.1	5	-0.03	2.0E-01	excluded
TP53I3	52810	ES	4	3	5	0.01	2.0E-01	included
ABCD3	3814	ES	3	1	4	0.00	2.0E-01	included
ING3	81587	ES	5	4.1	6	-0.02	2.0E-01	excluded
EEF1D	85459	ES	3	1	8.2	0.00	2.0E-01	included
MARCH6	71565	ES	4:05	3	6	0.00	2.0E-01	included
GMFB	27592	ES	3	2	4.1	0.00	2.0E-01	excluded
YY1AP1	8108	ES	7	6	8	0.00	2.0E-01	excluded
IBTK	76836	ES	12	11	13	0.00	2.0E-01	included
TRIT1	1934	ES	2:3.1:6.1:6.2:7.1:7.2:8	1	10	-0.01	2.0E-01	excluded
PLSCR4	67142	ES	7:08	6	9	0.00	2.0E-01	excluded
ACAD8	19556	ES	2	1	3	0.00	2.0E-01	excluded
DNAJB6	82513	ES	6	5	7	0.00	2.0E-01	excluded
POLM	79457	ES	8	6.2	9.1	0.01	2.0E-01	included
C16orf62	34326	ES	10	8	11.1	0.00	2.0E-01	excluded
PPP4R2	65626	ES	5	2	6	0.00	2.0E-01	excluded
PLEKHA5	20663	ES	9	8	10	0.00	2.0E-01	included
DUOXA1	30398	ES	1.2:1.3:2.2:3	1.1	5	0.03	2.0E-01	included
GRB2	43442	ES	5	4	6	0.00	2.0E-01	included
ABI2	57022	ES	9.2	8	10	0.00	2.0E-01	excluded
CRELD1	63289	ES	10	9	11	-0.01	2.0E-01	excluded
EIF4G1	67884	ES	4	3.2	5	0.00	2.0E-01	excluded
GPR124	83355	ES	12:13:14	11	15	0.00	2.0E-01	excluded
C19orf48	51223	ES	3	1	4.2	-0.02	2.0E-01	excluded
DMKN	49196	ES	7	6.4	11	-0.01	2.0E-01	excluded
ING4	19914	ES	3:04	2	5.1	0.03	2.0E-01	included
CEP70	67006	ES	3.2:3.3	2	4	0.01	2.0E-01	included
TOP3B	61272	ES	5:07	4	8	0.00	2.0E-01	excluded
OSGEPL1	56531	ES	7	6	8	0.00	2.0E-01	excluded
HNRNPR	1046	ES	5	4	6	0.00	2.0E-01	included
EDEM2	59069	ES	10:11	9	13	0.00	2.0E-01	excluded
ATXN3	28974	ES	2	1	3.1	0.01	2.0E-01	included
DMGDH	72620	ES	3.1:3.2:4:5:6:7	2	8	-0.01	2.0E-01	excluded
ADHFE1	84005	ES	07:08.1	6	9	0.00	2.0E-01	included
TGS1	83879	ES	11:12.1	10	12.2	0.00	2.0E-01	included
LGALS3BP	43955	ES	3.1:3.2:4.1:4.2:5.2:5.3	2.2	5.4	0.00	2.0E-01	excluded
PCSK4	46516	ES	6	5	7.1	-0.03	2.0E-01	excluded
ANAPC15	17574	ES	3.2:3.3:4.2	1.2	5	0.00	2.0E-01	excluded
FAM98C	49643	ES	5	4	6	0.00	2.0E-01	included
ERGIC1	74566	ES	5	3	6.1	0.00	2.0E-01	excluded
JMJD6	43621	ES	5.1:5.2	4	6	-0.02	2.0E-01	excluded
ABLIM1	13205	ES	23	22	25.2	0.00	2.0E-01	excluded
PHYKPL	74860	ES	2	1	3	-0.03	2.0E-01	excluded
HNRNPA1	120409	ES	7.1:7.2	6.2	8	-0.01	2.0E-01	excluded
REEP5	72994	ES	4	3	5	0.00	2.0E-01	excluded
MLST8	33220	ES	10.1	8.2	11.1	0.00	2.0E-01	excluded
MUM1	46455	ES	13.3	13.1	14	0.00	2.0E-01	excluded
LMO3	20623	ES	9.2:11.2	9.1	12	0.01	2.0E-01	included
TPD52	84276	ES	9:10	8	11	0.00	2.0E-01	excluded
ZNF691	2137	ES	2.1	1	4	0.03	2.0E-01	included
HHAT	9695	ES	7:08	6	9	0.00	2.0E-01	excluded
TTC8	28787	ES	6	2	7	-0.03	2.0E-01	excluded
RRM1	14037	ES	4	2	5	0.00	2.0E-01	included
TCTN1	24483	ES	2	1.1	4	-0.01	2.0E-01	excluded
LRIF1	4130	ES	2.2	1	3	-0.02	2.0E-01	excluded
HDAC5	41774	ES	16:17	15	18	0.00	2.0E-01	included
PDCD5	48889	ES	4.1:4.2:5.1:5.2:5.3	3	5.5	0.01	2.0E-01	included
INTS12	70249	ES	4	1	5	-0.02	2.0E-01	excluded

TMEM230	58637	ES	3	1.2	4	0.02	2.0E-01	included
SEPHS1	10793	ES	9	8	10	0.00	2.0E-01	excluded
FAM222B	39986	ES	8	7.2	9.1	0.00	2.0E-01	excluded
IL17RE	63245	ES	9	8	10	0.00	2.0E-01	excluded
APTX	86081	ES	6.3:7.2:7.3	6.2	7.4	0.02	2.0E-01	included
EEF1A1	485240	ES	1.4:3:4:5	1.3	6	0.00	2.0E-01	excluded
IL6ST	72084	ES	4.2:5:6	4.1	7	0.00	2.0E-01	included
STK25	58387	ES	4	3.2	5	0.00	2.0E-01	excluded
STAU2	84173	ES	3:04	2	7	-0.01	2.0E-01	excluded
MDH2	117319	ES	4	1	5	-0.03	2.0E-01	excluded
HHLA3	3404	ES	1.3:2.2	1.2	3	0.00	2.0E-01	included
GPS1	44289	ES	1.4:1.5	1.1	2.3	0.00	2.0E-01	excluded
C3orf14	65497	ES	2	1.1	3	0.01	2.0E-01	included
DHRS4	26782	ES	4:05:06	3	7.1	0.02	2.0E-01	included
ZNF707	85478	ES	10	8.2	11	-0.02	2.0E-01	excluded
STAT5A	41033	ES	5	4	6	0.01	2.0E-01	included
ATP6V0A1	41044	ES	22	21.2	23	0.00	2.0E-01	excluded
MCM8	58659	ES	13	11	14	-0.01	2.0E-01	excluded
ACY1	390894	ES	8.1:8.2:9:10:11:12	7.2	15	0.02	2.0E-01	included
NLN	72251	ES	3	1	4	0.00	2.0E-01	included
EIF4G3	952	ES	18.1	17	19	-0.01	2.0E-01	excluded
C14orf159	28859	ES	5.2:6	4	7	0.04	2.0E-01	included
UBE2F	58170	ES	6	5	7	0.00	2.0E-01	excluded
SUSD4	9918	ES	4	1.3	6	0.00	2.0E-01	included
SZRD1	807	ES	03:04.1	1	4.2	0.00	2.0E-01	excluded
EXD2	28130	ES	5	4	6	-0.01	2.0E-01	excluded
ANXA6	74150	ES	6.3:7.1:7.2:8:9:10:11:<br>12:13.1:13.2:14:15.1	6.2	15.2	0.00	2.0E-01	included
PFKFB3	10701	ES	15	14	17	0.00	2.0E-01	included
HECTD2	12518	ES	11	10	12	0.01	2.0E-01	included
ZNF540	49543	ES	6	5	7	-0.01	2.0E-01	excluded
CPNE1	59201	ES	3	1.1	5	0.00	2.0E-01	excluded
PHF20L1	85195	ES	12	11	13	0.00	2.0E-01	included
PDE4A	47523	ES	14:15:16	13	17	0.00	2.0E-01	excluded
PDPK1	33289	ES	13:14	12	15	0.00	2.0E-01	excluded
ZNF410	28327	ES	15	13	16	-0.01	2.0E-01	excluded
AKAP8L	48085	ES	4.1	3	4.4	0.01	2.0E-01	included
CTBP1	68464	ES	2.2	1	3.1	0.00	2.0E-01	excluded
UQCC1	59128	ES	3:6.1:6.2:7.1:7.2:8:9	1	10.1	0.00	2.0E-01	excluded
BMS1	99441	ES	18	17	19	0.01	2.0E-01	included
THOC1	44447	ES	4	3	6	0.00	2.0E-01	included
SEC22C	64292	ES	10	9	12	0.00	2.0E-01	excluded
NUP85	43387	ES	7	6	8	0.00	2.0E-01	included
VWA9	31206	ES	1.3:2.1	1.2	2.2	0.03	2.0E-01	included
TNFRSF10C	83066	ES	5	4.2	6	0.00	2.0E-01	included
COX14	21651	ES	2	1.1	4	0.00	2.0E-01	excluded
CALCOCO2	42234	ES	2:03	1	6	0.00	2.0E-01	included
MAPKAPK5	24543	ES	11	10	12	0.00	2.0E-01	excluded
TLE2	205979	ES	17:18:19:20	16.1	21	0.02	2.0E-01	included
SLMAP	65394	ES	25	24	26	-0.03	2.0E-01	excluded
YPEL3	36072	ES	3.2:4.1	3.1	5	0.00	2.0E-01	excluded
LY96	84219	ES	2	1	3	0.01	2.0E-01	included
MBNL1	67318	ES	10	9	11	0.02	2.0E-01	included
DDX56	79535	ES	7	6	8.1	0.00	2.0E-01	included
GUF1	69147	ES	9	8	10	-0.02	2.0E-01	excluded
RBFOX2	61987	ES	6	5.2	7	0.00	2.1E-01	excluded
PPP1CB	53073	ES	3	2	4	0.00	2.1E-01	excluded
ST6GAL1	68069	ES	5	4	6	0.00	2.1E-01	excluded
TRIM28	52480	ES	2:03	1	4	0.00	2.1E-01	included
ZNF799	47799	ES	3	1	4	-0.02	2.1E-01	excluded
SSX2IP	3617	ES	16.3	16.1	17	0.00	2.1E-01	excluded
CTNND1	16002	ES	3:4.2:4.3	2.1	5	-0.01	2.1E-01	excluded

TNPO1	72479	ES	6:07	5	8	0.00	2.1E-01	included
DNAJB14	70086	ES	4	3	5	0.00	2.1E-01	excluded
TRMT10B	86432	ES	5	4.1	6.1	0.00	2.1E-01	included
SEC23A	27353	ES	2	1	4.1	0.00	2.1E-01	included
KRIT1	80426	ES	10	9	11	0.00	2.1E-01	excluded
CMC2	37728	ES	9	5	12	-0.01	2.1E-01	excluded
FOXP1	65605	ES	26:28:00	25	29	0.00	2.1E-01	excluded
SMIM7	48197	ES	3:05	2	7.1	0.00	2.1E-01	excluded
CAMK2D	70417	ES	16	14.2	18	0.00	2.1E-01	excluded
WIBG	22292	ES	4	2	5	-0.02	2.1E-01	excluded
CSRP2	23512	ES	4	3	5	0.00	2.1E-01	excluded
ZNF846	47402	ES	7	6.1	8.1	-0.01	2.1E-01	excluded
WWP2	37318	ES	7	6	9	0.00	2.1E-01	included
ADD1	68611	ES	5	4	6	0.00	2.1E-01	excluded
FBXO7	61935	ES	3.2	1	4	-0.01	2.1E-01	excluded
WDR6	64805	ES	3.2	1	4.2	-0.02	2.1E-01	excluded
CDC27	42057	ES	3	2	4	0.00	2.1E-01	included
ZBTB49	68663	ES	4	3.2	5	-0.03	2.1E-01	excluded
HBS1L	77800	ES	4	3	7.1	0.00	2.1E-01	excluded
FLOT2	40014	ES	3	2	6	0.02	2.1E-01	included
PPAP2A	72037	ES	2:03	1	4	0.03	2.1E-01	included
POLR1E	86405	ES	5	4	6	0.00	2.1E-01	excluded
RPF2	77253	ES	3.2	2	4	0.00	2.1E-01	included
PGM2	69007	ES	8	7	9	0.00	2.1E-01	included
TBC1D31	85034	ES	19	18	20	-0.04	2.1E-01	excluded
SEPT11	69623	ES	2:03	1	4	-0.01	2.1E-01	excluded
ZNF211	52320	ES	2.2:3.1:3.2:4	2.1	5	0.01	2.1E-01	included
MLLT10	10973	ES	16	15	17	0.02	2.1E-01	included
DNAJA3	33723	ES	2:3:4:5.1	1	5.2	0.00	2.1E-01	excluded
TNPO1	72480	ES	5	4	6	0.00	2.1E-01	excluded
CD22	49112	ES	12	11	13	0.01	2.1E-01	included
KLHL20	9022	ES	3	2	4	0.00	2.1E-01	excluded
ALDH2	24538	ES	2	1	3	-0.01	2.1E-01	excluded
KCTD10	24299	ES	3	2.2	4.4	0.00	2.1E-01	excluded
LY6E	85392	ES	3	2	5.1	0.00	2.1E-01	included
DNAJC24	14813	ES	4	2	7	-0.01	2.1E-01	excluded
SRPK2	81284	ES	20	19	21.1	-0.02	2.1E-01	excluded
SDHD	18736	ES	3.2:4	3.1	5	0.01	2.1E-01	included
NAIP	72433	ES	14	13	15	-0.01	2.1E-01	excluded
VMP1	191195	ES	10	9	11	-0.01	2.1E-01	excluded
AP2S1	50604	ES	3.2:4.1	1.1	4.2	0.02	2.1E-01	included
SLC25A39	41841	ES	2.2:3:4:5.1:5.2	1	6	0.00	2.1E-01	excluded
LIMCH1	69115	ES	36	35	37	0.01	2.1E-01	included
TSPAN3	31978	ES	3:04	2.2	5	0.00	2.1E-01	excluded
RPS3A	70828	ES	2.2:3.1	1.3	4.1	0.00	2.1E-01	included
MAGI3	4273	ES	8	7	9	-0.04	2.1E-01	excluded
C10orf137	13425	ES	7	6	8	-0.02	2.1E-01	excluded
PHKA1	89508	ES	28	27	30	0.00	2.1E-01	included
BIN1	55183	ES	14:15:16	13	17	0.00	2.1E-01	included
LRRC28	32638	ES	7.1:7.2	6	8	0.01	2.1E-01	included
ZNF2	54492	ES	2	1	3	-0.02	2.1E-01	excluded
INPP5K	38318	ES	5.2	4	7	0.02	2.1E-01	included
SLC41A3	66573	ES	4:06	3	7	-0.01	2.1E-01	excluded
ST7L	4210	ES	15	14	16.1	-0.01	2.1E-01	excluded
ZNF473	51159	ES	3.1:3.2	2.2	4	-0.01	2.1E-01	excluded
PRRC2B	87935	ES	16	15	17	0.01	2.1E-01	included
USP3	31037	ES	5	3.2	7	0.00	2.1E-01	excluded
CPPED1	34059	ES	03:04.1	2	4.2	0.00	2.1E-01	included
GSS	59055	ES	5.1:5.3:6	4	7	0.00	2.1E-01	included
MAGI1	65535	ES	15	14	16	0.01	2.1E-01	included
OAS2	24611	ES	2.1	1	3	0.00	2.1E-01	excluded
CSRP1	9385	ES	7.2:8:9.1	7.1	9.2	0.00	2.1E-01	included

MKS1	42647	ES	16:17.1:17.2:17.3	15	18	0.00	2.1E-01	included
RBMS2	22464	ES	8	6	9	0.00	2.1E-01	included
PHYKPL	74859	ES	4:07	3	8.1	0.00	2.1E-01	excluded
TCP1	78319	ES	11	10	12	0.00	2.1E-01	excluded
SMARCA1	90045	ES	13	12	14	-0.01	2.1E-01	excluded
YAF2	21153	ES	3.2:5.2:7	2	9.1	0.00	2.1E-01	excluded
HSPA8	19200	ES	4.2:5.3:6.1	4.1	6.2	0.01	2.1E-01	included
IPO4	26887	ES	5.2	4.1	6	0.00	2.1E-01	included
NPRL3	32807	ES	4:05	2	6	0.01	2.1E-01	included
SLC25A39	41842	ES	2.1:2.2:3:4:5.2	1	6	0.00	2.1E-01	excluded
TMCO6	73699	ES	4.3:4.5	4.1	5.2	0.00	2.1E-01	included
HSCB	61553	ES	2.1:3.1:3.2	1	5	0.00	2.1E-01	included
SNX14	76929	ES	5	4	6	0.00	2.1E-01	included
LMO3	20624	ES	11.2	9.1	12	0.01	2.1E-01	included
BCAS3	42871	ES	29	28	30	0.02	2.1E-01	included
DMKN	49193	ES	7:08:10	6.4	11	0.03	2.1E-01	included
TRIT1	1932	ES	2:3.1:4:6.1:6.2:7.1:7.2	1	10	0.00	2.1E-01	excluded
SENP7	65946	ES	6	5	7	0.01	2.1E-01	included
TACC2	13345	ES	9	8	11	-0.02	2.1E-01	excluded
SHF	30414	ES	8.1:8.2:9.1	6	10	0.01	2.1E-01	included
SDCCAG8	10459	ES	2:03:04	1	5	0.00	2.1E-01	included
ZNF148	66549	ES	4	3.2	5	-0.03	2.1E-01	excluded
FAM156A	89182	ES	4	3.5	5	0.00	2.1E-01	excluded
PHF20L1	85194	ES	15	14	16	0.01	2.1E-01	included
CTSB	82664	ES	8	7	9	0.00	2.1E-01	included
RPAIN	38690	ES	05:06.1	3	7	-0.01	2.1E-01	excluded
CHEK1	19317	ES	3	2.3	4	0.01	2.1E-01	included
TMEM66	83266	ES	2.1:2.2:2.4	1.2	3	-0.01	2.1E-01	excluded
ZNF217	95980	ES	5	4	6	0.01	2.1E-01	included
SACM1L	64410	ES	1.3	1.1	2	-0.01	2.1E-01	excluded
METTL6	63576	ES	3	2	4	0.00	2.1E-01	included
GSTM1	4070	ES	4.2	3	5	0.05	2.1E-01	included
PREPL	53438	ES	8	7	9	0.00	2.1E-01	excluded
PSMF1	58476	ES	5	4	6	0.00	2.1E-01	included
COL1A2	484835	ES	33:34:35	31	36	0.01	2.1E-01	included
THNSL2	54472	ES	7:08:10	6	11	0.03	2.1E-01	included
FAM21C	11375	ES	22	21	23	0.01	2.1E-01	included
RPAP1	30098	ES	22.1	21	23.2	0.00	2.1E-01	included
MRPL55	10127	ES	2.2	1.2	2.9	0.02	2.1E-01	included
IKBKB	83691	ES	2:3:4.1:5:6.1:6.2:7	1.4	9	0.00	2.1E-01	included
C3orf33	67351	ES	4	3	5	0.00	2.1E-01	excluded
ELMOD3	54214	ES	4:05:06	3	7.1	0.00	2.1E-01	excluded
CBWD6	86491	ES	6	5	7	0.02	2.1E-01	included
MFF	57809	ES	8:09	7	11	0.01	2.1E-01	included
SMIM7	48191	ES	6.1	5	7.1	0.00	2.1E-01	included
STAG1	66934	ES	16	15	17	0.00	2.1E-01	included
NFS1	59216	ES	11	10	12	-0.01	2.1E-01	excluded
UBXN11	1262	ES	4	2	5	-0.02	2.1E-01	excluded
KLRG2	81962	ES	2:03:04	1	5	0.00	2.1E-01	included
MTR	10405	ES	2	1	3	0.01	2.1E-01	included
HEATR5B	100123	ES	34	33	35	0.01	2.1E-01	included
NBPF10	4452	ES	72:73:74:75:76:77	71	78	0.02	2.1E-01	included
ATG16L2	17665	ES	4.3:5:6.1:6.3:7:8:9:10:11:12:13	3	14	0.00	2.1E-01	included
DNAH14	9984	ES	6	5	7	0.00	2.1E-01	excluded
GALNT10	74214	ES	12	11	13	-0.01	2.1E-01	excluded
SDHD	18739	ES	3.1:3.2	2	5	0.00	2.1E-01	excluded
GATAD2A	48638	ES	7:8:9:10:11	6.2	12	0.00	2.1E-01	excluded
CAMK2D	70416	ES	17	14.2	18	0.01	2.1E-01	included
ADH5	70028	ES	2	1	3	0.00	2.1E-01	included
TIMMDC1	66306	ES	5	4	6	0.00	2.1E-01	included
CORO1C	24252	ES	8:09	7	10	0.00	2.1E-01	excluded

MAP2K4	39302	ES	4	3	5	-0.01	2.1E-01	excluded
MARK3	29445	ES	18	17	19	-0.02	2.2E-01	excluded
ABAT	33906	ES	8	7	9	0.01	2.2E-01	included
DNM1L	21049	ES	9	8	10	0.00	2.2E-01	included
SUGP1	48621	ES	5.2:6.1	4	6.2	0.00	2.2E-01	included
AMOTL1	18368	ES	2	1	3	0.02	2.2E-01	included
PML	31641	ES	5:6.1:6.4:6.5:6.6:6.7	4	6.8	0.00	2.2E-01	included
FAM105B	71590	ES	2	1	3	-0.01	2.2E-01	excluded
UBR4	880	ES	105	104	106	0.00	2.2E-01	included
TBC1D23	65818	ES	4	3	5	0.00	2.2E-01	included
USP14	44438	ES	3:04	2	5	0.00	2.2E-01	included
IL32	33438	ES	1.4:1.5:1.6	1.1	1.9	0.00	2.2E-01	included
SLMAP	65401	ES	14	12	15.2	0.02	2.2E-01	included
CLCC1	3977	ES	6.1:6.2:7.1:7.2:8:9	5	10	0.00	2.2E-01	included
PTBP3	87242	ES	5	4.2	6	-0.02	2.2E-01	excluded
RNPS1	33260	ES	2.3:3	1.1	4	0.00	2.2E-01	included
SIK3	18878	ES	10	9	11	-0.01	2.2E-01	excluded
CEP72	71445	ES	5	4	6	-0.01	2.2E-01	excluded
PPA2	70244	ES	2	1	4	0.00	2.2E-01	included
PSMC5	43006	ES	7	6	8	0.00	2.2E-01	included
INADL	3243	ES	36:37:00	35	38.1	0.01	2.2E-01	included
ACVR1B	21879	ES	6	5	7	-0.01	2.2E-01	excluded
SLA	85216	ES	8	7	9	0.00	2.2E-01	included
ZNF559	47281	ES	3.1:3.2:3.3	2.2	4	0.03	2.2E-01	included
SAR1B	73416	ES	10	9	11	0.00	2.2E-01	excluded
ZNF185	90409	ES	11	10	13	-0.03	2.2E-01	excluded
CCDC132	80464	ES	4:5:6:7:8:9:10:11:12:1	2.1	14	0.00	2.2E-01	included
WDR44	89938	ES	3	2	4	0.01	2.2E-01	included
USP54	12168	ES	7:8:9:10	6	11	0.00	2.2E-01	included
CES4A	36916	ES	12:13	11	14	-0.02	2.2E-01	excluded
ELMO2	59677	ES	12	11	13	0.00	2.2E-01	included
TRDMT1	10925	ES	3	2	4	-0.03	2.2E-01	excluded
SEPT2	58357	ES	8	7	9	-0.01	2.2E-01	excluded
MLPH	58118	ES	7	6	8	0.01	2.2E-01	included
MBTD1	42522	ES	14	13	15	0.00	2.2E-01	included
ACTR6	23909	ES	6	4	7	0.00	2.2E-01	excluded
CRELD2	62759	ES	8	7	9	0.00	2.2E-01	excluded
QPCTL	50516	ES	3	2	4	0.00	2.2E-01	included
CPNE1	59194	ES	1.2:2.1:2.2	1.1	3	0.00	2.2E-01	included
UBE2D3	70127	ES	13	12	14	0.00	2.2E-01	included
RNASE4	26460	ES	3	1.2	4	-0.02	2.2E-01	excluded
ZNF576	50224	ES	1.2:1.3:1.5	1.1	1.6	0.00	2.2E-01	included
MPDZ	85877	ES	27	26	29	0.02	2.2E-01	included
EPS8	20585	ES	10	9	13	0.00	2.2E-01	included
DTX3L	66446	ES	3	2	4	0.00	2.2E-01	excluded
ARHGAP30	8546	ES	1.2:3:4:5:6	1.1	7	0.00	2.2E-01	included
NPNT	70261	ES	5	4	6	0.00	2.2E-01	excluded
EBPL	25915	ES	2	1	4.1	0.01	2.2E-01	included
HSPH1	25576	ES	13	12	14	0.00	2.2E-01	excluded
TBL2	79986	ES	2.3	1	4.3	0.00	2.2E-01	included
CLUAP1	33589	ES	4	3	6	0.00	2.2E-01	included
AKAP2	87182	ES	10	8	11	0.00	2.2E-01	included
OGG1	63163	ES	7.2	6.1	8	-0.02	2.2E-01	excluded
PRKRIP1	97666	ES	6	4	7	0.02	2.2E-01	included
RAB1A	53789	ES	7	5	8	-0.03	2.2E-01	excluded
MBNL1	67323	ES	9	7	11	0.00	2.2E-01	excluded
INO80E	36012	ES	6.1:6.2:6.3:10	5	11	-0.02	2.2E-01	excluded
C8orf59	84338	ES	2.1:2.2	1	3.2	0.01	2.2E-01	included
CCDC88B	16646	ES	25.1:25.2	24	26	-0.01	2.2E-01	excluded
TRIM16L	39639	ES	2	1.3	3	-0.02	2.2E-01	excluded
CARS	13945	ES	6	5.2	7	0.00	2.2E-01	excluded
ATXN2L	35851	ES	22.2:22.6	22.1	22.7	0.02	2.2E-01	included

FAM114A1	69037	ES	2	1	3	0.02	2.2E-01	included
PPP2R1A	51421	ES	6	5	7	0.00	2.2E-01	included
C14orf159	28857	ES	9	8	10.1	0.00	2.2E-01	excluded
LUC7L3	42482	ES	03:04.2	2	5	-0.01	2.2E-01	excluded
AES	46660	ES	4:05	3.2	6	0.00	2.2E-01	excluded
FAM173B	71550	ES	2	1	3.1	0.00	2.2E-01	excluded
ZNF415	51681	ES	6.1:7.1:7.2:8.1:8.2	4	9	0.04	2.2E-01	included
CCT8	60306	ES	5	4	6	0.00	2.2E-01	included
KLC1	29488	ES	13.3:14.1	13.2	15	-0.01	2.2E-01	excluded
CNIH1	27588	ES	3	1	5	0.00	2.2E-01	included
ACOT9	88693	ES	12	11	13.1	0.00	2.2E-01	excluded
IL6ST	72088	ES	4.1:4.2:5:6:7:8:10:11:<br>12:13:14:15:16	3.2	17	0.00	2.2E-01	included
CTDSPL	64002	ES	4	3	5	-0.02	2.2E-01	excluded
FAM98C	49642	ES	7	6	8	0.00	2.2E-01	excluded
DARS2	9026	ES	15	14	16	0.00	2.2E-01	included
SFTA3	27275	ES	3:4.1:4.2	2	6	0.01	2.2E-01	included
BATF	28504	ES	2.2:3.1	2.1	3.2	0.01	2.2E-01	included
LRRFIP2	63973	ES	6:7:8:9:10:11:12:13:1	5	18	0.00	2.2E-01	excluded
RBM23	26652	ES	6	5	7	0.02	2.2E-01	included
ARL13B	65701	ES	2:04	1	5.1	0.01	2.2E-01	included
ZDHHC15	89528	ES	2	1	3	0.02	2.2E-01	included
SNX1	31094	ES	7	6	8	0.00	2.2E-01	included
CDK14	80405	ES	5	4	6	0.00	2.2E-01	excluded
RPTOR	44084	ES	3	2	4	0.00	2.2E-01	included
DNAJC11	498	ES	4	3	5	0.00	2.2E-01	excluded
CASC4	30337	ES	4	3	5	0.00	2.2E-01	included
GTF2H3	25106	ES	4	3	5	0.00	2.2E-01	excluded
VPS35	36305	ES	3.1:3.2	2	4	0.00	2.2E-01	excluded
RIMKLB	20204	ES	3	2	4	-0.01	2.2E-01	excluded
IFNGR2	60415	ES	2.2	1	4	0.00	2.2E-01	excluded
SEMA4G	12847	ES	15.1	14	15.3	-0.01	2.2E-01	excluded
PTGES3	93183	ES	5	4.2	7	0.02	2.2E-01	included
RMDN2	53236	ES	14	13	15	0.00	2.2E-01	included
GTF2H2C	72392	ES	7	6	8	0.00	2.2E-01	included
SPATA7	28694	ES	7	6.2	9	0.00	2.2E-01	excluded
HMGN1	107414	ES	7	6.2	9	0.02	2.2E-01	included
THBS3	8034	ES	3:04	2.1	5	0.00	2.2E-01	excluded
COBL	79730	ES	9	8	12	-0.01	2.2E-01	excluded
HYOU1	19088	ES	2	1.2	3	0.01	2.2E-01	included
AGTRAP	677	ES	03:04.2	1	5	0.01	2.2E-01	included
NVL	9945	ES	9	8	10	0.00	2.2E-01	excluded
C12orf10	22016	ES	3	2.2	4	0.00	2.2E-01	excluded
SGCE	80505	ES	4	1	5	0.00	2.2E-01	included
EPHX2	83163	ES	7	6	8	0.00	2.2E-01	included
ENTPD6	58869	ES	02:03.2	1	4	0.02	2.2E-01	included
ZNF426	47356	ES	6	5	7	-0.01	2.2E-01	excluded
TRIT1	1936	ES	2:3.1:4:6.1:7.1:7.2:8:9	1	10	-0.01	2.2E-01	excluded
TAF6	80897	ES	6	5.2	7	0.00	2.2E-01	included
ABHD14B	65143	ES	3.1:3.2:3.3	2.2	6.1	0.02	2.2E-01	included
RBM39	59245	ES	7:09	6	10	0.00	2.2E-01	excluded
DHRS4	26787	ES	3:4:5:6	2	7.1	0.01	2.2E-01	included
HNRNPD	69703	ES	2	1.3	3	0.01	2.2E-01	included
TCIRG1	17287	ES	5.3	5.1	6	0.00	2.2E-01	excluded
TBP	78494	ES	2.1:2.2	1	3	-0.01	2.2E-01	excluded
GGA3	43406	ES	3:4:5:6	2	8	-0.01	2.2E-01	excluded
ZNF480	51437	ES	4	3	5.1	0.00	2.2E-01	included
ARRB1	17833	ES	13	12	15.2	0.02	2.2E-01	included
RAB17	58121	ES	3	2.3	4	0.00	2.2E-01	excluded
CNOT10	63826	ES	4	3	5	0.00	2.2E-01	included
SNX15	16730	ES	7	6	8	0.00	2.2E-01	included
DCAF8	8448	ES	7.2:8.1:8.2	7.1	9	-0.02	2.2E-01	excluded

TMEM143	50738	ES	4:05	2	6	0.01	2.2E-01	included
ESM1	72019	ES	2	1	3	0.01	2.2E-01	included
SEPT4	42703	ES	7.1:7.2	3	8	0.03	2.2E-01	included
MRPL3	66794	ES	2	1	3	0.01	2.2E-01	included
GSTO2	13053	ES	5:08	4	9	0.00	2.2E-01	excluded
GLOD4	38276	ES	2	1	4.2	0.00	2.2E-01	excluded
EIF1AD	16977	ES	1.2:2.1	1.1	2.2	0.02	2.2E-01	included
ZNF707	85484	ES	2:4.2:5.1:5.2	1	6	-0.02	2.2E-01	excluded
REXO2	18836	ES	5.2:5.3	4	6	0.00	2.2E-01	included
ZNF720	36291	ES	2:03	1.2	5.1	-0.02	2.2E-01	excluded
THEMIS2	1353	ES	3:4.1:4.3	2	5	0.02	2.2E-01	included
UFD1L	61058	ES	3	2	4	0.00	2.2E-01	excluded
LUC7L3	42487	ES	2	1	5	0.00	2.2E-01	excluded
POLR2I	49344	ES	2	1.2	3	0.00	2.2E-01	included
PER1	39098	ES	15	14	16	0.00	2.2E-01	excluded
PPP6C	87548	ES	1.2:2	1.1	3	0.00	2.2E-01	included
SLC25A17	62379	ES	4:5.1:5.2	2.2	7.2	0.00	2.2E-01	excluded
CD74	99691	ES	9	4	10.1	0.00	2.2E-01	excluded
RTN3	16522	ES	8	7	9	0.00	2.2E-01	included
BTBD1	32249	ES	6	5	7	0.00	2.2E-01	excluded
SNX1	139165	ES	4.1:4.2:5:6:7:8:9:10.1:10.2:11:12:13.1:14:15	3	16.1	0.03	2.2E-01	included
M6PR	20218	ES	4:05	3	6	0.00	2.2E-01	excluded
KLHDC4	37957	ES	5:6.1:6.2	4.2	7.1	-0.03	2.2E-01	excluded
TRAF4	39977	ES	7:8:9.1:9.2	6	9.3	0.00	2.2E-01	excluded
TYSND1	12031	ES	2:03	1	4	0.00	2.2E-01	included
BBC3	50634	ES	3:04	1	5	-0.01	2.2E-01	excluded
TULP3	19734	ES	11	10.1	12	-0.01	2.2E-01	excluded
ZNF655	80688	ES	4:5.1:5.2:5.3	3.2	7	0.03	2.2E-01	included
SMPD4	55310	ES	4.1:4.2:5:6:7:8	3	9	0.00	2.2E-01	excluded
TBC1D15	23424	ES	4	1	5	0.00	2.3E-01	excluded
TRIM37	42725	ES	24	23	25.1	-0.01	2.3E-01	excluded
MLH3	28469	ES	8	6	9	-0.02	2.3E-01	excluded
CHP1	30076	ES	2	1	3	0.00	2.3E-01	included
SNURF	29715	ES	3.1:6:7:8	2	9	0.02	2.3E-01	included
POLK	72537	ES	3	1	4	0.01	2.3E-01	included
ZNF74	61153	ES	2:03	1.3	4	-0.04	2.3E-01	excluded
BZW2	78866	ES	1.2:2	1.1	3	0.00	2.3E-01	included
LMAN2L	54577	ES	2	1	4	0.01	2.3E-01	included
IGHMBP2	17351	ES	4	3	5	0.00	2.3E-01	excluded
ZMYND15	38585	ES	8	7	9	0.01	2.3E-01	included
HARS	73728	ES	4:5:6.1:6.2	2	7	0.00	2.3E-01	included
MAGIX	89091	ES	2.3	2.1	3	0.02	2.3E-01	included
LPCAT3	20061	ES	7	6	8	0.00	2.3E-01	included
PDE8B	72566	ES	14	13	16	0.00	2.3E-01	included
ZNF707	85480	ES	7.2:8.1	7.1	8.2	0.01	2.3E-01	included
SFXN5	53948	ES	12:13	11	14	-0.01	2.3E-01	excluded
HNRNPR	1048	ES	2.1:2.2	1	3	0.01	2.3E-01	included
NPHP4	354	ES	22:23	21	24	-0.02	2.3E-01	excluded
ZNF146	49374	ES	2	1	3	-0.02	2.3E-01	excluded
LYRM9	39870	ES	6.3	6.1	6.5	0.00	2.3E-01	included
HEXA	31549	ES	6	4	7	0.02	2.3E-01	included
HDAC11	63477	ES	7	6.2	8	0.00	2.3E-01	excluded
LCK	1583	ES	8	7	9	-0.01	2.3E-01	excluded
PTPN2	44725	ES	7	6	9.1	0.00	2.3E-01	included
CDK20	86780	ES	5:06	4	7	-0.01	2.3E-01	excluded
SUOX	22342	ES	4	1	5.2	0.03	2.3E-01	included
TRIM23	72240	ES	2	1	3	0.00	2.3E-01	included
TRIT1	1974	ES	02:03.1	1	6.1	0.03	2.3E-01	included
CFLAR	56797	ES	10:11:12	8.1	13	0.00	2.3E-01	excluded
GINS3	36636	ES	3	1	4	-0.02	2.3E-01	excluded
BCL7B	79951	ES	5	3	6	-0.01	2.3E-01	excluded

FGFR2	13308	ES	15:17.1:17.2	14	18	0.00	2.3E-01	included
NSFL1C	58505	ES	5.1:5.2	4	7.2	0.00	2.3E-01	excluded
RAD51D	40254	ES	7	5	8	-0.01	2.3E-01	excluded
PCGF3	68407	ES	4	3.2	5.1	0.03	2.3E-01	included
GGT5	61396	ES	2:03	1	4	0.00	2.3E-01	included
FHL2	54828	ES	4	3.2	5.1	-0.03	2.3E-01	excluded
LYZ	23316	ES	3	2.1	4	0.00	2.3E-01	included
HOMER3	48538	ES	7	6	8	0.00	2.3E-01	included
MRPL55	10161	ES	1.2:2.4:2.5	1.1	2.9	0.00	2.3E-01	excluded
CAPN10	58292	ES	2:3.1:3.2:4:5:6:7:9	1	11	-0.03	2.3E-01	excluded
CMC2	37736	ES	4.3:4.4	2	5	0.00	2.3E-01	excluded
MLF1	67439	ES	1.2:4.1:4.2	1.1	5	-0.02	2.3E-01	excluded
NT5C2	12992	ES	5	4	6	-0.02	2.3E-01	excluded
SLC52A2	85577	ES	1.3:1.4:2.2	1.2	2.3	0.00	2.3E-01	excluded
SLC25A17	62371	ES	4	2.2	5.1	0.00	2.3E-01	included
SLC50A1	7942	ES	5	4	6	0.00	2.3E-01	excluded
LMCD1	63053	ES	3	2	4	0.00	2.3E-01	included
STAU1	59743	ES	2	1	3	-0.02	2.3E-01	excluded
SCRN3	56021	ES	4	3	5	0.00	2.3E-01	included
PLEKHA4	50821	ES	17:18	16	19	0.00	2.3E-01	included
ANGPTL2	87615	ES	2	1	3	0.00	2.3E-01	excluded
POT1	81644	ES	7	6	8	0.01	2.3E-01	included
TMEM165	69327	ES	2	1	3	0.00	2.3E-01	included
NCOA1	52826	ES	24:25.1	23	25.2	0.01	2.3E-01	included
PPP3CC	83013	ES	11	10	12	0.00	2.3E-01	included
TRAP1	33630	ES	4	1	6.1	0.00	2.3E-01	included
CARF	56973	ES	6.1:6.2	5.2	7	0.01	2.3E-01	included
CD8A	54445	ES	7	6	8	0.01	2.3E-01	included
SPI1	15730	ES	2.1:2.2:3:4.1	1	5	0.00	2.3E-01	included
RNASE4	26461	ES	1.2:3	1.1	4	-0.01	2.3E-01	excluded
WHSC1	68531	ES	13.3:13.4	13.1	14	0.00	2.3E-01	included
PPWD1	72225	ES	4	3	5.1	-0.02	2.3E-01	excluded
MTG2	60051	ES	2	1	3	-0.01	2.3E-01	excluded
SCRN2	42124	ES	3	2.2	4.2	0.00	2.3E-01	included
TPGS2	45271	ES	5	4.2	6	0.00	2.3E-01	excluded
DPM1	95974	ES	7:08	6	9	-0.01	2.3E-01	excluded
PARP8	71986	ES	8	4	10	0.00	2.3E-01	included
AIG1	77974	ES	6	4	7	0.00	2.3E-01	excluded
MAGI1	65534	ES	19.2	18	20	-0.03	2.3E-01	excluded
OAT	13399	ES	2	1	3	0.00	2.3E-01	excluded
BROX	9897	ES	6	5	7	0.01	2.3E-01	included
C11orf49	15636	ES	2	1	3	0.00	2.3E-01	included
FN1	57364	ES	41	40.4	42	0.00	2.3E-01	included
ALDH1A1	86606	ES	7.2:8:9.1	7.1	9.2	0.00	2.3E-01	excluded
PILRB	80932	ES	11:12.1	9	12.2	0.01	2.3E-01	included
DGUOK	54006	ES	3:4:5:6	1	7	-0.01	2.3E-01	excluded
DTWD1	30558	ES	2	1.1	3	-0.01	2.3E-01	excluded
CDH24	26700	ES	9	8	10	0.03	2.3E-01	included
POLM	79466	ES	4:05	2	6.1	0.00	2.3E-01	included
BRF2	83360	ES	3.1:3.2	2	4	0.00	2.3E-01	excluded
PAM	72907	ES	3	2	4	0.00	2.3E-01	included
CTTN	17404	ES	11	10	13	-0.01	2.3E-01	excluded
PACS2	29638	ES	4	3	5	0.00	2.3E-01	excluded
CSNK1G3	73145	ES	13	12	14	-0.02	2.3E-01	excluded
DCTN6	83283	ES	4	2	5	0.00	2.3E-01	included
TMEM68	83874	ES	1.2:2:3.1	1.1	4	-0.01	2.3E-01	excluded
ADIRF	12443	ES	2	1	3	0.00	2.3E-01	excluded
ZNF550	52296	ES	3	2.2	4	0.00	2.3E-01	included
BCORL1	90062	ES	12	11	13	0.00	2.3E-01	excluded
ZNF669	10511	ES	3	2.2	4	0.01	2.3E-01	included
UQCRC2	35525	ES	6	4	7	0.00	2.3E-01	excluded
FHL1	90192	ES	8	7	10	0.00	2.3E-01	excluded

NUBPL	27125	ES	9	8	10	0.01	2.3E-01	included
TGM2	59374	ES	3	2	4	0.00	2.3E-01	excluded
ZNF544	52435	ES	3.3:4.1:4.2:5.2:6.1	3.2	6.2	-0.02	2.3E-01	excluded
TMEM189	59774	ES	5.1:5.2	3	6	0.00	2.3E-01	included
PIGT	59559	ES	2.1:2.2:4	1	5.2	-0.02	2.3E-01	excluded
NSUN2	71516	ES	6	5	7	0.00	2.3E-01	excluded
PICALM	18174	ES	4	3	5	0.00	2.3E-01	included
CORO1B	387269	ES	4:5.1:5.3:5.4:5.5:5.6:5.	3	7	0.00	2.3E-01	included
ATF2	56081	ES	8:9:10.1:11:12:13:14	6	16	-0.01	2.3E-01	excluded
NUP54	69591	ES	2:04	1	5	0.00	2.3E-01	excluded
MAX	27954	ES	5.3:5.4:5.5	5.1	5.8	0.00	2.3E-01	included
ATXN3	28900	ES	10.3:13.1	10.2	13.2	0.00	2.3E-01	included
PGGT1B	73023	ES	6:07	5	8	0.00	2.3E-01	excluded
UBE2J2	48	ES	6.1	5	7	0.00	2.3E-01	included
NDUFAF7	53225	ES	3	2	4.1	0.00	2.3E-01	included
LRRFIP1	58138	ES	4:5:6:7:8:9:10:11:12	3	13	0.00	2.3E-01	included
OPA1	68140	ES	5	4	6	-0.02	2.3E-01	excluded
PPIL2	61251	ES	5	4	6	0.00	2.3E-01	excluded
SLC25A26	65545	ES	12	11	13.1	0.00	2.3E-01	included
FGR	1336	ES	7.2:8	7.1	9	0.00	2.3E-01	excluded
CRYZL1	60460	ES	7:09	6	10	0.00	2.3E-01	excluded
TRAF3	29427	ES	2	1	3	0.01	2.3E-01	included
KIAA1191	74643	ES	4:05:06	3	7	0.00	2.3E-01	included
FZD6	84806	ES	4	3.4	5	0.00	2.3E-01	excluded
COPS7B	57945	ES	6	5	7.1	0.00	2.3E-01	included
ARID1A	1297	ES	18.1:18.2	17	19	0.00	2.3E-01	included
CDK5RAP2	87382	ES	34	33	35	-0.01	2.3E-01	excluded
GRN	522185	ES	4:5:6.2:7:8:9	3	11	0.00	2.3E-01	included
COL23A1	74866	ES	6	5	7	0.01	2.3E-01	included
MBOAT2	52610	ES	2:03	1	4	0.01	2.3E-01	included
UBE2W	84192	ES	8	6	9	0.00	2.3E-01	included
EIF3M	14858	ES	2.1:2.2	1	5	-0.02	2.3E-01	excluded
ZNF83	51507	ES	9.6	7	10.1	-0.01	2.3E-01	excluded
IFI27L1	29061	ES	06:07.1	4.3	8	-0.02	2.3E-01	excluded
GORASP1	64152	ES	6.1:6.2:7.1	5	7.2	0.00	2.3E-01	included
ZNF562	47387	ES	4	3	5	0.01	2.3E-01	included
GUSB	79862	ES	4.1	2	5.1	0.01	2.3E-01	included
YWHAE	38299	ES	2:03	1	7	0.00	2.3E-01	excluded
FAM114A2	74200	ES	2.2:3:4.1	2.1	4.2	0.00	2.3E-01	included
CD74	74082	ES	09:10.1	4	10.2	0.00	2.3E-01	excluded
PSMD10	89847	ES	2	1	3.1	0.00	2.3E-01	included
DIP2A	60946	ES	2:03	1	4	0.00	2.3E-01	excluded
PML	31653	ES	5:6.1:6.2:6.4:6.7	4	6.8	0.00	2.3E-01	included
MED23	77618	ES	14	13.2	15	0.01	2.3E-01	included
FARP1	26166	ES	22	21	23	0.00	2.3E-01	excluded
R3HDM2	22573	ES	14	13	15	0.00	2.3E-01	excluded
ZNF821	37502	ES	8.1	7.1	9.1	0.00	2.3E-01	included
NCF2	9187	ES	5	4	6	0.00	2.3E-01	excluded
DCP2	73003	ES	2:03	1	4	-0.01	2.3E-01	excluded
NBPF10	7271	ES	3.2:4.1:4.2:6:7:8	3.1	9	0.03	2.3E-01	included
C16orf95	37937	ES	4:05	3	6	-0.02	2.3E-01	excluded
HIF1A	27802	ES	15	14	16	0.00	2.3E-01	included
RAE1	59892	ES	10	9	11	0.00	2.3E-01	included
HNRNPA2B1	318763	ES	7:08	6	10	-0.02	2.3E-01	excluded
FAM86A	33891	ES	4	3.1	5.2	0.01	2.3E-01	included
SPATA13	25478	ES	5	4	6	0.01	2.3E-01	included
HYDIN	37421	ES	22	21.1	23	0.02	2.3E-01	included
BCL2L11	54954	ES	10	7	11	-0.01	2.4E-01	excluded
RNF14	73853	ES	2.2:3	2.1	4	-0.03	2.4E-01	excluded
ELP3	83203	ES	6	5	7	0.00	2.4E-01	included
POLD4	17169	ES	3	2	4	0.00	2.4E-01	included
CLASRP	50391	ES	16	15.2	17	0.00	2.4E-01	excluded

PSMG4	75186	ES	5.1:5.4:5.5	4	5.6	0.02	2.4E-01	included
CNN2	46363	ES	5.2	4.2	6	0.00	2.4E-01	excluded
SMAD4	45564	ES	11:12:13	10	14	0.00	2.4E-01	included
ZC3H14	28726	ES	12	10	15	0.01	2.4E-01	included
WDR6	64804	ES	3.1:3.2	1	4.2	-0.02	2.4E-01	excluded
IFNGR2	60413	ES	2.2:3	1	4	0.00	2.4E-01	excluded
PHF15	73405	ES	12	11	13.2	0.02	2.4E-01	included
DYX1C1	30738	ES	4	3.2	5	0.00	2.4E-01	excluded
ACTB	264671	ES	3	1	5	0.02	2.4E-01	included
SLC30A6	53150	ES	15	14	16	0.00	2.4E-01	included
FAM214B	86243	ES	10.2:11.1	10.1	11.2	0.00	2.4E-01	included
MAP4K4	54760	ES	17:18	16.2	19	-0.01	2.4E-01	excluded
FLII	39591	ES	18:19:20:21:22:23:24:<br>25:26:27:28:29:30:31.	17	31.2	0.00	2.4E-01	included
NLRC5	36517	ES	29	28	30	0.01	2.4E-01	included
SH3GLB2	87813	ES	4	3	5	0.00	2.4E-01	included
SLC25A40	80344	ES	3	2	4	-0.02	2.4E-01	excluded
GRIPAP1	89055	ES	4:05	3	6	0.00	2.4E-01	excluded
TMEM256-PLSC	38937	ES	8	7	9.1	0.01	2.4E-01	included
SH3PXD2A	13026	ES	12	11	13	0.01	2.4E-01	included
MDH2	80160	ES	6	5	7	0.00	2.4E-01	included
CPNE1	59185	ES	8.1:8.2	7	9	0.01	2.4E-01	included
FAM86A	33881	ES	6:07	5.2	8	0.00	2.4E-01	included
GATSL3	61712	ES	2	1.1	4	-0.01	2.4E-01	excluded
MLF1IP	71315	ES	11	10	13	-0.02	2.4E-01	excluded
RUNX1	60506	ES	8	6	10	0.01	2.4E-01	included
OBSCN	10200	ES	49:50:51:52:53:54.1:5	48	55	0.02	2.4E-01	included
EIF2AK1	78711	ES	2	1	3	0.00	2.4E-01	excluded
PMF1	8197	ES	8.1:8.2	7	10	0.00	2.4E-01	excluded
LETMD1	21751	ES	3.2:3.3:4	2	5	0.04	2.4E-01	included
CCNI	69628	ES	2	1	3	0.00	2.4E-01	excluded
COPS7B	57961	ES	4.4	3	5	0.00	2.4E-01	included
SPATA20	42427	ES	5	4.2	6	0.01	2.4E-01	included
RNH1	13676	ES	3	2	4.3	0.02	2.4E-01	included
RNF121	17463	ES	2	1	3	-0.01	2.4E-01	excluded
DDX19B	37355	ES	05:02.2	3	9	0.01	2.4E-01	included
COPS5	84062	ES	4.1	2.3	5	0.00	2.4E-01	included
DTX3	22662	ES	1.3	1.1	1.5	0.02	2.4E-01	included
SEMA3F	64965	ES	1.2:3.1	1.1	3.2	0.00	2.4E-01	excluded
RBM14	17086	ES	2	1	4.1	-0.01	2.4E-01	excluded
STRAP	20590	ES	2	1	3	0.00	2.4E-01	excluded
MCFD2	53478	ES	5	3	6	0.02	2.4E-01	included
GLT1D1	25191	ES	7:8:9:10	5	11	-0.02	2.4E-01	excluded
MARS	22602	ES	6	5.2	7.1	0.00	2.4E-01	included
CEP95	43071	ES	5	4.2	6	0.02	2.4E-01	included
TOM1L1	42541	ES	8	7	9.1	0.00	2.4E-01	included
ARSA	62896	ES	1.5	1.2	2	0.01	2.4E-01	included
SDCCAG3	88164	ES	2	1	4	-0.02	2.4E-01	excluded
ZDHHC8	61145	ES	4:05	3	6	0.00	2.4E-01	included
RNF34	24868	ES	2:3:4:5:6	1	7.1	0.01	2.4E-01	included
KDM5C	89203	ES	3:05	1	6	0.00	2.4E-01	excluded
ERCC2	50434	ES	6:07	5	8	0.00	2.4E-01	excluded
RAB6B	66861	ES	3.2:4:5:6:7:8:9.1	3.1	9.2	0.00	2.4E-01	included
SZRD1	810	ES	2:03	1	4.2	0.00	2.4E-01	excluded
RAB6A	17711	ES	5:07:08	4	9	0.00	2.4E-01	excluded
LSR	49090	ES	3	2.2	6	0.00	2.4E-01	included
CNOT8	74252	ES	3	2	5	0.00	2.4E-01	included
NECAP2	826	ES	4	3	5	0.00	2.4E-01	included
PDZD11	89371	ES	2	1	3	0.00	2.4E-01	included
CASP6	70333	ES	2:03:04	1	5	0.00	2.4E-01	excluded
TM6SF1	32250	ES	8	7	9	0.00	2.4E-01	excluded
LOXL3	54112	ES	4:05	3	6	0.02	2.4E-01	included

COL6A2	206509	ES	13:14:15	12	16	0.00	2.4E-01	included
RABGGTB	3514	ES	5:06	4.1	7	-0.01	2.4E-01	excluded
SDHC	8665	ES	2:03	1	5	-0.01	2.4E-01	excluded
OXA1L	26617	ES	9	8	10	0.00	2.4E-01	included
PPP6R3	17310	ES	24:26.1	23	26.2	0.00	2.4E-01	excluded
ACAD8	19554	ES	3	2	4	0.01	2.4E-01	included
PTCD2	72463	ES	3:04:05	2	6	0.00	2.4E-01	excluded
TMX2	15914	ES	4	2	5.1	0.01	2.4E-01	included
SFTA3	27270	ES	3:4.1:4.2:5	2	6	-0.02	2.4E-01	excluded
ENGASE	43994	ES	4	3	5	-0.01	2.4E-01	excluded
AP5M1	27652	ES	3	1	4	0.00	2.4E-01	excluded
TMEM87A	30137	ES	3	1	4	0.00	2.4E-01	included
TOMM40L	8618	ES	5	4	6	0.00	2.4E-01	excluded
FAM13A	69916	ES	10	9	11	-0.02	2.4E-01	excluded
ANKRD28	63630	ES	6	5	7	0.01	2.4E-01	included
PRPS1	89832	ES	3.1	2	4	0.00	2.4E-01	included
DMKN	101868	ES	8:09:11	6.4	12	0.01	2.4E-01	included
SCPEP1	94617	ES	10.1:10.2	9	11	0.03	2.4E-01	included
CEP89	48913	ES	12	11	13	-0.01	2.4E-01	excluded
CARKD	26257	ES	4:05	3	6	0.00	2.4E-01	included
CELF2	10745	ES	8	7	9	-0.01	2.4E-01	excluded
NEIL1	31857	ES	05:06.1	4	6.2	-0.03	2.4E-01	excluded
HAUS1	45387	ES	4:05:06	2.1	7	0.00	2.4E-01	included
PPP2R4	87845	ES	5:06	4	7	-0.01	2.4E-01	excluded
POC1B	23629	ES	12	10	13	-0.01	2.4E-01	excluded
TOLLIP	13832	ES	3.1:3.2	1.2	4	0.00	2.4E-01	excluded
EVA1C	60349	ES	2	1	3	0.00	2.4E-01	excluded
LRRFIP2	63975	ES	6:7:8:10:11:12:13:14	5	18	0.00	2.4E-01	excluded
TMX3	45758	ES	7	6	8	0.00	2.4E-01	excluded
EMC4	29842	ES	2.3:4.1:4.2	2.2	6	0.00	2.4E-01	included
RABEP1	38673	ES	2	1	3	0.00	2.4E-01	excluded
TMEM260	27645	ES	10	9	11	-0.01	2.4E-01	excluded
LYPLA2	1076	ES	7	6	8.1	0.00	2.4E-01	included
DDX55	25092	ES	2.1:2.2:3:4	1	5.1	0.02	2.4E-01	included
PAK1	17952	ES	16:17.1	15	17.2	0.00	2.4E-01	included
RELA	16901	ES	5	4.2	6.1	0.00	2.4E-01	included
FAM111A	16028	ES	3.1	2	3.3	0.02	2.4E-01	included
SLC25A19	43431	ES	6	5	7	0.00	2.4E-01	excluded
GGCT	79136	ES	5	3	7	0.00	2.4E-01	included
ANAPC1	54973	ES	5	4	6	0.00	2.4E-01	excluded
CYFIP2	74336	ES	21	20	22	0.00	2.4E-01	excluded
NUPL1	25501	ES	5	4.1	7	-0.01	2.4E-01	excluded
MIB2	194	ES	10.1:10.2	9	11	0.00	2.4E-01	included
IAH1	52632	ES	03:04.1	2	4.2	0.00	2.4E-01	included
ENOPH1	69707	ES	3	2	4	0.00	2.4E-01	excluded
APBB1	14118	ES	13	12	14.2	0.00	2.4E-01	excluded
HNRNPC	26554	ES	2.2:2.4:2.6	1	3.2	0.00	2.4E-01	excluded
DYX1C1	30737	ES	6	5	7	0.01	2.4E-01	included
PRMT2	60955	ES	8:09	6.1	10	0.00	2.4E-01	excluded
MEGF8	50158	ES	30	29	31	-0.01	2.4E-01	excluded
ARHGEF10L	863	ES	14:15	13	16	0.00	2.4E-01	excluded
CTCF	37087	ES	3:04	2	5	0.00	2.4E-01	included
IRF3	51014	ES	1.4:2	1.1	3	0.00	2.4E-01	excluded
CSNK1D	44310	ES	3.1:3.2	1	4	-0.03	2.4E-01	excluded
PIAS2	45423	ES	3	2	4	0.00	2.4E-01	included
PRTFDC1	11018	ES	9	8	10	0.01	2.4E-01	included
EBPL	25906	ES	4.1:4.2	2	6	0.00	2.4E-01	excluded
TIE1	2160	ES	7:09	6	10	0.00	2.4E-01	included
ITGAV	56488	ES	6:07	5	8	0.00	2.4E-01	included
RPUSD3	63227	ES	8	7	9	0.00	2.4E-01	included
GORASP2	55918	ES	3	1	4.1	0.00	2.4E-01	excluded
STOX1	11964	ES	3.1:3.2	2	4.1	0.01	2.4E-01	included

HPS4	61509	ES	7	6	8.2	0.01	2.4E-01	included
TRIP10	47081	ES	11.1:11.2:12.1	10	12.2	0.00	2.4E-01	excluded
CTDSP2	22769	ES	3.2:4:5.2:6:7:8.1	3.1	8.2	0.00	2.4E-01	excluded
IFT88	25428	ES	24	23	25	-0.01	2.4E-01	excluded
UBE3A	93616	ES	2:3:4.1:4.2:5.1:5.2	1	6.2	0.03	2.4E-01	included
TIMM50	49837	ES	2:3:4:6:7:8	1	9	0.03	2.4E-01	included
SIRT6	46775	ES	3	1	4	0.00	2.4E-01	excluded
TMEM175	68431	ES	4.1:4.2	3	5.1	0.02	2.4E-01	included
IFNAR2	60391	ES	09:10.1	8	10.2	0.00	2.4E-01	excluded
WWOX	37677	ES	7:08:09	5	13	0.00	2.4E-01	excluded
GALNT2	10235	ES	11	10	12	0.00	2.4E-01	included
RAP1GAP	992	ES	22.1	21	22.4	0.01	2.4E-01	included
PDGFC	70957	ES	2:03	1	4.1	0.01	2.4E-01	included
CTSB	97874	ES	2:3.1:3.2	1.1	5.3	0.00	2.4E-01	included
TNIP1	74136	ES	18:20	17	21.1	0.00	2.4E-01	excluded
SPIDR	83787	ES	5	4	8	0.00	2.4E-01	excluded
HNRNPA1	485363	ES	3:4:5:6.1:6.2:10:11.1:	2	11.3	0.01	2.4E-01	included
TMPO	23849	ES	6:07:08	5.1	9	0.01	2.5E-01	included
DMTF1	80308	ES	5	4.2	6	0.00	2.5E-01	included
PPP2R3C	27211	ES	2	1.3	3	0.00	2.5E-01	included
MAP4	64561	ES	15	14	16	0.00	2.5E-01	excluded
METTL15	14789	ES	7	3	8	0.00	2.5E-01	included
ZNF394	80653	ES	2	1	3	0.01	2.5E-01	included
DHX35	59386	ES	3	2	4	0.01	2.5E-01	included
MLLT4	78453	ES	32.1:32.2	31	33.1	0.00	2.5E-01	excluded
MYO5C	30649	ES	22:23	21	24	0.00	2.5E-01	included
EXT1	84991	ES	1.2:2	1.1	3	0.00	2.5E-01	excluded
SLC25A12	55957	ES	5	4	6	0.01	2.5E-01	included
ERCC8	72170	ES	3	2	4	-0.02	2.5E-01	excluded
DLG1	68294	ES	10	7	11	-0.01	2.5E-01	excluded
ANKRD6	77008	ES	6	5	7	0.01	2.5E-01	included
ATG7	63386	ES	17	16	18	0.00	2.5E-01	excluded
SNRPD2	50510	ES	04:05.1	1.3	5.2	-0.01	2.5E-01	excluded
RANBP3	46998	ES	2:4.1:5:7:8	1	10.2	-0.01	2.5E-01	excluded
POLR3C	7294	ES	12:13:14	11	15	0.00	2.5E-01	included
IP6K2	64771	ES	8.1:11.1	7	11.2	0.00	2.5E-01	included
ZNF273	79844	ES	2	1.2	3	-0.03	2.5E-01	excluded
MARC2	9876	ES	2	1	3	0.00	2.5E-01	excluded
PTMS	293234	ES	3.1:3.2:4	2	5	0.01	2.5E-01	included
FAM136A	53891	ES	1.3	1.1	2	0.01	2.5E-01	included
SPECC1L	61398	ES	15	14	16	0.00	2.5E-01	included
ALKBH6	49328	ES	1.3	1.1	2	0.01	2.5E-01	included
DYNC1I2	55954	ES	6	4	7.3	0.00	2.5E-01	included
MANBAL	59342	ES	03:04.1	1	4.2	0.00	2.5E-01	excluded
C2CD5	20734	ES	26:27.2	25	28	0.01	2.5E-01	included
GNPAT	10274	ES	2	1	3	0.00	2.5E-01	included
ETFA	31945	ES	3	1	4	-0.01	2.5E-01	excluded
AMDHD2	33283	ES	4	3	5	0.00	2.5E-01	included
UEVLD	14672	ES	5:06	4	7	0.00	2.5E-01	included
CADM1	18853	ES	9	8	10	0.00	2.5E-01	included
DPF2	16818	ES	4	3	5	0.00	2.5E-01	included
SLMAP	65411	ES	12	11	15.2	-0.02	2.5E-01	excluded
PDXK	192507	ES	3	2	4	-0.03	2.5E-01	excluded
TAB2	78073	ES	8	7	9	0.00	2.5E-01	excluded
AIDA	9893	ES	8:09	7	10	0.00	2.5E-01	excluded
ZNF302	48986	ES	3:4:5.2:6.1	2	6.2	0.00	2.5E-01	excluded
STX3	16035	ES	13	11	14	0.00	2.5E-01	excluded
SGCE	80509	ES	2	1	4	0.00	2.5E-01	included
CLCN3	71153	ES	14	13	15	0.02	2.5E-01	included
AMACR	71705	ES	3	2	4.1	-0.01	2.5E-01	excluded
ADAM10	30900	ES	2:3:4:5:6:7:8.1:8.2:9.1 :9.2:10:11:12:13:14:1	1	16.2	0.00	2.5E-01	excluded

FAM32A	48145	ES	2	1.3	3	0.00	2.5E-01	included
ALG3	67854	ES	3	2.2	4	0.00	2.5E-01	included
NAE1	36861	ES	3.1:4:5:6	1	7	0.00	2.5E-01	excluded
GNB2L1	75088	ES	2.1:2.2:2.3:3:5:6:7.2:8	1.1	8.2	0.00	2.5E-01	excluded
CHEK2	61542	ES	5:06	3	9	0.00	2.5E-01	excluded
UBXN1	16398	ES	8.1	7	8.3	0.00	2.5E-01	included
TCEA3	1063	ES	2.2	1	3	0.00	2.5E-01	excluded
ERO1LB	10371	ES	6	5	7.1	0.00	2.5E-01	excluded
PHF17	70585	ES	6	5	7.1	0.00	2.5E-01	included
NSL1	9746	ES	5	4	6	0.01	2.5E-01	included
GCAT	62159	ES	2.2	1	3	0.00	2.5E-01	included
MAOA	88873	ES	2	1	3	-0.01	2.5E-01	excluded
TMBIM1	57474	ES	3	1	4	0.00	2.5E-01	excluded
GABPB1	30576	ES	2:03	1	4	0.01	2.5E-01	included
HSCB	96110	ES	3.2:4	3.1	5	-0.02	2.5E-01	excluded
CC2D2A	68808	ES	32	31	33	0.00	2.5E-01	included
LIPT1	54676	ES	5	3	6	-0.02	2.5E-01	excluded
RUVBL2	50858	ES	14	13	15	0.00	2.5E-01	excluded
UBA3	65567	ES	4:5:6:7	3	8	0.00	2.5E-01	included
NIPA2	29681	ES	5	4	6	0.00	2.5E-01	included
ABHD4	26615	ES	4	3.3	5	0.00	2.5E-01	included
ZNF691	2132	ES	2.3	2.1	4	0.02	2.5E-01	included
RABL2A	55061	ES	6:07	5.2	8.1	-0.01	2.5E-01	excluded
RAB17	58124	ES	2.2:3:4:5:6.1	2.1	6.2	-0.01	2.5E-01	excluded
PPP1R12C	52020	ES	15.2:16:17.1:17.2	15.1	18	0.00	2.5E-01	excluded
ZNF584	52452	ES	4.2	3	5	-0.01	2.5E-01	excluded
KDM8	35675	ES	4:5:6:7:8	3	9	0.00	2.5E-01	excluded
NEMF	27449	ES	4.1	3	5	0.00	2.5E-01	excluded
PPP2R5C	29323	ES	7	5	10	0.01	2.5E-01	included
TMUB2	41805	ES	03:04.2	2.5	4.3	0.02	2.5E-01	included
MLPH	58117	ES	8:09	7	11	0.00	2.5E-01	included
PMF1	8204	ES	5	1	6	0.00	2.5E-01	excluded
POLK	72529	ES	14.1:14.2	13.1	15	0.00	2.5E-01	included
HAGH	33147	ES	2	1.2	3	0.01	2.5E-01	included
CTNND1	15933	ES	21:22.1	20	22.2	0.01	2.5E-01	included
ITPA	100225	ES	2	1.2	4	-0.03	2.5E-01	excluded
EEFSEC	66643	ES	4	3	5	0.00	2.5E-01	excluded
C21orf59	60364	ES	2	1	3	0.00	2.5E-01	included
TDRD10	7836	ES	8	7	9	-0.02	2.5E-01	excluded
WWOX	37680	ES	5:7:8:9	4	13	0.00	2.5E-01	included
NUP62	51131	ES	1.2:1.5	1.1	2.1	-0.01	2.5E-01	excluded
ACTR3	55078	ES	4:05	3	6	0.00	2.5E-01	included
IFI16	8400	ES	8	7	9	0.01	2.5E-01	included
CERS5	21660	ES	17.2	16	18	0.00	2.5E-01	excluded
GFRA2	82910	ES	4.2:5	3.2	6	0.00	2.5E-01	excluded
CENPK	72215	ES	10	9	11	-0.01	2.5E-01	excluded
CDK10	38116	ES	5	4	6	-0.02	2.5E-01	excluded
METTL9	34525	ES	2	1.2	3	0.00	2.5E-01	excluded
NXN	38281	ES	7	6	8	0.00	2.5E-01	included
KLC1	29476	ES	15	13.3	18	-0.01	2.5E-01	excluded
ERBB2IP	72255	ES	26	25	27	0.00	2.5E-01	included
MRPL55	10098	ES	2.4:2.5	2.2	2.9	0.00	2.5E-01	included
CDC14A	3884	ES	17	16.1	18	-0.01	2.5E-01	excluded
ZNF655	80695	ES	8	3.2	9	-0.03	2.5E-01	excluded
SLMAP	65395	ES	19:20	18.1	21	0.00	2.5E-01	excluded
PSMA6	27232	ES	3	2.1	4	0.00	2.5E-01	excluded
CENPT	37132	ES	8.1:8.2:9	7.2	10.1	0.00	2.5E-01	included
DNAJA4	32077	ES	5	2.3	6.1	0.00	2.5E-01	included
ARHGAP27	41967	ES	10	9.2	11	0.00	2.5E-01	excluded
PLXDC2	10958	ES	3	2	4	0.00	2.5E-01	excluded
LTBP3	16866	ES	2:3:4:5:6	1.2	7	0.00	2.5E-01	included
DMKN	49200	ES	7:11	6.4	12	0.02	2.5E-01	included

MDM2	22973	ES	9:10:11	8.2	12.1	0.00	2.5E-01	excluded
ELMOD3	54212	ES	7.3	7.1	8	-0.01	2.5E-01	excluded
MTO1	76750	ES	5	3	6	0.01	2.5E-01	included
KCNN4	50238	ES	03:04.1	1	4.2	0.01	2.5E-01	included
APOO	88704	ES	4	3	5.1	0.00	2.5E-01	excluded
SEPT10	54909	ES	4:05:06	3.1	7	0.00	2.5E-01	included
RASA4	81122	ES	17	16	18	0.02	2.5E-01	included
CPNE1	59196	ES	1.2:2.2	1.1	3	0.00	2.5E-01	included
TBC1D5	63667	ES	5	1	7	0.01	2.5E-01	included
ENDOV	44064	ES	6.2	2.4	7	0.02	2.5E-01	included
GLS2	22453	ES	2:3:4.1:4.2	1	5	0.00	2.5E-01	excluded
CHMP3	54441	ES	5	4	6.1	0.00	2.5E-01	included
ECHDC1	77462	ES	8	7	10.2	0.00	2.5E-01	excluded
PSME3	41149	ES	11	10	12.2	0.00	2.5E-01	included
DYNC1LI1	63817	ES	3:04	2	5	0.00	2.5E-01	excluded
BABAM1	48256	ES	4:05:06	3	8	0.00	2.5E-01	included
PHB2	20040	ES	8	7.1	9.2	0.00	2.5E-01	included
OASL	24820	ES	4	3	5	-0.01	2.5E-01	excluded
HIVEP1	75359	ES	6	5	7	0.01	2.5E-01	included
SDHAF2	16225	ES	5.2	4.1	6	0.00	2.5E-01	excluded
PTS	18762	ES	4:05	3	6	0.00	2.5E-01	excluded
MAMDC4	88226	ES	15:16	14	17	0.01	2.5E-01	included
UGP2	53758	ES	7	6	9	0.00	2.5E-01	excluded
FAM179B	27391	ES	8	7	9	0.02	2.5E-01	included
PLSCR1	67161	ES	8.1:8.3	7	9.1	0.00	2.5E-01	excluded
GTF2H1	14604	ES	4	3	5	0.00	2.5E-01	included
HIRA	61052	ES	18:19:20:21	17	22	0.00	2.5E-01	excluded
HACE1	77104	ES	17:18:19:20:21	15	22	0.00	2.5E-01	excluded
FAS	12479	ES	7	6	8	0.00	2.5E-01	included
STAU1	59740	ES	3:04	1	5	-0.03	2.5E-01	excluded
ARNT	7515	ES	2	1	3	0.01	2.6E-01	included
CLN3	35725	ES	9:10	8	11	0.00	2.6E-01	included
DDX58	86065	ES	3	1	5	0.00	2.6E-01	excluded
SNX11	42172	ES	5:06	4	7	0.00	2.6E-01	excluded
DCAF4	28235	ES	8:9:10.1:10.2:11.1:11.	7.2	12	0.00	2.6E-01	included
ZNF28	51628	ES	5	3	7.1	-0.02	2.6E-01	excluded
NBPF10	7270	ES	3.2:4.1:4.2:5:6:7:8	3.1	9	0.00	2.6E-01	included
UBXN4	55450	ES	3.1:3.2:3.3	2	4	0.02	2.6E-01	included
POLR3H	62431	ES	5.1:5.2	3	6	0.01	2.6E-01	included
SAP30L	74219	ES	2	1	3.1	0.00	2.6E-01	excluded
TMX2	15913	ES	3.3:4	2	5.1	0.00	2.6E-01	included
RBM7	18827	ES	2:3.1:3.2	1	4.1	0.00	2.6E-01	excluded
CIRBP	46443	ES	7.2:7.3	5.2	7.5	0.00	2.6E-01	excluded
BRCA1	41191	ES	15.1:15.2:16:17:18:19	13	20	0.00	2.6E-01	included
RBM7	18826	ES	2	1	3.1	0.00	2.6E-01	excluded
PHB2	20051	ES	3.1:4.2:5.1:5.3:6.1:6.2	2	7.1	0.01	2.6E-01	included
RANBP1	61141	ES	5	4	6	0.00	2.6E-01	excluded
MNAT1	27782	ES	6	5	7	0.00	2.6E-01	included
MKS1	42654	ES	3	2	4	0.00	2.6E-01	excluded
PCYT2	44234	ES	1.3	1.1	2	0.00	2.6E-01	included
HCLS1	66394	ES	7	6	8	0.00	2.6E-01	excluded
ARMC8	66967	ES	3	1	4	0.02	2.6E-01	included
ST3GAL3	2223	ES	14:15.1:16.2	13	19.1	0.00	2.6E-01	excluded
ELMO2	59679	ES	9.1:9.2	8	10	0.00	2.6E-01	excluded
DDB2	15676	ES	3	2	4	0.00	2.6E-01	excluded
ILK	14173	ES	3	1.4	4	0.00	2.6E-01	included
GPATCH4	8297	ES	5.4	5.1	6	0.00	2.6E-01	excluded
PRKRIR	17887	ES	3	2	4	-0.01	2.6E-01	excluded
ARHGEF9	89308	ES	2.4:4	2.3	5	-0.02	2.6E-01	excluded
PELP1	38556	ES	4	3	5	0.00	2.6E-01	excluded
COASY	41071	ES	1.3	1.1	1.5	0.00	2.6E-01	included
TUFT1	7635	ES	6	5	7	0.00	2.6E-01	excluded

MST1R	64927	ES	11	10	12	-0.02	2.6E-01	excluded
FKBP10	40946	ES	6.3:6.4	6.1	7	0.00	2.6E-01	excluded
CTNS	38478	ES	3:04:05	2.2	7	0.00	2.6E-01	excluded
RRBP1	58739	ES	3.1:3.2:3.3:3.4	2	4	0.00	2.6E-01	excluded
RBM22	74108	ES	3.2:4	3.1	6.2	0.00	2.6E-01	excluded
AKNA	87321	ES	7	6	8	0.00	2.6E-01	included
RWDD4	71279	ES	2:3.1:3.2	1	4	0.00	2.6E-01	included
RIC8B	24165	ES	13:14:15:16	11	17	0.02	2.6E-01	included
DLG1	68290	ES	21	20.1	23	0.00	2.6E-01	included
PQLC1	46258	ES	7:09	5	10	-0.01	2.6E-01	excluded
TRPC6	18430	ES	6	5	7	0.00	2.6E-01	included
PNPLA8	81411	ES	02:04.1	1	4.2	0.01	2.6E-01	included
VHL	63312	ES	2	1	3	-0.01	2.6E-01	excluded
PLRG1	70897	ES	7	6.2	8	0.00	2.6E-01	excluded
LGMN	29010	ES	04:05.1	3	5.2	0.00	2.6E-01	included
FAM189B	8047	ES	6.2:7:8:9	5	10	0.00	2.6E-01	excluded
WWP1	84362	ES	6:07:08	5	9	0.00	2.6E-01	included
ALPK1	70369	ES	12	11	13	0.00	2.6E-01	excluded
TP53	39040	ES	10.2	9	11	0.00	2.6E-01	excluded
UBE2D3	70129	ES	9.1:9.2	8.2	10	0.00	2.6E-01	excluded
HPSE	69785	ES	6	5	7	0.00	2.6E-01	included
C19orf43	47856	ES	2.2	1.2	3	0.00	2.6E-01	included
HNRNPA2B1	79039	ES	2	1	3	0.00	2.6E-01	excluded
ABCC1	34211	ES	20	19	21	0.00	2.6E-01	included
GAK	68419	ES	2:3:5.2:6	1	7	0.00	2.6E-01	included
SERINC4	115971	ES	5:7:8:9:10	4	11	-0.01	2.6E-01	excluded
NDUFV1	17253	ES	2	1.3	3	0.00	2.6E-01	excluded
GNG2	27528	ES	6.2	4	9	0.01	2.6E-01	included
IST1	37523	ES	6:7:8:9	5	10	0.00	2.6E-01	included
PVR	50341	ES	6.1:6.2	5	7	0.01	2.6E-01	included
SMC1A	89217	ES	2:03	1	4	0.00	2.6E-01	excluded
RFT1	65293	ES	3	2	4	0.00	2.6E-01	excluded
NUBP1	33989	ES	5	4	6	0.00	2.6E-01	excluded
SLC25A12	55959	ES	4	3	6	0.00	2.6E-01	included
FNBP1L	3791	ES	10	9	12	0.00	2.6E-01	included
FBXO28	9933	ES	4	3	5	0.00	2.6E-01	included
HNRNPUL1	50037	ES	8:09	7	10	0.00	2.6E-01	included
ENO2	20014	ES	4:05	3	6	0.00	2.6E-01	included
PDXDC1	34117	ES	10	9	11	0.00	2.6E-01	included
CSNK1G3	73146	ES	11	10	12	-0.01	2.6E-01	excluded
CCT6B	40225	ES	3	2	4	-0.02	2.6E-01	excluded
NVL	9946	ES	7	6	8	0.00	2.6E-01	included
C2ORF15	54674	ES	3	2	4	-0.01	2.6E-01	excluded
PBX3	87591	ES	7	6	8	0.00	2.6E-01	included
FBXL12	47422	ES	3.2	2.4	5	-0.01	2.6E-01	excluded
CD99L2	90360	ES	4:05:06	2	7	0.00	2.6E-01	included
SF3B1	56680	ES	4.3	4.1	5	0.00	2.6E-01	included
NSUN7	69094	ES	11	10	12	-0.01	2.6E-01	excluded
ENOSF1	44468	ES	6:07	5	8	0.02	2.6E-01	included
DAG1	64886	ES	3:04	2.1	7	0.00	2.6E-01	included
LIAS	69063	ES	3:4:5:6	2	7	0.01	2.6E-01	included
MMAA	70764	ES	5	4	6	0.01	2.6E-01	included
PTTG1IP	60841	ES	4:05:06	2	7	0.00	2.6E-01	included
EEF1D	98105	ES	7.2	1	8.1	0.00	2.6E-01	included
MAN2C1	31868	ES	7.1:7.2:8	6	9	0.00	2.6E-01	included
R3HDM4	46352	ES	2:3.1:3.3	1	3.4	0.01	2.6E-01	included
POLDIP3	62538	ES	3.1:3.2	2	4	0.03	2.6E-01	included
MIIP	697	ES	8	7	9	0.00	2.6E-01	included
TOE1	2677	ES	4	3	5	0.00	2.6E-01	included
EIF4G1	67921	ES	2.2:2.4:3.2	1	5	0.01	2.6E-01	included
SAMHD1	59331	ES	4	3	5	0.00	2.6E-01	excluded
PIP4K2C	22654	ES	4	3.2	6	0.00	2.6E-01	excluded

ARMC10	81153	ES	9	8	10	0.00	2.6E-01	excluded
AGBL3	81869	ES	18:19	17	20.1	-0.02	2.6E-01	excluded
POMT1	87955	ES	2	1.1	3	-0.01	2.6E-01	excluded
TMED5	3778	ES	4.3	4.1	5	0.00	2.6E-01	excluded
LONP1	46918	ES	1.5:1.6:1.7:2:3:4.1	1.4	4.2	0.00	2.6E-01	included
ACBD4	41947	ES	4	3.5	5	0.00	2.6E-01	included
DERL2	38709	ES	3:4.1:4.2:4.3:5.2	2	6	-0.02	2.6E-01	excluded
MDM2	23164	ES	3	2.1	5.1	0.00	2.6E-01	included
PNISR	100610	ES	9.3	9.1	10	0.01	2.6E-01	included
PXMP2	25288	ES	4:05	3.2	7.1	0.00	2.6E-01	excluded
MTRR	71537	ES	4.1:4.2	2	5	-0.02	2.6E-01	excluded
RBM4B	17110	ES	4	2.2	5	0.00	2.6E-01	excluded
GFER	33181	ES	2.2	1	3	0.00	2.6E-01	included
NAT9	43301	ES	05:06.1	4	6.2	0.01	2.6E-01	included
BEND5	101576	ES	4	3	5	0.01	2.6E-01	included
MPDU1	39004	ES	5.2	3.2	6.1	0.00	2.6E-01	included
MVP	35960	ES	3	2.3	4	0.00	2.6E-01	excluded
CLASP2	63878	ES	29	28	30	0.00	2.6E-01	excluded
NSRP1	40084	ES	5:06	3	8	-0.03	2.6E-01	excluded
OLFML3	4321	ES	2	1	3	0.00	2.6E-01	included
TKT	65301	ES	6.1:6.2:7.1	5	7.2	0.00	2.6E-01	included
EDEM2	59070	ES	7	6	8.2	0.00	2.6E-01	included
PTPRS	46844	ES	15:16:18:19	14	20	-0.01	2.6E-01	excluded
SERGEF	14556	ES	13	12	14	0.00	2.6E-01	excluded
TCTN1	24472	ES	6.1:6.2	5	7.1	0.00	2.6E-01	excluded
FTSJ1	88968	ES	2:3:4:5	1	6	0.00	2.6E-01	excluded
MBD1	45524	ES	8	7	9	0.00	2.7E-01	included
CDH5	36705	ES	3.2:4:5:6.1	3.1	6.2	0.00	2.7E-01	excluded
AMT	64865	ES	5	4	6	0.00	2.7E-01	excluded
PRKACA	47959	ES	4:5:6:7:8:9:10:11.1	3	11.2	0.00	2.7E-01	included
OSBPL1A	44894	ES	3.2:4	3.1	6	0.00	2.7E-01	excluded
LSM1	83372	ES	3:04	2	5	0.00	2.7E-01	excluded
CCDC53	24023	ES	5	2	6	-0.01	2.7E-01	excluded
EIF4G3	954	ES	17	16.2	19	0.00	2.7E-01	included
RAB17	58125	ES	2.2:2.3:4:5:6.1	2.1	6.2	0.00	2.7E-01	excluded
DDX42	42992	ES	3	1.3	4	0.00	2.7E-01	excluded
ABHD11	80026	ES	4	3	5	0.00	2.7E-01	included
PML	31649	ES	6.1:6.4:6.6:6.7	4	6.8	0.01	2.7E-01	included
ZSWIM7	39402	ES	2	1.2	4	0.00	2.7E-01	included
GANAB	16379	ES	3	2.2	4	0.00	2.7E-01	included
EIF5A2	67622	ES	4.1:4.2	3	5	0.00	2.7E-01	included
EIF1AD	16969	ES	1.2:2.3:2.4	1.1	3.1	0.01	2.7E-01	included
RTN3	16530	ES	2:3.1:3.2	1	4	0.00	2.7E-01	excluded
SNCA	69931	ES	5	4.2	6.1	0.00	2.7E-01	excluded
ASAH1	82845	ES	8	7	9	0.00	2.7E-01	included
ACAD9	66674	ES	1.2:2.1	1.1	2.2	-0.01	2.7E-01	excluded
SARS2	49730	ES	14	13	15	0.00	2.7E-01	included
CHID1	13814	ES	6.1:6.2:6.3	5.2	7	-0.01	2.7E-01	excluded
ACAD10	24535	ES	10	9	11	0.00	2.7E-01	excluded
PIGX	68259	ES	7	6.1	8	0.00	2.7E-01	included
NASP	2742	ES	5:6:7.2:8	3	10	0.00	2.7E-01	included
SHC1	7857	ES	2.3:2.4:3:4:5.1	2.2	5.2	0.00	2.7E-01	excluded
SEC24C	12176	ES	8:09:10	7	11	0.00	2.7E-01	included
CBR4	71140	ES	2.1:2.2:3	1	4	0.03	2.7E-01	included
SMN2	72409	ES	9.1	7	10	0.01	2.7E-01	included
CLUAP1	33587	ES	12.3	12.1	12.5	0.00	2.7E-01	included
TIMM23	11550	ES	4:5.1:5.2	3	6	0.00	2.7E-01	excluded
TSPAN4	13799	ES	5.2	3	7	0.00	2.7E-01	included
ECHDC2	3028	ES	3	2.1	5.1	0.02	2.7E-01	included
PPP6R3	17325	ES	2:03	1	4	0.00	2.7E-01	excluded
PRKRIP1	81088	ES	9	8	10	0.00	2.7E-01	included
TMEM50B	60421	ES	3:04	2	5	0.00	2.7E-01	excluded

SLC37A3	81984	ES	13	12	14	0.00	2.7E-01	included
ZSCAN32	33556	ES	04:05.2	2.2	6.1	-0.02	2.7E-01	excluded
PEX1	80440	ES	3:04:05	2	6	0.00	2.7E-01	excluded
SQSTM1	74938	ES	9.2:10:11.1	9.1	11.2	0.00	2.7E-01	excluded
NOB1	37309	ES	3	2	4	0.00	2.7E-01	included
C19orf82	47381	ES	2:03	1	4.1	-0.03	2.7E-01	excluded
PFDN5	22011	ES	2	1	5	0.01	2.7E-01	included
FAM184A	77363	ES	17	16	18	-0.01	2.7E-01	excluded
C11orf30	17892	ES	21	20	22	0.00	2.7E-01	excluded
MPV17	52968	ES	9:10	8	12	0.00	2.7E-01	included
MFN1	67691	ES	13:14	12	15	0.00	2.7E-01	included
PAICS	69359	ES	4	3	5	0.00	2.7E-01	included
ARF3	21502	ES	2	1	3	0.00	2.7E-01	included
FAM160B2	82936	ES	2	1	3	-0.02	2.7E-01	excluded
TRAPPC4	19069	ES	2.1:2.2:2.3:2.4:3.1:3.2	1	4.1	-0.02	2.7E-01	excluded
DMKN	49176	ES	8:09:11	7	12	0.02	2.7E-01	included
HSP90AA1	29334	ES	5	4	6	0.00	2.7E-01	excluded
CASD1	80490	ES	3	2	4	0.00	2.7E-01	excluded
LRRC28	32708	ES	3:05:06	2	7.1	0.00	2.7E-01	excluded
NDUFA12	23735	ES	4	3	5.1	0.00	2.7E-01	excluded
TSGA10	54667	ES	7	5	8	0.01	2.7E-01	included
MARCH8	11363	ES	2	1	4	-0.02	2.7E-01	excluded
MEGF6	315	ES	31	30	32	-0.02	2.7E-01	excluded
KLHDC3	76214	ES	3	2.2	4	0.00	2.7E-01	excluded
CCDC47	42984	ES	2	1	3	0.00	2.7E-01	excluded
UBE2J2	52	ES	4	2.1	5	0.00	2.7E-01	excluded
COMT	61103	ES	5	4	6.2	0.00	2.7E-01	included
CCDC148	55663	ES	7	6	8	-0.01	2.7E-01	excluded
TMEM258	16284	ES	3.1:3.2	2.3	4	0.01	2.7E-01	included
UTP15	72487	ES	2	1	3.1	0.00	2.7E-01	excluded
ZNF83	51512	ES	08:09.6	6.3	10.1	0.02	2.7E-01	included
STAG3	80919	ES	5:06	4	7	0.00	2.7E-01	included
RPL13	38088	ES	02:03.1	1.4	3.2	0.00	2.7E-01	excluded
SMS	88680	ES	3:04	2	5	0.00	2.7E-01	included
RRAGD	76999	ES	2	1	3	0.00	2.7E-01	included
DNTTIP2	3797	ES	3	2	4	0.00	2.7E-01	excluded
MAPKAP1	87580	ES	13	12	14	0.00	2.7E-01	included
CSDE1	4335	ES	4	2	5	0.00	2.7E-01	included
PAF1	49811	ES	8	7	9	0.00	2.7E-01	included
PPARG	63420	ES	12	11	13	0.00	2.7E-01	excluded
MUTYH	2612	ES	6.2:6.3:6.4:6.5:7:8:9:10:11:12:13:14:15:16:1	5	19	0.01	2.7E-01	included
IDNK	86687	ES	4	1.2	5	0.02	2.7E-01	included
CUX1	81077	ES	6	5	7	0.00	2.7E-01	included
MRPL55	10145	ES	2.5:2.6:2.8	1.1	2.9	0.00	2.7E-01	excluded
SIRT2	49712	ES	5	4	7	0.00	2.7E-01	included
RCAN1	60497	ES	6	5	7.2	0.01	2.7E-01	included
ELMO2	59683	ES	3	2	4.1	0.02	2.7E-01	included
PRMT5	26670	ES	1.2:2	1.1	3	0.01	2.7E-01	included
SLTM	30917	ES	8	7	9	0.01	2.7E-01	included
USP25	60221	ES	20	18	21	0.01	2.7E-01	included
MAL	54484	ES	2:03	1	4	0.00	2.7E-01	included
USP39	54321	ES	4	3	5.1	0.00	2.7E-01	excluded
R3HDM4	46349	ES	2	1	3.1	0.02	2.7E-01	included
ATP6V1E1	60981	ES	2	1	3	0.00	2.7E-01	included
SUMF2	79818	ES	2	1	3	0.00	2.7E-01	included
CYB5RL	3130	ES	8:09	7	10	-0.01	2.7E-01	excluded
TMEM70	84217	ES	3	2.1	4	0.01	2.7E-01	included
DTNA	45111	ES	20	17	22	0.00	2.7E-01	excluded
LYRM5	20816	ES	2.1	1.1	3.1	0.00	2.7E-01	excluded
C1orf27	9227	ES	9	8	10	0.00	2.7E-01	included
GRAP	39688	ES	6	4	7	0.00	2.7E-01	included

FTSJ2	78605	ES	4	2	5	0.01	2.7E-01	included
RABGEF1	79898	ES	5	3	6	0.02	2.7E-01	included
GOT2	36688	ES	3.1	2	4	0.00	2.7E-01	excluded
MRPL55	10095	ES	2.4:2.5:2.6	2.2	2.9	0.00	2.7E-01	included
POLK	72525	ES	14.1:14.2:15	13.1	16	0.00	2.7E-01	included
KIAA0226L	25830	ES	16	15	17	0.00	2.7E-01	excluded
ZNF562	47394	ES	2.1:2.2:3	1	4	0.00	2.7E-01	included
SFXN2	12978	ES	2.1:2.2	1	4.1	-0.02	2.7E-01	excluded
CHD1L	7376	ES	10:11:12	9	13	0.00	2.7E-01	excluded
PICALM	18168	ES	19:20	18	21	0.00	2.7E-01	excluded
DHX40	42781	ES	2.2:3	2.1	4	0.00	2.7E-01	included
CASK	88864	ES	20:21	19.1	22	-0.02	2.7E-01	excluded
CLEC4A	20180	ES	2:03	1	4	0.01	2.7E-01	included
RBM39	59237	ES	20	19.2	21	0.00	2.7E-01	excluded
ACADM	3489	ES	6:07	4.2	8	0.00	2.7E-01	excluded
ADH5	70027	ES	4.1	3	5.1	0.00	2.7E-01	excluded
ABCA3	33267	ES	11	10	12	0.00	2.7E-01	included
MARK3	29447	ES	17	16	18	0.02	2.7E-01	included
ITGA2	71991	ES	20	19	21	0.00	2.7E-01	included
EMID1	61577	ES	2:3:4:5	1	6	0.00	2.7E-01	excluded
SNF8	42269	ES	05:07.1	2	9	0.00	2.7E-01	included
ASUN	20853	ES	2:03	1	4	0.00	2.7E-01	excluded
TFPT	51790	ES	4	3	5	0.00	2.7E-01	excluded
AUH	86824	ES	4	3	5	0.00	2.7E-01	excluded
NECAP2	827	ES	2	1.2	3	0.00	2.7E-01	included
CD302	55705	ES	4	3	5	-0.01	2.7E-01	excluded
RANBP3	46996	ES	4.1:5:6:7:8	1	10.2	-0.03	2.7E-01	excluded
FOLH1	15819	ES	6	5	7.1	0.00	2.8E-01	excluded
EIF4G3	956	ES	16.2	15	17	0.00	2.8E-01	excluded
RARA	40860	ES	6:07	3	8	0.00	2.8E-01	excluded
OSBPL9	2974	ES	11	9.2	12	0.00	2.8E-01	included
ELAC2	39329	ES	7	6	8	0.00	2.8E-01	included
PCNX	28192	ES	26	25	27	0.00	2.8E-01	included
SPATA6	2923	ES	4	3	5.2	0.00	2.8E-01	included
CD74	74079	ES	06:07.1	4	9	0.00	2.8E-01	included
ADCY4	26974	ES	8.1:8.2	7	9	0.01	2.8E-01	included
UMPS	66530	ES	5:06	4.2	7	0.00	2.8E-01	excluded
MGLL	66625	ES	7	6	8	0.01	2.8E-01	included
LRRC28	32711	ES	3	2	5	-0.01	2.8E-01	excluded
ATG4A	89848	ES	10	9.2	11	0.00	2.8E-01	included
ENTPD1	12654	ES	6	5	7	0.00	2.8E-01	included
ASB7	32738	ES	5.1	4	6	0.00	2.8E-01	excluded
CAMLG	99505	ES	2	1	4	0.01	2.8E-01	included
MKS1	42653	ES	4:05	3	6.2	0.00	2.8E-01	excluded
TUSC2	65016	ES	2.2	1	3.1	0.00	2.8E-01	included
RWDD4	71277	ES	02:03.1	1	3.2	0.00	2.8E-01	included
CCDC25	83176	ES	9	8.1	10	0.00	2.8E-01	included
SPRY4	73867	ES	2	1	3	0.01	2.8E-01	included
UBAP2	86136	ES	12	11	13	0.01	2.8E-01	included
POR	80141	ES	6.2:7:9.1	6.1	9.2	0.00	2.8E-01	excluded
HAUS1	45386	ES	2.2:4:5:6	2.1	7	-0.03	2.8E-01	excluded
RNF216	78684	ES	3	2	4	0.00	2.8E-01	excluded
KIAA0430	34178	ES	8.1:8.2:8.3	7	9	-0.01	2.8E-01	excluded
PI4KB	7594	ES	2:04:05	1	6	0.00	2.8E-01	excluded
TRO	89258	ES	3	2.2	4	0.01	2.8E-01	included
GANC	30144	ES	6	5	7	0.01	2.8E-01	included
DCTD	71242	ES	3	1.2	5	-0.01	2.8E-01	excluded
CNOT8	74263	ES	1.5:2	1.4	3	0.01	2.8E-01	included
CYTH1	43894	ES	10:11.1:13.2	9	14	0.00	2.8E-01	included
MTX2	56124	ES	2	1	3	-0.01	2.8E-01	excluded
PALM	46315	ES	8	7	9	0.01	2.8E-01	included
COX4I2	58897	ES	4	3	5	-0.01	2.8E-01	excluded

NDUFB2	82026	ES	7	3	10	0.00	2.8E-01	included
RPS6KC1	9782	ES	9	8	10	-0.02	2.8E-01	excluded
PRUNE	7547	ES	3:04:05	1	6	0.00	2.8E-01	included
PPOX	8577	ES	4:5:6:7:8:9	3	10	0.00	2.8E-01	excluded
CYP20A1	57006	ES	6.2	5	7	-0.01	2.8E-01	excluded
NEK11	66784	ES	20	19	21	0.01	2.8E-01	included
PFDN5	22000	ES	2:4.1:4.2:5	1	6.2	0.01	2.8E-01	included
AP1S3	57766	ES	3	2.1	6.1	0.00	2.8E-01	included
TATDN1	85088	ES	03:04.1	2	5	0.00	2.8E-01	included
ATG16L2	17659	ES	4.3:5:6.1:6.3:6.4:7:8:9:10:11:12:13	3	14	0.01	2.8E-01	included
PDHX	14973	ES	5:6:7:8:9	4	10	0.01	2.8E-01	included
EFCAB6	62588	ES	11:12	10	13.1	-0.01	2.8E-01	excluded
CPNE1	59204	ES	1.2:2.2	1.1	5	-0.01	2.8E-01	excluded
SEC62	67598	ES	2	1	3	0.00	2.8E-01	excluded
INPP4A	54634	ES	5.2:6:7:8:9:10:11:12:13:16.2:17:18.1:18.2:19	5.1	22.2	0.01	2.8E-01	included
FAM118B	19373	ES	2	1	3	-0.01	2.8E-01	excluded
LCN10	123361	ES	3	2.2	4.2	0.02	2.8E-01	included
SNRPG	53885	ES	6	5	7	0.00	2.8E-01	excluded
CASP10	56805	ES	8:09	7	10	0.00	2.8E-01	included
DAPK2	31074	ES	13.4:14:15.1	13.3	15.2	0.00	2.8E-01	included
TAGLN	18898	ES	1.3	1.1	2.3	0.00	2.8E-01	included
CD74	74083	ES	3	2	4	0.00	2.8E-01	included
PTPRB	23389	ES	9	8	10	0.00	2.8E-01	included
ULK3	31766	ES	2.2:2.3:3.1	1	3.2	0.00	2.8E-01	included
NACA	22497	ES	3.1:3.3	2.2	4.2	0.00	2.8E-01	included
UBL4A	90617	ES	2	1	3.2	0.00	2.8E-01	included
TPPP3	37061	ES	1.3	1.1	2.2	-0.01	2.8E-01	excluded
STK25	58388	ES	2.2:3.2	2.1	4	0.00	2.8E-01	excluded
ERC1	19655	ES	22	21	23	0.00	2.8E-01	excluded
ZFAND4	11370	ES	9.1:10:11	7	12	0.01	2.8E-01	included
SGCE	80497	ES	14	13.1	15	0.00	2.8E-01	excluded
SUGP2	48554	ES	8	7	9.1	0.00	2.8E-01	included
TLE3	31416	ES	7.2:8.1	7.1	8.2	-0.02	2.8E-01	excluded
C14orf80	29665	ES	7	6.2	8	0.00	2.8E-01	included
TFDP2	67095	ES	7	6	8	-0.01	2.8E-01	excluded
ACSF3	38065	ES	1.2:3.2	1.1	4	0.01	2.8E-01	included
FGFR2	13315	ES	14:15	7	17.1	0.00	2.8E-01	excluded
IDE	12535	ES	2:3:4:5	1	6	-0.04	2.8E-01	excluded
TOMM7	78941	ES	4	3	5	0.00	2.8E-01	excluded
COQ9	36558	ES	4.2:5	4.1	6	0.00	2.8E-01	excluded
ZNF419	52271	ES	4.1	3.2	5.1	0.04	2.8E-01	included
HAUS1	45383	ES	2.2:4:5	2.1	6	-0.03	2.8E-01	excluded
SNED1	58309	ES	27.1	26	28	-0.01	2.8E-01	excluded
M1AP	54124	ES	5	4	6	-0.01	2.8E-01	excluded
HERC4	11915	ES	23:24	22	25	0.00	2.8E-01	excluded
CEP19	68254	ES	2	1	3	-0.01	2.8E-01	excluded
GPS1	44288	ES	1.3:1.4:1.5	1.1	2.3	0.00	2.8E-01	excluded
MOCS1	76067	ES	11.1:11.2:12.1	10	12.2	0.00	2.8E-01	included
RPS2	33173	ES	4	3	5	0.00	2.8E-01	excluded
SIGLEC7	51311	ES	2:3:4:5	1	6	-0.01	2.8E-01	excluded
PSMD9	24916	ES	2.2:3.1	1	4	0.00	2.8E-01	included
ANAPC15	17572	ES	3.2:4.1:4.2	1.2	5	0.00	2.8E-01	excluded
CAMK2B	79495	ES	15	14.2	17	0.00	2.8E-01	excluded
ARHGAP5	27133	ES	03:04.1	2	4.2	-0.01	2.8E-01	excluded
MLF1	67430	ES	8	7	9.1	0.00	2.8E-01	included
CYTH1	43892	ES	10:11.1:11.2:13.1:13.2	9	14	0.00	2.8E-01	included
FAM193A	68582	ES	19	18	21	0.00	2.8E-01	excluded
PDK1	55980	ES	10	9	11	-0.01	2.8E-01	excluded
DONSON	60447	ES	4.1:4.2	3.2	5	0.00	2.8E-01	included
BCL6	68082	ES	8	7	9	0.00	2.8E-01	included

GAB3	90658	ES	9	8	10	0.01	2.8E-01	included
TFCP2	21802	ES	6	5	7	0.00	2.8E-01	included
RPS6KB1	42839	ES	5	4	6.2	0.00	2.8E-01	excluded
C2orf44	52799	ES	3	2	4	-0.01	2.8E-01	excluded
FRMD8	16850	ES	4	3	5	0.00	2.8E-01	excluded
FUK	37391	ES	10	9	11	-0.01	2.8E-01	excluded
PELP1	38552	ES	14.2:15:16.1	14.1	16.2	0.00	2.8E-01	excluded
NAV2	14697	ES	34	33	35	0.01	2.8E-01	included
C17orf80	43223	ES	4	3	5	0.00	2.8E-01	excluded
DHX40	42778	ES	8:09:10	7	11	0.00	2.8E-01	excluded
NPRL3	32809	ES	3:04	2	6	0.02	2.8E-01	included
ZBTB7B	101303	ES	3:04:05	2.2	6.2	-0.02	2.8E-01	excluded
GATSL3	61706	ES	6:07:08	5	9	0.00	2.8E-01	included
NPHP1	54929	ES	20	19	21	-0.01	2.8E-01	excluded
PDE9A	60720	ES	7	5	8	0.01	2.8E-01	included
TBXAS1	81969	ES	19	18	20	-0.01	2.8E-01	excluded
WDR7	45633	ES	2:3:4:5:6:7:8:9:10:11:12:13:14:15:16:18:19:20:21:22:23:24:25:26:	1	28	0.00	2.8E-01	excluded
TMEM159	34428	ES	5	4	7	0.00	2.8E-01	included
SMIM8	76949	ES	1.2:2.1:2.2	1.1	3	0.00	2.8E-01	included
DCAF8	8449	ES	8.1:8.2	7.1	9	-0.01	2.8E-01	excluded
HSD11B1L	46873	ES	7	5.3	8	-0.02	2.8E-01	excluded
THOC6	33359	ES	12	11	13	0.00	2.8E-01	included
ZNF451	76585	ES	13:14:15:16.1	12	17	0.00	2.8E-01	excluded
ANKRD49	18353	ES	2.6	2.2	2.8	0.01	2.8E-01	included
VPS33A	24942	ES	2	1	3	0.00	2.8E-01	excluded
TRIM16	39366	ES	4:05	3	6.2	-0.01	2.8E-01	excluded
SLC17A5	76762	ES	9	8	10	0.00	2.8E-01	included
LIAS	69061	ES	10	9	11	0.00	2.8E-01	excluded
NEK4	65262	ES	2.1:2.2	1	3	0.00	2.9E-01	included
TXNRD1	24103	ES	10	6.2	11	0.00	2.9E-01	included
TYW3	3474	ES	3:04:05	1	6	0.00	2.9E-01	included
TBC1D15	23416	ES	8	7	9	0.00	2.9E-01	included
ANKRD50	70530	ES	2	1	3	0.00	2.9E-01	excluded
HNRNPC	26538	ES	8.2	7.2	8.4	0.00	2.9E-01	excluded
DNAJB5	86226	ES	2.3	1	2.5	-0.01	2.9E-01	excluded
YAF2	21136	ES	3.1:3.2:5.2:6:7:8	2	9.1	0.00	2.9E-01	included
SIN3B	48216	ES	10	9	11.2	-0.02	2.9E-01	excluded
NOL10	52664	ES	2	1	3	0.00	2.9E-01	included
IL32	33441	ES	1.4:1.5	1.1	1.9	0.01	2.9E-01	included
ME2	45553	ES	14:15	13	16	0.00	2.9E-01	included
ATP6V0A1	41052	ES	2.2:3:4:5:6.1:6.2:7:8:9	2.1	10.2	-0.01	2.9E-01	excluded
ATG13	15583	ES	14	12	15	0.00	2.9E-01	excluded
MARS	22604	ES	5.1:5.2:6	4	7.1	0.00	2.9E-01	included
ADAM9	83487	ES	17:18	16	19	0.00	2.9E-01	included
MRPL55	10167	ES	2.2	1.1	2.9	0.02	2.9E-01	included
GABARAPL1	20396	ES	2.7:2.8:2.9:2.10:2.12:2.13:2.14	2.6	3	0.00	2.9E-01	excluded
RAVER2	3282	ES	7.2:8:9:10.1	6	10.2	0.00	2.9E-01	included
TBC1D7	75380	ES	5	4.1	6.1	0.00	2.9E-01	included
CASP7	13170	ES	3.1:3.2	2	4	0.01	2.9E-01	included
CCNL1	67389	ES	4.1	3	5	-0.02	2.9E-01	excluded
COPS3	39468	ES	9	8	10	0.00	2.9E-01	included
NBPF10	7264	ES	17:18:19:20:21:22	16	23	0.00	2.9E-01	excluded
WDR41	72590	ES	2:04	1.1	5	0.00	2.9E-01	excluded
SCOC	70649	ES	4	3.2	6	0.00	2.9E-01	excluded
TMEM106C	21397	ES	3:4.1:4.2:5.1:5.2:6:7	2.2	8	0.00	2.9E-01	included
ABCB8	82295	ES	10	9	12	0.00	2.9E-01	excluded
DCUN1D4	69269	ES	12	11	13	-0.01	2.9E-01	excluded
PQLC3	52678	ES	5	4	7	0.01	2.9E-01	included
HFE	75599	ES	2.1:2.2	1	3	0.01	2.9E-01	included

RPL23	40608	ES	5.1:5.3:5.4	4	5.5	0.00	2.9E-01	included
FAM193B	74800	ES	7	6	8	-0.02	2.9E-01	excluded
GLB1	63834	ES	5:06	4.2	7	0.00	2.9E-01	excluded
FUK	37396	ES	2:3:4.2:5:6.1:7:8	1	9	0.00	2.9E-01	included
CANT1	43978	ES	2	1	3.1	-0.01	2.9E-01	excluded
PLEKHO1	7427	ES	6	5	7	0.00	2.9E-01	included
ZNF821	37500	ES	8.1	7.2	9.1	-0.01	2.9E-01	excluded
YBEY	60914	ES	3	2.2	4	0.01	2.9E-01	included
CYBB	88785	ES	5:06	4	7	0.00	2.9E-01	excluded
PLA2G6	62209	ES	6	5	7	0.00	2.9E-01	included
GNB5	30641	ES	6	5.1	7	0.00	2.9E-01	included
HNRNPA1	22150	ES	6.2:6.3:7.2:9.1	6.1	9.2	0.00	2.9E-01	excluded
NAA30	27654	ES	4	3	5	0.00	2.9E-01	excluded
SIRT3	13604	ES	3.1:3.2	2.2	4	-0.01	2.9E-01	excluded
SH3BP5	63572	ES	4:5.2:8:9.1:10:11.1	3	11.2	0.00	2.9E-01	included
BNIP1	74580	ES	3	2	4	-0.01	2.9E-01	excluded
LRTOMT	17542	ES	2.1:2.2:3:5	1	6.1	0.01	2.9E-01	included
TM7SF3	92999	ES	9	8	11	-0.02	2.9E-01	excluded
KLHDC4	37955	ES	6.2:7.1	6.1	7.2	0.00	2.9E-01	included
NDEL1	39192	ES	5.2:6:7:8:9:10:11:12.1	5.1	12.2	0.00	2.9E-01	excluded
RABL2B	62920	ES	4	3.2	5	0.00	2.9E-01	excluded
NKIRAS2	40984	ES	5.6:6	5.3	7	0.03	2.9E-01	included
BDH2	70180	ES	5	4.2	6	0.00	2.9E-01	excluded
IL1RAP	68108	ES	10.1	9	11	-0.01	2.9E-01	excluded
FDXR	43331	ES	04:06.3	3.2	6.4	0.00	2.9E-01	excluded
STAG1	66933	ES	29	28	30	0.00	2.9E-01	included
BMF	29947	ES	6.1:6.2	5.2	7	0.00	2.9E-01	excluded
LDLR	47624	ES	14	13	15	0.00	2.9E-01	included
CACFD1	88068	ES	3	2	4	0.00	2.9E-01	excluded
NAPA	50658	ES	2:03:04	1	5	-0.01	2.9E-01	excluded
VSTM2L	59364	ES	2:03	1	4	0.00	2.9E-01	excluded
VAMP7	90683	ES	6	5	7	0.00	2.9E-01	excluded
RAB4B	50004	ES	5	4	6	0.00	2.9E-01	included
MAX	27949	ES	5.3:5.4:5.5:5.6	5.1	5.8	0.00	2.9E-01	excluded
ANXA3	69642	ES	4	2	5	0.00	2.9E-01	included
MUC1	7963	ES	5	4.3	6.1	0.00	2.9E-01	included
INO80C	45178	ES	5.1:5.2:6:7	1	8	0.00	2.9E-01	included
TRMT6	58658	ES	3	2	4	0.00	2.9E-01	excluded
VPS29	24445	ES	02:03.1	1	5	0.00	2.9E-01	included
N4BP2L2	25599	ES	2.1:2.2	1.3	3.1	0.00	2.9E-01	included
RNPS1	33257	ES	2.3:2.4	1.1	3	0.00	2.9E-01	excluded
TBC1D4	26052	ES	15	14	16	0.00	2.9E-01	excluded
COQ6	28349	ES	5	4	6	-0.01	2.9E-01	excluded
EPHB6	82069	ES	6	5.3	7.1	-0.01	2.9E-01	excluded
APOBEC3C	62271	ES	2	1	3	0.00	2.9E-01	included
ETV7	75975	ES	2:03	1	4	-0.01	2.9E-01	excluded
PPIE	1905	ES	7.1:7.2	6	8	-0.02	2.9E-01	excluded
LMO7	26066	ES	10:11:12	9	13	0.01	2.9E-01	included
PSME3	41152	ES	5:06	4	7	0.00	2.9E-01	excluded
MRPL49	16789	ES	2	1	3.1	0.00	2.9E-01	included
C14orf159	28845	ES	13	12.2	14	0.00	2.9E-01	excluded
DGKA	22302	ES	13	12	14	0.00	2.9E-01	excluded
TMEM57	1189	ES	5:06	4	7	0.00	2.9E-01	included
DGCR8	61132	ES	8	7	9	0.00	2.9E-01	excluded
ZNF211	52310	ES	6	5	7	-0.02	2.9E-01	excluded
SYTL3	78284	ES	8:9:10:11:12	7	13	0.00	2.9E-01	excluded
TBC1D7	75381	ES	3	2.2	4.1	-0.01	2.9E-01	excluded
ATP5J	60268	ES	1.3:1.4:1.5:2	1.2	3	0.02	2.9E-01	included
ARHGAP44	39326	ES	7	6	8	0.00	2.9E-01	included
FTSJ1	88969	ES	3:04:05	1	6	0.00	2.9E-01	excluded
UBE2D4	79381	ES	2	1	3	-0.02	2.9E-01	excluded
KIAA1598	13243	ES	17:18	16	20.1	0.00	2.9E-01	included

QDPR	68856	ES	6	5	7	0.00	2.9E-01	included
IGHMBP2	17354	ES	2.1	1	3	0.00	2.9E-01	included
HDGFRP3	32273	ES	2:03	1	4	0.00	2.9E-01	excluded
CCDC57	44298	ES	2	1	3	-0.03	2.9E-01	excluded
MFSD7	68395	ES	4:05	3.2	6	0.01	2.9E-01	included
TEX30	26216	ES	5	4	6	-0.01	2.9E-01	excluded
ARFGAP2	15647	ES	4.2:5:6.2:7:8	3	9	0.00	2.9E-01	included
RPS6KB2	17207	ES	5.1:7	4	8	0.00	2.9E-01	included
MAP4	64560	ES	17	16	18	0.00	2.9E-01	excluded
ZBTB40	1009	ES	5:06	4	7	0.00	2.9E-01	included
ZNF83	51506	ES	08:09.6	7	10.1	0.03	2.9E-01	included
MORN2	53297	ES	4.1:4.2	3.2	5	0.00	2.9E-01	excluded
COL1A1	316103	ES	20:21:22:25:26:27:28:<br>29:30:31:32:33:34:35:	19	41	-0.02	2.9E-01	excluded
PML	31646	ES	5:6.1:6.2:6.4:6.6:6.7	4	6.8	0.01	2.9E-01	included
PQLC1	46262	ES	7	5	9	0.00	2.9E-01	excluded
TBC1D3	40569	ES	9	8	10	-0.01	2.9E-01	excluded
SFTA3	27272	ES	04:02.2	2	6	0.02	2.9E-01	included
MRPL55	10119	ES	2.4:2.5:2.6	1.2	2.9	0.00	2.9E-01	excluded
ODF2	87758	ES	10	9.1	11	0.02	2.9E-01	included
MAD2L1	70469	ES	2:03	1	4	0.00	2.9E-01	excluded
TCF7L2	13155	ES	6.2	5	8	0.01	2.9E-01	included
SNX14	76930	ES	5:06	4	7	0.01	2.9E-01	included
KTN1	27634	ES	42	41	43	0.01	2.9E-01	included
UBE2D3	70139	ES	2.4:3.2	2.3	3.3	0.01	2.9E-01	included
BFAR	34095	ES	4:05	2	6	-0.02	2.9E-01	excluded
CECR5	60969	ES	3:04:05	2	6	0.00	2.9E-01	excluded
MRPL55	10107	ES	2.2:2.4:2.5:2.6:2.7:2.8	1.2	2.9	0.00	2.9E-01	included
LPHN2	3570	ES	24	23	25	0.00	2.9E-01	excluded
GRID1	12412	ES	15	14	16	0.00	2.9E-01	excluded
ALS2	56918	ES	24	23	25	-0.01	2.9E-01	excluded
THYN1	19546	ES	6	5	7	0.00	2.9E-01	included
HDAC10	62801	ES	9	8	10	0.00	2.9E-01	included
DRG2	39555	ES	4:5.1:6.1:6.2:6.3:6.4:7<br>:8.1:8.2:9	3.2	10	0.03	2.9E-01	included
CYTH1	43893	ES	10:11.1:12:13.2	9	14	0.00	2.9E-01	included
TRAPPC4	19073	ES	2.1:2.2:2.3	1	4.1	-0.01	2.9E-01	excluded
ALDH1A3	32744	ES	4	3	5	0.00	2.9E-01	excluded
CD46	9662	ES	7:08	6	9	-0.02	2.9E-01	excluded
PTK2	85308	ES	33	32	34	0.00	2.9E-01	included
KLF2	48150	ES	2	1	3	0.00	2.9E-01	excluded
MRPL55	10112	ES	2.2:2.4:2.5:2.6:2.8	1.2	2.9	0.00	2.9E-01	included
MYH10	39196	ES	17	16	18	0.00	2.9E-01	included
ELAC2	39331	ES	1.2:2.1	1.1	2.2	0.00	2.9E-01	excluded
C1orf63	1145	ES	5.1:5.2:6.1	4.2	6.2	-0.03	2.9E-01	excluded
CPSF3L	97	ES	5.2:6.2	4	7.1	0.00	2.9E-01	included
CTNND1	15988	ES	5	2.1	6	-0.01	2.9E-01	excluded
TPD52L2	60171	ES	7	5	8	0.00	2.9E-01	included
ANKRD6	77006	ES	11	10.1	12	0.00	2.9E-01	excluded
RNF34	24869	ES	3:4:5:6	1	7.1	0.00	2.9E-01	included
ANAPC10	70750	ES	2	1.2	3	0.00	2.9E-01	included
HPS5	14591	ES	23	22	24	-0.01	3.0E-01	excluded
PTPRU	1430	ES	21	19	22	0.00	3.0E-01	excluded
MTFR1	83987	ES	3	2	5	0.00	3.0E-01	excluded
ZMYND11	10588	ES	10:11.1:11.2	9	12	0.00	3.0E-01	excluded
CLCC1	3981	ES	02:03.1	1	3.2	0.02	3.0E-01	included
DEPDC4	23918	ES	2	1	3	0.02	3.0E-01	included
UBE3C	82507	ES	2:03	1	4	0.00	3.0E-01	excluded
ZSWIM7	39406	ES	2:03	1.1	4	0.02	3.0E-01	included
ARL16	44153	ES	2.3:3:4	2.2	5	-0.01	3.0E-01	excluded
AGK	82045	ES	3	2	4	0.00	3.0E-01	excluded
ARL1	23953	ES	3	1	5	0.00	3.0E-01	excluded

BZW2	78867	ES	2	1.1	3	0.00	3.0E-01	included
CTC1	39145	ES	20	19	21	0.01	3.0E-01	included
STX3	16040	ES	5	4	6	0.00	3.0E-01	included
DDX23	21484	ES	7:08	6	9.1	0.00	3.0E-01	included
ELOVL1	2167	ES	4	3	5	0.00	3.0E-01	included
SIL1	73576	ES	2:03	1	4	0.00	3.0E-01	included
RABGAP1	87502	ES	4	3	5	-0.01	3.0E-01	excluded
PML	31655	ES	5:6.1:6.4:6.7	4	6.8	0.00	3.0E-01	included
MPV17	52970	ES	7:08	6.3	9	0.00	3.0E-01	excluded
ZNF415	51679	ES	7.1:7.2	6.1	9	0.02	3.0E-01	included
RNF40	36190	ES	3.2:4:5:6:7:8:9:10.1	3.1	10.2	0.00	3.0E-01	excluded
PDIA3	30313	ES	2	1	4	0.00	3.0E-01	excluded
OGDH	79551	ES	3:05	2	7	0.00	3.0E-01	included
RNF38	86349	ES	6	5	7	0.00	3.0E-01	included
NOP58	56946	ES	4	3	5	-0.01	3.0E-01	excluded
FAM171A2	41857	ES	4.1	3	5	0.01	3.0E-01	included
CHCHD10	61318	ES	2.1:2.2	1	3.2	0.00	3.0E-01	included
FOPNL	34204	ES	3.2	2	4	0.01	3.0E-01	included
PFKM	21424	ES	11	10	12	0.00	3.0E-01	excluded
TARDBP	633	ES	3:04	2	5	0.01	3.0E-01	included
SLC7A8	26714	ES	7	6	8	0.00	3.0E-01	excluded
AMT	64868	ES	2	1	4	0.00	3.0E-01	included
DEF8	38190	ES	3	2.1	4	0.00	3.0E-01	included
CROT	80325	ES	4	3	5.1	0.00	3.0E-01	included
ABCB8	82316	ES	2.2	1	5	0.00	3.0E-01	excluded
NDUFC1	70621	ES	3	2.2	5	-0.01	3.0E-01	excluded
GTF3C5	88006	ES	9.1	8	10	0.01	3.0E-01	included
RPE	57246	ES	5	4	6	0.01	3.0E-01	included
CDADC1	25880	ES	4.1:4.2	3	6	0.00	3.0E-01	excluded
NSFL1C	58506	ES	5.2	4	7.2	-0.01	3.0E-01	excluded
PQLC1	46260	ES	9	5	10	-0.01	3.0E-01	excluded
RETSAT	54198	ES	9	8	10	0.00	3.0E-01	included
INO80E	36015	ES	6.1:6.2:6.3	5	11	0.02	3.0E-01	included
RPS25	19054	ES	2.2:3.1	2.1	3.2	-0.01	3.0E-01	excluded
RAP2A	26150	ES	2	1	3	-0.01	3.0E-01	excluded
ATF1	21713	ES	6:07	4	8	0.00	3.0E-01	included
PPIF	12307	ES	5	4.1	6.2	0.00	3.0E-01	excluded
FAM13C	11831	ES	11:12	10	13.1	-0.02	3.0E-01	excluded
ABI2	57019	ES	13.1:13.2	12	14	0.00	3.0E-01	excluded
GABARAPL1	20394	ES	2.7:2.8:2.10:2.11:2.12:<br>2.13:2.14	2.6	3	0.00	3.0E-01	excluded
NQO1	37303	ES	4	3	6.1	0.02	3.0E-01	included
SNX17	53000	ES	2.2:3	1	5	0.00	3.0E-01	excluded
NFATC3	37185	ES	12	9.1	13	0.01	3.0E-01	included
FAM86A	33880	ES	7	6	8	0.00	3.0E-01	included
ADAM9	83486	ES	18	17	19	0.00	3.0E-01	excluded
BPGM	81854	ES	2	1	4	0.00	3.0E-01	included
SMN1	72424	ES	8	7	9.3	0.02	3.0E-01	included
SMN1	72426	ES	4	3	5	0.00	3.0E-01	excluded
BLNK	12677	ES	7	6	8	0.00	3.0E-01	excluded
RPS6KA4	16650	ES	10.2:11.2:12:13:14:15.	10.1	15.2	-0.01	3.0E-01	excluded
ASNS	80572	ES	03:04.1	1	4.2	0.00	3.0E-01	excluded
ANP32E	7434	ES	5.1:5.2	4	6	0.00	3.0E-01	included
SLC25A43	89952	ES	3	2	4	-0.01	3.0E-01	excluded
BCORL1	90063	ES	10	9	11	0.01	3.0E-01	included
CAPN10	58288	ES	2:3.1:3.2:4:5:6:7:9:10.	1	11	-0.03	3.0E-01	excluded
TOM1	61969	ES	3	2	5	0.00	3.0E-01	included
TNFSF13	38974	ES	4.1:4.2	3	5	0.01	3.0E-01	included
CHD1L	7377	ES	7	6	8	0.00	3.0E-01	included
NDUFS7	46469	ES	3	2.3	4	0.00	3.0E-01	excluded
ETFDH	70998	ES	2	1	3	0.00	3.0E-01	excluded
ASCC2	61686	ES	3	1	5	0.01	3.0E-01	included

CHMP1A	38102	ES	2	1	3	0.00	3.0E-01	included
ECSIT	47722	ES	3	1	4	0.01	3.0E-01	included
SPOP	42315	ES	3	1	6	-0.01	3.0E-01	excluded
NSDHL	90394	ES	2	1	3	-0.01	3.0E-01	excluded
MVB12A	48304	ES	5	4	6	0.00	3.0E-01	included
GIPC1	47979	ES	6	5	7	0.00	3.0E-01	included
NAV2	14701	ES	24	22	25	0.02	3.0E-01	included
DMKN	49172	ES	8:10	7	11	0.01	3.0E-01	included
OSBPL1A	44885	ES	23:24:25	22	26	0.00	3.0E-01	excluded
RNASE1	26469	ES	2.3:3.1	1	3.2	0.02	3.0E-01	included
STAG2	90037	ES	4	1	5	0.01	3.0E-01	included
GIGYF2	234074	ES	26	25	27	0.00	3.0E-01	included
CAPNS1	49362	ES	4	3	5	0.00	3.0E-01	excluded
PDXDC1	34120	ES	7.2:8:9:10:11:12:13:1<br>4:15:16:17:18.1:19:20	7.1	22	0.00	3.0E-01	included
CSNK1A1	74046	ES	9	8.2	10	0.00	3.0E-01	included
EMP1	20543	ES	2	1	3	0.00	3.0E-01	included
TMEM143	50751	ES	3.1:3.2	2	4	0.02	3.0E-01	included
CMC2	37704	ES	11	10	12	0.00	3.0E-01	excluded
ALDOA	36043	ES	7.2:8	6.2	9	0.00	3.0E-01	excluded
NDUFA10	58239	ES	12	10	13	0.00	3.0E-01	included
LRIG3	22772	ES	15	14	16	0.00	3.0E-01	excluded
TMEM165	69326	ES	3:04:05	1	6	0.00	3.0E-01	excluded
TMEM116	24564	ES	6	4	7	-0.02	3.0E-01	excluded
PPP2R4	87846	ES	6	4	7	-0.01	3.0E-01	excluded
ZNF384	19924	ES	05:06.1	4	6.2	0.00	3.0E-01	included
SFTA3	121945	ES	2:3:4.2:5	1.3	6	0.02	3.0E-01	included
NPIPB3	93925	ES	12.7:13.1	12.6	13.3	-0.02	3.0E-01	excluded
ZNF268	25346	ES	10.1:10.2	9	11	0.00	3.0E-01	included
SLC30A6	53152	ES	11.2	10	12	0.01	3.0E-01	included
AASDH	69347	ES	7:08	6	9	0.00	3.0E-01	excluded
RPL12	87633	ES	3	2	4	0.00	3.0E-01	excluded
LETMD1	21744	ES	4:05:06	3.2	7	-0.01	3.0E-01	excluded
SLC12A9	81006	ES	3:04	2	5	0.01	3.0E-01	included
ZNF561	47370	ES	04:06.1	3	6.2	-0.01	3.0E-01	excluded
GALNT11	82407	ES	7	6	8.1	0.00	3.0E-01	excluded
UCHL5	9248	ES	9	8	10	-0.01	3.0E-01	excluded
UBE2I	33062	ES	4.2	2	5.2	0.01	3.0E-01	included
CAPN10	58275	ES	9:10.1:10.2	7	11	0.00	3.0E-01	included
UBE2F	58183	ES	5:07	2	9	0.00	3.0E-01	excluded
PIP4K2C	22655	ES	2.2:3.1	2.1	3.2	0.00	3.0E-01	excluded
CD44	14986	ES	7:8:9.1:9.2:10:11:12.1<br>:13:14:15	5	16.1	0.01	3.0E-01	included
ZNF562	47390	ES	3	2.2	4	0.01	3.0E-01	included
DYNC1LI2	36835	ES	5.2:6:7.1:8:9:10:11:12	5.1	13.2	0.00	3.0E-01	excluded
MPZL1	8872	ES	3	2	4	0.00	3.0E-01	included
ARHGAP17	35667	ES	4	3	5	0.00	3.0E-01	excluded
UBE2K	69076	ES	4:05	3	6	0.00	3.0E-01	excluded
VPS9D1	38134	ES	2.2:3:4	1	5	0.00	3.0E-01	included
CIRBP	46444	ES	6	5.2	7.5	0.00	3.0E-01	excluded
SLC35C2	59670	ES	3.4:3.5:4:5:6	3.3	7.1	0.00	3.0E-01	included
MROH6	85427	ES	10	9.2	11	0.01	3.0E-01	included
PRMT7	37225	ES	5	4.2	6	0.01	3.0E-01	included
SNUPN	31882	ES	3	1.1	4	0.01	3.0E-01	included
DYRK1A	60577	ES	13.1:13.2:13.3	12	14	0.00	3.0E-01	excluded
KIFC3	100254	ES	6	5	7	0.02	3.0E-01	included
ACSS1	58861	ES	12.2:13:14:15.1	12.1	15.2	0.00	3.0E-01	excluded
UHRF2	85821	ES	11	10	12	-0.01	3.0E-01	excluded
GTF2H2	72437	ES	9:10:11:12:13:14:15:1	8	17.2	0.00	3.0E-01	included
KMT2E	81273	ES	6	5	7	0.00	3.1E-01	included
RPE	57256	ES	2.1:2.2	1.2	3	0.00	3.1E-01	excluded
SMEK2	53625	ES	10	9	11	-0.01	3.1E-01	excluded

LIN54	69754	ES	6	5.2	7	0.00	3.1E-01	excluded
ARMCX6	89658	ES	3	2	4	0.00	3.1E-01	excluded
NR3C1	73887	ES	6.1:6.2:6.3	3	7.1	0.00	3.1E-01	excluded
DDB2	15674	ES	4:5:6:7	3	8	0.00	3.1E-01	included
PMF1	8200	ES	6	5	7	-0.02	3.1E-01	excluded
ATP11A	26313	ES	29	28	30.3	-0.02	3.1E-01	excluded
FLNB	65416	ES	40	39	42	0.00	3.1E-01	excluded
ABI1	11048	ES	5	4	7	-0.01	3.1E-01	excluded
UBA3	65566	ES	4	3	5	0.00	3.1E-01	included
FNBP1L	3790	ES	10:11	9	12	0.00	3.1E-01	included
RIC3	14231	ES	2:3.1:4:5:7.2	1	8	0.01	3.1E-01	included
CPT1B	62875	ES	8.1:8.2	7	9	0.00	3.1E-01	excluded
BRF1	29624	ES	2	1.2	3	0.00	3.1E-01	excluded
TOM1L2	39521	ES	4:05:06	3	7	0.00	3.1E-01	excluded
UCK1	87959	ES	5	4	7	0.00	3.1E-01	excluded
CARKD	26258	ES	3:4:5:6	1	7.1	0.00	3.1E-01	excluded
CRELD2	390168	ES	9	7	10	0.00	3.1E-01	included
SLC12A9	81004	ES	7	6	8	0.00	3.1E-01	excluded
MST4	90121	ES	8	7	9	0.00	3.1E-01	excluded
BTN2A2	75644	ES	4	3.2	5	0.00	3.1E-01	included
KPNB1	42090	ES	9:10	8	11	0.00	3.1E-01	included
MRS2	75509	ES	4	3	6.1	0.00	3.1E-01	included
TOE1	2678	ES	2	1	3	0.00	3.1E-01	included
ABCC4	26111	ES	18	17	19	0.00	3.1E-01	included
INSIG2	55092	ES	2	1	3	0.00	3.1E-01	excluded
RAB5B	22330	ES	4	3	5	0.00	3.1E-01	excluded
ASXL1	58955	ES	6	5	8	-0.02	3.1E-01	excluded
TCF7L2	13153	ES	7	5	8	0.00	3.1E-01	included
MRS2	75513	ES	2	1	3	0.00	3.1E-01	included
PPHLN1	21227	ES	6	5	7	0.01	3.1E-01	included
SHBG	39019	ES	5.1:5.2:6.1:6.2:7:8	4.2	9	0.01	3.1E-01	included
TOM1L2	39515	ES	5:06	4	7	0.00	3.1E-01	included
DDX58	86062	ES	6	5	7	0.00	3.1E-01	included
USP35	18020	ES	2:3:4.2:5:6	1	7.1	-0.01	3.1E-01	excluded
ZNF569	49517	ES	05:06.1	4	7	0.01	3.1E-01	included
SMPD4	55316	ES	4.1:4.2:5:6:7:8:9	3	10	0.00	3.1E-01	included
GAS8	38203	ES	6	5	7.1	0.01	3.1E-01	included
RANGAP1	62418	ES	10	9	11	0.00	3.1E-01	included
TMEM199	39892	ES	5	4	6.1	0.00	3.1E-01	excluded
ITPKC	49957	ES	4	3	5	0.00	3.1E-01	included
TMEM159	34432	ES	2.1	1	2.3	0.00	3.1E-01	excluded
NDUFAF6	84595	ES	13	11	14	0.00	3.1E-01	included
PUM2	52777	ES	5	4.2	6	0.01	3.1E-01	included
CLN6	31365	ES	3:4:5.1:5.2:6	2	7.1	0.00	3.1E-01	excluded
HNRNPA1	212646	ES	3:4:5:6.1:6.2:6.3:7.2:9.1:9.2:10:11.2	2	11.3	0.00	3.1E-01	excluded
NUPL2	78962	ES	7	6	8	0.00	3.1E-01	excluded
RGS12	68644	ES	7.2	5	8	0.01	3.1E-01	included
DOCK8	85702	ES	30	29	31	0.01	3.1E-01	included
LSM5	79192	ES	3	2.1	4.1	0.00	3.1E-01	included
SLC39A9	28147	ES	7.1	6	7.3	-0.03	3.1E-01	excluded
GTF2H1	14605	ES	2	1	3	0.00	3.1E-01	included
EBPL	25902	ES	5	4.2	6	0.00	3.1E-01	included
VAC14	37413	ES	2	1	3	0.00	3.1E-01	excluded
PACRGL	68894	ES	6:8:9:12	5	13.1	-0.02	3.1E-01	excluded
CREBBP	33634	ES	5	4	6	-0.01	3.1E-01	excluded
TEX10	87082	ES	3.1:3.2	1	4	0.00	3.1E-01	excluded
C16orf13	32924	ES	2	1	3	0.00	3.1E-01	excluded
ALCAM	65992	ES	8:09	7	10	0.00	3.1E-01	excluded
RAB7A	66658	ES	4.1:4.2:5.1:5.2	3.2	6	0.00	3.1E-01	included
ABCC6	34223	ES	20	19	21	0.00	3.1E-01	excluded
TSEN15	9207	ES	04:05.1	3	6	0.00	3.1E-01	excluded

DYNC1LI2	36836	ES	4	3	5.1	0.00	3.1E-01	excluded
ATXN2	24515	ES	24:25:00	23	26	-0.01	3.1E-01	excluded
WNK1	19616	ES	14	13.2	15	-0.03	3.1E-01	excluded
TPM1	31007	ES	2.2	1	3.1	0.00	3.1E-01	excluded
SZRD1	804	ES	2	1	3	0.00	3.1E-01	excluded
UBAP2	86142	ES	2	1.2	4	0.01	3.1E-01	included
TUBB6	44683	ES	4.2	3	5.3	0.00	3.1E-01	excluded
ZNF791	47813	ES	2.1:2.2	1	3	-0.01	3.1E-01	excluded
BAG4	83375	ES	2	1	3	-0.01	3.1E-01	excluded
TBCB	49355	ES	04:05.1	2.3	6	-0.02	3.1E-01	excluded
MBNL1	67321	ES	9:10	7	11	0.00	3.1E-01	excluded
KCNRG	25923	ES	2	1	3	0.00	3.1E-01	included
MAZ	35950	ES	3.1:3.2:4.2	2.2	5.1	0.00	3.1E-01	included
TCEB1	84213	ES	5	1.1	6	-0.02	3.1E-01	excluded
CDK7	72323	ES	5.1:5.2:6	4	7	0.00	3.1E-01	included
NAB1	56582	ES	8	6	9.1	0.00	3.1E-01	included
GIPC1	47987	ES	2:03	1	5	0.00	3.1E-01	excluded
STYXL1	80151	ES	4:05	3	6	0.00	3.1E-01	included
MDM2	23289	ES	3:5.1:5.2:6	2.1	7	0.00	3.1E-01	included
RELA	16910	ES	3	2	4.1	0.00	3.1E-01	excluded
HBS1L	77806	ES	3:04	2	7.1	0.00	3.1E-01	included
SNX3	77148	ES	2	1.2	4	0.00	3.1E-01	included
ALDOA	36044	ES	8	6.2	9	0.00	3.1E-01	included
ANK3	11848	ES	46	45	47	0.00	3.1E-01	excluded
CANT1	43977	ES	3.1	1	3.3	-0.01	3.1E-01	excluded
HNRNPC	26560	ES	2.2	1	2.4	0.00	3.1E-01	included
PUM2	52776	ES	6	4.2	7	0.00	3.1E-01	excluded
ACSS2	59047	ES	4:5.1:6:7:8	3	10	0.00	3.1E-01	excluded
MGME1	58752	ES	3:04	2.2	5	0.00	3.1E-01	excluded
ILDR1	66416	ES	4:05	3	6	0.00	3.1E-01	excluded
GOLM1	86749	ES	5	4.2	6	0.00	3.1E-01	included
CLK1	56754	ES	11	10	12	0.00	3.1E-01	included
HNRNPM	47235	ES	11	10	12	0.00	3.1E-01	included
NUP35	56473	ES	8	7	9	-0.01	3.1E-01	excluded
SHMT2	22556	ES	4	1	6.1	0.00	3.1E-01	included
TRNT1	62982	ES	1.2:1.4	1.1	2	0.00	3.1E-01	excluded
THNSL2	54471	ES	9	8	11	-0.02	3.1E-01	excluded
CDC20B	72026	ES	11	10	12	0.01	3.1E-01	included
DAD1	26609	ES	2	1.3	3	0.00	3.1E-01	included
CAMK2G	12242	ES	19.2	18	21	0.00	3.1E-01	excluded
C5orf45	74982	ES	2.3:3.1:3.2	2.1	4	0.00	3.1E-01	excluded
YAF2	21135	ES	5.1:5.2:6:7:8	2	9.1	0.00	3.2E-01	included
FAM63B	30910	ES	6	5.1	7	0.00	3.2E-01	excluded
OS9	22704	ES	5.1:5.2:5.3:7.1:7.2:7.3:7.4:8:9.1	4	9.2	0.00	3.2E-01	included
ATG16L1	58038	ES	8	7	10	-0.02	3.2E-01	excluded
IDS	90293	ES	13	11	14	0.00	3.2E-01	excluded
ATG4A	89852	ES	3:04	2	5	0.01	3.2E-01	included
EML2	50497	ES	22	21	23	0.00	3.2E-01	included
RUVBL1	66641	ES	11	10	12	0.00	3.2E-01	excluded
DGKZ	15546	ES	11	10.2	12	0.00	3.2E-01	excluded
TM7SF3	92997	ES	10	9	11	0.00	3.2E-01	included
LMBR1	82486	ES	4.1:5:7.2	2.1	9	0.00	3.2E-01	excluded
TRAIP	64925	ES	4:5:6:7:8	3	9	-0.01	3.2E-01	excluded
CADPS2	81609	ES	22	21	23	0.00	3.2E-01	included
ATG16L2	17663	ES	4.1:4.2:4.3:5:6.1:6.3:7:8:9:10:11:12:13	3	14	0.00	3.2E-01	included
RPGR	88810	ES	10	9	11	0.00	3.2E-01	included
ZC3H18	38002	ES	4	3	5	-0.01	3.2E-01	excluded
CUL2	11222	ES	22	21	23	0.00	3.2E-01	excluded
LPCAT3	20065	ES	3:04	1	5.1	0.00	3.2E-01	included
JAM2	60255	ES	3	2	4	0.00	3.2E-01	excluded

ETV1	78835	ES	13	12	14	0.00	3.2E-01	excluded
TBC1D10A	61721	ES	4.2	1	5	0.00	3.2E-01	included
ALDH3B1	17274	ES	4:05	3	6	0.00	3.2E-01	included
P4HA1	12123	ES	2	1	3	-0.02	3.2E-01	excluded
RNF41	22406	ES	2	1.2	3	0.00	3.2E-01	excluded
MTHFD2	54022	ES	7	6	8	0.00	3.2E-01	included
RCBTB2	25864	ES	4	3	5	0.01	3.2E-01	included
ARL13B	65689	ES	3.2:4	2	5.1	0.02	3.2E-01	included
EPHA4	57752	ES	3	2.2	4	0.00	3.2E-01	included
ZNF780B	49857	ES	3	2	4	0.01	3.2E-01	included
SLCO2B1	17829	ES	3:04:05	1	6	0.00	3.2E-01	included
COPS7A	19946	ES	4:06	2.4	7	-0.02	3.2E-01	excluded
POM121	79929	ES	4	3	6	-0.02	3.2E-01	excluded
DCAF5	28126	ES	1.2:3	1.1	4	0.00	3.2E-01	excluded
MAP4K1	49674	ES	14.2:15:16:17:18:19:20:21:22:23:24.1:24.3:	14.1	29.2	0.00	3.2E-01	included
RBM42	95126	ES	5	4	6.2	0.01	3.2E-01	included
GTF2H2C	72396	ES	2	1.2	3	0.02	3.2E-01	included
PGAP3	40674	ES	3	2	4	0.00	3.2E-01	included
IFI27	29074	ES	5	4.1	6.1	0.00	3.2E-01	excluded
SPAG9	42495	ES	16	15	17	0.00	3.2E-01	included
LPHN2	3563	ES	29:30:00	27	31	0.00	3.2E-01	included
FLI1	19420	ES	6	5	7	0.00	3.2E-01	excluded
ABCA1	87109	ES	2	1	3	0.01	3.2E-01	included
CD276	31613	ES	5:06	4.2	7	-0.01	3.2E-01	excluded
CCNL2	160	ES	6.1:7.1	5	7.2	0.03	3.2E-01	included
AIFM1	90073	ES	11.2	10	11.4	0.00	3.2E-01	included
CHMP2B	65661	ES	2	1	3.1	0.00	3.2E-01	included
SFI1	61869	ES	11:12:13:14:15	10	16	0.00	3.2E-01	included
HERC3	69902	ES	11:12	10.2	13	0.00	3.2E-01	included
ZNF554	46626	ES	6	5	7	-0.02	3.2E-01	excluded
COX4I1	156375	ES	5.1:5.3	4.1	5.4	-0.01	3.2E-01	excluded
BRD1	62756	ES	5	3.2	6	0.00	3.2E-01	included
FLAD1	7868	ES	3	2.2	4.1	0.00	3.2E-01	excluded
BBS5	55867	ES	3:04	2	5	0.00	3.2E-01	excluded
CDC27	42055	ES	3:04	2	5	0.00	3.2E-01	excluded
HNRNPM	47237	ES	3	2	4	0.00	3.2E-01	excluded
ELP2	45218	ES	12	11	13	0.00	3.2E-01	excluded
GSAP	80194	ES	26	25	27	0.00	3.2E-01	excluded
RAB7A	66661	ES	3.2:4.1:4.2:5.1	3.1	5.2	0.00	3.2E-01	included
KCTD20	75977	ES	5	4	6	0.00	3.2E-01	included
PTPN1	59777	ES	2	1	3	0.00	3.2E-01	included
SCP2	3046	ES	4	3	5	0.01	3.2E-01	included
EIF3E	84875	ES	03:04.1	1	4.2	-0.02	3.2E-01	excluded
MRPL27	42377	ES	5.3:5.5	5.1	5.6	0.00	3.2E-01	included
ELK3	23826	ES	3	2	4	0.00	3.2E-01	excluded
APOPT1	29464	ES	2	1	5	0.02	3.2E-01	included
SMAP1	76646	ES	12	11	13	0.00	3.2E-01	included
TTLL11	87449	ES	3	2	4.1	-0.02	3.2E-01	excluded
CALM2	53491	ES	3	2	4	-0.01	3.2E-01	excluded
MRPL55	10159	ES	1.2:2.2:2.4:2.5	1.1	2.9	0.00	3.2E-01	included
AMD1	77248	ES	3:04:05	1	6	0.00	3.2E-01	excluded
CHP1	30074	ES	6	4	7	0.00	3.2E-01	included
GABARAPL1	20409	ES	2.7:2.8:2.10:2.12	2.6	3	0.00	3.2E-01	included
SLC38A6	27789	ES	18:20.1	17	20.2	-0.02	3.2E-01	excluded
PAPD5	36368	ES	7	6	8	0.00	3.2E-01	included
KANK2	47637	ES	2.1:2.2:2.3	1	3	0.02	3.2E-01	included
ZNF148	66550	ES	3.2:4	3.1	5	-0.01	3.2E-01	excluded
MLPH	58113	ES	14	13	15	-0.01	3.2E-01	excluded
ARPC1A	80603	ES	5	4	6	0.01	3.2E-01	included
NDUFAF1	30087	ES	4	3	5	-0.01	3.2E-01	excluded
SEC22C	64297	ES	5:6.1:6.2	3	7	0.01	3.2E-01	included

EDC3	31734	ES	2:03	1	5	0.00	3.2E-01	included
SPP1	69875	ES	4	3	5	0.00	3.2E-01	included
PER2	58221	ES	11	10	12	0.00	3.2E-01	included
YKT6	79481	ES	6	5	7	0.00	3.2E-01	excluded
LARS	73908	ES	2	1	3	0.00	3.2E-01	excluded
XRRA1	17791	ES	13	10	14	0.03	3.2E-01	included
IDE	12533	ES	22	21	23	-0.01	3.2E-01	excluded
FAM21A	11565	ES	18	17	19	-0.01	3.2E-01	excluded
HNRNPA1	22148	ES	6.2:6.3:7.2:8:9.1	6.1	9.2	0.01	3.2E-01	included
NUFIP2	40042	ES	2	1	3	0.00	3.2E-01	included
SUGP2	48547	ES	13	12.3	14	0.02	3.2E-01	included
FAXDC2	74237	ES	6	5.2	7	0.00	3.2E-01	excluded
RBM14	17081	ES	4.1:4.2	1	5	0.00	3.2E-01	included
SLC1A4	53776	ES	6	5	7	0.00	3.2E-01	included
USP8	30587	ES	6	5	7	0.00	3.2E-01	included
NUSAP1	30079	ES	10	9	11	-0.01	3.2E-01	excluded
FOS	28498	ES	2.4	2.2	2.6	0.00	3.2E-01	excluded
POMT1	87945	ES	5.2	4	6	0.00	3.2E-01	excluded
VPS29	24439	ES	2	1	3.1	0.00	3.2E-01	excluded
C5orf45	74981	ES	2.2:2.3:3.1:3.2	2.1	4	0.00	3.2E-01	excluded
PPIP5K1	30275	ES	2	1	3.2	0.02	3.2E-01	included
FAM49B	85139	ES	11	10	12	0.00	3.2E-01	included
XRCC1	50206	ES	2	1	3	0.00	3.2E-01	excluded
C8orf59	84336	ES	2.1:2.2:2.3	1	3.2	0.02	3.2E-01	included
SLAIN2	69215	ES	7	6	8	0.00	3.2E-01	excluded
SCG5	29821	ES	5	4.2	6	0.00	3.2E-01	excluded
C14orf159	28865	ES	05:02.2	1	8	-0.01	3.2E-01	excluded
FOXK2	44408	ES	9	8	10	0.01	3.2E-01	included
MRE11A	18334	ES	17.1:17.2	16	18	0.00	3.2E-01	included
NUP107	22964	ES	6	5.2	7	0.00	3.2E-01	included
DUS2	37177	ES	2	1	3.2	-0.01	3.2E-01	excluded
MTMR10	29792	ES	8	7	9	-0.01	3.2E-01	excluded
CD79B	43025	ES	3	2.2	4	-0.01	3.2E-01	excluded
ZCCHC4	68962	ES	6:07:08	5	9	0.00	3.2E-01	included
PIGU	59031	ES	12	11	13	0.00	3.2E-01	excluded
RPS6KA4	16649	ES	10.2:11.1:11.2:12:13:1	10.1	15.2	0.00	3.2E-01	excluded
GK	88741	ES	3	2	5	0.00	3.2E-01	excluded
DCTD	71245	ES	1.2:2.1:2.2:3:4	1.1	5	0.00	3.2E-01	included
TNC	87358	ES	12:13:15:16:19	11	20	0.03	3.2E-01	included
ATP5G2	99402	ES	04:02.2	2	6	-0.03	3.2E-01	excluded
NCOR2	25145	ES	46.1	45	47	-0.01	3.2E-01	excluded
GNB2L1	75072	ES	2.1:2.2:2.3:3:4.1:4.2:5 :6:7.2:8.1	1.1	8.2	-0.01	3.2E-01	excluded
ZSCAN18	52404	ES	3.3:4	3.2	5	0.00	3.3E-01	included
ACADVL	38852	ES	4	3.3	5	0.00	3.3E-01	included
FAN1	29779	ES	3	2	4	0.00	3.3E-01	excluded
DDX55	25089	ES	10	9	11	0.00	3.3E-01	included
DNAJB6	82514	ES	3	2	4	0.00	3.3E-01	included
IST1	37520	ES	7:8:9:10	6	11	0.00	3.3E-01	excluded
APTX	86088	ES	6.2:6.3:7.2:7.3	5.2	7.4	0.02	3.3E-01	included
PMS1	56548	ES	7	6	8	-0.01	3.3E-01	excluded
TYW3	3477	ES	2	1	3	0.00	3.3E-01	excluded
SCOC	70648	ES	7	6	8	0.00	3.3E-01	excluded
RILPL1	25080	ES	7	6	9	0.00	3.3E-01	excluded
CASP8	56828	ES	6	5.2	7	0.00	3.3E-01	included
LTBR	19855	ES	6	5	7	0.00	3.3E-01	excluded
CTNND1	15966	ES	5:06:07	2.1	8	0.00	3.3E-01	excluded
ZFYVE19	30050	ES	8	7	9	0.01	3.3E-01	included
TOR2A	87660	ES	2	1	3.1	0.01	3.3E-01	included
ARCN1	19031	ES	2	1	3.1	0.00	3.3E-01	excluded
EXOC3L1	36962	ES	3:04	2	5	0.00	3.3E-01	excluded
RSRC1	67423	ES	6	5	7	0.00	3.3E-01	included

NBEAL2	64486	ES	46	45	47	0.00	3.3E-01	included
NUMB	28291	ES	11:12	10	14	0.00	3.3E-01	included
ASB13	10662	ES	5	4	6	-0.01	3.3E-01	excluded
TM9SF4	58939	ES	4	3	5.1	0.00	3.3E-01	included
APOL1	62041	ES	3.2:5	1	7	0.00	3.3E-01	included
RABGEF1	79899	ES	5:06	3	7	0.02	3.3E-01	included
SCARB2	69596	ES	4:05:06	2	7	0.00	3.3E-01	excluded
SRP54	27180	ES	4.2:5:6:7.1	4.1	7.2	0.00	3.3E-01	included
MSR1	82782	ES	4:05	3	6	0.00	3.3E-01	excluded
ICAM2	43049	ES	1.2:2	1.1	3.2	0.00	3.3E-01	included
MYLK	66482	ES	32	31	33	0.00	3.3E-01	excluded
PALLD	71129	ES	17	16	18	0.00	3.3E-01	excluded
ZNF512	53028	ES	2	1	3	-0.01	3.3E-01	excluded
FBXO4	71890	ES	2:03	1	4	-0.01	3.3E-01	excluded
ARMC10	81160	ES	8	6	9	0.00	3.3E-01	excluded
TATDN1	85089	ES	4.1	2	5	-0.01	3.3E-01	excluded
OSBPL2	60052	ES	13	12	14	0.00	3.3E-01	included
SNRK	64342	ES	3:04	1	5	0.00	3.3E-01	excluded
PIGH	28072	ES	3.1:3.2	2.3	5	0.00	3.3E-01	included
NARS	45640	ES	5.2:6:7:8.1	5.1	8.2	0.00	3.3E-01	included
C3orf17	66189	ES	2	1	3.1	0.01	3.3E-01	included
DERL1	85031	ES	2:5:6:7	1	8	0.00	3.3E-01	excluded
INTS7	9724	ES	3	2	4	0.00	3.3E-01	included
MDH1B	57156	ES	2	1	3	-0.03	3.3E-01	excluded
P4HTM	391671	ES	6.1:6.2	5	7.1	0.02	3.3E-01	included
ATG13	15584	ES	3	2.2	4.1	-0.01	3.3E-01	excluded
CNN3	3824	ES	5	4	6	0.00	3.3E-01	included
COX6B1	49246	ES	4	3	5	0.00	3.3E-01	excluded
SCIN	78817	ES	14	13	15	0.00	3.3E-01	included
ATF2	56080	ES	7:8:9:10.1:11:12:13:1	6	16	0.00	3.3E-01	excluded
NSMAF	83945	ES	4	3	6	0.00	3.3E-01	excluded
CHURC1	27912	ES	3.2	2	4	0.00	3.3E-01	included
RPS6KB1	42836	ES	8	7	10	-0.02	3.3E-01	excluded
SHMT1	39618	ES	4	3	5	0.00	3.3E-01	included
CPVL	99754	ES	15	14	17	-0.02	3.3E-01	excluded
PRPF3	7466	ES	4:05	3	6	0.01	3.3E-01	included
SSR2	8166	ES	3	2	4.1	0.00	3.3E-01	excluded
CRYZ	3470	ES	3	1	4	0.00	3.3E-01	included
CTNND1	15986	ES	2.2:2.3:5	2.1	6	0.01	3.3E-01	included
DCUN1D4	69272	ES	9.3	9.1	10	0.01	3.3E-01	included
GPHN	28035	ES	4	3	5	0.00	3.3E-01	excluded
UQCRC2	35524	ES	5:06	4	7	0.01	3.3E-01	included
MAZ	35944	ES	3.2:4.2:5.1:7.1:7.2:7.3	3.1	7.4	0.00	3.3E-01	excluded
NFATC3	37187	ES	5	4.1	6	0.00	3.3E-01	included
CD44	15054	ES	15	5	16.1	0.00	3.3E-01	included
RBMS3	63802	ES	13	12	14	-0.02	3.3E-01	excluded
ARL6IP1	34269	ES	5	4	6	0.00	3.3E-01	included
RFTN1	63650	ES	5	4	6	0.00	3.3E-01	excluded
ARPC1B	80612	ES	2:03	1	4.2	0.00	3.3E-01	excluded
C1RL	20075	ES	3.2:4.1	3.1	4.2	0.00	3.3E-01	included
MIB2	196	ES	8	7	9	0.00	3.3E-01	excluded
COPS3	39472	ES	5	4	6.2	0.00	3.3E-01	excluded
YTHDF3	83977	ES	5.1:5.2	4.1	6	0.00	3.3E-01	excluded
SEPP1	71903	ES	5:06	4	7	0.00	3.3E-01	excluded
RAP1B	22941	ES	4:05	3.2	6	0.00	3.3E-01	included
VPS18	30059	ES	5	4	6	0.00	3.3E-01	included
TMEM108	66829	ES	5	4.2	7	0.00	3.3E-01	included
ZNF317	47267	ES	5.2	4	6	0.01	3.3E-01	included
TSSC1	52560	ES	3	2	4	0.00	3.3E-01	included
MTR	10403	ES	21	20	22	0.00	3.3E-01	included
AP1M1	48149	ES	02:03.1	1.1	3.2	0.00	3.3E-01	excluded
FARSB	57753	ES	3	2	4	0.00	3.3E-01	excluded

TINF2	26936	ES	2:3:4:5	1	6.1	0.00	3.3E-01	included
OPN3	10432	ES	2	1	4	0.01	3.3E-01	included
ULK3	31767	ES	2.2:2.3:2.4	1	3.2	0.01	3.3E-01	included
COPS8	58097	ES	7	6	8.1	0.00	3.3E-01	included
TRAPPC2L	38047	ES	4.1:4.2:4.3:4.4	3.2	5.1	0.01	3.3E-01	included
HTATIP2	14709	ES	3	2.1	4	0.00	3.3E-01	included
C1QTNF1	43988	ES	6.2	2.2	7	0.00	3.3E-01	excluded
RHPN2	48923	ES	4	3	5	0.00	3.3E-01	excluded
C11orf30	17894	ES	10	9	11	0.00	3.3E-01	excluded
FBLIM1	774	ES	6:07	5.2	8	0.00	3.3E-01	excluded
TMEM205	47667	ES	2.3:2.5	2.2	2.6	0.00	3.3E-01	included
NOD1	79116	ES	14	13	15	0.00	3.3E-01	excluded
HNRNPA1	301523	ES	8:9.1:9.2	6.2	10	-0.01	3.3E-01	excluded
ATE1	13325	ES	4	3	5	0.00	3.3E-01	excluded
PEMT	39492	ES	9.2	8	10	0.00	3.3E-01	excluded
EIF4G1	67883	ES	5:06	3.2	7	0.00	3.3E-01	excluded
VPS39	30131	ES	3	2	4	0.02	3.3E-01	included
CD46	9664	ES	7	6	8	0.01	3.3E-01	included
KLHL3	73483	ES	12:13:14:15	10	16	-0.01	3.3E-01	excluded
GPD2	55631	ES	6:8:9:10	4	11	0.00	3.3E-01	included
STARD3	40657	ES	7.1:7.2	6	8	0.02	3.3E-01	included
COL4A4	57794	ES	43	42	44	0.00	3.3E-01	excluded
FTSJ1	88970	ES	2	1	3	0.00	3.3E-01	included
SMPDL3B	1362	ES	4	3.2	5	0.00	3.3E-01	excluded
MFSD1	67452	ES	14	13	15	0.00	3.3E-01	excluded
TIMM23	11551	ES	5.2	3	6	0.00	3.3E-01	excluded
LETMD1	21792	ES	2:04	1.2	5	0.00	3.3E-01	included
ORC4	55524	ES	10	9	11	0.00	3.3E-01	included
VDAC2	12274	ES	4	3	5	0.00	3.3E-01	included
NAPRT1	85431	ES	11.5:11.7	11.4	11.8	0.00	3.3E-01	included
NIF3L1	56772	ES	6	5	7	0.00	3.3E-01	excluded
CMPK1	2907	ES	5	4	6	0.00	3.3E-01	included
TMEM120B	24894	ES	7:08	6	9	-0.01	3.3E-01	excluded
PTK2	85312	ES	24:25:26:27:28	23	29	0.00	3.3E-01	excluded
HSPH1	25577	ES	6:07	5	8	0.00	3.3E-01	included
MAPK12	62811	ES	6	5	7	-0.01	3.3E-01	excluded
NSD1	74742	ES	6:07	4.3	8	0.00	3.3E-01	included
PAK6	29960	ES	12	11	13.1	0.00	3.3E-01	included
TRIT1	1916	ES	10	9	11	0.00	3.3E-01	included
SERPING1	15875	ES	2.2	1	3.2	0.00	3.3E-01	excluded
MAP3K8	11124	ES	2	1	3.2	0.01	3.3E-01	included
D2HGDH	58420	ES	7.1:7.3:8:9	6	11.2	-0.03	3.3E-01	excluded
MLF1	67443	ES	2	1.1	5	0.00	3.4E-01	excluded
ZNF613	51384	ES	4	3	5	-0.01	3.4E-01	excluded
SFTA3	121947	ES	2:05	1.3	6	0.00	3.4E-01	excluded
RCOR3	9711	ES	5	4	6	-0.01	3.4E-01	excluded
STAU2	84179	ES	3:4:7:8	2	11	0.00	3.4E-01	excluded
BTBD7	29032	ES	5:06	3	7	0.00	3.4E-01	excluded
SPOP	42312	ES	2	1	3	-0.02	3.4E-01	excluded
MAP4K3	53332	ES	3	1	4.1	0.00	3.4E-01	excluded
DIS3L2	57992	ES	11	10.2	12	-0.01	3.4E-01	excluded
MANBAL	59343	ES	2.2:3	1	4.2	0.01	3.4E-01	included
OCIAD1	69243	ES	3:04:05	2.1	6	-0.03	3.4E-01	excluded
ZNF415	51678	ES	8.2	6.1	9	-0.02	3.4E-01	excluded
RBM27	73911	ES	9	8	10	-0.01	3.4E-01	excluded
ARL1	23950	ES	3:05:06	1	7.1	0.00	3.4E-01	excluded
HOPX	69382	ES	1.2:2	1.1	4.6	0.02	3.4E-01	included
LGR4	14756	ES	2	1	3	0.00	3.4E-01	included
TAPT1	68831	ES	5.2:6	5.1	7	0.00	3.4E-01	excluded
FAM221A	78998	ES	2	1	4	-0.02	3.4E-01	excluded
CEP57L1	77166	ES	11	10.1	12	0.01	3.4E-01	included
OSBPL2	60053	ES	5	4	6	0.00	3.4E-01	included

RGL1	9196	ES	14	13	15	0.00	3.4E-01	excluded
POLR2E	46387	ES	2.2	1	3	-0.02	3.4E-01	excluded
SYTL3	78283	ES	10:11	9	12	0.01	3.4E-01	included
NAGPA	33872	ES	9	8	10	0.00	3.4E-01	included
RIC3	14227	ES	2:3.1:4:5	1	7.2	0.00	3.4E-01	excluded
VEGFA	76331	ES	7.1:7.2:7.3:8.1	6	9.1	0.00	3.4E-01	excluded
AKIRIN1	1864	ES	3	2	4	0.00	3.4E-01	included
FHL2	54826	ES	7	5.1	8	0.00	3.4E-01	excluded
STK16	57667	ES	3.1:3.2:4	2.2	5	-0.01	3.4E-01	excluded
ZNF248	11305	ES	3	2	4	-0.02	3.4E-01	excluded
GPR116	76430	ES	20	19	21	0.00	3.4E-01	excluded
LRRC28	32678	ES	5:6:7.1:7.2:8:10:11	2	12	0.00	3.4E-01	included
CD3D	18988	ES	3	2	4	0.01	3.4E-01	included
API5	15452	ES	3	1	4	0.00	3.4E-01	excluded
NDRG3	59305	ES	3:04:05	2.2	6	0.00	3.4E-01	included
FAM107B	10824	ES	11	10	12	0.00	3.4E-01	included
SNRPD3	61422	ES	3.2:4.1	3.1	4.2	0.00	3.4E-01	included
STRIP1	4095	ES	7	6	8	0.00	3.4E-01	included
MSH6	53508	ES	1.2:3:4	1.1	5.1	0.00	3.4E-01	excluded
ZMYND10	65029	ES	5	4	6	0.00	3.4E-01	excluded
CALCOCO2	42225	ES	8	7	9	0.00	3.4E-01	excluded
OSBPL5	13954	ES	6.1:6.2	5.2	7	0.00	3.4E-01	included
C6orf203	77124	ES	2	1.2	3	-0.02	3.4E-01	excluded
XPA	87022	ES	6	5	7	0.01	3.4E-01	included
SPATA6L	85768	ES	3:04:05	2	6	-0.01	3.4E-01	excluded
ZKSCAN7	64370	ES	1.2:2	1.1	3	0.01	3.4E-01	included
C9orf85	86581	ES	2:03	1	4	0.00	3.4E-01	included
CSAD	21957	ES	8:09	7.2	10.1	0.00	3.4E-01	included
PLA2G6	62207	ES	11:12	10	13	0.02	3.4E-01	included
C6orf211	78159	ES	2	1	3	0.00	3.4E-01	excluded
SEPT2	58375	ES	4:05	2	7	0.00	3.4E-01	excluded
FAM3A	90641	ES	02:03.2	1.4	4	0.00	3.4E-01	included
GALM	53268	ES	2:3:4:5	1	6	-0.02	3.4E-01	excluded
CAPN10	58279	ES	9	7	11	-0.02	3.4E-01	excluded
APAF1	23873	ES	18	17	19	-0.02	3.4E-01	excluded
TRA2A	78976	ES	2:3.1:3.2	1	4	-0.01	3.4E-01	excluded
CSTF3	14889	ES	2	1	4.1	0.00	3.4E-01	included
RPAIN	38686	ES	4:05	3	6.1	0.00	3.4E-01	excluded
PTPN18	55343	ES	2:3:4:5:6	1	7	0.01	3.4E-01	included
FBXO4	71891	ES	2	1	4	0.01	3.4E-01	included
CCDC122	25774	ES	3:04	2	5	0.01	3.4E-01	included
CPSF7	16203	ES	4:6.1:6.2:6.3	3	7.1	-0.01	3.4E-01	excluded
PSEN2	10033	ES	11	10	12	0.00	3.4E-01	included
KIAA0040	9047	ES	3	2	4	-0.01	3.4E-01	excluded
RAB18	11072	ES	4:05	3	6	0.00	3.4E-01	excluded
ARFGAP2	15652	ES	4.2:5:6.1:6.2:7	3	8	0.01	3.4E-01	included
TRIP6	81015	ES	2.2	1	3	0.02	3.4E-01	included
ST6GALNAC6	87683	ES	6	5	7	0.00	3.4E-01	excluded
COX16	28179	ES	2	1	4	0.00	3.4E-01	excluded
FAM131A	67935	ES	6.2	2	6.4	0.01	3.4E-01	included
METTL23	43634	ES	1.4:1.5:3	1.3	4.1	-0.01	3.4E-01	excluded
PCDH1	73833	ES	6	5	7	0.01	3.4E-01	included
GLT8D2	24091	ES	4	2	5	0.01	3.4E-01	included
ZNF706	84749	ES	3.4	2	4	0.00	3.4E-01	excluded
CUX1	81078	ES	4	3	5	0.00	3.4E-01	included
PBXIP1	7848	ES	2.2:3.1:3.2:4:5.1:5.2:6.	2.1	6.4	0.00	3.4E-01	included
HMHA1	46380	ES	4.2	2	7	0.00	3.4E-01	excluded
DDB1	16157	ES	5:6:7:8:9:10:11:12:13:<br>14:15:16:17:18:19:20	4	21	0.00	3.4E-01	excluded
REPIN1	82245	ES	2.2:3.2:4.2	2.1	5.2	0.01	3.4E-01	included
ARFIP1	70860	ES	3:05:06	2	7	0.00	3.4E-01	excluded
DEF8	38194	ES	2.1	1	4	0.01	3.4E-01	included

PDCD10	67560	ES	4.2	1.1	5	0.01	3.4E-01	included
TRA2A	78975	ES	2	1	3.1	0.02	3.4E-01	included
PXK	65437	ES	19	17.2	20	0.00	3.4E-01	excluded
DAGLB	78731	ES	4	3	5	0.00	3.4E-01	included
SDHD	18738	ES	3.1:3.2:4	2	5	0.03	3.4E-01	included
RHOC	4241	ES	1.2:2.2:2.3:2.4	1.1	3	0.00	3.4E-01	excluded
AP1G1	37490	ES	2:03:04	1	5	-0.01	3.4E-01	excluded
ERMARD	78485	ES	18	16	19	0.00	3.4E-01	included
FIS1	81054	ES	3	2.1	4	0.00	3.4E-01	excluded
PLEKHG4	37005	ES	1.5:1.7	1.4	2	0.01	3.4E-01	included
COTL1	37853	ES	2	1	3	0.00	3.4E-01	included
MST4	90120	ES	11:12.1	10	12.2	0.00	3.4E-01	included
RAB6A	17706	ES	9	8	10	0.00	3.4E-01	excluded
MDFI	76117	ES	4	3	5	0.00	3.4E-01	excluded
LUC7L3	42483	ES	3	2	5	0.00	3.4E-01	included
ACY1	65154	ES	06:07.1	5	7.2	0.00	3.4E-01	included
PDE4DIP	4427	ES	9	8.3	10	0.01	3.4E-01	included
MAP3K4	78356	ES	24	23	25	-0.01	3.4E-01	excluded
RNF10	24770	ES	15	14	16	0.00	3.4E-01	included
C7orf43	80909	ES	8	7	9.2	0.00	3.4E-01	excluded
PLEKHA1	13355	ES	15.1	14	16	-0.02	3.4E-01	excluded
RARS	74465	ES	5	4.2	6	0.00	3.4E-01	included
PTPRA	58568	ES	9	8	10	0.00	3.4E-01	excluded
CYGB	43592	ES	5	4	6	0.00	3.4E-01	included
MTIF3	25538	ES	5	4.2	6	-0.01	3.4E-01	excluded
EMC4	29851	ES	2.1:2.2	1	3.1	0.00	3.4E-01	included
ADK	12260	ES	4	3	5	0.00	3.4E-01	included
BCOR	88829	ES	6	5	7	0.01	3.4E-01	included
TESK2	2684	ES	2	1	3	0.00	3.4E-01	included
ACN9	80558	ES	2	1	3	0.00	3.4E-01	included
EPS8L1	52011	ES	18:19:20:21	17	22	0.00	3.4E-01	included
CORO7	33671	ES	10:11:12:13:14:15:16:	9	18.2	0.00	3.4E-01	included
PPP1R9A	80521	ES	15.1:15.2:16:17:18.1	14	19	-0.01	3.4E-01	excluded
KLHL7	78955	ES	4	1	5	0.00	3.4E-01	included
SIPA1	390173	ES	12	11	13	0.00	3.5E-01	included
SLC23A2	58632	ES	8:09:10	7	11	0.00	3.5E-01	excluded
ASCC2	61685	ES	3:04	1	5	-0.02	3.5E-01	excluded
CLCN7	33101	ES	2	1	3	0.00	3.5E-01	included
RPGR	88809	ES	12	11	13	0.00	3.5E-01	excluded
RHOT1	40185	ES	19.2:19.3:20	19.1	22	0.00	3.5E-01	excluded
GOLIM4	67565	ES	7	6	8	0.00	3.5E-01	excluded
SPI1	15731	ES	2.2:3:4.1	1	5	0.00	3.5E-01	included
MUTYH	2653	ES	6.5:7:8:9	5	10	0.01	3.5E-01	included
RPS6KB2	17206	ES	5.1:6.2:7	4	8	0.01	3.5E-01	included
RPUSD3	63228	ES	3	2	4	0.00	3.5E-01	excluded
GNB2	80986	ES	3.2:4.1:4.2:5:6	1	7	0.00	3.5E-01	included
EIF4B	21922	ES	4	3	5.2	0.00	3.5E-01	included
PFDN5	22005	ES	5	1	6.2	0.00	3.5E-01	excluded
WSB1	39837	ES	2.1:2.2:3:4	1	5.1	0.00	3.5E-01	excluded
NDUFB4	66377	ES	2.1	1.1	2.3	0.00	3.5E-01	included
CCNT2	55411	ES	6.1:6.2:6.3	5	7.1	-0.02	3.5E-01	excluded
ERAP2	72873	ES	4	3	5.1	0.01	3.5E-01	included
RBBP8	44788	ES	19.2	18	20	0.00	3.5E-01	included
TAF9	72348	ES	3.2	2	5	0.00	3.5E-01	excluded
SPRYD3	21934	ES	2	1	3	0.00	3.5E-01	included
NPHP1	54935	ES	03:04.1	2	5	0.00	3.5E-01	included
MCCC1	67787	ES	5	4	7	0.01	3.5E-01	included
CDC14A	3885	ES	9	8	10	0.00	3.5E-01	excluded
HM13	58893	ES	11:12.1	10	13	-0.02	3.5E-01	excluded
PHF14	78802	ES	3	2	4	0.00	3.5E-01	included
MTSS1L	37410	ES	7	6	8	0.01	3.5E-01	included
DPP9	46825	ES	5	3	6	-0.02	3.5E-01	excluded

IL10RA	18955	ES	4	3	5	0.00	3.5E-01	included
NME6	64631	ES	1.2:1.3:2.1	1.1	3.2	0.00	3.5E-01	included
GFRA2	82907	ES	4.2	3.2	5	0.00	3.5E-01	excluded
ST3GAL3	2272	ES	12	9	13	-0.01	3.5E-01	excluded
CS	22422	ES	2	1	4.1	0.00	3.5E-01	excluded
ZNF185	90404	ES	12	11	13	-0.01	3.5E-01	excluded
ATG12	73036	ES	3	1.1	4	0.00	3.5E-01	included
KCTD20	75978	ES	3:04:05	2	6	0.00	3.5E-01	excluded
NAA30	27655	ES	2	1	3	-0.01	3.5E-01	excluded
TTC38	62702	ES	6	5	7	0.00	3.5E-01	excluded
FMNL2	55606	ES	27	26	28	-0.01	3.5E-01	excluded
GIPC1	47984	ES	2:03:04	1	5	0.00	3.5E-01	included
DERA	20596	ES	7	6	8.1	0.00	3.5E-01	included
SEPT2	58358	ES	5	4	7	0.00	3.5E-01	included
KIAA0586	107574	ES	34	33	36	-0.01	3.5E-01	excluded
CSNK1D	44309	ES	6	5	7	0.00	3.5E-01	excluded
CSNK1A1	74048	ES	4	2.2	5	0.00	3.5E-01	excluded
SMEK1	28881	ES	3:04	2	5	0.00	3.5E-01	included
EVC	99903	ES	24	22.1	25	-0.02	3.5E-01	excluded
MTCH1	76001	ES	2	1	3	0.00	3.5E-01	excluded
OCLN	72378	ES	5	4	6	0.00	3.5E-01	excluded
NUCB2	14524	ES	17	16	18	0.00	3.5E-01	excluded
NBPF12	7359	ES	12.2:13:14:15:16:17.1	12.1	17.2	-0.01	3.5E-01	excluded
ST3GAL3	2210	ES	16.2	15.1	19.1	0.00	3.5E-01	included
FAM13A	69918	ES	4.1	3	5.1	0.01	3.5E-01	included
CD44	14989	ES	7:8:9.2:10:11:12.1:13:	5	16.1	0.00	3.5E-01	included
ABAT	33907	ES	5	4	6	0.00	3.5E-01	excluded
RIC3	14232	ES	3.1:4:5:7.2	1	8	0.00	3.5E-01	excluded
TSEN2	63430	ES	7	6	8	0.01	3.5E-01	included
OS9	22705	ES	5.1:5.2:7.1:7.2:7.3:7.4:	4	9.2	0.00	3.5E-01	included
PAPD4	72634	ES	11	10	12	0.00	3.5E-01	excluded
SNX11	42174	ES	5	4	6	-0.02	3.5E-01	excluded
RRBP1	290840	ES	3.3:4:6:7:8:9:10:11:12<br>:13:14:15:16:17:18	3.2	19	-0.01	3.5E-01	excluded
SLC7A8	26713	ES	8:9:10:11:12	7	13	0.00	3.5E-01	included
SUN1	78526	ES	12	11	13	-0.01	3.5E-01	excluded
EXOSC1	12700	ES	6:07	5	8	0.00	3.5E-01	excluded
MMAB	24328	ES	2:04	1	6	0.00	3.5E-01	included
TXNL4A	46287	ES	6	3	7.2	0.00	3.5E-01	included
MPPE1	44654	ES	4	3.2	5	0.00	3.5E-01	excluded
CYP4B1	2842	ES	02:03.2	1	5	0.00	3.5E-01	included
CYB5A	45805	ES	5	4	6	0.00	3.5E-01	included
FCGRT	50968	ES	3:07	2.2	8.1	0.03	3.5E-01	included
ANXA11	12351	ES	3.2	1.1	4	0.00	3.5E-01	excluded
STX6	9135	ES	2:03	1	4	0.00	3.5E-01	included
COL13A1	11996	ES	42	41	43	0.01	3.5E-01	included
ECHDC2	319383	ES	12:13	11	14	0.00	3.5E-01	excluded
ING4	19918	ES	2:03	1	4	0.02	3.5E-01	included
POR	80139	ES	14.2:15.1:15.2:16.1	14.1	16.2	0.00	3.5E-01	excluded
DSCR3	60554	ES	4	3	5	0.00	3.5E-01	included
SLC7A7	26626	ES	2.3:3	1	4	0.00	3.5E-01	included
CD44	14978	ES	10	9.2	11	0.00	3.5E-01	included
LZTR1	61209	ES	3	2	4	0.00	3.5E-01	excluded
DHRS4	26781	ES	4	3	5	0.00	3.5E-01	excluded
UBE2G2	60831	ES	7	6	8	-0.01	3.5E-01	excluded
SLC37A3	81990	ES	7	6	8	0.00	3.5E-01	excluded
RAB31	44616	ES	8:09	7	10	0.00	3.5E-01	included
COPS7A	19944	ES	5:06	2.4	7	0.02	3.5E-01	included
PAPLN	28276	ES	25:26:27	24	28	0.00	3.5E-01	excluded
FOLH1	15817	ES	21	20	22	0.02	3.5E-01	included
FAM120AOS	86914	ES	4	3	5	0.01	3.5E-01	included
IL2RG	89396	ES	2:03:04	1	5	0.00	3.5E-01	included

MRPL55	10092	ES	2.5:2.6:2.8	2.2	2.9	0.00	3.5E-01	included
BLOC1S6	30441	ES	4.1	3	7.1	0.00	3.5E-01	excluded
PSMC3	15750	ES	3	2	4	0.00	3.5E-01	excluded
MTG2	60049	ES	4:05:06	3	7	0.00	3.5E-01	included
SYNE2	27858	ES	78	77	79	0.00	3.5E-01	included
GOLPH3L	7504	ES	3	2	4	0.00	3.5E-01	excluded
GRAMD1A	49012	ES	19	18	20	0.00	3.5E-01	included
CRELD1	63291	ES	3:04	2	5.1	0.00	3.5E-01	included
TMEM175	68432	ES	4.2	3	5.1	0.00	3.5E-01	excluded
FASTK	82341	ES	2	1.1	3.1	0.00	3.5E-01	excluded
DDHD2	83385	ES	6	5	7.1	0.00	3.5E-01	included
EBF1	74398	ES	14	13	15	0.00	3.5E-01	excluded
UBE2K	69077	ES	2:03:04	1	5	0.00	3.5E-01	included
NDUFS7	46470	ES	2.3:3	2.2	4	0.00	3.5E-01	excluded
C1S	20069	ES	7	6.2	8	0.00	3.5E-01	included
TMEM68	83871	ES	6:07	5	8	0.00	3.5E-01	included
RPL13A	264941	ES	2.1:2.2	1	3	0.01	3.5E-01	included
RNF141	14357	ES	3	2	4	0.00	3.5E-01	included
NNT	71965	ES	3	2	4	0.00	3.5E-01	included
UBE2D4	79375	ES	05:01.1	3	7.1	0.02	3.5E-01	included
PLAT	83579	ES	2	1	3	0.00	3.5E-01	excluded
ACAA1	64028	ES	3	2	4	0.00	3.5E-01	excluded
CCBL1	87786	ES	2:03	1	4	0.02	3.5E-01	included
PUS7L	21241	ES	1.2:1.3:2	1.1	3	-0.02	3.5E-01	excluded
VAMP7	90684	ES	3	2.2	4	0.00	3.5E-01	included
CNPY3	76199	ES	2	1	3	0.00	3.5E-01	excluded
MTFR1	83985	ES	4	3	5	0.00	3.5E-01	included
LGMN	29009	ES	6	5.2	7	0.00	3.5E-01	included
DMKN	49170	ES	8:12	7	13	0.02	3.5E-01	included
EPB41L2	77536	ES	22	21	23	0.00	3.5E-01	included
TMUB2	41825	ES	2.5	1	4.3	0.02	3.6E-01	included
MON1A	64929	ES	2	1	3	-0.01	3.6E-01	excluded
ARAP3	73826	ES	20	19	21	0.00	3.6E-01	included
SMAD5	73475	ES	2	1	3	-0.02	3.6E-01	excluded
HUS1	79617	ES	5	4	6	0.00	3.6E-01	included
SIPA1L1	28199	ES	15	14	16	-0.01	3.6E-01	excluded
UBE2F	58157	ES	11:12.1	10	13	0.00	3.6E-01	excluded
TMEM237	56852	ES	7:08	6	9	0.00	3.6E-01	excluded
COPS2	30515	ES	2:03	1	4	0.00	3.6E-01	excluded
GLMN	3757	ES	14	13	15	0.00	3.6E-01	excluded
PODN	3051	ES	5:6:7:8	4	9	0.00	3.6E-01	included
NEIL2	82635	ES	2.1	1.1	3	-0.01	3.6E-01	excluded
ERBB2IP	72260	ES	23	21	24.1	-0.01	3.6E-01	excluded
ARRB2	38570	ES	5	4.2	6.1	0.00	3.6E-01	included
SPATA7	28692	ES	7:08	6.2	9	-0.01	3.6E-01	excluded
SH2D4A	82872	ES	3	1	4	0.00	3.6E-01	excluded
TK2	36718	ES	5	4	6	0.00	3.6E-01	excluded
PPARD	75915	ES	2	1	4.1	0.00	3.6E-01	excluded
RABGAP1	87501	ES	5	3	6	0.00	3.6E-01	included
BCL2L13	60996	ES	4:06:07	3	8.1	0.00	3.6E-01	included
EPB41L2	77575	ES	17:18:20.1:20.2:21:22	14	23	0.00	3.6E-01	included
COL8A1	65802	ES	2	1	3	0.01	3.6E-01	included
SLC9B2	70175	ES	16	15	17.1	0.02	3.6E-01	included
MACF1	1889	ES	29	28	30	0.00	3.6E-01	included
TTLL3	63223	ES	4:5:6.3:6.5:7	3	8.1	-0.01	3.6E-01	excluded
ADCK3	10039	ES	8.1:8.2:9:10	7	11	0.00	3.6E-01	included
SKA2	42755	ES	4.1	1.1	5	0.02	3.6E-01	included
CD44	14981	ES	8:9.2:10:11	7	12.1	0.03	3.6E-01	included
IYD	78144	ES	6.1:6.2	5	7	0.00	3.6E-01	included
NKIRAS2	40983	ES	5.5:5.6:6	5.3	7	0.03	3.6E-01	included
IFNAR2	60392	ES	9	8	10.2	-0.01	3.6E-01	excluded
CXCL12	11347	ES	2.2:3.1:5.1	2.1	5.2	0.01	3.6E-01	included

C14orf2	29535	ES	4	2	5	0.00	3.6E-01	excluded
CREM	11266	ES	8	4	9.2	0.00	3.6E-01	excluded
ZCCHC10	73332	ES	2	1	4	-0.02	3.6E-01	excluded
TPD52L1	77415	ES	08:09.1	6	10	0.01	3.6E-01	included
GUCD1	61412	ES	6	5	7.1	-0.01	3.6E-01	excluded
ZNF706	84741	ES	5	4	6	0.00	3.6E-01	excluded
ACSF2	42397	ES	3	1	4	0.00	3.6E-01	excluded
ECD	12132	ES	6	5	7	0.00	3.6E-01	included
ARPC1A	80604	ES	3	2	4	0.00	3.6E-01	included
ERI2	34396	ES	9	8	10	0.00	3.6E-01	excluded
AGO2	85284	ES	17	16	18	0.00	3.6E-01	excluded
NKIRAS2	40982	ES	5.4:5.5:5.6:6	5.3	7	0.03	3.6E-01	included
DTNB	52870	ES	6:07	5	8	0.00	3.6E-01	excluded
CALCOCO1	22110	ES	8	7	9	0.00	3.6E-01	included
CIZ1	87720	ES	5	4	6	0.00	3.6E-01	excluded
SRP9	9996	ES	3	2	5	-0.01	3.6E-01	excluded
LRCH3	68325	ES	15	14	16	-0.02	3.6E-01	excluded
C16orf62	34327	ES	6	5	7.2	0.00	3.6E-01	excluded
PRICKLE3	89101	ES	9.2:10.1	9.1	10.2	0.00	3.6E-01	included
LYRM7	73221	ES	3:04	2	5	0.00	3.6E-01	included
SOD2	78308	ES	6	5.1	7	0.00	3.6E-01	excluded
SUPT20H	25666	ES	14	13	15	0.00	3.6E-01	included
DCUN1D2	26379	ES	2:03	1	4	0.00	3.6E-01	included
BIN3	83049	ES	4	3	6	0.00	3.6E-01	included
BRE	53056	ES	2	1	3	0.01	3.6E-01	included
TRAPPC11	71287	ES	2.2:3:4:5:6:7:8:9:10.1	2.1	10.2	0.00	3.6E-01	excluded
EML4	53355	ES	4	3	5	0.00	3.6E-01	excluded
TBCB	49353	ES	4:5.1:5.2	2.3	6	0.00	3.6E-01	excluded
RNF121	17453	ES	5:6.1:6.2:7:8	3	9	-0.01	3.6E-01	excluded
ZNF7	85662	ES	5.2	4.2	6.1	-0.01	3.6E-01	excluded
G6PC3	41768	ES	2.1:2.2	1.1	3	0.00	3.6E-01	included
GTF2A2	30938	ES	2	1.1	3	0.00	3.6E-01	excluded
LILRB1	51921	ES	14	13	15	0.00	3.6E-01	excluded
MECOM	67571	ES	22	21	23	0.00	3.6E-01	excluded
PDE8A	32339	ES	10	9	11	0.01	3.6E-01	included
IDH3A	32016	ES	03:04.1	2	4.2	-0.01	3.6E-01	excluded
C6orf106	75787	ES	4	3	5	0.00	3.6E-01	included
CYB561D1	4023	ES	2:03	1.2	4.2	-0.01	3.6E-01	excluded
HARS2	73752	ES	2.2:3:4:5	1.3	6.1	0.00	3.6E-01	excluded
STOML1	31622	ES	8.2	7	9	0.00	3.6E-01	excluded
TG	319588	ES	2.2:3	2.1	4	-0.02	3.6E-01	excluded
ZNF468	51631	ES	5	4	6	0.01	3.6E-01	included
SLC6A9	2544	ES	6	2	7	0.01	3.6E-01	included
MKL1	62353	ES	9	8	10	0.00	3.6E-01	excluded
CLNS1A	271082	ES	3:04	2	6	0.02	3.6E-01	included
PPIP5K1	30269	ES	27:28:00	26	29	0.00	3.6E-01	included
PIGT	59549	ES	4	3	5.2	0.00	3.6E-01	excluded
LGALS1	62144	ES	3:04	2.2	5	0.01	3.6E-01	included
CCDC64	24716	ES	6:07	5	8	-0.01	3.6E-01	excluded
TPGS2	45274	ES	02:04.1	1	4.2	0.00	3.6E-01	included
LRRC37A3	43081	ES	7:08	6	9	0.01	3.6E-01	included
CORO1A	36090	ES	11	10	12	0.00	3.6E-01	included
KLHL5	69043	ES	3	2.2	4	0.00	3.6E-01	excluded
CLTB	74654	ES	3	2	4	0.00	3.6E-01	excluded
ZNF254	48840	ES	5	4	6	-0.01	3.6E-01	excluded
UBAC2	26180	ES	3:04	2	5	0.00	3.6E-01	included
DDX20	4193	ES	3	2	4.2	0.00	3.6E-01	excluded
PRR5L	15426	ES	10	9	11	0.01	3.6E-01	included
SEPT10	54915	ES	2	1	3.1	0.00	3.6E-01	included
CMC2	37731	ES	6	5	9	0.00	3.6E-01	included
P4HB	44183	ES	2.2:3:4:5:6:7:8:9:10:1	2.1	11.2	0.00	3.6E-01	included
INTS1	78577	ES	29.2:30.1	29.1	30.2	0.00	3.6E-01	excluded

CLN6	31366	ES	3:4:5.1:6	2	7.1	0.00	3.6E-01	excluded
PAK4	49764	ES	2.2:2.3	1	4	0.01	3.6E-01	included
EXOC6	12545	ES	22	21	23	0.00	3.6E-01	excluded
FCHSD1	73821	ES	12:13.1	11	13.2	0.00	3.6E-01	included
PCCB	66917	ES	4	3	6	0.00	3.6E-01	excluded
RIT1	8133	ES	3	1	4	0.00	3.6E-01	excluded
TRMT10B	86431	ES	4.2:5	4.1	6.1	0.00	3.6E-01	included
UBXN11	101233	ES	3:04:05	2	7	0.02	3.6E-01	included
PRDM2	724	ES	8:09:10	7	12	-0.03	3.6E-01	excluded
GSK3B	66364	ES	9	8	10	0.00	3.6E-01	included
HFE	75578	ES	3	2.2	5.1	0.00	3.6E-01	excluded
ERBB2IP	72269	ES	15:16:17.1:17.2:18:19	14	22	0.00	3.6E-01	included
ADAP2	40131	ES	4	3.2	5	0.00	3.6E-01	included
CYTH1	43895	ES	6	5	7	0.00	3.6E-01	included
FBXO25	82547	ES	10	9	11	0.00	3.6E-01	included
ZCCHC11	3010	ES	5	4	6	0.00	3.6E-01	excluded
PDPK1	33290	ES	06:07.1	4.1	8	0.00	3.6E-01	excluded
WBP2	43519	ES	9	8.2	10.1	0.00	3.6E-01	included
TBC1D13	87784	ES	3	2	4	-0.01	3.6E-01	excluded
ARHGAP8	62631	ES	11	10	14	0.00	3.6E-01	excluded
TFDP2	67101	ES	4	3.1	6	-0.01	3.7E-01	excluded
CALM3	50568	ES	5	2	6.1	0.00	3.7E-01	excluded
GBAS	79769	ES	6	5	7	0.00	3.7E-01	included
CLN6	31362	ES	5.1:5.2:6:7.1	4	7.2	0.01	3.7E-01	included
TK2	36721	ES	4	2.4	5	0.00	3.7E-01	included
FKBP9	79205	ES	3	1	4	0.00	3.7E-01	excluded
TUBGCP2	13537	ES	3.2	2	4.2	0.00	3.7E-01	included
KPTN	50650	ES	4.2:5	4.1	6	0.01	3.7E-01	included
MUTYH	2600	ES	11:12:13:14:15	10	16	0.00	3.7E-01	included
DMKN	49155	ES	11	9	12	0.01	3.7E-01	included
DBNL	79397	ES	03:04.1	2.2	5.2	0.00	3.7E-01	included
ZNF772	52265	ES	2.1:3	1	5	0.02	3.7E-01	included
MSR1	82780	ES	11	9	12	0.00	3.7E-01	excluded
SPG21	31150	ES	6	5	7	0.00	3.7E-01	included
MRPL55	10140	ES	2.2:2.4:2.5:2.6:2.8	1.1	2.9	0.00	3.7E-01	included
GEMIN2	27355	ES	7	6	8	-0.01	3.7E-01	excluded
CCDC91	20920	ES	4:05:06	3	8.2	0.01	3.7E-01	included
TMEM107	39120	ES	3.2:3.3:3.4	2	3.7	0.04	3.7E-01	included
PIK3C2A	14518	ES	2	1	3	-0.01	3.7E-01	excluded
ANKRD65	168	ES	1.4:2	1.3	3	0.03	3.7E-01	included
UEVLD	14674	ES	5	4	6	0.00	3.7E-01	included
RPL39L	68072	ES	4	3	5	0.00	3.7E-01	excluded
SUMO1	56934	ES	6.1:6.2	5.2	8.1	0.00	3.7E-01	included
ASPSCR1	44259	ES	7.1:8:9	6	10	0.00	3.7E-01	excluded
SLC25A17	62364	ES	6	5.2	7.2	0.00	3.7E-01	excluded
NUP50	62641	ES	6	5	7	0.00	3.7E-01	excluded
PHF20	59255	ES	2	1	3	-0.02	3.7E-01	excluded
ZMYND8	59718	ES	9	8.2	10	0.00	3.7E-01	excluded
RNF121	17459	ES	04:06.1	3	6.2	0.01	3.7E-01	included
PEX14	620	ES	3	2	4	0.00	3.7E-01	included
LRRCC1	84318	ES	10	9	11	0.00	3.7E-01	excluded
CYB561D2	96394	ES	2	1.5	3	0.00	3.7E-01	included
LASP1	40614	ES	3	2	5	0.00	3.7E-01	included
TPD52L1	77413	ES	8:9.1:9.2	6	10	0.00	3.7E-01	included
TMEM164	89873	ES	4:05	3	6	0.00	3.7E-01	included
NEURL4	38917	ES	3	2	4	0.01	3.7E-01	included
MARCH7	55703	ES	7	6	8	0.00	3.7E-01	excluded
VWA9	31202	ES	2.3:3.1	2.2	3.2	0.00	3.7E-01	excluded
FANCL	53653	ES	9.1	8	10	0.00	3.7E-01	included
ATP2A2	24420	ES	7	6	8	0.00	3.7E-01	excluded
XAF1	38790	ES	8	6	9.1	0.01	3.7E-01	included
ISCA2	28407	ES	3.1	2	4.1	0.00	3.7E-01	included

CDH1	37245	ES	11	10	12	0.00	3.7E-01	included
USP30	24272	ES	6:07	5	8	0.00	3.7E-01	included
NIPSNAP1	61620	ES	5	4	6	0.00	3.7E-01	excluded
SPATA6L	85759	ES	11:12	10	14	0.02	3.7E-01	included
LMBR1L	21528	ES	3:4.1:4.2:5	1	6.1	0.00	3.7E-01	included
CASP1	18523	ES	4:05	3.4	6	0.00	3.7E-01	excluded
BRCC3	90677	ES	8	7	9	-0.01	3.7E-01	excluded
ALDOC	39926	ES	7	6	8	0.00	3.7E-01	included
LEPROTL1	83276	ES	3	1	4	0.00	3.7E-01	included
RPLP0	24729	ES	5.1:5.2:5.3	4.2	6.2	-0.01	3.7E-01	excluded
WDR1	68784	ES	3:04:05	2	6	0.00	3.7E-01	excluded
SEPT10	54912	ES	4:05	3.1	6	0.00	3.7E-01	excluded
SMARCE1	40881	ES	2.2:3	1	4.1	0.00	3.7E-01	excluded
KIAA0101	31117	ES	2	1	3	0.00	3.7E-01	included
YAF2	21115	ES	6:07	5.2	9.1	-0.02	3.7E-01	excluded
MS4A6A	16059	ES	6	5.1	7	0.00	3.7E-01	excluded
RNF214	18905	ES	13	12	14	0.00	3.7E-01	included
HNRNPC	26541	ES	5:6.1:6.2	4.2	6.3	0.00	3.7E-01	included
ARMC10	81164	ES	2	1	5.1	0.01	3.7E-01	included
MTUS1	82823	ES	5	3	8	0.01	3.7E-01	included
IL1RL2	54787	ES	4:05	3	6	-0.01	3.7E-01	excluded
C11orf49	15622	ES	4:07	3	10	-0.03	3.7E-01	excluded
ANP32E	7437	ES	4	3	5.1	0.00	3.7E-01	included
RNF34	24870	ES	2	1	3	-0.01	3.7E-01	excluded
ERRFI1	537	ES	3.2	2	3.4	-0.01	3.7E-01	excluded
BRCC3	90676	ES	9	7	10	0.00	3.7E-01	excluded
IL4R	35688	ES	7	6	8	0.00	3.7E-01	excluded
MCRS1	21590	ES	3:05	1	6	0.00	3.7E-01	excluded
NSUN2	71517	ES	3	2	4	0.00	3.7E-01	included
MORF4L1	32134	ES	4:05	3.1	7	0.00	3.7E-01	included
GOLT1B	20701	ES	7	6	8	0.00	3.7E-01	included
SLC35A1	76963	ES	3	2	4	0.00	3.7E-01	included
CBWD5	86506	ES	8	7	10	0.00	3.7E-01	included
SCFD1	27062	ES	21	20	22	0.00	3.7E-01	excluded
ATP6V0E2	82208	ES	5	4.2	6	0.00	3.7E-01	excluded
FEZ2	53199	ES	5	4	6	0.00	3.7E-01	excluded
ADAM15	7904	ES	21.2:22.1	20	23	-0.01	3.7E-01	excluded
EPRS	9855	ES	10	9	11	0.00	3.7E-01	included
MRPL55	10143	ES	2.2:2.5:2.6:2.8	1.1	2.9	0.00	3.7E-01	included
COPRS	40165	ES	4	3	5	0.00	3.7E-01	excluded
ENO2	20013	ES	6:07	5	8	0.00	3.7E-01	excluded
TLK2	42902	ES	17	16	18	0.00	3.7E-01	excluded
MKRN1	82003	ES	4:05	1	6	0.00	3.7E-01	excluded
DYM	45469	ES	9	8	10	0.00	3.7E-01	excluded
ARID4B	10342	ES	23	22	24	0.01	3.7E-01	included
WDR45	89082	ES	07:02.2	5	9.2	0.00	3.7E-01	excluded
PAOX	13555	ES	7	6	8	0.01	3.7E-01	included
STK4	59509	ES	5	4	6	0.00	3.7E-01	included
GRAMD1A	49014	ES	5	4	6	0.00	3.7E-01	excluded
VPS41	79291	ES	9	8	10	0.00	3.7E-01	excluded
UBXN2A	52796	ES	8	7	9	0.00	3.7E-01	excluded
CC2D2A	68810	ES	1.2:3.1:3.2	1.1	3.3	0.00	3.7E-01	included
ST7	81561	ES	17	16.2	18	0.00	3.7E-01	excluded
METTL23	43632	ES	1.4:2:3	1.3	4.1	0.00	3.7E-01	excluded
SLC22A18	13937	ES	6:07	5	8	0.00	3.7E-01	excluded
NASP	2754	ES	2	1	3	0.02	3.7E-01	included
SFI1	61870	ES	11:12:14:15	10	16	0.00	3.7E-01	included
RPL18	50776	ES	05:06.1	4	6.2	0.00	3.7E-01	excluded
DEF8	38193	ES	2.1:2.2	1	4	0.01	3.7E-01	included
ICAM2	43050	ES	2	1.1	3.2	0.00	3.8E-01	included
CLN3	35732	ES	6	5	7	0.00	3.8E-01	excluded
ARMC6	48571	ES	2.2:5:6	1.1	7	-0.01	3.8E-01	excluded

ADCK3	10040	ES	8.2:9:10	7	11	-0.01	3.8E-01	excluded
METTL13	8996	ES	2.2:3.1	2.1	3.2	0.00	3.8E-01	excluded
SUMF2	79788	ES	8	7	10.2	0.00	3.8E-01	excluded
NPRL3	32811	ES	3:04	2	5	0.00	3.8E-01	excluded
TANGO2	61124	ES	7.1:7.2	6	8	-0.01	3.8E-01	excluded
PTPN2	44723	ES	8	7	9.1	0.00	3.8E-01	excluded
NPIPB3	93938	ES	12.1:12.2:12.3:12.4:12	11	13.3	-0.01	3.8E-01	excluded
WIBG	22293	ES	4	2	6	-0.01	3.8E-01	excluded
ZWINT	11812	ES	5.2:6.1	5.1	6.2	0.01	3.8E-01	included
MRPS25	63542	ES	3.2:4.1	3.1	4.2	0.03	3.8E-01	included
SHANK3	62903	ES	18.2:20	18.1	21	0.00	3.8E-01	included
CIRBP	46445	ES	5.2:7.2	5.1	7.3	0.00	3.8E-01	excluded
KPNA1	66438	ES	6	5	7	-0.01	3.8E-01	excluded
CLTC	42787	ES	3.2:4:5:6:7:8:9:10:11:<br>12:13:14:15:16:17:18:	3.1	22.2	0.00	3.8E-01	excluded
ARPP19	30672	ES	04:05.2	2.6	6	-0.01	3.8E-01	excluded
NDRG1	85237	ES	4	3	5.1	0.00	3.8E-01	excluded
SNX7	3840	ES	9	8	10	0.00	3.8E-01	excluded
DDX19A	37380	ES	2:03	1	4	0.00	3.8E-01	excluded
GPI	48959	ES	6	5	7	0.00	3.8E-01	included
SMG7	9183	ES	3	1	4	0.01	3.8E-01	included
RRBP1	58741	ES	3.1:3.2:3.3	2	4	0.00	3.8E-01	excluded
CTSH	32139	ES	3	2	4	0.00	3.8E-01	excluded
MCRS1	21591	ES	2	1	3	0.00	3.8E-01	excluded
CD86	66421	ES	4	3	5	0.00	3.8E-01	included
SEC14L1	43711	ES	22.2:22.4	22.1	22.5	0.00	3.8E-01	included
RSL1D1	34042	ES	2.1:2.2:3:4:5:6.1	1.1	6.2	0.00	3.8E-01	excluded
NEMF	27448	ES	18	17	19	0.00	3.8E-01	included
ARFGAP2	15661	ES	3:4.2:5:6.1:6.2:7:8	2	9	0.01	3.8E-01	included
CAMKK2	24854	ES	15	14	16	0.01	3.8E-01	included
SMAD4	45565	ES	8:09	7	10	0.00	3.8E-01	included
RBM4B	17111	ES	3	2.2	4	0.00	3.8E-01	excluded
ASNSD1	300787	ES	3.3:4.1:4.2:5	1	6	-0.01	3.8E-01	excluded
HNRNPC	26547	ES	2.6	2.4	3.2	0.00	3.8E-01	included
TTC8	28771	ES	7:8.1:8.2:9:10.1:11:14	2	17	0.00	3.8E-01	excluded
DUOXA1	30391	ES	4	3	5	0.00	3.8E-01	included
TEC	69205	ES	14	13	15	0.01	3.8E-01	included
PIGG	68362	ES	9.1:9.2	8	10.1	0.00	3.8E-01	excluded
VRK2	53647	ES	5.2:6.1	5.1	6.2	0.00	3.8E-01	excluded
CCNG1	74437	ES	2.2:3.1	2.1	3.2	0.00	3.8E-01	excluded
NACA	22492	ES	3.1:3.2:3.3:3.4:3.5	2.2	4.2	0.00	3.8E-01	included
AKAP8L	48080	ES	4.4	4.1	5	0.01	3.8E-01	included
MYO1E	30933	ES	5	4	6	0.00	3.8E-01	excluded
TMEM230	58642	ES	1.2:3	1.1	4	0.00	3.8E-01	included
CCDC159	47684	ES	8:09:10	7	11	0.00	3.8E-01	included
ZFPL1	16752	ES	3.1:3.2	2.1	4	-0.01	3.8E-01	excluded
EEF1D	85445	ES	8.3:9:10.1:11:12.1:12.	8.2	13.2	-0.01	3.8E-01	excluded
SPIDR	83775	ES	25	24	26	0.00	3.8E-01	excluded
RHOT1	40178	ES	20	19.3	22	0.02	3.8E-01	included
PPAP2A	72039	ES	2	1	4	0.01	3.8E-01	included
GTDC1	55502	ES	13:14	12	15	0.00	3.8E-01	included
CAMK2G	12246	ES	16	14	17	0.01	3.8E-01	included
ATIC	57353	ES	3	2	4	0.00	3.8E-01	included
REPS1	77949	ES	12.2:12.3:13	11	14	0.00	3.8E-01	included
SF3B2	16984	ES	5	4	6	0.00	3.8E-01	excluded
IPO5	26157	ES	10	9.2	11	0.00	3.8E-01	excluded
RRAS2	14468	ES	4	2	7.2	0.00	3.8E-01	excluded
MRPS25	63547	ES	3.1	2	4.1	0.00	3.8E-01	included
MAT2B	74454	ES	4	3	5	0.00	3.8E-01	excluded
CDK7	72326	ES	5.1:5.2:6:7:8	4	9	0.00	3.8E-01	excluded
GPS1	44290	ES	1.5	1.1	2.3	0.00	3.8E-01	excluded
ZNF827	70766	ES	2	1	3	0.00	3.8E-01	included

ZFYVE28	68563	ES	7	6	10	0.00	3.8E-01	excluded
USP8	30590	ES	6:7:8:9:10.1	5	11	0.00	3.8E-01	included
SCRN1	79102	ES	4.2	3	5	0.00	3.8E-01	excluded
NCOR2	25148	ES	20	19	21	0.01	3.8E-01	included
PDCD5	48885	ES	4.1:4.2:5.1:5.3:5.4	3	5.5	0.00	3.8E-01	included
GIPC1	47985	ES	3:04	1	5	-0.01	3.8E-01	excluded
CEL	88011	ES	10	9	11	0.00	3.8E-01	excluded
MAGOHB	20473	ES	4:05	3	6	0.00	3.8E-01	included
ACSF2	42398	ES	2	1	4	0.00	3.8E-01	excluded
TEAD4	19745	ES	4	3	5	0.00	3.8E-01	excluded
CTSH	32143	ES	2:4:5:6:7.1	1.1	7.2	0.00	3.8E-01	included
PTK7	76249	ES	11	10	12	0.00	3.8E-01	excluded
NUDT5	10770	ES	4	3	5	0.00	3.8E-01	excluded
HAUS4	26674	ES	5:06:07	4	8	0.00	3.8E-01	excluded
MAGI1	65531	ES	24	23.1	25	0.00	3.8E-01	excluded
RMND5B	74836	ES	4.2:5.1	4.1	5.2	0.01	3.8E-01	included
ATRIP	64660	ES	6	5	7	0.00	3.8E-01	included
EXOC5	27647	ES	4	3.2	5	0.00	3.8E-01	included
THOP1	46625	ES	13	12	14	0.00	3.8E-01	excluded
ARRB2	38574	ES	4.2	1	5	0.00	3.8E-01	included
GGA2	35588	ES	7.2:8:9:10:11:12:13:14:15:16:17.1	7.1	17.2	0.00	3.8E-01	excluded
SUMF2	79806	ES	4:06	3	7	-0.02	3.8E-01	excluded
HNRNPA1	22149	ES	6.2:7.1:7.2:9.1	6.1	9.2	0.00	3.8E-01	included
MOGS	54072	ES	2	1.3	3.1	0.00	3.8E-01	included
LETMD1	21760	ES	4:05:06	2	7	-0.01	3.8E-01	excluded
FCHSD2	17673	ES	19	18.1	20	0.00	3.8E-01	included
MLLT3	85981	ES	12	11	13	0.00	3.8E-01	included
KTN1	27633	ES	46	45	47	0.00	3.8E-01	included
GANAB	16381	ES	2.1:2.2:3:4	1	5.1	0.00	3.8E-01	included
TUFT1	7638	ES	4	1	5	0.00	3.8E-01	excluded
COPS5	84058	ES	10	9	11.1	0.00	3.8E-01	included
TTC17	15458	ES	2	1	3	0.00	3.8E-01	excluded
ANKRD54	62168	ES	4	2	5.1	0.00	3.8E-01	excluded
SMOX	58625	ES	5:6.1:6.2:6.3:7	4	9	0.00	3.8E-01	excluded
KIF9	64503	ES	2.2:2.3:3	2.1	4	0.00	3.8E-01	included
RPS15A	34258	ES	3.2:3.3:4	2.2	5.1	0.01	3.8E-01	included
BBIP1	13092	ES	4:5.1:5.2	3	7	0.01	3.8E-01	included
SMPD1	14107	ES	2.5	2.3	3	0.00	3.8E-01	included
IKBKB	83692	ES	3:4.1:5:6.1:6.2:7	1.4	9	0.00	3.8E-01	included
SLC39A6	45210	ES	2	1.1	3	0.00	3.8E-01	included
SUN1	78535	ES	10:11	9.1	12	0.00	3.8E-01	included
C14orf159	28868	ES	05:02.2	1	7	0.00	3.8E-01	excluded
WWP2	37321	ES	2.2:3	1.1	4.3	-0.01	3.8E-01	excluded
POMGNT1	2788	ES	9	8	10	0.00	3.8E-01	included
HAUS4	26675	ES	6:07	4	8	0.01	3.8E-01	included
YIPF2	47603	ES	6	5	7	0.00	3.8E-01	included
MDH1	53739	ES	4:5:6:7	1	8	0.00	3.8E-01	included
P4HA1	12121	ES	12.2:13.2	12.1	14	0.00	3.8E-01	included
PRDX3	13258	ES	2.2:3.1	2.1	3.2	0.00	3.8E-01	excluded
MYO9A	31494	ES	2	1	3	0.00	3.8E-01	excluded
RNPS1	33259	ES	2.3:2.4:3	1.1	4	0.00	3.8E-01	excluded
HAX1	7820	ES	2.1:2.2:2.3:3.1	1	3.2	0.01	3.8E-01	included
USP54	12166	ES	19	18	20	-0.01	3.8E-01	excluded
IL33	85816	ES	5	4	6	0.00	3.8E-01	excluded
HNRNPR	1044	ES	6	5	7	0.00	3.9E-01	excluded
TATDN1	138619	ES	1.2:2:3:4.1:4.2:5:6	1.1	7	0.01	3.9E-01	included
DDX31	87988	ES	2	1	4	-0.01	3.9E-01	excluded
AASDH	69348	ES	4	3	5	0.00	3.9E-01	included
PLBD1	20552	ES	2	1	3	0.00	3.9E-01	excluded
COPZ1	22167	ES	6	5	8	0.00	3.9E-01	excluded
ZNF707	85485	ES	4.2:5.1:5.2	1	6	0.03	3.9E-01	included

GABARAPL1	20395	ES	2.10:2.11:2.12:2.13:2.	2.6	3	0.00	3.9E-01	excluded
TNFSF13	38975	ES	03:04.1	2.2	4.2	0.00	3.9E-01	included
HSPB2-C11orf52	18705	ES	2	1	3	0.00	3.9E-01	excluded
TLE2	46648	ES	10.5:11:12:13:14:15:1 6.1:16.2:17:18:19:20:	10.4	23.2	0.00	3.9E-01	included
CHIT1	9445	ES	10	9	11	0.02	3.9E-01	included
FAM216A	24429	ES	3	2	4	0.00	3.9E-01	included
TPD52	84282	ES	3	1	5	0.00	3.9E-01	excluded
DNAJC10	56463	ES	18	17	19	0.00	3.9E-01	included
BCS1L	57549	ES	1.4:1.5:1.6	1.1	2	0.00	3.9E-01	included
HN1L	33120	ES	5	4	6	0.00	3.9E-01	excluded
ACAA1	64023	ES	5:06	4	7	0.00	3.9E-01	excluded
HAGHL	32976	ES	3	2.2	4	0.00	3.9E-01	included
THOC1	44445	ES	5	4	6	0.01	3.9E-01	included
BOD1L1	68797	ES	2	1	3	0.02	3.9E-01	included
KCTD5	33294	ES	3	2	4	0.00	3.9E-01	excluded
MAZ	35943	ES	3.2:4.2:5.1:6.1:7.1:7.2:	3.1	7.4	0.00	3.9E-01	excluded
HARS	73725	ES	5:6.1:6.2	4	7	0.00	3.9E-01	included
PRMT10	70796	ES	2	1	3	0.01	3.9E-01	included
TAZ	90588	ES	6	5	7	0.00	3.9E-01	included
XPR1	9132	ES	11	10	12	0.00	3.9E-01	included
DHRS11	40498	ES	1.2:2.1	1.1	2.2	0.00	3.9E-01	included
TOM1L1	42546	ES	4	3.3	6.1	0.00	3.9E-01	included
ENTPD1	12658	ES	4	2	5	0.00	3.9E-01	included
ZFAND6	32172	ES	4	2	6	-0.02	3.9E-01	excluded
CEP85	1245	ES	3	2	4	0.00	3.9E-01	included
SNX11	42173	ES	6	4	7	0.00	3.9E-01	excluded
ITGB1	11188	ES	18	16	19	0.01	3.9E-01	included
ECD	12133	ES	2	1	3	0.00	3.9E-01	excluded
YTHDF3	83978	ES	4.2:5.2	4.1	6	0.00	3.9E-01	excluded
MARS	22617	ES	3:4:5.1:5.2:6:7.1	2	7.2	0.00	3.9E-01	included
FUZ	51077	ES	3	2.3	4	0.00	3.9E-01	excluded
HHAT	9697	ES	5	4	6	-0.01	3.9E-01	excluded
SEPSECS	68955	ES	2:03	1	4	0.00	3.9E-01	included
METAP2	23790	ES	9	8	10	0.00	3.9E-01	excluded
MDM2	22969	ES	10:11	9	12.1	0.00	3.9E-01	excluded
PPP1R21	53526	ES	17	16	18	0.00	3.9E-01	included
COL16A1	1493	ES	44	43	45.1	-0.02	3.9E-01	excluded
PFAS	39148	ES	5	4	6	0.01	3.9E-01	included
TNKS1BP1	15829	ES	6.2:7:8:9:10:11:12.1	6.1	12.2	0.00	3.9E-01	excluded
NF2	61658	ES	3	2	4	0.00	3.9E-01	excluded
SLK	13030	ES	13	12	14	-0.01	3.9E-01	excluded
TOM1L1	42547	ES	3.1:3.2:3.3	2	4	0.01	3.9E-01	included
RPS15	46486	ES	02:03.2	1.5	3.3	-0.02	3.9E-01	excluded
GATSL3	61707	ES	6:08	5	9	0.00	3.9E-01	included
SKP2	71804	ES	3	2	4	0.00	3.9E-01	included
GIT2	24381	ES	17.2:18.1:18.2	17.1	20	0.01	3.9E-01	included
TEX9	30761	ES	3:04:05	2	6	0.00	3.9E-01	included
ACAP3	74	ES	21	20	22	0.00	3.9E-01	excluded
ZNF385A	22179	ES	7	6	8	0.00	3.9E-01	excluded
MLPH	58116	ES	8:09:10	7	11	0.00	3.9E-01	included
CEP164	18915	ES	14	13	15	-0.02	3.9E-01	excluded
CHI3L2	4149	ES	4.2	3.2	5	0.01	3.9E-01	included
KIF16B	58722	ES	25	24	26	0.00	3.9E-01	excluded
RPGRIP1L	36424	ES	24	23	25	-0.02	3.9E-01	excluded
TTC8	28775	ES	6:7:8.1:8.2:9:11:14:15	2	17	0.00	3.9E-01	included
APOBEC3B	62270	ES	5	4	6.1	0.00	3.9E-01	included
TRPM4	50906	ES	3:04:05	2	6	0.00	3.9E-01	included
ASAH1	82847	ES	4	3	5	0.00	3.9E-01	included
TMEM143	50745	ES	3.1:3.2:4	2	5	0.03	3.9E-01	included
ENAH	9993	ES	4	3	5	0.00	3.9E-01	included
ZNF266	47348	ES	04:01.1	2	6.2	-0.02	3.9E-01	excluded

TRIQK	84504	ES	05:06.1	2.1	6.2	0.02	3.9E-01	included
STK3	84656	ES	12	11	13.1	0.00	3.9E-01	excluded
KPNB1	42091	ES	1.2:2	1.1	4	0.00	3.9E-01	excluded
RNF14	73854	ES	3	2.1	4	0.00	3.9E-01	included
ABCB9	25003	ES	9	8	10	-0.01	3.9E-01	excluded
TBC1D1	69015	ES	20:21.1	19	21.2	0.00	3.9E-01	excluded
PCMTD2	60205	ES	2.2:3:4:5.1	2.1	5.2	0.00	3.9E-01	excluded
GEMIN7	50398	ES	2	1.1	3	-0.02	3.9E-01	excluded
IKBKB	83699	ES	2	1.4	3	0.01	3.9E-01	included
GLRB	70965	ES	12	11	13	-0.01	3.9E-01	excluded
CCAR1	11959	ES	4	3	5	-0.01	3.9E-01	excluded
VPS8	67977	ES	3	2	4	-0.03	3.9E-01	excluded
PPFIA1	17394	ES	17	16	18	0.00	3.9E-01	included
WDR81	38366	ES	6	5	7	0.00	3.9E-01	included
LMBR1L	21524	ES	5	3	6.1	0.01	3.9E-01	included
ZNF335	59652	ES	2:03:04	1	5	0.00	3.9E-01	included
TFDP1	26389	ES	13.2:13.3	12	14	0.01	3.9E-01	included
DCAF6	91280	ES	13:01.1	10	15	0.02	3.9E-01	included
NARFL	32985	ES	3.1	2.4	4	0.00	3.9E-01	excluded
ANO10	64348	ES	6	5	7	0.00	3.9E-01	excluded
FPGT	3452	ES	3	2.1	4	-0.01	3.9E-01	excluded
C17orf85	38491	ES	8	7	9	0.01	3.9E-01	included
NSF	42037	ES	4	3	5	0.00	3.9E-01	included
ZDHHC15	89527	ES	7	6	8	0.01	3.9E-01	included
MAGED2	89254	ES	5.2:5.4	5.1	5.5	-0.01	3.9E-01	excluded
GNPDA1	73860	ES	3.2:4:5.1	3.1	5.2	0.00	3.9E-01	included
ASNSD1	56514	ES	2	1	3.3	0.00	3.9E-01	included
NFIX	47906	ES	12	11	13	-0.01	3.9E-01	excluded
LDLR	47627	ES	4	3	5	0.00	3.9E-01	included
TNFAIP2	29436	ES	10	9.2	11	0.00	3.9E-01	excluded
STK38L	20871	ES	3	2	4.1	-0.01	3.9E-01	excluded
TEP1	26439	ES	5:06	4	7	0.00	3.9E-01	excluded
MTHFD2	54027	ES	2:03	1	4	0.00	3.9E-01	excluded
STAT6	22525	ES	2.2:4	2.1	5	0.00	3.9E-01	excluded
EIF4ENIF1	61858	ES	12	11.2	13	0.00	3.9E-01	included
PEX11A	32438	ES	2	1	3.1	-0.01	3.9E-01	excluded
ATP6AP2	88833	ES	2	1	3	0.00	3.9E-01	included
COPS7A	19945	ES	3:04:06	2.4	7	0.02	3.9E-01	included
NDUFB4	66376	ES	1.2:2.1	1.1	2.3	0.00	3.9E-01	excluded
SLC6A9	2542	ES	6:07	2	8	-0.01	3.9E-01	excluded
RNF7	67078	ES	1.2:2	1.1	3	0.01	3.9E-01	included
TMX3	45763	ES	5	4	6	0.00	3.9E-01	excluded
FAM172A	72787	ES	2	1	3	-0.01	3.9E-01	excluded
UBE2H	81756	ES	4:05	3	6	0.00	3.9E-01	included
CLN3	35733	ES	6:7:8:9:10	5	11	0.00	4.0E-01	included
CGRRF1	27594	ES	2	1	3	0.00	4.0E-01	included
CLDND1	65784	ES	3.4	1	4.1	0.00	4.0E-01	excluded
TAF1	89423	ES	37	36	38	0.01	4.0E-01	included
STARD3	40662	ES	5	4.1	6	-0.01	4.0E-01	excluded
CERS5	21679	ES	8	1	9	0.00	4.0E-01	excluded
NECAP2	823	ES	9.2:9.4	9.1	9.5	0.02	4.0E-01	included
PCCB	66903	ES	13	12	14	0.00	4.0E-01	excluded
RAB3GAP1	55432	ES	26	25	27.1	0.00	4.0E-01	included
MAX	27945	ES	5.2:5.3:5.5:5.6:5.7	5.1	5.8	0.00	4.0E-01	excluded
ALG5	25652	ES	04:05.1	3	5.2	0.00	4.0E-01	included
HNRNPH1	74907	ES	5	4	6	0.00	4.0E-01	included
VAPA	44623	ES	2	1	3	0.00	4.0E-01	excluded
KIAA1586	76576	ES	3	2	4	-0.01	4.0E-01	excluded
SAE1	50627	ES	4:05	3	6	0.00	4.0E-01	excluded
ARL8B	63023	ES	6:07	5	8	0.00	4.0E-01	included
GAS7	39265	ES	11:12:13:14:15:16:17:	10	18.2	0.00	4.0E-01	included
DBI	55111	ES	1.3:3.1	1.2	3.2	0.00	4.0E-01	excluded

HADHA	52887	ES	4	3	5	0.00	4.0E-01	excluded
YWHAE	38293	ES	6	3	7	0.00	4.0E-01	included
DCTN6	83280	ES	5	4	6	0.00	4.0E-01	excluded
DHRS4	26784	ES	4:05	3	7.1	0.01	4.0E-01	included
PRMT2	60956	ES	8:09:10	6.1	11	0.00	4.0E-01	excluded
C1orf109	1819	ES	3.3:4.1	3.2	4.2	-0.02	4.0E-01	excluded
C1D	53819	ES	2.1:2.2	1.2	3.1	0.00	4.0E-01	included
TRAF3IP3	9685	ES	14	13	15	0.00	4.0E-01	included
PLEKHA7	14511	ES	2.2:3:4:5:6:7:8:9:10:1 1:12:13:14:15:16:17:1 8:19:20.1:20.2:21:22.1	2.1	22.2	0.00	4.0E-01	included
AP2B1	40324	ES	4.2	3	5	0.00	4.0E-01	excluded
MVK	24344	ES	2.2:3:5:6:7:8.1:9.1	1	9.2	0.00	4.0E-01	excluded
IDH3A	32028	ES	2:3:4.1:4.2:5:6.1:6.2:7 :8.2:9:10	1	11.1	0.00	4.0E-01	included
RBM25	93514	ES	11.3	11.1	12	0.01	4.0E-01	included
C14orf2	29531	ES	5:06	2	7.1	0.02	4.0E-01	included
DCAF4	28240	ES	5:06	4.2	7.1	0.00	4.0E-01	excluded
TSSK6	48646	ES	1.2:2.1	1.1	2.2	0.00	4.0E-01	included
TUBGCP4	30254	ES	14	13.2	15	-0.01	4.0E-01	excluded
STX8	39217	ES	5	2	6	0.01	4.0E-01	included
VAV1	47089	ES	22	21	23	0.00	4.0E-01	excluded
ANAPC5	24865	ES	8.1:8.3	7	9.1	0.00	4.0E-01	included
RDH11	28090	ES	2.1:2.2:3:4	1	5	0.00	4.0E-01	excluded
ADAM15	7903	ES	21.1:21.2:22.1	20	23	-0.01	4.0E-01	excluded
CYP27A1	57566	ES	2	1	3	0.00	4.0E-01	excluded
C5orf45	74983	ES	3.1:3.2	2.1	4	0.00	4.0E-01	excluded
PLA2G15	37202	ES	3	1	4.1	0.00	4.0E-01	included
RNH1	13677	ES	3	1	4.2	0.01	4.0E-01	included
DHX36	67333	ES	20	19	21	0.00	4.0E-01	excluded
MAEA	68483	ES	6	5.1	7	0.00	4.0E-01	included
LRRC49	31457	ES	10	7	11	0.01	4.0E-01	included
ACSM2B	34363	ES	2.1:2.2	1	3	0.01	4.0E-01	included
C2orf43	52791	ES	2	1	3	-0.01	4.0E-01	excluded
CTSH	32142	ES	1.2:2:4:5:6:7.1	1.1	7.2	0.00	4.0E-01	excluded
RAD17	72354	ES	6	5	7	0.00	4.0E-01	excluded
RBM5	64959	ES	6.3	6.1	7	0.02	4.0E-01	included
TWSG1	44601	ES	4:05	3	6.1	0.00	4.0E-01	included
QDPR	68855	ES	7	5	8.1	-0.01	4.0E-01	excluded
CYYR1	60293	ES	3	2	4	0.00	4.0E-01	excluded
SLC25A3	23855	ES	7	6	8	0.00	4.0E-01	excluded
CRYZ	3469	ES	2:03	1	4	0.01	4.0E-01	included
PCCB	66921	ES	3	2	6	0.00	4.0E-01	excluded
IARS	86836	ES	3	2	4	0.00	4.0E-01	excluded
C4orf29	70562	ES	4	3	6	0.01	4.0E-01	included
PSMA2	79319	ES	3	2	4	0.00	4.0E-01	included
INTS6	25947	ES	8	5	9	0.01	4.0E-01	included
AIFM1	90078	ES	2	1	4	0.00	4.0E-01	included
PRSS16	75683	ES	10	9	11	0.01	4.0E-01	included
GABARAP	38869	ES	1.2:1.5	1.1	2.1	0.02	4.0E-01	included
BCS1L	57551	ES	1.4:1.5	1.1	2	0.00	4.0E-01	included
TMEM245	87161	ES	7	6	8	0.00	4.0E-01	included
ZBTB25	27886	ES	7.2	6	8	0.00	4.0E-01	excluded
SETX	87977	ES	24	23	25	0.00	4.0E-01	included
TBC1D9B	74992	ES	3	2	4	0.00	4.0E-01	included
TMTC4	26197	ES	5:06	3	7	0.00	4.0E-01	excluded
MCCC1	67784	ES	5:07	4	8	0.00	4.0E-01	excluded
UBA2	48970	ES	4:05:06	1	7	0.00	4.0E-01	excluded
FOLR2	17587	ES	2	1	3	0.00	4.0E-01	excluded
SCFD1	27073	ES	4:06:07	3	8	0.00	4.0E-01	included
GNE	86343	ES	3	2	4	0.00	4.0E-01	excluded
PBX1	8792	ES	10	9	12	0.00	4.0E-01	included

USP10	37860	ES	5:06	4	7	0.00	4.0E-01	included
DECR1	84402	ES	8.1	7	9	0.00	4.0E-01	included
SLC25A17	62366	ES	5.1:5.2	4	7.2	0.00	4.0E-01	included
TRA2B	68038	ES	3	1	4	0.00	4.0E-01	excluded
ZNF780A	49867	ES	4	3	5	0.00	4.0E-01	excluded
CAPN1	16805	ES	8	7	9	0.00	4.0E-01	included
METTL10	13407	ES	6	5	7	-0.01	4.0E-01	excluded
PTDSS1	84626	ES	3:04	2	5	0.00	4.0E-01	excluded
SNF8	42252	ES	3	2	5	0.00	4.0E-01	included
CDK5RAP2	87387	ES	14	13	15	0.01	4.0E-01	included
NPHP4	357	ES	13:14.1	11	14.2	0.00	4.0E-01	excluded
CAMK1	63175	ES	3	2	4	-0.01	4.0E-01	excluded
TAPT1	68832	ES	2	1	3	0.00	4.0E-01	included
ANXA6	74154	ES	6.1:6.2:6.3:7.1:7.2:8:9 :10:11:12:13.1:13.2:1 4:15.1:15.2:16:17	5	18	0.00	4.0E-01	excluded
ANXA11	12349	ES	3.2:4	1.1	5.3	0.00	4.0E-01	excluded
PMM2	33947	ES	07:09.1	3	9.2	0.00	4.0E-01	included
BSDC1	1594	ES	4.1:4.2:4.3:5	3	6.1	0.00	4.0E-01	included
TMED1	47593	ES	3.1:3.2	1	4	0.00	4.0E-01	excluded
SLC25A39	41843	ES	2.2:3:4:5.2	1	6	0.00	4.0E-01	excluded
CALCOCO2	42231	ES	3	2	6	-0.02	4.0E-01	excluded
LUC7L3	42481	ES	3:4.1:4.2	2	5	0.00	4.0E-01	excluded
ATP9A	59803	ES	5:6:7:8:9	4	10	0.00	4.0E-01	included
ARNTL	14445	ES	6.2	5	7	0.00	4.0E-01	excluded
SH3YL1	52498	ES	19	18	20	0.01	4.0E-01	included
SPAG16	57335	ES	07:08.1	5	9	0.00	4.0E-01	excluded
ACSS2	59044	ES	4:5.1:6:7:8:10:11:12:1	3	16	0.00	4.0E-01	excluded
BFAR	34097	ES	3:04	2	5	0.00	4.0E-01	excluded
TMEM41B	14312	ES	6	5	7.1	0.00	4.0E-01	included
SKA2	42733	ES	4.1:4.2	2	5	0.01	4.0E-01	included
CNOT8	74258	ES	1.5:2:3	1.4	5	0.00	4.0E-01	excluded
ZNF90	95106	ES	6	3	7	0.02	4.0E-01	included
PXK	65447	ES	2:03:04	1	5	0.00	4.1E-01	included
NOXA1	88303	ES	5:06	4	7	0.00	4.1E-01	included
RAB43	66695	ES	6	5	7	0.00	4.1E-01	excluded
TJAP1	76280	ES	2	1	5	0.00	4.1E-01	included
PLCB4	58678	ES	38	37	39	0.00	4.1E-01	included
TM6SF1	32257	ES	4.2:5.2:5.3:6.2:7:8	4.1	9	-0.01	4.1E-01	excluded
GRB10	79726	ES	5.2	1	11	-0.02	4.1E-01	excluded
SLC2A6	88069	ES	8	7	9	0.01	4.1E-01	included
CALCRL	56490	ES	2	1	3	-0.01	4.1E-01	excluded
CYFIP2	74370	ES	6.2:8.1:10.1:10.2:10.3	6.1	11	0.00	4.1E-01	included
CACNA2D1	80256	ES	30:31:00	29.2	32	0.00	4.1E-01	included
ASF1B	47965	ES	2	1	3.1	0.00	4.1E-01	included
H2AFV	79574	ES	3	2	4.1	0.00	4.1E-01	excluded
STAU1	59739	ES	2:03:04	1	5	-0.02	4.1E-01	excluded
NFYC	2022	ES	13	12	14	0.00	4.1E-01	included
SUMF2	79803	ES	5.1:5.2:6	3	7	0.01	4.1E-01	included
ACSL5	13113	ES	10	9	11	0.00	4.1E-01	excluded
IL32	33373	ES	4	3	5.1	0.00	4.1E-01	excluded
DENND1A	87517	ES	23	21	24	-0.01	4.1E-01	excluded
PPP6R2	96242	ES	3	2	5	0.01	4.1E-01	included
MAN2B2	68721	ES	6.2:7.1	6.1	7.2	0.00	4.1E-01	included
CTTN	133810	ES	11	9	13	0.01	4.1E-01	included
PET100	47119	ES	2	1	3.1	0.00	4.1E-01	included
ATP2A3	38516	ES	21.1	20	23.1	-0.01	4.1E-01	excluded
TULP3	19738	ES	6	5	8	0.00	4.1E-01	excluded
URI1	48865	ES	4	3	5	0.00	4.1E-01	excluded
SYNRG	40529	ES	10	9	11	0.00	4.1E-01	included
TDRD7	87007	ES	13	12	14	0.00	4.1E-01	excluded
UTP14A	90055	ES	6	5	8	0.00	4.1E-01	excluded

ACAD9	66680	ES	1.2:2.1:2.2:2.3	1.1	3.2	0.00	4.1E-01	excluded
RABAC1	50103	ES	4	3	5	0.00	4.1E-01	included
OCLN	72383	ES	4	3	6	0.01	4.1E-01	included
PDE9A	60723	ES	5:06:07	3	8	0.00	4.1E-01	excluded
ZNF410	28334	ES	6.1:6.2	4.2	7.1	0.00	4.1E-01	included
NME7	8912	ES	12	11.1	13	0.00	4.1E-01	excluded
MTO1	76751	ES	4	3	5	-0.01	4.1E-01	excluded
ARPP19	30690	ES	2.5:2.6	2.2	5.2	0.01	4.1E-01	included
ARMC1	83980	ES	4	3	5	0.00	4.1E-01	included
PARD3	11217	ES	5	4	7	-0.01	4.1E-01	excluded
LUC7L3	42485	ES	03:04.2	1	5	-0.01	4.1E-01	excluded
ASNSD1	56513	ES	02:03.2	1	3.3	0.00	4.1E-01	included
SEMA4F	54141	ES	3	1	4	0.00	4.1E-01	included
NBPF11	7326	ES	25.1:25.2:25.3	24	26	0.00	4.1E-01	excluded
GSTZ1	28585	ES	8	7.2	9	0.00	4.1E-01	excluded
KTN1	27639	ES	2	1	3.2	0.01	4.1E-01	included
NF1	40149	ES	45.2:46	44.2	47	0.00	4.1E-01	excluded
ZNF180	50335	ES	4	2.2	6	-0.02	4.1E-01	excluded
PML	31648	ES	5:6.1:6.4:6.6:6.7	4	6.8	0.00	4.1E-01	included
ZNF512	53022	ES	7	6	8	0.00	4.1E-01	included
SCP2	3050	ES	2	1	3	0.00	4.1E-01	excluded
EIF3E	84880	ES	2.2	1	3	0.00	4.1E-01	excluded
GEMIN6	53290	ES	5.1	4	6	0.00	4.1E-01	included
DHFR	72657	ES	5	4	6.1	0.00	4.1E-01	excluded
CNDP2	45815	ES	3	2.3	4	0.00	4.1E-01	included
TRIT1	1918	ES	9	8	10	0.00	4.1E-01	included
GSTZ1	28591	ES	3	1	5	0.00	4.1E-01	excluded
CTDP1	46247	ES	11	10	12	0.01	4.1E-01	included
OXNAD1	63644	ES	4	3.2	6	0.00	4.1E-01	excluded
FCHSD2	17674	ES	12	11	13	0.00	4.1E-01	excluded
KCNAB2	368	ES	16	15	17	0.00	4.1E-01	excluded
CLASP1	55166	ES	40	39	41	0.00	4.1E-01	excluded
TFEB	76125	ES	6	5.2	7	0.00	4.1E-01	included
HAUS3	68549	ES	4.2:5.1	4.1	5.2	-0.01	4.1E-01	excluded
YWHAB	59489	ES	2	1	3	0.00	4.1E-01	excluded
SNAPIN	7752	ES	2	1	3	0.00	4.1E-01	included
SAE1	50624	ES	8	6	9	0.00	4.1E-01	included
SLC25A39	41838	ES	04:05.1	3	5.2	0.00	4.1E-01	included
UAP1	8751	ES	9.2	8	10	-0.02	4.1E-01	excluded
CNOT2	23378	ES	3.1:3.2:4	2	6	0.00	4.1E-01	excluded
CERS5	21683	ES	5	1	8	0.00	4.1E-01	included
CCT3	8239	ES	3	1	4	-0.02	4.1E-01	excluded
TAF1C	37823	ES	6.1	5.2	7	0.00	4.1E-01	excluded
NDRG2	26498	ES	06:07.2	5.2	8.1	0.00	4.1E-01	included
SAR1B	73418	ES	6	5.2	8	0.02	4.1E-01	included
SIRT3	13599	ES	6.1	5.3	8.1	0.00	4.1E-01	included
NLRP1	38724	ES	8	7	9	0.00	4.1E-01	excluded
LARS	73905	ES	5	4	6	0.00	4.1E-01	excluded
CLN6	31363	ES	5.1:6:7.1	4	7.2	0.00	4.1E-01	included
ANXA6	74149	ES	7.2:8:9	7.1	10	0.00	4.1E-01	included
FBXO28	9935	ES	3	2	4	0.00	4.1E-01	excluded
SRL	33637	ES	3	1	4	0.03	4.1E-01	included
SNW1	28622	ES	2.1:2.2:3:4	1	5	0.00	4.1E-01	included
ARMCX4	89654	ES	10.1	9.2	11.1	0.03	4.1E-01	included
KLC1	29485	ES	13.3:15	13.2	18	-0.01	4.1E-01	excluded
D2HGDH	58419	ES	7.1:7.2:7.3:8:9	6	11.2	-0.02	4.1E-01	excluded
SRP54	27181	ES	4.1:4.2	3	5	0.00	4.1E-01	excluded
RAP1B	22938	ES	6	5	7.1	0.00	4.1E-01	excluded
G6PC3	41765	ES	2.1	1.2	3	0.01	4.1E-01	included
DCAF6	8888	ES	3	2	4	0.00	4.1E-01	included
ARHGDIA	44194	ES	5	4	6	0.00	4.1E-01	included
GOLGA5	29012	ES	12	11	13	0.00	4.1E-01	included

SLC25A30	25809	ES	3	1	4	0.00	4.1E-01	excluded
CSDE1	4339	ES	2	1	4	0.00	4.1E-01	included
CTSH	32141	ES	2:3:4:5:6:7.1	1.1	7.2	0.00	4.1E-01	excluded
DHRSX	88403	ES	5	4	6	0.01	4.1E-01	included
TXNL4B	37543	ES	4	3	5	0.00	4.1E-01	excluded
LEF1	70295	ES	5	4	6.1	0.00	4.2E-01	excluded
TTC7B	28826	ES	18	17	19	-0.01	4.2E-01	excluded
ADCY6	21466	ES	15	14	16	0.00	4.2E-01	excluded
CPSF3L	91	ES	5.2:6.1:6.2:7.2:8	4	9	-0.01	4.2E-01	excluded
UBE2J2	57	ES	2.1:4	1.1	5	-0.02	4.2E-01	excluded
ASCC2	61674	ES	6:7:8:9.1:9.2	5	10	0.00	4.2E-01	excluded
CSF2RA	88371	ES	12:13	11	14	0.00	4.2E-01	excluded
TCF7L2	13154	ES	6.1:6.2	5	8	-0.01	4.2E-01	excluded
MAEA	68478	ES	7	6	9	0.00	4.2E-01	excluded
ATXN3	28932	ES	4.2:5	3.2	6	0.00	4.2E-01	excluded
GPR56	94167	ES	5.2	1	7.2	-0.01	4.2E-01	excluded
DCUN1D5	18476	ES	2:03:04	1.1	5	0.00	4.2E-01	included
NAA40	16551	ES	4.2	3	5	-0.01	4.2E-01	excluded
UBXN11	101231	ES	4:05:06	2	7	0.01	4.2E-01	included
ZNF346	74702	ES	5.1:5.2	4	6.2	0.00	4.2E-01	included
ELP4	14824	ES	10	9	11	0.00	4.2E-01	excluded
DNLZ	88156	ES	2	1	3	0.00	4.2E-01	excluded
SLC27A4	87739	ES	2.2:3:4:5:6:7:8:9:10.1	2.1	10.2	0.00	4.2E-01	excluded
ARFGAP2	15664	ES	3:4.2:5:6.2:7:8	2	9	0.00	4.2E-01	included
UACA	31441	ES	8:09:10	7	11	0.00	4.2E-01	included
MAPKAP1	87587	ES	2	1	3	0.00	4.2E-01	included
CCND3	76157	ES	6	5	7	0.00	4.2E-01	included
DHRS4	26785	ES	5	3	7.1	0.00	4.2E-01	included
CNST	10497	ES	3	2	4	0.01	4.2E-01	included
TAF1C	37838	ES	2.1:2.2	1	3	0.00	4.2E-01	included
RAB17	58123	ES	2.2:2.3:3:4:5:6.1	2.1	6.2	0.00	4.2E-01	excluded
ACTR10	27683	ES	2:03	1	4	0.00	4.2E-01	excluded
APOC1	50370	ES	4	3.2	5.1	0.00	4.2E-01	included
CAMK2B	79514	ES	14.1:14.2:15:17	12	21.1	-0.01	4.2E-01	excluded
COL1A1	316105	ES	20:21:22:23:24:25:26:<br>27:28:29:30:31:33:34:<br>35:36:37:38:39:40	19	41	0.01	4.2E-01	included
TMEM107	39114	ES	3.2:3.4:3.5:3.6	2	3.7	0.02	4.2E-01	included
SMC1A	89216	ES	4:05:06	3	7	0.00	4.2E-01	excluded
RRN3	34140	ES	10	9	11.1	0.00	4.2E-01	included
NHP2L1	62449	ES	3.2	2	4	0.01	4.2E-01	included
NUDT1	78610	ES	3.2:3.3	1	4	0.00	4.2E-01	excluded
ANXA2	30949	ES	6	5	7	0.00	4.2E-01	included
PRPSAP2	39665	ES	13	12.1	14	0.00	4.2E-01	included
C5orf28	71952	ES	5	3.2	6	0.01	4.2E-01	included
PLLP	36539	ES	2	1	3	0.00	4.2E-01	included
ABHD5	64354	ES	3:04	2	5	0.00	4.2E-01	included
HSDL2	87245	ES	5:06	4	7	0.00	4.2E-01	included
PHF12	40029	ES	4	3	5	0.00	4.2E-01	included
RPS6KA1	1287	ES	12	11	13	0.00	4.2E-01	included
ACOX3	68767	ES	17	16	18	-0.01	4.2E-01	excluded
FOSL1	16944	ES	2:3.1:3.2	1	4	0.00	4.2E-01	included
ATP2B4	9451	ES	19	18	20	0.00	4.2E-01	excluded
SNX11	42182	ES	03:01.1	1.2	6	0.01	4.2E-01	included
DSCR3	60558	ES	2	1	3	0.00	4.2E-01	excluded
GIGYF2	58019	ES	12	10	13	0.00	4.2E-01	excluded
INTS4	17993	ES	17:18.2	16	19	-0.01	4.2E-01	excluded
RBM42	49231	ES	4	3.2	6.2	0.00	4.2E-01	excluded
RABL6	88217	ES	14.2:15	14.1	16.2	0.00	4.2E-01	excluded
MAD1L1	78599	ES	2.1:2.2:3:4	1	5	0.00	4.2E-01	included
MRPL55	10125	ES	2.2:2.5	1.2	2.9	0.00	4.2E-01	included
NDUFB2	97765	ES	8	5	10	0.01	4.2E-01	included

R3HCC1L	12753	ES	7	6	8	0.00	4.2E-01	included
SLC8B1	24642	ES	2	1	3.1	0.00	4.2E-01	included
TJP3	46729	ES	3.2	1	4	0.00	4.2E-01	included
GBA	8045	ES	2.2:3	1	4	0.00	4.2E-01	included
GPATCH11	53214	ES	3:04	2	5	0.01	4.2E-01	included
C19orf82	47378	ES	3	2	4.1	-0.03	4.2E-01	excluded
QTRTD1	66248	ES	2:3.1:3.2:4:5	1	6	0.01	4.2E-01	included
CARD14	44029	ES	16	15	17.1	0.00	4.2E-01	included
EIF4E2	58002	ES	3	2	4	0.00	4.2E-01	included
PRKCQ	10704	ES	16	15	17	0.00	4.2E-01	included
GUCY1B3	70950	ES	3	1	5	0.00	4.2E-01	excluded
PPFIBP1	20895	ES	03:04.1	2	5	0.00	4.2E-01	included
TMEM71	85186	ES	6	5.2	7	-0.01	4.2E-01	excluded
OSBPL9	2976	ES	10	7	11	0.00	4.2E-01	excluded
CRTC2	7760	ES	2:3:4:5	1.2	6	0.00	4.2E-01	included
LSM1	83374	ES	3	2	4	0.00	4.2E-01	excluded
RTCA	3878	ES	3	2.1	4	0.00	4.2E-01	included
CCNL1	67387	ES	4.1:4.2:4.3	3	5	0.01	4.2E-01	included
RIPK4	60675	ES	08:09.1	7	9.2	0.00	4.2E-01	excluded
DMTF1	80307	ES	6	4.2	7	0.00	4.2E-01	excluded
CABIN1	61391	ES	9	8	10	0.00	4.2E-01	excluded
BRD9	71463	ES	18.1:18.2	17	20	0.00	4.2E-01	included
MRPL55	10131	ES	2.2:2.4:2.5:2.6:2.7:2.8	1.1	2.9	0.00	4.2E-01	included
CDIP1	33761	ES	4	3.2	5.1	0.00	4.2E-01	excluded
MLH3	28467	ES	11	10.2	12	0.00	4.2E-01	included
DAG1	64883	ES	3:04:05	2.1	7	0.00	4.2E-01	included
CAMTA2	38640	ES	3	1	4	0.00	4.2E-01	included
PRIMPOL	71305	ES	09:10.1	7	10.2	0.00	4.2E-01	included
NAPG	44630	ES	2	1	3	0.00	4.2E-01	included
PCMTD2	60206	ES	2.2:3:4	2.1	5.2	0.00	4.2E-01	excluded
FYN	77274	ES	12	10	13	0.00	4.2E-01	included
CDC23	73522	ES	2	1	3.1	0.00	4.2E-01	excluded
GLYCTK	65206	ES	5.2:6.1	4	6.3	-0.01	4.2E-01	excluded
TMOD2	30624	ES	8	7	9	0.00	4.2E-01	excluded
RAN	25206	ES	1.3	1.1	2.1	0.00	4.2E-01	included
NIF3L1	95742	ES	1.2:3.2	1.1	3.3	0.02	4.2E-01	included
RUVBL2	50865	ES	1.4:1.5:1.6	1.2	2	-0.02	4.2E-01	excluded
G3BP2	69550	ES	9	8	10	0.00	4.2E-01	included
COL1A2	265306	ES	15:37	14	38	-0.01	4.2E-01	excluded
UIMC1	74688	ES	7.1:7.2	6.1	8	-0.01	4.2E-01	excluded
MDM2	22999	ES	5.2:6:7:8.1:8.2:9:10:1<br>1:12.1:12.2:12.3	5.1	12.4	0.00	4.2E-01	excluded
NUDT6	70524	ES	6	5	7	0.00	4.2E-01	included
RELT	17694	ES	6:07	5	8	0.00	4.2E-01	included
SNF8	42263	ES	3:5:7.1:7.2	2	9	0.01	4.2E-01	included
UBAP1	86152	ES	5	1	6	0.00	4.2E-01	excluded
METTL16	38401	ES	5	4	6	0.00	4.2E-01	excluded
PLA2G6	62206	ES	12	11	13	0.01	4.2E-01	included
FUCA2	77981	ES	3:04	2	5	0.00	4.2E-01	excluded
FCGRT	50964	ES	4	3	7	0.00	4.2E-01	included
NEDD9	75342	ES	6.1:6.2	5	7	0.02	4.2E-01	included
PRSS22	33312	ES	3.2:4	3.1	5	0.00	4.2E-01	included
ASXL1	58956	ES	4	3	5	-0.01	4.2E-01	excluded
ZNF559	47286	ES	3.1:4:5	2.2	6.1	0.02	4.2E-01	included
MLH1	63946	ES	1.2:2.1:2.2	1.1	4.1	0.00	4.2E-01	excluded
PELI3	17035	ES	3	2	4.1	0.01	4.3E-01	included
NUP93	36499	ES	24	23	25	0.00	4.3E-01	excluded
UBAP1	86154	ES	2	1	5	-0.03	4.3E-01	excluded
HSF1	85558	ES	12	11	13	0.00	4.3E-01	included
CARS	13948	ES	3	1	4	0.01	4.3E-01	included
TRDMT1	10885	ES	6	5	7	0.01	4.3E-01	included
NARF	44404	ES	7	6	9	0.00	4.3E-01	included

CD74	74084	ES	2:03	1	4	0.00	4.3E-01	included
CERS4	94932	ES	11	9	12	-0.02	4.3E-01	excluded
SSR2	8149	ES	6.1	5	7	0.00	4.3E-01	excluded
PCCB	66911	ES	6:7.1:7.2	3	8	0.00	4.3E-01	included
HARS2	73746	ES	4:05	3	6.1	0.00	4.3E-01	excluded
OTUB1	16563	ES	4.2:5:6	3	7.1	0.00	4.3E-01	excluded
SLC4A5	54036	ES	27	26	28	0.00	4.3E-01	excluded
CCDC64	24715	ES	7	6	8	0.01	4.3E-01	included
LETMD1	21749	ES	3.2	2	4	-0.01	4.3E-01	excluded
USP3	31053	ES	3.2:4:5:7:8:9:10	1	11	0.00	4.3E-01	excluded
SERAC1	78268	ES	12	11	13	0.00	4.3E-01	excluded
GOLM1	86750	ES	3.2:4.1	3.1	4.2	0.00	4.3E-01	included
UBE2L3	61231	ES	3	2	4	0.00	4.3E-01	included
PHB2	20050	ES	3.1:3.2:4.1:4.2:5.1:5.3:	2	7.1	0.00	4.3E-01	included
YIF1A	17012	ES	2	1	3	0.00	4.3E-01	included
TMED4	79540	ES	4.1	3	5	0.00	4.3E-01	excluded
IMPA1	84299	ES	6.1:6.2	5	8	0.00	4.3E-01	included
DCTN2	22645	ES	3:04	2	10	0.00	4.3E-01	excluded
WBSCR22	80003	ES	6	5	7	0.00	4.3E-01	excluded
MUTYH	2651	ES	6.3:6.4:6.5:7:8:9	5	10	0.01	4.3E-01	included
BAD	16614	ES	3	1.3	4	0.00	4.3E-01	excluded
PDGFC	70955	ES	5:06	4.3	7	0.00	4.3E-01	included
MICU1	12095	ES	9	8	10.1	0.00	4.3E-01	included
MTMR10	29791	ES	18.1:18.3	16	18.4	0.00	4.3E-01	excluded
SRP72	69362	ES	7:08	6	9	0.00	4.3E-01	included
THSD1	25997	ES	2:03	1	4	0.00	4.3E-01	included
DLG1	68292	ES	16:17:18	15	19	0.00	4.3E-01	excluded
ORAI2	81092	ES	2.1:2.2	1	3	0.00	4.3E-01	excluded
IMMT	54410	ES	6.2:6.3	5	7.2	0.00	4.3E-01	excluded
MOCS2	72000	ES	6	5	7.1	0.00	4.3E-01	included
UBL7	31720	ES	1.4	1.2	3	0.00	4.3E-01	excluded
FAM114A2	74206	ES	1.3	1.1	2.1	0.00	4.3E-01	excluded
FNDC1	78298	ES	10	9	11	0.00	4.3E-01	excluded
PXK	65450	ES	2	1	3	0.00	4.3E-01	excluded
NUP35	56475	ES	6.1:6.2	5	7	0.00	4.3E-01	included
CCL4L1	40441	ES	3.1:3.2:3.13	1.1	3.14	0.00	4.3E-01	excluded
DOCK9	26177	ES	52:53:00	51.1	54	0.00	4.3E-01	excluded
LAMP2	90002	ES	3:04	1	5	0.00	4.3E-01	excluded
ST3GAL3	2199	ES	19.1:19.2:20	16.2	21	0.00	4.3E-01	excluded
ATG4D	47539	ES	5	3.2	6	0.02	4.3E-01	included
ALOX5	11359	ES	13	12	14	0.00	4.3E-01	included
RAD17	72360	ES	5	3.2	6	0.00	4.3E-01	excluded
TBC1D22A	62730	ES	6	1	7	0.00	4.3E-01	included
GSTM2	4062	ES	7	6	9	0.00	4.3E-01	excluded
INPP5K	38325	ES	4:5.1:5.2	1	7	0.00	4.3E-01	excluded
POLM	79458	ES	6.3:7	6.2	8	0.01	4.3E-01	included
PACRGL	68885	ES	7	6	8	-0.01	4.3E-01	excluded
RTN2	50465	ES	8	7	9	0.00	4.3E-01	included
HDAC11	63503	ES	5:6.1:6.2:7:8	4	9	0.00	4.3E-01	included
COX7A2	76782	ES	3	2.2	4.1	0.00	4.3E-01	excluded
TCOF1	74070	ES	22	21	23	0.01	4.3E-01	included
SCMH1	2050	ES	8	6	9	-0.02	4.3E-01	excluded
SLC37A4	19078	ES	2.1	1	3.1	0.00	4.3E-01	excluded
HDAC2	77303	ES	3	1	4	0.00	4.3E-01	included
SYNE2	27855	ES	104	103	105	0.00	4.3E-01	included
GGA3	43407	ES	4:05:06	2	8	0.00	4.3E-01	excluded
RAD52	19643	ES	9	8	10	0.02	4.3E-01	included
ACTG2	53999	ES	5	4.1	7.2	0.00	4.3E-01	excluded
AGAP6	564718	ES	3	2	4	-0.01	4.3E-01	excluded
DNASE1L1	90579	ES	2.2:3.2	1	4	0.01	4.3E-01	included
C12orf4	19768	ES	7	6	8	0.00	4.3E-01	included
RPLP1	31401	ES	2:03	1	4	0.00	4.3E-01	included

SUSD1	87239	ES	15	14	17	0.00	4.3E-01	included
MTHFSD	37924	ES	4.1	2.2	5	0.00	4.3E-01	included
NUP98	13996	ES	32:33:00	31	34	0.00	4.3E-01	included
STAP2	46792	ES	3	2	4	0.00	4.3E-01	included
PIK3IP1	61843	ES	2:03	1	4	0.00	4.3E-01	excluded
TSPAN7	88818	ES	4	1	7	0.00	4.3E-01	excluded
CORO7	33695	ES	3:4:5.1:5.2:6.1:6.2:7.1	1.2	9	0.00	4.3E-01	excluded
THUMPD2	53336	ES	8	7	9	0.01	4.3E-01	included
ABI1	11045	ES	10	9	11.1	0.00	4.3E-01	included
PRDM2	723	ES	10	9	12	-0.02	4.3E-01	excluded
CCT6A	79782	ES	3	2	4	0.00	4.3E-01	included
MAP9	70918	ES	2	1	3	0.00	4.3E-01	included
TTLL4	57562	ES	9	8	10	0.00	4.3E-01	included
MRPL43	12857	ES	4	3.1	5	0.01	4.3E-01	included
KIFC2	85625	ES	14.2:15:16.1	14.1	16.2	0.00	4.3E-01	excluded
PNPLA6	47112	ES	18	17	19	0.00	4.3E-01	excluded
YDJC	61232	ES	4	3	5	-0.01	4.3E-01	excluded
SPOP	42316	ES	2	1	6	-0.02	4.4E-01	excluded
RPS3	17847	ES	4.2:4.3	3.2	5	0.00	4.4E-01	excluded
PHF21A	15538	ES	3	2	4	0.01	4.4E-01	included
ANO10	64349	ES	3:04	2	5	0.00	4.4E-01	included
ATP5G1	42239	ES	3	2	4.1	0.00	4.4E-01	included
EMCN	70089	ES	6:07:08	5	9	0.00	4.4E-01	excluded
TUBD1	42812	ES	5:06:07	3	8.1	-0.01	4.4E-01	excluded
HPSE	69788	ES	5:06	4	7	0.00	4.4E-01	excluded
CHRD	67960	ES	11	10	12	0.00	4.4E-01	included
TSEN15	9206	ES	5.1:5.2	3	6	-0.01	4.4E-01	excluded
NDRG2	26508	ES	4.5:4.6	4.1	5.2	0.01	4.4E-01	included
SCUBE2	14294	ES	17	16	18	0.00	4.4E-01	included
NONO	89419	ES	4	1	5	0.00	4.4E-01	excluded
ATG4B	58405	ES	4	3	6	0.00	4.4E-01	included
PRDX5	16639	ES	2:03	1	4	0.00	4.4E-01	included
SLC24A1	31237	ES	7:08:09	5	10	0.00	4.4E-01	excluded
CLN3	35719	ES	17	15	18	0.00	4.4E-01	excluded
PHYHD1	87801	ES	8	7	9.1	0.00	4.4E-01	included
ZNF480	51439	ES	3:04	2	5.1	0.00	4.4E-01	included
DICER1	29161	ES	29	28	30	0.00	4.4E-01	included
TTLL3	63220	ES	5:6.3:6.4:6.5:7	3	8.1	-0.01	4.4E-01	excluded
CCT7	53967	ES	3:4:5.1:5.2:7	1	8	0.02	4.4E-01	included
GALNT1	45195	ES	3	2	4.1	0.00	4.4E-01	included
IKBKG	90652	ES	9	8.2	10	0.00	4.4E-01	included
MBD1	45511	ES	18.1:18.4	17	18.5	-0.01	4.4E-01	excluded
ARIH2	64787	ES	3	2	4.2	-0.01	4.4E-01	excluded
PWWP2A	74410	ES	3	2.1	4	-0.01	4.4E-01	excluded
PLEKHB2	55371	ES	8.2:9.1:9.2	8.1	10	-0.01	4.4E-01	excluded
RIPK2	84386	ES	2	1	3	0.01	4.4E-01	included
TAF1C	37825	ES	3	2.2	4	0.00	4.4E-01	included
EEF1D	98101	ES	7.2:8.1	1	8.2	0.00	4.4E-01	included
CLN3	35720	ES	16	15	18	0.00	4.4E-01	excluded
NPIPB3	94006	ES	8.5:8.7:8.8:9:10:11:12 .1:12.2:12.3:12.4:12.5:	8.4	13.3	-0.02	4.4E-01	excluded
FOLH1	15818	ES	9	8	10	0.00	4.4E-01	excluded
PRIMPOL	71308	ES	5:06	4	7	0.00	4.4E-01	included
YAF2	21144	ES	7:08	2	9.1	0.00	4.4E-01	excluded
MAP2K4	39307	ES	3:04	1	5	0.00	4.4E-01	included
CDK17	23829	ES	2	1	3	0.00	4.4E-01	excluded
IL32	33434	ES	1.4:1.5:1.8	1.1	1.9	0.00	4.4E-01	included
SAE1	50621	ES	8:09	6	10	0.00	4.4E-01	excluded
CCDC88A	53615	ES	26:27:00	25	28	0.00	4.4E-01	included
HMGN1	60623	ES	6.1:6.2	5	9	0.00	4.4E-01	excluded
NIPSNAP3B	87105	ES	4	3	5	-0.01	4.4E-01	excluded
VPS41	79292	ES	5	4	6	0.00	4.4E-01	excluded

DCAF13	84822	ES	3	2.1	4	0.00	4.4E-01	excluded
NQO2	75157	ES	11	10	12	0.00	4.4E-01	included
FAM213B	292	ES	05:06.2	4.2	6.3	0.00	4.4E-01	excluded
ELP4	14826	ES	11	9	12	0.00	4.4E-01	included
RPP40	75237	ES	5	3	6	0.00	4.4E-01	included
POP4	48847	ES	3.1:3.2	2.1	4	0.00	4.4E-01	excluded
SEC31A	69733	ES	24	23	25.1	0.00	4.4E-01	included
RPL5	3768	ES	4	3	5	0.00	4.4E-01	excluded
IL2RG	89395	ES	6	5	7	0.00	4.4E-01	excluded
TFAP4	33641	ES	3:04:05	1	6	0.00	4.4E-01	included
ZMYND11	10590	ES	9	8	10	0.00	4.4E-01	included
ZNF254	48841	ES	4:05	3	6	0.01	4.4E-01	included
PRPF39	27398	ES	4.1:4.2:4.3	3	5	-0.02	4.4E-01	excluded
TRDMT1	10900	ES	3:04	2	5	0.02	4.4E-01	included
GCC2	54879	ES	3	2	4	0.00	4.4E-01	excluded
EYA3	1372	ES	4	3	5	0.00	4.4E-01	excluded
TRMT11	77440	ES	4	3	5	0.00	4.4E-01	included
ICA1	78793	ES	9	7	10	0.00	4.4E-01	excluded
UGP2	53752	ES	10	9	11	0.00	4.4E-01	excluded
C1orf85	8228	ES	3.1:3.2:4:5.2:6.1	2.1	6.2	0.00	4.4E-01	excluded
RPLP1	31403	ES	2	1	3	0.00	4.4E-01	included
MAPK1	61258	ES	6	5	7	0.00	4.4E-01	included
PUM1	1446	ES	13	12	14.1	0.00	4.4E-01	excluded
NPHP4	359	ES	7	6	8	0.00	4.4E-01	excluded
PLEKHA5	20659	ES	16:17:19	15	21	0.00	4.4E-01	excluded
HMGN1	60619	ES	6.2:7:8.2	5	9	0.00	4.4E-01	included
DCN	23656	ES	8:9:10:11	7	12	0.00	4.4E-01	excluded
RAB3GAP2	9870	ES	3:04	2	5	0.00	4.4E-01	included
LITAF	34024	ES	8.2	7.1	10	0.00	4.4E-01	excluded
MTX2	56123	ES	3:04:06	1	7	0.00	4.4E-01	included
VWA9	31215	ES	2.2:2.3	1.1	3.1	0.02	4.4E-01	included
C11orf54	18324	ES	02:03.1	1	3.2	0.02	4.4E-01	included
MTMR14	63118	ES	10:11	9	12.1	0.00	4.4E-01	excluded
MEGF6	314	ES	36:37.1	35	37.2	0.00	4.4E-01	included
P4HA1	12120	ES	13.1:13.2	12.1	14	0.00	4.4E-01	included
TRIP12	57854	ES	3:4.1:4.2:5	2	6	0.00	4.4E-01	excluded
DPH2	2497	ES	3.1:3.2	2.1	4	-0.01	4.4E-01	excluded
TUT1	16355	ES	3	2	4	0.00	4.4E-01	excluded
CSAD	21962	ES	8:9:10.1:10.4:10.5	7.2	11	0.01	4.4E-01	included
NAE1	36868	ES	3.1:4:6	1	7	0.00	4.4E-01	excluded
COPS3	39473	ES	3.1:3.2	1	4	0.02	4.4E-01	included
PGD	610	ES	4	3.2	5	0.00	4.4E-01	excluded
INTS4	17998	ES	3.1:3.2	2	4	0.01	4.4E-01	included
MTSS1	85111	ES	13:14.1	11	14.2	-0.01	4.4E-01	excluded
RNH1	13669	ES	5.3:6.1:6.2:7:8:9.1	5.2	9.2	0.00	4.4E-01	excluded
RACGAP1	21629	ES	07:08.1	5.4	9	0.00	4.4E-01	excluded
RSAD1	42402	ES	5	4	6	0.00	4.4E-01	included
CCAR1	11957	ES	05:06.1	3	6.2	0.00	4.4E-01	included
ARPC1B	80611	ES	2	1	3	-0.01	4.4E-01	excluded
USP16	60303	ES	2	1	3.1	0.00	4.4E-01	included
MUTYH	2609	ES	6.4:6.5	5	7	0.00	4.4E-01	included
PTPN23	64512	ES	3	2	4	0.00	4.4E-01	included
TATDN1	85097	ES	2	1.1	4.1	-0.01	4.4E-01	excluded
MANBAL	59341	ES	2.2:3:4.1	1	4.2	0.00	4.4E-01	excluded
DDX11	20977	ES	8	7.2	9	-0.01	4.4E-01	excluded
NOMO2	34244	ES	6	5	7	0.00	4.4E-01	excluded
CD300A	43254	ES	4	1	5	0.01	4.4E-01	included
KANK2	47639	ES	2.1:2.2	1	3	-0.01	4.4E-01	excluded
TC2N	28885	ES	10	9	11	0.00	4.4E-01	included
ANKRD36	54597	ES	73	72	74	-0.01	4.4E-01	excluded
STX3	16039	ES	11	10	13	0.00	4.5E-01	excluded
MKNK1	2814	ES	4	3	5	0.01	4.5E-01	included

ZNF720	94156	ES	1.2:2:3	1.1	5.1	-0.02	4.5E-01	excluded
IRF7	13713	ES	5.1	3.2	5.3	-0.01	4.5E-01	excluded
NSFL1C	58504	ES	06:07.1	4	7.2	0.00	4.5E-01	excluded
CHN2	79086	ES	14	13	15	0.00	4.5E-01	excluded
THOC5	61611	ES	14	13	15	0.00	4.5E-01	excluded
CSF1R	74060	ES	7	6	8	0.00	4.5E-01	included
RAB40B	44417	ES	2	1	3	-0.02	4.5E-01	excluded
RNF41	22404	ES	4.1:4.2	3	5	0.00	4.5E-01	included
POLM	79463	ES	6.2:6.3:7	6.1	8	0.02	4.5E-01	included
SLFN5	40292	ES	4	3	5	0.00	4.5E-01	excluded
CYB561A3	16167	ES	5.1:5.2:6.1:6.2	4.2	6.3	0.00	4.5E-01	excluded
SUB1	71658	ES	4.1:4.2	3	5	0.00	4.5E-01	included
PPIL6	77194	ES	8	6	9	0.00	4.5E-01	excluded
TSEN54	43457	ES	2	1	3	0.00	4.5E-01	included
C11orf54	18326	ES	2	1	3.1	0.01	4.5E-01	included
GTPBP10	80390	ES	6	5	7	0.00	4.5E-01	included
ITGA3	42349	ES	5	4	6	0.00	4.5E-01	included
ZNF140	25334	ES	3	2	4	-0.01	4.5E-01	excluded
CPSF3L	82	ES	10	9	11.2	0.00	4.5E-01	included
RBM42	49234	ES	3.2:4:6.2:6.3:7:8:9.1	3.1	9.2	0.00	4.5E-01	excluded
POLR3E	35552	ES	4	3	5.1	0.00	4.5E-01	included
UBE2Q2	31895	ES	5:6:7:8:9	3	10	0.00	4.5E-01	included
C12orf23	24181	ES	3.1:3.2	2.2	4.2	-0.01	4.5E-01	excluded
BYSL	76153	ES	3	2	4	0.00	4.5E-01	included
NPHP4	358	ES	12:13	11	14.2	0.00	4.5E-01	excluded
TMED10	28488	ES	4	3	5	0.00	4.5E-01	excluded
CD46	9653	ES	12:13	11	14	0.00	4.5E-01	included
KCTD7	79893	ES	2	1	3	0.00	4.5E-01	included
NPNT	70262	ES	3	2	4	-0.01	4.5E-01	excluded
C17orf62	44360	ES	4.2:4.3:5.2:6	1.1	7.1	0.00	4.5E-01	excluded
NAE1	36877	ES	2.1	1	3.1	-0.01	4.5E-01	excluded
SUN3	79620	ES	3	2	4	0.00	4.5E-01	included
ICAM2	43046	ES	1.2:3.2	1.1	3.3	-0.01	4.5E-01	excluded
PLCB3	16610	ES	03:04.1	1	4.2	0.00	4.5E-01	excluded
BRF1	29622	ES	12.2:12.3:13.2:14.2:15 .2:16:17:18:19:20.1	12.1	20.2	0.00	4.5E-01	included
PTCD3	54400	ES	2	1	3	0.00	4.5E-01	included
CBWD3	86514	ES	6	5	7	0.01	4.5E-01	included
NT5C3B	40957	ES	1.2:2	1.1	4	0.00	4.5E-01	included
ERC1	19658	ES	14	13	15	0.00	4.5E-01	included
MFSD8	70555	ES	6	5	7	0.01	4.5E-01	included
HNRNPA1	120412	ES	6.2:8:9.1	6.1	9.2	0.00	4.5E-01	excluded
ARNT	7509	ES	17.2:18:19:20:21.1:21.	17.1	22.2	0.00	4.5E-01	excluded
NPEPPS	42085	ES	6.2	5	7.1	0.00	4.5E-01	excluded
SFI1	61873	ES	5	3	6	-0.01	4.5E-01	excluded
USP28	18805	ES	21	20	22	-0.01	4.5E-01	excluded
SH3BP5	63567	ES	6:07	5.2	8	-0.01	4.5E-01	excluded
PIGG	68369	ES	3	2	4	0.00	4.5E-01	included
GCFC2	54160	ES	5	4	6	0.00	4.5E-01	included
RAD18	63079	ES	3	2	4.1	0.00	4.5E-01	excluded
IRF3	50990	ES	5.1:5.2:6.2	4	7	0.00	4.5E-01	excluded
IRF3	50998	ES	1.5:1.6:2	1.4	3	0.02	4.5E-01	included
CASP3	71297	ES	8	7	9	0.00	4.5E-01	included
RPLP0	24731	ES	5.3	4.2	6.2	0.00	4.5E-01	excluded
RDH13	52004	ES	4	3	5.1	0.00	4.5E-01	included
FZD6	84803	ES	5:6.1:6.2	3.4	6.3	0.00	4.5E-01	excluded
ACTR10	27684	ES	2	1	4	0.00	4.5E-01	included
FRMD4A	10815	ES	30	29	31	-0.01	4.5E-01	excluded
TBC1D15	23425	ES	2:03	1	5	0.00	4.5E-01	excluded
NARF	44399	ES	8	7	9	0.00	4.5E-01	included
LETMD1	21763	ES	3.2	2	7	-0.01	4.5E-01	excluded
DMKN	49189	ES	7:08:12	6.4	13	0.01	4.5E-01	included

NAP1L1	23488	ES	3:04	2	5	0.00	4.5E-01	included
PPIE	1906	ES	7.1	6	8	0.00	4.5E-01	included
CCNL1	67382	ES	7:08	6	9	-0.01	4.5E-01	excluded
COPZ1	22164	ES	9.2:10.1	9.1	10.4	0.00	4.5E-01	excluded
RBM39	59247	ES	7	6	10	0.00	4.5E-01	excluded
FAM173A	32965	ES	4.1	3	4.3	0.00	4.5E-01	included
ZNF680	79828	ES	2:03	1	5	0.01	4.5E-01	included
FAM185A	81134	ES	1.2:1.3:2	1.1	3	-0.01	4.5E-01	excluded
EZH1	41117	ES	3	2	4.1	0.00	4.5E-01	included
EVI5L	47191	ES	12	11	13	0.01	4.5E-01	included
NRF1	81752	ES	12	11	13	0.00	4.5E-01	included
KIAA1429	84565	ES	17	16	18	0.00	4.5E-01	included
ST3GAL3	2480	ES	4	3.1	6	0.01	4.5E-01	included
PARN	34079	ES	4	3.2	5.1	0.00	4.5E-01	included
NDUFA10	58241	ES	4.2:5	4.1	6.1	0.00	4.5E-01	included
NSMCE2	85127	ES	2	1	3	0.01	4.5E-01	included
MAN2A2	32516	ES	17.2	16	18	0.00	4.5E-01	included
NDUFAF5	58707	ES	9	8	10	0.01	4.5E-01	included
EIF1AX	88666	ES	2	1	3	0.00	4.5E-01	included
LGALS3BP	43933	ES	5.2:5.3:5.4	4.2	6	0.00	4.5E-01	excluded
GNB2L1	190581	ES	4.1:4.2:5:6	3	9	0.00	4.6E-01	excluded
SLC2A8	87629	ES	9	8	10	0.00	4.6E-01	included
FAM86A	33889	ES	4	3.1	6	0.01	4.6E-01	included
FXR1	67747	ES	2:05:06	1	7	-0.01	4.6E-01	excluded
KLHDC1	27437	ES	2	1	3	0.02	4.6E-01	included
IL32	33431	ES	1.4:1.5:1.6:1.8	1.1	1.9	0.00	4.6E-01	included
RASSF5	9587	ES	6	5	7	0.00	4.6E-01	excluded
YAF2	21134	ES	4:5.1:5.2:6:7:8	2	9.1	0.00	4.6E-01	included
HSD11B1L	46909	ES	02:04.1	1	4.2	-0.01	4.6E-01	excluded
DMKN	49163	ES	9	8	11	0.03	4.6E-01	included
RCBTB2	25867	ES	2:03:04	1	5	0.01	4.6E-01	included
COX20	10473	ES	2	1	3	0.00	4.6E-01	excluded
GTF2A2	30937	ES	4	3	5	0.00	4.6E-01	excluded
RAB2B	26581	ES	4:05	3	6	0.00	4.6E-01	excluded
SLC35B2	76381	ES	2:03	1	4	0.00	4.6E-01	excluded
MAD1L1	78596	ES	6	5	7	0.00	4.6E-01	included
FAIM3	9601	ES	2.1:2.2	1	3	0.01	4.6E-01	included
GALNT10	74217	ES	6	4	7	0.00	4.6E-01	excluded
NONO	89418	ES	2:04	1	5	0.00	4.6E-01	excluded
PLAT	83573	ES	07:08.1	6	8.2	0.00	4.6E-01	excluded
IMMT	54411	ES	6.3	5	7.2	0.00	4.6E-01	excluded
IFI27L1	29057	ES	7.1:7.2	6	8	-0.01	4.6E-01	excluded
ADD1	68608	ES	18.2	17	19	0.00	4.6E-01	included
MGRN1	33783	ES	11:12	10	13	0.00	4.6E-01	excluded
NACA	93187	ES	3.3:3.5	2.2	4.2	0.00	4.6E-01	excluded
EPOR	47693	ES	4.2	3	5	0.02	4.6E-01	included
PIK3C3	45320	ES	2	1	3	0.00	4.6E-01	excluded
SMC4	67481	ES	19	18	20	0.00	4.6E-01	included
ARL2BP	36538	ES	03:04.1	2	4.2	0.00	4.6E-01	included
CCDC58	66431	ES	2:03	1.1	4	0.00	4.6E-01	included
WWP1	84363	ES	4:5:6:7:8	3	9	0.00	4.6E-01	included
FAM134C	41087	ES	7	6	8	0.00	4.6E-01	included
TRPM4	50900	ES	17	16	18	0.00	4.6E-01	excluded
TBC1D31	85035	ES	17	16	18	0.01	4.6E-01	included
PLEKHG2	49824	ES	19:20.1:20.2	18	20.3	0.00	4.6E-01	included
CLIP1	24951	ES	11.2:12	11.1	13	0.00	4.6E-01	excluded
DERA	20598	ES	4.1:4.2	3	5	0.00	4.6E-01	excluded
TLDC1	37851	ES	4	3	5	0.00	4.6E-01	excluded
LTBP4	49934	ES	25:26:27	24	28	0.00	4.6E-01	excluded
SFTA3	27282	ES	3	2	4.2	-0.01	4.6E-01	excluded
DDX19A	37378	ES	5.1:5.2	4	8.1	0.00	4.6E-01	excluded
PICK1	62192	ES	5	4	6	0.00	4.6E-01	included

FLII	39593	ES	7	6	8	0.00	4.6E-01	included
STEAP3	55098	ES	8	7	9	0.00	4.6E-01	excluded
SFTA3	27262	ES	3:4.1:4.2	2	5	-0.01	4.6E-01	excluded
ZDHHC20	25453	ES	12:13.1	11	13.2	-0.02	4.6E-01	excluded
NELL2	21291	ES	19	18	20	0.00	4.6E-01	excluded
GOSR1	40120	ES	2.2:3	2.1	4	0.00	4.6E-01	included
CD46	9654	ES	12	11	14	0.00	4.6E-01	included
RPAIN	38691	ES	05:06.1	3	7	-0.01	4.6E-01	excluded
GAA	44021	ES	2.2	1	3	-0.01	4.6E-01	excluded
TANC1	55689	ES	6:07	5	8	0.00	4.6E-01	included
SEPT9	43743	ES	5	1	10.1	0.00	4.6E-01	included
CRK	38304	ES	2.2:3.1	2.1	3.2	0.00	4.6E-01	included
ISYNA1	48437	ES	02:03.2	1.3	4	0.00	4.6E-01	included
OPTN	10780	ES	04:05.1	3	5.2	-0.02	4.6E-01	excluded
TTLL1	62566	ES	10	9	11	0.00	4.6E-01	included
ARHGEF10L	864	ES	8	7	9	0.00	4.6E-01	included
ATG16L1	58037	ES	8:09	7	10	0.00	4.6E-01	excluded
NARF	44402	ES	7:08	6	9	0.02	4.6E-01	included
DOPEY1	76853	ES	39	38	40	-0.01	4.6E-01	excluded
TRAPPC4	19070	ES	2.1:2.2:2.3:3.1:3.2	1	4.1	0.00	4.6E-01	excluded
RSAD1	42401	ES	7	6	8.1	0.00	4.6E-01	excluded
MBNL2	26141	ES	9:10	8	11	-0.02	4.6E-01	excluded
GABARAPL1	20398	ES	2.10:2.12:2.13:2.14	2.6	3	0.00	4.6E-01	excluded
LRRFIP1	58139	ES	6:7:8:9:10:11:12	3	13	0.00	4.6E-01	included
NR4A1	21891	ES	5.1:5.2	4.1	6.1	0.01	4.6E-01	included
KIF13B	83254	ES	39	38	40	-0.01	4.6E-01	excluded
RPRD1B	59373	ES	2	1	3	0.00	4.6E-01	excluded
MET	81544	ES	10.2	9	11	0.00	4.6E-01	excluded
MAP2K4	39300	ES	10	9	11	0.00	4.6E-01	included
RPAIN	38693	ES	6.1	3	7	0.00	4.6E-01	included
QDPR	68854	ES	6:07	5	8.1	0.00	4.6E-01	excluded
PXN	24749	ES	13:14.1	9	14.2	0.01	4.6E-01	included
UBE3B	24310	ES	19	18	20	-0.01	4.6E-01	excluded
ZGPAT	60162	ES	5.2:6.1	5.1	6.2	-0.01	4.6E-01	excluded
SRP19	72989	ES	2	1	3	0.00	4.6E-01	excluded
SMPD4	55294	ES	11:13:14	10	15.1	-0.01	4.6E-01	excluded
NUP35	56476	ES	6.1	5	7	0.00	4.6E-01	included
HSPE1	56695	ES	2	1	3.1	0.00	4.6E-01	included
BMP1	82986	ES	19	18.2	20	0.00	4.6E-01	excluded
SLC16A4	4108	ES	3:04	2	5	0.00	4.6E-01	included
TFRC	68216	ES	3:04	2	5	0.00	4.6E-01	included
HOMER1	72631	ES	6:07:08	5	9	0.00	4.6E-01	included
DPAGT1	19113	ES	7:08	6	9.1	0.00	4.6E-01	excluded
PRSS36	36244	ES	13.2:14	13.1	15	0.01	4.6E-01	included
FKBP8	48447	ES	4	3	5	0.00	4.6E-01	included
APCDD1	44626	ES	3.2:4.1	3.1	4.2	0.00	4.6E-01	excluded
CDK10	38127	ES	3	2.1	5	0.01	4.6E-01	included
GLS2	22444	ES	4.1:4.2	3	5	-0.01	4.6E-01	excluded
FBXW9	47842	ES	4	3	5	0.00	4.6E-01	included
CCDC113	36632	ES	2:03	1	4	0.00	4.6E-01	excluded
RPLP1	31402	ES	3	1	4	0.00	4.6E-01	included
SPATS2L	56738	ES	6	4	7	0.00	4.6E-01	excluded
SSR2	8154	ES	4.2:4.3:7	4.1	8	0.00	4.6E-01	excluded
COG6	25715	ES	3	1	4.1	0.00	4.6E-01	excluded
MRI1	95017	ES	3	2	4	-0.01	4.6E-01	excluded
NQO1	37301	ES	4:05	3	6.1	0.00	4.6E-01	excluded
PORCN	88981	ES	8	6	9	0.00	4.6E-01	included
KIAA1432	85796	ES	14	13	15	0.01	4.6E-01	included
KIAA0319L	1725	ES	4	3	5	0.00	4.6E-01	included
GTF3C5	88009	ES	2.1:2.2:2.3	1	3	0.00	4.6E-01	included
ATG4A	89851	ES	7:08	6	9.1	0.00	4.6E-01	excluded
NOL8	86858	ES	6.2:6.3	5	7	0.01	4.6E-01	included

IL4R	35689	ES	4	3.2	5	0.01	4.6E-01	included
TNFRSF11A	45701	ES	7:08:09	6	10	0.00	4.6E-01	excluded
TMEM110	65288	ES	2	1	3	0.01	4.7E-01	included
ARRB1	17832	ES	14:15.1	12	15.2	0.00	4.7E-01	included
SMPD4	55301	ES	5	4.2	6	0.00	4.7E-01	included
FERMT2	27561	ES	3	2	4	0.00	4.7E-01	included
IRF3	50991	ES	5.1:6.2	4	7	0.00	4.7E-01	excluded
HAUS1	45384	ES	4:05	2.1	6	0.00	4.7E-01	included
ESRP1	98000	ES	14	13	15	-0.01	4.7E-01	excluded
EXTL3	83223	ES	3.2:4.1	3.1	4.2	0.00	4.7E-01	included
YTHDF2	1394	ES	2	1.3	3	0.00	4.7E-01	excluded
TXNRD1	24106	ES	6.2	4.2	10	0.01	4.7E-01	included
RAP1B	22946	ES	2:3.1:3.2:4:5	1.1	6	0.00	4.7E-01	included
WRAP53	39046	ES	3	2.4	4	0.00	4.7E-01	excluded
CD97	47969	ES	5	4	7	0.01	4.7E-01	included
AURKB	39141	ES	5.3:6.2	5.2	7	0.00	4.7E-01	excluded
DPYD	3839	ES	2	1	3	0.00	4.7E-01	included
SF3A1	61726	ES	3.2:4.1	3.1	4.2	0.00	4.7E-01	included
SCFD1	27064	ES	7	6	8	0.00	4.7E-01	excluded
CMAS	20725	ES	2	1	3	0.00	4.7E-01	excluded
FRG1	71415	ES	6	4	7	-0.02	4.7E-01	excluded
ARHGAP8	62629	ES	11:13	10	14	0.01	4.7E-01	included
DNM1L	21053	ES	7	6	8	0.00	4.7E-01	included
ADAM15	7917	ES	20:22.1	19	23	0.04	4.7E-01	included
TMEM68	83873	ES	3.1	2	4	0.00	4.7E-01	included
AKR1A1	2698	ES	2	1	3	0.00	4.7E-01	excluded
NT5C3A	79214	ES	4.2	3	5	0.00	4.7E-01	included
GLG1	37567	ES	5.2:6:7:8:9:10:11.1	5.1	11.2	0.00	4.7E-01	excluded
TULP3	19735	ES	9	8	10.1	0.00	4.7E-01	included
ESCO1	44771	ES	9	8	10	0.01	4.7E-01	included
NOL8	86864	ES	2.2	1.4	3	0.00	4.7E-01	excluded
GTF2H2C	72395	ES	5	4	6	0.00	4.7E-01	excluded
NDUFS1	57129	ES	3:4:5.1:6	1	7	0.00	4.7E-01	excluded
ATG16L2	17656	ES	4.3:5:6.1:6.2:6.3:6.4:7:8:9:10:11:12:13	3	14	0.01	4.7E-01	included
CTSA	59641	ES	2:03	1.4	4	0.00	4.7E-01	excluded
SEC13	63363	ES	2.2	1.1	3	0.00	4.7E-01	excluded
TMEM117	21283	ES	4	3	5	-0.01	4.7E-01	excluded
SAFB	46855	ES	3:04	2	5	0.00	4.7E-01	excluded
IQGAP1	32487	ES	2.2:3:4:5:6:7:8:9:10:11:12:13:14:15:16.1	2.1	16.2	0.00	4.7E-01	included
SNRPN	93601	ES	09:10.2	7	10.3	-0.02	4.7E-01	excluded
AGAP5	12171	ES	2	1	3	-0.01	4.7E-01	excluded
RNPS1	33258	ES	2.3	1.1	3	0.00	4.7E-01	included
UBE2Q2	31894	ES	4:5:6:7:8:9	3	10	0.00	4.7E-01	included
ADA	59483	ES	7	6	8	0.01	4.7E-01	included
PCBP4	65135	ES	4.1:4.2	2.2	5	0.01	4.7E-01	included
WDR27	101276	ES	23	22	24	-0.01	4.7E-01	excluded
MS4A4A	16082	ES	4	3	5	0.00	4.7E-01	excluded
NF1	40151	ES	31	30	32	-0.01	4.7E-01	excluded
ORAOV1	17372	ES	4.1	3	5.1	0.00	4.7E-01	excluded
PPWD1	72229	ES	3	1	5.1	0.00	4.7E-01	excluded
MAGOHB	20477	ES	2.1	1.2	3	0.00	4.7E-01	excluded
FMR1	90277	ES	10.1	9	11.1	0.00	4.7E-01	included
PCGF6	12995	ES	4:05:06	3	7	0.00	4.7E-01	excluded
STK3	84661	ES	7:10:11	6	13.1	0.00	4.7E-01	excluded
VEZT	23789	ES	2	1	4	0.00	4.7E-01	excluded
CTNNAL1	87156	ES	10:11	9	12	0.00	4.7E-01	excluded
VAMP7	90685	ES	2.2:3	2.1	4	0.00	4.7E-01	included
ZNF506	48690	ES	6	3	7.1	-0.01	4.7E-01	excluded
SIRT2	49715	ES	2	1	3.2	0.01	4.7E-01	included
ERGIC3	59178	ES	5	4	6	0.01	4.7E-01	included

NDUFB6	86067	ES	3	2	4	0.00	4.7E-01	excluded
SKA2	42752	ES	03:04.1	1.1	5	0.01	4.7E-01	included
INTS8	84576	ES	19	18	20.1	0.00	4.7E-01	excluded
SCFD1	27066	ES	5.1:5.2	4	6	0.00	4.7E-01	excluded
VRK3	51150	ES	5	3	6	0.00	4.7E-01	excluded
CHIT1	9446	ES	4	3	5	0.00	4.7E-01	included
ABCC5	67817	ES	24	23	25	-0.01	4.7E-01	excluded
TWF1	21276	ES	3	2	4	0.00	4.7E-01	excluded
LRRFIP2	63976	ES	7:8:10:11:12:13:14	5	18	0.00	4.7E-01	excluded
AGFG1	57822	ES	6	5	8	0.00	4.7E-01	included
CWF19L1	12791	ES	12	11	13	0.00	4.7E-01	excluded
FAM192A	36535	ES	1.2:3.2	1.1	4.2	0.00	4.7E-01	excluded
GMPR2	26920	ES	6.2:7.1	6.1	7.2	-0.02	4.7E-01	excluded
NDUFA3	95377	ES	03:04.1	2	5.1	0.01	4.7E-01	included
RBM42	49232	ES	3.2:4:5:6.1:6.2:6.3:7:8	3.1	9.2	0.00	4.7E-01	excluded
OS9	22703	ES	5.1:6:7.1:7.2:7.3:7.4:8	4	9.2	0.00	4.7E-01	excluded
SLC25A27	76417	ES	09:10.1	7	10.2	0.00	4.7E-01	excluded
ADPGK	31591	ES	2:03	1.1	4	0.00	4.7E-01	included
GPHN	28024	ES	11	10	12	0.00	4.7E-01	included
SMN2	72410	ES	8	7	10	0.01	4.7E-01	included
BRMS1L	27245	ES	2	1	3	0.00	4.7E-01	excluded
MAGOHB	20474	ES	4	3	6	0.00	4.7E-01	included
ARL16	44155	ES	2.3:3	2.2	5	0.00	4.7E-01	included
TMPRSS13	18953	ES	2:03	1	4	0.00	4.7E-01	excluded
ZNF793	49539	ES	6:07	5.2	8.2	0.00	4.7E-01	included
KLC1	29472	ES	14.1	13.3	14.3	0.00	4.7E-01	excluded
ARHGAP5	27130	ES	3:4.1:4.2	2	5	0.00	4.7E-01	excluded
KIAA1217	11003	ES	23	22	25.1	0.01	4.7E-01	included
TK1	43787	ES	4	3	5	0.00	4.7E-01	included
KIAA1217	11004	ES	22:23:24	21	25.1	0.00	4.7E-01	excluded
MEI1	62455	ES	30	29	31	0.01	4.7E-01	included
EPHB4	80998	ES	16	15	17	0.00	4.7E-01	excluded
RBBP4	1623	ES	5	4	6	0.01	4.7E-01	included
YAF2	21145	ES	4:5.1:5.2:6:7	2	9.1	0.00	4.7E-01	excluded
RAB7L1	9564	ES	2	1.4	3	0.00	4.7E-01	included
INPP5K	38337	ES	2	1	4	0.00	4.7E-01	excluded
DMKN	49177	ES	8:11	7	12	-0.01	4.7E-01	excluded
TATDN1	85083	ES	4.2:5:6	4.1	7	0.00	4.7E-01	excluded
MTHFD2	54030	ES	2	1	3	0.00	4.8E-01	excluded
ZMYM3	89410	ES	24	23	25	0.00	4.8E-01	excluded
NAGK	117487	ES	8	6.2	9.1	-0.01	4.8E-01	excluded
TTC8	28777	ES	7:8.1:8.2:9:11:14:15:1	2	17	0.00	4.8E-01	included
PCM1	82840	ES	25	24	27	0.01	4.8E-01	included
EXOC3	71443	ES	5:06:07	4	8	0.00	4.8E-01	excluded
BRMS1	17021	ES	9	8	10.1	0.00	4.8E-01	included
THOC7	65512	ES	3	1	4	0.00	4.8E-01	included
MEGF8	50159	ES	13	12	14	0.00	4.8E-01	excluded
EPSTI1	25762	ES	8	7	9	-0.01	4.8E-01	excluded
TNS1	57433	ES	23:24	22	25	0.00	4.8E-01	included
OTUB1	16561	ES	06:07.1	5	7.2	0.00	4.8E-01	excluded
TBRG1	19227	ES	3:04	2	6	0.01	4.8E-01	included
TCEB1	84204	ES	5:06	1.2	7	0.00	4.8E-01	included
NCAPD3	19542	ES	4	3	5	0.00	4.8E-01	included
UBR1	30205	ES	6.2:7:8:9:10:11:12:13:	6.1	15	0.00	4.8E-01	included
ALPL	977	ES	5	4	6	0.00	4.8E-01	included
C3orf17	66142	ES	4.1:4.2:5.1:6.1:6.2:7	3.2	8	0.00	4.8E-01	excluded
MARK2	16545	ES	15.2:16.1	15.1	16.2	0.00	4.8E-01	included
C1orf27	9228	ES	4	3	5	0.00	4.8E-01	excluded
CDKN1B	20513	ES	02:03.1	1	3.2	0.00	4.8E-01	included
LYRM1	34416	ES	5:06	3	7	0.00	4.8E-01	excluded
NCSTN	8466	ES	2	1	3	0.00	4.8E-01	excluded
KALRN	66527	ES	40.2	39	41	-0.01	4.8E-01	excluded

PPT2-EGFL8	75758	ES	9	8	10.1	0.01	4.8E-01	included
UBE3A	93619	ES	2:03	1	6.2	0.01	4.8E-01	included
DDX52	40541	ES	4	2	5	0.00	4.8E-01	excluded
ATG4D	47540	ES	4.1:4.2	3.2	5	-0.01	4.8E-01	excluded
OARD1	76090	ES	3.2	2.2	4.1	0.00	4.8E-01	excluded
DRAP1	16953	ES	2.2:3.1	1	3.2	0.00	4.8E-01	included
ALG5	25651	ES	8	7	9	0.00	4.8E-01	excluded
PDLIM7	74779	ES	10.1:10.2	8	11	0.00	4.8E-01	included
NVL	9947	ES	5:06:07	4	8	-0.01	4.8E-01	excluded
ATPAF2	39536	ES	6	5	7	0.00	4.8E-01	included
BTBD19	2585	ES	3:04	2	6	-0.01	4.8E-01	excluded
PIAS1	31350	ES	4:05	1	6	0.00	4.8E-01	excluded
MARS	22615	ES	4:5.1:5.2	3	6	0.00	4.8E-01	excluded
RAD17	72366	ES	3.2	2.1	5	-0.01	4.8E-01	excluded
ETV7	75973	ES	5	4	6	0.00	4.8E-01	excluded
CNIH1	27583	ES	4	3	5	0.00	4.8E-01	excluded
CA5B	98313	ES	4	3	5	0.01	4.8E-01	included
FAM3A	90642	ES	2	1.4	4	0.00	4.8E-01	excluded
SS18	44964	ES	5:07	3	9	0.00	4.8E-01	excluded
DNMT3A	52858	ES	23	22	24	0.00	4.8E-01	excluded
ARNTL	14441	ES	14.2:15	13	16	0.00	4.8E-01	excluded
UBA2	48969	ES	3:4:5:6	1	7	0.00	4.8E-01	excluded
TCERG1	73912	ES	22	21	23	0.01	4.8E-01	included
SLC4A2	82331	ES	06:07.1	4	7.2	0.00	4.8E-01	included
UPP1	97462	ES	6.1:6.2:7	4	9	0.00	4.8E-01	excluded
EXOSC5	50066	ES	2	1	3	0.00	4.8E-01	included
NCF2	9188	ES	5:06	4	7	0.00	4.8E-01	included
HSPA8	19199	ES	4.2:5.2:5.3:6.1	4.1	6.2	0.00	4.8E-01	excluded
C1orf85	8226	ES	3.1:3.2:4:5.1:5.2:6.1	2.1	6.2	0.00	4.8E-01	excluded
SLMAP	65407	ES	12:13:14	11	15.2	0.01	4.8E-01	included
MEF2B	48598	ES	12	11	13	0.00	4.8E-01	excluded
IFI44L	3553	ES	2	1	3	-0.03	4.8E-01	excluded
BAI2	1501	ES	26	25	27	-0.01	4.8E-01	excluded
ARMC8	66963	ES	7	6	8	0.00	4.8E-01	excluded
D2HGDH	58417	ES	7.1:7.3:8:9:10:11.1	6	11.2	0.03	4.8E-01	included
FAM213B	291	ES	5:6.1:6.2	4.2	6.3	0.00	4.8E-01	excluded
CNOT8	74264	ES	2	1.4	3	0.00	4.8E-01	included
CTIF	45458	ES	2	1	3	-0.02	4.8E-01	excluded
TMTC2	23565	ES	4	1	5	0.00	4.8E-01	included
AIFM1	90089	ES	2:4:5:6:7:8:9:10	1	11.4	0.00	4.8E-01	included
CTNS	38475	ES	4:05:07	3	8	0.00	4.8E-01	included
ATG3	66108	ES	4.2:5.1	4.1	5.2	0.00	4.8E-01	included
RBM23	26653	ES	4	3	5	-0.01	4.8E-01	excluded
KLHL7	78957	ES	05:06.1	1	8	0.00	4.8E-01	included
CSF2RA	88372	ES	12	11	14	0.00	4.8E-01	excluded
MVP	35963	ES	2.3:3:4:5.1:6.1:6.2:7.1	2.2	7.2	0.00	4.8E-01	excluded
NAV2	14698	ES	27	26	28	0.00	4.8E-01	included
DPF2	16817	ES	5:6:8:9:10	4	11	0.00	4.8E-01	included
TTC17	15457	ES	18	17	19	0.00	4.8E-01	included
PPP2R1B	18675	ES	10	9	11	0.00	4.8E-01	included
TOM1L1	42556	ES	3.1:3.2:3.3:4	2	6.1	0.01	4.8E-01	included
MVK	24347	ES	2.2:3:7:8.1:9.1	1	9.2	0.00	4.8E-01	excluded
PLXNB1	64646	ES	5:6:7:8:9:10:11:12:13.<br>3:14:15:16:17:18:19:2	4	23.2	0.00	4.8E-01	excluded
HIGD1B	41898	ES	3	2.2	4	-0.01	4.8E-01	excluded
POLM	79460	ES	6.2:8	6.1	9.1	0.01	4.8E-01	included
DCK	69459	ES	5	4	6	0.00	4.8E-01	excluded
C14orf80	29661	ES	9	8	10	-0.01	4.8E-01	excluded
ZNF302	48989	ES	4:5.1:5.2	2	6.2	-0.01	4.8E-01	excluded
FRYL	69225	ES	62	60	63	0.01	4.8E-01	included
SLCO2B1	17824	ES	3:4:5:6:7	1	8	0.00	4.8E-01	excluded
NIPA2	29684	ES	1.2:3	1.1	4	0.02	4.8E-01	included

GTDC1	55505	ES	12	11	13	0.00	4.8E-01	included
IDS	90294	ES	10	9	11	0.00	4.8E-01	included
CEP170	10455	ES	10	9	11	0.01	4.8E-01	included
ARFRP1	60155	ES	7	6	8.1	0.00	4.8E-01	excluded
ZBTB49	68659	ES	5:06:07	3.2	8	-0.01	4.8E-01	excluded
DPP8	31174	ES	17	16	19	0.00	4.8E-01	included
PPP1R9A	80515	ES	21	19	22	0.00	4.8E-01	excluded
SFXN2	12969	ES	8:9:10:11	7	12	0.00	4.8E-01	included
TXNDC15	73436	ES	3	1.2	4	0.00	4.8E-01	included
ARHGAP30	8541	ES	3:04	1.2	5	0.00	4.8E-01	excluded
LRTOMT	17539	ES	3	2.2	5	0.00	4.8E-01	excluded
PLEKHM1	41974	ES	6	5	7	-0.01	4.8E-01	excluded
PHF21A	15537	ES	7	6	8	0.00	4.8E-01	included
ASPN	86875	ES	6:07	5	8	0.00	4.8E-01	included
STAG2	90035	ES	7	6	8	0.00	4.8E-01	excluded
RNH1	13680	ES	3	1	4.3	-0.01	4.8E-01	excluded
STAG3	80920	ES	5:6:7:8:9	4	10	-0.01	4.8E-01	excluded
APTX	86090	ES	6.2	5.2	7.4	-0.01	4.8E-01	excluded
PAX8	55052	ES	10	7	11	0.01	4.8E-01	included
ZNF451	76584	ES	14	13	15	0.00	4.8E-01	included
OSBPL2	60055	ES	3.1:3.2	2	4	0.00	4.8E-01	excluded
PRDM2	725	ES	9:10	7	12	0.02	4.8E-01	included
PMF1	8202	ES	2:03:04	1	5	0.02	4.8E-01	included
PPP6C	87546	ES	4:05	3	6	0.00	4.9E-01	excluded
UBA7	64921	ES	17	16	18	0.00	4.9E-01	included
GTF2A1	28671	ES	4	3	5	0.00	4.9E-01	included
DEPDC5	61903	ES	25:26:00	24	27.1	-0.01	4.9E-01	excluded
FAM86C1	17438	ES	3.2:4	2	5.1	0.01	4.9E-01	included
OPA1	68145	ES	4	3	6	0.00	4.9E-01	excluded
FANCL	53655	ES	6:7.1:7.2	5	8	0.00	4.9E-01	included
ANKS3	220918	ES	09:10.1	7.2	10.2	-0.01	4.9E-01	excluded
ITGB2	60851	ES	6.1	5.2	7	0.00	4.9E-01	excluded
RNF14	73848	ES	5	4	6	0.00	4.9E-01	included
CIAPIN1	36548	ES	7:08	6	9	0.00	4.9E-01	included
ATG16L1	58039	ES	3:04:05	2	6	0.00	4.9E-01	included
SLC25A29	29268	ES	2	1	3.7	0.00	4.9E-01	excluded
RABL2A	55060	ES	7	6	8.1	0.00	4.9E-01	included
TNRC6C	43748	ES	19	18	20	0.01	4.9E-01	included
HNRNPDL	69705	ES	8	7	9	-0.01	4.9E-01	excluded
POMT1	87939	ES	5.2:6:7:8.1:8.2	4	9	0.01	4.9E-01	included
LYRM4	75242	ES	4	2	7	0.00	4.9E-01	excluded
DDX19B	37362	ES	5.2	3	6	-0.02	4.9E-01	excluded
HEXA	31547	ES	9	8	10	0.00	4.9E-01	excluded
UQCRC2	35521	ES	15	14	16	0.00	4.9E-01	included
ZNF846	47409	ES	2.1:3.2:4.2:5	1	6.1	0.00	4.9E-01	included
ERBB2	40687	ES	8.1:9:10:11:12:13	6	14	0.00	4.9E-01	included
FES	32503	ES	2.3	2.1	3	0.00	4.9E-01	excluded
XAF1	38789	ES	7:08	6	9.1	0.01	4.9E-01	included
CCNDBP1	30223	ES	2:03	1	4	0.00	4.9E-01	excluded
ATP6V0E2	82217	ES	3	2	4.1	0.00	4.9E-01	excluded
GPNMB	78968	ES	2:03	1	4.1	0.00	4.9E-01	excluded
YAF2	21142	ES	3.2:5.2:7:8	2	9.1	0.00	4.9E-01	excluded
ATG13	15595	ES	2.1:2.2	1.1	4.1	0.02	4.9E-01	included
XRRA1	17787	ES	18.1	17	18.3	0.01	4.9E-01	included
FRMD8	16852	ES	3	2.1	4	0.00	4.9E-01	excluded
TGFB1I1	36271	ES	6	5	7	0.00	4.9E-01	excluded
XKRX	89620	ES	2	1	3	0.00	4.9E-01	excluded
ACPL2	67062	ES	9:10	5	11	0.00	4.9E-01	included
PTPRE	13468	ES	9	8.2	10	0.00	4.9E-01	included
RTN4	53593	ES	5:6.1:6.2	2.5	8	0.00	4.9E-01	excluded
NME6	64629	ES	1.2:1.3:2.1:2.2	1.1	3.2	0.00	4.9E-01	excluded
PSME3	41150	ES	8:09	7	10	0.00	4.9E-01	included

ACP5	47751	ES	2.2	1	3.2	-0.02	4.9E-01	excluded
RPS2	534147	ES	5	4	6.2	-0.01	4.9E-01	excluded
FN1	57398	ES	25	24	26	0.01	4.9E-01	included
DDX17	62242	ES	11.2:12.2	11.1	12.4	0.00	4.9E-01	excluded
NKIRAS1	63723	ES	4.1:4.2	2.2	5	0.00	4.9E-01	included
SDHC	8662	ES	3:04	2	5	-0.01	4.9E-01	excluded
UPF3A	26410	ES	4	3	5	-0.01	4.9E-01	excluded
NPAS1	50616	ES	08:09.2	7	10	0.02	4.9E-01	included
PI4KB	7597	ES	2:04	1	6	0.00	4.9E-01	excluded
DCUN1D4	69267	ES	12:13	11	14	0.02	4.9E-01	included
CATSPERG	49632	ES	7	6.2	8.1	0.00	4.9E-01	included
RBMS2	22469	ES	3	1	4	0.00	4.9E-01	included
TMEM107	39126	ES	2:3.1:3.2:3.4:3.5:3.6	1	3.7	0.00	4.9E-01	included
ECHDC1	77476	ES	1.2:4	1.1	6.1	0.02	4.9E-01	included
TMEM126B	18118	ES	3:04	1	5.1	0.00	4.9E-01	excluded
PEX1	80437	ES	14	13	15	0.00	4.9E-01	excluded
POC1B	23630	ES	11	10	12	0.00	4.9E-01	included
TMBIM4	22896	ES	8	7	9.1	0.00	4.9E-01	included
PPIE	1908	ES	3:4.1:4.2:5	2	6	0.01	4.9E-01	included
SLTM	30921	ES	03:04.1	2	4.2	0.00	4.9E-01	included
RNGTT	76983	ES	12	11	13	0.00	4.9E-01	excluded
HAUS7	90436	ES	12	11.2	13	0.01	4.9E-01	included
TMEM218	19284	ES	2	1.2	4.2	-0.01	4.9E-01	excluded
SMARCE1	40877	ES	03:04.1	2.2	6	0.00	4.9E-01	excluded
ARFIP2	14138	ES	4	3	5.1	0.00	4.9E-01	excluded
ZNF268	25372	ES	9:10.1:10.2:11	7	13	0.00	4.9E-01	excluded
STARD4	72965	ES	5	3	6.1	0.01	4.9E-01	included
IDH2	32464	ES	11	10	12	0.00	4.9E-01	excluded
LSM4	48417	ES	2	1	3	0.00	4.9E-01	excluded
TRA2A	78978	ES	02:03.1	1	4	0.00	4.9E-01	excluded
PNPO	42134	ES	4	3	5	0.00	4.9E-01	included
ARL16	44151	ES	3	2.3	5	-0.01	4.9E-01	excluded
ATP2C1	66768	ES	3.2:3.3	2	4	-0.01	4.9E-01	excluded
YAF2	21201	ES	05:02.2	1	9.1	0.00	4.9E-01	excluded
DMTN	82929	ES	8	7	9	0.00	4.9E-01	excluded
FAM3A	90631	ES	5.1:5.2:5.3	4	6	0.00	4.9E-01	excluded
ELP6	64528	ES	6	5	7	0.00	4.9E-01	excluded
RGS3	87296	ES	27	22.2	28	0.00	4.9E-01	excluded
UBN1	33870	ES	18	17	19	0.00	4.9E-01	excluded
ALG11	25978	ES	2:03	1	4	-0.01	4.9E-01	excluded
SLC11A2	21735	ES	9.1:9.2	7.2	10	0.00	4.9E-01	excluded
AAMDC	17980	ES	5	4	6	0.01	4.9E-01	included
IRF3	51029	ES	1.4	1.1	2	0.00	4.9E-01	included
PPM1K	69886	ES	7	6	8	0.00	4.9E-01	included
SEMA4B	32475	ES	3	2.2	4	0.00	4.9E-01	excluded
NUDT2	86163	ES	2.2	1	3	0.00	4.9E-01	excluded
CD74	74080	ES	6:7.1:8:9:10.1	4	10.2	0.00	4.9E-01	included
B2M	30361	ES	1.2:2.2	1.1	2.3	0.00	4.9E-01	excluded
APBB3	73660	ES	9	8	10	0.00	4.9E-01	excluded
MATR3	73580	ES	20	19.2	21	0.00	4.9E-01	included
IL18	18743	ES	3	2.2	4	0.00	4.9E-01	included
ARHGAP44	39316	ES	21	20	22	0.01	4.9E-01	included
TATDN1	85093	ES	1.2:2:3	1.1	4.1	-0.01	4.9E-01	excluded
C1orf63	1144	ES	5.1:5.2	4.2	6.1	0.02	4.9E-01	included
LRTOMT	17546	ES	5	1	6.1	0.01	4.9E-01	included
CNOT8	74278	ES	1.4:1.5:2:3	1.1	5	0.00	4.9E-01	included
RFC5	24685	ES	2.1:2.2	1	3	0.01	4.9E-01	included
FDXR	43320	ES	11	10.2	12.1	0.00	4.9E-01	excluded
KANSL3	54545	ES	18	17	19.1	0.00	5.0E-01	included
ANO10	64351	ES	3	2	4	0.00	5.0E-01	included
NACA	22496	ES	3.1:3.2:3.3	2.2	4.2	0.00	5.0E-01	excluded
TRIM3	14131	ES	4	2	5.1	0.00	5.0E-01	included

LDHA	14624	ES	5	4	6.1	0.00	5.0E-01	excluded
LRTOMT	17550	ES	2.1:2.2:3	1	5	0.00	5.0E-01	included
PRPSAP2	39671	ES	6.2	5	7	0.00	5.0E-01	excluded
ALDOA	36041	ES	8	7.2	9	0.00	5.0E-01	included
TMEM184B	62227	ES	4.1	3	5	0.00	5.0E-01	excluded
CCDC90B	18080	ES	1.3:2	1.2	4.2	0.01	5.0E-01	included
CYTH1	43904	ES	4	1	5	0.00	5.0E-01	excluded
SNX1	139173	ES	4.2:5:6:7:8:9:10.2:11:<br>12:13.1:14:15	3	16.1	-0.01	5.0E-01	excluded
APOL2	62014	ES	3	2.2	4	0.00	5.0E-01	included
RFFL	40233	ES	11	10	12	0.00	5.0E-01	excluded
BCAS4	59786	ES	5	3	7	0.00	5.0E-01	excluded
IL17RC	63283	ES	1.3	1.1	2	0.00	5.0E-01	excluded
IL6ST	72083	ES	9	8	10	0.00	5.0E-01	excluded
RBM14	17079	ES	3:4.1:4.2	1	5	0.01	5.0E-01	included
C1orf85	8233	ES	2.1	1	3.1	0.01	5.0E-01	included
TMC6	43767	ES	4.2	3	5.1	0.00	5.0E-01	excluded
TMEM205	47663	ES	2.6	2.2	3	0.00	5.0E-01	excluded
GOLGA4	63984	ES	5:6:7:8:9:10:11:12:13:<br>14:15:16:17:18:19:20:	4	25	0.00	5.0E-01	excluded
SETD3	29217	ES	07:08.1	6	10	0.00	5.0E-01	included
COQ6	28348	ES	8:9:10.1:10.3:11:12:1	6	14.1	0.00	5.0E-01	included
SMURF2	43075	ES	2	1	3	0.01	5.0E-01	included
NAA25	24574	ES	2	1	3	-0.02	5.0E-01	excluded
SPCS2	17804	ES	4:5.1:5.2	3	5.3	0.00	5.0E-01	excluded
PPA2	70200	ES	6	5	7	0.00	5.0E-01	excluded
CCNB1IP1	26422	ES	6	5	8	0.01	5.0E-01	included
AHI1	77891	ES	35	34	36	0.00	5.0E-01	included
SEC24D	70446	ES	16:17.2	15	18	0.00	5.0E-01	excluded
SDHD	18735	ES	4	3.2	5	0.00	5.0E-01	excluded
THEMIS2	1350	ES	4.1:4.2:4.3	3	5	0.01	5.0E-01	included
PXMP4	59012	ES	3.2	2	4	0.00	5.0E-01	excluded
NDUFS1	57123	ES	05:01.1	3	7	0.00	5.0E-01	excluded
NBPF10	7267	ES	5:6:7:8:9	4.2	10	0.00	5.0E-01	included
PAX8	55051	ES	09:02.2	7	11	0.01	5.0E-01	included
MTMR14	63127	ES	3:5.1:5.2:6:7:8:9:10:1	1	12.1	0.00	5.0E-01	excluded
NFS1	59218	ES	8	5	9	0.00	5.0E-01	included
LINC00999	11313	ES	6	5	7	0.00	5.0E-01	excluded
TTC26	81944	ES	17	16	18	0.00	5.0E-01	included
HIVEP1	75358	ES	8	7	9	0.00	5.0E-01	included
NUP43	78103	ES	4	3	5	0.00	5.0E-01	included
ASB6	87871	ES	2	1	3	0.00	5.0E-01	excluded
PAK4	49765	ES	2.2	1	4	0.01	5.0E-01	included
NDRG2	26499	ES	7.2	5.2	8.1	0.00	5.0E-01	included
GMPPA	95768	ES	1.2:1.3:2.1	1.1	2.2	0.01	5.0E-01	included
RFC4	68062	ES	9	8	10	0.00	5.0E-01	excluded
BPTF	43117	ES	5:06	4	7	0.02	5.0E-01	included
ABI2	57020	ES	13.2	12	14	0.00	5.0E-01	excluded
SNX13	78883	ES	6	5	8	0.00	5.0E-01	included
SRSF9	24757	ES	3	2	4	0.00	5.0E-01	excluded
TNFAIP1	39886	ES	2	1	3	0.00	5.0E-01	included
TAF1C	37830	ES	3:4:5.1:5.2:6.1	2.2	7	0.00	5.0E-01	included
ATP6AP2	88830	ES	4	3	5	0.00	5.0E-01	excluded
HCFC2	24092	ES	9	8	10	0.00	5.0E-01	excluded
AP4B1	4306	ES	3:04	2	5.1	0.00	5.0E-01	excluded
FAM134C	41099	ES	3:04	2.1	5	-0.01	5.0E-01	excluded
ARRB2	38573	ES	02:04.2	1	5	0.00	5.0E-01	included
GPHN	28030	ES	6	5	8	0.00	5.0E-01	excluded
PARL	67808	ES	8	7	9	0.00	5.0E-01	included
YAF2	21139	ES	4:5.1:5.2:7:8	2	9.1	0.00	5.0E-01	excluded
SERPINB8	45739	ES	2	1.1	3	-0.01	5.0E-01	excluded
UBE2J2	65	ES	2.1	1.1	5	0.00	5.0E-01	included

HDAC9	78894	ES	11	10	12	0.01	5.0E-01	included
PNKP	51105	ES	14	13	15	0.00	5.0E-01	included
TRIM6	14054	ES	3.1:3.2:3.3:3.4	2	4.1	0.01	5.0E-01	included
FAM73B	87821	ES	8	7	9	0.00	5.0E-01	excluded
NME6	64602	ES	1.2:1.3:3.2	1.1	4	0.00	5.0E-01	excluded
CKLF	36730	ES	3.1:3.2	1	4	0.01	5.0E-01	included
CMAS	20724	ES	4	3	5	0.00	5.0E-01	excluded
OFD1	88523	ES	6	5	7	0.00	5.0E-01	included
NUP62	51125	ES	1.4:1.5	1.2	2.1	-0.01	5.0E-01	excluded
YWHAH	61910	ES	2	1	4	0.00	5.0E-01	included
CRISPLD1	84229	ES	6	5	7	0.00	5.0E-01	included
HARS2	73744	ES	6.3	6.1	7	0.00	5.0E-01	excluded
C5orf22	71633	ES	8	7.1	9	0.01	5.0E-01	included
POLR2J3	81115	ES	4.5	4.3	8	-0.01	5.0E-01	excluded
WSB2	24696	ES	4:05	3.2	6	0.00	5.0E-01	excluded
TTC23	32625	ES	2.2:2.3:3:4	2.1	6	0.01	5.0E-01	included
UBE2G1	38533	ES	2	1	3	0.00	5.0E-01	excluded
TTPAL	59467	ES	4:05	3.2	6	0.00	5.0E-01	included
CD44	15110	ES	6	5	7	-0.01	5.0E-01	excluded
CDK7	72318	ES	7	6	8	0.00	5.0E-01	included
GPR56	36587	ES	5.1:5.2	3.1	7.2	-0.01	5.0E-01	excluded
TFDP2	67091	ES	9	8	10	0.00	5.0E-01	included
ERGIC3	59176	ES	10	8	12	0.00	5.0E-01	excluded
PCNP	65953	ES	4	3	5	0.00	5.0E-01	included
MPV17	52972	ES	6.1:6.2:6.3	3.2	7	-0.01	5.0E-01	excluded
VPS45	7422	ES	14	13	15	0.00	5.0E-01	included
DCTN6	83282	ES	3:04	2	5	0.00	5.0E-01	included
SEPT9	43738	ES	10.3:16.1:17.1:17.2	10.2	17.3	0.00	5.0E-01	excluded
NCK2	54846	ES	5	4.2	6	0.00	5.0E-01	excluded
BLMH	40108	ES	3	2.1	4	0.00	5.0E-01	excluded
VCL	12255	ES	4:5:6:7:8:9:10:11:12:1<br>3:14:15:16:17:18.1	3	18.2	0.00	5.0E-01	included
TMEM176B	82259	ES	2.2:4.2	2.1	4.3	0.00	5.0E-01	included
SLCO2A1	66863	ES	9	8	10	0.00	5.0E-01	included
YIPF1	3078	ES	4	2	5	0.00	5.0E-01	excluded
IKZF2	57294	ES	13	10	14	0.00	5.0E-01	excluded
TTC26	81952	ES	3	2	4	0.00	5.0E-01	excluded
TAX1BP1	79061	ES	15:16.1:16.2:17	14	18	0.00	5.0E-01	excluded
ARHGAP22	11487	ES	11.1:11.2:12	10	13	0.00	5.0E-01	included
ZMYND8	59716	ES	19.1	18	20	0.00	5.0E-01	excluded
NDUFS1	57119	ES	5.1	4	6	0.00	5.0E-01	excluded
LIPH	68015	ES	5	4	6	0.00	5.0E-01	included
IQCA1	58091	ES	17	16	18	0.00	5.0E-01	included
PTPRF	2191	ES	12	11	13	0.00	5.0E-01	excluded
TDRKH	7668	ES	4	3	5	0.00	5.0E-01	excluded
OCLN	72381	ES	4:05	3	6	0.00	5.0E-01	excluded
ATXN3	28937	ES	4.2	3.2	5	0.00	5.0E-01	included
TFDP2	67097	ES	6:08:09	5	10	0.00	5.0E-01	excluded
THAP8	49333	ES	2	1.2	3	0.00	5.0E-01	excluded
BLOC1S5	75280	ES	3	2	4	0.00	5.0E-01	excluded
PGAP2	14009	ES	8	6	10	0.01	5.0E-01	included
ZNF383	49474	ES	5	4	7	0.00	5.0E-01	included
IL6ST	72107	ES	2	1	3.2	0.00	5.0E-01	excluded
NOX4	18239	ES	10:11:12:13:14:15:16:	8	19	0.00	5.0E-01	excluded
SUGP1	48620	ES	5.1:5.2:6.1	4	6.2	0.00	5.0E-01	included
MAVS	58611	ES	3	2	4	0.00	5.0E-01	included
AFAP1L2	13200	ES	5	4	6	-0.01	5.0E-01	excluded
RPUSD4	19370	ES	4.2:5.1	4.1	5.2	0.00	5.0E-01	included
E2F6	52687	ES	04:05.1	3	5.2	-0.01	5.0E-01	excluded
SPIDR	83779	ES	12	11	16	0.00	5.0E-01	included
ATG16L1	58035	ES	11.2:12.1	11.1	12.2	0.00	5.0E-01	excluded
TULP3	19736	ES	7	6	8	0.00	5.0E-01	excluded

SMIM8	76951	ES	1.2:2.1	1.1	3	0.00	5.1E-01	excluded
VIPAS39	28598	ES	4	3	5	0.00	5.1E-01	excluded
AFMID	43847	ES	5:06:12	2	13	0.00	5.1E-01	included
TMEM106C	21399	ES	3:4.1:4.2:5.1:6:7	2.2	8	0.00	5.1E-01	included
PDLIM5	69981	ES	5	2.2	6.1	0.00	5.1E-01	included
PLEKHA2	83473	ES	9:10:11:12:13.1	8	13.2	0.00	5.1E-01	excluded
GIT2	24371	ES	18.1:18.2:19	17.2	20	0.00	5.1E-01	included
WDR41	72577	ES	08:09.1	7	9.2	0.00	5.1E-01	excluded
DDX5	43064	ES	6.2:7:8.1	4.1	8.2	0.01	5.1E-01	included
TCEANC	88508	ES	4	3	5	0.01	5.1E-01	included
SULT1A1	35824	ES	3	2	6.2	0.00	5.1E-01	included
CCDC113	36631	ES	3	2	4	0.00	5.1E-01	excluded
C17orf62	44388	ES	3.2	1.1	4.2	0.00	5.1E-01	included
STYXL1	80155	ES	1.2:2.1	1.1	2.2	0.01	5.1E-01	included
SH3BGR	60640	ES	5	4	6	-0.01	5.1E-01	excluded
PON3	80528	ES	7:8.1:8.2	6	9	0.00	5.1E-01	excluded
NAT14	52111	ES	2.2:2.4	2.1	2.5	0.00	5.1E-01	excluded
MTDH	84631	ES	7	6	8	0.00	5.1E-01	excluded
VKORC1	36233	ES	4.2	2	6	0.00	5.1E-01	included
OS9	22702	ES	5.1:5.2:5.3:6:7.1:7.2:7.3:7.4:8:9.1	4	9.2	-0.02	5.1E-01	excluded
HDAC8	89480	ES	3.1:3.2:4.1:5:8.1:8.2:11:12:13:14.1	2	14.2	0.00	5.1E-01	excluded
FUZ	51079	ES	2.3:2.4:3	2.2	4	-0.01	5.1E-01	excluded
PACSIN2	62563	ES	4	1	6	0.00	5.1E-01	excluded
SLC43A2	38353	ES	4.1:4.2:4.3	3	5	0.00	5.1E-01	excluded
RRM2B	84773	ES	3.1:3.2	1	4	0.00	5.1E-01	excluded
ZSWIM7	94448	ES	7.2:7.3:7.5	7.1	7.7	-0.01	5.1E-01	excluded
TANK	55737	ES	13	12	14	0.00	5.1E-01	included
SLC25A23	47041	ES	14	10	15	0.01	5.1E-01	included
FIBP	16939	ES	4	3	5.1	0.00	5.1E-01	included
EIF4G3	955	ES	16.1:16.2	15	17	0.00	5.1E-01	excluded
PMP22	39344	ES	6	5.1	7	0.00	5.1E-01	excluded
DDX46	73426	ES	13	12	14	0.01	5.1E-01	included
DGUOK	54013	ES	2:04	1	7	-0.01	5.1E-01	excluded
TXNDC17	38766	ES	3	2.1	4	0.00	5.1E-01	excluded
GPR157	558	ES	3	2	4	-0.01	5.1E-01	excluded
SCLY	58200	ES	4:6:7:8:9.1	3.2	10	0.00	5.1E-01	included
PTP4A2	1530	ES	6	3	7	0.00	5.1E-01	excluded
CARKD	26261	ES	3:04:05	1	6	0.00	5.1E-01	excluded
TMEM68	83870	ES	7	6	8	0.00	5.1E-01	included
SH3GL1	46799	ES	4.2:5.1	4.1	5.2	0.00	5.1E-01	excluded
COQ2	69778	ES	5	4	6	0.00	5.1E-01	included
SLC30A5	72304	ES	2	1	3	0.00	5.1E-01	included
SCFD1	27078	ES	3	2	4	0.00	5.1E-01	included
TMED5	3779	ES	3	2	4.1	0.00	5.1E-01	excluded
ADAM10	30898	ES	9.2:10:11:12:13:14:15	9.1	16.2	0.00	5.1E-01	excluded
FAM120C	89239	ES	13:14	12	15	-0.01	5.1E-01	excluded
GNB2L1	264679	ES	4.2:5:7.2:8.1:8.2	3	9	0.00	5.1E-01	excluded
TMC8	43768	ES	2.2:3	2.1	4	0.00	5.1E-01	included
JAZF1	79071	ES	4	2	5.1	0.00	5.1E-01	excluded
NUF2	8776	ES	11	10	12	0.00	5.1E-01	excluded
FAM195B	44175	ES	4.1	3.3	4.3	0.00	5.1E-01	included
SLC4A5	54038	ES	26:28:00	25	29	0.00	5.1E-01	included
GPHN	28036	ES	2	1	3	-0.02	5.1E-01	excluded
UBALD1	33773	ES	2.3	1.2	2.5	0.00	5.1E-01	excluded
CPSF3L	88	ES	5.2:6.2:7.1:7.2:8	4	9	0.00	5.1E-01	excluded
LMBR1	82471	ES	15:16:17:18	14	19	0.00	5.1E-01	excluded
ANKRD9	29421	ES	2.1	1	2.3	0.01	5.1E-01	included
ATF2	56092	ES	3	2	4	0.00	5.1E-01	included
ADCK1	28633	ES	4:05	3	6	0.00	5.1E-01	excluded
C3orf14	65496	ES	1.2:2	1.1	3	-0.01	5.1E-01	excluded

TRIM5	14077	ES	9	7	11.3	0.01	5.1E-01	included
YEATS4	23317	ES	3:04	2	5	0.00	5.1E-01	excluded
CCNYL1	57202	ES	5	4	6	-0.01	5.1E-01	excluded
UQCC1	59105	ES	6.1:6.2	3	7.1	0.00	5.1E-01	included
MARK1	9871	ES	17	16	18	0.01	5.1E-01	included
RAB3A	48402	ES	2	1	3	0.00	5.1E-01	included
NDRG2	26495	ES	11	10	12	0.00	5.1E-01	excluded
CLASP1	55169	ES	33	32	34	-0.01	5.1E-01	excluded
SBDS	79907	ES	2.1	1	3	-0.01	5.1E-01	excluded
HAX1	7823	ES	2.2:2.3	1	3.2	-0.01	5.1E-01	excluded
IMMP1L	14820	ES	2	1	7	0.00	5.1E-01	excluded
RPS3A	70827	ES	1.4:2.2:3.1	1.3	4.1	0.00	5.1E-01	included
AMY2B	3910	ES	10	9	11	0.00	5.1E-01	included
MRPL55	10147	ES	2.2:2.8	1.1	2.9	0.01	5.1E-01	included
NDUFA7	47220	ES	3	2	4	0.00	5.1E-01	included
MMS19	12721	ES	7.1:7.2	6.2	8	0.00	5.1E-01	included
NDUFS1	57126	ES	4	3	5.1	0.00	5.1E-01	included
KANSL3	54558	ES	5.1:5.2:6:7	3.2	9	0.00	5.1E-01	excluded
MAPK8IP3	33125	ES	10	9	11	0.00	5.1E-01	included
MED12L	67297	ES	39:40:41:42	38	43	0.00	5.1E-01	included
DBNL	79434	ES	2.1:2.2:3	1.1	4.1	0.00	5.1E-01	excluded
NRCAM	81401	ES	30	29	33	0.00	5.1E-01	included
GIT2	24387	ES	17.1:17.2	15	18.2	-0.01	5.1E-01	excluded
PKN2	3701	ES	10	9	11	0.00	5.1E-01	included
TRIT1	1928	ES	2:3.1:4:6.1	1	7.1	0.01	5.1E-01	included
PXK	65438	ES	18.1	17.2	20	0.00	5.1E-01	included
C6orf89	75996	ES	6	4	7	0.01	5.1E-01	included
ACTR1A	12958	ES	3	2	4	0.00	5.1E-01	excluded
USP8	30596	ES	2	1.2	3	-0.02	5.1E-01	excluded
UPF3A	93461	ES	3:04	2	5	0.00	5.1E-01	excluded
DHRS4	26794	ES	3	2	4	0.00	5.1E-01	excluded
ACAA1	64024	ES	5	4	7	0.00	5.1E-01	excluded
GNB2L1	75085	ES	2.1:2.2:2.3:2.4:3:5:6:7	1.1	8.2	0.00	5.1E-01	excluded
RBM39	59239	ES	11:12.1	10	12.2	0.00	5.1E-01	excluded
ALAS1	65183	ES	02:03.1	1	3.2	-0.01	5.1E-01	excluded
CCDC174	63531	ES	8:09	7	10	0.00	5.2E-01	included
TG	85207	ES	36	35	37	0.00	5.2E-01	excluded
TGFBR1	87051	ES	8	7	9	0.00	5.2E-01	excluded
FN3KRP	44419	ES	2	1	3	0.00	5.2E-01	excluded
SYNCRIP	76935	ES	2	1	3	0.00	5.2E-01	included
YAF2	21138	ES	5.2:6:7:8	2	9.1	0.00	5.2E-01	included
PSMG1	60601	ES	4	3	5	0.00	5.2E-01	excluded
MCAM	19137	ES	14	13.3	15.1	0.00	5.2E-01	excluded
MEAF6	1799	ES	7:8.1:9.1	5	9.2	0.00	5.2E-01	included
ARL13B	65698	ES	2	1	4	-0.01	5.2E-01	excluded
IP6K2	64773	ES	8.1:8.2	7	11.2	0.00	5.2E-01	included
CASP8	56821	ES	10:11	8	13	0.00	5.2E-01	excluded
POGK	8824	ES	4	3	5	0.00	5.2E-01	included
PFDN5	22004	ES	2:05	1	6.2	0.01	5.2E-01	included
SLC25A23	47043	ES	9	8	10	0.00	5.2E-01	excluded
GYS1	50846	ES	2	1.2	3	0.00	5.2E-01	excluded
LRRC28	32652	ES	5:6:8:10:11	3	12	0.01	5.2E-01	included
CLASP2	63879	ES	27	26	28	0.00	5.2E-01	excluded
MTPN	81895	ES	2:03	1	4	0.00	5.2E-01	included
ADIPOR1	9429	ES	07:08.1	6	8.2	0.00	5.2E-01	excluded
LARS2	64399	ES	4	3	5	0.00	5.2E-01	excluded
DPM1	59794	ES	6	5	7	0.00	5.2E-01	excluded
HSPA13	60212	ES	3	2	4	0.00	5.2E-01	excluded
RPS6KB2	17205	ES	5.1:6.1:6.2:7	4	8	0.00	5.2E-01	included
ZNF426	47357	ES	3	2.2	4	-0.01	5.2E-01	excluded
SMUG1	22133	ES	1.2:2.2:2.3:3	1.1	4.1	-0.01	5.2E-01	excluded
POC1B	23639	ES	3.1	2.2	4	-0.01	5.2E-01	excluded

RPAP1	30097	ES	22.1:22.2:23.1	21	23.2	0.00	5.2E-01	included
ARFIP2	14136	ES	5.2:6	5.1	7	0.00	5.2E-01	included
TBC1D3F	40553	ES	10:11.1	9	12	0.00	5.2E-01	excluded
FAM3B	60656	ES	3.2	1	4	0.00	5.2E-01	excluded
RPN1	66652	ES	4	3	5	0.00	5.2E-01	excluded
MLLT4	78451	ES	32.2:33.1	31	33.2	0.00	5.2E-01	excluded
H2AFV	79576	ES	2	1	3	0.00	5.2E-01	included
BAD	16615	ES	2	1.3	3	0.00	5.2E-01	excluded
FAM21C	11378	ES	11	10	12	-0.01	5.2E-01	excluded
ZNF131	71928	ES	7.1:7.2:7.3:7.4	6	8	0.00	5.2E-01	excluded
GOT2	36690	ES	2	1	3.1	0.00	5.2E-01	excluded
NAE1	36874	ES	2.1:3.1	1	4	0.01	5.2E-01	included
ZNF714	48752	ES	6.2	4.2	7	-0.01	5.2E-01	excluded
RAD51	30023	ES	06:07.1	3	7.2	0.00	5.2E-01	excluded
FUZ	51090	ES	2.1:2.2	1.2	3	0.01	5.2E-01	included
PLAUR	50233	ES	7	6	8	0.00	5.2E-01	excluded
DUSP22	75134	ES	3	2	5	0.00	5.2E-01	excluded
TMEM234	1576	ES	2:03:04	1	5.1	0.00	5.2E-01	excluded
BNIP3L	83129	ES	4:05	1	6	0.00	5.2E-01	included
TLK2	42906	ES	6	4.2	7	0.00	5.2E-01	excluded
RNF170	83745	ES	5	4	6	0.00	5.2E-01	included
DMTF1	80295	ES	17.2:17.3	16	18	0.00	5.2E-01	excluded
STARD5	32208	ES	2	1.1	3	0.01	5.2E-01	included
FDXR	43318	ES	11:12.1	10.2	12.2	0.00	5.2E-01	excluded
HSPA12B	58595	ES	3:04	2	5	0.00	5.2E-01	excluded
NUP54	69592	ES	2:03	1	5	0.00	5.2E-01	excluded
PAQR4	33338	ES	2.2:2.3	1.2	3	0.00	5.2E-01	excluded
LAS1L	89321	ES	9	8	10	0.01	5.2E-01	included
IP6K1	64916	ES	1.2:2	1.1	3	0.00	5.2E-01	included
SMAGP	21835	ES	4	3.3	6.1	0.00	5.2E-01	included
RUFY2	11942	ES	2	1.1	3	0.00	5.2E-01	included
TUSC3	82773	ES	10	9	12	0.00	5.2E-01	excluded
RBMX	90224	ES	4:05	3.2	6	0.00	5.2E-01	excluded
CCNL2	162	ES	6.1	5	7.2	0.00	5.2E-01	included
PSME1	534013	ES	1.2:2	1.1	3	0.00	5.2E-01	excluded
HMCES	96506	ES	4	3	6	0.01	5.2E-01	included
LRRC28	32677	ES	3:5:6:7.1:7.2:8:10:11	2	12	0.00	5.2E-01	included
GLRB	70969	ES	6:7:8:9:10:11:12	3	13	0.00	5.2E-01	excluded
DHFR	72658	ES	2	1	3	0.00	5.2E-01	included
RBM38	59898	ES	4	3	6	0.00	5.2E-01	included
CPSF3L	86	ES	5.2:6.1:6.2:7.1:7.2:8	4	9	0.00	5.2E-01	included
MRPL2	76237	ES	6	5	7	0.00	5.2E-01	excluded
TTYH3	78632	ES	5.2	4	6	0.00	5.2E-01	included
AKT2	49874	ES	11	10	12	0.00	5.2E-01	included
RAPGEF6	73231	ES	31	30	32	0.00	5.2E-01	included
FAM60A	20987	ES	4:05:06	1	7	0.00	5.2E-01	excluded
PPARD	75910	ES	4.2:5	4.1	6	0.00	5.2E-01	excluded
ASAH1	82846	ES	6	5	7	0.00	5.2E-01	excluded
UBXN11	1254	ES	6	5	7	-0.01	5.2E-01	excluded
EXOC6	12546	ES	12:13:14	11	15	0.00	5.2E-01	excluded
TINAGL1	1478	ES	5.1	3	5.3	0.00	5.2E-01	excluded
ZNF207	40207	ES	3.3:4:5.1	3.2	5.2	0.00	5.2E-01	included
GNB2L1	75064	ES	3	2.4	5	0.00	5.2E-01	included
SLC19A2	8922	ES	2	1	3	0.00	5.2E-01	included
MTHFSD	37925	ES	3	2.2	5	0.00	5.2E-01	included
CSNK2A1	58462	ES	3	1	4	0.00	5.2E-01	excluded
ZNF148	66553	ES	3.2:5:6:7	3.1	8	0.00	5.2E-01	excluded
PLAU	12253	ES	4	3	5	0.00	5.3E-01	included
BTN3A3	75664	ES	7	6	8	0.01	5.3E-01	included
SUOX	22344	ES	2	1	5.2	0.01	5.3E-01	included
ARMC8	66970	ES	2.1:2.2	1	3	0.01	5.3E-01	included
TRIP12	57855	ES	4.1:4.2:5	2	6	0.00	5.3E-01	excluded

SZRD1	811	ES	3	1	4.2	-0.01	5.3E-01	excluded
IMMT	54407	ES	6.2:6.3:7.1	5	7.2	0.00	5.3E-01	excluded
CLNS1A	17961	ES	3	2	4	0.00	5.3E-01	excluded
RAD50	73290	ES	11	10	12	0.00	5.3E-01	included
TRIP10	47084	ES	11.2	10	12.2	0.00	5.3E-01	included
LGALS3BP	234087	ES	2.3:2.4:3.1:3.2	2.2	4.1	-0.01	5.3E-01	excluded
HINT2	546794	ES	3	2	4	0.00	5.3E-01	excluded
BSG	46305	ES	4	1	5	-0.01	5.3E-01	excluded
BACE1	18909	ES	7	6	8	0.00	5.3E-01	excluded
TMCO3	26381	ES	8:9:10:11:12:13.1	7	13.2	0.00	5.3E-01	excluded
SNX14	76926	ES	14:15:17:18:19:20:21	13	22	0.00	5.3E-01	excluded
PPP1R13B	29520	ES	17	16	18	0.00	5.3E-01	included
TCEANC	88509	ES	3:04	2	5	-0.01	5.3E-01	excluded
ATP5J	60262	ES	2	1.5	3	0.00	5.3E-01	included
PEX1	80438	ES	12	11	13	0.00	5.3E-01	excluded
DLST	28437	ES	6	5.2	7.1	0.00	5.3E-01	included
FXYD6	18945	ES	4	2	6	0.00	5.3E-01	included
OGDH	79552	ES	3:04	2	7	0.00	5.3E-01	included
HOOK2	47866	ES	18	17	19	0.00	5.3E-01	excluded
ZNF506	48687	ES	7.1:7.2:7.3	3	8	0.01	5.3E-01	included
RNF180	72197	ES	4	3	5.1	0.00	5.3E-01	included
COPS7B	57944	ES	9.2	8	10	0.00	5.3E-01	excluded
GSTM1	4067	ES	6:07	5	8	0.00	5.3E-01	included
ITSN1	60471	ES	27:28:00	26	29	-0.01	5.3E-01	excluded
NOL8	86849	ES	10	9	11	0.00	5.3E-01	excluded
POLR1C	76303	ES	7	6	8	0.00	5.3E-01	excluded
ZNF548	52245	ES	2:03	1	4	-0.01	5.3E-01	excluded
SETD8	25072	ES	2	1	3	0.00	5.3E-01	excluded
COA4	17737	ES	2.2	1	4.2	0.00	5.3E-01	included
MRVI1	14367	ES	12	11.3	13	0.00	5.3E-01	included
IPO5	26160	ES	7	6.2	8	0.00	5.3E-01	included
CAPN10	58270	ES	12	11	13	0.00	5.3E-01	included
QDPR	68859	ES	2	1	3	0.00	5.3E-01	included
CHD1L	7378	ES	2:3:4:5	1	6	0.00	5.3E-01	included
MTERFD1	84620	ES	8	7	9	0.00	5.3E-01	excluded
EHF	14962	ES	10	9	11	0.01	5.3E-01	included
BID	61006	ES	4:05	2	7	0.00	5.3E-01	included
FIG4	77213	ES	08:09.1	6	10	0.00	5.3E-01	excluded
DUS4L	81345	ES	5.1	4	6	0.01	5.3E-01	included
RRP12	12699	ES	04:05.2	3	6	0.00	5.3E-01	excluded
NREP	72978	ES	10	9	11	0.00	5.3E-01	excluded
MFSD11	43678	ES	6:07	5.3	8	0.00	5.3E-01	included
POC1B	23631	ES	7:08	6	9	0.00	5.3E-01	excluded
RPAIN	38695	ES	5	3	7	0.00	5.3E-01	excluded
CPSF3L	85	ES	5.1:5.2:6.1:6.2:7.1:7.2:	4	9	0.00	5.3E-01	excluded
CAMK2B	79496	ES	14.1:14.2:15:16:17:18	12	21.1	0.02	5.3E-01	included
FAM3A	90640	ES	2:3.1:3.2	1.4	4	-0.02	5.3E-01	excluded
DCTD	96837	ES	2.1:2.2	1.2	5	0.00	5.3E-01	included
KRAS	20820	ES	6	5	7	-0.01	5.3E-01	excluded
ADAM15	7932	ES	2	1	3.2	0.00	5.3E-01	included
PIGA	88556	ES	2.1:2.2	1	3	0.00	5.3E-01	excluded
REEP5	72993	ES	5	3	6	0.00	5.3E-01	excluded
GSS	59054	ES	5.1:5.2:5.3:6	4	7	0.00	5.3E-01	included
TRPC4AP	59061	ES	3	2	4	0.00	5.3E-01	excluded
MTMR12	71647	ES	15:16	14	17	0.00	5.3E-01	excluded
NFIC	46682	ES	4	3	5	0.00	5.3E-01	excluded
DAZAP2	21817	ES	4.1:4.2:4.3:4.4:4.5:4.6	3	5	0.00	5.3E-01	excluded
NACA	22493	ES	3.1:3.3:3.4:3.5	2.2	4.2	0.00	5.3E-01	excluded
ZNF180	50329	ES	7	6	8	0.00	5.3E-01	included
ZNF655	80685	ES	4:5.3:6.1:6.2	3.2	7	-0.01	5.3E-01	excluded
FDPS	8059	ES	02:03.1	1.2	3.2	-0.02	5.3E-01	excluded
PDCD1LG2	85791	ES	4	3	5	0.00	5.3E-01	included

LDHA	14623	ES	6.1:6.2	4	7	0.00	5.3E-01	excluded
SEC24D	70449	ES	7	6	8	0.00	5.3E-01	excluded
CCT3	8238	ES	4	1	5	0.00	5.3E-01	excluded
IFNAR2	60393	ES	7	6	8	0.00	5.3E-01	excluded
NGFRAP1	89733	ES	2.2	1	3.2	0.00	5.3E-01	excluded
GRIK5	50114	ES	4	3	5	0.00	5.3E-01	excluded
ARMC6	48580	ES	2.2:3	1.1	5	0.00	5.3E-01	included
CDK5RAP2	87383	ES	25	24	26	0.00	5.3E-01	excluded
OSCP1	1779	ES	11.2:12:13.1	11.1	13.2	0.00	5.3E-01	excluded
TNFRSF25	412	ES	6.3:6.4:6.5:7.1	6.2	7.2	0.00	5.3E-01	included
LTBR	19852	ES	08:09.1	7	10	0.01	5.3E-01	included
PAOX	13559	ES	2	1	3	0.00	5.3E-01	excluded
EXOSC1	12702	ES	6	5	7	0.00	5.3E-01	included
RPL26	39177	ES	3	2.2	4.1	0.00	5.3E-01	included
PABPC4	1895	ES	11	10.1	12	0.00	5.3E-01	excluded
BTN2A2	75645	ES	3.1:3.2:4	2	5	-0.01	5.3E-01	excluded
YME1L1	11059	ES	4	3.3	5	0.00	5.3E-01	excluded
RABL2B	62923	ES	2.1:2.2:2.3	1	3.1	-0.01	5.3E-01	excluded
RAD51C	42715	ES	7	6	8	0.00	5.3E-01	included
SLC22A17	26736	ES	3	2.2	4	0.00	5.3E-01	excluded
FAM21C	11379	ES	3	2	4	0.00	5.3E-01	included
RAB34	39957	ES	9.2:10:11.1	9.1	11.2	0.00	5.3E-01	excluded
USP3	31040	ES	3.2:4:5:7:8:9:10:11	1	12	0.01	5.3E-01	included
NUP153	75453	ES	12	11	13	0.00	5.3E-01	included
TSC22D1	25784	ES	1.2:3:4.1	1.1	4.2	0.00	5.3E-01	included
ALDH4A1	871	ES	13	12	14	-0.01	5.3E-01	excluded
TSC2	33198	ES	5	4	6	0.00	5.3E-01	included
PIGG	68364	ES	5	4	6	0.00	5.3E-01	excluded
TM7SF3	20860	ES	2	1	3	0.00	5.3E-01	excluded
LDLRAD3	15420	ES	2	1	3	0.00	5.3E-01	excluded
CASP10	56809	ES	7:08	5	9	0.00	5.3E-01	excluded
HTRA2	54104	ES	3	2	4	0.00	5.3E-01	included
ATG4D	47537	ES	4.1:4.2:5	3.2	6	-0.01	5.3E-01	excluded
CDC42BPA	10050	ES	13	12	14	-0.01	5.3E-01	excluded
TXNL4A	46281	ES	07:02.2	3	9	0.01	5.3E-01	included
UBE2Q2	31891	ES	11:12:13	10	14	0.00	5.3E-01	included
FAT1	71409	ES	27	26	28	0.00	5.3E-01	included
FAM13A	69914	ES	10:11	9	12	0.00	5.3E-01	excluded
MOCS1	76075	ES	3.3	1	4	0.00	5.3E-01	excluded
TCEB1	84214	ES	1.2:4	1.1	6	0.00	5.4E-01	included
GRB2	43440	ES	6	5	7	0.00	5.4E-01	included
TOP3A	39608	ES	12	11	13	0.00	5.4E-01	included
SEC24D	70451	ES	6	5	8	0.00	5.4E-01	excluded
CDK5RAP1	58981	ES	12	11	13	0.00	5.4E-01	included
FBL	49849	ES	4	3	5	0.00	5.4E-01	included
PMPCB	81178	ES	10:11:12.1:12.2	9	12.3	0.00	5.4E-01	included
ASCC1	12085	ES	7	6	8	0.00	5.4E-01	included
MFSD2A	1980	ES	02:03.2	1	4	0.00	5.4E-01	included
PTK7	76250	ES	9:10:11	8.1	12	0.00	5.4E-01	included
FAM219B	31795	ES	3.1:3.2:4	2	5.1	0.00	5.4E-01	included
ZNF85	48736	ES	3.1:3.2	2	6	0.00	5.4E-01	included
RPS3A	70824	ES	1.4:2.2:3.1:3.2	1.3	4.1	0.01	5.4E-01	included
SH3TC1	68760	ES	4.1:4.2	2	5	0.00	5.4E-01	included
RPS6KB2	17204	ES	6.2	5.1	7	0.00	5.4E-01	excluded
LGALS3BP	234086	ES	2.4:2.5:3.1:3.2	2.2	4.1	0.00	5.4E-01	included
UBTF	41833	ES	11	10	12	0.00	5.4E-01	excluded
UXT	88940	ES	3	2	4	0.00	5.4E-01	excluded
VPS26A	11981	ES	5	3	6	0.00	5.4E-01	excluded
BAI2	1504	ES	7	6.2	8	0.00	5.4E-01	excluded
PCNP	65961	ES	2.3	1	3	0.00	5.4E-01	included
VPS53	38225	ES	5:6:7:9:10:11:12	4	13	0.00	5.4E-01	included
KIF2A	72178	ES	19	18	20	0.00	5.4E-01	excluded

APAF1	23872	ES	25	24	26	0.00	5.4E-01	excluded
ALKBH1	28615	ES	3	2	4	0.00	5.4E-01	excluded
FOPNL	34201	ES	4	2	5	0.00	5.4E-01	included
GPR89A	7316	ES	4	1	5	0.00	5.4E-01	excluded
SCAMP2	31771	ES	3:4:5:6:7.1:7.2:8.1	2	8.2	0.00	5.4E-01	excluded
CUL3	57778	ES	4	1	5	0.00	5.4E-01	excluded
DECR1	84404	ES	03:04.1	1	4.2	-0.02	5.4E-01	excluded
STX10	47927	ES	3.3:4:5.1	3.2	5.2	0.00	5.4E-01	included
POC5	72543	ES	6	5	7	0.00	5.4E-01	included
HAT1	55962	ES	4	3	5	0.00	5.4E-01	included
TFDP1	26391	ES	13.2	12	14	0.01	5.4E-01	included
GTF2H2C	72398	ES	1.2:2	1.1	3	-0.01	5.4E-01	excluded
DDX19B	37351	ES	4:5.2:6:7	3	9	0.00	5.4E-01	included
RPGR	88807	ES	14.1:14.2:14.3	13	16	0.02	5.4E-01	included
IFT122	66729	ES	6	5	7	-0.01	5.4E-01	excluded
DTX2	80180	ES	2	1	4	0.01	5.4E-01	included
PDE9A	60731	ES	3	2	5	0.00	5.4E-01	included
UPP1	79639	ES	5:6.1:6.2:7	4	9	0.01	5.4E-01	included
PBK	83190	ES	4:05	3	6	0.00	5.4E-01	included
RAB34	39956	ES	9.2:9.3:10:11.1	9.1	11.2	0.00	5.4E-01	excluded
POFUT2	60873	ES	8.3	8.1	8.5	-0.01	5.4E-01	excluded
WDR86	82375	ES	5.1:5.2	4	6	-0.01	5.4E-01	excluded
RNF121	17454	ES	4:6.1:6.2:7:8	3	9	0.00	5.4E-01	included
ACAT1	18602	ES	5	4	7	0.00	5.4E-01	excluded
ALDH3A2	39760	ES	2	1.5	3	0.00	5.4E-01	included
ARID2	21311	ES	21	20.1	22	0.00	5.4E-01	excluded
DUOXA1	30395	ES	1.2:1.3:2.2:3:4	1.1	5	-0.01	5.4E-01	excluded
KDSR	45710	ES	6	5	7	0.00	5.4E-01	included
RBM6	64945	ES	3.2:4:5:7	2	8	0.00	5.4E-01	included
MFSD8	70553	ES	7	6	8	0.01	5.4E-01	included
NDRG2	26500	ES	6	5.2	7.2	0.01	5.4E-01	included
TRIM37	42729	ES	2	1	3	0.00	5.4E-01	included
OGFOD2	25013	ES	6.3:8.1	6.2	8.3	0.00	5.4E-01	included
SZRD1	806	ES	3	1	4.1	-0.01	5.4E-01	excluded
HSF4	36943	ES	14.1	13	14.3	0.00	5.4E-01	excluded
PPAP2A	72035	ES	3	2	4	0.01	5.4E-01	included
COPE	48514	ES	8	7	9	0.00	5.4E-01	included
MDM2	23017	ES	5.1:5.2:6:7:8.1:8.2:9	3	10	0.00	5.4E-01	excluded
MARC2	9875	ES	6:07:08	5	9	0.00	5.4E-01	excluded
COQ7	34288	ES	4.1	3	5	0.00	5.4E-01	included
ERMP1	85802	ES	14	13	15	0.00	5.4E-01	excluded
RPS23	72694	ES	3.2:3.4:3.5	3.1	3.6	0.00	5.4E-01	excluded
TATDN3	9758	ES	08:09.2	7	9.3	0.00	5.4E-01	included
TPM1	31006	ES	2.1:2.2	1	3.1	0.00	5.4E-01	included
TNFSF13	38976	ES	2.2:3	1	4.1	0.01	5.4E-01	included
TAF2	85011	ES	3	2	4	0.00	5.4E-01	included
RAP1B	22944	ES	1.2:3.1	1.1	3.2	0.00	5.4E-01	excluded
ACSL1	71326	ES	6	2	7	0.00	5.4E-01	included
ARHGAP5	27131	ES	03:04.2	2	5	-0.01	5.4E-01	excluded
ETV1	78833	ES	17	16	18	0.00	5.4E-01	excluded
CALML4	31351	ES	3	2	4	0.01	5.4E-01	included
GSR	83298	ES	9	8	10	0.00	5.4E-01	included
HNRNPK	86715	ES	7	6	8	0.00	5.4E-01	included
RABL6	88219	ES	13.2:14.1:14.2:15:16.2<br>:17:18.1:19.1	13.1	19.2	0.00	5.4E-01	included
BBS5	55866	ES	8	7	9	0.01	5.4E-01	included
EPG5	45359	ES	36:37:00	35	38	0.00	5.4E-01	included
SEMA4F	54130	ES	8	7	9	0.00	5.4E-01	included
DUOX1	30406	ES	2	1	3	-0.02	5.4E-01	excluded
CARD16	18545	ES	4	3	5	0.01	5.4E-01	included
TAPBPL	19859	ES	2	1	3	0.00	5.4E-01	excluded
NPC1	44837	ES	10:11	9	12	0.00	5.4E-01	included

GIGYF2	58025	ES	3.2	1	5	-0.01	5.4E-01	excluded
USB1	36627	ES	7	4	8	0.00	5.4E-01	excluded
DROSHA	71627	ES	7	6	8	-0.01	5.4E-01	excluded
MRPL55	10105	ES	2.2	1.2	2.5	-0.01	5.4E-01	excluded
ATG13	15587	ES	3:4.1:4.2	1.1	5	0.00	5.4E-01	included
PCBP2	213888	ES	6	5	7	0.00	5.4E-01	excluded
CCBL2	3704	ES	2	1	3	-0.01	5.4E-01	excluded
RAB7A	66663	ES	3.2:5.1	3.1	5.2	0.00	5.4E-01	excluded
COPS4	69764	ES	11	10	12	0.00	5.4E-01	included
FAM3A	90633	ES	5.3	4	6	0.00	5.4E-01	excluded
B3GNTL1	44430	ES	8	6	9	0.00	5.4E-01	included
EYA2	59705	ES	13:14	12	15	0.00	5.4E-01	included
SNX1	139189	ES	7:8:9:10.2:11:12:14:1	3	16.1	0.00	5.4E-01	excluded
ZNF33A	11311	ES	2	1	3	0.00	5.4E-01	included
INPP5K	38324	ES	2:4:5.1:5.2	1	7	-0.01	5.4E-01	excluded
NCBP1	87020	ES	6	5	7	0.00	5.4E-01	included
INO80E	36014	ES	6.3:10	5	11	0.00	5.5E-01	excluded
SLC25A36	67055	ES	3	2.2	4.1	0.00	5.5E-01	excluded
PMS1	56546	ES	11.1:11.2	10	12	0.00	5.5E-01	included
SMN2	72412	ES	4	3	5	0.00	5.5E-01	excluded
NOP2	19894	ES	3.2:4.1	3.1	4.2	0.00	5.5E-01	excluded
SLC25A32	84813	ES	4	3	5	0.00	5.5E-01	included
OSBPL3	79026	ES	12	11	13	0.01	5.5E-01	included
INO80	30067	ES	27	26	28	0.00	5.5E-01	excluded
WIPI1	43139	ES	2	1	3	0.00	5.5E-01	included
ST8SIA4	72885	ES	2	1	3	0.00	5.5E-01	excluded
CD44	15001	ES	12.1:13:14:15	5	16.1	0.00	5.5E-01	included
C17orf62	44346	ES	5.2	4.3	6	0.00	5.5E-01	included
KDM1B	75462	ES	14:15	13	16	0.00	5.5E-01	excluded
MGRN1	33784	ES	11	10	13	0.00	5.5E-01	excluded
TRMT13	3871	ES	5	4	6.1	0.01	5.5E-01	included
HMGCR	72515	ES	14	13	15	0.00	5.5E-01	excluded
PTPN6	20036	ES	3	1	4.2	0.00	5.5E-01	included
LOH12CR1	20507	ES	3	2	4	0.00	5.5E-01	included
PCGF2	40588	ES	3.2:5	2.2	6	0.00	5.5E-01	included
PDGFC	70958	ES	3	1	4.1	0.00	5.5E-01	included
NAE1	36875	ES	3.1	1	4	0.00	5.5E-01	included
NOP14	68621	ES	16.3:17:18.1:18.2	16.2	18.3	0.00	5.5E-01	included
OLA1	56018	ES	9:10:11	8	12	0.00	5.5E-01	excluded
WDR19	69052	ES	5	4	6	0.01	5.5E-01	included
NEK1	71149	ES	15	14	16	0.00	5.5E-01	excluded
EFTUD2	41903	ES	2	1.1	3	0.00	5.5E-01	excluded
ATE1	13322	ES	7	6	8	0.00	5.5E-01	excluded
CYFIP1	29678	ES	5	4	6	0.00	5.5E-01	excluded
STAU2	84158	ES	19	18.1	20	0.01	5.5E-01	included
GRPEL2	74030	ES	3.1	2	4	0.00	5.5E-01	included
ASL	79866	ES	13	12	14	0.00	5.5E-01	included
STAU1	59738	ES	4	3	5	0.00	5.5E-01	included
DHX38	37546	ES	2.2:3:4:5:6:7:8:9:10:1 1:12:13:14:15:16:17.1	2.1	17.2	0.00	5.5E-01	included
RPN2	59334	ES	3	2	4	0.00	5.5E-01	excluded
PTPN18	55344	ES	3:4:5:6	1	7	0.01	5.5E-01	included
ZBTB38	67069	ES	4	2	5	-0.01	5.5E-01	excluded
ZBTB25	27885	ES	7.1:7.2	6	8	-0.01	5.5E-01	excluded
FAM120B	78493	ES	4:05	3	6	0.00	5.5E-01	included
UBE2W	84194	ES	4	3	5	0.00	5.5E-01	included
TMEM143	50739	ES	3.1:3.2:5	2	6	0.01	5.5E-01	included
TAZ	90586	ES	7	6	8.1	-0.01	5.5E-01	excluded
ITGB1	11189	ES	17	16	19	0.00	5.5E-01	included
PCBP4	65129	ES	9.3:10:11	9.1	12	0.00	5.5E-01	included
CDC42BPA	10046	ES	24	21	25	0.00	5.5E-01	included
UBXN11	1251	ES	10	9	11	0.00	5.5E-01	excluded

NT5C3B	40956	ES	2:03	1.1	4	0.00	5.5E-01	excluded
SFTA3	121940	ES	2:4.1:4.2:4.3:5	1.3	6	0.01	5.5E-01	included
MTMR14	63110	ES	20	19	21	0.00	5.5E-01	included
GIPC1	47983	ES	3	1	4	0.00	5.5E-01	included
PYROXD2	100771	ES	13	12	14	-0.01	5.5E-01	excluded
OS9	22713	ES	5.1:5.2:7.1:7.4:8:9.1	4	9.2	0.00	5.5E-01	excluded
OAZ2	31129	ES	5	4	6	0.00	5.5E-01	excluded
ZNF846	47407	ES	2.1:3.2:4.1:4.2:5	1	6.1	0.00	5.5E-01	included
TXN2	96153	ES	4:05	3.2	6	-0.01	5.5E-01	excluded
SLC9B2	70176	ES	9	8	10	-0.01	5.5E-01	excluded
CCNC	77082	ES	3	2	4	0.00	5.5E-01	included
MTMR4	42686	ES	8	6.1	9	0.00	5.5E-01	excluded
SLC15A3	16132	ES	5	4	6	0.00	5.5E-01	excluded
TAB3	88760	ES	9.5	9.1	11	0.01	5.5E-01	included
FBXO16	83218	ES	3	2.2	5	-0.01	5.5E-01	excluded
TMEM143	50736	ES	3.1:3.2:4:5	2	6	0.01	5.5E-01	included
RAB7A	66656	ES	4.1:4.2	3.2	5.1	0.00	5.5E-01	included
EIF1AD	16973	ES	1.2:2.2:2.3	1.1	2.4	0.01	5.5E-01	included
GSTM1	4066	ES	7	6	8	0.00	5.5E-01	excluded
C19orf54	49986	ES	7.3:8.1:8.2:8.3:8.4:8.5:8.6:8.7:8.9	7.2	8.1	0.00	5.5E-01	included
SEPT10	54914	ES	3.1	1	4	0.00	5.5E-01	excluded
SLAIN2	69213	ES	7:08	6	9	0.00	5.5E-01	excluded
RPS2	33171	ES	5	4	6.1	0.00	5.5E-01	included
PATZ1	61848	ES	5.2	4	6	-0.01	5.5E-01	excluded
PODXL	81823	ES	4.2:4.3:4.4:4.6:4.7	4.1	4.8	0.00	5.5E-01	included
ZDHHC24	17066	ES	6	5	7	-0.01	5.5E-01	excluded
MSL1	40844	ES	4	3.1	5	-0.01	5.5E-01	excluded
CD300A	43251	ES	8	7	9	0.01	5.5E-01	included
GSR	83301	ES	8:09	7	10	0.00	5.5E-01	included
SS18	44982	ES	3	2	9	0.00	5.5E-01	excluded
KIAA0753	38761	ES	9	8	10	0.00	5.5E-01	excluded
CPSF2	28990	ES	4	3	5	0.00	5.5E-01	included
EXOSC2	87906	ES	4	3	5	0.00	5.5E-01	included
KBTBD8	65552	ES	2:03	1	4	0.00	5.5E-01	excluded
ERBB3	22360	ES	11:12:13:14:15:16:17.1:17.2:18:19:20.2:21:22:23:24.2:24.4:25:26	10	27.2	0.00	5.5E-01	included
RAB11FIP1	83366	ES	4	3	5.1	-0.01	5.5E-01	excluded
BCL2L13	60988	ES	07:08.1	6	9	0.00	5.5E-01	excluded
AGFG2	80957	ES	4:05	3	6	0.00	5.5E-01	included
PAPOLG	53669	ES	10	9	11	0.00	5.5E-01	included
LHPP	13402	ES	7	5	8	0.00	5.5E-01	included
NAPA	50663	ES	2	1	3	0.00	5.5E-01	excluded
FBXO16	83217	ES	9	8	10	0.00	5.5E-01	excluded
TSPAN14	12369	ES	7:08:09	6	10	0.00	5.5E-01	included
NME7	8913	ES	8	7	9	0.00	5.5E-01	excluded
ATP6V0A1	41054	ES	2.2:3:4:5:6.2:7:8:9:10.	2.1	10.2	0.00	5.5E-01	included
SRI	80358	ES	7.2:8.1	7.1	8.2	0.00	5.5E-01	excluded
CCDC101	35755	ES	5	4	6	0.00	5.6E-01	excluded
DNAJC18	73611	ES	3	2	4	0.00	5.6E-01	included
CAPNS1	49365	ES	2.4:3:4:5:6:7.2:8.1	2.3	8.2	0.00	5.6E-01	included
DNAJC11	496	ES	9.2:10:11:12:13:14:15	9.1	17.2	0.00	5.6E-01	excluded
DMPK	50527	ES	2.2	1	2.4	0.01	5.6E-01	included
DMTF1	80294	ES	17.1:17.2:17.3	16	18	0.00	5.6E-01	included
FAM134C	41089	ES	6	5	7	0.00	5.6E-01	included
STAU2	84171	ES	3:04:05	2	7	0.01	5.6E-01	included
TMEM218	19283	ES	2:03	1.2	4.2	0.01	5.6E-01	included
SIRT5	75396	ES	6	4	7	0.00	5.6E-01	included
HNRNPC	26540	ES	6.2:6.3:7.1	6.1	7.2	0.00	5.6E-01	included
ARHGAP4	90533	ES	5	4	6.2	0.00	5.6E-01	included
TEK	86045	ES	2	1	3	0.00	5.6E-01	excluded

DHCR24	3180	ES	7	6	8	0.00	5.6E-01	excluded
RBM42	49226	ES	5	4	6.1	0.00	5.6E-01	excluded
TREM1	76109	ES	3.1	2	4	0.01	5.6E-01	included
NAP1L1	23496	ES	2	1	5	0.01	5.6E-01	included
TMUB2	41799	ES	4.4:4.5:4.8	4.3	5	0.00	5.6E-01	excluded
CUX1	81080	ES	3	1	4	0.00	5.6E-01	included
BMP1	82994	ES	6	5.2	7	0.00	5.6E-01	included
ANAPC11	44221	ES	3.3:5	3.2	6	0.00	5.6E-01	included
TCTN1	24481	ES	3.1:3.2	1.1	4	0.00	5.6E-01	excluded
COPS7B	57964	ES	3:4.1:4.2:4.3:4.4:5	1	6	0.01	5.6E-01	included
REEP5	72996	ES	3:04	2	5	0.00	5.6E-01	excluded
SEPHS1	10796	ES	2	1	3	0.00	5.6E-01	excluded
TCF4	45618	ES	16.1:16.2:17	13	20.2	0.00	5.6E-01	excluded
NEIL2	82633	ES	1.2:1.3:2.1	1.1	3	0.01	5.6E-01	included
ICMT	386	ES	4	2	5	0.00	5.6E-01	included
FAM3A	90628	ES	7.1:7.2	6	8	0.01	5.6E-01	included
PTP4A2	1537	ES	3	2	6	0.00	5.6E-01	included
PPCDC	31831	ES	3.2:4	2	5	0.00	5.6E-01	included
C14orf79	29590	ES	2.4	2.2	3.2	0.01	5.6E-01	included
PLA2G15	37201	ES	2:03	1	4.1	0.00	5.6E-01	included
RNF121	17455	ES	6.1:6.2:7:8	3	9	0.00	5.6E-01	included
PRKAR2A	64781	ES	9	8	10	0.00	5.6E-01	included
SPCS2	17806	ES	2	1	3	0.00	5.6E-01	included
PARD3B	57099	ES	21	20	22	0.00	5.6E-01	included
ACTR10	27679	ES	6.1:6.2:7.1:7.2	5	8	0.00	5.6E-01	excluded
RHOT1	40180	ES	19.3	19.1	20	0.01	5.6E-01	included
RCAN3	1116	ES	4.1:4.2	3	5	0.00	5.6E-01	included
ABCD4	28381	ES	7	6	8.2	0.00	5.6E-01	included
CDIP1	33757	ES	6.1:6.2	5.2	7	0.00	5.6E-01	included
CHMP7	83075	ES	3	2.2	5	0.00	5.6E-01	included
RCOR3	9710	ES	11	10	12	0.00	5.6E-01	included
CHMP7	83074	ES	3:04	2.2	5	0.00	5.6E-01	excluded
OXNAD1	63643	ES	4:05	3.2	6	-0.01	5.6E-01	excluded
CBWD5	86500	ES	13:14	12.1	15	0.00	5.6E-01	included
RBBP6	35645	ES	11:12:13:14:15:16:17	10	18	0.00	5.6E-01	excluded
ECD	12130	ES	7	6	8	0.00	5.6E-01	included
TCTN1	24482	ES	3.2	1.1	4	0.00	5.6E-01	excluded
RPRD1A	45205	ES	2:03	1	4	0.00	5.6E-01	excluded
CIB2	31999	ES	3	2	4	0.00	5.6E-01	excluded
ZNF302	48985	ES	4:5.1:5.2:6.1	2	6.2	0.00	5.6E-01	excluded
TBC1D17	51116	ES	4	3	5	-0.01	5.6E-01	excluded
TMEM237	56853	ES	7	6	9	0.00	5.6E-01	excluded
HAGH	33148	ES	1.2:2	1.1	3	0.00	5.6E-01	excluded
MIB2	192	ES	11	10.2	12.1	0.00	5.6E-01	included
CCT4	53707	ES	4	3	5	0.00	5.6E-01	included
HMOX2	33751	ES	2.2:3	2.1	7	0.00	5.6E-01	excluded
NASP	2744	ES	07:02.2	3	10	0.00	5.6E-01	included
PTCD2	72459	ES	5	4	6	0.00	5.6E-01	included
BID	61008	ES	4	2	5	0.00	5.6E-01	excluded
CSPP1	84078	ES	20	19	21	0.00	5.6E-01	excluded
DDX46	73427	ES	3	2	4	0.00	5.6E-01	included
MTMR12	71652	ES	3	1	4	-0.01	5.6E-01	excluded
BLOC1S6	30448	ES	03:07.1	1	7.2	0.00	5.6E-01	excluded
CLCN4	88459	ES	3:04	2	5	0.00	5.6E-01	included
LAS1L	89323	ES	2	1	3	0.00	5.6E-01	included
CD164	77189	ES	5:06	4	7.1	0.00	5.6E-01	excluded
CC2D2A	68811	ES	1.2:1.3:3.2	1.1	3.3	0.00	5.6E-01	included
TRA2B	68039	ES	2	1	3	-0.01	5.6E-01	excluded
DLG1	68284	ES	26	25.1	27	0.00	5.6E-01	excluded
ZNF346	74717	ES	3:4:5.1:5.2:6.2	1	7	0.00	5.6E-01	excluded
RNF4	68574	ES	8.1	6	9.1	0.00	5.6E-01	excluded
ACAD9	66670	ES	2.1:2.2:2.3	1.2	3.2	0.00	5.6E-01	included

SDCBP	83929	ES	6.1:6.2	5	7.1	0.00	5.6E-01	included
SRR	38384	ES	2:3:4:5:6	1	7	0.00	5.6E-01	excluded
PCK2	26825	ES	2.2:2.3:3	2.1	4	0.00	5.6E-01	excluded
ARL13B	65685	ES	3.2:4:5.1	2	6	0.00	5.6E-01	excluded
IDH3A	32029	ES	2:4.1:4.2:5:6.1:6.2:7:8	1	11.1	0.00	5.6E-01	included
CANX	74920	ES	5	4	6	0.00	5.6E-01	excluded
ATXN3	28960	ES	3.1:3.2:4.2:5	2	6	0.00	5.6E-01	included
MR1	9136	ES	4:05	3.2	6	0.00	5.6E-01	excluded
E2F6	52686	ES	6:07	5.2	8	0.00	5.6E-01	excluded
STXBP2	47125	ES	13	12	14	0.00	5.6E-01	included
RAP1GDS1	69998	ES	7	5	8	0.01	5.6E-01	included
CNOT8	74279	ES	1.2:1.3:1.4:2:3	1.1	5	0.00	5.6E-01	included
DMKN	49202	ES	7:08:09	6.4	12	-0.01	5.6E-01	excluded
SMIM8	76952	ES	2.1	1.1	3	0.00	5.6E-01	excluded
GLB1	63835	ES	3:4.2:5:6	1	7	0.00	5.7E-01	included
RGS5	8773	ES	4:05	2	6.1	0.00	5.7E-01	excluded
PIGT	59570	ES	05:02.2	1	7	-0.01	5.7E-01	excluded
ATG5	77118	ES	4	3	5	0.00	5.7E-01	included
PER1	39095	ES	20	19.2	21	0.00	5.7E-01	excluded
TMEM176B	82260	ES	3	2.1	4.3	0.00	5.7E-01	excluded
TMEM218	19264	ES	6	5.3	7.1	0.00	5.7E-01	included
CSNK1G3	73150	ES	2.2	1	3	0.00	5.7E-01	excluded
GPAT2	54512	ES	19:20	18	21	-0.01	5.7E-01	excluded
TUBD1	42811	ES	4:5:6:7	3	8.1	-0.01	5.7E-01	excluded
POSTN	25671	ES	21	20	22	0.01	5.7E-01	included
TMEM232	72940	ES	3:04:05	2	6	0.00	5.7E-01	excluded
KRAS	20822	ES	4:05	2	7	0.00	5.7E-01	excluded
LAMTOR1	17558	ES	1.2:2.1	1.1	2.2	0.00	5.7E-01	excluded
ZNF706	84750	ES	3.2	2	4	0.00	5.7E-01	excluded
APAF1	23875	ES	8:9:10:11:12:13:14:15<br>:16:17:19:20:21:22:23	7	27	0.00	5.7E-01	included
NEK11	66785	ES	14	13	15	0.00	5.7E-01	excluded
RBM6	64943	ES	4:5:6:7	2	8	0.00	5.7E-01	excluded
NFIB	85892	ES	12.1:13	11	15	0.01	5.7E-01	included
ZFAT	85266	ES	6	5	7	0.00	5.7E-01	included
CYBRD1	55937	ES	3	2	4	0.00	5.7E-01	excluded
ERGIC3	59175	ES	11	8	12	0.00	5.7E-01	excluded
LRRFIP2	63974	ES	7:8:9:10:11:12:13:14	5	18	0.00	5.7E-01	excluded
TPD52L2	60172	ES	4	3	5	0.00	5.7E-01	excluded
SRSF1	94623	ES	3.3	3.1	3.5	-0.01	5.7E-01	excluded
ZNF415	51675	ES	7.2:8.1:8.2	6.1	9	-0.01	5.7E-01	excluded
GNAI2	64971	ES	6	2	7	0.00	5.7E-01	included
WDR18	46359	ES	9	8	10	0.00	5.7E-01	excluded
LRRC28	32697	ES	3:5:6:7.1:7.2:8:10	2	12	0.00	5.7E-01	excluded
NUMB	28289	ES	11:12:13	10	14	0.00	5.7E-01	included
PXK	65446	ES	5:06:07	4	8	0.00	5.7E-01	excluded
MYO5C	30648	ES	26	25	27	0.00	5.7E-01	included
NT5C3B	40958	ES	2	1.1	4	0.00	5.7E-01	included
PCK2	26828	ES	2.3	2.1	4	0.00	5.7E-01	excluded
SEC24C	12182	ES	3:4:5:6:7:9:10:11:12:1<br>3:14:15:16:17	2	18	0.00	5.7E-01	included
KIAA0391	27217	ES	4	3.2	5	0.00	5.7E-01	included
IFI16	8399	ES	8	7	10	-0.01	5.7E-01	excluded
ORC3	76969	ES	3:04	2	5	0.00	5.7E-01	included
DIO1	3084	ES	2.1:2.2:3	1.5	4	0.00	5.7E-01	excluded
MRPL55	10160	ES	2.2:2.4:2.5	1.1	2.9	0.00	5.7E-01	included
ZNF687	7587	ES	5:06	4	7	0.00	5.7E-01	excluded
ANKS3	33820	ES	5.1:5.2:6	3	8	0.00	5.7E-01	included
MTM1	90338	ES	5	4	6	0.00	5.7E-01	included
LPHN2	3568	ES	27:28:00	26	31	0.00	5.7E-01	included
TRAPPC4	19065	ES	2.3:2.4:3.1	2.2	3.2	0.00	5.7E-01	included
SLC12A9	81007	ES	2:3:4:5	1.2	6	0.00	5.7E-01	included

NACA	22486	ES	4.3:5:6.1	4.2	6.2	0.00	5.7E-01	excluded
YPEL3	36073	ES	4.1	3.1	5	0.00	5.7E-01	excluded
BBX	66010	ES	13	12	14	0.00	5.7E-01	included
MORN4	12732	ES	4	3	5	0.00	5.7E-01	included
PARP2	26428	ES	5:06	4	7	0.00	5.7E-01	included
ELMO3	36975	ES	5	4	6	0.00	5.7E-01	included
SEC11A	32313	ES	6:08	5	9	0.01	5.7E-01	included
LOXL3	54109	ES	8	7	9	0.00	5.7E-01	excluded
PPP6R3	17322	ES	7:8:9:10	6	11	0.00	5.7E-01	included
NAT10	14947	ES	9:10:11:12:13:14:15:1 6:17:18:19:20:21:22:2 3:24:25:26:27:28:29.1	8	29.2	0.00	5.7E-01	excluded
LRRC6	85182	ES	14	13.2	15	-0.01	5.7E-01	excluded
SLC18B1	77751	ES	5	4	6	0.00	5.7E-01	included
GEMIN2	27354	ES	10	9	11	0.00	5.7E-01	included
PCNXL4	27769	ES	2.1	1	3	0.01	5.7E-01	included
ST5	14271	ES	16	15	17	0.00	5.7E-01	excluded
ALPK1	70371	ES	4	3.2	5	0.00	5.7E-01	excluded
ILF3	47578	ES	6	5	7	0.00	5.7E-01	excluded
SPIRE2	38154	ES	14:15	13	16	0.00	5.7E-01	included
IMMT	54408	ES	6.3:7.1	5	7.2	0.00	5.7E-01	excluded
SMARCA4	47612	ES	32	31	33	0.00	5.7E-01	included
STT3A	19305	ES	3	1	4	0.00	5.7E-01	excluded
EMC4	29843	ES	2.3:3.1:3.2:4.2	2.2	6	0.00	5.7E-01	included
PCSK7	18901	ES	14	13	15	0.00	5.7E-01	excluded
LRIF1	4129	ES	2.2:2.3	1	3	-0.01	5.7E-01	excluded
LCMT1	35668	ES	6:07	5	8	0.00	5.7E-01	excluded
ZNF177	47308	ES	12	11	13	-0.02	5.7E-01	excluded
ACSL1	71325	ES	8	7	9	0.00	5.7E-01	included
PMM2	33950	ES	5.1:5.2	3	6	0.00	5.7E-01	excluded
HDAC8	89499	ES	3.1:3.2	2	4.1	0.00	5.7E-01	excluded
PIGB	30719	ES	2:03:04	1	5	0.00	5.7E-01	included
UBA52	48475	ES	2	1.3	4.2	0.00	5.7E-01	excluded
RAB8B	31020	ES	6	5	7	0.00	5.7E-01	excluded
SSFA2	56441	ES	19	18	20	0.00	5.7E-01	excluded
SLC25A23	47042	ES	13	10	14	-0.01	5.7E-01	excluded
DCTD	71237	ES	2.2:3	2.1	5	0.00	5.7E-01	included
SIK3	18876	ES	20	19	21	0.00	5.7E-01	included
BSDC1	1593	ES	6.2:7:8.1	6.1	8.2	0.00	5.7E-01	excluded
STOM	87440	ES	2	1	3	0.00	5.7E-01	included
LASP1	40612	ES	4	3	5	0.00	5.7E-01	excluded
CRK	38303	ES	2.2:2.3:3.1	2.1	3.2	0.00	5.7E-01	included
MRPL55	10165	ES	2.5	1.1	2.9	0.00	5.7E-01	included
SMIM7	48198	ES	5	2	7.1	0.00	5.7E-01	excluded
MOK	29389	ES	04:06.1	3	7	0.01	5.7E-01	included
MUM1	46458	ES	5.1	4	6	0.01	5.7E-01	included
SKP1	73359	ES	6	4	7	0.00	5.7E-01	included
POC1B	23634	ES	6:7:8:9	4	10	0.00	5.7E-01	included
COPE	48519	ES	4:05	3	6	-0.01	5.7E-01	excluded
ZNF564	47805	ES	2	1	4	0.01	5.7E-01	included
TPM1	30992	ES	5.2	4	6	0.00	5.7E-01	included
ANKRD27	48899	ES	4	3	5	0.01	5.7E-01	included
RPRD1B	59371	ES	3	2	4	0.00	5.7E-01	excluded
DOCK9	26176	ES	59	58	60	0.00	5.7E-01	excluded
NSUN5	97503	ES	2:3.1:3.2:4	1	5	0.00	5.7E-01	excluded
MEF2D	8280	ES	5	4	7	0.00	5.7E-01	included
BRPF3	75961	ES	9:10:11.1:11.2	8	12	0.00	5.7E-01	included
EML2	50499	ES	20	18	21	0.00	5.8E-01	excluded
FAM150A	83818	ES	4	3	5	0.00	5.8E-01	excluded
MBNL2	26146	ES	5:6.1:6.2:6.3:7	4	8	0.00	5.8E-01	excluded
ADCK1	28634	ES	5	3	6	0.00	5.8E-01	included
SAR1A	12040	ES	03:04.1	1	4.2	0.00	5.8E-01	excluded

SLC35B4	81845	ES	4	3	5	0.00	5.8E-01	included
ACOT8	59630	ES	2:04	1	5	0.00	5.8E-01	included
KIDINS220	52602	ES	28	27	29	0.00	5.8E-01	included
NADK2	71809	ES	11	9	12	0.00	5.8E-01	included
MIPOL1	27301	ES	11:12:13:14	10	15	0.00	5.8E-01	included
IL6ST	72074	ES	12	11	13	0.00	5.8E-01	excluded
VPS26A	11982	ES	4	3	5	0.00	5.8E-01	excluded
SMEK2	53624	ES	12	11	13	0.00	5.8E-01	excluded
ANP32E	7438	ES	04:05.1	1.3	5.2	0.00	5.8E-01	included
C3orf18	65063	ES	5	4.3	6.2	0.02	5.8E-01	included
TEX264	65105	ES	1.2:3	1.1	4	0.01	5.8E-01	included
EYA2	59706	ES	10	9	11	0.00	5.8E-01	excluded
SPAG6	10981	ES	3:04:05	2.1	6	0.01	5.8E-01	included
DDX19A	37381	ES	3	1	4	0.00	5.8E-01	excluded
R3HDM4	46358	ES	3.1	1	3.3	0.00	5.8E-01	excluded
PTCD2	72460	ES	4:05	3	6	0.00	5.8E-01	excluded
ERBB2	40682	ES	32	31	33	0.00	5.8E-01	excluded
SFTA3	27269	ES	4.2:4.3:5	2	6	0.00	5.8E-01	included
DVL3	67838	ES	2	1	3	0.00	5.8E-01	excluded
SMIM7	48200	ES	3	2	5	0.00	5.8E-01	excluded
SENP5	68261	ES	7	6	8	-0.01	5.8E-01	excluded
ALDH3A2	39759	ES	4	3	5	-0.01	5.8E-01	excluded
CDK4	22740	ES	2:3.1:3.2:4:5.1:5.2	1	7	0.00	5.8E-01	included
LGR4	14755	ES	3:04	2	5.1	0.00	5.8E-01	excluded
HSCB	61549	ES	2.1:3.1:3.2:4	1	5	0.00	5.8E-01	included
LRRFIP2	63979	ES	6:7:8:10:14	5	18	0.00	5.8E-01	included
PPP2R3C	27200	ES	7.1	6.1	8.1	0.00	5.8E-01	excluded
VTI1B	28085	ES	2	1	3	0.00	5.8E-01	excluded
RBFOX2	61983	ES	13	12	14	0.00	5.8E-01	included
RFXANK	48608	ES	5	4.2	6	0.00	5.8E-01	included
FIP1L1	69313	ES	11	10	12	0.00	5.8E-01	excluded
ATP8B1	45648	ES	3	2	4	0.00	5.8E-01	included
MCCC1	67780	ES	8	7	9	0.00	5.8E-01	included
COX18	69478	ES	2.2	1	3	0.00	5.8E-01	included
ZNF133	58778	ES	5.1:5.2:6.1:6.2:8.1	1	8.2	-0.01	5.8E-01	excluded
TSPAN14	12376	ES	3:05	1	6	0.01	5.8E-01	included
DNAJA3	33722	ES	3	1	4	0.00	5.8E-01	included
WDR77	4166	ES	3:04	2	5	0.00	5.8E-01	included
PGS1	43879	ES	3.1:3.2	1	4	0.00	5.8E-01	included
L3MBTL2	62412	ES	16	15	17	0.00	5.8E-01	included
RAB5B	22328	ES	5	4	6	0.00	5.8E-01	included
ZSWIM7	39408	ES	1.2:2	1.1	4	0.00	5.8E-01	included
ACBD4	41944	ES	10	9.2	12	0.00	5.8E-01	included
MAP4	64552	ES	23:24.1	22	24.2	0.00	5.8E-01	excluded
POLDIP3	62539	ES	3.2	2	4	0.00	5.8E-01	excluded
NVL	9948	ES	6:07	4	8	0.00	5.8E-01	included
CNOT1	36672	ES	26	25	27	0.00	5.8E-01	excluded
DBI	55118	ES	1.2:3.2	1.1	5	0.00	5.8E-01	excluded
THOC5	61617	ES	3	1	4	-0.01	5.8E-01	excluded
TMEM131	54605	ES	13	12	14	0.00	5.8E-01	excluded
CASP1	18525	ES	4	3.4	5	0.00	5.8E-01	included
GNB2L1	190579	ES	4.2:5:6:7.2:8.1:8.2	3	9	-0.01	5.8E-01	excluded
PTK2	85317	ES	9.1	8	10	0.00	5.8E-01	excluded
DUOX1	30403	ES	12.3:13	12.2	14	0.00	5.8E-01	included
GPBP1	72132	ES	3.1	2	6	0.00	5.8E-01	excluded
SMPD4	55300	ES	11	10	14	0.01	5.8E-01	included
FBXL20	40643	ES	8	7	9	0.00	5.8E-01	excluded
TRPT1	16578	ES	7.2	6	8	0.02	5.8E-01	included
WDFY3	69811	ES	45	44	46	0.01	5.8E-01	included
RAB7A	66662	ES	3.2:4.1:5.1	3.1	5.2	-0.01	5.8E-01	excluded
KANSL3	54560	ES	5.2:6:7	3.2	9	0.00	5.8E-01	included
PLD1	67638	ES	15	14	17	0.00	5.8E-01	included

MBD4	66722	ES	2.2:3.1	2.1	4	0.00	5.8E-01	excluded
RAP1GDS1	69997	ES	6:07	5	8	0.00	5.8E-01	included
QTRTD1	66246	ES	5	4	6	0.00	5.8E-01	excluded
GPD1L	63812	ES	2	1	3	0.00	5.8E-01	included
SLC11A2	21736	ES	08:09.1	7.2	10	0.00	5.8E-01	included
UBA52	48485	ES	1.2:2	1.1	4.2	0.00	5.8E-01	included
IP6K2	64757	ES	11.4:11.5:11.6:11.7	11.2	11.9	0.00	5.8E-01	included
OARD1	76089	ES	2.3:3.2	2.2	4.1	0.00	5.8E-01	excluded
HMCES	66715	ES	5	4	6	0.00	5.8E-01	included
SERAC1	78267	ES	16.1	15	16.5	0.00	5.8E-01	included
EIF1AD	16968	ES	2.2:2.3:2.4	1.1	3.1	0.00	5.8E-01	included
FANCD2	63308	ES	38	37	39	0.00	5.9E-01	excluded
SEC31A	69739	ES	5.2	4	6	0.00	5.9E-01	included
STT3A	19306	ES	2	1	3	0.00	5.9E-01	excluded
OS9	22699	ES	5.3:6	5.2	7.1	0.00	5.9E-01	included
ATP6V0A1	41045	ES	19:21.1	18	21.2	0.00	5.9E-01	excluded
MTFP1	61751	ES	3.3	2	4	-0.01	5.9E-01	excluded
TTC7A	53487	ES	10	9	11	0.00	5.9E-01	included
HIP1	80120	ES	24:25:00	23	26	0.00	5.9E-01	excluded
RGS6	28221	ES	15	14	16	0.01	5.9E-01	included
UBR4	882	ES	90	89	91	0.00	5.9E-01	excluded
FDXR	43322	ES	7.1:7.2	6.4	8	0.00	5.9E-01	included
UBXN2B	83926	ES	3	2	4	0.00	5.9E-01	excluded
CXorf38	88845	ES	1.2:1.3:2.3	1.1	3	0.00	5.9E-01	included
ZNF263	33513	ES	6	2	7	-0.01	5.9E-01	excluded
GANAB	16378	ES	6	5.1	7	0.00	5.9E-01	excluded
NRP1	11199	ES	19	18	20	0.00	5.9E-01	included
SEPT2	58372	ES	3	2	4	0.01	5.9E-01	included
AFMID	43810	ES	7:08	6	12	0.01	5.9E-01	included
GOLGA4	63987	ES	5:6:7:8:9:10:11:12:13:<br>14:15:16:17:18:20:21:	4	25	-0.01	5.9E-01	excluded
PIEZO2	44635	ES	50	49	51	0.00	5.9E-01	included
GTPBP3	48293	ES	5.2:6.1:6.3	5.1	6.4	0.00	5.9E-01	included
ASCC1	12086	ES	5	4	6	0.00	5.9E-01	included
OS9	22698	ES	6	5.3	7.1	0.00	5.9E-01	excluded
STRBP	87503	ES	8	7	9	0.00	5.9E-01	excluded
HARS2	73762	ES	2.2	1.3	3	0.00	5.9E-01	excluded
HMBS	19105	ES	4	1.1	5.1	0.00	5.9E-01	included
CRCP	79875	ES	3:05:06	1.2	7	0.00	5.9E-01	excluded
PDE9A	60742	ES	2:3:5:6:7	1	8	0.00	5.9E-01	excluded
EPN2	39705	ES	2.1:2.3	1	4	0.00	5.9E-01	excluded
ASCC2	61680	ES	6:07	5	8	0.01	5.9E-01	included
EIF3K	49679	ES	6	5	7.1	0.00	5.9E-01	included
FCHSD2	17677	ES	2	1	3	0.00	5.9E-01	excluded
ACSF3	38064	ES	02:03.2	1.1	4	0.00	5.9E-01	included
SREBF2	62464	ES	17:18	16	19	0.00	5.9E-01	excluded
EFNA3	7938	ES	5	4	6	0.01	5.9E-01	included
WHSC1L1	83395	ES	16	15	17	0.01	5.9E-01	included
AMD1	77244	ES	3:4:5:6	1	7	0.00	5.9E-01	excluded
CEP152	30503	ES	22	21	23	0.00	5.9E-01	included
ATE1	13326	ES	3:04	2	5	0.00	5.9E-01	included
PSME2	26865	ES	02:03.1	1	3.2	0.00	5.9E-01	included
ALDH3A2	39756	ES	10	9	13.1	-0.02	5.9E-01	excluded
TCTN1	24467	ES	9	8	10	0.00	5.9E-01	excluded
FDFT1	82649	ES	7	6.4	8.1	0.00	5.9E-01	excluded
ANXA7	12146	ES	3.2:4	3.1	5	0.00	5.9E-01	included
RCOR3	9712	ES	3	2.2	4	0.00	5.9E-01	included
TMEM161B	72734	ES	11.6	11.4	12.1	-0.01	5.9E-01	excluded
VPS29	24442	ES	4	1	5	0.00	5.9E-01	included
ARFGAP2	15645	ES	4.2:5:6.1:6.2:7:8	3	9	0.00	5.9E-01	included
RIPK1	75164	ES	4	3	5	0.00	5.9E-01	included
ERBB2	40683	ES	8.2:9:10:11:12:13	8.1	14	0.00	5.9E-01	included

ZNF7	85661	ES	5.1:5.2	4.2	6.1	-0.01	5.9E-01	excluded
ECHDC2	3032	ES	2.2:5.1:6.1	2.1	6.2	0.00	5.9E-01	excluded
C18orf8	44826	ES	5	4	6	0.00	5.9E-01	excluded
HM13	58892	ES	11:12.1:12.2	10	13	0.01	5.9E-01	included
LILRA1	51926	ES	6:07	5	8	0.00	5.9E-01	excluded
C14orf80	29662	ES	8:09	7	10	-0.01	5.9E-01	excluded
MAP3K5	77919	ES	15	14	16	0.00	5.9E-01	included
FAS	12483	ES	3:04	2	5	0.00	5.9E-01	excluded
TCOF1	74071	ES	18	17	20	0.01	5.9E-01	included
CDC27	42051	ES	9.2:10:11:12.1:12.2:13	8	15	0.00	5.9E-01	excluded
DAG1	64887	ES	4	2.1	7	0.00	5.9E-01	included
FAM179B	27390	ES	15	14	16	0.00	5.9E-01	included
RWDD4	71273	ES	7	6	8	0.00	5.9E-01	excluded
GUCY1B3	70946	ES	4	3	5	0.00	5.9E-01	included
LYVE1	14359	ES	02:03.2	1.1	4	0.00	5.9E-01	excluded
TFB2M	10493	ES	3	2	4	0.00	5.9E-01	included
TARS2	7477	ES	6:07	5	10	0.00	5.9E-01	excluded
RABL6	88216	ES	14.2:15:16.1	14.1	16.2	0.00	5.9E-01	excluded
MYNN	67579	ES	9	7	10	0.00	5.9E-01	included
ACADM	3499	ES	3:4.2:6:7	2.2	8	0.00	5.9E-01	excluded
ATG16L2	17662	ES	4.3:5:6.1:6.2:6.3:7:8:9 :10:11:12:13	3	14	0.00	5.9E-01	included
SIRT2	49708	ES	15.2:16:17	15.1	18	0.00	5.9E-01	excluded
BAP1	65215	ES	4	3	5	0.00	5.9E-01	excluded
PACRGL	68884	ES	10	9	13.1	0.00	5.9E-01	excluded
TARS2	7476	ES	6:7:8:9	5	10	0.00	5.9E-01	excluded
LMBR1	82472	ES	13	12	14	0.00	5.9E-01	included
UTP14A	90056	ES	4	3	5	0.00	5.9E-01	excluded
AACS	25176	ES	18	17	19	0.00	5.9E-01	included
CAMK2D	70418	ES	15	14.2	18	0.00	5.9E-01	excluded
AACS	25178	ES	4	3	5	0.00	5.9E-01	excluded
RBM39	59246	ES	9	6	10	0.00	5.9E-01	excluded
SYNC	1630	ES	4	3	5	0.01	5.9E-01	included
PFDN5	22003	ES	2:03:05	1	6.2	0.00	5.9E-01	included
METAP2	23794	ES	2.2	1	3	0.00	5.9E-01	included
DCTD	96838	ES	1.2:2.1:2.2	1.1	5	-0.01	5.9E-01	excluded
PTP4A2	1523	ES	6:07	3	8	0.00	5.9E-01	excluded
MRPL55	10132	ES	1.2:2.4:2.5:2.6:2.7:2.8	1.1	2.9	0.00	5.9E-01	excluded
MIPOL1	27323	ES	5:6:8.1:8.2:9:10	1	11	0.00	5.9E-01	excluded
SSFA2	56444	ES	4	2	5	0.00	5.9E-01	included
MRPL42	23702	ES	3.1:3.2	2.2	4	0.00	5.9E-01	excluded
LRRC48	39530	ES	2	1	3	0.01	5.9E-01	included
HMBS	19100	ES	12.1	11.1	12.3	0.00	5.9E-01	excluded
NCOR1	39424	ES	03:04.2	2	5	0.00	5.9E-01	included
ITGA2	71990	ES	24	23	25	0.00	5.9E-01	excluded
COX5A	31814	ES	3	1	4	0.00	5.9E-01	excluded
CCNB1IP1	26423	ES	3.2	1	4	0.00	6.0E-01	excluded
NEBL	10964	ES	30:31:00	29	32	0.00	6.0E-01	excluded
RHOT1	40184	ES	20:21	19.1	22	0.00	6.0E-01	excluded
MXI1	13082	ES	6	5	7	0.00	6.0E-01	excluded
WDYHV1	85065	ES	4	3	5	0.00	6.0E-01	included
CDK4	22737	ES	3.1:3.2	2	4	0.00	6.0E-01	included
POMZP3	80186	ES	6:07	5	8	0.00	6.0E-01	excluded
BRPF3	75958	ES	11.1:11.2	10	12	0.00	6.0E-01	included
ASCC2	61676	ES	8:9.1:9.2	5	10	0.00	6.0E-01	excluded
NUP54	69584	ES	12	11	13	0.00	6.0E-01	excluded
PARP8	71980	ES	22	21	23	0.00	6.0E-01	included
MVP	233980	ES	2.3:3:7.1	2.2	7.2	0.01	6.0E-01	included
NAA60	33532	ES	8	7	9	0.00	6.0E-01	excluded
ITGAL	36133	ES	2.3:4.2:5:6:7:8:9.1:10: 11:12:13:14:15:16:17.	2.2	18.2	0.00	6.0E-01	included
ABR	38291	ES	23.2:24:25:26:27.1	23.1	27.2	0.00	6.0E-01	excluded

PIAS2	45425	ES	2	1	3	0.00	6.0E-01	excluded
AHI1	77895	ES	29	28	30.1	0.00	6.0E-01	included
HAUS7	90440	ES	7.1	5	8	0.00	6.0E-01	included
CXorf38	88846	ES	1.3:2.3	1.1	3	0.00	6.0E-01	excluded
TRIQK	84513	ES	4	2.1	6.2	0.00	6.0E-01	excluded
CD99L2	90359	ES	6:07:08	5	9	0.00	6.0E-01	included
CORO7	33711	ES	2.2	1.2	3	0.00	6.0E-01	excluded
ZNF341	59016	ES	3	2	4	0.00	6.0E-01	included
PUM2	52775	ES	5:06	4.2	7	0.00	6.0E-01	excluded
MYD88	64038	ES	2	1	3.1	0.00	6.0E-01	included
TMEM150A	54304	ES	6:07	5	8	-0.01	6.0E-01	excluded
AP2M1	67841	ES	7	6	8	0.00	6.0E-01	excluded
TRMT10B	86441	ES	3.2	2	4.1	0.00	6.0E-01	included
QTRTD1	66249	ES	3.1:3.2:4:5	1	6	0.00	6.0E-01	excluded
CEP170B	29576	ES	2	1	3	0.00	6.0E-01	included
MED15	61169	ES	12	11	13	0.01	6.0E-01	included
MRPL55	10163	ES	2.2:2.5	1.1	2.9	0.00	6.0E-01	included
FGGY	3213	ES	5:06	4	7	0.00	6.0E-01	included
OGFOD1	36465	ES	8	7	9	0.00	6.0E-01	excluded
BIN3	83051	ES	3:04	2.2	6	0.00	6.0E-01	included
BRD1	62755	ES	4:05	3.2	6	0.00	6.0E-01	excluded
ATF2	56066	ES	11	10.1	12	0.00	6.0E-01	included
CRTAC1	12750	ES	13	12	14	0.00	6.0E-01	included
IRF3	51000	ES	2	1.4	3	0.00	6.0E-01	excluded
SLC25A17	62385	ES	3.1:3.2	2.2	4	0.00	6.0E-01	excluded
DIS3L2	57987	ES	20	19	21	0.00	6.0E-01	excluded
TG	85208	ES	26	25	27	0.00	6.0E-01	included
DHRS4	26780	ES	6	5	7.1	0.00	6.0E-01	excluded
MATR3	73582	ES	8:09	7.1	10	0.00	6.0E-01	excluded
ARHGAP39	85636	ES	7	6	8	0.00	6.0E-01	excluded
ENSA	7500	ES	3.2:3.3:4	3.1	5.1	0.00	6.0E-01	excluded
KLC4	76225	ES	14	13	15	0.00	6.0E-01	excluded
TAGAP	78297	ES	5	4	6	0.00	6.0E-01	included
LRRC37B	40172	ES	11	10	12	0.00	6.0E-01	included
SUGP1	48627	ES	2.1	1	3	0.00	6.0E-01	excluded
ECHDC1	77465	ES	9	6.3	10.2	0.00	6.0E-01	included
NBPF11	7337	ES	10:11:12:13	8.3	14	0.01	6.0E-01	included
NFATC1	46245	ES	3	2	4	0.00	6.0E-01	excluded
ANXA5	70490	ES	4	3	5	0.00	6.0E-01	included
IFI44	3556	ES	7.1:8	6	9	0.00	6.0E-01	included
CENPH	72307	ES	5	4.2	6	0.00	6.0E-01	included
SLMO1	44689	ES	2.2	1	3.2	0.01	6.0E-01	included
ZDHHC20	25455	ES	12	11	13.1	-0.01	6.0E-01	excluded
HACL1	63585	ES	12	11	13	0.00	6.0E-01	excluded
NR3C2	70803	ES	6:07	5	8	0.00	6.0E-01	included
LMBR1L	21532	ES	3:05	1	6.1	0.00	6.0E-01	excluded
MCFD2	53479	ES	4	3	6	0.00	6.0E-01	excluded
GEMIN7	50396	ES	2	1.2	3	0.01	6.0E-01	included
PAPLN	28279	ES	21.2	20	22	0.00	6.0E-01	included
HKR1	49495	ES	8.1:8.2:11	7	12	-0.01	6.0E-01	excluded
FYN	77277	ES	4	2	6	0.00	6.0E-01	included
GLT8D1	65251	ES	2	1.3	4	-0.01	6.0E-01	excluded
TCEA1	83859	ES	2	1	4.1	0.00	6.0E-01	excluded
C14orf159	28867	ES	7	1	8	0.00	6.0E-01	excluded
RBM6	64941	ES	3.1:3.2:4:5:6:7	2	8	0.00	6.0E-01	excluded
ZNF341	59014	ES	11	10	12	0.00	6.0E-01	excluded
SLC6A13	19595	ES	2.1	1	3	0.00	6.0E-01	excluded
ZNF91	48817	ES	3	2	4	0.00	6.0E-01	included
ZNF286A	39382	ES	1.3:1.4	1.1	2	0.01	6.0E-01	included
OSCP1	1780	ES	2	1	4	0.00	6.0E-01	included
CAPRIN2	20944	ES	13	12	14	0.00	6.0E-01	excluded
DHPS	47830	ES	08:09.1	7	9.2	0.00	6.0E-01	excluded

DCP1A	65309	ES	6	5	7	0.00	6.0E-01	included
YAF2	21140	ES	5.1:5.2:7:8	2	9.1	0.00	6.0E-01	included
NAE1	36860	ES	2.1:3.1:4:5:6	1	7	0.00	6.0E-01	excluded
C19orf82	47382	ES	3	1	4.1	-0.01	6.0E-01	excluded
GNE	86342	ES	5	4	6	0.00	6.0E-01	excluded
TM2D2	83475	ES	1.2:2.1	1.1	2.2	0.00	6.0E-01	included
REEP5	72997	ES	3	2	5	0.00	6.0E-01	excluded
PI4KB	7593	ES	2:3:4:5	1	6	0.00	6.0E-01	excluded
ZNF846	47403	ES	5	4.2	6.1	0.01	6.0E-01	included
FOXP1	65608	ES	7:08	6	10	0.01	6.0E-01	included
TRPM4	50904	ES	3:04	2	5	0.00	6.0E-01	excluded
TMEM25	19020	ES	3.2:3.3	2.3	4	0.00	6.0E-01	included
RBM42	49223	ES	5:6.1:6.2	4	6.3	0.00	6.0E-01	included
RHOT1	40183	ES	19.3:20:21	19.1	22	0.00	6.0E-01	excluded
AK3	85777	ES	3	2	4	0.00	6.0E-01	included
BSDC1	1595	ES	4.2:4.3:5	3	6.1	0.00	6.0E-01	included
NRP1	11203	ES	5	4	6	0.00	6.1E-01	excluded
APPBP2	42860	ES	2	1	4	0.00	6.1E-01	included
IL32	33386	ES	1.6:1.8	1.5	1.9	0.00	6.1E-01	excluded
BCAS3	42885	ES	3:06	2	7	0.00	6.1E-01	excluded
CHD7	83952	ES	3.2:4:5:6:7:8:9:10:11:<br>12:13.1:14:15:16:17:1<br>8:19:20:21:22:23:24:2<br>5:26:27:28:29:30:31:3	3.1	36.2	0.00	6.1E-01	excluded
CYB561A3	16170	ES	5.1:5.2:6.1	4.2	6.3	0.00	6.1E-01	included
GTPBP10	80395	ES	4	3	5	0.00	6.1E-01	excluded
GGT2	121589	ES	5:06	4	7	-0.01	6.1E-01	excluded
SMIM7	48193	ES	05:06.1	2	7.1	0.00	6.1E-01	excluded
PMM2	33940	ES	6:7:8:9.1	3	9.2	0.00	6.1E-01	included
LTA4H	23823	ES	18.2	17	19	0.00	6.1E-01	excluded
RANBP3	46999	ES	4.1:5:7:8	1	10.2	0.01	6.1E-01	included
TRAFD1	24579	ES	5	4	6.1	-0.01	6.1E-01	excluded
IFNAR2	60394	ES	2.1:2.2:3	1	4	0.00	6.1E-01	excluded
PTPRK	77497	ES	19:20	18	21	0.00	6.1E-01	excluded
ACADM	3487	ES	12	11	13.1	0.00	6.1E-01	included
FAM195A	32926	ES	4	2	5	0.00	6.1E-01	excluded
PREPL	53436	ES	9	8	10	0.00	6.1E-01	included
RPS15A	34257	ES	3.1:3.2:3.3:4	2.2	5.1	0.00	6.1E-01	excluded
GIT2	24376	ES	17.2:18.1:18.2	17.1	19	0.00	6.1E-01	included
RANBP3	47004	ES	2:4.1:7:8	1	10.2	0.00	6.1E-01	excluded
RPS6KA1	1292	ES	2	1	4	0.00	6.1E-01	included
ASS1	87901	ES	3	1	4	0.00	6.1E-01	excluded
TMUB2	41797	ES	4.4:4.5:4.7:4.8	4.3	5	0.00	6.1E-01	excluded
DMKN	49152	ES	15:16	13	18.1	0.00	6.1E-01	included
TAX1BP1	79063	ES	16:01.1	14	18	0.00	6.1E-01	excluded
IDNK	86686	ES	6	5	7	0.00	6.1E-01	excluded
DHRS4L2	26804	ES	4:5:6:7	3	8.1	0.00	6.1E-01	excluded
SEPT2	58370	ES	4:07	2	9	-0.01	6.1E-01	excluded
IL32	33375	ES	3	2.4	4	0.00	6.1E-01	excluded
PASK	58327	ES	3:04:05	2	6	0.00	6.1E-01	excluded
PTP4A2	1535	ES	3:04:05	2	6	0.00	6.1E-01	excluded
NOMO3	34228	ES	6	5	7	0.00	6.1E-01	excluded
TANGO2	61125	ES	7.2	6	8	-0.01	6.1E-01	excluded
OS9	22711	ES	5.1:6:7.1:7.4:8:9.1	4	9.2	-0.01	6.1E-01	excluded
IRF5	81731	ES	9.1:9.2	8	10	0.00	6.1E-01	included
NT5C3B	40955	ES	1.2:2:3	1.1	4	0.00	6.1E-01	included
POGZ	7633	ES	4:05	3	6	0.00	6.1E-01	included
GUF1	69148	ES	2	1	3	-0.01	6.1E-01	excluded
GPATCH4	8296	ES	5.3:5.4	5.1	6	0.00	6.1E-01	excluded
AKTIP	36418	ES	2	1.1	3	0.00	6.1E-01	excluded
UEVLD	14671	ES	10	9	11	0.00	6.1E-01	excluded
ATG7	63384	ES	18	17	19	0.00	6.1E-01	included

SDHAF2	16228	ES	4.1	3	6	0.00	6.1E-01	excluded
RBM23	26654	ES	3:04	2	5	0.01	6.1E-01	included
NDEL1	39195	ES	4	3	5.1	0.00	6.1E-01	included
MDM2	23257	ES	3:5.1:5.2:6:7:8.1:8.2:9	2.1	10	0.00	6.1E-01	included
ARHGAP23	40576	ES	24	23	25	0.00	6.1E-01	excluded
PREPL	53441	ES	2.3	1.3	3	-0.01	6.1E-01	excluded
BRD8	73514	ES	3	2.1	4	0.00	6.1E-01	excluded
ARFGAP2	15654	ES	4.2:5:6.2:7	3	8	0.00	6.1E-01	included
ARHGAP21	11012	ES	25	24	26	0.00	6.1E-01	excluded
INPP5K	38317	ES	5.1:5.2	4	7	0.00	6.1E-01	included
MACF1	1886	ES	69	68	70	0.00	6.1E-01	included
RCBTB2	25863	ES	07:08.1	6	8.2	0.00	6.1E-01	included
YAF2	21209	ES	2	1	9.1	0.00	6.1E-01	included
GNE	86341	ES	10	9	11	0.00	6.1E-01	excluded
B4GALT4	66291	ES	4	3.2	5	0.00	6.1E-01	included
UGDH	69071	ES	4	2	5.1	0.00	6.1E-01	included
C19orf44	48173	ES	6	5	7.1	-0.01	6.1E-01	excluded
TCP1	78321	ES	4.2	3.2	5	0.00	6.1E-01	excluded
DOPEY1	76854	ES	27	26	28	0.00	6.1E-01	included
RANBP3	47006	ES	3	1	4.1	0.00	6.1E-01	excluded
COBLL1	55789	ES	8	7	9	0.00	6.1E-01	included
ZWILCH	31285	ES	3	2.2	4	0.00	6.1E-01	included
CHURC1	27911	ES	3.1:3.2	2	4	-0.01	6.1E-01	excluded
MAL	54483	ES	2	1	3	0.00	6.1E-01	included
KLC4	237505	ES	19	18	20	0.00	6.1E-01	excluded
GNB2	80996	ES	3.2:4.1	1	4.2	0.00	6.1E-01	excluded
FAM21A	11563	ES	27	26	29	0.01	6.1E-01	included
TATDN1	85094	ES	2:03	1.1	4.1	0.00	6.1E-01	included
PIGG	68368	ES	4:06	2	7	0.00	6.1E-01	included
TVP23B	39655	ES	5.1:5.2	4	6	0.00	6.1E-01	excluded
DIAPH1	73802	ES	18	17.1	20	0.00	6.1E-01	included
SGOL1	63702	ES	6.1:6.2	5	7	0.01	6.1E-01	included
CABLES1	44794	ES	7:08:09	6	10	0.00	6.1E-01	excluded
GLYCTK	65203	ES	5.2:6.1:6.2	4	6.3	0.00	6.1E-01	included
EZH1	41113	ES	5:06	4.2	7	0.00	6.1E-01	included
B4GALT4	66289	ES	6.2:7:8.1:8.2:9.1:9.3	6.1	9.4	0.00	6.1E-01	included
ARHGEF1	50100	ES	29	28	30	0.00	6.1E-01	excluded
ZNF286A	39379	ES	3	2	4	0.00	6.1E-01	included
IFI27L1	29065	ES	5	4.3	6	0.00	6.1E-01	included
CXADR	60228	ES	5	4	6	0.00	6.1E-01	included
AASS	81602	ES	21	19	22	0.00	6.1E-01	excluded
FARSA	47890	ES	6	5	7.1	0.00	6.1E-01	included
TSPAN17	74677	ES	2:03	1.1	4.1	0.00	6.1E-01	excluded
CEP250	59168	ES	15	14	16	0.00	6.1E-01	included
BTG3	60238	ES	4	3	5	0.00	6.1E-01	excluded
HSPA9	73558	ES	2.2	1	3	0.00	6.1E-01	included
ANKZF1	57651	ES	2:03:04	1.1	5	0.00	6.1E-01	included
TSPAN14	12375	ES	3:04:05	1	6	0.00	6.1E-01	excluded
MLF1	67441	ES	4.2	1.1	5	0.00	6.1E-01	included
LPIN1	52711	ES	18	17	19	0.00	6.2E-01	excluded
SVIL	11114	ES	16	15	17	0.00	6.2E-01	included
TIMM9	27711	ES	2	1	4.2	0.00	6.2E-01	included
C5orf45	74979	ES	4	2.1	6	-0.01	6.2E-01	excluded
UMPS	66536	ES	3	1	4.1	0.00	6.2E-01	excluded
ACYP2	53570	ES	12	10	13	0.00	6.2E-01	included
PRPF6	60185	ES	15	14	16	0.00	6.2E-01	included
CCDC90B	18081	ES	2	1.2	4.2	0.00	6.2E-01	included
DPP8	31170	ES	19	17	20	0.00	6.2E-01	included
CD53	4125	ES	5:06	4	7	0.00	6.2E-01	included
SKP1	73360	ES	5	4	7	0.00	6.2E-01	included
UBE2C	59604	ES	2	1.4	3.2	0.00	6.2E-01	included
CDK10	38121	ES	4	2.2	5	-0.01	6.2E-01	excluded

P2RX4	24844	ES	2	1	3	0.00	6.2E-01	excluded
RPS15A	34260	ES	3.1:3.2:3.3	2.2	5.1	-0.01	6.2E-01	excluded
SDCBP	83930	ES	6.2	5	7.1	0.00	6.2E-01	included
PHF23	38858	ES	3	1	4	0.00	6.2E-01	included
NPTN	31604	ES	2:03:04	1	5	-0.02	6.2E-01	excluded
MUC1	7965	ES	4.1:4.2:4.3:5:6.1:6.2	3.4	7	0.00	6.2E-01	excluded
HSPA8	19197	ES	8.2:9:10.1	8.1	10.2	0.00	6.2E-01	excluded
HPCAL1	52658	ES	8.2:9	8.1	10	0.00	6.2E-01	included
ERBB3	22361	ES	1.2:4:5.1:6:7:8:9:10:1<br>1:12:13:14:15:16:17.1	1.1	17.2	0.00	6.2E-01	included
POSTN	25677	ES	17	16	18	0.01	6.2E-01	included
TRPM4	50903	ES	4	3	5	0.00	6.2E-01	included
CBWD3	86511	ES	13	12	14	0.00	6.2E-01	excluded
AKIP1	14278	ES	4.1	3	5	0.00	6.2E-01	excluded
PDE9A	60739	ES	2	1	3	0.00	6.2E-01	excluded
FAM219B	31797	ES	4	2	5.1	0.00	6.2E-01	included
ZNF302	48987	ES	4:5.2:6.1	2	6.2	0.00	6.2E-01	included
H2AFY	73451	ES	4:5:6.2:6.3:8	3	9	0.00	6.2E-01	excluded
SOS2	27454	ES	9	8	10	0.00	6.2E-01	included
PDSS2	77131	ES	6:07	5	8	0.00	6.2E-01	included
EFCAB14	2825	ES	7	6	8	0.01	6.2E-01	included
ATP13A4	68137	ES	6:07	5	8	0.00	6.2E-01	included
TMEM175	68434	ES	03:04.2	1	5.1	0.00	6.2E-01	excluded
MYL5	68386	ES	1.3:1.4:2	1.2	4.1	0.00	6.2E-01	excluded
TCERG1	73913	ES	6	5	7	-0.01	6.2E-01	excluded
RAD51D	40247	ES	10	9	11	0.00	6.2E-01	included
ANXA11	12347	ES	3.2:5.1:5.2	1.1	5.3	0.00	6.2E-01	included
CEP250	59167	ES	21	20	22	0.00	6.2E-01	excluded
RGPD8	54990	ES	7:08	6	9	0.00	6.2E-01	excluded
ZNF143	14326	ES	4	3	6	0.00	6.2E-01	included
LRRFIP1	58142	ES	4:5:6:7	3	13	0.00	6.2E-01	included
ARHGEF4	55361	ES	6:7:8:9:10:11:12:13:1	4.1	14.3	0.00	6.2E-01	excluded
RSRC1	67425	ES	5	4	7	0.00	6.2E-01	included
SLC31A2	87258	ES	3	2	4	0.00	6.2E-01	included
SGCE	80498	ES	12	11	13.1	0.00	6.2E-01	excluded
ARF3	21501	ES	4	3	5	0.00	6.2E-01	included
MICAL3	61018	ES	8	7	9	0.00	6.2E-01	excluded
NEIL2	82629	ES	3	2.1	4	0.00	6.2E-01	excluded
ATG4A	89853	ES	4	2	5	0.00	6.2E-01	included
ANXA5	70488	ES	4:05:06	3	7.1	0.00	6.2E-01	included
ELP2	45223	ES	07:08.1	4.2	9	0.00	6.2E-01	excluded
TIPIN	31266	ES	4	3	5	0.00	6.2E-01	excluded
MRPL55	10151	ES	2.2:2.4:2.5:2.6	1.1	2.9	0.00	6.2E-01	included
SFTA3	27260	ES	3:4.2:4.3	2	5	0.00	6.2E-01	included
WDYHV1	85064	ES	6	5	7	0.00	6.2E-01	included
TSTD2	87016	ES	5:06	4	7	0.00	6.2E-01	included
EIF3J	30342	ES	6	5	7	0.00	6.2E-01	included
BSDC1	1596	ES	4.3:5	3	6.1	0.00	6.2E-01	included
SMPD4	55295	ES	11:12:14	10	15.1	-0.01	6.2E-01	excluded
TNFRSF1A	19835	ES	2	1	3.1	0.00	6.2E-01	included
SNX17	52993	ES	4	3	5	0.00	6.2E-01	included
SLC47A1	39747	ES	4	3	5	0.00	6.2E-01	excluded
NFS1	59217	ES	7:08	5	9	0.00	6.2E-01	included
UCHL5	9249	ES	4	3	5	0.00	6.2E-01	included
RAD52	19649	ES	4:05	2.2	6.1	-0.01	6.2E-01	excluded
RBM10	88906	ES	4	3	5	-0.01	6.2E-01	excluded
CASC1	20798	ES	4:06	1.1	7	0.00	6.2E-01	included
ADD3	13077	ES	15	14	16	0.00	6.2E-01	included
LRP5	17304	ES	7	6	8	0.00	6.2E-01	included
PSMD3	40816	ES	11	10	12	0.00	6.2E-01	excluded
IRF3	51010	ES	1.4:1.5:2	1.1	3	-0.01	6.2E-01	excluded
NUP160	15811	ES	3.1:3.3	2	4	0.00	6.2E-01	included

MKNK1	2813	ES	6	5	7.1	0.00	6.2E-01	excluded
ESRP1	84567	ES	14:15	13	16	0.00	6.2E-01	excluded
TG	319510	ES	25:26:27:28:29:31:32:<br>33:34:35:37:38:39:40:	24	46	0.00	6.2E-01	included
SLC12A4	37162	ES	4	1	5	0.00	6.2E-01	included
B3GAT3	16373	ES	2	1	3	0.00	6.2E-01	included
SELENBP1	7619	ES	7	5	8	0.00	6.2E-01	excluded
RAMP2	41123	ES	4.2	3	5	0.00	6.2E-01	excluded
ENTPD1	12655	ES	5:06	4	7	0.00	6.2E-01	excluded
ZNF222	50267	ES	4	3	6	0.00	6.2E-01	included
ANKS3	33835	ES	3	1.1	4	0.00	6.2E-01	excluded
CLEC16A	34005	ES	24	23	25	0.01	6.2E-01	included
IDH2	32465	ES	4:05	3	6	0.00	6.2E-01	excluded
NUDC	262810	ES	3:04	2	7	-0.01	6.2E-01	excluded
NDEL1	39191	ES	10	9	12.1	0.00	6.2E-01	excluded
SRP68	43550	ES	3	2.2	4	0.00	6.2E-01	included
MTX2	56122	ES	2:3:4:6	1	7	0.00	6.2E-01	included
NR4A1	21892	ES	4.2:5.2	4.1	6.1	0.01	6.2E-01	included
CD44	15212	ES	3.2:4:5:12.1:13:14:15:	3.1	16.2	0.00	6.2E-01	included
EXOSC10	643	ES	18	17	19	0.00	6.2E-01	included
NHEJ1	57591	ES	6	5	7	0.00	6.2E-01	excluded
SH3D21	1769	ES	5	4.2	6	0.00	6.2E-01	included
NSUN5	270163	ES	02:03.2	1	5	0.00	6.2E-01	excluded
ASTE1	66775	ES	5	4.1	6	0.01	6.2E-01	included
RPS3	17842	ES	4.2:4.3:5:6.1	3.2	6.2	0.00	6.2E-01	included
PMM2	33927	ES	6:07	3	9.1	0.00	6.2E-01	excluded
MRPL55	10118	ES	2.2:2.4:2.5:2.6	1.2	2.9	0.00	6.3E-01	included
SYT11	8129	ES	2	1	3	0.00	6.3E-01	included
EMP1	20539	ES	4.1:4.3:4.4	3	4.5	0.00	6.3E-01	included
RIC3	14224	ES	05:07.2	4	8	0.00	6.3E-01	included
ACLY	40961	ES	4:5:6:7:8:9:10	3	11	0.00	6.3E-01	excluded
STARD8	89356	ES	5	4	6	0.00	6.3E-01	excluded
MAPKBP1	30114	ES	3	2	4	0.00	6.3E-01	included
VPS33B	32533	ES	3	2	4	0.00	6.3E-01	included
ZNF329	52415	ES	5	4	6.1	-0.01	6.3E-01	excluded
CCDC117	61556	ES	3	2	4	0.00	6.3E-01	included
CCNC	77079	ES	10	9.2	11	0.00	6.3E-01	excluded
NCOA1	52830	ES	7	6	8	0.00	6.3E-01	included
DPY19L4	84572	ES	3	2	4	0.00	6.3E-01	excluded
ALAD	87280	ES	4	3	5	0.00	6.3E-01	included
GSTK1	82079	ES	6	5	7.1	0.00	6.3E-01	excluded
TARS	71675	ES	5	4	6.1	0.00	6.3E-01	excluded
GEMIN7	50397	ES	1.2:2	1.1	3	-0.01	6.3E-01	excluded
TRAPPC10	60803	ES	8	6	9	-0.01	6.3E-01	excluded
VAPB	59959	ES	3:04:05	2	6	0.00	6.3E-01	excluded
FAM60A	20986	ES	3:4:5:6	1	7	0.00	6.3E-01	excluded
COPS7B	57953	ES	4.1:4.2:4.3:4.4:5	3	6	0.00	6.3E-01	included
SFXN2	12979	ES	2.2	1	4.1	-0.01	6.3E-01	excluded
FAXDC2	74238	ES	5.2:6	4	7	0.00	6.3E-01	included
FAM173B	71548	ES	4	3.1	5	0.00	6.3E-01	excluded
CLEC2D	20263	ES	7.1	6.1	8	-0.01	6.3E-01	excluded
LRRC28	32650	ES	5:6:7.2:8:10:11	3	12	0.01	6.3E-01	included
UBL7	31723	ES	1.2:1.4	1.1	3	0.00	6.3E-01	excluded
SNTB2	37278	ES	3	2	4	0.00	6.3E-01	included
SAP30BP	43478	ES	12	11	13	0.00	6.3E-01	included
ERBB2	40686	ES	8.1:8.2:9:10:11:12:13	6	14	0.00	6.3E-01	included
GATAD2A	48637	ES	7	6.2	8	0.00	6.3E-01	included
CCDC88A	53617	ES	26	25	27	0.01	6.3E-01	included
ZNF410	28336	ES	5	4.2	6.1	0.00	6.3E-01	included
HBS1L	77811	ES	2:03	1	4	0.00	6.3E-01	excluded
NDRG1	85236	ES	5.2:6:7.1	5.1	7.2	0.00	6.3E-01	included
EHD2	50667	ES	2	1	3	0.00	6.3E-01	included

FYTTD1	68315	ES	6	5	7	0.00	6.3E-01	excluded
ZNF83	51514	ES	7	6.3	10.1	0.00	6.3E-01	excluded
ITM2A	89544	ES	2	1	3	0.00	6.3E-01	excluded
GPALPP1	25791	ES	10.1:10.2:10.3:10.4:10	9.1	11	0.00	6.3E-01	included
EIF1AD	16975	ES	1.2:2.3	1.1	2.4	-0.01	6.3E-01	excluded
CALCOCO2	42235	ES	2	1	6	0.00	6.3E-01	included
WDR45	89074	ES	11.1:11.2:12	10	13	0.00	6.3E-01	excluded
ZNF544	52430	ES	7.2:9:10.1	6.2	10.2	0.01	6.3E-01	included
HBS1L	77793	ES	5.2	4	7.1	0.00	6.3E-01	excluded
CEP68	53784	ES	1.2:2	1.1	3.1	0.00	6.3E-01	included
NME6	64586	ES	6	4	7	0.00	6.3E-01	included
RBM23	26655	ES	3	2	5	0.00	6.3E-01	included
SPRTN	10278	ES	3	2	4.1	0.00	6.3E-01	excluded
CLCN3	71154	ES	9	8	10	0.00	6.3E-01	excluded
MDH1B	57154	ES	4.2:5:6	4.1	7	0.00	6.3E-01	excluded
BRPF3	75966	ES	7:08	6.2	9	0.00	6.3E-01	excluded
NME6	64604	ES	3.2	1.1	4	0.00	6.3E-01	excluded
GFM1	67447	ES	6	5	7.1	0.00	6.3E-01	excluded
AP3S2	32454	ES	4:05	3	6	0.00	6.3E-01	included
BSDC1	1601	ES	4.2:4.3	3	5	0.00	6.3E-01	excluded
SREBF2	62465	ES	10	9	11	0.00	6.3E-01	excluded
RNF41	22402	ES	8	7.1	9	0.00	6.3E-01	excluded
C1D	53821	ES	1.2:2.1:2.2	1.1	3.1	0.00	6.3E-01	included
CCDC149	68954	ES	7:8:9:10:11:12:13	6	14	0.00	6.3E-01	excluded
SPECC1L	61399	ES	2	1	3	0.01	6.3E-01	included
OSTC	70305	ES	4	3	5	0.00	6.3E-01	excluded
PPP3CB	12157	ES	5	4	6	0.00	6.3E-01	excluded
FGD4	21037	ES	7.1	6	8	0.00	6.3E-01	included
TAZ	90595	ES	5	4	6	-0.01	6.3E-01	excluded
MRPL55	10113	ES	2.4:2.5:2.6:2.8	1.2	2.9	0.00	6.3E-01	excluded
ARFIP2	14135	ES	6	5.2	7	0.00	6.3E-01	included
ITGA5	22183	ES	2	1	3	0.00	6.3E-01	included
TRAP1	33623	ES	5	4	6.1	0.00	6.3E-01	included
PGAP2	14008	ES	8:09	6	10	0.00	6.3E-01	excluded
DEK	75484	ES	3.2:4.1	3.1	4.2	0.00	6.3E-01	excluded
RBM47	69093	ES	3.2:6:7.1	3.1	7.2	0.00	6.3E-01	excluded
H2AFY	73446	ES	7	6.3	9	0.00	6.3E-01	excluded
SESN3	18375	ES	5	4	6	0.00	6.3E-01	excluded
DGKZ	15554	ES	7:8.1:8.2:9.1:9.2	3	10.2	0.00	6.3E-01	included
TXNL4A	46283	ES	06:07.2	3	9	-0.01	6.3E-01	excluded
CLDN7	38897	ES	4	3	5	0.00	6.3E-01	included
SGSM3	62347	ES	3	2	4	0.00	6.3E-01	included
OGDH	79545	ES	8	7	9	0.00	6.3E-01	excluded
TRAPPC6A	50409	ES	2	1.2	3	0.00	6.3E-01	included
USP19	64837	ES	6.2	5.2	7	-0.01	6.3E-01	excluded
SSR2	8163	ES	4.1:4.2:4.3	2	7	-0.01	6.3E-01	excluded
CLASP1	55172	ES	29	28.1	31.1	-0.01	6.3E-01	excluded
NBAS	52721	ES	24:25:00	23	26	0.00	6.3E-01	excluded
NAA25	24572	ES	17	16	18	-0.01	6.3E-01	excluded
CMC2	37714	ES	9	5	10	0.00	6.3E-01	included
DEK	75485	ES	3.1:3.2	2	4.1	0.00	6.3E-01	included
NF2	61629	ES	15	14	17	0.00	6.3E-01	excluded
ABCB8	82314	ES	2.2:2.3	1	5	0.00	6.3E-01	included
AP3D1	46578	ES	23:24.1:24.2	22	25	0.00	6.3E-01	excluded
GRN	41850	ES	6.1:6.2:7:8:9	5	10	0.00	6.3E-01	included
TLE2	46645	ES	10.5:11:12:13:14:15:1<br>6.1:16.2:17:18:19:20:	10.4	23.2	0.00	6.3E-01	included
RNF216	78677	ES	4:5.1:5.2:6.1:6.2	2	7	0.00	6.3E-01	excluded
EXD2	28134	ES	3:04	1	6	0.00	6.3E-01	included
USP48	998	ES	23	22	24	0.00	6.3E-01	excluded
BCKDHA	50065	ES	2.2:3.1	2.1	3.2	0.00	6.3E-01	excluded
TMEM107	39132	ES	2:3.1:3.2:3.3:3.4	1	3.7	0.00	6.3E-01	included

GUSB	79861	ES	3.1:3.2:4.1	2	5.1	0.00	6.3E-01	included
RAB6A	17708	ES	6	4	7	0.00	6.3E-01	excluded
C17orf89	44113	ES	1.2:2.1	1.1	2.2	0.00	6.3E-01	included
XPC	63522	ES	4.1:4.2	3.2	5	0.00	6.4E-01	included
SLMO2	60015	ES	3.2	2	4	0.00	6.4E-01	included
MED24	40832	ES	5	4	6	0.00	6.4E-01	included
DCTD	96839	ES	2.1:2.2	1.1	5	0.01	6.4E-01	included
ATG9A	57641	ES	3.1:3.2:4	1	5	0.00	6.4E-01	excluded
NRBP1	53009	ES	9	8	10	0.00	6.4E-01	excluded
EIF1AD	16966	ES	1.2:2.1:2.2:2.3:2.4	1.1	3.1	0.00	6.4E-01	included
WHSC1	68533	ES	13.3	13.1	14	0.00	6.4E-01	included
UVRAG	17886	ES	16	15	17	0.00	6.4E-01	excluded
SEPT6	89978	ES	9:10	8	11.1	0.00	6.4E-01	included
PTER	10877	ES	2	1	3.2	-0.01	6.4E-01	excluded
GFPT1	53846	ES	9	8	10	0.00	6.4E-01	excluded
RCN2	31957	ES	2:03:04	1	5	0.00	6.4E-01	included
TTC8	28741	ES	12	11	14	0.00	6.4E-01	excluded
LIN54	69756	ES	4:5.1:5.2	3.2	6	-0.01	6.4E-01	excluded
POLK	72532	ES	10:11:12:13.1:14.1:14.	9	15	0.00	6.4E-01	excluded
TMCO4	917	ES	3	1	4	-0.01	6.4E-01	excluded
HHAT	9699	ES	4	2	5	0.00	6.4E-01	included
NME5	121823	ES	5	4	6	0.00	6.4E-01	excluded
ZYG11A	3016	ES	3	2	4	-0.01	6.4E-01	excluded
MFGE8	32406	ES	3	1	4	0.00	6.4E-01	included
SYTL3	78285	ES	8:09:12	7	13	0.00	6.4E-01	excluded
SFTA3	27267	ES	4.1:4.2:4.3:5	2	6	0.00	6.4E-01	excluded
MUC1	7976	ES	2.3:3.1:3.2:3.3:3.4:4.1:<br>4.2:4.3:5:6.1	2.2	6.2	0.00	6.4E-01	included
IL32	33444	ES	1.4	1.1	1.9	0.00	6.4E-01	included
LRRC61	82221	ES	3	1	4	0.00	6.4E-01	excluded
KAT7	42323	ES	5	4	6	0.00	6.4E-01	included
SLC35A2	89039	ES	2	1.2	3	0.00	6.4E-01	excluded
SF3A1	61729	ES	2:3.1:3.2:4.1:4.2:5:6:7<br>:8:9:10.1	1	10.2	0.00	6.4E-01	included
CDH3	37244	ES	2	1	3	0.00	6.4E-01	excluded
LIMK2	61839	ES	2	1	4	0.00	6.4E-01	excluded
ANKS3	33807	ES	5.1:5.2	4	6	0.00	6.4E-01	excluded
FMO5	7371	ES	7.1:7.2	6	8	-0.01	6.4E-01	excluded
PTPRM	44569	ES	19:21	18	22	0.00	6.4E-01	included
TCAIM	64361	ES	8	7.2	9	-0.01	6.4E-01	excluded
LIPT1	54681	ES	3	1	6	-0.01	6.4E-01	excluded
SIRT1	11910	ES	4	3	5	0.00	6.4E-01	excluded
SSBP2	72674	ES	6	5	7	0.00	6.4E-01	included
UBE2Z	42243	ES	3	2.2	4	0.00	6.4E-01	included
KIAA0195	43449	ES	4:5:6:8:9:10:11:12	2	13	0.00	6.4E-01	excluded
SELP	8933	ES	7:08	6	9.1	0.00	6.4E-01	excluded
HHLA3	3406	ES	2.1:2.2	1.1	3	-0.01	6.4E-01	excluded
SEPT9	43740	ES	6.2:10.1	6.1	10.2	0.00	6.4E-01	excluded
TAF1C	37818	ES	10.2:11:12:13.1:13.2:1	10.1	14.2	0.00	6.4E-01	excluded
ARL16	44149	ES	4	3	5	-0.01	6.4E-01	excluded
GCA	55772	ES	7:8:9:10.1	6	10.2	0.00	6.4E-01	excluded
FAM193B	74802	ES	8	6	9	-0.01	6.4E-01	excluded
MED15	61170	ES	8	7	9	0.00	6.4E-01	excluded
AMN1	21013	ES	2:03:05	1	6	-0.01	6.4E-01	excluded
SPRYD7	25916	ES	2	1	3	0.00	6.4E-01	excluded
HKR1	49486	ES	14:17.1	13	17.2	0.00	6.4E-01	included
RBPJ	68987	ES	2.2:2.3:4.2	1	5	0.00	6.4E-01	included
CDK20	86782	ES	5	4	6	0.00	6.4E-01	included
SUMF2	79792	ES	5.1:5.2:6	4	7	0.00	6.4E-01	included
NDUFAF6	84601	ES	4:05	3	6	0.00	6.4E-01	included
FRG1B	58884	ES	5	3	6	0.01	6.4E-01	included
TMEM44	68162	ES	14.2	13.2	15	0.00	6.4E-01	excluded

ZNF608	73153	ES	4	3	5	0.00	6.4E-01	excluded
HESX1	65373	ES	3	2	4	0.00	6.4E-01	excluded
NUDT7	37665	ES	3.1:4	2	5	0.01	6.4E-01	included
MSTO1	91198	ES	9	8	10	0.00	6.4E-01	included
PRPSAP2	39669	ES	7	5	8	0.00	6.4E-01	included
KIAA1522	1634	ES	7:08	6	9	0.00	6.4E-01	included
TMEM25	19016	ES	4	3.3	5	0.00	6.4E-01	included
ABCC1	34212	ES	18	17	19	0.00	6.4E-01	included
KCNC3	51174	ES	5	4	6.1	0.00	6.4E-01	excluded
PRIMPOL	71307	ES	5	4	6	0.00	6.4E-01	included
SEC24C	12179	ES	4	3	5	0.00	6.4E-01	excluded
TMEM220	39285	ES	5	4	6	0.00	6.4E-01	included
PRMT7	37226	ES	3	1.1	4.2	-0.01	6.4E-01	excluded
MCM5	61972	ES	5	4	6	0.00	6.4E-01	included
NOSTRIN	55840	ES	6	5	8	0.00	6.4E-01	excluded
HPS4	61507	ES	10.3	10.1	10.5	0.00	6.4E-01	included
LDHA	14622	ES	5:6.1:6.2	4	7	-0.01	6.4E-01	excluded
EIF2AK1	78710	ES	4	3	5	0.00	6.4E-01	included
EIF3K	49680	ES	06:07.1	4	8	0.00	6.4E-01	excluded
POLR2B	69389	ES	5	4	6	0.00	6.4E-01	included
TMPRSS13	18951	ES	6	5	7.1	0.00	6.4E-01	excluded
TAF1D	18321	ES	05:06.1	4	7	0.00	6.4E-01	included
PKM	31512	ES	6	5.2	7	0.00	6.4E-01	excluded
NUP85	43385	ES	8	7	9	0.00	6.4E-01	excluded
MCPH1	82576	ES	6	5	7	0.00	6.4E-01	included
IL15RA	10681	ES	3:04	2	5	0.01	6.4E-01	included
TGFB1I1	36269	ES	7	6	8	0.00	6.4E-01	included
BRF2	83358	ES	5	4	6	0.00	6.4E-01	excluded
CEP70	67002	ES	3.2:3.3:4:5:6	2	7.1	0.00	6.4E-01	included
FOCAD	85988	ES	19	18	20	0.00	6.4E-01	excluded
SLC25A20	64782	ES	4:05	3	6	0.00	6.4E-01	excluded
ZCCHC17	1465	ES	2	1.1	4	0.00	6.4E-01	included
ADHFE1	84007	ES	7	6	8.1	0.01	6.4E-01	included
YWHAE	38298	ES	3:06	1	7	0.01	6.4E-01	included
TMX2	15912	ES	3.2:3.3:4	2	5.1	0.00	6.4E-01	included
SCNN1B	35580	ES	9	8	10	0.00	6.4E-01	included
IFI27L1	29062	ES	06:07.1	4.3	8	0.00	6.4E-01	excluded
LCMT1	35670	ES	2	1	3.1	0.00	6.4E-01	excluded
ADPGK	31588	ES	1.3:2:3	1.2	4	0.00	6.4E-01	excluded
ENDOV	44063	ES	05:06.2	2.4	7	0.01	6.4E-01	included
EXOC7	43565	ES	8.1:8.2	7	9	-0.01	6.4E-01	excluded
GEMIN6	53289	ES	5.1:5.2	4	6	0.00	6.4E-01	excluded
FUZ	51080	ES	2.3:3	2.2	4	0.00	6.4E-01	included
APH1B	31025	ES	2	1	3	0.00	6.4E-01	included
UBE2F	58192	ES	2	1	5	0.00	6.5E-01	excluded
ATG12	73034	ES	2:03	1.1	4	0.00	6.5E-01	excluded
SLC46A1	39899	ES	3	2	4.1	0.00	6.5E-01	excluded
SMG5	8216	ES	10:11:12:13:14:15:16:17:18:19:20:21:22.1	9	22.2	0.00	6.5E-01	included
PSMD3	40817	ES	1.2:2.1	1.1	2.2	0.00	6.5E-01	included
SDHA	71421	ES	2	1	3	0.00	6.5E-01	included
ZFAND4	11371	ES	8	7	9.1	0.00	6.5E-01	excluded
LYRM7	73220	ES	4	3	5	0.00	6.5E-01	excluded
PDCD10	67561	ES	1.2:3.1:3.2:3.3	1.1	5	0.00	6.5E-01	included
NDRG2	26506	ES	4.2:4.3:4.5:4.6	4.1	5.2	0.00	6.5E-01	excluded
ENDOV	44071	ES	2.3:2.4:5:6.2	2.2	7	-0.01	6.5E-01	excluded
GPATCH1	48927	ES	4:05	3	6	0.00	6.5E-01	included
UQCRB	84616	ES	2	1	3	0.00	6.5E-01	excluded
MED31	38771	ES	4	3	5	0.00	6.5E-01	included
TPO	52538	ES	15	14	16	0.00	6.5E-01	excluded
LARP1B	70568	ES	19	18	20	0.00	6.5E-01	included
PRKD2	50582	ES	1.2:2.2	1.1	2.3	0.00	6.5E-01	excluded

GPR56	36581	ES	8.3:8.4:9	8.2	10	0.00	6.5E-01	included
RFWD2	9058	ES	5	4	6.1	0.00	6.5E-01	included
DPP8	31173	ES	17:18	16	19	0.01	6.5E-01	included
ARL13B	65686	ES	04:05.1	2	6	0.00	6.5E-01	included
NBPF10	7268	ES	6:7:8:9	4.2	10	0.01	6.5E-01	included
FLNB	65414	ES	41	40	42	0.00	6.5E-01	included
SIMC1	74638	ES	2:03:04	1	5	0.00	6.5E-01	excluded
IRF5	81732	ES	9.2	8	10	0.00	6.5E-01	included
KLHL32	77032	ES	6:07	5	8	0.00	6.5E-01	excluded
EPB41	1403	ES	20	19.1	21	0.00	6.5E-01	included
MPPE1	44650	ES	10	8	11	-0.01	6.5E-01	excluded
LZTR1	61207	ES	4	3	5	0.00	6.5E-01	excluded
SF3A1	61730	ES	2:3.1:4.2:5:6:7:8:9:10.	1	10.2	-0.01	6.5E-01	excluded
SYNE2	27849	ES	111	109	112	0.00	6.5E-01	excluded
FAM3A	90632	ES	5.2:5.3	4	6	0.00	6.5E-01	excluded
SRI	80359	ES	5	4	6	0.00	6.5E-01	excluded
C17orf72	43035	ES	2.1:2.2	1	3	0.00	6.5E-01	excluded
ZMAT5	61664	ES	2	1	3	0.00	6.5E-01	excluded
SGCE	80507	ES	2:03	1	4	0.00	6.5E-01	excluded
RNF138	45021	ES	3:04	2.2	5	0.00	6.5E-01	excluded
MEAF6	1803	ES	6:07	5	8.1	0.00	6.5E-01	excluded
SNRK	64341	ES	2:03:04	1	5	0.01	6.5E-01	included
DCTN5	35624	ES	4:05	2	6	0.00	6.5E-01	excluded
PPP6R3	17321	ES	11	10	12	0.00	6.5E-01	included
ATP7A	89538	ES	3:4:5:6:7:8:9:10:11:12<br>:13:14:15	2	16	0.00	6.5E-01	excluded
PQLC2	899	ES	2	1.3	3	0.00	6.5E-01	excluded
PDE4C	48410	ES	17	16	18	0.00	6.5E-01	excluded
YTHDF3	83979	ES	3	2	4.1	0.00	6.5E-01	included
MORF4L1	32133	ES	3.2:4:5	3.1	7	0.00	6.5E-01	included
KCNAB2	372	ES	13:14:15:17:18:19:21:<br>22.1:22.3	12	22.4	0.00	6.5E-01	excluded
ZNF568	49434	ES	3:04	2	5	0.00	6.5E-01	excluded
ZNF706	84740	ES	6	4	7	0.00	6.5E-01	included
DDX11	20976	ES	11.1	10	12.2	0.00	6.5E-01	excluded
YWHAE	38300	ES	3	1	7	-0.01	6.5E-01	excluded
ZBTB17	784	ES	3	2	4	0.00	6.5E-01	excluded
GRHL1	52649	ES	8	7	9	0.00	6.5E-01	included
SH2B1	35880	ES	3.3:3.6	3.2	4.1	0.00	6.5E-01	excluded
DNAL1	28306	ES	3:4:5:6	1	7	0.00	6.5E-01	excluded
HAUS2	30194	ES	3	2	5.1	0.00	6.5E-01	excluded
TRPC4	25686	ES	9.4:9.5:10.1:10.2:10.3	9.3	10.4	0.00	6.5E-01	included
ARF4	65385	ES	02:03.2	1	4	0.00	6.5E-01	included
ENO3	38621	ES	5:06	4	7	0.00	6.5E-01	included
EIF4G3	951	ES	23	22.1	24	0.00	6.5E-01	included
MOCS2	72002	ES	5	4	6	0.00	6.5E-01	included
SLMO2	60014	ES	3.1:3.2	2	4	-0.01	6.5E-01	excluded
GPR56	36589	ES	5.2	3.1	7.2	0.01	6.5E-01	included
MYO19	40491	ES	4:05:06	3	7	0.00	6.5E-01	excluded
ABI1	11043	ES	10:11.1	9	11.2	0.00	6.5E-01	included
RAD17	72362	ES	4	3.2	5	0.00	6.5E-01	excluded
TNFRSF1A	19831	ES	3.1:3.2	2	4.1	0.00	6.5E-01	excluded
CDH13	37785	ES	9	5.1	10	0.00	6.5E-01	included
TIMMDC1	66307	ES	3:04:05	2	6	0.00	6.5E-01	excluded
PSMD5	87397	ES	4	3	5	0.00	6.5E-01	excluded
ATXN3	28956	ES	3.1:3.2	2	4.2	0.00	6.5E-01	excluded
C1orf112	8956	ES	14	13	15.1	0.00	6.5E-01	excluded
DFFA	619	ES	4	3	5.1	0.00	6.5E-01	excluded
VKORC1L1	79852	ES	2	1	3	0.00	6.5E-01	included
ATP2A3	38514	ES	22	20	23.1	0.00	6.5E-01	excluded
SS18	44954	ES	6:09	3	11	0.01	6.5E-01	included
TCF25	38159	ES	18	17	19	0.00	6.5E-01	excluded

CTDSPL2	30341	ES	6	5	7	0.00	6.5E-01	included
CCNG1	74436	ES	6	5	7	0.00	6.5E-01	included
FGFR2	13316	ES	4:05	3	6	0.00	6.5E-01	included
PPP4C	94130	ES	5.1:5.2	4	6	-0.01	6.5E-01	excluded
TBC1D17	51117	ES	2	1	3	0.00	6.5E-01	excluded
YY1AP1	8107	ES	9	8	10.1	0.00	6.5E-01	excluded
STAT3	41038	ES	2.3	1	3	0.00	6.5E-01	included
HNRNPC	26553	ES	2.2:2.3:2.4:2.6	1	3.2	0.00	6.5E-01	excluded
GABPB1	30577	ES	3	1	4	0.00	6.5E-01	excluded
USMG5	13000	ES	3	2.2	4	0.00	6.5E-01	excluded
PNPO	42129	ES	5	4	6	0.00	6.5E-01	excluded
INPP1	56562	ES	2:03	1	4	0.00	6.5E-01	included
SMIM7	48199	ES	4	2	5	0.00	6.5E-01	included
POGZ	7634	ES	3	2	4	0.00	6.5E-01	included
CAMLG	73423	ES	3	1	4	0.00	6.5E-01	excluded
SNX14	76927	ES	7	6	8	0.00	6.5E-01	included
ZMYND8	59713	ES	21	20	22	0.00	6.5E-01	excluded
RBM25	28258	ES	6	5	7	0.00	6.5E-01	excluded
CCDC146	80188	ES	10	9	11	0.00	6.5E-01	excluded
SSR2	8161	ES	4.1:5	2	7	0.00	6.5E-01	excluded
ETFA	31933	ES	2:3:4:5	1	6	0.00	6.5E-01	included
CCNB1IP1	26421	ES	7.1	5	8	0.00	6.5E-01	excluded
NAB1	56584	ES	2:03	1	4.2	-0.01	6.5E-01	excluded
DIS3L	31264	ES	9	8	10	0.00	6.5E-01	excluded
SFTA3	27268	ES	3:4.2:4.3:5	2	6	0.00	6.5E-01	excluded
CHN1	56051	ES	8.2	7	10	0.00	6.5E-01	excluded
SERINC3	59472	ES	2	1	3	0.00	6.5E-01	excluded
USP14	44435	ES	4	3	5	0.00	6.5E-01	included
ELMO2	59684	ES	03:04.1	2	4.2	0.01	6.5E-01	included
DHDDS	1277	ES	5	4.2	6.1	0.00	6.5E-01	excluded
CASP10	56811	ES	3	2	4	0.00	6.5E-01	included
COPE	48516	ES	4:5:6:7	3	8	0.00	6.5E-01	included
MRPL37	3139	ES	03:04.1	1	4.2	0.00	6.5E-01	included
CRYZL1	60457	ES	12.1:12.2:13:14:16.2	11	17	0.00	6.6E-01	included
NUMA1	17516	ES	17:18	16	19	0.00	6.6E-01	excluded
UBL7	31721	ES	2	1.1	3	0.00	6.6E-01	included
DCAF8	8447	ES	7.2:7.3:8.1:8.2	7.1	9	0.01	6.6E-01	included
TTC7A	53486	ES	20	19	21	0.00	6.6E-01	included
NOP14	68622	ES	16.2:16.3:17:18.1:18.2	16.1	18.3	0.00	6.6E-01	excluded
COL14A1	85019	ES	18	17	19	0.00	6.6E-01	excluded
SEC24D	70452	ES	4	3	5	0.00	6.6E-01	excluded
MGRN1	33782	ES	12	11	13	0.00	6.6E-01	included
DCTN2	22642	ES	7:09	2	10	0.00	6.6E-01	excluded
CAST	72855	ES	12:13	11.2	14	0.00	6.6E-01	included
TMEM254	12337	ES	4:05	3	6	-0.01	6.6E-01	excluded
GABARAPL1	20408	ES	2.7:2.8:2.9:2.10:2.12	2.6	3	0.00	6.6E-01	included
GLYCTK	65211	ES	3	2	4	-0.01	6.6E-01	excluded
ZCWPW1	80944	ES	17	16	18	0.00	6.6E-01	included
MTMR11	7415	ES	13	12	14	0.00	6.6E-01	included
TYMP	96247	ES	2.2:2.3:3:4	1	5	-0.01	6.6E-01	excluded
IFT20	39882	ES	5	4	6.1	0.00	6.6E-01	included
PACRGL	68910	ES	6	5	8	0.01	6.6E-01	included
BBS4	31578	ES	2	1.1	5	0.00	6.6E-01	excluded
ATXN3	28917	ES	9:10.1:10.2	8.2	13.2	0.00	6.6E-01	excluded
STK38L	20873	ES	2	1	4.1	0.00	6.6E-01	included
ITFG3	32856	ES	13.1	12	14	0.00	6.6E-01	included
ANK3	11850	ES	40	39	41	0.00	6.6E-01	excluded
RCHY1	69525	ES	2	1.2	3.1	0.00	6.6E-01	excluded
COX4I1	156372	ES	4.2:5.1:5.2:5.3	4.1	5.4	0.00	6.6E-01	included
MEF2D	8276	ES	7	5	8	0.00	6.6E-01	included
DNAL1	28305	ES	2.2:3:4:5:6	1	7	0.00	6.6E-01	excluded
POLD3	17774	ES	3	2	4	0.00	6.6E-01	excluded

MEST	81807	ES	11	10	12	0.00	6.6E-01	excluded
PCSK2	58728	ES	3	2.2	4	0.00	6.6E-01	included
ARHGEF10	82560	ES	24	23	25	0.00	6.6E-01	included
HMGA1	75771	ES	4.2:5	4.1	6	0.00	6.6E-01	excluded
SDHAF2	16231	ES	2:3:4.1:5.2	1	6	0.00	6.6E-01	excluded
EPB41	1414	ES	7	6	8	0.00	6.6E-01	included
ISCU	24230	ES	5.3:5.4:5.5:5.6	5.1	6	0.00	6.6E-01	excluded
SLC38A9	72051	ES	14	13	15	0.00	6.6E-01	excluded
HMGN1	107416	ES	6.2:7	5	9	0.00	6.6E-01	included
SEC16A	88175	ES	24	23.12	25	0.00	6.6E-01	included
DLAT	18712	ES	2.2:3:4:5.1	2.1	5.2	0.00	6.6E-01	excluded
CSAD	21958	ES	8:9:10.1:10.2:10.3:10.	7.2	11	0.01	6.6E-01	included
TFAP4	33642	ES	2	1	3	0.00	6.6E-01	excluded
SH3D19	70837	ES	9	8.2	10	0.00	6.6E-01	included
SCAPER	31953	ES	12	11	13	0.00	6.6E-01	excluded
CALU	81712	ES	2:03:04	1	6	0.01	6.6E-01	included
CCNT2	55413	ES	6.1:6.2	5	7.1	0.01	6.6E-01	included
TNC	87351	ES	12:13:14:15:16:18:19	11	20	-0.01	6.6E-01	excluded
NENF	9730	ES	2	1	3	0.00	6.6E-01	included
RAB18	11074	ES	3:04	2	5	0.00	6.6E-01	included
SSR1	75257	ES	10	9.1	11.1	0.00	6.6E-01	excluded
CDK7	72327	ES	5.1:6:7:8	4	9	0.00	6.6E-01	excluded
ZCCHC6	86764	ES	21	20	22	0.00	6.6E-01	included
C14orf80	29663	ES	8	7	10	0.00	6.6E-01	included
STAU2	84177	ES	3:4:5:7:8	2	11	0.00	6.6E-01	included
IRAK1	90548	ES	6	5	7	0.00	6.6E-01	excluded
DPF2	16816	ES	5:6:7:8:9:10	4	11	0.00	6.6E-01	included
TAMM41	63409	ES	5:06	4	7	0.00	6.6E-01	included
EPN1	52140	ES	3	2	4	0.00	6.6E-01	excluded
ERP29	24569	ES	3	1	4	0.00	6.6E-01	included
OS9	22709	ES	5.1:5.2:7.1:7.3:7.4:8:9.	4	9.2	0.00	6.6E-01	excluded
LIMS1	54890	ES	10	8	11	0.00	6.6E-01	included
FBXO7	61934	ES	3.1:3.2	1	4	0.00	6.6E-01	included
ATG5	77116	ES	4:05:06	3	7	0.00	6.6E-01	included
ZDHHC13	14686	ES	2	1	3	0.00	6.6E-01	excluded
ZKSCAN1	80868	ES	2:3.1:3.2:3.3:4	1	5	0.00	6.6E-01	included
DCTD	71243	ES	2.1	1.2	5	0.00	6.6E-01	excluded
FAXDC2	74234	ES	6:07	5.2	9.1	0.00	6.6E-01	excluded
COX10	39337	ES	3.1	2	4	0.00	6.6E-01	included
MADD	15722	ES	18	17	19	0.00	6.6E-01	included
RPAP3	21340	ES	2	1	3	0.00	6.6E-01	included
PORCN	88982	ES	7	6	9	0.00	6.6E-01	included
NOP2	19890	ES	5	4.5	6	-0.01	6.6E-01	excluded
STAG1	66937	ES	5:06	4	7	0.00	6.6E-01	excluded
ORC3	76968	ES	15	14	16	0.00	6.6E-01	included
AGTRAP	669	ES	5	3	6.1	0.00	6.6E-01	included
UNC50	54646	ES	1.2:1.4	1.1	1.5	0.00	6.6E-01	included
RCAN3	1122	ES	3	2	4.1	0.00	6.6E-01	included
PLEKHA1	13354	ES	15.1:15.2	14	16	0.00	6.6E-01	excluded
OPTN	10783	ES	3:04	2	5.2	0.00	6.6E-01	included
NACA	93186	ES	3.3:3.4:3.5	2.2	4.2	0.00	6.6E-01	excluded
PMM2	33915	ES	8	7	9.1	0.00	6.6E-01	excluded
RBM33	82440	ES	6	5	7.1	0.00	6.6E-01	excluded
MFN2	696	ES	2	1	3	0.00	6.6E-01	included
CAPN5	17942	ES	3.2:5:6:7:8:9:10:11:12<br>:13:14:15.1	3.1	15.2	0.00	6.6E-01	included
PHYKPL	74857	ES	5	4	7	0.00	6.6E-01	included
IL17RE	63246	ES	3:04:05	2.2	6	0.00	6.6E-01	included
ARNT	7511	ES	16	15	17.1	0.00	6.6E-01	excluded
ACADSB	13386	ES	3	2	4	0.00	6.6E-01	included
CASC1	20786	ES	6	4	7	0.00	6.6E-01	included
ANKRD16	10668	ES	6	5	7.1	0.00	6.6E-01	excluded

GIT2	24372	ES	18.2:19	17.2	20	0.00	6.6E-01	excluded
CHIC2	69318	ES	4	3	5	0.00	6.7E-01	excluded
LSMD1	39064	ES	3	2	5.3	0.00	6.7E-01	excluded
GOSR1	40119	ES	3	2.2	4	0.00	6.7E-01	included
COL1A1	409535	ES	20:21:22:23:24:25:26:<br>27:28:29:30:31:32:33:	19	41	0.01	6.7E-01	included
CSRP1	9386	ES	5.2:6.1	5.1	6.2	0.00	6.7E-01	included
OSBPL9	2978	ES	6.1:6.2	5	7	0.00	6.7E-01	excluded
GPHN	28025	ES	7	6	8	0.00	6.7E-01	excluded
YY1AP1	8113	ES	4.1	3.2	6	0.00	6.7E-01	excluded
C19orf43	47858	ES	1.2:2.1	1.1	2.2	0.00	6.7E-01	included
GPRASP1	89703	ES	2:03	1	4	0.01	6.7E-01	included
SNRNP27	53862	ES	5	4	6.1	0.00	6.7E-01	excluded
AASDH	69352	ES	2	1	3	0.00	6.7E-01	included
TARS2	7480	ES	4.1:4.2	3.2	5	0.00	6.7E-01	included
CASP3	71300	ES	2	1	4	-0.01	6.7E-01	excluded
C7orf55-LUC7L2	81959	ES	6	5	7	-0.01	6.7E-01	excluded
HSD17B4	73088	ES	2	1.1	4	0.00	6.7E-01	included
LY6E	85389	ES	4	2	5.2	0.00	6.7E-01	included
EMP3	50727	ES	3	1	4.1	0.00	6.7E-01	included
ASCC3	77093	ES	3	2	5	0.00	6.7E-01	excluded
JMJD6	43618	ES	5.1:5.2:6	4	7.1	0.00	6.7E-01	included
RNF13	67240	ES	2:03	1	4	0.01	6.7E-01	included
CYCS	79028	ES	2	1.1	3	0.00	6.7E-01	excluded
C5orf45	74965	ES	2.2:2.3:3.1:3.2:4:5.1:5.	2.1	6	0.01	6.7E-01	included
MSL3	88488	ES	6	5	7	0.00	6.7E-01	excluded
SNRK	64343	ES	4	1	5	0.00	6.7E-01	excluded
AP1G1	37491	ES	2	1	5	0.00	6.7E-01	included
YIF1B	49609	ES	4	3.2	5	0.00	6.7E-01	excluded
MCCC1	67775	ES	18:19	17	20	0.01	6.7E-01	included
CORO7	33675	ES	5.1:5.2:6.1:6.2	4	7.1	0.00	6.7E-01	excluded
LINC00999	91517	ES	3:04	2	5	0.00	6.7E-01	included
CLNS1A	17965	ES	2	1	3	0.00	6.7E-01	included
C19orf60	48490	ES	3.1:3.2	2	4	0.00	6.7E-01	included
FAM168A	17696	ES	4	3	5	0.00	6.7E-01	excluded
MRRF	87468	ES	8	7	9	0.00	6.7E-01	excluded
POLR1B	55001	ES	9:10	8	11.1	0.00	6.7E-01	excluded
ADK	12259	ES	8	7	9	0.00	6.7E-01	included
IL33	85817	ES	3:04:05	2.2	6	0.00	6.7E-01	included
RFWD2	9060	ES	4	3	6.1	0.00	6.7E-01	included
HAPLN3	32399	ES	5	4	6	-0.01	6.7E-01	excluded
RYR1	102720	ES	94	93	95	0.01	6.7E-01	included
SLC39A9	28148	ES	4	3	5	0.00	6.7E-01	included
CA4	42851	ES	4:05	3	6	0.00	6.7E-01	included
CTSB	97873	ES	2:04	1.1	5.3	0.00	6.7E-01	included
PNPLA8	81415	ES	2	1	4.1	-0.01	6.7E-01	excluded
KLHDC1	27435	ES	7	6	8	0.00	6.7E-01	excluded
SNX11	42179	ES	3.1:4:5	1.2	6	0.00	6.7E-01	excluded
KLHDC2	27442	ES	9	8	10	0.00	6.7E-01	included
HDAC10	62806	ES	3	2	4	0.00	6.7E-01	excluded
CYLD	36395	ES	7	6	8.2	0.00	6.7E-01	included
HSD17B1	41060	ES	4	3	5.2	0.00	6.7E-01	included
ANKRD11	38084	ES	2	1	3	0.00	6.7E-01	included
GTF2I	80088	ES	10	9	11.1	0.00	6.7E-01	included
IRF7	13710	ES	4:5.1:5.2	3.2	5.3	-0.01	6.7E-01	excluded
SNX11	42177	ES	3.1:3.2:4:5	1.2	6	0.00	6.7E-01	included
FOPNL	34194	ES	4:05	2	6	0.00	6.7E-01	included
TACC2	13343	ES	14	13	15.1	0.00	6.7E-01	included
TWF2	65189	ES	8	7	9	0.00	6.7E-01	excluded
TFDP2	67094	ES	8	6	9	0.00	6.7E-01	excluded
ISYNA1	48439	ES	2	1.3	3.2	0.00	6.7E-01	excluded
GSTA4	76481	ES	4:5.1:5.2	3.2	6	0.00	6.7E-01	included

MAN1C1	1194	ES	2	1	3	0.00	6.7E-01	included
LILRB2	51858	ES	12	11	13	0.00	6.7E-01	excluded
EDC3	31732	ES	2:03:04	1	5	0.00	6.7E-01	excluded
ABI2	57024	ES	6:07	5.3	8	0.00	6.7E-01	included
CCM2L	58925	ES	7	6	8	0.00	6.7E-01	excluded
SNX11	42183	ES	3.1:4	1.2	6	0.00	6.7E-01	included
EIF2B1	25099	ES	6	5.3	7	0.00	6.7E-01	excluded
TMEM161A	48586	ES	5.1:5.2:6	4	7	0.00	6.7E-01	included
GINS4	83518	ES	4:05	3.1	6.1	0.00	6.7E-01	included
RBBP6	35646	ES	11:12:13:14:15:17	10	18	0.00	6.7E-01	excluded
WDR48	64136	ES	2:3.2:4.1:4.2:5.2:6:7	1	8	0.00	6.7E-01	included
PLEKHG4	37002	ES	11:12:13:14:15:16:17:	10	20	0.00	6.7E-01	included
ZNF28	51626	ES	6.2	5	7.1	-0.01	6.7E-01	excluded
SLC30A6	53154	ES	7	6	8	0.00	6.7E-01	excluded
CCAR1	11956	ES	14	13	15	0.00	6.7E-01	included
HMGA1	75775	ES	1.3	1.1	3.1	0.00	6.7E-01	excluded
FBXW9	47843	ES	2.1:2.2	1.2	3	0.00	6.7E-01	included
OLA1	56019	ES	3	1	4	0.00	6.7E-01	included
CALD1	81859	ES	17	16	18	0.00	6.7E-01	excluded
LDLR	47629	ES	3	2	4	0.00	6.7E-01	included
CISH	65091	ES	2	1	3	0.00	6.7E-01	excluded
TMEM30A	76785	ES	3	1	4	0.00	6.7E-01	included
BCL2L13	60992	ES	6:07	4	8.1	0.00	6.7E-01	excluded
FXR1	67755	ES	7:8:9:10:11:12:13:14:	1	16	0.01	6.7E-01	included
C7orf43	80911	ES	3	2.2	4.1	0.00	6.7E-01	included
ANAPC5	24864	ES	8.1:8.2:8.3	7	9.1	0.00	6.7E-01	included
TPD52	84278	ES	9	8	11	0.00	6.7E-01	excluded
SLC25A29	29263	ES	2:3.4:3.5:3.6	1	3.7	0.00	6.7E-01	included
ANAPC11	44213	ES	5	3.3	6	0.00	6.7E-01	included
HERC6	69891	ES	15	14	16	0.00	6.7E-01	included
ZNF688	36159	ES	2	1.2	4	0.00	6.7E-01	excluded
CTNS	38474	ES	5	4	7	0.00	6.7E-01	included
LRRFIP2	63980	ES	7:8:10:14	5	18	0.00	6.7E-01	excluded
TNFRSF1A	19832	ES	3.1:3.2:4.1	2	4.2	0.00	6.7E-01	included
HSCB	61545	ES	4	3.2	5	0.00	6.7E-01	excluded
RHOC	4248	ES	2.3	1.1	3	0.00	6.7E-01	excluded
RAB27A	30715	ES	4	3.2	5	0.00	6.7E-01	excluded
DPY19L3	48876	ES	20	19	21	0.00	6.7E-01	excluded
SDHC	8661	ES	4	3	5	0.00	6.7E-01	included
TMEM260	27646	ES	5	4	6	0.00	6.7E-01	excluded
MLH1	63947	ES	2.1:2.2	1.1	4.1	0.00	6.7E-01	excluded
MED24	40834	ES	4	3	5	0.00	6.7E-01	excluded
MMAB	24321	ES	9.1	8	9.3	0.01	6.7E-01	included
SRRT	81020	ES	3	2	4	0.00	6.7E-01	excluded
TATDN3	9764	ES	4	3	5	0.00	6.7E-01	included
TRIM16L	39637	ES	7	6.2	8.1	0.00	6.7E-01	included
SLC25A23	47040	ES	13:14	10	15	0.01	6.7E-01	included
ATHL1	13638	ES	09:10.1	8.2	10.2	0.00	6.7E-01	excluded
ASNS	80566	ES	2.3:3:4.1:4.2	1	5	0.01	6.8E-01	included
RNF216	78678	ES	3:4:5.2:6.1:6.2	2	7	0.00	6.8E-01	included
DDX5	43065	ES	4.1:4.2	3	5	0.00	6.8E-01	included
NFIA	3227	ES	6:7:8:9	5	10	0.00	6.8E-01	included
NCOA7	77430	ES	5	4	6	0.00	6.8E-01	included
SRC	59350	ES	8	7	9	0.00	6.8E-01	excluded
IFI16	8398	ES	9	7	10	0.00	6.8E-01	excluded
TATDN3	9757	ES	8:9.1:9.2	7	9.3	-0.01	6.8E-01	excluded
BRF2	83361	ES	3.1	2	4	0.00	6.8E-01	included
WDR25	29306	ES	5	2	6	0.00	6.8E-01	excluded
PIGT	59568	ES	2.1:2.2:4:5.2:6	1	7	0.00	6.8E-01	excluded
FYN	77275	ES	11	10	13	0.00	6.8E-01	excluded
BRD9	71469	ES	7	6.2	8	0.00	6.8E-01	included
PPIP5K1	30270	ES	27	26	29	-0.01	6.8E-01	excluded

ST3GAL3	2296	ES	9	6	13	0.00	6.8E-01	included
CYB561	42930	ES	9	8	10.1	0.00	6.8E-01	included
SPECC1	39796	ES	6	5	7	-0.01	6.8E-01	excluded
CD6	16139	ES	9:10.1:10.2	8	11	0.00	6.8E-01	excluded
PIP5K1A	7577	ES	13:14	12	15	0.00	6.8E-01	excluded
U2AF1L4	49275	ES	3.1:3.2	2.2	5	-0.01	6.8E-01	excluded
CAAP1	86033	ES	1.2:2:3:4.2:5	1.1	6	0.00	6.8E-01	excluded
AMD1	77247	ES	3	1	4	0.00	6.8E-01	excluded
ASNS	80567	ES	3:4.1:4.2	1	5	0.00	6.8E-01	excluded
BAK1	75760	ES	6	5	7	0.00	6.8E-01	included
EPN1	52139	ES	7	6	8	0.00	6.8E-01	excluded
ZFYVE27	12740	ES	3:04:05	2.2	6	0.00	6.8E-01	excluded
LETMD1	21790	ES	03:02.2	1.2	5	0.00	6.8E-01	included
NAA40	16550	ES	4.1:4.2	3	5	0.01	6.8E-01	included
FMNL2	55607	ES	16	15	17	0.00	6.8E-01	included
NUP160	15810	ES	3.1:3.2:3.3	2	4	0.00	6.8E-01	excluded
ATP8A1	69142	ES	15	14	16	0.00	6.8E-01	included
MRS2	75510	ES	3:04	2	6.1	0.00	6.8E-01	excluded
HSD11B1L	46886	ES	4.2:5.2:5.3:7	1	8	-0.01	6.8E-01	excluded
SEPT4	42701	ES	7.1:7.2	4	8	-0.01	6.8E-01	excluded
TWF1	21279	ES	1.3	1.1	2	0.00	6.8E-01	included
ZNF268	25348	ES	10.1:10.2:11:12	9	13	0.00	6.8E-01	excluded
AKAP10	39781	ES	11	10	12	0.00	6.8E-01	included
EPS8	20586	ES	5	4	6	0.00	6.8E-01	excluded
ACAA1	64020	ES	7	6	8.1	0.00	6.8E-01	excluded
SUPT3H	76397	ES	3:04	2	5	0.00	6.8E-01	included
ZNF75D	90177	ES	4:05	3	6	0.00	6.8E-01	excluded
YAF2	21170	ES	2	1	5.2	0.00	6.8E-01	included
DEPDC1B	72155	ES	12	11	13	0.00	6.8E-01	included
OCLN	72380	ES	4	3	5	0.00	6.8E-01	included
CUEDC1	42625	ES	4.1:4.2	1	5	0.00	6.8E-01	included
TSPAN17	74674	ES	5	4.2	6.1	0.00	6.8E-01	excluded
CTH	3411	ES	3	2	4	0.00	6.8E-01	excluded
HDAC7	21372	ES	10.2:11	10.1	12	0.00	6.8E-01	included
UQCC1	59088	ES	11.1:11.2	10.1	12.1	0.00	6.8E-01	included
NEDD4L	45674	ES	4:8:9:10:11:12:13:14:<br>15:16:17:19:20:21:22:<br>23:24:26:27:28:29:30:	1	35	0.01	6.8E-01	included
PIGK	3520	ES	2:03:04	1	5	0.00	6.8E-01	included
PTPRA	58570	ES	8	7	10	0.00	6.8E-01	included
TBC1D25	88991	ES	4	3	5	0.00	6.8E-01	excluded
VEZT	23782	ES	6.1:6.2:9	4	11	0.00	6.8E-01	excluded
MTHFSD	37932	ES	3:05:07	2.2	8	0.00	6.8E-01	included
PPIE	1909	ES	04:01.1	2	6	0.00	6.8E-01	included
CRAT	87829	ES	2	1	4.1	-0.01	6.8E-01	excluded
PPA2	70233	ES	2:4:5:7:9	1	11	0.00	6.8E-01	excluded
RSL1D1	34039	ES	2.1:2.2:3:4:5	1.1	6.1	0.00	6.8E-01	included
LRR1	27423	ES	6	5	7	0.00	6.8E-01	included
COPS7A	19941	ES	10	9	11	0.00	6.8E-01	excluded
RAD1	71741	ES	6:07	5	8	-0.01	6.8E-01	excluded
ARID1B	78238	ES	3	2	4	0.00	6.8E-01	excluded
ACOX1	43539	ES	3	2	5	0.00	6.8E-01	included
LRRC20	12050	ES	4	3	5	0.00	6.8E-01	included
PILRA	80939	ES	3:04	2	5	0.00	6.8E-01	excluded
HES6	58213	ES	1.3	1.1	2.1	0.00	6.8E-01	included
METTL16	38402	ES	4:05	3	6	0.00	6.8E-01	included
TMEM230	58638	ES	2	1.2	4	0.00	6.8E-01	excluded
PAIP1	71961	ES	6	5	7	0.00	6.8E-01	excluded
POC5	72542	ES	13	12	14	0.00	6.8E-01	included
GMPR2	26925	ES	3.2:4	3.1	5	-0.01	6.8E-01	excluded
LRMP	20765	ES	20	19	21	0.00	6.8E-01	excluded
CBWD2	55055	ES	8	7	9	0.00	6.8E-01	excluded

KIAA0586	27717	ES	35	34	36	0.01	6.8E-01	included
DNM1L	21061	ES	3	2	5	0.00	6.8E-01	included
SEMA4B	32477	ES	2.2	1	4	0.00	6.8E-01	included
TJAP1	76274	ES	10	9	11	0.00	6.8E-01	excluded
GALK2	30527	ES	8	7.2	10	0.00	6.8E-01	excluded
NUP155	71827	ES	23	22	24	0.00	6.8E-01	included
PTPN13	69836	ES	19:20	18	22	0.00	6.8E-01	excluded
ASNSD1	300789	ES	2:3.2:3.3:4.1:5	1	6	0.00	6.8E-01	excluded
PILRB	80933	ES	10:11	9	12.2	0.00	6.8E-01	included
SEMA4F	54138	ES	5:06	4	7	-0.01	6.8E-01	excluded
NKIRAS1	63725	ES	2.2:4.1	2.1	4.2	-0.01	6.8E-01	excluded
FHL2	54839	ES	2.2:2.3:3.2	2.1	5.1	0.00	6.8E-01	included
SVIL	11116	ES	10:11:12	9	13	0.01	6.8E-01	included
DDX3X	88856	ES	4	3	5	0.00	6.8E-01	included
FIZ1	52114	ES	2.2:3.1	2.1	3.2	0.00	6.9E-01	excluded
SSX2IP	3618	ES	14	13	15	0.00	6.9E-01	excluded
MTMR14	63123	ES	5.1:5.2:6:7	3	8	0.00	6.9E-01	included
XYLT2	42368	ES	9	8	10	0.00	6.9E-01	included
VPS53	38234	ES	3.2:4:5:6:7:9:10:11:12:13:14:15:16:17.1:17.2:18:19:20.1:21:22:23	3.1	23.2	0.00	6.9E-01	included
LIPT1	54679	ES	3:05	1	6	-0.01	6.9E-01	excluded
ACY1	65153	ES	8.2:9:10	8.1	11	0.00	6.9E-01	excluded
SEC14L2	61745	ES	4:5.1:5.2	3	6.2	0.00	6.9E-01	excluded
WDR75	56505	ES	3	2	4.2	0.00	6.9E-01	excluded
MTMR14	63120	ES	6	5.2	7	0.00	6.9E-01	excluded
ARMCX5	89699	ES	2.6	2.4	2.8	0.01	6.9E-01	included
ELMO3	36977	ES	4	3	5	0.00	6.9E-01	excluded
GALK1	43497	ES	3	2	4.1	0.00	6.9E-01	excluded
RIC8B	24169	ES	13	11	17	-0.01	6.9E-01	excluded
SPIDR	83788	ES	3	2.3	4	0.00	6.9E-01	included
ATP6V0D1	37070	ES	7	4	8	0.00	6.9E-01	included
SLC35D2	86968	ES	7:08:09	6.1	10	0.00	6.9E-01	excluded
UEVLD	14669	ES	12:13	11	14	0.00	6.9E-01	included
CYP2S1	50024	ES	4:05:06	3	7	0.00	6.9E-01	included
SCN4B	18966	ES	2:03	1	4	0.00	6.9E-01	included
CD36	80232	ES	12	11	13	0.00	6.9E-01	included
DSCR3	60559	ES	2:03:04	1	5	0.00	6.9E-01	excluded
STOML1	31625	ES	3	2	4	0.00	6.9E-01	excluded
CAPZA2	81552	ES	2	1	3	0.00	6.9E-01	included
OGDH	79546	ES	6	5	7	0.00	6.9E-01	excluded
GZMB	27021	ES	3.2:4	1	5.1	0.00	6.9E-01	included
EIF4G3	959	ES	11	9	12	0.00	6.9E-01	included
MUC1	8006	ES	2.3:3.1:3.2:3.3:3.4:4.1:	2.2	4.3	0.00	6.9E-01	included
SERPING1	15872	ES	3.2	2.2	4	0.00	6.9E-01	excluded
NDUFB3	56782	ES	2.2	1	3	0.00	6.9E-01	excluded
C1orf63	1148	ES	4.2:5.1	4.1	5.2	0.00	6.9E-01	excluded
ZDHHC16	12709	ES	2	1	4	0.00	6.9E-01	included
CADPS2	81610	ES	19	18	20	0.00	6.9E-01	excluded
YAF2	21117	ES	6	5.2	9.1	-0.01	6.9E-01	excluded
PACRGL	68908	ES	6:07	5	8	0.00	6.9E-01	excluded
ARMC6	48572	ES	1.2:5:6	1.1	7	-0.01	6.9E-01	excluded
ENOSF1	123118	ES	15:16:17	13	18.1	-0.01	6.9E-01	excluded
SUB1	71659	ES	4.2	3	5	0.00	6.9E-01	excluded
ERG	60597	ES	6	5	7	0.00	6.9E-01	included
STOML1	31624	ES	5	4	6	0.00	6.9E-01	excluded
CALM2	53490	ES	5	4	6	0.00	6.9E-01	included
TRMU	62708	ES	11	10	12	0.00	6.9E-01	excluded
PARD3B	57100	ES	17	16	18.1	0.00	6.9E-01	included
SCAMP4	46562	ES	5	4	6	0.00	6.9E-01	included
GHDC	41024	ES	4.3:5.1	4.2	5.2	0.00	6.9E-01	excluded
FDFT1	82656	ES	3.2	2.2	5.2	0.00	6.9E-01	included

RBM6	64947	ES	7	2	8	0.00	6.9E-01	excluded
GTF2H3	25107	ES	2.2	1	3	0.00	6.9E-01	included
PAQR3	69662	ES	2	1	4	0.00	6.9E-01	excluded
TMTC4	26199	ES	2	1	3	0.01	6.9E-01	included
MORF4L2	89768	ES	04:05.1	3.2	5.2	0.00	6.9E-01	included
DPP8	31179	ES	10:11:12:13	9	14	0.00	6.9E-01	excluded
TNRC6A	35650	ES	13	12	14	0.00	6.9E-01	excluded
DHRS7	27772	ES	2	1	3	0.00	6.9E-01	excluded
ACSL5	13112	ES	23	22	24	0.00	6.9E-01	included
CEP112	43099	ES	8:09	7	10	0.00	6.9E-01	included
EDNRA	70787	ES	3	2	4	0.00	6.9E-01	included
MGST3	8802	ES	3	1.2	5	0.00	6.9E-01	included
UBA52	48484	ES	1.2:1.3:2	1.1	4.2	0.00	6.9E-01	included
PTPLAD1	31188	ES	5	1.2	7	0.00	6.9E-01	included
MBOAT7	51805	ES	2	1	3	0.00	6.9E-01	included
MRPL55	10069	ES	03:04.1	2.9	4.2	0.00	6.9E-01	included
DHPS	47833	ES	3.3:4	3.2	5	0.00	6.9E-01	excluded
TAF1	89424	ES	35	34	36	0.00	6.9E-01	excluded
AMZ2	43130	ES	4	3.2	5	0.00	6.9E-01	included
CBWD2	55054	ES	12	11	13	0.00	6.9E-01	included
PAMR1	15418	ES	7:08	6	9	0.00	6.9E-01	excluded
EIF3L	62170	ES	5:06	4	7	0.00	6.9E-01	excluded
TCF7L2	13156	ES	4	3	5	0.01	6.9E-01	included
ABI1	11050	ES	4:05	3	7	0.00	6.9E-01	excluded
SEC24C	12177	ES	9:10	7	11	0.00	6.9E-01	included
MRPL55	10156	ES	2.5:2.6	1.1	2.9	0.00	6.9E-01	excluded
DMKN	49154	ES	15	13	16	0.00	6.9E-01	excluded
YWHAH	61909	ES	3	1	4	0.00	6.9E-01	excluded
ARHGAP8	62633	ES	10	9	11	0.00	6.9E-01	included
UBE2F	58160	ES	10	9	11	0.00	6.9E-01	excluded
NR4A2	55621	ES	4	2	5.1	0.00	6.9E-01	excluded
TTLL3	63219	ES	4:5:6.3:6.4:6.5:7	3	8.1	0.00	6.9E-01	included
PYCR1	44244	ES	7	6	8.1	0.00	6.9E-01	included
ZW10	18800	ES	3:04:05	2	6	0.00	6.9E-01	included
BNIP1	74579	ES	5	4	6	0.00	6.9E-01	excluded
SELENBP1	7623	ES	4.1	3	5	0.00	6.9E-01	excluded
PLEKHA5	20662	ES	12	11	13	0.00	6.9E-01	excluded
CLTC	42788	ES	3.1:3.2	2	4	0.00	7.0E-01	included
SNX6	27162	ES	6	5	7	0.00	7.0E-01	included
CLEC1A	20302	ES	2:03	1	4	0.00	7.0E-01	excluded
KDM2B	24882	ES	17:18:19:20:21:22:23:	16	26.1	0.00	7.0E-01	excluded
EIF3H	84960	ES	5	3.2	6	0.00	7.0E-01	included
TBC1D15	23418	ES	7:08	6	9	0.00	7.0E-01	excluded
STAT3	41036	ES	2.1:2.2:2.3	1	3	0.00	7.0E-01	included
CEP70	67004	ES	4:05:06	2	7.1	0.01	7.0E-01	included
MRPL49	16786	ES	3.1:3.3:3.4:3.5	2	3.6	0.00	7.0E-01	excluded
H2AFY	73452	ES	4:5:6.3:8	3	9	0.00	7.0E-01	excluded
RBM42	49229	ES	05:06.1	3.2	6.2	0.00	7.0E-01	excluded
YAF2	21164	ES	3.2:5.2	2	9.1	0.00	7.0E-01	excluded
UBA52	48480	ES	2	1.2	4.2	0.00	7.0E-01	included
TTC8	28769	ES	6:7:8.1:8.2:9:10.1:11:	2	17	0.00	7.0E-01	excluded
ARHGAP4	90525	ES	16	15	17	0.00	7.0E-01	included
SIRT7	44237	ES	4	3	5	0.00	7.0E-01	excluded
ENKD1	37096	ES	5	4	6	0.00	7.0E-01	excluded
PDE6B	100561	ES	22	21	23.1	0.00	7.0E-01	excluded
PIK3IP1	61842	ES	5.1	4	6	0.00	7.0E-01	included
KIAA0101	31113	ES	4	3	5	0.00	7.0E-01	included
RABL3	66383	ES	3	2	4	0.00	7.0E-01	excluded
STAG3	97624	ES	5:6:7:8:9:10	4	11	-0.01	7.0E-01	excluded
SNAP23	30173	ES	08:09.1	6	10	0.00	7.0E-01	included
RAN	25203	ES	4	3	5	0.00	7.0E-01	excluded
ZNF266	47347	ES	3	2	4.1	0.00	7.0E-01	excluded

BPHL	75167	ES	2	1.2	3	0.01	7.0E-01	included
ZNF433	94995	ES	2	1	4.2	0.01	7.0E-01	included
HNRNPA2B1	79037	ES	12.2	11	12.4	-0.01	7.0E-01	excluded
CINP	29413	ES	6	4	7	0.00	7.0E-01	included
NR3C2	70805	ES	6	5	7	0.00	7.0E-01	included
SEPN1	1195	ES	3	2	4	0.00	7.0E-01	excluded
ELMOD3	54216	ES	4	3	5	0.00	7.0E-01	excluded
SNX1	139176	ES	4.1:4.2:5:6:8:9:10.2:1<br>1:12:13.1:14:15	3	16.1	-0.01	7.0E-01	excluded
FBXL12	47430	ES	2.4	2.2	5	0.00	7.0E-01	included
ALKBH2	24275	ES	3	2.2	4	0.00	7.0E-01	excluded
CSNK1G3	73148	ES	2.2:3:4	1	5	0.00	7.0E-01	included
NBPF11	7345	ES	5	4	8.1	0.01	7.0E-01	included
RAB3IP	23350	ES	8.2:9.1:9.2:10	8.1	11	0.01	7.0E-01	included
DCTN5	35625	ES	5	2	6	0.00	7.0E-01	excluded
CBWD5	86507	ES	6	5	7	0.00	7.0E-01	included
GK5	67108	ES	10	9	11	-0.01	7.0E-01	excluded
SMOX	58626	ES	5:6.1:6.3:7	4	9	0.00	7.0E-01	excluded
INO80E	36013	ES	6.2:6.3:10	5	11	-0.01	7.0E-01	excluded
CDC27	42050	ES	9.1:9.2:10:11:12.1:12.	8	15	0.00	7.0E-01	excluded
CHCHD6	66604	ES	5	4	6.2	0.00	7.0E-01	excluded
THBS3	8032	ES	4	3	5	0.00	7.0E-01	included
PXMP2	25284	ES	6	5	7.1	0.00	7.0E-01	excluded
PDCD6IP	63890	ES	9	8.2	10	0.00	7.0E-01	excluded
DFFB	346	ES	5	4.3	6	0.01	7.0E-01	included
SCEL	26079	ES	17	16.5	18	-0.01	7.0E-01	excluded
PLXNB1	64644	ES	5:6:7:8:9:10:11:12:13.<br>1:13.2:13.3:14:15:16:<br>17:18:19:20:21:22:23.	4	23.2	0.00	7.0E-01	excluded
SMIM7	48196	ES	4:05	2	7.1	0.00	7.0E-01	excluded
ASTE1	66774	ES	7	6	8	0.00	7.0E-01	excluded
SFTA3	121941	ES	2:3:4.2:4.3:5	1.3	6	0.00	7.0E-01	included
TREX1	64681	ES	2.3:2.4:2.6:2.7	2.2	2.8	0.00	7.0E-01	excluded
ZNF586	52341	ES	3	1	4	-0.01	7.0E-01	excluded
SLC25A32	84818	ES	3	2	4	0.00	7.0E-01	excluded
SHOC2	13100	ES	3	2	4	0.00	7.0E-01	excluded
TRPC6	18431	ES	4	3	5	0.00	7.0E-01	excluded
HARS	73737	ES	3.2	2	4	0.00	7.0E-01	excluded
BIVM	26221	ES	8	7	9	0.00	7.0E-01	included
HSDL2	87248	ES	2	1	3	0.00	7.0E-01	excluded
STX16	59979	ES	4.1:4.2:4.3	1.4	5.1	0.00	7.0E-01	included
NT5C3A	79215	ES	3:4.1:4.2	1	5	0.00	7.0E-01	excluded
ACACB	24283	ES	48	47	49	0.01	7.0E-01	included
DECR2	32888	ES	5.1:5.3	4.2	6	-0.01	7.0E-01	excluded
YAF2	21161	ES	4:5.1:5.2	2	9.1	0.00	7.0E-01	excluded
METTL16	38400	ES	9	8	10	0.00	7.0E-01	excluded
RPS9	51826	ES	4.1	3	4.5	0.00	7.0E-01	included
BTK	89637	ES	13.2:14:15:16	13.1	17	0.00	7.0E-01	included
SLC35E2B	207	ES	2	1	3	0.01	7.0E-01	included
GPR63	77027	ES	2	1	3	0.00	7.0E-01	included
PTCD2	72468	ES	3	2	4	0.00	7.0E-01	included
STIM1	14032	ES	13	12	14	0.00	7.0E-01	included
GAK	68423	ES	03:05.2	1	6	0.00	7.0E-01	excluded
FLNA	499333	ES	37:38:39:40:41:43:44:	36	46	0.00	7.0E-01	excluded
MAD1L1	78600	ES	2.2:3:4	1	5	0.00	7.0E-01	excluded
ENDOV	44072	ES	2.3:2.4:6.2	2.2	7	0.00	7.0E-01	excluded
TTI2	83332	ES	3	2	4	0.00	7.0E-01	excluded
RNF212	68453	ES	8:09:10	7	11	0.00	7.0E-01	excluded
VAV2	88085	ES	6	5	7	0.00	7.0E-01	excluded
KIAA1715	56101	ES	5:06	3	7	0.00	7.0E-01	included
TVP23C	39360	ES	4.1:4.2	3	5	0.00	7.0E-01	excluded
SMPD4	55302	ES	4.1:4.2:5	3	6	0.00	7.0E-01	excluded

RAB28	68794	ES	4	3	5	0.00	7.0E-01	excluded
CORO7	33713	ES	1.2:3:4:5.1	1.1	5.2	0.00	7.0E-01	included
ATP5J	60265	ES	1.4:1.5:2	1.3	3	0.00	7.0E-01	included
WDPCP	53730	ES	2	1	3	0.01	7.0E-01	included
CENPM	62469	ES	5	4.2	7	0.00	7.0E-01	included
ETS1	19410	ES	9.2:10.1:10.2	9.1	11	0.00	7.0E-01	included
NOL8	86859	ES	6.3	5	7	0.00	7.0E-01	included
PON3	80531	ES	3	2.2	4	0.00	7.0E-01	included
ZNF81	88945	ES	1.2:2	1.1	3	-0.01	7.0E-01	excluded
ZNF707	85489	ES	3	1	6	0.01	7.0E-01	included
RSF1	17968	ES	3	2	4	0.00	7.1E-01	included
GIN1	72908	ES	5:06:07	4	8	0.00	7.1E-01	excluded
IMPA1	84295	ES	9	8	10	0.00	7.1E-01	excluded
TIA1	53871	ES	13:14.1:14.2	12	15	0.00	7.1E-01	excluded
PANK4	282	ES	2.5:3.1	2.4	3.2	0.00	7.1E-01	included
LETMD1	21786	ES	2:3.2:3.3:4	1.2	5	0.00	7.1E-01	included
KRAS	20821	ES	4:05:06	2	7	0.00	7.1E-01	excluded
SFTA3	27259	ES	4.1:4.2:4.3	2	5	0.00	7.1E-01	included
SLC29A2	17026	ES	9	8	10.1	0.00	7.1E-01	included
C12orf75	24137	ES	2:03	1	4	0.00	7.1E-01	excluded
PLEKHA5	20651	ES	26:28:00	25.2	29	0.00	7.1E-01	included
SARS2	49733	ES	8	6	9	0.00	7.1E-01	included
TMEM106C	21403	ES	2.2:3:4.1	2.1	4.2	0.00	7.1E-01	included
CSAD	21952	ES	14	13	15	0.00	7.1E-01	included
MAN2C1	31874	ES	3.2:4:6:7.1	2	7.2	0.00	7.1E-01	included
GABARAPL1	20390	ES	3	2.6	4.1	0.00	7.1E-01	included
NCKAP1	56467	ES	2	1	3	0.00	7.1E-01	included
STAG3	80921	ES	7:08:09	4	10	0.00	7.1E-01	excluded
BCR	61300	ES	17	16	18	0.00	7.1E-01	excluded
MINPP1	12454	ES	4:05	3	6	0.00	7.1E-01	excluded
DMKN	49208	ES	5	4	6.4	0.00	7.1E-01	included
RPSA	64183	ES	3.1:3.2	2	4	-0.01	7.1E-01	excluded
CDC25B	58602	ES	10	9	11	0.00	7.1E-01	excluded
CEP63	66882	ES	12	11	13	0.00	7.1E-01	included
SLC25A20	64783	ES	2	1	3	0.00	7.1E-01	excluded
HSH2D	48140	ES	5	4	6.1	0.00	7.1E-01	included
NCKAP5	55400	ES	14:15	13	16	0.01	7.1E-01	included
LAMP1	26369	ES	4	3	5	0.00	7.1E-01	excluded
NFU1	53852	ES	3:04	2	5	0.00	7.1E-01	included
CD44	14985	ES	6:7:8:9.1:9.2:10:11:12 .1:13:14:15	5	16.1	0.00	7.1E-01	included
PHLDB1	19036	ES	25	24	26	0.00	7.1E-01	included
METAP2	23793	ES	2.1:2.2	1	3	0.00	7.1E-01	included
DIAPH1	73805	ES	6.2:7:8.1	6.1	8.2	0.00	7.1E-01	included
NUDT14	29604	ES	3	2	4	0.00	7.1E-01	excluded
UBE2D3	70147	ES	2.2:2.3:3.2	2.1	3.3	0.00	7.1E-01	excluded
MRPL52	26640	ES	3	2	5	0.00	7.1E-01	excluded
SAR1B	73419	ES	3	2	4	0.00	7.1E-01	excluded
ALDH7A1	191781	ES	15	14	16	0.00	7.1E-01	excluded
GNB2L1	264678	ES	4.1:4.2:5:7.2:8.1:8.2	3	9	0.00	7.1E-01	excluded
HNRNPA1	212641	ES	3:4:5:6.1:6.2:6.3:7.2:9 .1:9.2:10:11.1:11.2	2	11.3	0.00	7.1E-01	included
ZCCHC11	573887	ES	17:18:19:20:21	3.2	22	-0.01	7.1E-01	excluded
SPIDR	83781	ES	8	5	9.1	0.00	7.1E-01	excluded
SLC37A3	81989	ES	9	8	10.2	0.00	7.1E-01	excluded
PMS1	56551	ES	04:05.1	3	6	0.00	7.1E-01	excluded
PPM1D	42861	ES	6	5	7	0.00	7.1E-01	included
UIMC1	74687	ES	6.2:7.1:7.2	6.1	8	-0.01	7.1E-01	excluded
CCDC134	62463	ES	4:05:06	3	7	0.00	7.1E-01	included
RHOC	4244	ES	2.1:2.2:2.3	1.1	3	0.00	7.1E-01	excluded
TSC1	87999	ES	5	4	6	0.00	7.1E-01	included
AP3M2	83560	ES	2	1.6	3	0.00	7.1E-01	included

COPS7B	57960	ES	4.3:4.4	3	5	0.00	7.1E-01	included
ASF1B	47963	ES	3.1:3.2	2	4	0.00	7.1E-01	excluded
ARFGAP3	62551	ES	4:05	3	6	0.00	7.1E-01	included
EIF1AD	16967	ES	1.2:2.2:2.3:2.4	1.1	3.1	0.00	7.1E-01	excluded
SLC47A1	39742	ES	7:8:9:10.2:11:12:13:1	6	17	0.00	7.1E-01	included
ADD1	68607	ES	18.1:18.2	17	19	0.00	7.1E-01	excluded
ADCK1	28630	ES	8	7	9	0.00	7.1E-01	excluded
FAM120A	86918	ES	8	7	9	0.00	7.1E-01	excluded
PCBP2	22057	ES	12	11.2	14.1	0.00	7.1E-01	excluded
SNX7	3842	ES	2	1	3	0.00	7.1E-01	included
REEP4	82941	ES	7	6	8	0.00	7.1E-01	excluded
HIRA	61051	ES	23	22	24	0.00	7.1E-01	excluded
SNX14	76925	ES	14:15:16:17:18:19:20:	13	22	0.00	7.1E-01	excluded
PPP2R3C	27206	ES	5.1:5.2	4.2	6.1	0.00	7.1E-01	excluded
CD164	77191	ES	5	4	7.1	0.00	7.1E-01	excluded
THBS3	8031	ES	6	5	7.1	0.00	7.1E-01	excluded
BUB3	13392	ES	5:06:07	4	8.2	0.00	7.1E-01	excluded
SKA2	42747	ES	2:3:4.1:4.2	1.1	5	0.00	7.1E-01	excluded
TRIM14	87033	ES	02:03.1	1	3.2	0.00	7.1E-01	included
HFE	75580	ES	2.1:2.2:3	1	5.1	0.00	7.1E-01	excluded
POLDIP3	62527	ES	3.2:4:6:7.1:7.2:8:9:10.	2	10.2	0.00	7.1E-01	included
ZFYVE27	12739	ES	3:04	2.2	5	0.00	7.1E-01	excluded
COL6A2	257694	ES	17:18	16	19	0.00	7.1E-01	included
ATAD2	85055	ES	27	26	28	0.00	7.1E-01	excluded
ZYG11B	3015	ES	9:10	8	12	0.00	7.1E-01	excluded
PPP6R3	17326	ES	3	1	4	0.00	7.1E-01	excluded
IFI27L1	29071	ES	2	1	4.3	0.00	7.1E-01	excluded
ZKSCAN7	64371	ES	2	1.1	3	0.00	7.1E-01	excluded
MBD4	66721	ES	2.2:3.1:3.2	2.1	4	0.00	7.1E-01	excluded
ATP6V1E1	60980	ES	5	4	6	0.00	7.1E-01	included
KIAA0195	43447	ES	7	6	8	0.00	7.1E-01	excluded
GLIPR2	86325	ES	6	5	7	0.00	7.1E-01	included
ZNF706	84748	ES	3.2:3.4	2	4	0.00	7.1E-01	included
TLE2	46653	ES	10.4:10.5:11	10.1	12	0.00	7.1E-01	included
DUOXA1	30399	ES	2.2:3	1.1	5	0.01	7.1E-01	included
C12orf4	19769	ES	4	3	5	0.00	7.1E-01	excluded
CCDC126	78982	ES	2	1	5	0.00	7.1E-01	excluded
IGF2BP2	68031	ES	11	10	12	0.00	7.1E-01	excluded
HPCAL1	52659	ES	6	5	7	0.00	7.1E-01	included
G3BP1	74193	ES	4	3.2	5	0.00	7.1E-01	included
RBBP6	35644	ES	16	15	17	0.00	7.1E-01	included
TLE4	86664	ES	14:15	13.2	17	0.00	7.2E-01	included
TAX1BP1	79065	ES	15	14	16.1	0.00	7.2E-01	excluded
CTNND1	101761	ES	21	20	22.2	0.01	7.2E-01	included
ZCWPW1	80946	ES	16	15	18	0.00	7.2E-01	included
HDAC11	63484	ES	6.1:6.2:7:8:9	5	10	0.00	7.2E-01	excluded
PPP2R1B	18677	ES	5:06	3	7	0.00	7.2E-01	included
M1AP	54125	ES	4:05	3	6	0.00	7.2E-01	excluded
SMARCC1	64537	ES	4	3	5	0.00	7.2E-01	excluded
KIAA0319L	1724	ES	7	6	8	0.00	7.2E-01	excluded
ADAM15	7895	ES	22.1:22.2	21.2	23	-0.01	7.2E-01	excluded
SGSM2	38396	ES	2	1	3	0.00	7.2E-01	excluded
TEP1	26437	ES	31	30	32	-0.01	7.2E-01	excluded
PGF	28458	ES	5	4	6	0.00	7.2E-01	included
BHMT2	72628	ES	4	3	5	0.00	7.2E-01	excluded
UBAP2	86135	ES	20	19	21.2	0.00	7.2E-01	excluded
ATG13	15599	ES	2.2	1.1	3	0.00	7.2E-01	included
EHBP1	53719	ES	9	8	10	0.00	7.2E-01	excluded
PFDN5	93147	ES	4.2	2	5	-0.01	7.2E-01	excluded
MRPL48	17723	ES	6:08	5	10	0.00	7.2E-01	included
RAVER2	3280	ES	9	8	10.1	0.00	7.2E-01	included
NCDN	1728	ES	1.3	1.1	2	0.00	7.2E-01	included

METTL5	55892	ES	2:03	1.4	4	0.00	7.2E-01	excluded
TARS2	7475	ES	8:09	7	10	0.00	7.2E-01	included
ABCD4	28391	ES	4.1	2	5	0.00	7.2E-01	excluded
DHX38	37545	ES	3:4:5:6:7:8:9:10:11:12<br>:13:14:15:16:17.1:17.	2.2	18	0.00	7.2E-01	excluded
EMC1	893	ES	3	2.1	4	0.00	7.2E-01	excluded
APOL1	62023	ES	4	3.2	5	0.00	7.2E-01	excluded
ZNF226	50293	ES	4	3.3	5	0.00	7.2E-01	excluded
LRWD1	81099	ES	2	1.1	3	0.00	7.2E-01	excluded
ABCD4	28396	ES	2	1	3	0.00	7.2E-01	excluded
ATG13	15598	ES	2.1:2.2	1.1	3	0.00	7.2E-01	excluded
ATG5	77117	ES	5:06	3	7	0.00	7.2E-01	excluded
LSR	49083	ES	07:08.1	6	8.2	0.00	7.2E-01	excluded
ETNK2	9469	ES	4	2	5	0.00	7.2E-01	included
ZKSCAN1	80869	ES	3.1:3.2:3.3:4	1	5	0.00	7.2E-01	excluded
TATDN1	85086	ES	3:4.1:4.2	2	5	0.00	7.2E-01	included
C19orf60	48491	ES	3.2	2	4	0.00	7.2E-01	excluded
ZNF302	48991	ES	04:05.2	2	6.2	0.00	7.2E-01	included
FEZ2	53197	ES	9	7	10	0.00	7.2E-01	excluded
SNF8	42250	ES	4	3	5	0.00	7.2E-01	excluded
SLC41A3	66575	ES	4	3	6	0.00	7.2E-01	excluded
DENND5A	14307	ES	2	1	4	0.00	7.2E-01	included
HNRNPH3	11931	ES	03:04.1	2	5	0.00	7.2E-01	included
ACTN1	28116	ES	21	20	22	0.00	7.2E-01	excluded
DUOX1	30404	ES	13	12.2	14	0.00	7.2E-01	excluded
AP4M1	80891	ES	5	4	6	0.00	7.2E-01	excluded
C9orf3	86947	ES	15	14	16	0.00	7.2E-01	included
HSD3B7	36212	ES	6	5	7	0.00	7.2E-01	included
CRTAP	63841	ES	4	3	5	0.00	7.2E-01	included
EIF1AD	16972	ES	1.2:2.1:2.2:2.3	1.1	2.4	-0.01	7.2E-01	excluded
FAM49B	85157	ES	6:09	4	10	0.00	7.2E-01	excluded
RAP1A	4179	ES	4	2	5	0.00	7.2E-01	excluded
HNRNPAB	74845	ES	5.3:6	5.2	7	0.00	7.2E-01	excluded
TCTN3	12649	ES	8:09	7	10.1	0.00	7.2E-01	excluded
RPS6KA5	28834	ES	3	1	4	0.01	7.2E-01	included
AGTRAP	680	ES	2	1	3	0.00	7.2E-01	included
UCHL1	69107	ES	7	6	8.1	0.00	7.2E-01	excluded
AFG3L2	205724	ES	3	2	4	0.00	7.2E-01	excluded
NEDD1	23837	ES	2.3:3:4.2	2.2	5	0.00	7.2E-01	included
RDH11	28088	ES	5	4	6	0.00	7.2E-01	excluded
PPIL3	56765	ES	4.2	3	6	0.00	7.2E-01	included
UBE4B	596	ES	8	7	9	0.00	7.2E-01	excluded
IREB2	32092	ES	3	2.2	4	0.00	7.2E-01	excluded
NAA60	33536	ES	7	6.2	8	0.00	7.2E-01	excluded
TNIK	67631	ES	17	16	18	0.00	7.2E-01	excluded
GUSB	79858	ES	3.2:4.1:5.1	3.1	6	0.00	7.2E-01	excluded
IREB2	32091	ES	6	5	7	0.00	7.2E-01	excluded
C20orf96	58440	ES	2.2:3	1	4	0.00	7.2E-01	included
ZNF83	51505	ES	9.5:9.6	7	10.1	0.01	7.2E-01	included
PAPD4	72636	ES	2	1.4	3	0.00	7.2E-01	included
DDX50	11972	ES	2.2	1	3	0.00	7.2E-01	included
PLTP	59649	ES	6	5	7	0.00	7.2E-01	excluded
ASMTL	88388	ES	5	4	6	0.00	7.2E-01	included
PAX8	55053	ES	3:04	2	5	0.00	7.2E-01	excluded
NCSTN	8464	ES	7:08:09	6	10	0.00	7.2E-01	excluded
GTDC1	55506	ES	8.1:9	7	10	0.00	7.2E-01	included
GTPBP4	10609	ES	4	3	5	0.00	7.2E-01	excluded
IFI16	8397	ES	8:09	7	10	0.00	7.2E-01	included
YAF2	21137	ES	3.2:5.2:6:7:8	2	9.1	0.00	7.2E-01	included
PTK7	76248	ES	13.2:14	13.1	15	0.00	7.2E-01	included
TRO	89256	ES	12.3	12.1	13	0.01	7.2E-01	included
PANK1	12495	ES	6	5	7	0.00	7.2E-01	included

RAB31	44617	ES	6	1	7	0.00	7.2E-01	excluded
LRRFIP1	58143	ES	6:07	3	13	0.00	7.2E-01	included
ILVBL	48058	ES	4	3	5	0.00	7.2E-01	excluded
CTNND1	15950	ES	3:4.1:4.2	2.1	4.3	0.00	7.2E-01	excluded
FLNA	90564	ES	32	31	33	0.00	7.2E-01	included
VPS16	58563	ES	10:11:12.2:12.4	9	13	0.00	7.2E-01	excluded
DNAJC22	21573	ES	2	1	3	0.01	7.2E-01	included
KCTD15	48947	ES	3	2.3	4	0.00	7.2E-01	included
BCL2L13	96061	ES	6	4	8.1	0.00	7.2E-01	excluded
BDH1	68301	ES	3	2	4	-0.01	7.2E-01	excluded
PARP12	81976	ES	7	6	8	0.00	7.2E-01	included
RNF207	382	ES	11:12	10	13	0.00	7.2E-01	included
SIRT5	75395	ES	5:06	4	7	0.00	7.2E-01	excluded
RNF41	22405	ES	4.2	3	5	0.00	7.2E-01	excluded
KDM4C	85834	ES	5.1	4	6	0.00	7.3E-01	excluded
DDX19B	37352	ES	5.2:6:7	3	9	0.00	7.3E-01	included
PRSS23	18203	ES	4:05	3	6	0.00	7.3E-01	excluded
NARG2	30963	ES	4.1:4.2:5	3	6	0.00	7.3E-01	excluded
IDH3A	32012	ES	10	9	11.1	0.00	7.3E-01	excluded
NOS3	82280	ES	4:05	3	6	0.00	7.3E-01	included
YAF2	21150	ES	4:5.1:5.2:7	2	9.1	0.00	7.3E-01	included
PRMT7	37223	ES	6	5	7	0.00	7.3E-01	excluded
NPRL2	65044	ES	3.1:3.2	2.1	3.4	0.00	7.3E-01	included
MTO1	76749	ES	4:05	3	6	0.01	7.3E-01	included
UQCC1	59089	ES	8:09	7.2	10.1	0.00	7.3E-01	included
NT5C3B	40953	ES	9	8	10	0.00	7.3E-01	excluded
PI4KB	7595	ES	4:05	1	6	0.00	7.3E-01	excluded
CYB5D1	39066	ES	1.2:2:3	1.1	4.1	0.00	7.3E-01	included
ING4	19916	ES	3	2	4	0.00	7.3E-01	excluded
ASB6	87870	ES	4	3	5	0.00	7.3E-01	included
C17orf72	43029	ES	2.1:2.2:3:4.1:4.2	1	5	0.00	7.3E-01	included
PEX26	61023	ES	4	3	5	0.00	7.3E-01	included
CCDC106	52126	ES	2	1.5	3	0.00	7.3E-01	included
HEXA	31546	ES	12	10	13.1	0.01	7.3E-01	included
AKAP9	80412	ES	35:36:00	34.1	37	0.00	7.3E-01	excluded
ZNF605	25320	ES	2	1	3	0.01	7.3E-01	included
SSH3	17167	ES	2.2	1	3.1	0.00	7.3E-01	excluded
SNX11	42178	ES	2:3.1:4:5	1.2	6	0.00	7.3E-01	included
PRDM10	19467	ES	19.1:19.2	18	20	0.00	7.3E-01	excluded
RPP38	91486	ES	2	1.2	3.1	-0.01	7.3E-01	excluded
ZFAND1	84306	ES	07:08.1	6	8.2	-0.01	7.3E-01	excluded
POLR1B	55019	ES	3.1:3.2	1.3	4	0.00	7.3E-01	included
ZNF438	11134	ES	10	8	11.1	0.00	7.3E-01	excluded
RRBP1	290839	ES	3.3:3.4:4:6:7:8:9:10:1<br>1:12:13:14:15:16:17:1	3.2	19	0.00	7.3E-01	included
PDE2A	17617	ES	8:09	7.2	10.1	0.00	7.3E-01	excluded
HACL1	63607	ES	3:4:5:6:7:8:9	2	11	0.00	7.3E-01	included
TRPC6	18432	ES	3:04	2	5	0.00	7.3E-01	included
ATG7	63388	ES	6	5	7	0.00	7.3E-01	excluded
CCDC90B	18077	ES	2:03	1.2	4.2	0.00	7.3E-01	included
TMX2	15894	ES	4	3.3	5.1	0.00	7.3E-01	included
BMP2K	69646	ES	9	8	10	0.00	7.3E-01	excluded
LTBP2	28409	ES	26	25	27	0.00	7.3E-01	excluded
CHRD	67959	ES	15	14	16	0.00	7.3E-01	included
AP1G2	99354	ES	5	4	6	0.01	7.3E-01	included
EIF2A	67286	ES	4	2	5.1	0.00	7.3E-01	excluded
FAM227B	30547	ES	5	4	6	0.00	7.3E-01	included
SUMF1	62987	ES	8	7	9	0.00	7.3E-01	included
COPS7B	57968	ES	3:05	1	6	0.00	7.3E-01	excluded
TLE2	46652	ES	10.3:10.4:10.5:11	10.1	12	0.00	7.3E-01	included
MTMR2	18404	ES	4	3	6	-0.01	7.3E-01	excluded
CD34	9669	ES	3	2	4	0.00	7.3E-01	included

NT5C3A	79216	ES	03:04.2	1	5	0.00	7.3E-01	included
SMARCC2	22394	ES	7	6	8	0.00	7.3E-01	included
ZNF720	36289	ES	4	3	5.1	0.00	7.3E-01	excluded
A2M	20222	ES	3	2	4	0.00	7.3E-01	included
ZNF691	2127	ES	3	2.3	4	0.00	7.3E-01	excluded
DYRK2	22919	ES	2	1	3	0.01	7.3E-01	included
AKAP8	48073	ES	4	3	5	0.00	7.3E-01	excluded
SRSF4	1422	ES	8:09	7	10	0.00	7.3E-01	included
RSL1D1	34046	ES	2.1:2.2	1.1	3	0.00	7.3E-01	excluded
SFXN5	53949	ES	6	5	7	0.00	7.3E-01	excluded
ALKBH8	18571	ES	14:15	13	16.1	0.00	7.3E-01	included
TRMU	62712	ES	4	3	5	0.00	7.3E-01	excluded
FAF1	2939	ES	20	19	21	0.00	7.3E-01	included
TMEM107	39115	ES	3.1:3.2:3.3:3.4:3.5	2	3.7	-0.01	7.3E-01	excluded
HHAT	9700	ES	4:05	2	6	0.00	7.3E-01	included
BCLAF1	77906	ES	13	12	14	0.00	7.3E-01	included
DENND3	85329	ES	9	8	10	0.00	7.3E-01	included
ZNF562	47385	ES	5	4	6.1	0.00	7.3E-01	included
UBE2Q2	31890	ES	11:12	10	13	0.00	7.3E-01	excluded
TMCO6	73701	ES	3	2.1	4.1	0.00	7.3E-01	included
ABI1	11052	ES	2	1	3	0.00	7.3E-01	included
HSPBP1	52054	ES	5.2:6:7:8.1	5.1	8.2	0.00	7.3E-01	included
PSMA5	4011	ES	2	1	3	0.00	7.3E-01	included
FUOM	13554	ES	3	1	4	0.00	7.3E-01	excluded
TMEM57	1190	ES	2:3:4:5:6	1	7	0.00	7.3E-01	excluded
PHKA1	89507	ES	28:29:00	27	30	0.00	7.3E-01	included
CCT5	71557	ES	3:04:05	1.2	6	0.00	7.3E-01	excluded
C15orf40	32248	ES	2:03	1	4	0.00	7.3E-01	excluded
BLVRB	49904	ES	4	3.1	5	0.00	7.3E-01	excluded
PDXDC1	34126	ES	7.1:7.2:8	6.2	9	0.00	7.3E-01	included
ATP6V1D	28052	ES	3:4:5:6	2	7	0.00	7.3E-01	included
APP	60289	ES	4	2	5	0.00	7.3E-01	excluded
TMEM234	1577	ES	2:03	1	5.1	0.00	7.3E-01	included
AASDH	69344	ES	12	11.1	13	0.00	7.3E-01	included
ZBTB25	27887	ES	3:04:05	1	6	0.00	7.3E-01	excluded
WDR11	13289	ES	9	8	10.1	0.00	7.3E-01	excluded
MCCC1	67776	ES	19	17	20	0.00	7.3E-01	excluded
MAN2A2	32515	ES	17.1:17.2	16	18	0.00	7.3E-01	excluded
PITPNA	38340	ES	5:07	4	8	0.00	7.3E-01	excluded
COPS3	39469	ES	7	6.2	8	0.00	7.3E-01	included
UMPS	66538	ES	2	1	3	0.00	7.3E-01	included
SEPT7	79237	ES	9.2:10:11:12:13.1	9.1	13.2	0.00	7.3E-01	included
LMBR1	82495	ES	2.1:4.1	1	5	0.00	7.3E-01	included
MRPL55	10116	ES	2.2:2.8	1.2	2.9	0.01	7.3E-01	included
APBB3	73682	ES	6.6	6.2	6.9	0.00	7.3E-01	excluded
HMBS	19097	ES	12.3	12.1	13	0.00	7.3E-01	excluded
BAIAP2	44100	ES	2:03:05	1	6	0.00	7.4E-01	excluded
MRPL10	42104	ES	2.2	1.1	3	0.00	7.4E-01	excluded
USP8	30597	ES	1.2:2	1.1	3	-0.01	7.4E-01	excluded
AMZ2	43134	ES	1.2:1.4	1.1	1.5	0.00	7.4E-01	excluded
NQO1	37302	ES	5	3	6.1	0.00	7.4E-01	included
ATG10	72683	ES	8	7	9.1	0.00	7.4E-01	excluded
BTN2A1	75672	ES	2	1	3	0.00	7.4E-01	included
TROVE2	9261	ES	3.2	1	4	0.01	7.4E-01	included
TGM1	26940	ES	2.2:3:4:5:6:7:8	2.1	9	0.00	7.4E-01	excluded
PLEKHB2	55380	ES	4.2	1	5	0.00	7.4E-01	excluded
COPZ1	22162	ES	9.2:10.1:10.2:10.3	9.1	10.4	-0.01	7.4E-01	excluded
USP39	54319	ES	12	11	13	0.00	7.4E-01	excluded
ARHGAP42	18420	ES	5	4	6	0.00	7.4E-01	excluded
GALNT2	10236	ES	3	2	4	0.00	7.4E-01	excluded
PDE8A	32346	ES	9	8	11	0.00	7.4E-01	excluded
USP2	19141	ES	2	1	4	0.00	7.4E-01	included

PYCR1	44246	ES	6	5	7	0.00	7.4E-01	included
ODF2	87757	ES	9.2:10	9.1	11	0.00	7.4E-01	excluded
GNB5	30642	ES	6:07	5.1	8	0.00	7.4E-01	included
RPL18	50778	ES	2	1	3.1	0.00	7.4E-01	included
ALDH3B1	17272	ES	9.2:10:11:12:13:14.1	9.1	14.2	0.00	7.4E-01	excluded
HYOU1	19087	ES	4	3	5	0.00	7.4E-01	excluded
ARPP19	30674	ES	4	2.6	5.2	0.00	7.4E-01	included
OBSCN	10202	ES	29	28	30	0.00	7.4E-01	excluded
PIP5K1A	7579	ES	10	9	11	0.00	7.4E-01	included
AGPAT6	83526	ES	5	4	6	0.00	7.4E-01	included
AIMP2	78704	ES	2:04	1.2	5	0.01	7.4E-01	included
CEP70	67001	ES	3.1:3.2:3.3:4:5:6	2	7.1	0.00	7.4E-01	excluded
TATDN3	9759	ES	8	7	9.3	0.00	7.4E-01	included
DMKN	49194	ES	7:08:09	6.4	11	-0.01	7.4E-01	excluded
PDLIM7	74782	ES	6	5	7	0.00	7.4E-01	included
ANXA7	91718	ES	3.1:3.2:4:5	1	7	0.00	7.4E-01	excluded
CSTF3	14888	ES	3	1	4.1	0.00	7.4E-01	included
AP3S1	73040	ES	6:07	5	8	0.00	7.4E-01	excluded
BUB3	13391	ES	5:6:7:8.1	4	8.2	0.00	7.4E-01	excluded
CHCHD4	63517	ES	2:03	1	4	0.00	7.4E-01	excluded
NAA60	33538	ES	6.2	4	7	0.00	7.4E-01	included
JMJD6	43620	ES	6	4	7.1	0.00	7.4E-01	included
FAM189B	8049	ES	2	1	3	0.00	7.4E-01	included
RABL5	97665	ES	3.2	1	5	0.00	7.4E-01	excluded
P4HTM	64788	ES	6.2:7.1	6.1	7.2	0.00	7.4E-01	included
RPS6	214604	ES	1.2:1.4	1.1	1.6	0.00	7.4E-01	excluded
CREB1	57180	ES	7.2:9:12.1	7.1	12.2	0.00	7.4E-01	included
ELL	48443	ES	4	3	5	0.00	7.4E-01	excluded
UBXN11	1263	ES	3	2	4	0.00	7.4E-01	excluded
PBRM1	65234	ES	29	28	30	0.00	7.4E-01	included
TMEM66	83264	ES	2.1:2.2:2.3:2.4	1.2	3	0.00	7.4E-01	excluded
CCDC58	66430	ES	1.2:2:3	1.1	4	0.00	7.4E-01	excluded
CCDC51	64651	ES	1.2:2.1	1.1	2.2	-0.01	7.4E-01	excluded
FCHSD2	17676	ES	4	3	5	0.00	7.4E-01	included
DTNA	45113	ES	16	13	17	0.00	7.4E-01	included
SAE1	50622	ES	9	6	10	0.00	7.4E-01	excluded
WWP2	37322	ES	2.2	1.1	4.3	0.00	7.4E-01	included
STPG1	1106	ES	4	3.2	5	0.01	7.4E-01	included
PRRC2C	8990	ES	23:24	22	25	0.00	7.4E-01	excluded
RGN	88902	ES	5	4	6	-0.01	7.4E-01	excluded
MFAP3	74208	ES	2	1.1	3	0.00	7.4E-01	excluded
ACSF2	42388	ES	5.2:6.1	5.1	6.2	0.00	7.4E-01	excluded
EPB41L2	77546	ES	18:20.1:20.2:21	17	22	0.00	7.4E-01	included
VAV2	88083	ES	28	27	29	0.00	7.4E-01	included
R3HDM1	55446	ES	4	3	7	0.00	7.4E-01	excluded
NFIA	3225	ES	13	12	14	0.00	7.4E-01	excluded
FAM126A	78946	ES	11	10	12	-0.01	7.4E-01	excluded
PIGU	59032	ES	3	2	4	0.00	7.4E-01	excluded
KDM4B	46833	ES	13	10	14	0.00	7.4E-01	included
VAPB	59961	ES	3:04	2	5	0.00	7.4E-01	excluded
ANKMY1	58253	ES	14:15	13	16	0.00	7.4E-01	excluded
GYG1	67206	ES	6	5	8	0.00	7.4E-01	included
FAM134A	57616	ES	2	1	3	0.00	7.4E-01	included
UBE2N	23688	ES	4.4	4.2	4.6	0.00	7.4E-01	included
ZNF506	48686	ES	6:7.1:7.2:7.3	3	8	0.00	7.4E-01	included
DDX19A	37373	ES	8.1	5.2	9	0.00	7.4E-01	included
RPS15A	34262	ES	3.3	2.2	5.1	0.00	7.4E-01	excluded
OGDHL	11525	ES	2:03	1	4	0.00	7.4E-01	included
PAIP1	71960	ES	11	10	12	0.00	7.4E-01	included
SDHAF2	16224	ES	5.1:5.2	4.1	6	0.00	7.4E-01	included
MARS	22609	ES	4:5.1:5.2:6	3	7.1	0.00	7.4E-01	included
DHRS4L2	26801	ES	5:06:07	4	8.1	0.01	7.4E-01	included

EPN2	39698	ES	09:10.1	8	10.2	0.00	7.4E-01	included
PAPOLA	29209	ES	5:06	4	7.1	0.00	7.4E-01	excluded
CRCP	79872	ES	5	3	6	0.00	7.4E-01	included
NT5C2	12994	ES	4	2	5	0.00	7.4E-01	excluded
D2HGDH	95816	ES	7.3	7.1	8	0.01	7.4E-01	included
VPS4B	45714	ES	8	7	9	0.00	7.4E-01	excluded
LARP1B	70571	ES	5	4	6	0.00	7.4E-01	included
ST3GAL4	19398	ES	9	8.2	10	0.00	7.4E-01	included
CCDC84	19051	ES	9	8	10	0.00	7.4E-01	included
MFF	57817	ES	3	1	5	0.00	7.4E-01	excluded
TRAF3	29425	ES	7:08:09	6	10	0.00	7.4E-01	excluded
PFDN5	93153	ES	02:04.2	1	5	0.00	7.4E-01	included
DDX31	87986	ES	20:21	19.2	22	0.00	7.4E-01	included
AMACR	71703	ES	6	4.2	7.1	0.00	7.4E-01	excluded
EPB41L5	55147	ES	25.1:25.2	24	26	0.00	7.4E-01	excluded
DOK4	36571	ES	4	3	5	0.00	7.4E-01	included
KLHL36	37854	ES	3.2:4	3.1	5	0.00	7.4E-01	excluded
LSM14A	48954	ES	5	4	6	0.00	7.4E-01	excluded
DPP8	31171	ES	18	17	19	-0.01	7.4E-01	excluded
SMPD4	55324	ES	3:4.1:4.2:5:6:7:8	2	9	0.00	7.4E-01	included
PORCN	88980	ES	7:08	6	9	0.00	7.4E-01	excluded
KANSL3	54548	ES	6:07:08	5.2	9	0.00	7.5E-01	included
TPM1	30991	ES	6	5.2	7	0.00	7.5E-01	included
C19orf66	47452	ES	4.1:4.2:4.3	3	5	0.00	7.5E-01	excluded
FKBP5	75920	ES	9	8	10.1	0.00	7.5E-01	excluded
SMIM7	48195	ES	05:06.1	2	7.1	0.00	7.5E-01	included
NUDT7	37666	ES	3.1	2	5	-0.01	7.5E-01	excluded
CD40	59661	ES	6	5	7	0.00	7.5E-01	included
RPS6KA1	1290	ES	8:09	4	10	0.00	7.5E-01	excluded
SLC27A2	30570	ES	4	3	5	0.00	7.5E-01	included
OPTN	215727	ES	12	11	13	0.00	7.5E-01	included
MAPKAP1	87586	ES	3	1	4	-0.01	7.5E-01	excluded
ACOT8	59627	ES	3.2	2	4	0.00	7.5E-01	included
APAF1	23874	ES	8:9:10:11:12:13:14:15<br>:16:17:18:19:20:21:22	7	27	0.00	7.5E-01	excluded
TJAP1	76279	ES	2:03	1	5	0.01	7.5E-01	included
ADCK5	85595	ES	2	1	3	0.00	7.5E-01	included
EIF4E	70013	ES	3	1	4	0.00	7.5E-01	excluded
VPS37A	82796	ES	4	1	5	0.00	7.5E-01	excluded
SEZ6L2	35978	ES	4:05	3.2	6	0.00	7.5E-01	included
SAP30BP	43481	ES	9	8	10	0.00	7.5E-01	excluded
MNT	38399	ES	2:03:04	1	5	0.00	7.5E-01	included
VDAC3	83721	ES	8.2:9:10:11.1	8.1	11.2	0.00	7.5E-01	excluded
DNAJA3	33721	ES	2:03	1	4	0.00	7.5E-01	excluded
MAL	54481	ES	3	2	4	0.00	7.5E-01	included
FES	32501	ES	10.1:10.2	9	11.2	0.00	7.5E-01	excluded
SEPT2	58369	ES	3:04:07	2	9	0.00	7.5E-01	excluded
DCTD	71249	ES	2.1:2.2:3	1.1	5	0.00	7.5E-01	included
TRPC4	25683	ES	9.5:10.1:10.2:10.3:10.	9.4	10.6	0.00	7.5E-01	excluded
PLEKHM1	41976	ES	4.1:4.2	3	5	-0.01	7.5E-01	excluded
SHQ1	65620	ES	3.2	1	4	0.00	7.5E-01	excluded
ATG4D	47535	ES	5:06	3.2	7	0.00	7.5E-01	included
TAMM41	63411	ES	2	1	3	0.00	7.5E-01	excluded
MSRA	82605	ES	6	5	8	0.00	7.5E-01	excluded
NOX4	18237	ES	16:17:18:19:20	15	22.1	0.00	7.5E-01	included
VPS26A	11979	ES	10	9	11	0.00	7.5E-01	included
HAUS2	30192	ES	3:04	2	5.1	0.00	7.5E-01	excluded
PEPD	48939	ES	8:09	7	10	0.00	7.5E-01	excluded
MDM2	23089	ES	5.1:5.2:6:7:8.1:8.2	3	9	0.00	7.5E-01	excluded
FAM45A	13253	ES	5	4	6	0.00	7.5E-01	excluded
FLYWCH1	33316	ES	7:08	6	9	0.00	7.5E-01	included
C17orf72	43027	ES	4.2:5	4.1	6	0.00	7.5E-01	included

LUC7L	119069	ES	9	8	10.1	0.00	7.5E-01	included
COL3A1	266550	ES	36.1:36.2:37:38:39:40:41:43:44:47	35	48.1	0.00	7.5E-01	included
CAP2	75436	ES	6:07:08	4	10	0.00	7.5E-01	included
ZFYVE20	63552	ES	10	9	11	0.00	7.5E-01	included
PLEK2	28059	ES	3	2	4	0.00	7.5E-01	included
ASB8	21439	ES	5.2	4.1	5.4	0.00	7.5E-01	excluded
PLA2G7	76425	ES	3	2	4	0.00	7.5E-01	included
SPATA6	2921	ES	10	9	11	0.00	7.5E-01	excluded
ACOT8	59626	ES	3.1:3.2	2	4	0.00	7.5E-01	excluded
ATP2B1	23648	ES	19	18	20	0.00	7.5E-01	excluded
NT5DC2	65227	ES	4	3	5	0.00	7.5E-01	excluded
PPCDC	31834	ES	3.2	2	4	0.00	7.5E-01	excluded
ZNF223	50275	ES	8	7.1	9.1	0.01	7.5E-01	included
SLC22A5	73268	ES	3:04	1	5	0.00	7.5E-01	included
LAIR1	51878	ES	6.1	4.4	7.1	-0.01	7.5E-01	excluded
MINK1	38600	ES	2	1	3	0.00	7.5E-01	excluded
TESK2	2683	ES	11	10	12	0.00	7.5E-01	included
PDE2A	17616	ES	30:31:32:33:34.1	29	34.2	0.00	7.5E-01	included
LPCAT3	20063	ES	6	5.1	7	0.00	7.5E-01	included
ORC4	55530	ES	2.2:4:5:6	2.1	7	0.00	7.5E-01	included
LGALS9	39857	ES	6	4	7	0.00	7.5E-01	excluded
ANP32E	7435	ES	5.1:5.2:6	4	7	0.00	7.5E-01	excluded
RNF216	78679	ES	4:5.2:6.1:6.2	2	7	0.00	7.5E-01	excluded
DCN	23662	ES	7:08:09	3	10	0.00	7.5E-01	included
C19orf55	49293	ES	2:03	1	4	0.00	7.5E-01	included
SOAT1	9111	ES	2	1	3	0.00	7.5E-01	included
UBE2F	58155	ES	13	12.1	14	0.00	7.5E-01	included
TOP2B	63749	ES	25	24	27	0.00	7.5E-01	included
KLHDC4	37968	ES	4.1:4.2:6.1:6.2	1	7.1	0.00	7.5E-01	excluded
CALM3	50566	ES	6.3:7:8.1	6.2	8.2	0.00	7.5E-01	included
TSC2	33194	ES	27:28.1	26	28.2	0.00	7.5E-01	included
GYPC	55181	ES	2	1	3	0.00	7.5E-01	included
MARCH6	71567	ES	2:03:04	1	5	0.00	7.5E-01	excluded
GTF2H2	72438	ES	5	4	6	0.00	7.5E-01	excluded
PPP3CA	70095	ES	3:4:5:6:7:8	2	9	0.00	7.5E-01	excluded
RARS	74467	ES	2	1	3	0.00	7.5E-01	excluded
AKIRIN1	1866	ES	2	1	3	0.00	7.5E-01	included
E4F1	33235	ES	8.2:9:10:11.1	8.1	11.2	0.00	7.5E-01	excluded
SNX17	52992	ES	9	8	10	0.00	7.5E-01	included
CIAPIN1	36552	ES	6	5	7	0.00	7.5E-01	included
BACE2	60650	ES	8	7	9	0.00	7.5E-01	excluded
COPS7B	57958	ES	4.1:4.2:4.3:4.4	3	5	-0.01	7.5E-01	excluded
ATXN3	28920	ES	9	8.2	10.1	0.00	7.5E-01	excluded
RABL2B	62928	ES	2.2:2.3:3.1	1	3.2	0.00	7.5E-01	excluded
YWHAE	38295	ES	2:3:4:5:6	1	7	0.00	7.5E-01	excluded
CDK10	38126	ES	4	2.1	5	0.01	7.5E-01	included
SEC22A	66466	ES	3.1:3.2:4:5	2.2	6	0.00	7.5E-01	included
VTI1B	28084	ES	3	1	4	0.00	7.5E-01	excluded
C20orf24	59292	ES	4.1	3	5	0.00	7.5E-01	included
BLVRB	49903	ES	3.2:4	3.1	5	0.01	7.5E-01	included
RDH13	52003	ES	8	7	9.1	0.00	7.5E-01	included
MBIP	27247	ES	7	6	8	0.00	7.6E-01	included
ECHDC2	3033	ES	5.1:6.1	2.1	6.2	0.00	7.6E-01	excluded
DCTN5	35626	ES	4	2	5	0.01	7.6E-01	included
C3orf17	66158	ES	3.1:4.1:4.2:5.1:6.1:6.2:	1	8	0.00	7.6E-01	excluded
EDNRA	70788	ES	3:04	2	5	0.00	7.6E-01	excluded
MDH1B	57153	ES	5:06	4.2	7	0.00	7.6E-01	excluded
FOSB	50458	ES	2.2:2.3:2.5	1	3.1	0.00	7.6E-01	included
TATDN1	138627	ES	3:4.1:5:6	1.1	7	-0.01	7.6E-01	excluded
SEPHS1	10795	ES	8	7	10	0.00	7.6E-01	excluded
TBC1D22A	62729	ES	5:06	1	7	0.00	7.6E-01	included

TMEM161B	72745	ES	3	1	4	0.00	7.6E-01	excluded
SF1	16687	ES	1.3	1.1	3	0.00	7.6E-01	excluded
ARHGAP12	11155	ES	5	4	6	0.00	7.6E-01	excluded
SMURF2	43073	ES	7	6	8	0.00	7.6E-01	excluded
NBPF3	968	ES	3:4:5:6	2	7	0.00	7.6E-01	excluded
WNT5B	19669	ES	6	5	7	0.00	7.6E-01	excluded
TMX2	15911	ES	3.1:3.2:3.3:4	2	5.1	0.01	7.6E-01	included
TNFAIP2	29437	ES	4	3.2	5	0.00	7.6E-01	excluded
ZNF791	47814	ES	2.1	1	3	0.00	7.6E-01	excluded
BTAF1	12524	ES	25:26.1	24	26.2	0.00	7.6E-01	excluded
SDHC	8666	ES	2	1	5	0.01	7.6E-01	included
PMM2	33931	ES	6	3	7	0.00	7.6E-01	included
B2M	30360	ES	1.2:2.1:2.2	1.1	2.3	0.00	7.6E-01	included
C3orf17	66155	ES	2:3.1:3.2:4.1:4.2:5.1:6.	1	8	0.01	7.6E-01	included
GIT2	24377	ES	17.2:18.2	17.1	19	0.00	7.6E-01	excluded
EAF1	63577	ES	3	2	4	0.00	7.6E-01	included
GNB2L1	190582	ES	4.2:5:6	3	9	0.00	7.6E-01	excluded
TXNL4A	46290	ES	2	1	7.2	0.00	7.6E-01	included
ECHDC1	77470	ES	5	4	6.1	0.00	7.6E-01	included
KLHDC4	37979	ES	2	1	4.1	0.00	7.6E-01	included
SULT1A1	35819	ES	4	3	6.2	0.00	7.6E-01	included
HNRNPC	26555	ES	2.4:2.6	1	3.2	0.00	7.6E-01	excluded
SLC35F2	18593	ES	7	4.2	8	0.00	7.6E-01	excluded
SDCBP	83932	ES	6.2:7.1	5	7.2	0.00	7.6E-01	excluded
SLC35B2	76380	ES	3	2	4	0.00	7.6E-01	included
GPR64	88617	ES	29	28	30	0.00	7.6E-01	excluded
ZBTB44	19500	ES	6	5.1	7.1	0.00	7.6E-01	included
TRIM4	80865	ES	2	1	3	0.00	7.6E-01	included
ATP11A	26310	ES	29:30.1:30.2	28	30.3	0.00	7.6E-01	included
TFRC	68219	ES	3	2	4	0.00	7.6E-01	included
CLYBL	26189	ES	4	3	5	0.00	7.6E-01	included
KLHDC2	27440	ES	10	9	11	0.00	7.6E-01	excluded
CDK1	11856	ES	5.1	4	6.1	0.00	7.6E-01	included
EIF3C	190575	ES	2.4:3:4:5.2:6:7:8:9:10:	1	13	0.00	7.6E-01	excluded
ASCC2	61675	ES	7:8:9.1:9.2	5	10	0.00	7.6E-01	excluded
TUBG1	41101	ES	5	4	6	0.00	7.6E-01	excluded
UNC13B	86247	ES	38	37	39	0.00	7.6E-01	included
AFAP1L1	74027	ES	18	17	19	0.00	7.6E-01	excluded
KCTD17	62079	ES	7	6	8	-0.01	7.6E-01	excluded
IFI27L1	29060	ES	6:7.1:7.2	4.3	8	0.00	7.6E-01	excluded
POLDIP3	62528	ES	4:6:7.1:7.2:8:9:10.1	2	10.2	0.00	7.6E-01	included
PFKFB2	9620	ES	4	3.2	5	0.00	7.6E-01	excluded
PCK2	26826	ES	2.3:3	2.1	4	0.00	7.6E-01	excluded
ELN	80042	ES	25	24.2	26.1	0.00	7.6E-01	included
MTG1	13560	ES	6:07	5	8	0.00	7.6E-01	excluded
SOAT1	9112	ES	2:03	1	4	0.00	7.6E-01	excluded
MRC2	42910	ES	24	23	25	0.00	7.6E-01	included
POLK	72531	ES	13.1	12	14.1	0.00	7.6E-01	excluded
CAMK2G	12248	ES	15	14	16	0.00	7.6E-01	excluded
PSMA2	79322	ES	2:03	1	4	0.00	7.6E-01	included
RBMS2	22470	ES	2	1	3	0.00	7.6E-01	included
TMPRSS13	18952	ES	3	2	4	0.00	7.6E-01	excluded
ZNF561	47369	ES	5.2	4	6.2	0.00	7.6E-01	included
KHDRBS3	85274	ES	4:5:6:7	3	8	0.00	7.6E-01	included
DUS2	37175	ES	6:07	5	8	0.00	7.6E-01	included
CNIH1	27580	ES	6.1	5	7	0.00	7.6E-01	excluded
MSRB1	33165	ES	2.3	2.1	2.5	0.00	7.6E-01	included
PPARA	62688	ES	7	6	8	0.00	7.6E-01	excluded
GGT5	115560	ES	8:9:10.1:10.2	7	11	0.00	7.6E-01	excluded
SLC35A3	3864	ES	3	1	4	0.01	7.6E-01	included
CAPN10	58284	ES	2:3.1:3.2:4:5:6:7:9:10.	1	11	0.00	7.6E-01	excluded
MED15	61184	ES	5:07	3.1	8	0.00	7.6E-01	included

CXorf23	88651	ES	11	10.2	12	0.00	7.6E-01	excluded
CES4A	36915	ES	12	11	13	0.00	7.6E-01	excluded
NME6	64584	ES	5.1:5.2:6	4	7	0.00	7.6E-01	included
ANKS3	33805	ES	7.2	6	8	0.00	7.6E-01	included
RNF185	61830	ES	4:05	3	6	0.00	7.6E-01	excluded
FAM57A	38252	ES	3	2	4	0.00	7.6E-01	excluded
SLC30A6	53162	ES	3:04:06	2	8	0.00	7.6E-01	included
HSDL2	87247	ES	4:05	3	6	0.00	7.6E-01	excluded
TP53BP2	9931	ES	2:03	1	4	0.00	7.6E-01	excluded
MAATS1	66356	ES	2:03	1.1	4	0.00	7.6E-01	excluded
USMG5	13003	ES	3	1	4	0.00	7.6E-01	excluded
ARMC2	77153	ES	3	2	4	0.00	7.7E-01	excluded
DDX19B	37353	ES	6:07	3	9	0.00	7.7E-01	included
TSFM	22760	ES	7	6	8	0.00	7.7E-01	excluded
STAU2	84180	ES	4:07:08	2	11	0.00	7.7E-01	included
KDM1A	1031	ES	10	9	12	0.00	7.7E-01	included
AP2M1	67842	ES	3:04	2.2	5	0.00	7.7E-01	included
IQCK	34339	ES	3	2	4	0.00	7.7E-01	excluded
CDK7	72336	ES	3	2.2	4	0.00	7.7E-01	excluded
RABL2A	55064	ES	2	1	3.1	-0.01	7.7E-01	excluded
MBD1	45513	ES	18.1	17	18.5	0.00	7.7E-01	included
TMEM144	70983	ES	7	6.1	8	0.00	7.7E-01	included
NOL6	86121	ES	4	3	5	0.00	7.7E-01	excluded
VMP1	42799	ES	4:05	3	6	0.00	7.7E-01	excluded
SERP2	25781	ES	4	3	6	0.00	7.7E-01	excluded
TMEM117	21282	ES	7	6	8	0.00	7.7E-01	included
BTBD11	24194	ES	10:11	9	12	0.00	7.7E-01	excluded
POFUT1	58943	ES	2:03:04	1	6	0.00	7.7E-01	excluded
VEZT	23765	ES	05:06.1	4	6.2	0.00	7.7E-01	excluded
MFSD10	68615	ES	10:11	9	12.1	0.00	7.7E-01	excluded
PTRH1	87649	ES	5	4	6	0.00	7.7E-01	excluded
PTPRK	77499	ES	17	16.2	18	0.00	7.7E-01	included
RFFL	40234	ES	9	8	10	0.00	7.7E-01	included
PHC1	20216	ES	4	3	5	0.00	7.7E-01	included
RAD18	63077	ES	8	7	9	0.00	7.7E-01	excluded
WBP2	43521	ES	7	6	8.1	0.00	7.7E-01	excluded
ST7L	4213	ES	7:08	6	9	0.00	7.7E-01	included
BEST1	16322	ES	6:7:8.1:8.2:9:10	5	11.1	0.00	7.7E-01	excluded
RBM38	59896	ES	5	4	6	0.00	7.7E-01	included
METTL23	43655	ES	1.3:1.4	1.1	2	0.00	7.7E-01	included
RPS15A	34259	ES	3.3:4	2.2	5.1	0.00	7.7E-01	excluded
LYST	10364	ES	13	12	14	0.00	7.7E-01	included
SLC38A9	72055	ES	7	3.2	8	0.00	7.7E-01	excluded
EWSR1	61585	ES	7	6	8	0.00	7.7E-01	excluded
APEH	64894	ES	2:03	1	4	0.00	7.7E-01	excluded
ERCC2	50432	ES	6:07:08	5	9	0.00	7.7E-01	excluded
NEK9	28487	ES	2	1	3	0.00	7.7E-01	included
NAA40	16549	ES	5:06:07	4.2	8	0.00	7.7E-01	included
NDEL1	39193	ES	5.2:6:7:8:9:10:12.1	5.1	12.2	0.00	7.7E-01	excluded
TBC1D1	69018	ES	14	12	15	0.00	7.7E-01	excluded
USMG5	13002	ES	2.2:3	1	4	0.00	7.7E-01	included
STEAP4	80363	ES	4.1	3	5	0.00	7.7E-01	excluded
PILRB	80931	ES	11:12.1	9	12.2	0.00	7.7E-01	included
DDX24	29052	ES	2.1:2.2	1	3	0.00	7.7E-01	excluded
NACA	22489	ES	3.5	3.3	4.2	0.00	7.7E-01	included
ASNSD1	300784	ES	2:3.1:3.2:3.3:4.1:4.2:5	1	6	0.00	7.7E-01	excluded
CPSF6	23309	ES	2.2:4:5:6.1:6.2:7:8.1:8.2:9:10:11.1	2.1	11.2	0.00	7.7E-01	excluded
PDXDC1	34124	ES	7.1:7.2	6.2	8	0.00	7.7E-01	included
ZNF664	25125	ES	3	2.2	4	0.00	7.7E-01	included
NUDT7	37667	ES	03:01.1	1	5	0.00	7.7E-01	excluded
GPR89A	7317	ES	3	1	4	0.00	7.7E-01	included

PEX16	15523	ES	4	3	5	0.00	7.7E-01	excluded
CTNND1	16005	ES	4.3	2.1	5	0.00	7.7E-01	included
DHRS4L2	26806	ES	4	3	8.1	0.00	7.7E-01	excluded
CEP78	86657	ES	15	14	16.1	0.01	7.7E-01	included
FASTK	82340	ES	1.2:2	1.1	3.1	0.00	7.7E-01	excluded
WDR45B	44413	ES	4	3	5	0.00	7.7E-01	excluded
MX2	60662	ES	7	6	8	0.00	7.7E-01	included
KLHL8	69856	ES	4.1:4.2:5	3	6	0.00	7.7E-01	included
PHB2	20048	ES	3.1:3.2:4.1:4.2:5.1:5.2:<br>5.3:6.1:6.2	2	7.1	0.00	7.7E-01	included
BSDC1	1600	ES	4.1:4.2:4.3	3	5	0.00	7.7E-01	included
RBM6	64939	ES	3.1:3.2	2	4	0.01	7.7E-01	included
SLC25A45	16830	ES	7	6.2	8	0.00	7.7E-01	included
ZNF544	52429	ES	7.1:7.2:9:10.1	6.2	10.2	0.00	7.7E-01	excluded
SPATA2	59753	ES	3	1	4	0.00	7.7E-01	excluded
OPTN	10784	ES	3	2	5.2	0.00	7.7E-01	excluded
MRPL55	10152	ES	1.2:2.4:2.5:2.6	1.1	2.9	0.00	7.7E-01	excluded
PDGFC	70959	ES	2	1	3	0.00	7.7E-01	included
BCL7C	36195	ES	03:04.1	2	4.2	0.00	7.7E-01	excluded
LDB2	68841	ES	10	9.2	11.2	0.00	7.7E-01	excluded
CPSF3L	120	ES	4:5.1:5.2:6.1:6.2	1	7.1	-0.01	7.7E-01	excluded
CREM	11293	ES	4	1	15	-0.01	7.7E-01	excluded
GTPBP8	66130	ES	4:05	1	6	0.00	7.7E-01	excluded
AFF1	69852	ES	4:05	3	6	0.00	7.7E-01	included
SUPT20H	25661	ES	26	25	27	0.00	7.7E-01	excluded
DDHD2	83386	ES	4	3	5	0.00	7.8E-01	excluded
SH3GLB2	87811	ES	8	6	9	0.00	7.8E-01	included
TXNDC11	34032	ES	4	3	5	0.00	7.8E-01	included
VEGFA	76327	ES	7.1:7.2:7.3:8.1:8.2	6	9.1	0.00	7.8E-01	included
CLPP	47025	ES	4	3	5	0.00	7.8E-01	included
GGCX	54288	ES	2	1	3.1	0.00	7.8E-01	excluded
DIP2A	60944	ES	6.2	5	7	0.00	7.8E-01	excluded
MRPL21	17345	ES	2.1:2.2	1	3	0.00	7.8E-01	included
KIAA0196	85121	ES	2:03	1	4	0.00	7.8E-01	included
RNF13	67241	ES	3	1	4	0.00	7.8E-01	excluded
SUMF2	79787	ES	8:09	7	10.2	0.00	7.8E-01	excluded
ZNF468	51634	ES	3.2	2	4	0.00	7.8E-01	excluded
EMCN	70092	ES	5	4	6	0.00	7.8E-01	included
ADCK4	49952	ES	6.3:7:8	6.2	9	0.00	7.8E-01	included
LTBP1	53183	ES	26	25	27	0.00	7.8E-01	included
CLN6	31361	ES	5.1	4	6	0.00	7.8E-01	excluded
SS18	44961	ES	4:8.1:8.2	3	9	0.00	7.8E-01	excluded
POLR2J3	273766	ES	2	1	4.3	0.00	7.8E-01	included
EPS8L1	52014	ES	5	4	6	0.00	7.8E-01	excluded
DNAJC3	26127	ES	5	4	6	0.00	7.8E-01	included
CLPB	17603	ES	1.3:2:4.1	1.2	4.2	0.00	7.8E-01	included
ETV1	78834	ES	15	14	16	0.00	7.8E-01	excluded
ADAM8	13522	ES	22	21	23	0.00	7.8E-01	included
RNASE1	26468	ES	2.2:2.3:3.1	1	3.2	0.00	7.8E-01	included
COX10	39336	ES	4	3.1	5	0.00	7.8E-01	excluded
MEF2A	32718	ES	5	4.2	6	0.00	7.8E-01	included
SCFD1	27067	ES	5.2	4	6	0.00	7.8E-01	included
TSC2	33193	ES	27	26	28.1	0.00	7.8E-01	included
PDHA1	88633	ES	7	6.1	8	0.00	7.8E-01	excluded
FLVCR2	28513	ES	7:8:9:10:11:12	4	13	0.00	7.8E-01	excluded
PODXL	81822	ES	4.2:4.4:4.5:4.6:4.7	4.1	4.8	0.00	7.8E-01	included
VMP1	42801	ES	4	3	5	0.00	7.8E-01	excluded
GPR89A	7311	ES	8	7.2	9	0.00	7.8E-01	excluded
ACVRL1	21873	ES	3:04:05	2.2	6	0.00	7.8E-01	included
FAM13B	73499	ES	19	18	20	0.00	7.8E-01	excluded
GFM2	72503	ES	14	13	15	0.00	7.8E-01	included
IRF3	51025	ES	1.4:1.5	1.1	2	-0.01	7.8E-01	excluded

CFLAR	56800	ES	3.2	1	5.2	0.00	7.8E-01	excluded
CSDE1	4336	ES	3	2	4	0.00	7.8E-01	excluded
BRF1	29621	ES	12.2:12.3:13.2:14.1:14<br>.2:15.2:16:17:18:19:2	12.1	20.2	0.00	7.8E-01	included
ZNF544	52431	ES	7.1:7.2	6.2	10.2	0.00	7.8E-01	included
SEC31A	69732	ES	26.1	25.1	28	0.01	7.8E-01	included
PML	31660	ES	5:6.1:6.2	4	6.4	0.00	7.8E-01	included
CDK7	72335	ES	4	2.2	5.1	0.00	7.8E-01	included
GUSB	79859	ES	3.1:3.2:4.1:4.2	2	5.1	0.01	7.8E-01	included
TMEM67	84540	ES	02:03.1	1.3	3.2	0.00	7.8E-01	excluded
ATP2B1	23647	ES	21	20	22	0.00	7.8E-01	excluded
STAT3	41037	ES	2.2:2.3	1	3	0.00	7.8E-01	included
ANKEF1	58684	ES	2	1	3	0.00	7.8E-01	included
CC2D2B	12662	ES	15:16:17:18:19:20	14	21	-0.01	7.8E-01	excluded
EPB41L2	77538	ES	20.2:21	20.1	22	0.00	7.8E-01	excluded
SLC25A29	29261	ES	3.2:3.5	2	3.7	0.00	7.8E-01	excluded
CHN1	56052	ES	7	6	8.2	0.00	7.8E-01	included
CD44	14988	ES	6:7:8:9.2:10:11:12.1:1	5	16.1	0.00	7.8E-01	included
DLST	28435	ES	06:07.1	5.2	7.2	0.00	7.8E-01	included
ZBTB49	68658	ES	4:5:6:7	3.2	8	0.00	7.8E-01	excluded
PRDX1	2693	ES	2.2:3:4.1	2.1	4.2	0.00	7.8E-01	excluded
XRRA1	17795	ES	10:11:12	9	13	0.00	7.8E-01	excluded
TBCEL	19170	ES	6.1:6.2:7	5	8	0.00	7.8E-01	excluded
BAIAP2	44101	ES	3:05	1	6	0.00	7.8E-01	excluded
NKG7	51323	ES	2.4	2.2	3	0.00	7.8E-01	included
TG	319503	ES	25:27:28:29:31:32:33:<br>34:35:36:37:38:39:40:	24	46	0.00	7.8E-01	excluded
ZNF23	37436	ES	6.1	5.2	7.1	-0.01	7.8E-01	excluded
GBA	8043	ES	4:05	3	6	0.00	7.8E-01	excluded
SH2B1	35881	ES	3.6	3.2	4.1	0.00	7.8E-01	excluded
SEPSECS	68957	ES	2	1	3	-0.01	7.8E-01	excluded
RANBP10	37105	ES	7	3	8	0.00	7.8E-01	included
FCGRT	50960	ES	5:06	4	7	0.00	7.8E-01	included
COX16	28178	ES	3	1	4	0.00	7.8E-01	included
SLC39A9	28150	ES	3	2	4	0.00	7.8E-01	included
SCMH1	2058	ES	6	5	8	0.01	7.8E-01	included
CLTA	86333	ES	5	4	7.1	0.00	7.8E-01	included
ERG	60594	ES	10	9	11.1	0.00	7.8E-01	included
C1orf50	2115	ES	1.2:1.3:2.2	1.1	3	0.00	7.8E-01	included
PTPLAD1	31187	ES	4:05	1.2	7	0.00	7.8E-01	excluded
DPH2	2496	ES	4	3.2	5	0.00	7.8E-01	excluded
EPHX2	83166	ES	2.1:2.2	1	3	0.00	7.8E-01	included
GAPVD1	87568	ES	16	15	17	0.00	7.8E-01	included
RBM6	64942	ES	3.2:4:5:6:7	2	8	0.00	7.8E-01	included
ISCU	24244	ES	2	1	3	0.00	7.8E-01	included
MROH6	85426	ES	12	11	13	0.00	7.8E-01	excluded
MED6	28184	ES	6	5.2	7	0.00	7.8E-01	included
MARK4	50420	ES	16	15	17	0.00	7.8E-01	excluded
ASNSD1	300790	ES	2:3.3:4.1:5	1	6	0.00	7.8E-01	included
NSMCE2	85126	ES	6	3	7	0.00	7.8E-01	included
EIF2AK4	29936	ES	15	14	16	0.00	7.8E-01	excluded
CRTC2	7761	ES	1.2:2:3:4:5:6:7:8:9:10:	1.1	11.2	0.00	7.8E-01	excluded
METTL23	43633	ES	2:03	1.3	4.1	0.00	7.8E-01	excluded
GALK2	30535	ES	2.1:2.2	1	6	0.00	7.8E-01	excluded
UBE2W	84193	ES	7	6	9	0.00	7.8E-01	included
CPSF7	16201	ES	5.1:5.2:6.1:6.2:6.3	3	7.1	0.00	7.8E-01	excluded
MOB4	56703	ES	4	2	5.1	0.00	7.8E-01	included
KDM5C	89194	ES	4:05	3	6	0.00	7.8E-01	included
KANSL3	54550	ES	6:07	5.2	9	0.00	7.8E-01	included
PIGX	68260	ES	5	4	6.1	0.00	7.8E-01	excluded
RPS6KC1	9783	ES	6:07	5	8	0.00	7.8E-01	included
BBX	66007	ES	16:17	15	18	0.00	7.8E-01	excluded

F11R	8521	ES	7	6	8	0.00	7.8E-01	included
FKBP4	19714	ES	2	1	3	0.00	7.8E-01	excluded
FBLN7	54982	ES	5	4	6	0.00	7.8E-01	included
STAT1	56597	ES	3	2.2	4	0.00	7.8E-01	included
RNASE1	26471	ES	2.2:2.3	1	3.1	0.00	7.8E-01	included
SOAT1	9109	ES	3	2	4	0.00	7.8E-01	included
UBXN11	101230	ES	3:4:5:6	2	7	0.00	7.8E-01	included
CPSF3L	81	ES	10:11.1	9	11.2	0.00	7.8E-01	included
UPP1	79644	ES	1.2:2.1:2.2	1.1	2.3	0.00	7.8E-01	included
ADAP2	40133	ES	3.2	2	4	0.00	7.9E-01	included
CLN6	31360	ES	5.1:5.2	4	6	0.00	7.9E-01	excluded
ZNF177	47313	ES	4:05:06	3	7	0.00	7.9E-01	included
ZNF528	51458	ES	5	4	6	0.00	7.9E-01	excluded
TIMM50	49835	ES	5	4	6	0.00	7.9E-01	excluded
SUMO1	56931	ES	7	6.2	8.1	0.00	7.9E-01	excluded
GPR132	29599	ES	4	3	6	-0.01	7.9E-01	excluded
STK35	58539	ES	3	2	4	0.00	7.9E-01	included
E2F6	52693	ES	3	1	5.1	0.00	7.9E-01	included
HARS2	73751	ES	2.1:2.2:3:4:5	1.3	6.1	0.00	7.9E-01	excluded
GMFG	49774	ES	06:07.1	4	7.3	0.00	7.9E-01	excluded
TKT	65306	ES	6.2	5	7.1	0.00	7.9E-01	included
LMBR1	82473	ES	8	7.2	9	0.00	7.9E-01	excluded
AGTPBP1	86736	ES	7	6	8	0.00	7.9E-01	excluded
RPS6KB1	42835	ES	9	7	10	0.00	7.9E-01	included
ODF2L	3673	ES	16	15	17	0.00	7.9E-01	included
KDELR2	78737	ES	5	4	6	0.00	7.9E-01	excluded
LRRC20	12048	ES	5	4	6	0.00	7.9E-01	excluded
APBB3	73681	ES	6.5:6.6	6.2	6.9	0.00	7.9E-01	excluded
HDAC8	89474	ES	3.1:3.2:4.1:5:6.1:8.1:8.2:11:12:13:14.1	2	14.2	0.00	7.9E-01	excluded
HNRNPA1	264681	ES	3:4:5:6.1:6.2:8:9.1:9.2:10:11.1:11.2	2	11.3	0.00	7.9E-01	excluded
C5orf45	74984	ES	3.2	2.1	4	0.00	7.9E-01	included
SLC47A1	39737	ES	16:17	15	18.1	0.00	7.9E-01	excluded
ARF4	65383	ES	2	1	3.2	0.00	7.9E-01	included
PAOX	13558	ES	4	3	5	0.00	7.9E-01	included
RHOC	4236	ES	1.2:1.3:2.2	1.1	2.3	0.00	7.9E-01	included
CAPN10	58282	ES	2:3.1:3.2:4:5:6:7:8:9:10.1:10.2	1	11	0.00	7.9E-01	included
ZSCAN32	33564	ES	2.2:3:4:5.2	1.2	6.1	0.00	7.9E-01	included
MTERF	80409	ES	2	1	4	0.00	7.9E-01	included
PARP16	31166	ES	2:03	1	4	0.00	7.9E-01	excluded
HNRNPH3	11929	ES	3:4.1:4.2	2	5	0.00	7.9E-01	included
SNX16	84311	ES	4	3	5	0.00	7.9E-01	included
MBNL2	26148	ES	5:6.1:6.2:6.3	4	8	0.00	7.9E-01	included
RTN3	16532	ES	3.2	1	4	0.00	7.9E-01	excluded
R3HCC1L	12757	ES	6	4	8	0.00	7.9E-01	included
FDFT1	82652	ES	5.2	2.2	6.3	0.00	7.9E-01	excluded
PIGG	68363	ES	9.1	8	10.1	0.00	7.9E-01	excluded
C19orf54	49978	ES	7.3:8.1:8.2:8.3:8.4:8.5:8.7:8.8:8.9	7.2	8.1	0.00	7.9E-01	excluded
PBX1	8791	ES	10:11	9	12	0.00	7.9E-01	included
C5orf45	74966	ES	2.3:3.1:3.2:4:5.1:5.2	2.1	6	0.01	7.9E-01	included
SETD5	63095	ES	5	4	6	0.01	7.9E-01	included
METTL21A	57193	ES	6	5	7	0.00	7.9E-01	included
GIN1	72909	ES	3	2	4	0.00	7.9E-01	excluded
DYX1C1	30736	ES	10	9	11	0.00	7.9E-01	included
PDE9A	60748	ES	2:3:5:7	1	8	0.00	7.9E-01	excluded
PRKAG2	82392	ES	15	14.1	16	0.00	7.9E-01	excluded
SEC11A	32318	ES	5:07	4	8	0.00	7.9E-01	excluded
FAM84A	52720	ES	2	1	3	0.00	7.9E-01	included
ACBD5	11070	ES	3	2.3	4	0.00	7.9E-01	excluded

FAXDC2	74231	ES	7	6	9.1	0.00	7.9E-01	excluded
SULT1A1	235340	ES	06:02.2	3	8	0.01	7.9E-01	included
CD46	9655	ES	9	8	10	0.00	7.9E-01	included
STRADA	42977	ES	3	2.2	6	0.00	7.9E-01	included
POLR3E	35554	ES	03:05.1	2	5.2	0.00	7.9E-01	excluded
DNAJA1	86092	ES	3	2	4	0.00	7.9E-01	excluded
TCEAL4	89748	ES	3.2:4.2:5.1	3.1	5.2	0.00	7.9E-01	excluded
FER	72933	ES	10:11	9	12	0.00	7.9E-01	excluded
GOLGA4	63985	ES	5:6:7:8:9:10:11:12:13:<br>14:15:16:17:18:20:21:	4	25	0.00	7.9E-01	excluded
WDR35	52756	ES	12	10	13	0.00	7.9E-01	excluded
LRSAM1	87636	ES	20	19	21	0.00	7.9E-01	excluded
HSF4	36948	ES	10.2	9	11.2	0.00	7.9E-01	included
USP35	18019	ES	4.2	3	5	0.00	7.9E-01	included
OS9	22710	ES	5.1:5.2:5.3:6:7.1:7.4:8	4	9.2	0.00	7.9E-01	excluded
CAAP1	86034	ES	2:3:4.2:5	1.1	6	0.00	7.9E-01	included
FBXO10	86410	ES	7	6	8	0.00	7.9E-01	excluded
PHTF1	4279	ES	7	6	8	0.00	7.9E-01	excluded
XPNPEP1	13074	ES	2	1	3	0.00	7.9E-01	included
NME6	64585	ES	5.2:6	4	7	0.00	7.9E-01	included
H2AFY	73455	ES	4:5:6.3:7	3	9	0.00	7.9E-01	included
DEF8	38192	ES	2.1:3	1	4	0.00	7.9E-01	included
CLCN2	67939	ES	13:14.1	12	14.2	0.00	7.9E-01	excluded
TMEM107	39128	ES	2:3.1:3.2:3.3:3.4:3.5	1	3.7	0.00	7.9E-01	included
MYO5C	30653	ES	10	9	11	0.00	7.9E-01	included
PRMT2	60958	ES	8:9:10:11	6.1	12	0.00	7.9E-01	excluded
CCNI	69627	ES	04:05.1	2	5.2	0.00	7.9E-01	excluded
PRMT7	37221	ES	13:14:15	12	16	0.00	7.9E-01	excluded
HAVCR2	74315	ES	3	2	4	0.00	7.9E-01	excluded
TMEM205	47661	ES	2.5:2.6	2.2	3	0.00	7.9E-01	excluded
CDK10	38120	ES	4	2.2	6	0.00	7.9E-01	excluded
DDX39A	47974	ES	8	7.2	9	0.00	7.9E-01	excluded
KLHL7	78956	ES	3:4:5:6.1	1	8	0.00	7.9E-01	included
ALDH16A1	50939	ES	2:03:04	1	5	0.00	7.9E-01	excluded
SYVN1	16797	ES	6:7.1:7.2	5	8	0.00	7.9E-01	excluded
TRPT1	16577	ES	7.1:7.2	6	8	0.00	7.9E-01	included
GOSR1	40117	ES	8	7.2	9	0.00	7.9E-01	excluded
EPB41	1401	ES	21	20	22	0.00	7.9E-01	included
NSUN5	97504	ES	03:02.2	1	5	0.01	7.9E-01	included
UBR1	30204	ES	23	22	24	0.00	7.9E-01	excluded
CBWD5	86502	ES	13	12.1	14	0.00	8.0E-01	included
RSU1	10879	ES	1.3	1.1	2	0.00	8.0E-01	excluded
GDPD1	42771	ES	7	6	8	0.00	8.0E-01	excluded
TKT	65299	ES	14	13	15	0.00	8.0E-01	excluded
FOSB	50454	ES	2.5	2.3	3.1	0.00	8.0E-01	excluded
NIN	27494	ES	29	28	30	0.00	8.0E-01	excluded
EPS15	2958	ES	16:17	15	18	0.00	8.0E-01	excluded
CTNND1	15977	ES	3	2.1	4.1	0.00	8.0E-01	excluded
PRMT5	26663	ES	2	1.2	3	0.00	8.0E-01	excluded
FAM184A	77362	ES	19	18	20	0.00	8.0E-01	included
EIF4G1	67893	ES	2.4:3.2	2.2	5	0.00	8.0E-01	excluded
DIABLO	24935	ES	3	2.4	4	0.00	8.0E-01	included
FPGS	87669	ES	15	14	16	0.00	8.0E-01	excluded
PHC1	20217	ES	2.2	1	3	0.00	8.0E-01	included
GALM	53269	ES	2:04:05	1	6	0.00	8.0E-01	excluded
SGSH	44036	ES	3:04	2.2	5	0.00	8.0E-01	included
GPAA1	85530	ES	2	1	3.1	0.00	8.0E-01	excluded
SLC30A2	1228	ES	3	2	4	0.00	8.0E-01	excluded
RBM25	28259	ES	2	1	3	0.00	8.0E-01	excluded
WDR27	78476	ES	4:05:06	3	7	0.00	8.0E-01	excluded
HSCB	61551	ES	2.1:3.1:4	1	5	0.00	8.0E-01	excluded
PIGF	53468	ES	3	2	4	0.00	8.0E-01	excluded

DPYSL3	73959	ES	2.2:3:4:5:6:7:8:9:10:1	2.1	12	0.00	8.0E-01	included
ELL2	72833	ES	4.2:5:6:7:8.1	4.1	8.2	0.00	8.0E-01	excluded
AGTRAP	667	ES	4.1:4.2:5	3	6.1	0.00	8.0E-01	included
SNX17	52998	ES	2.2:3:4	1	5	0.00	8.0E-01	included
MED16	46340	ES	4	3.2	5	0.00	8.0E-01	included
RPL17	45482	ES	4	3.4	5	0.00	8.0E-01	included
ASB8	21438	ES	5.1:5.2	4.1	5.4	0.00	8.0E-01	included
PXMP2	25285	ES	4	3.2	5	0.00	8.0E-01	included
BRCA1	41187	ES	24	22	25	0.00	8.0E-01	excluded
LDB2	68845	ES	9.2:10	9.1	11.2	0.00	8.0E-01	excluded
FCHSD2	17675	ES	6	5	7	0.00	8.0E-01	excluded
MTG2	60048	ES	4	3	5	0.00	8.0E-01	excluded
BFAR	34094	ES	3:04:05	2	6	0.00	8.0E-01	excluded
GGCT	79125	ES	6.1	5	7	0.00	8.0E-01	included
ZNF846	47411	ES	2.1:3.2:4.1:4.2	1	6.1	0.00	8.0E-01	included
TEAD1	14435	ES	11	10	12	0.00	8.0E-01	excluded
DPP8	31176	ES	17:19	16	20	0.00	8.0E-01	included
TOP3A	39613	ES	2:3.1:3.2	1	5.1	0.00	8.0E-01	excluded
CCT4	53705	ES	5	4	6	0.00	8.0E-01	included
STARD3NL	79286	ES	2	1	3	0.00	8.0E-01	included
HYPK	30321	ES	3	2.2	4.1	0.00	8.0E-01	included
BUB3	13393	ES	2	1	3	0.00	8.0E-01	excluded
AFMID	43805	ES	8:9:10:11.1	6	12	0.00	8.0E-01	included
KDM3B	73542	ES	8	7.3	9	0.00	8.0E-01	excluded
LSR	49084	ES	7	6	8.2	0.00	8.0E-01	excluded
NSRP1	40103	ES	2	1	3	0.00	8.0E-01	included
CCDC90B	18076	ES	1.3:2:3	1.2	4.2	0.00	8.0E-01	excluded
C7orf43	80908	ES	08:09.1	7	9.2	0.00	8.0E-01	excluded
TAF1A	9886	ES	3	2	4	0.00	8.0E-01	included
ITCH	59023	ES	4	3	5	0.00	8.0E-01	included
ME1	76870	ES	2:03	1	4	0.00	8.0E-01	excluded
LRRFIP2	63977	ES	6:7:8:9:10:14	5	18	0.00	8.0E-01	excluded
SLC29A1	76376	ES	2	1	5.3	0.00	8.0E-01	excluded
APCDD1	44629	ES	2	1	3.1	0.00	8.0E-01	excluded
FGD5	63534	ES	18	17	19	0.00	8.0E-01	excluded
LGALS3BP	43967	ES	2.4:2.5	2.2	3.1	0.00	8.0E-01	included
CCDC90B	18084	ES	1.5	1.2	2	0.00	8.0E-01	included
APLP2	19484	ES	4:5.1:5.2	1	6.1	0.00	8.0E-01	included
AIF1L	87921	ES	5	2.2	6.2	0.00	8.0E-01	included
DERA	20599	ES	4.2	3	5	0.00	8.0E-01	included
FTO	36429	ES	3	2	4	0.00	8.0E-01	excluded
SLC4A5	54041	ES	7	6	8	0.00	8.0E-01	included
POP4	48845	ES	3.2:4	3.1	5	0.00	8.0E-01	included
IRF3	50986	ES	6.2	5.1	7	0.00	8.0E-01	included
MRPL55	10123	ES	2.2:2.4:2.5	1.2	2.9	0.00	8.0E-01	excluded
TIMM10B	14147	ES	2	1	3.1	0.00	8.0E-01	included
ST3GAL3	2300	ES	7.2	6	9	0.00	8.0E-01	excluded
BECN1	41140	ES	5:06	4	7	0.00	8.0E-01	excluded
SHBG	39015	ES	7:08	6.2	9	0.00	8.0E-01	excluded
TEX261	53912	ES	3	2	4	0.00	8.0E-01	excluded
SLC38A6	27791	ES	18:19	17	20.2	0.00	8.0E-01	excluded
HSPH1	25578	ES	5	4.2	6	0.00	8.0E-01	included
SDAD1	69570	ES	4	3.3	5	0.00	8.0E-01	included
RIF1	55572	ES	31	30	32	0.00	8.0E-01	included
IRF3	50984	ES	6.1:6.2	5.1	7	0.00	8.0E-01	excluded
SMG1	93908	ES	4	3	5	0.00	8.0E-01	excluded
VPS35	36306	ES	3.2	2	4	0.00	8.0E-01	excluded
TDRD7	87009	ES	2	1	3	0.00	8.0E-01	excluded
UBE2D4	79372	ES	6	5.1	7.1	0.00	8.0E-01	excluded
PACRGL	68913	ES	6:08	5	9	-0.01	8.0E-01	excluded
GLS2	22452	ES	2	1	3	0.00	8.0E-01	included
DYNC1I2	55947	ES	7.3:8	4	9	0.00	8.0E-01	included

SDHC	8664	ES	2:03:04	1	5	0.01	8.0E-01	included
NARG2	30966	ES	2	1.2	3	0.00	8.0E-01	excluded
MIB2	189	ES	19.1:19.2:19.3	18	20	0.00	8.0E-01	excluded
PCYT1A	68234	ES	4	3	5	0.00	8.0E-01	included
HNRNPC	26544	ES	3.3:4.1	3.2	5	0.00	8.0E-01	excluded
GUK1	10184	ES	10:11.2:11.3	9.2	12	0.00	8.0E-01	included
DBNL	79390	ES	5.2	4.1	6	0.00	8.0E-01	excluded
VPS33B	32535	ES	2	1	3	0.00	8.0E-01	excluded
CAMK2D	70414	ES	16:17	14.2	18	0.00	8.0E-01	included
IFT122	66738	ES	5:07	3	8.2	0.00	8.0E-01	excluded
TP53INP2	59033	ES	2	1	3	0.00	8.0E-01	included
RGL3	47698	ES	6	5	7	0.00	8.1E-01	included
OS9	22718	ES	2.2	1	4	0.00	8.1E-01	included
SATB1	63673	ES	14	13	15	0.00	8.1E-01	excluded
MDK	15569	ES	3	2.4	4.1	0.00	8.1E-01	included
GSTM4	4054	ES	2.5:3	2.3	4	0.00	8.1E-01	included
RPAIN	38689	ES	4	3	5	0.00	8.1E-01	excluded
SLC37A4	19074	ES	11	10	12	0.00	8.1E-01	included
DMKN	49198	ES	7:8:9:11	6.4	12	0.01	8.1E-01	included
ZNF330	70668	ES	5	4	6	0.00	8.1E-01	included
SEC11A	32319	ES	5:06	4	8	0.00	8.1E-01	excluded
SS18	44958	ES	9	3	11	0.00	8.1E-01	included
MYO18A	40038	ES	41	40	42	0.00	8.1E-01	included
WASH4P	32781	ES	5.1	4	6.1	0.00	8.1E-01	included
CLK4	74870	ES	12	11	13	0.00	8.1E-01	included
TMEM106A	41699	ES	6	5	7	0.00	8.1E-01	included
ARL6IP4	25025	ES	3.4:4.1	3.3	4.2	0.00	8.1E-01	included
FAM45A	13255	ES	4	3	5	0.00	8.1E-01	included
BTNL9	75042	ES	2	1	3	0.00	8.1E-01	included
NCOA2	84118	ES	14	13	15.1	0.00	8.1E-01	included
VEGFA	76341	ES	6:8.1:8.2	5	9.1	0.00	8.1E-01	excluded
TLK2	42905	ES	5:06	4.2	7	0.00	8.1E-01	excluded
ZMAT1	89676	ES	2	1	3	0.00	8.1E-01	excluded
AASDHPPT	18561	ES	3	2	4	0.00	8.1E-01	excluded
IL1R1	54779	ES	8:9:10:11:12:13.1:13.2:13.3:13.4	7	13.5	0.00	8.1E-01	included
KIAA1328	45291	ES	5	4	6.1	0.00	8.1E-01	excluded
STAT6	22527	ES	2.2:4:5	2.1	6	0.00	8.1E-01	excluded
SLC25A29	29258	ES	3.2:3.5:3.6	2	3.7	0.00	8.1E-01	included
DEPDC5	61898	ES	44	43	45	0.00	8.1E-01	excluded
MED16	46334	ES	14	13	15	0.00	8.1E-01	excluded
PSMA6	27224	ES	9	8.2	10	0.00	8.1E-01	excluded
SLC35A2	89038	ES	3	1.2	4.1	0.00	8.1E-01	excluded
AP2B1	40323	ES	4.1:4.2	3	5	0.00	8.1E-01	included
COPE	48520	ES	4	3	6	0.00	8.1E-01	included
NFATC3	37188	ES	2	1	3	0.00	8.1E-01	included
GTF2IRD2B	80107	ES	8	7	9	0.00	8.1E-01	included
ACOT8	59628	ES	2:3.1:3.2:4	1	5	0.00	8.1E-01	included
LILRB4	51930	ES	13.1:13.2	12.2	14	0.01	8.1E-01	included
CDKL2	69547	ES	3:04:05	2	6	0.00	8.1E-01	included
OAZ2	31130	ES	2:03	1	4	0.00	8.1E-01	included
APOBR	35749	ES	3.2:4.1	3.1	4.2	0.00	8.1E-01	excluded
ZNF7	85670	ES	3	1	4.2	0.00	8.1E-01	included
ZNF277	81443	ES	3	2.2	4	0.00	8.1E-01	included
SLC16A4	4105	ES	6	5	7	0.00	8.1E-01	excluded
C17orf62	44344	ES	6	5.2	7.1	0.00	8.1E-01	excluded
TLK1	55926	ES	17	16	18	0.00	8.1E-01	included
BBS1	17062	ES	2	1.3	3	0.00	8.1E-01	included
ZNF559	47284	ES	3.1:3.2:3.3:4:5	2.2	6.1	0.00	8.1E-01	excluded
PPM1A	27778	ES	3	2	5.1	0.00	8.1E-01	included
AMBRA1	15576	ES	10	9	11	0.00	8.1E-01	excluded
MACF1	1882	ES	105	104	106	0.00	8.1E-01	excluded

AAAS	22019	ES	7	6	8	0.00	8.1E-01	excluded
TAMM41	63407	ES	9.2:10	9.1	11.1	0.00	8.1E-01	excluded
CD320	47213	ES	2.2	1	3.1	0.00	8.1E-01	excluded
DNAJC2	81188	ES	12:13	11	14	0.00	8.1E-01	excluded
SLAIN1	26094	ES	8	7	9	0.00	8.1E-01	excluded
ZSCAN32	33558	ES	2.2	1.2	3	0.00	8.1E-01	excluded
E2F5	84328	ES	4.2:5.1	4.1	5.2	0.00	8.1E-01	excluded
SLC12A4	37159	ES	20	19	21	0.00	8.1E-01	excluded
CDC42BPA	10042	ES	36	35	37	0.00	8.1E-01	included
PPP2R4	87857	ES	4:05	3.1	6	0.00	8.1E-01	excluded
GOLGA4	63988	ES	3	2	4	0.00	8.1E-01	excluded
SLC26A7	84437	ES	9	8	10	0.00	8.1E-01	excluded
NCEH1	67651	ES	2.1	1	3	0.00	8.1E-01	included
RNF216	78680	ES	3:4:5.1:5.2:6.2	2	7	0.00	8.1E-01	excluded
LLGL2	43464	ES	3	1	4	0.00	8.1E-01	excluded
C1orf159	24	ES	5.1	4.4	5.5	0.00	8.1E-01	included
ORC4	55531	ES	4:05:06	2.1	7	0.00	8.1E-01	excluded
FOXRED1	19375	ES	5	4.2	6	0.00	8.1E-01	included
RAB2A	83947	ES	2	1	3	0.00	8.1E-01	included
THOC5	61613	ES	11	10	12.1	0.00	8.1E-01	excluded
FASTK	82339	ES	3.1:4	2	5.1	0.00	8.1E-01	excluded
UQCC2	75764	ES	2	1	3	0.00	8.1E-01	excluded
TSPAN31	22732	ES	2.1:2.2:3	1	5.1	0.00	8.1E-01	included
PPP2R4	87859	ES	4	3.1	6	0.00	8.1E-01	excluded
HNRNPA1	212638	ES	3:4:5:6.1:6.2:7.1:7.2:8 :9.1:9.2:10:11.1:11.2	2	11.3	0.00	8.1E-01	excluded
ZC3HC1	81760	ES	13	12.2	14	0.00	8.1E-01	included
PRMT1	51042	ES	4.2:5	4.1	6	0.00	8.1E-01	included
YBEY	60916	ES	2.2:3	2.1	4	0.00	8.1E-01	included
ARHGEF1	50102	ES	6	5	7	0.00	8.1E-01	excluded
FHL2	54834	ES	2.3:3.2	2.2	5.1	0.00	8.1E-01	included
PMM2	33951	ES	04:05.2	3	6	0.00	8.1E-01	included
FKBP9	79206	ES	2	1	3	0.00	8.1E-01	included
RABGEF1	79897	ES	8	7	9	0.00	8.1E-01	excluded
STRADA	42972	ES	5	3	6	0.00	8.1E-01	included
CEP68	53780	ES	5	4.1	6	0.00	8.2E-01	excluded
SELENBP1	7618	ES	6:07	5	8	0.00	8.2E-01	excluded
GMPR2	26923	ES	5	3.2	6.1	0.00	8.2E-01	included
NAA60	33531	ES	09:10.2	8	10.3	0.00	8.2E-01	included
TG	319468	ES	25:26:27:31:32:33:34: 35:37:38:39:40:41:42:	24	46	0.00	8.2E-01	included
ECSIT	47721	ES	4:05	3	6.1	0.00	8.2E-01	included
KIAA1033	24124	ES	2	1	3	0.00	8.2E-01	excluded
UBE2G2	60832	ES	5	4	6	0.00	8.2E-01	excluded
ANAPC15	17576	ES	3.2	1.2	4.1	0.00	8.2E-01	excluded
PSMG4	75182	ES	5.3:5.4	5.1	5.6	0.00	8.2E-01	included
CLASRP	50393	ES	3.2:4:5.1	3.1	5.2	0.00	8.2E-01	included
TBC1D22A	62726	ES	9	8	10	0.00	8.2E-01	included
LIG1	50695	ES	06:07.2	5	8	0.00	8.2E-01	excluded
NARG2	30964	ES	4.2:5	3	6	0.00	8.2E-01	excluded
SEC11A	32316	ES	6	5	8	0.00	8.2E-01	excluded
HERC4	11918	ES	5	3	7	0.00	8.2E-01	included
RNF216	78676	ES	3:4:5.1:5.2:6.1:6.2	2	7	0.00	8.2E-01	included
SMPD4	55296	ES	11:14	10	15.1	0.00	8.2E-01	included
PDCD5	48891	ES	4.1:4.2:5.1:5.3	3	5.5	0.00	8.2E-01	included
PFKFB3	10699	ES	16	15	17	0.00	8.2E-01	included
PHTF2	80209	ES	17	16	18	0.00	8.2E-01	excluded
DNAJB14	70087	ES	2	1	3	0.00	8.2E-01	included
SLC9A8	59750	ES	5	4	6	0.00	8.2E-01	excluded
TIMM9	27710	ES	2:03	1	4.2	0.00	8.2E-01	included
ICA1	78789	ES	15	14	16	0.00	8.2E-01	included
CALML4	31353	ES	2:03:04	1	5	0.00	8.2E-01	excluded

DCTN2	22639	ES	19	18	20	0.00	8.2E-01	included
RAP1B	22937	ES	7.1	6	8.1	0.00	8.2E-01	excluded
HSPA12B	58597	ES	3	2	4	0.00	8.2E-01	included
ADAL	30237	ES	4	3	5	0.00	8.2E-01	included
AGTRAP	668	ES	4.2:5	3	6.1	0.00	8.2E-01	included
TMEM2	86572	ES	6	5	7	0.00	8.2E-01	excluded
IQCA1	58093	ES	8	7	9	0.00	8.2E-01	included
SORBS2	71384	ES	12	11.2	13	0.00	8.2E-01	included
CCND3	76161	ES	4	2	5	0.00	8.2E-01	excluded
MYL5	68388	ES	1.2:1.3:1.4:2	1.1	4.1	0.00	8.2E-01	excluded
GTF2IRD2	80095	ES	8	7	9	0.00	8.2E-01	included
IL10RB	60397	ES	3	2	4	0.00	8.2E-01	excluded
SNX1	139186	ES	4.1:4.2:5:6:7:8:9:10.2:11:12:14:15	3	16.1	0.00	8.2E-01	excluded
NID1	10365	ES	10:11:12	9	13	0.00	8.2E-01	included
ZNF671	52333	ES	2	1.1	3.1	0.00	8.2E-01	excluded
SMARCD2	43017	ES	3	1.1	4	0.00	8.2E-01	excluded
ANKFY1	38529	ES	4	3	5	0.00	8.2E-01	excluded
APOL1	62022	ES	6.1	5	7	0.00	8.2E-01	excluded
RAB28	68793	ES	7	6	9	0.00	8.2E-01	included
SLC35A1	76960	ES	6	5	7	0.00	8.2E-01	excluded
ZNF585B	49465	ES	5	2	6.2	0.00	8.2E-01	included
MAP4K4	54761	ES	18	16.2	19	0.00	8.2E-01	excluded
PPIA	79569	ES	2.1	1	3	0.00	8.2E-01	included
CLCC1	3975	ES	6.1:6.2:7.1:7.2	5	8	0.00	8.2E-01	included
ST3GAL5	262521	ES	10	9.2	12	0.00	8.2E-01	excluded
FAS	12477	ES	8	7	9.2	0.00	8.2E-01	included
STRBP	87505	ES	4	3	5	0.00	8.2E-01	excluded
MDM4	9479	ES	11	10	12	0.00	8.2E-01	excluded
DTNA	45101	ES	26.1	25.2	27.2	0.00	8.2E-01	included
ECHDC1	77464	ES	6.4:10.1	6.3	10.2	0.00	8.2E-01	excluded
SLC24A1	31235	ES	6	5	7	0.00	8.2E-01	included
DLG4	38848	ES	21	20	22	0.00	8.2E-01	included
NEDD4L	45654	ES	25	24	26	0.00	8.2E-01	excluded
NPRL3	32812	ES	4	2	5	0.00	8.2E-01	excluded
NMT1	41936	ES	5	4	6	0.00	8.2E-01	included
DNAJC4	16599	ES	05:06.2	4	7.1	0.00	8.2E-01	excluded
SS18	44968	ES	5	3	9	0.00	8.2E-01	excluded
MANBAL	59340	ES	2.2	1	3	0.00	8.2E-01	included
HS1BP3	52782	ES	2	1	3.1	0.00	8.2E-01	included
TNPO3	81738	ES	13	12.2	14	0.00	8.2E-01	included
SFXN2	12973	ES	6	5	7	0.00	8.3E-01	included
MEF2D	8274	ES	11	9	12	0.00	8.3E-01	excluded
BCAS3	42874	ES	20	19	21	0.00	8.3E-01	included
DENND1A	87518	ES	13	12	14	0.00	8.3E-01	excluded
C7orf63	80377	ES	20	19	21	0.00	8.3E-01	excluded
INCA1	38645	ES	2.2:3	2.1	4	0.00	8.3E-01	excluded
STRN	53211	ES	8	7	9	0.00	8.3E-01	included
ST7	81567	ES	5:6:7:8:9:11.1:12	1	13	0.00	8.3E-01	included
KIAA2026	85813	ES	5	4	6	0.00	8.3E-01	included
SLC16A4	4106	ES	4	3	5	0.00	8.3E-01	excluded
NOX4	18235	ES	16:17:18:19:20:21	15	22.1	0.00	8.3E-01	included
LARP7	70391	ES	3:04	1	5.1	0.00	8.3E-01	included
LGALS8	10391	ES	2:3.1:3.2:3.3	1	5	0.00	8.3E-01	included
ACAD9	66681	ES	2.1:2.2:2.3	1.1	3.2	0.00	8.3E-01	included
NBPF12	7354	ES	29:30:31:32:33:34:35:36:37:38:39:40:41:42:43:44:45:46:47:48:49:	28	57	0.00	8.3E-01	included
GNPDA2	69151	ES	3	2	4	0.00	8.3E-01	excluded
PIGB	30718	ES	10	9	11	0.00	8.3E-01	included
POLR3H	62432	ES	04:05.1	3	6	0.00	8.3E-01	included
TRMT1	47919	ES	3:04	2.4	5	0.00	8.3E-01	included

ITGB7	21973	ES	12:13	11	14	0.00	8.3E-01	included
PBXIP1	7851	ES	2.1:2.2:3.1	1	3.2	0.00	8.3E-01	excluded
TSEN15	9208	ES	5.1	3	6	0.00	8.3E-01	excluded
SNX14	76924	ES	16	15	17	0.00	8.3E-01	included
TMEM175	68435	ES	2:03	1	5.1	0.00	8.3E-01	excluded
POLR1B	55009	ES	3.1:3.2:4:5:6:7:8:9:10	1.3	11.1	0.00	8.3E-01	included
COX5A	31811	ES	04:05.1	3	6	0.00	8.3E-01	excluded
NSUN5	270161	ES	4	3.2	5	0.00	8.3E-01	excluded
ZNF331	51729	ES	10	9	11	0.00	8.3E-01	excluded
MED9	39483	ES	2.1	1	3	0.00	8.3E-01	excluded
NUP62CL	89819	ES	5	4	6	0.00	8.3E-01	excluded
ATG7	63389	ES	4	3	5	0.00	8.3E-01	excluded
VPS53	38221	ES	10	9	11	0.00	8.3E-01	included
LMBR1L	21535	ES	2	1	3	0.00	8.3E-01	excluded
WDR11	13290	ES	2.2:3:4:5:6:7:8:9:10.1	2.1	10.2	0.00	8.3E-01	included
INPP1	56563	ES	3	1	4	0.00	8.3E-01	excluded
COPZ1	22172	ES	3.1:3.2	1	4	0.00	8.3E-01	excluded
SNX1	31092	ES	13.1	12	14	0.00	8.3E-01	excluded
PAFAH1B1	38409	ES	4	3	5	0.00	8.3E-01	included
ATP6V1H	83835	ES	2.2:4.1:4.2	2.1	4.3	0.00	8.3E-01	excluded
RINT1	81296	ES	4.2:5.1	4.1	5.2	0.00	8.3E-01	included
JMJD8	32953	ES	2	1	3	0.00	8.3E-01	included
DOCK8	85708	ES	2	1	3	0.00	8.3E-01	included
IRF7	13709	ES	5.1	4	5.3	0.00	8.3E-01	included
RABL5	81060	ES	4	3.2	5	0.00	8.3E-01	included
GUSB	79863	ES	3.1:3.2	2	4.1	0.00	8.3E-01	included
ZNF791	47812	ES	3	1	4	0.00	8.3E-01	included
RAD51AP1	19782	ES	9.2:10.1	9.1	11	0.00	8.3E-01	excluded
ZNF256	52378	ES	2	1	3	0.00	8.3E-01	included
CDKAL1	75498	ES	4	3.2	5	0.00	8.3E-01	included
MAPK7	39722	ES	4	3	5	0.00	8.3E-01	excluded
NPHP4	355	ES	20	19	21	0.00	8.3E-01	excluded
C19orf54	49974	ES	7.3:8.1:8.2:8.3:8.4:8.5:8.6:8.7:8.8:8.9	7.2	8.1	0.00	8.3E-01	excluded
PLRG1	70899	ES	2	1	4	0.00	8.3E-01	included
BAK1	75761	ES	2	1	3	0.00	8.3E-01	included
SORD	30381	ES	5.2	4	6	0.00	8.3E-01	included
OCIAD1	69232	ES	10	9.1	11	0.00	8.3E-01	excluded
PDK1	55984	ES	7	4	8	0.00	8.3E-01	excluded
HNRNPA1	212639	ES	3:4:5:6.1:6.2:6.3:7.2:8:9.1:9.2:10:11.1:11.2	2	11.3	0.00	8.3E-01	included
RPL17-C18orf32	45476	ES	6	5	7	0.00	8.3E-01	excluded
PDLIM2	83027	ES	3	2.2	4	0.00	8.3E-01	included
POLR2F	62184	ES	3	2.2	4.1	0.00	8.3E-01	excluded
SDHAF2	16232	ES	3:4.1:5.2	1	6	0.00	8.3E-01	included
WDR45	89085	ES	6	5	7.2	0.00	8.3E-01	excluded
CCNY	11298	ES	5	4	6	0.00	8.3E-01	included
USP3	31054	ES	2:3.2:5:7:8:9:10	1	11	0.00	8.3E-01	excluded
PTPLAD1	31185	ES	9	8	10	0.00	8.3E-01	included
CD82	15478	ES	6	5	7	0.00	8.3E-01	included
AFMID	94696	ES	12	6	13	0.00	8.3E-01	excluded
PARM1	69515	ES	2	1	3	0.00	8.3E-01	included
C18orf8	44828	ES	4:06	3	7	0.00	8.3E-01	included
R3HDM2	22578	ES	12	11	13	0.00	8.3E-01	included
FLI1	19421	ES	4	3	5	0.00	8.3E-01	included
C12orf45	24116	ES	3	2	4	0.00	8.3E-01	included
ST7L	4212	ES	14	13	15	0.00	8.3E-01	included
OS9	22706	ES	5.1:5.2:5.3:6:7.1:7.3:7.	4	9.2	0.00	8.3E-01	included
STK25	58389	ES	3.2	2.1	4	0.00	8.3E-01	included
SYPL2	4012	ES	5	4	6	0.00	8.3E-01	excluded
RAB11A	31242	ES	2.2:3.1	2.1	3.2	0.00	8.3E-01	excluded
MVK	24339	ES	5:06	3	7	0.00	8.3E-01	excluded

ABLIM2	68747	ES	21:22	18.2	23	0.00	8.3E-01	excluded
CCNC	77084	ES	2	1.2	3	0.00	8.3E-01	included
SKIV2L2	72032	ES	2:03	1	4	0.00	8.3E-01	included
IARS	86835	ES	31	30	32	0.00	8.3E-01	excluded
METTL23	43657	ES	1.3	1.1	2	0.00	8.3E-01	excluded
DGKZ	15564	ES	7:8.1:8.2:9.2	2	10.2	0.00	8.3E-01	included
SP3	56015	ES	3	2	4	0.00	8.3E-01	included
FXR1	67754	ES	6:7:8:9:10:11:12:13:1	1	16	0.00	8.3E-01	excluded
MYO19	40489	ES	11.2:12	11.1	13	0.01	8.3E-01	included
EVL	29246	ES	7	5	8	0.00	8.3E-01	included
PITRM1	10629	ES	2	1	3	0.00	8.3E-01	included
ARCN1	19028	ES	3.2:4:5:6:7:8:9:10:11.	3.1	11.2	0.00	8.3E-01	included
ASPSCR1	44256	ES	16	15.2	17	0.00	8.3E-01	included
STOML2	86238	ES	6	5	7	0.00	8.3E-01	included
PPP2R3C	27203	ES	6.1:7.1	5.2	8.1	0.00	8.3E-01	excluded
DTWD2	73063	ES	5	4	6	0.00	8.3E-01	included
TMF1	65563	ES	10:11.1	9	11.2	0.00	8.3E-01	included
HMGN1	60618	ES	6.1:6.2:7:8.2	5	9	0.00	8.3E-01	included
MCCC1	67790	ES	4:07	2	8	0.00	8.4E-01	excluded
CACNB3	21479	ES	6	5	7	0.00	8.4E-01	excluded
DTNA	45110	ES	19:20	17	22	0.00	8.4E-01	included
DHRS4	26793	ES	3	2	7.1	0.00	8.4E-01	included
PQLC3	52675	ES	6	5	7	0.00	8.4E-01	excluded
DTNA	45114	ES	14:15	13	17	0.00	8.4E-01	excluded
PDHA1	88635	ES	2	1	3	0.00	8.4E-01	included
INADL	3244	ES	37	35	38.1	0.00	8.4E-01	included
OBSCN	10203	ES	20:21	19	22	0.00	8.4E-01	included
BDH1	68299	ES	4	3	5.1	0.00	8.4E-01	excluded
DBNL	79419	ES	2.1:2.2:3:4.1	1.1	5.2	0.00	8.4E-01	excluded
SNX1	139179	ES	4.1:4.2:5:6:7:8:9:10.1:<br>10.2:11:12:14:15	3	16.1	0.00	8.4E-01	excluded
PARP6	31529	ES	10.2:11.1	10.1	11.2	0.00	8.4E-01	excluded
DMKN	49179	ES	8:09	7	12	0.00	8.4E-01	included
CPSF3L	140	ES	2.1	1	4	0.00	8.4E-01	excluded
TMEM194B	56574	ES	7.1:7.2	6	8	0.00	8.4E-01	excluded
NEO1	31598	ES	22	21	23	0.00	8.4E-01	included
NMNAT3	67040	ES	5:07	3	8	0.00	8.4E-01	excluded
PDDC1	13747	ES	7:8.1:8.2:8.3:8.4	6.2	8.5	0.00	8.4E-01	excluded
SNX11	42184	ES	2	1.2	3.1	0.00	8.4E-01	included
CA12	31027	ES	3	2	4	0.00	8.4E-01	excluded
UQCC1	59161	ES	2	1	3	-0.01	8.4E-01	excluded
SNX1	31099	ES	4.1:4.2:5:6	3	7	0.00	8.4E-01	excluded
RASSF3	22860	ES	3	2	4	0.00	8.4E-01	included
FUT8	28015	ES	4	3	5	0.00	8.4E-01	included
CLN3	35724	ES	10	9	11	0.00	8.4E-01	included
POGZ	7631	ES	5:6:7.1:7.2:8:9	4	10	0.00	8.4E-01	included
FASTK	82338	ES	3.1:3.2:4	2	5.1	0.00	8.4E-01	excluded
ALDH7A1	73170	ES	13:14	12	15	0.00	8.4E-01	excluded
PIK3CB	67021	ES	10	9	11	0.00	8.4E-01	included
NBR1	41695	ES	21:22	20	24	0.00	8.4E-01	excluded
TRPC4AP	59058	ES	9.1:9.2	8	10	0.00	8.4E-01	excluded
WWOX	37684	ES	2	1.1	3	0.00	8.4E-01	excluded
ST6GALNAC2	43601	ES	2	1	3	0.00	8.4E-01	excluded
ZFAT	85264	ES	17:18.1:18.2	16	19.1	0.00	8.4E-01	excluded
TOX4	26591	ES	3.1	2	5.1	0.00	8.4E-01	excluded
PQLC1	94856	ES	6	5	10	0.00	8.4E-01	excluded
KDM1A	1032	ES	3	2	4	0.00	8.4E-01	excluded
PFKFB4	64707	ES	12	11.2	14	0.00	8.4E-01	excluded
DUOXA1	30396	ES	2.2:3:4	1.1	5	0.00	8.4E-01	excluded
VKORC1	36232	ES	4.1:4.2	2	6	0.00	8.4E-01	excluded
APOC1	50371	ES	2.2	1	3.2	0.00	8.4E-01	included
AGK	82041	ES	14	13	15	0.00	8.4E-01	excluded

SUMF2	79808	ES	4	3	7	0.00	8.4E-01	included
CRYZL1	60456	ES	12.2:13:14:16.1:16.2	11	17	0.00	8.4E-01	included
CNNM3	54581	ES	2	1	3	0.00	8.4E-01	excluded
SAMM50	62604	ES	05:06.1	4	6.2	0.00	8.4E-01	included
ATP5A1	300055	ES	4.1:4.2:5	3.2	6.1	0.00	8.4E-01	included
TBL1X	88450	ES	6	5	7	0.00	8.4E-01	included
CAPN7	63559	ES	3	1	4	0.00	8.4E-01	included
ZNF19	37454	ES	5.1:5.2	4	7	0.00	8.4E-01	included
DCTD	71234	ES	6	5	7	0.00	8.4E-01	included
SDCBP	83931	ES	6.1:6.2:7.1	5	7.2	0.00	8.4E-01	excluded
ARMCX2	89669	ES	02:03.1	1	3.2	0.00	8.4E-01	included
HDHD2	45439	ES	6	5	7	0.00	8.4E-01	excluded
PMS1	56550	ES	5.1	4	6	0.00	8.4E-01	excluded
ATP6V1D	28051	ES	4	3	5	0.00	8.4E-01	included
PMPCA	88167	ES	5	4	6.1	0.00	8.4E-01	excluded
CDC27	42053	ES	9.2:10:11:12.2:13:14	8	15	0.00	8.4E-01	excluded
NCOR1	39420	ES	27:28:29:30	26	31	0.00	8.4E-01	included
RBM6	64953	ES	4:05	2	7	0.00	8.4E-01	included
ALDH2	24537	ES	4	3	5	0.00	8.4E-01	excluded
CDPF1	62695	ES	3.3:4	3.2	5	0.00	8.4E-01	excluded
DNAJC4	16598	ES	5:6.1:6.2	4	7.1	0.00	8.4E-01	included
LRP8	3060	ES	10	9	11	0.00	8.4E-01	included
RNPS1	33261	ES	3	1.1	4	0.00	8.4E-01	excluded
PNISR	77056	ES	2	1	3	0.00	8.4E-01	included
ZNF195	13985	ES	2:03	1	4	0.00	8.4E-01	excluded
UGP2	53756	ES	7:08	6	9	0.00	8.4E-01	included
NT5DC2	65226	ES	6	5	7	0.00	8.5E-01	excluded
BANK1	70102	ES	8	6	9	0.00	8.5E-01	included
UXS1	54859	ES	2:3.2:4:5:6:8	1	9	0.00	8.5E-01	included
ACSL1	71324	ES	10	9	11	0.00	8.5E-01	excluded
TMEM41A	68012	ES	3:04	2	5	0.00	8.5E-01	included
ATG13	15593	ES	2.2:3	1.1	4.1	0.00	8.5E-01	included
AP2S1	50601	ES	4.2	3.2	5	0.00	8.5E-01	excluded
GOLGA4	63986	ES	5:6:7:8:9:10:11:12:13:<br>14:15:16:17:18:19:20:	4	25	0.00	8.5E-01	excluded
EZH1	41110	ES	6	5	7	0.00	8.5E-01	included
C16orf58	36281	ES	2	1.3	3	0.00	8.5E-01	excluded
DAGLB	78728	ES	7	6	8	0.00	8.5E-01	included
CARF	56975	ES	5.1:5.2:6.1:6.2	4.2	7	0.00	8.5E-01	included
LYPLA1	83863	ES	3:4:5:6:7	2	8	0.00	8.5E-01	excluded
PLA2G7	76423	ES	3:04:05	2	6	0.00	8.5E-01	included
RASA3	26404	ES	5	4	6	0.00	8.5E-01	excluded
GTF2A2	30935	ES	5	4	6	0.00	8.5E-01	included
CARD8	50711	ES	9:10.1:10.2	8	11	0.00	8.5E-01	included
MLX	41072	ES	3	2	4	0.00	8.5E-01	excluded
LAMA5	60064	ES	66	65	67	0.00	8.5E-01	excluded
GAL3ST4	80915	ES	3	2.2	4	0.00	8.5E-01	excluded
PSMB5	26690	ES	2:03	1.2	4	0.00	8.5E-01	excluded
SFI1	61871	ES	7:08	6	9	0.00	8.5E-01	excluded
MFNG	62114	ES	2	1.1	3	0.00	8.5E-01	excluded
HNRNPC	26543	ES	3.3:4.1:4.2	3.2	5	0.00	8.5E-01	included
RPS3	17843	ES	05:06.1	3.2	6.2	0.00	8.5E-01	included
SPATA13	25476	ES	12	11	13	0.00	8.5E-01	included
MATN2	84635	ES	8	7	9	0.00	8.5E-01	excluded
SMTN	61815	ES	15:16	14	17	0.00	8.5E-01	included
CEP120	73135	ES	23	22	24	0.00	8.5E-01	included
HSF4	36946	ES	10.2:11.1	9	11.2	0.00	8.5E-01	included
OPTN	10782	ES	04:05.1	2	5.2	0.00	8.5E-01	excluded
EIF4E	70012	ES	06:07.1	4	7.2	0.00	8.5E-01	excluded
SUGP1	48624	ES	3	2.1	4	0.00	8.5E-01	included
RNF14	73843	ES	6	5	7	0.00	8.5E-01	included
NUMB	28288	ES	13	12	14	0.00	8.5E-01	excluded

DNAAF2	27430	ES	2	1	3	0.00	8.5E-01	included
FAM13A	69913	ES	14	13	15	0.00	8.5E-01	excluded
NF2	61661	ES	2:03	1	4	0.00	8.5E-01	excluded
PPT1	1995	ES	2:03:04	1	5.2	0.00	8.5E-01	included
ABHD10	66068	ES	5	4	6	0.00	8.5E-01	included
EVA1C	60347	ES	2:03	1	4	0.00	8.5E-01	excluded
PLSCR1	67170	ES	2:03	1	4	0.00	8.5E-01	excluded
ZNF283	50251	ES	5	4	6	0.00	8.5E-01	included
TBRG1	19228	ES	3	2	6	0.00	8.5E-01	included
FXR1	67748	ES	5:06	1	7	0.00	8.5E-01	included
PODXL	81825	ES	3	2.2	4.1	0.00	8.5E-01	excluded
PFKFB3	10698	ES	18.1	17	20	0.00	8.5E-01	excluded
LAMTOR5	4116	ES	1.3	1.1	2.2	0.00	8.5E-01	excluded
USP45	77066	ES	18	16	19	0.00	8.5E-01	excluded
ZNF717	65639	ES	3	1	4.2	0.00	8.5E-01	excluded
ZNF691	2133	ES	2.1:2.2:2.3:3	1	4	0.00	8.5E-01	excluded
GTDC1	55501	ES	14	13	15	0.00	8.5E-01	excluded
RAB3IP	23348	ES	11	10	12	0.00	8.5E-01	included
ARVCF	61109	ES	11	10	12	0.00	8.5E-01	included
ENY2	84891	ES	1.2:1.3:2.1	1.1	2.2	0.00	8.5E-01	excluded
DRAM1	24006	ES	4:05:06	3	7	0.00	8.5E-01	included
PPIG	55874	ES	6	5.2	7	0.00	8.5E-01	excluded
NBPF11	7328	ES	21:22	20.2	23	0.00	8.5E-01	excluded
POLDIP3	62526	ES	3.1:3.2:4:6:7.1:7.2:8:9	2	10.2	0.00	8.5E-01	included
HSD17B4	73087	ES	1.2:2	1.1	4	0.00	8.5E-01	included
TINF2	26935	ES	2	1	3	0.00	8.5E-01	included
PTP4A1	76599	ES	5	4	6	0.00	8.5E-01	excluded
C11orf70	18439	ES	4	3	5.1	0.00	8.5E-01	included
ZNF562	47391	ES	3:04	2.2	5	0.00	8.5E-01	excluded
WFDC2	59583	ES	2:03	1	4.3	0.00	8.5E-01	included
TMEM201	566	ES	8	7	9	0.00	8.5E-01	included
RPP38	10863	ES	1.2:2	1.1	3.1	0.00	8.5E-01	excluded
VPS45	7423	ES	5:06	4	7	0.00	8.5E-01	included
INPP5K	38329	ES	04:05.2	1	7	0.00	8.5E-01	excluded
PRR5	62619	ES	2	1	3.2	0.00	8.5E-01	included
TYROBP	49316	ES	2	1.2	3	0.00	8.5E-01	included
CA2	84356	ES	4	2	5	0.00	8.5E-01	included
MDM2	22972	ES	9	8.2	10	0.00	8.5E-01	included
PFDN5	93151	ES	04:02.2	1	6.2	0.00	8.5E-01	included
VPS54	53763	ES	10	9	11	0.00	8.5E-01	included
MCAT	62569	ES	3	2	4	0.00	8.5E-01	included
TBCK	70269	ES	6:07	5	9.1	0.00	8.5E-01	excluded
TNFRSF1A	19830	ES	3.2:4.1	3.1	4.2	0.00	8.5E-01	excluded
DEAF1	13723	ES	11	10	12	0.00	8.5E-01	excluded
MARK3	29452	ES	9	8	10	0.00	8.5E-01	excluded
RNF40	36188	ES	9:10.1:10.2	8	11	0.00	8.5E-01	included
THOC3	74620	ES	3	2	4	0.00	8.5E-01	excluded
SAFB	46854	ES	4	3	5	0.00	8.5E-01	included
MTMR1	90354	ES	17	16	18	0.00	8.5E-01	excluded
ZNF268	25382	ES	9	7	10.1	0.00	8.5E-01	included
PAAF1	17744	ES	4.2	3.2	5	0.00	8.5E-01	excluded
RNF123	64911	ES	03:04.2	1	5	0.00	8.6E-01	excluded
BCKDHB	76829	ES	6	5	7	0.00	8.6E-01	included
GBP3	3708	ES	8:09	7	10	-0.01	8.6E-01	excluded
HMGN1	60624	ES	6.2	5	9	0.00	8.6E-01	excluded
GRB10	79722	ES	6	5.2	11	0.00	8.6E-01	excluded
OGG1	63173	ES	2:03:04	1	5	0.00	8.6E-01	included
ZUFSP	77334	ES	5	4	6	0.00	8.6E-01	excluded
CCDC14	66493	ES	2.8:2.9	2.6	3	0.01	8.6E-01	included
HFE	75585	ES	2.1:2.2:3:5.1:5.2	1	6	0.00	8.6E-01	excluded
PSMB7	87533	ES	5	4	6	0.00	8.6E-01	excluded
TOLLIP	13829	ES	4:05:06	3.2	8	0.00	8.6E-01	included

GLRB	70972	ES	6:7:8:9:10:11	3	13	0.00	8.6E-01	included
NFYA	76094	ES	3	2	4	0.00	8.6E-01	excluded
STAG3	101025	ES	10	4	11	0.00	8.6E-01	included
TOP1MT	85416	ES	12.2:13.1	12.1	13.2	0.00	8.6E-01	included
EDNRA	70785	ES	4	3	5	0.00	8.6E-01	excluded
TCF12	30793	ES	4	3	6	0.00	8.6E-01	included
AP3M2	83559	ES	7	6.1	8	0.00	8.6E-01	excluded
PDCD6IP	63893	ES	3	1	4	0.00	8.6E-01	excluded
HINT1	73218	ES	3.2	2.1	4	0.00	8.6E-01	excluded
ADCK4	49951	ES	8	7	9	0.00	8.6E-01	included
SIL1	73577	ES	2	1	4	0.00	8.6E-01	included
HOMER1	72632	ES	4:05:06	3	7	0.00	8.6E-01	included
IFT20	39884	ES	2.1:2.2	1	3	0.00	8.6E-01	excluded
CDC14B	86978	ES	15	13	16	0.00	8.6E-01	included
RAB17	58126	ES	2.1:2.2:2.3	1	3	0.00	8.6E-01	included
POLL	12894	ES	04:05.1	1.5	6.1	0.00	8.6E-01	included
ZNF195	13977	ES	11:12	5.1	13	0.00	8.6E-01	excluded
ZDHHC17	23509	ES	2	1	3	0.00	8.6E-01	excluded
ELK1	88936	ES	4.2:5:6.1	4.1	6.2	0.00	8.6E-01	excluded
CCT3	8236	ES	3:04	1	5	0.00	8.6E-01	included
SLBP	68501	ES	3	2	4.2	0.00	8.6E-01	included
TACC2	13344	ES	12	11	13	0.00	8.6E-01	included
FAS	12482	ES	4	3	5	0.00	8.6E-01	excluded
WASF2	1330	ES	8	7	9	0.00	8.6E-01	excluded
NOL10	52663	ES	4	3	5	0.00	8.6E-01	included
MAMLD1	90334	ES	5:06	4	7	0.00	8.6E-01	included
LEO1	30635	ES	6:07	5	8	0.00	8.6E-01	included
LYRM4	75244	ES	2	1	7	0.00	8.6E-01	included
SLC7A6	37211	ES	7	6	8	0.00	8.6E-01	excluded
TMEM116	24560	ES	7	4	8	0.00	8.6E-01	included
GNAS	135422	ES	6	5	7	0.00	8.6E-01	excluded
COQ10B	56685	ES	3	1	4	0.00	8.6E-01	included
ATXN3	28945	ES	4.2:5:6	3.2	8.1	0.00	8.6E-01	included
IL32	33415	ES	1.9	1.1	2.1	0.00	8.6E-01	excluded
TMEM107	39116	ES	3.2:3.3:3.4:3.5	2	3.7	0.00	8.6E-01	included
TBC1D14	68731	ES	13	12	14	0.00	8.6E-01	excluded
ERCC1	50445	ES	08:10.1	7	11	0.00	8.6E-01	included
AXIN2	43093	ES	7	6	8	0.00	8.6E-01	included
INPP5B	1851	ES	13	12	14	0.00	8.6E-01	excluded
SMN1	72423	ES	9.1	7	9.3	0.00	8.6E-01	included
ADAM15	7908	ES	20:21.1	19	21.2	0.00	8.6E-01	included
ZNF691	2134	ES	2.1:2.3:3	1	4	0.00	8.6E-01	included
FAM96A	31085	ES	2	1	3	0.00	8.6E-01	excluded
ADA	59484	ES	4:05:06	3	7	0.00	8.6E-01	excluded
PCMTD1	83810	ES	3.1:3.2:5	1	6	0.00	8.6E-01	included
FAM3A	90629	ES	7.2	6	8	0.00	8.6E-01	excluded
ARFGAP1	60112	ES	10	9	11	0.00	8.6E-01	included
MPRIP	39459	ES	10	9	11	0.00	8.6E-01	included
RBM5	64961	ES	4	3	5	0.00	8.6E-01	excluded
BSDC1	1606	ES	3:4.2:4.3	2	5	0.00	8.6E-01	excluded
ACY1	390893	ES	8.1:11:14	7.2	15	0.00	8.6E-01	excluded
SLC25A30	25806	ES	4	3	5	0.00	8.6E-01	included
OSBPL9	2969	ES	17	15	18	0.00	8.6E-01	excluded
HACL1	63588	ES	6:07	5	8	0.00	8.6E-01	excluded
ABHD17A	46556	ES	4.3:5.1	4.2	5.2	0.00	8.6E-01	included
ZCCHC7	86398	ES	1.2:2	1.1	3	0.00	8.6E-01	included
CCDC90B	18078	ES	1.3:1.4:1.5:2	1.2	4.2	0.01	8.6E-01	included
TRIP12	57856	ES	3	2	4.1	0.00	8.6E-01	included
RASSF4	91547	ES	12	11	13	0.00	8.6E-01	included
RASSF4	11351	ES	7.2	6	8	0.00	8.6E-01	included
ZSWIM4	47939	ES	7	6	8	0.00	8.6E-01	excluded
ZNF706	84739	ES	5:06	4	7	0.00	8.6E-01	included

SUMF2	79802	ES	4:5.1:5.2:6	3	7	0.00	8.6E-01	included
MIOX	62832	ES	8:9.1:9.2	7	10	0.00	8.6E-01	excluded
CD47	66015	ES	8	7	11	0.00	8.6E-01	excluded
TK2	36722	ES	3	2.4	4	0.00	8.6E-01	included
DGKZ	15562	ES	7:8.1:8.2:9.1:9.2	2	10.2	0.00	8.6E-01	included
KAT7	42324	ES	4:05	3	6	0.00	8.6E-01	excluded
SLC30A6	53168	ES	2:03:04	1	6	0.00	8.6E-01	included
TATDN1	138620	ES	2:3:4.1:4.2:5:6	1.1	7	0.00	8.6E-01	included
PXK	65451	ES	2:03	1	4	0.00	8.6E-01	included
NRF1	81754	ES	4	3.2	5	0.00	8.6E-01	included
UBXN11	1252	ES	7:8:9:10	6	11	0.00	8.6E-01	included
SNAP23	30176	ES	8	6	9.1	0.00	8.6E-01	excluded
HGSNAT	83765	ES	3	2	4	0.00	8.6E-01	excluded
RBMS2	22463	ES	12:13.1	11	14	0.00	8.6E-01	excluded
ZNF263	33511	ES	3:4:5.1:6	2	7	0.00	8.6E-01	excluded
ENSA	7501	ES	4	3.1	5.1	0.00	8.6E-01	included
FKBP14	79103	ES	2	1	3	0.00	8.6E-01	included
PIP4K2A	10986	ES	7	6	8	0.00	8.6E-01	excluded
ATXN2	24517	ES	24	23	25	0.00	8.7E-01	excluded
ZFAT	85265	ES	14	13	15	0.00	8.7E-01	excluded
LY6E	85391	ES	4	2	5.1	0.00	8.7E-01	excluded
SCFD1	27083	ES	2	1.1	3	0.00	8.7E-01	included
KLHL2	71037	ES	5	4	7	0.00	8.7E-01	included
HARS2	73761	ES	2.1:2.2	1.3	3	0.00	8.7E-01	included
STARD3	40665	ES	3	2	4.1	0.00	8.7E-01	included
ARHGAP8	62634	ES	5:06:07	4	9	0.00	8.7E-01	included
GALNT10	74215	ES	9.3:10	9.2	11	0.00	8.7E-01	included
GALK2	30536	ES	2.2	1	6	0.00	8.7E-01	included
NUDT7	37664	ES	4	3.1	5	0.00	8.7E-01	excluded
DNM1L	21054	ES	6:7:8:9	5	10	0.00	8.7E-01	excluded
YY1AP1	8114	ES	3.2	2	4.1	0.00	8.7E-01	excluded
ZNF662	64333	ES	04:05.1	3.2	5.2	0.00	8.7E-01	excluded
LY6E	85386	ES	04:05.1	2	5.2	0.00	8.7E-01	included
SEPT2	58371	ES	7	2	9	0.00	8.7E-01	included
POMGNT2	64340	ES	2	1	3	0.00	8.7E-01	included
BTF3L4	2995	ES	3	2	4	0.00	8.7E-01	excluded
SSH3	17166	ES	2.1:2.2	1	3.1	0.00	8.7E-01	excluded
HAGH	33146	ES	6	5	7	0.00	8.7E-01	excluded
ARL13B	65688	ES	3.1:3.2:4	2	5.1	0.00	8.7E-01	excluded
GLYCTK	65209	ES	5.2	4	6.1	0.00	8.7E-01	excluded
CITED1	89455	ES	2.2:4	1	5	0.00	8.7E-01	included
IL1R1	54781	ES	8:9:10:11:12:13.1:13.	7	13.5	0.00	8.7E-01	included
CAPRIN2	20949	ES	12	11	13	0.00	8.7E-01	included
DUS2	37176	ES	4	3.2	5	0.00	8.7E-01	excluded
FAM173B	71547	ES	6	5	7	0.00	8.7E-01	excluded
NDUFV1	17252	ES	3	1.3	4	0.00	8.7E-01	excluded
POLA2	16809	ES	2	1	3	0.00	8.7E-01	included
CAP2	75452	ES	4	3	6	0.00	8.7E-01	excluded
HOMER3	48539	ES	5:6:7:8:9.1	4.2	9.2	0.00	8.7E-01	excluded
FAM110A	58468	ES	03:04.2	1	4.3	0.00	8.7E-01	included
PPA2	70199	ES	8	7	9	0.00	8.7E-01	excluded
YIPF6	89353	ES	2	1	3	0.00	8.7E-01	excluded
SCFD1	27070	ES	4:06	3	7	0.00	8.7E-01	included
SAR1A	12039	ES	2.2:3:4.1	1	4.2	0.00	8.7E-01	included
NDRG1	85250	ES	2.2:2.3:3:4:5.1:5.2:6:7.1:7.2:8:10:11:12:13:1	2.1	16.2	0.00	8.7E-01	included
ETF1	73553	ES	5	4	6	0.00	8.7E-01	excluded
NGLY1	63757	ES	6	5	7	0.00	8.7E-01	included
POGZ	7632	ES	5:6:7.2:8:9	4	10	0.00	8.7E-01	included
ZNF544	52436	ES	3.3:4.1:5.2:6.1	3.2	6.2	0.00	8.7E-01	included
DGKZ	15556	ES	7:8.1:8.2:9.2	3	10.2	0.00	8.7E-01	included
ITSN1	60473	ES	25	23	26	0.00	8.7E-01	excluded

LPHN2	3571	ES	15	14	16	0.00	8.7E-01	excluded
SPIRE1	44697	ES	6	4	7	0.00	8.7E-01	included
CCNL2	161	ES	6.1:6.2	5	7.2	0.00	8.7E-01	included
FN1	57370	ES	40.4:41	39	42	0.00	8.7E-01	included
ATP6V0E2	82214	ES	4.1:4.2	2	6	0.00	8.7E-01	included
ADAM15	7909	ES	20	19	21.2	0.00	8.7E-01	excluded
FNBP1	87878	ES	13:14.1	10.3	14.2	0.00	8.7E-01	excluded
HACL1	63598	ES	4	3	5	0.00	8.7E-01	excluded
DNAJA3	33724	ES	04:05.1	1	5.2	0.00	8.7E-01	included
PRSS16	75687	ES	4:5:6:7:8	3	9	0.00	8.7E-01	included
POLB	83710	ES	12	11	13.2	0.00	8.7E-01	included
SGCE	80499	ES	10	9	11	0.00	8.7E-01	included
OS9	22707	ES	5.1:6:7.1:7.3:7.4:8:9.1	4	9.2	0.00	8.7E-01	excluded
WDR41	72578	ES	8:9.1:9.2	7	10	0.00	8.7E-01	included
PLEKHG3	27904	ES	3:04	2	5	0.00	8.7E-01	included
DSCR3	60555	ES	3:04	2	5	0.00	8.7E-01	included
CLNS1A	17959	ES	5	4	6	0.00	8.7E-01	included
CENPN	37745	ES	4	3.1	5.1	0.00	8.7E-01	included
TIAM1	60333	ES	13	12	14	0.00	8.7E-01	excluded
CTSS	7505	ES	4	3	5	0.00	8.7E-01	included
CRTC1	48504	ES	4	3	5	0.00	8.7E-01	excluded
UPF3A	93462	ES	3	2	5	0.00	8.7E-01	included
EMC4	29845	ES	2.3:3.1:3.2	2.2	6	0.00	8.7E-01	excluded
DNM1L	21048	ES	17	16	18	0.00	8.7E-01	excluded
C9orf85	86580	ES	4	1	5	0.00	8.7E-01	included
PLTP	59650	ES	5:06	3	7	0.00	8.7E-01	included
EFEMP1	53639	ES	2.2	1	3	0.00	8.7E-01	included
NQO2	75158	ES	8	7	9	0.00	8.7E-01	included
ISCU	24233	ES	5.3:5.4	5.1	6	0.00	8.7E-01	included
NFKBIA	27238	ES	2.2	1	3	0.00	8.7E-01	excluded
PRMT5	26661	ES	4.1:4.2:5:6:7	3	8	0.00	8.7E-01	excluded
CLTA	86331	ES	05:06.1	4	7.1	0.00	8.7E-01	excluded
SKA2	42750	ES	4.1:4.2	1.1	5	0.00	8.7E-01	included
RBM6	64951	ES	3.1:3.2:4:5	2	7	0.00	8.7E-01	included
DET1	32382	ES	4	3.6	5	0.00	8.7E-01	excluded
SSH1	24258	ES	12	11	13	0.00	8.7E-01	excluded
ZFYVE27	12742	ES	2.2:3	2.1	4	0.00	8.7E-01	excluded
ULK3	31769	ES	2.2	1	3.2	0.00	8.7E-01	included
SHROOM1	73309	ES	5	4	6	0.00	8.7E-01	excluded
CLASP1	55171	ES	28.2:30	28.1	31.1	0.00	8.7E-01	included
CCDC25	83183	ES	2	1	4	0.00	8.7E-01	included
SGK1	77776	ES	10	9	11	0.00	8.7E-01	excluded
PNPLA4	88441	ES	3	1	4	0.00	8.7E-01	excluded
DNAJC25	87230	ES	2.2	1	3.2	0.00	8.7E-01	included
EML4	53354	ES	7	6	8	0.00	8.7E-01	excluded
STAT6	22528	ES	4:05	2.1	6	0.00	8.7E-01	excluded
SRRM1	1130	ES	11	10.2	12.1	0.00	8.7E-01	excluded
ZSWIM7	39400	ES	2:03	1.2	4	0.00	8.7E-01	included
ZBTB49	68661	ES	4:05	3.2	6	0.00	8.7E-01	excluded
ZFAND6	32174	ES	5	1	6	0.00	8.7E-01	included
PGS1	43875	ES	4	3.2	5	0.00	8.8E-01	excluded
CHMP5	86099	ES	7	6	8	0.00	8.8E-01	included
DEAF1	13725	ES	5	4	6	0.00	8.8E-01	excluded
MTHFSD	37931	ES	4.1:5:7	2.2	8	0.00	8.8E-01	excluded
SEC11A	32315	ES	7	5	8	0.00	8.8E-01	included
MTFMT	31157	ES	3:04	2	5	0.00	8.8E-01	excluded
ZFP1	37588	ES	4.1	3	5	0.00	8.8E-01	excluded
PTK2	85307	ES	35	34	36.1	0.00	8.8E-01	included
MAPK7	39725	ES	3:05	1	6	0.00	8.8E-01	included
C12orf4	19770	ES	2.1:2.2	1	3	0.00	8.8E-01	excluded
NADSYN1	17428	ES	9.2:10	9.1	11	0.00	8.8E-01	excluded
TK2	36720	ES	3:04	2.4	5	0.00	8.8E-01	included

MAPK14	75947	ES	11	9.1	12	0.00	8.8E-01	included
ZNF195	13981	ES	11	5.1	13	0.00	8.8E-01	included
H2AFY	73454	ES	4:5:6.2:6.3:7	3	9	0.00	8.8E-01	included
ARL13B	65684	ES	3.1:3.2:4:5.1	2	6	0.00	8.8E-01	excluded
RCN2	31958	ES	2:03	1	5	0.00	8.8E-01	included
NEDD1	23841	ES	2.2:2.3	1	3	0.00	8.8E-01	included
UBE2G1	38530	ES	4:05	3	6	0.00	8.8E-01	included
IFT46	19024	ES	12	11	13	0.00	8.8E-01	included
RPL34	70302	ES	2	1.2	3	0.00	8.8E-01	excluded
TXNRD2	61093	ES	3.1:3.2	1	4	0.00	8.8E-01	included
MSI2	42619	ES	6	5	7	0.00	8.8E-01	included
UBE2E2	63713	ES	6	4	7	0.00	8.8E-01	excluded
ACY1	390892	ES	8.1:8.2:9:10:11:14	7.2	15	0.00	8.8E-01	included
TUBD1	42810	ES	7	6	8.1	0.00	8.8E-01	excluded
PTPRM	44570	ES	19:20	18	22	0.00	8.8E-01	excluded
CASP8	56818	ES	10	9	11	0.00	8.8E-01	included
NACA	22494	ES	3.1:3.2:3.3:3.5	2.2	4.2	0.00	8.8E-01	included
GMFG	49783	ES	3.2:4:5	2	6	0.00	8.8E-01	included
MINK1	38597	ES	22	21	23	0.00	8.8E-01	excluded
DCTN1	54049	ES	7.2:8	5	9	0.00	8.8E-01	excluded
MYL6	22380	ES	2.2:3.1	2.1	3.2	0.00	8.8E-01	excluded
DGKD	101805	ES	29	28	30	0.00	8.8E-01	excluded
NRF1	81753	ES	8	7	9	0.00	8.8E-01	excluded
PKP4	55684	ES	5	3	9	0.00	8.8E-01	included
GNB2L1	127737	ES	7.2:8.1:8.2	7.1	9	0.00	8.8E-01	included
LCLAT1	53117	ES	3	2	4	0.00	8.8E-01	excluded
PDE2A	17623	ES	5:6:7.1:7.2:8:9	4.2	10.1	0.00	8.8E-01	excluded
SLBP	68503	ES	2	1	3	0.00	8.8E-01	included
ADAM8	13524	ES	17	16	18	0.00	8.8E-01	included
SDHA	71419	ES	13:14	12	15	0.00	8.8E-01	included
MDM2	22984	ES	8.1:8.2	7	9	0.00	8.8E-01	excluded
NONO	89417	ES	2:03:04	1	5	0.00	8.8E-01	included
CDS1	69810	ES	2	1	3	0.00	8.8E-01	included
STRA13	44266	ES	3.1	2	4.1	0.00	8.8E-01	excluded
TIA1	53876	ES	5	4	7	0.00	8.8E-01	included
NAGK	53918	ES	2:03	1	4	0.00	8.8E-01	included
CPNE2	36524	ES	2	1	3.2	0.00	8.8E-01	excluded
CD44	15267	ES	3.1:3.2	2.1	4	0.00	8.8E-01	excluded
PAK1	17955	ES	3	2	4	0.00	8.8E-01	included
RPL30	84640	ES	2.1	1.3	3.1	0.00	8.8E-01	excluded
ACTR10	27676	ES	10:11	9	12	0.00	8.8E-01	excluded
SEPT9	43744	ES	4	1	5	0.00	8.8E-01	excluded
BET1L	13579	ES	4.1	3	4.3	0.00	8.8E-01	included
DCAF10	86447	ES	5	4	6	0.00	8.8E-01	excluded
NDUFA10	58243	ES	2	1	3.1	0.00	8.8E-01	included
CDC27	42052	ES	9.1:9.2:10:11:12.2:13:	8	15	0.00	8.8E-01	excluded
KCTD10	24298	ES	03:04.3	2.2	4.4	0.00	8.8E-01	excluded
TMEM8A	32872	ES	1.2:2.1	1.1	2.2	0.00	8.8E-01	excluded
FN1	57367	ES	40.2:40.3:40.4:41	39	42	0.00	8.8E-01	excluded
DDX5	43063	ES	4.2:5:6.1:6.2:7:8.1	4.1	8.2	0.00	8.8E-01	excluded
ZCCHC17	1463	ES	2:03	1.1	4	0.00	8.8E-01	included
SKA2	42732	ES	3:4.1:4.2	2	5	0.00	8.8E-01	excluded
HACL1	63604	ES	3:4:5:8:9:10	2	11	0.00	8.8E-01	included
AKAP17A	88394	ES	4.3	4.1	5	0.00	8.8E-01	excluded
SMPD4	55307	ES	4.1:4.2	3	5	0.00	8.8E-01	excluded
WBP1	54066	ES	02:03.1	1	3.2	0.00	8.8E-01	excluded
AATF	40502	ES	2.2:3:4.1	2.1	4.2	0.00	8.8E-01	excluded
DEPDC5	61904	ES	7	6	8	0.00	8.8E-01	included
SLC6A13	19594	ES	03:04.2	2.1	6	0.00	8.8E-01	excluded
RTN3	16523	ES	5	4	6	0.00	8.8E-01	excluded
ARPP19	30673	ES	5.2	2.6	6	0.00	8.8E-01	excluded
SWAP70	14332	ES	4	2	5	0.00	8.8E-01	excluded

LGALS3BP	43937	ES	3.2:4.1:4.2:5.2	3.1	5.3	0.00	8.8E-01	included
SORBS1	12636	ES	14:16	13	17	0.01	8.8E-01	included
HPSE	69782	ES	10	9	11	0.00	8.8E-01	included
SH3YL1	52502	ES	12:13	10	15	0.00	8.8E-01	excluded
MVK	24338	ES	4:05:06	3	7	0.00	8.8E-01	excluded
ATG13	15590	ES	4.1:4.2	1.1	5	0.00	8.8E-01	excluded
SPP1	69873	ES	5	4	6	0.00	8.8E-01	excluded
TPD52L2	60170	ES	6:07	5	8	0.00	8.8E-01	excluded
GLIPR2	86326	ES	2:03	1	4	0.00	8.8E-01	included
PPIL3	56763	ES	5.2	3	6	0.00	8.8E-01	excluded
PSMD1	57923	ES	21	20	22	0.00	8.8E-01	excluded
ATP11C	90245	ES	32	29	33	0.00	8.8E-01	included
ACP2	15688	ES	5.3:6.3	5.1	8	0.00	8.8E-01	excluded
PRC1	32532	ES	3	2	4	0.00	8.8E-01	excluded
TM7SF2	16762	ES	4:05	3	6	0.00	8.8E-01	excluded
ACY1	65152	ES	12:13	11	14	0.00	8.8E-01	included
RASSF4	11350	ES	7.1:7.2	6	8	0.00	8.8E-01	excluded
CSGALNACT1	82883	ES	6	5	8	0.00	8.8E-01	excluded
ZNF268	25351	ES	10.1:10.2:11	9	13	0.00	8.8E-01	excluded
ITPK1	29022	ES	3	2.2	4	0.00	8.8E-01	included
NMU	69332	ES	7	6	8	0.00	8.8E-01	included
ACRBP	19905	ES	3	2	4	0.00	8.8E-01	included
VOPP1	79756	ES	11	9	12	0.00	8.8E-01	included
FXR1	67751	ES	5	1	6	0.00	8.8E-01	excluded
EIF3E	84878	ES	3	1	4.2	0.00	8.8E-01	excluded
TRAF5	9716	ES	7:08:09	6	10	0.00	8.8E-01	included
FANK1	13450	ES	2:05	1	6.1	0.00	8.8E-01	included
SSR2	8153	ES	5:07	4.1	8	0.00	8.8E-01	excluded
BTRC	12872	ES	3	2	4	0.00	8.8E-01	excluded
WDR59	37575	ES	3	2	4	0.00	8.8E-01	excluded
RAPGEF4	55992	ES	32:33:34	31	35	0.00	8.8E-01	included
PICK1	62193	ES	3	2.2	4	0.00	8.8E-01	included
STX16	59985	ES	1.5:2	1.4	5.1	0.00	8.8E-01	included
MUC15	14741	ES	2.2	1	3	0.00	8.8E-01	excluded
RPP14	100831	ES	2.2	1.2	3	0.00	8.9E-01	excluded
XRRA1	17797	ES	10	9	13	0.00	8.9E-01	included
LRP5	17303	ES	9	8	10	0.00	8.9E-01	excluded
PLAC9	12341	ES	4	3	5	0.00	8.9E-01	included
SH3YL1	52503	ES	11:13	10	15	0.00	8.9E-01	excluded
MTERFD1	84622	ES	7	6	9	0.00	8.9E-01	excluded
DGCR2	61038	ES	2	1	3	0.00	8.9E-01	included
HSF2	77374	ES	11	10	12	0.00	8.9E-01	excluded
CPZ	68775	ES	8	7	9	0.00	8.9E-01	included
ZNF569	49518	ES	1.2:2	1.1	4	0.00	8.9E-01	excluded
FBXL2	63857	ES	7:08	3	9	0.00	8.9E-01	excluded
ST3GAL3	2271	ES	11:12	9	13	0.00	8.9E-01	included
PTGES3	93182	ES	6	4.2	7	0.00	8.9E-01	included
NDOR1	88286	ES	5	4	6	0.00	8.9E-01	included
TUBD1	42808	ES	07:08.1	6	9	0.00	8.9E-01	excluded
SLCO2B1	17831	ES	3:04	1	5	0.00	8.9E-01	included
CCDC90B	18074	ES	1.3:1.4:1.5:2:3	1.2	4.2	0.00	8.9E-01	excluded
SLC47A1	39745	ES	7:8:9:10.2:11:12:13:1	6	17	0.00	8.9E-01	excluded
POLI	45580	ES	5	4.2	6	0.00	8.9E-01	excluded
GLUL	9151	ES	1.4	1.1	2	0.00	8.9E-01	excluded
ZNF74	61154	ES	2	1.3	4	0.00	8.9E-01	excluded
NSMF	88325	ES	6	4	7	0.00	8.9E-01	excluded
GLE1	87769	ES	4	3	5	0.00	8.9E-01	included
TOM1L2	39527	ES	4	3	5	0.00	8.9E-01	excluded
OS9	22712	ES	5.1:5.2:5.3:7.1:7.4:8:9.	4	9.2	0.00	8.9E-01	included
TDG	24084	ES	3	1	4	0.00	8.9E-01	included
SHANK3	62902	ES	24	23	25	0.00	8.9E-01	included
TSN	55180	ES	6	5	7	0.00	8.9E-01	excluded

HSD17B10	89224	ES	5.1	4	6	0.00	8.9E-01	included
TIMMDC1	66322	ES	1.2:2	1.1	3	0.00	8.9E-01	included
PCK2	26820	ES	11	10	12.1	0.00	8.9E-01	excluded
SF3A3	1853	ES	3:04	2	5	0.00	8.9E-01	excluded
DLD	81385	ES	3:04	2	5	0.00	8.9E-01	excluded
CCND1	17365	ES	2:03	1	4.1	0.00	8.9E-01	included
ZNF140	25324	ES	07:08.1	5.3	8.2	0.00	8.9E-01	excluded
ZNF821	37501	ES	7.2:8.1	7.1	9.1	0.00	8.9E-01	excluded
RNF185	61829	ES	4	3	5	0.00	8.9E-01	excluded
CAPZB	906	ES	11	10	12	0.00	8.9E-01	included
C5orf22	71634	ES	5	4.3	6	0.00	8.9E-01	excluded
RBMS2	22462	ES	12:13.1:13.2	11	14	0.00	8.9E-01	included
PHLDB2	66067	ES	10	9	11	0.00	8.9E-01	excluded
ATP7A	89537	ES	10	9	11	0.00	8.9E-01	excluded
SUMF2	79786	ES	08:10.1	7	10.2	0.00	8.9E-01	excluded
YAF2	21141	ES	3.1:3.2:5.2:7:8	2	9.1	0.00	8.9E-01	included
RPL7A	88035	ES	3.2:4.1	3.1	4.2	0.00	8.9E-01	included
WDR19	69053	ES	3	2	4	0.00	8.9E-01	included
TMEM106C	21392	ES	4.1:4.2	3	5.1	0.00	8.9E-01	excluded
RPTOR	44083	ES	14:15:16:17	13.1	18	0.00	8.9E-01	included
EMID1	61576	ES	11	10	12	0.00	8.9E-01	included
PALLD	71126	ES	25	24	26	0.00	8.9E-01	excluded
TXNDC11	34031	ES	6	5	7	0.00	8.9E-01	included
VPS4B	45715	ES	4.1	3	5	0.00	8.9E-01	included
MOCS2	72003	ES	5:06	4	7.1	0.00	8.9E-01	excluded
ZFAT	85263	ES	18.2:19.1	18.1	19.2	0.00	8.9E-01	excluded
XRN1	67114	ES	3:04:05	2	6	0.00	8.9E-01	included
PRPSAP2	39668	ES	6.2:7	5	8	0.00	8.9E-01	included
BBX	66008	ES	17	15	18	0.00	8.9E-01	excluded
LRCH1	25843	ES	16	15	17	0.00	8.9E-01	excluded
ATF7IP2	33978	ES	8:09	7	10	0.00	8.9E-01	excluded
LAMP5	58680	ES	3	2	4	0.00	8.9E-01	included
WBP1	54068	ES	2	1	3.1	0.00	8.9E-01	excluded
TOM1	61953	ES	11	10	12	0.00	8.9E-01	included
R3HDM4	46347	ES	4.1	3.4	5.1	0.00	8.9E-01	excluded
ADAMTS13	88053	ES	17:18	16	19	0.00	8.9E-01	excluded
TOM1	61955	ES	7.1:7.2	6.2	8	0.00	8.9E-01	excluded
LMO3	20632	ES	6.3	3	9.1	0.00	8.9E-01	included
CNOT8	74281	ES	1.4:2:3	1.1	5	0.00	8.9E-01	excluded
TSSC1	52552	ES	6	4	7	0.00	8.9E-01	excluded
RABL2B	62927	ES	2.1:2.2:2.3:3.1	1	3.2	0.00	8.9E-01	excluded
SUMF1	62989	ES	4:05:06	2	7	0.00	8.9E-01	included
CLN3	35727	ES	9:10:11	8	12.1	0.00	8.9E-01	excluded
CALU	81704	ES	8	7	9	0.00	8.9E-01	included
QKI	78413	ES	4	3	5	0.00	8.9E-01	excluded
USP3	31055	ES	3.2:5:7:8:9:10	1	11	0.00	8.9E-01	excluded
KLHL7	78954	ES	3:04	1	5	0.00	8.9E-01	excluded
ZNF266	47345	ES	4.1:5	3	6.2	0.00	8.9E-01	excluded
SUN2	62261	ES	9	8	10.2	0.00	8.9E-01	excluded
ARNTL	14440	ES	14.1:14.2:15	13	16	0.00	8.9E-01	excluded
ZDHHC3	64393	ES	2.2:3	2.1	4	0.00	8.9E-01	excluded
CD99L2	90362	ES	4:05	2	6	0.00	8.9E-01	included
MCM7	80882	ES	9:10:11:12:13:14:15.1	8	15.2	0.00	8.9E-01	included
PCBP2	22058	ES	12	11.2	13	0.00	8.9E-01	included
RTN4IP1	77121	ES	5:06:07	4	8	0.00	8.9E-01	included
BRD8	73513	ES	4	2.1	5.1	0.00	8.9E-01	excluded
MAP2K5	31333	ES	21	20	22	0.00	8.9E-01	excluded
COPZ1	22170	ES	4:05	3.2	6	0.00	8.9E-01	included
PIGG	68361	ES	9.1:9.2:9.3	8	10.1	0.00	8.9E-01	excluded
ARAF	88923	ES	7:08	6.1	9	0.00	8.9E-01	excluded
SMIM14	69074	ES	3	2	4	0.00	8.9E-01	included
NOL8	86853	ES	7:08:09	5	10	0.00	8.9E-01	included

ACOT8	59631	ES	4	1	5	0.00	9.0E-01	excluded
LRRC28	32684	ES	3:5:6:8:10:11	2	12	0.00	9.0E-01	included
SLC7A6	37213	ES	3	2.2	4	0.00	9.0E-01	excluded
SNX11	42176	ES	2:3.1:3.2:4:5	1.2	6	0.00	9.0E-01	excluded
CRYZ	3467	ES	8	7	9	0.00	9.0E-01	excluded
RAD17	72365	ES	2.2:3.2	2.1	5	0.00	9.0E-01	excluded
MFGE8	32407	ES	2	1	3	0.00	9.0E-01	excluded
CCT3	8234	ES	5:06	4	7	0.00	9.0E-01	included
ITGA3	42346	ES	25	24	26	0.00	9.0E-01	included
MTF2	3772	ES	2.1:2.2:2.3:3	1	4	0.00	9.0E-01	excluded
PAK4	49758	ES	5.1:5.2	4	6	0.00	9.0E-01	excluded
ACTR3	55082	ES	3	1	4	0.00	9.0E-01	included
NELFA	68543	ES	2	1	3	0.00	9.0E-01	excluded
MLH1	63937	ES	4.1:4.2	2.2	5	0.00	9.0E-01	included
TXLNG	88577	ES	2:03	1	4	0.00	9.0E-01	excluded
SYNJ2	78248	ES	24	23	25	0.00	9.0E-01	excluded
CD4	19972	ES	3	2	4	0.00	9.0E-01	included
TEAD4	19747	ES	3	2.2	4	0.00	9.0E-01	included
MS4A4A	16081	ES	7	6	8	0.00	9.0E-01	excluded
NR1H3	15703	ES	4	3.5	5.2	0.00	9.0E-01	excluded
ZNF19	37455	ES	5.2	4	7	0.00	9.0E-01	excluded
GSTM4	4053	ES	2.4:2.5:3	2.3	4	0.00	9.0E-01	included
DTNA	45109	ES	20:21	17	22	0.00	9.0E-01	excluded
TRIM6	14061	ES	3.1:3.2:3.3:3.4:4.1	2	5	0.00	9.0E-01	excluded
PITPNB	61522	ES	12	11	13	0.00	9.0E-01	included
NPHP3	66813	ES	15	14	16	0.00	9.0E-01	included
WLS	3364	ES	3.1:3.2	1	4	0.00	9.0E-01	included
ZNF76	75906	ES	5	4	6	0.00	9.0E-01	included
RFC1	69057	ES	5	4	6	0.00	9.0E-01	excluded
C2orf43	52789	ES	3	2	5	0.00	9.0E-01	included
DHX8	41707	ES	2	1	3	0.00	9.0E-01	excluded
WIPF2	40853	ES	5:06	4.2	7	0.00	9.0E-01	included
ALPL	978	ES	3	1	4	0.00	9.0E-01	excluded
USHBP1	48250	ES	3.1	2	3.3	0.00	9.0E-01	excluded
LRRC28	32665	ES	4	3	5	0.00	9.0E-01	included
GABARAPL1	20410	ES	2.10:2.12	2.6	3	0.00	9.0E-01	included
NFATC2IP	35903	ES	4:05:06	3	7.2	0.00	9.0E-01	included
KIAA0753	38763	ES	4	3	6	0.00	9.0E-01	included
UBE2B	73396	ES	3:04	2	5	0.00	9.0E-01	excluded
CHRAC1	85283	ES	2	1	3	0.00	9.0E-01	included
TARS	71673	ES	6.1:6.2:7.1	4	7.2	0.00	9.0E-01	excluded
DPH2	2499	ES	3.2	2.1	4	0.00	9.0E-01	excluded
NEDD4L	45658	ES	18:19	17	20	0.00	9.0E-01	included
CNOT8	74276	ES	1.2:1.3:1.4:1.5:2:3	1.1	5	0.00	9.0E-01	excluded
TOPORS	86066	ES	2	1	3	0.00	9.0E-01	included
MARK3	29450	ES	17	16	19	0.00	9.0E-01	excluded
CLTA	86332	ES	6.1	4	7.1	0.00	9.0E-01	excluded
STX5	16444	ES	4	2.2	5	0.00	9.0E-01	included
LPCAT3	20066	ES	2	1	3	0.00	9.0E-01	included
IP6K2	64769	ES	09:11.1	7	11.2	0.00	9.0E-01	excluded
MED27	87971	ES	6	5	7	0.00	9.0E-01	included
RABGEF1	79900	ES	6	3	7	0.00	9.0E-01	excluded
HINT1	73216	ES	2.2:2.3:3.1:3.2	2.1	4	0.00	9.0E-01	excluded
NAA16	25740	ES	13.3	13.1	14	0.00	9.0E-01	included
ZNF211	52322	ES	2.2:4	2.1	5	0.00	9.0E-01	excluded
PGPEP1	48421	ES	5.1	4	6.1	0.00	9.0E-01	included
MAGI3	4272	ES	22.1	21	23	0.00	9.0E-01	included
TRAPPC4	19062	ES	2.4:3.1:3.2	2.3	4.1	0.00	9.0E-01	included
ACAA1	64027	ES	4	2	5	0.00	9.0E-01	included
GIT2	24382	ES	17.2:18.2	17.1	20	0.00	9.0E-01	excluded
ADPGK	31590	ES	1.2:1.3:2:3	1.1	4	0.00	9.0E-01	included
RPP38	10864	ES	2	1.1	3.1	0.00	9.0E-01	excluded

TPD52	84277	ES	10	8	11	0.00	9.0E-01	excluded
GPR162	19974	ES	2.1:2.2	1	3	0.00	9.0E-01	included
DAP	71571	ES	3	2	4	0.00	9.0E-01	included
DSN1	59310	ES	3.1:3.2	2	4	0.00	9.0E-01	excluded
ZNF415	51690	ES	3	1.1	4	0.00	9.0E-01	excluded
CTSE	9583	ES	2	1	3	0.00	9.0E-01	included
C16orf58	36280	ES	4:05	3	6	0.00	9.0E-01	excluded
XPNPEP1	13073	ES	4	3	5	0.00	9.0E-01	excluded
POLR3GL	7278	ES	4	3	5	0.00	9.0E-01	included
TMEM237	56854	ES	4	3	5	0.00	9.0E-01	excluded
BSCL2	16411	ES	3	2.2	5	0.00	9.0E-01	included
APLP2	19481	ES	6.1:6.2:7:8	5.2	10	0.00	9.1E-01	included
LDB2	68850	ES	4	2	5	0.00	9.1E-01	included
ARHGEF11	8338	ES	39	38	40	0.00	9.1E-01	included
SMIM8	76950	ES	2.1:2.2	1.1	3	0.00	9.1E-01	included
ANAPC15	17573	ES	4.1:4.2	1.2	5	0.00	9.1E-01	excluded
ORC4	55528	ES	4	2.1	5	0.00	9.1E-01	included
DMXL2	30618	ES	17:18	16	19	0.00	9.1E-01	included
AKAP10	39783	ES	03:04.1	2	4.2	0.00	9.1E-01	included
DAP3	8122	ES	4	3	5.1	0.00	9.1E-01	included
NEIL2	82637	ES	1.3	1.1	2.1	0.00	9.1E-01	included
TSC2	33196	ES	5:06:07	4	8	0.00	9.1E-01	excluded
SERGEF	14560	ES	6	5	7	0.00	9.1E-01	excluded
ATP2A3	38513	ES	21.1:21.2:22	20	23.1	0.00	9.1E-01	included
C14orf79	29592	ES	2.2:2.4	2.1	3.2	0.00	9.1E-01	included
HSD17B11	69862	ES	3	2	4	0.00	9.1E-01	included
ABCC1	34214	ES	17	16	18	0.00	9.1E-01	included
NAV1	9392	ES	13	12	14	0.00	9.1E-01	excluded
HKR1	49500	ES	8.1:8.2	7	12	0.00	9.1E-01	included
SGK3	84034	ES	16	15	17	0.00	9.1E-01	included
C5orf45	74969	ES	2.2:2.3:4:5.1:5.2	2.1	6	0.00	9.1E-01	excluded
METTL15	14786	ES	11	10.2	12.1	0.00	9.1E-01	included
DUS4L	81344	ES	5.1:5.2	4	6	0.00	9.1E-01	excluded
TTLL1	62568	ES	4	3	5.1	0.00	9.1E-01	included
TNFAIP2	29438	ES	3.1:3.2:4	2.2	5	0.00	9.1E-01	excluded
TOR2A	87658	ES	3.3	3.1	3.5	0.00	9.1E-01	included
ERG	60593	ES	11.1	10	12	0.00	9.1E-01	excluded
TMEM33	69136	ES	3	2	5	0.00	9.1E-01	included
MTMR10	29795	ES	4	3	5	0.00	9.1E-01	excluded
AUTS2	79911	ES	13	12	14	0.00	9.1E-01	excluded
COL12A1	76773	ES	3:4:5:6:7:8:9:10:11:12:13:14:15:16:17	2	18	0.00	9.1E-01	included
NDRG3	59304	ES	4:05	3	6	0.00	9.1E-01	included
CCDC53	24025	ES	3	2	6	0.00	9.1E-01	excluded
RAD51D	40248	ES	8	7	9	0.00	9.1E-01	included
ATG13	15594	ES	3	1.1	4.1	0.00	9.1E-01	excluded
PEMT	39494	ES	6:07	4	8	0.00	9.1E-01	included
KIAA0430	34168	ES	15	14	16	0.00	9.1E-01	excluded
BRCA1	41200	ES	9:10:11.1:11.2	8.2	12	0.00	9.1E-01	included
TNFAIP2	29440	ES	2.2:3.1	2.1	3.2	0.00	9.1E-01	excluded
SYT17	34298	ES	2.3:2.5	1	2.6	0.00	9.1E-01	included
PARD3	11211	ES	20	19.3	21	0.00	9.1E-01	included
CCDC25	83181	ES	3	2	4	0.00	9.1E-01	excluded
SMARCA2	85726	ES	33	32	34	0.00	9.1E-01	included
KLHDC2	27438	ES	11	10	12	0.00	9.1E-01	excluded
ANXA11	12348	ES	1.2:5.2	1.1	5.3	0.00	9.1E-01	included
SUCLA2	25851	ES	6	5	7	0.00	9.1E-01	included
PAPLN	28281	ES	19.1:19.2	18	20	0.00	9.1E-01	excluded
ADAM15	7914	ES	20:21.1:21.2:22.1	19	23	0.00	9.1E-01	excluded
PIGT	59561	ES	4	1	5.2	0.00	9.1E-01	included
PRPSAP2	39666	ES	9	8	10	0.00	9.1E-01	excluded
WDR41	72588	ES	2:03:04	1.1	5	0.00	9.1E-01	excluded

NME4	32880	ES	3.2:3.3	2	5	0.00	9.1E-01	included
MORN1	255	ES	2	1	3	0.00	9.1E-01	included
NCAPG2	82527	ES	28	27	29	0.00	9.1E-01	excluded
UCK2	8814	ES	4.3	3	5	0.00	9.1E-01	included
ABI2	57055	ES	5.3:8:10:11:12	5.2	14	0.00	9.1E-01	excluded
NUDT7	37668	ES	02:03.1	1	5	0.00	9.1E-01	included
STARD3	40661	ES	4.2:5	4.1	6	0.00	9.1E-01	included
MDM2	22997	ES	7:8.1:8.2	6	9	0.00	9.1E-01	excluded
PPA2	70206	ES	4:5:7:8	2	9	0.00	9.1E-01	excluded
NDRG2	26493	ES	16	15.2	17	0.00	9.1E-01	included
PPP2R5D	76205	ES	2:3.2:3.3:3.4:4.1	1	4.2	0.00	9.1E-01	included
DAG1	64884	ES	4:05	2.1	7	0.00	9.1E-01	excluded
RNF135	40140	ES	2	1	5	0.00	9.1E-01	included
ZNF561	47374	ES	2	1	3	0.00	9.1E-01	included
ZNF226	50296	ES	3.1	1	3.3	0.00	9.1E-01	included
RTFDC1	59882	ES	3	1	4	0.00	9.1E-01	excluded
GREB1L	44769	ES	13	12	14	0.00	9.1E-01	included
FAM118B	19372	ES	5	4	6	0.00	9.1E-01	included
FCGRT	50967	ES	3:04:07	2.2	8.1	0.00	9.1E-01	excluded
ADCK3	10038	ES	14	13	15	0.00	9.1E-01	excluded
BRCC3	90675	ES	8:09	7	10	0.00	9.1E-01	excluded
FBXL2	63848	ES	11.3:11.4:12:13.1	11.2	13.2	0.00	9.1E-01	excluded
PCBP4	65127	ES	9.3	9.1	10	0.00	9.1E-01	included
MMP19	22278	ES	3.1:3.2:4.1:4.2	2	5	0.00	9.1E-01	included
AMPD2	4049	ES	2.2	1	4	0.00	9.1E-01	excluded
STRADB	56841	ES	4	3	5	0.00	9.1E-01	included
OSBPL9	2981	ES	5	4	6.1	0.00	9.1E-01	excluded
WSB1	39843	ES	2.1:2.2:3	1	4	0.00	9.1E-01	included
RNGTT	76984	ES	2	1	3	0.00	9.1E-01	included
ANKDD1A	31142	ES	12	11	13	0.00	9.1E-01	included
DCP2	73002	ES	8	7	9	0.00	9.1E-01	included
CALCRL	56489	ES	4	3	5	0.00	9.1E-01	excluded
FIG4	77212	ES	7:08	6	9.1	0.00	9.1E-01	excluded
HEATR6	42846	ES	13.2:14:15:16.1	13.1	16.2	0.00	9.1E-01	included
MRPL55	10099	ES	2.5	2.2	2.9	0.00	9.1E-01	excluded
LRRK1	32750	ES	23	22	24	0.00	9.1E-01	included
LRRC37B	40173	ES	7:08	6	9	0.00	9.1E-01	included
RIC8B	24167	ES	13:14	11	17	0.00	9.1E-01	included
DHPS	47834	ES	4	3.2	5	0.00	9.1E-01	included
INTS4	17995	ES	5.1:5.2	4	6	0.00	9.1E-01	excluded
PSMD9	24911	ES	5.1	4	6.1	0.00	9.1E-01	included
PRMT3	14723	ES	1.3	1.1	2	0.00	9.1E-01	excluded
RAB6A	17710	ES	6:07:08	4	9	0.00	9.1E-01	excluded
MAN2C1	31870	ES	5	4	6	0.00	9.1E-01	excluded
ATP9B	46236	ES	9	8	10	0.00	9.1E-01	excluded
PMM2	33920	ES	6:07:08	3	9.1	0.00	9.1E-01	included
MED16	46338	ES	13	11	15	0.00	9.1E-01	excluded
PNPT1	53632	ES	2:03	1	4	0.00	9.1E-01	excluded
ICAM1	47490	ES	03:04.1	1	4.2	0.00	9.1E-01	excluded
TEX30	26219	ES	3.2	1	3.4	0.00	9.1E-01	included
SUMF2	79801	ES	4	3	6	0.00	9.1E-01	included
MBNL1	67316	ES	11	9	13	0.00	9.1E-01	excluded
C3orf17	66173	ES	2:3.1:4.1:4.2:5.1:6.1:6.	1	8	0.00	9.1E-01	included
CELF2	10746	ES	5	4.2	6	0.00	9.1E-01	excluded
WLS	3368	ES	2	1	3.1	0.00	9.1E-01	included
CANX	74921	ES	4:05	3	6	0.00	9.1E-01	included
WDR48	64122	ES	10:11	9	12	0.00	9.1E-01	excluded
ENTPD6	58868	ES	3.1:3.2	1	4	0.00	9.1E-01	included
CLCN2	67940	ES	3	2	4	0.00	9.1E-01	included
BANP	37993	ES	9	6.2	10	0.00	9.1E-01	included
CBLB	66004	ES	5	4	6	0.00	9.1E-01	included
PHLPP2	37477	ES	14	13	15	0.00	9.1E-01	included

HERC2	29747	ES	52	51	53	0.00	9.1E-01	included
SIRT2	49711	ES	6	4	7	0.00	9.1E-01	excluded
SELP	8935	ES	7	6	8	0.00	9.2E-01	excluded
PKM	31513	ES	5.2:6:7:8:9.1	5.1	9.2	0.00	9.2E-01	included
SNX1	139175	ES	7:8:9:10.2:11:12:13.1:	3	16.1	0.00	9.2E-01	excluded
DTNB	52872	ES	5:06:07	4	8	0.00	9.2E-01	excluded
IP6K1	64915	ES	2	1.2	3	0.00	9.2E-01	included
NOX4	18234	ES	16:17:18	15	19	0.00	9.2E-01	excluded
EMC4	29849	ES	2.1:2.2:2.3	1	3.1	0.00	9.2E-01	included
SMIM7	48194	ES	05:06.1	2	7.1	0.00	9.2E-01	included
ATP5A1	300056	ES	3.3:4.2:5	3.2	6.1	0.00	9.2E-01	excluded
GZMB	27018	ES	4	3.2	5.1	0.00	9.2E-01	included
CYP4B1	2840	ES	3.2	2	5	0.00	9.2E-01	excluded
NAA25	24573	ES	5	4	6	0.00	9.2E-01	included
HARS	73723	ES	8	7	9	0.00	9.2E-01	excluded
CYB5RL	3132	ES	8	7	9	0.00	9.2E-01	included
C3orf17	66157	ES	2:3.1:4.1:4.2:5.1:6.1:6.	1	8	0.00	9.2E-01	excluded
ATP6V0A1	41051	ES	4	3	5	0.00	9.2E-01	excluded
EZH1	41119	ES	2	1	3	0.00	9.2E-01	excluded
NDEL1	39190	ES	10:11	9	12.1	0.00	9.2E-01	excluded
DPP8	31175	ES	17:18:19	16	20	0.00	9.2E-01	included
MTMR14	63150	ES	2	1	3	0.00	9.2E-01	included
ASH2L	83370	ES	15	14	16	0.00	9.2E-01	excluded
USP28	18808	ES	2:03	1	4	0.00	9.2E-01	included
PPIA	97441	ES	5.1:6	4	7	0.00	9.2E-01	included
TPD52L1	77411	ES	8	6	9.1	0.00	9.2E-01	included
GPHN	28023	ES	13:14	12	15	0.00	9.2E-01	excluded
TAF1D	18317	ES	12.3	12.1	12.5	0.00	9.2E-01	excluded
CORO1A	36091	ES	2	1	3	0.00	9.2E-01	excluded
ATG9A	57645	ES	3.1:3.2	1	4	0.00	9.2E-01	excluded
SF1	16684	ES	4	3	5	0.00	9.2E-01	included
LCN2	87708	ES	4	3	6.1	0.00	9.2E-01	included
ATPAF2	39537	ES	3	2	4	0.00	9.2E-01	excluded
ACPL2	67066	ES	3:04	1	5	0.00	9.2E-01	excluded
LIMCH1	69120	ES	27:28:29:30.2	26	31	0.00	9.2E-01	excluded
WBP11	20560	ES	6:07	5	8	0.00	9.2E-01	included
ATP5A1	300060	ES	4.2:4.3	3.2	6.1	0.00	9.2E-01	excluded
ADAM15	7910	ES	20:21.1:21.2:22.1:22.2	19	23	0.01	9.2E-01	included
LRBA	70815	ES	40	39	41	0.00	9.2E-01	included
SEC23A	27351	ES	5	4.1	6	0.00	9.2E-01	included
RNF168	68246	ES	2	1	3	0.00	9.2E-01	excluded
GYS1	50844	ES	3	2	4	0.00	9.2E-01	excluded
UBE3A	29722	ES	4.1:4.2:5.1:5.2	3	6.2	0.00	9.2E-01	included
UBA52	48479	ES	1.3:2	1.2	4.2	0.00	9.2E-01	excluded
ZFAND5	86598	ES	03:04.1	1	4.2	0.00	9.2E-01	included
SLC26A11	44040	ES	2.1:2.2	1	3.2	0.00	9.2E-01	excluded
DMKN	101871	ES	8:09	6.4	12	0.00	9.2E-01	included
CCDC25	83178	ES	8.1	6	9	0.00	9.2E-01	included
RNF43	42673	ES	3	1.4	4	0.00	9.2E-01	included
AP3S2	32455	ES	5	3	6	0.00	9.2E-01	included
ZNF302	48992	ES	3	2	4	0.00	9.2E-01	included
PNO1	53829	ES	3	2	4	0.00	9.2E-01	excluded
MAPK8	11477	ES	08:11.1	6	11.2	0.00	9.2E-01	included
SMPD4	55297	ES	14	10	15.1	0.00	9.2E-01	excluded
TOM1L1	42558	ES	3.3:4	2	6.1	0.00	9.2E-01	included
MBNL2	26143	ES	9	8	11	0.00	9.2E-01	excluded
PACRGL	68911	ES	6:07:08	5	9	0.00	9.2E-01	excluded
CENPT	37142	ES	6.3	5	7.2	0.00	9.2E-01	included
KIAA1217	11008	ES	16:17:18	15	19	0.00	9.2E-01	included
NEK3	25995	ES	4	3.2	5	0.00	9.2E-01	excluded
GAPVD1	87565	ES	20	19.1	21.1	0.00	9.2E-01	excluded
TBC1D3	40567	ES	17:18.1	16	19	0.00	9.2E-01	excluded

TMEM123	18452	ES	4	1	5	0.00	9.2E-01	included
ERG	60596	ES	8:09	7	11.1	0.00	9.2E-01	excluded
CCDC90B	18067	ES	03:04.1	2	4.2	0.00	9.2E-01	included
NME6	64603	ES	1.2:3.2	1.1	4	0.00	9.2E-01	included
PFDN5	21991	ES	3	2	5	0.00	9.2E-01	excluded
ENOSF1	44465	ES	14	13	15	0.00	9.2E-01	included
PPP4R1	44609	ES	5:06	4.2	7	0.00	9.2E-01	included
VPS8	67973	ES	22:23	21	24	0.00	9.2E-01	excluded
TRAPPC4	19066	ES	2.3:3.1	2.2	3.2	0.00	9.2E-01	included
MCRS1	21589	ES	2:03:05	1	6	0.00	9.2E-01	excluded
HAX1	7822	ES	2.1:2.2:2.3	1	3.2	0.00	9.2E-01	included
PPP2R1B	18679	ES	3	2	5	0.00	9.2E-01	excluded
TRA2B	68037	ES	2:03	1	4	0.00	9.2E-01	excluded
RHCE	1185	ES	7:08:09	6	10	0.00	9.2E-01	included
KDM4C	85831	ES	20	19	22	0.00	9.2E-01	included
PPOX	8570	ES	9	8	10	0.00	9.2E-01	included
ZNF512	53027	ES	3	1	4.1	0.00	9.2E-01	included
ACBD5	11068	ES	8	7	9	0.00	9.2E-01	excluded
BSCL2	16407	ES	10	9	11	0.00	9.2E-01	included
HABP4	86970	ES	3:04:05	2	6	0.00	9.2E-01	excluded
ADAM15	7924	ES	5.2:6:7:8:9:10:11.1:11 .2:11.3:12:13.1	5.1	13.2	0.00	9.2E-01	excluded
ZNF415	51676	ES	7.1:7.2:8.2	6.1	9	0.00	9.2E-01	included
ABCA10	43179	ES	13.1:13.2	12	14	0.00	9.2E-01	included
OS9	22708	ES	5.1:5.2:5.3:7.1:7.3:7.4:	4	9.2	0.00	9.2E-01	included
REEP5	72992	ES	4:05	3	6	0.00	9.2E-01	included
ESRP1	97999	ES	15	13	16	0.00	9.2E-01	excluded
COMMD9	15422	ES	2	1	3	0.00	9.2E-01	excluded
ALG2	87057	ES	2	1	3	0.00	9.2E-01	excluded
ERG	60595	ES	8:09:10	7	11.1	0.00	9.2E-01	included
EPB41L2	77564	ES	20.1:20.2:21	14	22	0.00	9.3E-01	excluded
MMS19	12719	ES	12:13	11	14	0.00	9.3E-01	excluded
CLNS1A	17962	ES	3:04	2	5	0.00	9.3E-01	excluded
LTBP4	49932	ES	29	28	31	0.00	9.3E-01	excluded
WDR48	64128	ES	3.2	2	4.1	0.00	9.3E-01	included
PPA2	70202	ES	5	4	7	0.00	9.3E-01	excluded
MT1G	36490	ES	2.2	1	2.4	0.00	9.3E-01	excluded
WDR20	29347	ES	5	4	7.1	0.00	9.3E-01	excluded
IGSF1	90117	ES	11	10	12	0.00	9.3E-01	included
ZNF263	33514	ES	04:05.1	2	6	0.00	9.3E-01	excluded
THAP6	69533	ES	4.1	3	5	0.00	9.3E-01	excluded
TIMMDC1	66323	ES	2	1.1	3	0.00	9.3E-01	included
ALPL	976	ES	10	9.2	12	0.00	9.3E-01	included
ADAM15	7915	ES	20:21.2:22.1	19	23	0.00	9.3E-01	excluded
LRRC28	32660	ES	5:6:7.1:7.2:8:10	3	12	0.00	9.3E-01	excluded
LRP8	3064	ES	6:07	5	9	0.00	9.3E-01	excluded
OSBPL5	13956	ES	5.2:6.1:6.2:7:8:9:10.1	5.1	10.2	0.00	9.3E-01	excluded
IRF3	50999	ES	1.5:2	1.4	3	0.00	9.3E-01	excluded
LRRC28	32709	ES	5:06	2	7.1	0.00	9.3E-01	excluded
BBS4	31567	ES	4	2	5	0.00	9.3E-01	excluded
SEC14L2	61741	ES	4	3	5.1	0.00	9.3E-01	included
NASP	2708	ES	7.2	6	8	0.00	9.3E-01	included
BAX	50840	ES	2	1	3	0.00	9.3E-01	included
RPS3	17840	ES	4.3:5	4.2	6.1	0.00	9.3E-01	excluded
SDR39U1	27010	ES	5	4.1	7	0.00	9.3E-01	included
LGALS8	10383	ES	8:09	7.3	10.1	0.00	9.3E-01	included
TATDN1	85084	ES	5:06	4.1	7	0.00	9.3E-01	included
MTHFSD	37927	ES	4.1:5:6:7	2.2	8	0.00	9.3E-01	included
HYAL1	64994	ES	5	4.5	6	0.00	9.3E-01	excluded
ADAM15	7921	ES	20	19	23	0.00	9.3E-01	excluded
METTL7A	21720	ES	3.2:3.4	3.1	3.5	0.00	9.3E-01	included
ZFPL1	16753	ES	3.1	2.1	4	0.00	9.3E-01	excluded

GPR137B	10368	ES	2:03	1	4.1	0.00	9.3E-01	included
ZNF148	66552	ES	3.2:4:5:6:7	3.1	8	0.00	9.3E-01	excluded
MAPRE2	45139	ES	5	4.1	6	0.00	9.3E-01	included
TLE2	46646	ES	10.5:11:12:13:14:15:1<br>6.2:17:18:19:20:21:22	10.4	23.2	0.00	9.3E-01	excluded
CARF	56970	ES	14	13	15	0.00	9.3E-01	excluded
MTHFD2L	69500	ES	7	6	8	0.00	9.3E-01	included
M1AP	54123	ES	9	8	10	0.00	9.3E-01	included
EIF3K	49681	ES	05:07.1	4	8	0.00	9.3E-01	excluded
RBM6	64944	ES	3.1:3.2:4:5:7	2	8	0.00	9.3E-01	included
FAM172A	72784	ES	11	10	12	0.00	9.3E-01	included
WBSCR22	80008	ES	5	4	6	0.00	9.3E-01	included
UBE2W	84195	ES	2	1	3	0.00	9.3E-01	excluded
WSB1	39836	ES	3	2.2	4	0.00	9.3E-01	included
PHB2	20052	ES	3.1:3.2:4.1:4.2:5.1:6.2	2	7.1	0.00	9.3E-01	excluded
SETD5	63101	ES	2	1	3	0.00	9.3E-01	included
NFIB	85891	ES	12.1:12.2:13	11	15	0.00	9.3E-01	included
HNRNPAB	74847	ES	5.2:5.3:6	5.1	7	0.00	9.3E-01	included
PSMC5	43008	ES	3	2.3	4	0.00	9.3E-01	excluded
C11orf80	17128	ES	5.1	4	6	0.00	9.3E-01	included
DHRS4L2	26803	ES	4:05:06	3	7	0.00	9.3E-01	excluded
COMMD4	31852	ES	4	3	5.2	0.00	9.3E-01	excluded
LMO7	26063	ES	30:31:00	29	32	0.00	9.3E-01	included
ZNF2	54491	ES	4	3	5.2	0.00	9.3E-01	included
NBPF10	4446	ES	85:86:87:88	84	89	0.00	9.3E-01	included
ECT2	67659	ES	2	1	3.2	0.00	9.3E-01	excluded
VIPAS39	28604	ES	1.3	1.1	2	0.00	9.3E-01	included
ALKBH8	18573	ES	10.2:11:12:13:14:15:1	10.1	16.2	0.00	9.3E-01	excluded
C14orf2	29527	ES	6	5	7.1	0.00	9.3E-01	excluded
DHDDS	1276	ES	6.1	5	8	0.00	9.3E-01	included
ZNF544	52427	ES	11	7.2	12	0.00	9.3E-01	excluded
DDO	77229	ES	4	3	5	0.00	9.3E-01	included
OPTN	10781	ES	4	3	5.2	0.00	9.3E-01	included
KLHDC4	37953	ES	9	8	10	0.00	9.3E-01	included
PYGL	27498	ES	2	1	3	0.00	9.3E-01	included
TSFM	22761	ES	3:04	2	5	0.00	9.3E-01	excluded
COG5	81339	ES	16	15	17	0.00	9.3E-01	excluded
ITGA6	55969	ES	7	6	8	0.00	9.3E-01	included
TMEM184B	62226	ES	4.1:4.2	3	5	0.00	9.3E-01	excluded
IQCB1	66405	ES	8:09:10	7	11	0.00	9.3E-01	excluded
PLD3	49897	ES	3	1.1	5.2	0.00	9.3E-01	included
ATG10	72684	ES	2	1.1	3.2	0.00	9.3E-01	excluded
SLC5A6	52951	ES	8	7	9	0.00	9.3E-01	excluded
TPM3	7795	ES	5.3:6.1	5.2	6.2	0.00	9.3E-01	excluded
DUS4L	81346	ES	3	2	4	0.00	9.3E-01	excluded
BSDC1	1605	ES	3:4.1:4.2:4.3	2	5	0.00	9.3E-01	included
NDUFC2	18005	ES	2.2:2.3:3.1	2.1	3.2	0.00	9.3E-01	included
RSPH3	78294	ES	3:04	2	5	0.00	9.3E-01	excluded
GTF2H2C	72399	ES	2	1.1	3	0.00	9.3E-01	excluded
FSTL1	66370	ES	3	2	4	0.00	9.3E-01	excluded
RER1	261	ES	4	3	5	0.00	9.3E-01	included
IL17RC	63266	ES	5.1:5.2:5.3	3.3	6	0.00	9.3E-01	excluded
ARHGAP17	35665	ES	10	9	11	0.00	9.3E-01	excluded
UXS1	54858	ES	2:3.1:3.2:4:5:6:8	1	9	0.00	9.3E-01	excluded
TCF7L2	13148	ES	15	14	17	0.00	9.3E-01	excluded
ABCC1	34210	ES	31	30	32	0.00	9.3E-01	included
TULP3	19739	ES	4	3	5	0.00	9.3E-01	excluded
HACL1	63591	ES	5	4	6	0.00	9.3E-01	included
CS	22419	ES	4.2:5.1	4.1	5.2	0.00	9.3E-01	included
PI4KB	7598	ES	4	1	6	0.00	9.3E-01	included
KLHL8	69859	ES	4.1:4.2	3	5	0.00	9.3E-01	excluded
WAPAL	12416	ES	15	14	16	0.00	9.3E-01	excluded

SKA2	42746	ES	1.2:2:3:4.1:4.2	1.1	5	0.00	9.3E-01	included
MYO5C	30647	ES	36	35	37	0.00	9.3E-01	included
JAZF1	79072	ES	3	2	5.1	0.00	9.3E-01	excluded
RAMP2	41122	ES	4.1:4.2	3	5	0.00	9.3E-01	excluded
RPS3A	70826	ES	2.1:2.2:3.1	1.3	4.1	0.00	9.4E-01	excluded
PSME2	26866	ES	2	1	3.2	0.00	9.4E-01	excluded
PPP2R3A	66897	ES	2	1	4	0.00	9.4E-01	included
EFS	26738	ES	2	1	3	0.00	9.4E-01	excluded
SUMO1	56943	ES	2	1	4	0.00	9.4E-01	included
EFCAB13	42072	ES	9:10	8	11	0.00	9.4E-01	included
CORO1B	387271	ES	4:5.1:5.3:5.5:5.6:5.7:6	3	7	0.00	9.4E-01	excluded
PLBD2	24643	ES	8	7	9	0.00	9.4E-01	excluded
EGFL7	88190	ES	7	6	8	0.00	9.4E-01	included
PLEKHA5	20658	ES	19:20	15	21	0.00	9.4E-01	excluded
NFATC1	46242	ES	10.1:10.2	9.1	11	0.00	9.4E-01	excluded
RIN2	58807	ES	6:07:08	5	9	0.00	9.4E-01	excluded
MAEA	68486	ES	2	1	5.1	0.00	9.4E-01	excluded
FAM49B	85156	ES	5:06:09	4	10	0.00	9.4E-01	excluded
RABEP2	35893	ES	7	6	8	0.00	9.4E-01	excluded
VRK3	51148	ES	8:09	7	10	0.00	9.4E-01	included
NSFL1C	58508	ES	2:03	1	4	0.00	9.4E-01	excluded
SNX1	139190	ES	4.1:4.2:5:6:8:9:10.2:1 1:12:14:15	3	16.1	0.00	9.4E-01	included
C11orf80	17122	ES	13	12	14	0.00	9.4E-01	excluded
TARS2	7481	ES	3.2:4.1	3.1	4.2	0.00	9.4E-01	included
GAPVD1	87567	ES	17	15	18	0.00	9.4E-01	excluded
ALKBH3	15466	ES	8	6	9	0.00	9.4E-01	excluded
TTC39B	85908	ES	5:07	2	8	0.00	9.4E-01	included
R3HCC1L	12756	ES	5:06	4	8	0.00	9.4E-01	included
PI4KB	7592	ES	3	2	4	0.00	9.4E-01	included
MRRF	87471	ES	6	4	7	0.00	9.4E-01	included
RBM15	4103	ES	2	1.1	3	0.00	9.4E-01	included
VTI1B	28083	ES	2:03	1	4	0.00	9.4E-01	excluded
DCTD	71251	ES	1.2:2.1	1.1	5	0.00	9.4E-01	excluded
TBC1D15	23421	ES	7	6	8	0.00	9.4E-01	included
TCEB1	84207	ES	6	1.2	7	0.00	9.4E-01	excluded
MCUR1	75402	ES	3	2	4	0.00	9.4E-01	excluded
C14orf28	27387	ES	3	2	4	0.00	9.4E-01	excluded
RNF181	54295	ES	3.2	2	4.1	0.00	9.4E-01	excluded
FER	72934	ES	5:06	4.2	7	0.00	9.4E-01	included
DDX5	43066	ES	3	1	4.1	0.00	9.4E-01	included
COPS3	39471	ES	05:06.1	4	6.2	0.00	9.4E-01	excluded
KLC4	76227	ES	7.2	6	8	0.00	9.4E-01	excluded
FAM86B1	82716	ES	1.2:3.1	1.1	3.2	0.00	9.4E-01	included
RPL34	70303	ES	1.2:2	1.1	3	0.00	9.4E-01	included
WIZ	48093	ES	3	2	4	0.00	9.4E-01	included
NLN	72250	ES	2:03	1	4	0.00	9.4E-01	excluded
PUM1	1450	ES	7:08	4	9	0.00	9.4E-01	excluded
CHID1	13815	ES	6.2:6.3	5.2	7	0.00	9.4E-01	excluded
UBE2D4	79379	ES	04:05.1	3	7.1	0.00	9.4E-01	included
SRP14	29941	ES	2.2	1	3	0.00	9.4E-01	included
NUP62	51130	ES	1.2:1.4:1.5	1.1	2.1	0.00	9.4E-01	included
MAP4	64556	ES	19	18	20	0.00	9.4E-01	excluded
VOPP1	79759	ES	3.1	1	9	0.00	9.4E-01	included
GTF2H2	72439	ES	2	1	3	0.00	9.4E-01	included
ACADM	3493	ES	5	4.2	6	0.00	9.4E-01	excluded
PDPR	37331	ES	18	17	19	0.00	9.4E-01	excluded
SLC30A6	53165	ES	2	1	3	0.00	9.4E-01	excluded
NSUN5	270162	ES	2:3.1:3.2	1	5	0.00	9.4E-01	excluded
SEC24C	12227	ES	2	1	3	0.00	9.4E-01	included
HELQ	69791	ES	6	5	7	0.00	9.4E-01	included
DMKN	101866	ES	7	6.4	8	0.00	9.4E-01	included

MYH10	39199	ES	6.1	5	7	0.00	9.4E-01	included
PTP4A3	85339	ES	04:05.1	2	5.2	0.00	9.4E-01	included
ARPC2	57447	ES	3	2	4	0.00	9.4E-01	excluded
MTMR12	71645	ES	16	15	17	0.00	9.4E-01	excluded
FAM134C	41092	ES	5:06:07	2.1	8	0.00	9.4E-01	excluded
CFLAR	56798	ES	11:12	8.1	13	0.00	9.4E-01	excluded
R3HDM4	46357	ES	02:03.1	1	3.3	0.00	9.4E-01	excluded
PRKCDBP	14097	ES	2	1	3	0.00	9.4E-01	excluded
XRN1	67112	ES	40	39	41	0.00	9.4E-01	excluded
ZFYVE27	12737	ES	9	8.2	10	0.00	9.4E-01	excluded
PEX7	77922	ES	9	8	10	0.00	9.4E-01	included
POLH	76310	ES	2	1	3	0.00	9.4E-01	excluded
RBM42	49233	ES	3.2:4:6.1:6.2:6.3:7:8:9	3.1	9.2	0.00	9.4E-01	included
PSMA6	27226	ES	8.2	7	9	0.00	9.4E-01	included
SMOX	58621	ES	8	7	9	0.00	9.4E-01	excluded
PI4KB	7596	ES	2:03:04	1	6	0.00	9.4E-01	excluded
CNTN4	62959	ES	12	11	13	0.00	9.4E-01	excluded
APOL1	62038	ES	3.2:4:5	1	7	0.00	9.4E-01	included
PIGT	59569	ES	2.1:4:5.2:6	1	7	0.00	9.4E-01	included
CHORDC1	18273	ES	4	2	5.1	0.00	9.4E-01	excluded
KIFC3	36612	ES	9:10:11	8	13	0.00	9.4E-01	excluded
CHMP3	54440	ES	6.2:7.1	6.1	7.2	0.00	9.4E-01	included
IST1	37522	ES	6:07:08	5	9	0.00	9.4E-01	excluded
EIF3M	14850	ES	4	3	5	0.00	9.4E-01	excluded
WWP2	37319	ES	3	2.2	4.3	0.00	9.4E-01	included
FXYD6	18943	ES	4:05	2	6	0.00	9.4E-01	included
ZNF791	47807	ES	4	3	5	0.00	9.4E-01	excluded
RTN3	16524	ES	5:06:07	4	8	0.00	9.4E-01	excluded
CARF	56971	ES	10.1	9	11	0.00	9.4E-01	included
AP1B1	61605	ES	17	16	18	0.00	9.4E-01	included
NOP9	26956	ES	8.2:9.1	8.1	9.2	0.00	9.4E-01	included
ASB1	58226	ES	3	2	4	0.00	9.4E-01	included
PIAS1	31349	ES	7	6	8	0.00	9.5E-01	included
IL11RA	86214	ES	5	4	6	0.00	9.5E-01	excluded
ARHGEF11	8339	ES	8	7	9	0.00	9.5E-01	excluded
SART3	24217	ES	10	9	11	0.00	9.5E-01	excluded
HAGHL	32975	ES	5.1:5.3	4	6.1	0.00	9.5E-01	included
KIDINS220	52606	ES	3	2	4	0.00	9.5E-01	excluded
WBSCR27	80036	ES	4	3	5	0.00	9.5E-01	excluded
METTL21A	57195	ES	5	4.2	7	0.00	9.5E-01	excluded
SLC4A7	63780	ES	26	25	27	0.00	9.5E-01	included
CSGALNACT1	82880	ES	10	9	11	0.00	9.5E-01	excluded
ZNF544	52428	ES	09:10.1	7.2	10.2	0.00	9.5E-01	excluded
LIMCH1	69127	ES	3	2	4	0.00	9.5E-01	included
SCYL3	8960	ES	12	11	13	0.00	9.5E-01	included
CCT2	23326	ES	14	13	15	0.00	9.5E-01	included
RPAIN	38679	ES	6.1	5	7	0.00	9.5E-01	included
POLR2J3	81119	ES	2	1	4.1	0.00	9.5E-01	included
MLH3	28468	ES	7:08	6	9	0.00	9.5E-01	included
PTGES3	22480	ES	4.1:4.2	3	5	0.00	9.5E-01	included
SUN1	78537	ES	10:11:12	9.1	13	0.00	9.5E-01	excluded
NVL	9951	ES	5	4	6	0.00	9.5E-01	included
PPOX	8571	ES	4:5:6:7	3	8	0.00	9.5E-01	excluded
UBE2I	33060	ES	4.2:4.3:4.4	2	5.2	0.00	9.5E-01	included
SDCCAG3	88162	ES	3	2	4	0.00	9.5E-01	included
NPRL3	32806	ES	3:04:05	2	6	0.00	9.5E-01	excluded
VWF	19816	ES	3	2	4	0.00	9.5E-01	included
HKR1	49504	ES	6	5	12	0.00	9.5E-01	excluded
BCL2L11	54965	ES	5	3.1	7	0.00	9.5E-01	excluded
GABARAPL1	20397	ES	2.7:2.8:2.10:2.12:2.13:	2.6	3	0.00	9.5E-01	included
HDAC8	89465	ES	6.1:8.1:8.2	5	11	0.00	9.5E-01	excluded
G6PC3	41770	ES	2.1	1.1	3	0.00	9.5E-01	excluded

IFNAR2	60395	ES	2.2:3	1	4	0.00	9.5E-01	excluded
HARS2	73747	ES	3	2.2	4	0.00	9.5E-01	excluded
DCAF4	28242	ES	2	1	3	0.00	9.5E-01	included
TIA1	53872	ES	13:14.2	12	15	0.00	9.5E-01	excluded
HMGN1	107415	ES	6.1:6.2:7	5	9	0.00	9.5E-01	included
LIMK2	61838	ES	4:5:6:7:8	2	9	0.00	9.5E-01	excluded
C9orf3	86948	ES	7:08	6	10	0.00	9.5E-01	excluded
PIGT	59551	ES	3	2.2	4	0.00	9.5E-01	excluded
FXR1	67750	ES	2:05	1	6	0.00	9.5E-01	excluded
TRIM16	39365	ES	8:09	7	10	0.00	9.5E-01	excluded
CASP8	56820	ES	9:10:11	8	13	0.00	9.5E-01	excluded
SNED1	58308	ES	30	29	31	0.00	9.5E-01	excluded
TCEA1	83853	ES	4.1:5.1:6:7:8	2	9	0.00	9.5E-01	included
KRI1	47551	ES	1.3	1.1	2	0.00	9.5E-01	excluded
VEGFB	115126	ES	4	3	5	0.00	9.5E-01	included
C5orf45	74986	ES	2.3	2.1	4	0.00	9.5E-01	excluded
DEAF1	13724	ES	7	6	8	0.00	9.5E-01	included
SEZ6L2	35980	ES	2	1	3.1	0.00	9.5E-01	excluded
ATF2	56067	ES	10.1:11	9	12	0.00	9.5E-01	included
MRPL48	17732	ES	2:03	1	4	0.00	9.5E-01	excluded
CBLL1	81372	ES	3	2	4.1	0.00	9.5E-01	excluded
RPS14	74090	ES	6	5	7	0.00	9.5E-01	excluded
FAM76A	1344	ES	3	2	4	0.00	9.5E-01	included
RAD51D	40263	ES	5	4	7	0.00	9.5E-01	included
EIF3M	14855	ES	2.2:3:4	1	5	0.00	9.5E-01	included
C21orf91	60240	ES	4	3	5.1	0.00	9.5E-01	excluded
NBPF20	155354	ES	99:100:101:102:103:1<br>04:105:106:107:108:1	98	111	0.00	9.5E-01	included
FBXO3	14932	ES	6	5	7	0.00	9.5E-01	excluded
TMEM135	18209	ES	4	3	5	0.00	9.5E-01	excluded
CCDC88A	53614	ES	29	28	30	0.00	9.5E-01	excluded
CDK5RAP1	58984	ES	8	7	9	0.00	9.5E-01	excluded
RCN2	31956	ES	4	3	5	0.00	9.5E-01	excluded
NAPG	44631	ES	2:3:4:5	1	6	0.00	9.5E-01	included
DRG2	39557	ES	4:5.1:5.2:6.1:6.2:6.4:7<br>:8.1:8.2:9	3.2	10	0.00	9.5E-01	excluded
ANKZF1	57649	ES	3	2	4	0.00	9.5E-01	excluded
ERCC8	72169	ES	6	5	7	0.00	9.5E-01	included
CNOT7	82788	ES	3.2	2	4	0.00	9.5E-01	included
PPP1R9A	80519	ES	15.1:15.2:16:17:18.1:1	14	19	0.00	9.5E-01	excluded
MED25	51093	ES	3:4:5:6:7	2	8	0.00	9.5E-01	included
NLE1	40282	ES	2.1	1.3	3.1	0.00	9.5E-01	excluded
NICN1	64872	ES	3	2	4	0.00	9.5E-01	included
GORASP2	55919	ES	2.1:2.2	1	4.1	0.00	9.5E-01	excluded
FN1	57362	ES	42	41	43	0.00	9.5E-01	excluded
CNBP	66706	ES	2.2:3.1	2.1	3.2	0.00	9.5E-01	excluded
CARF	56981	ES	5.1:5.2	3.1	6.1	0.00	9.5E-01	included
SLC7A7	26622	ES	7	6	8	0.00	9.5E-01	included
RHOC	4240	ES	1.2:1.3:2.2:2.3:2.4	1.1	3	0.00	9.5E-01	excluded
SMARCB1	61327	ES	4.1:4.2	3	5	0.00	9.5E-01	included
ZC3H14	28729	ES	4	3	5	0.00	9.6E-01	included
MGAT4B	74931	ES	4	3	5	0.00	9.6E-01	excluded
MIA3	9890	ES	4.2:5:6:8.1:10:11.2:12<br>:13:14:15:16:17:18:19<br>:20:21:22:23:24:25:26	4.1	30.2	0.00	9.6E-01	excluded
C17orf62	44389	ES	2	1.1	4.2	0.00	9.6E-01	included
CNOT2	23379	ES	3.2:4	2	6	0.00	9.6E-01	excluded
DGAT2	17877	ES	2	1	3	0.00	9.6E-01	excluded
DYNC1I2	55953	ES	05:07.2	4	7.3	0.00	9.6E-01	included
PSMG4	75187	ES	5.1:5.2:5.3:5.4	4	5.6	0.00	9.6E-01	included
H2AFV	79573	ES	4.1	3	5	0.00	9.6E-01	included
FNIP1	73238	ES	3	2	4	0.00	9.6E-01	included

BIN3	83047	ES	5	4	6	0.00	9.6E-01	included
DAPK1	86773	ES	24	23	25	0.00	9.6E-01	included
MDM2	23155	ES	4	3	5.1	0.00	9.6E-01	included
HIBCH	56560	ES	14	13	15	0.00	9.6E-01	excluded
TTC23	32618	ES	3:04	2.3	6	0.00	9.6E-01	included
GRAMD1A	260995	ES	14	13	15	0.00	9.6E-01	included
TATDN3	9760	ES	06:01.1	4	8	0.00	9.6E-01	excluded
DUS3L	46931	ES	3.3:4.1:4.2	3.2	4.3	0.00	9.6E-01	excluded
SCFD2	69308	ES	7	6	8	0.00	9.6E-01	included
NQO1	37304	ES	4	3	5	0.00	9.6E-01	included
HKR1	49492	ES	11	8.2	12	0.00	9.6E-01	excluded
LARGE	61942	ES	16	15	17	0.00	9.6E-01	included
MTMR1	90355	ES	13	11	14	0.00	9.6E-01	included
FYCO1	64425	ES	15	14	16	0.00	9.6E-01	excluded
DNASE2	47880	ES	4	3	5	0.00	9.6E-01	included
VEZT	23781	ES	5:6.1:6.2:9	4	11	0.00	9.6E-01	excluded
RQCD1	57498	ES	4	3	5	0.00	9.6E-01	included
FGD2	76006	ES	3:04	2.1	5	0.00	9.6E-01	included
POLB	83713	ES	3	2	4	0.00	9.6E-01	excluded
MYO15A	39584	ES	62:63	61	64	0.00	9.6E-01	excluded
TGM2	59376	ES	2	1	3	0.00	9.6E-01	excluded
STK16	57668	ES	3.2:4	2.2	5	0.00	9.6E-01	excluded
PARP9	66444	ES	06:07.1	4.1	7.2	0.00	9.6E-01	included
RABL6	88218	ES	13.2:14.1:14.2:15:16.1:16.2:17:18.1:19.1	13.1	19.2	0.00	9.6E-01	included
DCTD	71252	ES	2.1	1.1	5	0.00	9.6E-01	excluded
ODF2	87756	ES	10:11	9.2	12	0.00	9.6E-01	included
DAPK2	31081	ES	3	2	4.1	0.00	9.6E-01	included
CPSF7	16204	ES	6.1:6.2:6.3	3	7.1	0.00	9.6E-01	included
MDM2	22968	ES	11	10	12.1	0.00	9.6E-01	excluded
RNF216	78682	ES	3:4:5.2:6.2	2	7	0.00	9.6E-01	excluded
HIBADH	79057	ES	2	1	3	0.00	9.6E-01	included
SPG11	30348	ES	31:32.1	30	33	0.00	9.6E-01	excluded
PFKFB3	10697	ES	18.1:19	17	20	0.00	9.6E-01	included
PRDM5	70473	ES	15	14	16	0.00	9.6E-01	excluded
SENP7	65948	ES	5	4	6	0.00	9.6E-01	included
CLIC6	60498	ES	2	1	3	0.00	9.6E-01	included
VPS45	7421	ES	16	15	17	0.00	9.6E-01	excluded
PSMA3	27692	ES	3.2	2	4	0.00	9.6E-01	included
TIMM10	15856	ES	1.2:2.1	1.1	2.2	0.00	9.6E-01	excluded
SLC25A29	29266	ES	2:3.4:3.5	1	3.7	0.00	9.6E-01	excluded
TMBIM4	22901	ES	4.1:4.2:5	1	6	0.00	9.6E-01	excluded
WBSCR22	80005	ES	5:06	4	7	0.00	9.6E-01	included
TAF15	40342	ES	2:03	1.1	4	0.00	9.6E-01	included
CBWD1	85690	ES	11	10	12	0.00	9.6E-01	included
ATP6V1C1	84782	ES	2	1.2	3	0.00	9.6E-01	excluded
IYD	78146	ES	6.1	5	7	0.00	9.6E-01	excluded
RPS20	83892	ES	1.2:1.4	1.1	2.1	0.00	9.6E-01	included
DMKN	49173	ES	8:09	7	11	0.00	9.6E-01	excluded
C1QTNF1	43989	ES	4.2	2.2	6.2	0.00	9.6E-01	excluded
U2SURP	67125	ES	13	12	14	0.00	9.6E-01	excluded
SNF8	42266	ES	3:5:6:7.1	2	9	0.00	9.6E-01	included
PPP2R4	87847	ES	5	4	6	0.00	9.6E-01	excluded
ILVBL	48060	ES	3	1	4	0.00	9.6E-01	included
ZMYM2	25417	ES	3	2	4.1	0.00	9.6E-01	included
PPWD1	72230	ES	2	1	3	0.00	9.6E-01	excluded
C14orf159	28858	ES	6	5.2	7	0.00	9.6E-01	included
HERC4	11917	ES	9	8	10	0.00	9.6E-01	excluded
ZNF131	71931	ES	6	5.2	7.1	0.00	9.6E-01	excluded
GZMH	27015	ES	4	3	5	0.00	9.6E-01	included
RPS6KA1	1289	ES	8	4	9	0.00	9.6E-01	excluded
RAB1B	16997	ES	4	3	5	0.00	9.6E-01	excluded

POC1A	65180	ES	10	9	11	0.00	9.6E-01	excluded
GLB1	63836	ES	4.2:5:6	1	7	0.00	9.6E-01	included
LMF1	33034	ES	6:07	5.1	8.1	0.00	9.6E-01	included
NBPF10	4448	ES	84:85:86:87	83	88	0.00	9.6E-01	excluded
ARL16	44150	ES	3:04	2.3	5	0.00	9.6E-01	excluded
CHD1L	7379	ES	2:3:4:5:6:7	1	8	0.00	9.6E-01	excluded
PCYT1A	68235	ES	2	1	3	0.00	9.6E-01	excluded
WDR53	68248	ES	2.2:3	2.1	4	0.00	9.6E-01	included
DCAF4	28236	ES	8:9:10.2:11.1:11.2	7.2	12	0.00	9.6E-01	included
MTO1	76747	ES	10	9	11	0.00	9.6E-01	excluded
NFIC	46679	ES	10:11	9	12	0.00	9.6E-01	excluded
OGDHL	11524	ES	3	2	4	0.00	9.6E-01	excluded
BACE1	18911	ES	4.2:5.1	4.1	5.2	0.00	9.6E-01	included
RABL2B	62917	ES	9	7.1	10.1	0.00	9.6E-01	included
POR	80143	ES	6.1:6.2	5.1	7	0.00	9.6E-01	excluded
SRSF7	53285	ES	4.4	4.1	4.6	0.00	9.7E-01	excluded
RFC2	80073	ES	6	5	7	0.00	9.7E-01	included
CRYZL1	60458	ES	12.2:13:14:16.2	11	17	0.00	9.7E-01	excluded
STK24	26169	ES	4	3	5	0.00	9.7E-01	excluded
NXF1	16437	ES	3.1:3.2:4	2	5	0.00	9.7E-01	excluded
U2AF1L4	49279	ES	2.2	1	3.1	0.00	9.7E-01	included
ELP2	45221	ES	5:6:7:8.1	4.2	9	0.00	9.7E-01	included
APOL1	62021	ES	6.1:6.2	5	7	0.00	9.7E-01	excluded
SURF4	88038	ES	5.2:7.1	5.1	7.2	0.00	9.7E-01	included
DLD	81384	ES	4	3	5	0.00	9.7E-01	included
DCN	23661	ES	7:08	3	9	0.00	9.7E-01	excluded
MON2	22838	ES	38	37.2	39	0.00	9.7E-01	included
FBXL12	47421	ES	3.1:3.2	2.4	5	0.00	9.7E-01	included
MRPS25	63546	ES	3.1:3.2	2	4.1	0.00	9.7E-01	excluded
MCCC1	67789	ES	4:05:07	2	8	0.00	9.7E-01	included
FAM76A	1343	ES	5	4	6	0.00	9.7E-01	included
ZNF28	51627	ES	05:06.2	3	7.1	0.00	9.7E-01	included
ADAT1	37633	ES	5.2	4	6	0.00	9.7E-01	included
ZNF274	52416	ES	4	3	5	0.00	9.7E-01	excluded
STXBP1	87640	ES	19	18	20	0.00	9.7E-01	included
KLC2	16994	ES	7:8:9:10:11:12:13:14:<br>15:16.1:16.3	6	16.4	0.00	9.7E-01	included
CACTIN	46714	ES	10.3:10.5:10.6:10.7:10	10.2	10.9	0.00	9.7E-01	excluded
FBXO32	85067	ES	5:06	4	7	0.00	9.7E-01	included
SCMH1	2048	ES	10:11	9	12	0.00	9.7E-01	excluded
ZMYND8	59715	ES	19.1:19.2	18	20	0.00	9.7E-01	excluded
DDX19B	37348	ES	7	6	9	0.00	9.7E-01	excluded
LMF1	33040	ES	3.1:5.1:8.1:8.2	2	9	0.00	9.7E-01	included
SNURF	29713	ES	5	3.1	6	0.00	9.7E-01	included
IRF3	50985	ES	5.2:6.2	5.1	7	0.00	9.7E-01	excluded
PSMA3	27694	ES	2	1	3.1	0.00	9.7E-01	excluded
COMMD4	31851	ES	04:05.1	3	5.2	0.00	9.7E-01	excluded
UPP1	79640	ES	06:01.1	4	9	0.00	9.7E-01	excluded
PDCD10	67555	ES	5	4.2	6	0.00	9.7E-01	included
MEGF9	87389	ES	2:03	1	4	0.00	9.7E-01	included
TECPR1	80583	ES	2.2:3	2.1	4	0.00	9.7E-01	included
RABL2A	55065	ES	02:03.1	1	3.2	0.00	9.7E-01	excluded
UBR4	885	ES	60	59	61	0.00	9.7E-01	excluded
MOB4	56700	ES	5.1:5.2	4	6	0.00	9.7E-01	excluded
APBB1	14119	ES	10:11	9	12	0.00	9.7E-01	included
RPL35	87539	ES	4.1:4.2	3	5	0.00	9.7E-01	excluded
ST3GAL3	2297	ES	7.1:7.2:8	6	9	0.00	9.7E-01	excluded
SLC9A1	1314	ES	2	1	3	0.00	9.7E-01	excluded
ATL2	53250	ES	5	2	6	0.00	9.7E-01	excluded
LTBP4	107680	ES	16	15	17	0.00	9.7E-01	excluded
GOLT1B	20707	ES	2	1	5	0.00	9.7E-01	excluded
FN1	57369	ES	40.2:40.4:41	39	42	0.00	9.7E-01	excluded

ST7L	4214	ES	6	5	7	0.00	9.7E-01	excluded
ABHD14B	65145	ES	3.3	2.2	6.1	0.00	9.7E-01	included
PGLS	48324	ES	3	2	4	0.00	9.7E-01	included
TIMM17B	89020	ES	4.2:4.3	3	5	0.00	9.7E-01	included
SCPEP1	42601	ES	3.2:4:5	3.1	6	0.00	9.7E-01	excluded
C9orf41	86623	ES	4	3	5	0.00	9.7E-01	excluded
TDRD3	26023	ES	8	7	9	0.00	9.7E-01	excluded
ADAM15	7919	ES	20:21.2	19	23	0.00	9.7E-01	excluded
ATXN3	28978	ES	2:3.1:3.2:4.2	1	5	0.00	9.7E-01	included
LDB2	68846	ES	10	9.1	11.2	0.00	9.7E-01	excluded
ACY1	65150	ES	13:14	12	15	0.00	9.7E-01	excluded
ATP1B3	67081	ES	7	6	8	0.00	9.7E-01	included
RHOT1	40182	ES	19.2:19.3:20:21	19.1	22	0.00	9.7E-01	excluded
RFESD	72813	ES	6	5	7	0.00	9.7E-01	excluded
FAM92A1	84528	ES	3	2	4	0.00	9.7E-01	included
ATP6V0A1	41049	ES	5	4	6.2	0.00	9.7E-01	excluded
PTK2	85304	ES	38:39.4	37	39.5	0.00	9.7E-01	included
ST7	81569	ES	5	1	6	0.00	9.7E-01	included
TTC26	81950	ES	6.2:7	5	8.1	0.00	9.7E-01	excluded
UEVLD	14676	ES	4	3	5	0.00	9.7E-01	excluded
HDAC10	62799	ES	9:10	8	11	0.00	9.7E-01	excluded
MRRF	87472	ES	3	2.3	4	0.00	9.7E-01	excluded
OSTC	70306	ES	3:04	2	5	0.00	9.7E-01	included
DPP8	31169	ES	18:19	17	20	0.00	9.7E-01	excluded
ZSWIM7	39409	ES	2	1.1	4	0.00	9.7E-01	included
CNEP1R1	36363	ES	4	3	5	0.00	9.7E-01	excluded
AFMID	43802	ES	8:9:10:11.1:11.2	6	12	0.00	9.7E-01	included
RAB28	68792	ES	8	6	9	0.00	9.7E-01	excluded
TAF1B	52642	ES	3	2	4	0.00	9.7E-01	excluded
PSEN2	10034	ES	4	3	5	0.00	9.7E-01	excluded
PRMT5	26662	ES	03:04.1	2	4.2	0.00	9.7E-01	excluded
HELLS	12589	ES	13:14	11.2	16	0.00	9.7E-01	included
FAM73A	3536	ES	2	1	3	0.00	9.8E-01	included
MAPKBP1	30108	ES	11	10	12	0.00	9.8E-01	included
FAM92A1	84525	ES	10	9.3	12.1	0.00	9.8E-01	excluded
EZH2	82154	ES	16	15	17	0.00	9.8E-01	excluded
FOPNL	34192	ES	3.1:3.2:4:5	2	6	0.00	9.8E-01	included
ZNF438	11137	ES	6	4	7	0.00	9.8E-01	included
MRPL39	60252	ES	10	9	11	0.00	9.8E-01	included
IP6K2	64775	ES	9	7	11.1	0.00	9.8E-01	excluded
GNG5	3605	ES	2	1.3	3	0.00	9.8E-01	included
SEC24D	70447	ES	14	13	15	0.00	9.8E-01	included
STAG3	97625	ES	7:8:9:10	4	11	0.00	9.8E-01	included
ACTR2	53800	ES	3	2	5	0.00	9.8E-01	excluded
ZNF821	37499	ES	10	9.2	11	0.00	9.8E-01	included
SLC39A6	45209	ES	1.2:2	1.1	3	0.00	9.8E-01	included
VGLL4	63400	ES	5	3	8	0.00	9.8E-01	excluded
HSD17B10	89223	ES	5.1:5.2	4	6	0.00	9.8E-01	included
DMKN	49192	ES	7	6.4	13	0.00	9.8E-01	excluded
ZNF606	52382	ES	4.1	3.1	5	0.00	9.8E-01	excluded
PTP4A2	1531	ES	4:05	3	6	0.00	9.8E-01	excluded
IFT20	39885	ES	2.2	1	3	0.00	9.8E-01	included
ARHGAP4	90526	ES	7	6.2	8	0.00	9.8E-01	included
CCT7	53960	ES	04:05.1	3	5.2	0.00	9.8E-01	included
SCRN2	42123	ES	03:04.1	2.2	4.2	0.00	9.8E-01	included
RUVBL2	50866	ES	1.3:1.4:1.6	1.2	2	0.00	9.8E-01	included
ITSN1	60470	ES	27	26	28	0.00	9.8E-01	included
MAX	27956	ES	5.3:5.5	5.1	5.8	0.00	9.8E-01	included
ADAM15	7918	ES	20:21.1:21.2	19	23	0.00	9.8E-01	excluded
LARGE	61943	ES	13	12	14	0.00	9.8E-01	excluded
MEF2A	32719	ES	3	2	4.2	0.00	9.8E-01	excluded
RASSF4	11353	ES	4	3	6	0.00	9.8E-01	included

RFC2	80076	ES	2	1	3	0.00	9.8E-01	excluded
MRPL55	10141	ES	1.2:2.4:2.5:2.6:2.8	1.1	2.9	0.00	9.8E-01	included
NDRG2	26512	ES	4.5	4.1	5.2	0.00	9.8E-01	excluded
PSMD5	87398	ES	2	1	3	0.00	9.8E-01	excluded
EPS15L1	48167	ES	4.1:4.2:5:6.2:7.2	3	7.3	0.00	9.8E-01	included
PDPR	37334	ES	2.1:2.2:3.2	1	4	0.00	9.8E-01	included
RRP8	14159	ES	2.2:3.1:3.2:3.3	1	4	0.00	9.8E-01	excluded
DCTN1	54048	ES	07:02.2	5	9	0.00	9.8E-01	excluded
ZNF92	79848	ES	2:03	1	4	0.00	9.8E-01	included
XRCC6	62444	ES	3	2.3	4.1	0.00	9.8E-01	excluded
PDLIM5	69979	ES	9	8.2	14	0.00	9.8E-01	included
ZDHHC5	15887	ES	2	1	3	0.00	9.8E-01	excluded
TTC12	18777	ES	22:24.1	21	24.2	0.00	9.8E-01	included
TSEN2	63432	ES	6	5.3	7	0.00	9.8E-01	excluded
CRLS1	58665	ES	5	4	6	0.00	9.8E-01	included
PPP1R12B	9416	ES	9	8	10.1	0.00	9.8E-01	excluded
PRKACB	3590	ES	10	9	11	0.00	9.8E-01	excluded
CPSF6	23311	ES	2.2:4:5:6.1:6.2:8.1:8.2:9:10:11.1	2.1	11.2	0.00	9.8E-01	excluded
POLR1A	54394	ES	2:03	1	4	0.00	9.8E-01	included
KANK2	47638	ES	2.2:2.3	1	3	0.00	9.8E-01	excluded
EDC3	31729	ES	6	5	7	0.00	9.8E-01	included
CD6	16135	ES	13	12	14	0.00	9.8E-01	included
CCNL1	67388	ES	4.1:4.2	3	5	0.00	9.8E-01	included
TRIM55	83997	ES	5:6:7:8	4	11	0.00	9.8E-01	excluded
GUK1	10191	ES	3	2	5.3	0.00	9.8E-01	excluded
RPS11	50953	ES	2.3:3.1	2.2	3.2	0.00	9.8E-01	included
CARD8	50709	ES	12	11	13	0.00	9.8E-01	excluded
NDC1	3068	ES	8	7	9	0.00	9.8E-01	included
TSSC1	52557	ES	4:06	2	7	0.00	9.8E-01	excluded
KIFAP3	8965	ES	3	1	4	0.00	9.8E-01	included
PML	31664	ES	5	4	6.1	0.00	9.8E-01	included
ACPL2	67061	ES	6:09:10	5	11	0.00	9.8E-01	included
ACP1	52515	ES	2	1	3	0.00	9.8E-01	excluded
TM6SF1	32258	ES	5.2:5.3:6.2:7:8	4.1	9	0.00	9.8E-01	included
SEMA4D	86809	ES	17:18:19.1:19.2	16	20.2	0.00	9.8E-01	included
CDK7	72321	ES	5.1:5.2	4	6	0.00	9.8E-01	included
KARS	37640	ES	3:4:5:6:7:8:9:10:11:12:13:14:15.1	2	15.2	0.00	9.8E-01	excluded
ZNF714	48751	ES	6.1:6.2	4.2	7	0.00	9.8E-01	excluded
FAM73B	87820	ES	12:13	11	14	0.00	9.8E-01	excluded
ZSWIM8	12232	ES	21.2:22.1	21.1	22.2	0.00	9.8E-01	excluded
SPATA6L	85756	ES	15	14	16.1	0.00	9.8E-01	excluded
HDAC10	62797	ES	11	10	12	0.00	9.8E-01	excluded
FAM171A2	41856	ES	4.1:4.2	3	5	0.00	9.8E-01	included
ATXN3	28925	ES	7	6	8.1	0.00	9.8E-01	excluded
RHOT1	40177	ES	20:21	19.3	22	0.00	9.8E-01	excluded
DERL2	38711	ES	3:4.1:4.2:4.3	2	6	0.00	9.8E-01	included
ENAH	9989	ES	13	12	14	0.00	9.8E-01	included
SEC16A	88178	ES	24	23.12	26	0.00	9.8E-01	excluded
PARP1	10018	ES	3	2.1	4	0.00	9.8E-01	included
MZT2B	55332	ES	2:03	1	4	0.00	9.8E-01	included
ATG13	15592	ES	2.1:2.2:3	1.1	4.1	0.00	9.8E-01	included
NPIPA8	34237	ES	26:27:00	25	28	0.00	9.8E-01	excluded
CD55	9641	ES	3.1:3.2:3.3	2	4	0.00	9.8E-01	included
MPP7	11095	ES	6	5.2	7	0.00	9.8E-01	excluded
SMC4	67483	ES	2.2:2.3:3:4	2.1	5	0.00	9.8E-01	included
YAP1	18444	ES	5	4	6.1	0.00	9.8E-01	excluded
PGS1	43878	ES	2:3.1:3.2	1	4	0.00	9.8E-01	included
PLEKHB1	17701	ES	4	3.2	5	0.00	9.8E-01	excluded
STAT5A	41032	ES	13	12	14.2	0.00	9.8E-01	excluded
CCDC66	65345	ES	13	12	14	0.00	9.8E-01	included

INSIG1	82434	ES	3:04	2.2	5	0.00	9.9E-01	included
KDM5C	89207	ES	2.1:2.2	1	3	0.00	9.9E-01	included
ALDH16A1	50938	ES	2:03	1	4	0.00	9.9E-01	included
HSD11B1L	46911	ES	2:03	1	4.2	0.00	9.9E-01	included
TXNRD1	24104	ES	7:08	6.2	10	0.00	9.9E-01	included
PLS3	89925	ES	12	11	13	0.00	9.9E-01	excluded
CDC42SE2	73225	ES	2	1	3.1	0.00	9.9E-01	excluded
EEFSEC	66642	ES	6	5	7	0.00	9.9E-01	excluded
DCUN1D4	69273	ES	6	5	7	0.00	9.9E-01	included
NF2	61660	ES	2	1	3	0.00	9.9E-01	included
RBM4	17098	ES	3.2	1	4.1	0.00	9.9E-01	included
DCAF7	42940	ES	2:3:4.1:4.2:5:6	1	7	0.00	9.9E-01	included
XXYLT1	68174	ES	4	3	8	0.00	9.9E-01	included
CCZ1B	78767	ES	7	6	8	0.00	9.9E-01	included
HACL1	63599	ES	3:4:5:6:7:8:9:10	2	11	0.00	9.9E-01	included
TPST2	61519	ES	5	4	6	0.00	9.9E-01	excluded
CALCOCO2	42230	ES	4	2	6	0.00	9.9E-01	excluded
STX5	16445	ES	3	2.2	4	0.00	9.9E-01	included
NSMAF	83944	ES	5	3	6	0.00	9.9E-01	excluded
TDP1	28809	ES	20	19	21	0.00	9.9E-01	excluded
CDKL3	73384	ES	7:08:09	6	10.1	0.00	9.9E-01	included
CPSF3L	124	ES	4:5.2:6.1:6.2	1	7.1	0.00	9.9E-01	excluded
ATP5A1	300061	ES	4.1:4.2	3.2	6.1	0.00	9.9E-01	included
PSMA2	79321	ES	2	1	3	0.00	9.9E-01	excluded
CLN3	35742	ES	5:06	4	7	0.00	9.9E-01	excluded
TMEM66	83268	ES	1.2:2.1:2.2	1.1	2.3	0.00	9.9E-01	excluded
ZNF550	52294	ES	5.1:5.2	4	6	0.00	9.9E-01	excluded
ORAI3	36211	ES	2	1	3.1	0.00	9.9E-01	included
SWAP70	14333	ES	3	2	4	0.00	9.9E-01	included
ADAM15	7911	ES	20:21.2:22.1:22.2	19	23	0.00	9.9E-01	excluded
STXBP2	47124	ES	15	14	16	0.00	9.9E-01	included
SLMAP	65408	ES	12:14	11	15.2	0.00	9.9E-01	included
TMEM116	24558	ES	6:07	4	8	0.00	9.9E-01	excluded
SUMF1	62988	ES	3:4:5:6	2	7	0.00	9.9E-01	included
NMNAT3	67043	ES	3	2	5	0.00	9.9E-01	included
KANSL3	54554	ES	5.2:6:7:8	3.2	9	0.00	9.9E-01	included
HEPH	89332	ES	12:13:14	11	15	0.00	9.9E-01	included
DNAJC18	73613	ES	2	1	3	0.00	9.9E-01	excluded
KCNQ1	13926	ES	15	14	16	0.00	9.9E-01	excluded
LIG1	50693	ES	6:7.1:7.2	5	8	0.00	9.9E-01	excluded
RENBP	90538	ES	7	6	8	0.00	9.9E-01	excluded
GUCY1B3	70945	ES	7	6	8	0.00	9.9E-01	included
TM7SF3	101115	ES	10	8	11	0.00	9.9E-01	excluded
SLC25A41	47034	ES	3	2	4	0.00	9.9E-01	included
PSMB3	40592	ES	4.1:4.2	3	5	0.00	9.9E-01	excluded
LYPLA1	83862	ES	4	3	5	0.00	9.9E-01	included
CATSPERG	49633	ES	05:06.1	4	6.2	0.00	9.9E-01	excluded
TMEM167A	72697	ES	2	1	3	0.00	9.9E-01	included
SDHAF2	16226	ES	4.1:5.1:5.2	3	6	0.00	9.9E-01	included
CD6	16136	ES	10.1:10.2	9	11	0.00	9.9E-01	included
COL1A2	265305	ES	16:17:18:19:20:21:22:23:24:25:26:27:28:29:30:31:33:34:35:36	15	37	0.00	9.9E-01	excluded
RRP12	12696	ES	7:08	6	9	0.00	9.9E-01	excluded
AP3D1	46579	ES	23:24.2	22	25	0.00	9.9E-01	excluded
CLIP4	53090	ES	7	6	8	0.00	9.9E-01	excluded
PPP2R1B	18680	ES	2:03	1	5	0.00	9.9E-01	excluded
PPIA	79566	ES	6	5.1	7	0.00	9.9E-01	excluded
RNFT1	42842	ES	3:04	2	5	0.00	9.9E-01	included
MAPK11	62818	ES	3	2	4	0.00	9.9E-01	included
CIRBP	46439	ES	8.2	7.5	8.4	0.00	9.9E-01	included
CMC2	37735	ES	4.2:4.3:4.4	2	5	0.00	9.9E-01	included

AKR1A1	262727	ES	5.1	4	6	0.00	9.9E-01	included
ABHD17A	46561	ES	3	2.2	4.1	0.00	9.9E-01	excluded
ITM2B	25853	ES	3:04	2	5	0.00	9.9E-01	excluded
RPL18A	48381	ES	4.2:5.1	4.1	5.2	0.00	9.9E-01	excluded
IQGAP2	72553	ES	21	20	22	0.00	9.9E-01	excluded
GSTP1	17245	ES	6	5	7	0.00	9.9E-01	excluded
ADAL	30236	ES	6	5	7	0.00	9.9E-01	included
FYN	77276	ES	7.2:8	7.1	9	0.00	9.9E-01	excluded
TAF1B	52641	ES	8	7	9	0.00	9.9E-01	included
DMKN	101863	ES	8:09	6.4	11	0.00	9.9E-01	included
IDH3A	32032	ES	2:4.1:4.2:6.1:6.2:7:8.2	1	11.1	0.00	9.9E-01	included
USP46	69302	ES	4	1	6	0.00	9.9E-01	included
TNFRSF25	416	ES	6.2:6.3:6.4:6.5:7.1	5	7.2	0.00	9.9E-01	included
ARHGAP44	39321	ES	18:19	16	20	0.00	9.9E-01	included
BRPF1	63155	ES	9	8.2	10	0.00	9.9E-01	excluded
CDS2	58652	ES	10	9	11	0.00	9.9E-01	included
NVL	9941	ES	21	20	22	0.00	9.9E-01	included
DAGLB	78729	ES	5	4	6	0.00	1.0E+00	included
CDCA7L	78915	ES	4	3	5	0.00	1.0E+00	included
BIVM	26222	ES	2:03	1	4	0.00	1.0E+00	excluded
REPS1	77950	ES	12.3:13	11	14	0.00	1.0E+00	excluded
ACHE	81032	ES	4.1:4.2:4.3:4.4	3.5	5	0.00	1.0E+00	included
CXorf38	88842	ES	1.3:2.1:2.2:2.3	1.1	3	0.00	1.0E+00	excluded
PPA2	70222	ES	2:4:5:7:8:9	1	11	0.00	1.0E+00	excluded
RBBP4	1625	ES	4	3.2	6	0.00	1.0E+00	included
FAM21A	11558	ES	30.2:31:32.1	30.1	32.2	0.00	1.0E+00	excluded
ZNF841	51407	ES	5:06	4	7.1	0.00	1.0E+00	excluded
CPNE1	59193	ES	2.2	1.2	5	0.00	1.0E+00	included
GLS2	22449	ES	4.1:4.2:5:6:7:8.1	3	8.3	0.00	1.0E+00	included
ANKRD42	18054	ES	11	10	12.1	0.00	1.0E+00	excluded
PSMC2	81192	ES	5	4	6	0.00	1.0E+00	excluded
SLC43A1	15851	ES	7:08	6	9	0.00	1.0E+00	included
RBPJ	68985	ES	2.3:4.2	2.2	5	0.00	1.0E+00	included
PML	31661	ES	6.1:6.2	4	6.4	0.00	1.0E+00	included
MPPE1	44648	ES	9.1:9.2:10	8	11	0.00	1.0E+00	excluded
PER1	39097	ES	18	17	19.1	0.00	1.0E+00	included
METTL2B	81696	ES	2	1	4	0.00	1.0E+00	excluded
PEX1	80439	ES	9	8	10	0.00	1.0E+00	excluded
CDK2	22322	ES	4	3	6	0.00	1.0E+00	included
GUCY1A3	70925	ES	8.1:8.2:9	7	10	0.00	1.0E+00	excluded
NADK2	71808	ES	10:11	9	12	0.00	1.0E+00	included
CNTROB	39081	ES	12	11	13	0.00	1.0E+00	excluded
PPP1R12A	23529	ES	24	23	25	0.00	1.0E+00	included
MDM2	23102	ES	5.1:5.2:6:7:8.1:8.2:9:1<br>0:11:12.1:12.2	3	12.3	0.00	1.0E+00	excluded
ZNF180	50332	ES	4:06	2.2	7	0.00	1.0E+00	included
MROH1	85545	ES	33	32	34	0.00	1.0E+00	excluded
BSDC1	1607	ES	03:04.3	2	5	0.00	1.0E+00	excluded
TRIQK	84507	ES	04:06.1	2.1	6.2	0.00	1.0E+00	included
HDAC8	89501	ES	3.1:3.2:4.1	2	5	0.00	1.0E+00	included
RRP8	14160	ES	2.1:2.2:3.1:3.3	1	4	0.00	1.0E+00	excluded
MLLT4	78454	ES	32.2	31	33.1	0.00	1.0E+00	included
PPA2	70212	ES	4:05:07	2	9	0.00	1.0E+00	included
C11orf1	18689	ES	5	4	6	0.00	1.0E+00	excluded
DAG1	64882	ES	6	2.1	7	0.00	1.0E+00	excluded
NUDCD1	84885	ES	4	3	5	0.00	1.0E+00	excluded
ETFA	31939	ES	2:03:04	1	5	0.00	1.0E+00	excluded
INPP5J	61824	ES	3.3	3.1	4	0.00	1.0E+00	included
DBNL	79405	ES	2.1:2.2	1.1	3	0.00	1.0E+00	excluded
NFATC3	37186	ES	10	9.1	13	0.00	1.0E+00	included
GPR64	88618	ES	20:21	19	22	0.00	1.0E+00	included

PLCH2	276	ES	4.2:5.1:5.2:6:7:8:9:10:11:12:13:14:15:16:17:	4.1	19	NA	NA	included
MEGF6	313	ES	39	38	40	NA	NA	included
KCNAB2	370	ES	13:14:15:17:18:19:21:22.1:22.2:22.3	12	22.4	NA	NA	included
ACOT7	397	ES	1.2:5	1.1	6	NA	NA	included
TNFRSF25	432	ES	5:6.2:6.3:6.4:6.5:7.1:7.	4	8	NA	NA	included
RERE	545	ES	18:19:20:21:22:23:24	17	25	NA	NA	included
CLCN6	690	ES	3	2	4	NA	NA	included
KDM1A	1030	ES	11	9	12	NA	NA	included
EYA3	1370	ES	6:07	5	8	NA	NA	included
EYA3	1371	ES	6	5	8	NA	NA	included
EYA3	1374	ES	3	2	4	NA	NA	included
PTPRU	1431	ES	20	19	22	NA	NA	included
PTPRU	1432	ES	15	14	16	NA	NA	included
ZMYM4	1716	ES	10:11	9	12	NA	NA	included
CSF3R	1788	ES	15	14	16	NA	NA	included
CTPS1	2030	ES	3.2:4:5:6	1	7	NA	NA	included
CCDC30	2087	ES	20.2:22.1	20.1	22.2	NA	NA	included
ST3GAL3	2231	ES	12:13:14:15.1:16.2:19.1:19.2:20	9	21	NA	NA	included
ST3GAL3	2232	ES	10:13:14:15.1:16.2:19.1:19.2:20	9	21	NA	NA	included
ST3GAL3	2233	ES	13:14:15.1:16.2:19.1:1	9	21	NA	NA	included
ST3GAL3	2464	ES	4:06:09	3.1	13	NA	NA	included
NDC1	3067	ES	13.2:14	13.1	15	NA	NA	included
FAM151A	3147	ES	6.2:7:8.1	6.1	8.2	NA	NA	included
ACADM	3508	ES	3	2.2	4.2	NA	NA	included
ABCD3	3812	ES	12:13:14:15	11	16	NA	NA	included
VCAM1	3886	ES	5:06	4	7	NA	NA	included
SLC25A24	3939	ES	4	3	5	NA	NA	included
CLCC1	3974	ES	7.2:8:9:10:11:12:13.1	7.1	13.2	NA	NA	included
PTPN22	4290	ES	9:10:11:12:13:14:15:16:17:18:19:20	8	21.1	NA	NA	included
DENND2C	4327	ES	14.2:15:16.1	14.1	16.2	NA	NA	included
NBPF10	4450	ES	74:75:76:77:78:79:80:	73	82	NA	NA	included
NBPF12	7352	ES	60:61:62:63:64:65	59.3	66	NA	NA	included
MTMR11	7416	ES	8:9:10:11:12:13	7	14	NA	NA	included
SELENBP1	7617	ES	10.2:11.1	10.1	11.2	NA	NA	included
SHC1	7858	ES	2.2:2.3:2.4:3:4:5.1	2.1	5.2	NA	NA	included
ADAM15	7922	ES	11.3:12	11.2	13.1	NA	NA	included
ADAM15	7928	ES	5.1:5.2:6:7:8:9:10:11.	4	11.2	NA	NA	included
KIRREL	8367	ES	4:05	3	6	NA	NA	included
KIRREL	8369	ES	2:03	1.1	4	NA	NA	included
DUSP12	8732	ES	4	3.2	5	NA	NA	included
POU2F1	8865	ES	9	8	10	NA	NA	included
SELP	8937	ES	6	5	7	NA	NA	included
SELE	8945	ES	8:09	7	10	NA	NA	included
CFH	9277	ES	5	4	6	NA	NA	included
MDM4	9481	ES	3:4:5:8:9:10:11:12:13.	2	13.2	NA	NA	included
MDM4	9482	ES	3:4:5:9:10:11:12:13.1	2	13.2	NA	NA	included
DSTYK	9526	ES	4.2:5:6:7:8:9:10:11.1	4.1	11.2	NA	NA	included
KLHDC8A	9534	ES	4:05	3	6	NA	NA	included
CD46	9665	ES	2	1	3	NA	NA	included
TRAF3IP3	9684	ES	15:16.1:17:18	14	19	NA	NA	included
EPRS	9854	ES	12	11	13	NA	NA	included
EPRS	9856	ES	6	5	7	NA	NA	included
SUSD4	9914	ES	11.2:12.1	11.1	12.2	NA	NA	included
LGALS8	10387	ES	6	5	7.1	NA	NA	included
LGALS8	10389	ES	3.1:3.2:3.3	2	5	NA	NA	included
PFKFB3	10702	ES	12:13	11	14	NA	NA	included
ITIH5	10717	ES	12.2:14:15:16.1	12.1	16.2	NA	NA	included

SEC61A2	10763	ES	11	10	12	NA	NA	included
TRDMT1	10895	ES	3:04:05	2	6	NA	NA	included
TRDMT1	10902	ES	3:4:5:6:7:8	2	9	NA	NA	included
TRDMT1	10903	ES	4:5:6:7:8	2	9	NA	NA	included
ABI1	11047	ES	6	4	7	NA	NA	included
FAM21A	11566	ES	5.2:6:7	5.1	8	NA	NA	included
JMJD1C	11891	ES	12	11	13	NA	NA	included
TTC18	12141	ES	7.2:8:9:10:11:12:13:14.1:15.1:15.2:16:17:18:19:20:21:22:23:24.1	7.1	24.2	NA	NA	included
SEC24C	12192	ES	2:03:04	1	5	NA	NA	included
SEC24C	12193	ES	3:04	1	5	NA	NA	included
SEC24C	12196	ES	2:3:4:5:6:7:8:9:10:11:12:13:14:15:16:17	1	18	NA	NA	included
SEC24C	12197	ES	3:4:5:6:7:8:9:10:11:12:13:14:15:16:17	1	18	NA	NA	included
SEC24C	12201	ES	2:3:4:5:6:7:9:10:11:12:13:14:15:16:17	1	18	NA	NA	included
SEC24C	12202	ES	3:4:5:6:7:9:10:11:12:13:14:15:16:17	1	18	NA	NA	included
DYDC1	12354	ES	2	1.1	3	NA	NA	included
NOLC1	12940	ES	3.1:3.2:4.1	2	4.2	NA	NA	included
NOLC1	12941	ES	3.2:4.1	2	4.2	NA	NA	included
COL17A1	13033	ES	50	49	51	NA	NA	included
BBIP1	13095	ES	04:05.1	1	5.2	NA	NA	included
BBIP1	13096	ES	03:05.1	1	5.2	NA	NA	included
C10orf118	13187	ES	16	15	17	NA	NA	included
PTPRE	13466	ES	10	9	11	NA	NA	included
ADAM8	13523	ES	19:20.1	18	20.2	NA	NA	included
TOLLIP	13833	ES	1.2:3.1	1.1	3.2	NA	NA	included
TRIM3	14128	ES	7.2:8.1	7.1	8.2	NA	NA	included
ZNF143	14321	ES	7.1:7.2	6	8	NA	NA	included
CYP2R1	14481	ES	5.2:5.3:6.1:6.2	5.1	6.3	NA	NA	included
CYP2R1	14482	ES	5.2:5.3:6.2	5.1	6.3	NA	NA	included
LDHA	14639	ES	2.4:3:4:6.1	2.3	6.2	NA	NA	included
LDHA	14640	ES	2.4:4:6.1	2.3	6.2	NA	NA	included
LIN7C	14757	ES	3	2	4	NA	NA	included
NUP160	15807	ES	33:34.1	31	34.2	NA	NA	included
CYB561A3	16173	ES	5.1:5.2:6.1:6.2:6.3	4.2	6.4	NA	NA	included
CYB561A3	16176	ES	5.1:5.2:6.1:6.3	4.2	6.4	NA	NA	included
BEST1	16316	ES	8.2:9	8.1	10	NA	NA	included
BBS1	17056	ES	4:5.1:5.3:6.1:6.2	3	7	NA	NA	included
RBM14-RBM4	17101	ES	2	1	3.1	NA	NA	included
LRFN4	17142	ES	2.3:3.1	2.2	3.2	NA	NA	included
ADRBK1	17154	ES	13.2:14:15:16:17:18:19:20:21.1	13.1	21.2	NA	NA	included
SSH3	17162	ES	7.2:8:9:10:11:12.1:12.	7.1	12.3	NA	NA	included
SSH3	17163	ES	7.2:8:9:10:11:12.2	7.1	12.3	NA	NA	included
RAD9A	17179	ES	5.1:5.2:6	4	7	NA	NA	included
TBC1D10C	17189	ES	7.2:8	7.1	9	NA	NA	included
TBC1D10C	17190	ES	8	7.1	9	NA	NA	included
PPP6R3	17311	ES	25	23	26.2	NA	NA	included
TPCN2	17358	ES	17:18:19:20:21:22:23	16	24	NA	NA	included
IL18BP	17479	ES	3:4.1:4.2	2	4.4	NA	NA	included
CLPB	17596	ES	16	15	17	NA	NA	included
PDE2A	17618	ES	8:9:10.1:11:12:13:14:	7.2	17	NA	NA	included
UCP3	17759	ES	4:05	3	6.1	NA	NA	included
GDPD5	17864	ES	6:7:8:13	5.2	14	NA	NA	included
AMOTL1	18366	ES	4.4:5:6:7:8:9:10:11.1	4.3	11.2	NA	NA	included

DYNC2H1	18489	ES	16:17:18:19:20:21:22:23:24:25:26:27:28:29:30:31:32:33:34:35:36:37:38:39:40:41:42:43:44:45:46:47:48:49:50:51:52:53:54:55:56:57:58:59:60:61:62:63:65:66:67:68:69:70:71:72:	15	86	NA	NA	included
CASP1	18526	ES	3.3:3.4:4	3.2	5	NA	NA	included
CASP1	18532	ES	3.3:3.4:4:5:6:7:8	3.2	9.2	NA	NA	included
CASP1	18533	ES	3.3:3.4:5:6:7:8	3.2	9.2	NA	NA	included
SLC35F2	18591	ES	11	10	12	NA	NA	included
CUL5	18598	ES	3	2	4	NA	NA	included
RDX	18636	ES	7.2:8:9:10:11:12:13:1	7.1	15.2	NA	NA	included
RDX	18641	ES	3.1:3.2:4:5:6:7.1:7.2:8	2	11	NA	NA	included
RDX	18643	ES	4:5:6:7.1:7.2:8:9:10	2	11	NA	NA	included
MPZL3	18978	ES	2.1:2.2:3:4	1	5	NA	NA	included
VWA5A	19213	ES	10.1:11:12:13:14	9	15	NA	NA	included
ROBO3	19251	ES	22	21	23	NA	NA	included
TMEM218	19274	ES	4.3:4.4:5.2	4.2	5.3	NA	NA	included
CHEK1	19316	ES	11	10	12	NA	NA	included
FLI1	19419	ES	7:08	5	9	NA	NA	included
JAM3	19535	ES	5	4	6	NA	NA	included
ERC1	19660	ES	4:5:6:7:8	3.2	9	NA	NA	included
ERC1	19661	ES	4:5:6:8	3.2	9	NA	NA	included
NECAP1	20175	ES	4:05	3	6	NA	NA	included
OLR1	20337	ES	06:07.1	4	7.2	NA	NA	included
EPS8	20587	ES	2	1	4	NA	NA	included
TMTC1	20933	ES	12	11	13	NA	NA	included
FAM60A	20984	ES	3:04	1	5	NA	NA	included
FAM60A	20985	ES	4	1	5	NA	NA	included
TWF1	21275	ES	7	6	8	NA	NA	included
PFKM	21427	ES	8:9:10:12:13:14.1	7.3	14.2	NA	NA	included
PFKM	21428	ES	9:10:12:13:14.1	7.3	14.2	NA	NA	included
CACNB3	21474	ES	10	9	11	NA	NA	included
BIN2	21841	ES	13:14.1	12	14.2	NA	NA	included
TARBP2	22088	ES	1.2:3.1	1.1	3.2	NA	NA	included
PMEL	22312	ES	6:7.1:7.2:7.3:8	5.2	9	NA	NA	included
PMEL	22315	ES	03:04.1	2.3	5.1	NA	NA	included
NACA	22495	ES	3.1:3.3:3.5	2.2	4.2	NA	NA	included
CDK4	22736	ES	3.2:4:5.1	3.1	5.2	NA	NA	included
MON2	22840	ES	23	22	24	NA	NA	included
MSRB3	22873	ES	4:05	3	6	NA	NA	included
MDM1	22930	ES	3.1:3.2	2	3.4	NA	NA	included
MDM1	22931	ES	3.2	2	3.4	NA	NA	included
MDM2	22978	ES	8.2:9:10:11:12.1:12.2:12.3:12.4	8.1	12.5	NA	NA	included
MDM2	22985	ES	7:8.1:8.2:9:10:11	6	12.1	NA	NA	included
MDM2	23033	ES	5.1:5.2:6:7:8.1:8.2:9:1	3	12.1	NA	NA	included
MDM2	23086	ES	5.1:5.2:6	3	7	NA	NA	included
MDM2	23166	ES	3:5.1:5.2:6:7:8.1:8.2:9	2.1	12.1	NA	NA	included
CPSF6	23305	ES	6.2:7:8.1	6.1	8.2	NA	NA	included
CPSF6	23306	ES	6.2:8.1	6.1	8.2	NA	NA	included
KRR1	23466	ES	7	6	8	NA	NA	included
KITLG	23614	ES	2:3:4:5:6	1	7	NA	NA	included
NTN4	23805	ES	9	8	10	NA	NA	included
ANKS1B	23896	ES	2:3:4:5:6:7:8	1	9	NA	NA	included
RIC8B	24174	ES	3:5:7:8:9:10	1	11	NA	NA	included
ACACB	24284	ES	24	23.2	25	NA	NA	included
GIT2	24392	ES	10	8	11.1	NA	NA	included
GIT2	24393	ES	9	8	11.1	NA	NA	included
ATXN2	24525	ES	1.2:2	1.1	3	NA	NA	included

RNFT2	24670	ES	7.2:8:9:10:11:12.1:12.	7.1	12.3	NA	NA	included
C12orf43	24814	ES	5.2:6.1:6.2:7.1:7.2	5.1	7.3	NA	NA	included
C12orf43	24816	ES	5.2:6.1:6.2:7.2	5.1	7.3	NA	NA	included
KDM2B	24884	ES	10:11	9	13	NA	NA	included
KNTC1	24978	ES	28:29:30:31:32:33:34:<br>35:36:37:38:39:40:41:<br>42:43:44:45:46.2:47:4	27	52	NA	NA	included
DENR	24984	ES	7.2:8.1	7.1	8.2	NA	NA	included
HSPH1	25579	ES	4.2:5	4.1	6	NA	NA	included
WBP4	25725	ES	3	2	4	NA	NA	included
RB1	25856	ES	2:3:4:5:6	1	7	NA	NA	included
INTS6	25944	ES	10.2:12:13:14:15:16:1<br>7:18:19.1	10.1	19.2	NA	NA	included
NEK5	25983	ES	17:18:19	16	20	NA	NA	included
IPO5	26159	ES	9.2:11:12:13.1	9.1	13.2	NA	NA	included
ARHGEF7	26287	ES	16	15	17	NA	NA	included
APEX1	26447	ES	2.2:2.3:3:4:5.1	2.1	5.2	NA	NA	included
APEX1	26448	ES	2.2:3:4:5.1	2.1	5.2	NA	NA	included
RNF31	26872	ES	3.2:4.1:4.2:5:6:7:8:9:1	3.1	11.2	NA	NA	included
RNF31	26873	ES	3.2:4.1:5:6:7:8:9:10:1	3.1	11.2	NA	NA	included
AP4S1	27103	ES	5.1:5.2:7	4.1	8	NA	NA	included
AKAP6	27137	ES	13	12	14	NA	NA	included
MAP4K5	27482	ES	4:05:06	3	7	NA	NA	included
PSMC6	27550	ES	5	4	6	NA	NA	included
ACTR10	27677	ES	6.2:7.1	6.1	7.2	NA	NA	included
RHOJ	27818	ES	3.2:4.1:5.1	3.1	5.2	NA	NA	included
SYNE2	27857	ES	94	93	95	NA	NA	included
SYNE2	27859	ES	74.1:75.1	73	75.2	NA	NA	included
MAX	27958	ES	3	2	5.1	NA	NA	included
MAX	27960	ES	2:3:5.1:5.2:5.3:5.4:5.5	1.2	5.8	NA	NA	included
MAX	27961	ES	3:5.1:5.2:5.3:5.4:5.5:5.	1.2	5.8	NA	NA	included
MAX	27972	ES	2:3:5.1:5.5:5.6:5.7	1.2	5.8	NA	NA	included
MAX	27973	ES	3:5.1:5.5:5.6:5.7	1.2	5.8	NA	NA	included
MAX	28005	ES	03:05.1	1.2	5.8	NA	NA	included
MAX	28006	ES	03:05.1	1.2	5.8	NA	NA	included
ZFYVE26	28104	ES	9:10	8	11	NA	NA	included
AREL1	28414	ES	13	12	14	NA	NA	included
YLPM1	28428	ES	13	12	14	NA	NA	included
VIPAS39	28596	ES	5	4	6	NA	NA	included
DIO2	28652	ES	3.3:4:5.1	3.2	5.2	NA	NA	included
DIO2	28653	ES	3.3:5.1	3.2	5.2	NA	NA	included
NRDE2	28817	ES	2:03	1	4	NA	NA	included
C14orf159	28856	ES	10.2:11.1	10.1	11.2	NA	NA	included
ATXN3	28901	ES	10.3:12	10.2	13.2	NA	NA	included
ATXN3	28927	ES	6	5	8.1	NA	NA	included
ATXN3	28929	ES	5	4.2	6	NA	NA	included
MOK	29386	ES	6.1	5	7	NA	NA	included
XRCC3	29498	ES	2.1:2.2:3.2:4.1:4.2:5:6	1.1	10	NA	NA	included
XRCC3	29501	ES	3.2:4.1:4.2:5:6:9	1.1	10	NA	NA	included
TDRD9	29537	ES	29:30:31:32:33	28	34	NA	NA	included
GOLGA8R	29772	ES	5:6:7:8:9:10	4	11	NA	NA	included
DNAJC17	30044	ES	6	5.1	7.2	NA	NA	included
MAPKBP1	30105	ES	29.2:30	29.1	31	NA	NA	included
MAPKBP1	30110	ES	10:11	8	12	NA	NA	included
SPG11	30347	ES	37:38:39	36	40	NA	NA	included
FBN1	30500	ES	7.2:8:9:10:11:12:13:1<br>4:15:16:17:18:19:20:2<br>1:22:23:24:25:26:27:2	7.1	33	NA	NA	included
CCNB2	30930	ES	2:03	1	4	NA	NA	included
RAB11A	31240	ES	5.2:5.3:6.1	5.1	6.2	NA	NA	included
NOX5	31380	ES	2	1	4	NA	NA	included

TLE3	31423	ES	1.2:2.3:3.2:5:6:7.1:7.2:8.1:8.2:9:11:12:13:14.1:14.2:14.3:15.1	1.1	15.2	NA	NA	included
TLE3	31424	ES	1.2:2.3:3.2:5:6:7.1:8.1:8.2:9:11:12:13:14.1:1	1.1	15.2	NA	NA	included
TLE3	31425	ES	1.2:2.3:3.2:5:6:7.1:8.2:9:11:12:13:14.1:14.2:	1.1	15.2	NA	NA	included
TLE3	31429	ES	1.2:2.3:3.2:5:6:7.1:7.2:8.1:8.2:9:11:12:13:14	1.1	15.2	NA	NA	included
TLE3	31430	ES	1.2:2.3:3.2:5:6:7.1:8.1:8.2:9:11:12:13:14.1:1	1.1	15.2	NA	NA	included
TLE3	31431	ES	1.2:2.3:3.2:5:6:7.1:8.2:9:11:12:13:14.1:14.2:	1.1	15.2	NA	NA	included
TLE3	31436	ES	1.2:2.3:3.2:5:6:7.1:8.1:8.2:9:11:12:13:14.1:1	1.1	15.2	NA	NA	included
LRRC49	31452	ES	16	15.2	17	NA	NA	included
LRRC49	31472	ES	7:10	4.1	11	NA	NA	included
CPEB1	32234	ES	8	7.3	9	NA	NA	included
HOMER2	32243	ES	6.2:7.1	6.1	7.2	NA	NA	included
ZSCAN2	32305	ES	1.2:2.1:2.2	1.1	5.1	NA	NA	included
ZSCAN2	32306	ES	2.1:2.2	1.1	5.1	NA	NA	included
FES	32497	ES	17	16	18	NA	NA	included
MEF2A	32716	ES	11:12	10	14	NA	NA	included
TELO2	33104	ES	9.2:10:11:12:13:14:15	9.1	17.2	NA	NA	included
IFT140	33110	ES	4.2:5:6:7:8:9:10	4.1	11	NA	NA	included
NAA60	33539	ES	3.2:4:6.2	3.1	7	NA	NA	included
NAA60	33540	ES	04:06.2	3.1	7	NA	NA	included
PARN	34086	ES	3.2:4:5.1	3.1	5.2	NA	NA	included
KIAA0430	34166	ES	16	15	17	NA	NA	included
KIAA0430	34176	ES	8.3:9:10.1	8.2	10.2	NA	NA	included
ARL6IP1	34270	ES	3.2:4:5:6	3.1	7	NA	NA	included
CCP110	34317	ES	4:05	3	6	NA	NA	included
NPIPB4	34542	ES	9.17:9.18:10.2	9.16	10.3	NA	NA	included
UQCRC2	35522	ES	13:14	12	15	NA	NA	included
PDZD9	35526	ES	3	2	4	NA	NA	included
PDZD9	35527	ES	2:03	1	4	NA	NA	included
SCNN1B	35581	ES	7	6.2	8	NA	NA	included
CLN3	35721	ES	14:15:17:18	13	19.1	NA	NA	included
CLN3	35722	ES	14:15:16:18	13	19.1	NA	NA	included
CLN3	35723	ES	14:15:18	13	19.1	NA	NA	included
MAZ	35947	ES	3.2:4.2:5.1:6.1:7.1:7.2	3.1	7.3	NA	NA	included
MAZ	35948	ES	3.2:4.2:5.1:7.1:7.2	3.1	7.3	NA	NA	included
MAPK3	36085	ES	7.2:7.4	7.1	7.5	NA	NA	included
DCTPP1	36114	ES	1.2:3.2:4.1	1.1	4.2	NA	NA	included
ITGAL	36126	ES	19:20.2:21:22:23:24.1	18.2	24.2	NA	NA	included
ITGAL	36140	ES	2.3:4.2:5:6:7:8:9.1:10:11:12:13:14:15:16:17.	2.2	17.2	NA	NA	included
FBXL19	36206	ES	7	6	8.1	NA	NA	included
TGFB1I1	36272	ES	4.2	1	5	NA	NA	included
ORC6	36310	ES	5	4	6	NA	NA	included
LONP2	36336	ES	6	5	7	NA	NA	included
ADCY7	36376	ES	10.2:11.1:11.2:12:13	10.1	14	NA	NA	included
CYLD	36393	ES	10:11	9	12	NA	NA	included
CIAPIN1	36553	ES	3.3:4	3.2	5	NA	NA	included
CMTM1	36770	ES	1.2:1.3:1.4:5.1	1.1	5.2	NA	NA	included
CMTM1	36782	ES	1.2:1.3:1.4:5.1:5.2:6.1	1.1	6.2	NA	NA	included
CENPT	37130	ES	15.2:16.1	15.1	16.2	NA	NA	included
PLA2G15	37195	ES	2:3:4.1:5:6.1:6.2	1	7	NA	NA	included
PLA2G15	37196	ES	3:4.1:5:6.1:6.2	1	7	NA	NA	included
PLA2G15	37199	ES	3:4.1:5:6.2	1	7	NA	NA	included
CDH1	37249	ES	5	4	6	NA	NA	included
CYB5B	37287	ES	3:04	2	5.1	NA	NA	included

COG4	37402	ES	5.2:6.1	5.1	6.2	NA	NA	included
MLKL	37571	ES	4:5:6:7:8	3	9.1	NA	NA	included
FA2H	37572	ES	3:04	2	5	NA	NA	included
TMEM231	37619	ES	3	2.3	4	NA	NA	included
MON1B	37651	ES	3.3:4:5.1	3.2	5.2	NA	NA	included
DYNLRB2	37692	ES	5.2	3	6	NA	NA	included
CDH13	37781	ES	10	9	13	NA	NA	included
FBXO31	37938	ES	1.2:2:3:4:5:6:7:8.1	1.1	8.2	NA	NA	included
RNF166	38017	ES	1.2:3:5.2	1.1	5.3	NA	NA	included
GALNS	38033	ES	1.2:2.1:3.1	1.1	3.2	NA	NA	included
SCARF1	38357	ES	5.2:6.1:6.2:7	5.1	8	NA	NA	included
ARRB2	38568	ES	05:06.1	4.2	7	NA	NA	included
RNF167	38608	ES	3	2.6	4	NA	NA	included
DHX33	38700	ES	2:03:04	1	5	NA	NA	included
ALOX15B	39083	ES	10.2:11.1:11.2	10.1	12	NA	NA	included
GAS7	39266	ES	10:11:12:13:14:15:16	9.2	17	NA	NA	included
KIAA0100	39937	ES	4	3	5	NA	NA	included
DHRS13	40018	ES	1.3:2	1.1	3	NA	NA	included
MYO18A	40039	ES	32	31	33	NA	NA	included
TAOK1	40043	ES	16:17	15	18	NA	NA	included
NLE1	40279	ES	3.2:4	3.1	5	NA	NA	included
CCL16	40365	ES	2:03	1	4	NA	NA	included
ZNHIT3	40474	ES	2:3.1:3.2:4	1	6	NA	NA	included
MYO19	40485	ES	19:20:21.1:21.2:21.3:22:24:25:26:27.1	18	27.2	NA	NA	included
MYO19	40486	ES	19:20:21.2:21.3:22:24:25:26:27.1	18	27.2	NA	NA	included
MYO19	40487	ES	16.2:17:18:19:20:21.1	16.1	21.3	NA	NA	included
MYO19	40488	ES	16.2:17:18:19:20:21.2	16.1	21.3	NA	NA	included
PLXDC1	40624	ES	12:13	11	14.1	NA	NA	included
GRB7	40699	ES	5.2:6:7.1	5.1	7.2	NA	NA	included
BRCA1	41188	ES	15.1:15.2:16:17:18	13	19	NA	NA	included
BRCA1	41198	ES	11.1:11.2	10	12	NA	NA	included
FAM187A	41907	ES	2.2:3:4.1	2.1	4.2	NA	NA	included
SRSF1	42633	ES	2.2:3.1:3.2:3.3:3.4	2.1	3.5	NA	NA	included
SRSF1	42634	ES	2.2:3.1:3.3:3.4	2.1	3.5	NA	NA	included
SRSF1	42636	ES	2.2:3.1:3.3	2.1	3.5	NA	NA	included
MKS1	42645	ES	17.3:18:19.1	17.2	19.2	NA	NA	included
MTMR4	42685	ES	16	15	17	NA	NA	included
TRIM37	42727	ES	5.1:6:7	3	8	NA	NA	included
GDPD1	42772	ES	5	4	6	NA	NA	included
TUBD1	42817	ES	3:5:6:7	2	8.1	NA	NA	included
DCAF7	42939	ES	4.2:5:6:7:8.1	4.1	8.2	NA	NA	included
PSMD12	43111	ES	4	1	5	NA	NA	included
NOL11	43115	ES	5	4	6	NA	NA	included
GPRC5C	43248	ES	1.2:3.1:3.2	1.1	3.3	NA	NA	included
CD300A	43252	ES	4:05:06	1	7	NA	NA	included
TMC6	43762	ES	13:14.1:14.2:14.3:15:16:17:18:19:20.1	12	20.2	NA	NA	included
TMC6	43763	ES	13:14.1:14.3:15:16:17:18:19:20.1	12	20.2	NA	NA	included
TMC6	43764	ES	13:14.1:15:16:17:18:1	12	20.2	NA	NA	included
SYNGR2	43777	ES	3.1:3.2	1	4.1	NA	NA	included
SYNGR2	43778	ES	3.2	1	4.1	NA	NA	included
CYTH1	43896	ES	6:7:8:9:10:11.1:11.2:13.1:13.2:14:15:16.1	5	16.2	NA	NA	included
CYTH1	43898	ES	6:7:8:9:10:11.1:12:13.2:14:15:16.1	5	16.2	NA	NA	included
CYTH1	43900	ES	6:7:8:9:10:11.1:13.2:14:15:16.1	5	16.2	NA	NA	included
IMPA2	44665	ES	2.2:3.1	2.1	3.2	NA	NA	included
CEP76	44709	ES	4.1:4.2	3.2	5	NA	NA	included

DTNA	45132	ES	10:11.2:13:17:22	9	23	NA	NA	included
ZSCAN30	45158	ES	3.2:4.2	3.1	4.3	NA	NA	included
FHOD3	45256	ES	15.2:16:17:18:19:20.1	15.1	20.2	NA	NA	included
FHOD3	45257	ES	15.2:16:18:19:20.1	15.1	20.2	NA	NA	included
PSTPIP2	45363	ES	11	10	12	NA	NA	included
KATNAL2	45432	ES	4:5:6:7:8:9:10:11:12:1	3.2	14	NA	NA	included
RPL17-C18orf32	45477	ES	2	1	3	NA	NA	included
MBD1	45516	ES	17:18.1:18.2	16.2	18.3	NA	NA	included
NEDD4L	45655	ES	18:19:20	17	21	NA	NA	included
APC2	46498	ES	5.2:6:7:8:9:10:11.1	5.1	11.2	NA	NA	included
AP3D1	46580	ES	6.2:7:8.1	6.1	8.2	NA	NA	included
AP3D1	46581	ES	4.2:5:6.1:6.2:7:8.1:8.2	4.1	10	NA	NA	included
FZR1	46691	ES	6:07	5	8	NA	NA	included
DUS3L	46933	ES	3.2:3.3:4.1:4.2:4.3	3.1	5	NA	NA	included
MARCH2	47232	ES	3.2:4:5:6.1	3.1	6.2	NA	NA	included
MARCH2	47233	ES	3.2:4:6.1	3.1	6.2	NA	NA	included
ADAMTS10	47256	ES	8.2:9:10	8.1	11	NA	NA	included
TYK2	47511	ES	1.2:2:3:4:5	1.1	6	NA	NA	included
ATG4D	47542	ES	3.2:4.1:4.2:5:6:7	3.1	8	NA	NA	included
ATG4D	47543	ES	3.2:4.1:5:6:7	3.1	8	NA	NA	included
ATG4D	47544	ES	3.2:5:6:7	3.1	8	NA	NA	included
LDLR	47625	ES	4:05	3	6	NA	NA	included
FBXW9	47844	ES	1.2:2.1	1.1	2.2	NA	NA	included
ZNF333	48024	ES	5:06	4	8	NA	NA	included
CPAMD8	48221	ES	13:14:15:16	12	17.1	NA	NA	included
MRPL34	48274	ES	4.3:5.1	4.2	5.2	NA	NA	included
ZNF682	48714	ES	5.2:6	1	9	NA	NA	included
C19orf40	48916	ES	2	1.1	3.1	NA	NA	included
PDCD2L	48962	ES	4:05	3.2	6	NA	NA	included
LSR	49091	ES	1.2:2.1	1.1	2.2	NA	NA	included
CD22	49113	ES	11:12	10	13	NA	NA	included
RBM42	49222	ES	9.1:9.2	8	10	NA	NA	included
ETV2	49237	ES	2:03	1.5	4	NA	NA	included
PPP1R14A	49597	ES	3.1:4.1	2.2	4.2	NA	NA	included
PPP1R14A	49598	ES	2.2	1	3.1	NA	NA	included
MAP4K1	49671	ES	23	22	24.1	NA	NA	included
NUMBL	49941	ES	3	1	4	NA	NA	included
CEACAM1	50171	ES	3:04:05	2	8	NA	NA	included
VASP	50485	ES	3.2:4:5:6.1	3.1	6.2	NA	NA	included
CCDC61	50538	ES	7:8.1:9.1:9.2:10	6	11	NA	NA	included
TMEM143	50756	ES	2:04	1	5	NA	NA	included
TRPM4	50902	ES	9:10	8	11.1	NA	NA	included
CPT1C	51053	ES	21:22	20	23	NA	NA	included
SIGLEC7	51307	ES	2:3:4:5:6	1	7	NA	NA	included
ZNF665	51697	ES	2:03	1	4	NA	NA	included
LENG8	51900	ES	3.2:4:5:6:7:8:9:10:11:<br>12:13:14:15.1:15.2	3.1	15.3	NA	NA	included
LENG8	51901	ES	3.2:4:6:7:8:9:10:11:12<br>:13:14:15.1:15.2	3.1	15.3	NA	NA	included
ZNF548	52243	ES	4	1	5.1	NA	NA	included
NLRC4	53174	ES	5	4	6	NA	NA	included
MORN2	53302	ES	1.2:2	1.1	3.2	NA	NA	included
MORN2	53303	ES	2	1.1	3.2	NA	NA	included
THADA	53385	ES	27:29:00	26	30	NA	NA	included
THADA	53387	ES	12:13.1	11.2	13.2	NA	NA	included
MSH6	53507	ES	3	1.2	4	NA	NA	included
LGALSL	53766	ES	4.2	3	5	NA	NA	included
MTHFD2	54023	ES	3:04:05	2	6	NA	NA	included
MTHFD2	54025	ES	3	2	4	NA	NA	included
TTC31	54092	ES	7.2:7.3:8:9.1:9.2:9.3:9.	7.1	9.8	NA	NA	included
TTC31	54093	ES	7.2:8:9.1:9.2:9.3:9.5:9.	7.1	9.8	NA	NA	included
TTC31	54094	ES	7.2:7.3:8:9.1:9.3:9.5:9.	7.1	9.8	NA	NA	included

TTC31	54095	ES	7.2:8:9.1:9.3:9.5:9.7	7.1	9.8	NA	NA	included
LOXL3	54110	ES	7:8:9:10:11	6	12	NA	NA	included
SEMA4F	54131	ES	6	5	7	NA	NA	included
ELMOD3	54209	ES	7.3:8	7.1	9	NA	NA	included
KDM3A	54437	ES	5	4	6	NA	NA	included
CD8B	54458	ES	2:03:04	1	5	NA	NA	included
KANSL3	54542	ES	23.3:24:25.1	23.2	25.2	NA	NA	included
KANSL3	54544	ES	19.2:20:21:22:23.1	19.1	23.2	NA	NA	included
MAP4K4	54764	ES	8:9:10:11	7	12	NA	NA	included
RANBP2	54893	ES	3	2	4	NA	NA	included
BUB1	54939	ES	22:23.1	21	23.2	NA	NA	included
FBLN7	54983	ES	4:05:06	3	7	NA	NA	included
POLR1B	55005	ES	3.1:3.2:4:5	1.3	6	NA	NA	included
UGGT1	55260	ES	4:05	2	6	NA	NA	included
THSD7B	55464	ES	7.2:8:9:10:11:12:13:14:15:16:17:19:20:21:22:23:24:25:26:27.1	7.1	27.2	NA	NA	included
ACVR2A	55519	ES	1.2:1.3:2.1	1.1	2.2	NA	NA	included
GPD2	55629	ES	13.2:14:15:16:17:18	13.1	19	NA	NA	included
ATF2	56091	ES	5	4	6	NA	NA	included
AGPS	56135	ES	3:4:5:6:7:8:9:10:11:12:13:14:15:16:17:18.1	2	18.2	NA	NA	included
MPP4	56865	ES	16	15	17.2	NA	NA	included
ABI2	57071	ES	2	1	5.1	NA	NA	included
NRP2	57108	ES	16.2:19.2	16.1	20.2	NA	NA	included
NRP2	57109	ES	19.2	16.1	20.2	NA	NA	included
IKZF2	57317	ES	7.1:7.2:9	4.1	10	NA	NA	included
TMBIM1	57470	ES	8	7	9	NA	NA	included
TTLL4	57563	ES	1.2:2:3.2	1.1	3.3	NA	NA	included
NHEJ1	57592	ES	2.2:3.1	2.1	3.2	NA	NA	included
COPS7B	57971	ES	3:4.1:4.2:4.3:4.4	1	5	NA	NA	included
COPS7B	57975	ES	3	1	5	NA	NA	included
GIGYF2	58017	ES	15.3:16.1	15.2	16.2	NA	NA	included
GIGYF2	58020	ES	08:09.1	7	9.2	NA	NA	included
UQCC1	59095	ES	7.2:8	7.1	9	NA	NA	included
UQCC1	59097	ES	6.2:7.1:7.2:8:9:10.1:1	6.1	11.2	NA	NA	included
RALGAPB	59381	ES	10	9	11	NA	NA	included
NCOA3	59725	ES	14:15.1	13	15.2	NA	NA	included
CSE1L	59735	ES	3.2:4:5:6:7.1	3.1	7.2	NA	NA	included
SALL4	59805	ES	2.1:2.2	1	3	NA	NA	included
STX16	59980	ES	4.2:4.3	1.4	5.1	NA	NA	included
STX16	59982	ES	4.2	1.4	5.1	NA	NA	included
SAMSN1	60215	ES	4:5:6:7	3	8	NA	NA	included
USP25	60223	ES	8:9:10:11:12:13:14:15:16:17:18:20:21:22	7	23	NA	NA	included
USP25	60224	ES	8:9:10:11:12:13:14:15:16:17:18:21:22	7	23	NA	NA	included
CXADR	60231	ES	4:05:06	3	7.1	NA	NA	included
CXADR	60234	ES	3:4:5:6	2	7.1	NA	NA	included
CXADR	60236	ES	3:04	2	7.1	NA	NA	included
USP16	60301	ES	3.2:4:5:6:7.1:7.2:8:9:10:11:12:13.1	3.1	13.2	NA	NA	included
USP16	60302	ES	3.2:4:5:6:7.2:8:9:10:11:12:13.1	3.1	13.2	NA	NA	included
SCAF4	60337	ES	3	2	4	NA	NA	included
ITSN1	60474	ES	19.2:21	19.1	22	NA	NA	included
ITSN1	60475	ES	20	19.1	22	NA	NA	included
ITGB2	60850	ES	8	7	9	NA	NA	included
GNB1L	61082	ES	6.2:7.1	6.1	7.2	NA	NA	included
ZNF74	61151	ES	5	4	6	NA	NA	included
GAS2L1	61593	ES	4.1	3	4.3	NA	NA	included
NF2	61631	ES	11.1:11.2:12	10	13	NA	NA	included

NF2	61639	ES	5:6:7:8:9:10:11.1:11.2:12:13:14:15:16.1	4	17	NA	NA	included
NF2	61645	ES	5:6:7:8:9:10:11.1:11.2:12:13:14:15	4	17	NA	NA	included
NF2	61657	ES	5:06:07	4	8	NA	NA	included
TNRC6B	62341	ES	10	9	11	NA	NA	included
SERHL2	62516	ES	2.2:3:4:5.2:6:7:8.1	2.1	8.2	NA	NA	included
CERK	62718	ES	5	4	6	NA	NA	included
CPT1B	62874	ES	13.2:14.1	13.1	14.2	NA	NA	included
ITPR1	63016	ES	13:14:15:16:17:18:19:20:21:22:23.1:24:25:26.1:27.1:27.2:28:29:30:31:32:33:34:35:36:37:38:39:43.2:44:45:46:	12	55	NA	NA	included
CAMK1	63174	ES	11	10	12	NA	NA	included
CIDEC	63234	ES	5:06:07	4	8	NA	NA	included
CIDEC	63235	ES	6:07	4	8	NA	NA	included
XPC	63523	ES	3.2:4.1	3.1	4.2	NA	NA	included
FBXL2	63847	ES	11.4:12:13.1:13.2	11.3	13.3	NA	NA	included
PDCD6IP	63889	ES	12	11	13	NA	NA	included
VILL	64007	ES	4.3:5:6:7:8:9:10.1	4.2	10.2	NA	NA	included
WDR48	64121	ES	18.2:19	18.1	20.1	NA	NA	included
TTC21A	64167	ES	3.1:4.1:5	2	6	NA	NA	included
NBEAL2	64487	ES	26:27.1	25	27.2	NA	NA	included
MAP4	64563	ES	10.2:11:12.1	10.1	12.2	NA	NA	included
SLC26A6	64725	ES	5:6.2:7.1	4.2	7.2	NA	NA	included
MST1	64901	ES	2	1	3	NA	NA	included
TRAIP	64924	ES	5:6:7:8	4	9	NA	NA	included
PBRM1	65242	ES	19:20.1	18	20.2	NA	NA	included
GLT8D1	65252	ES	1.3:2	1.2	4	NA	NA	included
EOGT	65561	ES	11:12:13	10	14	NA	NA	included
CPOX	65788	ES	1.2:2.1	1.1	2.2	NA	NA	included
NAA50	66214	ES	4.3:5:6:7.1	4.2	7.2	NA	NA	included
GRAMD1C	66228	ES	10:11.1	9	11.2	NA	NA	included
GRAMD1C	66230	ES	4:05:06	3	9	NA	NA	included
RABL3	66380	ES	5.1:5.2	4	6	NA	NA	included
CD86	66419	ES	4:05	3	6	NA	NA	included
DIRC2	66452	ES	2	1	3	NA	NA	included
SEC61A1	66636	ES	4:05:06	3.2	7	NA	NA	included
MBD4	66723	ES	2.1:2.2:3.1:3.2:4:5	1	6	NA	NA	included
MBD4	66724	ES	2.1:2.2:3.1:4:5	1	6	NA	NA	included
UBA5	66821	ES	12	11	13.1	NA	NA	included
DBR1	66958	ES	3:04	2	5	NA	NA	included
WWTR1	67229	ES	3.2:4.1	3.1	4.2	NA	NA	included
IL1RAP	68111	ES	04:02.2	1.1	6	NA	NA	included
IL1RAP	68112	ES	4.2:5	1.1	6	NA	NA	included
TFRC	68214	ES	4	3	5	NA	NA	included
UBXN7	68243	ES	2:3:4:5	1	6	NA	NA	included
SMIM20	68973	ES	2	1	3	NA	NA	included
TBC1D1	69019	ES	2.2:4:5:6.1	2.1	6.2	NA	NA	included
WDR19	69050	ES	5:06	4	7	NA	NA	included
TEC	69206	ES	8	7	9	NA	NA	included
FRYL	69226	ES	61	60	63	NA	NA	included
USO1	69552	ES	4	3	5	NA	NA	included
ANTXR2	69673	ES	9:10:11:13	8	14	NA	NA	included
HPSE	69783	ES	9:10	8	11	NA	NA	included
EXOSC9	70507	ES	3.2:4	3.1	5	NA	NA	included
FAT4	70533	ES	11	10	12	NA	NA	included
GUCY1A3	70928	ES	5.4:6:7:8.1:8.2:9	5.3	10	NA	NA	included
TMA16	71018	ES	5:6.1:7.1	4	7.2	NA	NA	included
WWC2	71264	ES	6:7:8:9	5	10	NA	NA	included
WWC2	71266	ES	6	5	7	NA	NA	included

WWC2	71267	ES	4:05	2	6	NA	NA	included
TRAPPC11	71286	ES	12.3:13:14:15:16:17:1	12.2	18.2	NA	NA	included
EGFLAM	71844	ES	14	13	15	NA	NA	included
ZNF131	71932	ES	5.2:6:7.1:7.2:7.3:7.4:8	5.1	9.3	NA	NA	included
ZNF131	71936	ES	5.2:6:7.1:7.4:8:9.1:9.2	5.1	9.3	NA	NA	included
IL6ST	72076	ES	10:11:12:13:14:15:16	8	17	NA	NA	included
SREK1	72275	ES	1.2:3.1	1.1	3.2	NA	NA	included
WDR41	72580	ES	08:09.1	6	9.2	NA	NA	included
HOMER1	72633	ES	2:3:4:5	1	6	NA	NA	included
MEF2C	72754	ES	11	10.1	12.2	NA	NA	included
DTWD2	73064	ES	4:05	3	6	NA	NA	included
REEP2	73547	ES	3	2.2	4	NA	NA	included
SPATA24	73607	ES	2	1	3	NA	NA	included
HARS2	73748	ES	3:04:05	2.2	6.1	NA	NA	included
RELL2	73810	ES	6	5	7	NA	NA	included
DCTN4	74114	ES	9	8	10	NA	NA	included
ANXA6	74152	ES	6.2:6.3:7.1:7.2:8:9:10:<br>11:12:13.1	6.1	13.2	NA	NA	included
SAP30L	74220	ES	02:03.1	1	3.2	NA	NA	included
CYFIP2	74342	ES	8.1:10.1:10.2:10.3:11:<br>12:13.1:13.2	6.2	14	NA	NA	included
CYFIP2	74377	ES	5.3:6.1:6.2:8.1:10.1:10	5.2	10.3	NA	NA	included
NOP16	74652	ES	2:3.2:4.1:5.1	1	5.2	NA	NA	included
RNF130	74996	ES	2.2:3.1	2.1	3.2	NA	NA	included
MAPK9	75009	ES	10.1:11	7	12	NA	NA	included
NEDD9	75343	ES	6.2	5	7	NA	NA	included
CAP2	75445	ES	4:6:7:8:10:11	3	12	NA	NA	included
KDM1B	75465	ES	1.2:2:3:4:5:6:7:8:9:10:<br>11:12:13:14:15:16:17.	1.1	20	NA	NA	included
KDM1B	75473	ES	1.2:2:3:4:5:6:7:8:9:10:<br>11:12:13:14:15:16:17.	1.1	20	NA	NA	included
MBOAT1	75491	ES	5	4	6	NA	NA	included
ALDH5A1	75517	ES	2	1.3	3	NA	NA	included
PRSS16	75685	ES	5:6:7:8	4	9	NA	NA	included
PRSS16	75701	ES	3:4:5:6:7:8	2	9	NA	NA	included
PRSS16	75702	ES	4:5:6:7:8	2	9	NA	NA	included
BRPF3	75964	ES	7:8:9:10	6.2	11.1	NA	NA	included
PTK7	76252	ES	5	4	6	NA	NA	included
GPR116	76432	ES	10:11	9	12	NA	NA	included
ZNF451	76589	ES	3:7:8:9:10:11	2.2	12	NA	NA	included
COL12A1	76771	ES	52:53:00	51	54	NA	NA	included
CEP57L1	77170	ES	06:07.1	5	7.2	NA	NA	included
DCBLD1	77345	ES	8:9:10:11	7	12	NA	NA	included
SLC18B1	77752	ES	3.2:4:5:6:7	3.1	8	NA	NA	included
HBS1L	77796	ES	4:7.1:8.1	3	8.2	NA	NA	included
MYB	77825	ES	16:17	15.1	19.1	NA	NA	included
REPS1	77947	ES	16	15	17	NA	NA	included
SYNE1	78186	ES	90.2	89	91	NA	NA	included
MAP3K4	78361	ES	2:03	1	5	NA	NA	included
AGPAT4	78376	ES	3.1:5.1:5.3	2	6.1	NA	NA	included
AGPAT4	78380	ES	2	1	3.1	NA	NA	included
SUN1	78529	ES	10:11:12:13	9.1	14	NA	NA	included
SUN1	78541	ES	8:9.1:10:11:12:13	7	14	NA	NA	included
MICALL2	78573	ES	1.2:2.2:3:4:5:6.1	1.1	6.2	NA	NA	included
PMS2	78699	ES	11.2:12:13:14.1	11.1	14.2	NA	NA	included
PMS2	78700	ES	8:9:10:11.1:11.2	7	12	NA	NA	included
CHN2	79088	ES	8:09:10	7	12.3	NA	NA	included
CAMK2B	79522	ES	12:14.1	11	14.2	NA	NA	included
CAMK2B	79525	ES	4:05	3	6	NA	NA	included
DDX56	79537	ES	3	2	4.1	NA	NA	included
TNS3	79607	ES	16:17:18:19.1	14	19.2	NA	NA	included
EGFR	79745	ES	5	4	6	NA	NA	included

TBL2	79975	ES	4.1:4.2:4.3	1	5.1	NA	NA	included
TBL2	79978	ES	03:04.3	1	5.1	NA	NA	included
TBL2	79980	ES	4.3	1	5.1	NA	NA	included
MLXIPL	79993	ES	4.2:5:6.1	4.1	6.2	NA	NA	included
VPS37D	79994	ES	2:03	1	4	NA	NA	included
CD36	80230	ES	16	15	17	NA	NA	included
CD36	80231	ES	14:15	13	16	NA	NA	included
CD36	80233	ES	10.2:11	10.1	12	NA	NA	included
DMTF1	80311	ES	1.2:2:3:4.2	1.1	6	NA	NA	included
DMTF1	80312	ES	4.2	1.1	6	NA	NA	included
DBF4	80346	ES	08:09.1	7	9.2	NA	NA	included
C7orf63	80381	ES	9	8	11	NA	NA	included
TFPI2	80481	ES	3.4:4	3.3	5	NA	NA	included
CASD1	80491	ES	2:03	1	4	NA	NA	included
CUX1	81072	ES	17:18	16.2	19	NA	NA	included
KMT2E	81272	ES	20.2:21	20.1	22	NA	NA	included
CBLL1	81369	ES	6	5	7	NA	NA	included
CADPS2	81612	ES	12	11	13.2	NA	NA	included
CEP41	81801	ES	5	2	6	NA	NA	included
MEST	81806	ES	13	12	14	NA	NA	included
SLC37A3	81992	ES	3:4:5:6	2	7	NA	NA	included
SLC37A3	81993	ES	3:05:06	2	7	NA	NA	included
EPHB6	82070	ES	5.3:6:7.1:7.2:7.3	5.2	7.4	NA	NA	included
GALNT11	82410	ES	6	1	7	NA	NA	included
INSIG1	82435	ES	2.2:3	2.1	4	NA	NA	included
ZNF596	82543	ES	4.1:5:6.1	3	6.2	NA	NA	included
INTS9	83227	ES	7	6	8	NA	NA	included
KIF13B	83255	ES	4:05:06	3	7	NA	NA	included
TACC1	83445	ES	10.1:10.2	8	11.1	NA	NA	included
PLAT	83570	ES	10:11:12:13.1	9	13.2	NA	NA	included
PLAT	83571	ES	7:8.1:8.2	6	9	NA	NA	included
VDAC3	83722	ES	7	6	8.1	NA	NA	included
VDAC3	83724	ES	5.1:5.2	4.1	6	NA	NA	included
GDAP1	84224	ES	1.2:2.1	1.1	2.2	NA	NA	included
ANKRD46	84710	ES	5	4	6.1	NA	NA	included
RRM2B	84762	ES	3.2:4:5:6:7:8	3.1	9	NA	NA	included
RRM2B	84763	ES	2:3.1:3.2:4:5:6:7:8:9	1	10	NA	NA	included
RRM2B	84764	ES	3.1:3.2:4:5:6:7:8:9	1	10	NA	NA	included
RRM2B	84769	ES	2:3.1:3.2:4:5:6:7	1	8	NA	NA	included
RRM2B	84770	ES	3.1:3.2:4:5:6:7	1	8	NA	NA	included
ATP6V1C1	84783	ES	1.2:2:3	1.1	4	NA	NA	included
OXR1	84856	ES	7:8:9:10:11:12.1:13:1	6.2	17	NA	NA	included
OXR1	84857	ES	7:8:9:10:11:12.1:13:1	6.2	17	NA	NA	included
COL14A1	85020	ES	11:12	10	13	NA	NA	included
ATAD2	85060	ES	4	3.1	5	NA	NA	included
SLA	85220	ES	02:03.2	1	5	NA	NA	included
SLA	85222	ES	2	1	3.2	NA	NA	included
DOCK8	85703	ES	23	22	24	NA	NA	included
CD274	85790	ES	3	2	4	NA	NA	included
KDM4C	85832	ES	14.2:15	14.1	16	NA	NA	included
TTC39B	85905	ES	11	10	12	NA	NA	included
NOL6	86118	ES	23.2:24:25:26.1	23.1	26.2	NA	NA	included
UBAP1	86146	ES	8	7	9	NA	NA	included
TLN1	86280	ES	43	42	44	NA	NA	included
TLN1	86282	ES	38.2:39.1	38.1	39.2	NA	NA	included
TRMT10B	86428	ES	4.2:5:6.1	4.1	6.2	NA	NA	included
TRMT10B	86429	ES	05:06.1	4.1	6.2	NA	NA	included
AGTPBP1	86731	ES	18:19:20:21:22:23:24:	17	26	NA	NA	included
ZCCHC6	86767	ES	7:8:9:10.1	6	11	NA	NA	included
PHF2	86919	ES	12.3:13:14:15:16:17:1	12.2	18.2	NA	NA	included
PHF2	86920	ES	3.2:4:5:6:7:8:9:10:11:	3.1	12.2	NA	NA	included
AKAP2	87178	ES	12	11	13	NA	NA	included

AKAP2	87179	ES	11:12	10	13	NA	NA	included
DENND1A	87522	ES	2:03	1	4	NA	NA	included
GARNL3	87624	ES	29	28	30	NA	NA	included
SPTAN1	87773	ES	36.2:37	36.1	39	NA	NA	included
GTF3C5	88007	ES	4.2:5:6.1	4.1	6.2	NA	NA	included
CARD9	88160	ES	12.2:13.1	12.1	13.2	NA	NA	included
CSF2RA	88364	ES	13:14:15	12	17.1	NA	NA	included
CSF2RA	88365	ES	14:15	12	17.1	NA	NA	included
CSF2RA	88373	ES	10:11:12:13:14	9	15	NA	NA	included
CSF2RA	88374	ES	10:11:12:14	9	15	NA	NA	included
ZFX	88709	ES	8	7	9	NA	NA	included
ZFX	88718	ES	2:3:5:7	1	8	NA	NA	included
CASK	88869	ES	12	10	13	NA	NA	included
CASK	88870	ES	11	10	13	NA	NA	included
FTSJ1	88967	ES	7	6	8	NA	NA	included
GRIPAP1	89054	ES	06:07.1	5	7.2	NA	NA	included
CCNB3	89135	ES	6	5	7	NA	NA	included
CCNB3	89136	ES	5:6:7:8	4	9	NA	NA	included
KDM5C	89191	ES	27.2:28.1	27.1	28.2	NA	NA	included
TRO	89261	ES	2.2:3	1.1	4	NA	NA	included
ARHGEF9	89305	ES	11.2:12.1	11.1	12.2	NA	NA	included
TEX11	89388	ES	3	2	4	NA	NA	included
ZMYM3	89413	ES	6	5.2	7	NA	NA	included
HDAC8	89473	ES	4.1	3.2	5	NA	NA	included
ABCB7	89516	ES	4	3	5	NA	NA	included
PGK1	89542	ES	4.2:5:6:7.1	4.1	7.2	NA	NA	included
CHM	89565	ES	2:03:04	1	6	NA	NA	included
MAP7D3	90199	ES	7	6	8	NA	NA	included
RBMX	90228	ES	02:03.2	1	4	NA	NA	included
ZNF185	90402	ES	13:14:15	12	16	NA	NA	included
ZNF185	90403	ES	13:14	12	16	NA	NA	included
ARHGAP4	90530	ES	6.2:8:9	5	10	NA	NA	included
SNX1	139191	ES	4.2:5:6:8:9:10.2:11:12	3	16.1	NA	NA	included
SNX1	139192	ES	6:8:9:10.2:11:12:14:1	3	16.1	NA	NA	included
HNRNPA1	212644	ES	3:4:5:6.1:6.2:6.3:7.2:8 :9.1:9.2:10:11.2	2	11.3	NA	NA	included
RPS6	214601	ES	1.2:1.4:1.5	1.1	1.6	NA	NA	included
ASNSD1	300788	ES	2:3.1:3.2:3.3:4.1:5	1	6	NA	NA	included
ASNSD1	300791	ES	3.3:4.1:5	1	6	NA	NA	included
PGK1	317814	ES	4.1	3	7.2	NA	NA	included

The ES events were sorted by the t-test p-values.

Supplementary Table 4. Result of motif enrichment analysis to identify RNA-binding protein binding sites around downregulated ES events.

RNA binding protein	smallest_p_in_upstreamExon-3prime	smallest_p_in_upstreamExonIntron	smallest_p_in_upstreamIntron	smallest_p_in_targetExon-5prime	smallest_p_in_targetExon-3prime	smallest_p_in_downstreamIntron	smallest_p_in_downstreamExonIntron	smallest_p_in_downstreamExon-5prime
BRUNOL5.TGTGT[GT][GT]	6.5E-02	1.6E-02	5.0E-02	5.3E-01	4.3E-01	9.3E-30	1.1E-02	9.6E-02
BRUNOL4.[GT]GTGT[GT][GT]	9.1E-02	9.5E-03	2.6E-01	2.4E-01	6.3E-01	1.1E-23	1.9E-02	4.4E-02
RBM24.[AT]G[AT]GTG[AGT]	1.9E-01	1.3E-02	3.8E-02	4.4E-01	2.3E-01	9.9E-14	6.4E-03	2.2E-01
RBM38.[GT][GT]GTGT[GT]	7.7E-01	3.6E-02	3.3E-01	2.5E-01	6.0E-01	1.2E-12	6.4E-02	2.0E-01
RBMS1.[GT]ATATA[GC]	2.3E-01	2.9E-01	5.1E-02	1.0E+00	1.0E+00	3.0E-12	1.6E-03	1.0E+00
KHDRBS2.[AG]ATAAA[AC]	4.7E-01	3.5E-01	7.2E-02	4.3E-01	4.9E-01	1.8E-09	4.5E-01	1.0E+00
SRp20.[AT]C[AT][AT]C	4.2E-03	1.3E-02	6.2E-04	9.9E-01	1.0E+00	9.3E-03	1.9E-07	8.6E-01
ENOX1.[ACT][AG][GT]ACAG	1.9E-07	3.8E-04	2.6E-02	4.2E-01	2.1E-01	4.1E-02	5.2E-02	2.3E-05
RBMS3.[AC]TATA[GT][AC]	1.0E+00	3.6E-01	3.3E-05	2.4E-01	3.0E-01	4.7E-07	2.1E-01	1.0E+00
FUS.CGCGC	1.0E+00	7.3E-01	1.0E+00	4.0E-02	1.0E-06	8.7E-02	5.6E-01	6.2E-01
KHDRBS3.ATAAA[ACG]	3.6E-01	1.9E-01	4.7E-02	4.5E-01	8.2E-01	1.7E-06	4.3E-01	3.6E-01
RBMS3.[ACT]ATATA	5.7E-01	3.1E-02	7.6E-03	5.0E-01	5.5E-01	2.4E-06	6.2E-02	1.0E+00
HNRNPL.ACAC[AG]A[ACG]	2.6E-06	5.0E-01	4.5E-01	3.1E-01	1.0E+00	1.3E-01	1.3E-01	1.4E-01
SRp40.[CT][AG]C[AG][GT][AC]	3.8E-01	1.5E-05	3.5E-03	7.7E-01	8.9E-01	3.1E-06	1.2E-03	7.4E-01
KHDRBS1.ATAAAA[ACG]	3.9E-01	2.8E-01	6.7E-01	1.0E+00	1.0E+00	5.4E-06	6.9E-01	9.9E-02
HNRNPL.A[AC]A[CT]A[AC]A	2.1E-02	1.2E-01	3.7E-01	1.0E+00	6.9E-01	7.3E-06	6.5E-04	3.4E-03
RBM28.G[AT]GTAG[AGT]	1.2E-01	7.6E-02	3.6E-03	2.4E-01	3.5E-01	2.6E-05	7.5E-02	9.9E-02
ANKHD1.AGACG[AT][AT]	3.0E-05	1.8E-02	1.0E+00	1.0E+00	1.0E+00	1.0E+00	1.1E-01	2.1E-01
HNRPLL.[AG]CA[ACT]ACA	9.1E-04	4.1E-05	5.6E-02	1.0E+00	1.0E+00	4.6E-03	3.0E-03	3.1E-03
IGF2BP2.[ACG][AC]A[ACT][AT]CA	3.4E-03	4.4E-04	5.2E-01	6.7E-01	3.9E-01	9.1E-03	4.7E-04	5.7E-05
PCBP2.CC[CT][CT]CC[ACT]	1.4E-01	1.7E-03	2.9E-03	1.6E-04	7.9E-05	6.9E-04	2.6E-02	8.4E-01
PABPC1.A[AG]AAAA[AC]	8.9E-03	3.7E-02	4.6E-02	1.8E-01	3.2E-01	4.6E-03	8.1E-05	1.7E-01
SART3.A[AG]AAAA[AC]	8.9E-03	3.7E-02	4.6E-02	1.8E-01	3.2E-01	4.6E-03	8.1E-05	1.7E-01
RBM45.GACGA[AC][ACG]	9.3E-05	1.7E-01	2.0E-01	1.0E+00	1.0E+00	2.6E-01	2.7E-01	3.4E-01
SF2-ASF.[AG]GAAGAAC	1.0E+00	1.9E-01	1.0E+00	1.0E+00	1.0E+00	1.0E+00	1.0E+00	1.0E-04
PTB.CTCT[CT][CT]	9.4E-01	5.0E-01	1.0E-04	7.1E-01	9.2E-01	4.1E-02	1.6E-02	1.3E-01
ZCRB1.G[AG][ACT]TTAA	4.3E-01	2.7E-01	1.5E-01	3.6E-01	5.6E-02	1.7E-01	1.1E-04	9.5E-02
PCBP1.C[CT]TTCC	4.8E-01	1.1E-04	3.2E-04	1.4E-02	1.1E-01	3.7E-03	2.4E-03	1.4E-01
ESRP1.TGGTGG	2.8E-01	1.6E-02	4.2E-02	1.6E-01	1.1E-01	4.8E-02	1.2E-03	1.1E-04
SAMD4A.GC[GT]GG[ACT][AC]	1.6E-01	8.6E-03	1.6E-02	7.6E-02	3.3E-01	1.4E-04	4.3E-02	1.5E-01
RBM5.GA[AG]GG[AT][AG]	6.8E-01	1.7E-04	2.3E-01	3.8E-01	5.8E-01	1.8E-02	7.5E-02	4.5E-01
SNRPA.[AT]TGCAC[AG]	1.0E+00	2.0E-04	1.3E-02	1.0E+00	1.0E+00	8.7E-02	4.1E-01	3.0E-01
SNRNP70.[AG][AT]TCAAG	1.0E+00	3.0E-01	9.9E-02	4.9E-02	3.1E-02	3.9E-01	2.0E-04	1.0E+00
ZNF638.[CGT]GTT[GC][GT]T	2.2E-04	1.2E-02	4.6E-02	1.0E+00	6.3E-01	1.0E-01	2.7E-01	2.3E-01
KHDRBS1.TAAAA[ACG][ACG]	1.7E-01	2.0E-01	1.2E-01	6.2E-01	2.7E-01	2.4E-04	6.4E-03	1.4E-01
SRp55.[CT][CT][AT]C[AT][GC]G	1.3E-03	5.6E-04	2.4E-04	4.1E-02	3.1E-03	6.0E-03	9.8E-02	4.3E-02
BRUNOL6.TGTG[AGT][GT]G	5.0E-01	2.1E-02	1.1E-02	6.2E-01	1.0E+00	2.8E-04	4.4E-03	4.2E-01
FXR2.[AGT]GAC[AG][AG][AG]	3.7E-04	4.1E-01	4.9E-02	4.9E-01	2.7E-01	1.3E-01	3.3E-03	6.1E-01
PABPN1.A[AG]AAGA	1.3E-01	4.5E-03	5.7E-03	6.0E-01	1.6E-01	3.7E-04	1.9E-01	6.5E-01
RBM46.[AG]AT[GC]A[AT][AGT]	5.3E-01	9.7E-04	1.2E-01	8.3E-01	8.9E-01	3.7E-04	4.4E-02	2.1E-02
TARDBP.GAATG[AGT]	3.8E-01	3.8E-04	6.9E-03	4.3E-01	5.0E-01	5.1E-03	3.6E-03	2.8E-01
HNRNPK.CCA[AT][AC]CC	3.9E-03	6.8E-02	1.4E-02	1.0E+00	1.0E+00	4.8E-03	7.9E-02	3.8E-04
PCBP1.CC[AT][AT][ACT]CC	6.6E-02	2.0E-03	3.8E-04	8.2E-01	8.2E-01	9.0E-02	1.3E-01	5.1E-02
SFPQ.[GT]T[AG][AG]T[GT][GT]	9.7E-02	5.5E-02	4.6E-04	4.5E-01	8.9E-01	1.5E-03	1.0E-03	1.9E-01
PCBP3.TTT[CT]CC	8.4E-01	2.3E-01	4.6E-04	4.3E-01	4.2E-01	3.2E-02	6.1E-02	8.3E-01
HuR.TTT[AG][GT]TT	6.6E-01	6.8E-02	5.7E-04	1.0E+00	5.2E-01	3.1E-03	1.5E-01	1.5E-01
SRSF1.AGGA[GC][AC]	2.9E-03	8.2E-02	1.1E-01	2.9E-01	5.4E-01	6.0E-04	9.8E-03	9.3E-02
SRSF10.AGAGA[ACG][AC]	6.1E-01	9.9E-02	3.8E-01	1.0E+00	7.4E-01	3.0E-02	7.1E-02	6.9E-04
SRSF9.A[GT]GA[ACG][AC][AG]	1.1E-02	3.1E-02	4.2E-01	4.6E-01	6.1E-01	1.4E-01	7.2E-04	1.6E-01
HuR.TTTTTT[GT]	1.0E+00	4.9E-02	7.6E-03	1.0E+00	3.5E-01	1.2E-01	7.7E-04	3.2E-01
IGF2BP3.A[AC]A[ACT][AT]CA	1.2E-03	1.0E-03	1.6E-02	8.8E-01	5.3E-01	3.4E-03	9.3E-04	7.1E-03
SRp20.CTC[GT]TC[CT]	6.8E-01	1.2E-02	9.9E-04	1.0E+00	1.0E+00	3.0E-01	1.7E-01	7.0E-01
PTBP1.[ACT][CT]TTT[CT]T	1.2E-03	1.8E-03	1.5E-03	2.7E-01	4.2E-01	1.5E-02	1.7E-01	6.3E-01
RALY.TTTTTT[CGT]	6.2E-01	3.7E-02	3.0E-03	3.7E-01	1.4E-01	1.1E-02	1.3E-03	4.6E-01
PABPC3.[AG]AAAAC[AC]	1.0E+00	1.1E-02	9.1E-03	1.0E+00	4.5E-01	1.6E-03	1.3E-03	1.1E-01
U2AF2.TTTTT[CT]C	5.6E-01	4.1E-03	1.5E-03	1.2E-01	1.0E-01	2.4E-03	7.7E-02	1.0E+00
RBM5.[GC]AAGG[AG]G	7.7E-01	5.7E-02	2.1E-01	6.1E-01	1.3E-01	3.4E-02	1.5E-03	8.3E-01
TIA1.TTTTT[CGT][GT]	4.6E-02	7.9E-02	1.6E-03	1.9E-01	3.4E-01	5.4E-02	1.0E-02	7.0E-01
SRSF9.[GT]G[AG][AT]G[GC][AC]	2.0E-01	3.5E-03	7.3E-03	2.7E-01	2.4E-01	1.8E-03	1.8E-02	3.9E-03
HNRNPC.[ACT]TTTTT[GT]	1.5E-01	8.1E-02	1.9E-03	2.2E-01	1.3E-01	1.0E-01	1.3E-02	3.0E-01
HNRNPCL1.[ACT]TTTTT[GT]	1.5E-01	8.1E-02	1.9E-03	2.2E-01	1.3E-01	1.0E-01	1.3E-02	3.0E-01
SRSF2.GGAG[AT][AGT]	1.0E-01	4.0E-03	1.2E-01	7.1E-02	1.3E-01	2.5E-01	2.1E-03	8.0E-01
TIA1.[AT]TTTTT[CGT]	7.3E-01	1.2E-01	2.3E-03	6.1E-01	3.7E-01	4.4E-03	1.2E-02	3.0E-01
9G8.[AT]GGAC[AG]A	5.1E-01	9.9E-02	3.9E-01	1.0E+00	1.0E+00	2.3E-03	1.1E-01	5.5E-01
CPEB4.TTTTTT	3.0E-01	6.0E-02	2.3E-03	4.5E-01	1.9E-01	6.3E-03	7.8E-03	2.1E-01

HuR.TT[AGT]TTTT	6.0E-01	9.4E-03	1.1E-02	1.0E+00	5.3E-01	2.6E-03	1.6E-01	1.8E-01
SRSF1.GG[AG]GGA[ACG]	8.6E-03	7.2E-02	4.7E-02	6.0E-03	2.6E-03	3.8E-02	5.5E-01	9.1E-02
MATR3.[AC]ATCTT[AG]	4.6E-01	6.8E-03	9.9E-02	1.0E+00	3.6E-01	2.6E-03	5.7E-03	1.0E+00
RBM6.[ACT]ATCCA[AG]	4.2E-01	2.7E-03	4.7E-02	1.0E+00	7.5E-01	1.9E-02	6.7E-01	5.1E-01
PABPC4.AAAAAA[AG]	1.2E-01	5.9E-01	1.6E-01	3.0E-02	1.0E+00	6.1E-02	3.0E-03	4.3E-01
ESRP2.TGGG[AG]A[AGT]	2.1E-01	3.1E-03	5.2E-02	4.4E-02	2.1E-01	1.3E-01	3.0E-01	1.8E-01
MBNL1.GCTTGC	1.0E+00	4.6E-01	1.5E-01	4.0E-01	3.3E-01	3.9E-03	2.0E-01	4.8E-01
SRSF1.GGAGGA	5.5E-02	5.1E-02	2.0E-01	4.3E-03	7.6E-02	6.1E-02	4.3E-01	6.7E-02
CPEB2.C[ACT]TTTTT	1.0E+00	2.1E-02	7.8E-03	3.2E-02	2.6E-02	4.4E-03	5.6E-03	4.8E-01
HNRNPA1.[AGT]TAGGG[AT]	1.0E+00	5.9E-01	3.2E-01	3.0E-02	2.9E-01	5.5E-01	3.0E-01	4.8E-03
HNRNPA1L2.[AGT]TAGGG[AT]	1.0E+00	5.9E-01	3.2E-01	3.0E-02	2.9E-01	5.5E-01	3.0E-01	4.8E-03
HNRNPA2B1.[AGT]TAGGG[AT]	1.0E+00	5.9E-01	3.2E-01	3.0E-02	2.9E-01	5.5E-01	3.0E-01	4.8E-03
HNRNPH2.GGGAGGG	1.0E+00	4.8E-03	6.5E-01	3.3E-02	1.4E-01	3.8E-02	3.3E-01	2.3E-01
PPRC1.[GC][GC]GCGC[GC]	1.0E+00	8.7E-01	5.3E-01	2.6E-01	5.1E-03	9.9E-02	8.5E-01	6.1E-01
RBM41.[AT]TAC[AT]T[GT]	2.8E-01	2.1E-01	9.4E-03	1.0E+00	1.0E+00	9.4E-02	1.8E-01	5.7E-03
YBX1.AACATC[AGT]	1.7E-01	1.4E-02	4.3E-01	3.1E-01	3.5E-01	4.4E-01	5.8E-03	4.0E-01
RBM3.[AG]A[AGT]AC[GT]A	1.0E+00	6.5E-03	7.8E-01	1.0E+00	5.1E-01	7.0E-02	5.9E-03	5.3E-01
PABPC5.AGAAA[AGT]T	2.2E-01	4.8E-01	5.9E-01	1.0E+00	4.9E-01	2.7E-02	7.9E-02	6.1E-03
HuR.TT[GT][AG]TTT	6.6E-03	2.0E-01	7.4E-03	1.0E+00	1.0E+00	3.0E-02	1.5E-01	6.7E-01
YBX2.AACA[AT]C[AGT]	1.4E-02	1.4E-02	5.0E-02	2.3E-01	1.9E-01	1.9E-02	6.6E-03	6.4E-01
FXR1.A[CT]GAC[AG]	8.0E-03	2.9E-01	4.5E-02	1.0E+00	1.0E+00	2.9E-02	1.6E-01	8.0E-01
DAZAP1.TAG[GT][AT][AT][AG]	7.4E-01	2.3E-02	1.2E-02	5.4E-01	5.5E-01	8.3E-03	1.0E-01	5.9E-01
Tra2-beta.GAAAGAA	6.2E-02	8.6E-03	3.8E-01	1.0E+00	1.0E+00	1.7E-02	1.2E-01	5.8E-02
YBX1.AACATC	7.2E-02	2.6E-02	5.4E-01	4.0E-01	4.5E-01	4.8E-02	8.6E-03	5.1E-01
FMR1.[GT]GACA[AG]G	6.5E-01	1.5E-01	3.2E-01	2.7E-01	2.0E-01	1.7E-01	8.9E-03	7.4E-01
SRSF1.G[AG]AGGA	5.8E-02	9.2E-03	1.4E-01	1.6E-02	7.1E-02	3.0E-02	8.6E-02	8.5E-02
SRSF10.AGAGA[AG][AG]	1.0E+00	2.7E-02	2.8E-01	1.0E+00	7.5E-01	9.2E-03	2.3E-01	3.9E-02
SRSF10.AGAGA[ACG][ACG]	8.3E-01	7.7E-02	4.1E-01	1.0E+00	8.9E-01	3.5E-02	7.3E-02	9.7E-03
RBFOX1.[AT]GCATG[AC]	1.0E+00	9.4E-02	1.1E-02	1.4E-02	3.5E-01	4.6E-02	5.9E-02	5.6E-01
ZC3H14.TTT[AGT]TTT	6.0E-01	1.4E-02	1.2E-02	1.0E+00	5.1E-01	2.2E-02	2.1E-01	5.7E-01
LIN28A.[ACT]GGAG[AT]A	6.2E-01	1.3E-02	5.1E-01	9.9E-02	7.2E-02	2.6E-01	4.7E-02	1.0E+00
RBM47.GATGA[AT]	1.9E-01	2.2E-02	2.5E-02	1.7E-01	1.5E-01	1.5E-02	3.3E-01	1.7E-01
A1CF.[AT]TAATT[AG]	2.7E-01	4.7E-02	1.8E-02	1.0E+00	1.0E+00	2.2E-02	4.1E-01	3.0E-01
QKI.ACTAAC[ACG]	1.0E+00	2.2E-02	1.8E-02	1.0E+00	1.0E+00	2.1E-01	2.9E-01	1.0E+00
G3BP2.AGGAT[AGT][AG]	1.4E-01	4.6E-02	7.5E-01	1.0E+00	1.0E+00	9.1E-02	2.1E-01	2.0E-02
CNOT4.GACAGA	2.5E-02	2.8E-01	3.5E-01	6.0E-01	5.1E-01	6.9E-01	1.4E-01	6.1E-01
SRSF10.A[AG]AG[AG][AG][AG]	9.1E-02	8.1E-02	6.8E-02	4.1E-01	7.1E-01	1.5E-01	2.8E-02	3.9E-01
HuR.TT[AT]GTTT	3.9E-01	1.6E-01	5.5E-02	1.0E+00	1.0E+00	3.2E-02	9.9E-02	3.2E-01
SRSF1.GGA[GC]G[AG][ACG]	1.9E-01	9.4E-02	1.6E-01	4.7E-02	8.3E-02	7.7E-02	3.5E-01	6.3E-02
TUT1.[AC][AG]ATACT	1.0E+00	1.8E-01	2.0E-01	1.0E+00	1.0E+00	5.0E-02	4.9E-01	1.0E+00
LIN28A.[CT]GGAGG[AG]	9.5E-02	3.8E-01	5.8E-02	6.2E-01	6.3E-01	6.7E-02	7.5E-01	3.1E-01
ZC3H10.[GC][GC]AGCG[AC]	1.7E-01	1.1E-01	2.1E-01	4.4E-01	1.2E-01	6.0E-02	3.4E-01	1.0E-01
MSI1.TAG[GT][AT][AG]G	4.6E-01	3.2E-01	2.1E-01	3.5E-01	1.0E+00	6.6E-02	4.5E-01	1.0E+00
MSI1.TAGT[AT][AG]G	1.5E-01	3.2E-01	1.1E-01	1.0E+00	1.0E+00	6.9E-02	5.2E-01	1.0E+00
RBM42.AACTA[AC]G	8.4E-02	1.5E-01	1.5E-01	1.0E+00	1.0E+00	1.0E+00	1.7E-01	1.2E-01
RBM8A.[AG][CT]GCGC[CGT]	1.0E+00	9.7E-02	4.0E-01	2.6E-01	2.1E-01	3.2E-01	6.5E-01	5.3E-01
SRSF7.ACGACG	1.0E+00	1.0E+00	1.0E+00	1.0E+00	1.0E+00	1.0E+00	1.0E+00	2.2E-01
RBM4.GCGCG[GC][GC]	1.0E+00	6.6E-01	1.0E+00	1.0E+00	1.0E+00	1.0E+00	1.0E+00	1.0E+00
RBM4.GCGCG[GC]G	1.0E+00	1.0E+00	1.0E+00	1.0E+00	1.0E+00	1.0E+00	1.0E+00	1.0E+00

The 50bp of exonic region and the 250bp of the intronic region were analyzed in upstream and downstream of the input exons.
The rMAPS program provided the p-values of Wilcoxon's rank sum test for each sliding window between downregulated versus control exons.

Supplementary Table 5. List of 216 ES events with ASpedia annotation.

Gene Symbol	as_id	exon	chr	location	strand	as_description_id	gene_name	locus_group	gene_id	transcript_id	exon_inclusion_transcript_id	exon_exclusion_transcript_id	GO_BP	GO_CC	GO_MF	conservation_score
NUMA1	17515	18	chr11	11q13.4	-	ENSG0000	nuclear mit	protein-co	ENSG0000	ENST00000	ENST00000	ENST00000	M PHASE O	CYTOSKELE	STRUCTUR	hg19.100w
LSR	49086	5	chr19	19q13.12	+	ENSG0000	lipolysis sti	protein-co	ENSG0000	ENST00000	ENST00000	ENST00000	ORGAN DE	MEMBRANE;PLASMA		hg19.100w
TUBB3	38175	6.1:6.2	chr16	16q24.3	+	ENSG0000	tubulin bet	protein-co	ENSG0000	ENST00000	ENST00000	ENST00000555609				hg19.100w
KIAA1217	11009	12	chr10	10p12.2-p1	+	ENSG0000	KIAA1217	protein-co	ENSG0000	ENST00000	ENST00000	ENST00000	ORGAN DE	CYTOPLASM		hg19.100w
ARPC1B	80609	8	chr7	7q22.1	+	ENSG0000	actin relate	protein-co	ENSG0000	ENST00000	ENST00000	ENST00000432343		PROTEIN C	STRUCTUR	hg19.100w
RAC1	78720	4	chr7	7p22.1	+	ENSG0000	ras-related	protein-co	ENSG0000	ENST00000	ENST00000	ENST00000	RESPONSE TO EXTERN		PYROPHOS	hg19.100w
CACNB3	21476	9	chr12	12q13.12	+	ENSG0000	calcium vol	protein-co	ENSG0000	ENST00000	ENST00000	ENST00000	ION TRANS	PLASMA M	VOLTAGE G	hg19.100w
SSBP3	3144	7	chr1	1p32.3	-	ENSG0000	single stran	protein-co	ENSG0000	ENST00000	ENST00000	ENST00000371320,ENST00000417664,ENST0				hg19.100w
DYSF	53937	19	chr2	2p13.2	+	ENSG0000	dysferlin	protein-co	ENSG0000	ENST00000	ENST00000	ENST00000	SYSTEM PR	PLASMA MEMBRANE;		hg19.100w
LUC7L	32844	4	chr16	16p13.3	-	ENSG0000	LUC7 like	protein-co	ENSG0000	ENST00000	ENST00000	ENST00000418978,ENST00000443357				hg19.100w
CADM1	18851	10	chr11	11q23.3	-	ENSG0000	cell adhesi	protein-co	ENSG0000	ENST00000	ENST00000	ENST00000	POSITIVE R	PLASMA M	PROTEIN D	hg19.100w
SSBP4	48431	7	chr19	19p13.1	+	ENSG0000	single stran	protein-co	ENSG0000	ENST00000	ENST00000	ENST00000270061,ENST00000600628				hg19.100w
TBC1D15	23415	10	chr12	12q21.1	+	ENSG0000	TBC1 doma	protein-co	ENSG0000	ENST00000	ENST00000	ENST00000550746				hg19.100w
ITGB4	43489	35	chr17	17q25.1	+	ENSG0000	integrin su	protein-co	ENSG0000	ENST00000	ENST00000	ENST00000339591,EN		PLASMA MEMBRANE;P		hg19.100w
PCYT2	44230	7	chr17	17q25.3	-	ENSG0000	phosphate	protein-co	ENSG0000	ENST00000	ENST00000	ENST00000	PHOSPHOLIPID METAB		TRANSFER	hg19.100w
EXOC7	43569	7	chr17	17q25.1	-	ENSG0000	exocyst co	protein-co	ENSG0000	ENST00000	ENST00000	ENST00000405575,ENST00000607838				hg19.100w
SH3BP1	62140	16	chr22	22q13.1	+	ENSG0000	SH3 domai	protein-co	ENSG0000	ENST00000	ENST00000	ENST00000442465				hg19.100w
MROH1	85546	26	chr8	8q24.3	+	ENSG0000	maestro he	protein-co	ENSG0000	ENST00000	ENST00000	ENST00000326134,ENST00000398656,ENST0				hg19.100w
ARHGAP17	35664	18	chr16	16p12.1	-	ENSG0000	Rho GTPas	protein-co	ENSG0000	ENST00000	ENST00000	ENST00000289968,ENST00000441763,ENST0				hg19.100w
OSBPL3	79027	9	chr7	7p15.3	-	ENSG0000	oxysterol b	protein-co	ENSG0000	ENST00000	ENST00000	ENST00000313367,ENST00000353930,ENST0				hg19.100w
UNC5B	12054	8	chr10	10q22.1	+	ENSG0000	unc-5 netri	protein-co	ENSG0000	ENST00000	ENST00000	ENST00000335350				hg19.100w
CCNDBP1	30220	10.1	chr15	15q15.2	+	ENSG0000	cyclin D1 b	protein-co	ENSG0000	ENST00000	ENST00000	ENST00000566515		NUCLEUS		hg19.100w
ABLIM1	13209	16	chr10	10q25.3	-	ENSG0000	actin bindi	protein-co	ENSG0000	ENST00000	ENST00000	ENST00000	SYSTEM PR	INTRACELL	ACTIN BIN	hg19.100w
DYSF	53936	43	chr2	2p13.2	+	ENSG0000	dysferlin	protein-co	ENSG0000	ENST00000	ENST00000	ENST00000	SYSTEM PR	PLASMA MEMBRANE;		hg19.100w
PTK2	85305	39.2	chr8	8q24.3	-	ENSG0000	protein tyr	protein-co	ENSG0000	ENST00000	ENST00000	ENST00000	SIGNAL TR	INTRACELL	PROTEIN D	hg19.100w
IVNS1ABP	9218	9	chr1	1q25.3	-	ENSG0000	influenza v	protein-co	ENSG0000	ENST00000	ENST00000	ENST00000	RESPONSE	MEMBRANE	ENCLOSE	hg19.100w
GIT2	24370	19	chr12	12q24.11	-	ENSG0000	GIT ArfGAP	protein-co	ENSG0000	ENST00000	ENST00000	ENST00000	G PROTEIN	INTRACELLULAR ORGA		hg19.100w
ASAP2	52611	23	chr2	2p25.1	+	ENSG0000	ArfGAP wit	protein-co	ENSG0000	ENST00000	ENST00000	ENST00000281419				hg19.100w
SLAIN2	69214	8	chr4	4p11	+	ENSG0000	SLAIN moti	protein-co	ENSG0000	ENST00000	ENST00000	ENST00000510595,ENST00000512093				hg19.100w
MYO1B	56609	24	chr2	2q32.3	+	ENSG0000	myosin IB	protein-co	ENSG0000	ENST00000	ENST00000	ENST00000392316,ENST00000439065				hg19.100w
SCNN1A	19842	10	chr12	12p13	-	ENSG0000	sodium cha	protein-co	ENSG0000	ENST00000	ENST00000	ENST00000	SYSTEM PROCESS;ION TRANSPOR			hg19.100w
TMEM180	12954	6	chr10	10q24.32	+	ENSG0000	major facili	protein-co	ENSG0000	ENST00000	ENST00000	ENST00000238936				hg19.100w
SYNE1	78184	143	chr6	6q25.2	-	ENSG0000	spectrin re	protein-co	ENSG0000	ENST00000	ENST00000	ENST00000	ORGANELL	SYNAPSE;I	ACTIN BIN	hg19.100w
SRSF4	1424	6	chr1	1p35.3	-	ENSG0000	serine and	protein-co	ENSG0000	ENST00000	ENST00000	ENST00000	RNA META	NUCLEUS	RNA SPLICI	hg19.100w

Supplementar

Gene Svmbol	dbSNP_variant	COSMIC_variant	SPIDEX_variant	miRNA_binding_site	repeat.	NMD	COSMIC_NMD	dbSNP_NMD	protein_domain	protein_translational_modification	RBP_splicing_factor	RBP	tissue	as	isoform_PPI_a	isoform_PPI_b	isoform_subcellular_localization_id
NUMA1	rs2002420	chr11:7172	chr11:7172	NA			NA			Chain,1-21	FMR1,7.86	BUD13,3.0	brain:0.139;bonemarrow:0.219;pa				NA
LSR	rs1920913	chr19:3575	chr19:3574	NA			NA			Chain,1-64	FXR1,5.939	BCCIP,1.47	brain:-0.112;thyroid:0.075;stomac				NA
TUBB3	ay.phastCo	chr16:8999	chr16:8999	NA	RepeatMas	chr16:8999	NA		ENST00000	Chain,1-45	SRSF1,1.63	BUD13,2.1	bonemarrow:-0.319;spleen:-0.25				NA
KIAA1217	ay.phastCo	chr10:2476	chr10:2476	NA			NA		ENST00000376462,PF0		HNRNPA1,	AGGF1,8.6	brain:-0.19		ENST00000VPS52/FHL		NA
ARPC1B	rs150232928,chr7:989		chr7:98985	NA	RepeatMas	chr7:98986	NA			Chain,2-37	FMR1,9.92	BUD13,5.146e-19,chr7:98987526-98987616,9					NA
RAC1	rs140559382,chr7:643		chr7:64315	NA			NA		ENST00000	Chain,1-18	HNRNPA1,	BUD13,4.9	thyroid:-0.152;stomach:-0.066;pa				NA
CACNB3	ay.phastCo	chr12:4921	chr12:4921	NA			NA			Chain,1-484,chr12:49212713-4922			brain:-0.303;thyroid:0.12;stomach				NA
SSBP3	ay.phastCons:(E)0.824/		chr1:54722	NA			NA		ENST00000	Chain,1-38	FMR1,4.96	AGGF1,7.5	brain:0.07;		ENST00000SSBP3/LDB		NA
DYSF	rs1414000	chr2:71766	chr2:71766	NA			NA			Chain,1-2080,chr2:716		CREBBP,8.8	brain:-0.05		ENST00000CAV3/CAP		NA
LUC7L	ay.phastCo	chr16:2580	chr16:2580	NA			NA			Chain,1-37	HNRNPC,8.	AGGF1,9.1	brain:0.147;thyroid:-0.274;stomac				NA
CADM1	ay.phastCons:(E)0.543/		chr11:1150	NA			NA			Chain,45-4	HNRNPA1,	DDX55,1.0	brain:-0.395;thyroid:0.167;stomac				NA
SSBP4	ay.phastCo	chr19:1854	chr19:1854	NA			NA			Chain,1-38	HNRNPK,1.	AGGF1,2.2	brain:0.396;thyroid:0.321;stomach				NA
TBC1D15	rs3730116	chr12:7228	chr12:7228	NA			NA		ENST00000	Chain,2-69	IGF2BP1,3.	AKAP8L,2.3	thyroid:-0.395;stomach:-0.285;pa				NA
ITGB4	rs3680893	chr17:7375	chr17:7375	NA			NA			Chain,28-1	IGF2BP1,9.	CSTF2T,2.6	brain:-0.065;thyroid:0.391;stomac				NA
PCYT2	ay.phastCo	chr17:7986	chr17:7986	NA			NA			Chain,1-38	RBFOX2,5.	BUD13,7.3	brain:0.12;thyroid:-0.101;stomach				NA
EXOC7	ay.phastCo	chr17:7408	chr17:7408	NA			NA				HNRNPA1,	BUD13,3.7	brain:-0.13		ENST00000GRIN2B/AK		NA
SH3BP1	ay.phastCons:(E)0.369/		chr22:3804	NA	RepeatMasker,SINE,ch		NA			Chain,1-701,chr22:380		BUD13,1.792e-07,chr22:38044335-38044378,					NA
MROH1	ay.phastCons:(E)0.597/		chr8:14529	NA			NA			Chain,1-1641,chr8:145218730-145316640							NA
ARHGAP17	rs200789627,chr16:24		chr16:2494	NA			NA			Chain,1-88	HNRNPC,8.	BUD13,9.8	brain:-0.20		ENST00000ABL1/LCK/		NA
OSBPL3	rs1143397	chr7:24903	chr7:24901	NA			NA			Chain,1-88	U2AF1,6.13	BUD13,1.4	brain:-0.24		ENST00000NEDD8/YW		NA
UNC5B	rs3743693	chr10:7304	chr10:7304	NA			NA			Chain,27-9	IGF2BP1,1.	DNMT1,2.7	brain:-0.156;stomach:-0.247;bone				NA
CCNDBP1	ay.phastCons:(E)0.204/		chr15:4348	NA		chr15:4348	NA			Chain,2-36	IGF2BP1,8.	BUD13,2.7	brain:-0.078;thyroid:-0.095;smalli				NA
ABLIM1	ay.phastCons:(E)0.588/		chr10:1162	NA			NA			Chain,1-77	HNRNPK,5.	BUD13,1.4	brain:-0.38		ENST00000VIM/USHB		NA
DYSF	rs61746816,chr2:7184		chr2:71840	NA			NA			Chain,1-2080,chr2:71681129-7191			thyroid:-0.		ENST00000CAV3/CAP		NA
PTK2	ay.phastCons:(E)0.252/		chr8:14167	NA			NA			Chain,2-10	FMR1,1.14	BUD13,1.1	brain:-0.312;thyroid:-0.117;stoma				NA
IVNS1ABP	ay.phastCo	chr1:18527	chr1:18527	NA			NA		ENST00000	Chain,1-64	HNRNPC,1.	AGGF1,2.3	brain:0.152;thyroid:0.119;stomach				NA
GIT2	rs372157894,chr12:11		chr12:1103	NA			NA			Chain,1-75	HNRNPC,3.	BUD13,7.7	brain:-0.075;thyroid:-0.238;bonem				NA
ASAP2	ay.phastCons:(E)0.685/		chr2:95284	NA			NA			Chain,1-10	HNRNPC,7.	GRWD1,1.2	thyroid:-0.		ENST00000CTTN/SRC/		NA
SLAIN2	ay.phastCons:(E)0.713/		chr4:48385	NA	SelfChain,chr4:483941		NA		ENST00000	Chain,1-58	ELAVL1,1.8	AGGF1,1.9	thyroid:-0.193;stomach:-0.216;bo				NA
MYO1B	ay.phastCons:(E)0.256/		chr2:19226	NA			NA			Chain,1-11	HNRNPC,2.	AGGF1,3.7	brain:-0.24;stomach:0.207;bonem				NA
SCNN1A	rs7265755	chr12:6463	chr12:6458	NA	Interrupted Rpts,chr12		NA		ENST00000	Chain,1-669,chr12:6457042-64839			brain:0.5;t		ENST00000USP8/USP1		NA
TMEM180	rs1408975	chr10:1042	chr10:1042	NA			NA		ENST00000	Chain,1-51	RBM15,2.1	PRPF8,4.81	brain:-0.507;thyroid:-0.134;stoma				NA
SYNE1	rs144685965,chr6:152		chr6:15246	NA			NA			Chain,1-8797,chr6:152443574-152			brain:0.154;thyroid:0.141;stomach				NA
SRSF4	rs34597676,chr1:2948		chr1:29485	NA			NA			Chain,1-49	FMR1,4.75	BUD13,3.8	thyroid:-0.074;skin:-0.059;prostat				NA

Supplementar

Gene Symbol	isoform_subcellular_localization	tds.corr	tds.pval	erk.corr	erk.pval	ras.corr	ras.pval	ETE.pval	Lymph_node_metastasis.pval
NUMA1	NA	-0.54	7.2E-40	0.50	8.6E-33	-0.62	3.8E-52	7.9E-10	4.2E-09
LSR	NA	0.53	6.3E-37	-0.59	4.9E-49	0.49	8.7E-30	2.5E-11	5.0E-11
TUBB3	NA	0.52	1.2E-32	-0.45	8.0E-24	0.62	3.3E-47	4.5E-05	7.6E-07
KIAA1217	NA	0.58	2.8E-46	-0.29	2.6E-11	0.55	2.3E-38	1.0E-06	4.2E-07
ARPC1B	NA	0.47	1.2E-28	-0.52	1.2E-36	0.58	5.6E-45	3.4E-06	6.3E-05
RAC1	NA	-0.42	4.9E-23	0.35	7.7E-16	-0.58	1.9E-44	5.8E-02	1.4E-03
CACNB3	NA	-0.58	2.6E-44	0.32	3.7E-13	-0.55	2.5E-38	1.3E-04	5.0E-11
SSBP3	NA	0.57	1.9E-43	-0.39	7.4E-20	0.41	7.7E-21	3.8E-10	4.0E-09
DYSF	NA	-0.54	2.6E-33	0.41	1.8E-18	-0.61	1.1E-42	3.6E-07	3.2E-11
LUC7L	NA	0.38	6.9E-19	-0.55	9.3E-41	0.40	1.7E-19	4.1E-06	1.2E-04
CADM1	NA	0.44	1.1E-25	-0.45	3.1E-26	0.55	3.0E-39	4.1E-10	6.7E-08
SSBP4	NA	-0.53	1.6E-38	0.42	8.8E-23	-0.50	6.5E-32	7.6E-08	4.0E-11
TBC1D15	NA	0.54	2.6E-38	-0.41	1.3E-20	0.46	9.5E-26	8.9E-06	1.1E-05
ITGB4	NA	0.52	3.7E-36	-0.37	1.4E-17	0.52	2.0E-34	7.2E-06	4.6E-11
PCYT2	NA	-0.47	3.2E-29	0.51	3.3E-35	-0.37	1.5E-16	3.8E-08	1.4E-08
EXOC7	NA	0.41	1.7E-21	-0.50	2.0E-33	0.52	4.7E-35	1.4E-06	1.5E-03
SH3BP1	NA	0.48	8.8E-30	-0.47	2.1E-29	0.52	2.9E-34	2.1E-03	1.5E-04
MROH1	NA	-0.50	4.7E-33	0.32	1.5E-13	-0.52	3.1E-34	3.1E-07	4.3E-06
ARHGAP17	NA	0.35	3.1E-16	-0.51	3.8E-34	0.38	1.4E-17	1.7E-03	2.2E-04
OSBPL3	NA	0.52	3.9E-34	-0.45	1.0E-25	0.51	1.3E-31	5.3E-07	1.5E-09
UNC5B	NA	-0.52	4.0E-31	0.43	1.4E-20	-0.55	1.1E-33	6.8E-05	3.8E-04
CCNDBP1	NA	0.47	1.9E-28	-0.38	7.9E-19	0.50	7.1E-32	5.2E-04	4.0E-08
ABLIM1	NA	0.50	2.6E-31	-0.37	5.3E-17	0.42	5.2E-20	2.7E-05	5.0E-03
DYSF	NA	-0.44	3.4E-25	0.33	1.4E-13	-0.50	6.8E-31	2.0E-04	1.6E-04
PTK2	NA	0.48	9.7E-30	-0.29	5.6E-11	0.46	6.6E-26	1.4E-04	1.0E-03
IVNS1ABP	NA	0.34	2.2E-15	-0.47	2.9E-29	0.39	5.7E-19	7.6E-05	1.5E-03
GIT2	NA	-0.30	9.7E-12	0.25	2.2E-08	-0.48	5.0E-29	8.9E-03	1.2E-02
ASAP2	NA	-0.36	8.7E-17	0.22	4.6E-07	-0.48	5.9E-29	4.3E-05	1.2E-05
SLAIN2	NA	-0.40	1.4E-20	0.42	5.6E-23	-0.48	1.6E-28	3.0E-06	1.6E-04
MYO1B	NA	0.31	3.6E-12	-0.34	4.0E-15	0.48	1.8E-28	2.9E-02	1.2E-06
SCNN1A	NA	0.18	4.0E-05	-0.41	4.7E-22	0.48	4.7E-28	9.8E-05	1.5E-07
TMEM180	NA	0.46	8.9E-28	-0.46	6.3E-28	0.42	1.3E-21	4.7E-07	6.9E-04
SYNE1	NA	-0.47	8.5E-28	0.18	1.1E-04	-0.32	2.1E-12	6.9E-05	6.9E-05
SRSF4	NA	0.30	9.4E-12	-0.46	1.1E-27	0.42	3.5E-21	7.1E-04	1.1E-02

Gene Symbol	as_id	exon	chr	location	strand	as_description_id	gene_name	locus_group	gene_id	transcript_id	exon_inclusion_transcript_id	exon_exclusion_transcript_id	GO_BP	GO_CC	GO_MF	conservation_score
LCN12	127791	4	chr9	9q34.3	+	ENSG0000	lipocalin 12	protein-co	ENSG0000	ENST00000	ENST00000	ENST00000371633,ENST00000470501				hg19.100w
ACLY	40960	14	chr17	17q21.2	-	ENSG0000	ATP citrate	protein-co	ENSG0000	ENST00000	ENST00000	ENST00000	CELLULAR	PROTEIN C	TRANSFER	hg19.100w
BRD8	73509	10	chr5	5q31.2	-	ENSG0000	bromodom	protein-co	ENSG0000	ENST00000	ENST00000	ENST00000	REGULATION OF TRAN		TRANSCRIP	hg19.100w
SYTL2	18153	11.2	chr11	11q14.1	-	ENSG0000	synaptotag	protein-co	ENSG0000	ENST00000	ENST00000	ENST00000	VESICLE M	PLASMA MEMBRANE;P		hg19.100w
IGSF3	4366	6	chr1	1p13.1	-	ENSG0000	immunoglo	protein-co	ENSG0000	ENST00000	ENST00000	ENST00000318837,ENST00000369483				hg19.100w
GUSB	79855	6	chr7	7q11.21	-	ENSG0000	glucuronid	protein-co	ENSG0000	ENST00000	ENST00000	ENST00000	CARBOHYDRATE META		HYDROLAS	hg19.100w
FLNA	90565	30	chrX	Xq28	-	ENSG0000	filamin A	protein-co	ENSG0000	ENST00000	ENST00000	ENST00000	TRANSCRIP	INTRACELL	TRANSCRIP	hg19.100w
MAPK10	69826	9	chr4	4q21.3	-	ENSG0000	mitogen-ac	protein-co	ENSG0000	ENST00000	ENST00000	ENST00000	STRESS ACTIVATED PR		TRANSFER	hg19.100w
PTBP2	3832	12	chr1	1p21.3	+	ENSG0000	polypyrimi	protein-co	ENSG0000	ENST00000	ENST00000	ENST00000370197,ENST00000370198,ENST0				hg19.100w
GRB7	40698	16	chr17	17q12	+	ENSG0000	growth fac	protein-co	ENSG0000	ENST00000	ENST00000	ENST00000	TRANSMEMBRANE RE		MOLECULA	hg19.100w
SLC25A36	67052	6.1:6.2	chr3	3q23	+	ENSG0000	solute carri	protein-co	ENSG0000	ENST00000	ENST00000	ENST00000512506				hg19.100w
LGALS9	39858	5	chr17	17q11.2	+	ENSG0000	galectin 9	protein-co	ENSG0000	ENST00000	ENST00000	ENST00000	REGULATION OF I	KAP	SUGAR BIN	hg19.100w
DOCK9	26178	48	chr13	13q32.3	-	ENSG0000	dedicator o	protein-co	ENSG0000	ENST00000	ENST00000	ENST00000339416,ENST00000376460,ENST0				hg19.100w
DYSF	53938	7	chr2	2p13.2	+	ENSG0000	dysferlin	protein-co	ENSG0000	ENST00000	ENST00000	ENST00000	SYSTEM PR	PLASMA MEMBRANE;		hg19.100w
PPFIBP1	20893	11	chr12	12p11.23,	+	ENSG0000	PPFIA bind	protein-co	ENSG0000	ENST00000	ENST00000	ENST00000228425				hg19.100w
SYTL2	18148	13	chr11	11q14.1	-	ENSG0000	synaptotag	protein-co	ENSG0000	ENST00000	ENST00000	ENST00000	VESICLE M	PLASMA MEMBRANE;P		hg19.100w
DST	76560	106	chr6	6p12.1	-	ENSG0000	dystonin	protein-co	ENSG0000	ENST00000	ENST00000	ENST00000	CYTOSKELE	CYTOPLAS	PROTEIN C	hg19.100w
INCENP	16337	11	chr11	11q12.3	+	ENSG0000	inner centr	protein-co	ENSG0000	ENST00000	ENST00000	ENST00000	CELL DIVISI	INTRACELLULAR NON		hg19.100w
HAUS1	45389	3	chr18	18q21.1	+	ENSG0000	HAUS augm	protein-co	ENSG0000	ENST00000	ENST00000	ENST00000592206				hg19.100w
MAPK9	75012	3	chr5	5q35.3	-	ENSG0000	mitogen-ac	protein-co	ENSG0000	ENST00000	ENST00000	ENST00000	STRESS ACTIVATED PR		TRANSFER	hg19.100w
GEMIN2	27356	5	chr14	14q21.1	+	ENSG0000	gem nuclea	protein-co	ENSG0000	ENST00000	ENST00000	ENST00000	CELLULAR	MACROMO	RNA SPLICI	hg19.100w
FAM86C1	17441	3.2	chr11	11q13.4	+	ENSG0000	family with	protein-co	ENSG0000	ENST00000	ENST00000	ENST00000426628				hg19.100w
MAP3K4	78357	18	chr6	6q26	+	ENSG0000	mitogen-ac	protein-co	ENSG0000	ENST00000	ENST00000	ENST00000	REGULATION OF CATA		MAP KINAS	hg19.100w
ABI1	11032	12	chr10	10p12.1	-	ENSG0000	abl interact	protein-co	ENSG0000	ENST00000	ENST00000	ENST00000	CELL PROLI	SOLUBLE F	CYTOSKELE	hg19.100w
OPA1	68139	7	chr3	3q29	+	ENSG0000	OPA1, mito	protein-co	ENSG0000	ENST00000	ENST00000	ENST00000	TRANSPOR	ENVELOPE;	PYROPHOS	hg19.100w
MAP2	57224	16	chr2	2q34	+	ENSG0000	microtubul	protein-co	ENSG0000	ENST00000	ENST00000	ENST00000199940,EN		INTRACELLULAR NON		hg19.100w
CSF2RA	88369	13	chrX	Xp22.32 an	+	ENSG0000	colony stim	protein-co	ENSG0000	ENST00000	ENST00000	ENST00000417535,EN		PLASMA M	RECEPTOR	hg19.100w
BIN1	55200	13	chr2	2q14.3	-	ENSG0000	bridging int	protein-co	ENSG0000	ENST00000	ENST00000	ENST00000	CELL PROLI	INTRACELLULAR NON		hg19.100w
HIPK3	14893	14	chr11	11p13	+	ENSG0000	homeodom	protein-co	ENSG0000	ENST00000	ENST00000	ENST00000	NEGATIVE REGULATIO		TRANSFER	hg19.100w
PILRB	80935	10	chr7	7q22.1	+	ENSG0000	paired imm	protein-co	ENSG0000	ENST00000	ENST00000	ENST00000310771,ENST00000438028,ENST0				hg19.100w
LRRFIP2	63960	20	chr3	3p22.2	-	ENSG0000	LRR bindin	protein-co	ENSG0000	ENST00000	ENST00000	ENST00000336686,ENST00000416425,ENST0				hg19.100w
CAST	72863	8.2	chr5	5q15	+	ENSG0000	calpastatin	protein-co	ENSG0000	ENST00000	ENST00000	ENST00000338252,ENST00000341926,ENST0				hg19.100w
ZFAND1	84309	3	chr8	8q21.13	-	ENSG0000	zinc finger	protein-co	ENSG0000	ENST00000	ENST00000	ENST00000220669,ENST00000517353,ENST0				hg19.100w
KIF13A	75458	28	chr6	6p22.3	-	ENSG0000	kinesin fam	protein-co	ENSG0000	ENST00000	ENST00000	ENST00000259711,ENST00000358380,ENST0				hg19.100w
POLR2J2	81129	3.1	chr7	7q22.1	-	ENSG0000	RNA polym	protein-co	ENSG0000	ENST00000	ENST00000	ENST00000476151,ENST00000591000				hg19.100w
PRKDC	83791	81	chr8	8q11.21	-	ENSG0000	protein kin	protein-co	ENSG0000	ENST00000	ENST00000	ENST00000	CELLULAR	MACROMOLECULE	MET	hg19.100w

Gene Symbol	dbSNP_variant	COSMIC_variant	SPIDEX_variant	miRNA_binding_site	repeat.	NMD	COSMIC_NMD	dbSNP_NMD	protein_domain	protein_translational_modification	RBP_splicing_factor	RBP	tissue_as	isoform_PPI_a	isoform_PPI_b	isoform_subcellular_localization_id
LCN12	ay.phastCo	chr9:13984	chr9:13984	NA			NA		ENST00000	Chain,20-1	IGF2BP3,2.	CSTF2T,1.3	brain:-0.073;thyroid:0.319;testis:0			NA
ACLY	ay.phastCons:(E)0.756/		chr17:4004	NA			NA			Chain,1-11	FMR1,6.34	BUD13,3.8	brain:0.079;thyroid:0.113;stomach			NA
BRD8	rs3751438	chr5:13750	chr5:13750	NA			NA			Chain,1-12	IGF2BP1,8.	BUD13,3.6	brain:0.151	ENST00000	FSD2/MIS1	NA
SYTL2	rs1414769	chr11:8542	chr11:8542	NA			NA			Chain,1-934,chr11:85406244-8546			stomach:0.	ENST00000	PPP1CA	NA
IGSF3	rs1997357	chr1:11715	chr1:11714	NA	RepeatMasker,LINE,ch		NA			Chain,20-1194,chr1:11		DNMT1,2.9	thyroid:-0.162;stomach:-0.371;bo			NA
GUSB	rs367551992,chr7:654		chr7:65439	NA	SelfChain,chr7:654399		NA		ENST00000	Chain,23-6	HNRNPM,9	AGGF1,2.3	bonemarrow:0.104;prostate:0.057			NA
FLNA	rs387907371,chrX:153		chrX:15358	NA			NA		ENST00000	Chain,2-26	FMR1,2.17	BUD13,3.4	brain:-0.21	ENST00000	TRIO/ZNF6	NA
MAPK10	rs2009897	chr4:87024	chr4:87023	NA			NA		ENST00000	Chain,1-464,chr4:86938384-87115			brain:-0.22	ENST00000	RELA/STM	NA
PTBP2	rs2016355	chr1:97272	chr1:97270	NA			NA		ENST00000	Chain,1-53	ELAVL1,1.0	BUD13,1.116e-09,chr1:97270347-97270373,2				NA
GRB7	rs3717526	chr17:3790	chr17:3790	NA			NA		ENST00000	Chain,1-53	IGF2BP3,1.	BUD13,3.9	thyroid:-0.	ENST00000	LAX1/PKM	NA
SLC25A36	ay.phastCons:(E)0.313/		chr3:14068	NA		chr3:14068	NA		ENST00000	Chain,1-31	ELAVL1,1.0	BUD13,9.7	brain:-0.057;thyroid:-0.072;stoma			NA
LGALS9	rs2074763	chr17:2597	chr17:2596	NA			NA			Chain,1-35	HNRNPK,2.962e-10,ch		brain:0.114;thyroid:0.163;stomach			NA
DOCK9	ay.phastCons:(E)0.739/		chr13:9947	NA			NA					BUD13,6.1	thyroid:-0.101;stomach:-0.184;bo			NA
DYSF	rs7289889	chr2:71740	chr2:71738	NA			NA			Chain,1-2080,chr2:71681129-7191			brain:0.079	ENST00000	CAV3/CAP	NA
PPFIBP1	rs1389742	chr12:2780	chr12:2780	NA			NA			Chain,1-1011,chr12:27		GRWD1,3.5	brain:0.263;thyroid:0.374;stomach			NA
SYTL2	rs1996326	chr11:8542	chr11:8542	NA			NA			Chain,1-934,chr11:85406244-8546			brain:-0.18	ENST00000	PPP1CA;PP	NA
DST	ay.phastCons:(E)0.189/		chr6:56324	NA			NA				IGF2BP1,3.	AGGF1,7.6	brain:0.263	ENST00000	ORF35/OR	NA
INCENP	ay.phastCo	chr11:6190	chr11:6190	NA			NA			Chain,1-91	FMR1,8.96	BUD13,2.6	brain:0.236	ENST00000	AURKC/CB	NA
HAUS1	ay.phastCons:(E)0.289/		chr18:4368	NA	RepeatMas	chr18:4368	NA			Chain,1-27	IGF2BP1,2.	CHD4,1.860e-05,chr18:43685311-43685334,2				NA
MAPK9	ay.phastCons:(E)0.816/		chr5:17969	NA			NA		ENST00000	Chain,1-42	HNRNPK,9.	DNMT1,1.1	brain:0.108	ENST00000	JUN;IRS1/N	NA
GEMIN2	ay.phastCo	chr14:3959	chr14:3958	NA	Interrupted	chr14:3959	NA			Chain,1-28	SRSF1,5.42	AGGF1,1.481e-13,chr1		ENST00000	SRSF3/GAR	NA
FAM86C1	rs1465050	chr11:7150	chr11:7150	NA			NA		ENST00000	Chain,1-16	IGF2BP1,1.	CBX3,2.720	thyroid:0.391;stomach:0.344;skin:			NA
MAP3K4	ay.phastCons:(E)0.247/		chr6:16151	NA	RepeatMasker,Simple_		NA			Chain,1-16	HNRNPM,1	BUD13,9.325e-16,chr6:161518113-16151814				NA
ABI1	rs1483525	chr10:2704	chr10:2704	NA			NA				ELAVL1,1.7	BUD13,6.954e-08,chr1		ENST00000	EPS8/SPTA	NA
OPA1	rs112830022,chr3:193		chr3:19333	NA			NA			Chain,88-9	ELAVL1,6.6	BUD13,6.0	brain:0.372;stomach:-0.072;bone			NA
MAP2	ay.phastCo	chr2:21057	chr2:21057	NA			NA			Chain,1-1827,chr2:210517895-210			brain:0.74;	ENST00000	SRC/TUBA1	NA
CSF2RA	ay.phastCo	chrX:14193	chrX:14193	NA	Interrupted Rpts,chrX:		NA		ENST00000	Chain,20-90,chrX:1401654-142875			brain:0.065;thyroid:-0.413;stomac			NA
BIN1	rs34647988,chr2:1278		chr2:12780	NA			NA						thyroid:0.158;stomach:0.279;bone			NA
HIPK3	ay.phastCo	chr11:3336	chr11:3336	NA			NA			Chain,1-12	FXR1,1.452	PRPF8,2.77	brain:0.076	ENST00000	C1QA/LIMK	NA
PILRB	ay.phastCons:(E)0.086/(I)0.058;pha			NA	RepeatMasker,Simple_		NA				HNRNPK,4.	BUD13,1.5	brain:0.188;stomach:0.069;bonem			NA
LRRFIP2	rs1419973,	chr3:37136	chr3:37125	NA			NA			Chain,1-72	FXR1,6.551	AGGF1,4.5	brain:-0.078;thyroid:0.45;stomach			NA
CAST	rs3704013	chr5:96062	chr5:96058	NA			NA			Chain,1-70	IGF2BP1,2.	AGGF1,2.8	brain:-0.221;thyroid:0.078;stomac			NA
ZFAND1	rs5959334	chr8:82630	chr8:82627	NA			NA		ENST00000	Chain,1-26	HNRNPA1,	BUD13,4.0	brain:0.146;thyroid:0.267;stomach			NA
KIF13A	ay.phastCo	chr6:17794	chr6:17788	NA			NA		ENST00000	Chain,1-18	FMR1,5.77	BUD13,4.8	brain:0.761	ENST00000	ATF7IP/GT	NA
POLR2J2	ay.phastCons:(E)0.252/		chr7:10230	NA			NA		ENST00000	Chain,1-11	HNRNPH1,	CBX3,1.000	thyroid:0.218;stomach:0.441;bone			NA
PRKDC	ay.phastCons:(E)0.271/		chr8:48691	NA			NA		ENST00000	Phosphoryl	FMR1,7.23	BUD13,8.1	thyroid:0.0	ENST00000	AP1B1/HSP	NA

Gene Symbol	isoform_subcellular_localization	tds.corr	tds.pval	erk.corr	erk.pval	ras.corr	ras.pval	ETE.pval	Lymph_node_metastasis.pval
LCN12	NA	0.32	1.6E-13	-0.46	1.1E-27	0.28	6.1E-10	7.5E-03	7.6E-02
ACLY	NA	-0.46	1.3E-27	0.34	2.3E-14	-0.46	5.3E-26	1.1E-02	9.3E-06
BRD8	NA	-0.46	2.7E-27	0.34	3.2E-15	-0.33	7.7E-14	8.6E-06	3.0E-03
SYTL2	NA	0.48	3.3E-25	-0.43	2.6E-20	0.50	3.8E-27	1.4E-04	4.0E-05
IGSF3	NA	0.49	4.6E-27	-0.26	6.8E-08	0.39	1.9E-16	1.0E-03	5.9E-05
GUSB	NA	-0.45	8.3E-27	0.25	1.3E-08	-0.28	6.6E-10	2.9E-04	3.8E-05
FLNA	NA	-0.45	1.2E-26	0.22	4.7E-07	-0.43	4.9E-23	1.1E-04	1.8E-03
MAPK10	NA	0.47	1.4E-26	-0.32	2.2E-12	0.40	1.6E-18	3.1E-01	2.7E-02
PTBP2	NA	0.48	1.5E-26	-0.28	1.7E-09	0.22	7.1E-06	9.1E-04	5.3E-04
GRB7	NA	-0.43	4.0E-24	0.27	1.4E-09	-0.46	4.3E-26	1.5E-05	3.9E-04
SLC25A36	NA	-0.46	1.1E-25	0.18	1.3E-04	-0.31	5.6E-11	4.2E-02	2.4E-04
LGALS9	NA	-0.45	1.2E-25	0.40	3.8E-20	-0.43	2.8E-22	1.8E-03	3.2E-06
DOCK9	NA	-0.37	9.7E-18	0.24	3.8E-08	-0.46	1.2E-25	6.3E-04	2.9E-05
DYSF	NA	0.39	1.2E-17	-0.33	1.4E-12	0.48	1.6E-25	3.6E-05	1.3E-03
PPFIBP1	NA	0.40	1.7E-18	-0.40	3.3E-18	0.46	9.1E-24	5.8E-05	1.4E-03
SYTL2	NA	-0.44	1.1E-23	0.38	6.6E-17	-0.44	6.5E-22	1.9E-02	1.5E-04
DST	NA	0.43	1.4E-23	-0.32	2.7E-13	0.31	5.8E-12	2.2E-09	2.4E-09
INCENP	NA	-0.44	3.3E-23	0.27	4.0E-09	-0.43	7.3E-21	1.1E-02	2.3E-05
HAUS1	NA	0.07	1.1E-01	-0.42	5.6E-23	0.22	1.5E-06	3.5E-02	2.5E-02
MAPK9	NA	0.21	2.4E-06	-0.43	5.8E-23	0.29	2.5E-10	1.5E-02	2.3E-03
GEMIN2	NA	0.15	7.9E-04	-0.42	8.1E-23	0.38	2.0E-17	8.3E-03	3.3E-03
FAM86C1	NA	-0.41	3.0E-22	0.18	5.9E-05	-0.24	6.5E-08	6.3E-04	4.9E-02
MAP3K4	NA	0.34	3.1E-15	-0.41	1.1E-21	0.37	5.3E-17	1.0E-03	6.0E-04
ABI1	NA	0.41	2.5E-21	-0.29	1.7E-11	0.21	3.0E-06	1.8E-06	2.5E-08
OPA1	NA	0.42	3.2E-21	-0.29	2.0E-10	0.42	1.1E-20	1.6E-04	1.6E-05
MAP2	NA	-0.19	1.5E-05	0.19	1.8E-05	-0.42	3.5E-21	8.4E-02	9.6E-04
CSF2RA	NA	0.36	8.7E-17	-0.40	8.9E-21	0.33	7.7E-14	4.3E-05	3.8E-04
BIN1	NA	-0.40	1.3E-20	0.29	3.7E-11	-0.23	3.1E-07	2.8E-03	5.0E-05
HIPK3	NA	0.42	1.6E-20	-0.20	2.8E-05	0.31	4.8E-11	1.2E-03	1.3E-03
PILRB	NA	0.40	1.7E-20	-0.28	8.9E-11	0.34	5.5E-14	1.2E-02	7.5E-08
LRRFIP2	NA	-0.39	4.6E-20	0.22	5.5E-07	-0.38	2.3E-17	2.3E-03	7.6E-04
CAST	NA	0.34	9.1E-15	-0.39	8.0E-20	0.40	6.6E-20	2.5E-04	8.6E-05
ZFAND1	NA	-0.38	6.9E-19	0.18	6.0E-05	-0.40	1.3E-19	1.0E-01	3.1E-06
KIF13A	NA	-0.30	1.9E-11	0.25	6.2E-08	-0.41	2.4E-19	1.3E-08	5.8E-07
POLR2J2	NA	-0.38	9.0E-19	0.25	1.3E-08	-0.29	1.2E-10	5.1E-05	3.2E-01
PRKDC	NA	0.25	1.5E-08	-0.38	1.2E-18	0.29	2.1E-10	1.3E-02	9.7E-01

Gene Symbol	as_id	exon	chr	location	strand	as_description_id	gene_name	locus_group	gene_id	transcript_id	exon_inclusion_transcript_id	exon_exclusion_transcript_id	GO_BP	GO_CC	GO_MF	conservation_score
RWDD2B	60299	3	chr21	21q21.3	-	ENSG0000	RWD doma	protein-co	ENSG0000	ENST00000	ENST00000	ENST00000471269,ENST00000486719,ENST0				hg19.100w
NPIPB5	35567	3.3	chr16	16p12.2	+	ENSG0000	nuclear po	protein-co	ENSG0000	ENST00000	ENST00000	ENST00000541664,ENST00000542518				hg19.100w
LMO7	26067	12	chr13	13q22.2	+	ENSG0000	LIM domai	protein-co	ENSG0000	ENST00000	ENST00000	ENST00000341547,EN		CYTOPLASM;NUCLEUS		hg19.100w
HHLA3	3402	4	chr1	1p31.1	+	ENSG0000	HERV-H LT	protein-co	ENSG0000	ENST00000	ENST00000	ENST00000463058,ENST00000525653				hg19.100w
CLSTN1	575	11	chr1	1p36.22	-	ENSG0000	calsyntenin	protein-co	ENSG0000	ENST00000	ENST00000	ENST00000361311,ENST00000377298				hg19.100w
ARID1B	78237	12	chr6	6q25.3	+	ENSG0000	AT-rich inte	protein-co	ENSG0000	ENST00000	ENST00000	ENST00000275248,EN		PROTEIN C	DNA BINDI	hg19.100w
POLR2J3	81118	4.1	chr7	7q22.1	-	ENSG0000	RNA polym	protein-co	ENSG0000	ENST00000	ENST00000	ENST00000379340,ENST00000504157,ENST0				hg19.100w
APBB2	69101	8	chr4	4p14-p13	-	ENSG0000	amyloid be	protein-co	ENSG0000	ENST00000	ENST00000	ENST00000	CELL CYCLE	GROWTH C	TRANSCRIP	hg19.100w
PBX1	8790	11	chr1	1q23.3	+	ENSG0000	PBX homeo	protein-co	ENSG0000	ENST00000	ENST00000	ENST00000	TRANSCRIP	NUCLEUS		hg19.100w
ADAM15	7897	21.1:21.2	chr1	1q21.3	+	ENSG0000	ADAM met	protein-co	ENSG0000	ENST00000	ENST00000	ENST00000	CELL SUBSTRATE	ADHE	SH3 DOMA	hg19.100w
FAM195A	32927	3	chr16	16p13.3	+	ENSG0000	MAPK regu	protein-co	ENSG0000	ENST00000	ENST00000	ENST00000491999				hg19.100w
EXOC7	43573	7	chr17	17q25.1	-	ENSG0000	exocyst co	protein-co	ENSG0000	ENST00000	ENST00000	ENST00000357231,ENST00000467929,ENST0				hg19.100w
TTC8	28742	10.1	chr14	14q31.3	+	ENSG0000	tetratricop	protein-co	ENSG0000	ENST00000	ENST00000	ENST00000338104,ENST00000345383,ENST0				hg19.100w
TOP3B	61270	6	chr22	22q11.22	-	ENSG0000	topoisome	protein-co	ENSG0000	ENST00000	ENST00000	ENST00000444502,EN		NUCLEUS		hg19.100w
NPIPB4	35510	3.3	chr16	16p12.2	-	ENSG0000	nuclear po	protein-co	ENSG0000	ENST00000	ENST00000	ENST00000542133				hg19.100w
CCDC50	68126	6	chr3	3q28	+	ENSG0000	coiled-coil	protein-co	ENSG0000	ENST00000	ENST00000	ENST00000392456				hg19.100w
DDX52	40542	3	chr17	17q11.2	-	ENSG0000	zinc finger	protein-co	ENSG0000	ENST00000	ENST00000	ENST00000394367,ENST00000488402				hg19.100w
YBX3	20481	6	chr12	12p13.2	-	ENSG0000	Y-box bindi	protein-co	ENSG0000	ENST00000	ENST00000	ENST00000228251,ENST00000539204				hg19.100w
PKP2	21062	6	chr12	12p11.21	-	ENSG0000	plakophilin	protein-co	ENSG0000	ENST00000	ENST00000	ENST00000070846		INTRINSIC TO MEMBR		hg19.100w
GK	88735	23	chrX	Xp21.2	+	ENSG0000	glycerol kin	protein-co	ENSG0000	ENST00000	ENST00000	ENST00000378943,ENST00000427190,ENST0				hg19.100w
RPRD2	7472	4	chr1	1q21.2	+	ENSG0000	regulation	protein-co	ENSG0000	ENST00000	ENST00000	ENST00000369068				hg19.100w
DNM1L	21046	18	chr12	12p11.21	+	ENSG0000	dynamin 1	protein-co	ENSG0000	ENST00000	ENST00000	ENST00000	APOPTOSIS	INTRACELL	PYROPHOS	hg19.100w
SBF1	62828	29	chr22	22q13.33	-	ENSG0000	SET bindin	protein-co	ENSG0000	ENST00000	ENST00000	ENST00000	PROTEIN A	INTEGRAL	PHOSPHOP	hg19.100w
CFLAR	56799	10	chr2	2q33.1	+	ENSG0000	CASP8 and	protein-co	ENSG0000	ENST00000	ENST00000	ENST00000	POSITIVE REGULATION OF CELLUL			hg19.100w
TIA1	53874	6	chr2	2p13.3	-	ENSG0000	TIA1 cytoto	protein-co	ENSG0000	ENST00000	ENST00000	ENST00000	APOPTOSIS	GO;REGUL	SINGLE STR	hg19.100w
PILRA	80941	3	chr7	7q22.1	+	ENSG0000	paired imm	protein-co	ENSG0000	ENST00000	ENST00000	ENST00000	SIGNAL TRANSDUCTION			hg19.100w
THUMPD2	53337	4	chr2	2p22.1	-	ENSG0000	THUMP do	protein-co	ENSG0000	ENST00000	ENST00000	ENST00000454352,ENST00000510781				hg19.100w
MON2	22839	31	chr12	12q14.1	+	ENSG0000	MON2 hom	protein-co	ENSG0000	ENST00000	ENST00000	ENST00000	CELLULAR LOCALIZATION;ENDOSO			hg19.100w
ADAM15	7898	21.2	chr1	1q21.3	+	ENSG0000	ADAM met	protein-co	ENSG0000	ENST00000	ENST00000	ENST00000	CELL SUBSTRATE	ADHE	SH3 DOMA	hg19.100w
PAM	72906	14	chr5	5q21.1	+	ENSG0000	peptidylgly	protein-co	ENSG0000	ENST00000	ENST00000	ENST00000	MITOSIS;C	CYTOPLAS	CHROMATI	hg19.100w
SYK	86821	9	chr9	9q22.2	+	ENSG0000	spleen asso	protein-co	ENSG0000	ENST00000	ENST00000	ENST00000	PROTEIN C	PLASMA M	RECEPTOR	hg19.100w
COL6A3	58104	6	chr2	2q37.3	-	ENSG0000	collagen ty	protein-co	ENSG0000	ENST00000	ENST00000	ENST00000	ORGAN DE	COLLAGEN;EXTRACELL		hg19.100w
DLG1	68295	9	chr3	3q29	-	ENSG0000	discs large	protein-co	ENSG0000	ENST00000	ENST00000	ENST00000	CELL CELL	PLASMA M	PROTEIN K	hg19.100w
APP	60283	10	chr21	21q21.3	-	ENSG0000	amyloid be	protein-co	ENSG0000	ENST00000	ENST00000	ENST00000	CATION HO	PLASMA M	SERINE TYP	hg19.100w
TFIP11	61515	3	chr22	22q12.1	-	ENSG0000	tuftelin int	protein-co	ENSG0000	ENST00000	ENST00000	ENST00000405938		NUCLEAR SPECK;INTRA		hg19.100w
CLASP1	55177	22	chr2	2q14.2-q14	-	ENSG0000	cytoplasmi	protein-co	ENSG0000	ENST00000	ENST00000	ENST00000	REGULATIO	INTRACELL	CYTOSKELE	hg19.100w

Gene Svmbol	dbSNP_variant	COSMIC_variant	SPIDEX_variant	miRNA_binding_site	repeat.	NMD	COSMIC_NMD	dbSNP_NMD	protein_domain	protein_translational_modification	RBP_splicing_factor	RBP	tissue as	isoform_PPI_a	isoform_PPI_b	isoform_subcellular_localization_id
RWDD2B	rs3748103	chr21:3038	chr21:3038	NA			NA			Chain,1-319,chr21:303		DDX3X,6.7	brain:0.075;bonemarrow:-0.052;p			NA
NPIPB5	ay.phastCons:(E)0.088/		chr16:2253	NA	SelfChain,chr16:22523		NA			Chain,1-11	ELAVL1,2.7	ADAR,6.460e-07,chr16:22525313-22525325,1				NA
LMO7	ay.phastCons:(E)0.427/		chr13:7637	NA			NA				HNRNPA1,	AGGF1,2.1	brain:-0.27	ENST00000LMO7/UBA		NA
HHLA3	rs3681276	chr1:70832	chr1:70832	NA	Interrupted Rpts,chr1:		NA				PCBP2,1.11	YBX3,9.366	brain:0.064;stomach:-0.171;bone			NA
CLSTN1	ay.phastCons:(E)0.724/		chr1:97959	NA			NA		ENST00000	Chain,29-8	FXR1,2.137	BUD13,5.6	brain:-0.76	ENST00000CLSTN1/AP		NA
ARID1B	rs3736533	chr6:15749	chr6:15749	NA			NA			Chain,2-22	FMR1,4.22	BUD13,7.3	brain:-0.14	ENST00000SMARCC2/		NA
POLR2J3	ay.phastCons:(E)0.196/		chr7:10220	NA			NA		ENST00000	Chain,1-11	HNRNPK,2.	CBX3,3.380	brain:0.359;thyroid:0.064;bonema			NA
APBB2	rs7484777	chr4:40936	chr4:40936	NA			NA			Chain,1-75	HNRNPA1,	EFTUD2,1.835e-07,chr		ENST00000APLP2/SM		NA
PBX1	ay.phastCo	chr1:16478	chr1:16478	NA			NA		ENST00000	Chain,1-430,chr1:164529060-1648			brain:-0.515;thyroid:-0.219;stoma			NA
ADAM15	rs11589479,chr1:1550		chr1:15503	NA			NA			Chain,207-	HNRNPK,9.	DROSHA,1.	brain:-0.274;bonemarrow:-0.081;s			NA
FAM195A	rs369502374,chr16:69		chr16:6921	NA		chr16:6964	NA			Chain,1-16	HNRNPC,1.	CSTF2T,1.9	brain:-0.365;thyroid:-0.218;stoma			NA
EXOC7	rs3685557	chr17:7408	chr17:7408	NA			NA				HNRNPA1,	BUD13,3.7	brain:-0.457;stomach:0.131;pancr			NA
TTC8	rs119103286,chr14:89		chr14:8930	NA		chr14:8930	NA				U2AF2,2.96	PRPF8,1.87	brain:-0.111;thyroid:-0.133;stoma			NA
TOP3B	rs373549523,chr22:22		chr22:2232	NA		chr22:2232	NA			Chain,1-86	FMR1,2.41	AARS,7.935	brain:0.111;thyroid:-0.123;bonem			NA
NPIPB4	ay.phastCo	chr16:21858734,C>A		NA	SelfChain,chr16:21862		NA			Chain,1-11	ELAVL1,2.5	ADAR,1.56	stomach:0.365;bonemarrow:0.11;			NA
CCDC50	rs146587227,chr3:191		chr3:19108	NA			NA			Chain,2-30	HNRNPC,8.	AGGF1,1.0	thyroid:-0.618;stomach:-0.478;pa			NA
DDX52	rs182825918,chr17:35		chr17:3599	NA	RepeatMas	chr17:3599	NA				KHSRP,1.58	BUD13,1.6	bonemarrow:-0.068;fat:-0.074			NA
YBX3	rs111494814,chr12:10		chr12:1085	NA			NA			Chain,2-37	HNRNPA1,	AGGF1,3.6	skin:0.303;testis:-0.17;heart:0.116			NA
PKP2	rs1391594	chr12:3299	chr12:3299	NA	RepeatMasker,SINE,ch		NA		ENST00000	Chain,1-881,chr12:329		FASTKD2,3	fat:-0.101	ENST00000DSC1/DSG		NA
GK	ay.phastCons:(E)0.355/		chrX:30742	NA			NA				HNRNPA1,	BUD13,1.7	brain:-0.207;thyroid:0.463;stomac			NA
RPRD2	ay.phastCo	chr1:15041	chr1:15041	NA			NA			Chain,1-14	RBFOX2,4.	BUD13,5.7	brain:-0.09;thyroid:-0.13;stomach:			NA
DNM1L	rs2015993	chr12:3289	chr12:3289	NA			NA			Chain,1-73	HNRNPA1,	BUD13,4.1	brain:-0.05	ENST00000MAP3K10/		NA
SBF1	ay.phastCons:(E)0.537/		chr22:5089	NA			NA				FXR2,1.413	BUD13,1.1	brain:0.111;stomach:0.125;pancre			NA
CFLAR	rs13424615,chr2:2020		chr2:20200	NA	RepeatMasker,SINE,ch		NA			Chain,1-37	HNRNPA1,	AGGF1,2.208e-13,chr2:202005084-20200515				NA
TIA1	rs3751919	chr2:70454	chr2:70454	NA			NA			Chain,1-38	HNRNPC,1.	BUD13,6.6	stomach:-0.101;liver:0.162;fat:-0.1			NA
PILRA	rs2013400	chr7:99972	chr7:99971	NA			NA			Chain,20-2	HNRNPK,3.	CBX3phosp	stomach:0.	ENST00000SORBS1/PT		NA
THUMPD2	rs3709638	chr2:39996	chr2:39995	NA			NA		ENST00000	Chain,1-50	KHDRBS1,1	AGGF1,1.8	bonemarrow:-0.209;skin:-0.124;s			NA
MON2	rs146625340,chr12:62		chr12:6295	NA			NA		ENST00000	Chain,2-17	FXR1,4.865	BUD13,5.0	brain:-0.071;thyroid:0.169;stomac			NA
ADAM15	rs11589479,chr1:1550		chr1:15503	NA			NA			Chain,207-	HNRNPK,9.	DROSHA,1.	brain:-0.25	ENST00000GRB2/NCK		NA
PAM	ay.phastCons:(E)0.35/(I		chr5:10229	NA			NA			Chain,31-9	HNRNPU,3	AGGF1,2.3	brain:-0.29	ENST00000POMC/RAS		NA
SYK	rs1999086	chr9:93629	chr9:93627	NA			NA			Chain,1-63	IGF2BP1,7.	CHD4,1.17	thyroid:0.692;stomach:0.38;pancr			NA
COL6A3	ay.phastCo	chr2:23828	chr2:23828	NA			NA			Chain,26-7	FXR1,4.907	CBX3phosp	brain:-0.468;stomach:0.053;bone			NA
DLG1	rs184137321,chr3:196		chr3:19687	NA			NA		ENST00000	Chain,1-90	HNRNPC,1.	BUD13,1.8	brain:0.22;	ENST00000KIF13B/Dlg		NA
APP	rs2001547	chr21:2737	chr21:2735	NA			NA			Chain,18-6	HNRNPA1,	AGGF1,1.4	brain:0.42;	ENST00000HOMER2/H		NA
TFIP11	rs200883222,chr22:26		chr22:2690	NA	RepeatMasker,LTR,chr		NA					BUD13,3.1	brain:-0.14;stomach:0.076;pancre			NA
CLASP1	ay.phastCo	chr2:12220	chr2:12218	NA			NA			Chain,1-15	HNRNPC,1.	BUD13,6.0	brain:0.487	ENST00000MAPRE1/M		NA

Gene Symbol	isoform_subcellular_localization	tds.corr	tds.pval	erk.corr	erk.pval	ras.corr	ras.pval	ETE.pval	Lymph_node_metastasis.pval
RWDD2B	NA	-0.23	4.8E-07	0.39	1.2E-18	-0.25	1.0E-07	8.0E-03	9.6E-04
NPIPB5	NA	0.39	1.3E-18	-0.32	9.3E-13	0.39	4.8E-18	1.3E-05	1.7E-07
LMO7	NA	-0.36	2.5E-16	0.39	1.3E-18	-0.37	3.0E-16	2.9E-03	1.5E-03
HHLA3	NA	-0.33	1.6E-14	0.32	4.5E-13	-0.39	1.3E-18	7.3E-04	4.6E-04
CLSTN1	NA	0.22	7.2E-07	-0.37	1.1E-17	0.39	1.8E-18	3.6E-04	5.9E-03
ARID1B	NA	-0.38	2.7E-18	0.32	2.6E-13	-0.37	5.1E-16	7.4E-03	4.4E-03
POLR2J3	NA	-0.38	2.8E-18	0.23	2.4E-07	-0.28	5.2E-10	1.3E-04	2.9E-01
APBB2	NA	-0.31	3.6E-12	0.18	4.5E-05	-0.39	2.8E-18	4.4E-06	1.1E-02
PBX1	NA	0.32	2.6E-13	-0.34	9.7E-15	0.38	3.8E-18	4.1E-02	7.1E-01
ADAM15	NA	0.18	3.2E-05	-0.29	6.0E-11	0.38	8.3E-18	1.1E-01	3.4E-03
FAM195A	NA	0.21	2.4E-06	-0.37	1.4E-17	0.20	1.4E-05	2.6E-02	1.6E-01
EXOC7	NA	0.24	6.9E-08	-0.37	2.1E-17	0.30	1.8E-11	2.8E-02	1.3E-03
TTC8	NA	0.37	3.3E-17	-0.30	6.6E-12	0.35	3.8E-15	1.1E-04	2.9E-06
TOP3B	NA	0.18	1.1E-04	-0.37	4.9E-17	0.22	2.4E-06	1.6E-02	6.5E-02
NPIPB4	NA	0.38	6.3E-17	-0.31	2.4E-11	0.37	1.2E-15	1.0E-04	3.5E-06
CCDC50	NA	0.36	7.9E-17	-0.20	4.2E-06	0.29	1.2E-10	2.8E-01	1.0E-01
DDX52	NA	0.28	7.1E-10	-0.22	7.7E-07	0.37	9.4E-17	9.9E-05	7.9E-04
YBX3	NA	0.14	1.5E-03	-0.36	1.0E-16	0.11	1.6E-02	5.2E-05	1.8E-01
PKP2	NA	0.22	3.3E-06	-0.23	8.7E-07	0.39	1.5E-16	1.5E-02	1.7E-03
GK	NA	-0.36	1.5E-16	0.16	3.6E-04	-0.22	1.3E-06	1.1E-02	5.1E-06
RPRD2	NA	0.37	1.7E-16	-0.29	7.8E-11	0.33	4.7E-13	6.6E-04	7.4E-04
DNM1L	NA	-0.35	1.6E-15	0.36	1.7E-16	-0.24	2.8E-07	1.9E-03	8.7E-05
SBF1	NA	0.36	2.0E-16	-0.23	2.6E-07	0.22	1.1E-06	7.1E-04	5.9E-04
CFLAR	NA	0.06	1.5E-01	-0.26	2.2E-09	0.36	2.2E-16	3.0E-03	2.1E-05
TIA1	NA	0.21	1.7E-06	-0.35	3.9E-16	0.23	3.8E-07	1.5E-03	9.9E-03
PILRA	NA	0.30	8.4E-12	-0.35	4.3E-16	0.34	4.4E-14	2.1E-04	3.6E-02
THUMPD2	NA	0.22	1.0E-06	-0.36	4.6E-16	0.20	1.3E-05	1.2E-03	6.6E-02
MON2	NA	-0.29	7.3E-11	0.20	9.1E-06	-0.37	4.8E-16	2.0E-02	3.7E-01
ADAM15	NA	0.19	2.4E-05	-0.27	1.1E-09	0.36	9.8E-16	2.1E-01	4.3E-03
PAM	NA	-0.35	1.0E-15	0.11	1.9E-02	-0.31	8.0E-12	5.7E-04	6.1E-08
SYK	NA	-0.35	1.0E-15	0.29	2.4E-11	-0.11	1.4E-02	2.5E-02	3.1E-03
COL6A3	NA	-0.37	1.6E-15	0.34	2.6E-13	-0.30	4.7E-10	1.5E-07	3.0E-04
DLG1	NA	-0.29	1.6E-10	0.36	1.7E-15	-0.24	3.2E-07	9.1E-04	1.6E-04
APP	NA	-0.34	1.9E-15	0.25	9.4E-09	-0.24	1.0E-07	6.6E-03	2.6E-03
TFIP11	NA	0.29	2.0E-10	-0.28	8.2E-10	0.36	3.8E-15	3.1E-04	7.2E-02
CLASP1	NA	-0.37	3.8E-15	0.27	2.2E-08	-0.34	9.6E-13	2.9E-03	1.6E-02

Gene Symbol	as_id	exon	chr	location	strand	as_description_id	gene_name	locus_group	gene_id	transcript_id	exon_inclusion_transcript_id	exon_exclusion_transcript_id	GO_BP	GO_CC	GO_MF	conservation_score
BRE	53054	14	chr2	2p23.2	+	ENSG0000	brain and r	protein-co	ENSG0000	ENST00000	ENST00000	ENST00000	NEGATIVE	PROTEIN C	CYTOKINE	hg19.100w
ARAP1	17641	32	chr11	11q13.4	-	ENSG0000	ArfGAP wit	protein-co	ENSG0000	ENST00000	ENST00000	ENST00000	REGULATIO	CYTOPLAS	GTPASE AC	hg19.100w
FCHSD1	73817	19	chr5	5q31.3	-	ENSG0000	FCH and do	protein-co	ENSG0000	ENST00000	ENST00000	ENST00000522783				hg19.100w
ST7	81563	10	chr7	7q31.2	+	ENSG0000	suppressio	protein-co	ENSG0000	ENST00000	ENST00000	ENST00000265437,ENST00000323984,ENST0				hg19.100w
FAM86B1	82685	5	chr8	8p23.1	-	ENSG0000	family with	protein-co	ENSG0000	ENST00000	ENST00000	ENST00000529146				hg19.100w
YIPF1	3079	3	chr1	1p32.3	-	ENSG0000	Yip1 domai	protein-co	ENSG0000	ENST00000	ENST00000	ENST00000072644,EN		CYTOPLASMIC	MEMBR	hg19.100w
APOD	68181	3	chr3	3q29	-	ENSG0000	apolipopro	protein-co	ENSG0000	ENST00000	ENST00000	ENST00000	LIPID META	EXTRACELL	SUBSTRAT	hg19.100w
EHBP1	53718	18	chr2	2p15	+	ENSG0000	EH domain	protein-co	ENSG0000	ENST00000	ENST00000	ENST00000263991,ENST00000354487,ENST0				hg19.100w
PGAP2	14010	7	chr11	11p15.4	+	ENSG0000	post-GPI at	protein-co	ENSG0000	ENST00000	ENST00000	ENST00000278243,ENST00000396991,ENST0				hg19.100w
NPIPB5	35568	3.1	chr16	16p12.2	+	ENSG0000	nuclear po	protein-co	ENSG0000	ENST00000	ENST00000	ENST00000521555,ENST00000545375				hg19.100w
NMRK1	86630	3	chr9	9q21.13	-	ENSG0000	nicotinami	protein-co	ENSG0000	ENST00000	ENST00000	ENST00000376811				hg19.100w
INSR	47099	11	chr19	19p13.2	-	ENSG0000	insulin rece	protein-co	ENSG0000	ENST00000	ENST00000	ENST00000	CELLULAR	PLASMA M	PROTEIN T	hg19.100w
MAP2	57225	13	chr2	2q34	+	ENSG0000	microtubul	protein-co	ENSG0000	ENST00000	ENST00000	ENST00000199940		INTRACELLULAR	NON	hg19.100w
FGFR2	13318	4	chr10	10q26.13	-	ENSG0000	fibroblast g	protein-co	ENSG0000	ENST00000	ENST00000	ENST00000346997,EN		CELL SURFACE		hg19.100w
LIAS	69062	7	chr4	4p14	+	ENSG0000	lipoic acid	protein-co	ENSG0000	ENST00000	ENST00000	ENST00000261434,ENST00000340169				hg19.100w
RREB1	75253	12	chr6	6p24.3	+	ENSG0000	ras respons	protein-co	ENSG0000	ENST00000	ENST00000	ENST00000	TRANSCRIP	NUCLEUS;C	TRANSCRIP	hg19.100w
REPS1	77956	9.3	chr6	6q24.1	-	ENSG0000	RALBP1 ass	protein-co	ENSG0000	ENST00000	ENST00000	ENST00000258062,ENST00000431346,ENST0				hg19.100w
BIN1	55184	16	chr2	2q14.3	-	ENSG0000	bridging int	protein-co	ENSG0000	ENST00000	ENST00000	ENST00000	CELL PROLI	INTRACELLULAR	NON	hg19.100w
PICALM	18172	14.1:14.2	chr11	11q14.2	-	ENSG0000	phosphatid	protein-co	ENSG0000	ENST00000	ENST00000	ENST00000	RECEPTOR	MEMBRAN	PHOSPHOI	hg19.100w
PDDC1	13755	4.1:4.2	chr11	11p15.5	-	ENSG0000	glutamine	protein-co	ENSG0000	ENST00000	ENST00000	ENST00000534603				hg19.100w
ARHGEF10	82562	10	chr8	8p23.3	+	ENSG0000	Rho guanin	protein-co	ENSG0000	ENST00000	ENST00000	ENST00000262112,ENST00000349830,ENST0				hg19.100w
ZNF207	40205	10	chr17	17q11.2	+	ENSG0000	zinc finger	protein-co	ENSG0000	ENST00000	ENST00000	ENST00000321233,ENST00000341711,ENST0				hg19.100w
ABCA2	88259	4.1	chr9	9q34.3	-	ENSG0000	ATP bindin	protein-co	ENSG0000	ENST00000	ENST00000	ENST00000	RESPONSE	CYTOSKELE	ATPASE AC	hg19.100w
DTNB	52862	21	chr2	2p23.3	-	ENSG0000	dystrobrev	protein-co	ENSG0000	ENST00000	ENST00000	ENST00000406818,ENST00000407186,ENST0				hg19.100w
SUGT1	26006	7	chr13	13q14.3	+	ENSG0000	SGT1 homo	protein-co	ENSG0000	ENST00000	ENST00000	ENST00000	M PHASE O	CHROMOSOME;PROTE		hg19.100w
EPB41L1	59273	20	chr20	20q11.23	+	ENSG0000	erythrocyt	protein-co	ENSG0000	ENST00000	ENST00000	ENST00000202028,ENST00000338074,ENST0				hg19.100w
D2HGDH	58422	7.1:7.2:7.3	chr2	2q37.3	+	ENSG0000	D-2-hydrox	protein-co	ENSG0000	ENST00000	ENST00000	ENST00000	CELLULAR	CYTOPLAS	OXIDORED	hg19.100w
C4orf22	69683	6	chr4	4q21.21	+	ENSG0000	chromoso	protein-co	ENSG0000	ENST00000	ENST00000	ENST00000513920				hg19.100w
PAM	72902	23.2	chr5	5q21.1	+	ENSG0000	peptidylgly	protein-co	ENSG0000	ENST00000	ENST00000	ENST00000	MITOSIS;C	CYTOPLAS	CHROMATI	hg19.100w
NPIPB4	35511	3.1	chr16	16p12.2	-	ENSG0000	nuclear po	protein-co	ENSG0000	ENST00000	ENST00000	ENST00000520808,ENST00000536041,ENST0				hg19.100w
MAP4K4	54762	17	chr2	2q11.2	+	ENSG0000	mitogen-ac	protein-co	ENSG0000	ENST00000	ENST00000	ENST00000	PROTEIN MODIFICATIO		ATP BINDIN	hg19.100w
FIP1L1	69312	14	chr4	4q12	+	ENSG0000	factor inter	protein-co	ENSG0000	ENST00000	ENST00000	ENST00000358575,ENST00000504094,ENST0				hg19.100w
FMNL3	21605	6	chr12	12q13.12	-	ENSG0000	formin like	protein-co	ENSG0000	ENST00000	ENST00000	ENST00000293590,ENST00000335154,ENST0				hg19.100w
RAI14	71716	18	chr5	5p13.2	+	ENSG0000	retinoic aci	protein-co	ENSG0000	ENST00000	ENST00000	ENST00000265109,ENST00000397449,ENST0				hg19.100w
TTC31	54097	3	chr2	2p13.1	+	ENSG0000	tetratricop	protein-co	ENSG0000	ENST00000	ENST00000	ENST00000233623,ENST00000410003,ENST0				hg19.100w
PALLD	71133	14	chr4	4q32.3	+	ENSG0000	palladin, cy	protein-co	ENSG0000	ENST00000	ENST00000	ENST00000335742		INTRACELL	CYTOSKELE	hg19.100w

Gene Svmbol	dbSNP_variant	COSMIC_variant	SPIDEX_variant	miRNA_binding_site	repeat.	NMD	COSMIC_NMD	dbSNP_NMD	protein_domain	protein_translational_modification	RBP_splicing_factor	RBP	tissue	as	isoform_PPI_a	isoform_PPI_b	isoform_subcellular_localization_id
BRE	rs370258733,chr2:285		chr2:28521	NA			NA			Acetylation	HNRNPM,1	DGCR8,7.9	bonemarro		ENST00000	ATG2A/BA	NA
ARAP1	rs148121568,chr11:72		chr11:7239	NA			NA		ENST00000359373,PF0		FXR2,3.095	AGGF1,2.6	thyroid:0.4		ENST00000	ARPC3/XRC	NA
FCHSD1	rs2001196	chr5:14102	chr5:14102	NA	RepeatMasker,LINE,ch		NA			Chain,1-69	SRSF1,4.37	BUD13,6.2	pancreas:0.095;skin:0.062;prostat				NA
ST7	ay.phastCo	chr7:11677	chr7:11677	NA	Interrupted Rpts,chr7:		NA		ENST00000	Chain,1-58	FXR1,4.585	BUD13,1.7	brain:-0.11		ENST00000	GNB2L1;G	NA
FAM86B1	ay.phastCons:(E)0.4/(I)		chr8:12047	NA	RepeatMasker,RC,chr8		NA		ENST00000	Chain,1-29	HNRNPH1,	ADAR,6.51	testis:0.311;kidney:0.139;spleen:0				NA
YIPF1	ay.phastCons:(E)0.353/		chr1:54354	NA	RepeatMasker,SINE,ch		NA				IGF2BP2,3.	DDX3X,3.5	stomach:0.108;bonemarrow:-0.08				NA
APOD	ay.phastCons:(E)0.389/		chr3:19530	NA	Interrupted Rpts,chr3:		NA		ENST00000	Chain,21-108,chr3:195300764-195306272;Chain,21-189,chr3:195295							NA
EHBP1	rs199719337,chr2:632		chr2:63206	NA			NA			Chain,1-12	HNRNPA1,	AGGF1,3.1	brain:-0.24		ENST00000	UBC/PACSI	NA
PGAP2	ay.phastCons:(E)0.234/		chr11:3832	NA			NA			Chain,1-25	HNRNPU,1	CHD1,3.690e-08,chr11			ENST00000	CREB3L1;C	NA
NPIPB5	ay.phastCons:(E)0.088/		chr16:2253	NA	SelfChain,c	chr16:2253	NA			Chain,1-11	ELAVL1,2.7	ADAR,6.460e-07,chr16:22525313-22525325,1					NA
NMRK1	ay.phastCons:(E)0.201/		chr9:77692	NA	RepeatMasker,DNA,ch		NA		ENST00000	Chain,1-19	HNRNPK,3.610e-18,ch		brain:0.204;thyroid:0.122;stomach				NA
INSR	ay.phastCo	chr19:7143	chr19:7142	NA	Interrupted Rpts,chr19		NA			Chain,28-7	HNRNPC,7.	AGGF1,1.7	brain:0.424;stomach:0.223;bonem				NA
MAP2	rs1430898	chr2:21056	chr2:21056	NA			NA			Chain,1-1827,chr2:210517895-210			brain:0.243		ENST00000	SRC/TUBA1	NA
FGFR2	ay.phastCo	chr10:1233	chr10:1233	NA			NA			Chain,22-251,chr10:12		CREBBP,8.0	brain:-0.10		ENST00000	FGF1/FGF1	NA
LIAS	ay.phastCo	chr4:39469	chr4:39466	NA			NA		ENST00000	Chain,28-3	FXR1,2.680	BUD13,4.4	thyroid:0.05;stomach:0.084;pancr				NA
RREB1	rs372956114,chr6:724		chr6:72292	NA			NA			Chain,1-16	FMR1,1.90	BUD13,4.3	brain:0.416;thyroid:0.158;stomach				NA
REPS1	rs1419965	chr6:13924	chr6:13924	NA			NA			Chain,1-79	IGF2BP1,1.	ILF3,4.035e	brain:0.133		ENST00000	CFTR/REPS	NA
BIN1	rs34647988,chr2:1278		chr2:12780	NA			NA						brain:-0.357;bonemarrow:-0.482;p				NA
PICALM	rs1462855	chr11:8569	chr11:8569	NA			NA			Chain,2-65	FXR1,1.081	BUD13,6.9	brain:0.092;thyroid:0.186;stomach				NA
PDDC1	rs3725191	chr11:7740	chr11:7724	NA		chr11:7735	NA			Chain,39-2	EWSR1,6.4	BUD13,8.0	brain:0.231;thyroid:0.147;bonema				NA
ARHGEF10	rs1995142	chr8:18282	chr8:18247	NA			NA			Chain,1-1369,chr8:177		DDX24,9.6	brain:-0.411;thyroid:-0.106;bonem				NA
ZNF207	ay.phastCons:(E)0.48/(I		chr17:3069	NA			NA			Chain,1-47	IGF2BP1,2.	BUD13,2.9	thyroid:-0.279;stomach:-0.054;bo				NA
ABCA2	ay.phastCons:(E)0.35/(I)0.253;phas			NA			NA			Chain,1-24	FMR1,5.98	BUD13,3.4	brain:0.134;thyroid:0.242;bonema				NA
DTNB	ay.phastCo	chr2:25606	chr2:25602	NA			NA			Acetylation	HNRNPK,3.	PRPF8,2.56	brain:0.145;thyroid:-0.202;stomac				NA
SUGT1	ay.phastCo	chr13:5323	chr13:5323	NA	RepeatMasker,SINE,ch		NA			Chain,2-36	IGF2BP1,2.	BUD13,3.1	brain:-0.24		ENST00000	S100P/S10	NA
EPB41L1	ay.phastCons:(E)0.418/		chr20:3478	NA			NA			Chain,1-88	HNRNPU,1	BUD13,4.4	brain:-0.16		ENST00000	GRIA4/DRD	NA
D2HGDH	rs3769557	chr2:24268	chr2:24268	NA	RepeatMas	chr2:24268	NA			Chain,14-5	HNRNPC,3.	BUD13,1.9	brain:0.375;thyroid:0.127;bonema				NA
C4orf22	ay.phastCo	chr4:81504	chr4:81504	NA	Interrupted Rpts,chr4:		NA		ENST00000	Chain,1-233,chr4:8125		SMNDC1,8	brain:-0.796;thyroid:0.099;lung:-0				NA
PAM	ay.phastCons:(E)0.87/(I		chr5:10235	NA			NA			Chain,31-973,chr5:102202978-102			brain:0.07;skin:-0.115;prostate:0.0				NA
NPIPB4	ay.phastCo	chr16:21858734,C>A		NA	SelfChain,c	chr16:2186	NA			Chain,1-11	ELAVL1,2.5	ADAR,1.56	brain:-0.169;skin:-0.074;ovary:-0.3				NA
MAP4K4	ay.phastCo	chr2:10248	chr2:10247	NA			NA			Chain,2-12	FXR2,6.943	AGGF1,6.692e-44,chr2:102476190-10247621					NA
FIP1L1	rs3414707	chr4:54294	chr4:54294	NA			NA			Chain,1-59	FXR2,1.359	BUD13,2.841e-20,chr4:54294190-54294224,3					NA
FMNL3	ay.phastCo	chr12:5005	chr12:5005	NA			NA		ENST00000352151,PF0		IGF2BP1,2.	DNMT1,2.4	brain:-0.166;thyroid:-0.589;stoma				NA
RAI14	ay.phastCo	chr5:34814	chr5:34812	NA			NA			Chain,1-98	KHSRP,1.74	BUD13,5.2	brain:0.108		ENST00000	ITSN1/MD	NA
TTC31	ay.phastCo	chr2:74710	chr2:74710	NA			NA			Chain,1-51	HNRNPA1,	AGGF1,2.9	brain:0.498;stomach:-0.053;bone				NA
PALLD	rs1496310	chr4:16963	chr4:16963	NA	RepeatMasker,Low_co		NA				HNRNPU,6	CSTF2T,1.4	brain:0.137		ENST00000	UBC/EZR/A	NA

Gene Symbol	isoform_subcellular_localization	tds.corr	tds.pval	erk.corr	erk.pval	ras.corr	ras.pval	ETE.pval	Lymph_node_metastasis.pval
BRE	NA	0.32	1.6E-13	-0.34	4.1E-15	0.24	1.2E-07	1.3E-05	4.0E-05
ARAP1	NA	-0.34	5.9E-15	0.31	5.2E-13	-0.25	2.3E-08	7.6E-05	3.8E-06
FCHSD1	NA	0.24	7.4E-08	-0.34	5.9E-15	0.20	1.8E-05	1.3E-03	8.2E-02
ST7	NA	-0.34	6.7E-15	0.21	1.7E-06	-0.24	7.8E-08	2.4E-02	2.2E-06
FAM86B1	NA	0.24	9.6E-08	-0.34	7.7E-15	0.21	4.9E-06	5.8E-04	2.4E-01
YIPF1	NA	0.34	8.8E-15	-0.12	5.3E-03	0.13	5.0E-03	3.3E-02	3.5E-03
APOD	NA	0.17	1.8E-04	-0.29	9.0E-11	0.34	1.3E-14	4.2E-05	8.5E-08
EHBP1	NA	-0.33	2.8E-14	0.23	1.9E-07	-0.26	8.5E-09	1.1E-03	4.5E-04
PGAP2	NA	-0.23	3.5E-07	0.26	5.1E-09	-0.34	4.1E-14	3.2E-04	2.8E-07
NPIPB5	NA	0.27	2.8E-09	-0.25	2.4E-08	0.34	4.6E-14	2.6E-02	3.1E-03
NMRK1	NA	0.10	2.8E-02	-0.33	4.8E-14	0.25	1.6E-08	1.5E-04	8.2E-02
INSR	NA	-0.17	2.5E-04	0.33	3.1E-13	-0.35	5.2E-14	2.6E-01	5.4E-02
MAP2	NA	0.33	7.9E-14	-0.32	3.4E-13	0.32	5.5E-12	4.8E-02	2.5E-01
FGFR2	NA	0.25	9.2E-09	-0.33	1.2E-13	0.30	1.5E-11	4.7E-03	4.0E-01
LIAS	NA	-0.32	1.4E-13	0.16	3.1E-04	-0.24	9.6E-08	7.2E-01	6.2E-02
RREB1	NA	-0.33	1.8E-13	0.33	2.9E-13	-0.28	1.9E-09	3.7E-02	5.7E-02
REPS1	NA	-0.32	2.4E-13	0.16	2.6E-04	-0.25	5.2E-08	9.4E-03	4.9E-03
BIN1	NA	0.30	2.0E-11	-0.32	2.5E-13	0.26	8.9E-09	7.4E-03	2.2E-02
PICALM	NA	-0.23	3.4E-07	0.32	2.9E-13	-0.24	1.8E-07	2.3E-02	1.8E-05
PDDC1	NA	-0.35	3.2E-13	0.24	8.2E-07	-0.25	5.3E-07	5.6E-03	7.5E-04
ARHGEF10	NA	-0.30	1.0E-10	0.16	4.2E-04	-0.34	3.3E-13	3.0E-03	1.2E-03
ZNF207	NA	-0.32	4.4E-13	0.02	6.6E-01	-0.24	1.2E-07	3.5E-03	1.9E-01
ABCA2	NA	-0.34	6.1E-13	0.34	6.9E-13	-0.31	3.1E-10	7.8E-05	1.0E-02
DTNB	NA	0.31	6.9E-13	-0.18	6.6E-05	0.19	2.5E-05	3.9E-01	3.8E-03
SUGT1	NA	-0.31	8.0E-13	0.14	1.3E-03	-0.25	1.9E-08	4.9E-03	1.1E-01
EPB41L1	NA	0.31	1.2E-12	-0.27	1.0E-09	0.29	1.6E-10	7.1E-06	3.1E-05
D2HGDH	NA	-0.31	1.5E-12	0.02	6.8E-01	-0.21	2.9E-06	5.2E-03	3.3E-05
C4orf22	NA	-0.34	1.6E-12	0.18	2.2E-04	-0.23	2.5E-06	5.7E-03	6.5E-03
PAM	NA	-0.20	7.8E-06	0.18	7.8E-05	-0.32	1.6E-12	1.6E-01	7.3E-01
NPIPB4	NA	0.23	5.2E-07	-0.23	5.5E-07	0.33	1.7E-12	1.2E-01	6.4E-03
MAP4K4	NA	-0.30	6.3E-12	0.25	1.6E-08	-0.27	3.2E-09	1.7E-02	6.6E-04
FIP1L1	NA	0.27	1.0E-09	-0.24	3.3E-08	0.31	8.8E-12	7.3E-03	8.4E-03
FMNL3	NA	0.25	2.3E-08	-0.30	1.5E-11	0.25	4.7E-08	1.1E-02	1.3E-02
RAI14	NA	-0.10	4.0E-02	0.30	2.4E-11	-0.20	2.2E-05	9.6E-02	1.3E-02
TTC31	NA	-0.07	1.3E-01	0.29	2.5E-11	-0.02	6.6E-01	1.3E-02	4.5E-01
PALLD	NA	-0.30	3.0E-11	0.20	2.0E-05	-0.29	4.0E-10	1.5E-02	8.4E-07

Gene Symbol	as_id	exon	chr	location	strand	as_description_id	gene_name	locus_group	gene_id	transcript_id	exon_inclusion_transcript_id	exon_exclusion_transcript_id	GO_BP	GO_CC	GO_MF	conservation_score
CAPN3	30161	11	chr15	15q15.1	+	ENSG0000	calpain 3	protein-co	ENSG0000	ENST00000	ENST00000	ENST00000	ORGAN	DEVELOPMEN	CYSTEINE T	hg19.100w
SCRIB	85500	36	chr8	8q24.3	-	ENSG0000	scribbled p	protein-co	ENSG0000	ENST00000	ENST00000	ENST00000356994				hg19.100w
DMKN	49144	21	chr19	19q13.12	-	ENSG0000	dermokine	protein-co	ENSG0000	ENST00000	ENST00000	ENST00000462721,ENST00000472252				hg19.100w
TACC2	13344	12	chr10	10q26.13	+	ENSG0000	transformi	protein-co	ENSG0000	ENST00000	ENST00000	ENST00000369001			HORMONE	hg19.100w
MYO1B	56607	23	chr2	2q32.3	+	ENSG0000	myosin IB	protein-co	ENSG0000	ENST00000	ENST00000	ENST00000304164,ENST00000392318				hg19.100w
NUBPL	27126	3	chr14	14q12	+	ENSG0000	nucleotide	protein-co	ENSG0000	ENST00000	ENST00000	ENST00000552489				hg19.100w
PLEKHA4	50820	18	chr19	19q13.33	-	ENSG0000	pleckstrin h	protein-co	ENSG0000	ENST00000	ENST00000	ENST00000263265				hg19.100w
MACF1	1881	107	chr1	1p34.3	+	ENSG0000	microtubul	protein-co	ENSG0000	ENST00000	ENST00000	ENST00000442046,EN		CYTOPLASM		hg19.100w
TNIK	67629	22	chr3	3q26.2-q26	-	ENSG0000	TRAF2 and	protein-co	ENSG0000	ENST00000	ENST00000	ENST00000	STRESS ACTIVATED PR		TRANSFER	hg19.100w
SEC31A	69731	26.1:26.2	chr4	4q21.22	-	ENSG0000	SEC31 hom	protein-co	ENSG0000	ENST00000	ENST00000	ENST00000326950,ENST00000348405,ENST0				hg19.100w
ACPL2	67063	6	chr3	3q23	+	ENSG0000	2-phospho	protein-co	ENSG0000	ENST00000	ENST00000	ENST00000502783,ENST00000513528				hg19.100w
KCTD7	79882	13	chr7	7q11.21	+	ENSG0000	RAB guanin	protein-co	ENSG0000	ENST00000	ENST00000	ENST00000484547,ENST00000503687				hg19.100w
NT5C2	12992	5	chr10	10q24.32-q	-	ENSG0000	5'-nucleoti	protein-co	ENSG0000	ENST00000	ENST00000	ENST00000461461,ENST00000487			PHOSPHOR	hg19.100w
NASP	2706	9	chr1	1p34.1	+	ENSG0000	nuclear aut	protein-co	ENSG0000	ENST00000	ENST00000	ENST00000	CELL PROLI	NUCLEUS		hg19.100w
PLA2G6	62205	14	chr22	22q13.1	-	ENSG0000	phospholip	protein-co	ENSG0000	ENST00000	ENST00000	ENST00000	PHOSPHOLIPID METAB		PHOSPHOL	hg19.100w
NUMA1	17518	4	chr11	11q13.4	-	ENSG0000	nuclear mit	protein-co	ENSG0000	ENST00000	ENST00000	ENST00000	M PHASE O	CYTOSKELE	STRUCTUR	hg19.100w
C16orf13	32915	4	chr16	16p13.3	-	ENSG0000	methyltran	protein-co	ENSG0000	ENST00000	ENST00000	ENST00000301686,ENST00000568077				hg19.100w
TNC	87360	19	chr9	9q33.1	-	ENSG0000	tenascin C	protein-co	ENSG0000	ENST00000	ENST00000	ENST00000346706				hg19.100w
DOCK7	3256	24	chr1	1p31.3	-	ENSG0000	dedicator o	protein-co	ENSG0000	ENST00000	ENST00000	ENST00000251157,ENST00000454575				hg19.100w
MYO19	40481	23	chr17	17q11.2	-	ENSG0000	zinc finger	protein-co	ENSG0000	ENST00000	ENST00000	ENST00000586908				hg19.100w
NDUFAF6	84596	12	chr8	8q22.1	+	ENSG0000	NADH:ubiq	protein-co	ENSG0000	ENST00000	ENST00000	ENST00000286687,ENST00000520632,ENST0				hg19.100w
ZMIZ2	79559	9	chr7	7p13	+	ENSG0000	zinc finger	protein-co	ENSG0000	ENST00000	ENST00000	ENST00000	REGULATIO	INTRACELL	TRANSCRIP	hg19.100w
CARD8	50715	7.2	chr19	19q13.33	-	ENSG0000	caspase re	protein-co	ENSG0000	ENST00000	ENST00000	ENST00000	CELLULAR	CYTOPLAS	CASPASE R	hg19.100w
STRADA	42973	4	chr17	17q23.3	-	ENSG0000	STE20-rela	protein-co	ENSG0000	ENST00000	ENST00000	ENST00000	MACROMO	NUCLEUS;C	PHOSPHOT	hg19.100w
DNAJC5	60176	5	chr20	20q13.33	+	ENSG0000	DnaJ heat s	protein-co	ENSG0000	ENST00000	ENST00000	ENST00000470551				hg19.100w
DCAF6	8887	13.1	chr1	1q24.2	+	ENSG0000	DDB1 and	protein-co	ENSG0000	ENST00000	ENST00000	ENST00000367843				hg19.100w
TMEM116	24566	3	chr12	12q24.13	-	ENSG0000	transmemb	protein-co	ENSG0000	ENST00000	ENST00000	ENST00000354825,ENST00000355445,ENST0				hg19.100w
HSF1	85559	10	chr8	8q24.3	+	ENSG0000	heat shock	protein-co	ENSG0000	ENST00000	ENST00000	ENST00000400780,ENST00000530			TRANSCRIP	hg19.100w
TBC1D23	65817	15	chr3	3q12.1-q12	+	ENSG0000	TBC1 doma	protein-co	ENSG0000	ENST00000	ENST00000	ENST00000394144,ENST00000475134				hg19.100w
ZNF185	90401	15	chrX	Xq28	+	ENSG0000	zinc finger	protein-co	ENSG0000	ENST00000	ENST00000	ENST00000370270,ENST00000436			CATION BI	hg19.100w
CAMTA2	38634	7.1:7.2:7.3	chr17	17p13.2	-	ENSG0000	calmodulin	protein-co	ENSG0000	ENST00000	ENST00000	ENST00000571831,ENST00000573004,ENST0				hg19.100w
RPS6KB2	17203	6.1:6.2	chr11	11q13.2	+	ENSG0000	ribosomal	protein-co	ENSG0000	ENST00000	ENST00000	ENST00000	TRANSLATION;CELLUL		TRANSFER	hg19.100w
CADM1	18849	11	chr11	11q23.3	-	ENSG0000	cell adhesi	protein-co	ENSG0000	ENST00000	ENST00000	ENST00000	POSITIVE R	PLASMA M	PROTEIN D	hg19.100w
FN1	57398	25	chr2	2q35	-	ENSG0000	fibronectin	protein-co	ENSG0000	ENST00000	ENST00000	ENST00000323926,EN		CYTOPLASMIC PART;C		hg19.100w
FBLN5	28893	7	chr14	14q32.12	-	ENSG0000	fibulin 5	protein-co	ENSG0000	ENST00000	ENST00000	ENST00000	CELL SUBS	SOLUBLE F	RECEPTOR	hg19.100w
CYTH1	43891	12	chr17	17q25.3	-	ENSG0000	cytohesin 1	protein-co	ENSG0000	ENST00000	ENST00000	ENST00000	VESICLE M	PLASMA M	GTPASE RE	hg19.100w

Gene Svmbol	dbSNP_variant	COSMIC_variant	SPIDEX_variant	miRNA_binding_site	repeat.	NMD	COSMIC_NMD	dbSNP_NMD	protein_domain	protein_translational_modification	RBP_splicing_factor	RBP	tissue	as	isoform_PPI_a	isoform_PPI_b	isoform_subcellular_localization_id
CAPN3	ay.phastCo	chr15:4268	chr15:4268	NA			NA			Chain,1-821,chr15:42652004-4270			brain:-0.43		ENST00000	TFCP2/CEP	NA
SCRIB	rs370457362,chr8:144		chr8:14487	NA			NA			Chain,1-16	HNRNPK,5.	AKAP8L,4.6	brain:-0.526;stomach:-0.232;bone				NA
DMKN	ay.phastCons:(E)0.094/		chr19:3598	NA			NA			Chain,24-1	IGF2BP3,8.	EFTUD2,1.	thyroid:0.253;stomach:0.203;panc				NA
TACC2	rs1139750	chr10:1239	chr10:1239	NA	Interrupted Rpts,chr10		NA				HNRNPK,6.	AGGF1,7.551e-07,chr1			ENST00000	SMARCB1/	NA
MYO1B	ay.phastCons:(E)0.291/		chr2:19226	NA			NA			Chain,1-11	HNRNPC,2.	AGGF1,3.7	brain:-0.388;bonemarrow:-0.613;p				NA
NUBPL	ay.phastCo	chr14:3203	chr14:3203	NA	RepeatMasker,SINE,ch		NA			Chain,23-1	RBM15,3.9	AGGF1,4.527e-07,chr14:32034219-32034252,					NA
PLEKHA4	ay.phastCo	chr19:4934	chr19:4934	NA			NA			Chain,1-77	SRSF1,3.64	YBX3,1.193	brain:-0.228;thyroid:-0.059;bonem				NA
MACF1	ay.phastCo	chr1:39945	chr1:39945	NA			NA			Chain,1-73	FMR1,5.55	AGGF1,2.4	brain:0.105		ENST00000	SNAPIN/FH	NA
TNIK	ay.phastCons:(E)0.64/(I		chr3:17081	NA			NA			Chain,1-13	FMR1,1.83	BUD13,1.3	brain:-0.05		ENST00000	GOLGA4/N	NA
SEC31A	ay.phastCo	chr4:83765	chr4:83750	NA			NA			Chain,1-12	HNRNPC,2.	BUD13,4.8	brain:-0.117;pancreas:-0.105;skin:				NA
ACPL2	rs138761221,chr3:140		chr3:14097	NA		chr3:14098	NA			Chain,1-48	PCBP2,3.88	GTF2F1,1.4	brain:-0.055;thyroid:0.09;bonema				NA
KCTD7	ay.phastCo	chr7:66262	chr7:66260	NA	RepeatMasker,SINE,ch		NA				SRSF1,1.21	BUD13,2.180e-14,chr7:66260497-66260518,2					NA
NT5C2	ay.phastCons:(E)0.878/		chr10:1048	NA			NA			Chain,1-56	HNRNPA1,	BUD13,1.2	brain:-0.069;thyroid:-0.163;bonem				NA
NASP	ay.phastCons:(E)0.196/		chr1:46072	NA			NA			Acetylation	HNRNPA1,	BUD13,2.8	brain:-0.415;thyroid:-0.107;pancre				NA
PLA2G6	rs3732817	chr22:3852	chr22:3851	NA	RepeatMasker,SINE,ch		NA			Chain,1-80	HNRNPH1,	AGGF1,2.3	brain:0.462;thyroid:-0.078;pancre				NA
NUMA1	ay.phastCons:(E)0.246/		chr11:7174	NA	RepeatMasker,SINE,ch		NA				FMR1,2.07	BUD13,4.386e-07,chr11:71746953-71746976,					NA
C16orf13	ay.phastCons:(E)0.339/		chr16:6847	NA			NA			Chain,1-20	HNRNPK,1.	BUD13,2.9	brain:0.143;thyroid:0.215;pancrea				NA
TNC	ay.phastCo	chr9:11780	chr9:11780	NA			NA		ENST00000	Chain,20-1928,chr9:117783439-11			brain:-0.695;thyroid:-0.15;stomac				NA
DOCK7	ay.phastCo	chr1:63018	chr1:63009	NA			NA				HNRNPK,2.	AGGF1,1.1	brain:-0.16		ENST00000	SIRT7/LRCH	NA
MYO19	rs1932145	chr17:3485	chr17:3485	NA			NA				FMR1,3.07	AGGF1,2.7	brain:-0.771;stomach:0.069;skin:0				NA
NDUFAF6	rs34960210,chr8:9604		chr8:96047	NA		chr8:96048	NA		ENST00000	Chain,45-3	KHSRP,8.94	BUD13,1.6	stomach:0.101;bonemarrow:-0.20				NA
ZMIZ2	ay.phastCons:(E)0.361/		chr7:44798	NA			NA			Chain,1-92	HNRNPA1,	BCCIP,4.48	brain:0.354		ENST00000	BANP/SELV	NA
CARD8	rs2013617	chr19:4873	chr19:4873	NA			NA				RBM15,4.0	BUD13,4.3	brain:0.379;thyroid:0.279;pancrea				NA
STRADA	rs3580815	chr17:6180	chr17:6179	NA			NA			Chain,1-43	HNRNPA1,	ADAR,1.87	brain:-0.548;thyroid:-0.086;stoma				NA
DNAJC5	rs1411033	chr20:6256	chr20:6256	NA	SelfChain,c	chr20:6256	NA			Chain,1-19	IGF2BP2,4.	CSTF2,1.85	brain:0.271;thyroid:0.072;bonema				NA
DCAF6	ay.phastCo	chr1:16797	chr1:16797	NA	SelfChain,chr1:167992		NA			Chain,1-86	ELAVL1,1.0	BUD13,3.3	brain:0.231;thyroid:0.058;stomach				NA
TMEM116	ay.phastCons:(E)0.654/		chr12:1124	NA			NA				HNRNPU,5	BUD13,2.624e-13,chr12:112429565-1124296					NA
HSF1	ay.phastCo	chr8:14553	chr8:14553	NA			NA		ENST00000	Chain,1-52	FXR1,1.360	BUD13,4.7	stomach:-0.072;skin:-0.136;smalli				NA
TBC1D23	ay.phastCons:(E)0.498/		chr3:10002	NA			NA			Chain,1-69	FMR1,2.42	AGGF1,4.0	brain:-0.22		ENST00000	KRTAP10-9	NA
ZNF185	rs1913280	chrX:15210	chrX:15210	NA			NA			Chain,1-689,chrX:152083034-1521			brain:0.19;bonemarrow:0.079;pan				NA
CAMTA2	ay.phastCons:(E)0.885/		chr17:4884	NA			NA		ENST00000	Chain,1-12	HNRNPK,1.	BUD13,2.1	brain:-0.073;thyroid:-0.102;pancre				NA
RPS6KB2	rs2005305	chr11:6719	chr11:6719	NA	RepeatMas	chr11:6719	NA		ENST00000	Chain,1-48	HNRNPK,2.	BUD13,2.6	brain:-0.536;stomach:0.095;ovary				NA
CADM1	ay.phastCons:(E)0.513/		chr11:1150	NA			NA			Chain,45-4	HNRNPA1,	DDX55,1.0	brain:-0.651;thyroid:-0.109;stoma				NA
FN1	ay.phastCons:(E)0.405/		chr2:21625	NA			NA			Chain,32-2	IGF2BP1,0.	AGGF1,8.0	thyroid:-0.127;stomach:-0.217;bo				NA
FBLN5	ay.phastCo	chr14:9240	chr14:9240	NA	RepeatMasker,SINE,ch		NA			Chain,24-91,chr14:92403397-92409253;Chain,24-448,chr14:9233657							NA
CYTH1	rs3696251	chr17:7669	chr17:7668	NA			NA			Chain,1-39	FXR1,8.020	BUD13,4.2	brain:0.499;thyroid:0.584;stomach				NA

Gene Symbol	isoform_subcellular_localization	tds.corr	tds.pval	erk.corr	erk.pval	ras.corr	ras.pval	ETE.pval	Lymph_node_metastasis.pval
CAPN3	NA	0.32	3.6E-11	-0.25	2.9E-07	0.25	4.9E-07	2.0E-01	2.3E-02
SCRIB	NA	-0.29	5.6E-11	0.17	8.6E-05	-0.28	2.6E-10	8.8E-05	2.1E-06
DMKN	NA	-0.29	6.6E-11	0.07	1.2E-01	-0.12	7.7E-03	6.8E-02	4.6E-04
TACC2	NA	-0.28	8.9E-11	-0.11	1.8E-02	-0.02	7.0E-01	5.3E-01	8.7E-01
MYO1B	NA	0.29	1.6E-10	-0.27	3.7E-09	0.24	2.1E-07	1.4E-04	1.8E-01
NUBPL	NA	-0.29	1.8E-10	-0.06	1.8E-01	-0.12	9.6E-03	3.1E-01	1.4E-04
PLEKHA4	NA	-0.15	1.2E-03	0.13	3.6E-03	-0.29	2.6E-10	2.3E-01	5.2E-04
MACF1	NA	-0.12	6.3E-03	0.14	1.2E-03	-0.28	3.5E-10	3.4E-02	1.0E-01
TNIK	NA	0.29	5.9E-10	-0.12	1.1E-02	0.29	1.9E-09	1.6E-01	1.7E-01
SEC31A	NA	-0.20	7.0E-06	0.23	1.3E-07	-0.28	7.3E-10	6.6E-02	2.4E-01
ACPL2	NA	0.29	1.6E-09	-0.20	4.5E-05	0.30	7.7E-10	9.8E-05	3.9E-03
KCTD7	NA	0.00	1.0E+00	-0.18	6.9E-05	0.28	8.6E-10	4.7E-02	3.1E-02
NT5C2	NA	-0.05	2.9E-01	-0.27	8.8E-10	0.01	8.7E-01	7.1E-01	8.0E-01
NASP	NA	-0.24	5.8E-08	0.18	6.9E-05	-0.27	9.8E-10	1.2E-02	2.5E-03
PLA2G6	NA	-0.27	1.0E-09	-0.03	5.3E-01	-0.26	1.2E-08	3.8E-02	1.0E-03
NUMA1	NA	0.01	8.6E-01	-0.16	4.3E-04	0.27	1.1E-09	3.1E-02	2.1E-01
C16orf13	NA	-0.21	1.5E-06	0.26	2.1E-09	-0.13	3.6E-03	3.5E-02	7.4E-02
TNC	NA	-0.29	2.3E-09	0.17	5.1E-04	-0.14	6.6E-03	1.0E-01	1.2E-01
DOCK7	NA	-0.26	6.0E-08	0.17	5.7E-04	-0.29	4.3E-09	1.0E-02	5.2E-02
MYO19	NA	-0.04	3.5E-01	-0.26	4.3E-09	0.03	4.8E-01	1.1E-01	4.0E-01
NDUFAF6	NA	0.18	7.2E-05	-0.26	4.4E-09	0.17	2.2E-04	4.2E-03	3.3E-02
ZMIZ2	NA	-0.26	6.1E-09	-0.02	5.9E-01	-0.26	9.0E-09	4.4E-01	9.2E-04
CARD8	NA	-0.27	8.0E-09	0.19	3.5E-05	-0.24	3.2E-07	1.1E-01	1.4E-01
STRADA	NA	-0.27	9.0E-09	0.18	1.4E-04	-0.22	7.2E-06	1.2E-03	2.9E-04
DNAJC5	NA	0.25	9.9E-09	-0.23	1.1E-07	0.24	1.2E-07	4.7E-04	3.4E-04
DCAF6	NA	-0.19	1.6E-05	0.25	1.3E-08	-0.25	4.5E-08	1.1E-03	3.2E-02
TMEM116	NA	0.25	3.0E-08	-0.07	1.2E-01	0.27	1.4E-08	3.4E-01	3.4E-01
HSF1	NA	-0.25	1.7E-08	0.09	5.1E-02	-0.22	2.0E-06	2.6E-04	8.0E-04
TBC1D23	NA	-0.25	1.9E-08	0.05	3.0E-01	-0.17	3.3E-04	9.8E-01	9.8E-02
ZNF185	NA	0.20	1.2E-05	-0.22	1.3E-06	0.26	2.1E-08	3.7E-03	1.9E-01
CAMTA2	NA	-0.25	3.4E-08	0.10	2.9E-02	-0.19	7.0E-05	9.4E-03	3.4E-03
RPS6KB2	NA	-0.03	4.4E-01	-0.24	3.9E-08	0.08	7.4E-02	8.9E-01	2.2E-01
CADM1	NA	-0.23	1.7E-07	0.09	4.3E-02	-0.25	4.0E-08	7.7E-04	7.1E-04
FN1	NA	-0.09	4.6E-02	-0.17	1.4E-04	-0.25	4.8E-08	5.4E-02	2.4E-01
FBLN5	NA	-0.03	4.6E-01	-0.15	6.6E-04	0.24	9.0E-08	2.9E-03	1.9E-03
CYTH1	NA	0.17	1.3E-04	-0.13	4.8E-03	0.24	1.0E-07	5.4E-05	5.4E-03

Gene Symbol	as_id	exon	chr	location	strand	as_description_id	gene_name	locus_group	gene_id	transcript_id	exon_inclusion_transcript_id	exon_exclusion_transcript_id	GO_BP	GO_CC	GO_MF	conservation_score
PPIP5K1	30267	31	chr15	15q15.3	-	ENSG0000	diphosphoi	protein-co	ENSG0000	ENST00000	ENST00000	ENST00000348806,ENST00000360135				hg19.100w
EPOR	47692	4.1:4.2	chr19	19p13.2	-	ENSG0000	erythropoi	protein-co	ENSG0000	ENST00000	ENST00000	ENST00000586890		PLASMA M	RECEPTOR	hg19.100w
MFF	57810	9	chr2	2q36.3	+	ENSG0000	mitochond	protein-co	ENSG0000	ENST00000	ENST00000	ENST00000456345				hg19.100w
LEF1	70294	7	chr4	4q25	-	ENSG0000	lymphoid e	protein-co	ENSG0000	ENST00000	ENST00000	ENST00000265165,ENST00000504			DNA BINDI	hg19.100w
SMURF2	43074	4	chr17	17q23.3-q2	-	ENSG0000	SMAD spec	protein-co	ENSG0000	ENST00000	ENST00000	ENST00000	CATABOLIC	PROTEIN C	UBIQUITIN	hg19.100w
TERF1	84141	7	chr8	8q21.11	+	ENSG0000	telomeric r	protein-co	ENSG0000	ENST00000	ENST00000	ENST00000276603		NUCLEUS	DNA BINDI	hg19.100w
C14orf159	28858	6	chr14	14q32.11	+	ENSG0000	chromoso	protein-co	ENSG0000	ENST00000	ENST00000	ENST00000412671,ENST00000518868				hg19.100w
SMARCC2	22393	18	chr12	12q13.2	-	ENSG0000	SWI/SNF re	protein-co	ENSG0000	ENST00000	ENST00000	ENST00000	REGULATIO	PROTEIN C	TRANSCRIP	hg19.100w
PTK2B	83156	28	chr8	8p21.2	+	ENSG0000	protein tyr	protein-co	ENSG0000	ENST00000	ENST00000	ENST00000	PROTEIN C	CYTOPLAS	TRANSFER	hg19.100w
SPTAN1	87772	38	chr9	9q34.11	+	ENSG0000	spectrin al	protein-co	ENSG0000	ENST00000	ENST00000	ENST00000372739		CORTICAL	STRUCTUR	hg19.100w
FN1	57397	33	chr2	2q35	-	ENSG0000	fibronectin	protein-co	ENSG0000	ENST00000	ENST00000	ENST00000323926,EN		CYTOPLASMIC PART;C		hg19.100w
FGFR1	83430	6	chr8	8p11.23	-	ENSG0000	fibroblast g	protein-co	ENSG0000	ENST00000	ENST00000	ENST00000	INTRACELL	PLASMA M	TRANSFER	hg19.100w
UPF3B	89980	8	chrX	Xq24	-	ENSG0000	UPF3 regul	protein-co	ENSG0000	ENST00000	ENST00000	ENST00000276201				hg19.100w
ANXA6	74148	22	chr5	5q33.1	-	ENSG0000	annexin A6	protein-co	ENSG0000	ENST00000	ENST00000	ENST00000354546,ENST00000377751,ENST0				hg19.100w
LPIN1	52712	10	chr2	2p25.1	+	ENSG0000	lipin 1	protein-co	ENSG0000	ENST00000	ENST00000	ENST00000396098,EN		NUCLEUS		hg19.100w
EPOR	47693	4.2	chr19	19p13.2	-	ENSG0000	erythropoi	protein-co	ENSG0000	ENST00000	ENST00000	ENST00000588859		PLASMA M	RECEPTOR	hg19.100w
AP1G2	99354	5	chr14	14q11.2	-	ENSG0000	adaptor rel	protein-co	ENSG0000	ENST00000	ENST00000	ENST00000308724,EN		CYTOPLASMIC MEMBR		hg19.100w
SNX14	76924	16	chr6	6q14.3	-	ENSG0000	sorting nex	protein-co	ENSG0000	ENST00000	ENST00000	ENST00000314673,ENST00000369635,ENST0				hg19.100w
CD46	9652	13	chr1	1q32.2	+	ENSG0000	CD46 mole	protein-co	ENSG0000	ENST00000	ENST00000	ENST00000358170,EN		PLASMA M	RECEPTOR	hg19.100w
MYO5A	30660	35	chr15	15q21.2	-	ENSG0000	myosin VA	protein-co	ENSG0000	ENST00000	ENST00000	ENST00000358212,EN		LEADING EDGE;RUFFLE		hg19.100w
ABI1	11042	11.2	chr10	10p12.1	-	ENSG0000	abl interact	protein-co	ENSG0000	ENST00000	ENST00000	ENST00000	CELL PROLI	SOLUBLE F	CYTOSKELE	hg19.100w
AP1G1	37485	12	chr16	16q22.2	-	ENSG0000	adaptor rel	protein-co	ENSG0000	ENST00000	ENST00000	ENST00000	PROTEIN L	CYTOPLASMIC MEMBR		hg19.100w
TCF7	73350	6	chr5	5q31.1	+	ENSG0000	transcriptio	protein-co	ENSG0000	ENST00000	ENST00000	ENST00000	REGULATION OF TRAN		RNA POLY	hg19.100w
SPAG9	42494	30	chr17	17q21.33	-	ENSG0000	sperm asso	protein-co	ENSG0000	ENST00000	ENST00000	ENST00000	GAMETE G	INTEGRAL TO MEMBR		hg19.100w
THEMIS2	1354	3	chr1	1p35.3	+	ENSG0000	thymocyte	protein-co	ENSG0000	ENST00000	ENST00000	ENST00000373925				hg19.100w
AP1B1	61603	24	chr22	22q12.2	-	ENSG0000	adaptor rel	protein-co	ENSG0000	ENST00000	ENST00000	ENST00000356015,ENST00000357586,ENST0				hg19.100w
MYO9B	48232	37	chr19	19p13.11	+	ENSG0000	myosin IXB	protein-co	ENSG0000	ENST00000	ENST00000	ENST00000	SMALL GTP	ACTIN FILA	ACTIN BIN	hg19.100w
MALT1	45679	7	chr18	18q21.32	+	ENSG0000	MALT1 par	protein-co	ENSG0000	ENST00000	ENST00000	ENST00000	REGULATIO	NUCLEUS;C	UBIQUITIN	hg19.100w
COL16A1	1493	44	chr1	1p35.2	-	ENSG0000	collagen ty	protein-co	ENSG0000	ENST00000	ENST00000	ENST00000	REPRODUC	COLLAGEN	INTEGRIN B	hg19.100w
FBLN2	63511	11	chr3	3p25.1	+	ENSG0000	fibulin 2	protein-co	ENSG0000	ENST00000	ENST00000	ENST00000404922,EN		EXTRACELL	EXTRACELL	hg19.100w
SLK	13030	13	chr10	10q24.33-q	+	ENSG0000	STE20 like	protein-co	ENSG0000	ENST00000	ENST00000	ENST00000369755				hg19.100w
UAP1	8750	9.1:9.2	chr1	1q23.3	+	ENSG0000	UDP-N-ace	protein-co	ENSG0000	ENST00000	ENST00000	ENST00000	GLUCOSAMINE METAB		NUCLEOTID	hg19.100w
ARHGEF1	50101	15	chr19	19q13.2	+	ENSG0000	Rho guanin	protein-co	ENSG0000	ENST00000	ENST00000	ENST00000	CELL PROLI	PLASMA M	GUANYL N	hg19.100w
UAP1	8751	9.2	chr1	1q23.3	+	ENSG0000	UDP-N-ace	protein-co	ENSG0000	ENST00000	ENST00000	ENST00000	GLUCOSAMINE METAB		NUCLEOTID	hg19.100w
RALGPS2	9094	15	chr1	1q25.2	+	ENSG0000	Ral GEF wit	protein-co	ENSG0000	ENST00000	ENST00000	ENST00000367635,ENST00000480743				hg19.100w
MBNL1	67318	10	chr3	3q25.1-q25	+	ENSG0000	muscleblin	protein-co	ENSG0000	ENST00000	ENST00000	ENST00000	MULTICELL	NUCLEUS;C	RNA BINDI	hg19.100w

Gene Svmbol	dbSNP_variant	COSMIC_variant	SPIDEX_variant	miRNA_binding_site	repeat.	NMD	COSMIC_NMD	dbSNP_NMD	protein_domain	protein_translational_modification	RBP_splicing_factor	RBP	tissue as	isoform_PPI_a	isoform_PPI_b	isoform_subcellular_localization_id
PPIP5K1	ay.phastCo	chr15:4383	chr15:4382	NA	RepeatMasker,SINE,ch		NA			Chain,1-14	FXR1,4.734	CHD4,1.150e-05,chr15:43826552-43826563,1				NA
EPOR	rs1512260	chr19:1149	chr19:1149	NA			NA			Chain,25-5	SRSF1,5.07	BUD13,1.8	brain:-0.276;bonemarrow:0.413;s			NA
MFF	rs1437681	chr2:22822	chr2:22820	NA			NA			Chain,1-34	HNRNPA1,	BUD13,5.2	brain:-0.524;thyroid:-0.352;stoma			NA
LEF1	rs113018313,chr4:109		chr4:10900	NA			NA			Chain,1-39	IGF2BP1,2.	ILF3,1.159e	brain:0.812	ENST00000	SMAD4/AL	NA
SMURF2	rs148243478,chr17:62		chr17:6258	NA	SelfChain,c	chr17:6259	NA			Chain,1-748,chr17:625		EZH2,1.130	thyroid:0.122;stomach:0.064;panc			NA
TERF1	ay.phastCons:(E)0.145/		chr8:73939	NA	Interrupted Rpts,chr8:		NA			Acetylation	KHSRP,1.17	CHD1,4.62	brain:-0.58	ENST00000	ACD/NUM	NA
C14orf159	ay.phastCons:(E)0.057/		chr14:9161	NA	RepeatMasker,SINE,ch		NA				HNRNPC,8.	DKC1,4.526e-08,chr14:91623628-91623693,6				NA
SMARCC2	ay.phastCons:(E)0.807/		chr12:5656	NA			NA			Chain,1-12	FMR1,1.52	AGGF1,8.6	brain:-0.49	ENST00000	ITSN1/PEX	NA
PTK2B	rs1128435,	chr8:27301	chr8:27301	NA			NA			Chain,1-10	FXR1,5.926	AKAP8L,3.4	brain:-0.833;thyroid:-0.77;stomac			NA
SPTAN1	rs1848630	chr9:13137	chr9:13137	NA			NA		ENST00000	Chain,1-24	FMR1,5.10	AGGF1,1.8	brain:-0.748;pancreas:0.102;skin:0			NA
FN1	rs1996841	chr2:21624	chr2:21624	NA			NA			Chain,32-2	HNRNPA1,	BUD13,0.0	stomach:-0.144;bonemarrow:-0.0			NA
FGFR1	ay.phastCons:(E)0.289/		chr8:38285	NA			NA		ENST00000	Chain,22-6	HNRNPU,1	BUD13,4.553e-18,chr8		ENST00000	PIK3R1/GR	NA
UPF3B	ay.phastCo	chrX:11897	chrX:11897	NA			NA			Chain,1-48	KHSRP,2.28	AGGF1,3.3	brain:-0.13	ENST00000	NCBP1/MC	NA
ANXA6	rs3677083	chr5:15049	chr5:15048	NA			NA		ENST00000	Chain,2-67	FMR1,2.26	AGGF1,1.8	stomach:0.052;pancreas:0.308;col			NA
LPIN1	ay.phastCons:(E)0.284/		chr2:11913	NA			NA			Chain,1-89	FMR1,1.52	BUD13,2.9	brain:-0.37;thyroid:-0.551;stomac			NA
EPOR	rs1512260	chr19:1149	chr19:1149	NA			NA			Chain,25-5	SRSF1,5.07	BUD13,1.8	brain:-0.293;stomach:0.177;bone			NA
AP1G2	ay.phastCons:(E)0.363;		chr14:2403	NA			NA			Chain,1-78	RBM15,1.6	AGGF1,4.2	brain:0.73;thyroid:0.412;stomach:			NA
SNX14	ay.phastCo	chr6:86248	chr6:86246	NA			NA		ENST00000	Chain,1-94	FXR1,1.130	AGGF1,3.1	brain:-0.511;thyroid:-0.053;stoma			NA
CD46	rs68086922,chr1:2079		chr1:20795	NA			NA	chr1:207963626		Chain,35-3	HNRNPA1,	BUD13,1.9	brain:-0.07	ENST00000	CD46;U48	NA
MYO5A	ay.phastCons:(E)0.842/		chr15:5262	NA			NA			Chain,2-1855,chr15:52605896-528			brain:0.444;thyroid:0.464;stomach			NA
ABI1	rs141177976,chr10:27		chr10:2704	NA			NA				IGF2BP1,1.	BUD13,1.0	brain:-0.33	ENST00000	GAS7/NCK	NA
AP1G1	ay.phastCons:(E)0.674/		chr16:7179	NA			NA		ENST00000	Chain,2-82	FXR1,1.103	AGGF1,5.2	brain:-0.73	ENST00000	UBL7/AP1S	NA
TCF7	rs373694202,chr5:133		chr5:13347	NA			NA		ENST00000520958,PF0		HNRNPA1,	FAM120A,	bonemarro	ENST00000	SS18L1/UB	NA
SPAG9	ay.phastCo	chr17:4905	chr17:4905	NA			NA			Chain,1-13	FXR1,7.131	BUD13,2.6	brain:-0.31	ENST00000	MAP2K4/S	NA
THEMIS2	ay.phastCons:(E)0.231/		chr1:28203	NA			NA		ENST00000	Chain,1-64	HNRNPH1,	CHD4,1.16	brain:0.576;thyroid:0.089;stomach			NA
AP1B1	rs1918277	chr22:2972	chr22:2972	NA			NA				FMR1,4.01	BUD13,1.5	brain:-0.378;thyroid:0.28;stomach			NA
MYO9B	rs1132129	chr19:1732	chr19:1732	NA			NA				QKI,7.556e	AKAP8L,1.4	brain:-0.63	ENST00000	CALM3/MY	NA
MALT1	ay.phastCons:(E)0.826/		chr18:5637	NA			NA			Chain,2-82	HNRNPA1,	BUD13,8.4	brain:-0.37	ENST00000	MALT1/TR	NA
COL16A1	ay.phastCo	chr1:32145	chr1:32145	NA			NA			Chain,22-9	SRSF1,6.26	GRWD1,6.2	brain:-0.054;thyroid:0.29;pancrea			NA
FBLN2	ay.phastCo	chr3:13661	chr3:13661	NA			NA			Chain,28-1184,chr3:13611937-136			brain:-0.261;stomach:-0.053;bone			NA
SLK	ay.phastCons:(E)0.926/		chr10:1057	NA			NA			Chain,1-12	ELAVL1,3.4	BUD13,2.5	thyroid:-0.756;stomach:-0.744;bo			NA
UAP1	rs61743675,chr1:1625		chr1:16256	NA			NA		ENST00000	Chain,1-52	FXR1,1.165	BUD13,3.7	thyroid:-0.783;stomach:-0.69;panc			NA
ARHGEF1	rs2303797,	chr19:4240	chr19:4240	NA			NA			Chain,1-91	HNRNPK,8.	CSTF2T,1.9	thyroid:0.2	ENST00000	CNKSR1/AR	NA
UAP1	rs61743675,chr1:1625		chr1:16256	NA			NA		ENST00000	Chain,1-52	FXR1,1.165	BUD13,3.7	stomach:-0.599;pancreas:-0.706;s			NA
RALGPS2	ay.phastCo	chr1:17886	chr1:17885	NA			NA			Chain,1-58	KHSRP,2.08	BUD13,3.0	brain:-0.216;thyroid:-0.799;stoma			NA
MBNL1	ay.phastCo	chr3:15217	chr3:15216	NA			NA			Chain,1-38	HNRNPK,2.	BUD13,4.972e-41,chr3		ENST00000	TIAL1/DDX	NA

Gene Symbol	isoform_subcellular_localization	tds.corr	tds.pval	erk.corr	erk.pval	ras.corr	ras.pval	ETE.pval	Lymph_node_metastasis.pval
PPIP5K1	NA	-0.05	2.2E-01	-0.23	1.1E-07	0.16	3.4E-04	8.9E-02	3.4E-02
EPOR	NA	-0.23	1.4E-07	-0.16	2.7E-04	-0.08	9.6E-02	5.7E-01	3.5E-02
MFF	NA	0.15	6.4E-04	-0.23	2.0E-07	0.12	1.0E-02	3.9E-02	4.1E-02
LEF1	NA	-0.22	2.4E-06	0.14	1.8E-03	-0.24	2.2E-07	8.2E-01	3.7E-01
SMURF2	NA	0.06	1.9E-01	-0.12	6.9E-03	0.23	3.4E-07	1.4E-02	3.4E-02
TERF1	NA	-0.17	1.8E-04	0.15	6.4E-04	-0.23	3.7E-07	8.2E-02	8.0E-01
C14orf159	NA	-0.23	4.1E-07	-0.19	3.0E-05	0.01	7.7E-01	7.0E-01	4.3E-02
SMARCC2	NA	0.15	8.5E-04	-0.22	5.1E-07	0.15	1.5E-03	1.7E-01	1.5E-01
PTK2B	NA	0.05	2.3E-01	-0.22	5.9E-07	0.08	8.7E-02	2.5E-01	1.6E-01
SPTAN1	NA	-0.08	8.6E-02	-0.22	6.3E-07	-0.01	8.1E-01	1.5E-01	3.1E-01
FN1	NA	0.13	2.7E-03	-0.21	2.3E-06	0.22	7.3E-07	3.8E-01	8.7E-02
FGFR1	NA	0.22	1.6E-06	-0.19	2.4E-05	0.17	3.0E-04	1.7E-04	1.9E-04
UPF3B	NA	0.21	2.1E-06	-0.03	5.3E-01	0.08	6.7E-02	1.2E-01	1.5E-02
ANXA6	NA	-0.20	3.6E-06	0.01	9.1E-01	-0.20	1.3E-05	1.7E-01	2.2E-02
LPIN1	NA	0.22	4.0E-06	-0.21	8.6E-06	0.22	3.7E-06	1.4E-01	1.4E-01
EPOR	NA	-0.18	9.2E-05	-0.21	4.7E-06	0.02	6.6E-01	1.3E-01	1.4E-01
AP1G2	NA	0.20	6.6E-06	0.18	4.9E-05	0.00	9.3E-01	3.7E-01	5.1E-02
SNX14	NA	-0.09	3.5E-02	-0.10	2.2E-02	-0.20	1.1E-05	1.6E-02	5.1E-01
CD46	NA	-0.15	5.1E-04	0.03	5.5E-01	-0.20	1.7E-05	5.1E-01	1.8E-02
MYO5A	NA	-0.20	1.8E-05	0.11	1.8E-02	-0.19	9.5E-05	6.1E-01	6.8E-02
ABI1	NA	0.02	7.3E-01	-0.19	3.0E-05	0.02	6.7E-01	1.4E-01	6.0E-01
AP1G1	NA	0.08	9.3E-02	-0.18	2.4E-04	-0.02	7.5E-01	5.7E-01	2.0E-02
TCF7	NA	0.04	3.8E-01	0.17	4.2E-04	0.05	2.9E-01	4.0E-01	4.7E-01
SPAG9	NA	-0.16	4.5E-04	-0.09	5.3E-02	-0.11	2.1E-02	2.1E-01	2.2E-02
THEMIS2	NA	-0.14	2.6E-03	0.16	5.7E-04	-0.11	2.0E-02	1.8E-01	3.7E-02
AP1B1	NA	-0.14	1.2E-03	-0.05	2.5E-01	0.01	8.7E-01	9.7E-01	1.6E-01
MYO9B	NA	0.07	1.2E-01	-0.15	1.3E-03	0.07	1.7E-01	2.4E-01	6.6E-01
MALT1	NA	0.08	7.5E-02	-0.14	3.3E-03	0.09	7.3E-02	9.1E-01	1.4E-03
COL16A1	NA	0.07	1.3E-01	-0.03	5.4E-01	0.14	1.8E-03	2.5E-01	2.1E-01
FBLN2	NA	0.13	3.7E-03	-0.03	5.0E-01	-0.06	1.9E-01	7.9E-01	2.7E-01
SLK	NA	0.04	3.4E-01	0.12	7.9E-03	-0.10	3.2E-02	7.1E-01	6.6E-01
UAP1	NA	-0.06	2.1E-01	-0.06	1.7E-01	-0.12	8.4E-03	4.9E-02	7.4E-01
ARHGEF1	NA	-0.12	8.7E-03	-0.01	7.6E-01	-0.08	6.8E-02	1.6E-01	7.2E-02
UAP1	NA	-0.06	2.1E-01	-0.04	3.3E-01	-0.11	1.3E-02	5.7E-02	6.7E-01
RALGPS2	NA	0.03	4.8E-01	-0.04	4.3E-01	0.04	4.6E-01	6.1E-02	2.2E-02
MBNL1	NA	-0.02	6.4E-01	-0.04	3.8E-01	0.02	7.0E-01	7.1E-02	6.9E-01

Gene Symbol	as_id	exon	chr	location	strand	as_description_id	gene_name	locus_group	gene_id	transcript_id	exon_inclusion_transcript_id	exon_exclusion_transcript_id	GO_BP	GO_CC	GO_MF	conservation_score
MBNL1	67324	8	chr3	3q25.1-q25	+	ENSG0000	muscleblin	protein-co	ENSG0000	ENST00000	ENST00000	ENST00000	MULTICELL	NUCLEUS;C	RNA BINDI	hg19.100w
MAP3K7	77020	11	chr6	6q15	-	ENSG0000	mitogen-ac	protein-co	ENSG0000	ENST00000	ENST00000	ENST00000	TRANSFORMING GRO		MAP KINAS	hg19.100w

Gene Svmbol	dbSNP_variant	COSMIC_variant	SPIDEX_variant	miRNA_binding_site	repeat.	NMD	COSMIC_NMD	dbSNP_NMD	protein_domain	protein_translational_modification	RBP_splicing_factor	RBP	tissue as	isoform_PPI_a	isoform_PPI_b	isoform_subcellular_localization_id
MBNL1	ay.phastCons:	(E)0.9/(I)	chr3:15216	NA			NA			Chain,1-38	ELAVL1,1.7	BUD13,2.0	brain:-0.30	ENST00000	TIAL1/DDX	NA
MAP3K7	rs3720445	chr6:91254	chr6:91246	NA			NA			Chain,1-60	FMR1,4.24	BUD13,9.2	brain:-0.11	ENST00000	HSPA5/TNF	NA

Gene Symbol	isoform_subcellular_localization	tds.corr	tds.pval	erk.corr	erk.pval	ras.corr	ras.pval	ETE.pval	Lymph_node_metastasis.pval
MBNL1	NA	0.01	8.0E-01	-0.01	7.7E-01	0.00	9.4E-01	2.8E-01	1.9E-01
MAP3K7	NA	-0.02	6.3E-01	0.04	3.9E-01	-0.03	5.8E-01	5.1E-01	3.4E-01

Supplementary Table 6. Result of random forest classification with 23 ES events.

Gene Symbol	as_id	exons	MeanDecreaseGini
TUBB3	38175	6.1:6.2	32.21
DYSF	53937	19	30.41
KIAA1217	11009	12	27.01
EXOC7	43569	7	21.88
ARHGAP17	35664	18	21.41
LSR	49086	5	21.02
SSBP3	3144	7	20.99
PCYT2	44230	7	19.91
CACNB3	21476	9	16.70
ARPC1B	80609	8	16.83
SH3BP1	62140	16	16.40
UNC5B	12054	8	15.12
CADM1	18851	10	13.85
SSBP4	48431	7	12.45
SYTL2	18153	11.2	12.14
ITGB4	43489	35	11.24
ABLIM1	13209	16	11.22
NUMA1	17515	18	11.23
MROH1	85546	26	11.18
OSBPL3	79027	9	10.85
CCNDBP1	30220	10.1	10.06
TBC1D15	23415	10	7.84
LUC7L	32844	4	7.53
